# Transcriptome dynamics in early zebrafish embryogenesis determined by high-resolution time course analysis of 180 successive, individual zebrafish embryos

**DOI:** 10.1186/s12864-017-3672-z

**Published:** 2017-04-11

**Authors:** Han Rauwerda, Johanna F. B. Pagano, Wim C. de Leeuw, Wim Ensink, Ulrike Nehrdich, Mark de Jong, Martijs Jonker, Herman P. Spaink, Timo M. Breit

**Affiliations:** 1 RNA Biology & Applied Bioinformatics research group, Swammerdam Institute for Life Sciences, Faculty of Science, University of Amsterdam, Amsterdam, The Netherlands; 2 Institute Biology Leiden, Faculty of Science, Leiden University, Leiden, The Netherlands; 3 Present address: GenomeScan B.V., Plesmanlaan, Leiden, The Netherlands; 4 MAD/AB&RB, Swammerdam Institute for Life Sciences, University of Amsterdam, Science Park 904, 1098 XH Amsterdam, The Netherlands

**Keywords:** Maternal RNA, Individual transcriptome, Zebrafish, *Danio rerio*, Embryogenesis, Gastrulation

## Abstract

**Background:**

Recently, much progress has been made in the field of gene-expression in early embryogenesis. However, the dynamic behaviour of transcriptomes in individual embryos has hardly been studied yet and the time points at which pools of embryos are collected are usually still quite far apart. Here, we present a high-resolution gene-expression time series with 180 individual zebrafish embryos, obtained from nine different spawns, developmentally ordered and profiled from late blastula to mid-gastrula stage. On average one embryo per minute was analysed. The focus was on identification and description of the transcriptome dynamics of the expressed genes in this embryonic stage, rather than to biologically interpret profiles in cellular processes and pathways.

**Results:**

In the late blastula to mid-gastrula stage, we found 6,734 genes being expressed with low variability and rather gradual changes. Ten types of dynamic behaviour were defined, such as genes with continuously increasing or decreasing expression, and all expressed genes were grouped into these types. Also, the exact expression starting and stopping points of several hundred genes during this developmental period could be pinpointed. Although the resolution of the experiment was so high, that we were able to clearly identify four known oscillating genes, no genes were observed with a peaking expression. Additionally, several genes showed expression at two or three distinct levels that strongly related to the spawn an embryo originated from.

**Conclusion:**

Our unique experimental set-up of whole-transcriptome analysis of 180 individual embryos, provided an unparalleled in-depth insight into the dynamics of early zebrafish embryogenesis. The existence of a tightly regulated embryonic transcriptome program, even between individuals from different spawns is shown. We have made the expression profile of all genes available for domain experts. The fact that we were able to separate the different spawns by their gene-expression variance over all expressed genes, underlines the importance of spawn specificity, as well as the unexpectedly tight gene-expression regulation in early zebrafish embryogenesis.

**Electronic supplementary material:**

The online version of this article (doi:10.1186/s12864-017-3672-z) contains supplementary material, which is available to authorized users.

## Background

How and when genes are expressed is an important topic in contemporary life-sciences research. Over the past decades, supported by a steep increase in the quality of omics technologies, many new insights on gene-expression regulation were gained with respect to promotors, enhancers, and a large variety of trans acting factors such as, transcription factors and miRNAs. Furthermore, by adjusting for instance rates of transcription, splicing, polyadenylation, nuclear export, ribosome access, and transcription elongation, gene expression can be timed in a precise fashion. A cell thus has a broad repertoire to realize a fine-tuned system of transcription dynamics that allows for localized, synchronized and timely expression of needed transcripts and proteins. Given the fact that the transcription elongation is on average about 2 kb/min [1, 2], genes can be switched on in the order of minutes. Hence, studying transcriptome dynamics can be a challenge, also given de complexity of the cellular mechanisms plus the now widely appreciated additional regulatory roles for RNA other than just being a messenger between DNA and proteins. Scientists studying transcriptome dynamics favour embryogenesis as an experimental system, because it provides a biological system in which RNA plays an important role in many of the regulatory processes. For example, cells can respond to differences in morphogen concentrations and via this realize a highly localized gene expression [3–5].

A well-studied model organism in this context is zebrafish (*Danio rerio*). In the past decade a number of studies has been published that provides much insight in the overall processes of early embryogenesis. These studies entail; global inventories of the zebrafish transcriptome in early embryogenesis [6–9]; dynamics of small [10, 11] and long [9] non-coding RNAs; differential isoform usage pre and post MZT [12]; the start of zygotic gene expression [13, 14]; transcription and translation dynamics during the maternal-to-zygotic transition (MZT) [15]; the role of polyadenylation in RNA stability [14, 16]; the diversity in 3’ UTRs during development with respect to alternative polyadenylation [17, 18]; the dynamics of the methylation state of the zygotic genome [19, 20]; and changing chromatin signatures around MZT [21, 22]. Complemented with extensive in-situ experiments this has resulted in a rapidly increasing insight of the complex transcriptome dynamics in early zebrafish embryogenesis. An aspect that has not been studied comprehensively yet, is the behaviour of transcriptomes in individuals and the behaviour of individual gene expression during embryogenesis. Besides our own study on individual unfertilized zebrafish eggs [23], all of the studies mentioned above were based on pooled samples and quite dispersed time points. The usage of a poly-A+ protocol in some studies sometimes complicates the interpretation of the results, given the fact that many maternal RNAs lack a poly-A tail.

To investigate the transcriptome at the level of an individual, we performed a high-resolution time course gene-expression experiment, in which on average one embryo per minute was measured from late blastula to mid gastrula stages, covering about three hours developmental time. In total 180 individual embryos were interrogated. The start of this series is well after the onset of the MZT, which implies that the vast majority of maternal mRNA in the embryo is replaced by zygotic mRNA. We should, by employing a poly-A+ protocol, be able to gain insight into the embryonic transcriptome program and its dynamics. At the same time, we anticipated that we might be able to pinpoint the exact starting and/or stopping points of individual genes. Given that genes usually operate in pathways that are organized as cascades, knowing consecutive starting points might help reconstruct embryonic pathways. Based on our previous findings in zebrafish eggs [23] that maternal gene expression has a significant mother-specific component, we introduced nine spawns in our experiment.

Our finding here showed an extremely tight embryonic transcriptome program over all individual embryos and we succeeded in defining a clear gene-expression profile for each of the 6,734 expressed genes. This allowed us to classify the gene-expression profiles in ten biologically interpretable classes, including genes that, during this embryonic stage become activated or are switched off. The resolution of our time course was such that we were even able to identify several oscillating genes. Similar to what we observed in our study on unfertilized eggs [23], were the clear spawn-specific effects on gene-expression in many of the expressed genes.

As our aim was to investigate transcriptome dynamics; we did not analyse the behaviour of genes in biological pathways. This should be done by relevant experts in the domains these genes are involved. As we were able to pinpoint the starting and stopping of genes, we feel that our dataset might prove to be a valuable resource for biological experts by their attempts to unravel and reconstruct cellular pathways. Besides the transcriptome data, we also provide an intuitive web-resource for convenient access to the expression profiles of all expressed genes: http://rnabiology.nl/Dr-Browser.html.

## Results

### Experiment set-up

Gene expression in early zebrafish development is quite dynamic, as can be inferred from the many differentially expressed genes (DEGs) found in studies with up to eight developmental sample points covering early embryogenesis [7–10, 14]. Especially from the mid blastula stages onward to the early gastrula stage, characterized by the formation of the yolk syncytial layer and the morphodynamic loss of symmetry followed by progressing epiboly plus cellular differentiation and cell migration [24], many genes are switched on, off, or show other differential expression. Because studies on embryogenesis typically use quite distant sample points, as well as pools of staged embryos [7–9], it is still quite unclear what the expression dynamics, including the inter-individual transcriptome differences, are of each gene in this embryonic phase.

**Fig. 1:**
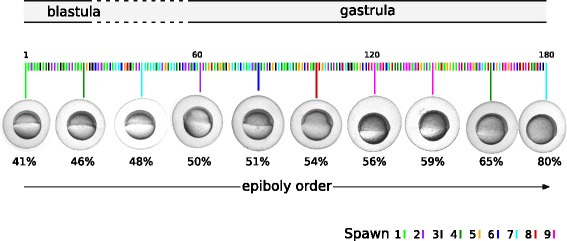
Individual transcriptomes were established of 180 embryos from 9 different spawns (parents) in a developmental stage ranging from late blastula to mid gastrula (approximately 5 to 8 h post fertilization, hpf). The developmental stage was samples with an average of one embryo per minute. Each embryo was photographed and several metrics were recorded (Additional file 1). Here ten embryos are shown with increasing epiboly from left to right

Hence, we designed and executed a high-resolution time course experiment with many individual zebrafish embryos. Using microarray technology, the gene expression of 180 individual zebrafish embryos in a developmental period ranging from 40% epiboly (late blastula, ~5 hours post fertilization (hpf) to 80% epiboly (mid gastrula stage, ~8 hpf) was measured (Fig. 1). Thus, we effectively measured the embryonic transcriptome via, on average, about one embryo per minute during this period. In this developmental period the onset of the zygotic transcription that in zebrafish roughly coincides with the mid blastula transition (MBT) [7, 13, 14, 25] is tightly coordinated with the clearance of maternal transcripts [26] and from the late blastula stage onwards maternal transcripts are reported to be almost entirely cleared [6, 27]. Hence, it is assumed that from late blastula phase onward, embryos primarily contain zygotically expressed transcripts. These mRNAs also should have a regular poly-A tail, which can be short or absent for maternal RNAs. Therefore, we used a poly-A+ protocol for our microarray analysis, thereby focusing on zygotic mRNAs. Moreover, the increasing epiboly provided us with a crude, gradual phenotypic marker of embryonic development. As fertilization took place at different time points due to the multiple pushes by which the mothers released their eggs and because there are significant differences in developmental speed between embryos, it is impossible to use timed samples to obtain a developmentally-ordered sample series. Thus, a crude phenotypic marker was needed for the basic ordering of the embryos based on their developmental progress. The percentage epiboly of a sample was determined by measuring the epibolic distance, from the animal pole to the progressing epibolic border, in the photographs of each embryo at the time of sampling (Additional files 1 and 2). Spawns from nine different zebrafish pairs were used (Additional file 3). All but one microarray (gFG_465103A06, due to surface effects) passed the minimum criteria for quality assessment of microarrays, therefore 179 samples were used in the further analyses.

### Epiboly-expressed genes

Transcriptome analysis typically starts with determining which genes are expressed somewhere during the investigated developmental stage. In our experiment, we identified 12,015 (17%) expressed transcripts and 58,291 (83%) non-expressed transcripts, which translates into 6,734 (30%) Ensembl-defined expressed and 15,938 (70%) Ensembl-defined non-expressed genes (Additional file 4). As expected, epiboly-expressed genes show a significantly higher expression than the non-expressed genes (Additional file 5).

Like reported before, pathway over-representation analyses revealed that gene expression in epiboly is rather focused on basal cellular processes such as splicing, translation or cell cycle (Table 1) and not so much on cellular signalling such as ligand-receptor interaction or cell adhesion. Most over-represented pathways were also found for maternal RNAs as present in zebrafish eggs, which is to be expected given the high overlap with maternally-expressed genes. However, this does not fit well with the generally-accepted theory that maternal RNA is only used for the pre-gastrula development, then is subsequently cleared and replaced by zygotic RNA that is specific for gastrula development.

**Table 1:** Top 10 over-represented KEGG pathways associated with the non-expressed (upper panel) and expressed (lower panel) genes in this time series and their associated p-values. The IE rank column gives the rank of the pathway in an overrepresentation analysis of respectively non-expressed and expressed genes in a study on individual non-fertilized zebrafish eggs [23]

	*p*-value	IE rank
Non expressed associated KEGG-Term		
Neuroactive ligand-receptor interaction	1.36E-38	1
Calcium signaling pathway	9.63E-17	2
Cytokine-cytokine receptor interaction	6.52E-09	4
Cell adhesion molecules (CAMs)	1.17E-05	3
ECM-receptor interaction	8.75E-04	6
MAPK signaling pathway	9.34E-04	8
Chondroitin sulfate biosynthesis	1.39E-03	-
Phosphatidylinositol signaling system	1.58E-03	-
GnRH signaling pathway	4.35E-03	11
Toll-like receptor signaling pathway	4.44E-03	14
Expressed associated KEGG-Term		
Spliceosome	3.83E-26	2
Ribosome	2.87E-25	1
Cell cycle	2.84E-10	11
Oxidative phosphorylation	2.07E-09	4
Proteasome	7.45E-07	17
DNA replication	8.68E-06	10
Ubiquitin mediated proteolysis	3.21E-05	3
RNA degradation	5.37E-05	5
Pyrimidine metabolism	1.43E-04	13
Nucleotide excision repair	3.24E-04	8

### A strict embryonic gene-expression program

**Fig. 2:**
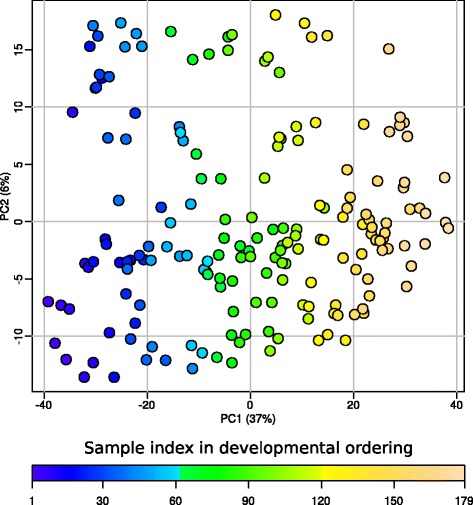
A Principal Component Analysis (PCA) plot of the expression levels of the expressed Ensembl genes. The variance explained by the components is indicated between brackets as percentages. The colouring of the embryos is by developmental order (cf. Materials & Methods, Establishing the Sample Order)

**Fig. 3:**
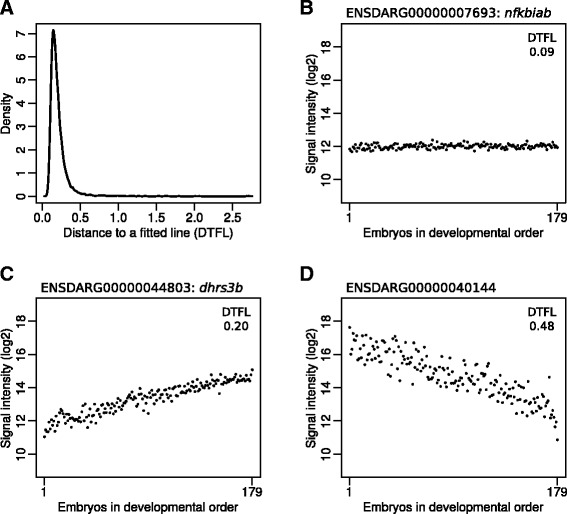
Tight gene-expression regulation in the late blastula and early gastrula stages in zebrafish embryogenesis. **a** Density of the median distance to a fitted lowess line (DTFL) of all expressed Ensembl genes. **b**-**d** Three examples showing the profiles of genes with an increasing DTFL. 98.3% of all expressed genes have a DTFL < 0.48

As measuring the percentage epiboly is quite error prone, for instance due to the orientation of the embryo under the microscope, plus the known fact that the increase of epiboly does not proceed at a constant pace during embryogenesis [25], we applied a bioinformatics procedure to determine the actual developmental ordering of the selected embryos. After data normalization, a developmental order for the embryos was established based on a training subset of continuously *increasing* gene-expression genes. We checked this approach by also establishing a developmental order using a training subset continuously *decreasing* gene-expression genes. We are confident that this approach is effective, because these independently obtained developmental orders are quite similar (Additional file 6). More importantly in both cases all test genes exhibited less variance over the whole developmental course as compared to the basic epiboly-estimated order (Additional file 6). The developmental order aligns almost perfectly with the Principal Component’s first axis (explaining 37% of the variance) in a PCA on all 6,734 epiboly-expressed Ensembl genes (Fig. 2). This implies that during this developmental period gene-expression is quite strictly regulated. It also appeared that the variation (on the PC2 axis, explaining 6% of the variance) decreased at the end of our time course as an indication of even more strict regulation (Fig. 2). The striking strict gene-expression regulation becomes apparent in the gene-expression plots of the ordered embryo samples (Fig. 3b-d). There are many genes that show an extreme consistent gene expression over all embryos (Fig. 3b). Also, most expressed genes show limited variability and small distance to a fitted line (DTFL): 96% ≤ 0.4 DTFL (Fig. 3a). These findings are even more amazing, if one realizes that the depicted samples are individual embryos from different spawns, selected on different days, and analysed individually by a complex laboratory technique. Apparently, there is quite a remarkable precise developmental program in place for gene expression during this embryonic development phase.

**Fig. 4:**
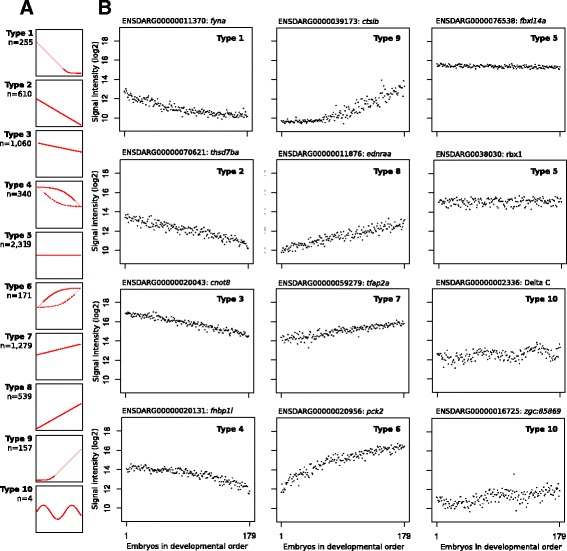
Categorization of gene-expression profiles in the late blastula and early gastrula stages (~5 hpf. to 8 hpf.). **a** 10 profile types are distinguished. n, number of genes. **b** Examples for each type of gene-expression profile

This is underlined by the fact that we were able, by just visual inspection of all gene-expression profiles, to identify four genes that displayed a clear oscillatory gene expression during this embryonic development: *deltaC*, *her1*, *her15.1* and *her7* (Fig. 4b and Additional file 7). All four genes are reported as oscillating genes, although usually in a later embryonic stage [28, 29]. These findings demonstrate that the ordering of embryonic samples it quite good and that the transcriptome organizations, even over embryos and spawns, is extremely regulated.

### Types of gene-expression profiles

Because the observed gene-expression profiles in our high-resolution time-course are quite distinct, it is possible to analyse the transcriptome dynamics by categorizing these profiles. For this, we defined ten overall types of gene-expression profiles, each of which represent a logical and biologically significant behaviour (Fig. 4). As can be expected, these gene-expression profile types blend together, so we applied arbitrary thresholds to three modes of dynamic behaviour: up, down or oscillatory gene expression; to continuous or discontinuous behaviour; and to the observation of ‘starting’ or ‘stopping’ of gene expression in this developmental stage.

Although this study covers about 3 hours of embryonic development and apart from the four oscillatory genes (Fig. 4, type 10), we did not encounter any gene that clearly peaked within this period, neither up nor down. This means that during early gastrulation gene-expression in general changes rather gradually. Also, about one-third of the expressed genes showed a constant expression profile (type 5), whereas one-third showed an increasing and one-third a decreasing gene expression. Within the latter groups there are several genes that were clearly switched-on (type 9; 2.3%) or switched-off (Fig. 4, type 1; 3.8%) during this embryonic stage. However, of the genes with changing expression, the gradually up (type 8) or gradually down (type 3) were most prevalent. If we examine the rate of change, it is clear that the starting (type 9) and stopping genes (Fig. 4, type 1) have the steepest slope, on average respectively 1.33 FC/hour (fold change per hour) and 1.28 FC/hour (Additional file 8) and the lowest intensities (Additional files 9 and 10).

For the starting (type 9) and stopping genes (type 1) the expression starting and stopping points are within this developmental stage. Overall is seems that there are relatively more genes starting in the first half of this phase and more stopping in the second half (Additional file 11). However, there are also many genes that have a considerable expression at the beginning of this phase. Given that we know the rate of gene-expression increase (type 7), as well as the first expression level, we can extrapolate to a predicted starting point (Additional file 11). This approach would place the far majority of gene-expression starting points before the moment of spawning. Expression of Type 5 genes does not change during this stage; here we used the maximum positive gene-expression rate we found in this study, to predict the starting point for the type 5 genes (Additional file 11). Again many gene-expression starting points are calculated well before spawning. So either the gene-expression rates in the earlier stages of embryogenesis are much higher than in the investigated stage, or there is massive polyadenylation of maternal mRNA that result in Poly-A+ mRNA.

### Mother-specific gene-expression variation

**Fig. 5:**
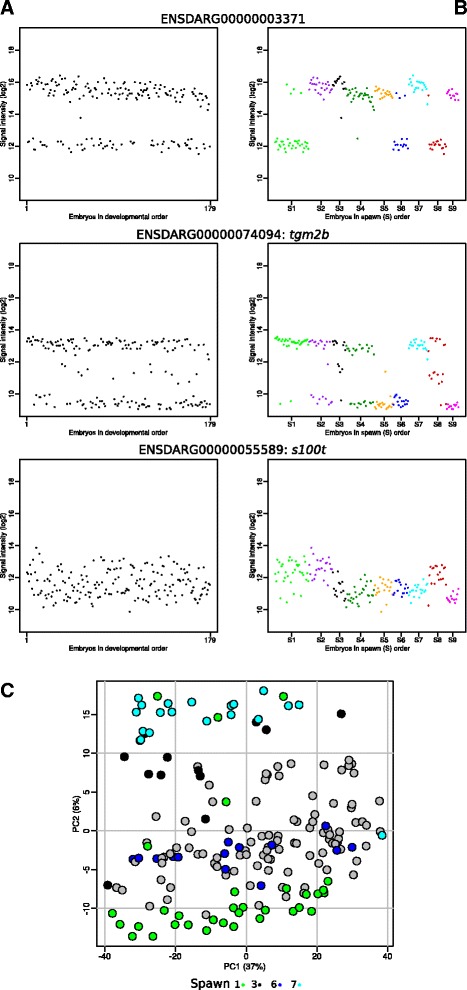
Multilevel gene-expression behaviour is highly spawn specific; examples of multilevel gene expression. **a** examples of genes with a two-level (*upper plot*), a three-level (*middle plot*) and a highly variable (*lower plot*) expression. **b** the same genes are ordered first as spawn then by developmental order. **c** the same Principal Component Analysis (PCA) plot of the expression levels of the expressed Ensembl genes as in Fig. 2, but with a colour scheme for four spawns as indicated, while the remaining five spawns are marked by in *grey*. Additional file 14 provides a PCA plot with all spawns marked with a different colour

During the analysis of our experiment, we noticed that a minority of the genes showed a clear multilevel expression profile (88 two-level and 5 three-level genes, Additional file 12). These genes, irrespective whether they have an overall increasing, decreasing on unchanged expression, are expressed at two or three expression levels, seemingly random throughout the time series (Fig. 5a). This seems an unlikely exception to the observed strict regulation of embryonic gene expression. It rapidly became clear that this phenomenon was related to spawns (i.e., mother and/or father) (Fig. 5b). Although there is a clear relation to spawn, it is not absolute as sometimes embryos from one spawn show different gene expression levels. It is obvious that maternal, and presumably paternal factors strongly influence the embryonic expression of these genes. Moreover, close inspection reveals that this spawn-specific effect might be present in many more genes, as also genes without an obvious multilevel gene-expression profile exhibit this phenomenon (Fig. 5b, *ENSDARG0000055589*, Additional file 13). The importance of this effect became clear after we labelled Spawn in the PCA of all expressed genes: all spawns show a strong tendency to group together and some spawns almost completely separate on the PCA-2 axis (Fig. 5c, Additional file 14). This means that in addition to a tight overall gen-expression regulation, embryos have a spawn-specific regulation.

## Discussion

In this study we set out to investigate the transcriptome dynamics of early zebrafish embryogenesis with a comprehensive experimental set-up that interrogated individual embryos about every minute during the circa 3 h period during epiboly from late blastula to the mid gastrula stage. We did so, because most studies into (zebrafish) embryogenesis are typically using pools of staged embryos plus quite dispersed time points, and we reasoned that a high-resolution time course could provide much insight into the true dynamics of the individual genes of the embryonic transcriptome. The fact that we were able to identify several oscillating genes shows the value of our approach. We set-out to prove that it should be feasible to pinpoint the starting and stopping points of individual genes. We were able to do so for the genes that started or stopped during the epibolic stage. Hence it is also possible to define consecutively starting, or stopping genes, which should be of great help reconstructing cellular processes and molecular pathways. In this study, we did not aim to unravel the role of individual genes nor that of the molecular pathways in zebrafish embryogenesis. This should be done by the appropriate experts on each molecular process. To facilitate them, we have not only uploaded our entire dataset to NCBI’s Gene Expression Omnibus with accession number GSE83395 (http://www.ncbi.nlm.nih.gov/geo/query/acc.cgi?acc=GSE83395), but also set-up a web resource in which experts can conveniently investigate the expression profiles of the genes of their choice: http://rnabiology.nl/Dr-Browser.html in combination with the expression in zebrafish oocytes from our previous study [23]. To gain perspective, it is important that by interpreting individual genes, one is aware what the common transcriptome dynamics are for all genes during the same developmental stage.

To put our study in context, we compared our finding with three other studies: Junker et al. [30] on genome-wide RNA tomography, which has one sample point (shield stage) that overlaps with our time-course; Aanes et al. [7] of which the 5.3-hpf sample point overlaps with the start of our time course; and our own study Rauwerda et al. [23] concerning maternal RNA in zebrafish eggs. In general, the findings of the current study were in line with the previous studies (Additional file 15). The genome-wide RNA tomography by Junker et al. provides an exciting opportunity to link the temporal gene expression data to embryonic location. Given that genes primarily function in the context of a cellular network this would allow the decomposition of the temporal transcriptome data from multicellular embryos to clusters of genes that operate together in pathways or cellular mechanisms. It was possible to look into this, as the RNA tomography data overlapped with one embryonic developmental sample point (shield stage) with our data. A similar number of expressed genes (7,358), with 70% overlap in our study were found in the overlapping shield-stage samples from Junker et al. (2014) (Additional file 15-B). Hence, we clustered the tomography data in 16 spatial gene-expression patterns and analysed which pathways were overrepresented in these spatial expression clusters. Genes of the KEGG Pathway “Ribosome”, which also is overrepresented in our time course experiment, were indeed found to be overrepresented in the same spatial expression cluster (Additional file 16). However, for many other overrepresented pathways from our expressed genes, we failed to find them back using the tomography data. We feel that the “Ribosome” pathway probably succeeded as these genes are highly expressed. Unfortunately, although the overall data of the tomography study is excellent, the data of the overlapping shield sample point is rather noisy compared to the other sample points, with only a few distinct gene-expression patterns. So, we believe that new tomography data could help decompose the whole-genome temporal transcriptome data, which could support the discovery of new pathways or new genes into known pathways.

In another comparison, also a high-percentage overlap (77% relative to our study) was found with the Aanes et al. (2011) study with also a similar relative gene expression for the overlapping genes per Aanes-defined gene-expression clusters in both studies (Additional file 15-D).

A comparison of the current results with our previous study [23] on maternal RNA revealed that 87% of the expressed genes were also found to be present as maternal RNA in egg (Additional file 15-F). Given the assumptions that in epiboly almost all maternal RNA is cleared and maternal RNA primarily functions in the pre-gastrula development, this is in line with the finding of Lee et al. (2013) that 74% of the early-zygotically transcribed genes still had a maternal contribution. There is however, no clear relation between the expression level for each gene as observed in eggs and that at the late blastula stage (Additional file 15 E-F). There is also no correlation between the genes that showed a distinct mother-specific effect in egg expression and a spawn-specific effect in epiboly expression. Altogether, the clearance and subsequent transcription of these genes seems a complicated aspect of the MZT.

The most impressive result of the high-resolution embryonic time course is the extremely tight regulation of gene expression, even over different embryos from different spawns. A technical conclusion is that pooling of embryos for experimentation poses no problem, if the staging of the individual embryos is done properly. The observed spawn-specific differences will obviously be lost, if eggs from different spawns are combined. At the same time, if spawns are not evenly distributed between pools, spawn-specific gene-expression differences might lead to false positive differential gene-expression conclusions.

More importantly, the presence of such a tightly regulated embryonic program for transcription, which took us by surprise, signals a rather robust system that in essence is resilient to the differences at many cellular levels that must occur between individuals. Notably, even though we classified the expressed genes into ten different types, virtually each individual gene has its own unique expression profile with respect to intensity, change and variability.

Our unique experimental set-up also allowed us to identify the starting and stopping points of gene-expression at an omics scale. Only several hundreds of genes appeared to be switched on or off during this period of embryogenesis, which seems to be quite a quite limited number given the multitude of developmental processes that occur. We must however consider that even though we found many genes expressed, we might miss out on several important developmental regulating genes as they could be below the detection limit of our experimental technique due to low gene expression or only be expressed just in a small fraction of the embryo cells. Regardless, if we accept the premise that maternal RNA is cleared previous to the gastrula stage, this means that, except for the identified 157 starting genes (type 9), all zygotic gene expression has to start prior to this embryonic stage. Although for some genes the maternal and zygotic contributions can be deconvoluted on the basis of their 3’UTR [17, 18], for many genes, polyadenylation of maternal transcripts and zygotic gene expression are confounded. So the expression levels and differences for each individual gene are a net result of those poly-A+ generating mechanisms, as well as specific RNA-degrading mechanisms. Collectively, the gene-expression changing rates are quite modest, on average about 1.3 FC/h. They do not explain the observed gene-expression levels as extrapolation of present genes predicts for most of the expressed genes a starting point well before fertilization. As, besides the oscillating genes, no genes with peaking gene-expression were found, we conclude that gene expression in this embryonic stage is unexpectedly gradual, which might be the sign of a complex, strictly regulated system.

Our final observation with respect to transcriptome dynamics concerns the spawn-specific gene-expression effects. Much alike we observed in our oocyte study, we detected several genes with a substantial spawn-specific difference. Moreover, it appeared that this property might be present in the majority, if not all genes, albeit often with extremely small differences. As the eggs in our previous study were unfertilized, we could identify this effect to be mother-specific. In the present study it is undoubtedly a mixed effect of several maternal and paternal epigenetic factors that collectively produce these differences in gene expression. This would also explain why in eggs multilevel expressed genes are almost always expressed at one level in all eggs of one mother, whereas in this study embryos from one spawn often show multilevel gene expression. Although we were unable to model all the gene-expression differences between the embryos, it is clear that given the strict regulation of the transcriptome, they will be related. As such, the spawn-specific differences provide an additional layer of information that could support the understanding of transcriptome regulation in embryogenesis.

## Conclusions

In our high-resolution time-course study, we determined that gene-expression in zebrafish embryogenesis from late blastula to mid-gastrula is tightly regulated and gene-expression changes are in general gradual with small fold changes. The fundamental processes that take place during this developmental period at the transcriptome level involve ~700 genes that are switched on and ~900 genes that are switched off. On top of, that some 1,450 genes show increasing and 1,400 genes show decreasing gene-expression, while 2,300 genes show no change in gene-expression. The resolution of the experiment was so high, that we were able to clearly identify four known oscillating genes. Additionally, it allowed us to precisely establish the expression starting and stopping points of several hundred genes. Furthermore, we found 93 genes that show an obvious multilevel expression profile over the embryo’s. The different expression levels of these genes could be linked to the spawns the embryos originated from. The fact that we were able to identify the different spawns by their gene-expression variance over all expressed genes, underlines the importance of spawn specificity, as well as the tight regulation in different individuals. Our unique experimental set-up of whole-transcriptome analysis of 180 individual embryos, provided an unparalleled in-depth insight into the dynamics of early zebrafish embryogenesis that we also made available to the scientific community.

## Methods

### Zebrafish embryos

Zebrafish were handled and maintained according to standard protocols (http://ZFIN.org). The local animal welfare committee (DEC) of the University of Leiden, the Netherlands specifically approved this study. All protocols adhered to the international guidelines specified by the EU Animal Protection Directive 86/609/EEC. Embryos were obtained by placing a female and a male, both of genotype ABTL, overnight in a tank separated by transparent, but watertight division. Approximately 1 h after the start of the light period the division was removed after which spawning and fertilization took place, generally in one or more pushes. Embryos were retrieved by transferring both animals to a different tank and sieving the remaining water. In total from 9 pairs, embryos were obtained (labelled Spawn, Fig. 1) that were retrieved on 5 separate days. Embryos were maintained at 28.5 °C in egg water (60 μg/ml Instant Ocean sea salts). In total 180 embryos were processed, from approx. 5 h post fertilization (hpf) to 8 hpf. During this period, embryos were, while kept at a temperature of 28.5 °C, one by one taken out of the medium and positioned along the anteroposterior axis under a stereo microscope (Leica MZ16 FA) in a 1.5% agarose gel (Sigma Aldrich IX-A) composed with egg water. After an image was taken the embryo was transferred to a 1.5 ml tube and exactly 30 s after the photograph was made the embryo was snap-frozen in liquid Nitrogen.

### Image processing

First from the metadata of the photograph a conversion factor of pixels to μm was determined. The embryo size, roundness, perimeter, height, width and epiboly were determined from the images using GraphPad Prism 6 software with which also the pixels belonging to the embryo and yolk were identified with a masking procedure (Additional file 1). Next, the height of the embryo plus yolk was determined as the diameter of the form in the animal-vegetal direction, the width as the diameter in the direction perpendicular to the direction in which the height was measured and the percentage epiboly was determined as the fraction of the epibolic distance and the height (Fig. 1b). Perimeter length was determined and roundness was calculated as the ratio of 4π*Area over the square of the perimeter. For each metric the maximum relative change was determined (Additional file 1). All individual images, rotated as to position the anterior-posterior axis of the embryo onto the x-axis of the image are given in Additional file 2.

### RNA extraction, amplification and labelling, hybridization, scanning

RNA was extracted from single embryos using the procedure described in (de Jong et al. 2010). The amount of RNA per μl was measured on the Nano- Drop ND-1000 (Thermo Scientific; Additional file 3). RNA integrity was assessed with the BioAnalyzer (Agilent Technologies) using the RNA pico 6000 kit (Agilent Technologies). Amplification and labelling of RNA was done in an identical fashion as described in [31] in a one-round mRNA amplification per zebrafish embryo using the Amino-allyl MessageAmp II kit (Applied Biosystems). Test samples were labelled with Cy3 and a reference sample was labelled with Cy5. The reference sample was made by pooling equimolar amounts of RNA from test samples and is mentioned here for completeness only, since in the analysis a one dye approach has been applied. The yield and CyDye incorporation were again measured with the NanoDrop ND-1000 (Additional file 1). The hybridization cocktail was made according to the manufacturer’s instructions (Nimblegen Arrays User’s Guide). Microarrays were designed using the approach described in [32] albeit with a few modifications (Additional file 4) and were produced as Nimblegen 135 k arrays. The array design has been submitted to NCBI's Gene Expression Omnibus with accession number GPL22016 (http://www.ncbi.nlm.nih.gov/geo/query/acc.cgi?acc=GPL22016). Hybridization took place for 17 h at 65 °C. In order to avoid confounding with spawn, samples have been randomized with respect to labelling batch and hybridization chamber. The microarrays were scanned in an ozone-free room with a DNA microarray scanner G2565CA (Agilent Technologies). Data was retrieved with Feature Extraction Software version 9.5.3 (Agilent Technologies).

### Data pre-processing & normalization

The quality of the microarray data was assessed via multiple quality-control checks, i.e., visual inspection of the scans, testing against criteria for foreground and background signals, testing for consistent performance of the labelling dyes, checking for spatial effects through pseudo-colour plots, and inspection of pre- and post-normalized data with box plots, ratio-intensity (RI) plots and PCA plots. Handling, analysis and visualization of all data was performed in R (http://cran.r-project.org/) using the Bioconductor packages affy (1.48.0), limma (3.26.9) and maanova (1.40.0) [33]. The Cy5 reference-sample channel was solely used for quality control and further analyses were performed with the Cy3 data only. Log2 transformed Cy3 data was subjected to a procedure, similar to the one described in [34], in which probes were identified as expressed or non-expressed probes. Here a log variance cut-off value of −2 was used to separate low and high variant probes and the per microarray cut-off for the likelihood of a probe to be expressed was set to 20%, thus avoiding too many false negatives. Hence, in this step for each array, individual probes were called ‘present’ or ‘not-present’. Next, we defined that a probe to be truthfully expressed must be called ‘present’ in at least 6 samples in one moving window of 30 with the samples ordered in epiboly stage to avoid false positives. Next all ‘present’ probes were quantile normalized and collapsed into transcripts using the R Bioconductor affy package. In the procedure no background removal was applied. Quantile normalization in this data set appeared to outperform other much used normalization approaches in a comparison using spike-in controls on the microarray (Additional file 17). Finally, when applicable, transcripts were merged into genes, using Ensembl (release 79) gene identifiers on the Zv9 assembly by calculating the mean of all transcripts that belong to a gene. Hence, 42% of the expressed genes was queried by 1 probe, 30% by two probes, 22% by three probes and 6% was queried by more than 3 probes.

### Establishing the sample order

In this experiment, on average one sample (embryo) per minute was analysed. Because in each spawn fertilization took place at different time points due to the multiple pushes by which the mothers released their eggs, the exact time of fertilization could not be established at a resolution below 15 min. We therefore first looked if a crude phenotypic marker would allow us to roughly order the embryos along developmental time. The metric with the largest relative change (percentage epiboly) was chosen as initial metric to roughly order the individual embryos in time. However, at around 50% epiboly, the rate at which the blastoderm extends over the yolk stalls, which makes epiboly an imprecise metric for the ordering of this entire time course. We assume that embryos with a low epiboly can indeed with confidence be positioned at the start of the time course of this experiment and, likewise, that embryos with a high epiboly are at the end of this time course. Thus, we selected 10 samples with the lowest epiboly and 10 samples with the highest epiboly. In these samples we looked for transcripts with a low expression level (mean log intensity below 12) in the low epiboly samples and for transcripts with a high expression level (mean intensity above 13) in the high epiboly samples. Next, we took the intersect of both sets and calculated the mean intensity in this intersect of 97 transcripts with increased expression for all 179 samples. The developmental order of the samples was then determined by ordering all samples according to the mean intensity of these intersecting transcripts from low to high. The ordering was tested to evaluate the overall variance of the expressed genes that were not used to order the samples. The whole procedure was tested by reversing the approach for transcripts with a high expression level (mean intensity above 13) in the low epiboly samples and for transcripts with a low expression level (mean log intensity below 12) in the high epiboly samples. The 121 transcripts with decreasing expression were used to order the samples and the remaining genes were evaluated for their overall variance in gene expression.

### Calculation of tightness of gene regulation

Given this developmental order we wanted to know how much individual embryos deviate from the gene expression trend in this period. For this we fitted a lowess line through all samples using the lowess function of R with a smoother span of 2/3. Per gene we calculated the median distance of each sample to this fit as the Distance to the Fitted Line (DTFL).

### Gene categorization

All Ensembl genes were assigned to exclusive gene-expression dynamics categories, which we call ‘types’. These 10 types are defined by three modes of behaviour: up, down or oscillatory gene expression; continuous or discontinuous behaviour; identification of switching-on or switching-off events (‘starting’ and ‘stopping’ genes) in this developmental stage. Oscillatory genes were checked with literature [28, 29]. We inspected all profiles by eye and because this appeared to be both more sensitive and selective than using a computer-based procedure, we added 4 genes to the oscillatory type by eye.

Up trends and down trends were identified by fitting a linear model through the profile using the R lm function. All profiles with an angle > 0.1° were categorized as ‘increasing’, all profiles with an angle < − 0.1° were categorized as ‘decreasing’.

The continuous and discontinuous trends were identified by comparing the linear fit from above with lowess fit. The lowess fit was made using the R lowess function with the default parameters (smoother span of 2/3). For each embryo the absolute distance to each fit was determined and per fit the average of the distances over all embryos was calculated. Finally, per gene the ratio of these two averages was taken. Genes with a ratio larger than 0.95 were categorized as continuous, genes with a ratio of 0.95 or smaller were categorized as discontinuous.

Starting and stopping genes were identified with all ‘increasing’ and ‘decreasing’ genes respectively. To call a gene starting we required that less than 7 out of the 15 first embryos were queried by probes that were called ‘present’; to call a gene stopping we required that less than 7 out of the 15 last embryos were queried by probes that were called ‘present’. By looking at the probes we consider the gene expression of individual embryos and can exclude the information on the developmental order that is present in the profile and in the ‘expressed’ calling of a gene. The tracing back from the gene to the probe level was done by first by choosing the transcript of that gene with the highest expression value and then choosing the most 3 prime ‘present’ probe of that transcript as the probe that reports the presence of a gene.

### Identification of starting and stopping points

Identification of starting and stopping points of genes was carried out on increasing starting and decreasing stopping genes, respectively (type 8, 9 and type 1, 2, Fig. 4). We devised a method that utilizes the observed behaviour that for a stopping gene the rate of decrease is lowest near the stopping point or, respectively for a starting gene the rate of increase is lowest when the gene starts. In other words, the starting and stopping gene expression profiles have a concave form. By defining the respective increase or decrease rate of a gene as the gradient that is determined by the embryos that lie between a minimum and the maximum deviation from the linear fit of all expression values and by inferring the ‘off’ level by probes that are labelled ‘not present’ the following geometric procedure allowed us to determine the starting and stopping points. First, for the starting and stopping genes a lowess fit (lof1), using the R lowess function with the standard parameters and a linear fit (lf1) are made on all samples (the red respectively the green line in Additional file 18) and the points at which these two lines intersect is determined. Next, for each sample the absolute difference of the sample’s expression value to the lowess fit and to the linear fit is calculated. These differences are ordered and the indices (sample number in developmental order) of the two smallest differences are identified (the two grey lines in Additional file 18). Also the index with the largest absolute difference that lies between the two former indices is determined (index L1, indicated by a solid blue line in Additional file 18). Next, a second linear fit is made (lf2); for stopping genes only the embryos with an index ranging from 1 to L1 are used; for starting genes this fit is made using the embryos ranging from L1 until index 179. Then, the intensity expression value at respectively the start or the end of the time course was determined by the median of the probe intensities of the ‘not-present’ probes in the first respectively the last 15 embryos (embryos marked with an asterisk in Additional file 18) and a horizontal line at this intensity level is drawn. Finally, starting and stopping points are calculated at the intersection of this horizontal line and lf2 by the minimum absolute difference between the 179 extrapolated point of the fitted lines. In order to ensure an actual starting or stopping behaviour this minimum was required to be smaller than 0.05 (stopping and starting points are indicated by the dotted blue line and by the labels ‘stop’ and ‘start’ in Additional file 18).

### Identification of multilevel genes

Identification of multilevel genes was done by firstly selecting the genes with a log2 variance higher than −0.1. To these genes a 2-means clustering was applied using the R kmeans function. The ratio of the within-cluster sum of squares and the between-cluster sum of squares was calculated and genes with a ratio smaller than 0.3 were selected for further analysis. Furthermore, the requirement was set for each cluster to span more than one third of the developmental time in this study. Finally, a visual inspection was done to discard apparently false positive genes that show rather temporal dynamics than a multilevel behaviour.

### Tomography data analysis

Data from the shield samples in the tomography study of [30] was compiled as one matrix with rows for each gene and with columns representing the slices in the animal-vegetal pole direction (50 slices), left right direction (56 slices) and ventral-dorsal direction (49 slices). The rows were clustered using a 16-means clustering with the R kmeans function. Next, the resulting spatial expression clusters are subjected to an overrepresentation analysis on KEGG pathways using the David Bioinformatics Resources 6.7 [35].

## Additional files

Additional file 1: Image metrics of all samples (embryos). For a description of the metrics cf. Material & Methods, paragraph “Image Processing”. (XLSX 25 kb)

Additional file 2: Images of all 179 embryos positioned in the animal pole - vegetal pole direction. (PDF 19069 kb)

Additional file 3: Sample information. Array = array identifier, ID = array index; Dye = Cy5 or Cy3; Slide = slide identifier; Location = location of array on slide; Day = Hybridization day; HybStation = Hybridization chamber; Epiboly = measured epiboly in percentage; Image = image index; AmpBlock = amplification block; Spawn = spawn the embryo belongs to; RIN = RIN value; Yield..ng. = yield in ng. (XLSX 24 kb)

Additional file 4: Number of expressed and non-expressed Ensembl genes and Vega, Refseq and Unigene genes. (XLSX 9 kb)

Additional file 5: Intensity distribution of the 6,734 expressed (red) and 15,938 non-expressed (green) unique Ensembl defined genes. (PDF 23 kb)

Additional file 6: Using two different training sets to establish developmental order. Page 1, A - Samples ordered by a training set of genes with continuously increasing gene-expression vs. samples ordered by a training set of genes with continuously decreasing gene-expression. Samples are colored by epiboly. B - *ENSDARG00000016725* ordered according to epiboly (bottom) and in developmental order (top); Page 2–123: test set of 121 continuously decreasing genes ordered by a training set of 97 continuously increasing genes. Page 124–221: test set of 97 continuously increasing genes ordered by a training set of 97 continuously decreasing genes. (PDF 1403 kb)

Additional file 7: Information on the Ensembl expressed genes. Ensembl_id = Ensembl identifier; ProbeID = probe set identifier; Designed_on = identifier on which the probe set was designed; REFSEQ = Refseq identifier; refseq.symbol = Refseq symbol; ENTREZ = Entrez identifier; refseq.descr = Refseq description; Unigene = Unigene identifier; unigene.symb = Unigene symbol; Unigene.descr = Unigene description; zfin = ZFIN identifier; zfin_descr = ZFIN description; probeNumber = number of probes in the probe set; Type = type (cf. Results); StartingPoint = starting point of gene expression; StoppingPoint = stopping point of gene expression; Angle = angle of expression rate corrected for starting and stopping position; FoldChange.per.hour = fold change per hour corrected for starting and stopping position. (XLSX 925 kb)

Additional file 8: Boxplots per type of absolute expression changes of genes over time, expressed as fold changes per hour. (PDF 37 kb)

Additional file 9: Intensity distributions per type. A) Left panel: top 10 highest gene expression intensities per type. Right panel: top 10 genes with the largest gene expression rates (expressed in angles) type. B) Scatterplot of gene expression rates, expressed as angles versus intensity. In each plot the genes belonging to the type are highlighted. (PDF 520 kb)

Additional file 10: Boxplots of median expression intensities per type. (PDF 11 kb)

Additional file 11: Starting and stopping points. A) Histogram of starting and stopping points in developmental time for genes belonging to type 1 and 9. B) Histogram of extrapolated starting points for type 5 and 7 genes. For type 7 genes the starting point was calculated from the observed expression rate for each gene. For type 5 genes we took the maximum positive expression rate we found in this experiment (2.98 FC/hr for *ENSDARG00000095866*) to calculate the starting point. The approximate time of spawning is indicated by a dashed line, the start of the time course in this experiment lies to the right of 5 hpf. (PDF 24 kb)

Additional file 12: Multi-level genes. On each page the left panel displays the expression intensities of a gene in developmental order, the right panel displays the same gene plotted in developmental order ordered per spawn. (PDF 354 kb)

Additional file 13: First two principal components of the intensity matrix of the expressed Ensembl genes. The variance explained by the components is indicated between brackets as percentages. Each embryo is colored by spawn as indicated. (PDF 26 kb)

Additional file 14: High-variance genes of type five with a log2 variance > −1. On each page the left panel displays the expression intensities of a gene in developmental order, the right panel displays the same gene plotted in developmental order ordered per spawn. (PDF 181 kb)

Additional file 15: Comparison of this study with the zebrafish tomography study of [30] *(study T)*, with the study on the early embryonic zebrafish transcriptome of [7] *(study EET)* and with the study on individual zebrafish eggs from five different mothers of [23] *(study IE)*. A) Scatter plot of log2 read counts in the shield stage of study T vs. the log2 expression intensity in this study of genes that are expressed in both studies. Read counts are calculated as the log2 of the sum of counts in all slices in study T and from this study the samples 85 to 95 in the developmental order are taken. The correlation between the two sets is 0.64. B) Venn diagram on Ensembl genes in study T and expressed Ensembl genes in this study. C) The distributions of log2 intensity values of Ensembl genes in this study (upper panel) that are per category in common with the Ensembl genes from study EET; in the lower panel for the same genes the distribution of read counts in the 7 categories of expression clusters in study EET are displayed. D) Per category comparison of number of expressed genes in this study and study EET. E) Scatter plot of log2 expression intensity in study IE (X-axis) and this study (Y-axis) of genes that are expressed in both studies. For this from study IE the median of all 24 eggs was taken and from this study the median of the first five samples was taken. The correlation between the two sets is 0.50. F) Venn diagram on study IE this study. Compared are expressed Ensembl genes from both studies. (PDF 377 kb)

Additional file 16: Temporal and spatial behavior of genes in 27 KEGG pathways. Each page displays three plots. The left plot shows the temporal expression in log2 intensity in our time course experiment for a pathway ordered in developmental time. The middle plot shows the spatial expression pattern in the tomography set [30] in log2 read counts. The three peaks represent the three axes in the embryo: respectively the animal-vegetal, left-right and ventral-dorsal axes. The right plot shows the number of genes of the pathway that belong to a spatial expression cluster. The spatial expression cluster 9 is the only spatial expression cluster in which an overrepresentation was found (KEGG pathway “Ribosome”). (PDF 1595 kb)

Additional file 17: Spike in controls were added to the samples and were hybridized to specific probes on the array. Based on the application of 3 widely used normalization methods and the resulting variance we decided to apply quantile normalization in this analysis. (PDF 13 kb)

Additional file 18: Example of the determination of a stopping point and a starting point. For an explanation see Methods, paragraph “Identification of starting and stopping points”. lf1 = linear fit on all samples; lof1 = lowest fit on all samples; grey lines indicate intersect of lf1 and lof1; L1: embryo with the largest difference between lf1 and lof1; for stopping genes lf2 = linear fit of L1 to 179; for starting genes lf2 = linear fit of 1 to L1; embryos marked with an asterisk: ‘not present’ probes in the last (stopping) 15 respectively in the first (starting) 15 embryos; horizontal black line: median probe intensity of embryos marked with an asterisk; dashed line: intersect of lf2 and the horizontal black line indicating the stopping respectively the starting point. (PDF 131 kb)

## Abbreviations

CAM: Cell adhesion molecules
DEC: Dierexperimentencommissie (Animal Welfare Committee)
DEG: Differentially Expressed Genes
DFTL: Distance to the Fitted Line
hpf: Hours post fertilization
lf: Linear fit
lof: Lowess fit
MBT: Mid Blastula Transition
MZT: Maternal-to-zygotic Transition
PCA: Principal component analysis
RI plots: Ratio-intensity plots
