# Supplementary figures and images for: Transcriptome dynamics in early zebrafish embryogenesis determined by high-resolution time course analysis of 180 successive, individual zebrafish embryos
Source: BMC Genomics. 2017 Apr 11;18:287. doi: 10.1186/s12864-017-3672-z (PMC5387192; doi:10.1186/s12864-017-3672-z)

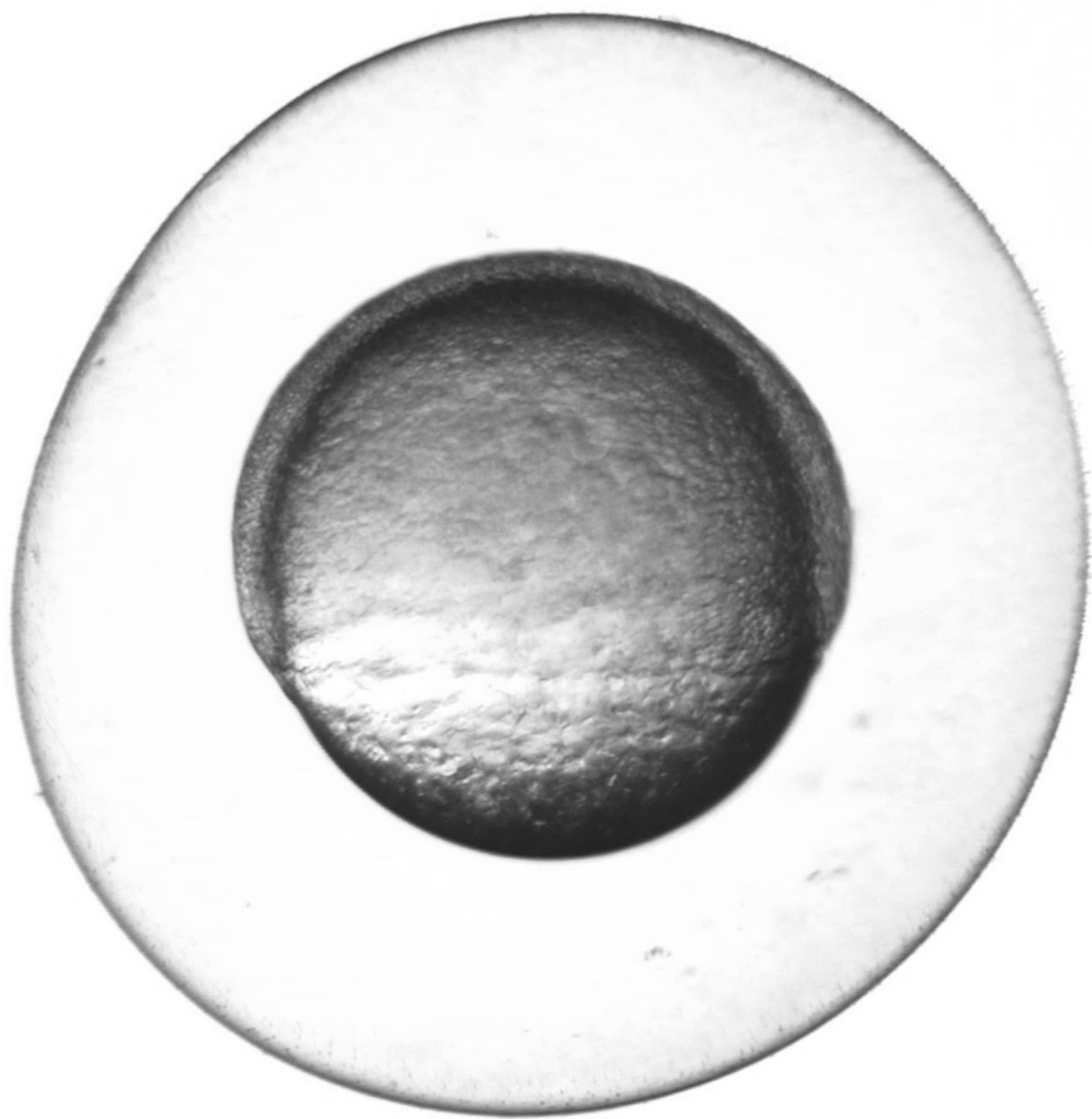

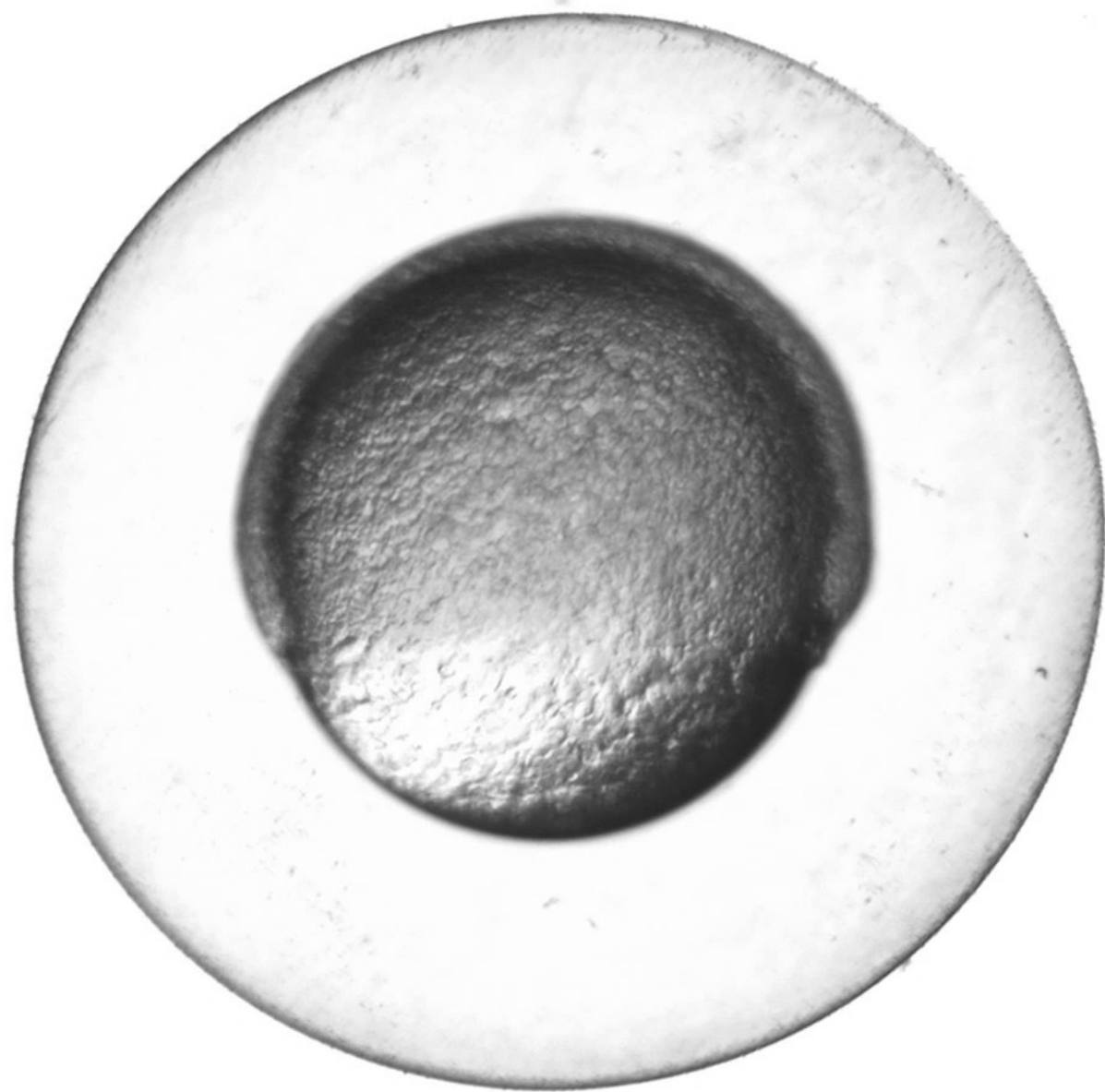

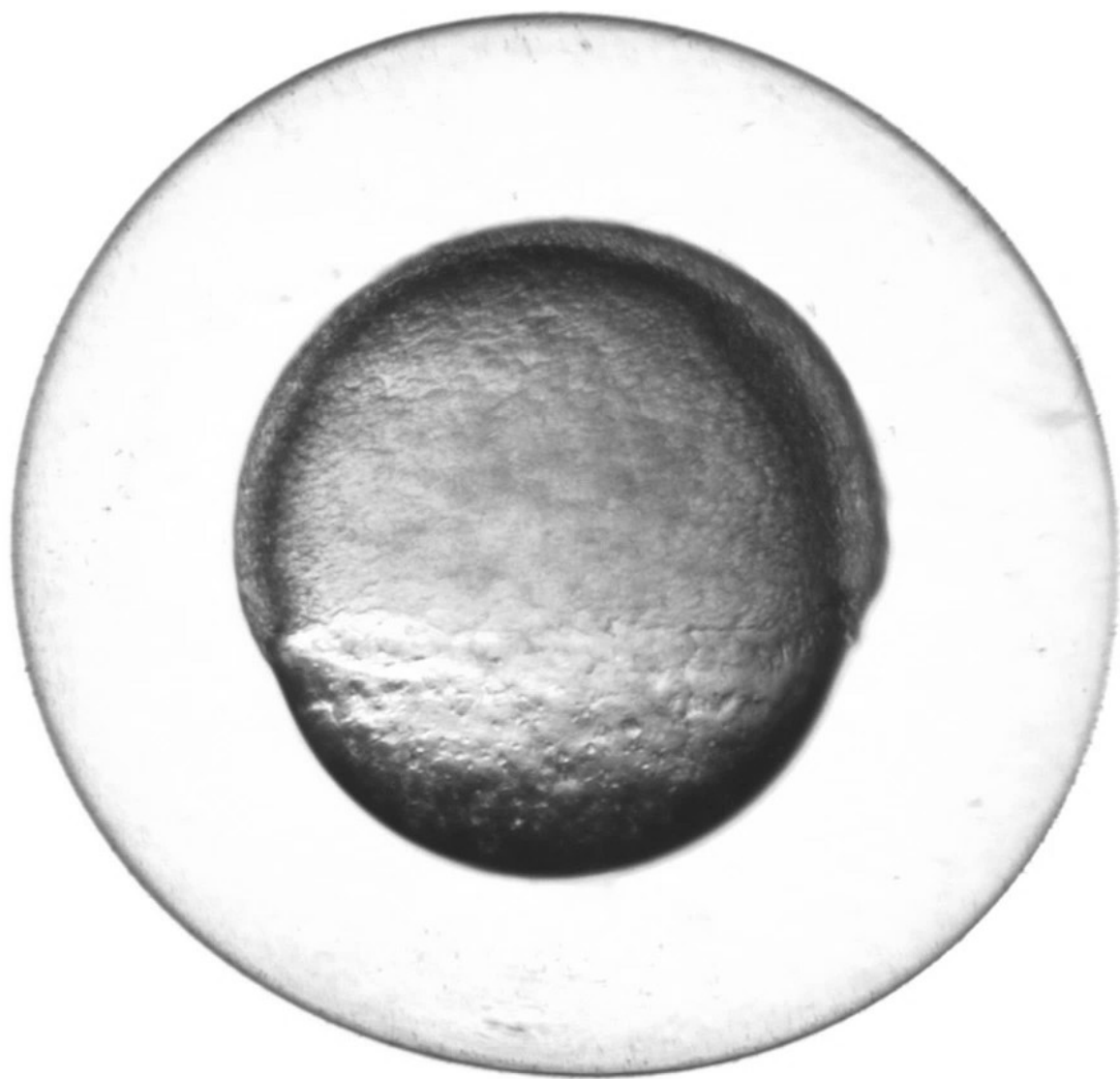

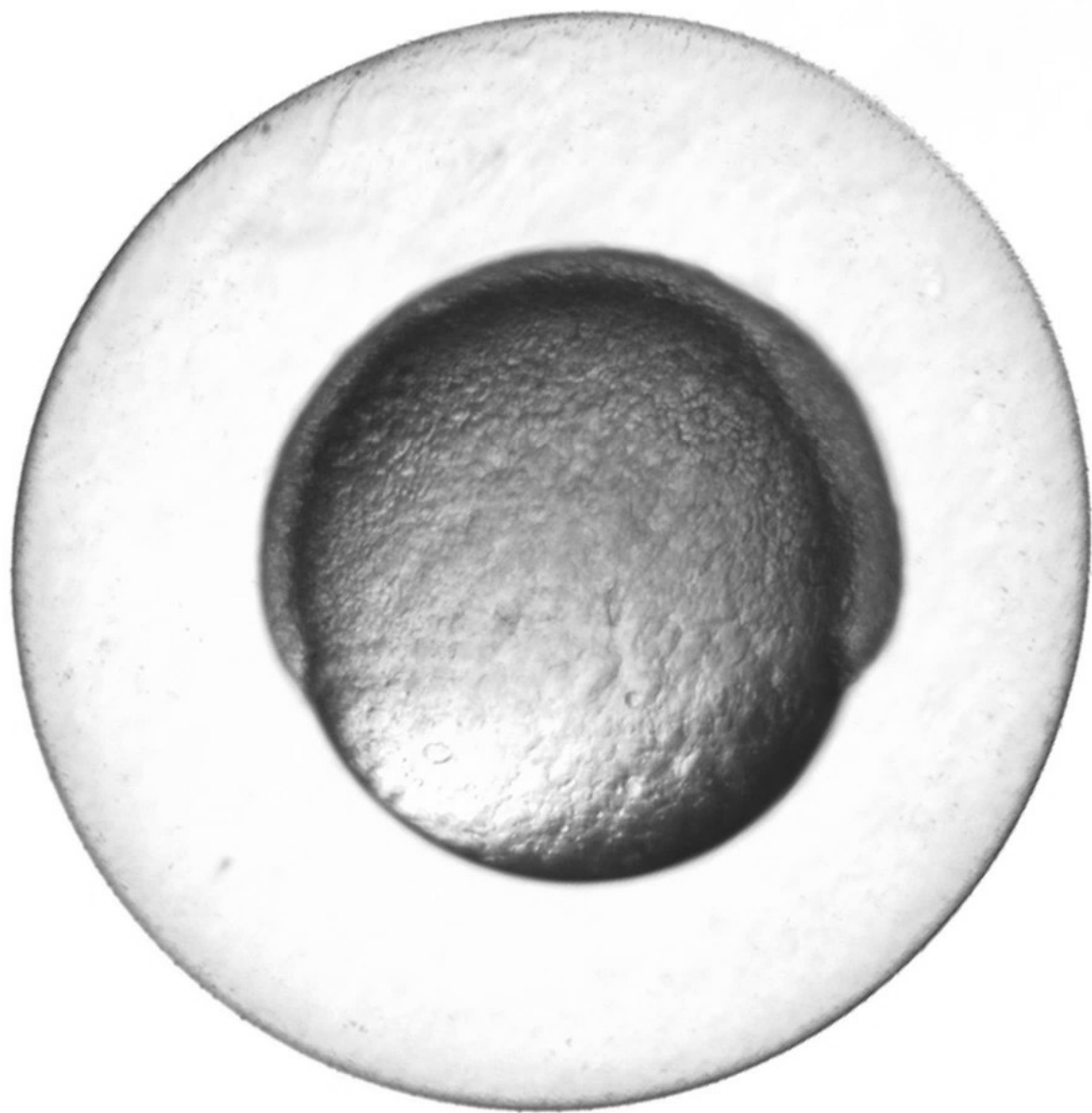

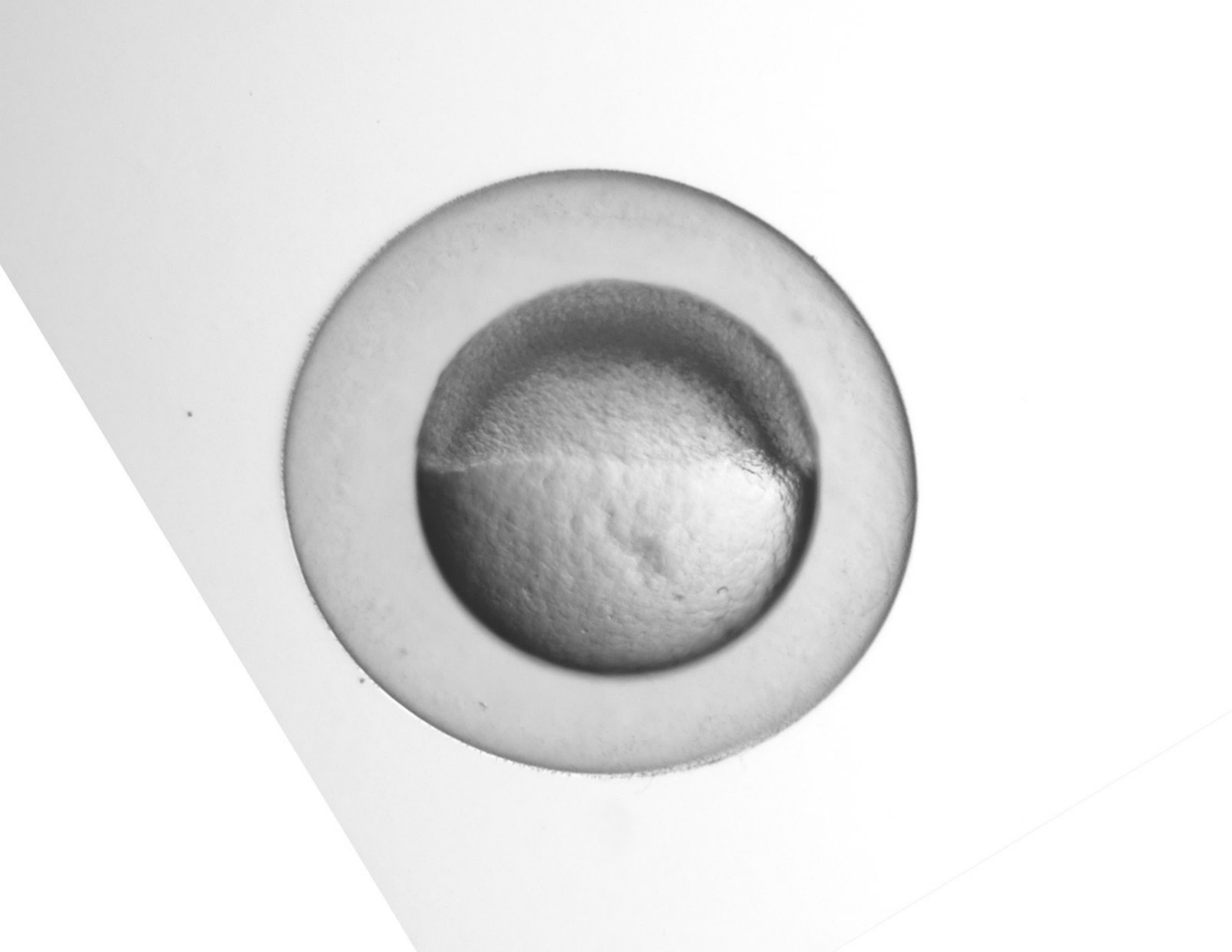

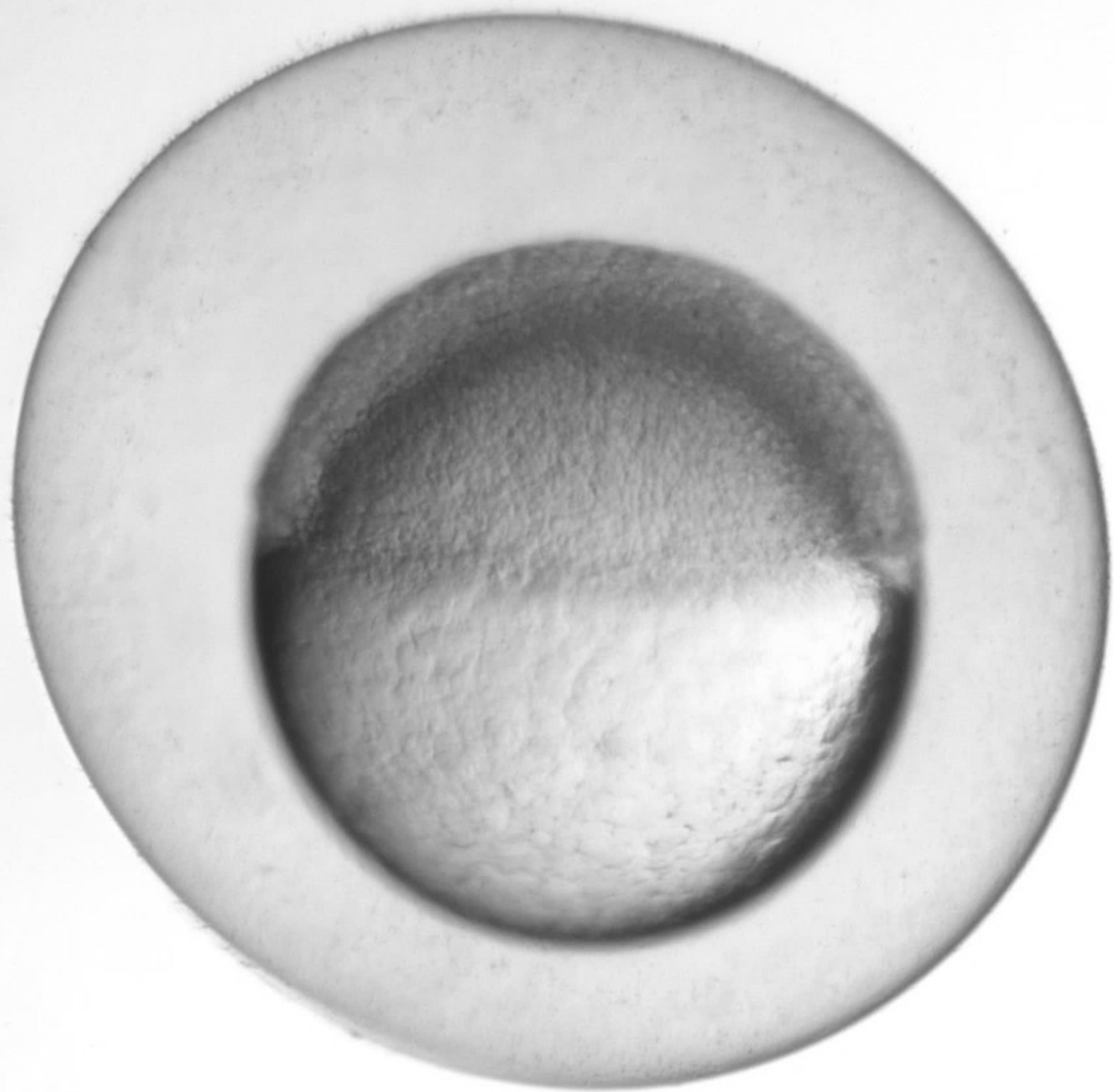

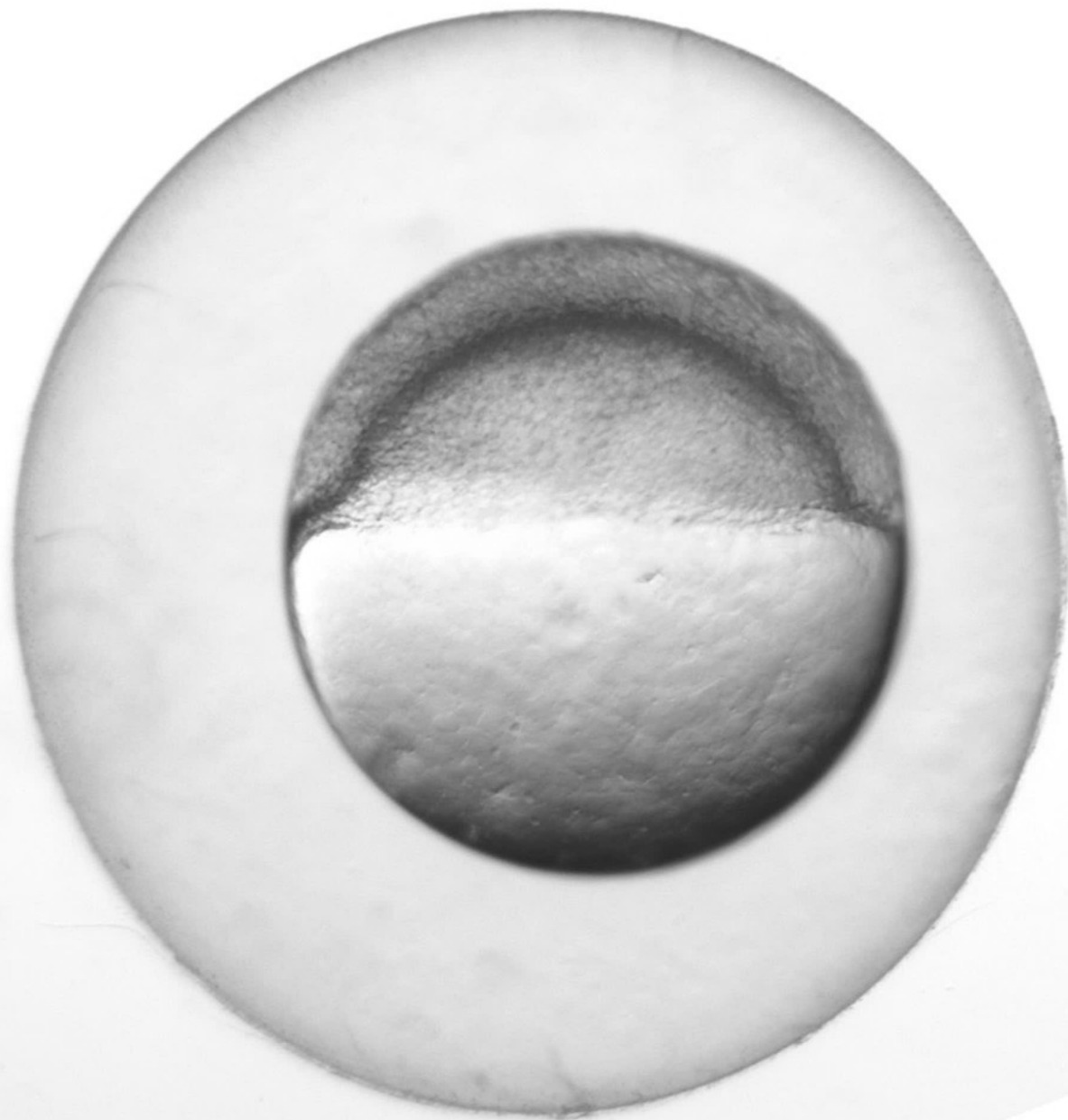

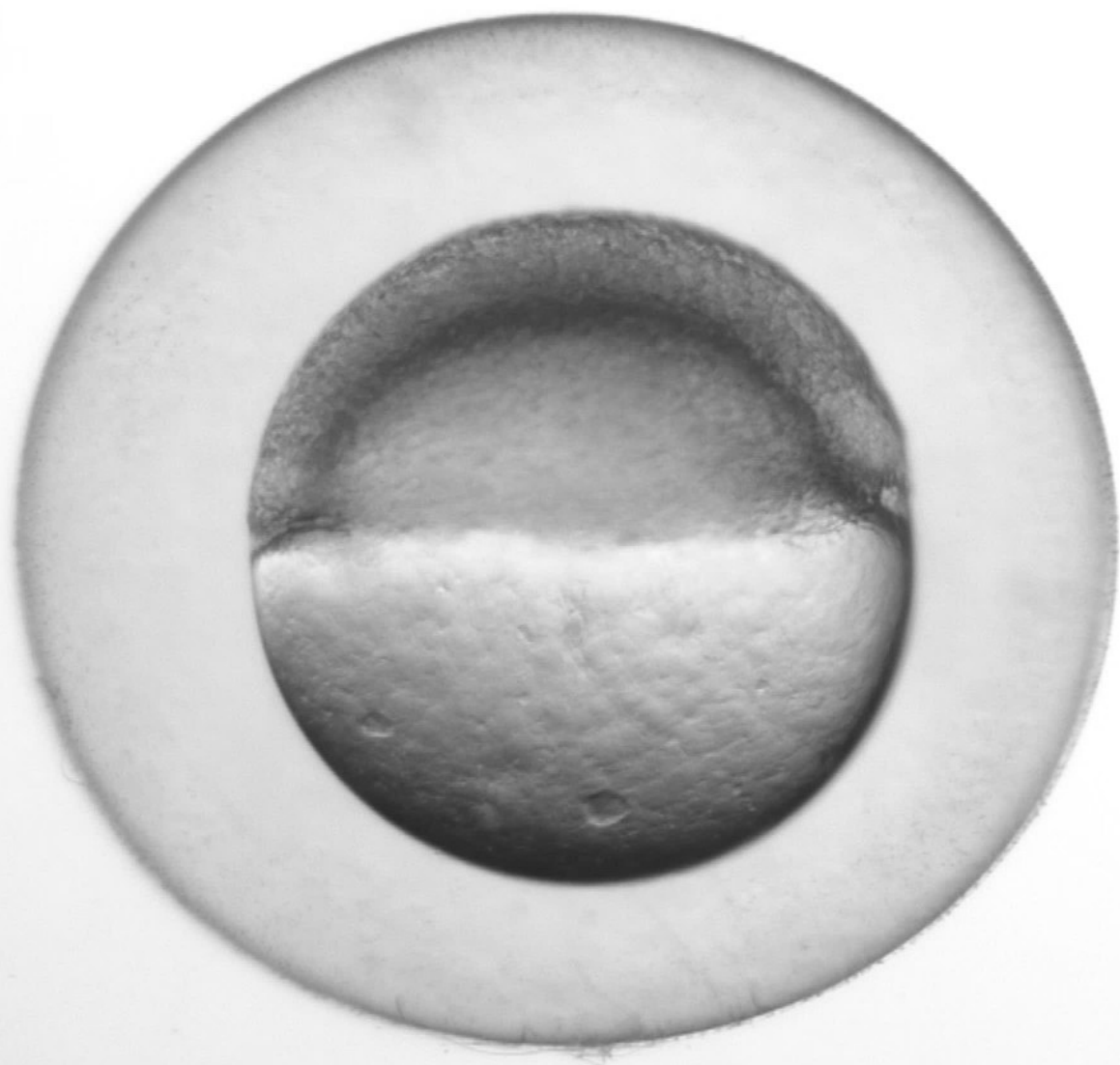

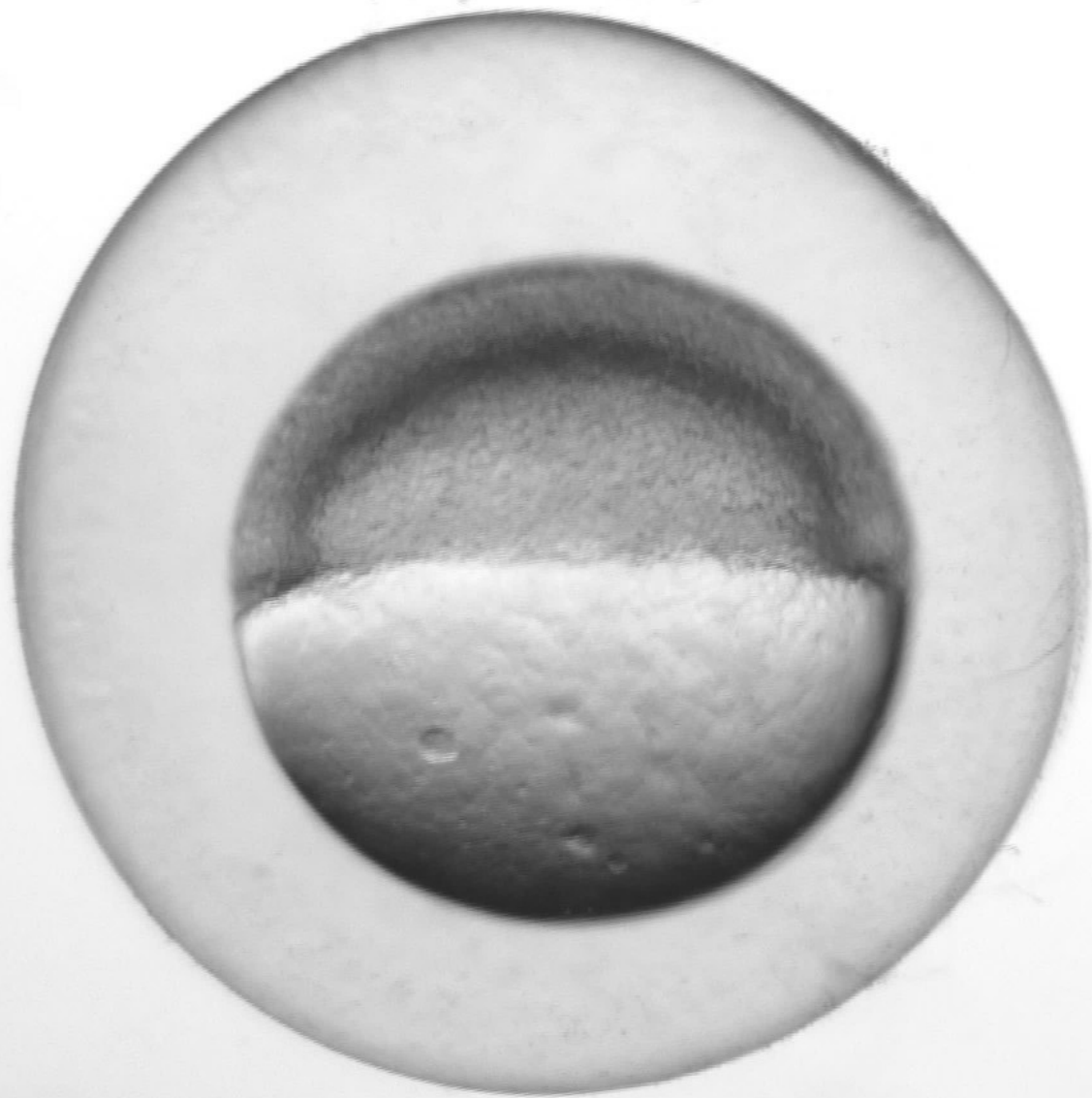

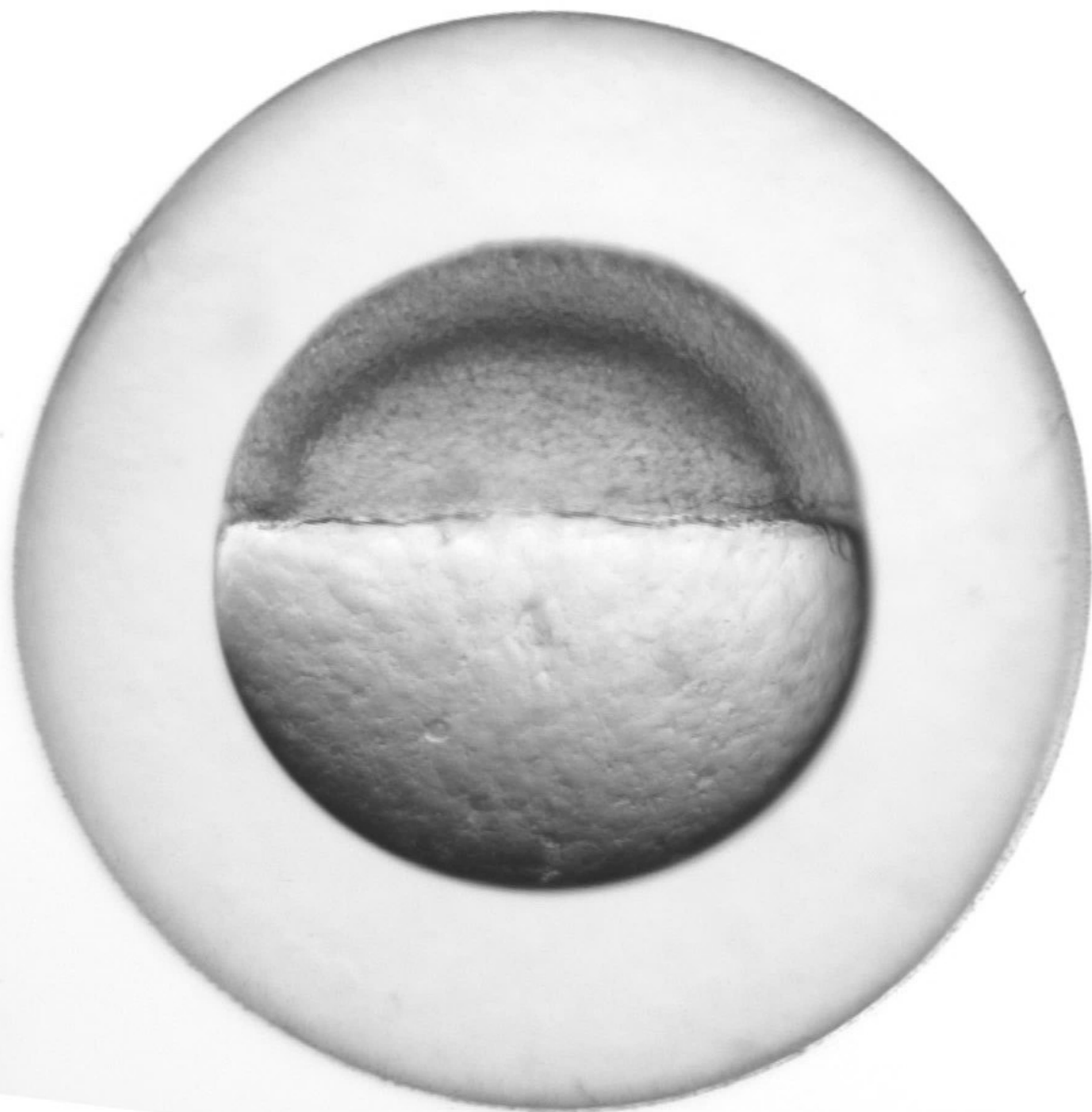

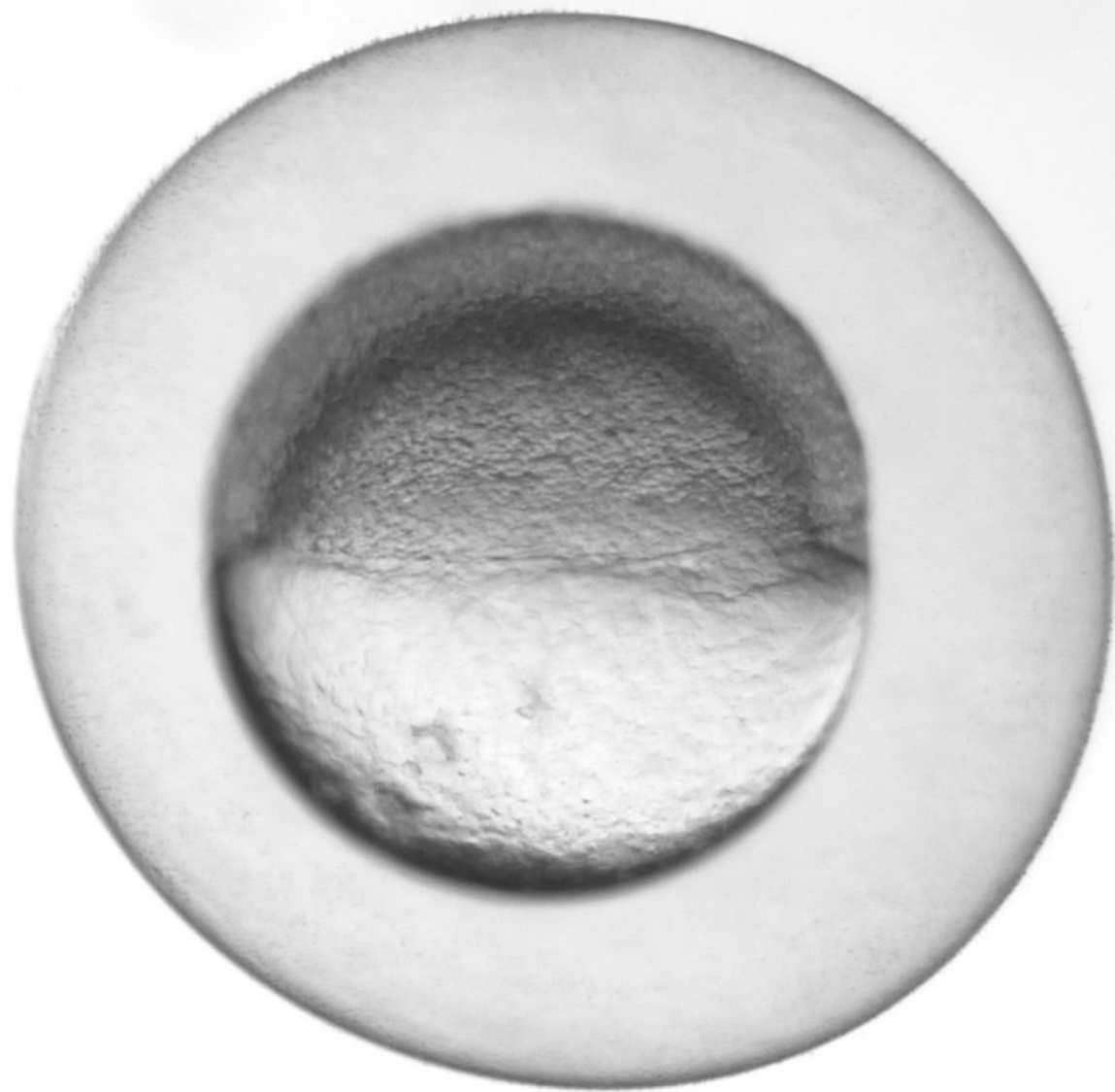

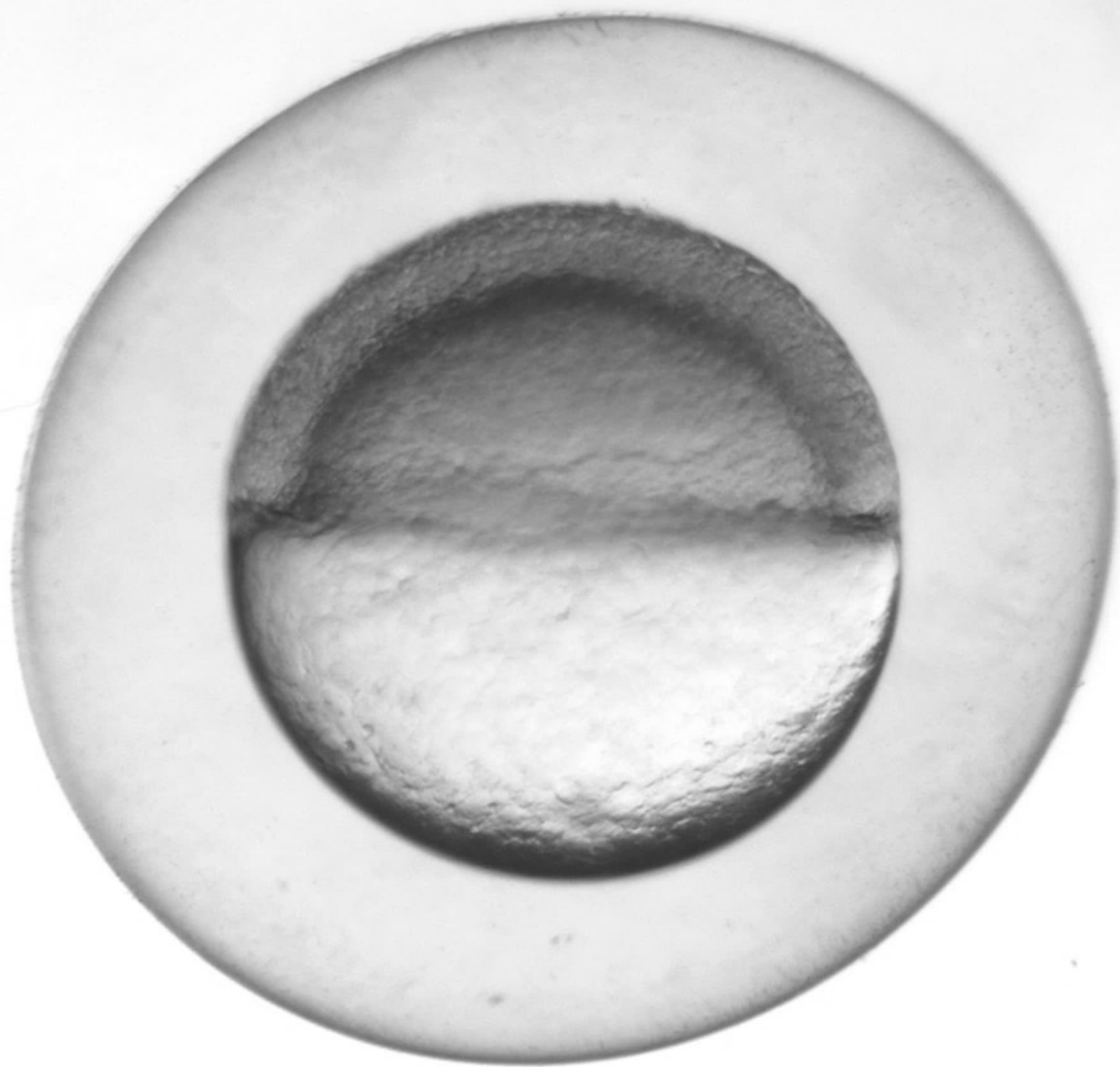

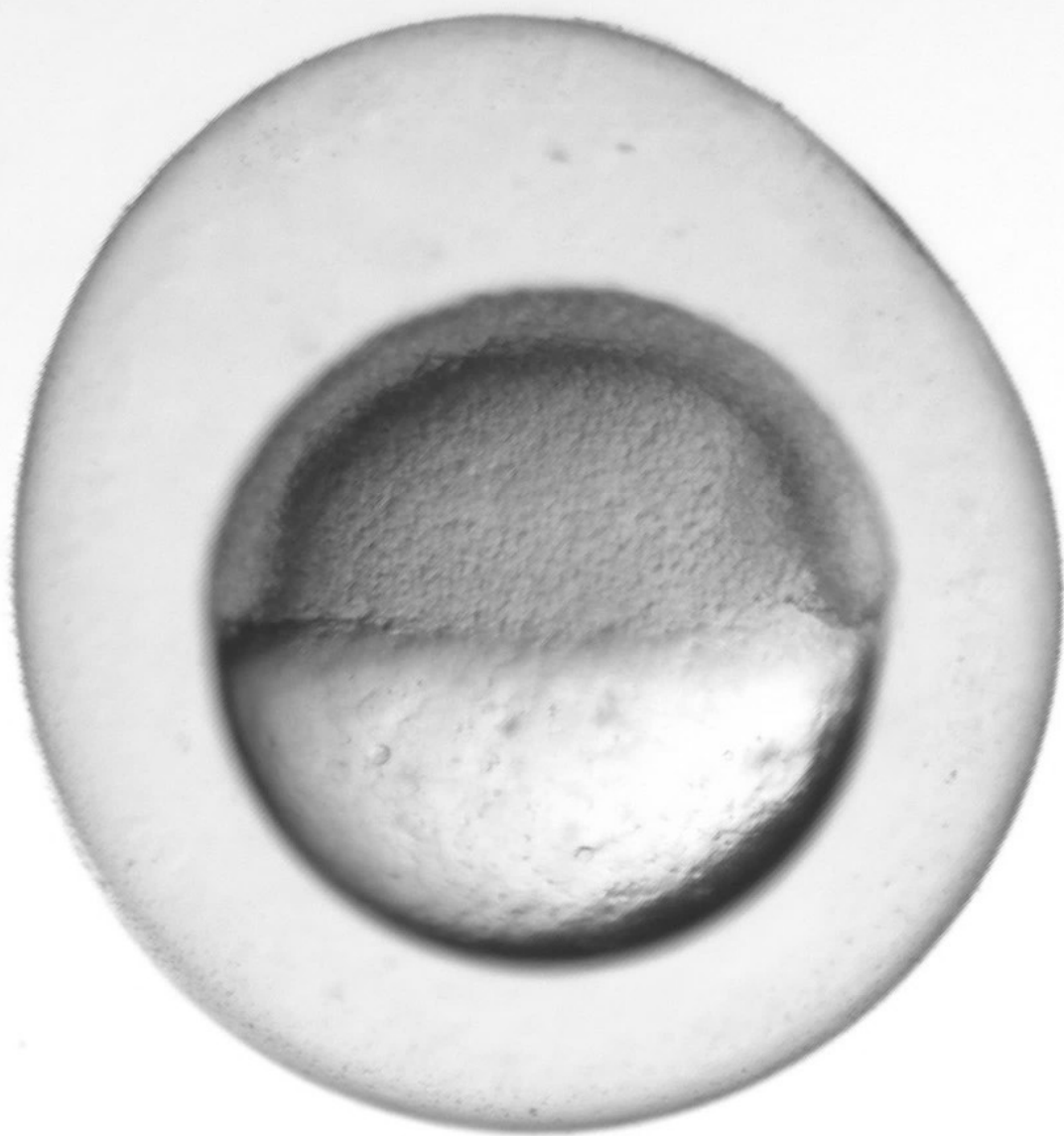

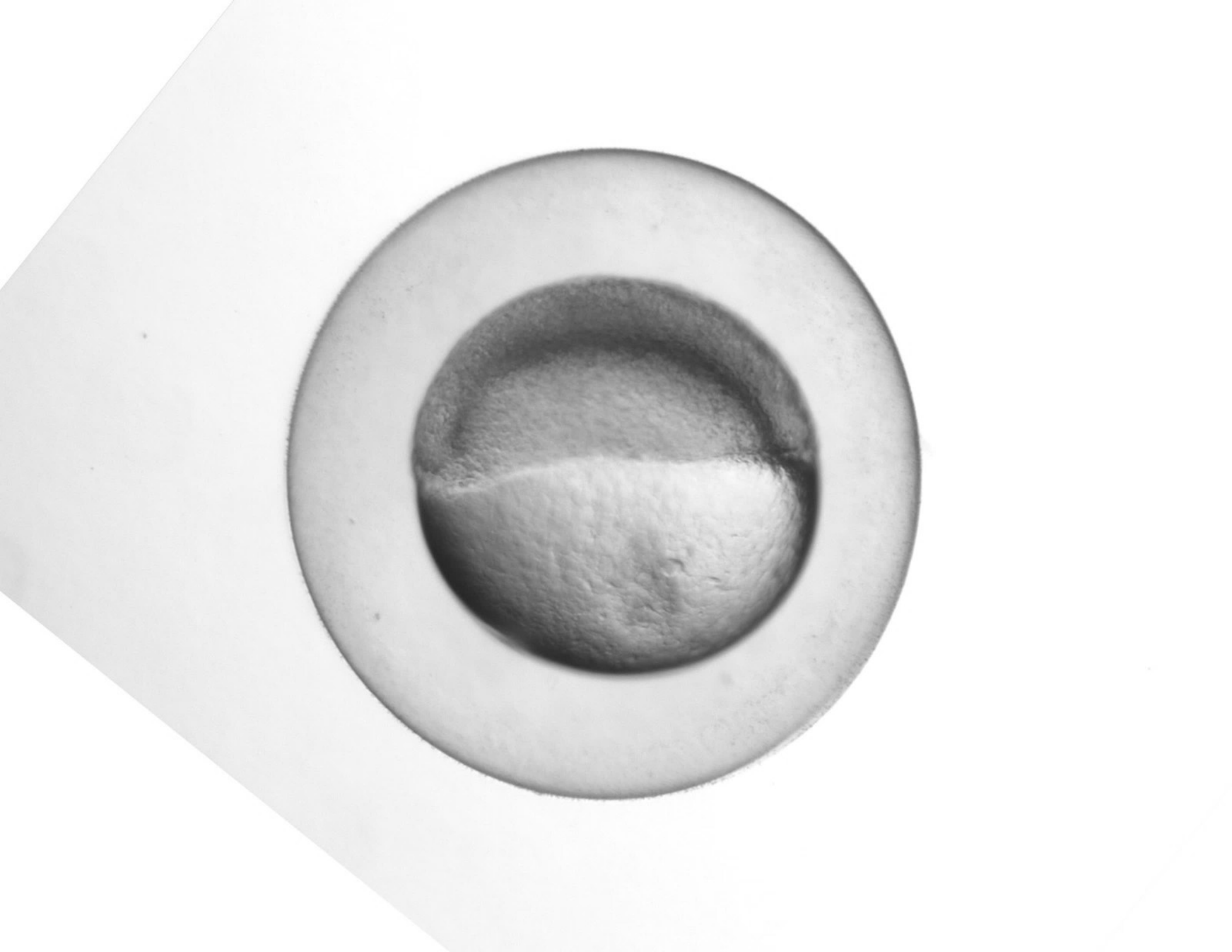

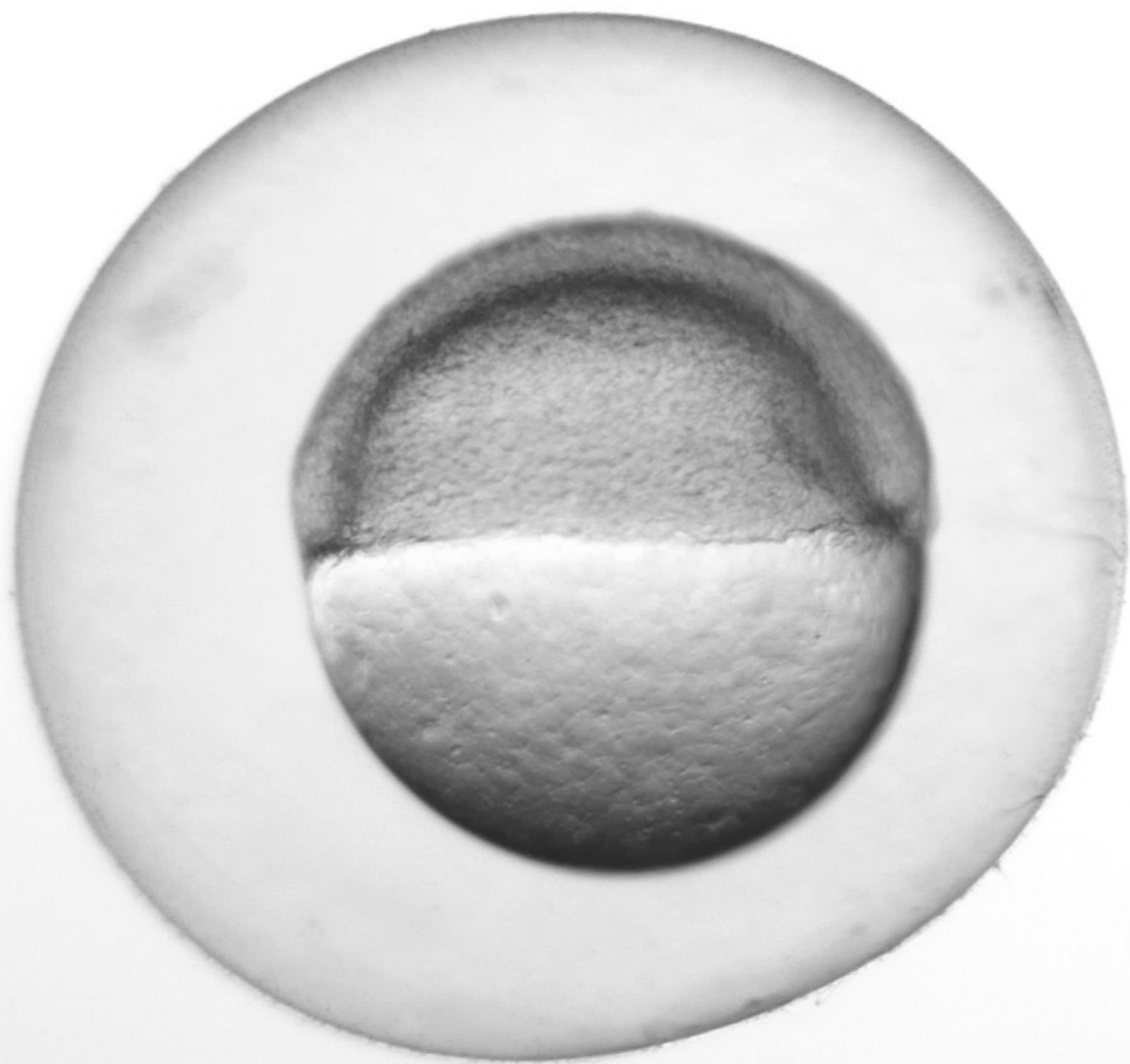

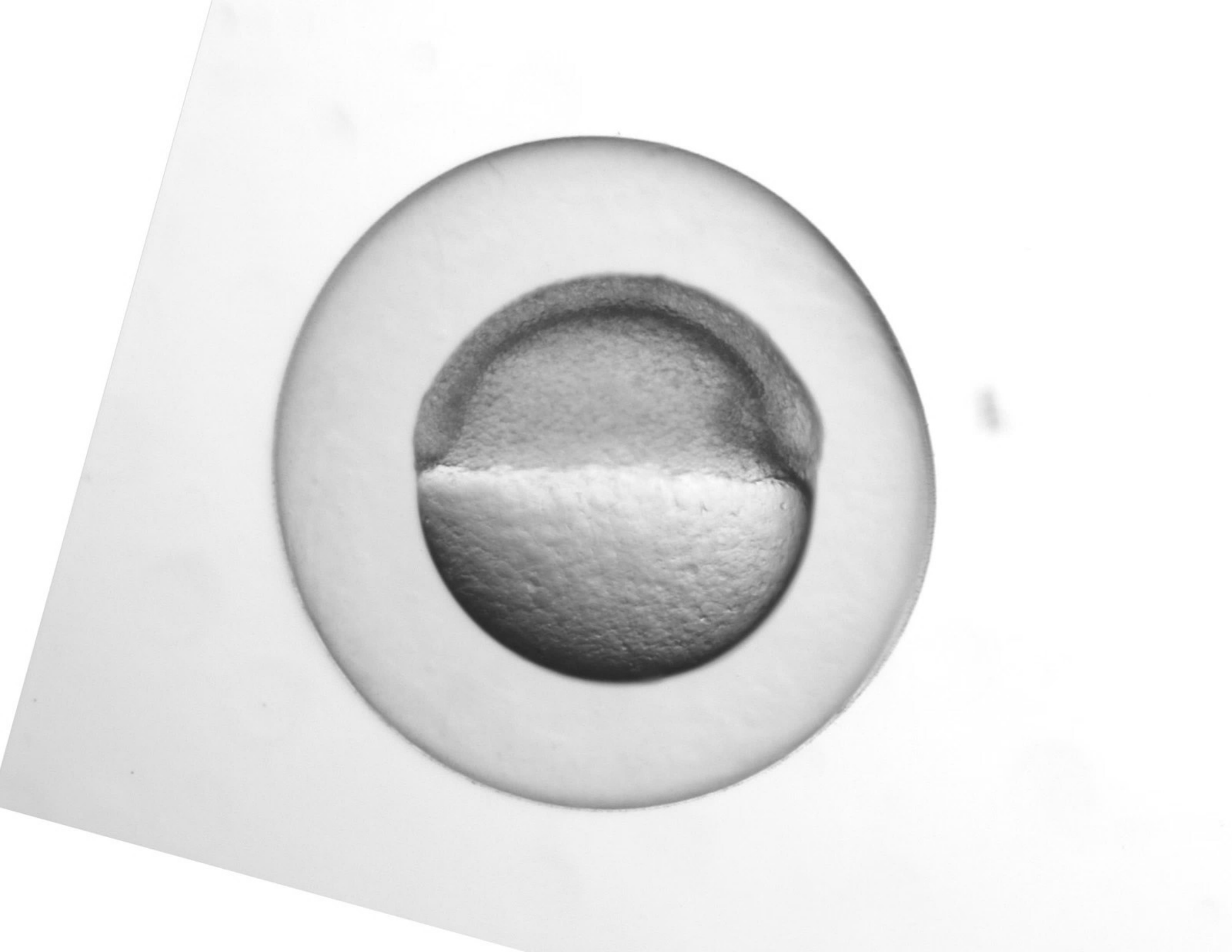

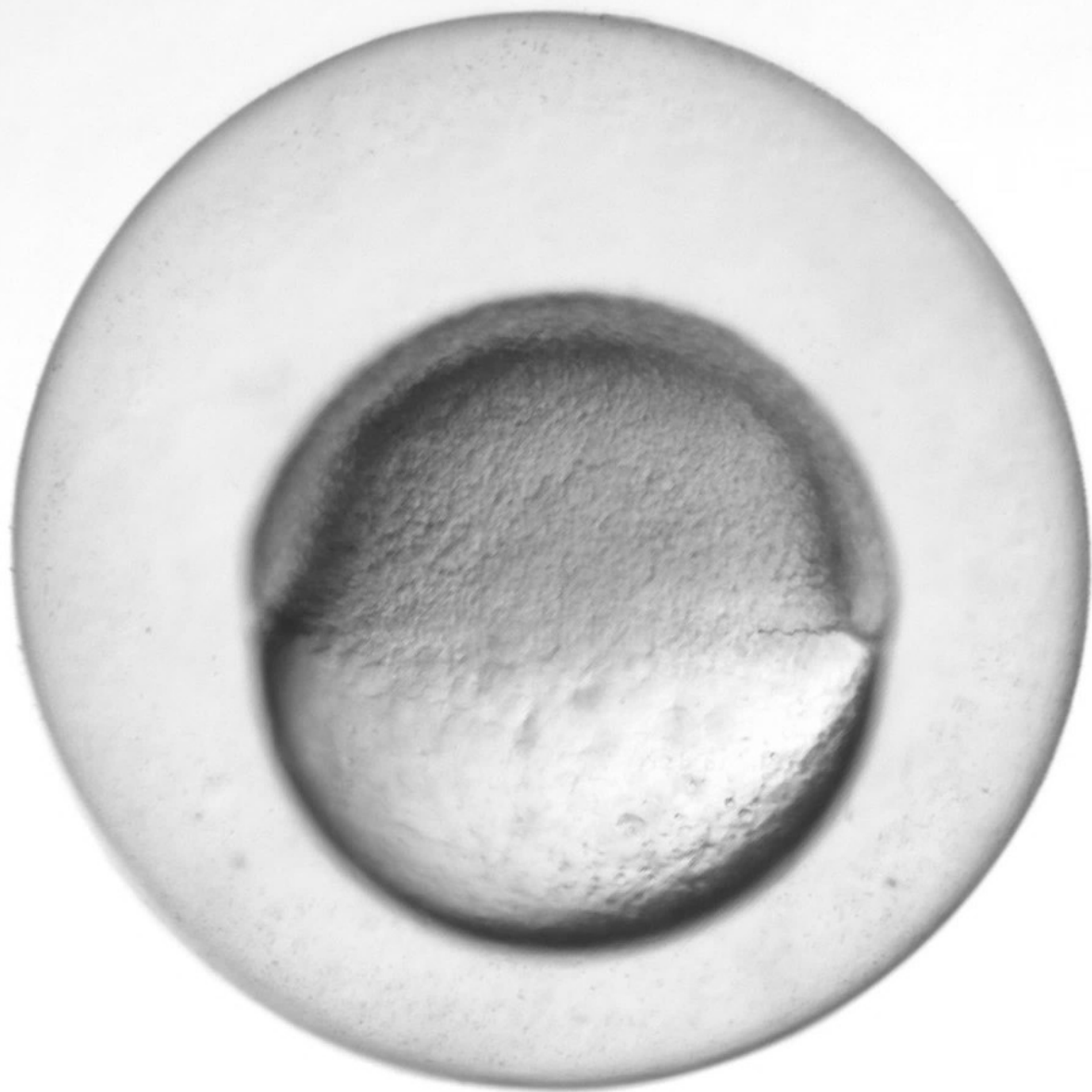

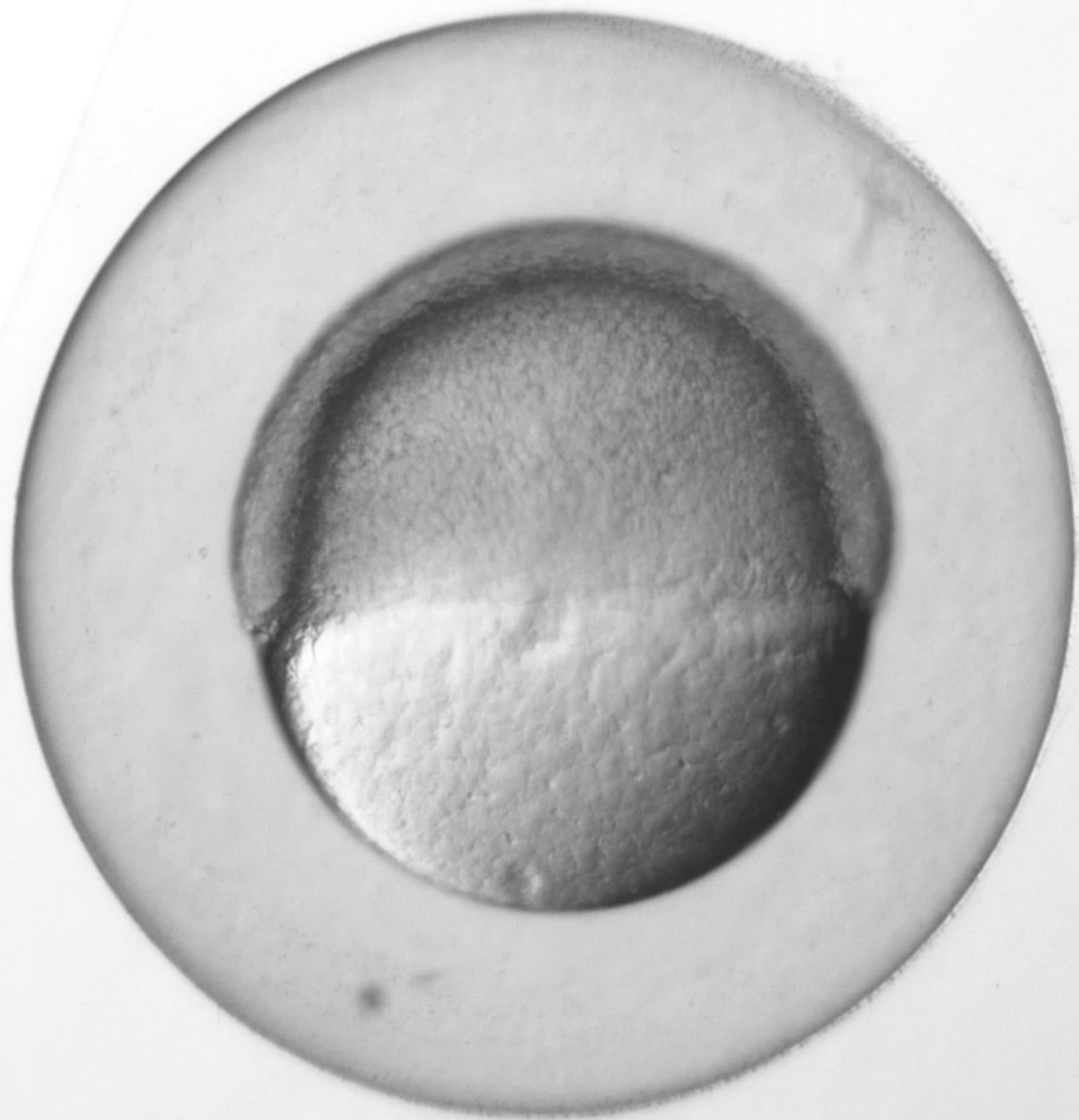

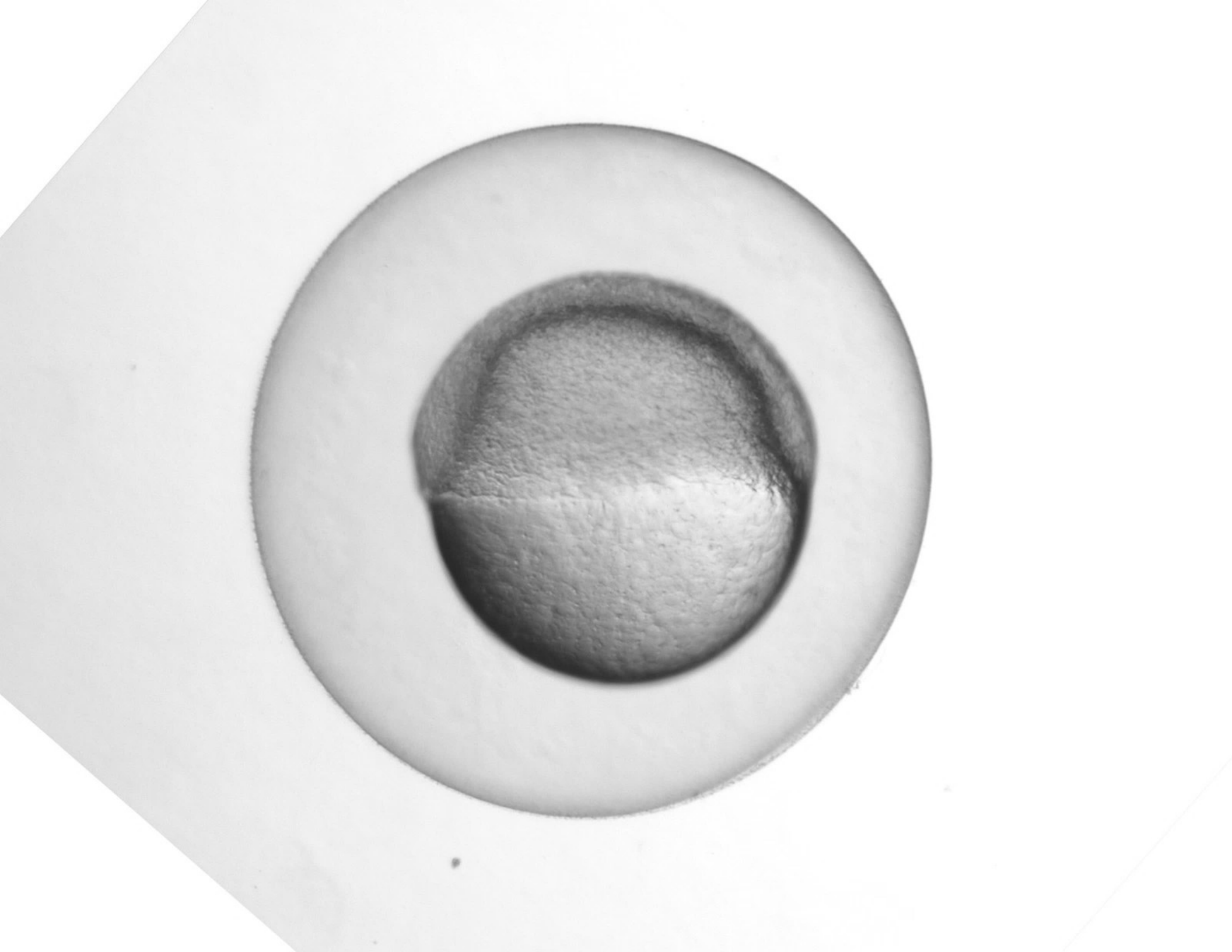

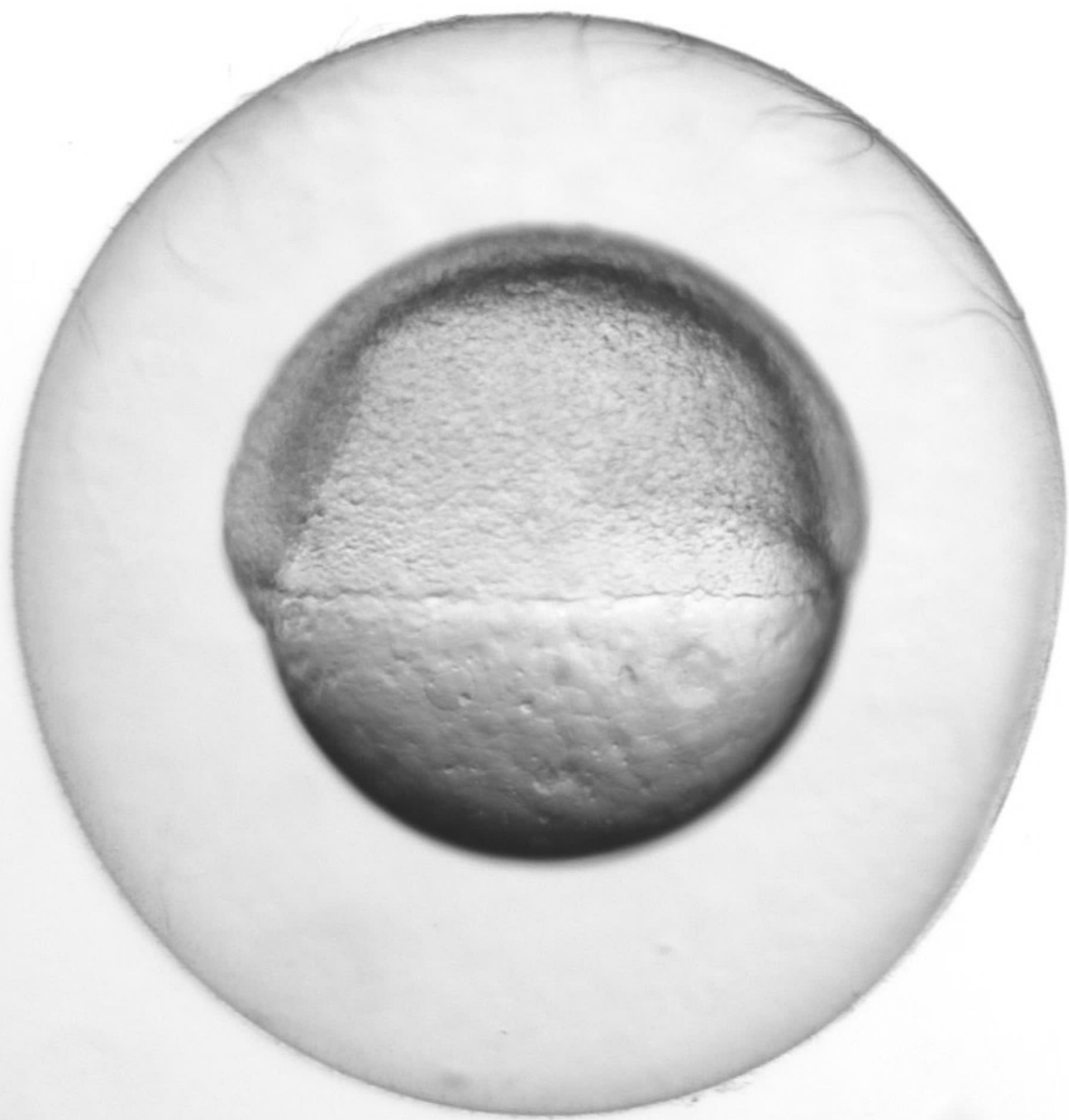

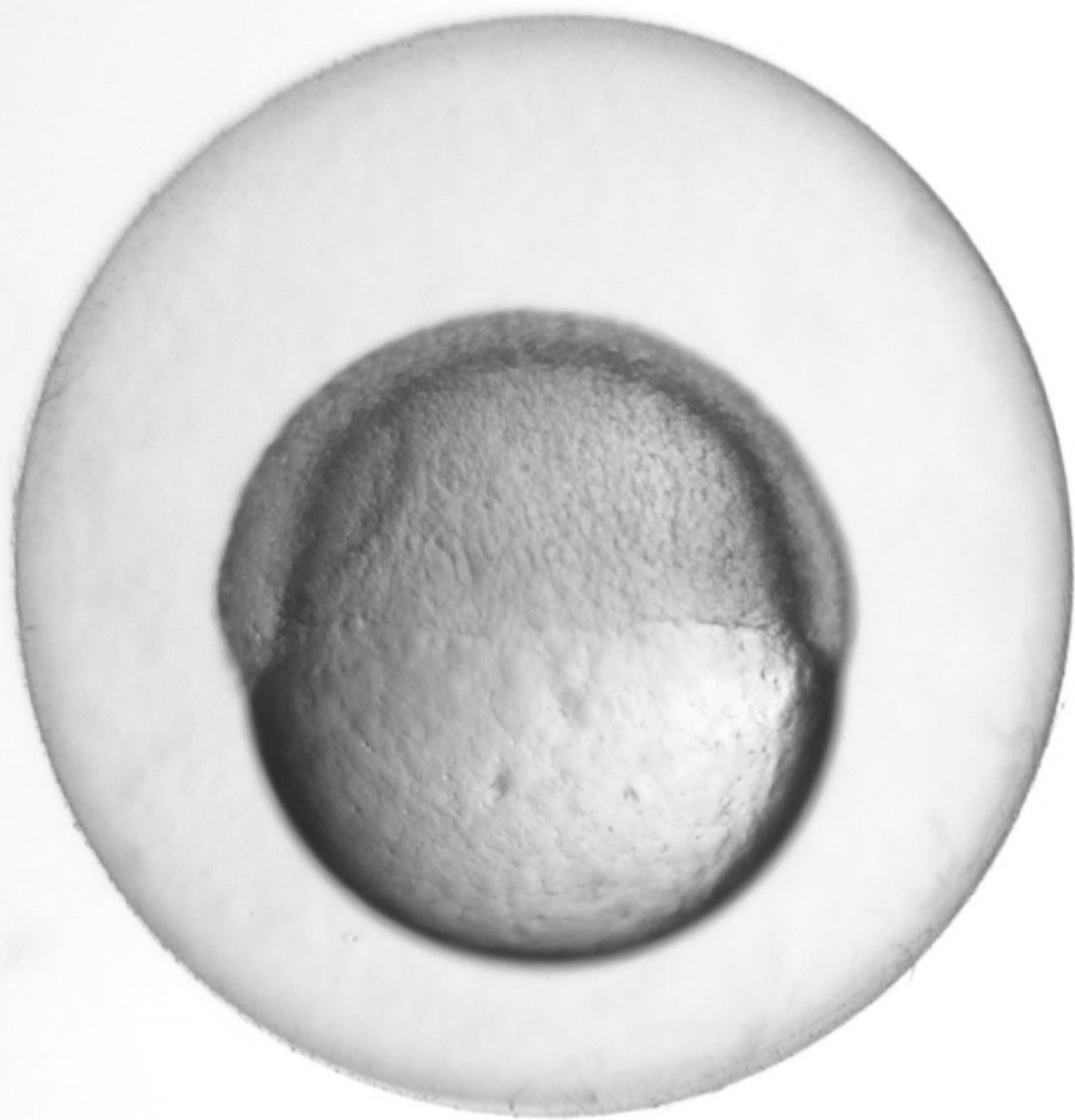

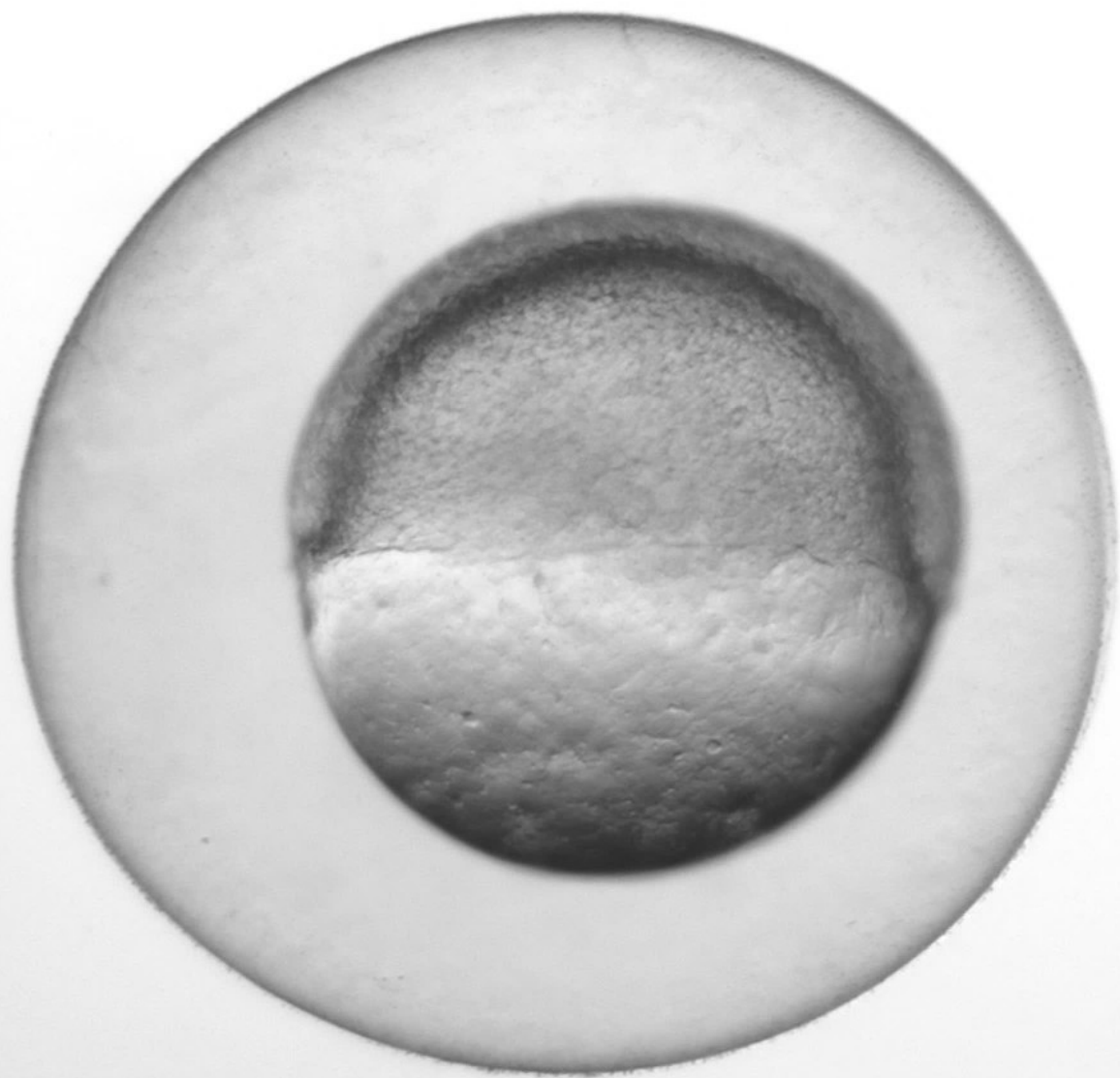

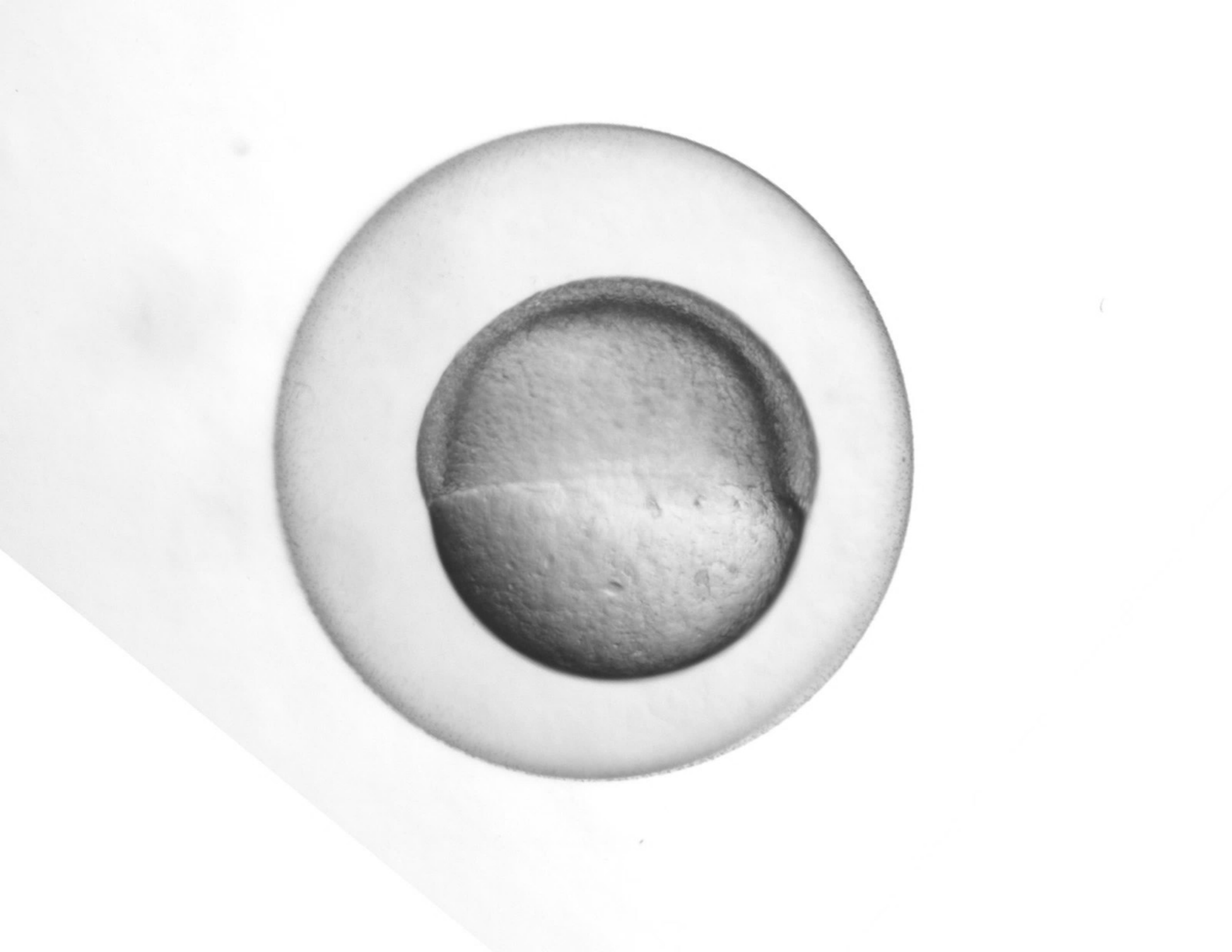

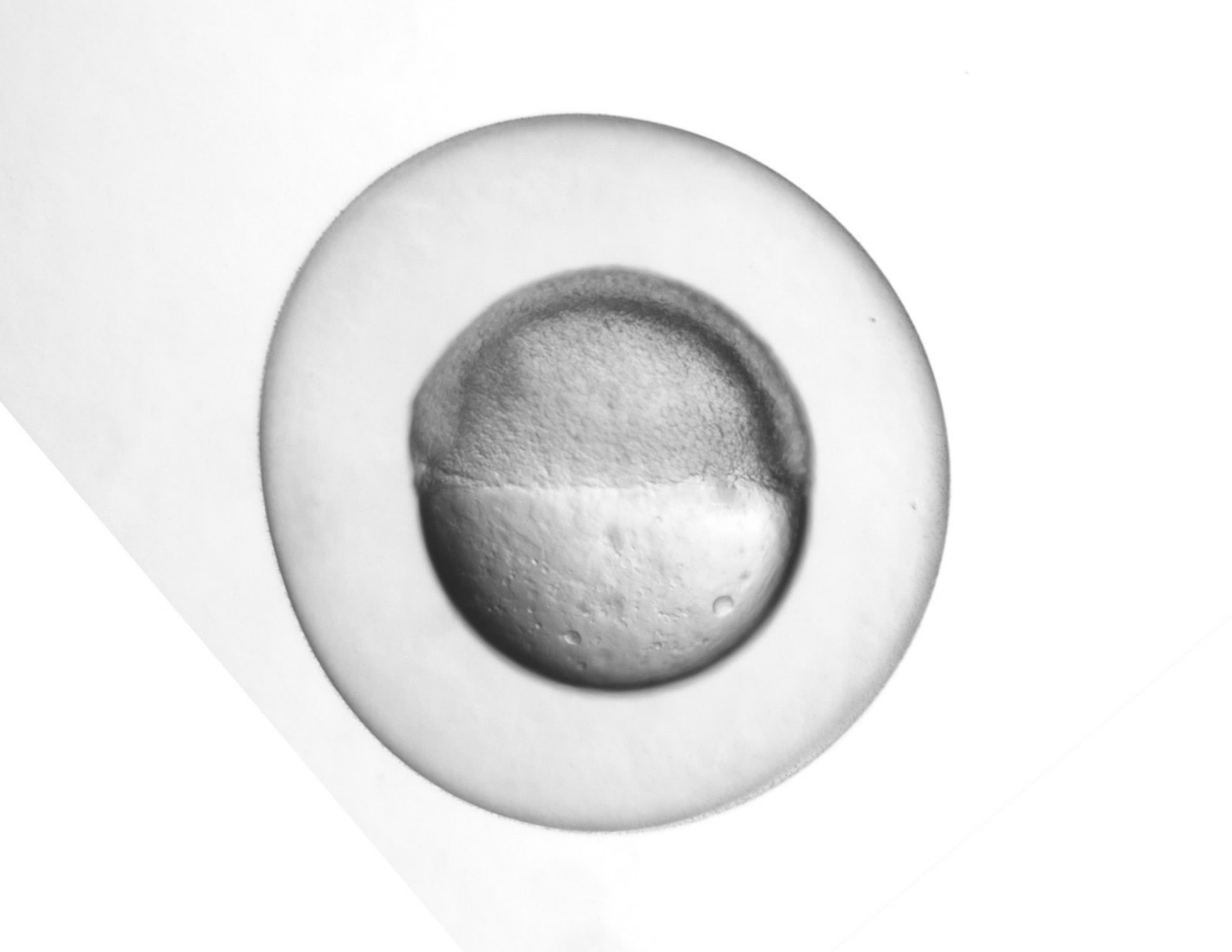

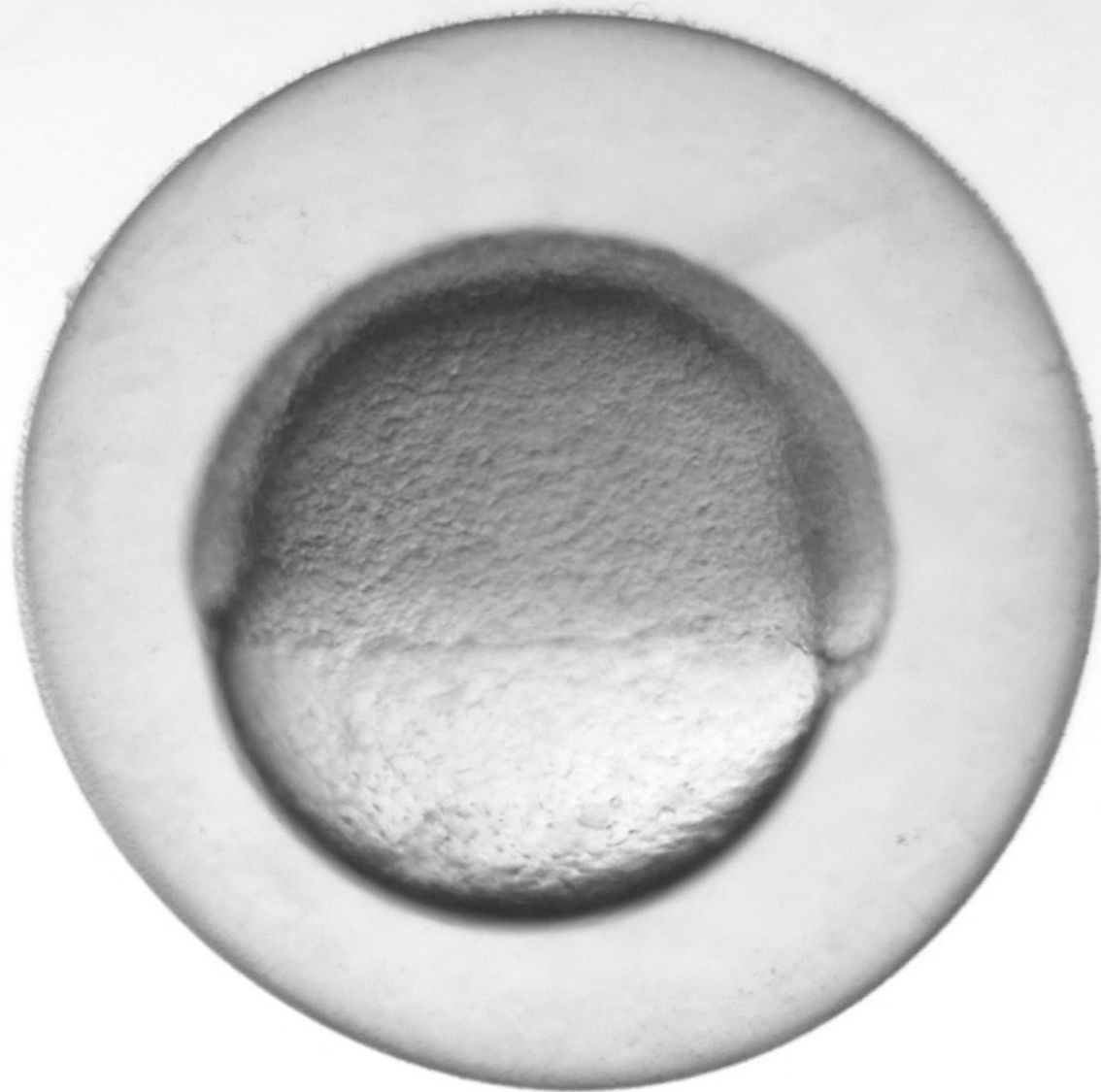

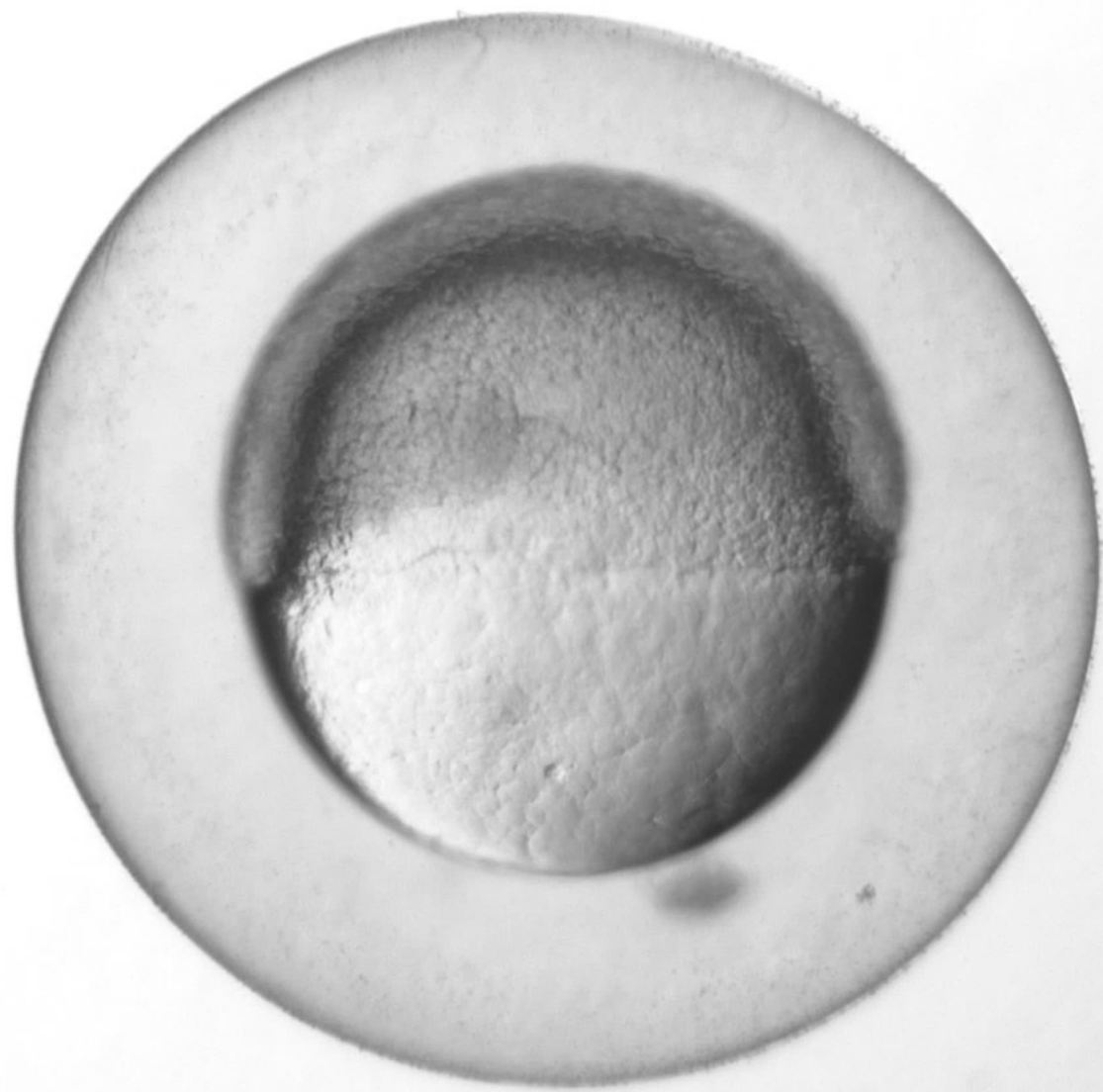

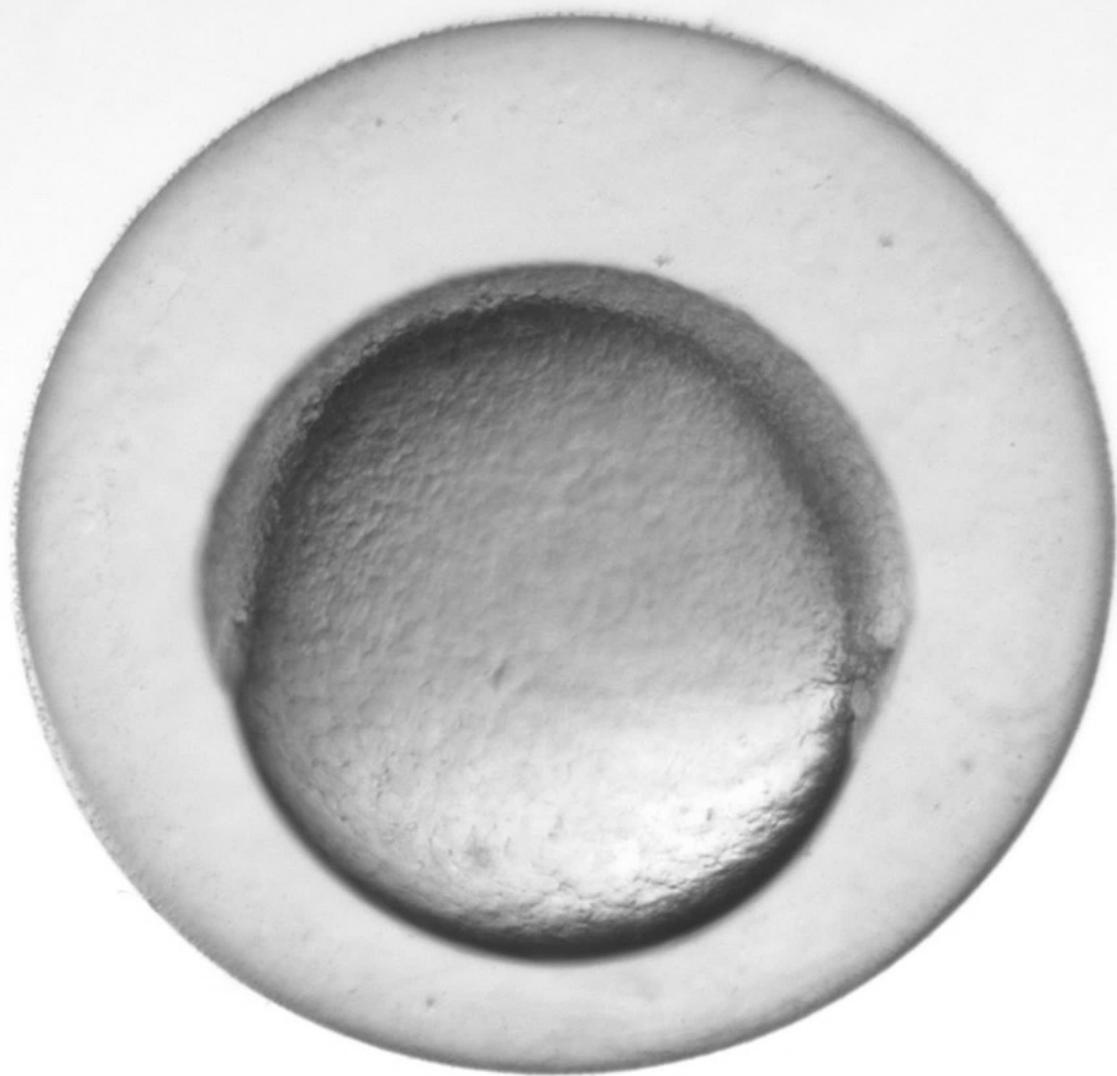

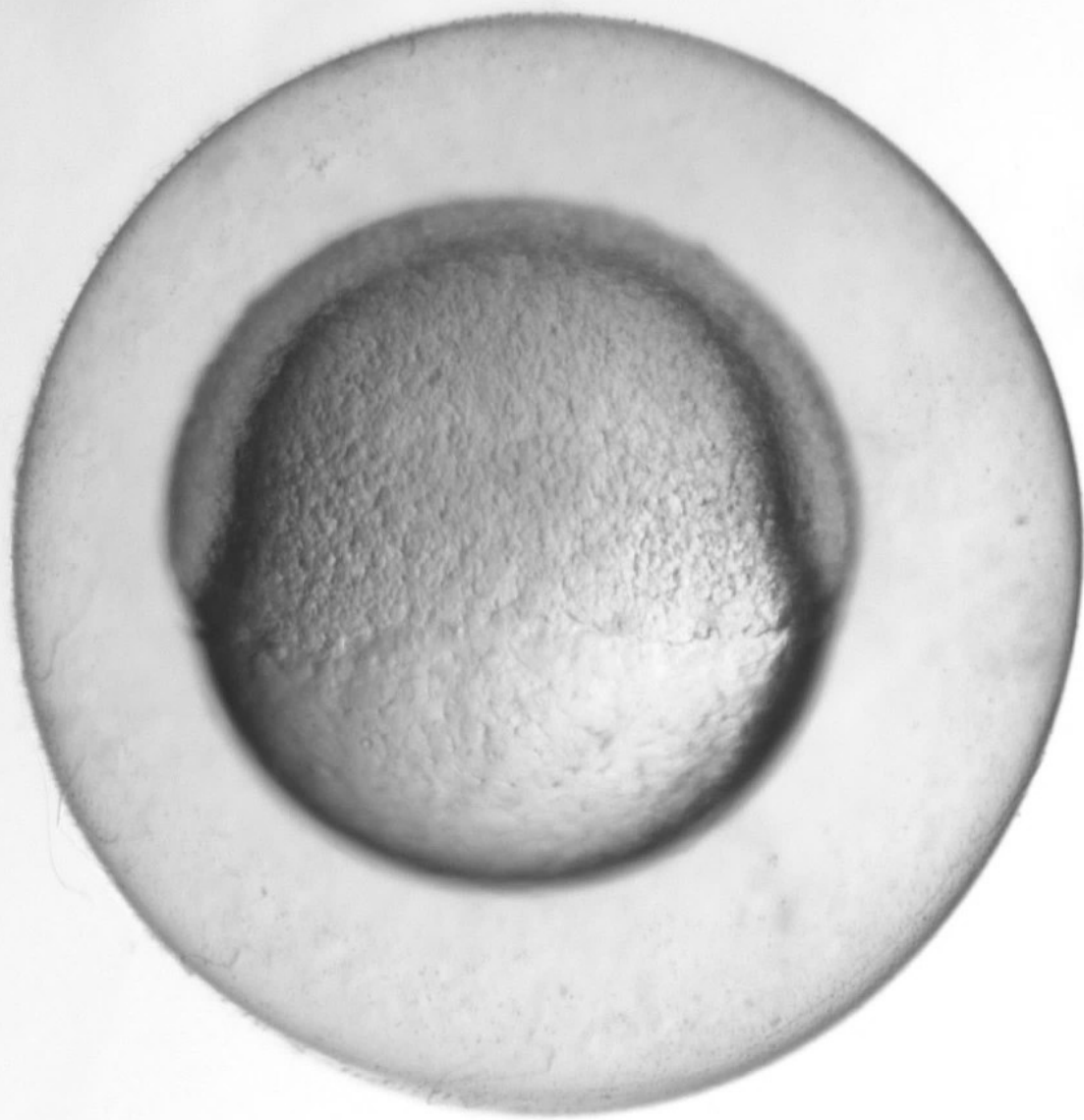

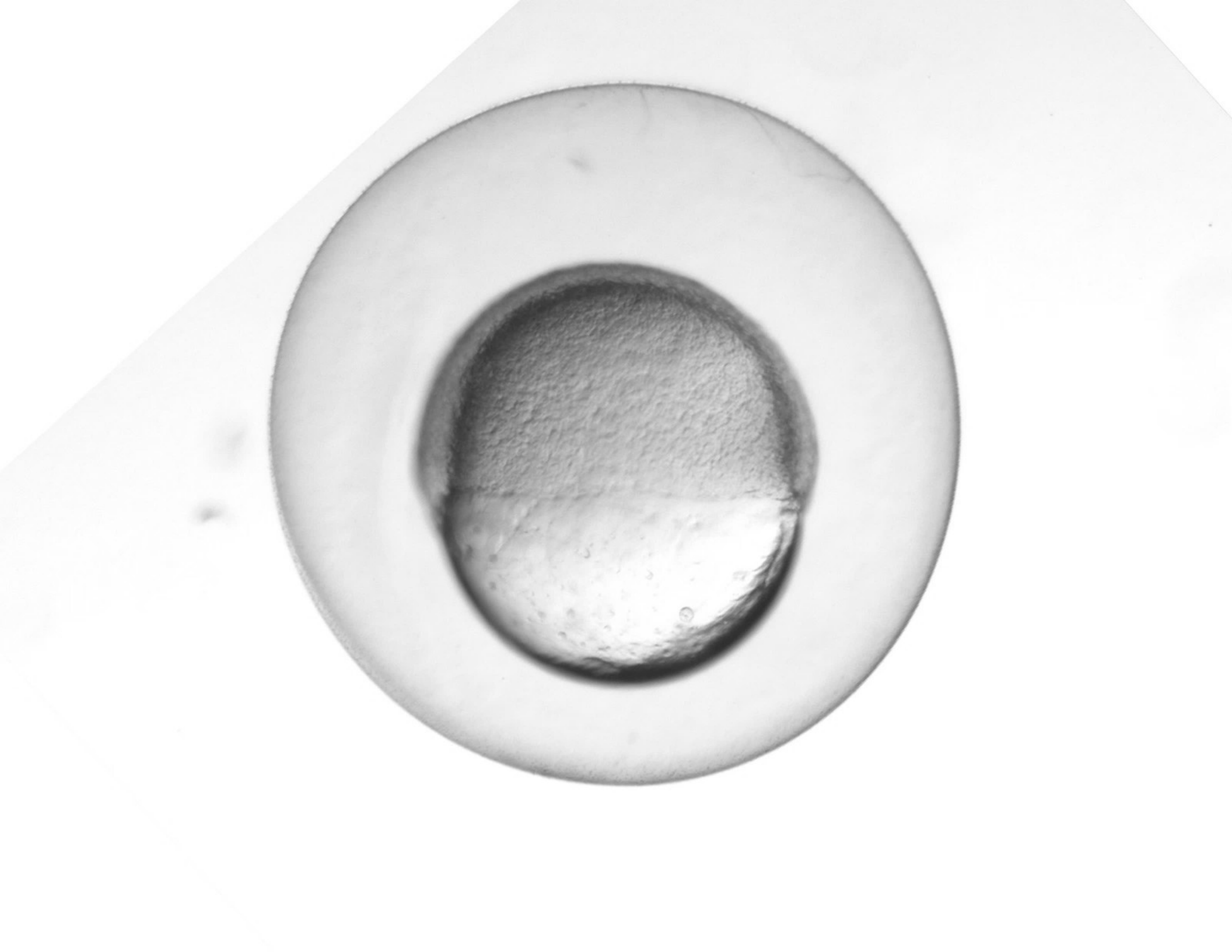

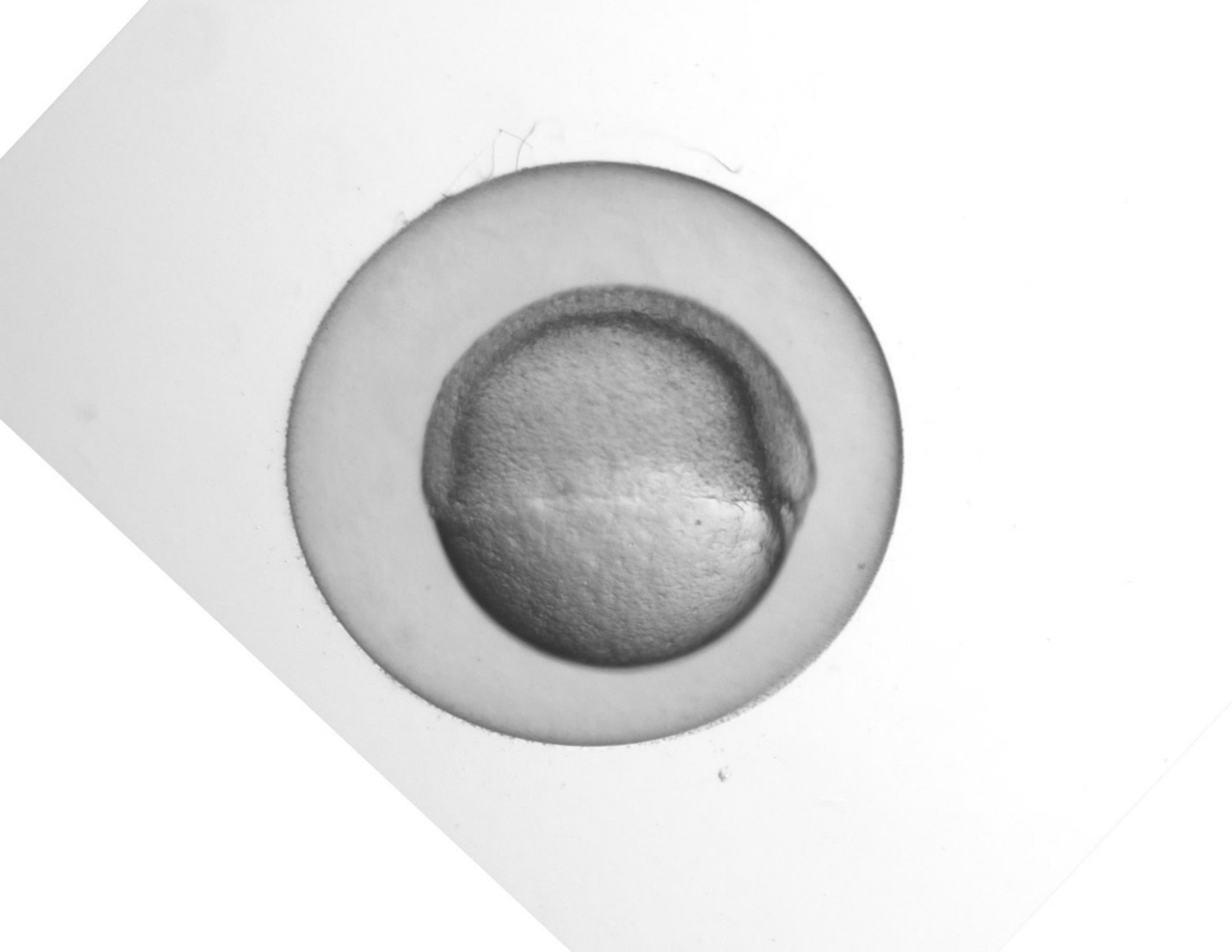

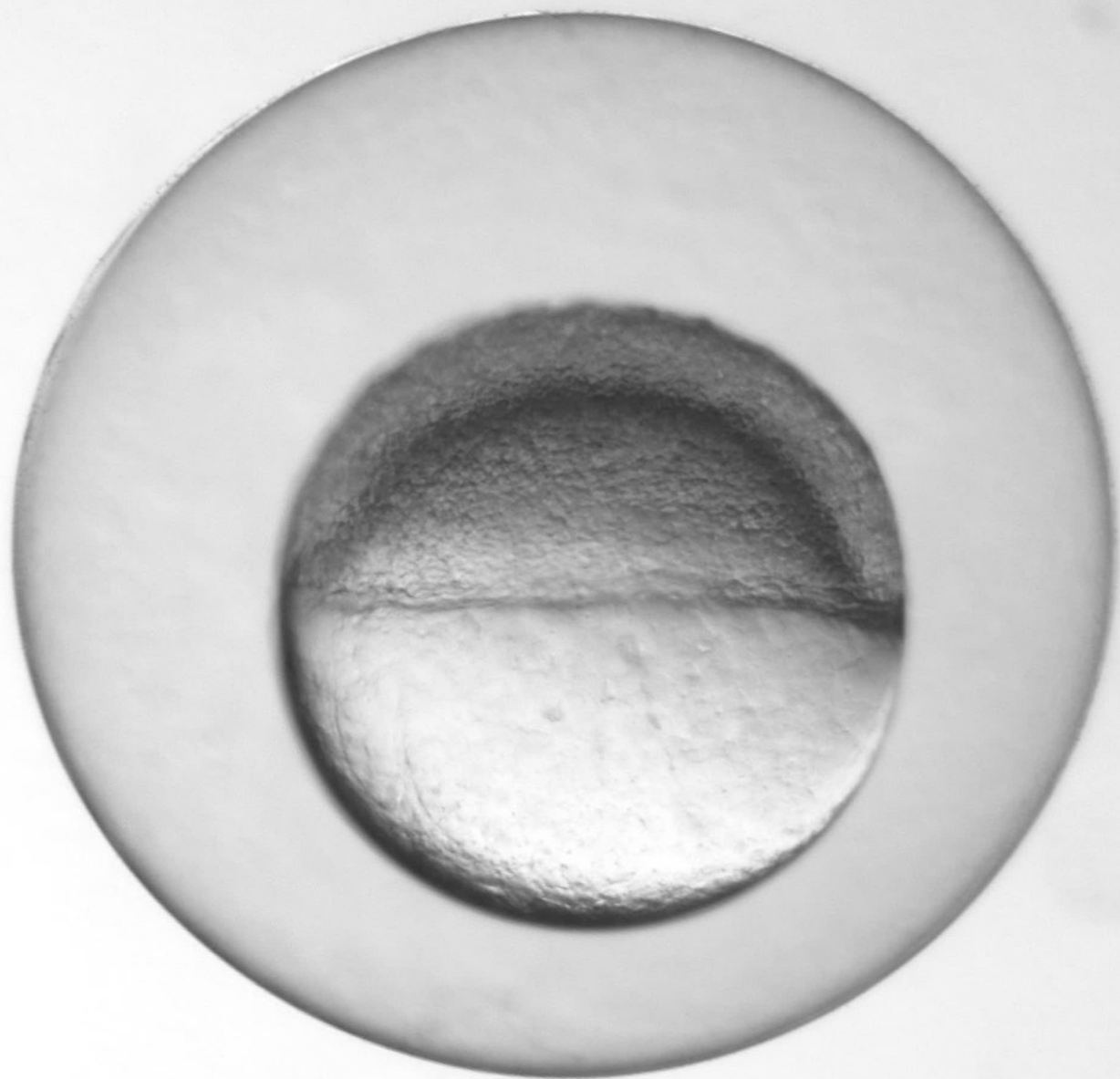

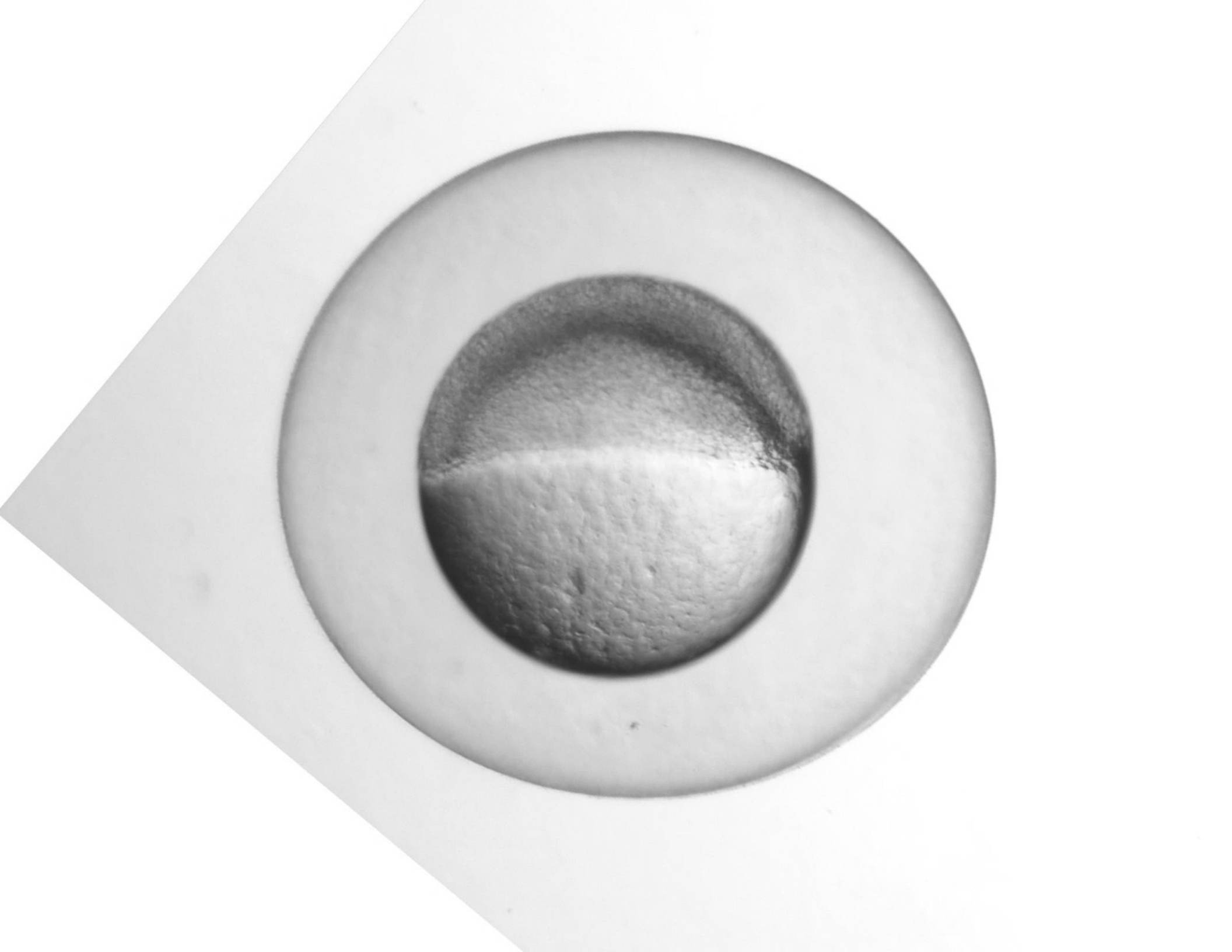

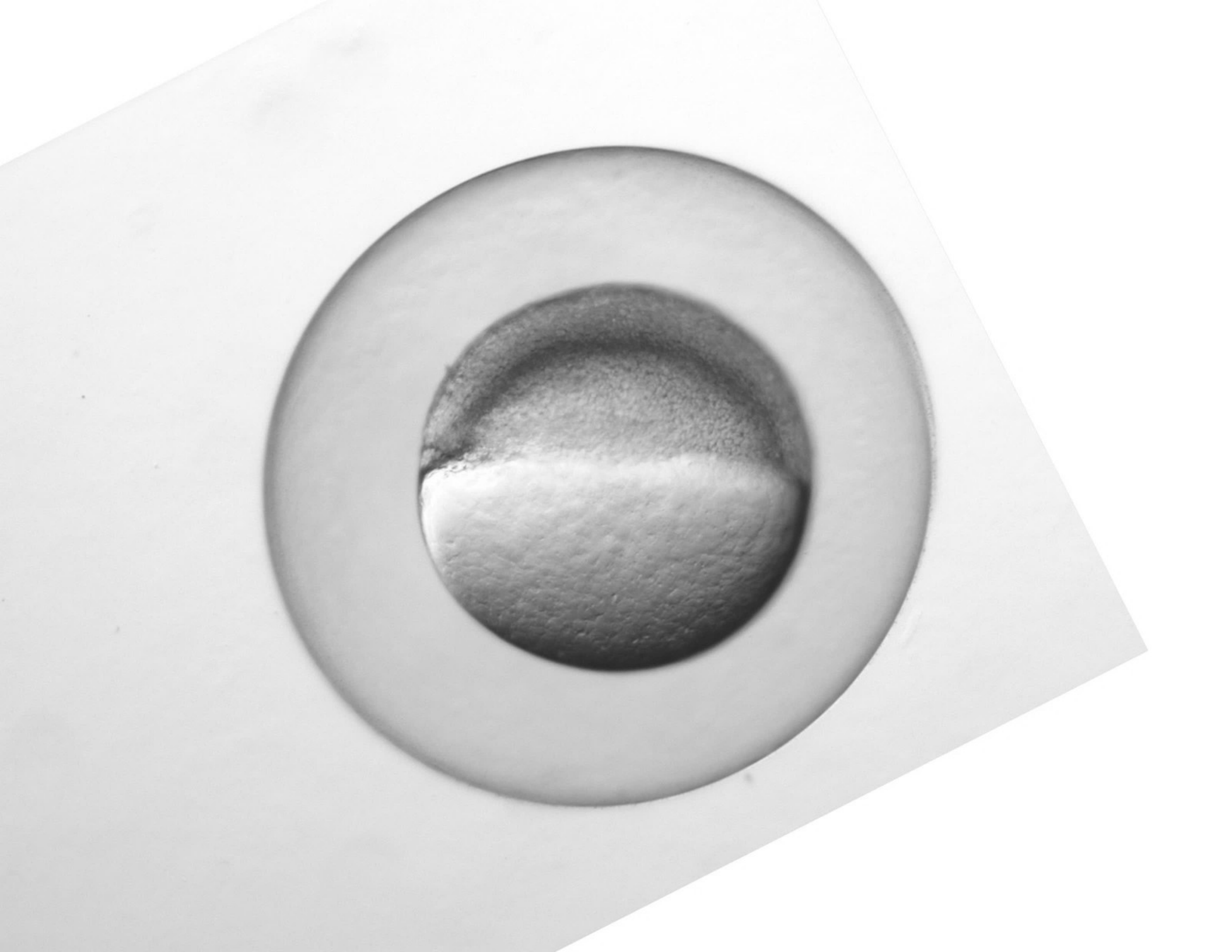

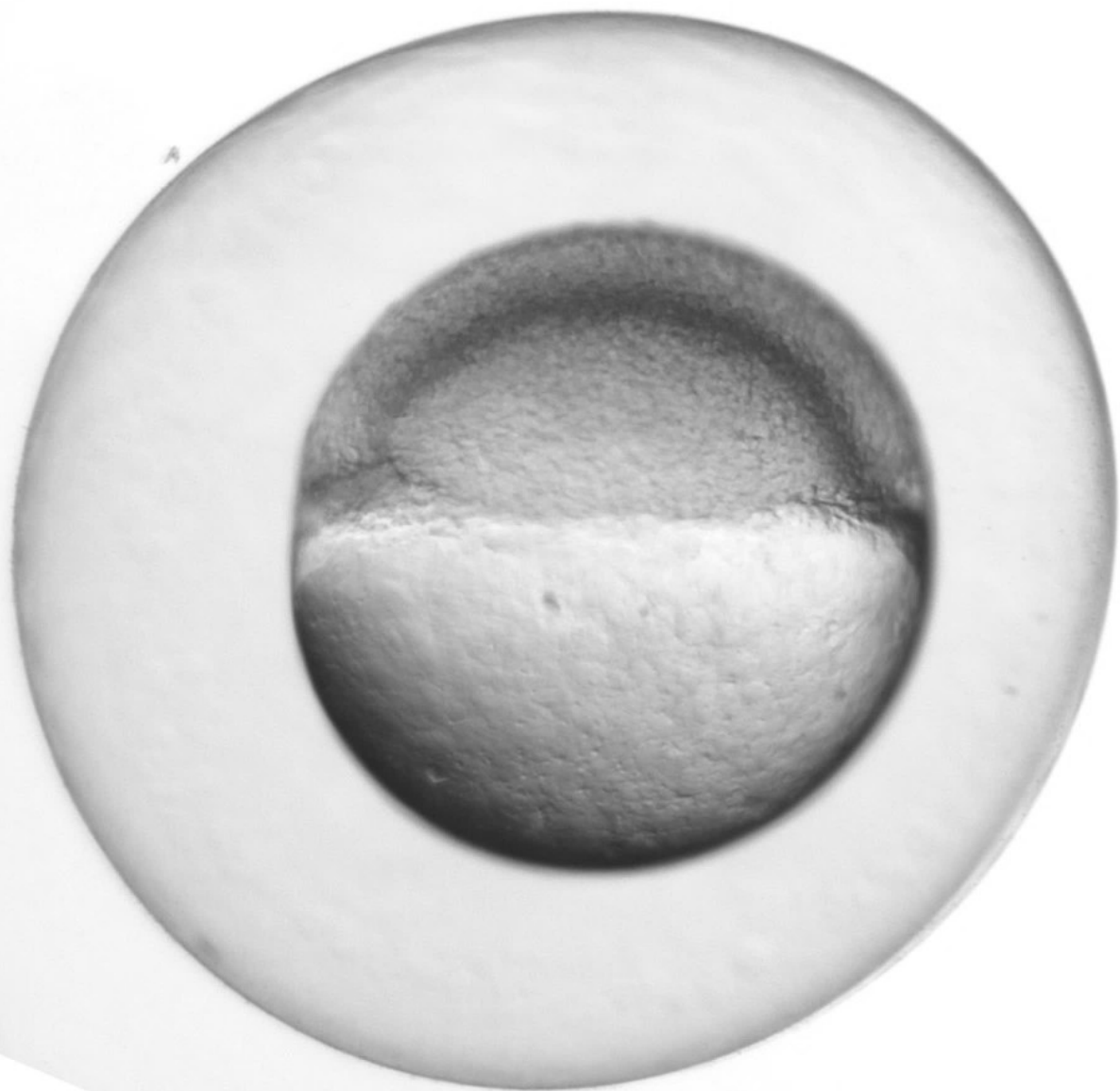

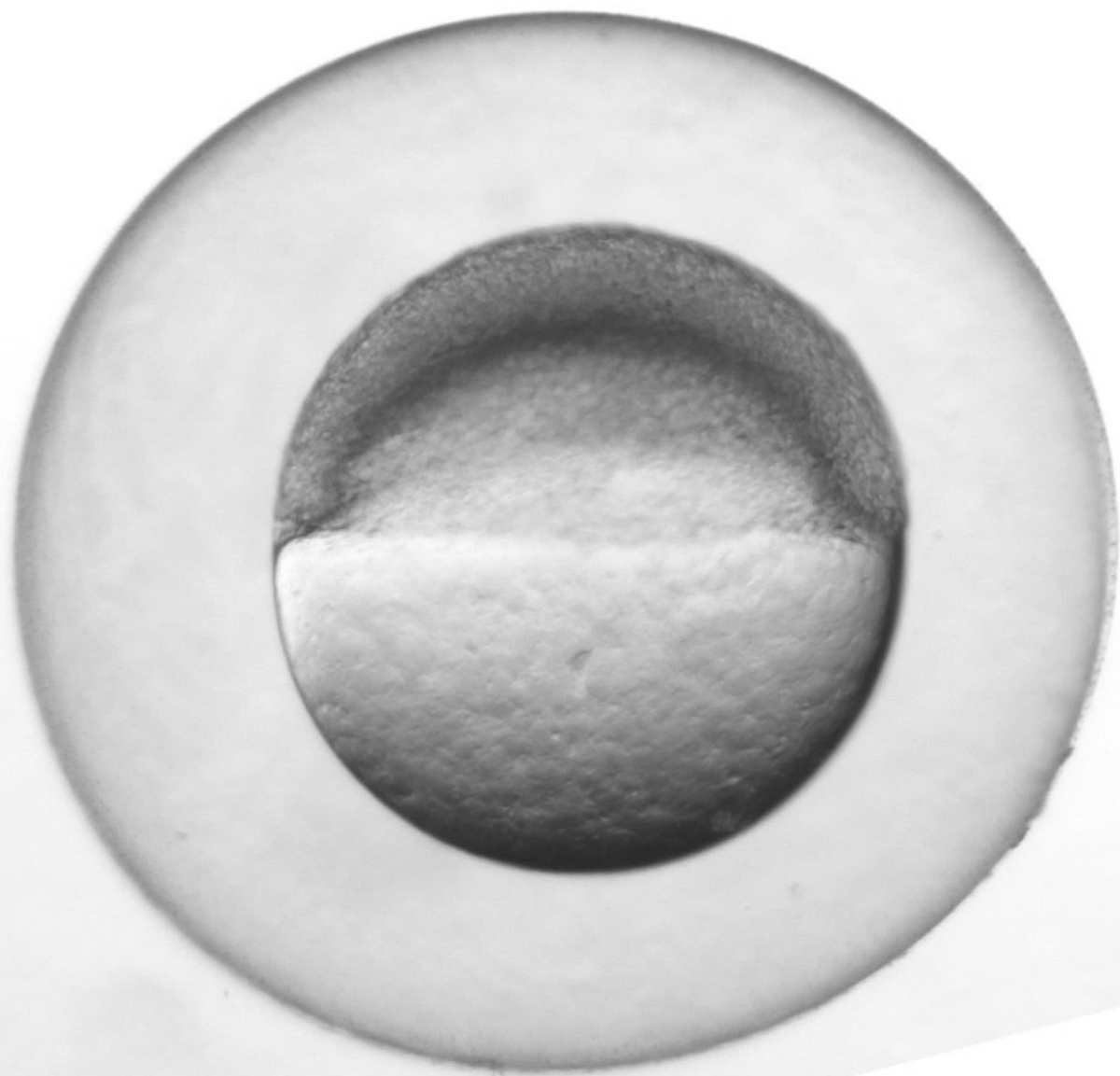

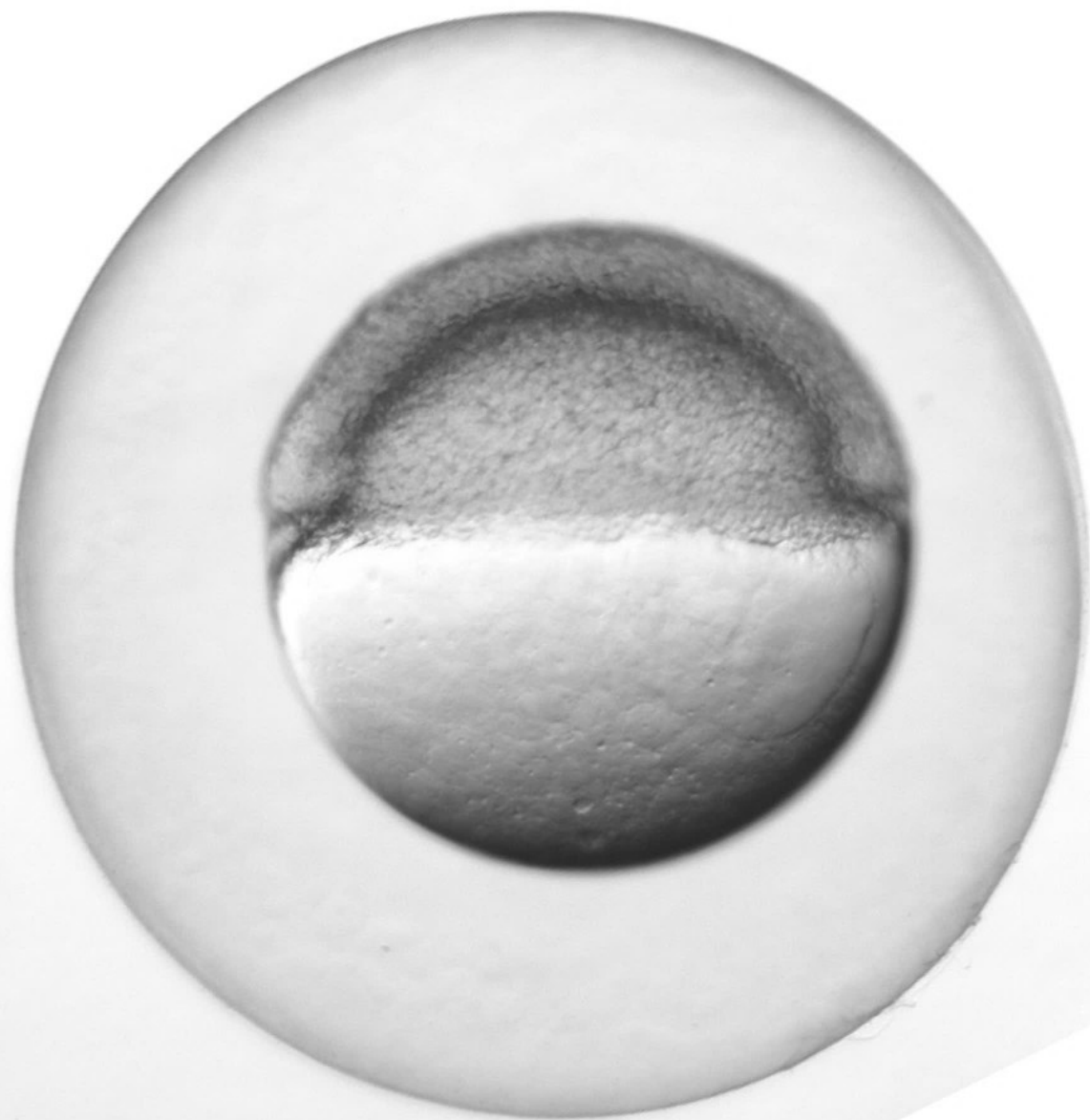

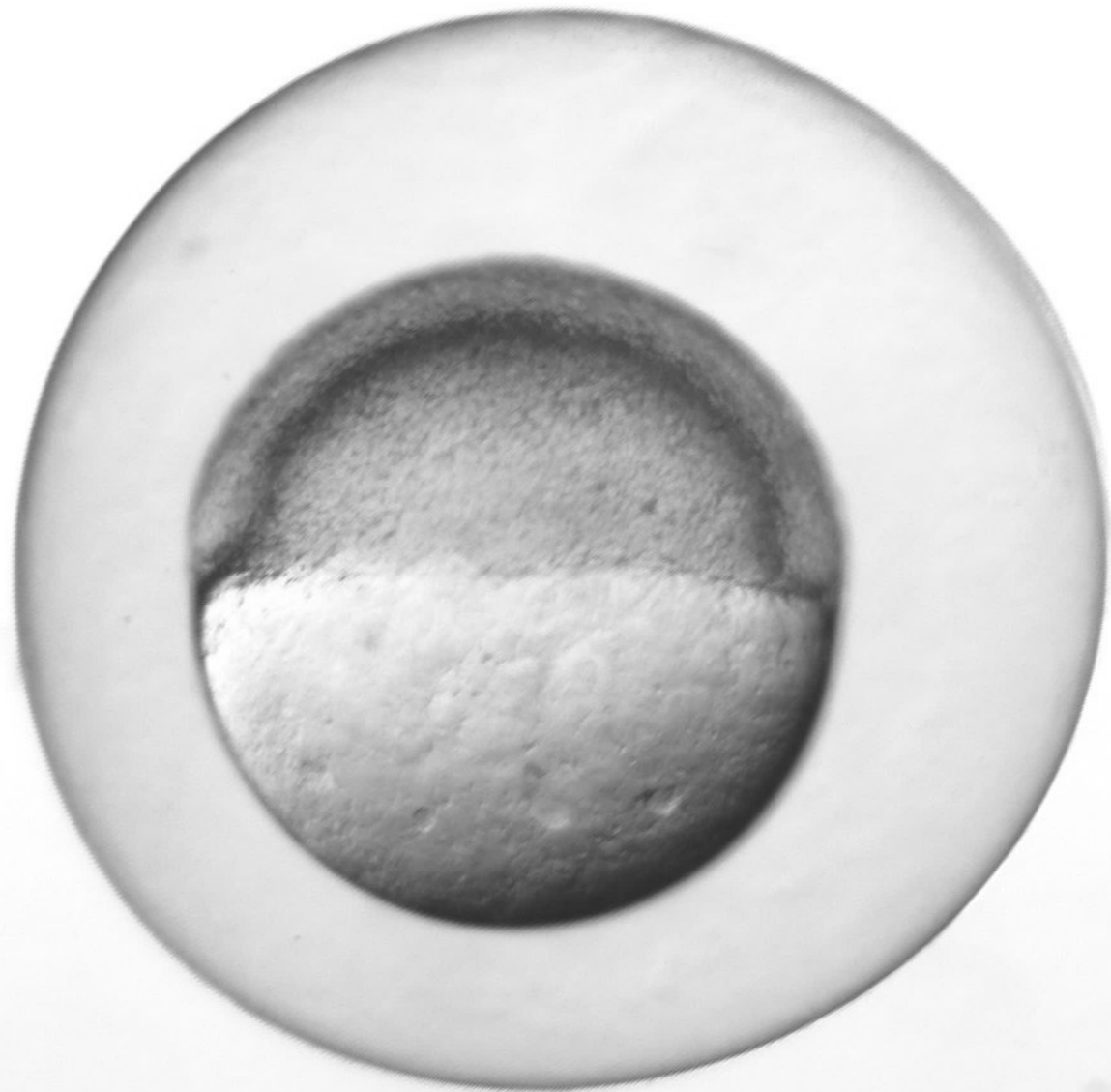

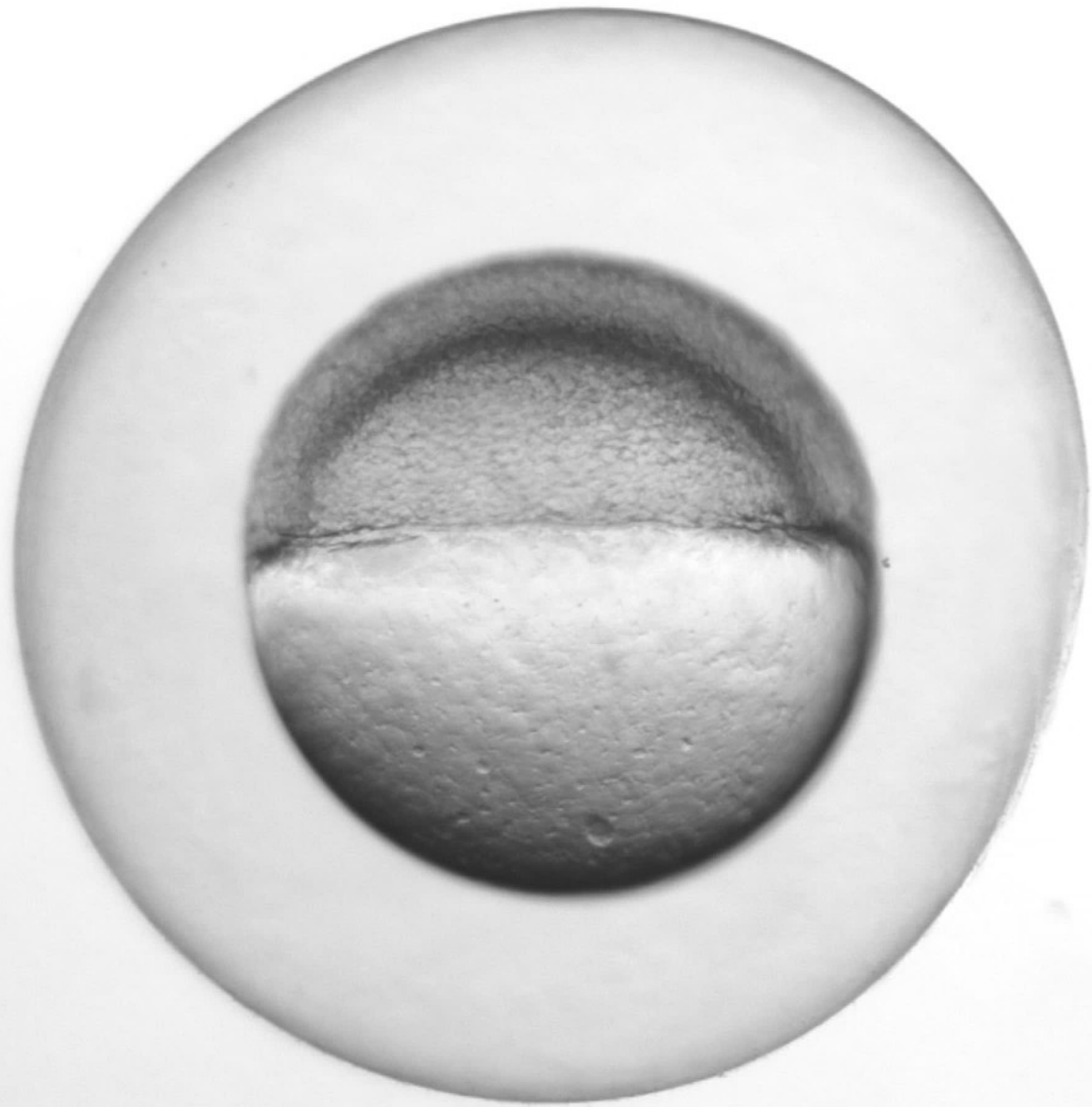

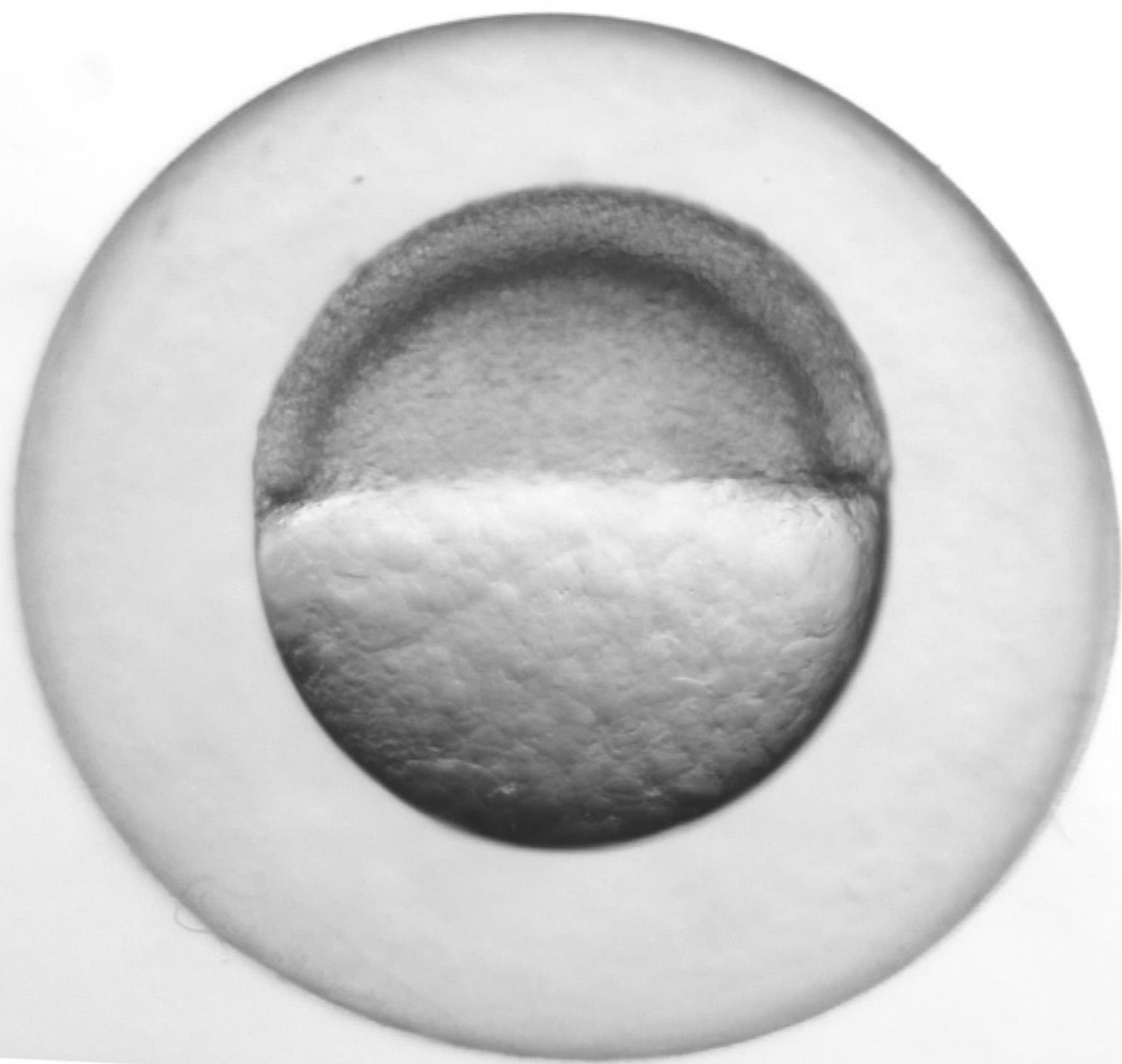

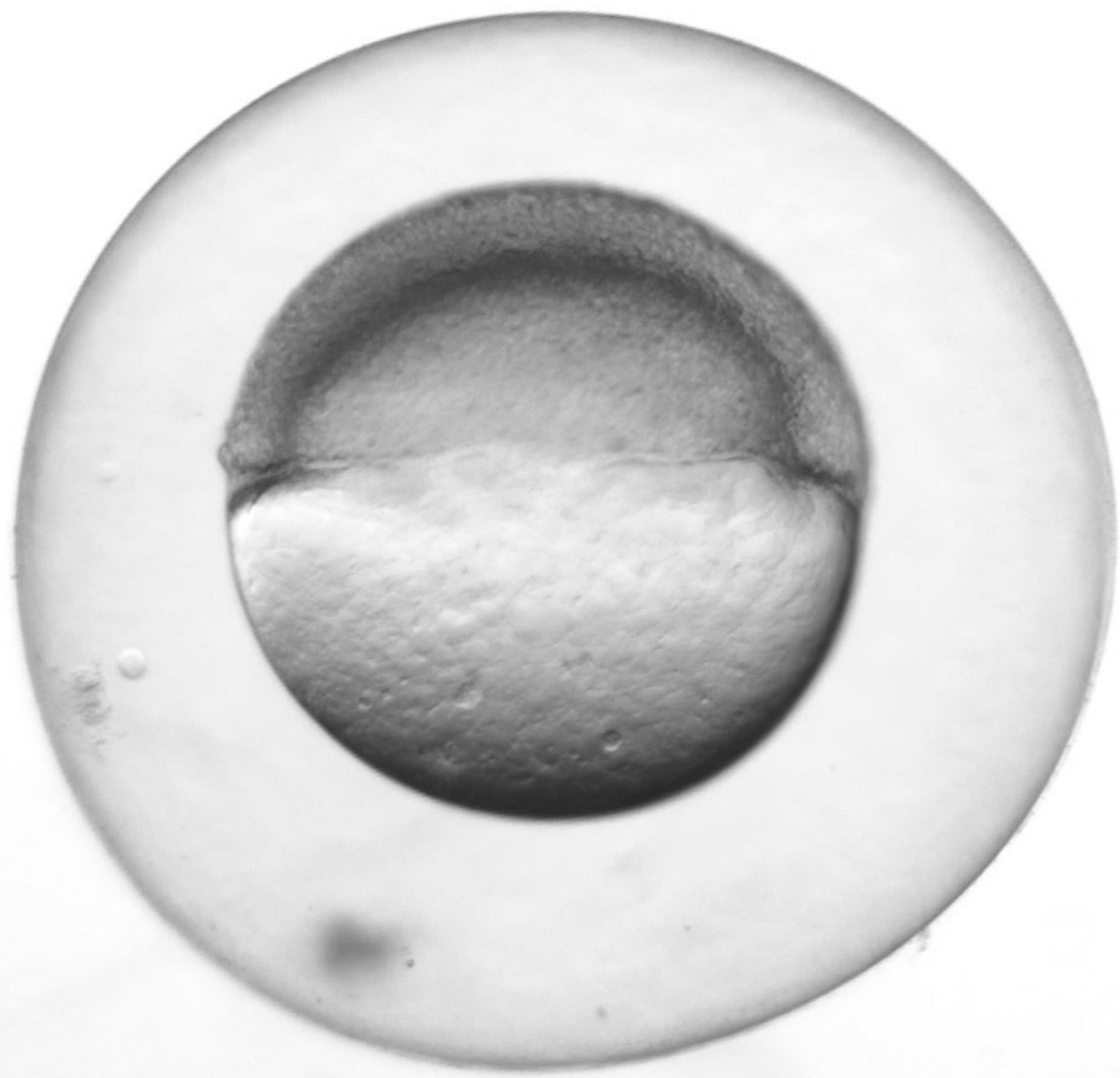

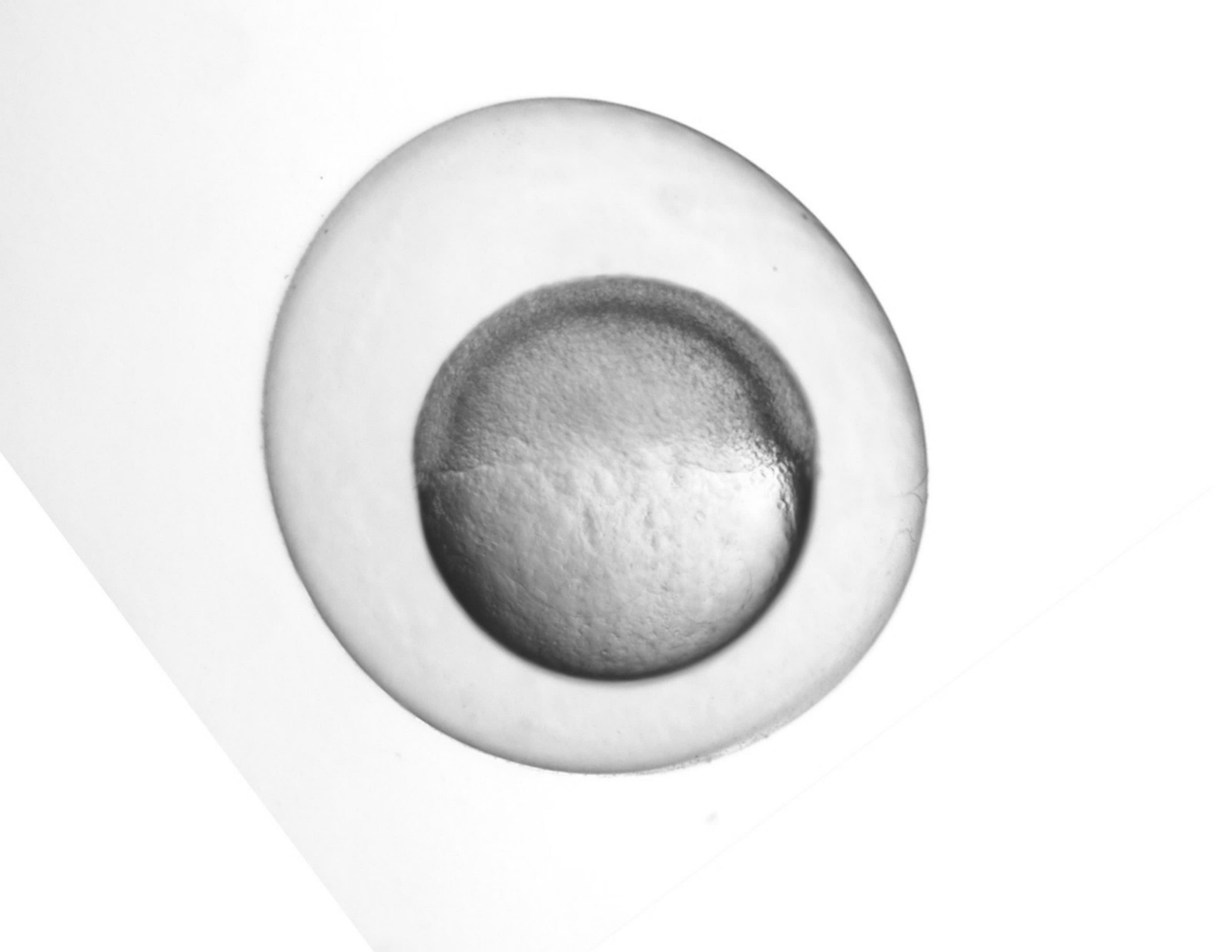

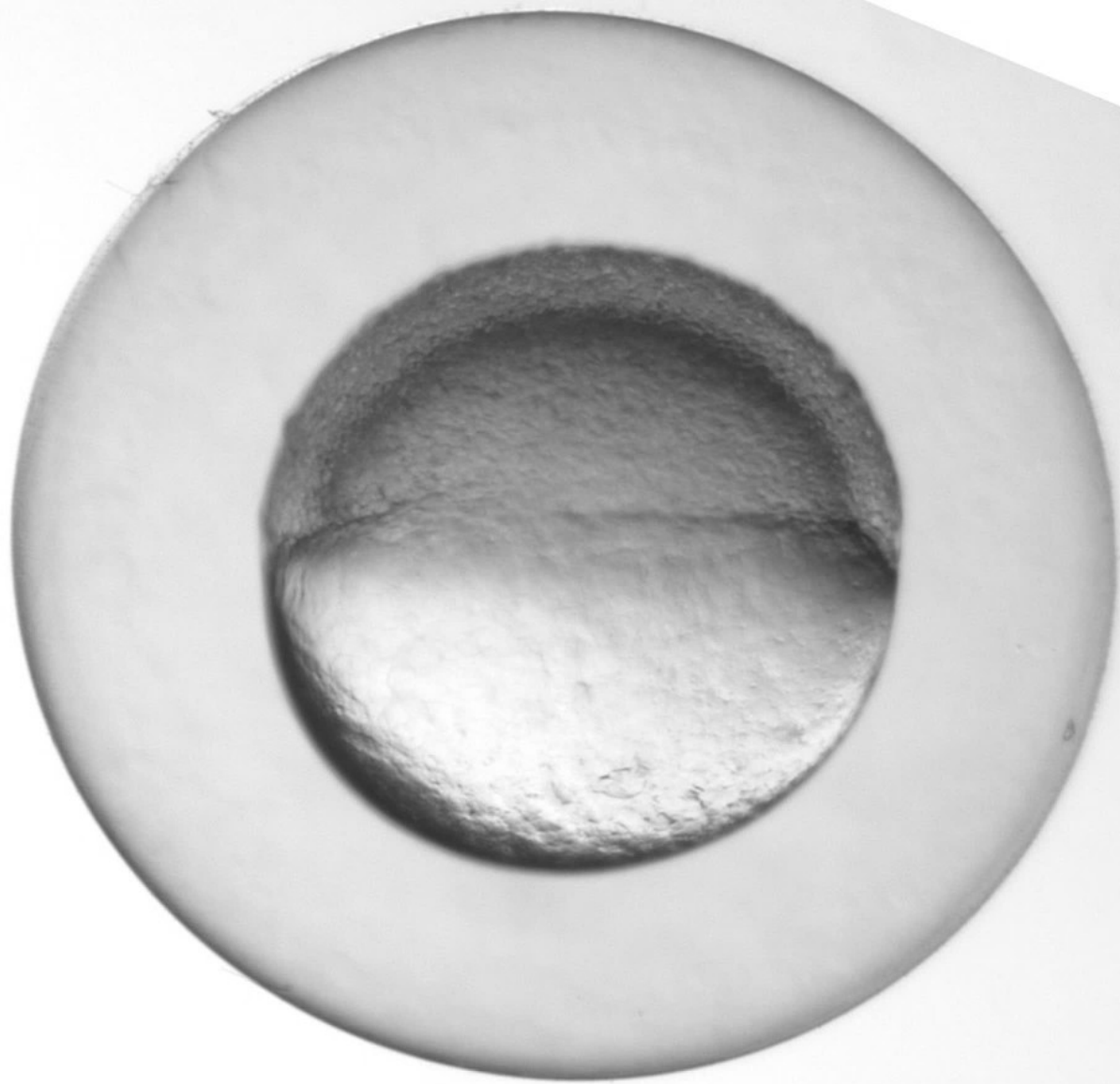

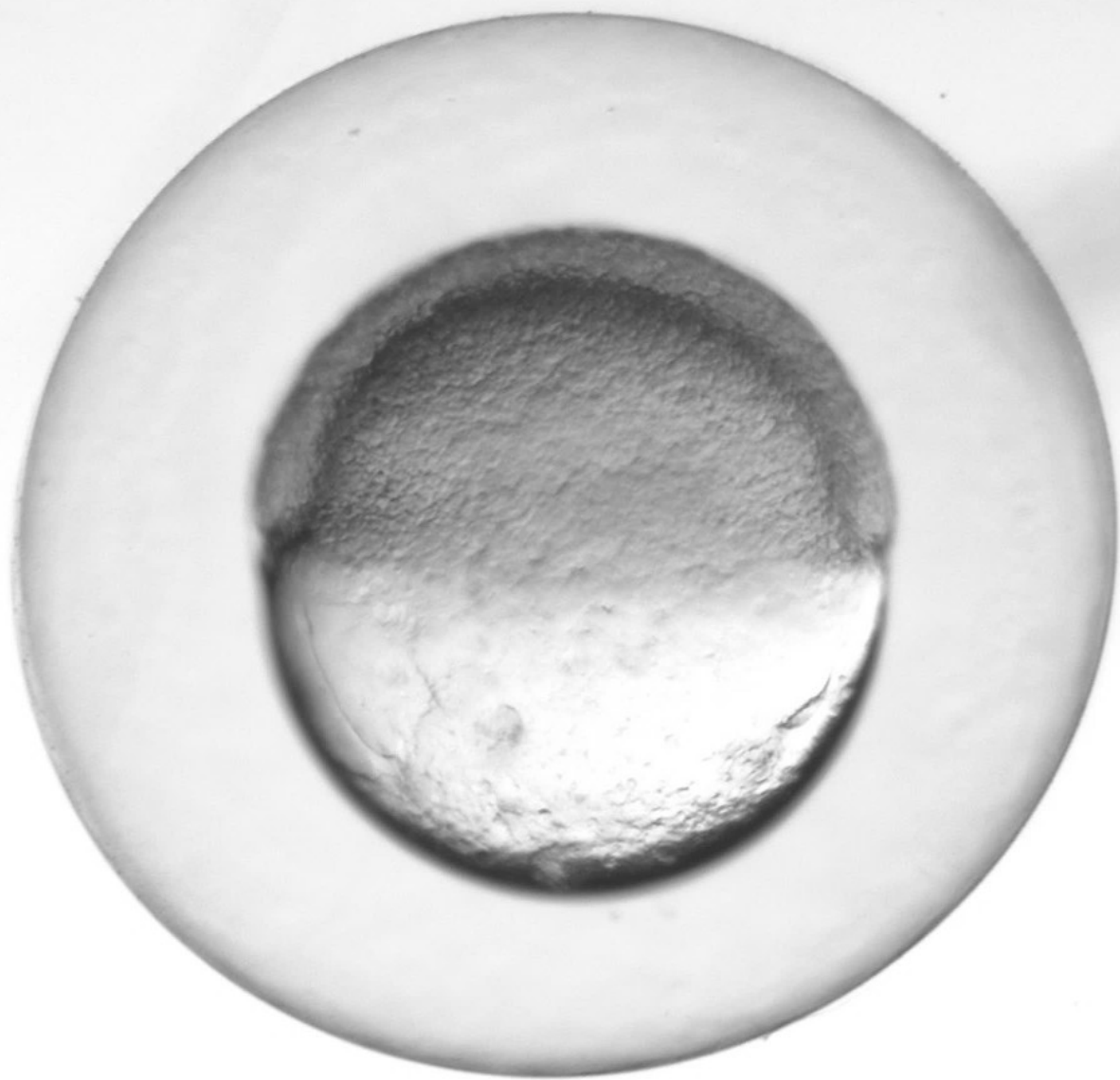

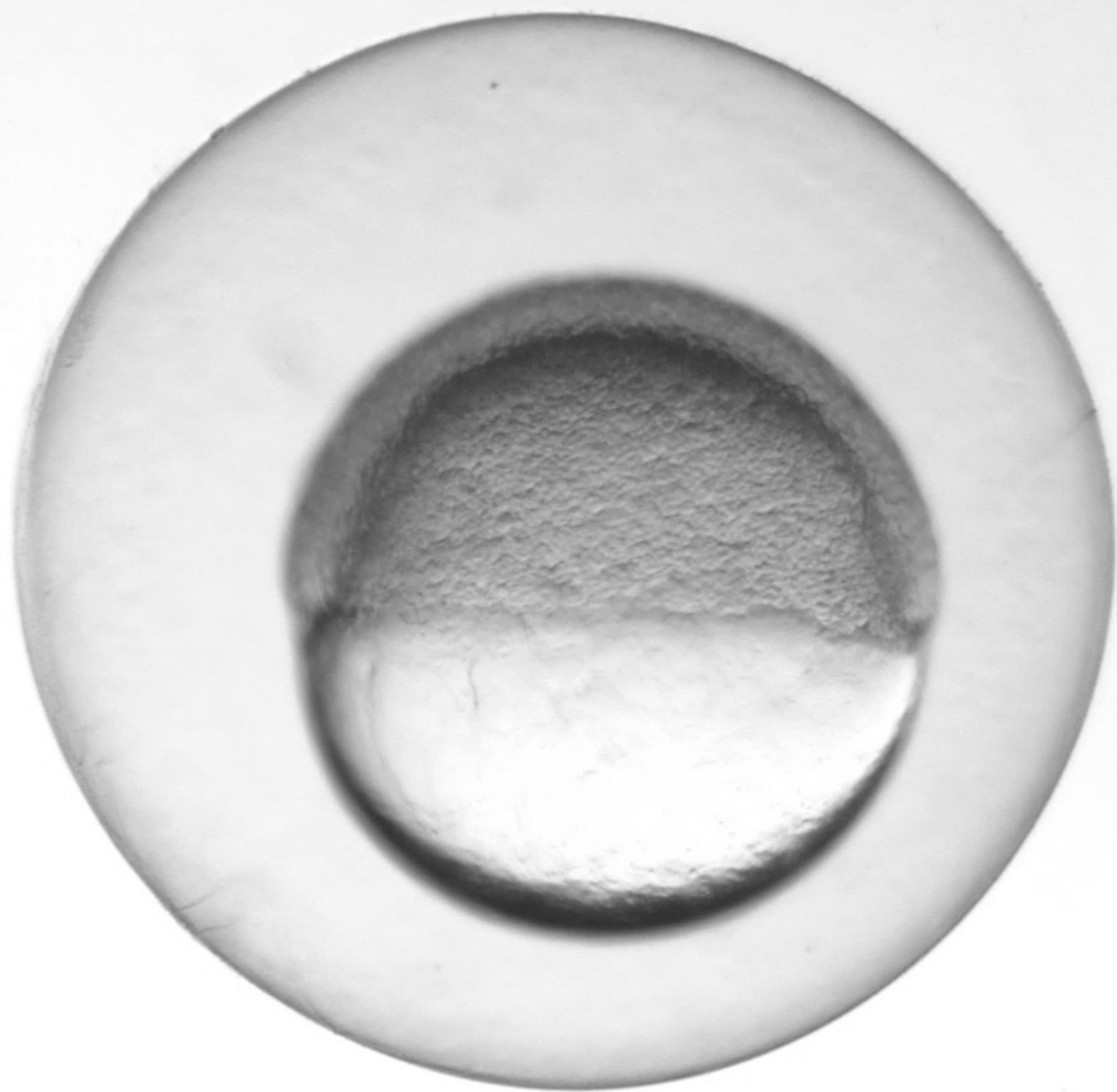

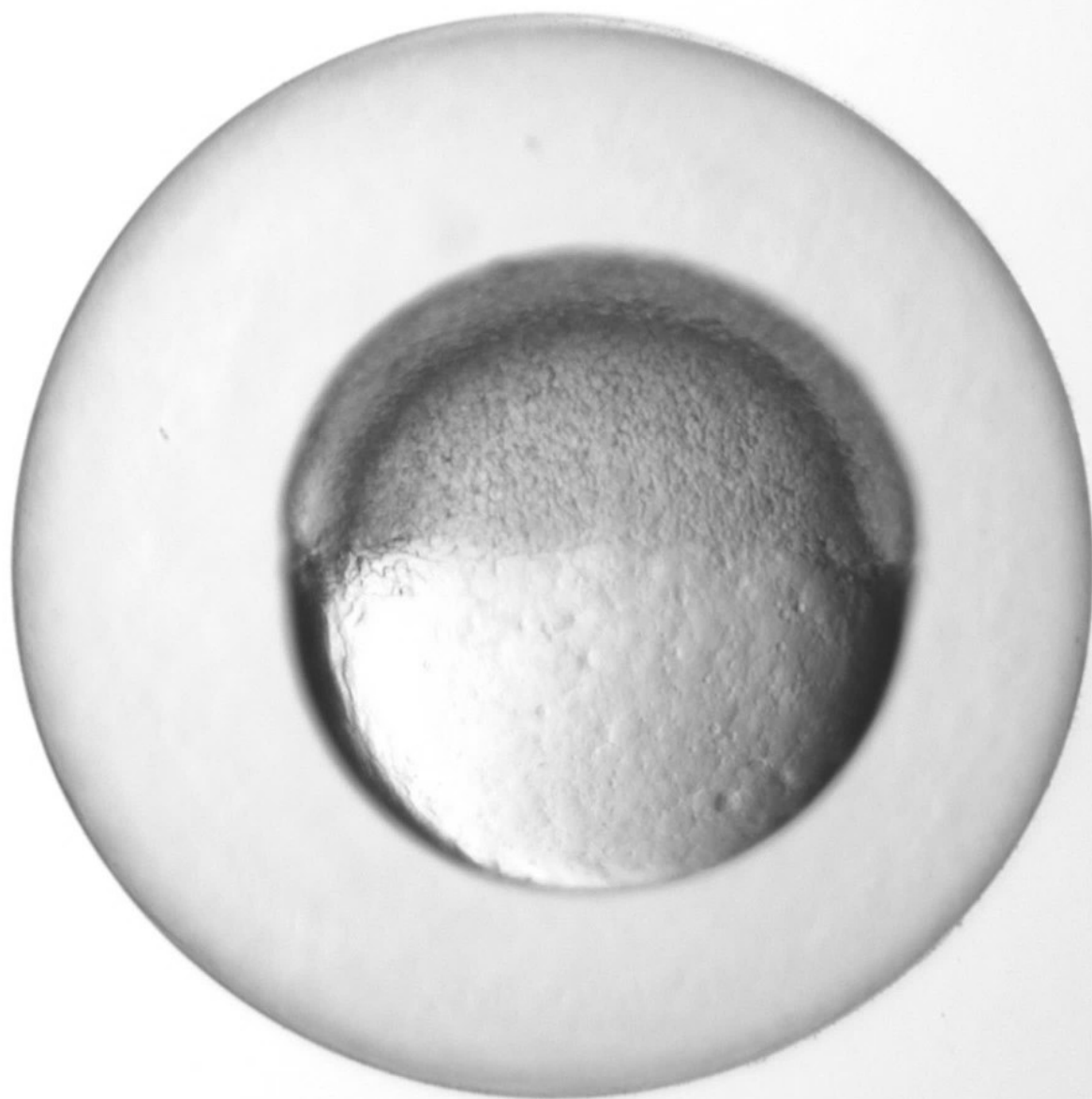

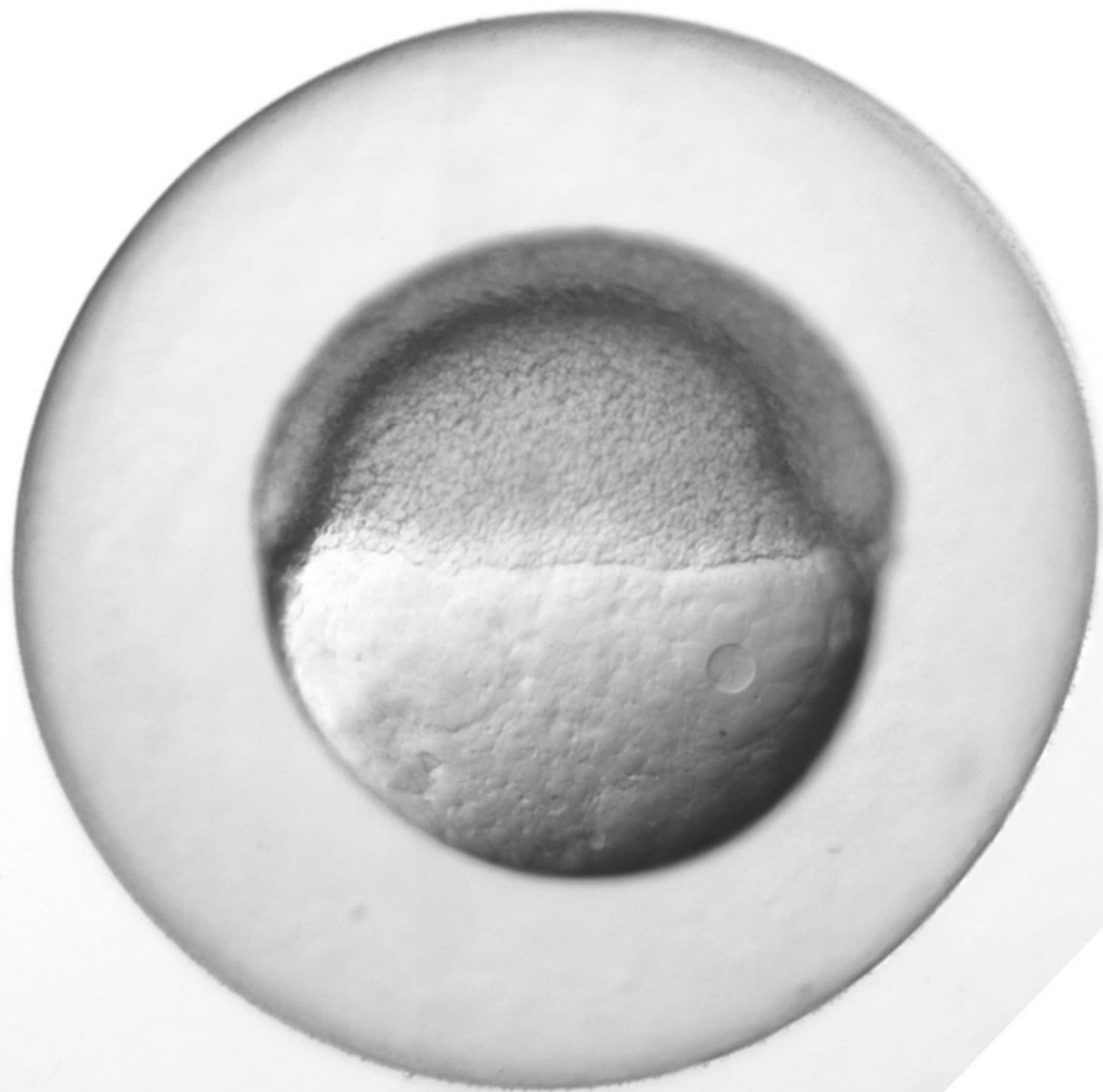

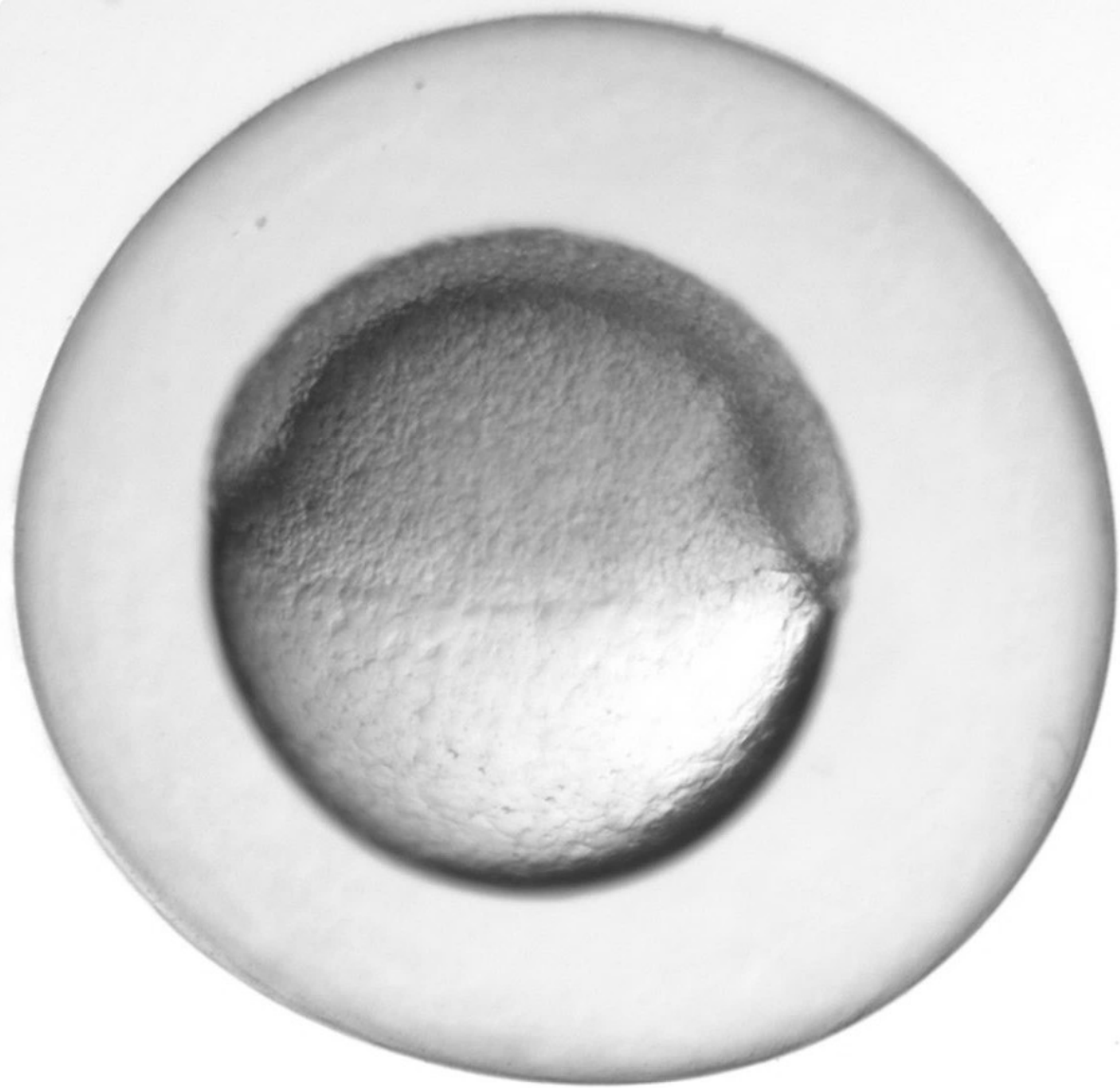

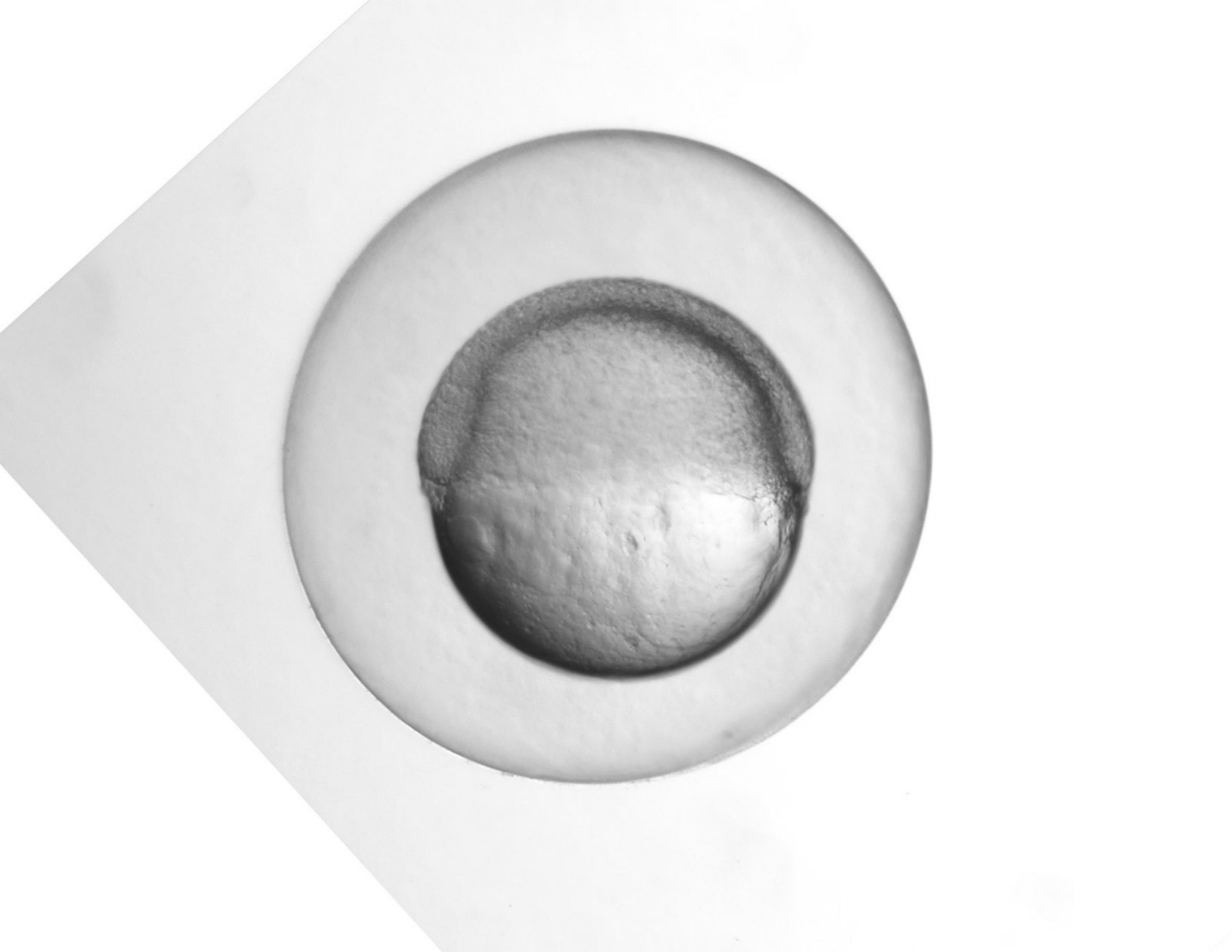

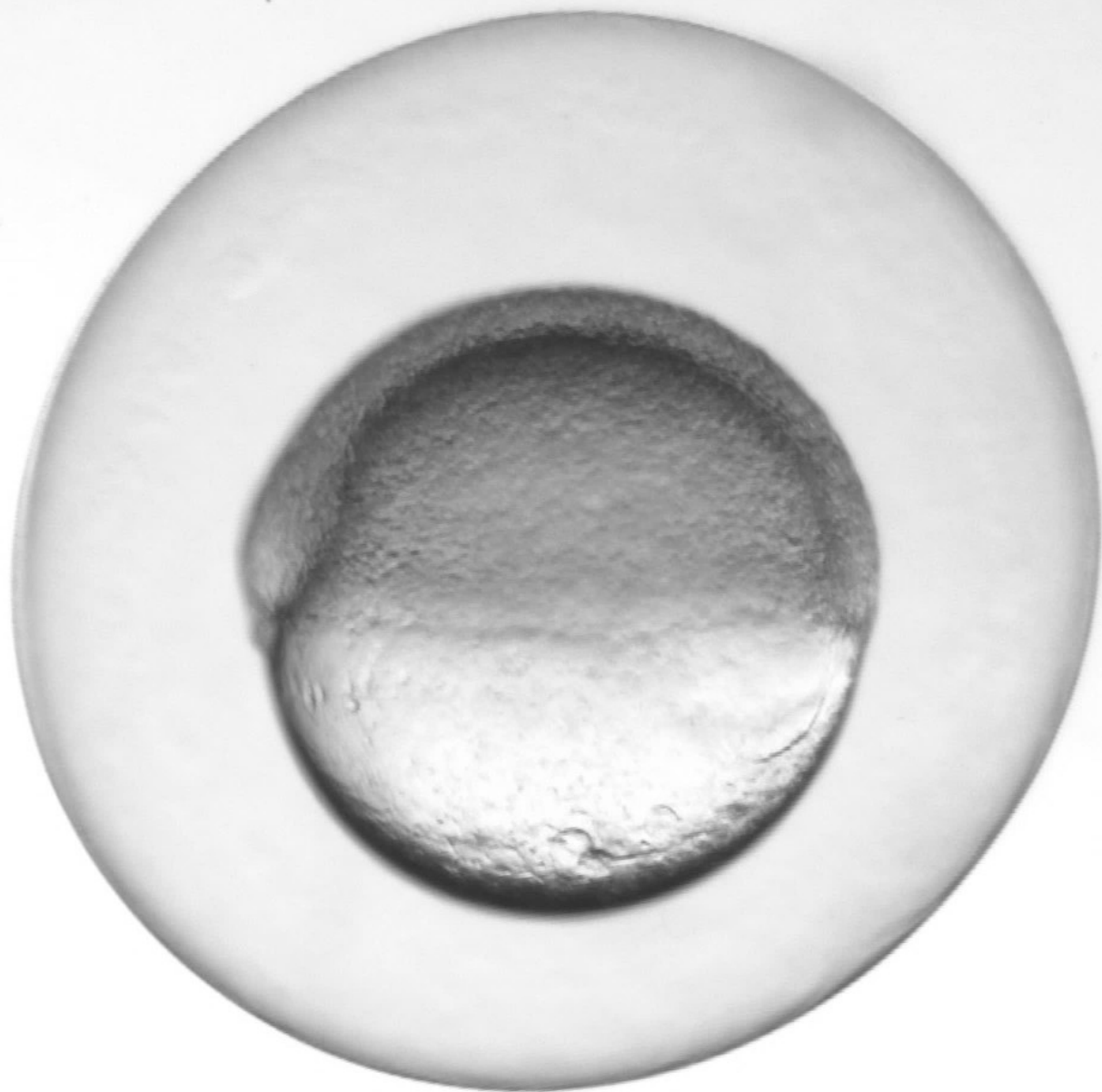

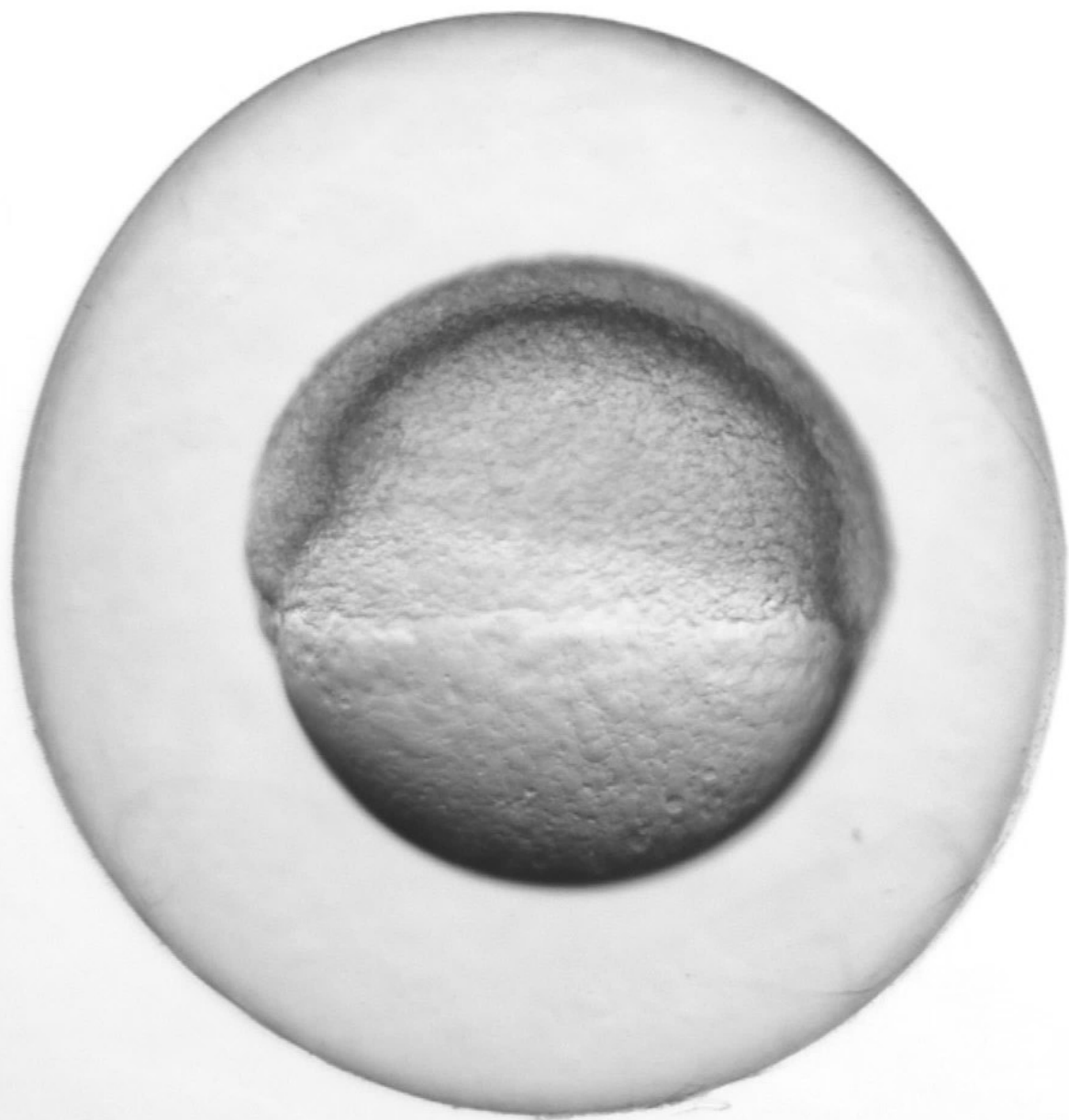

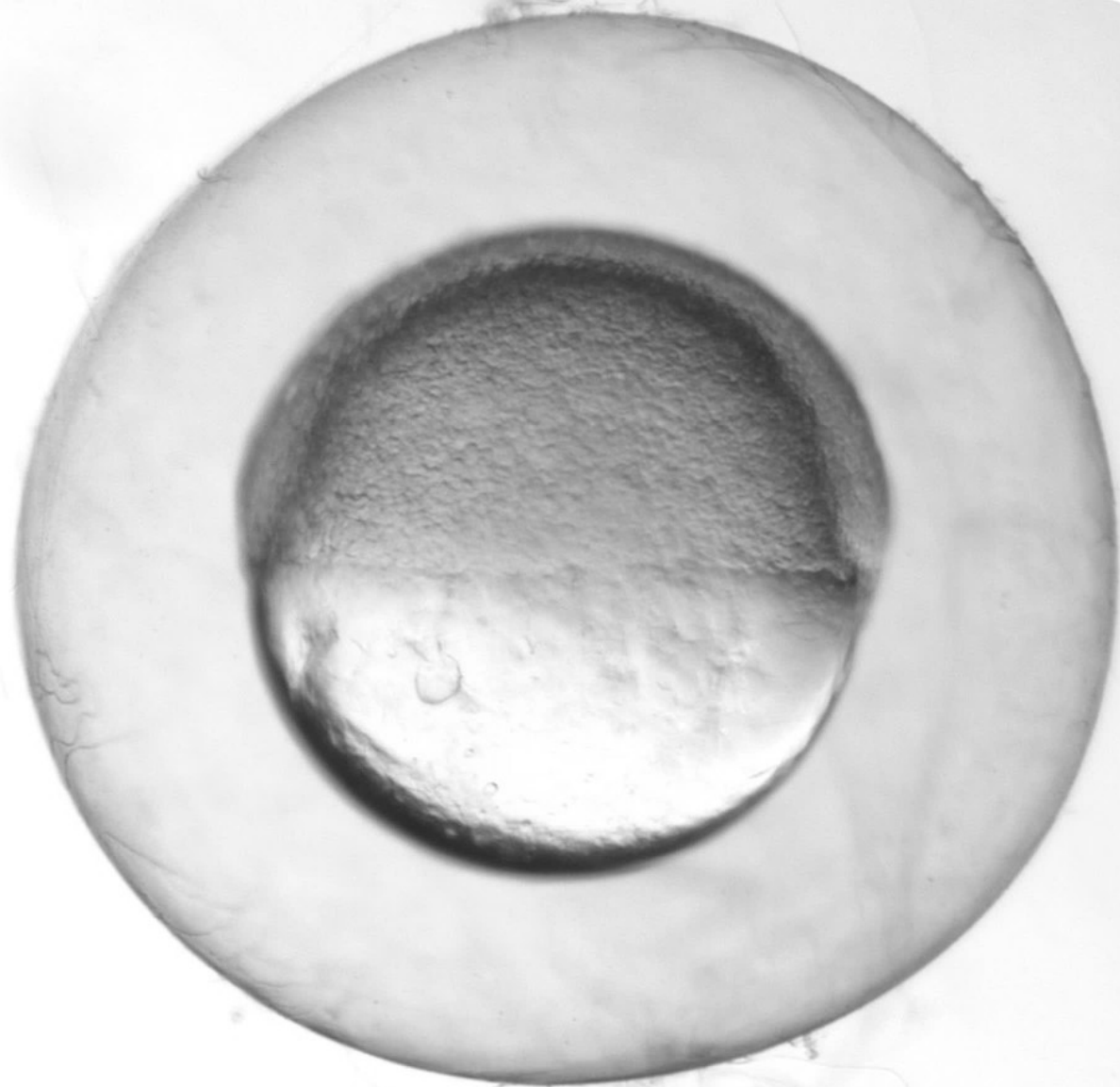

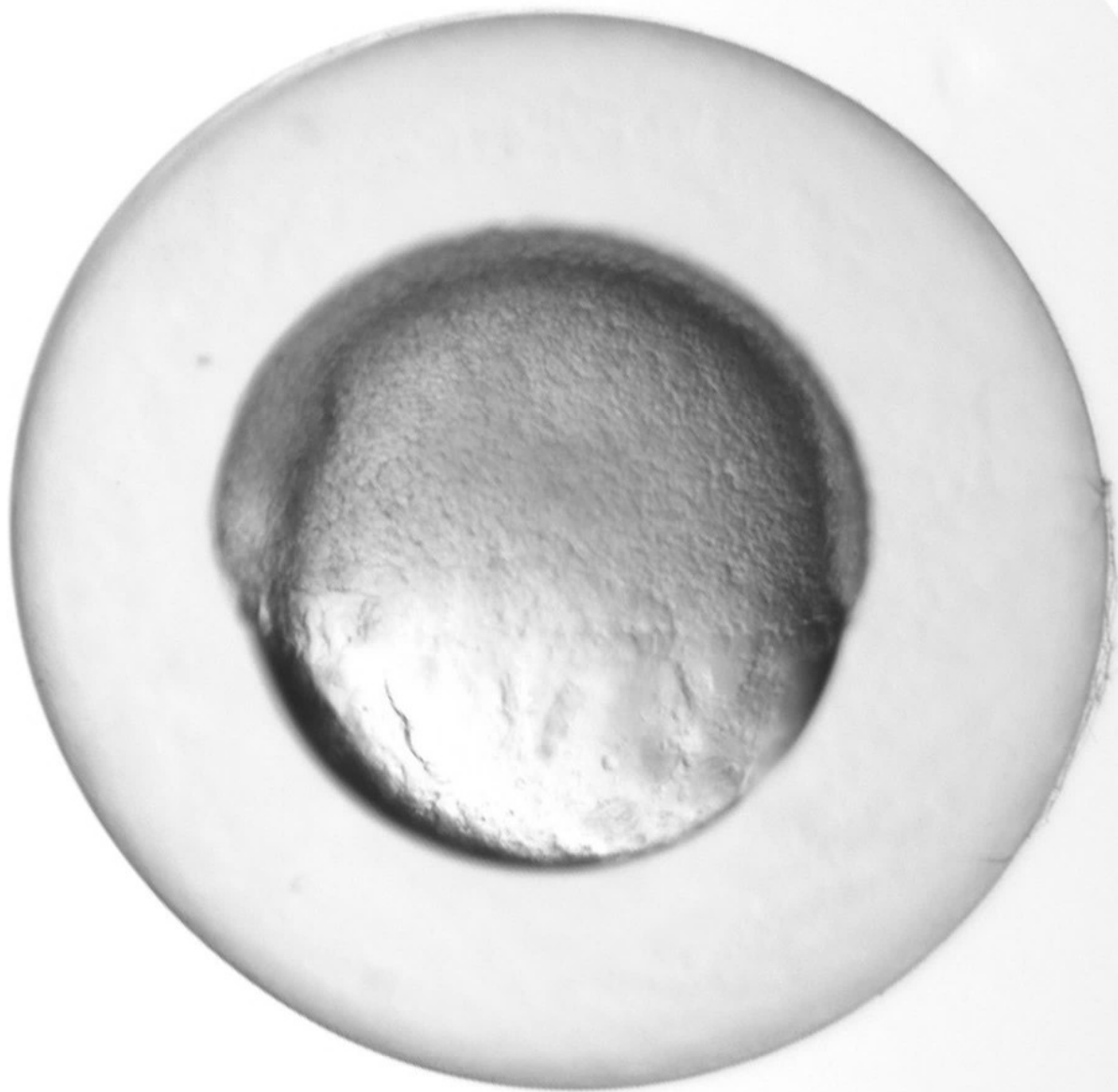

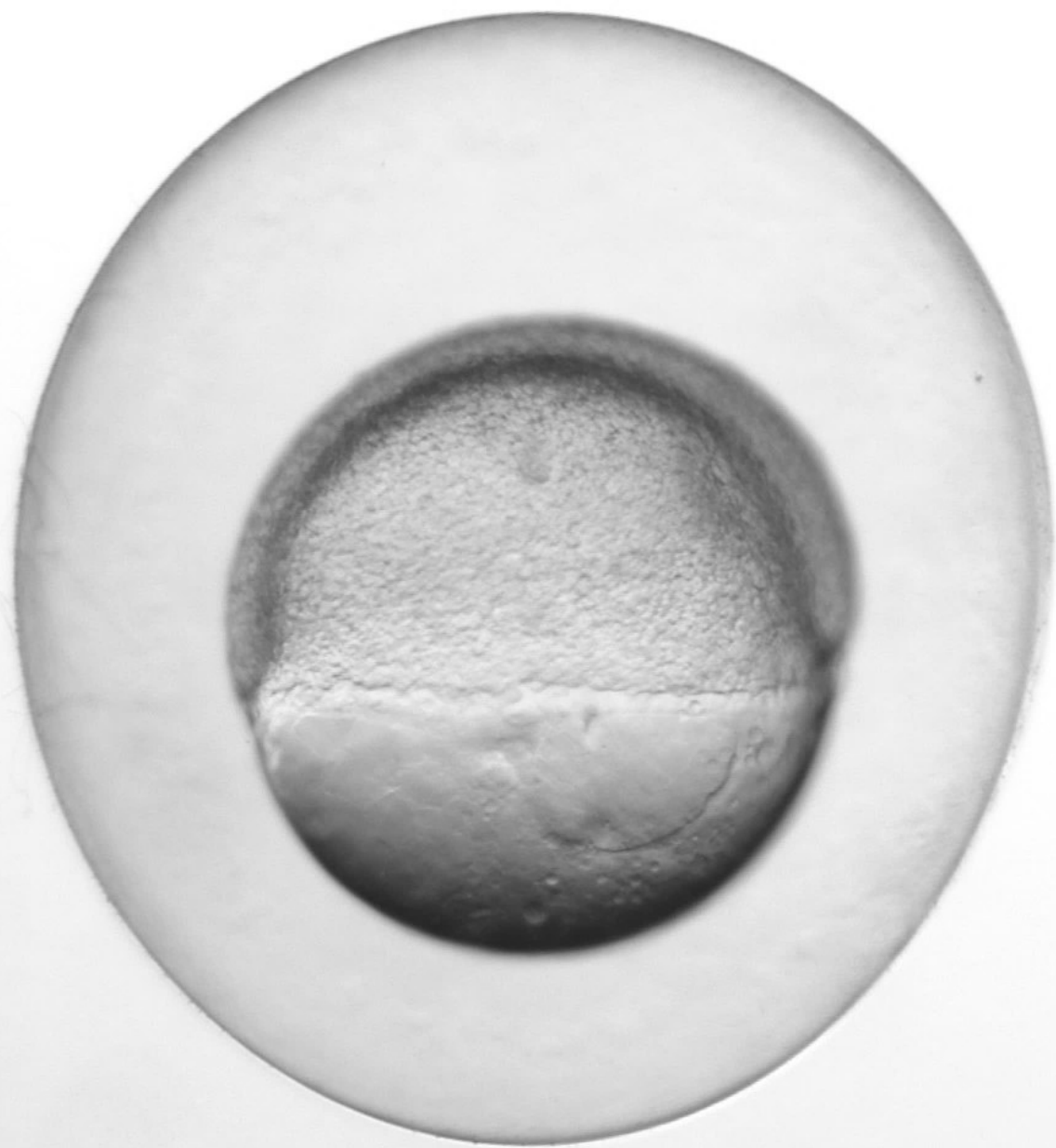

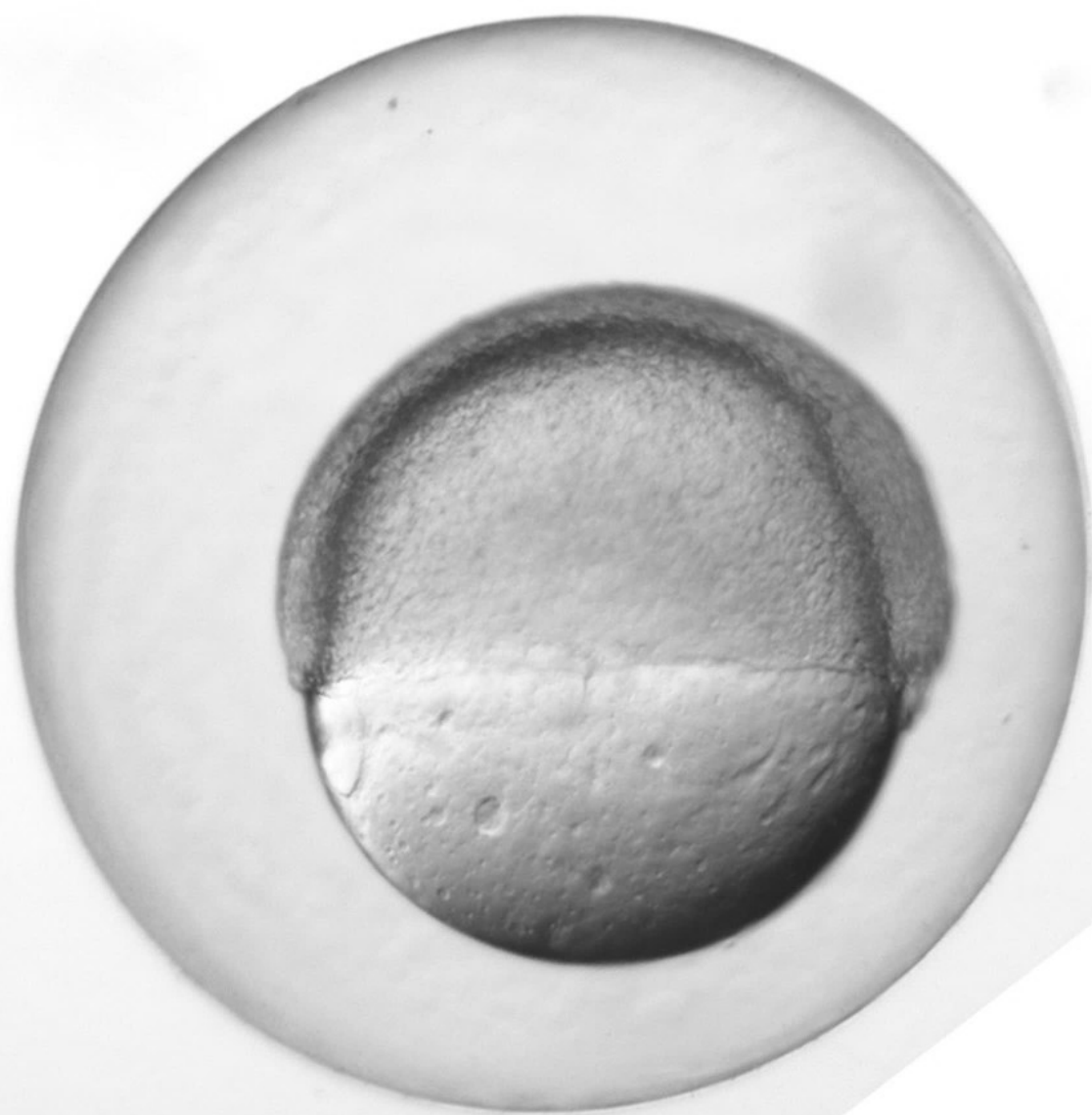

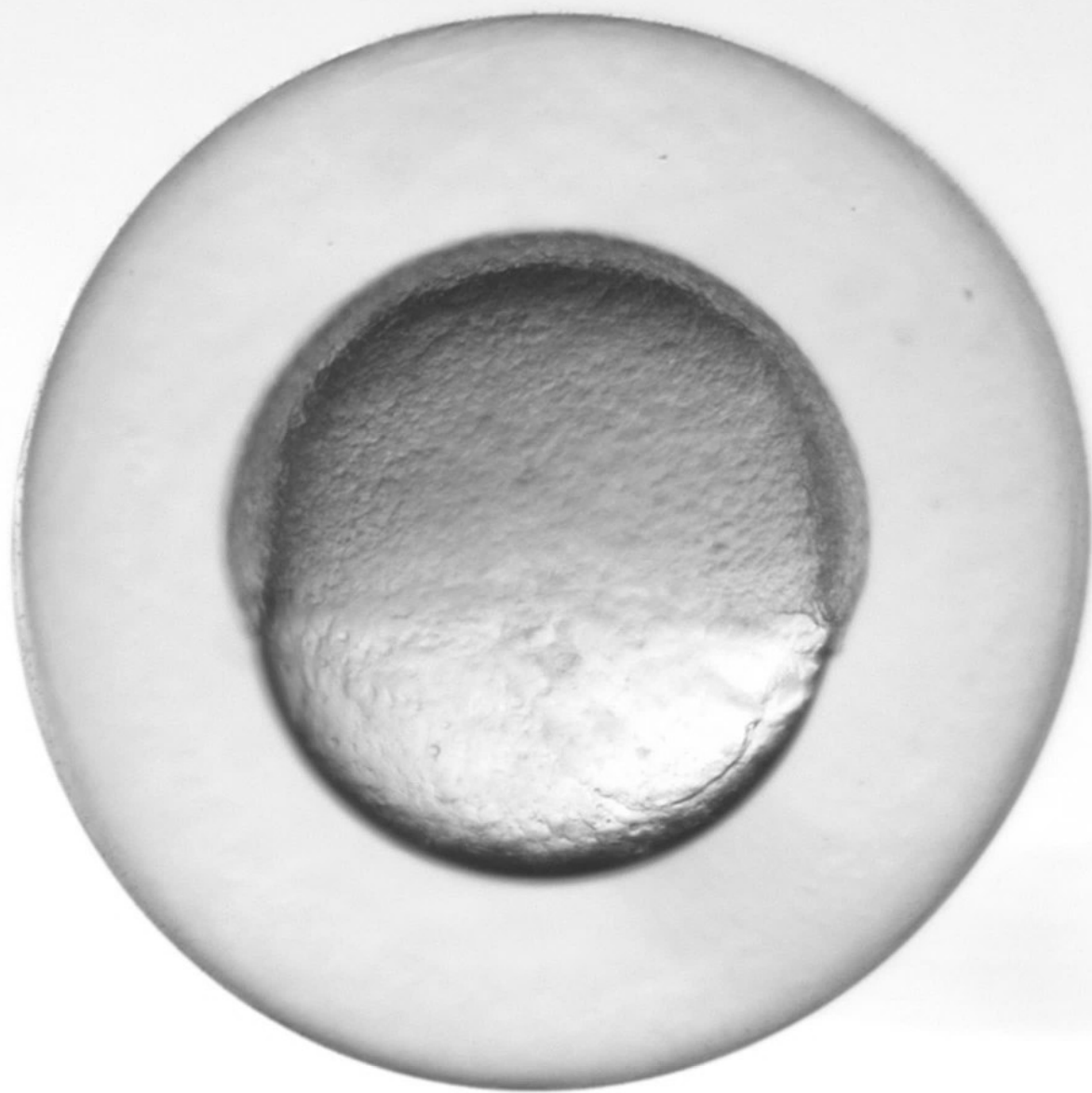

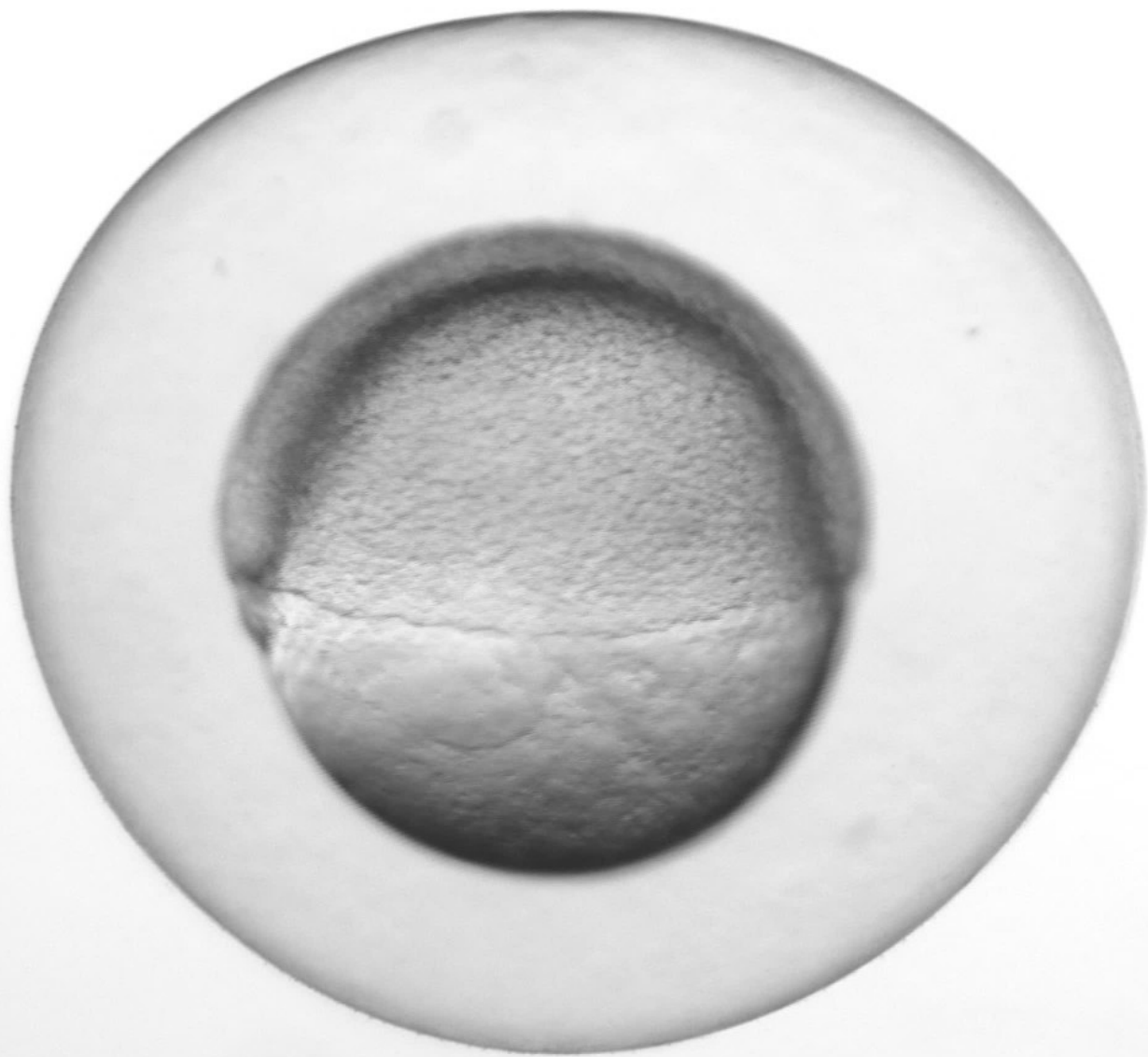

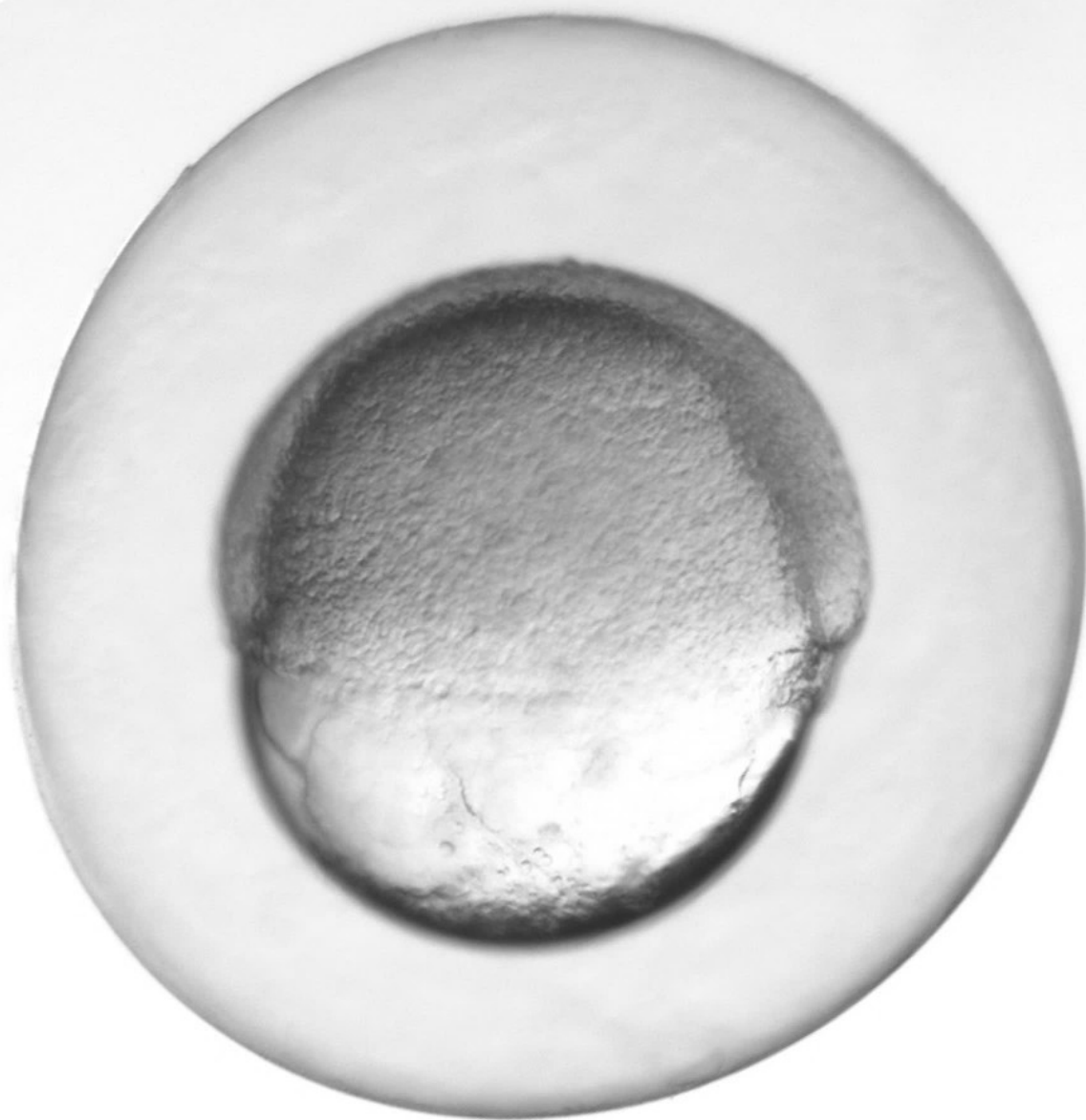

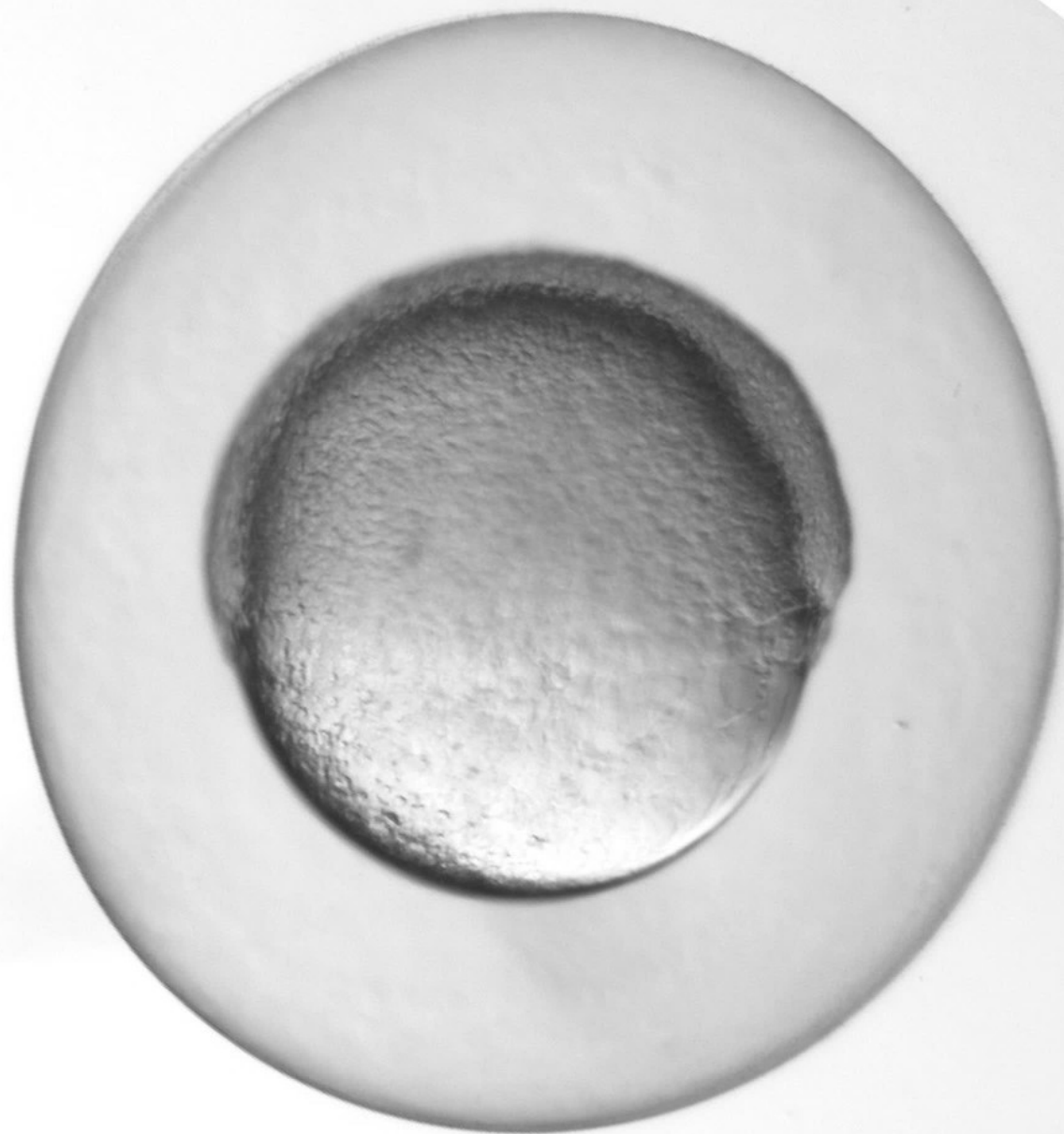

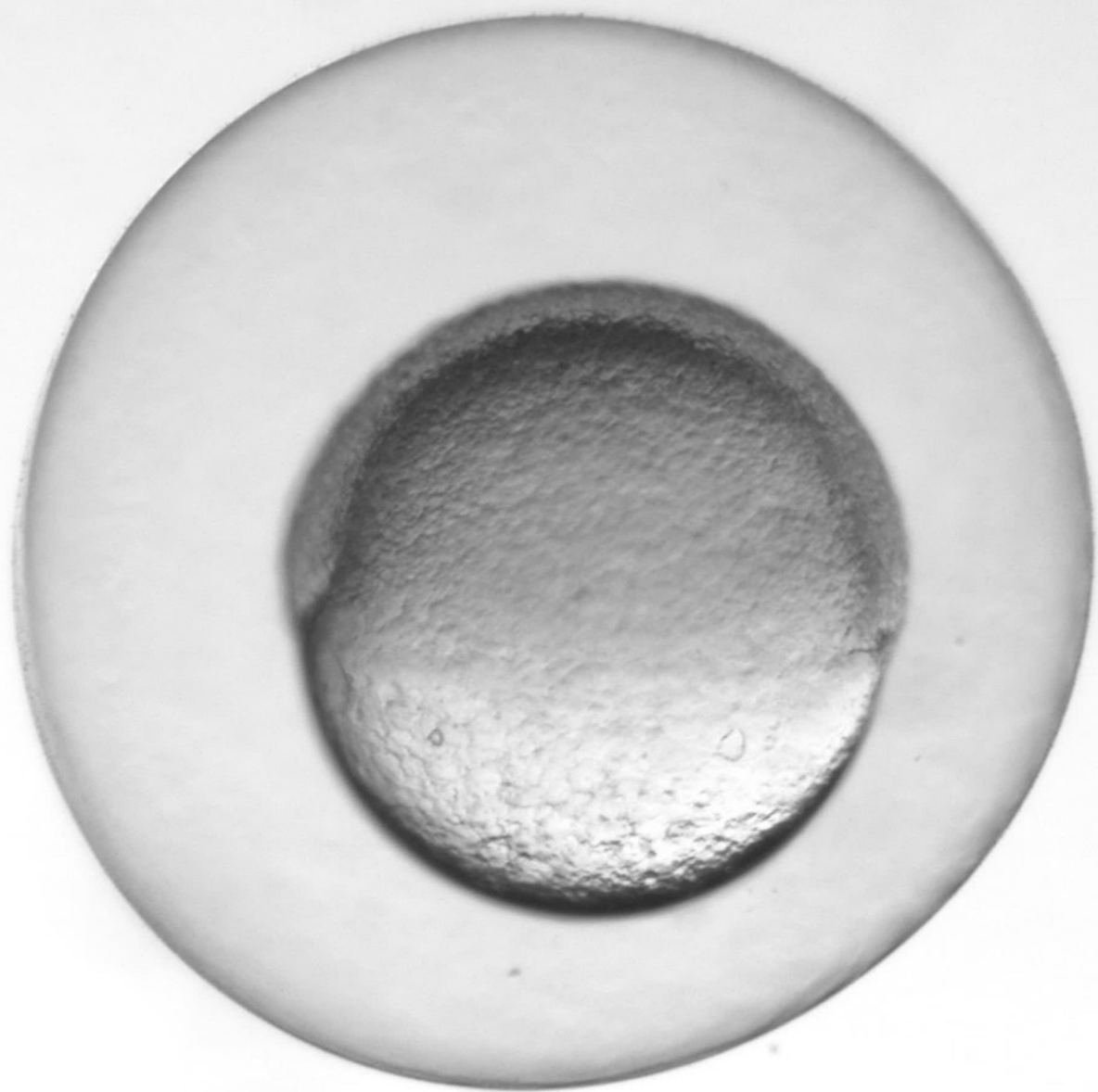

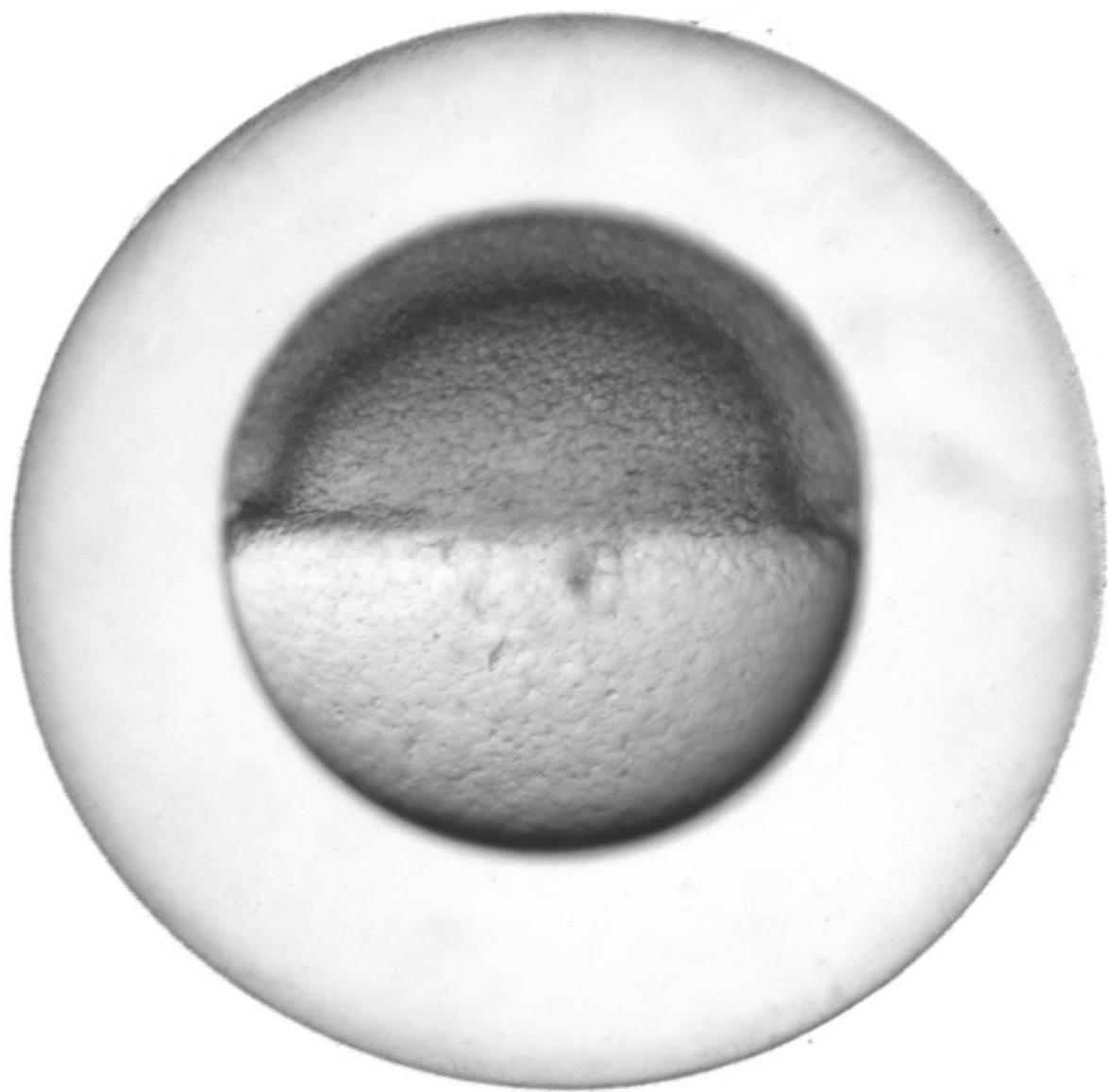

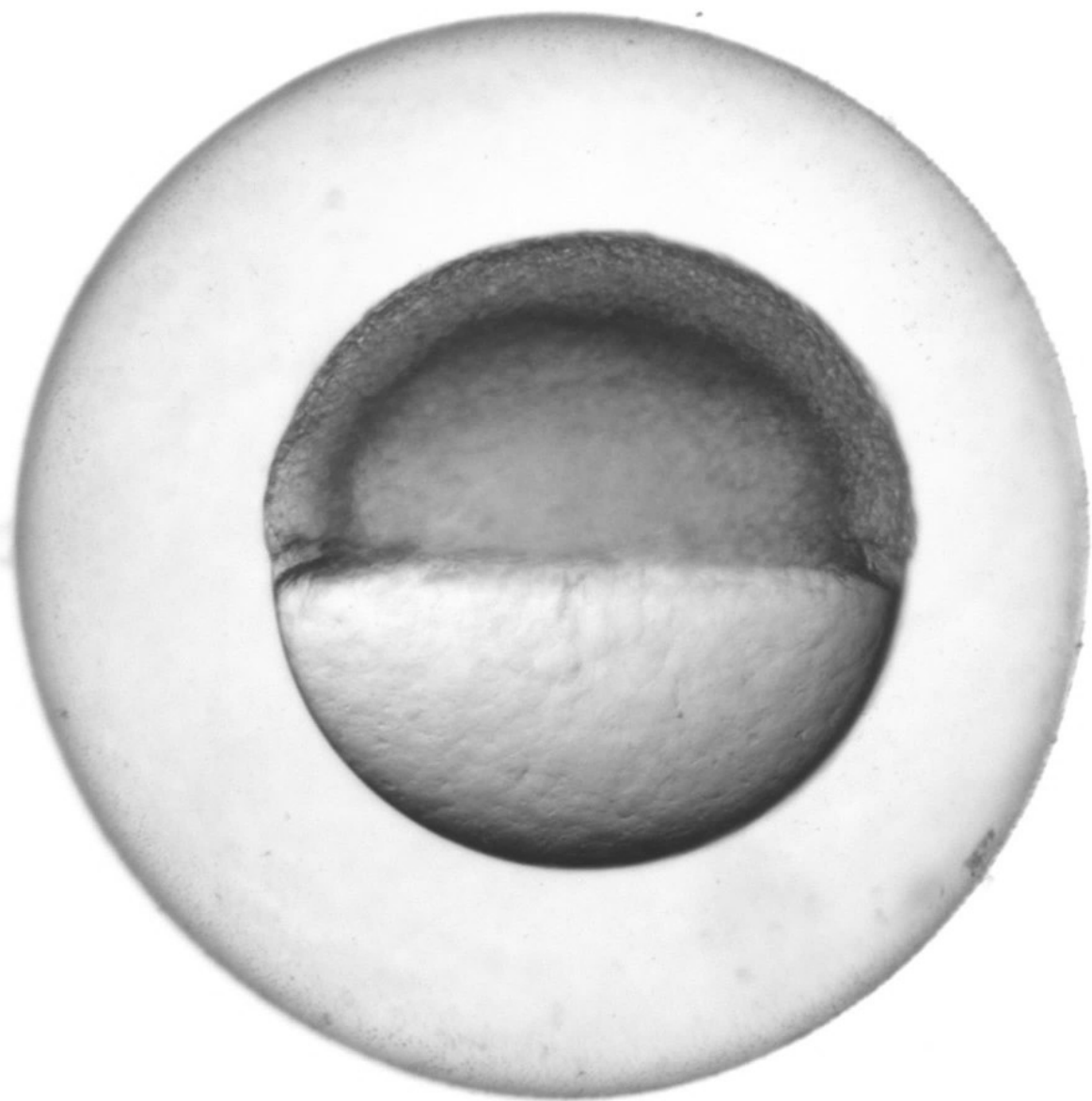

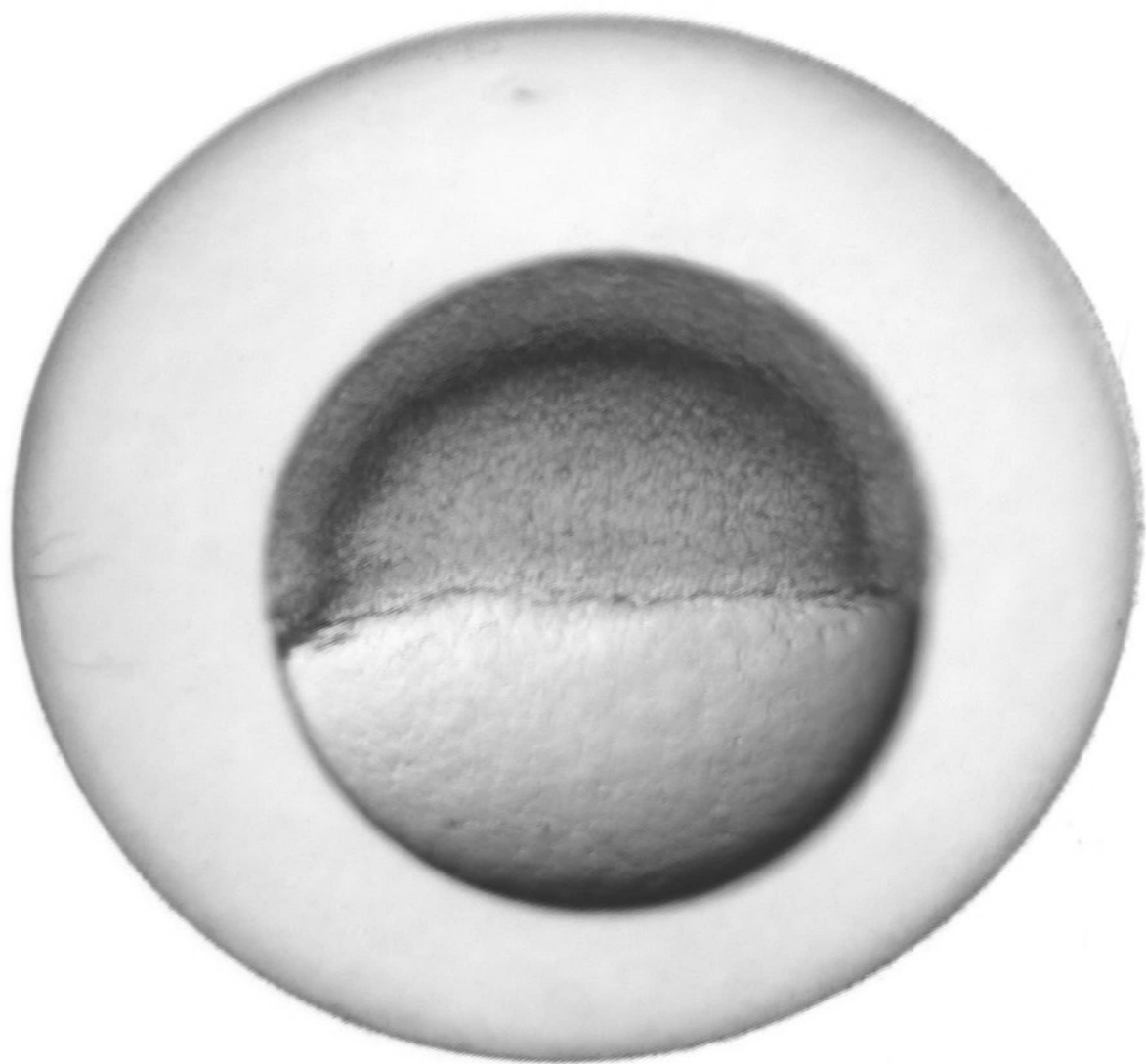

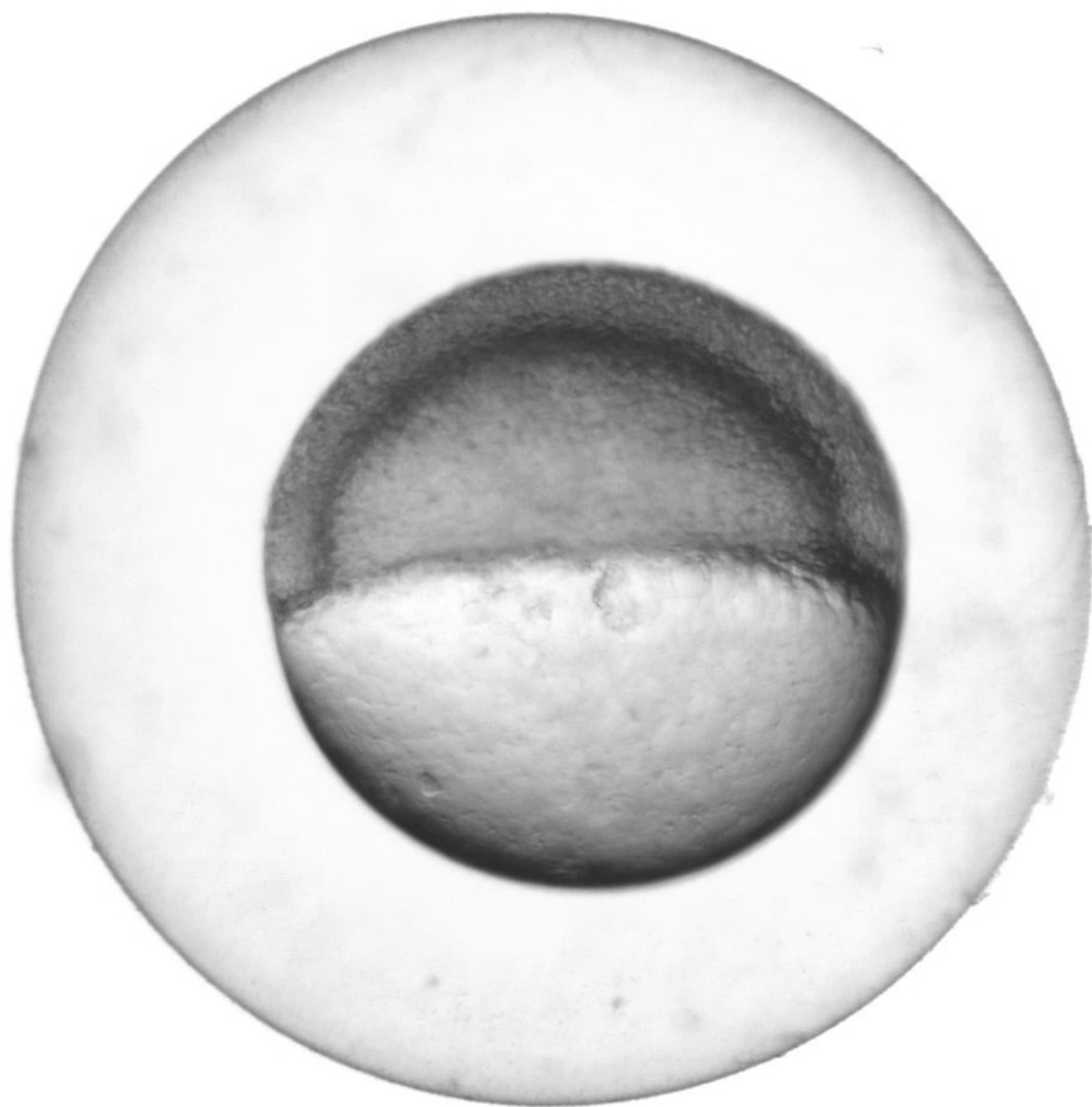

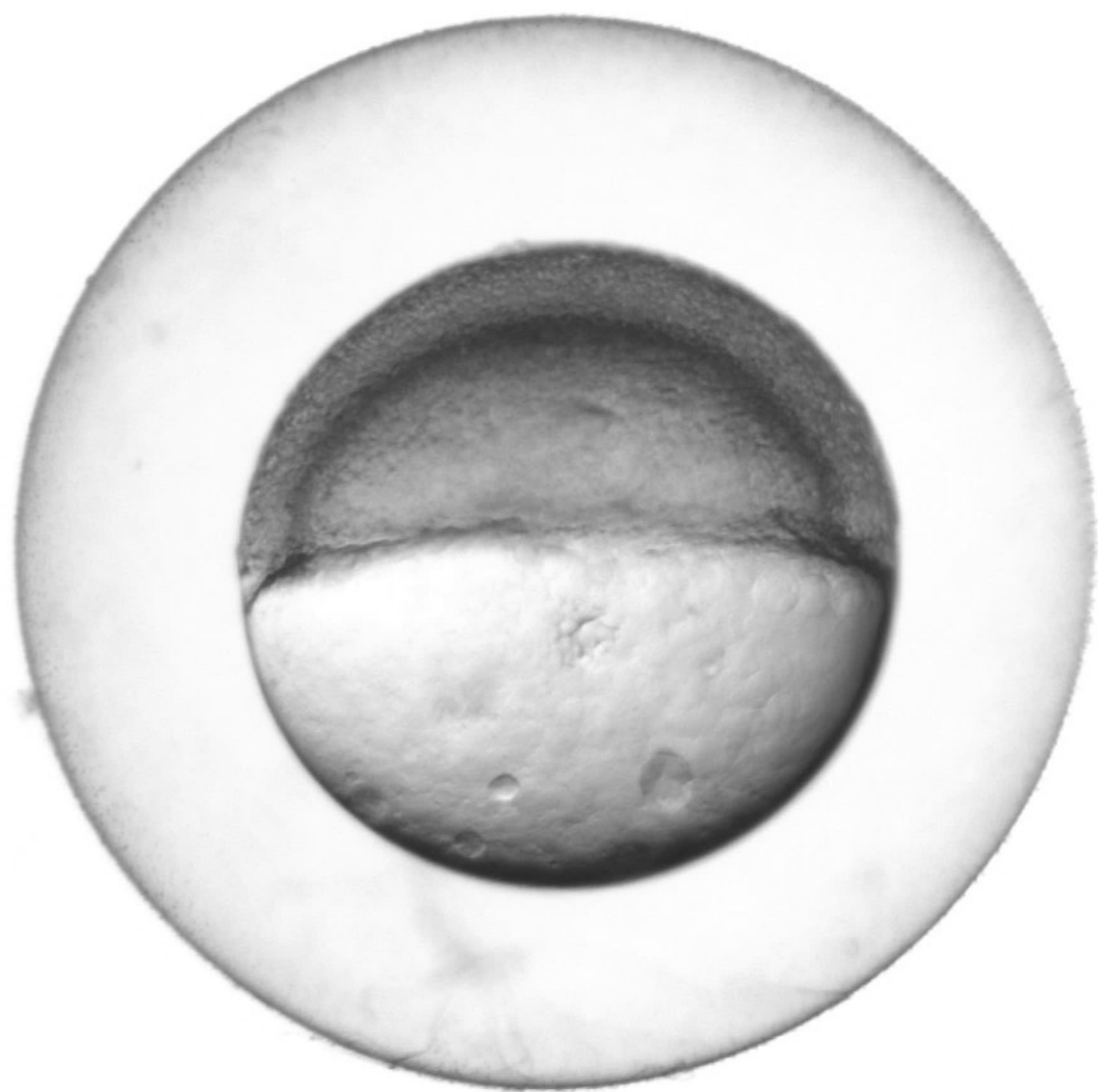

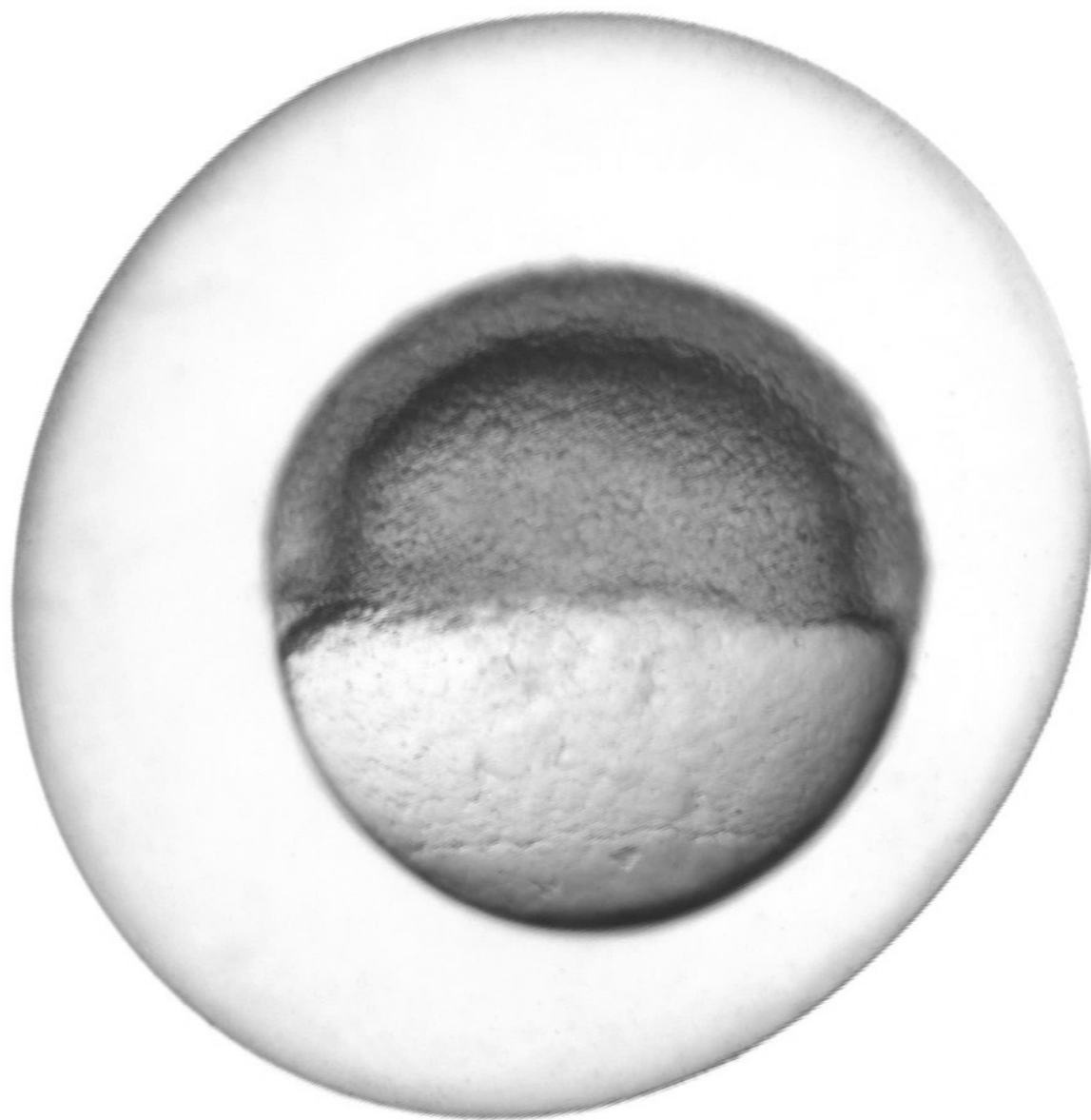

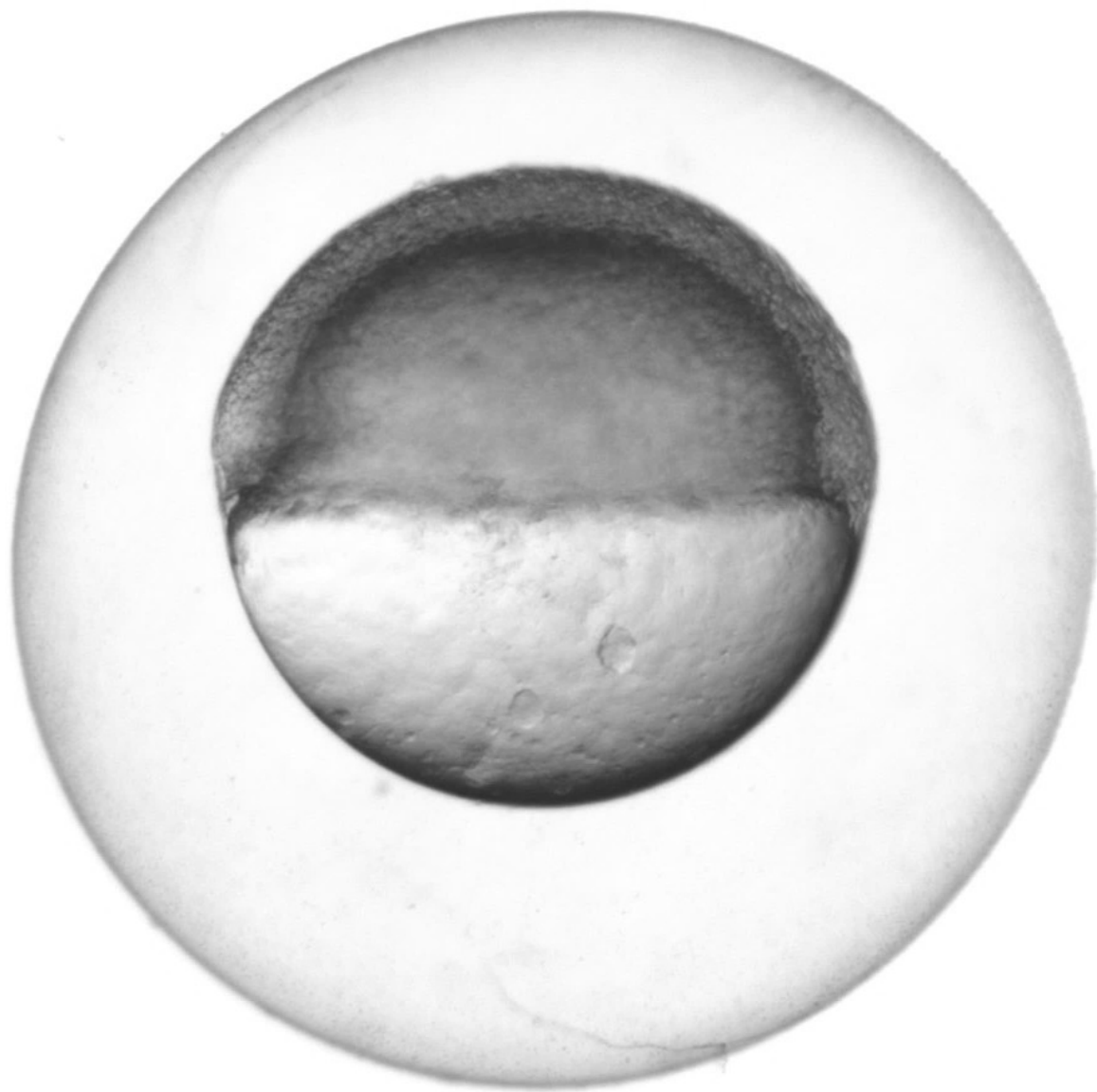

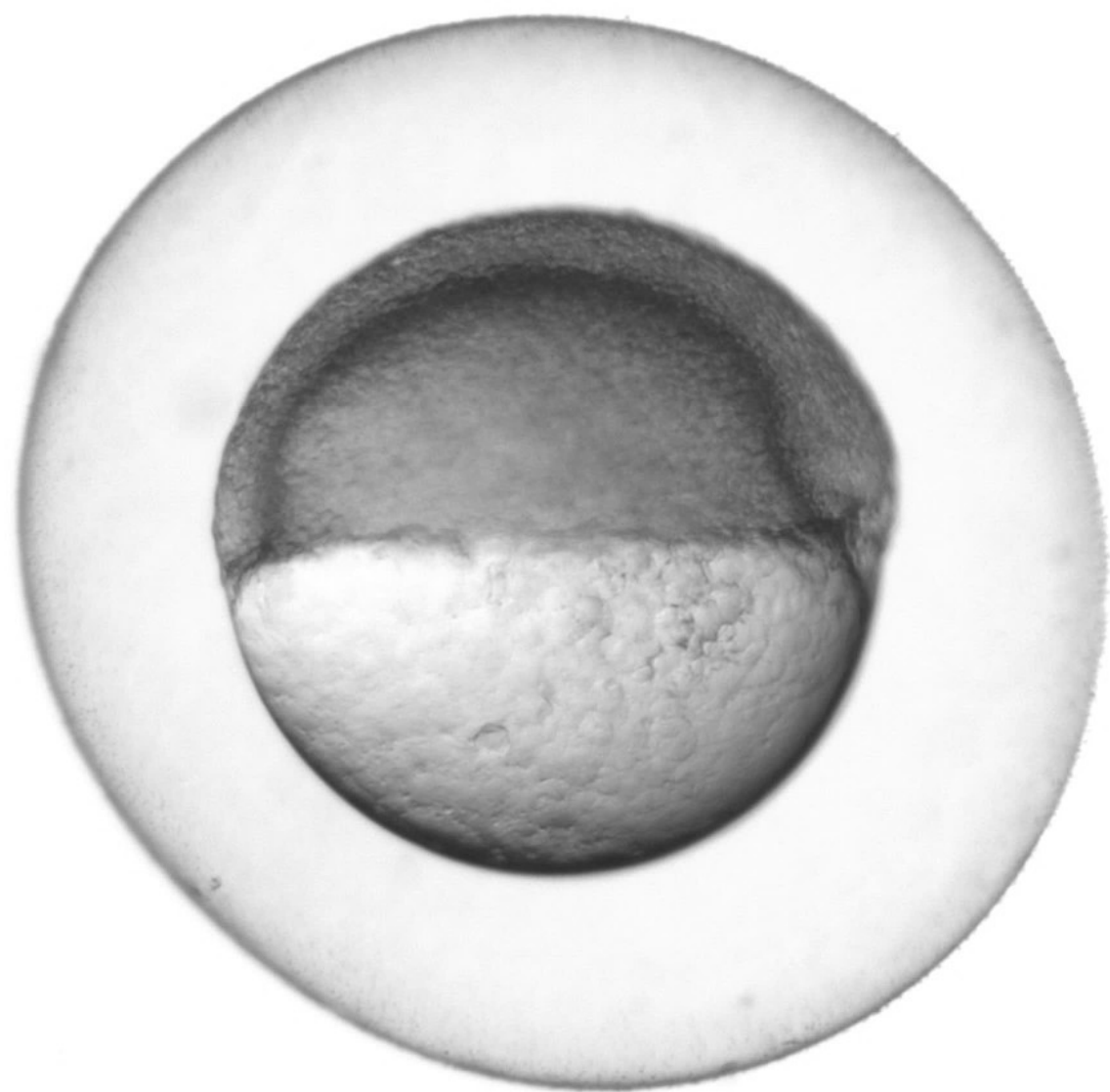

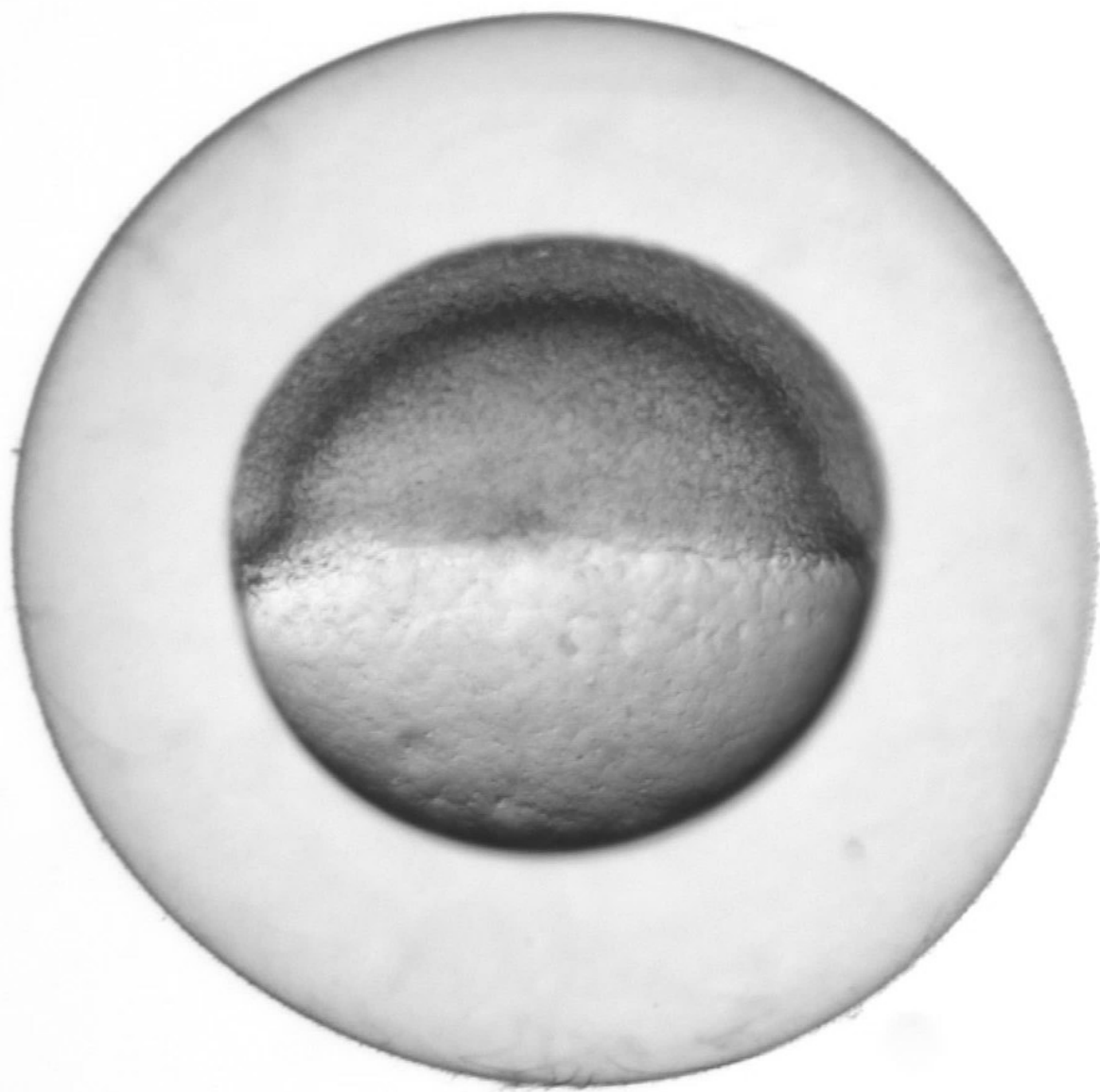

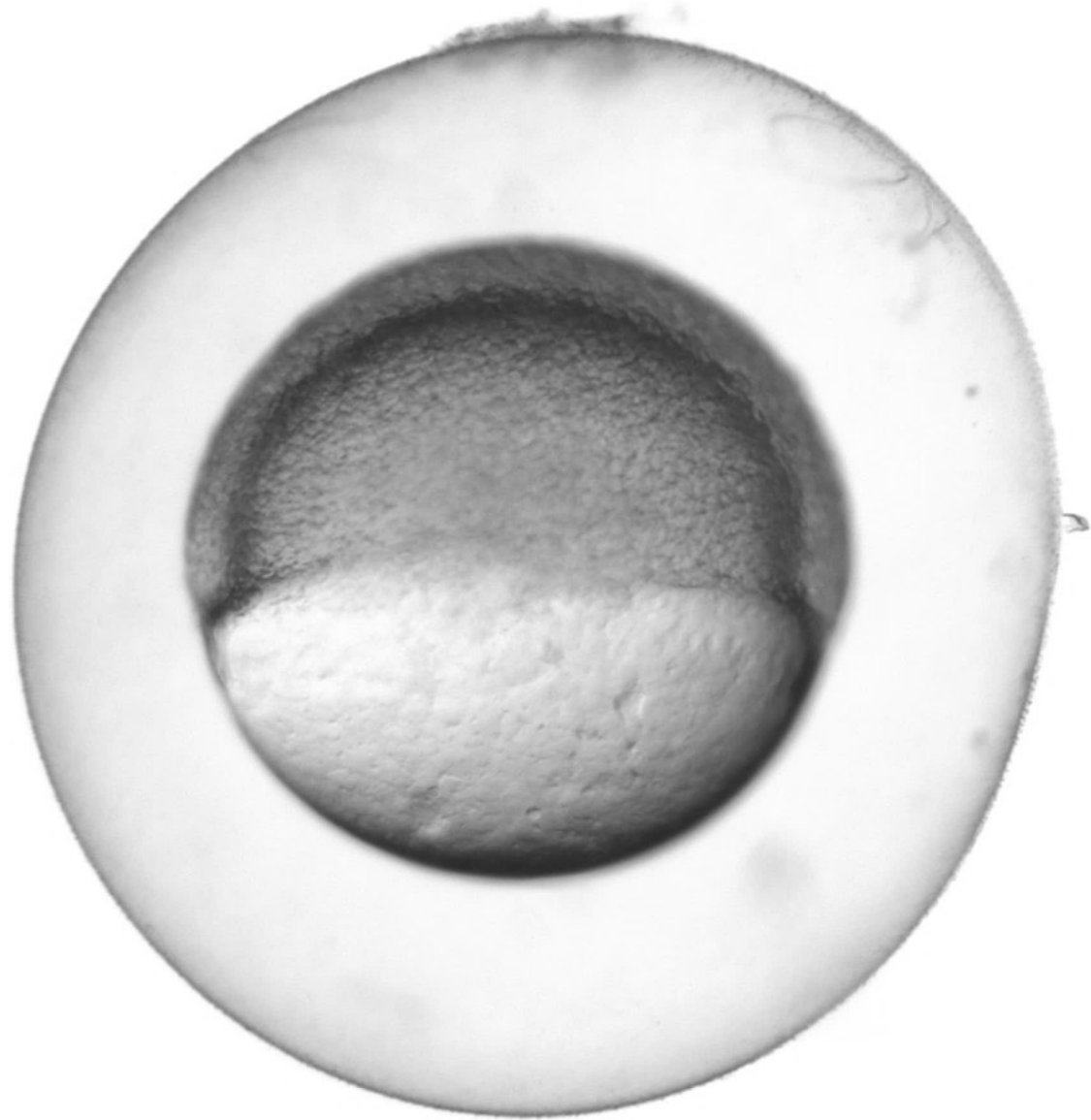

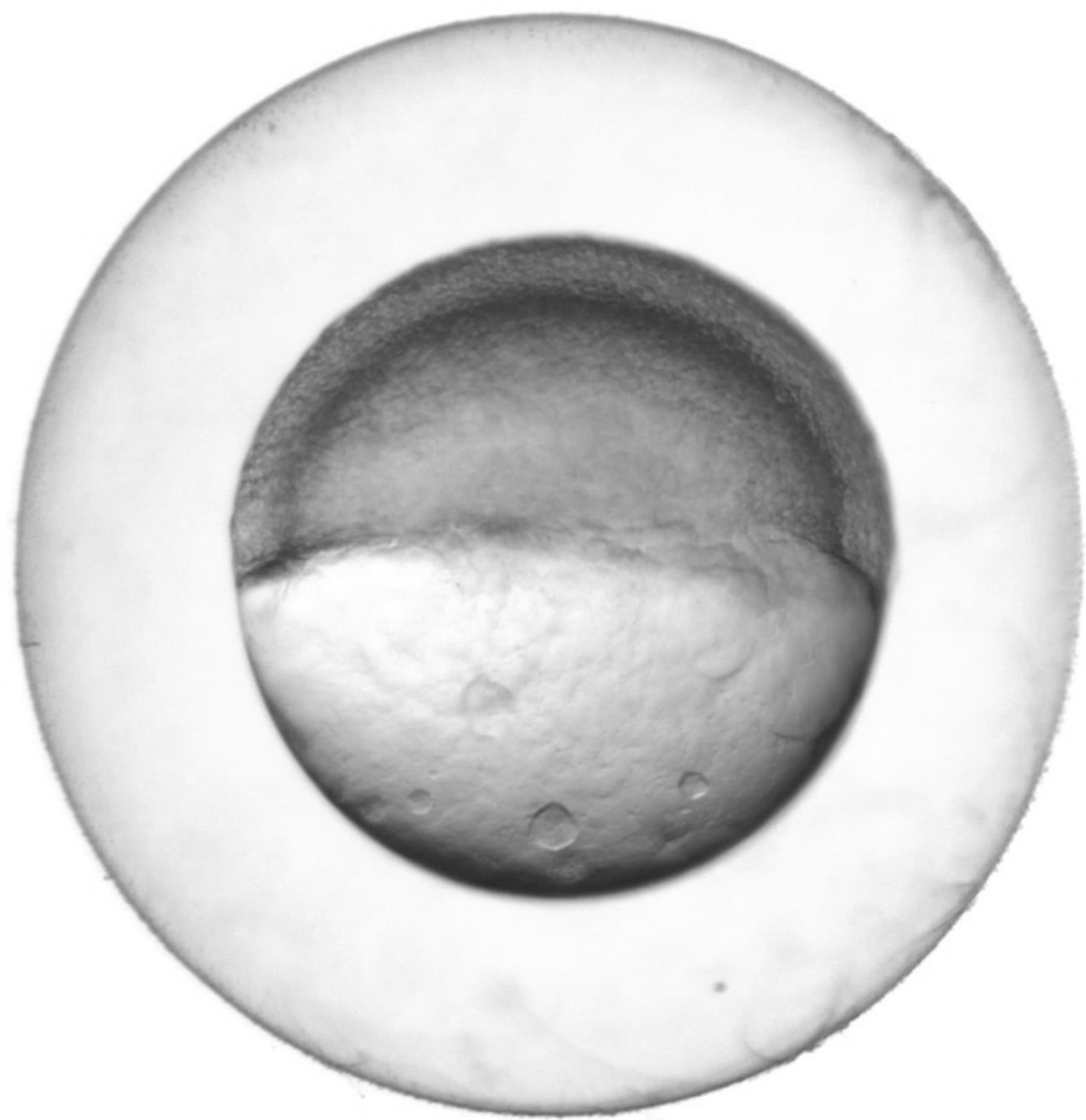

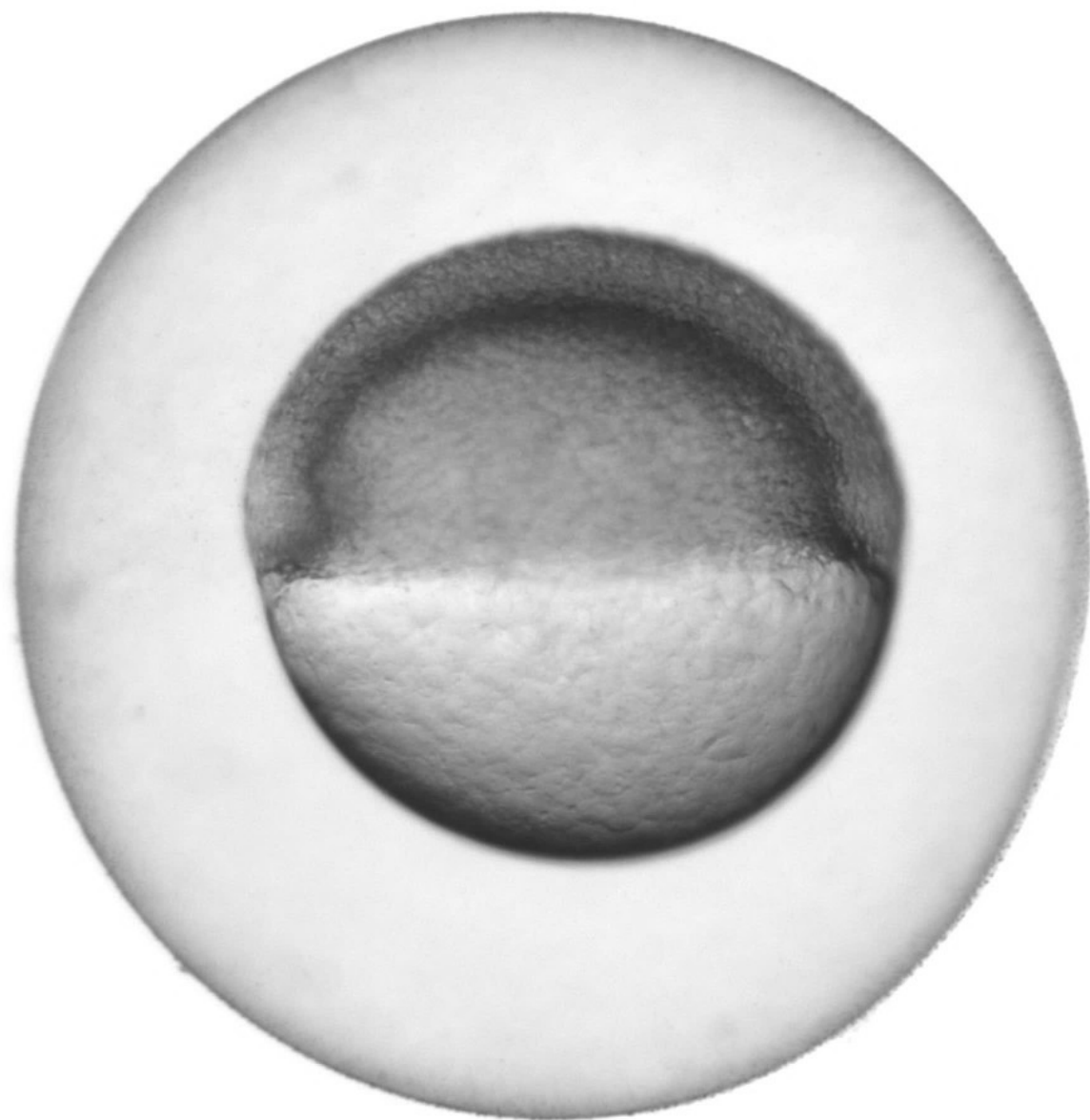

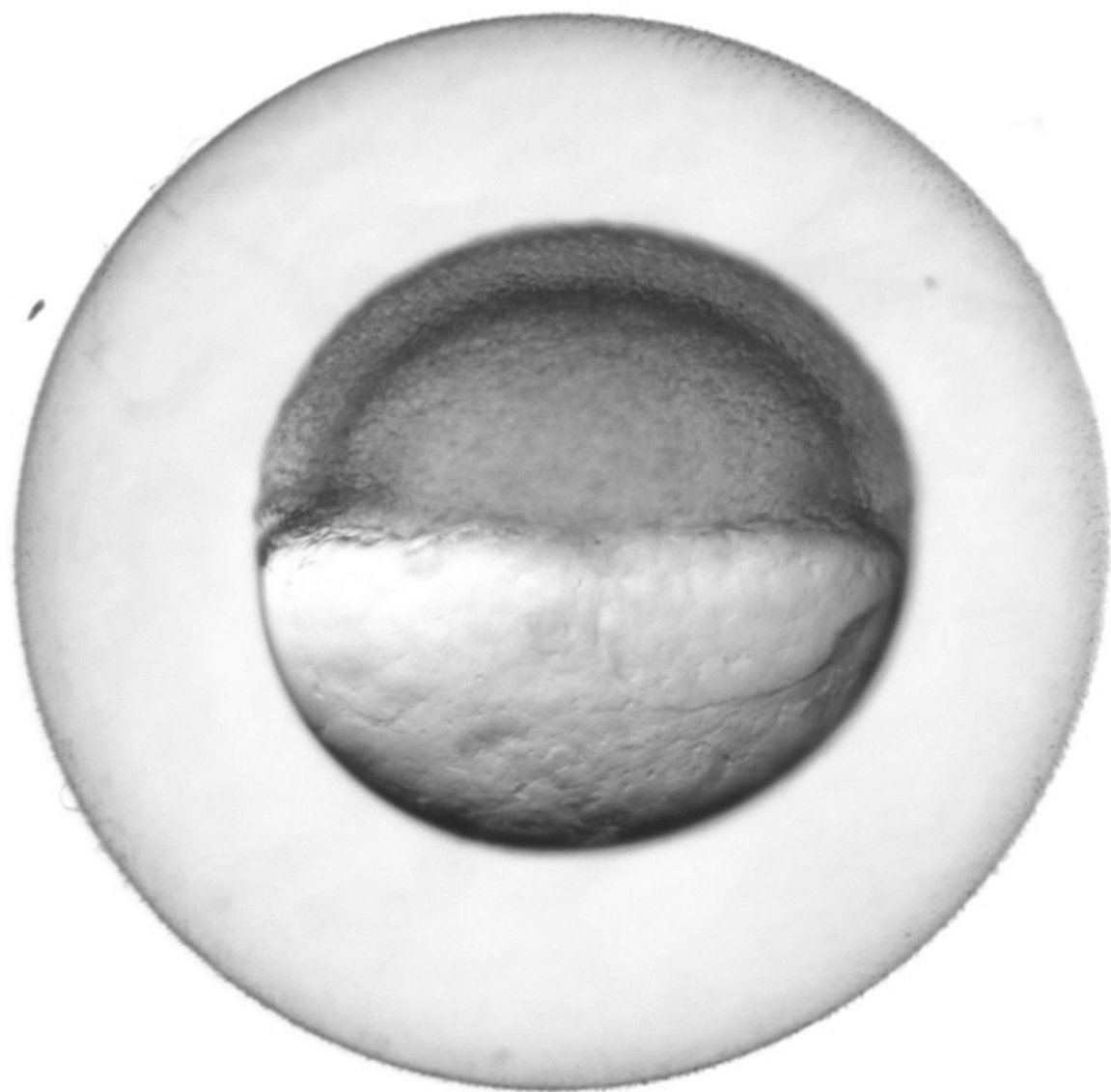

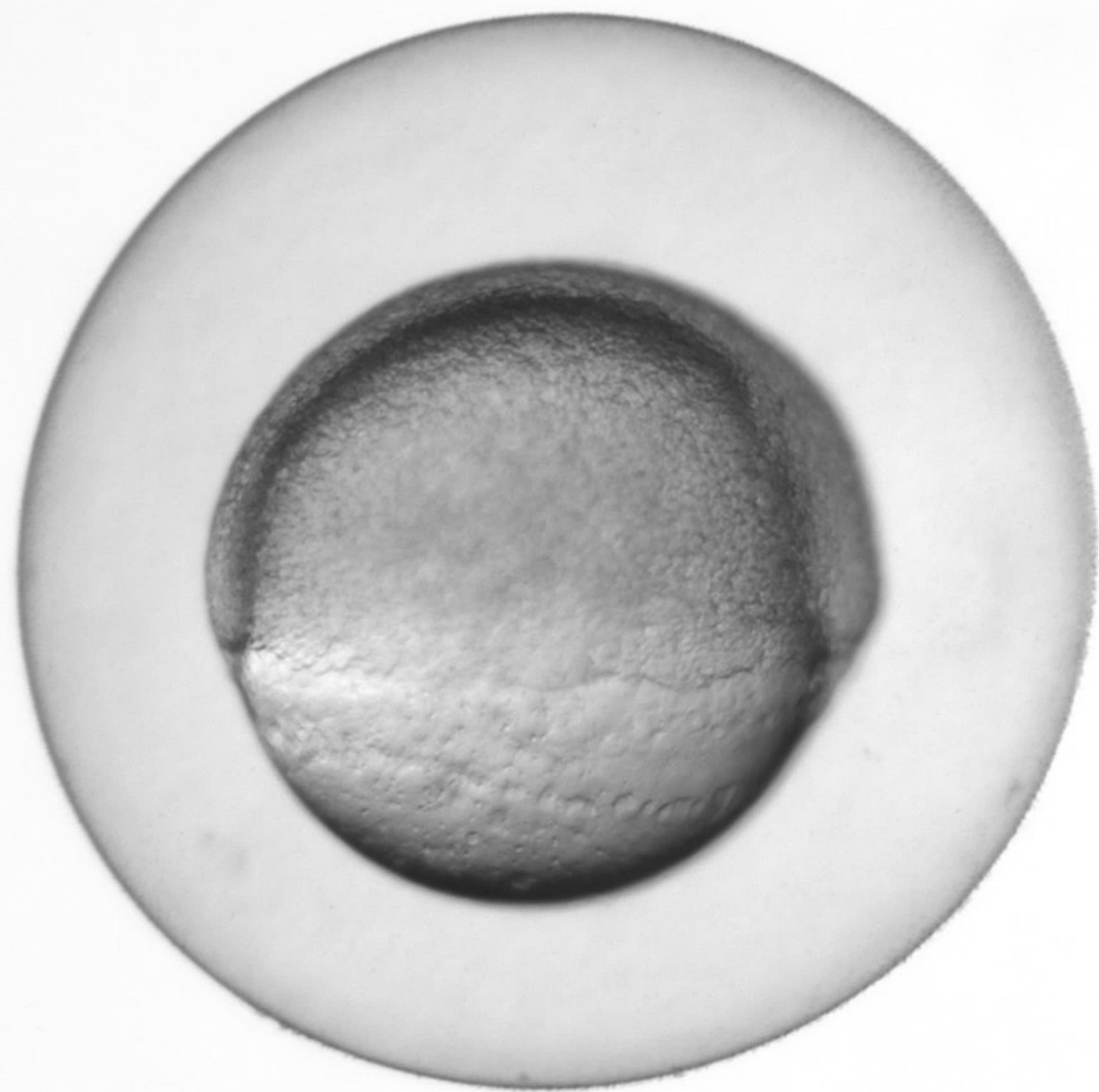

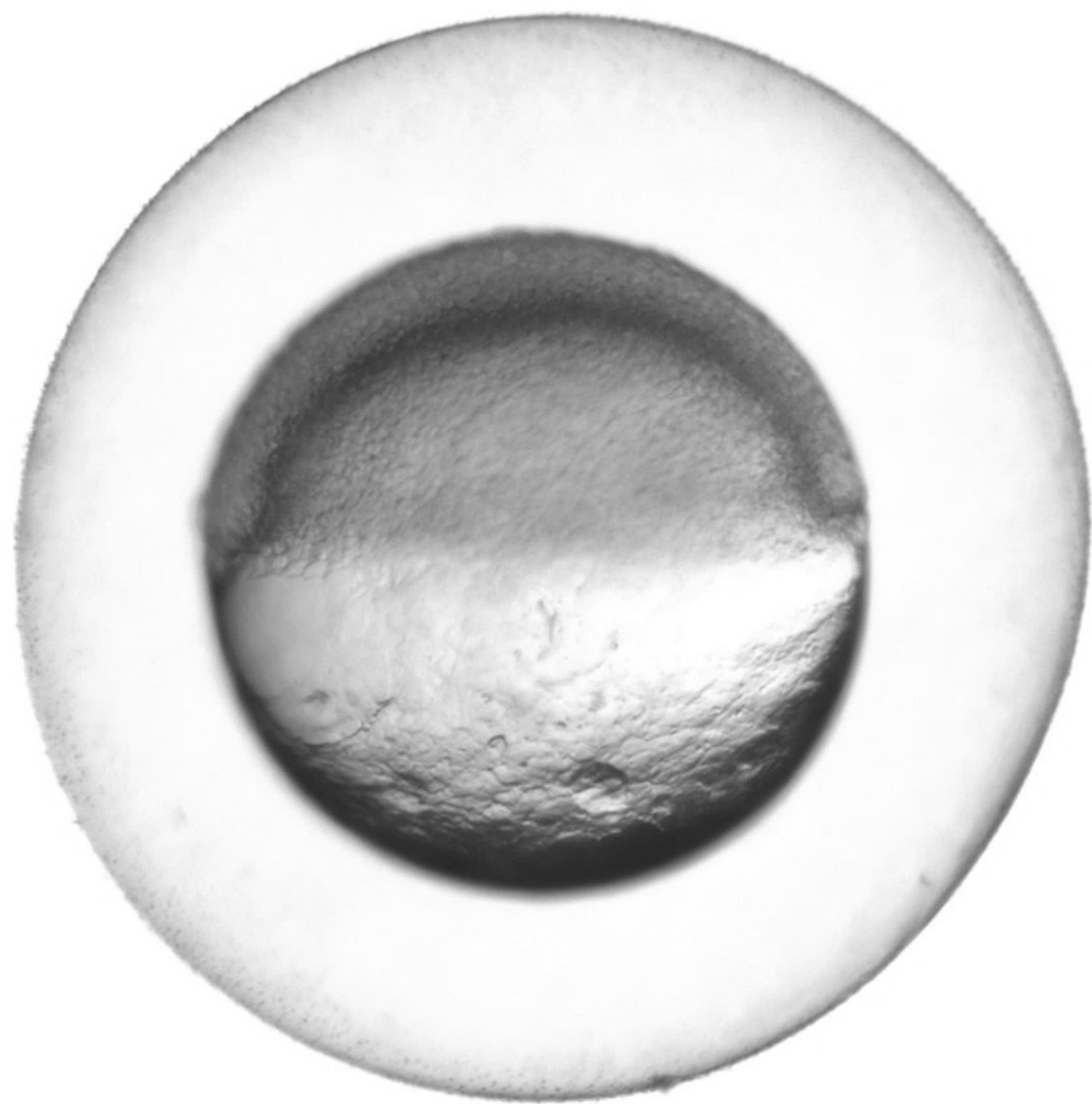

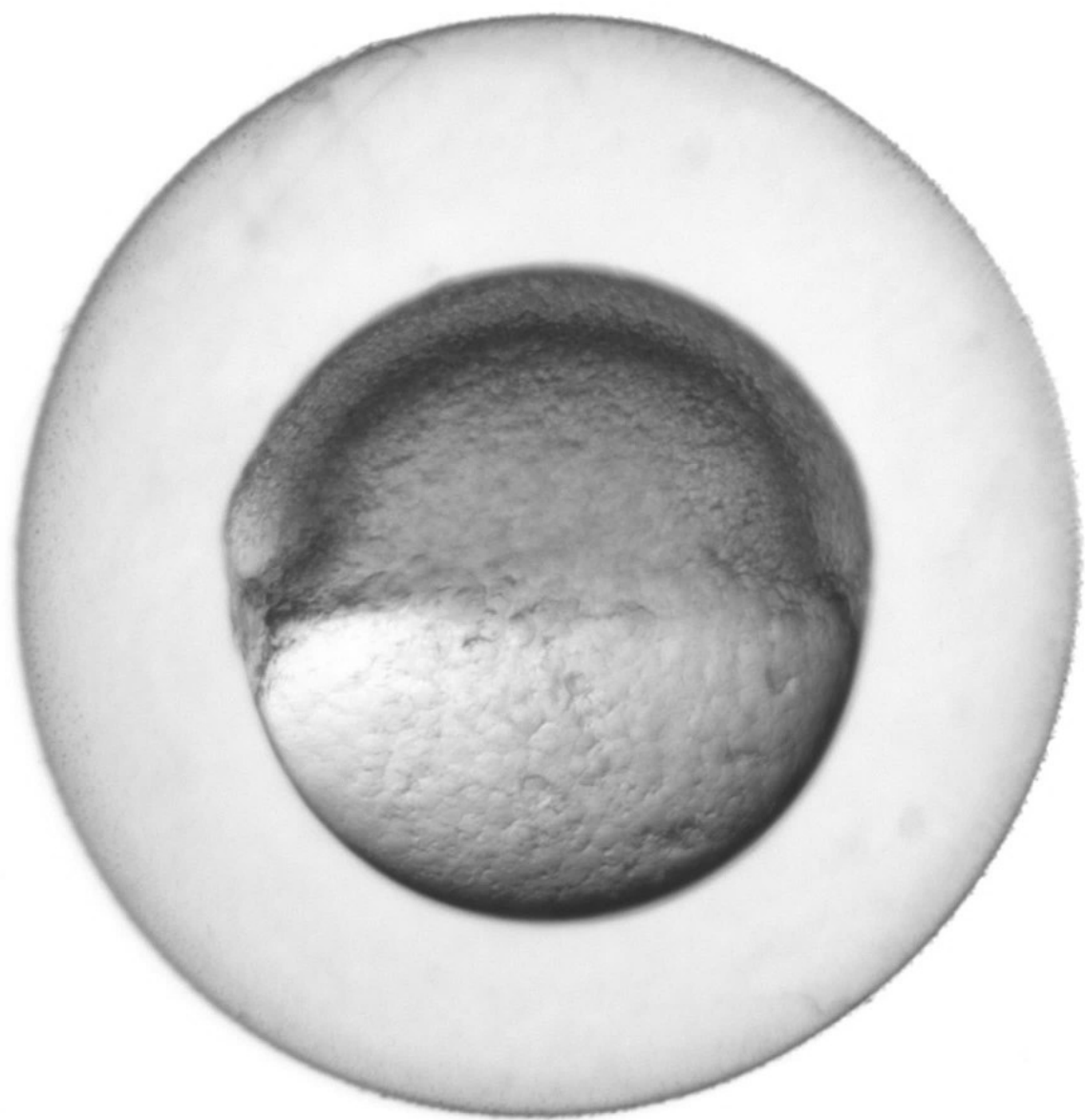

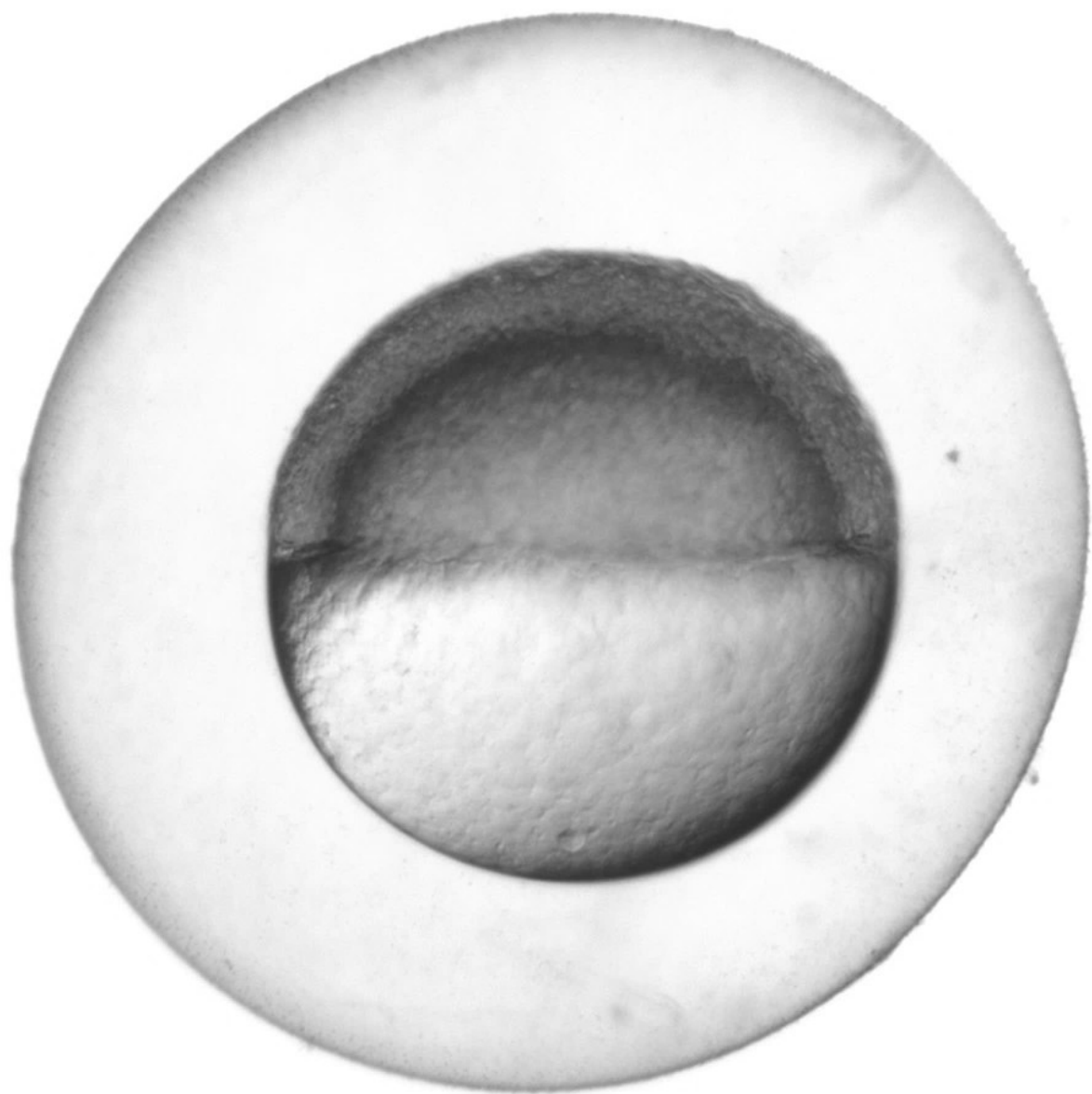

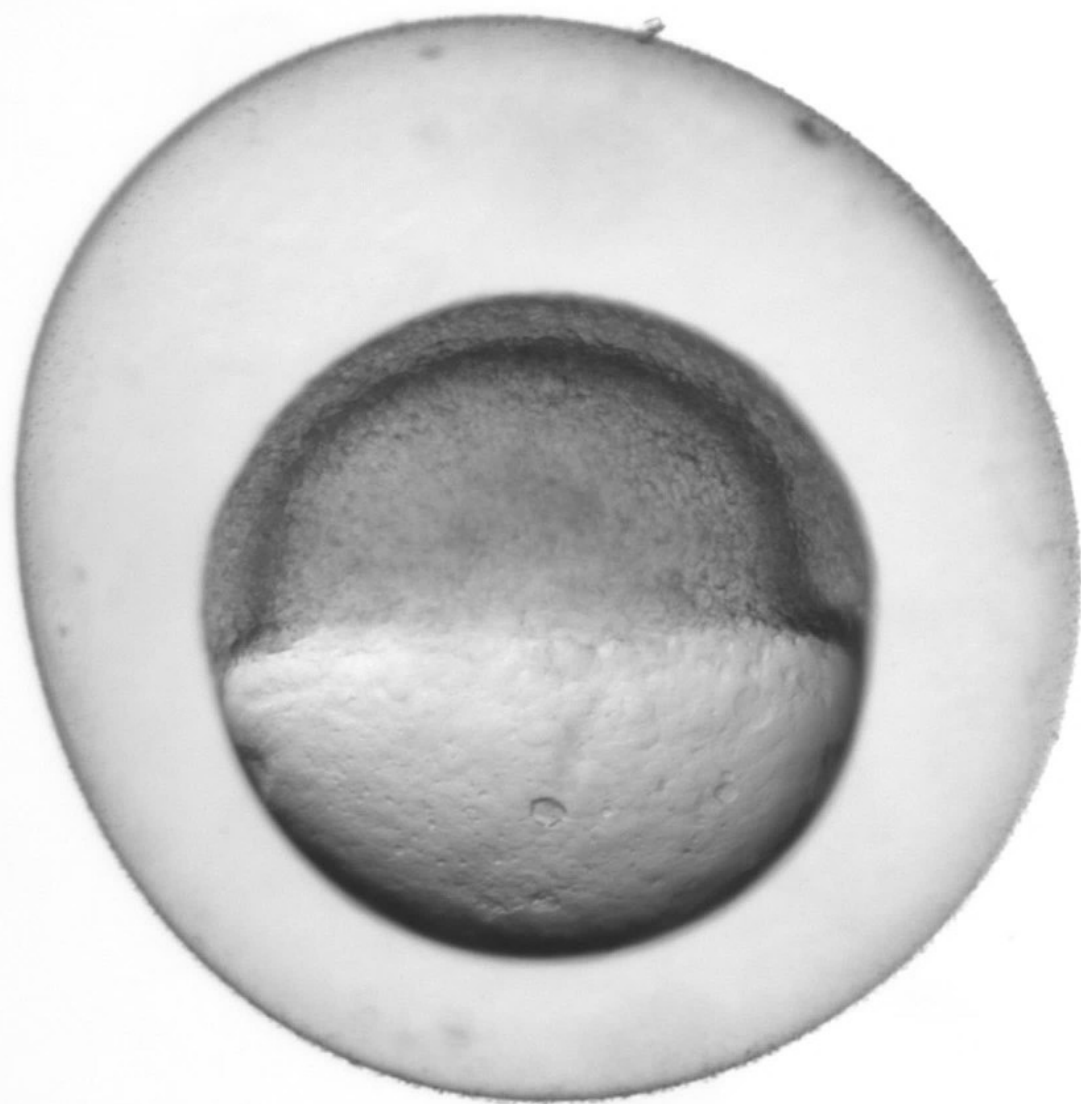

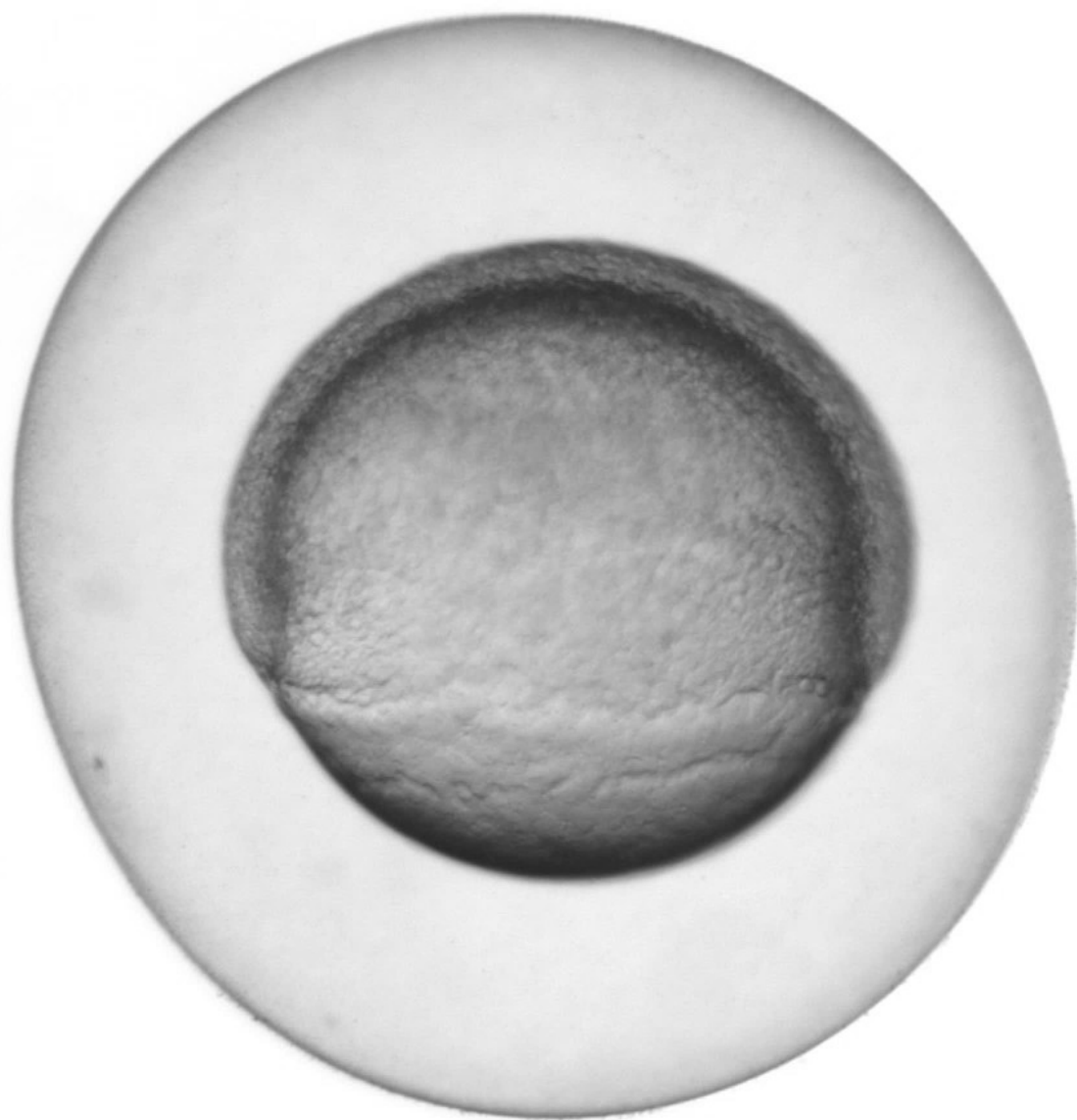

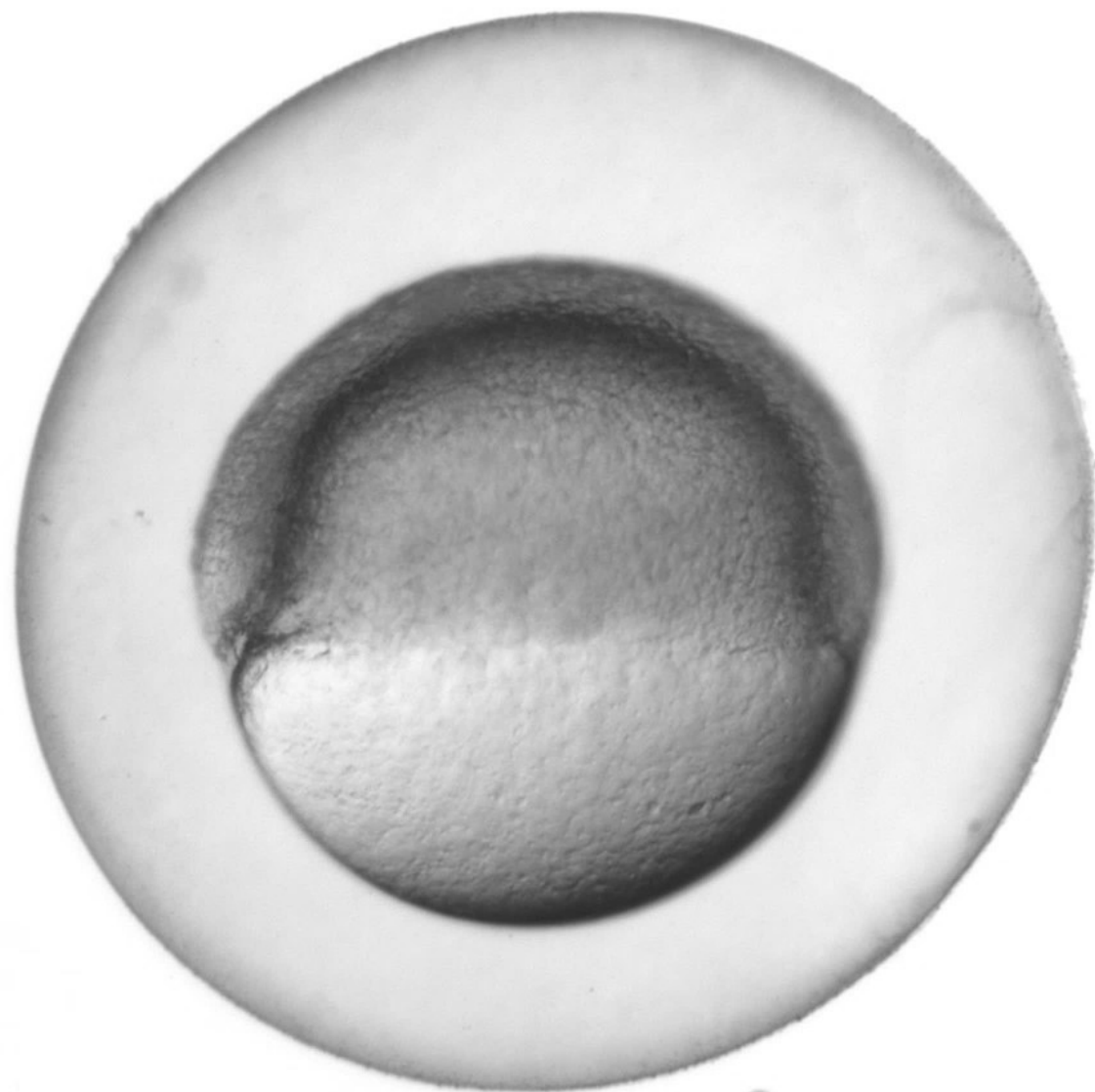

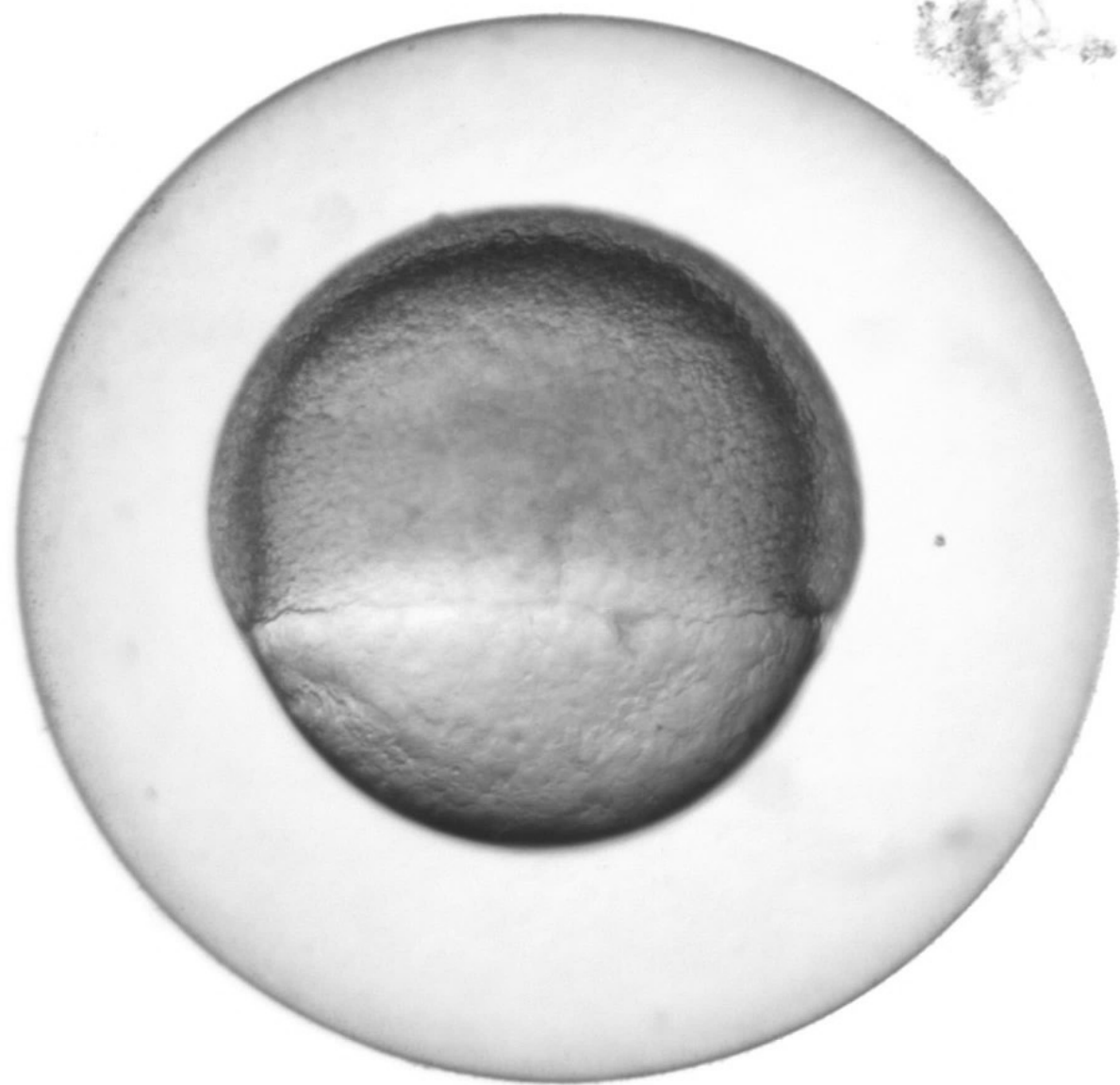

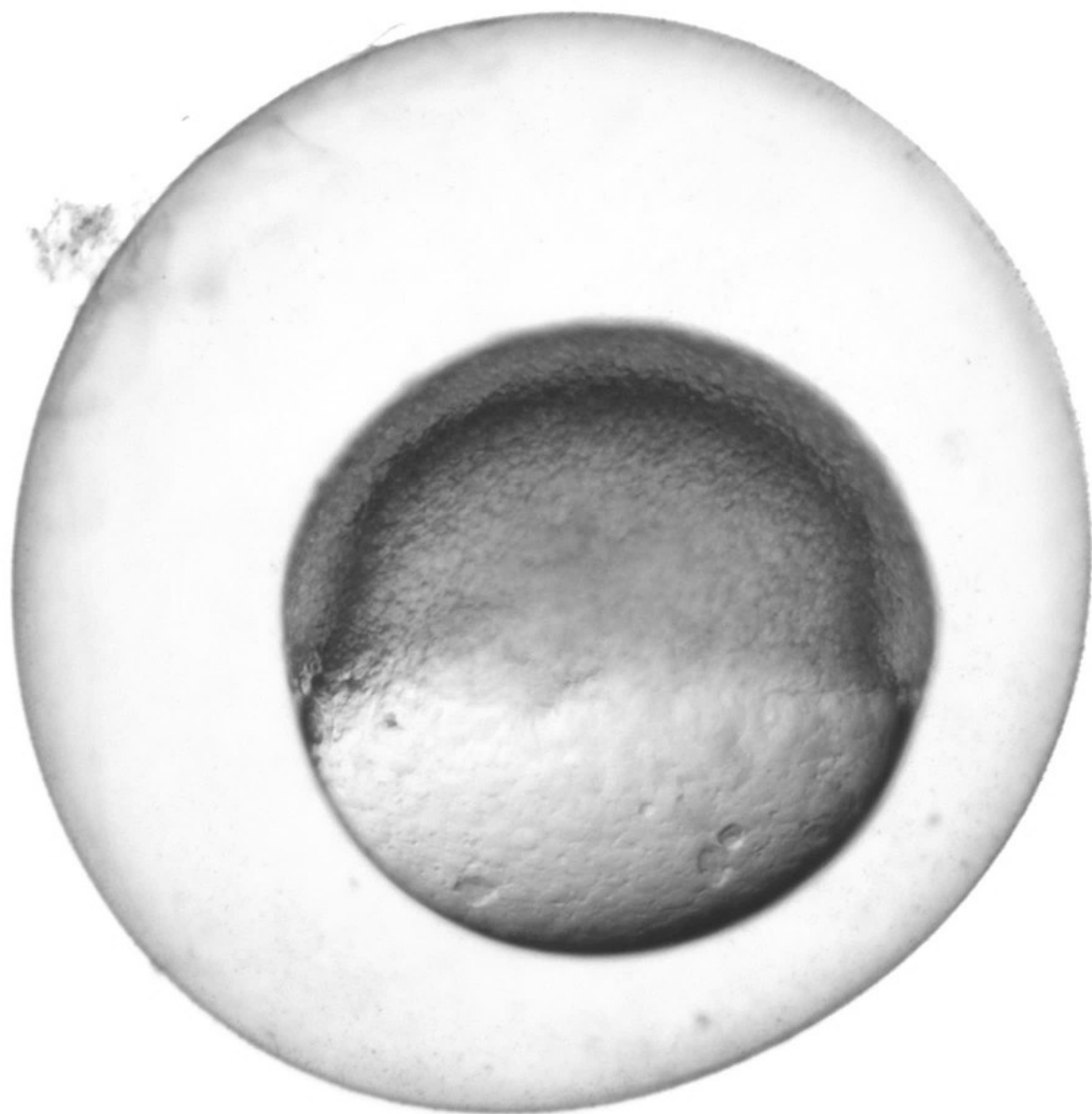

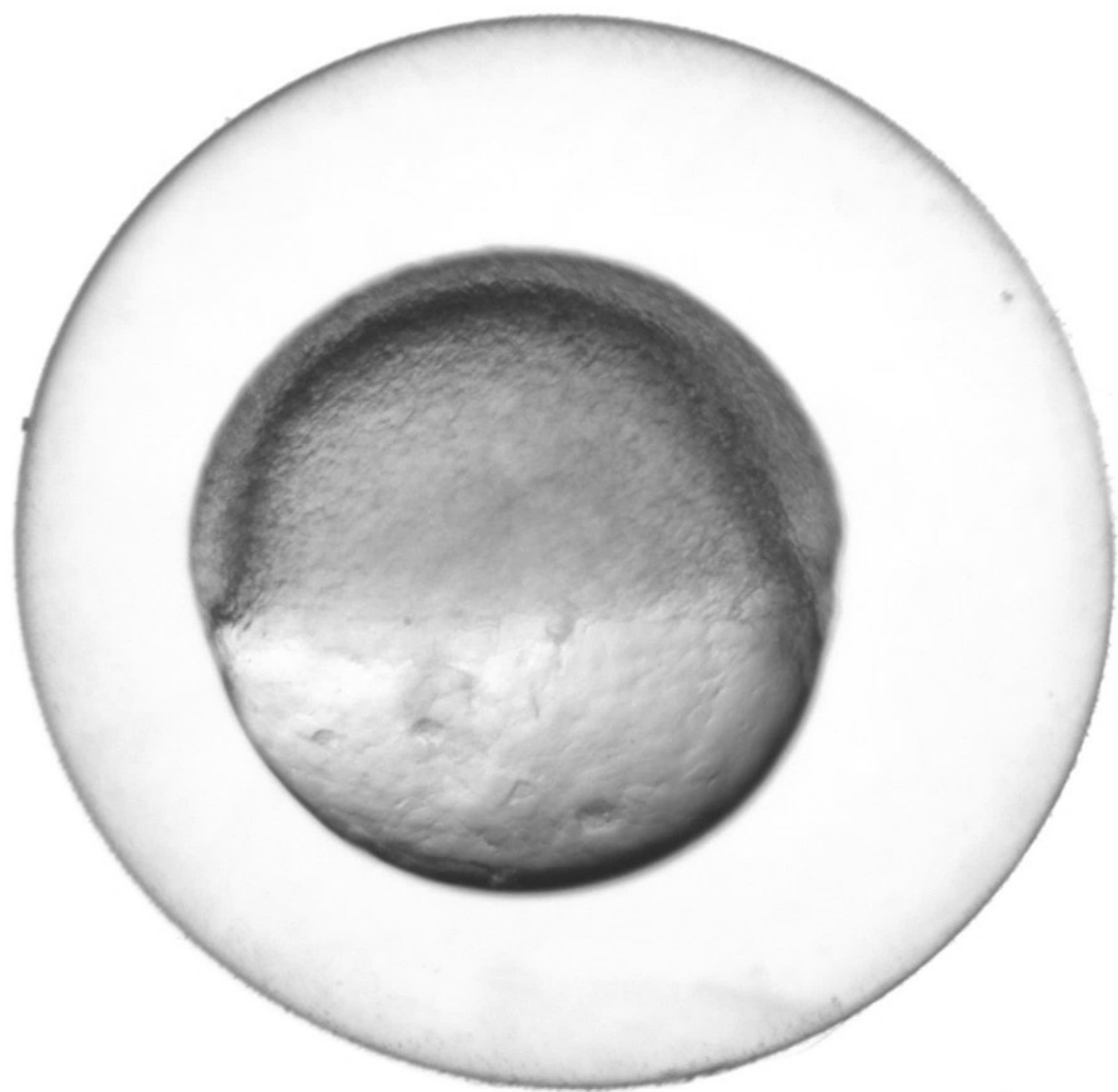

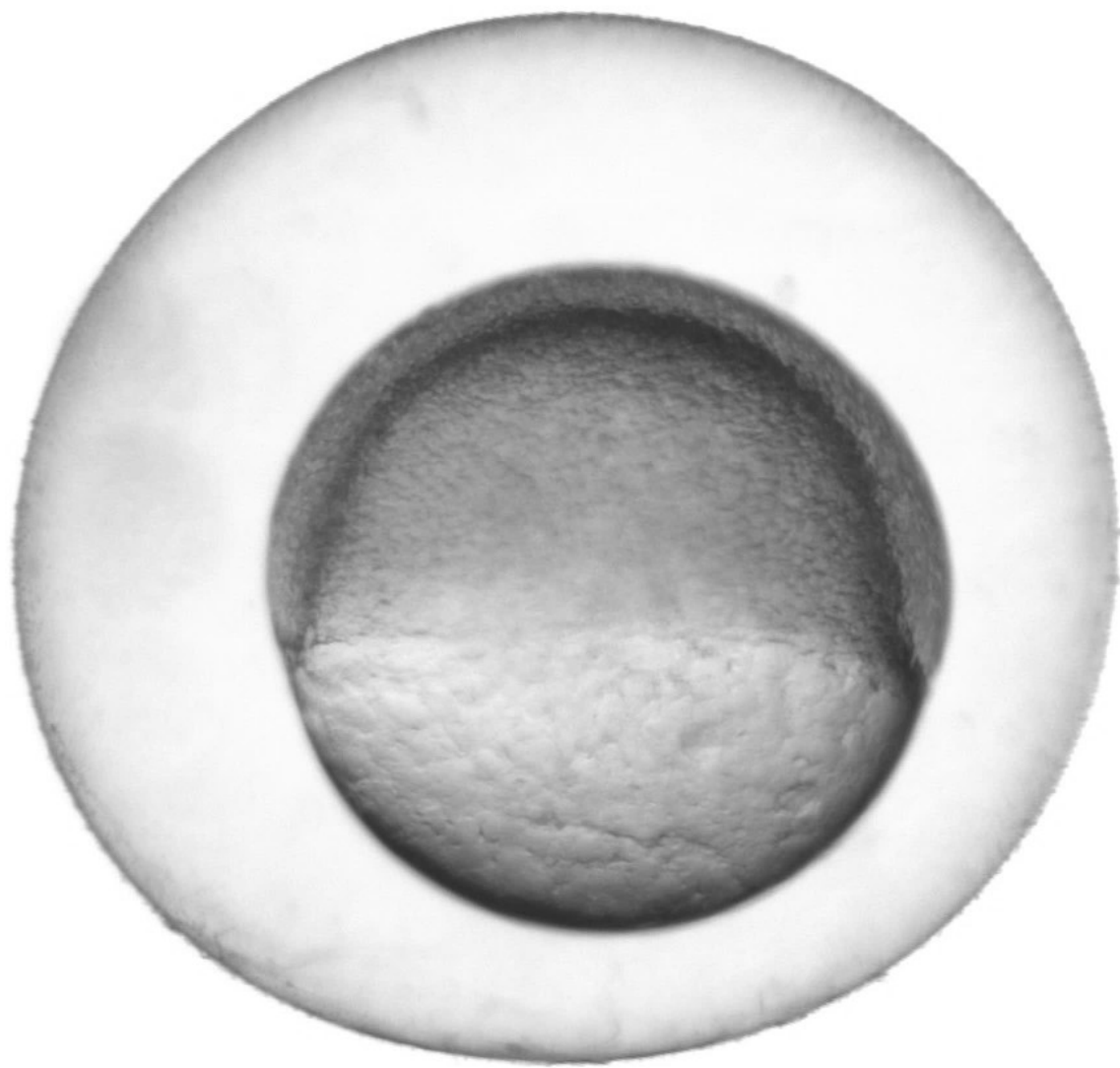

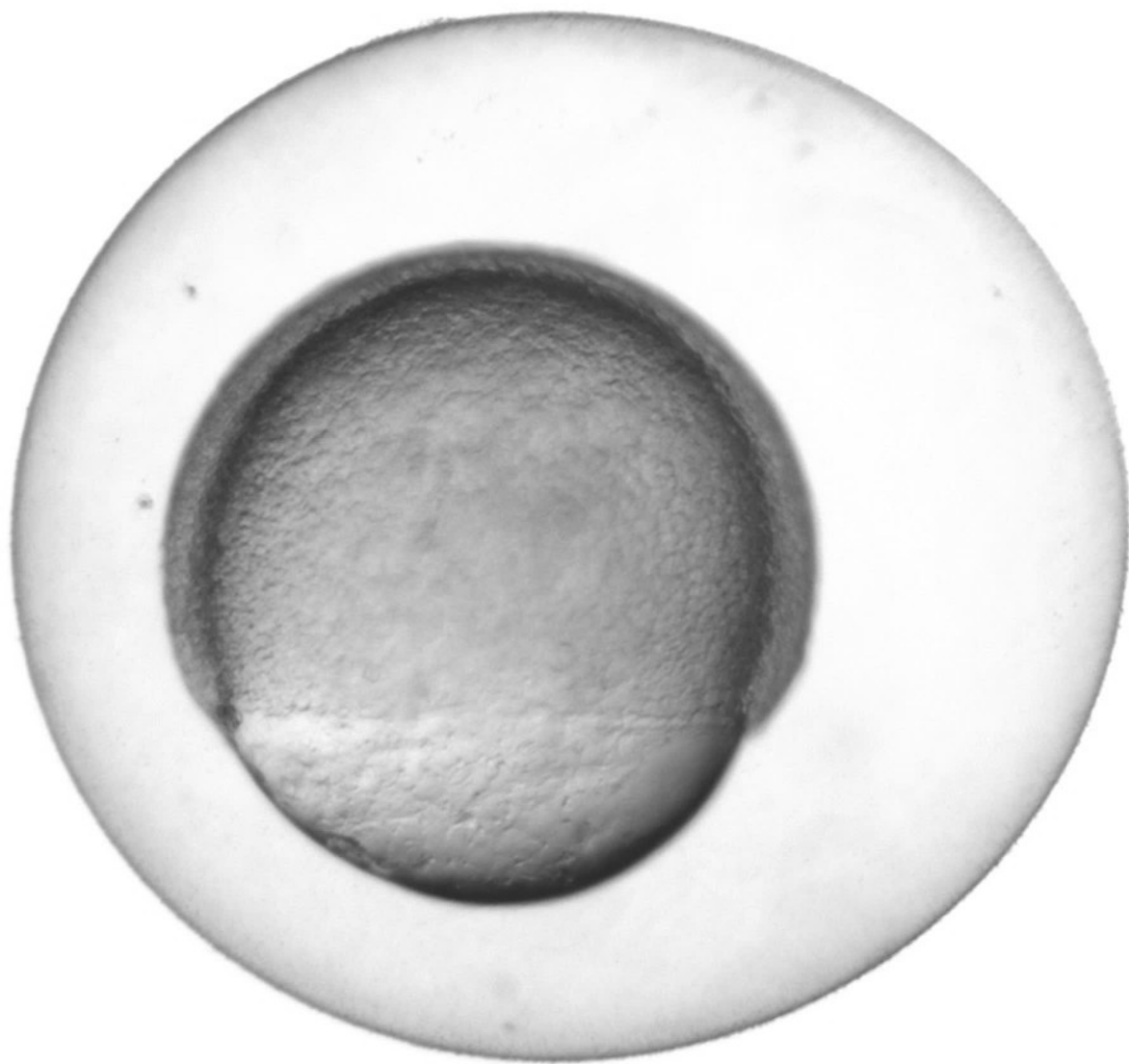

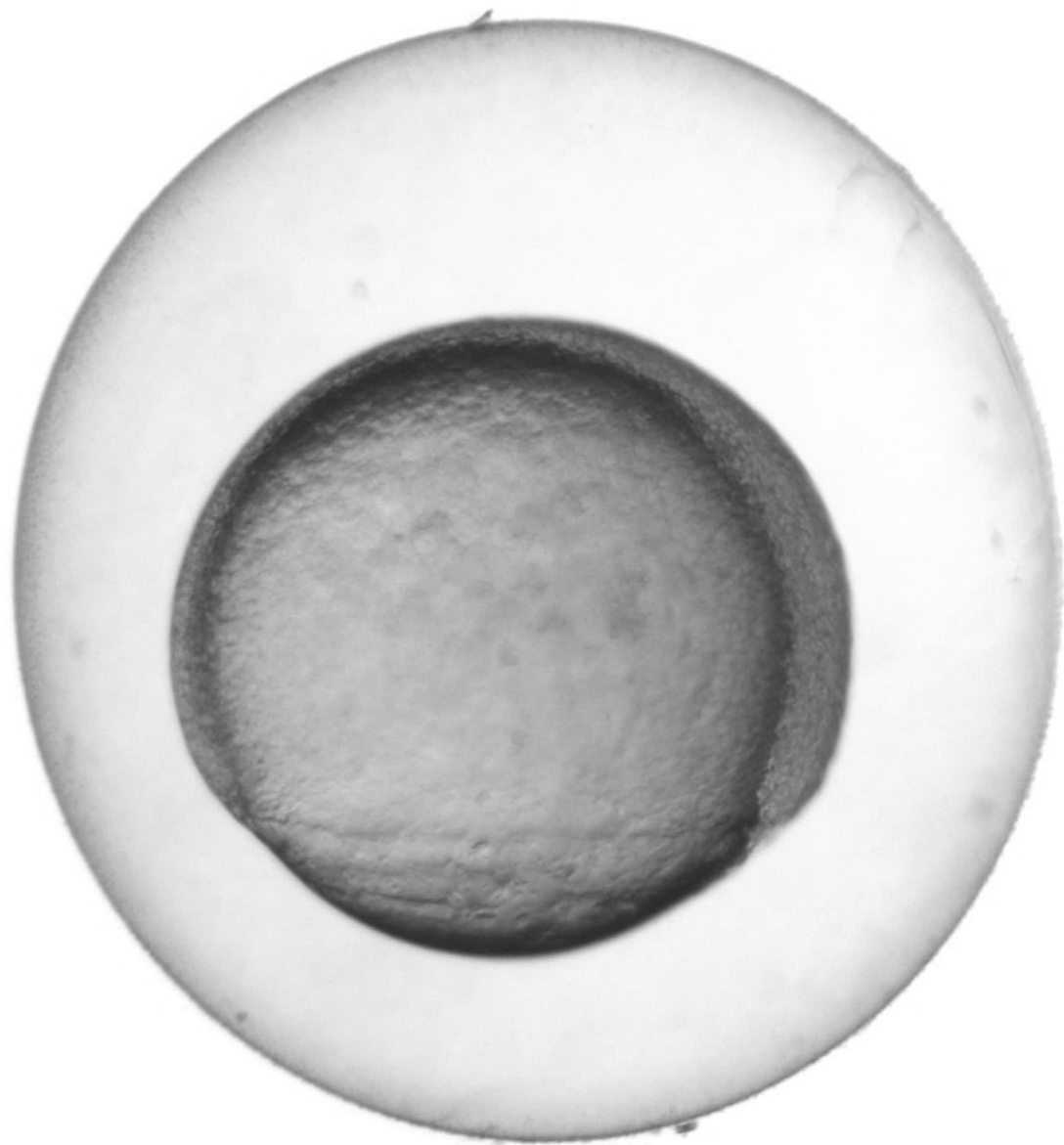

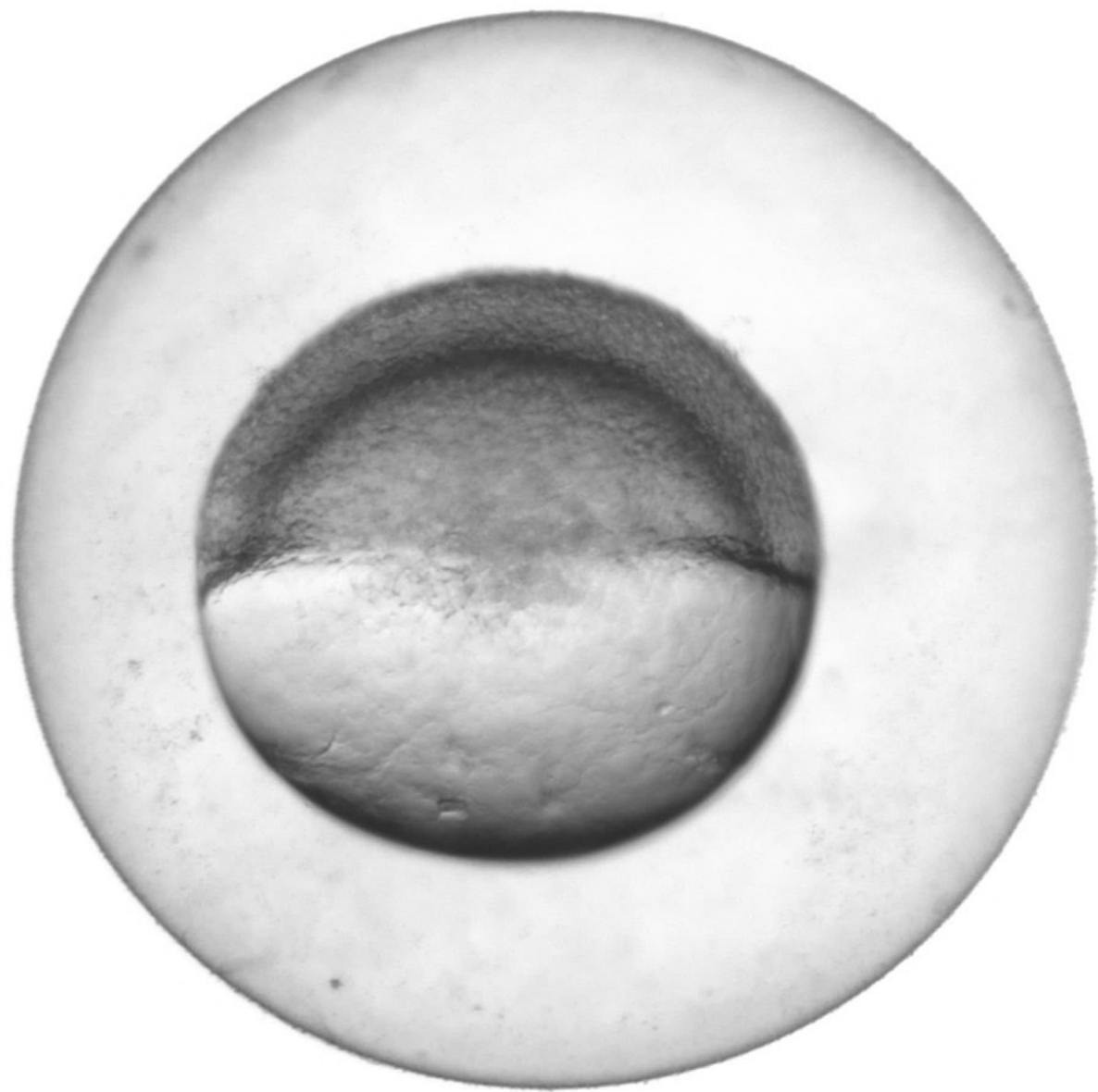

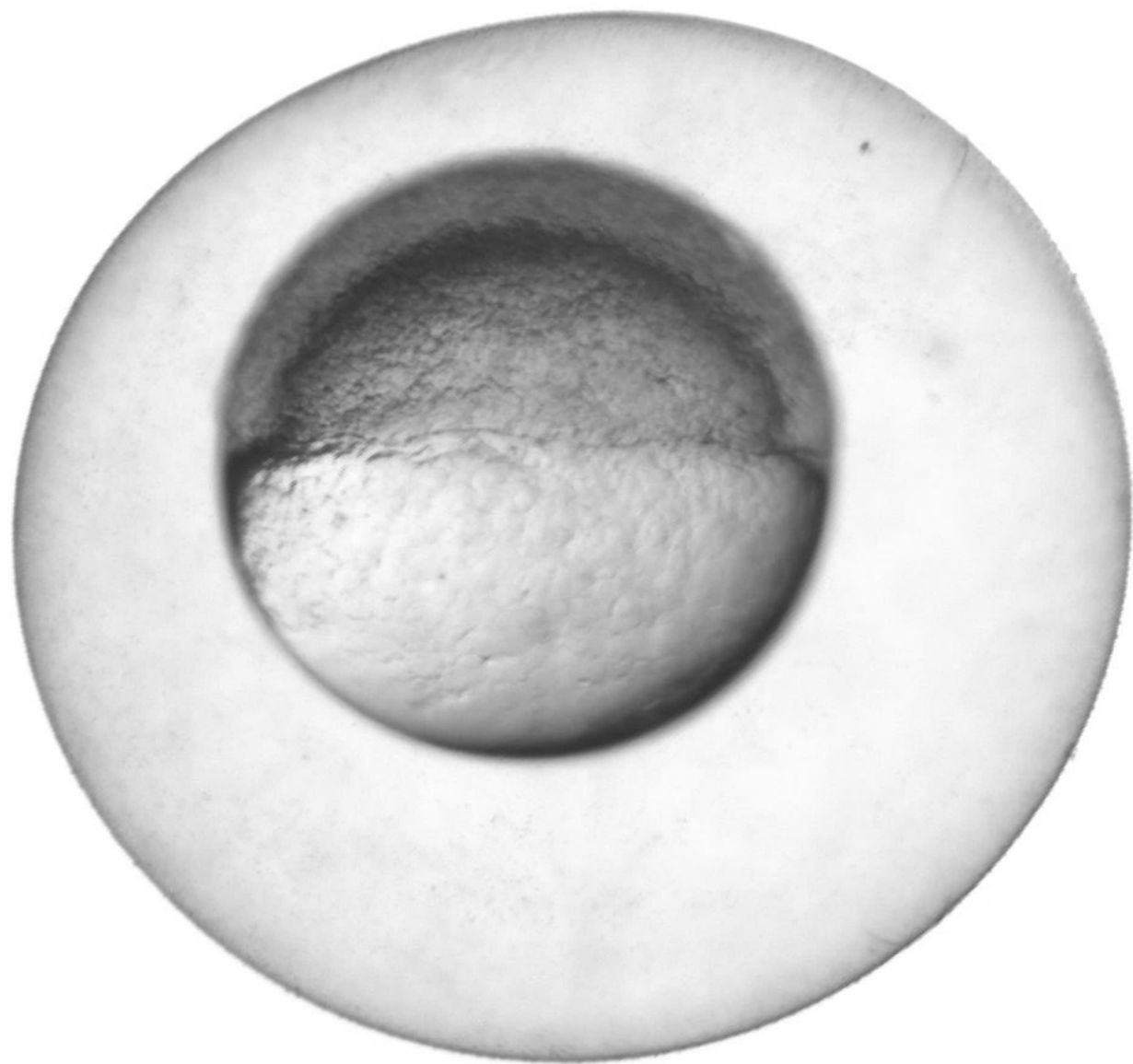

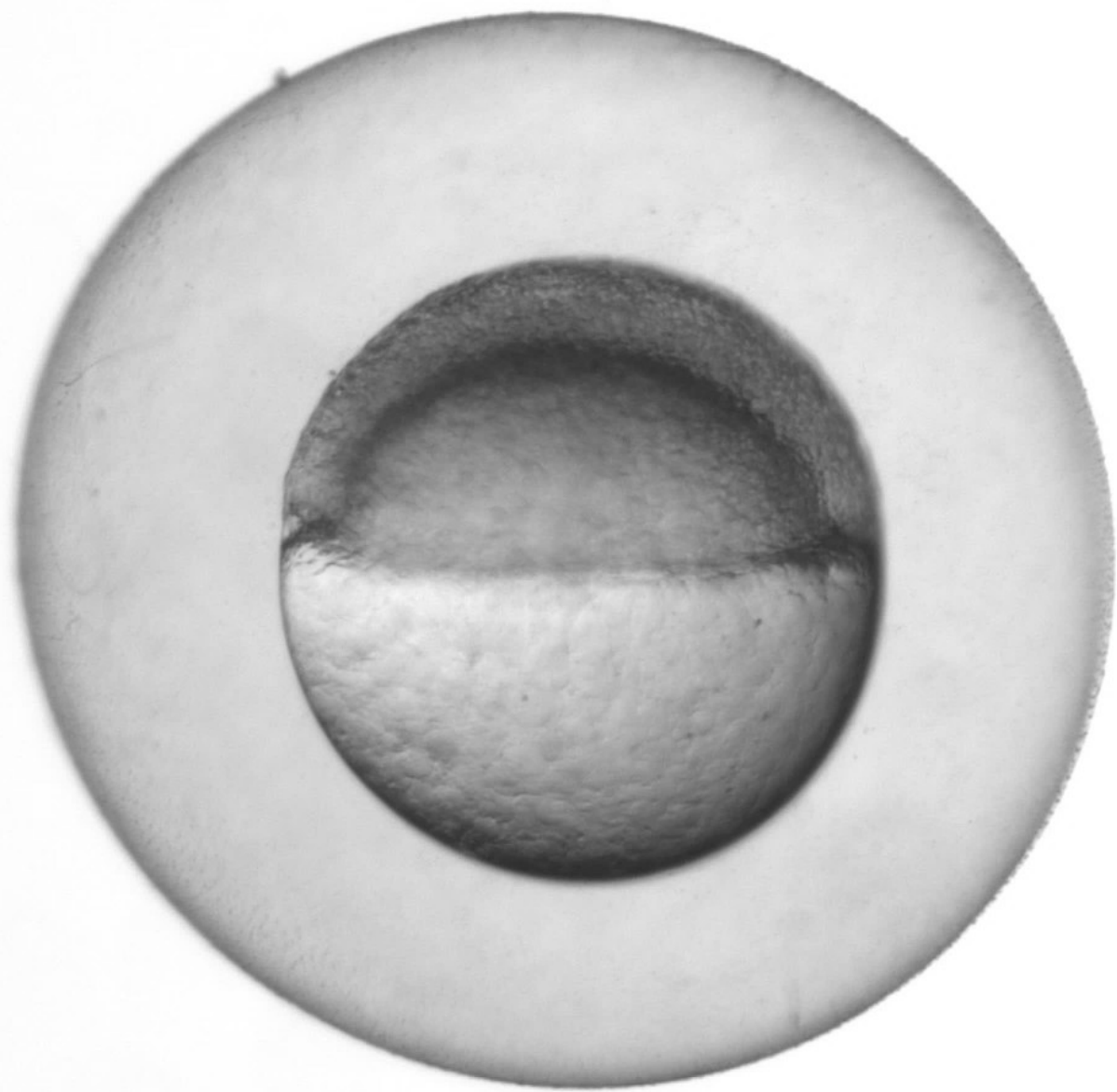

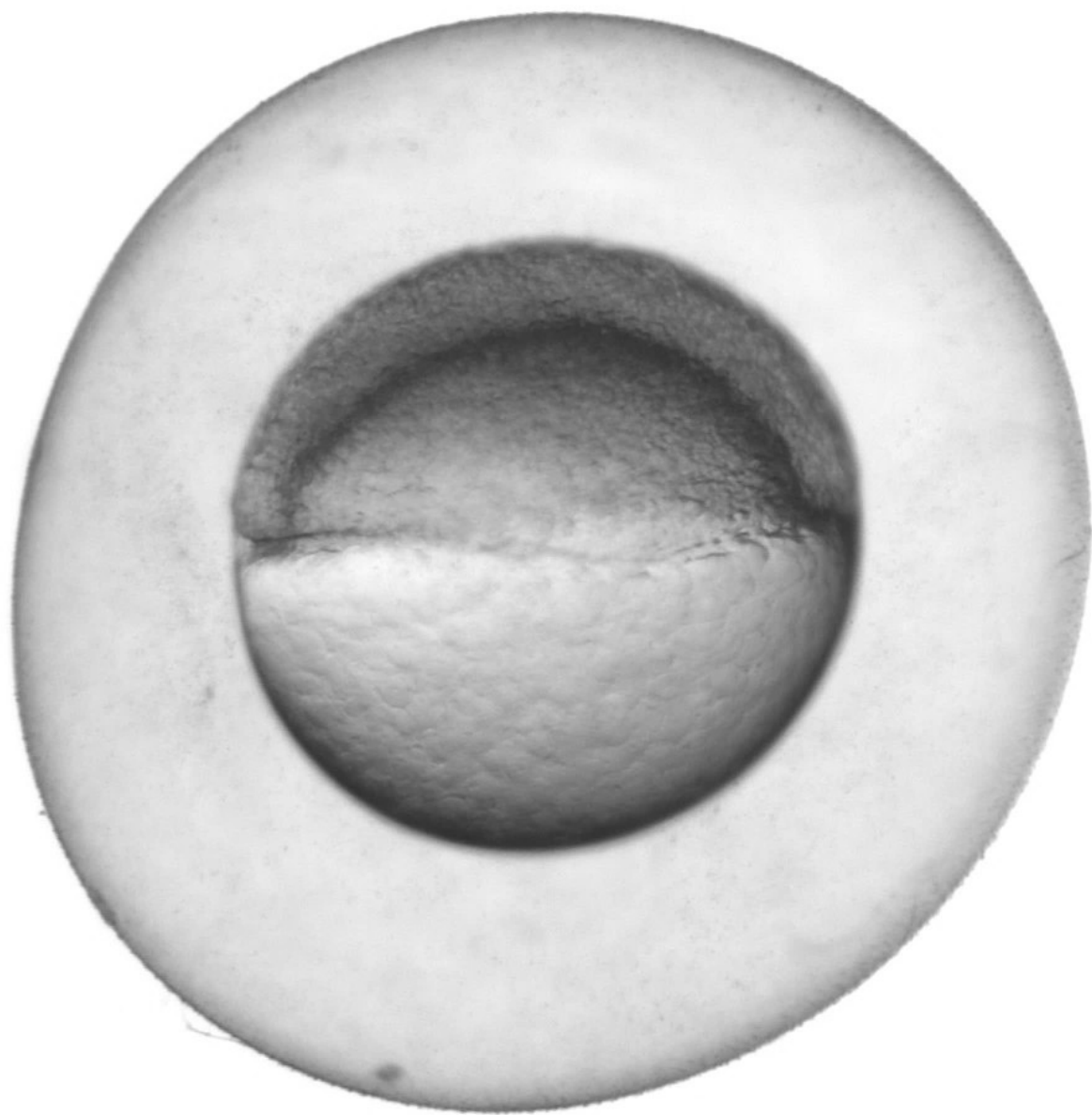

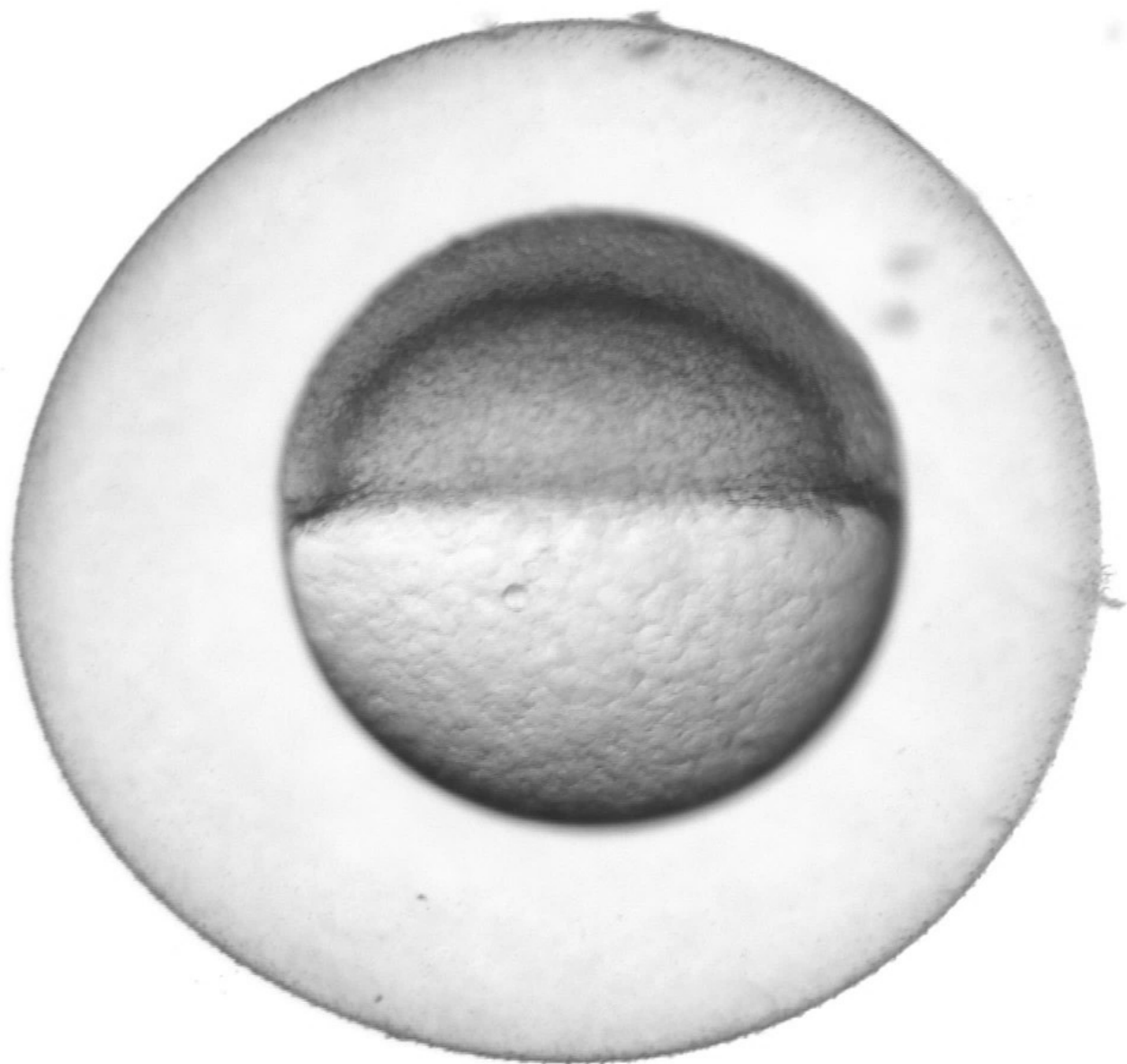

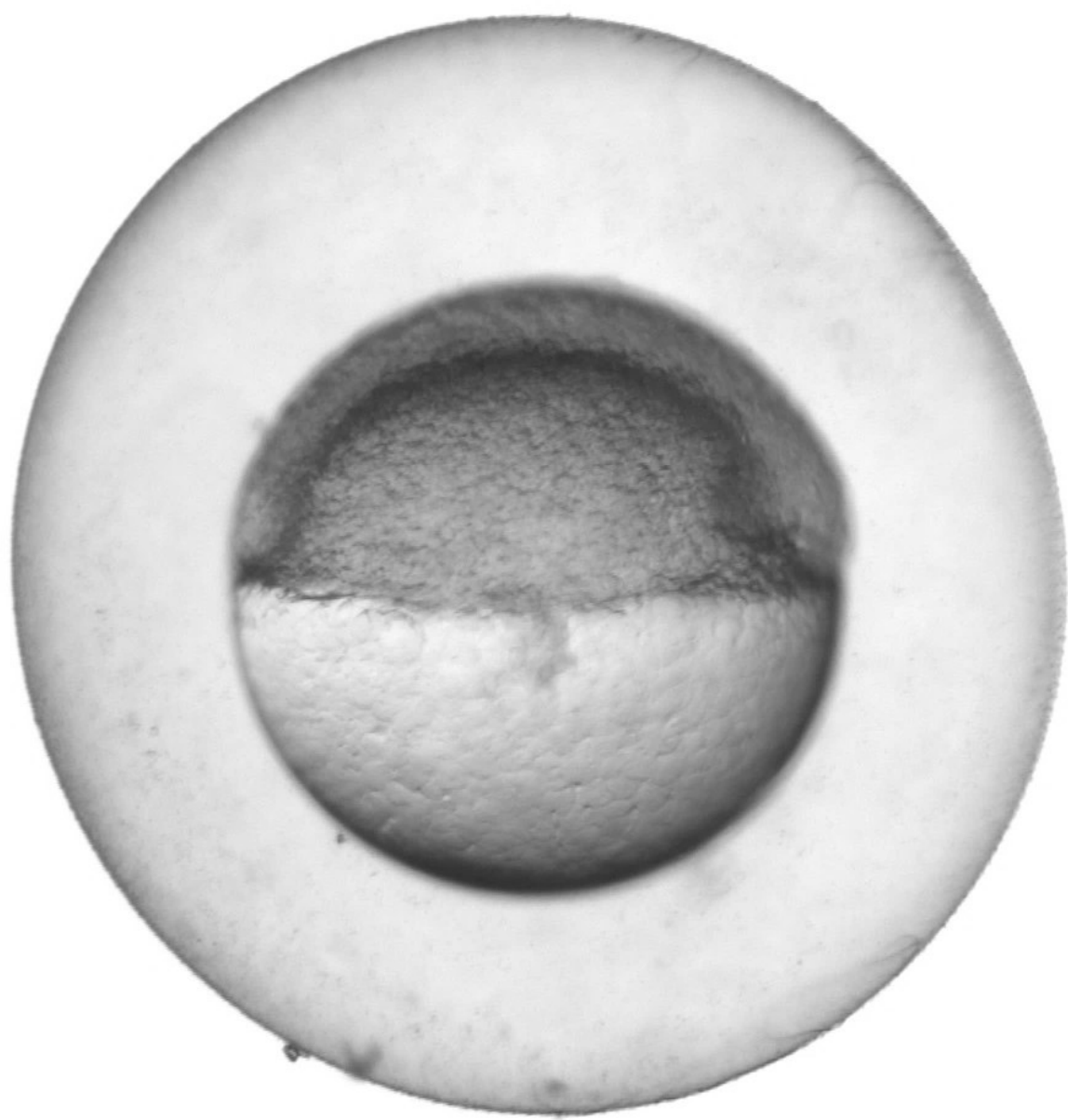

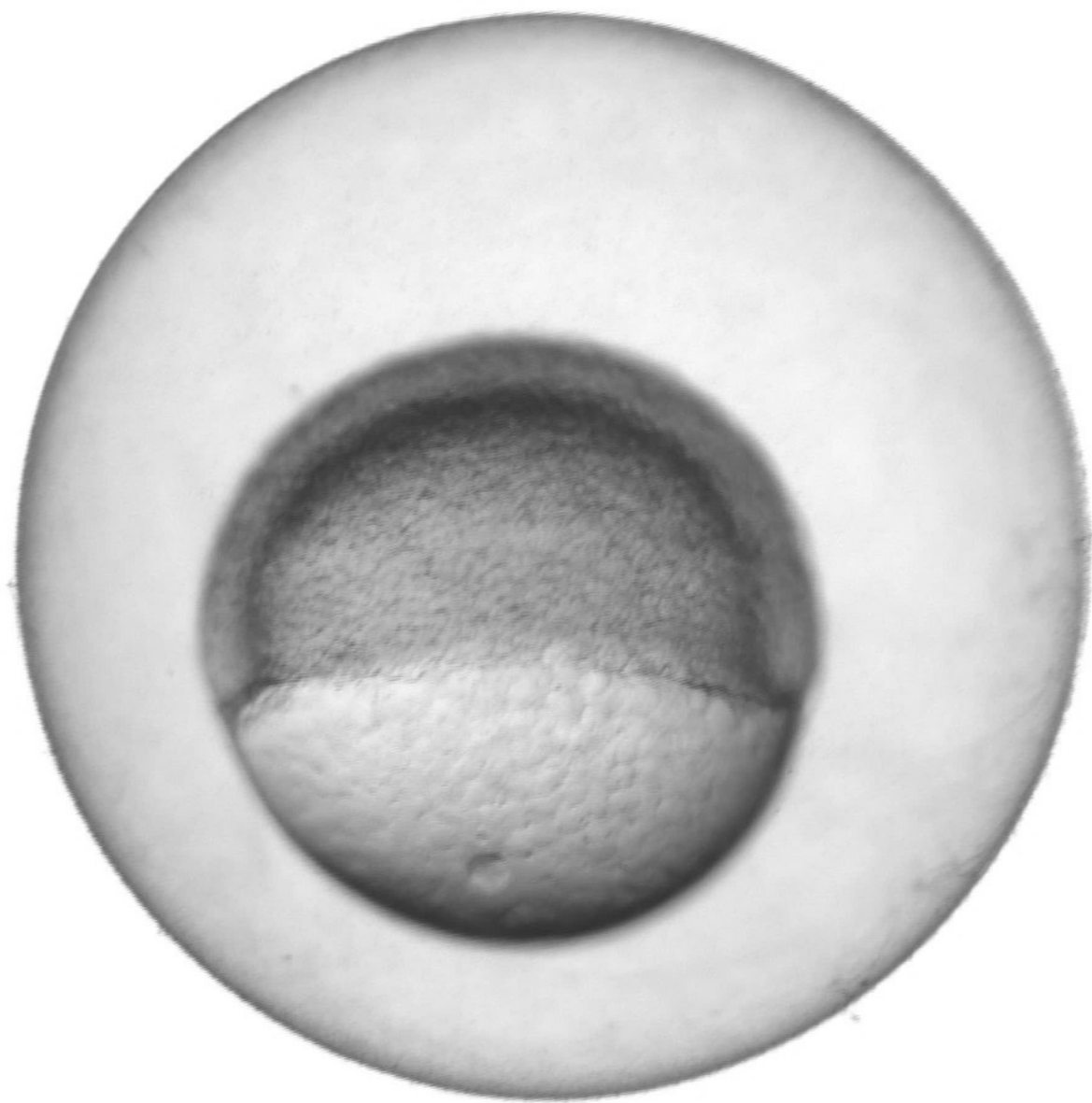

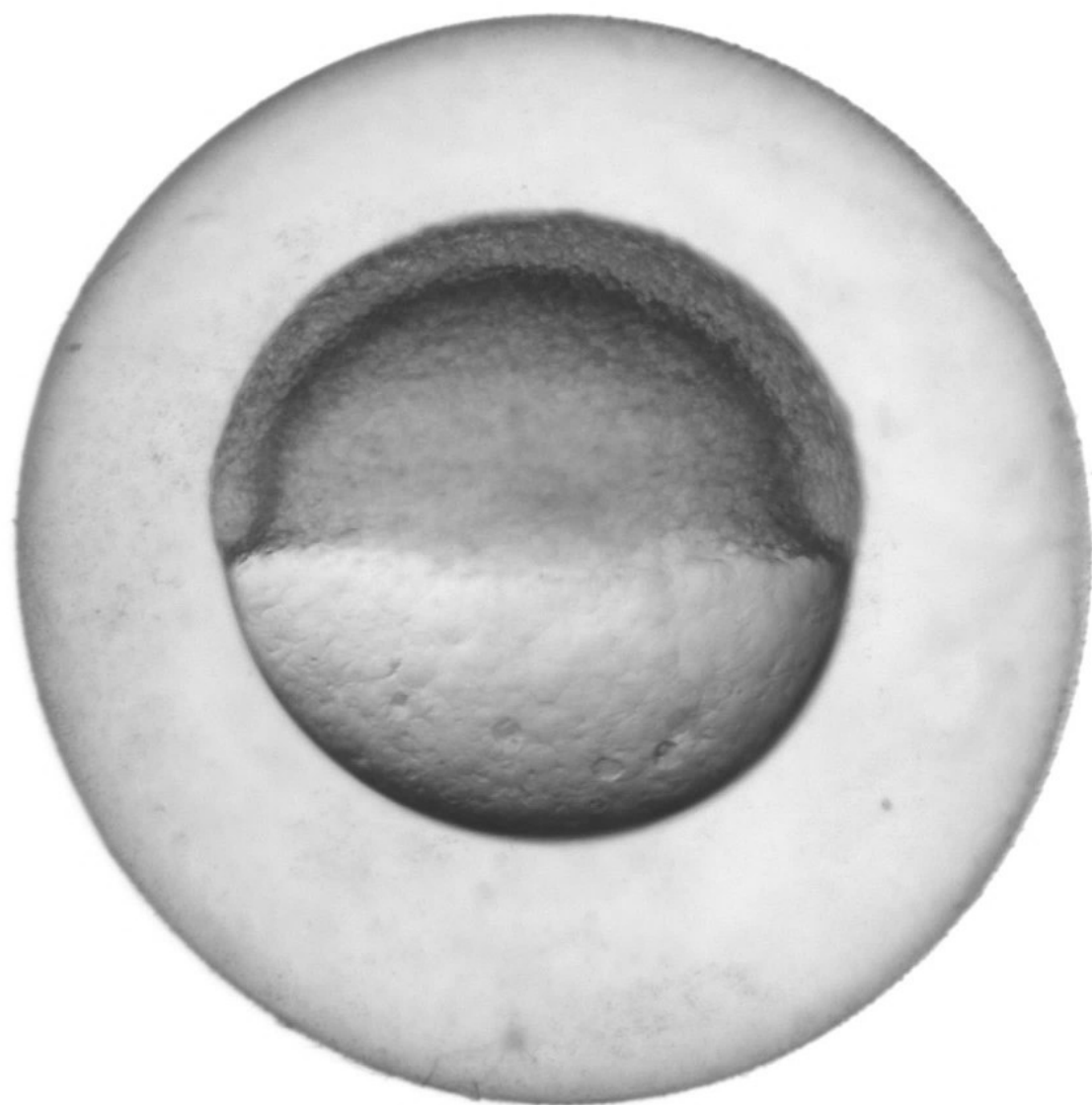

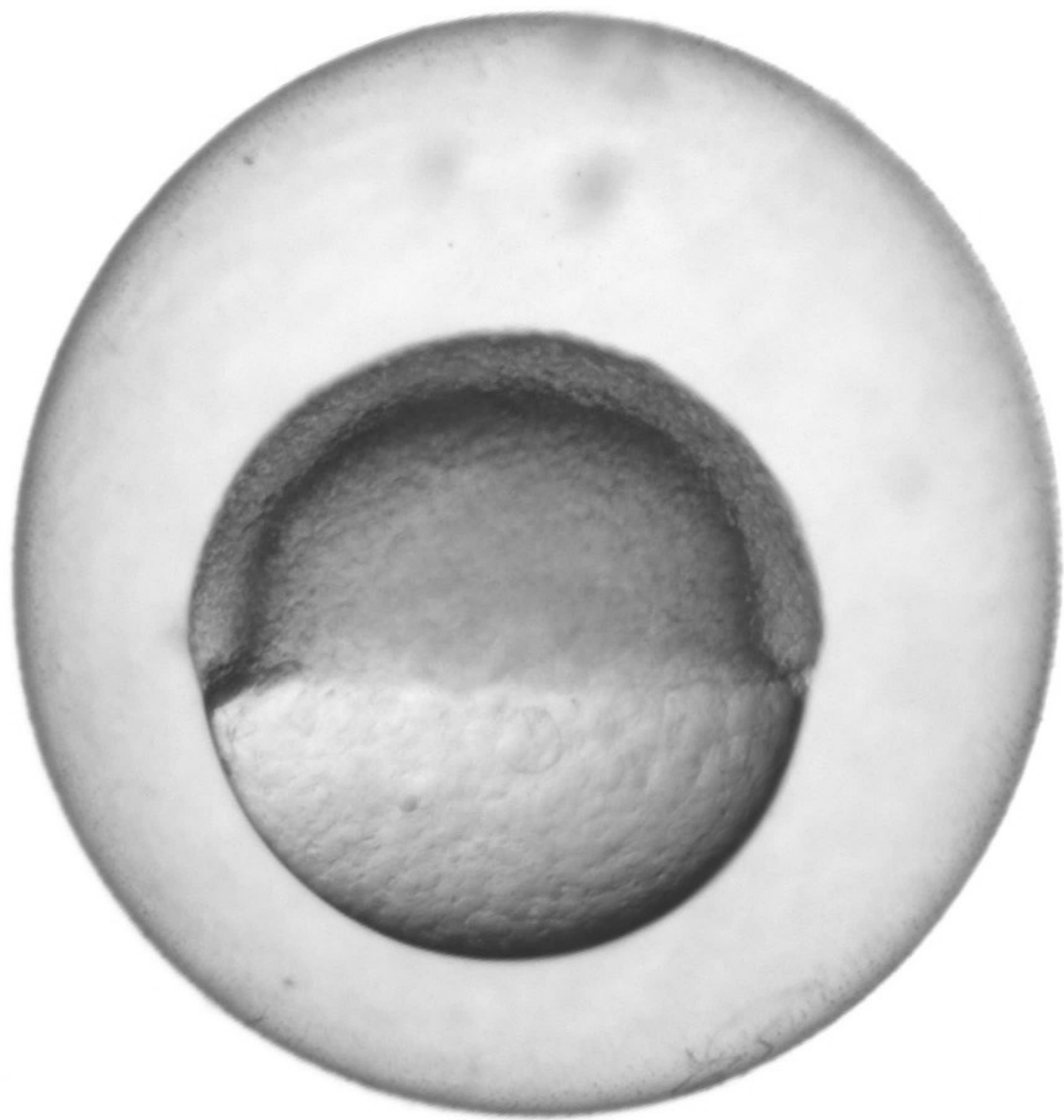

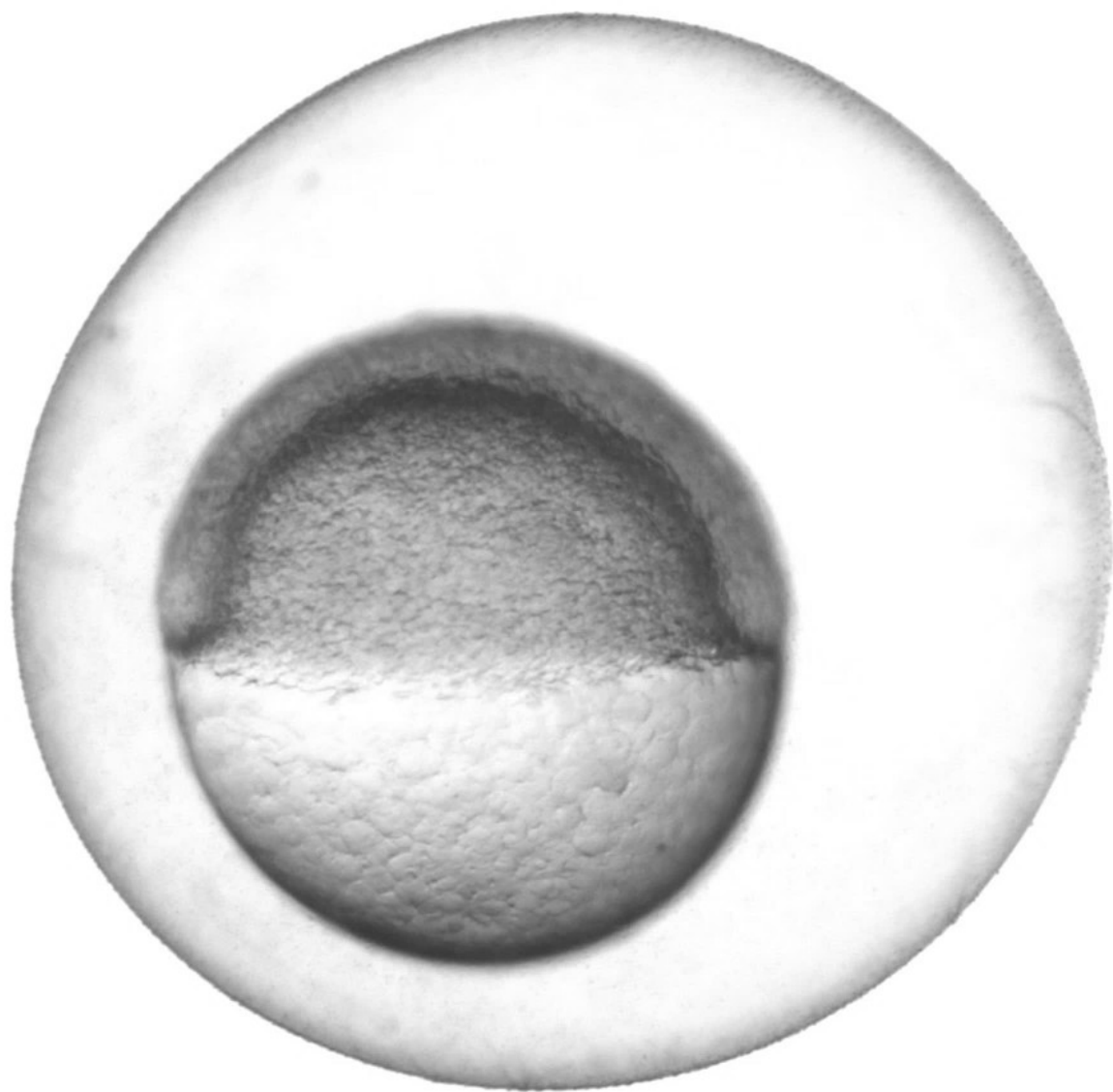

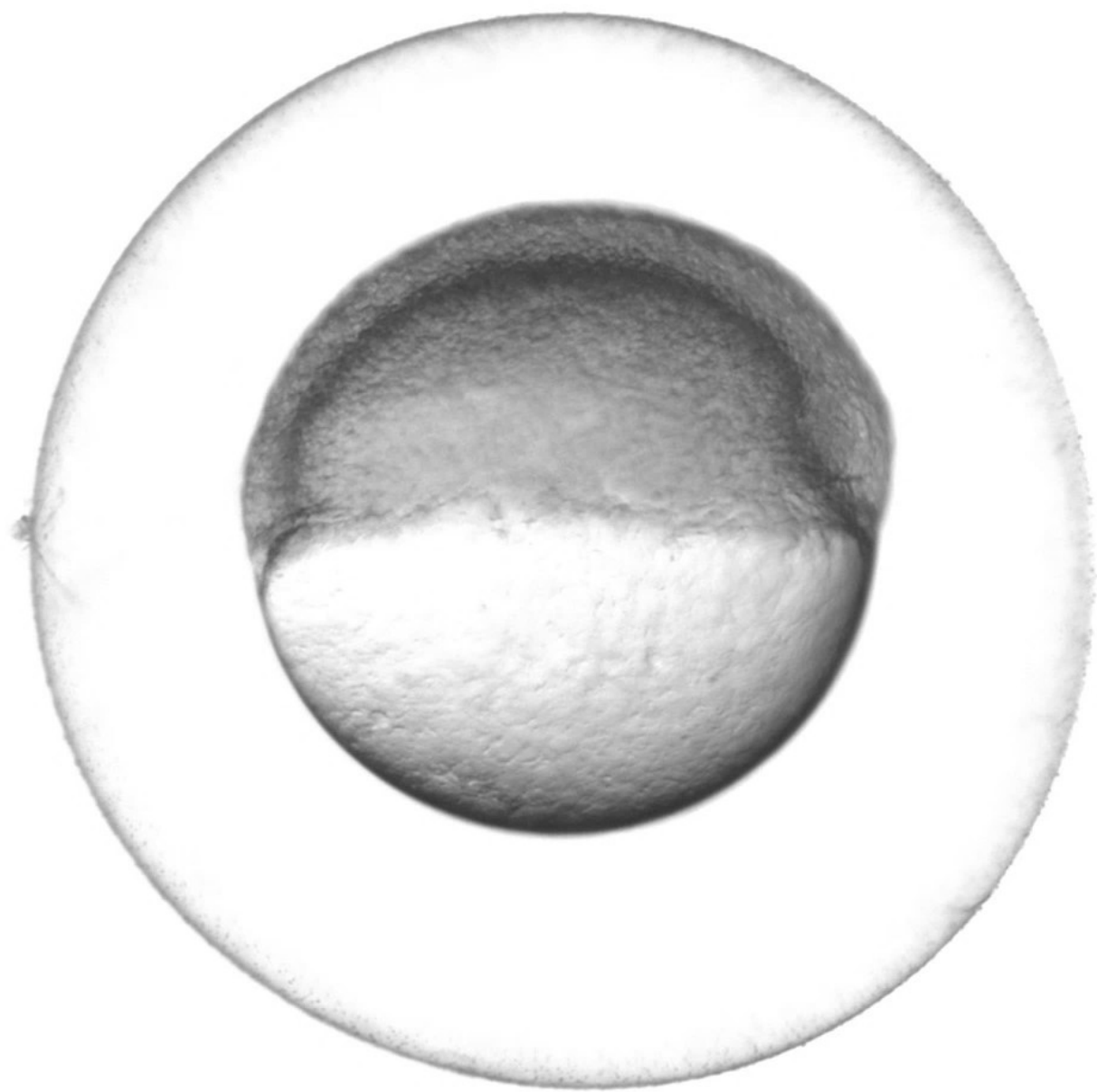

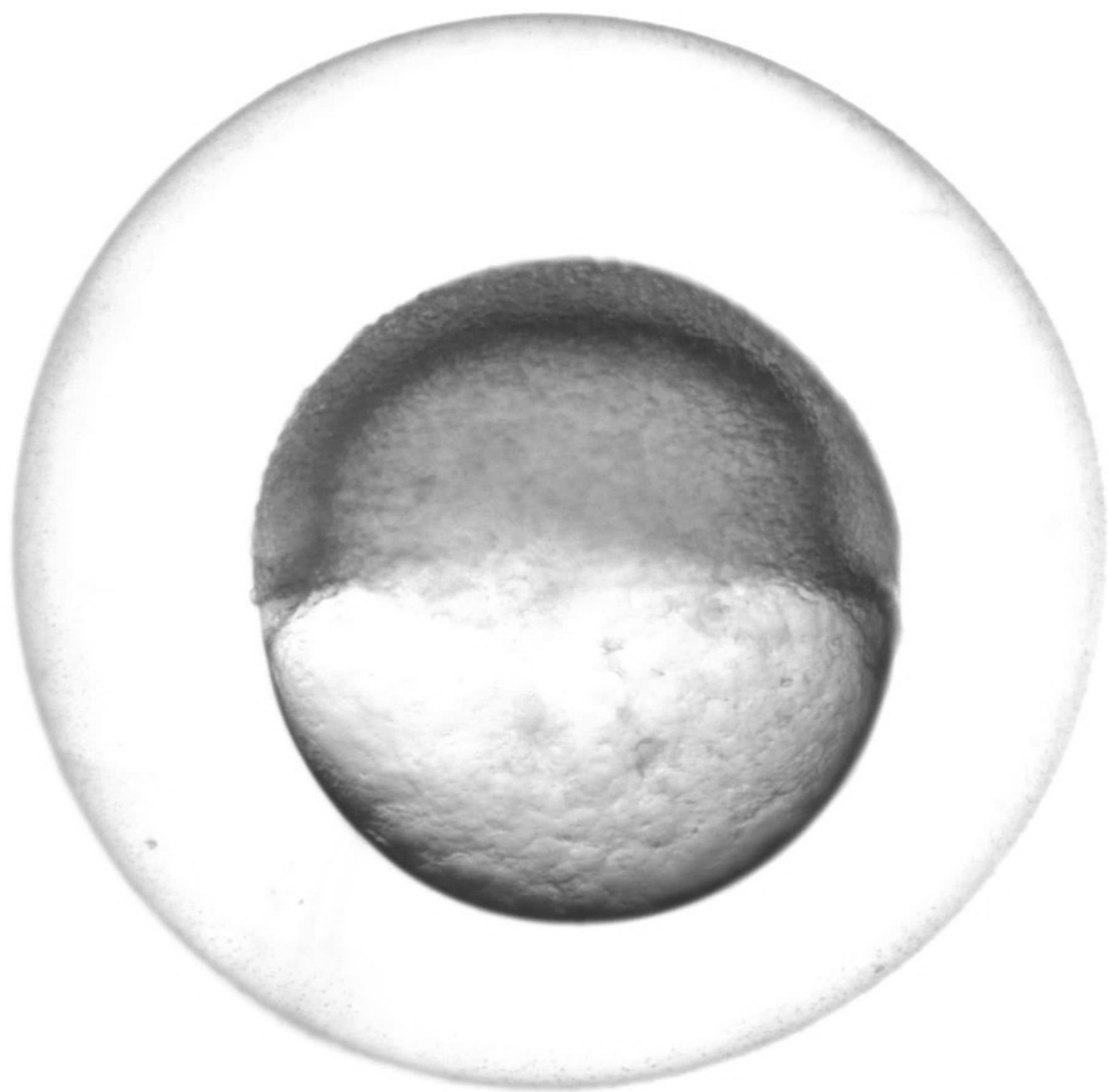

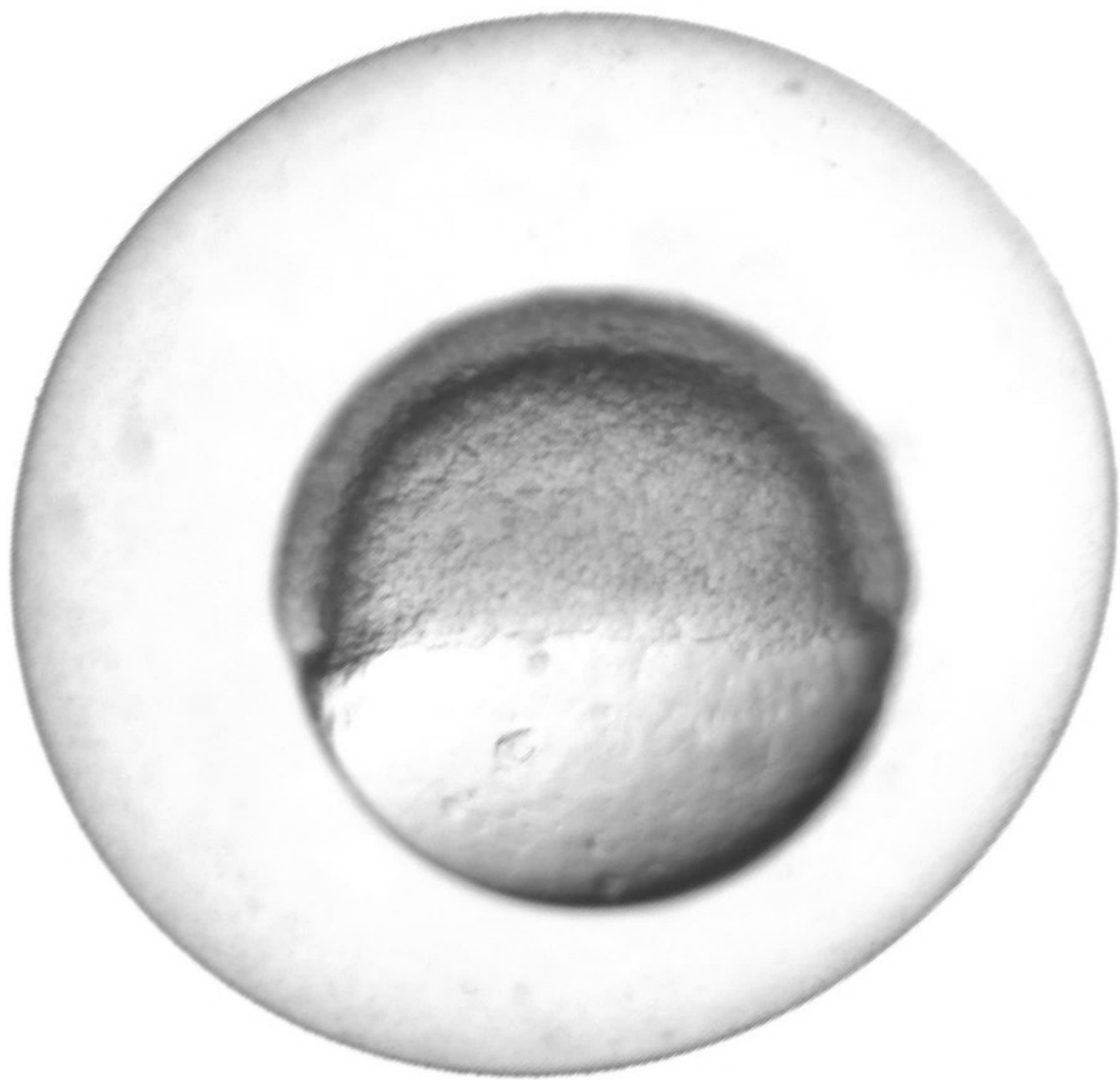

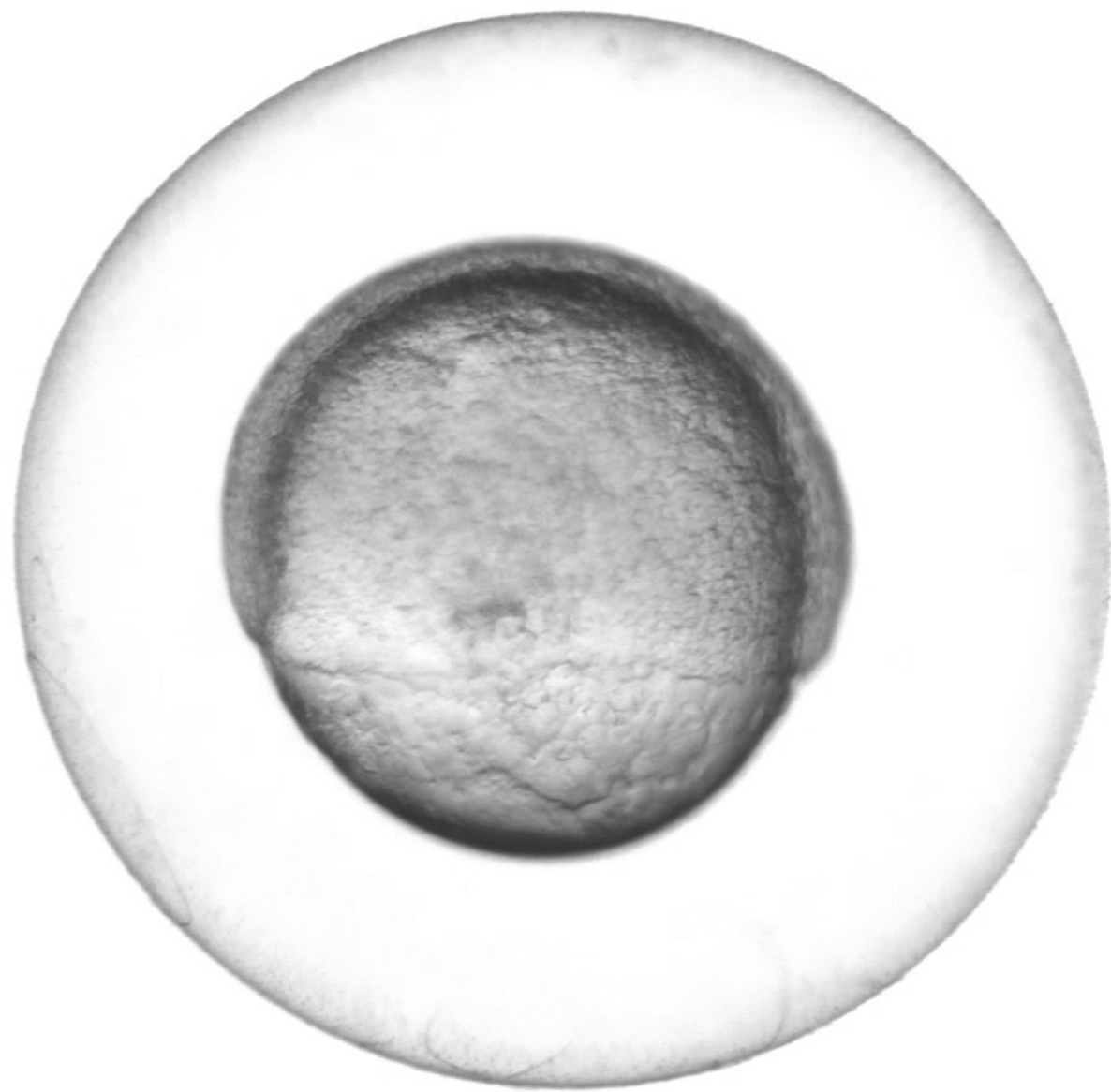

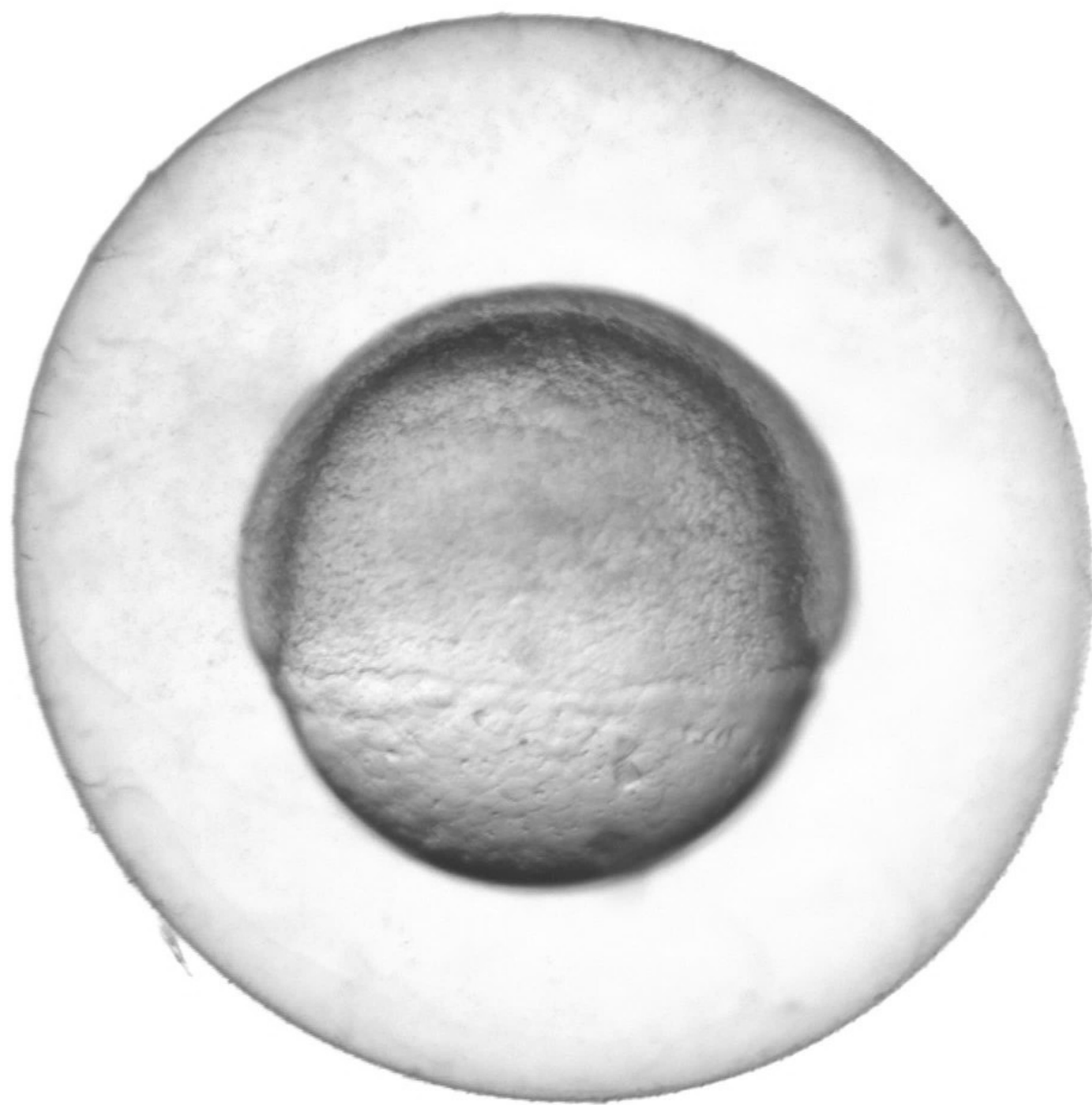

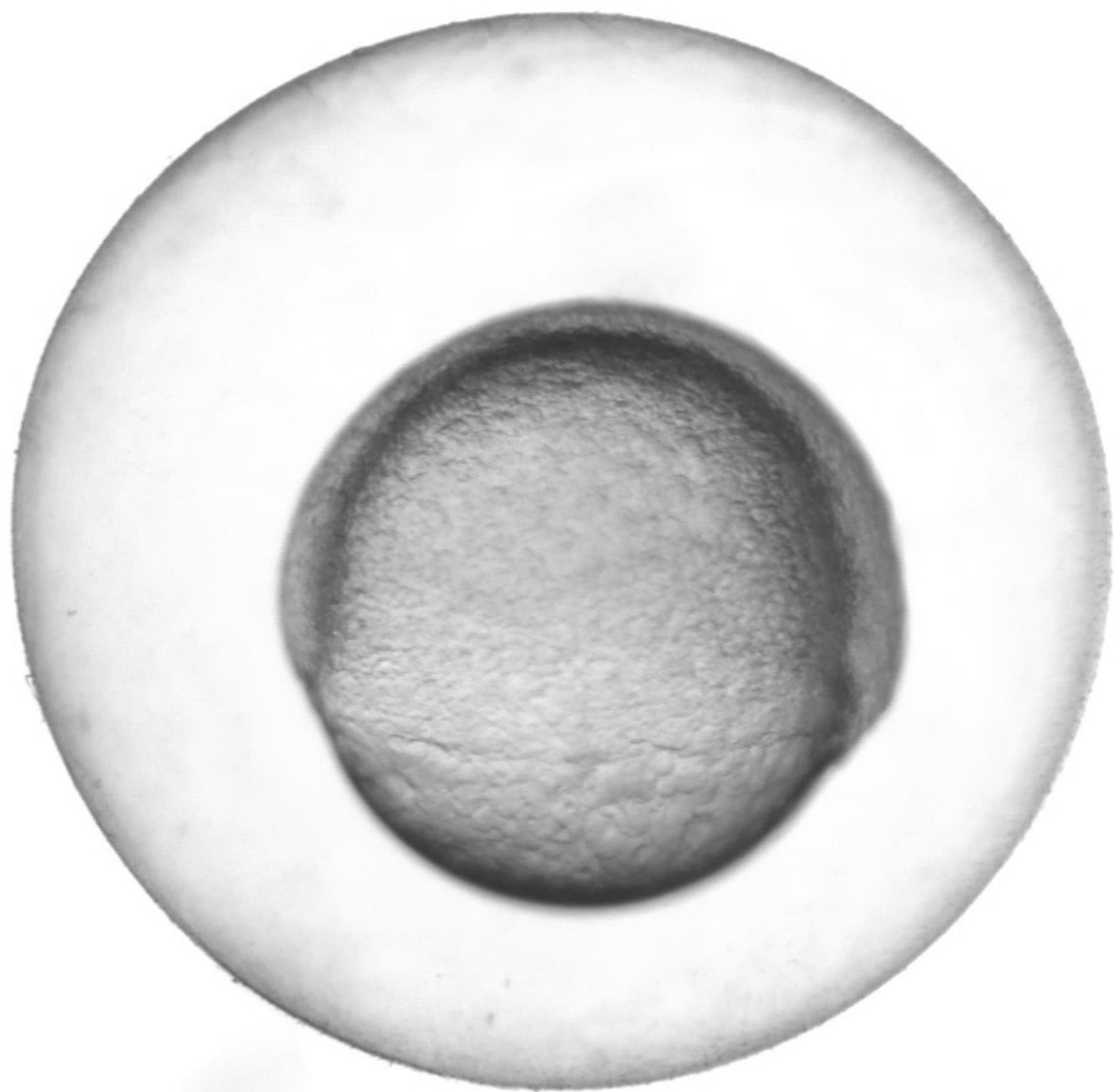

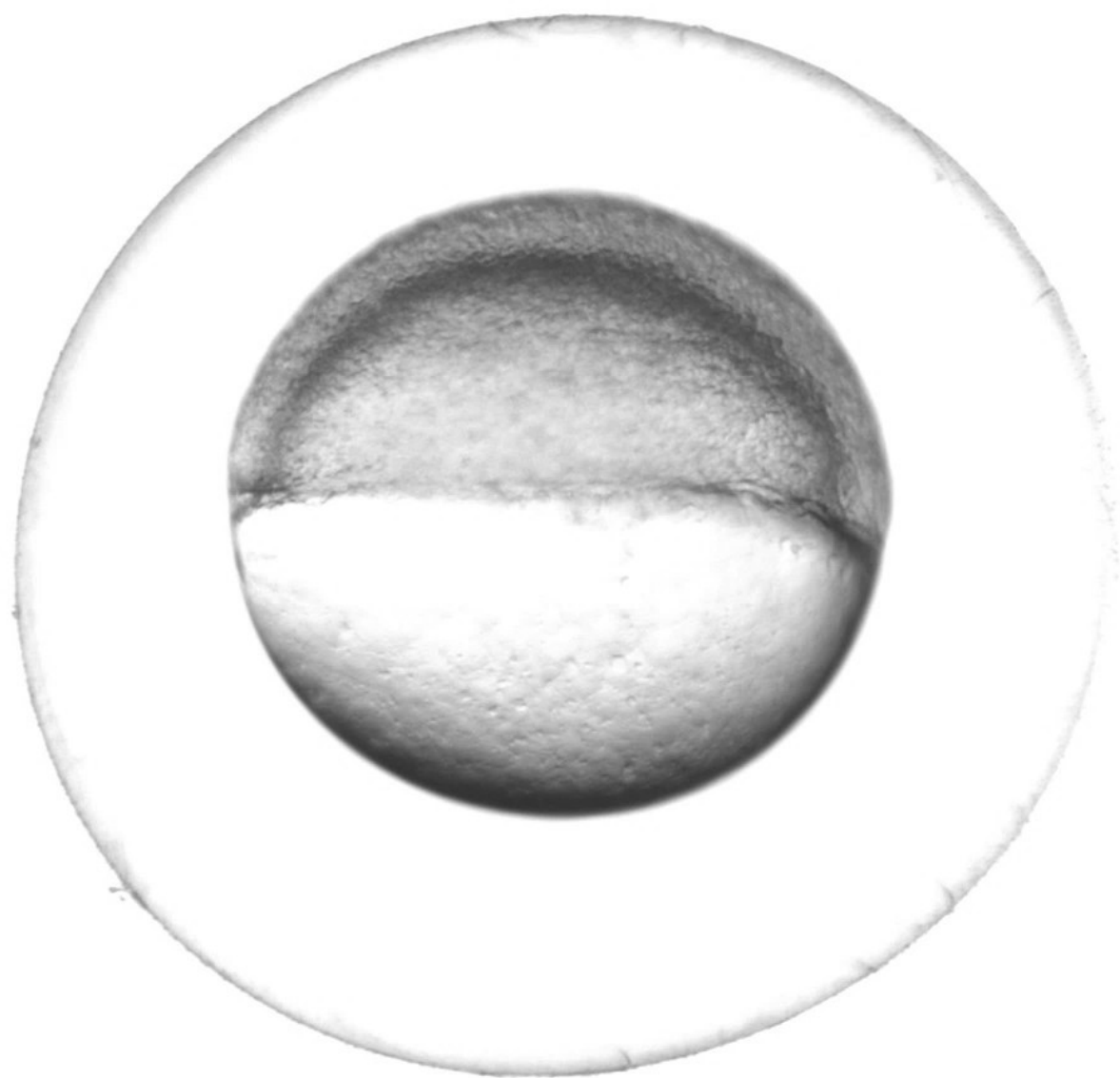

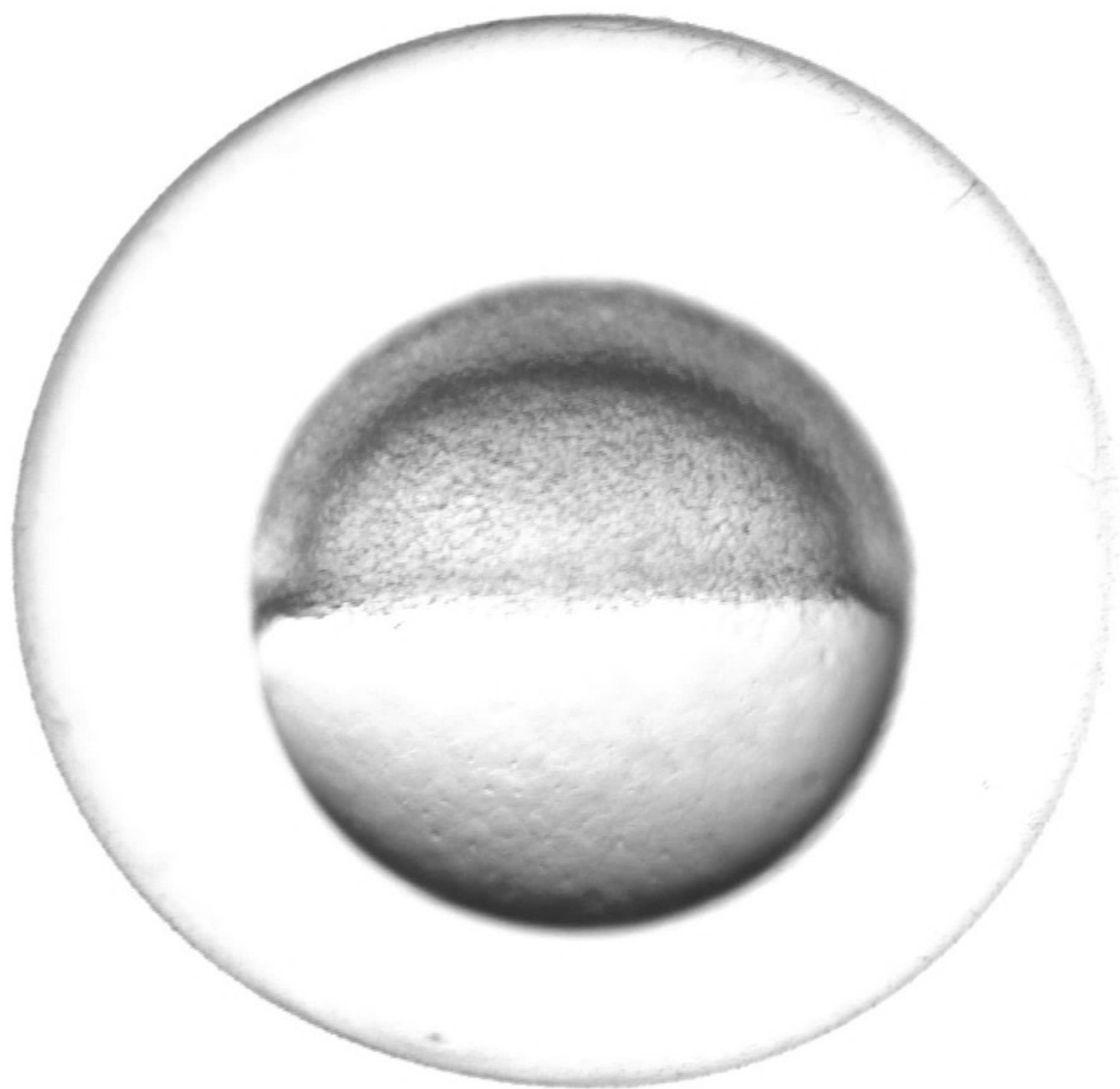

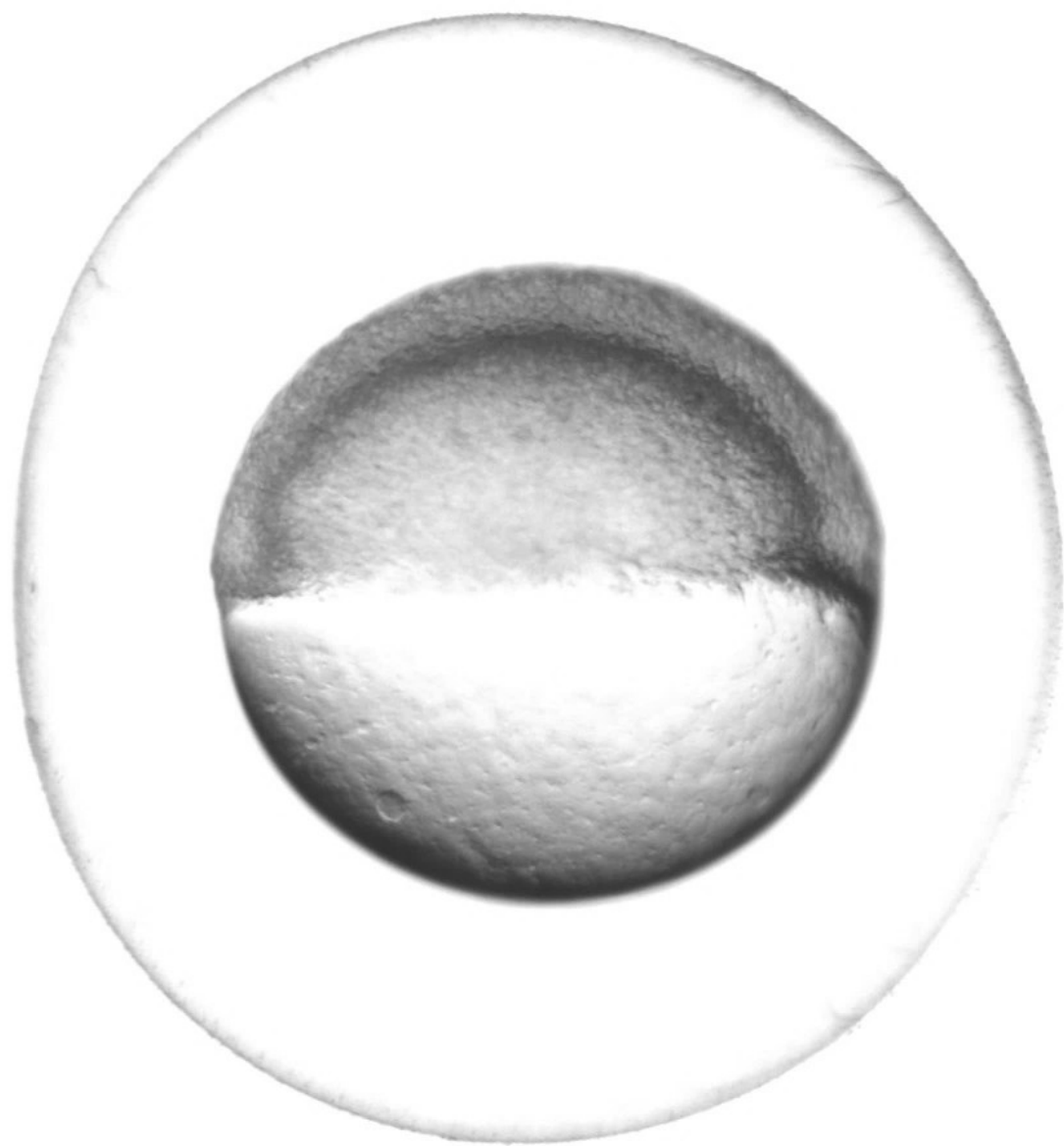

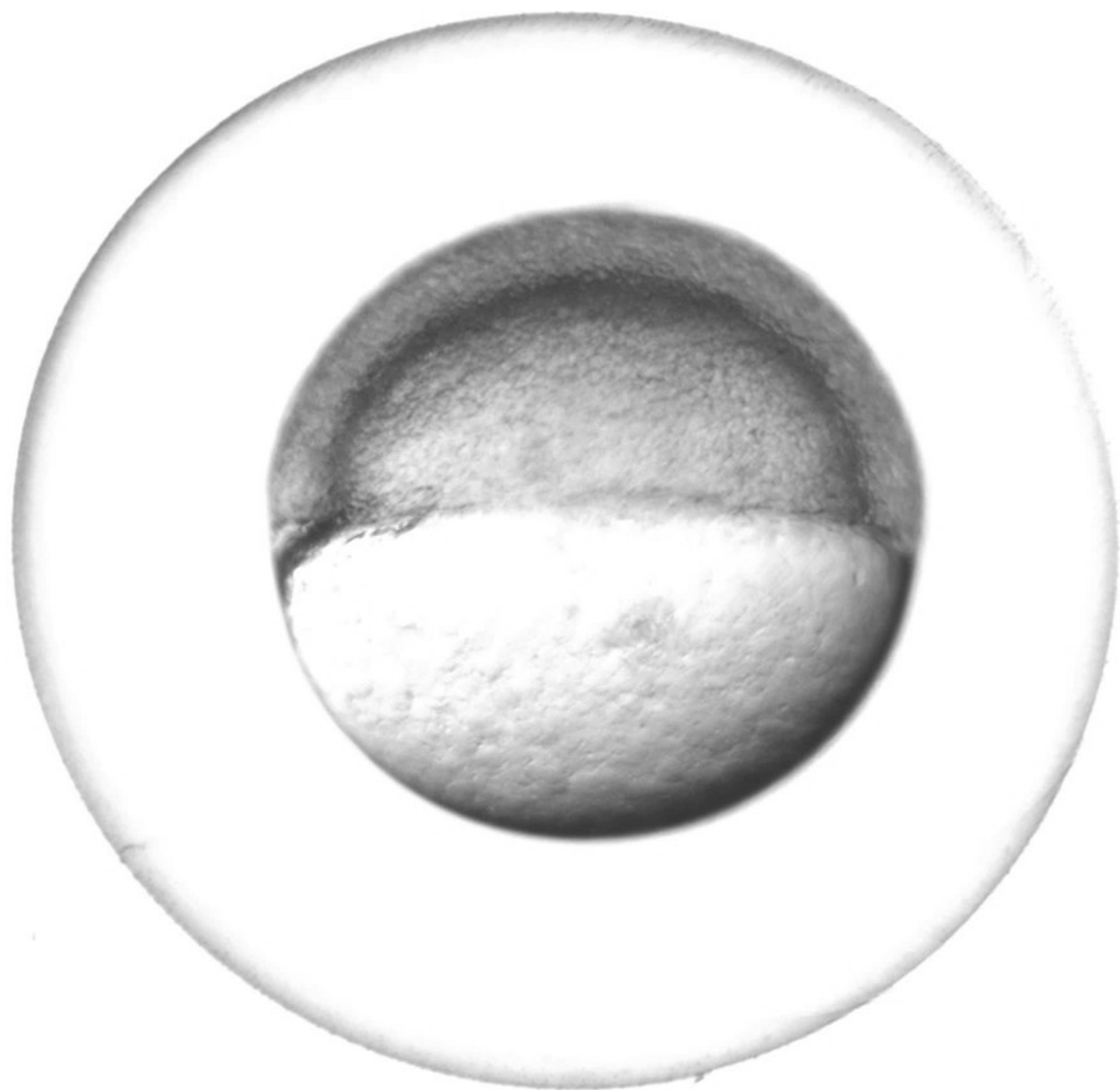

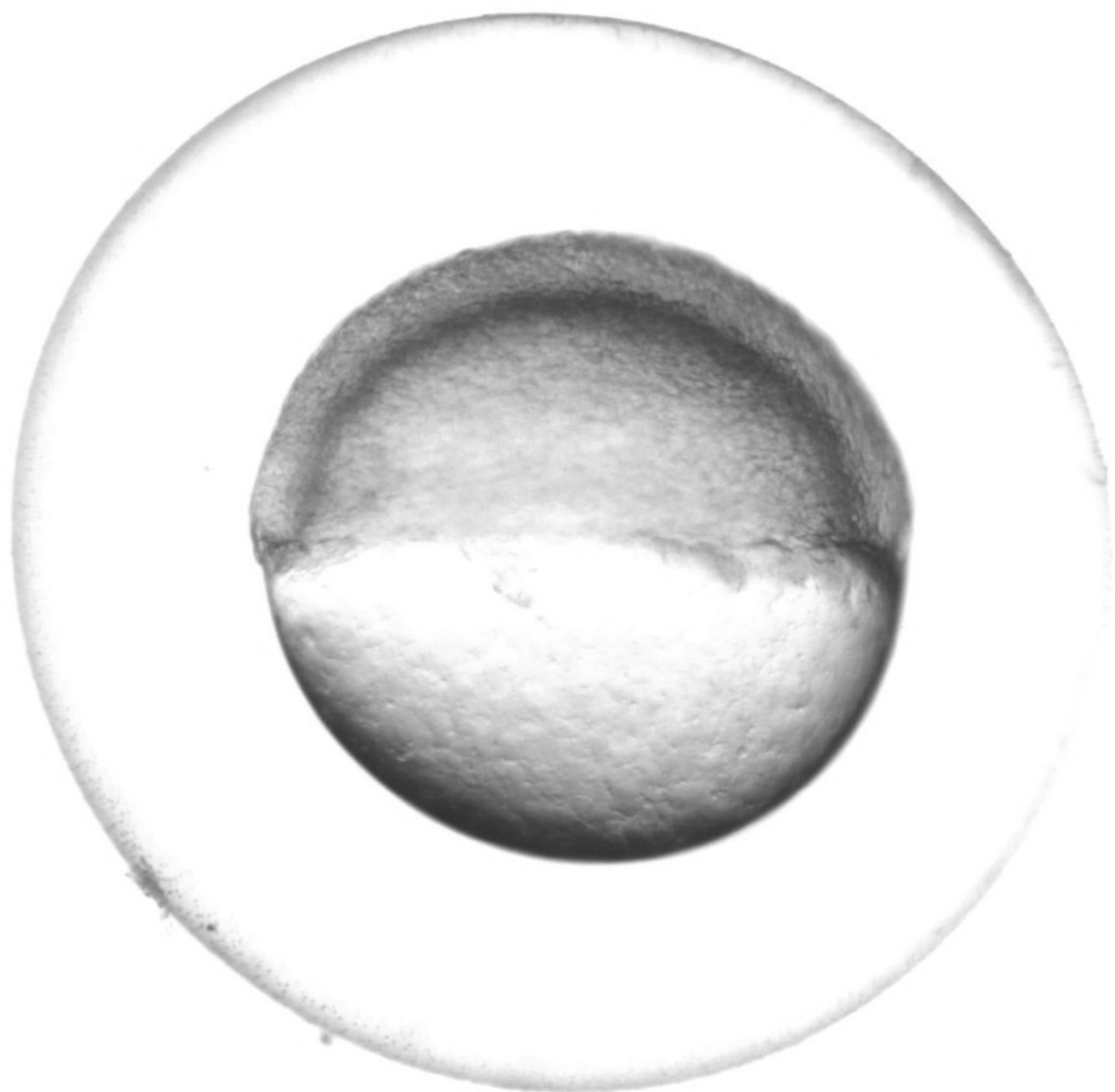

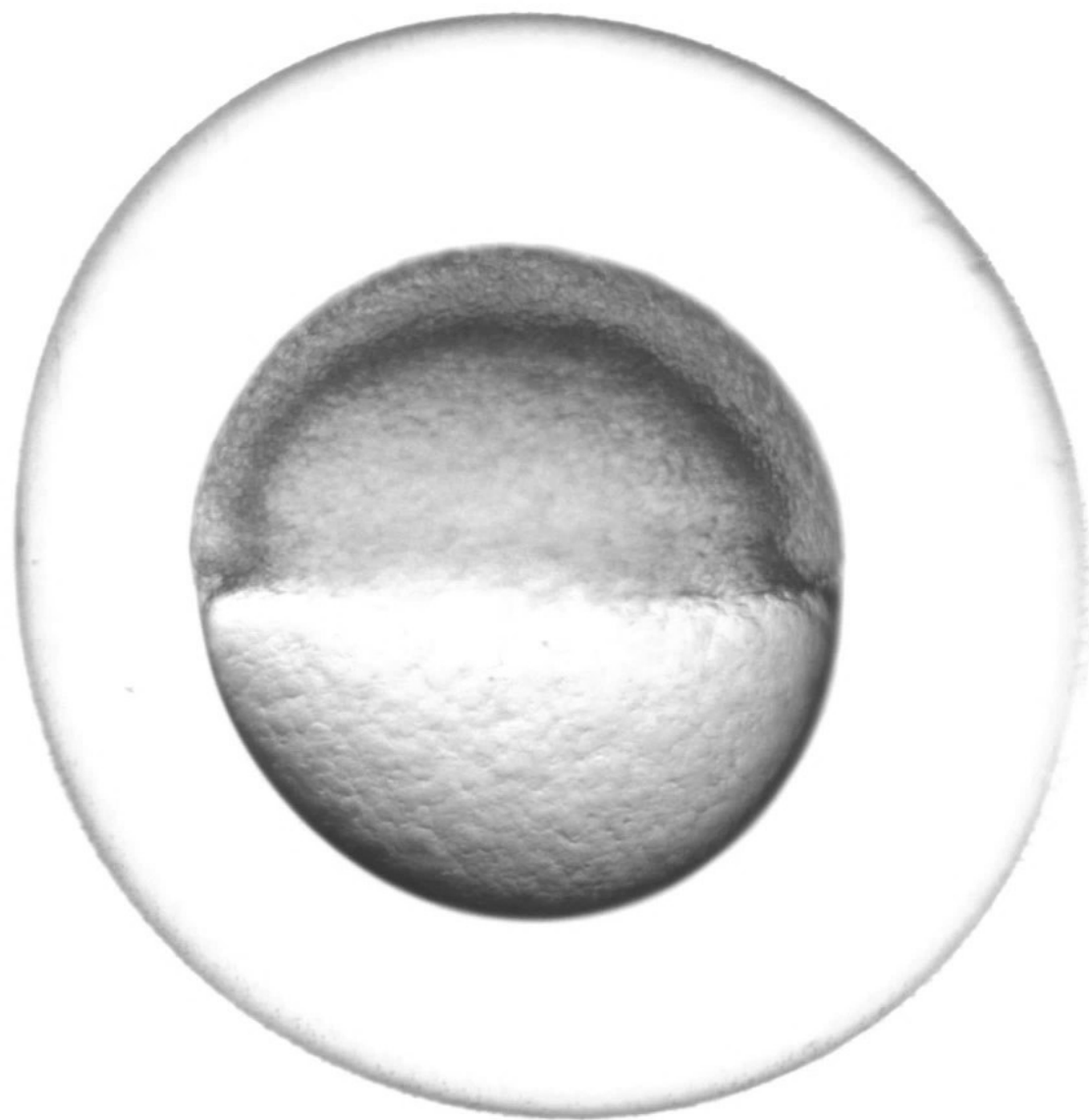

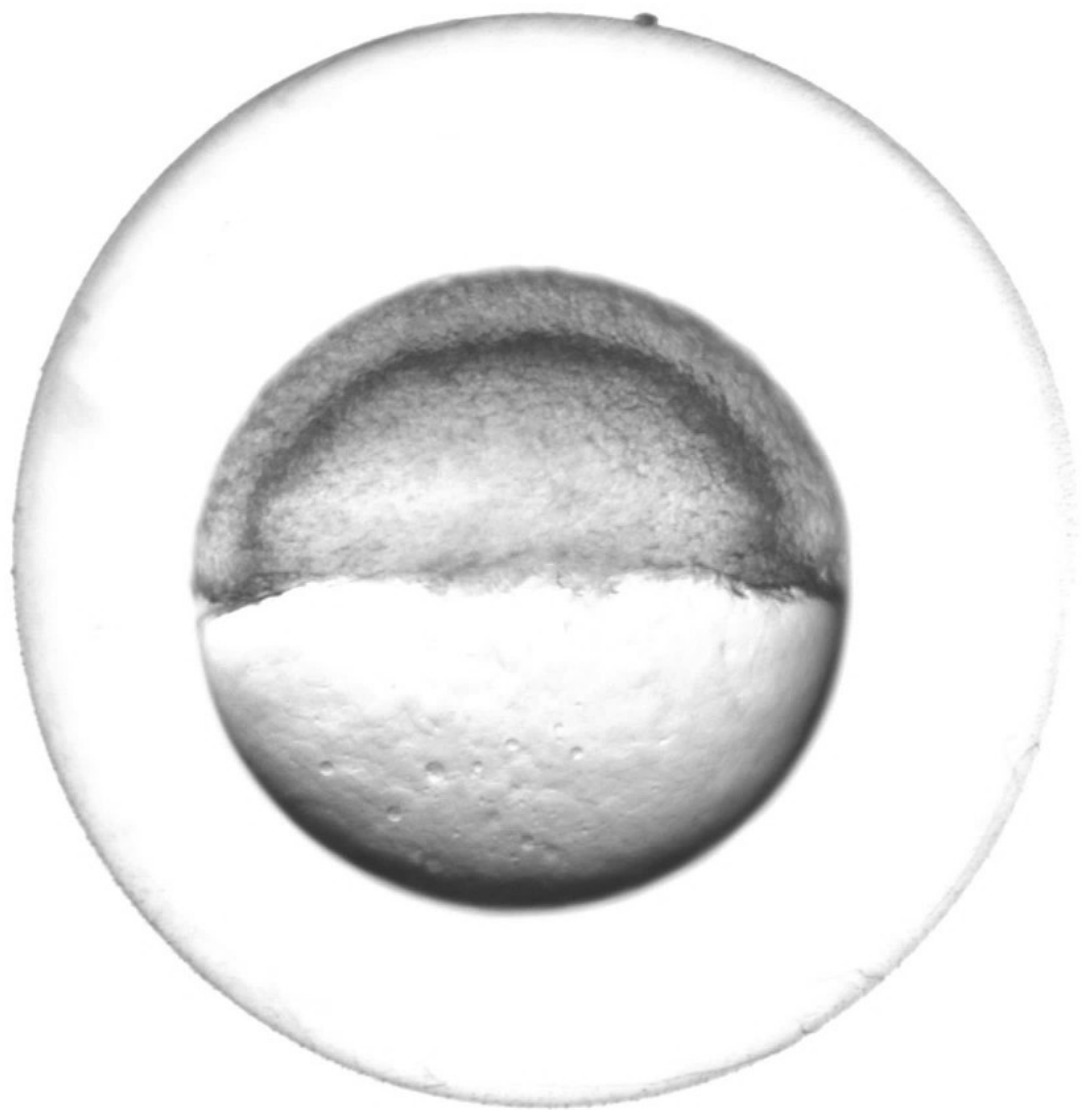

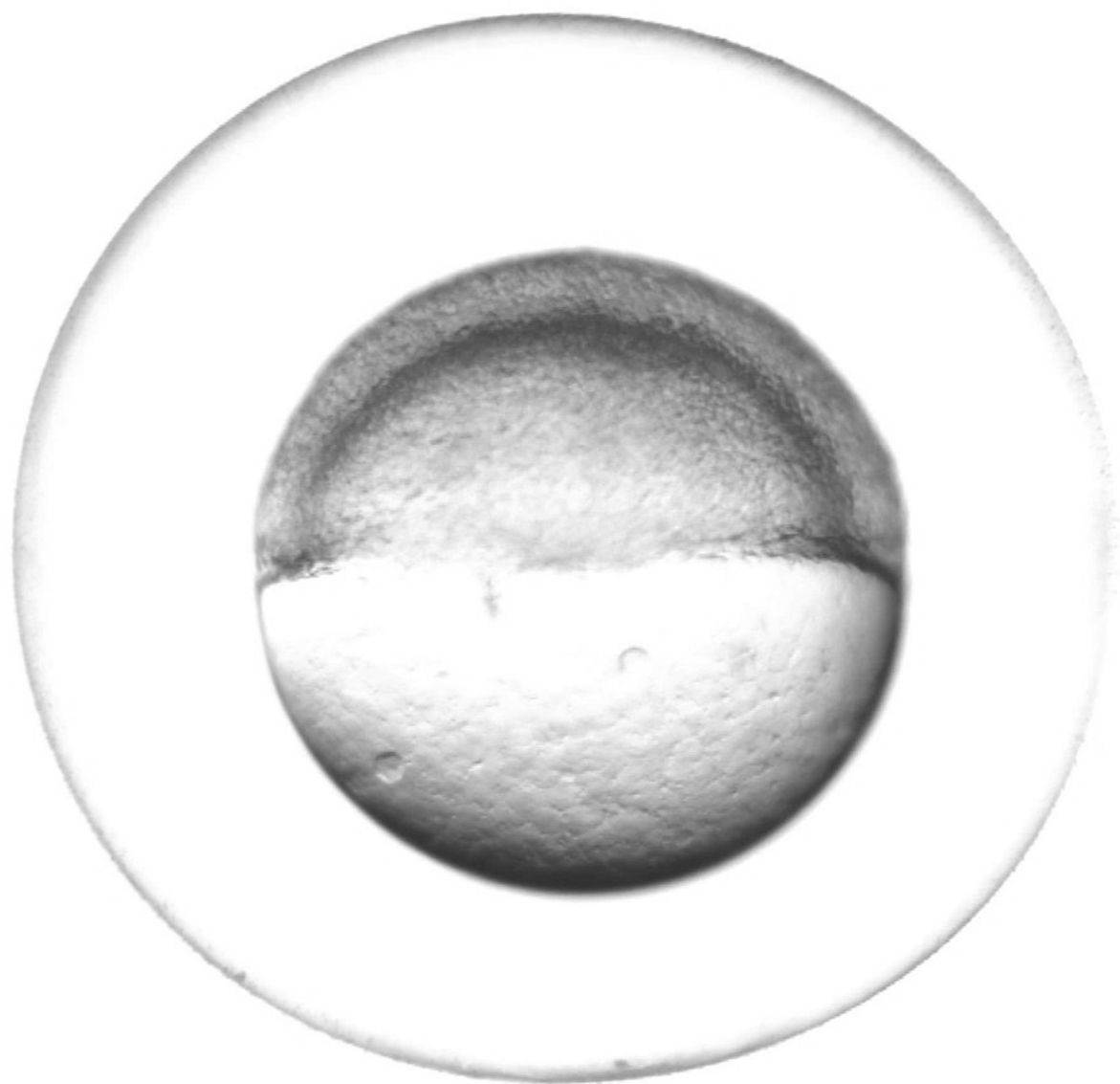

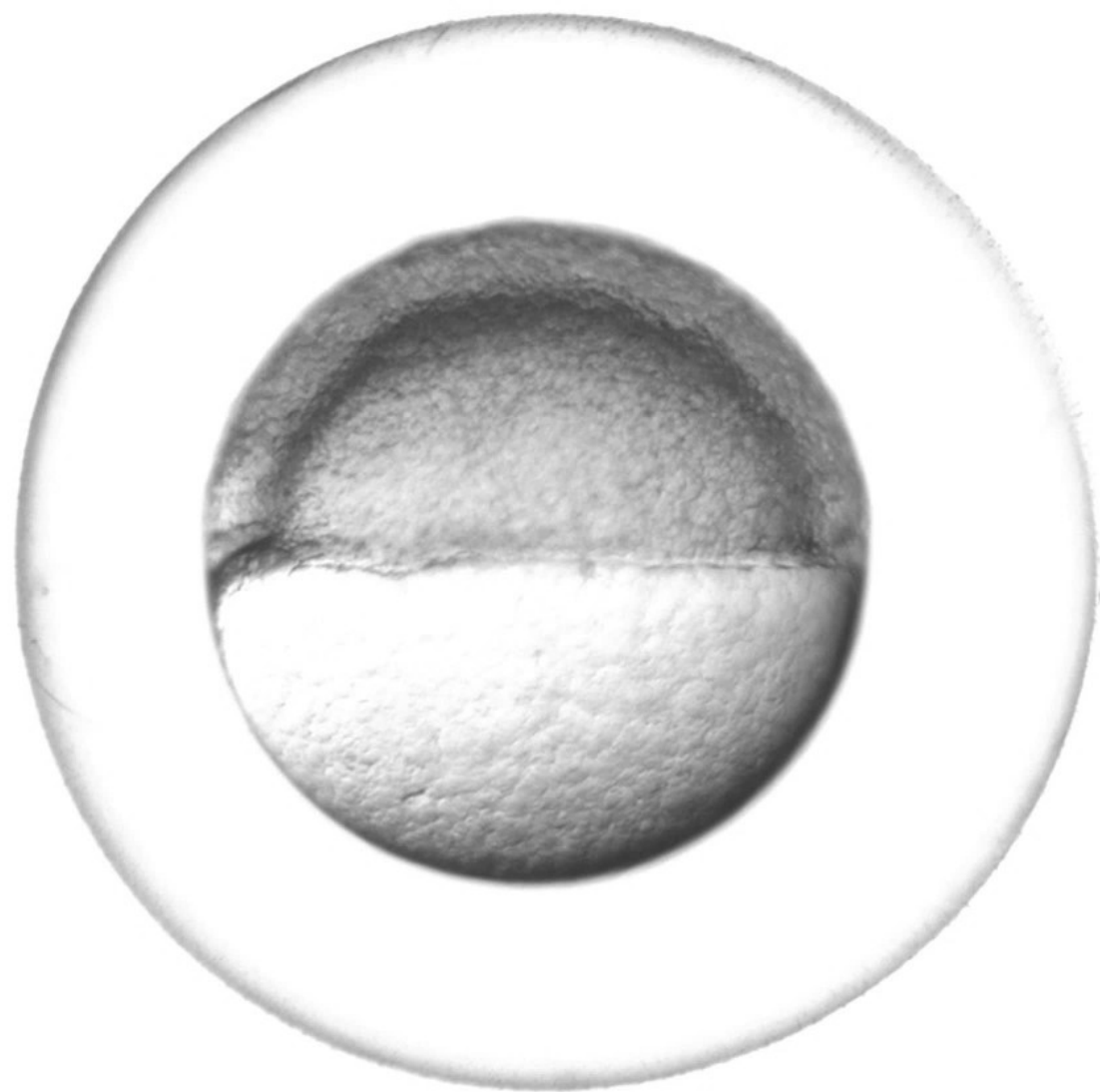

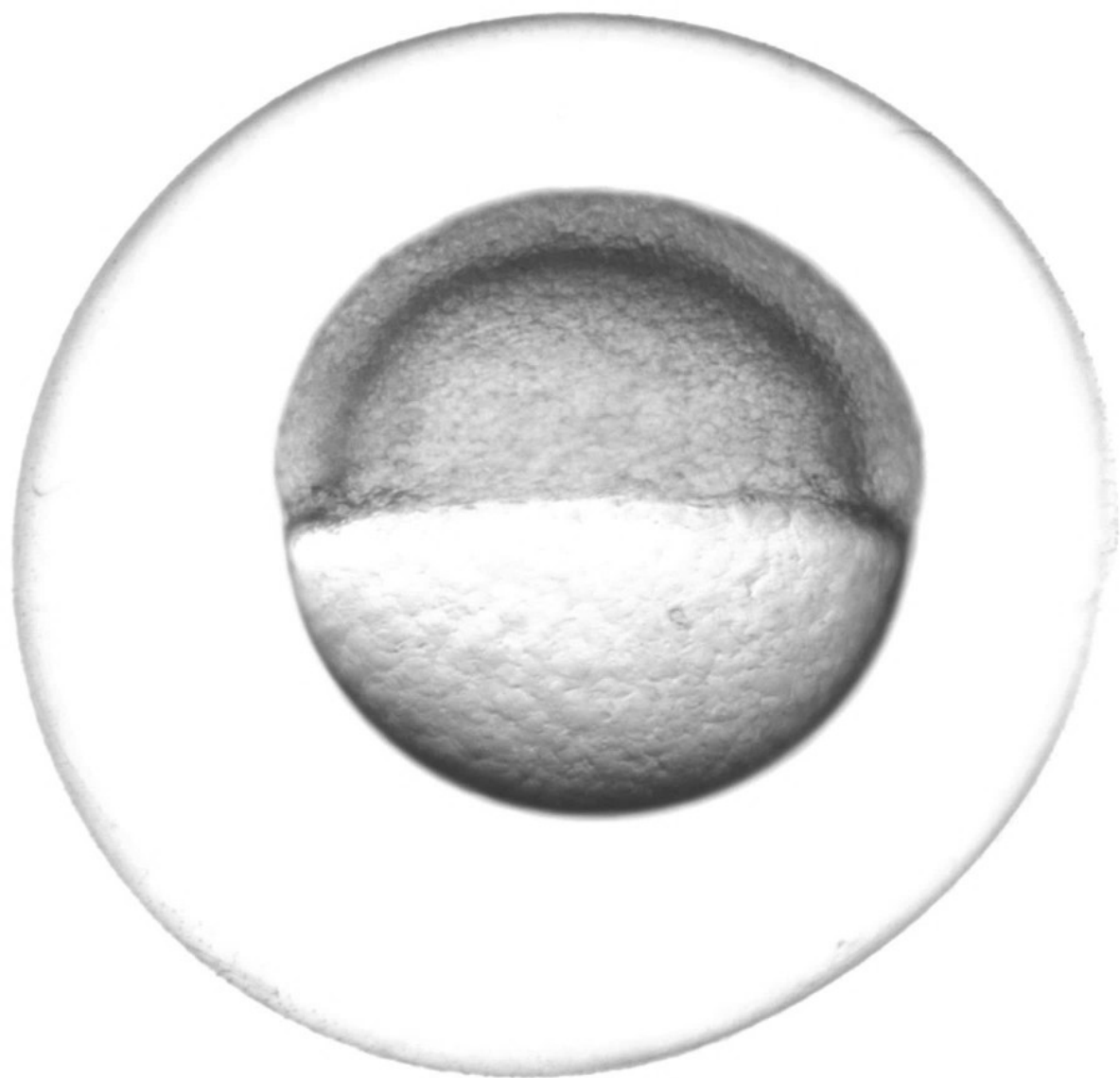

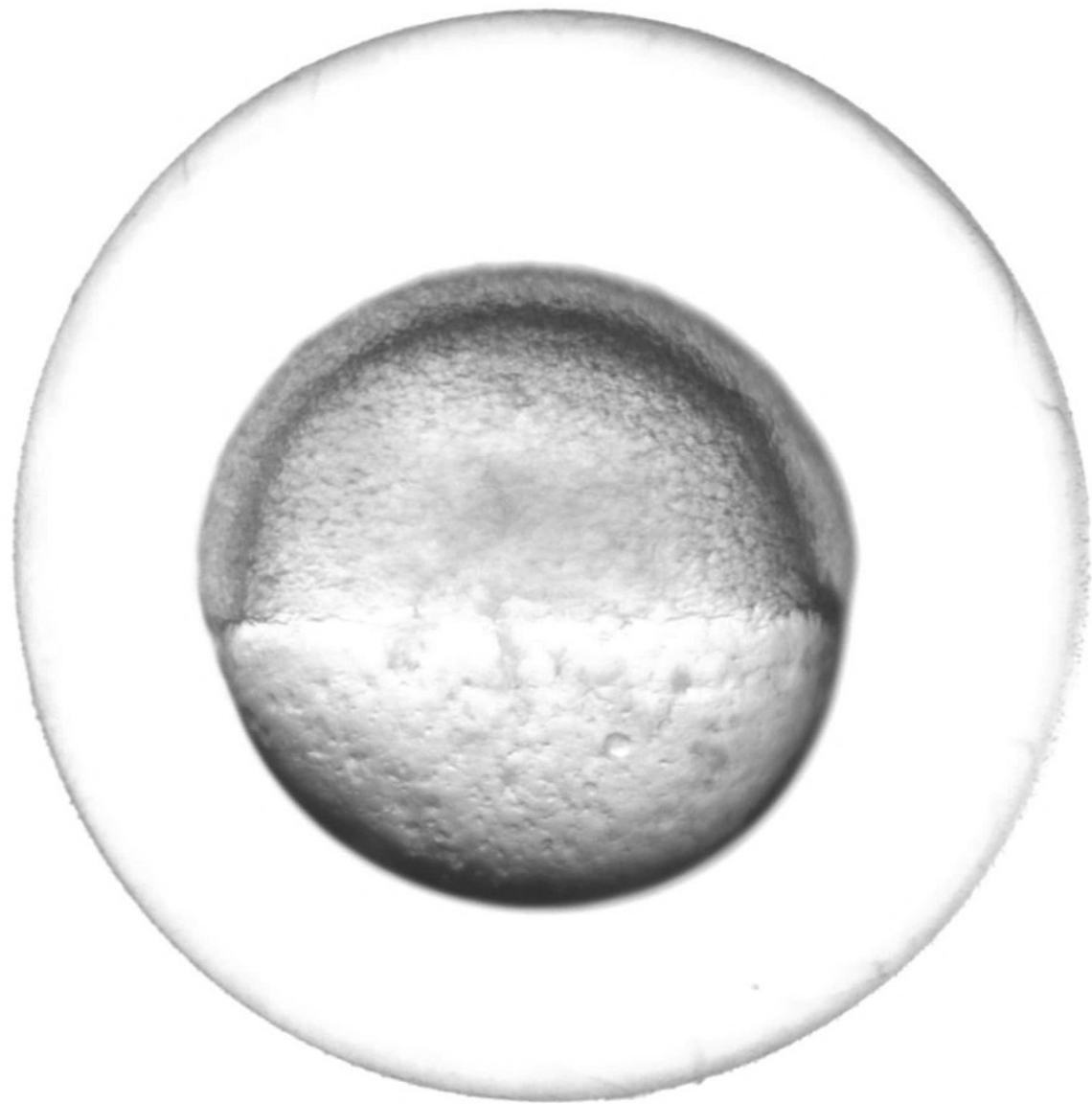

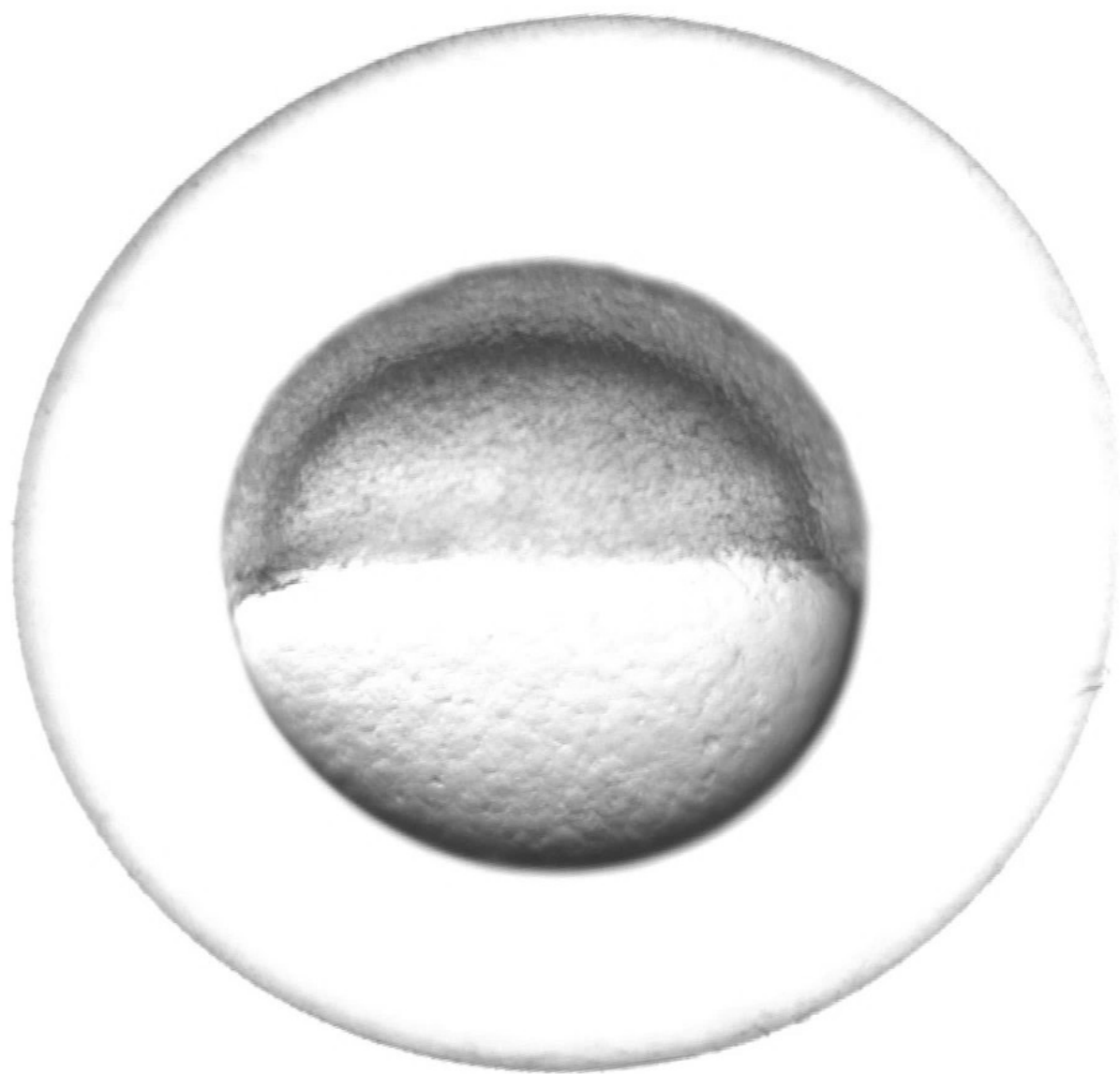

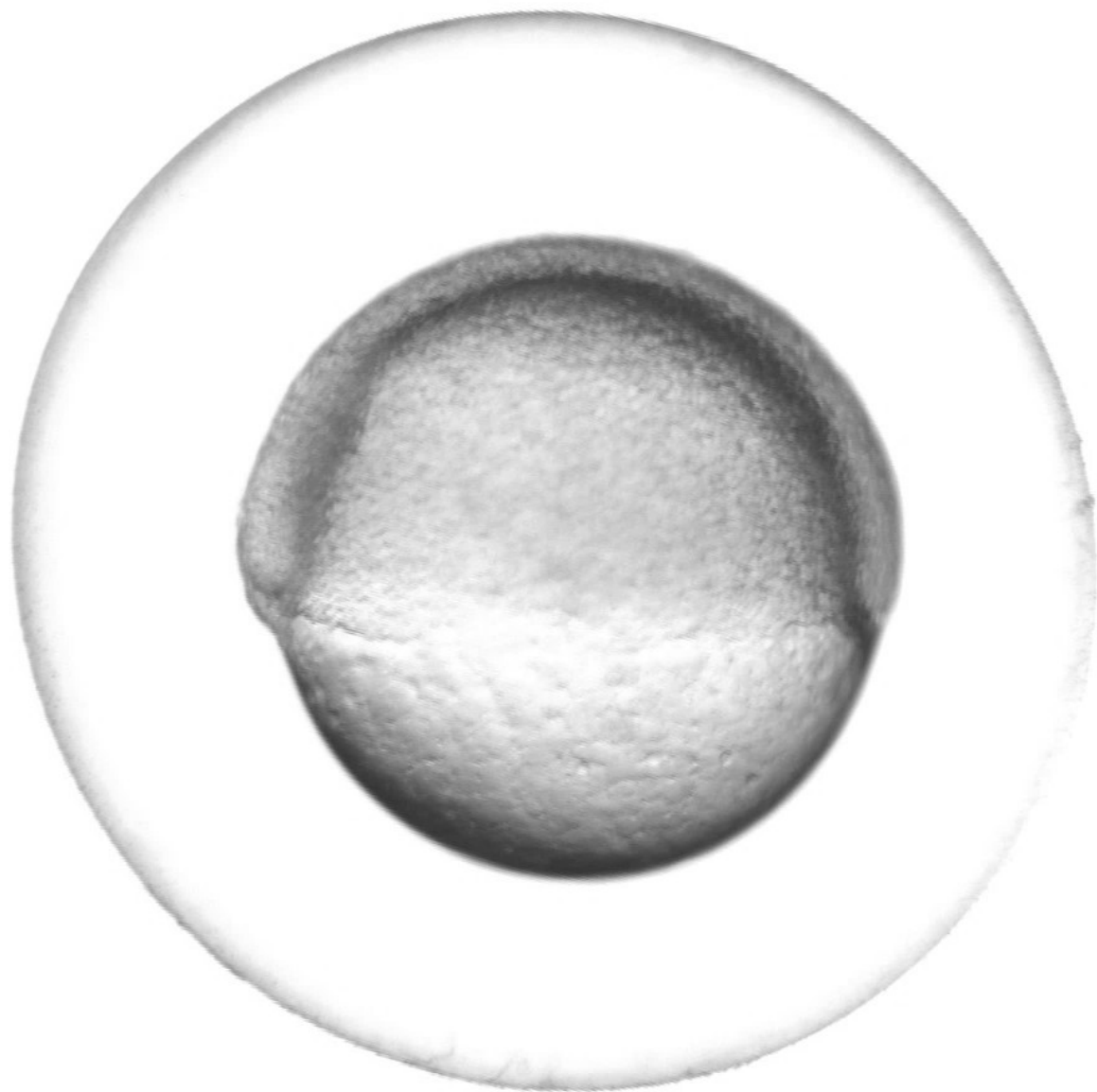

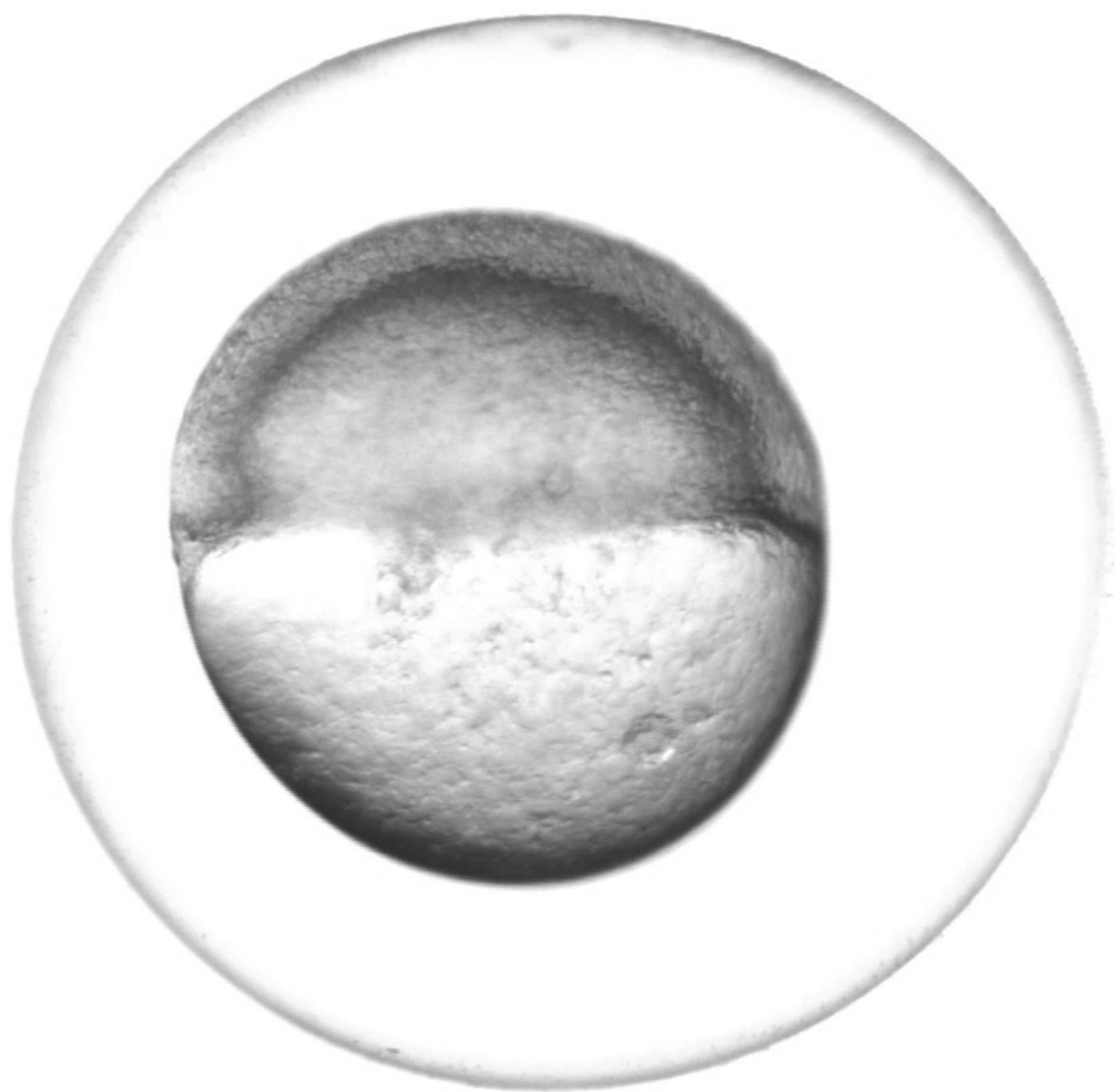

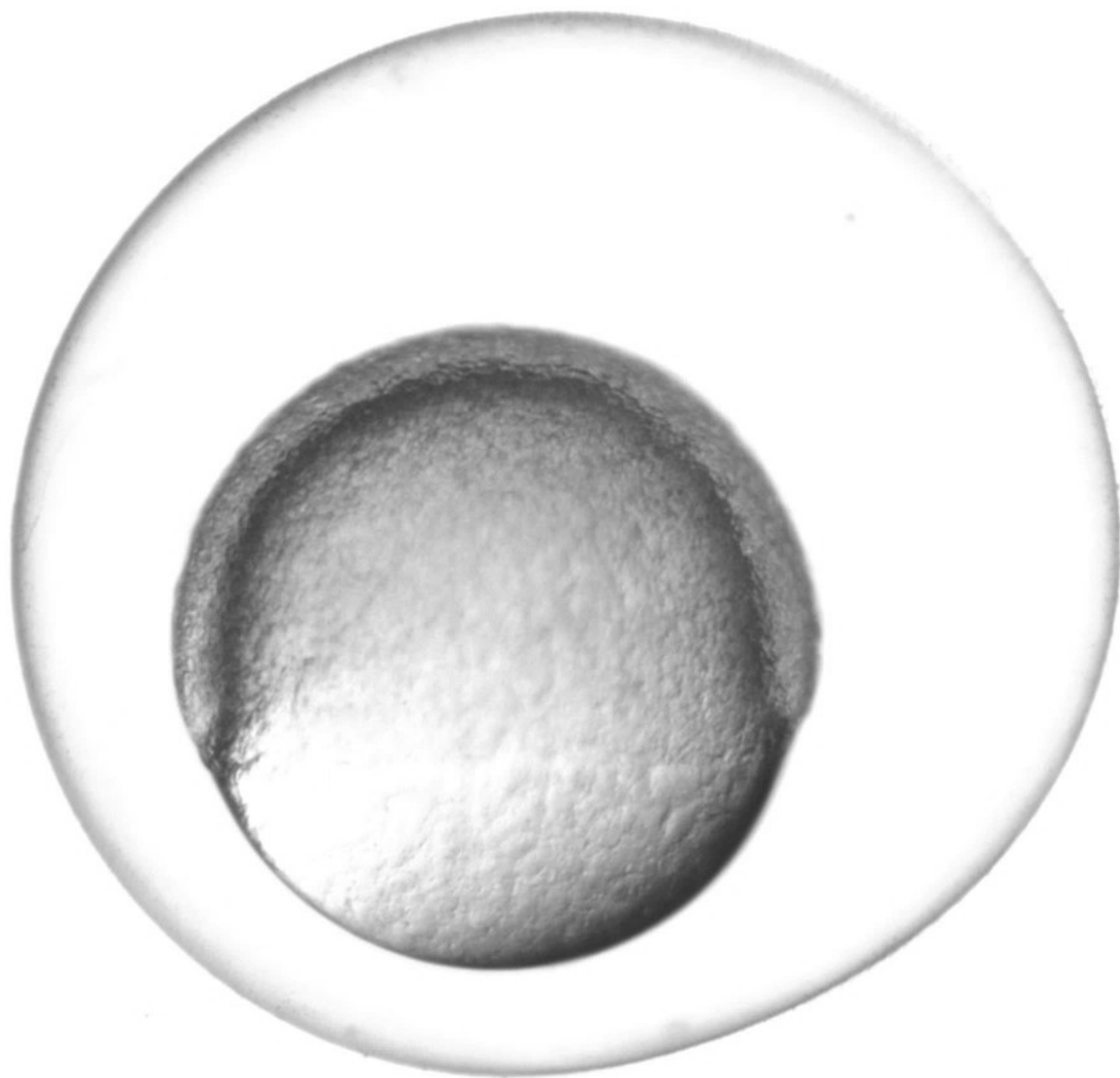

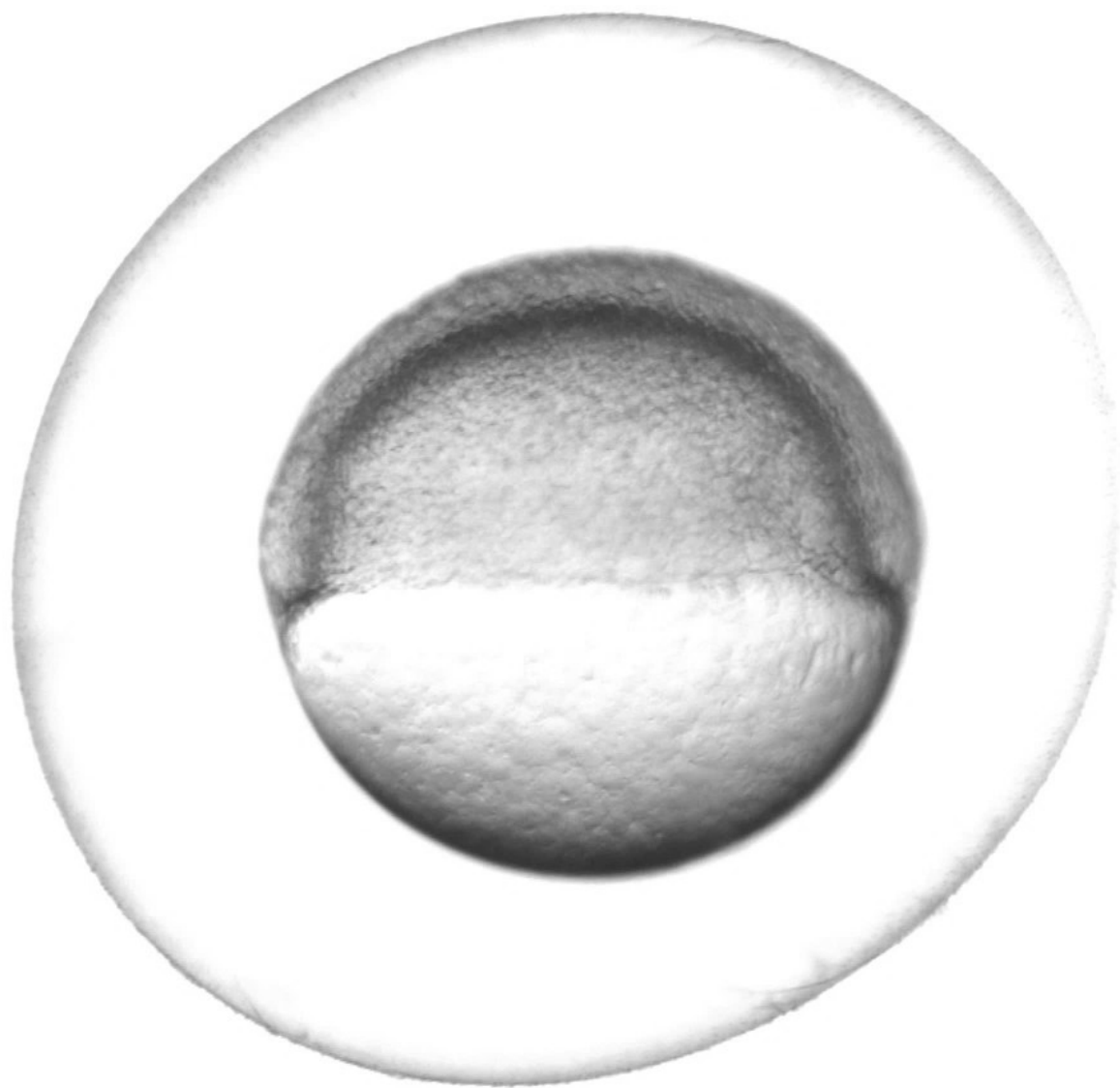

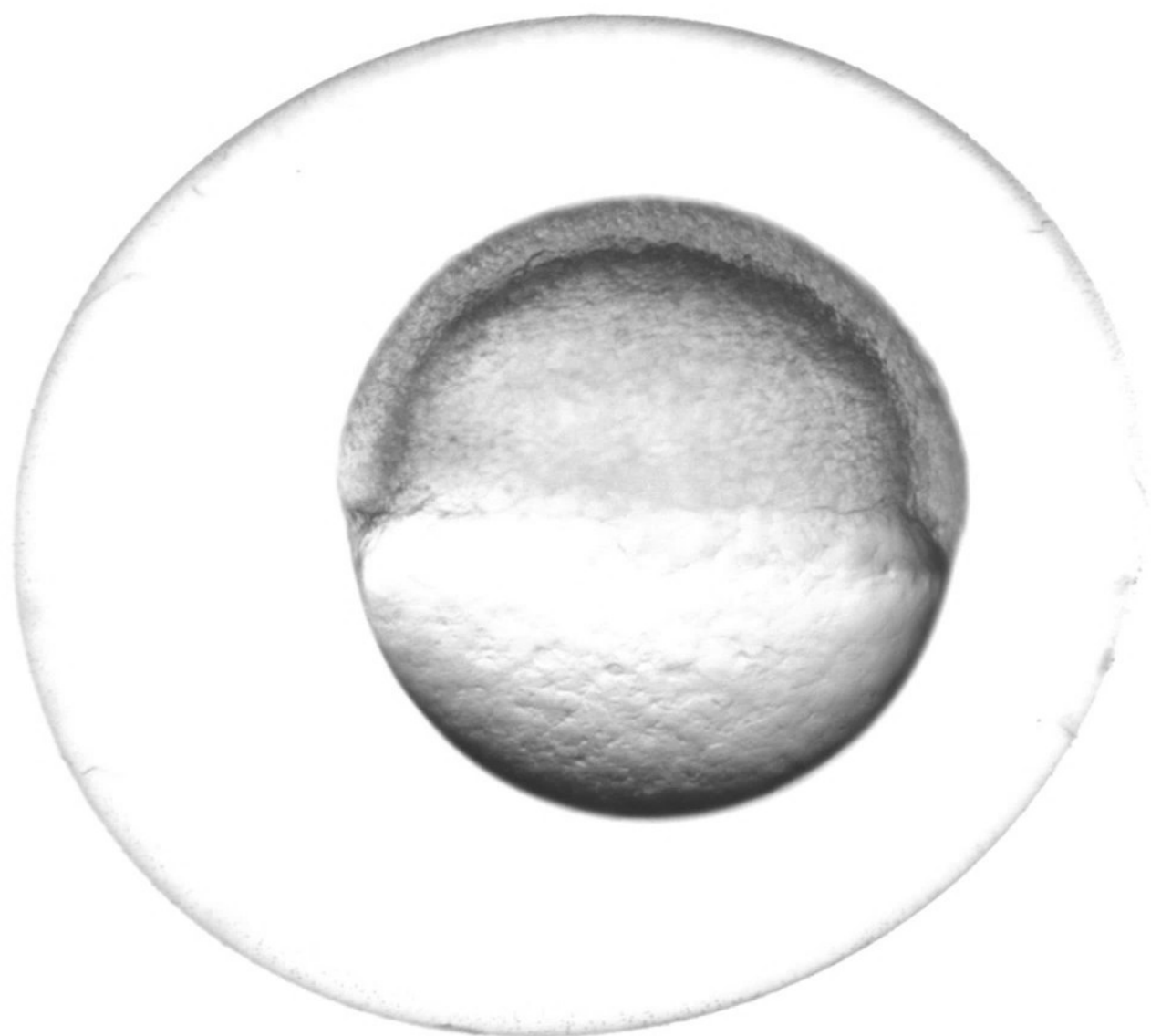

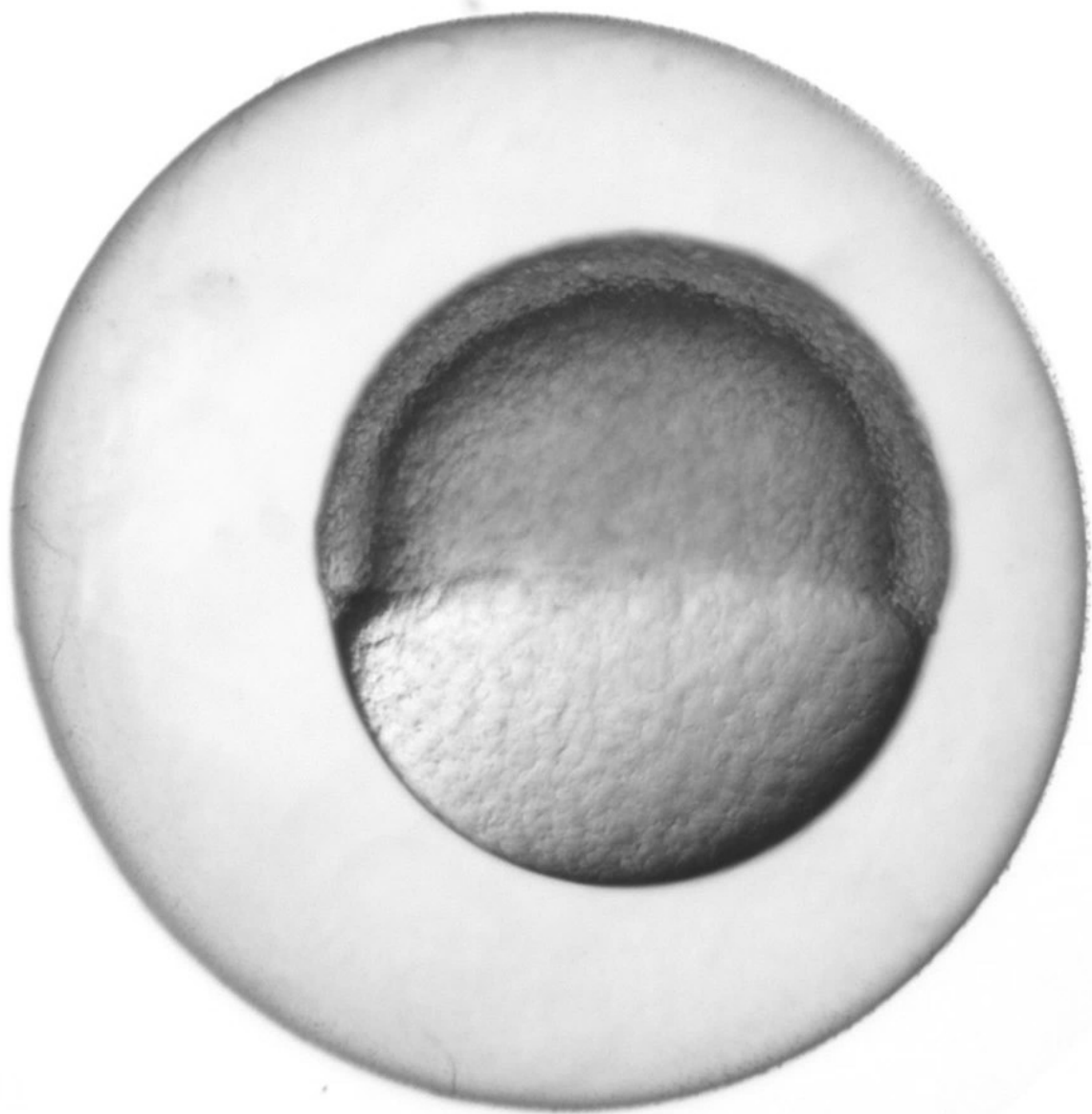

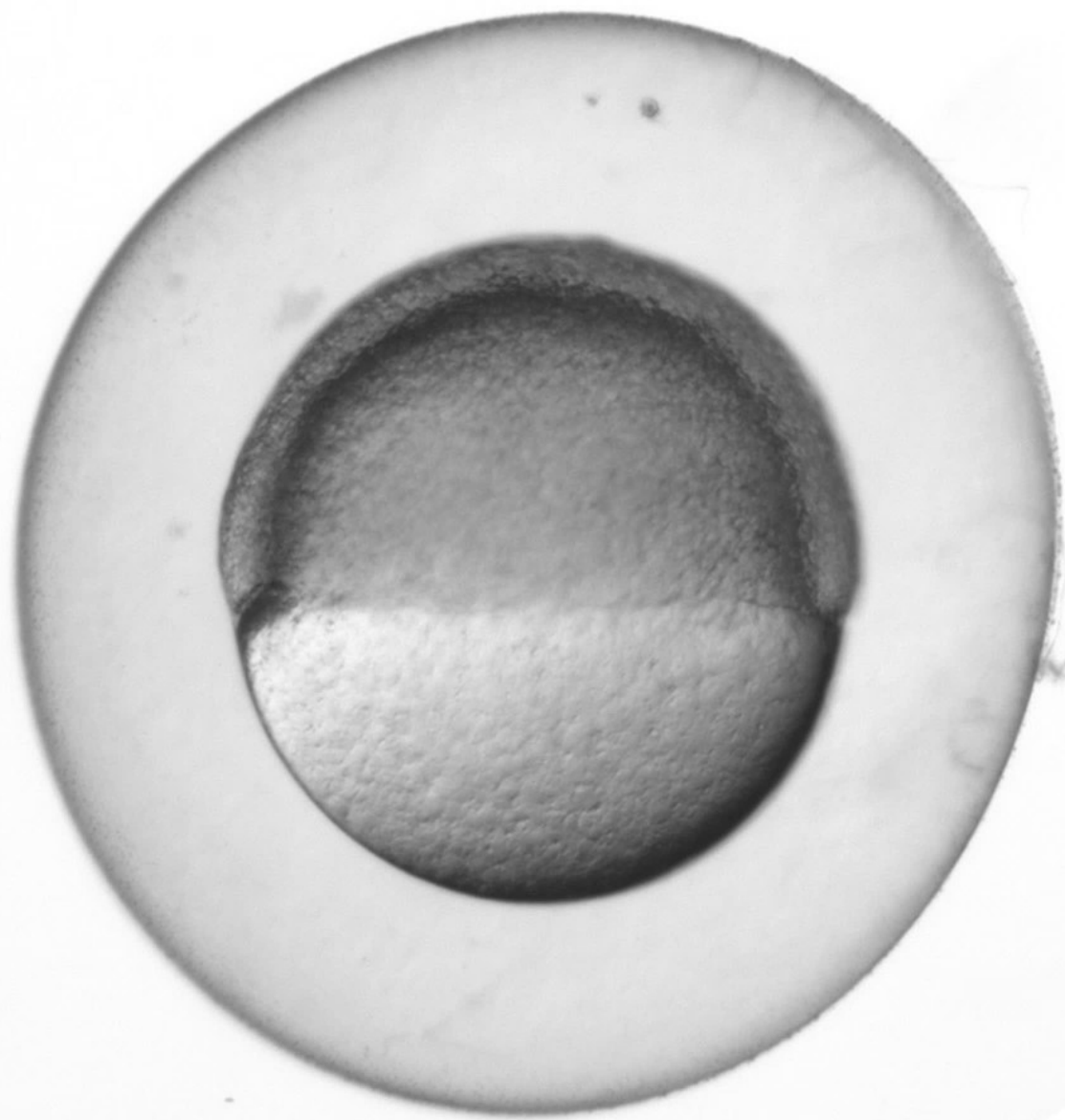

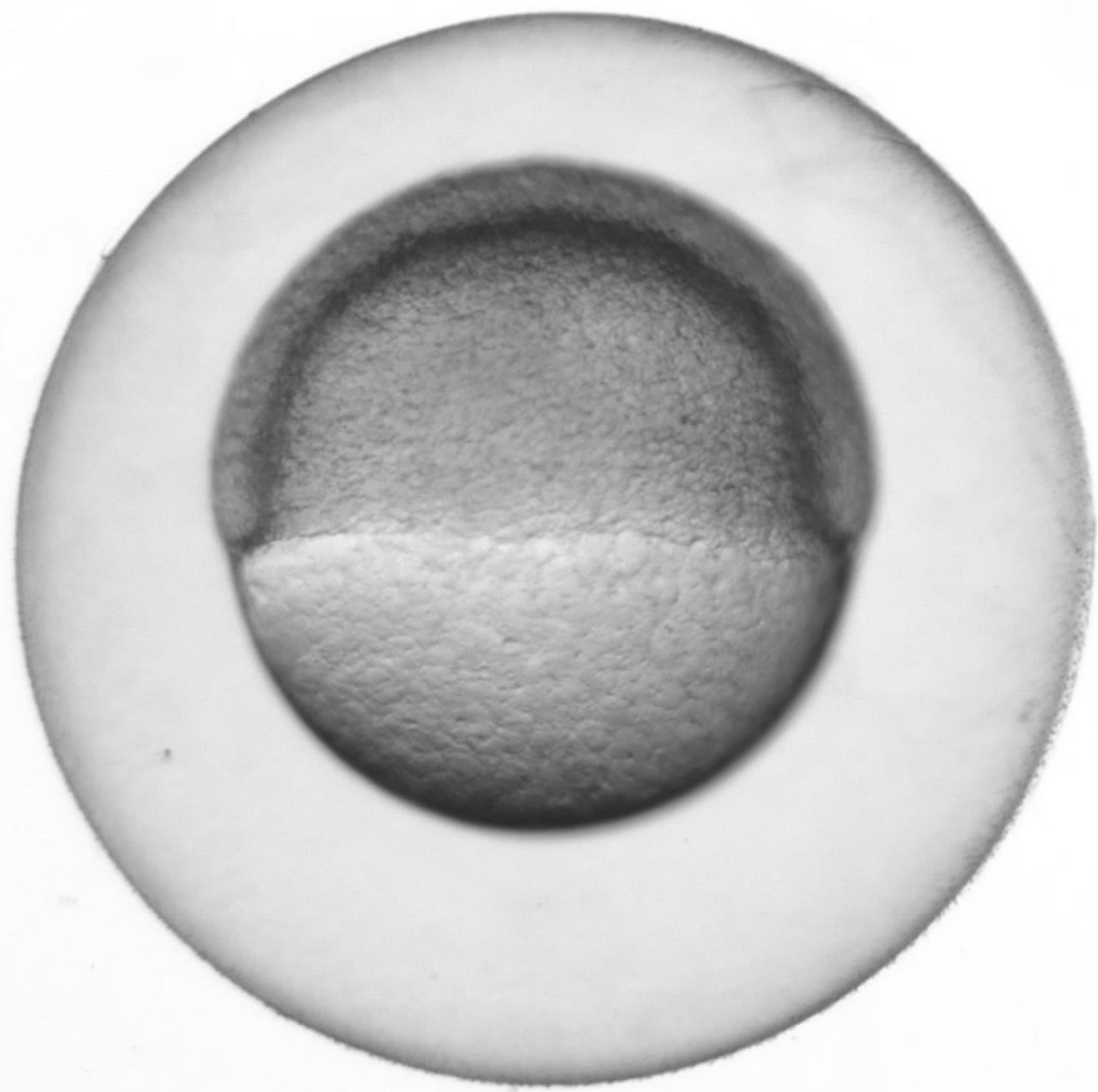

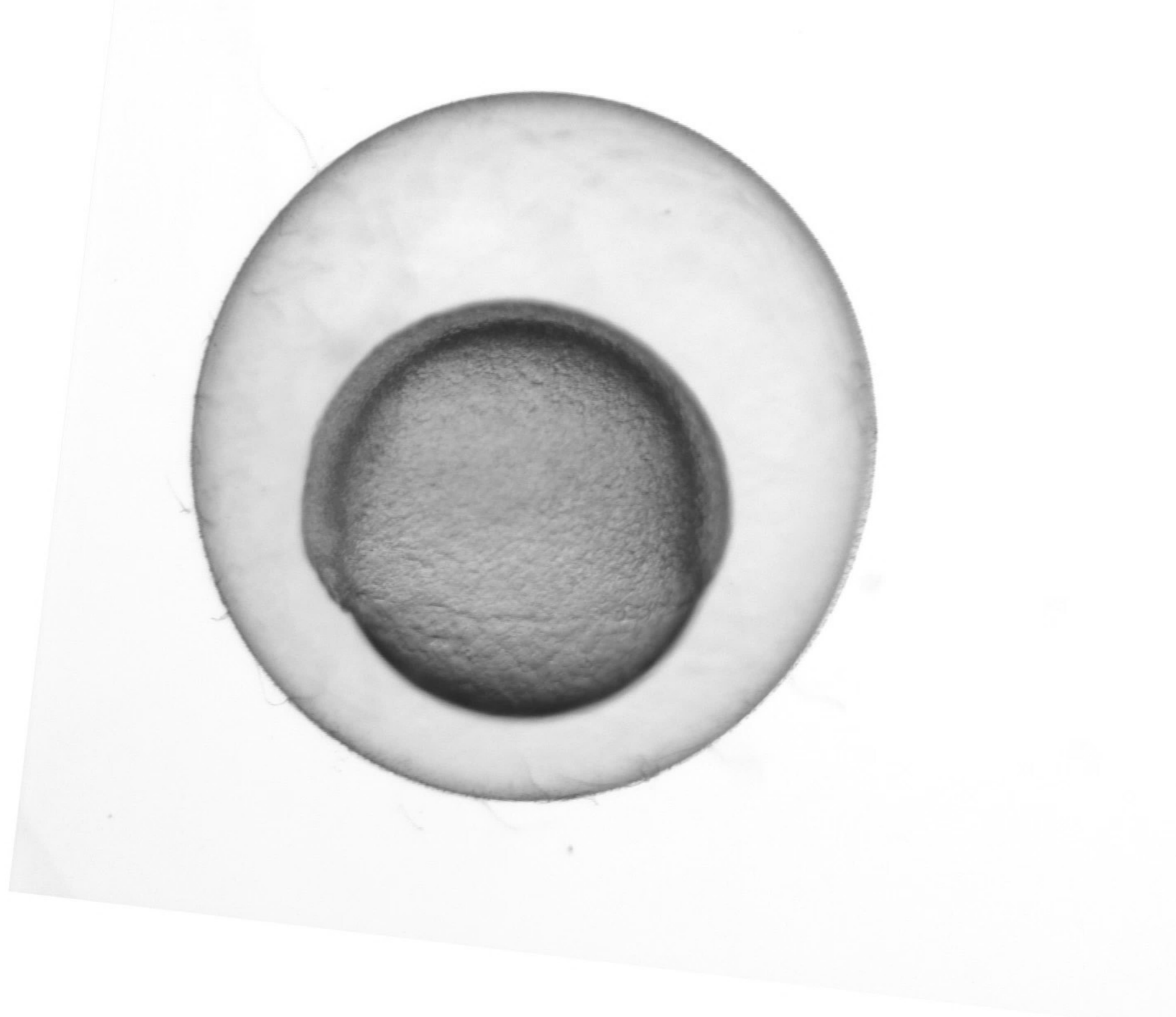

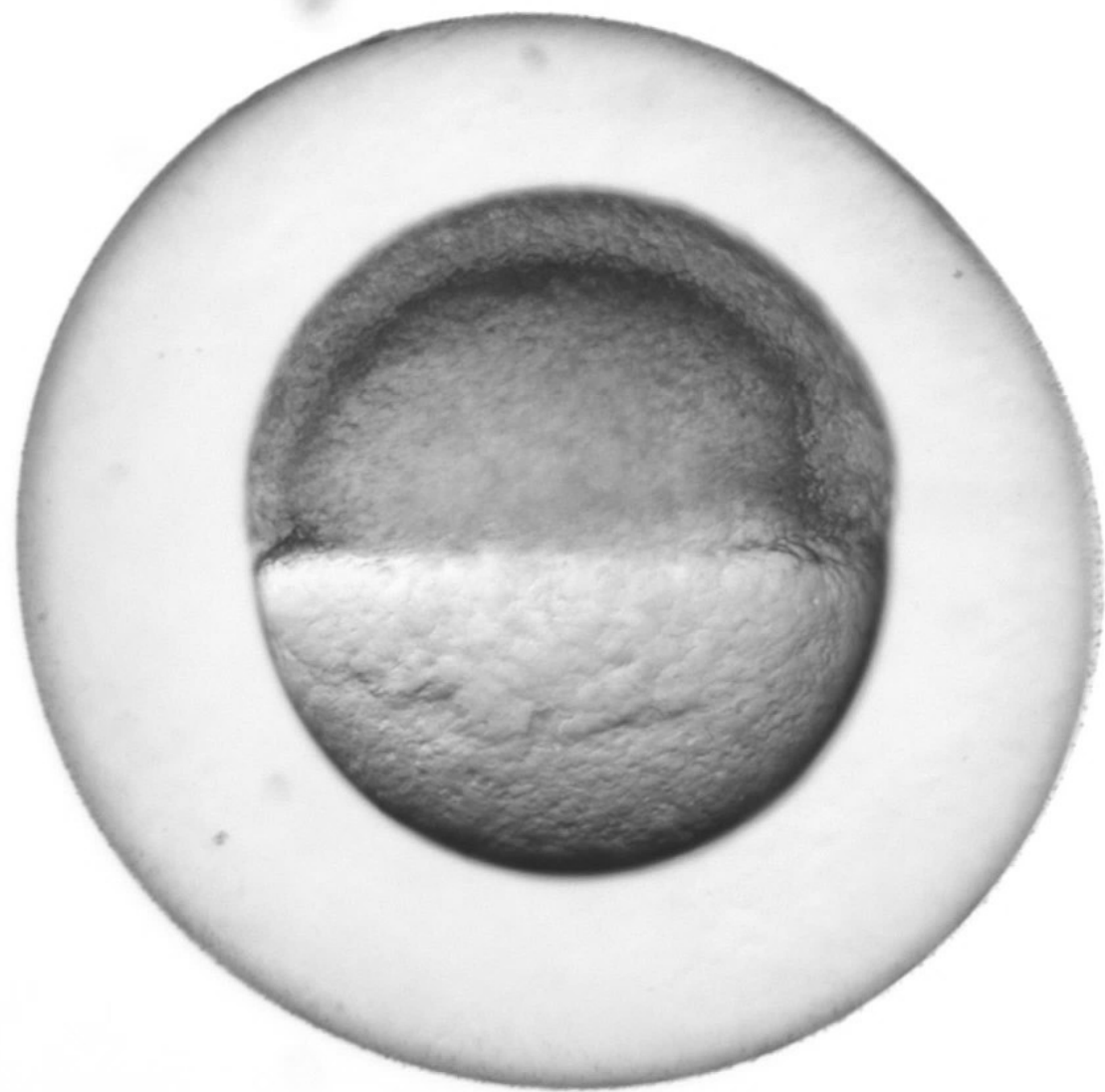

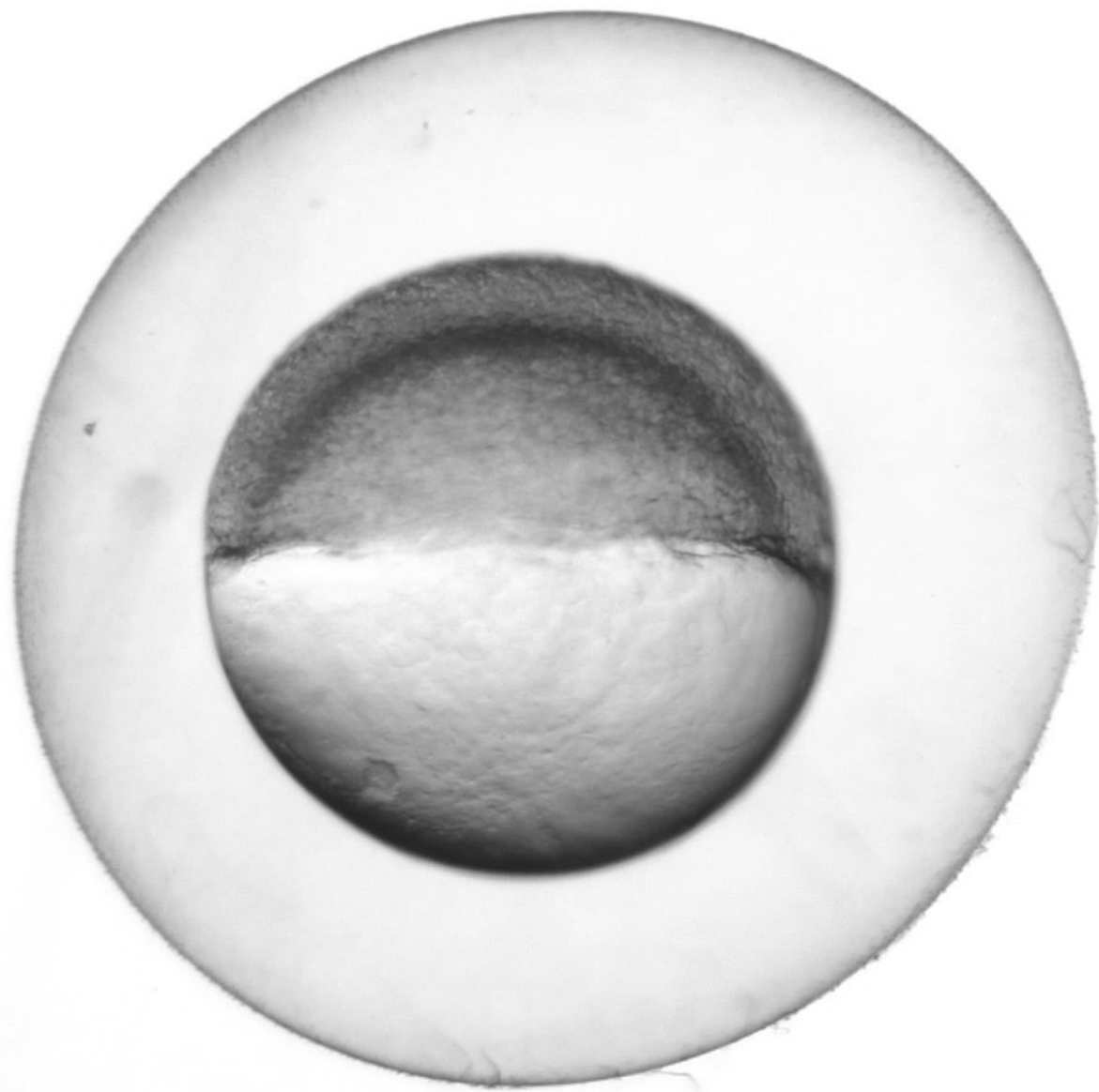

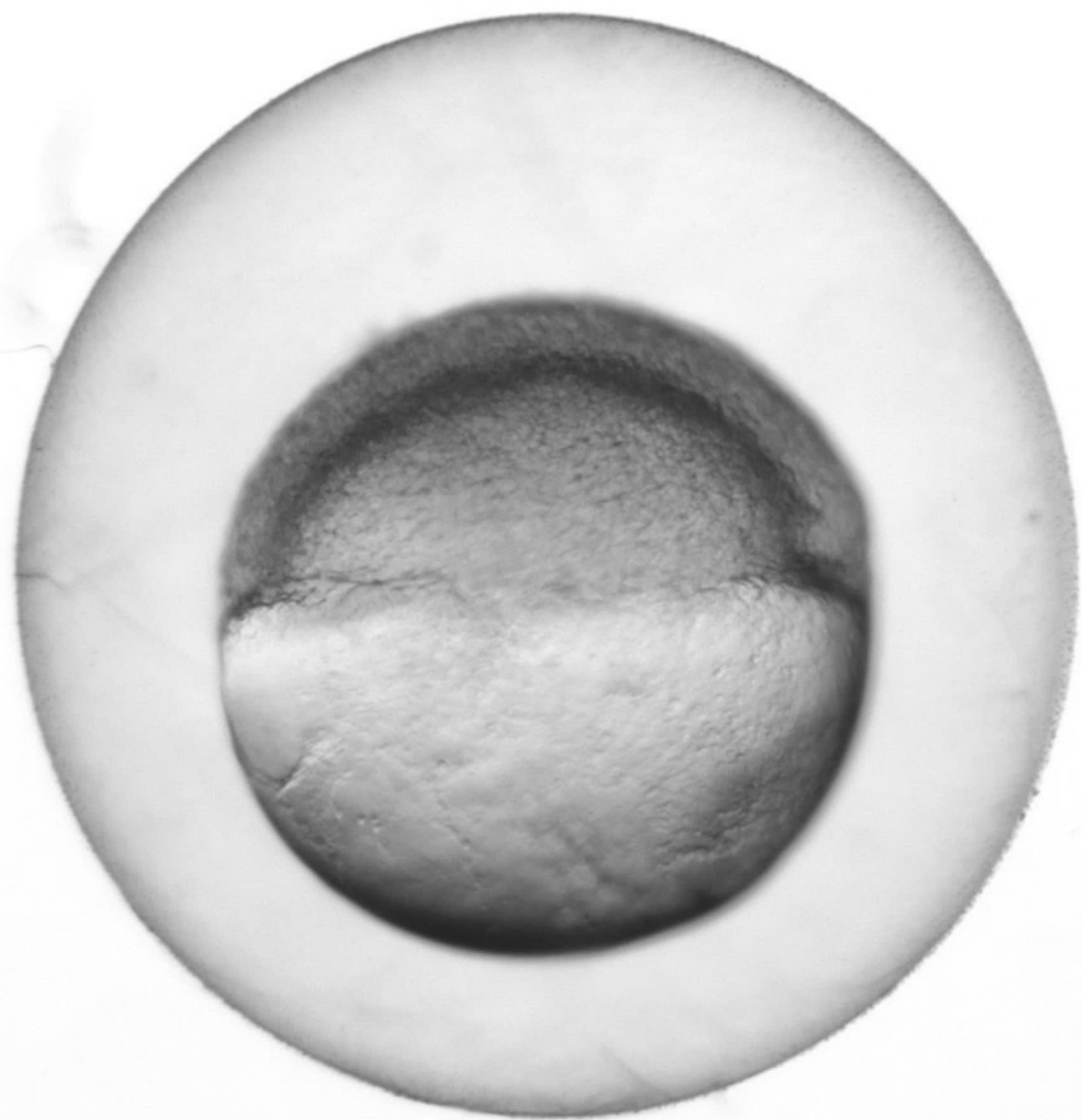

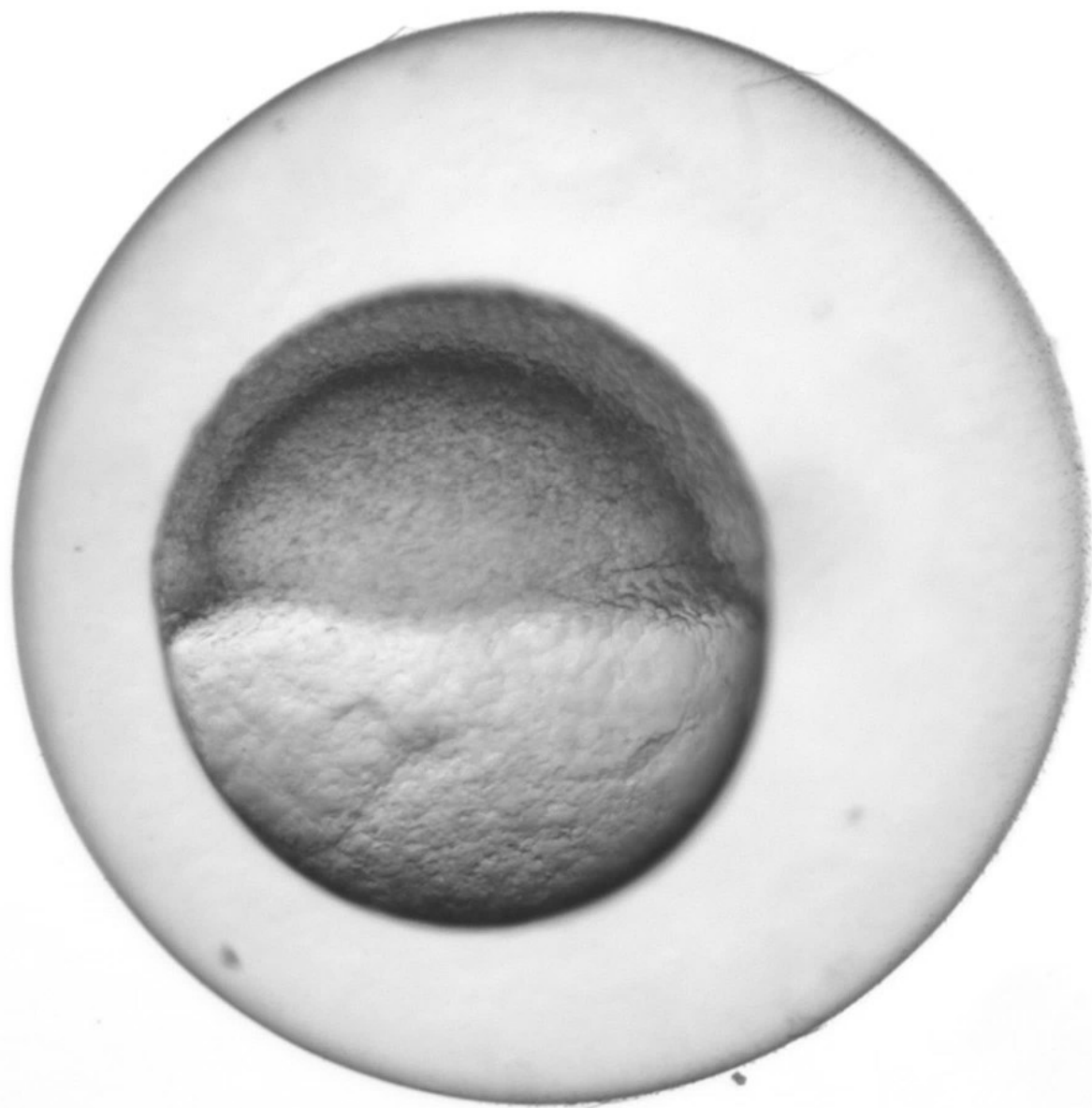

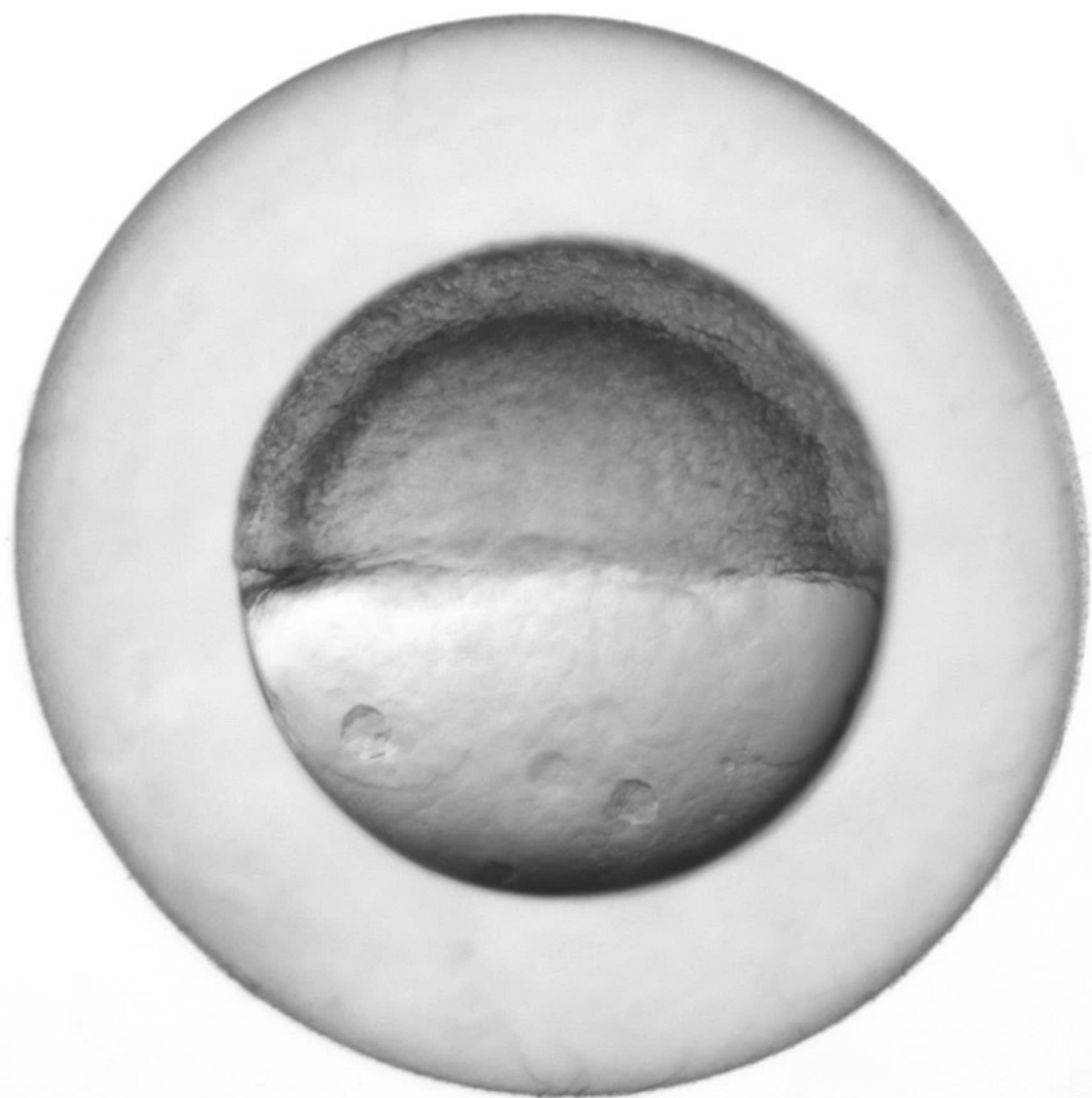

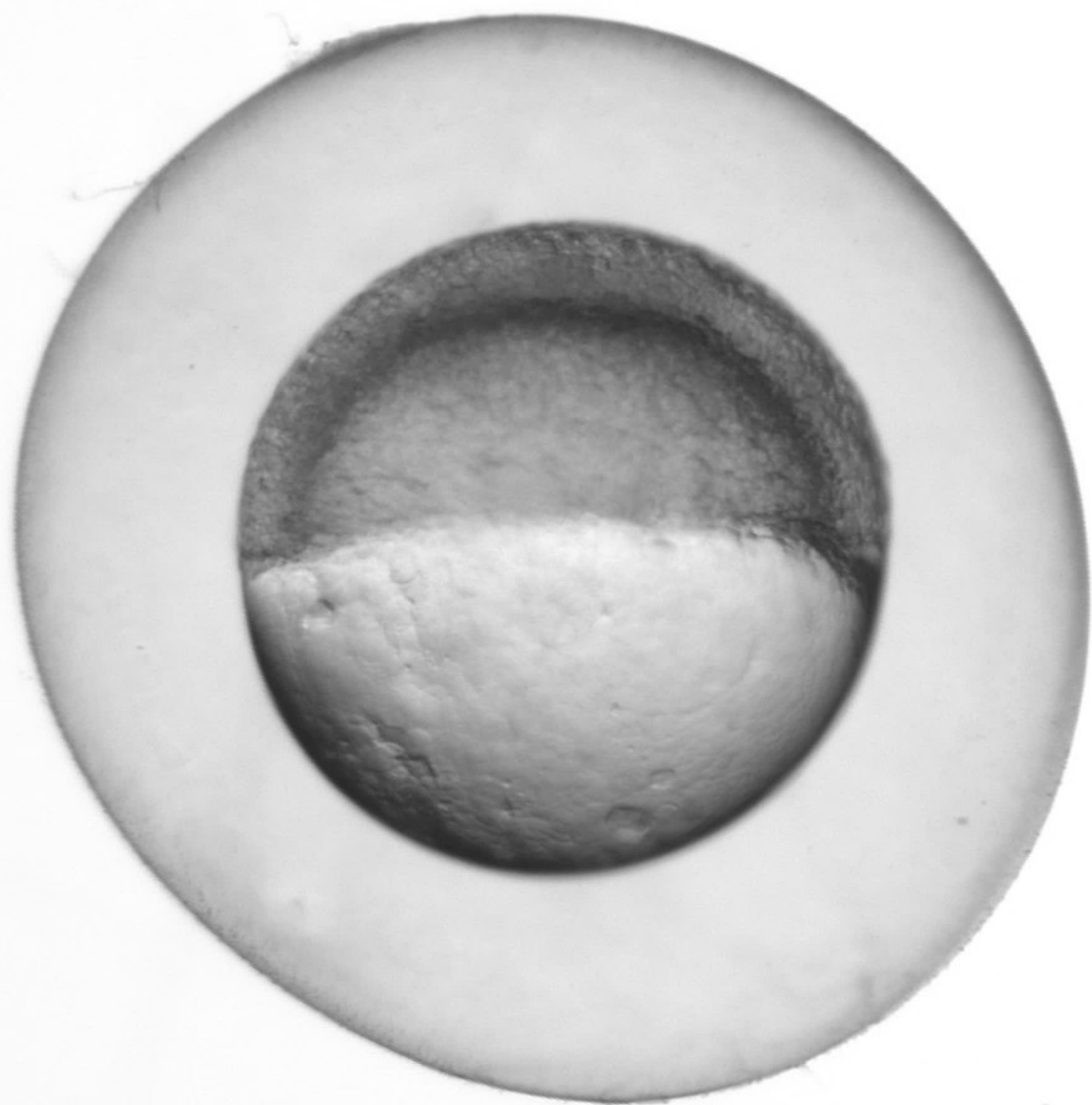

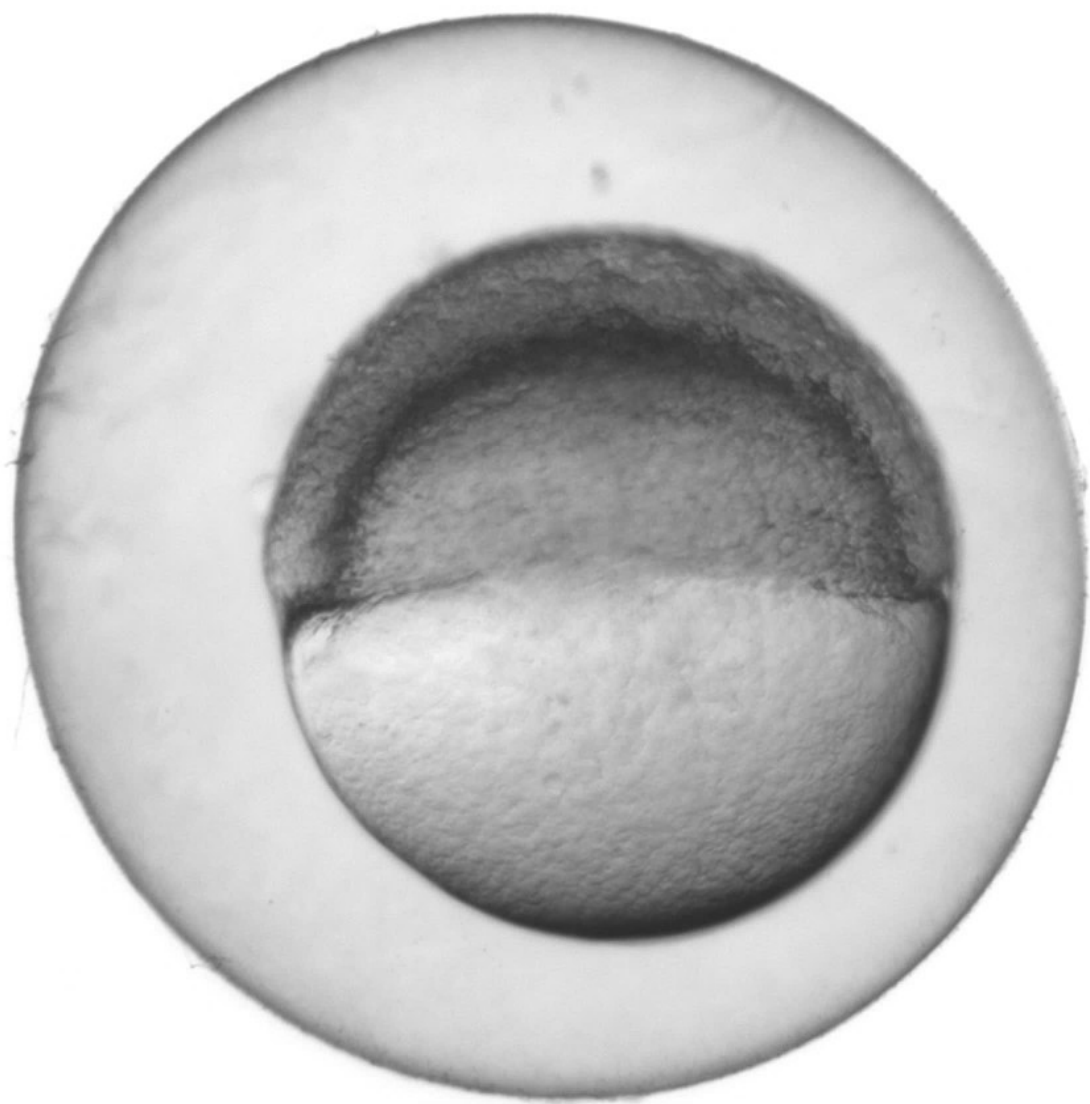

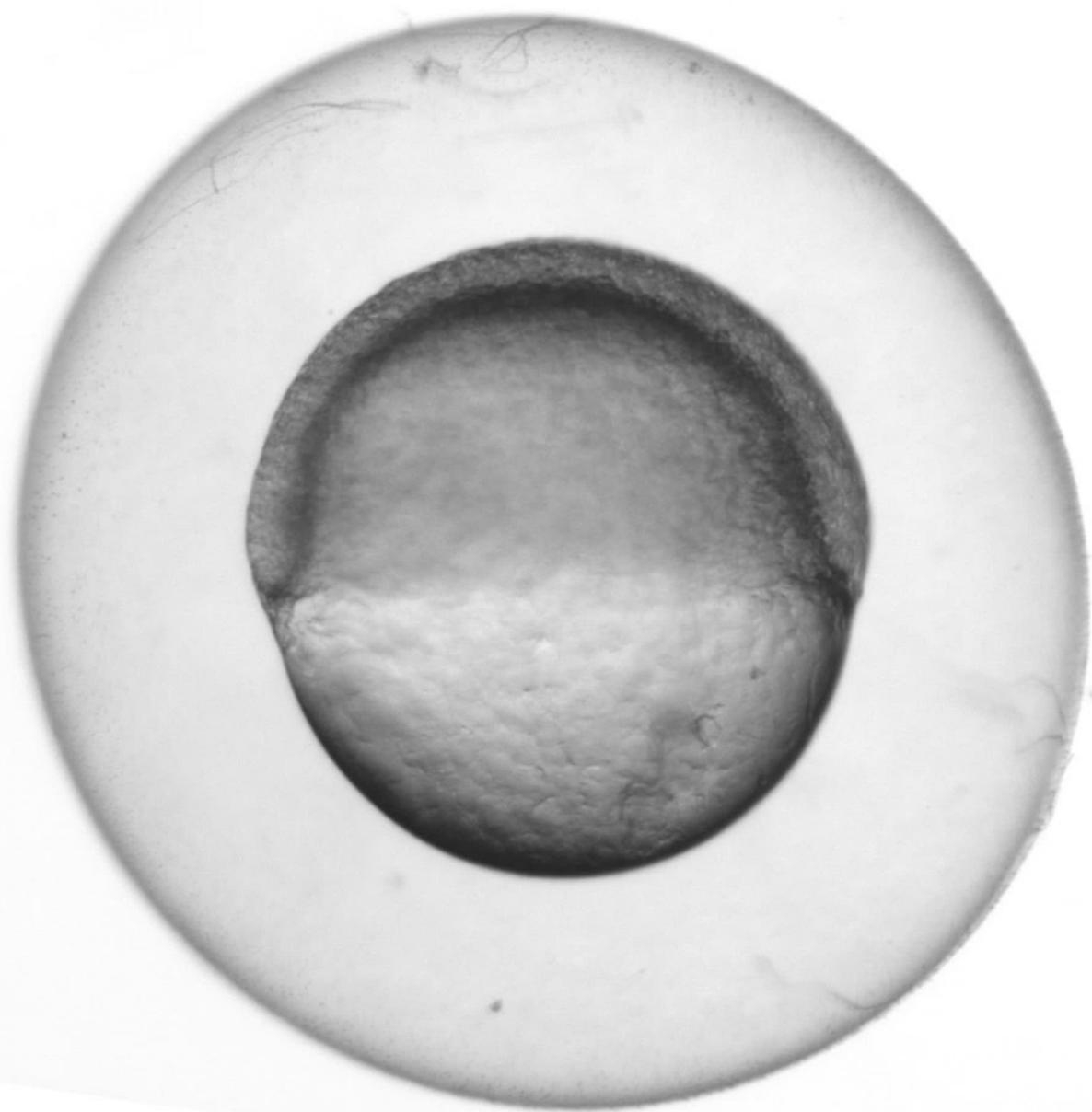

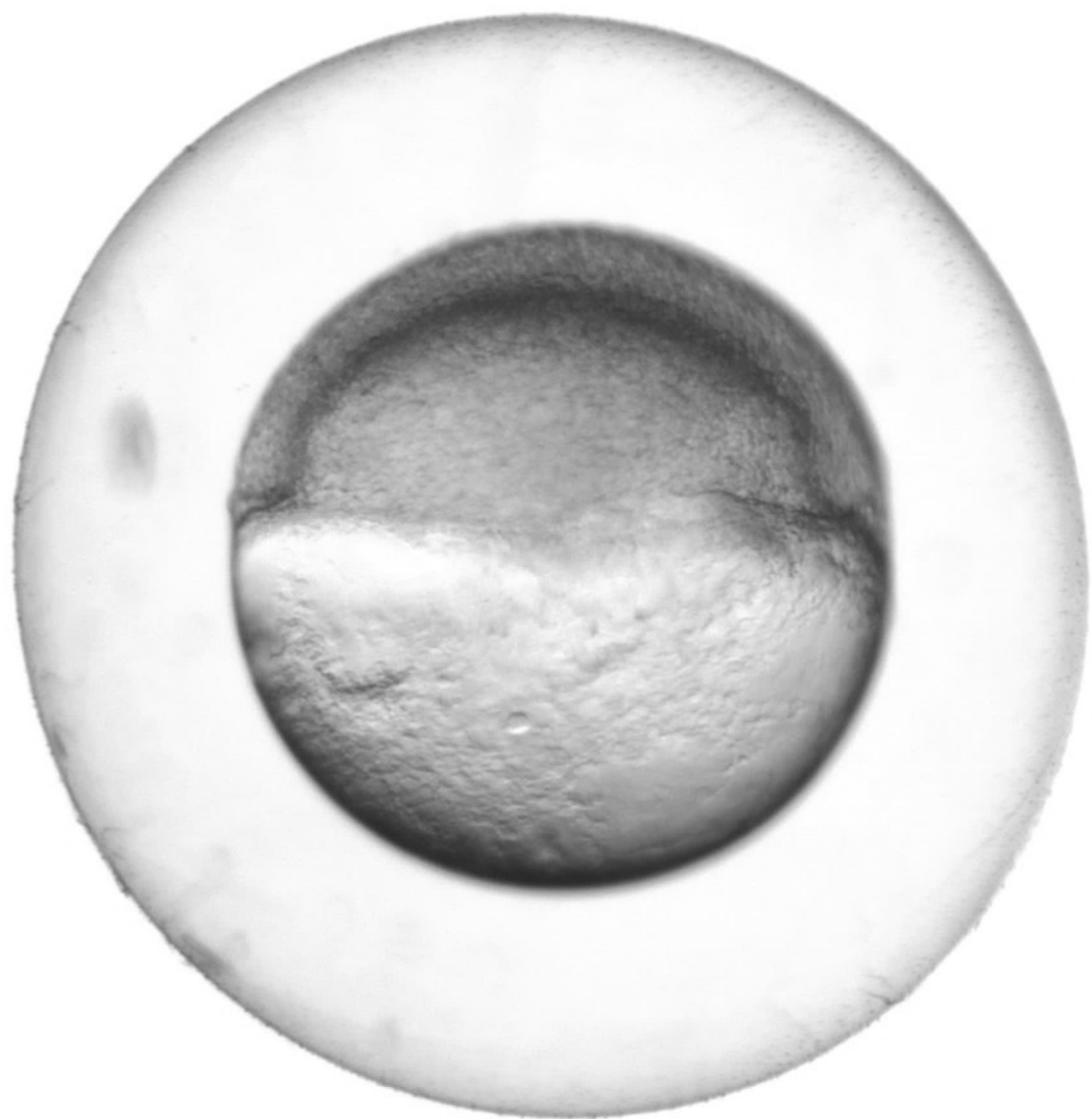

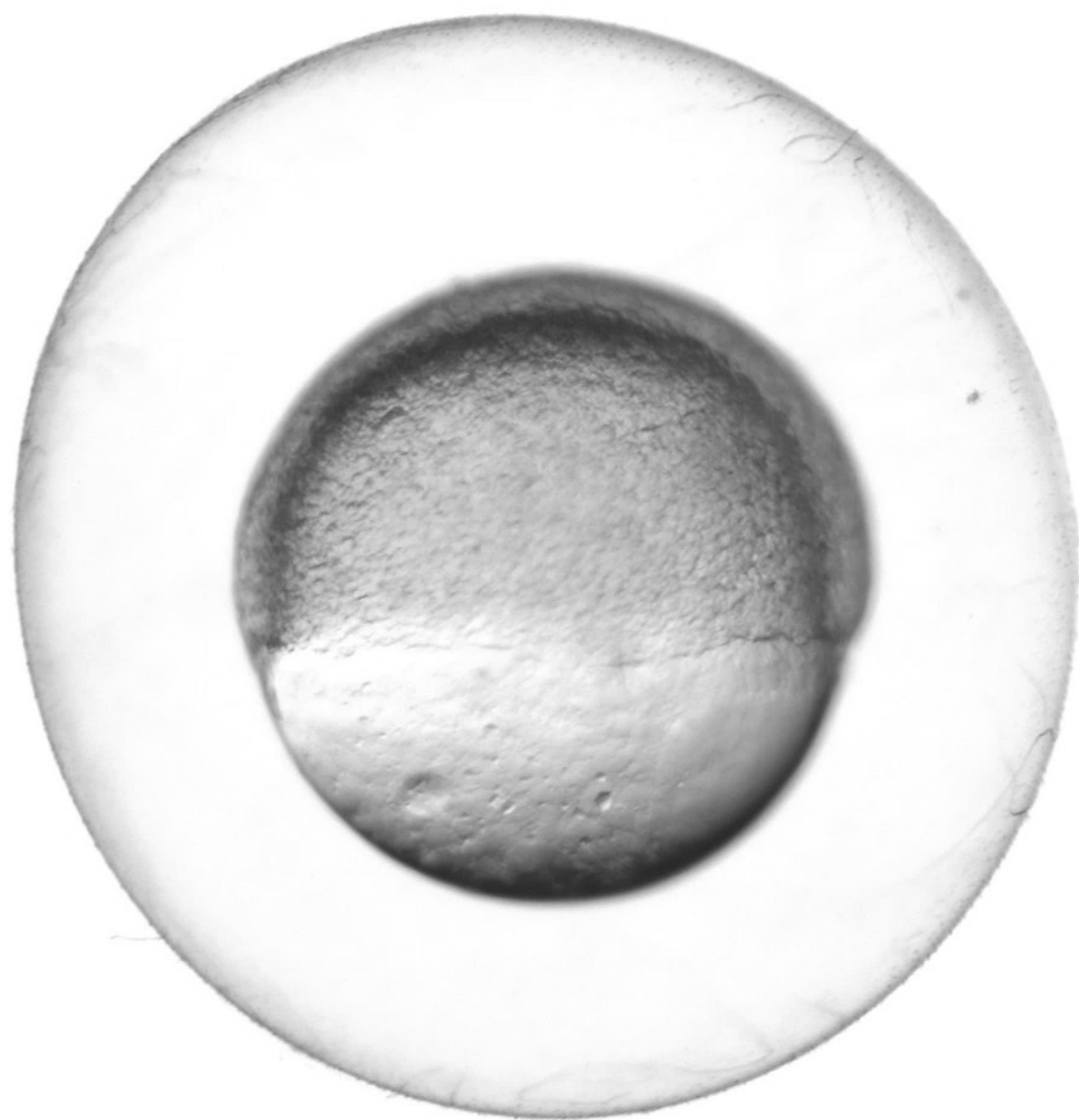

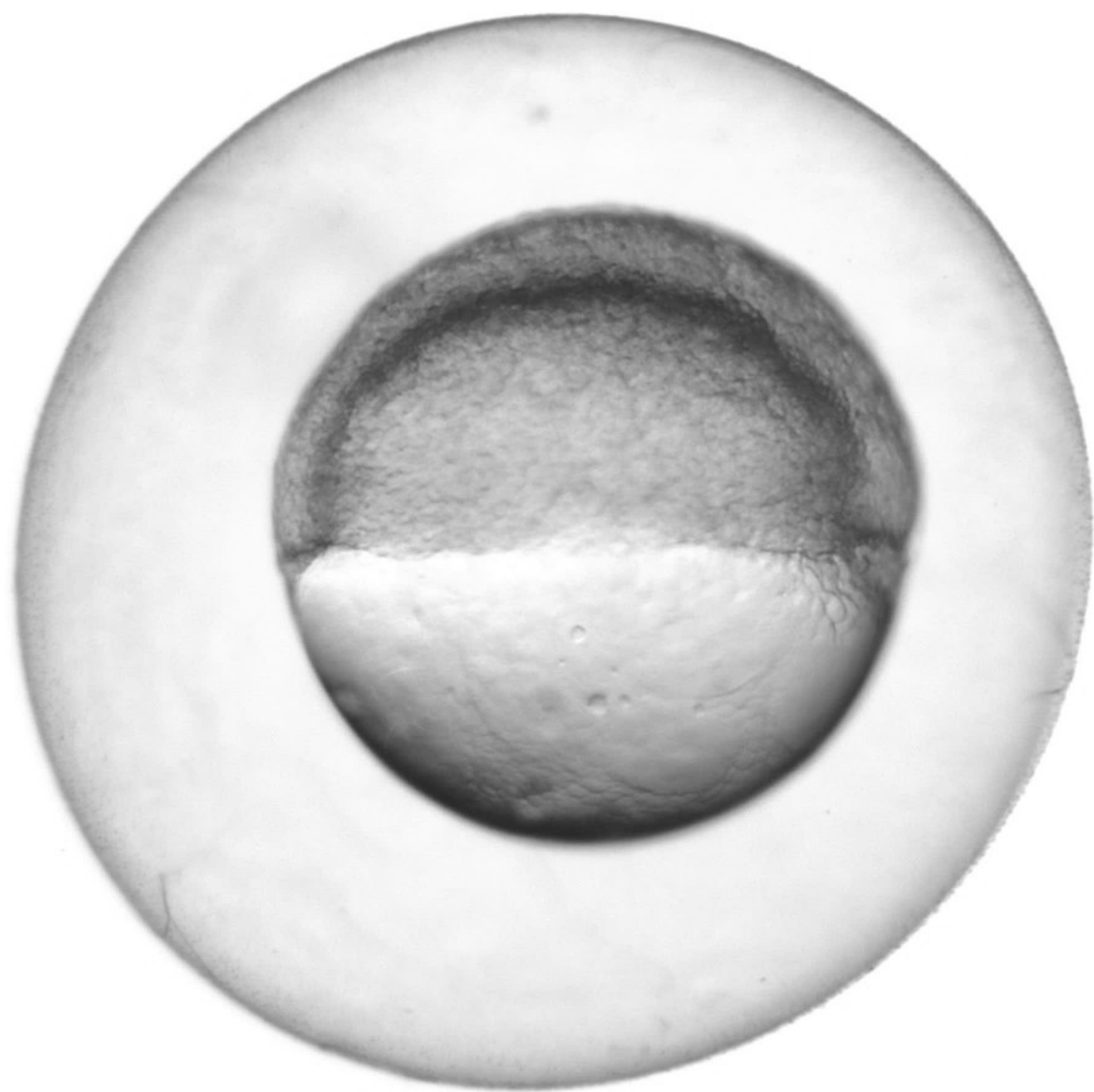

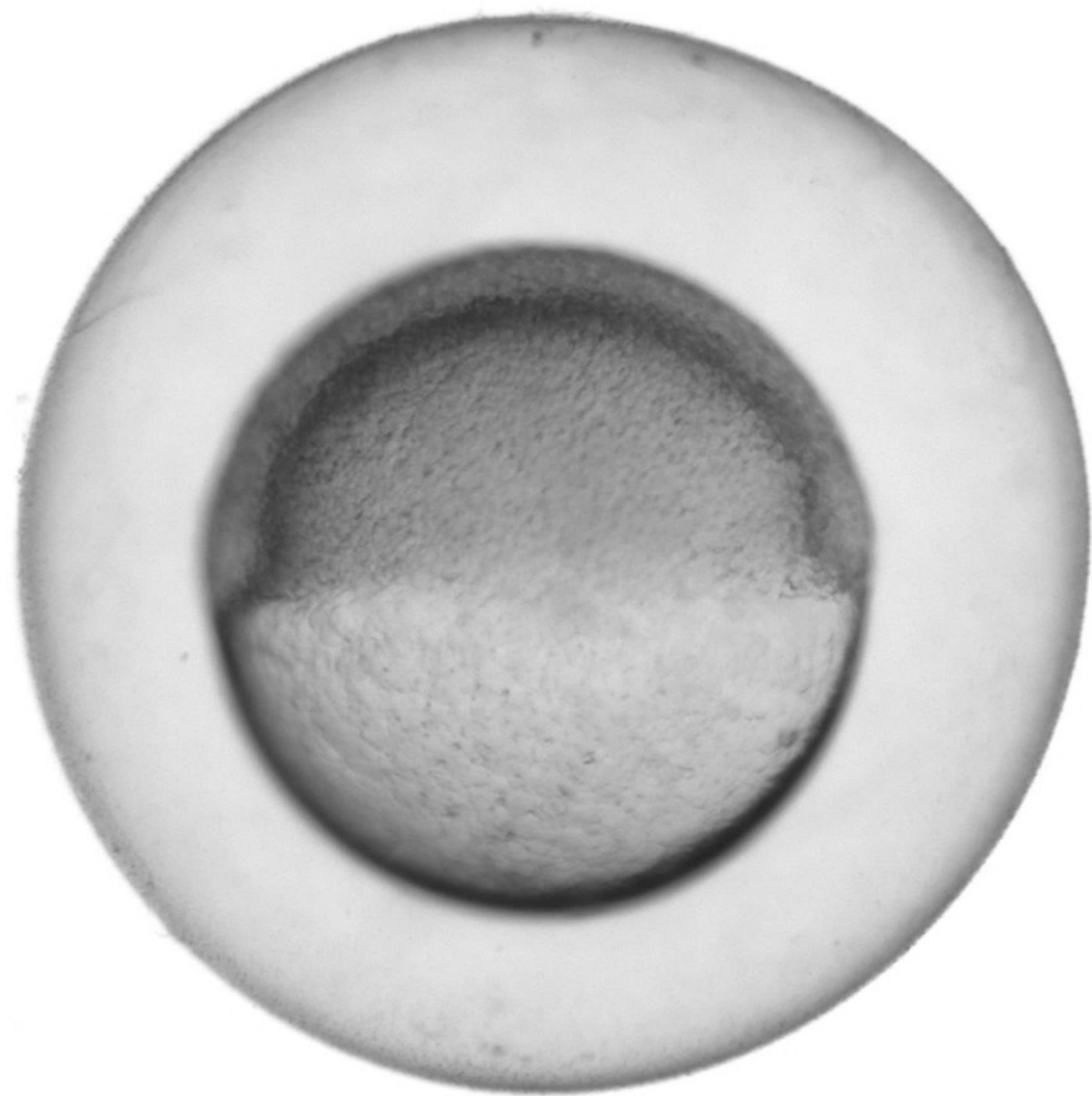

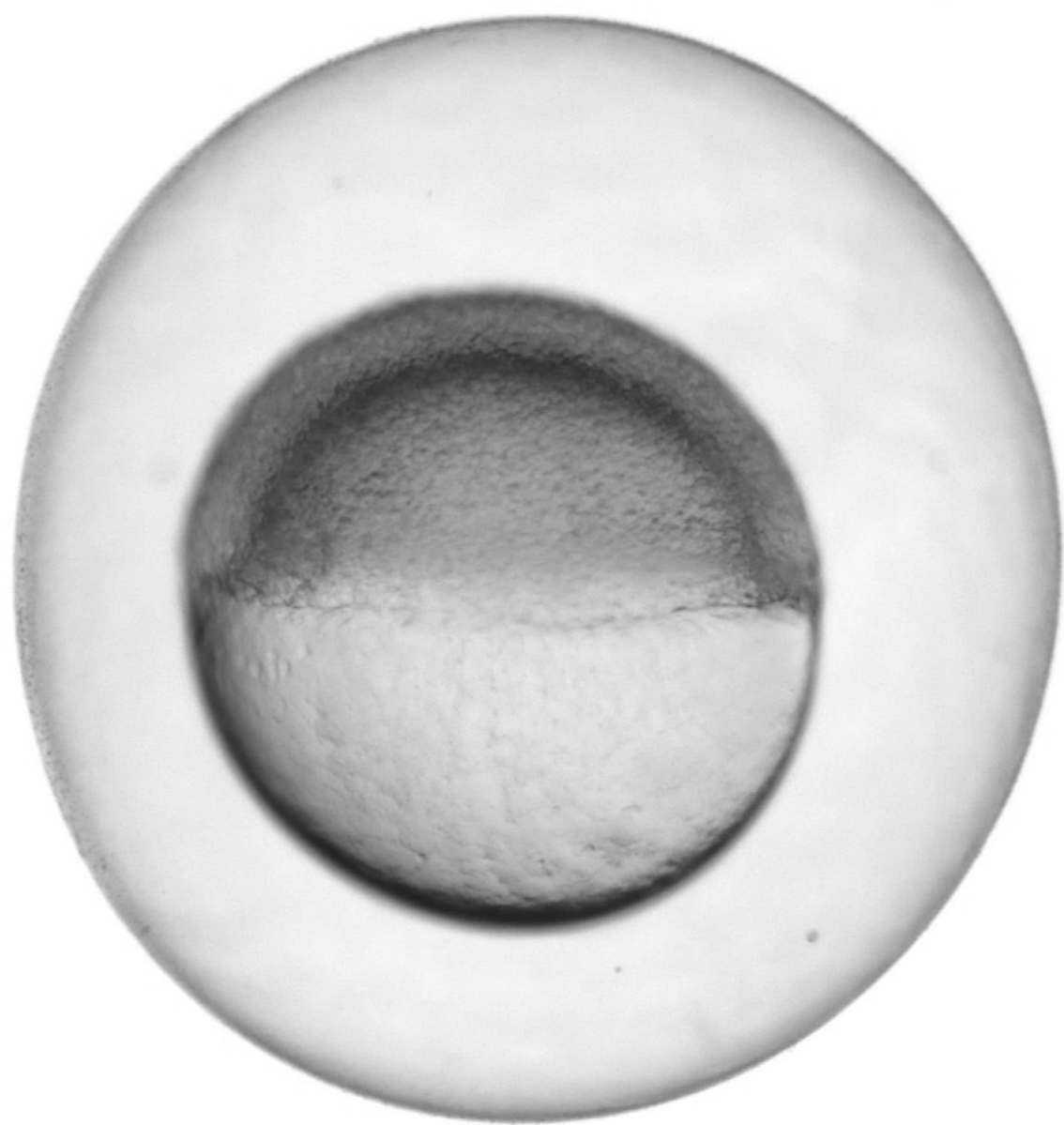

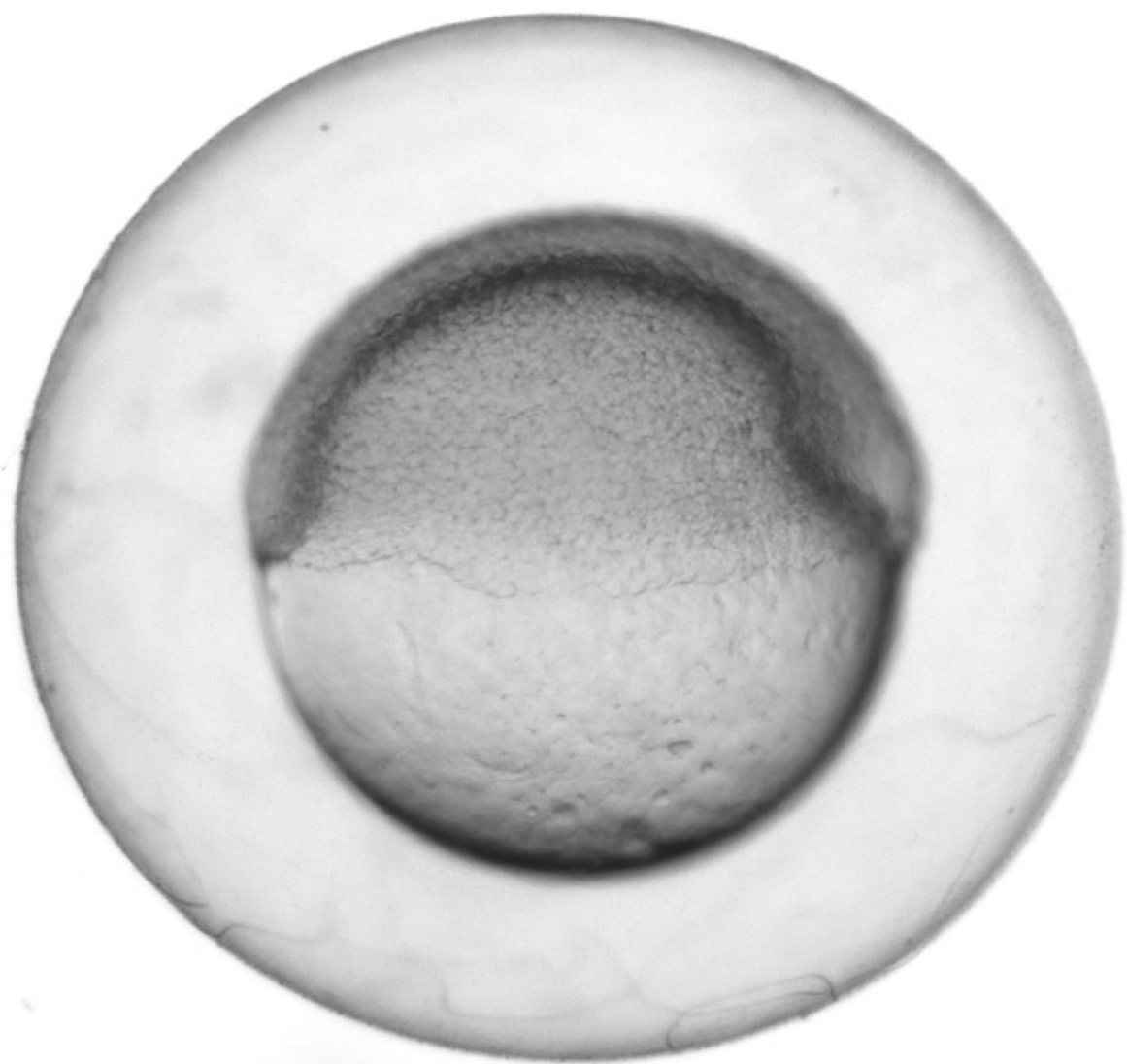

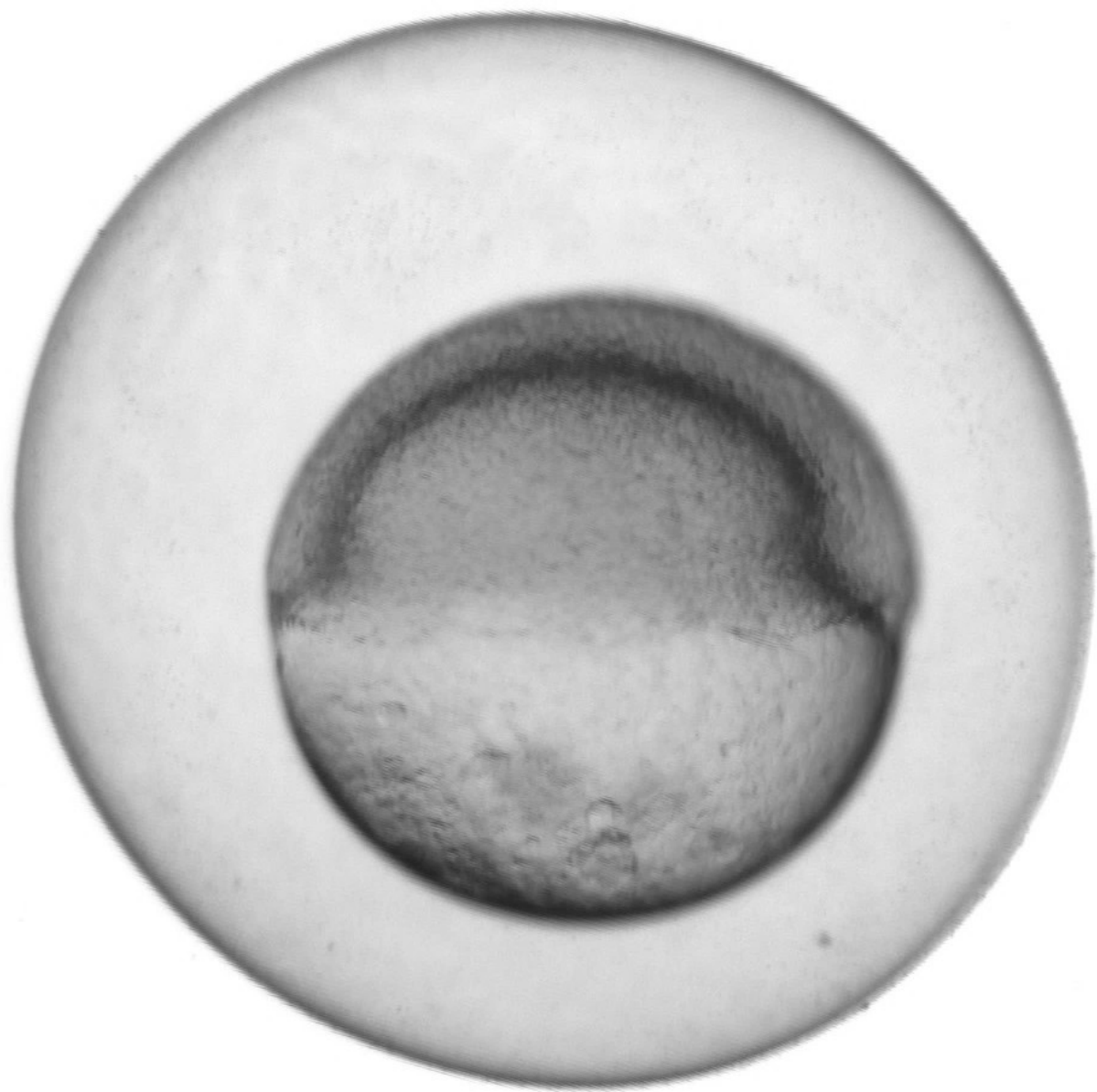

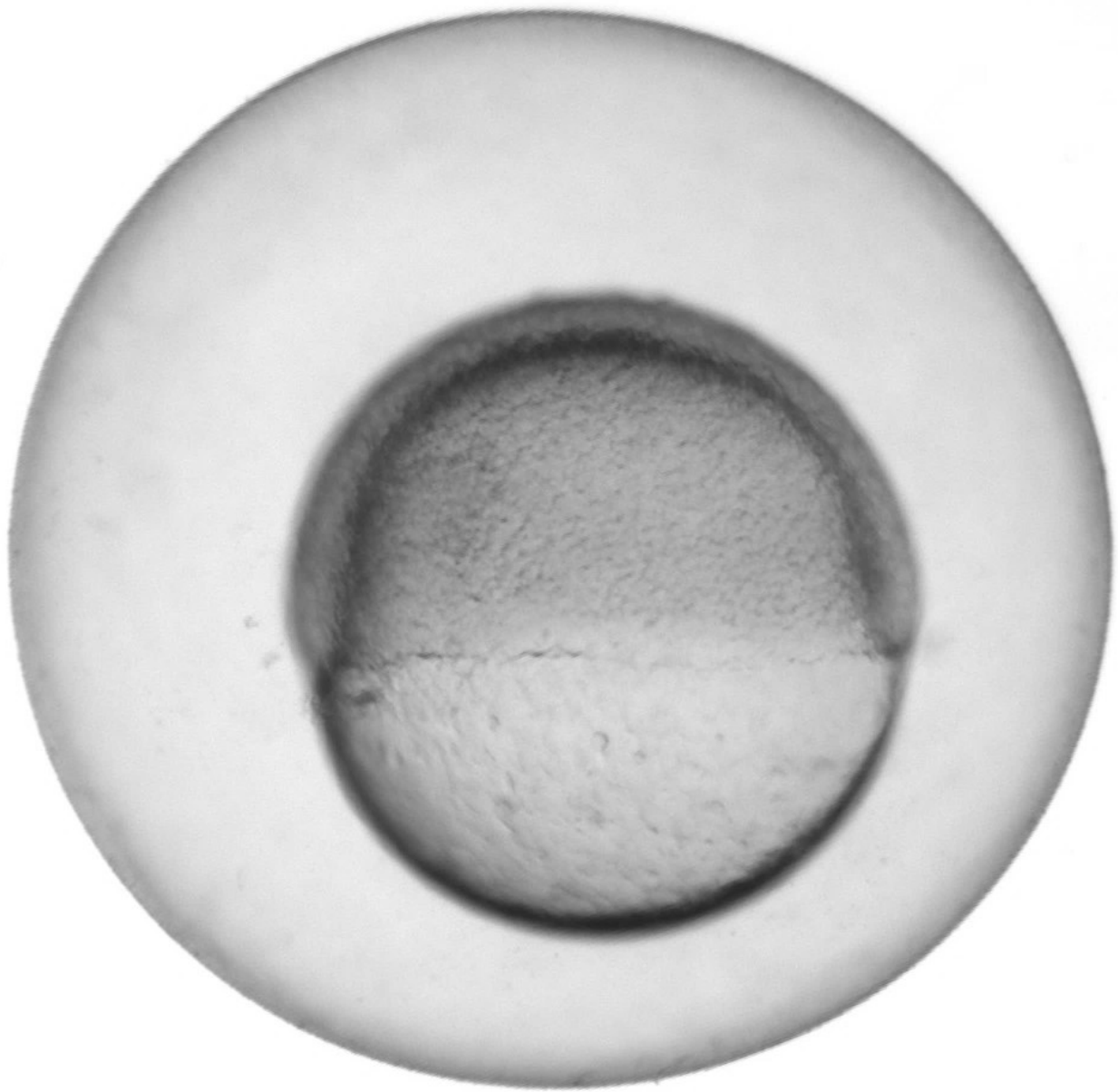

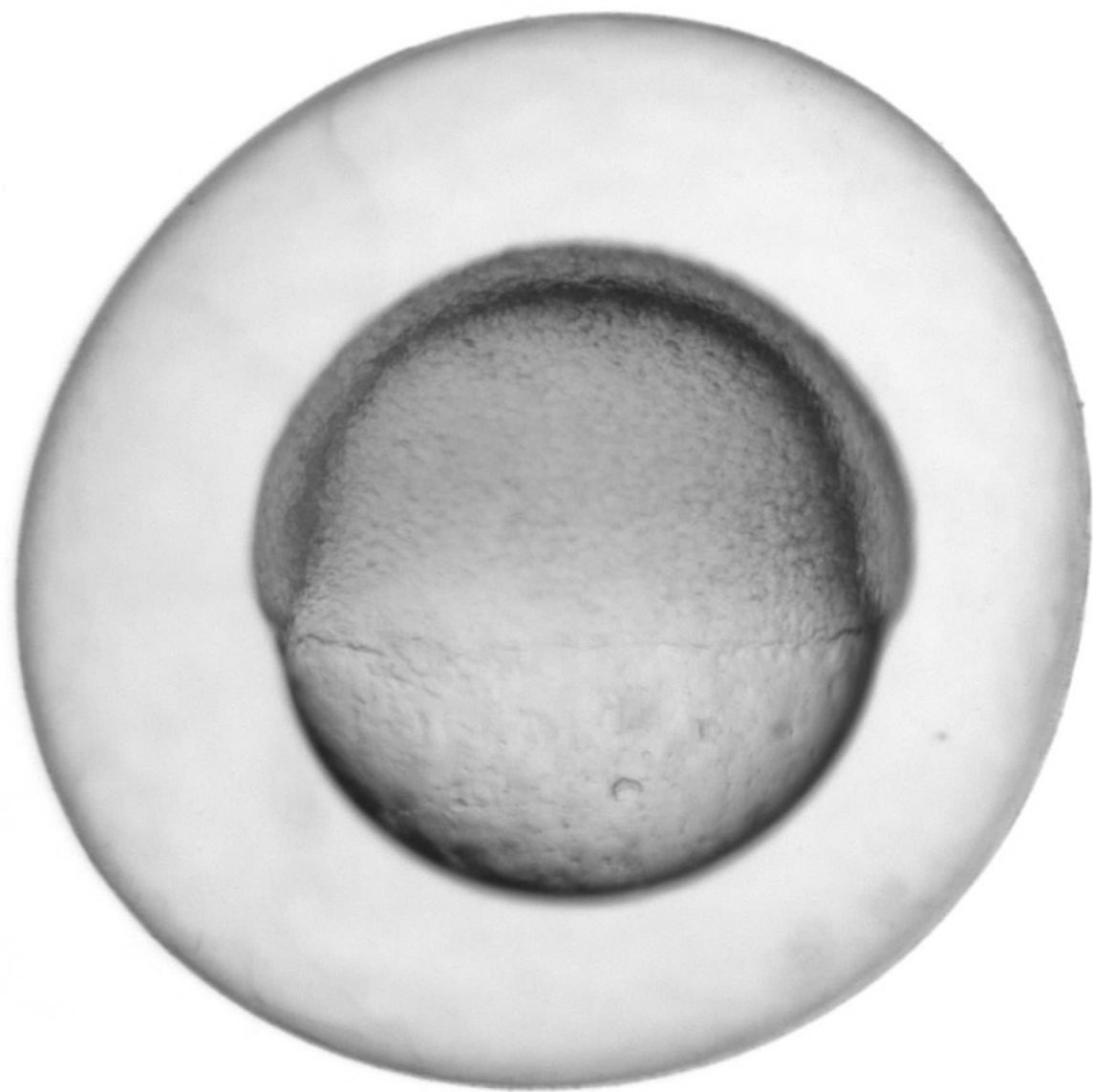

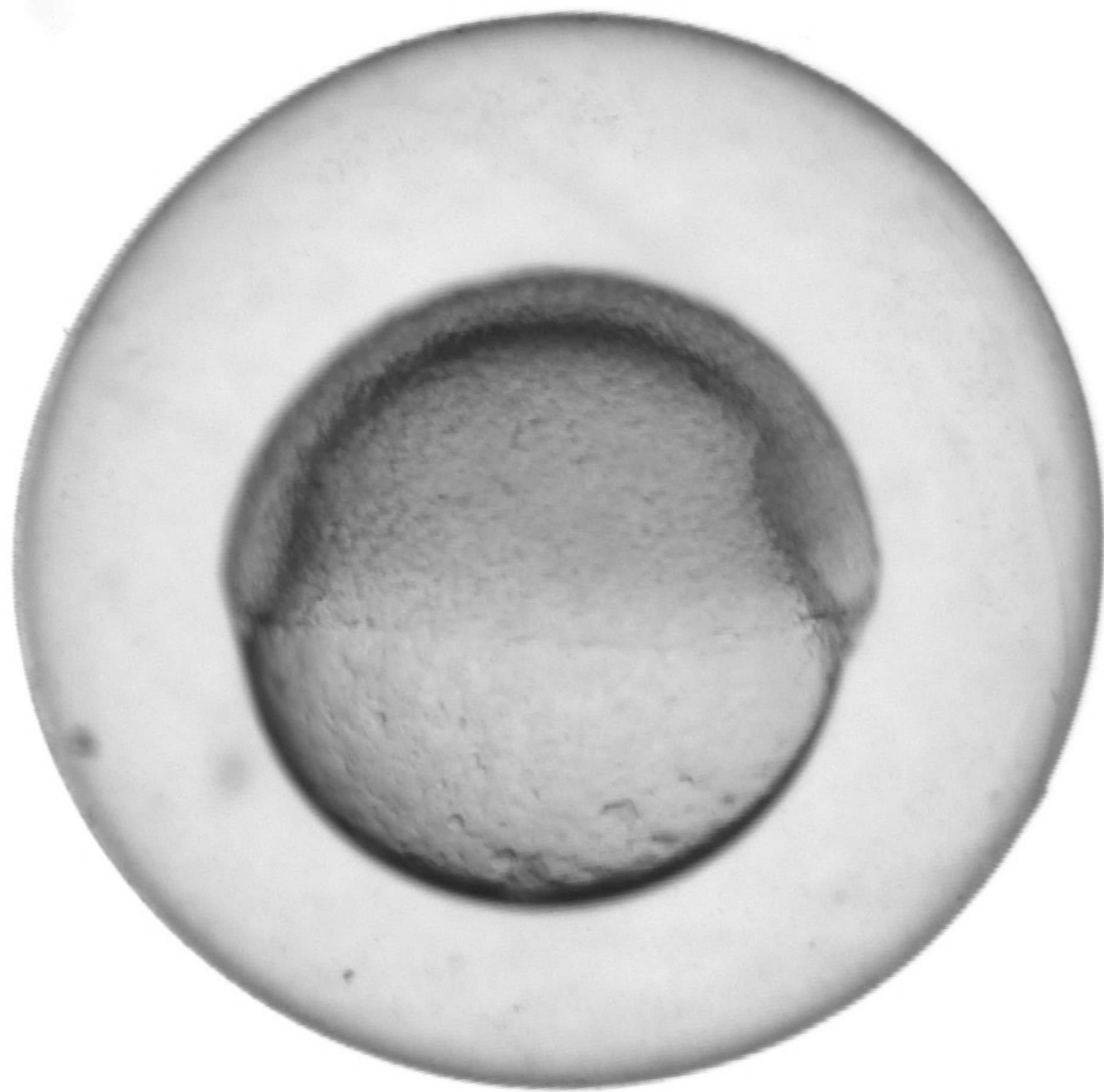

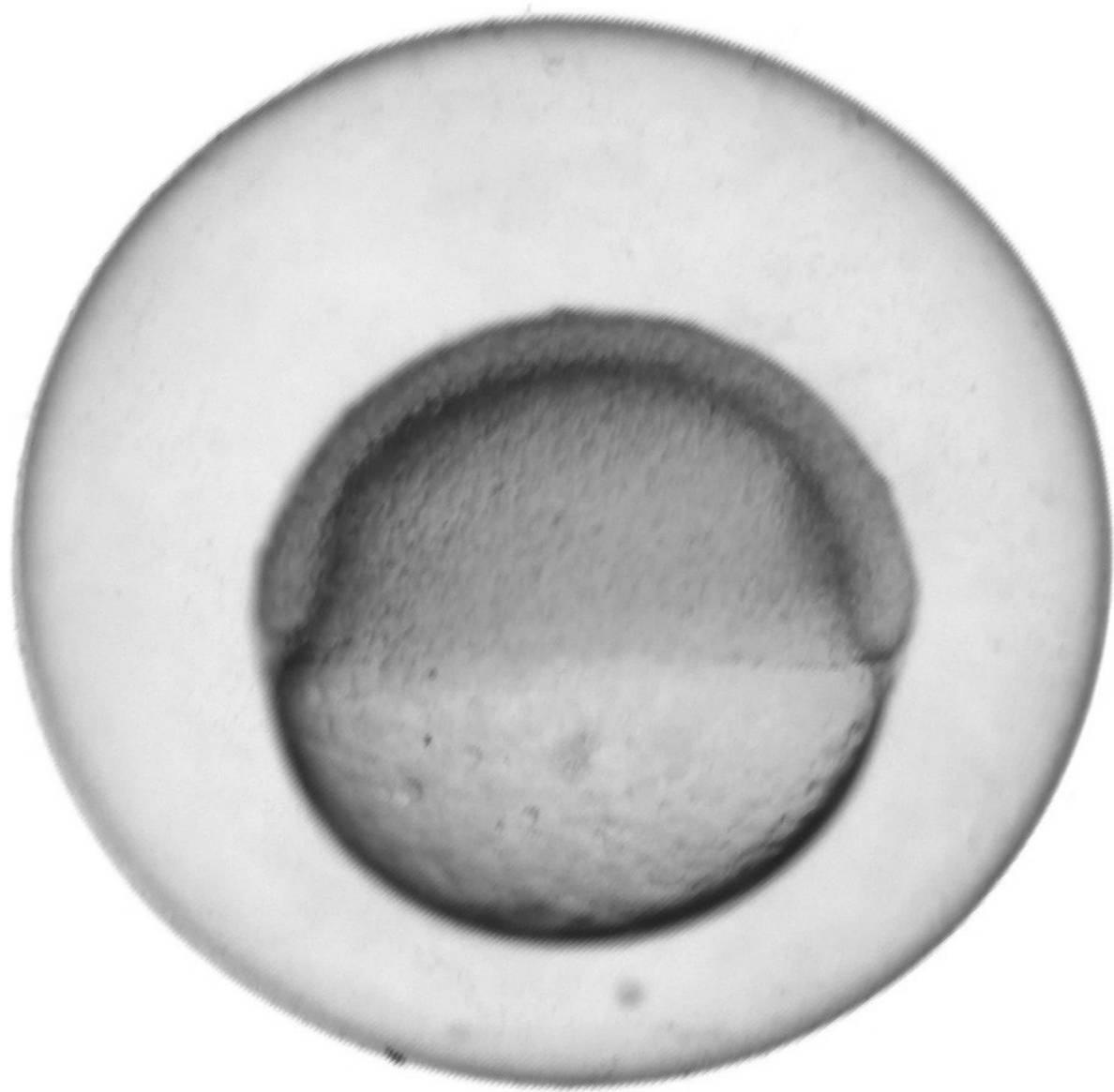

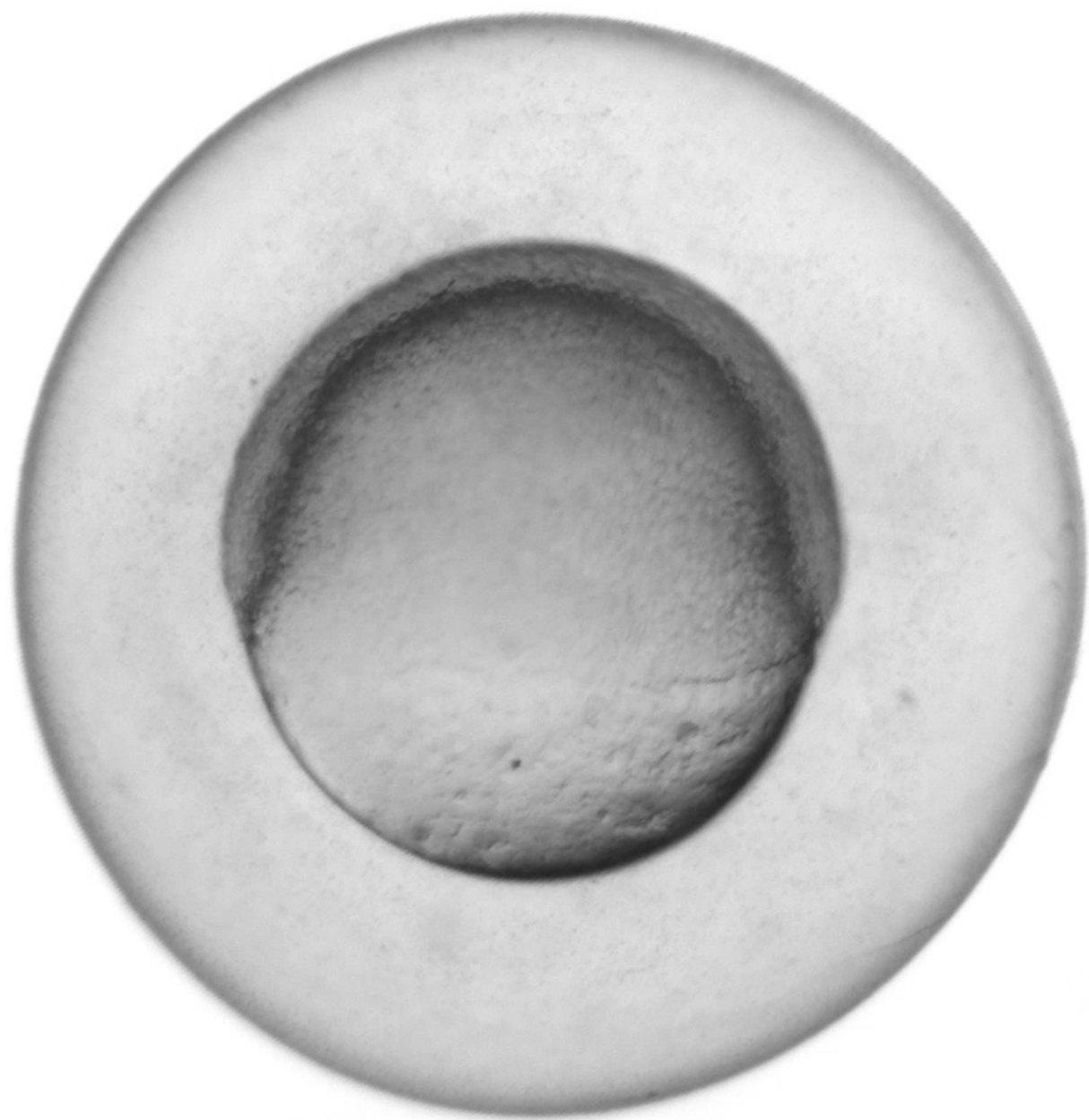

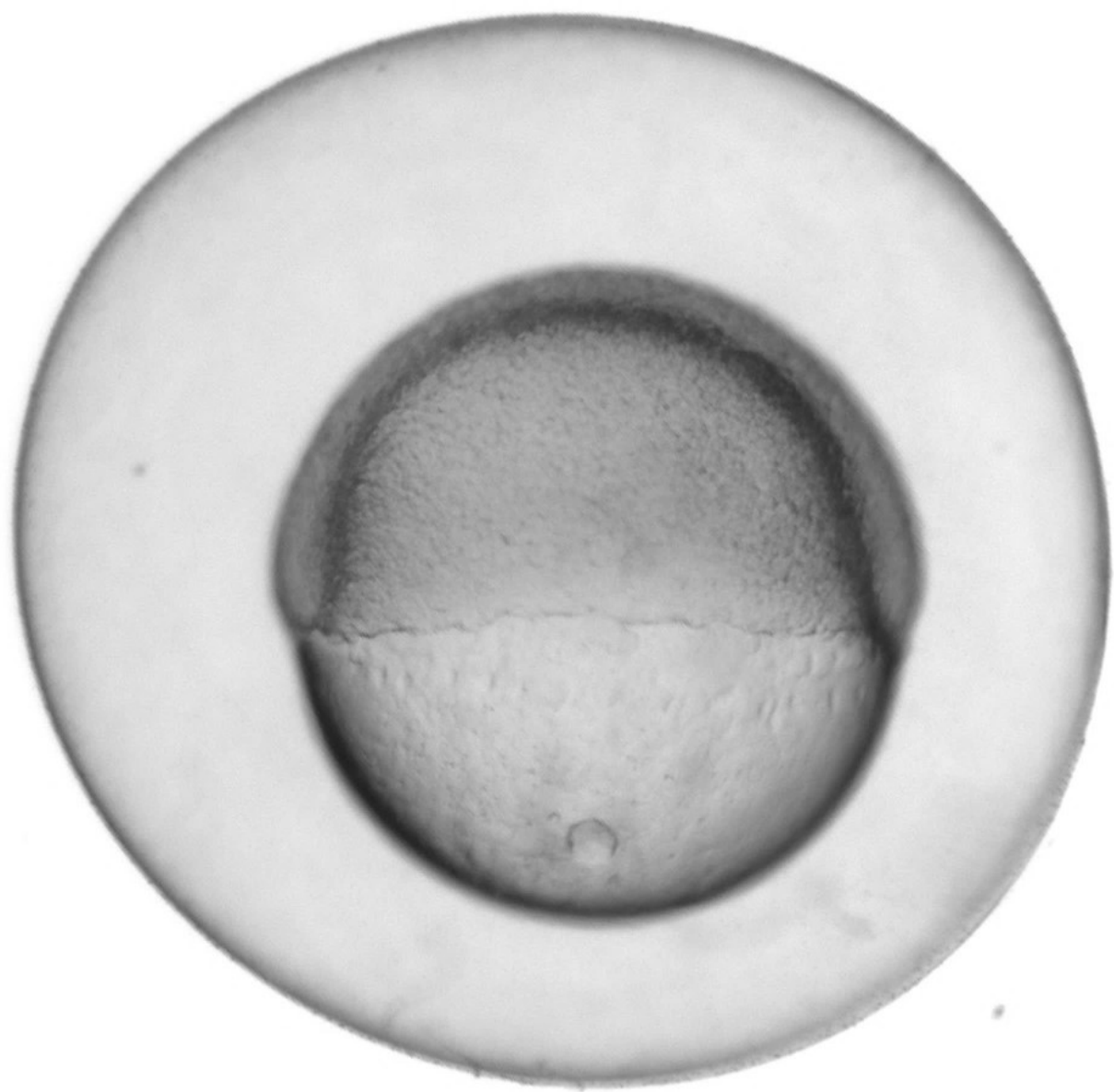

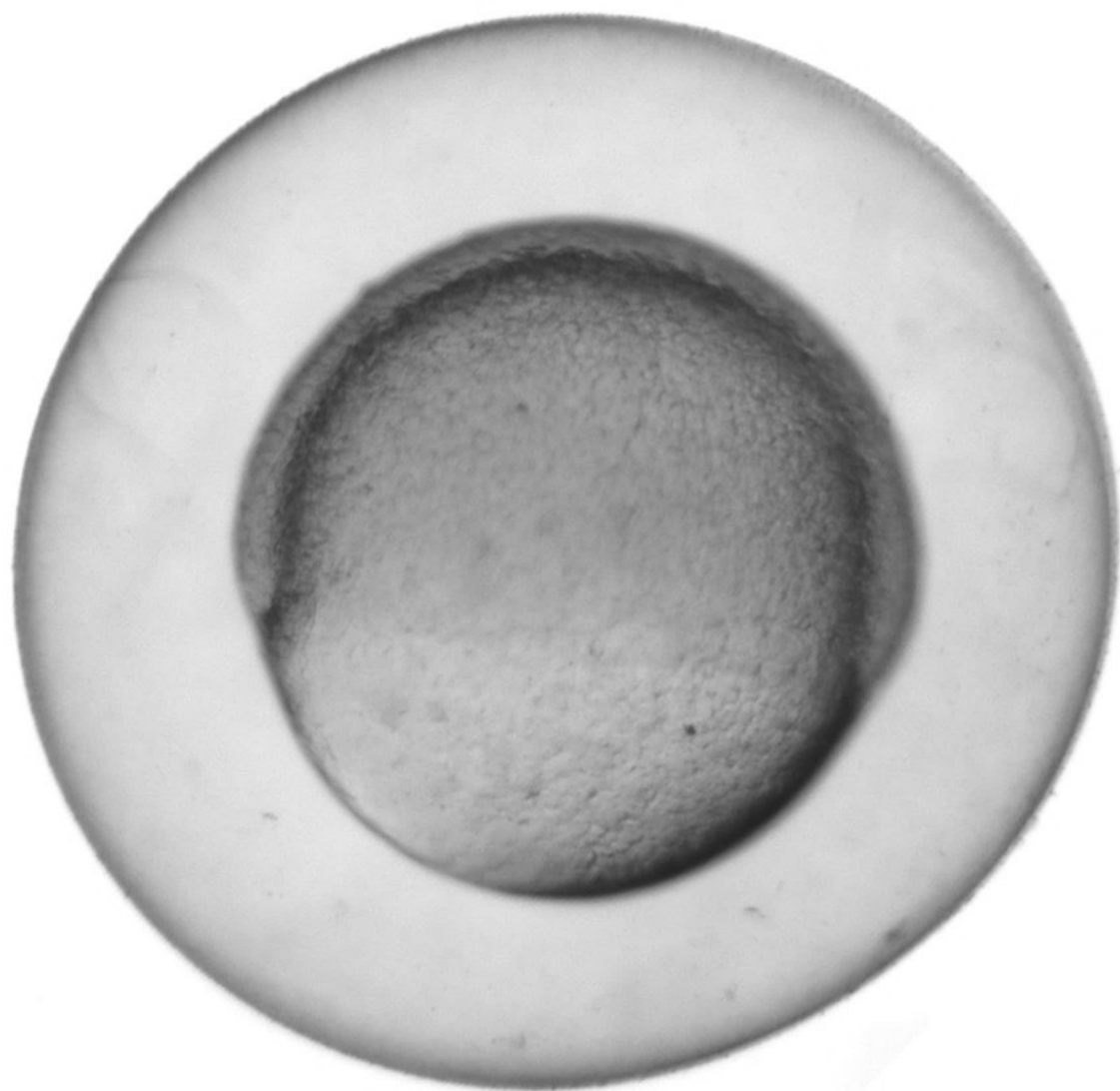

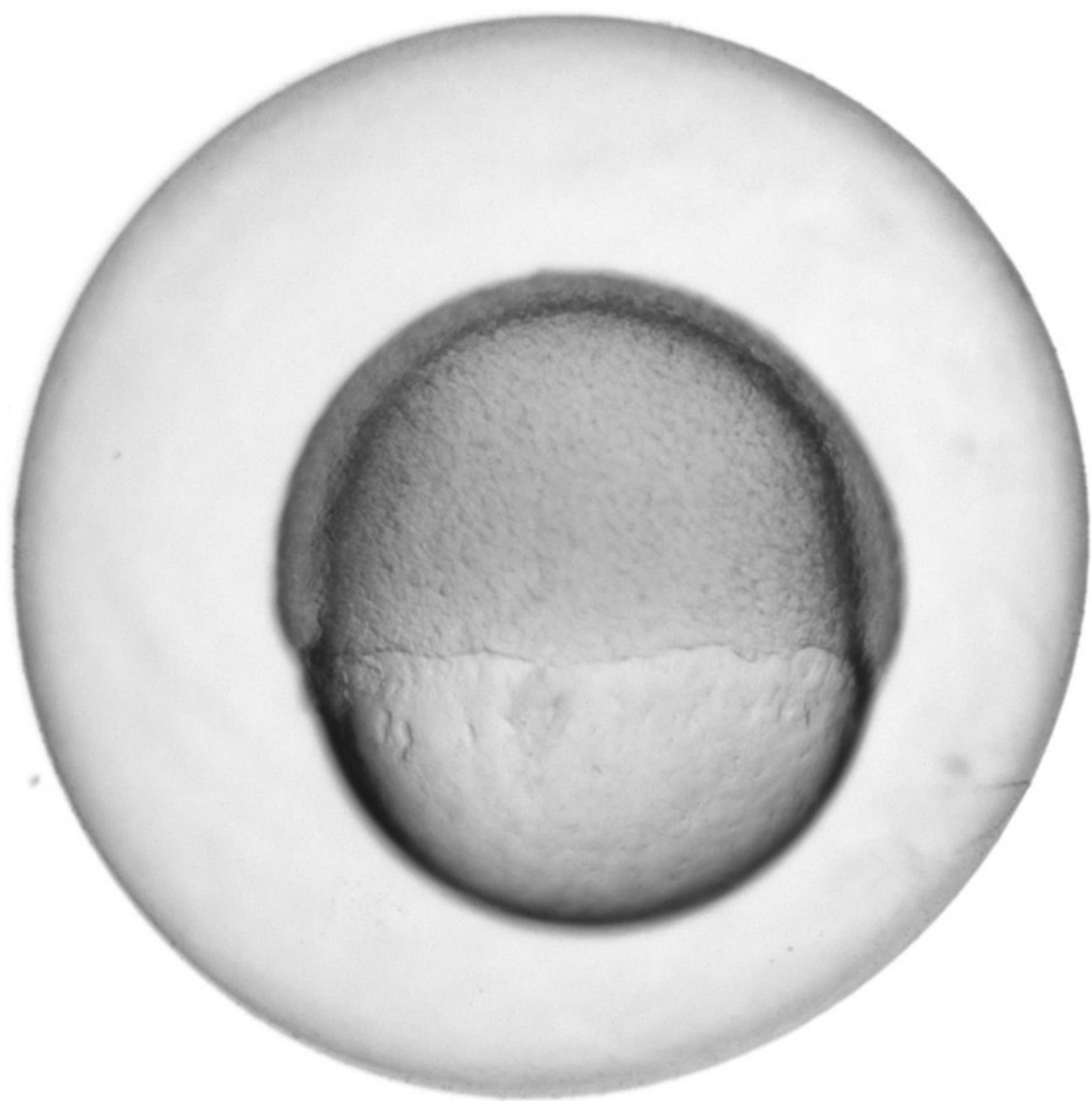

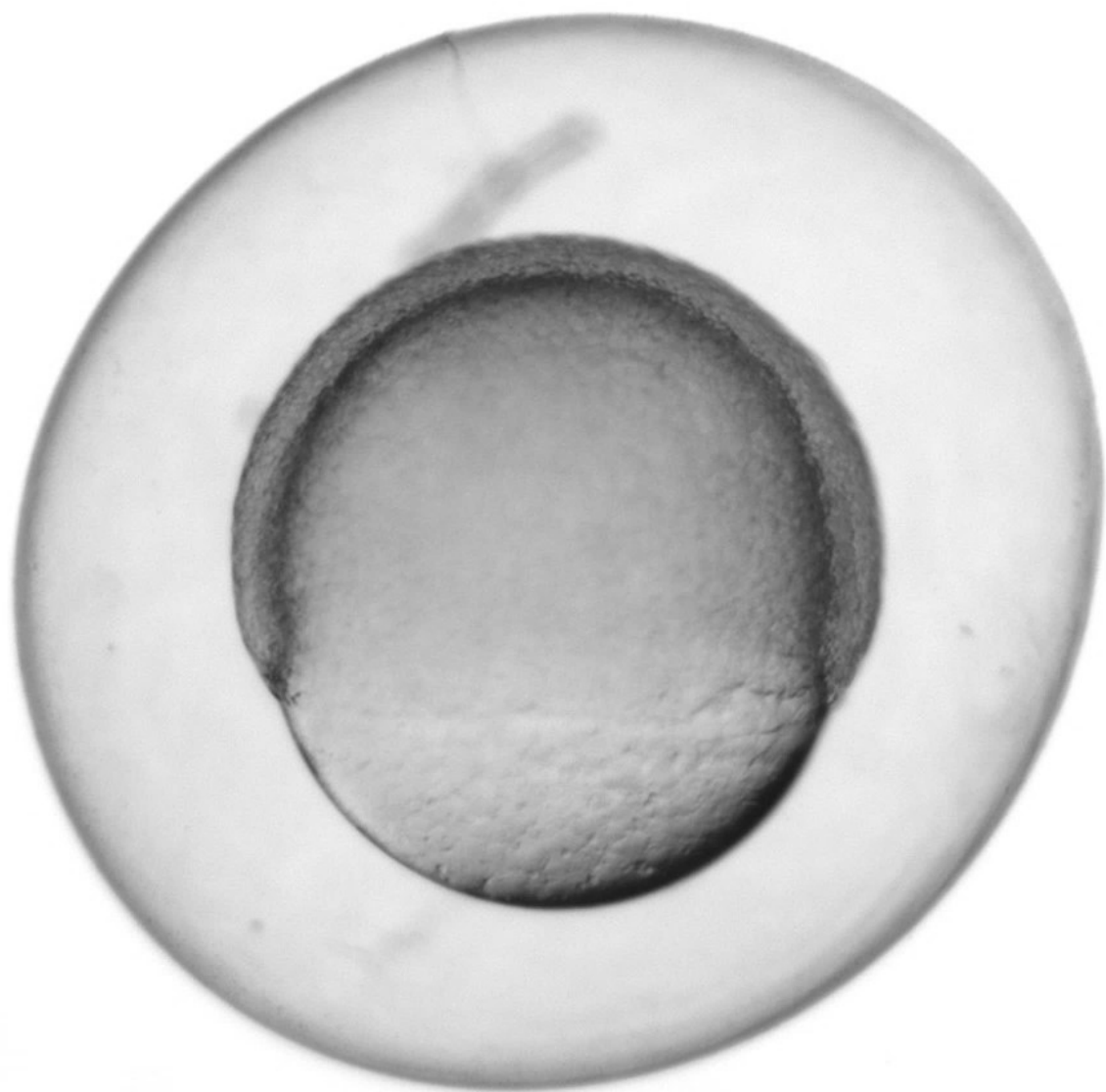

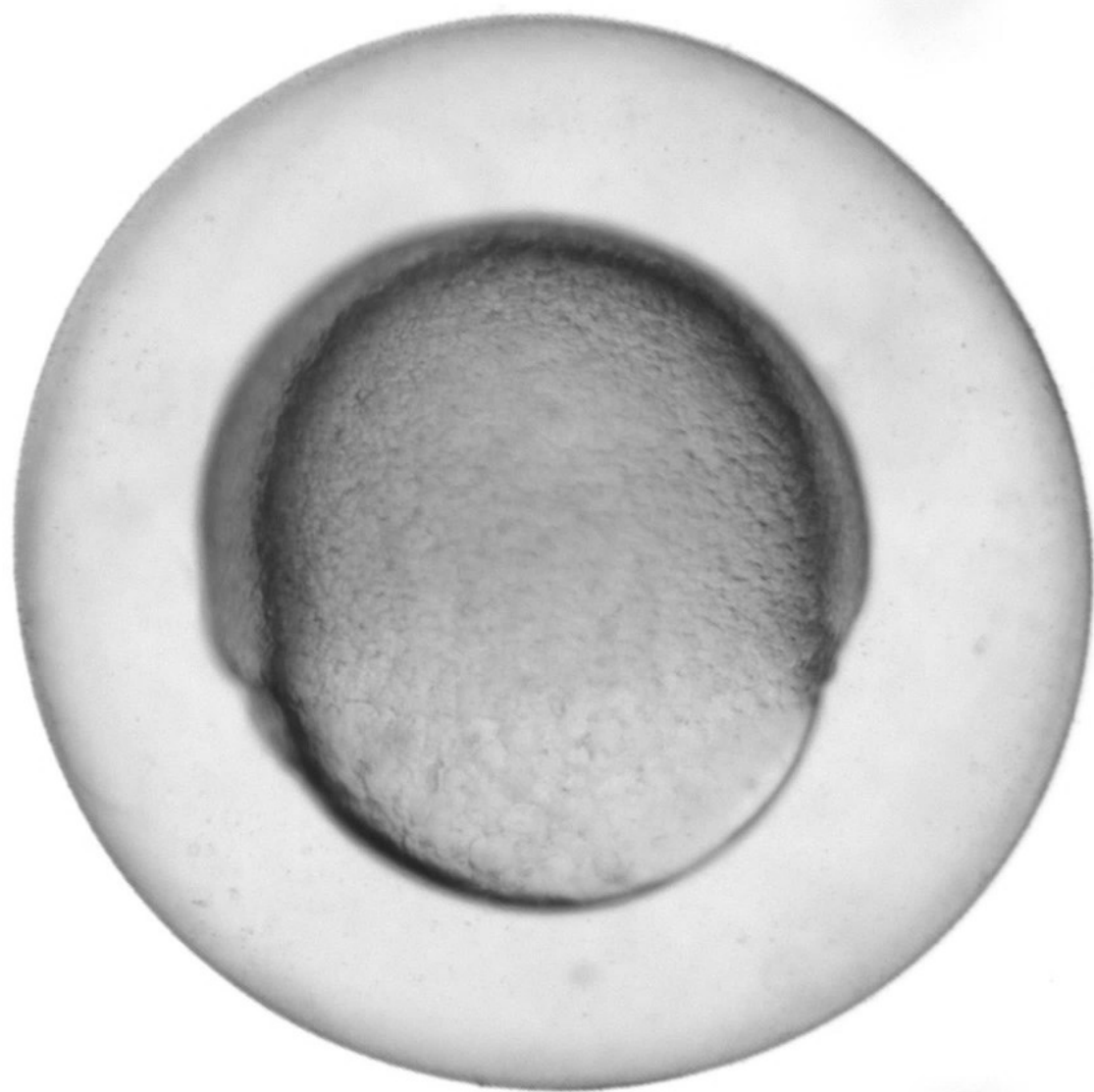

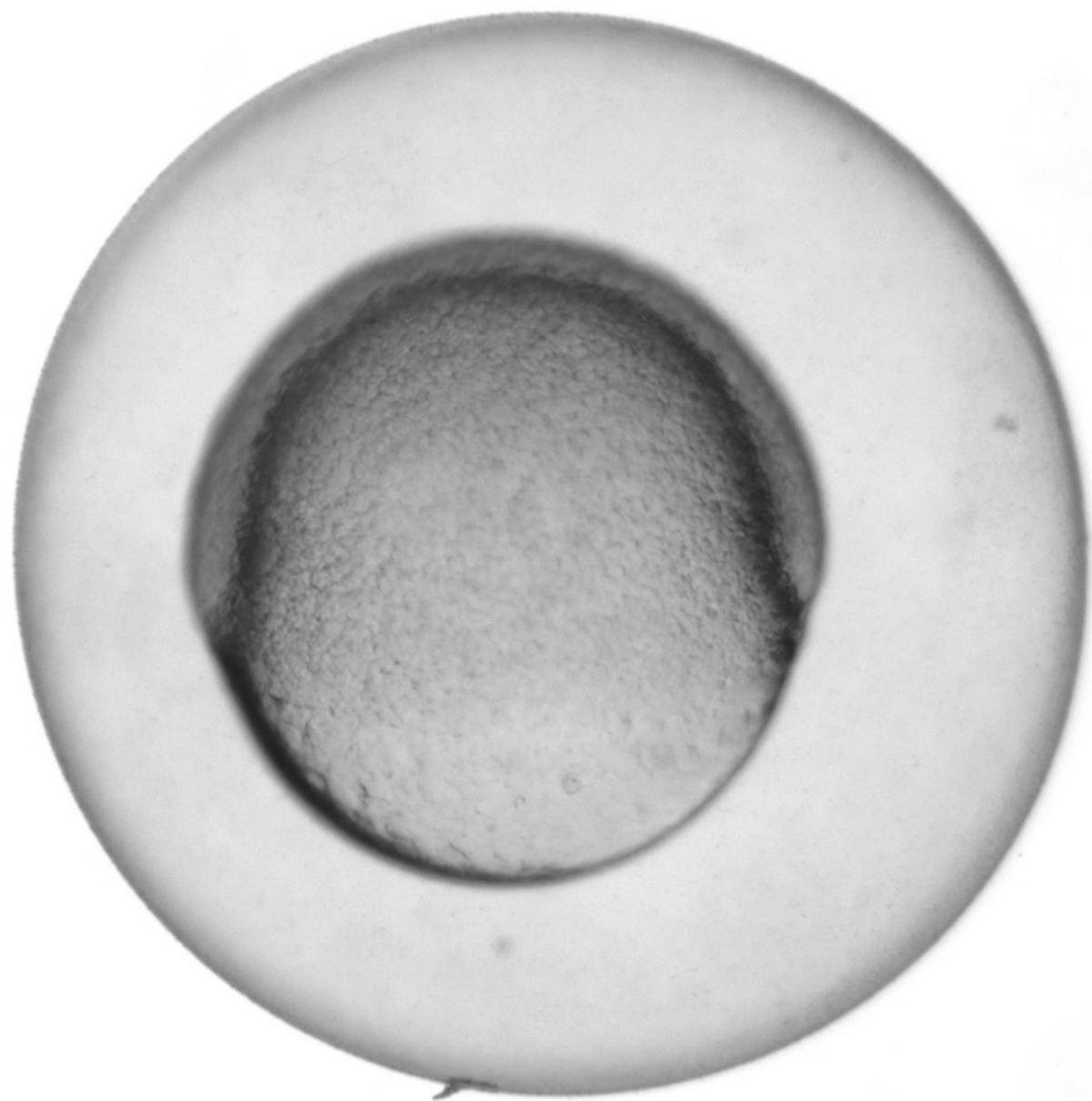

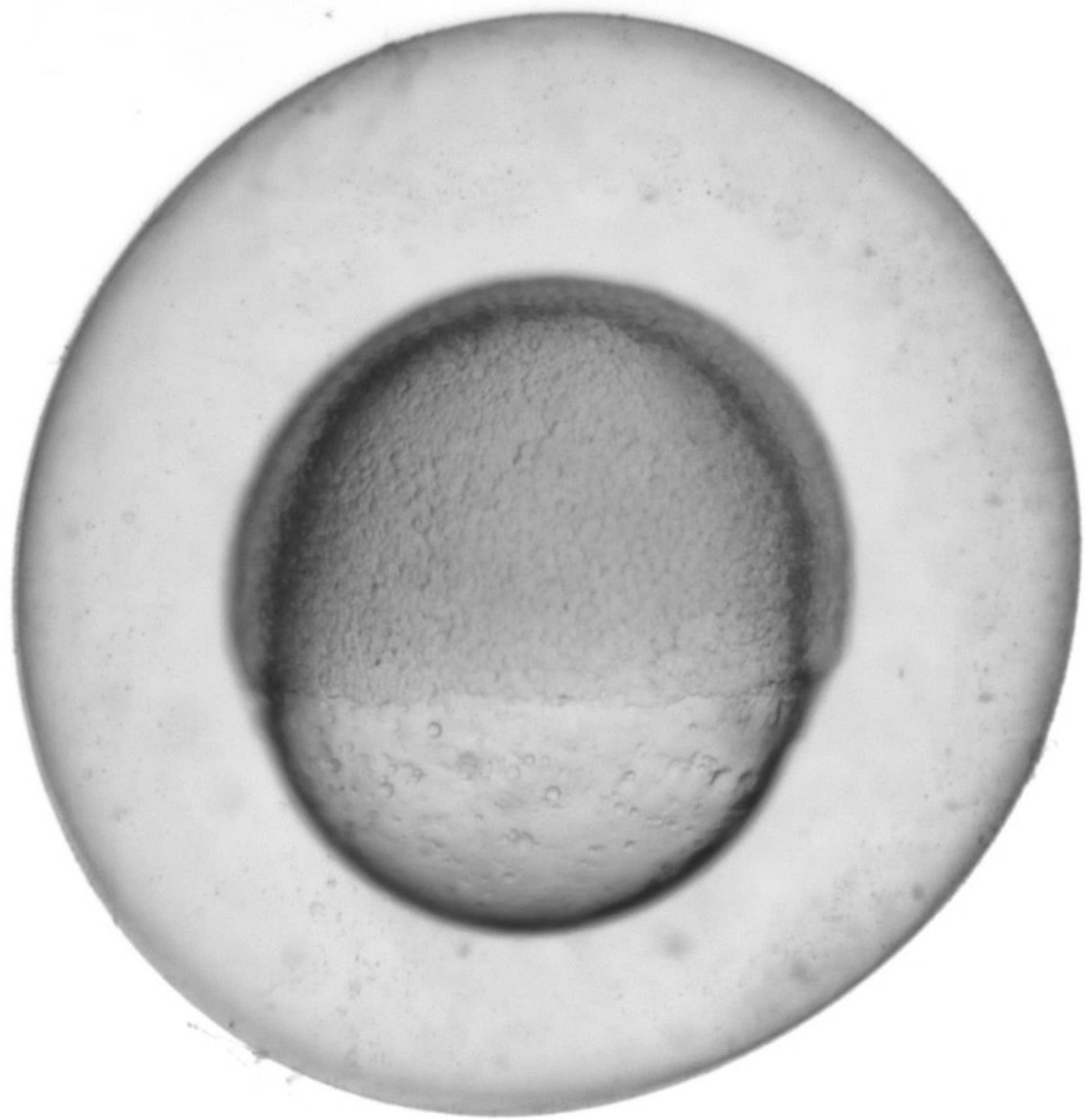

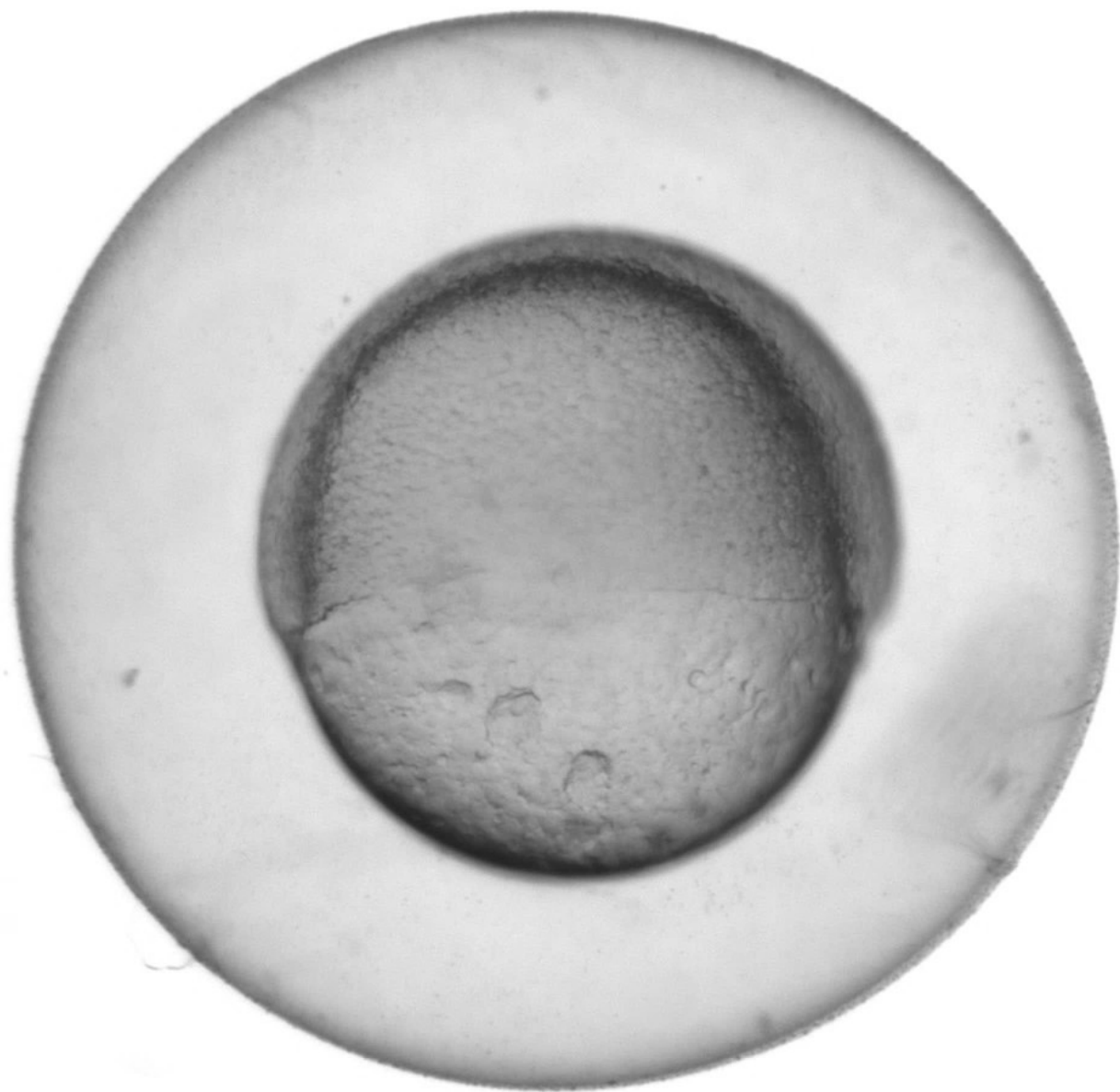

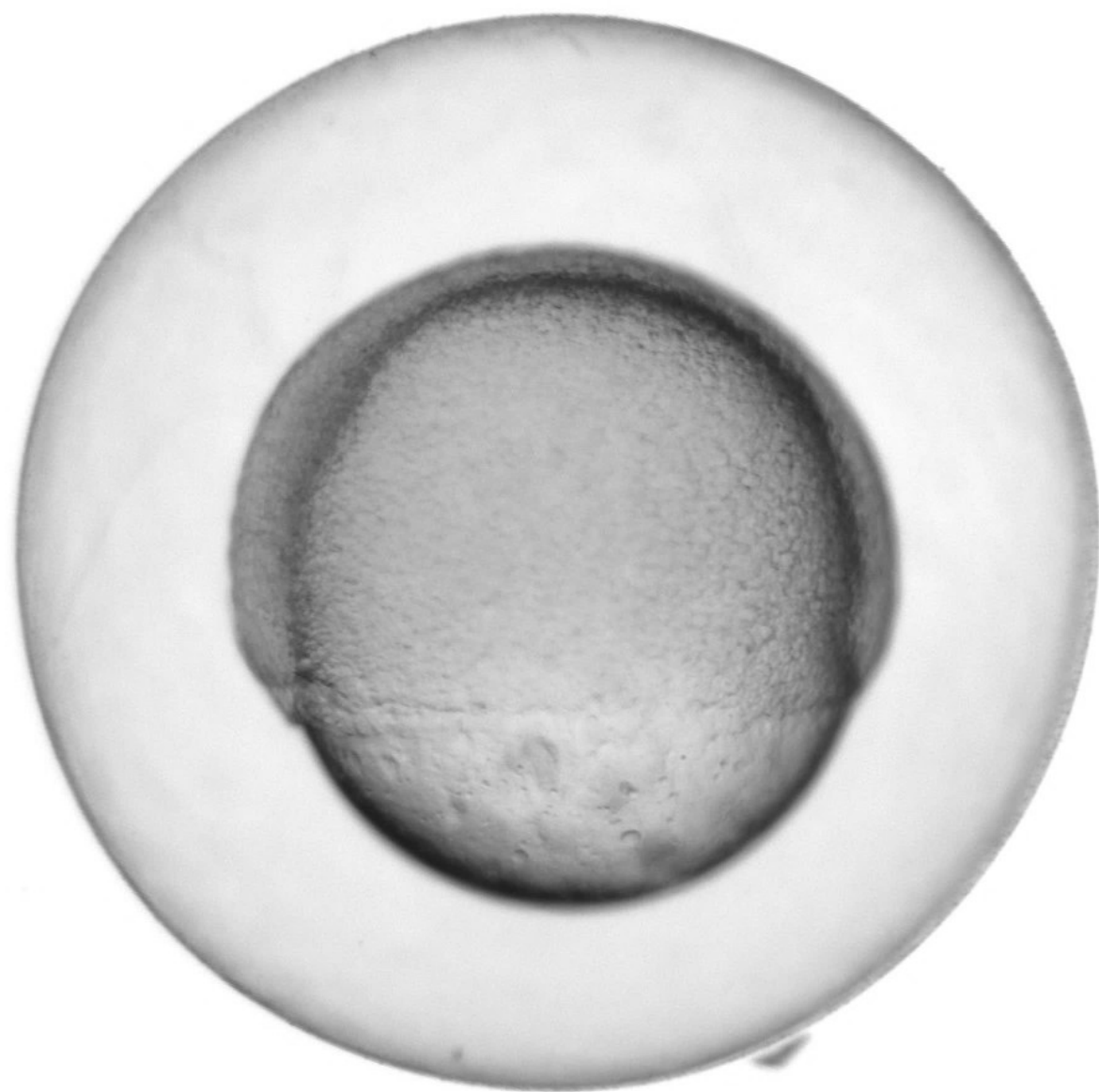

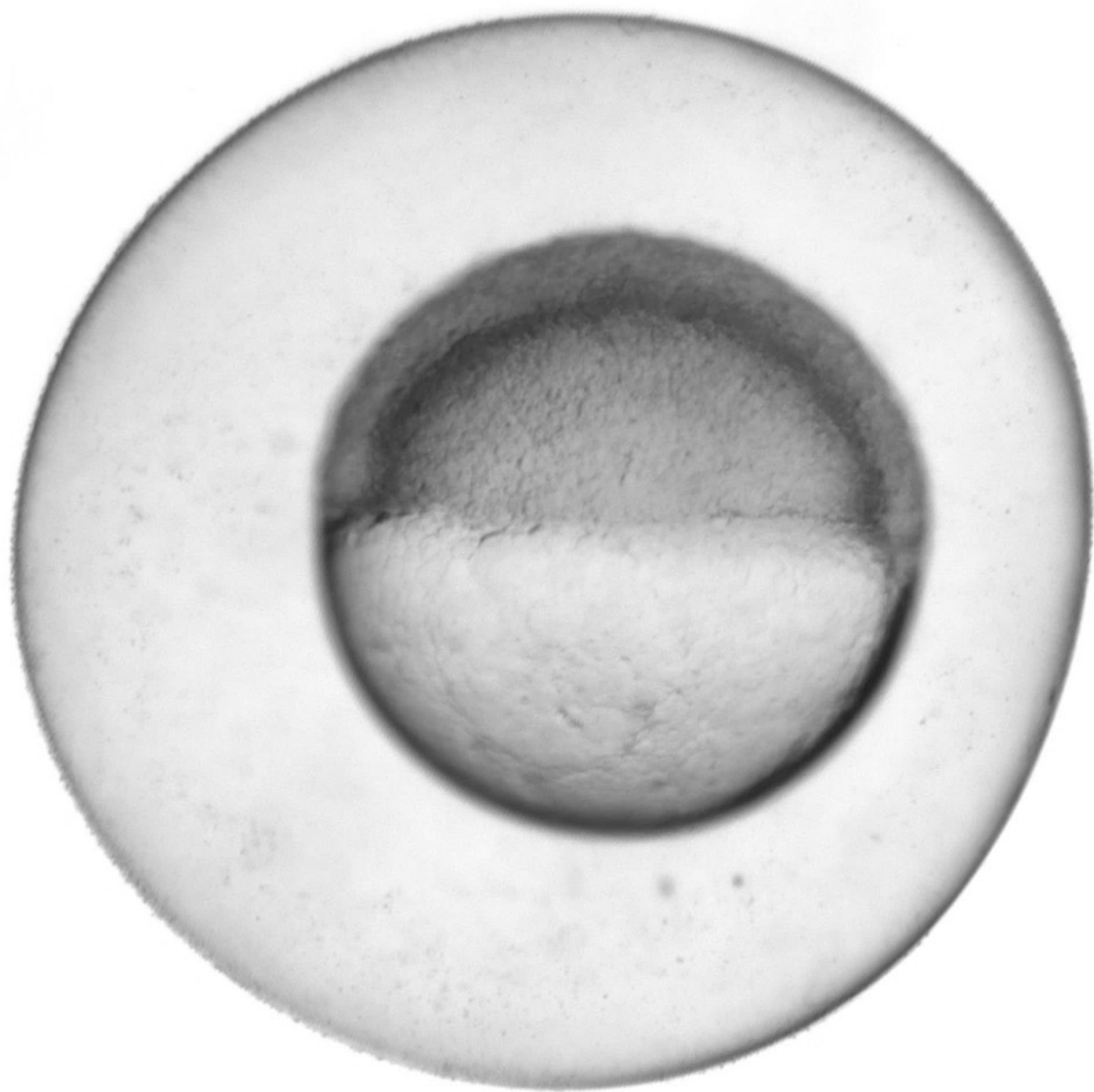

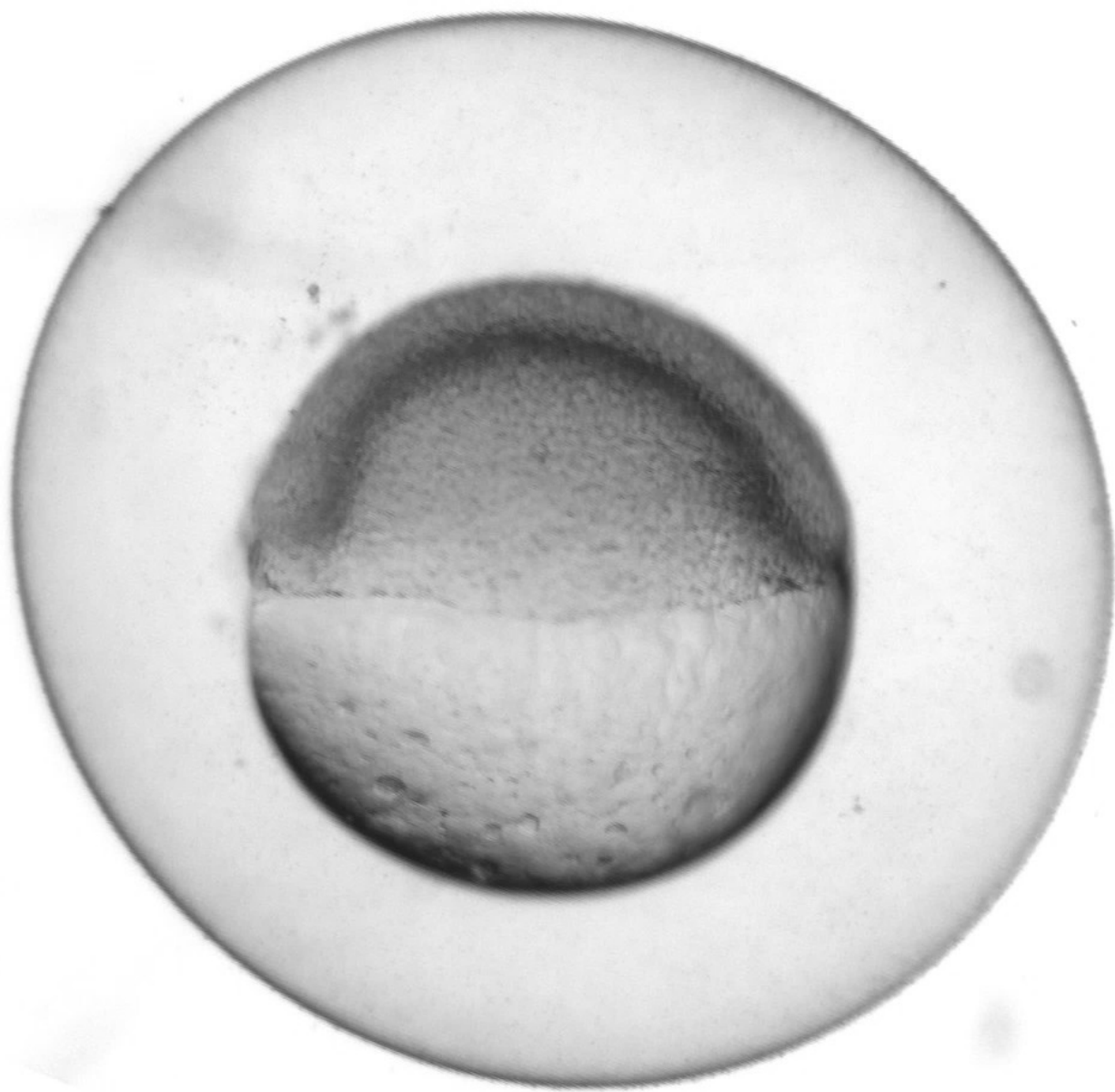

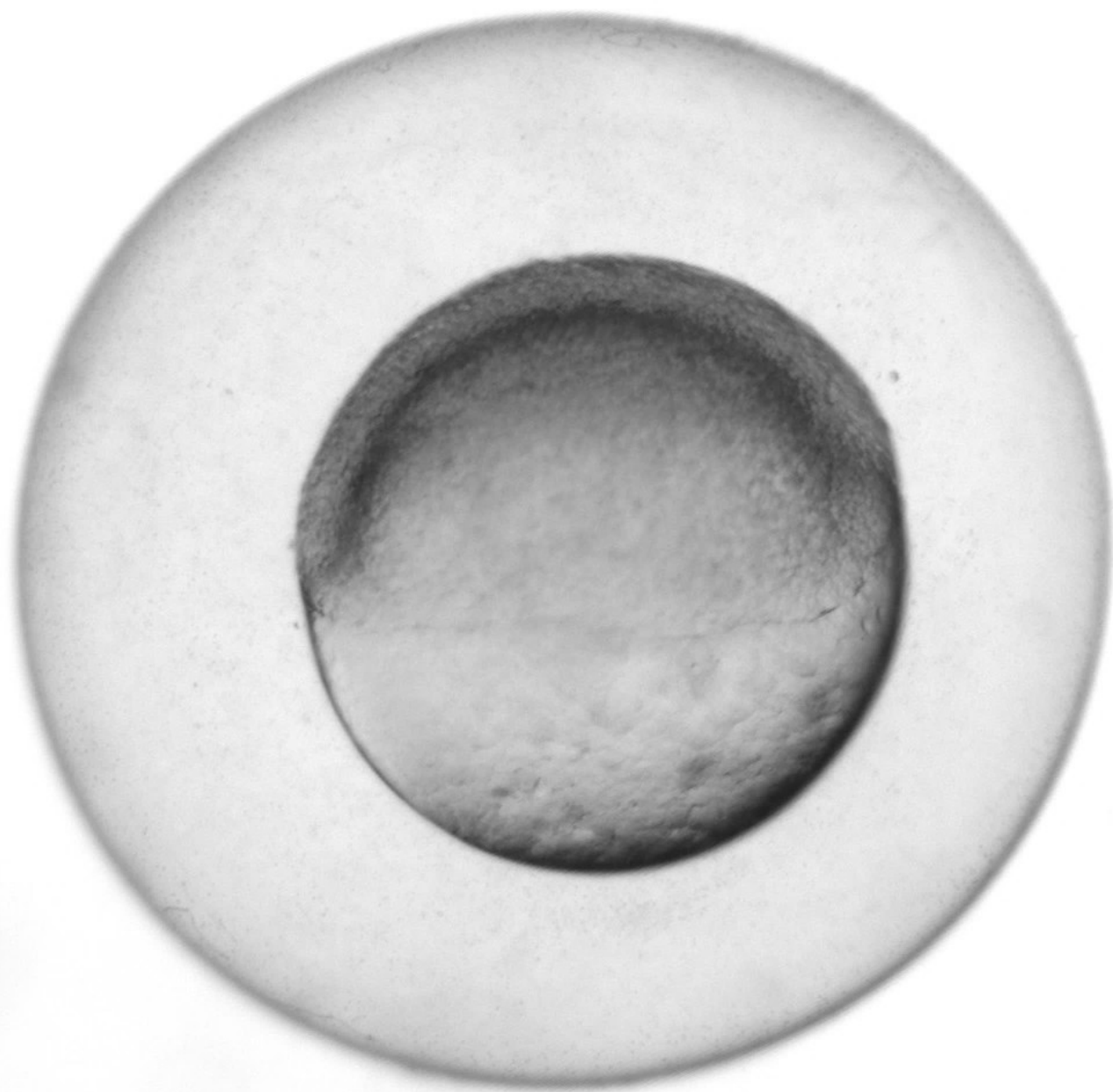

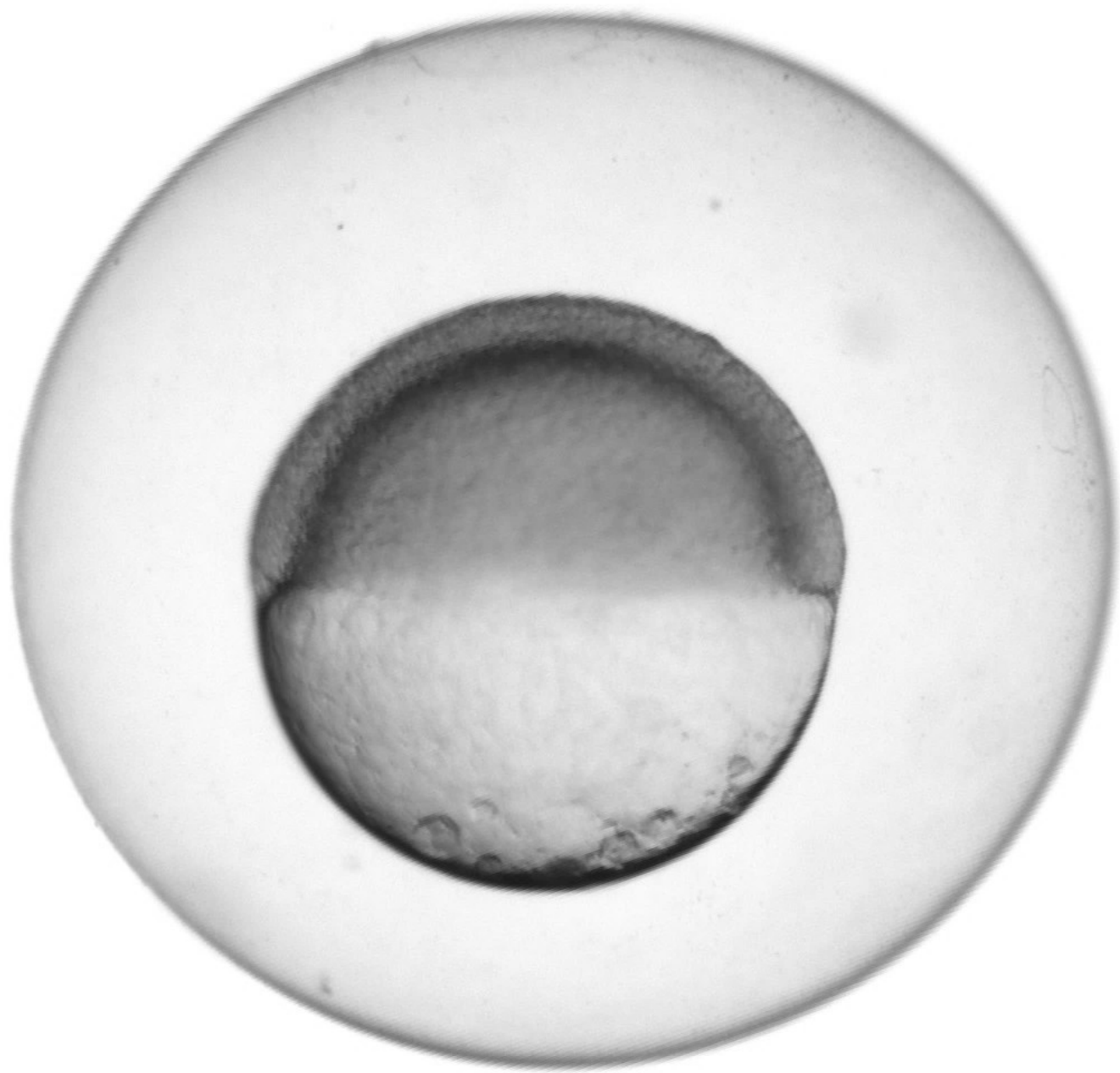

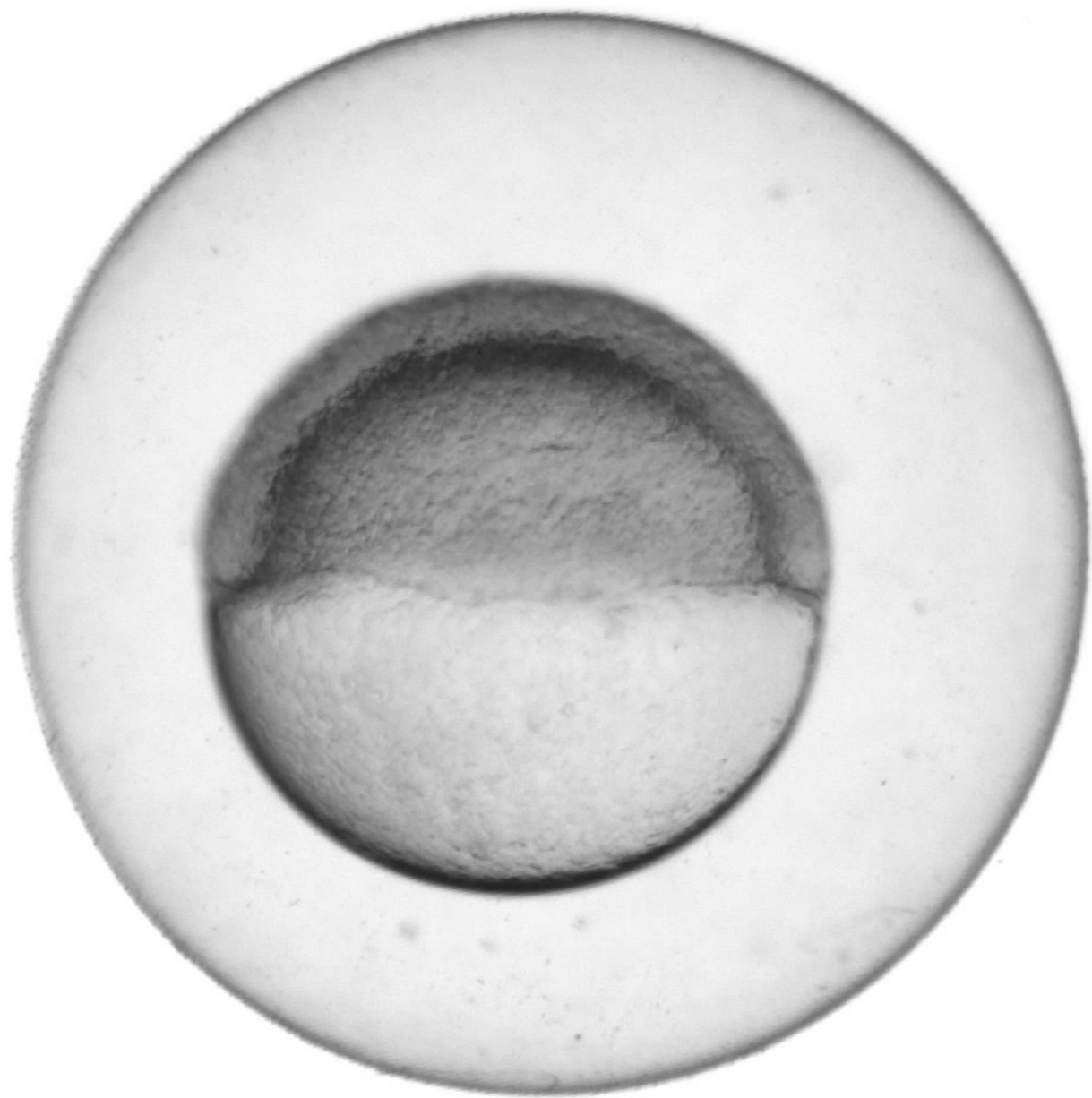

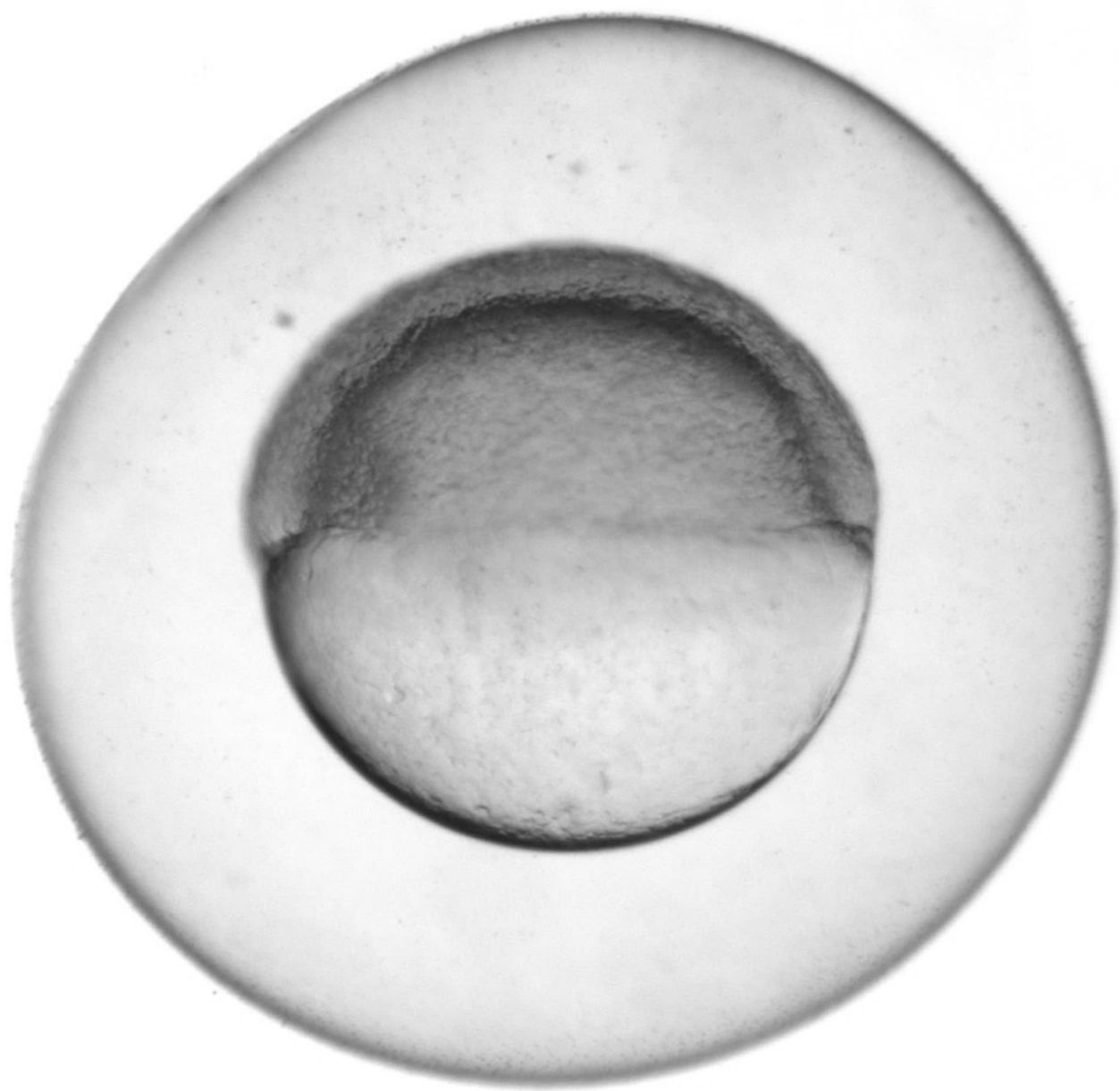

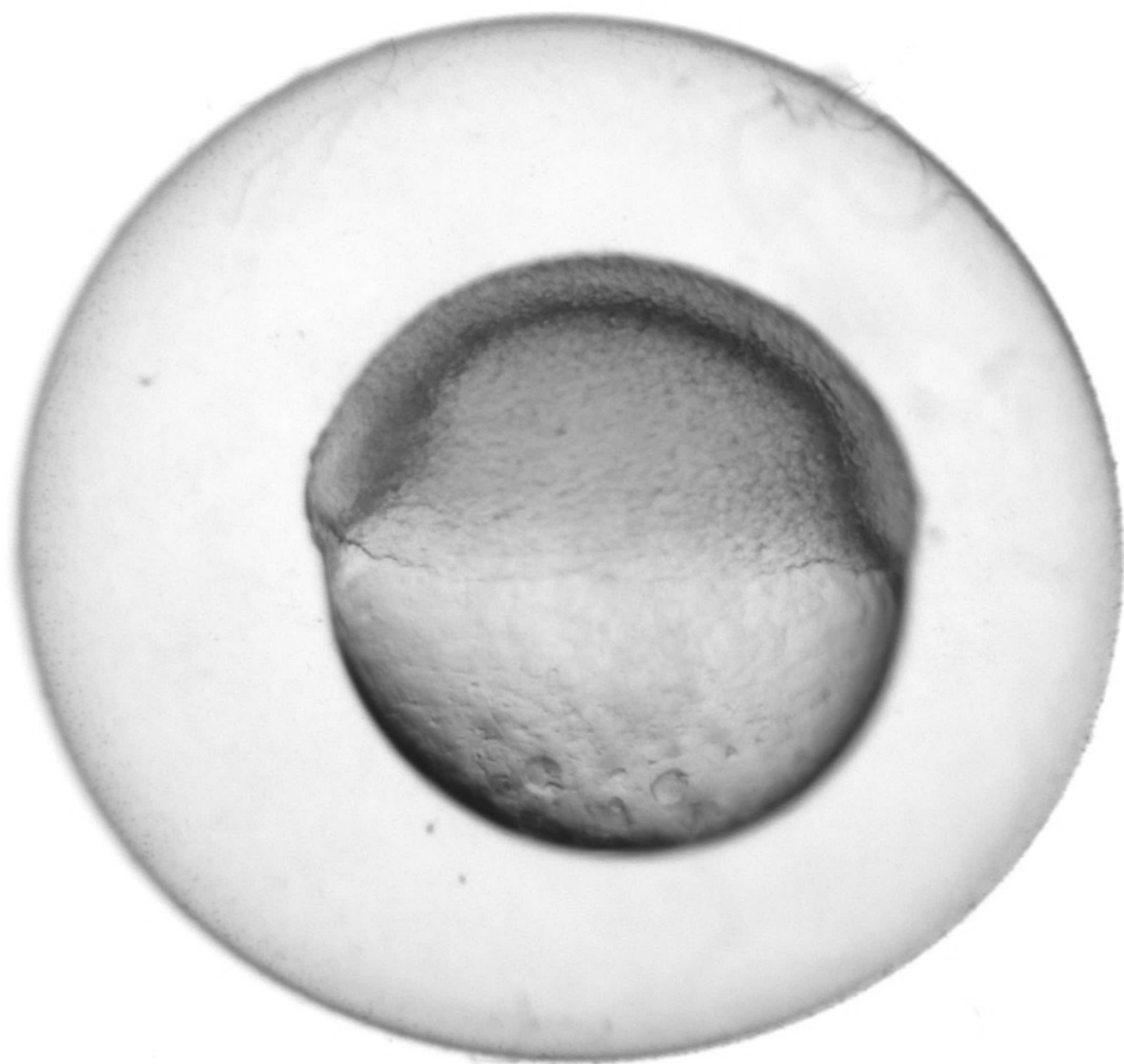

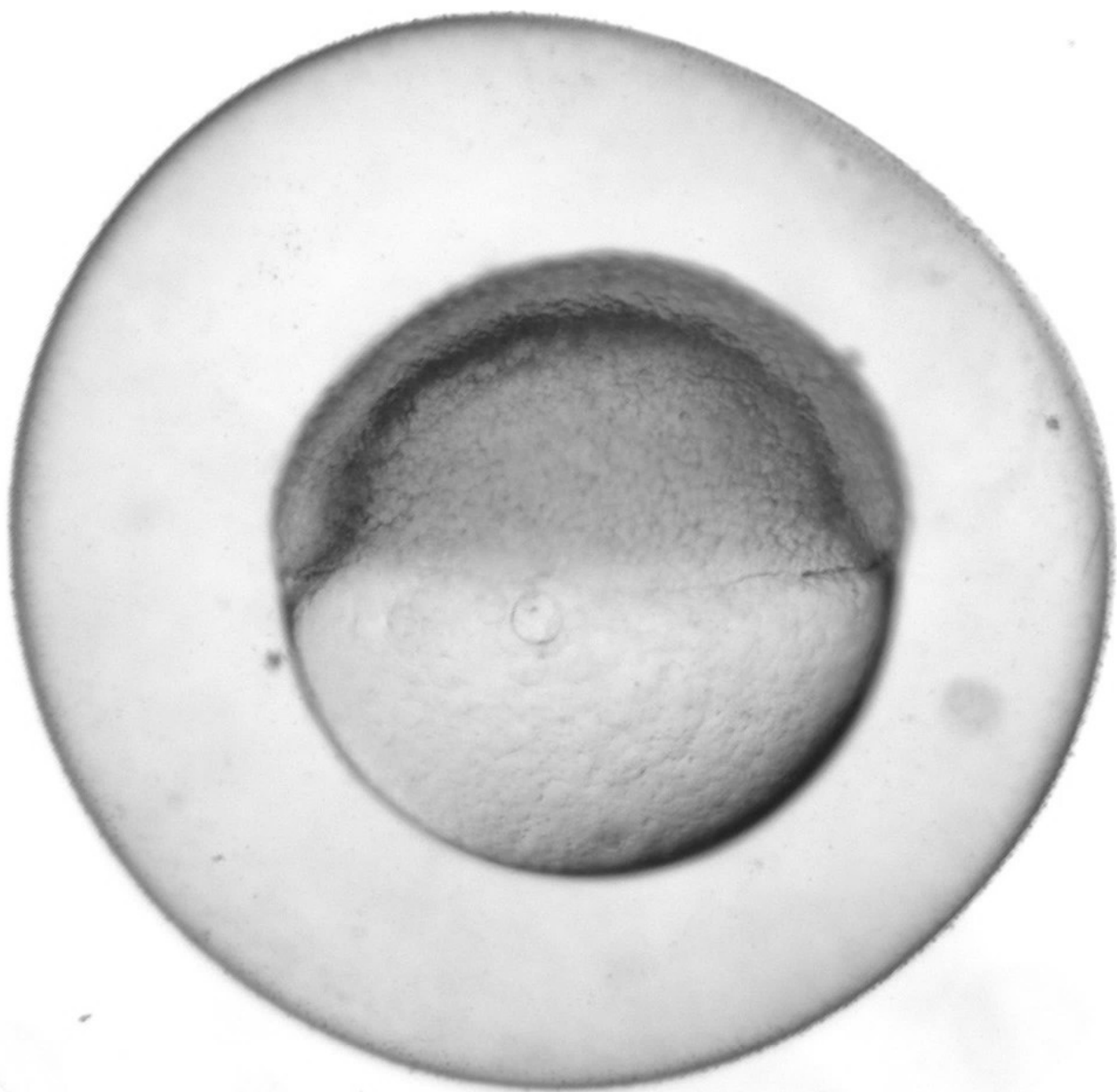

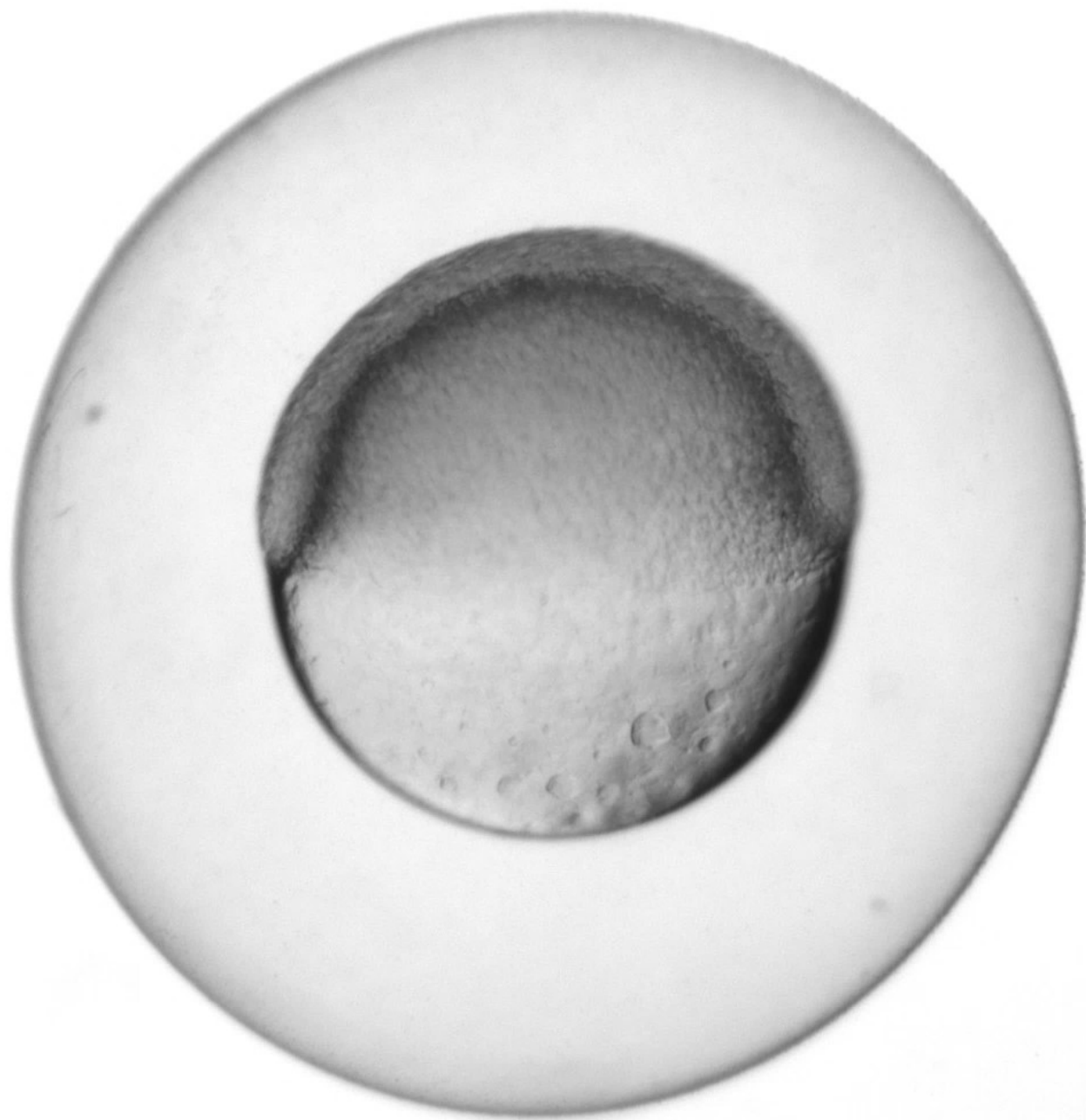

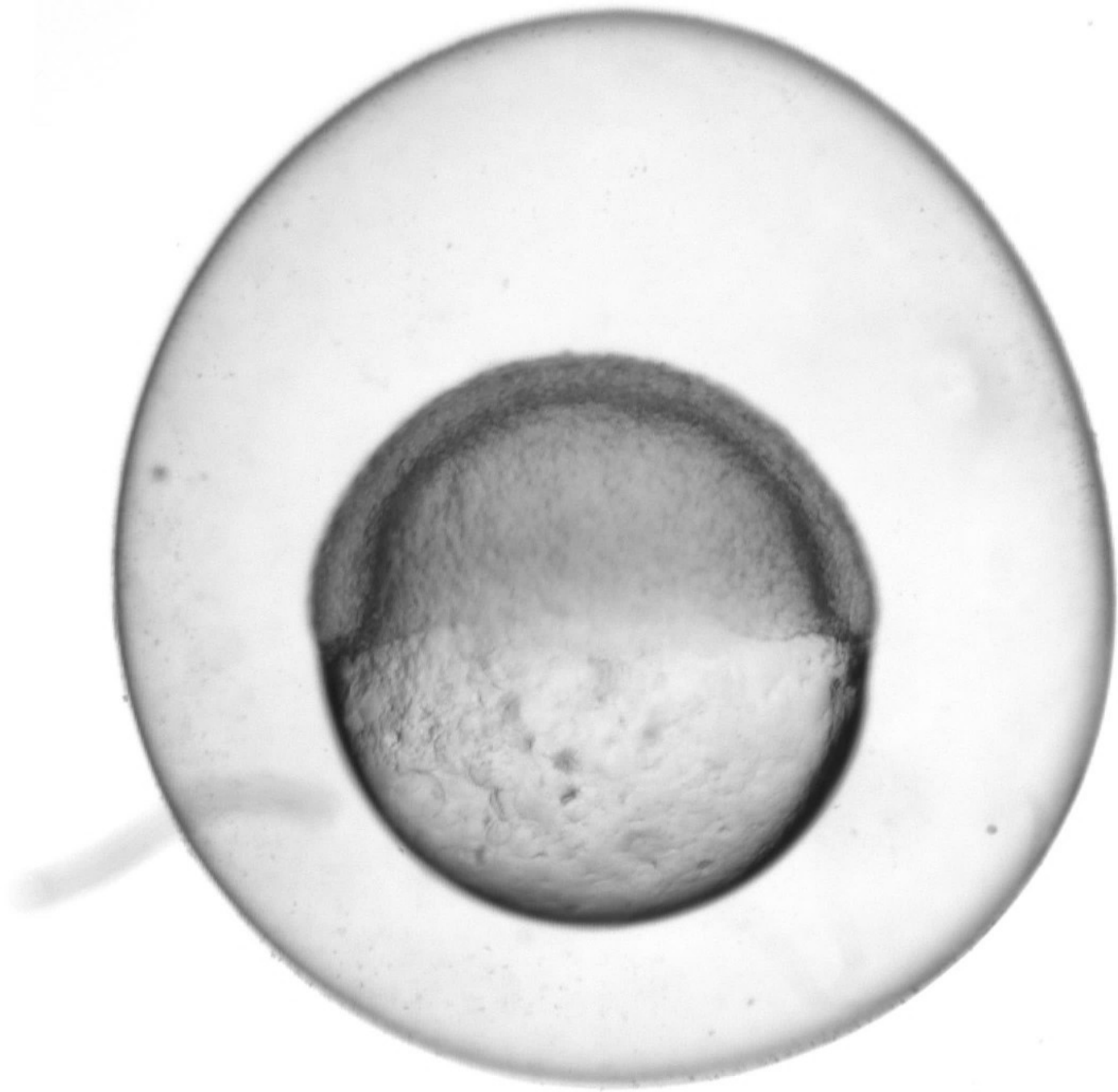

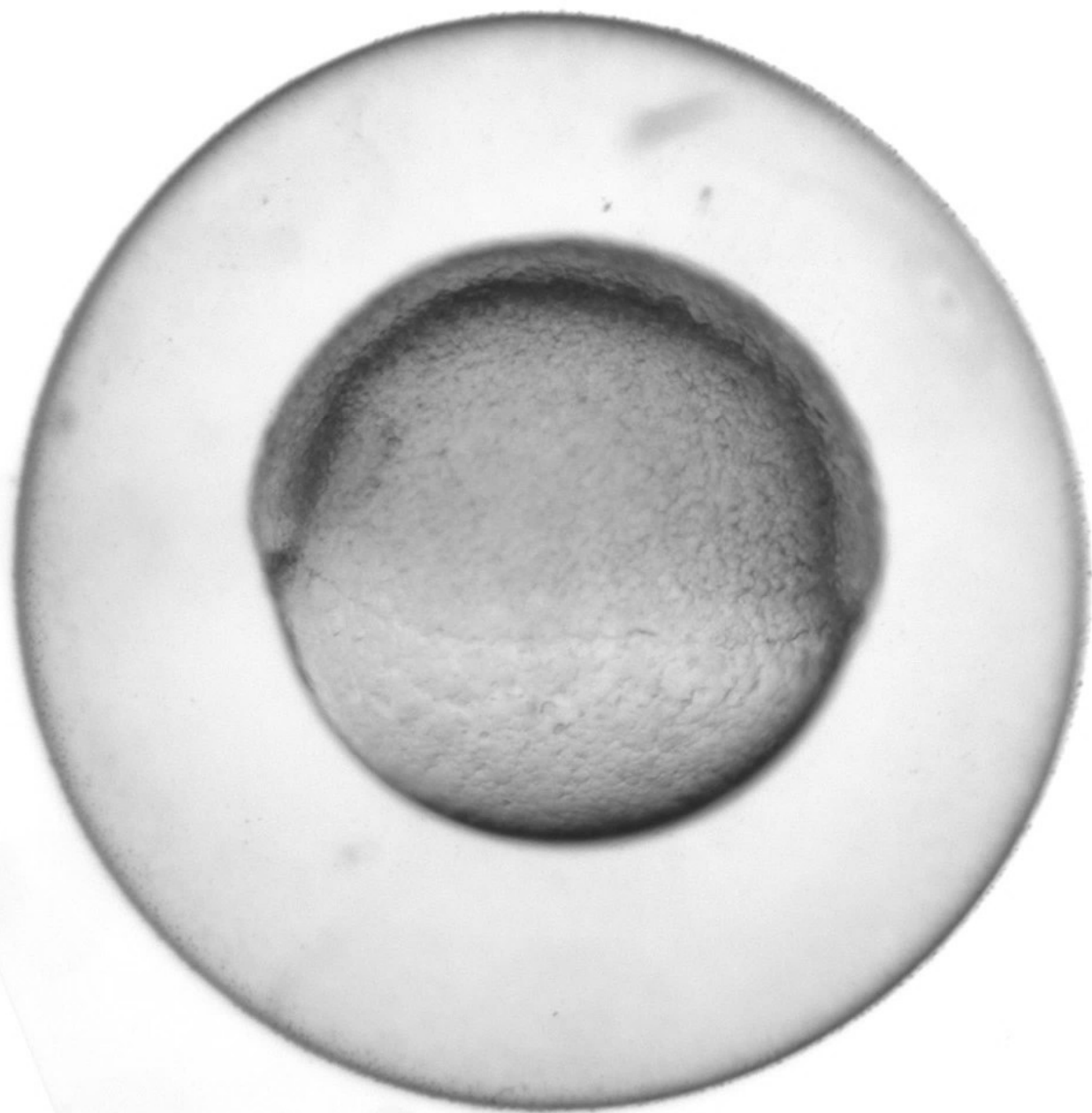

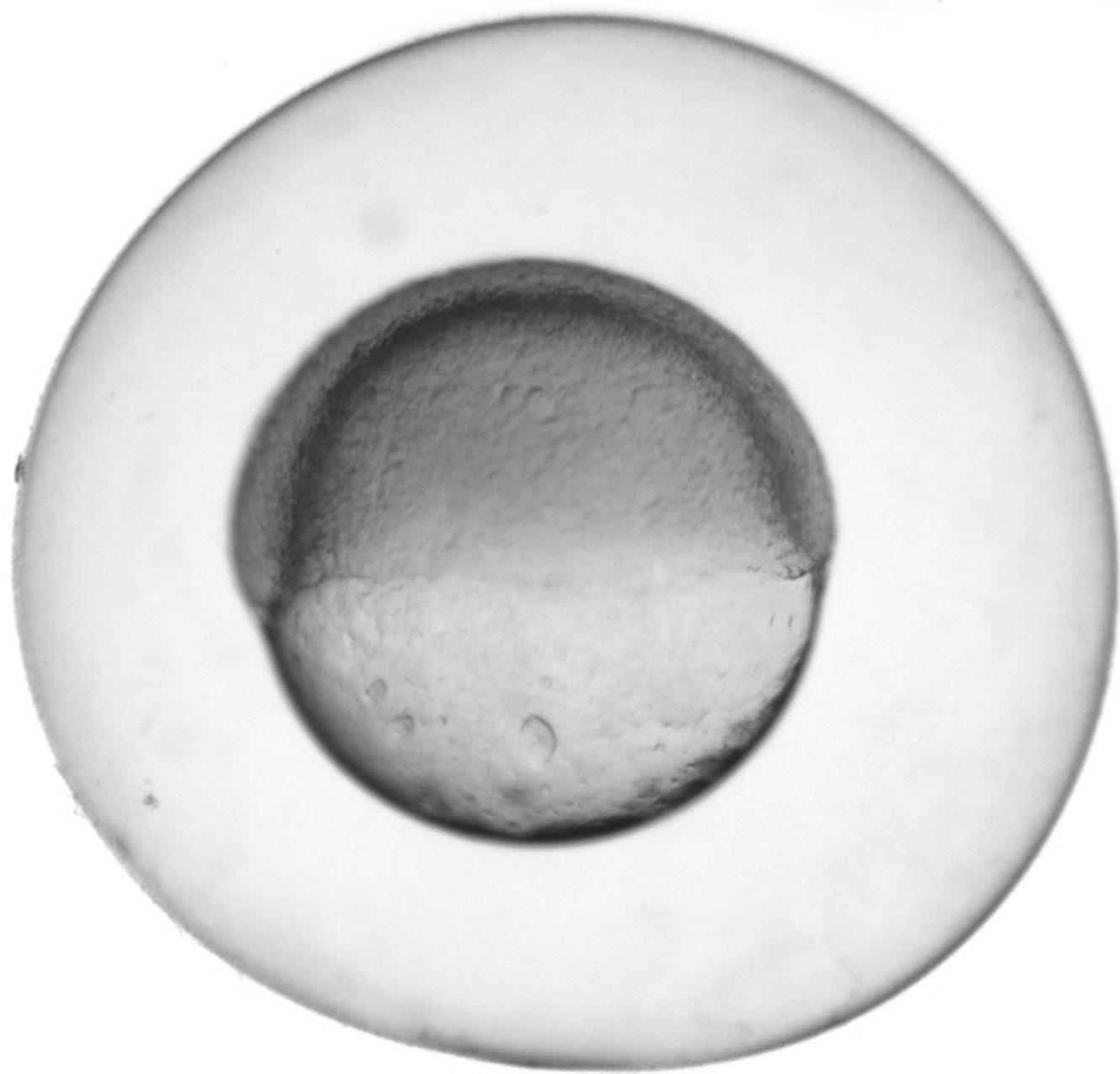

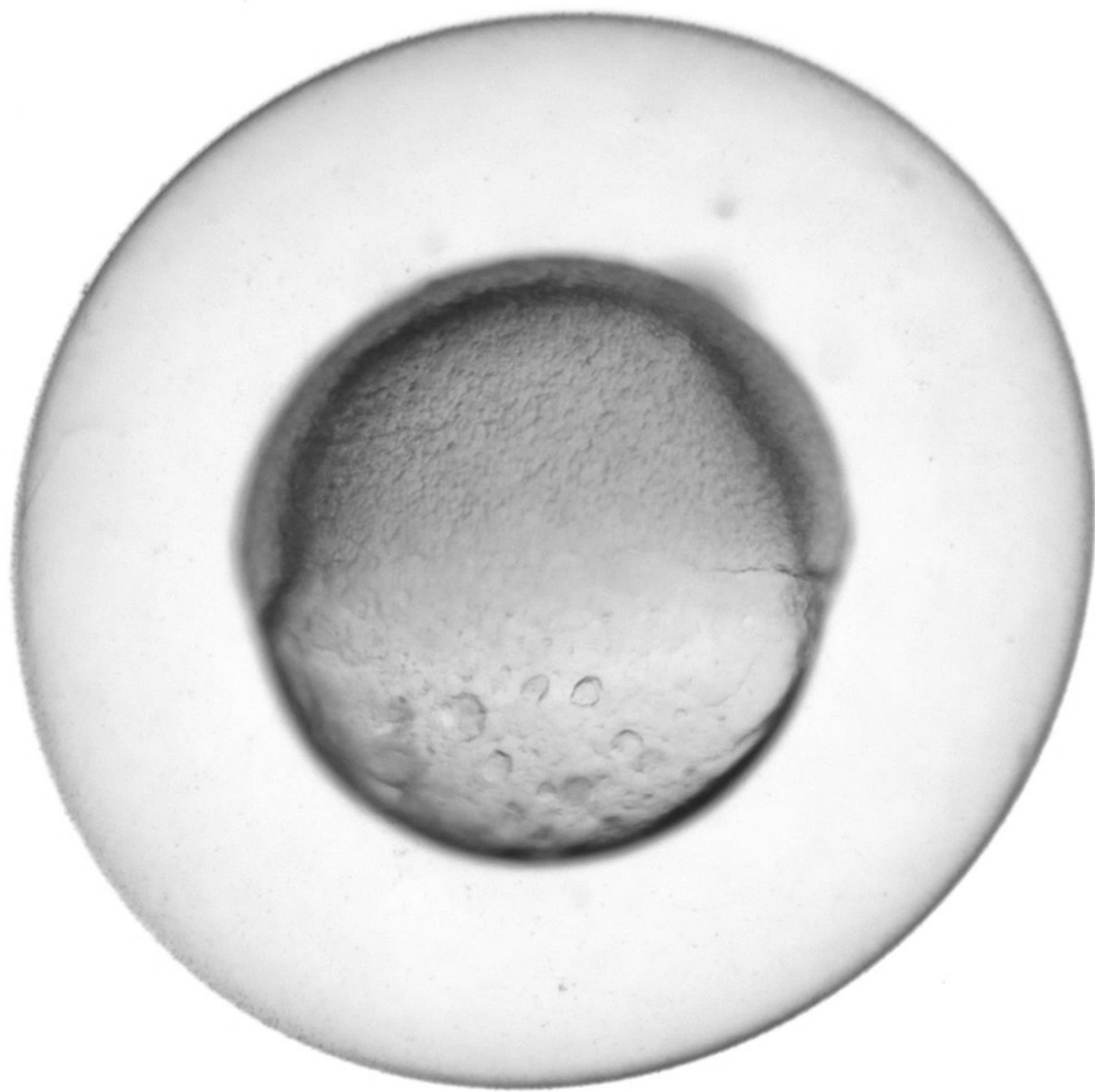

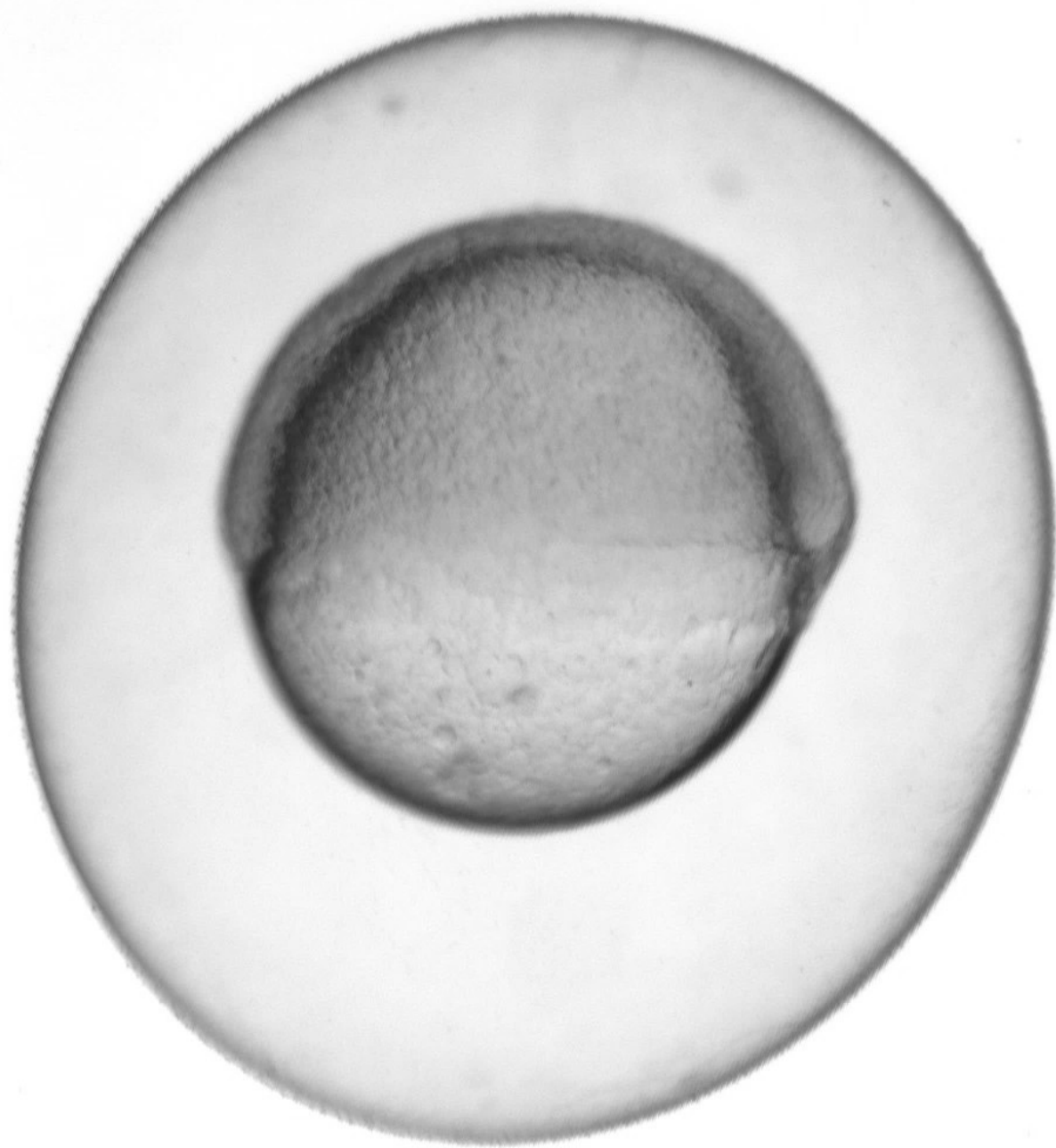

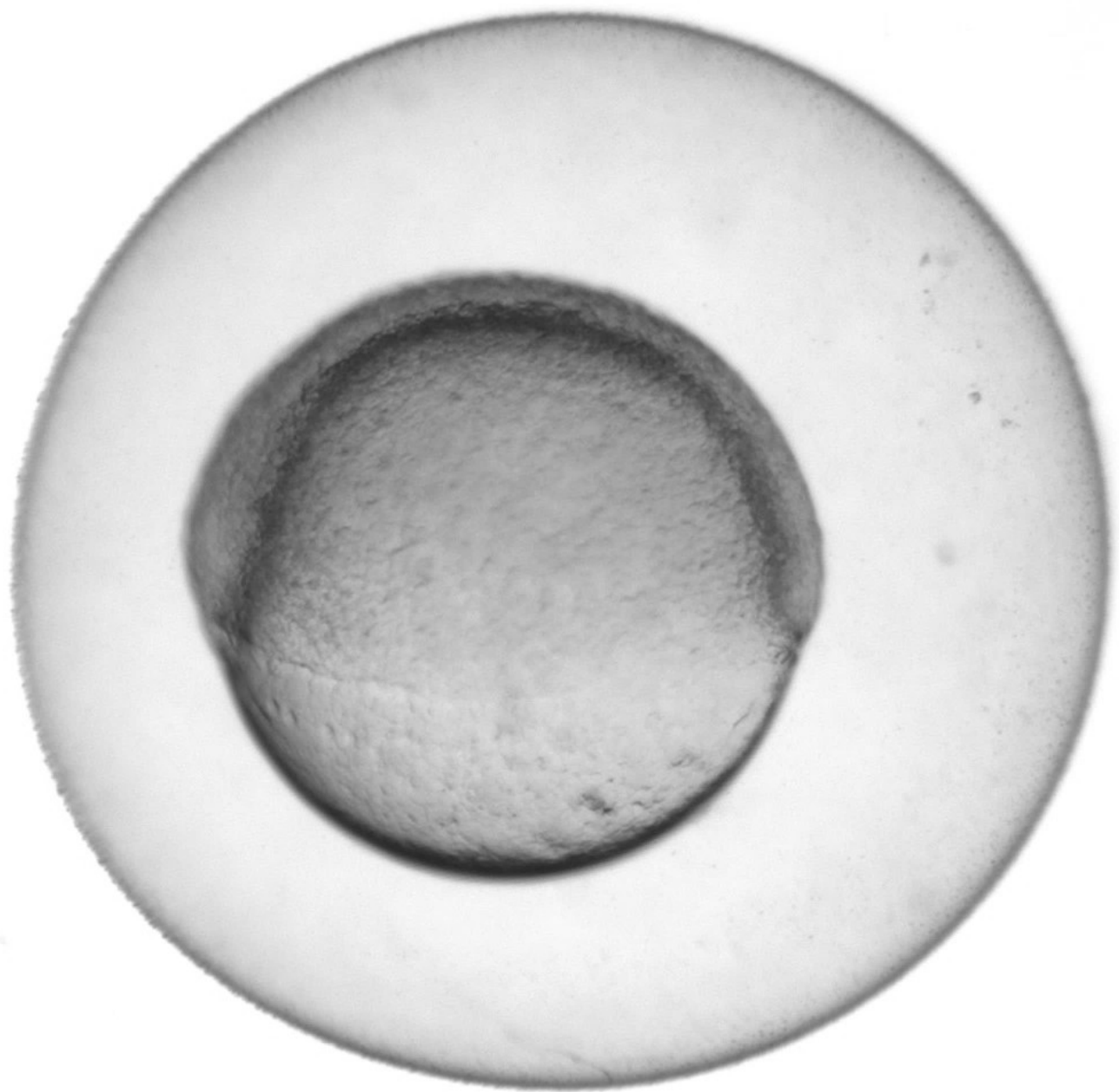

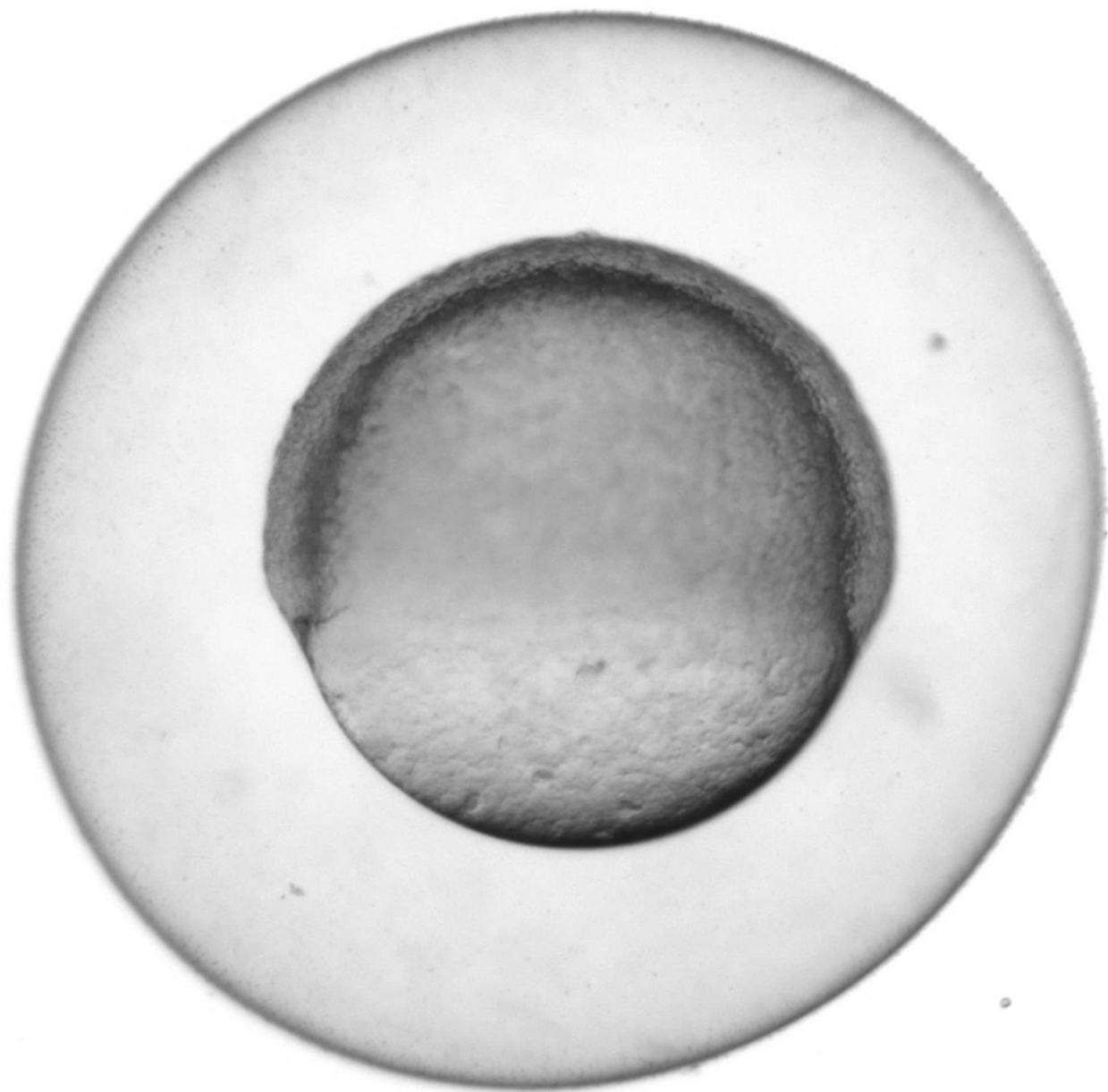

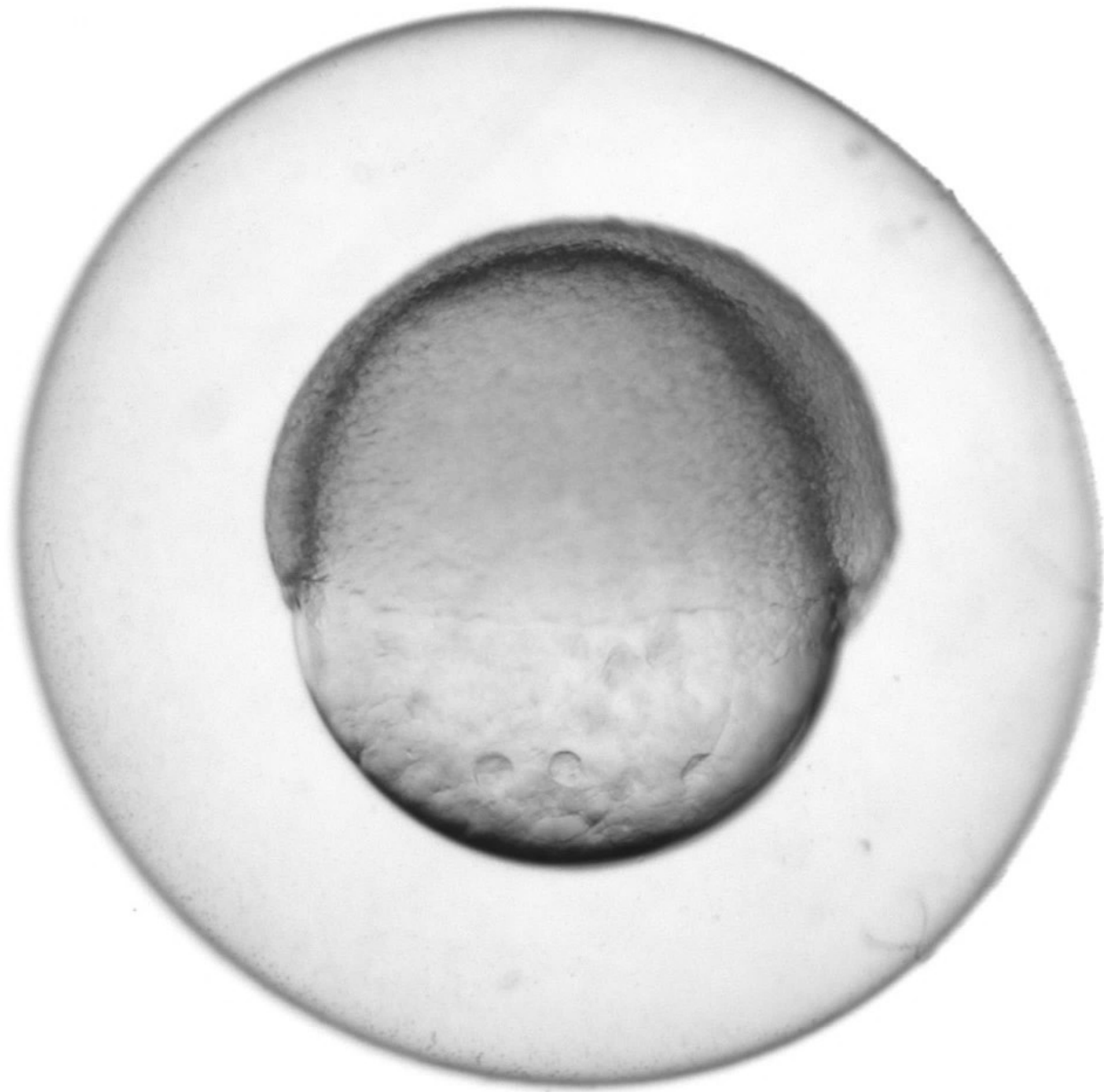

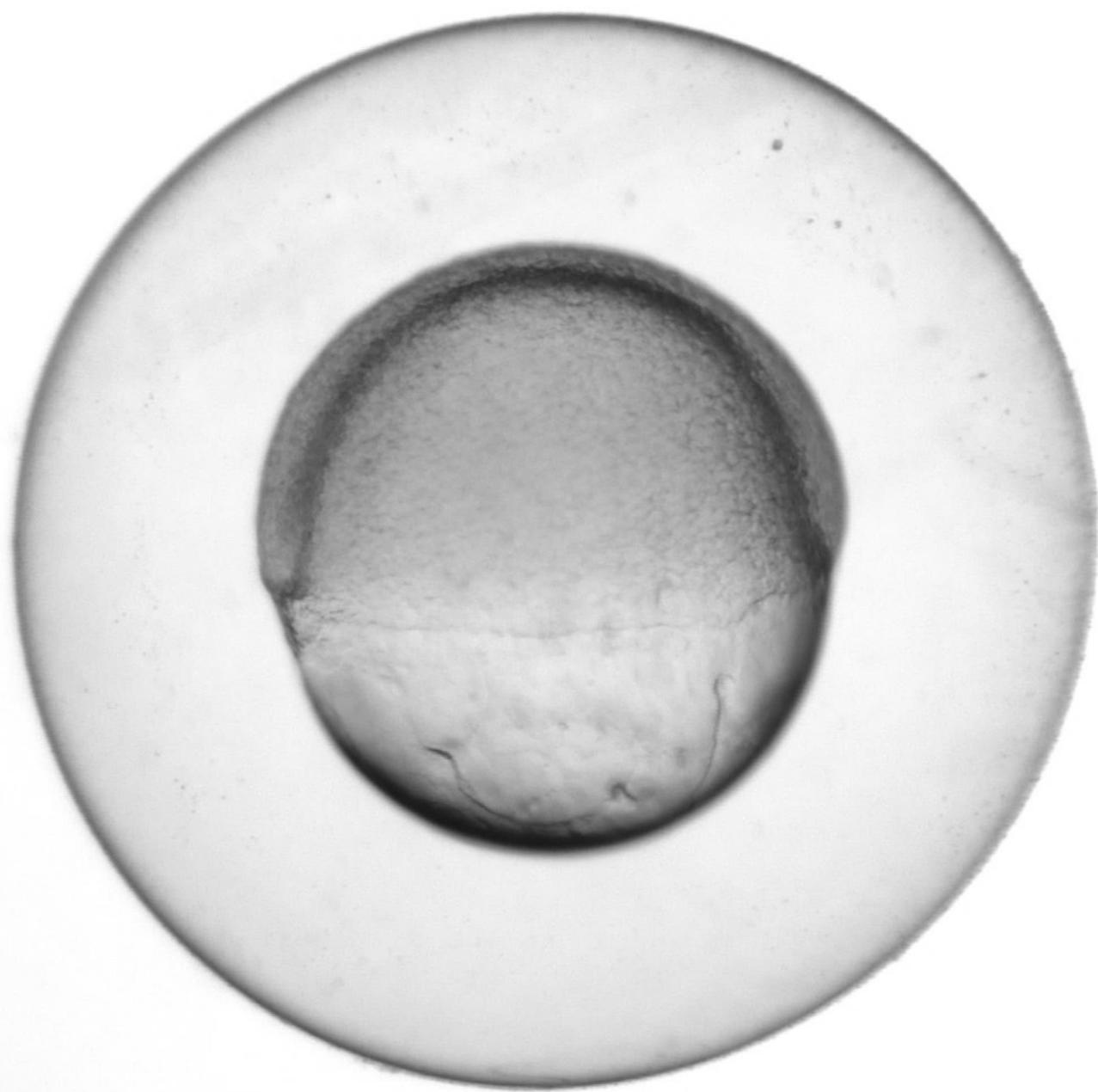

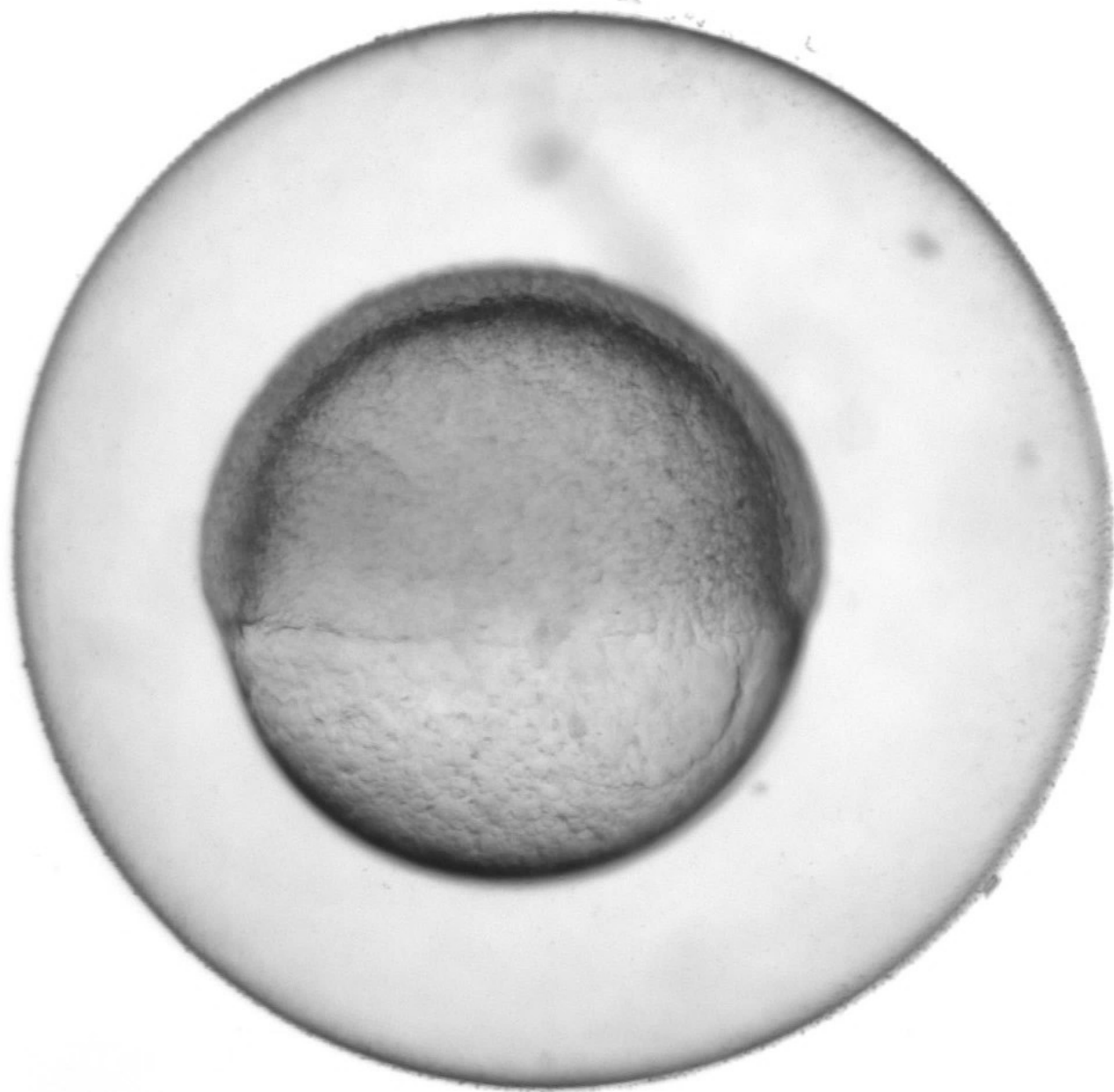

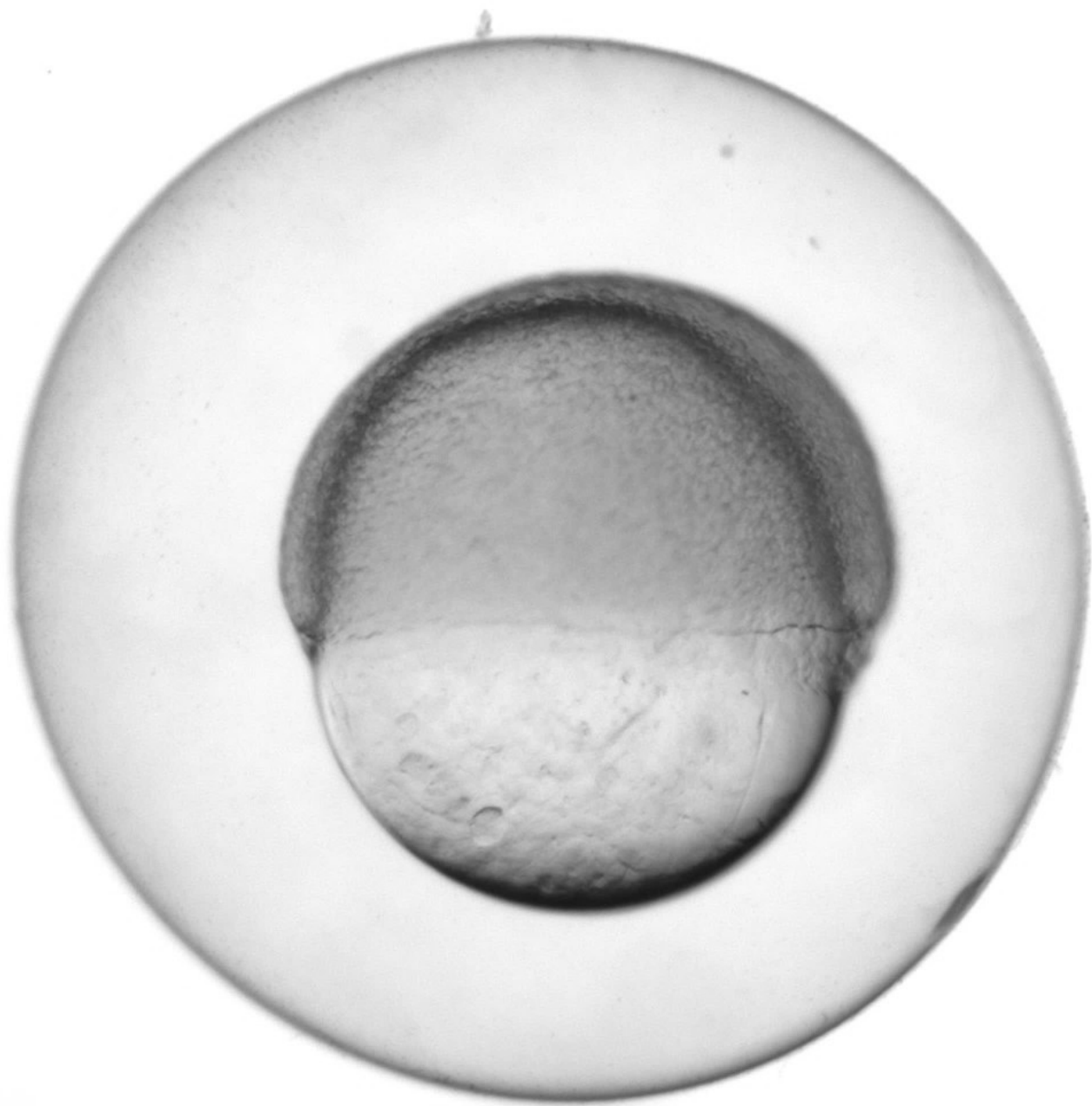

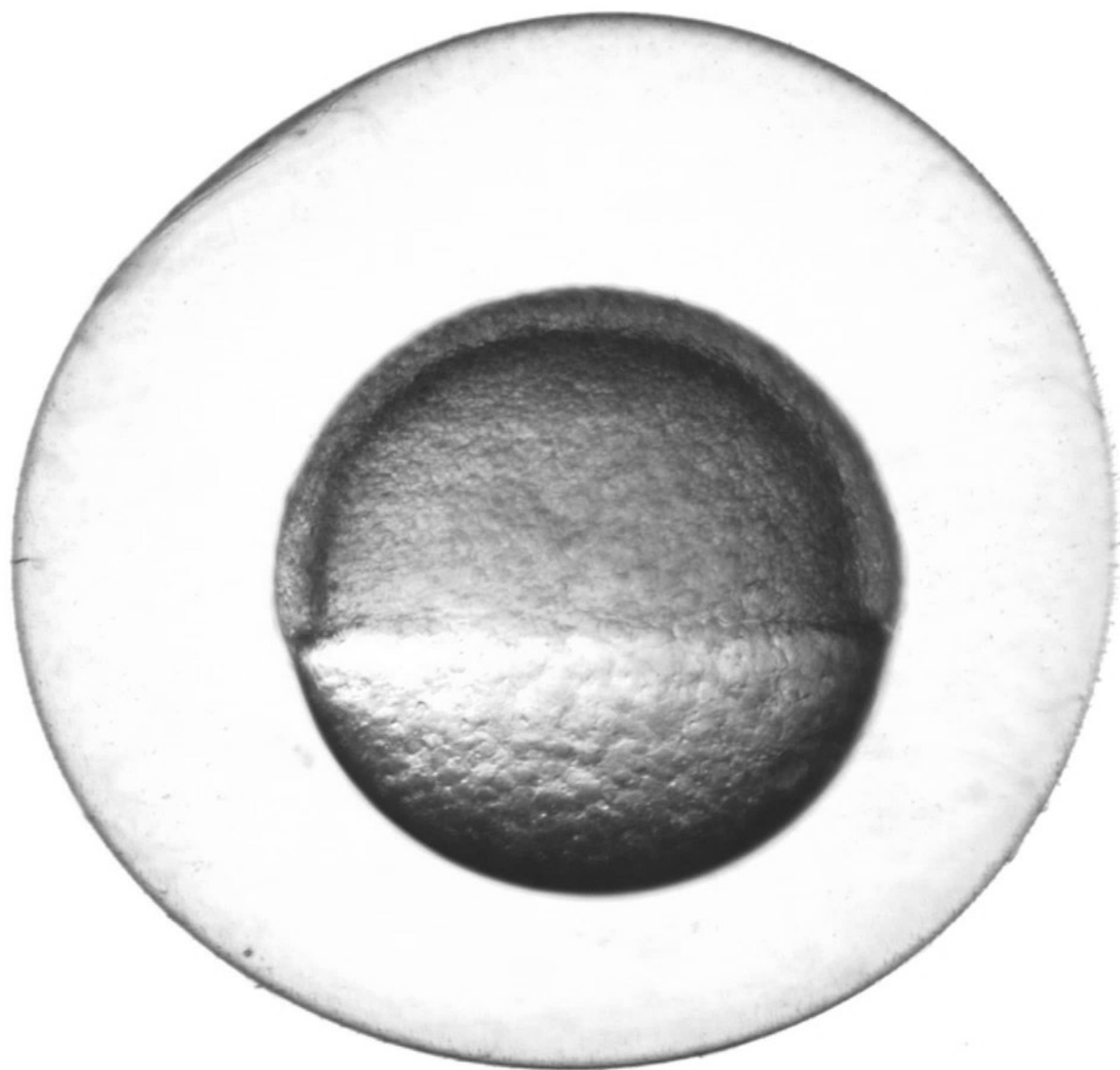

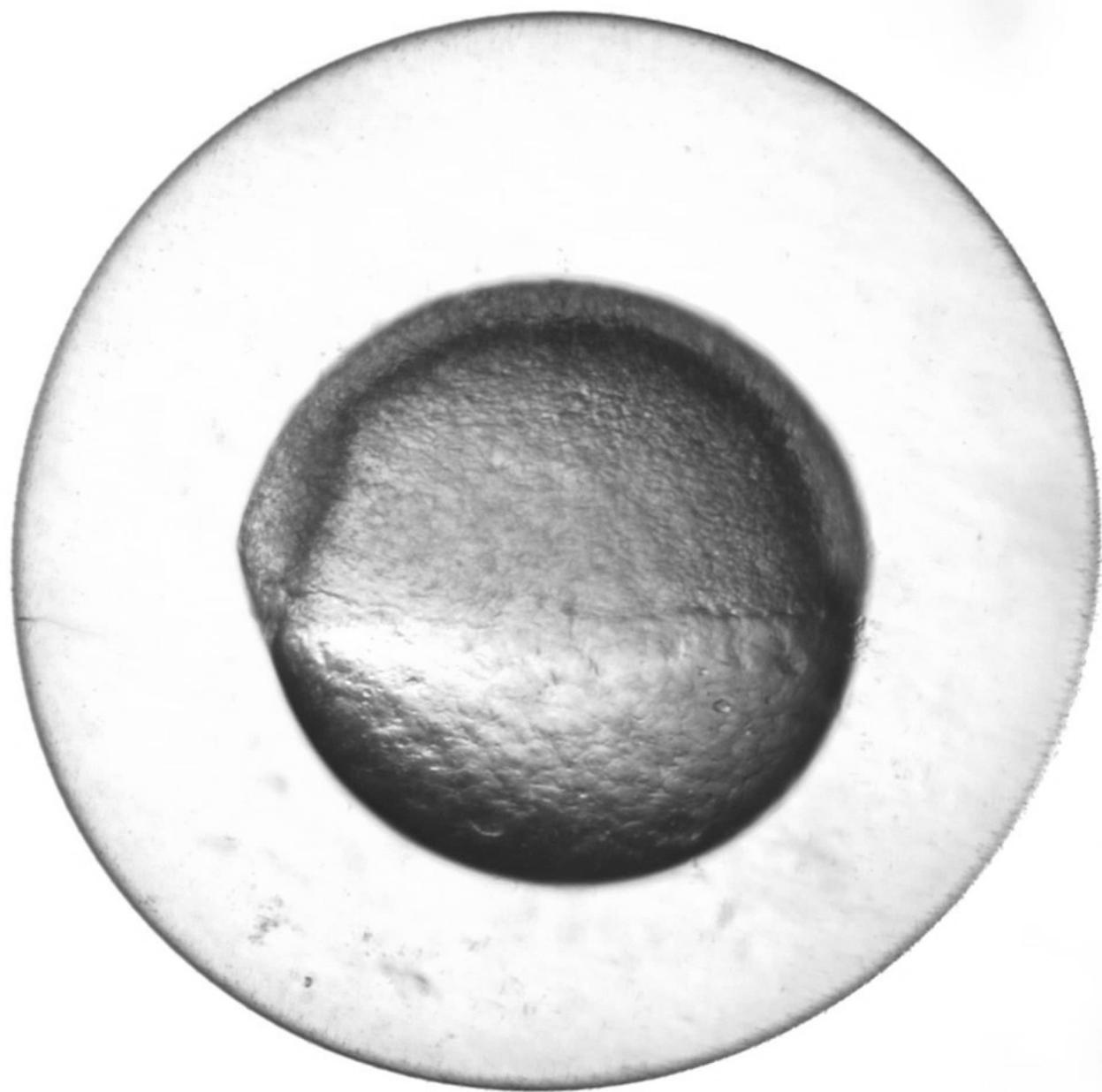

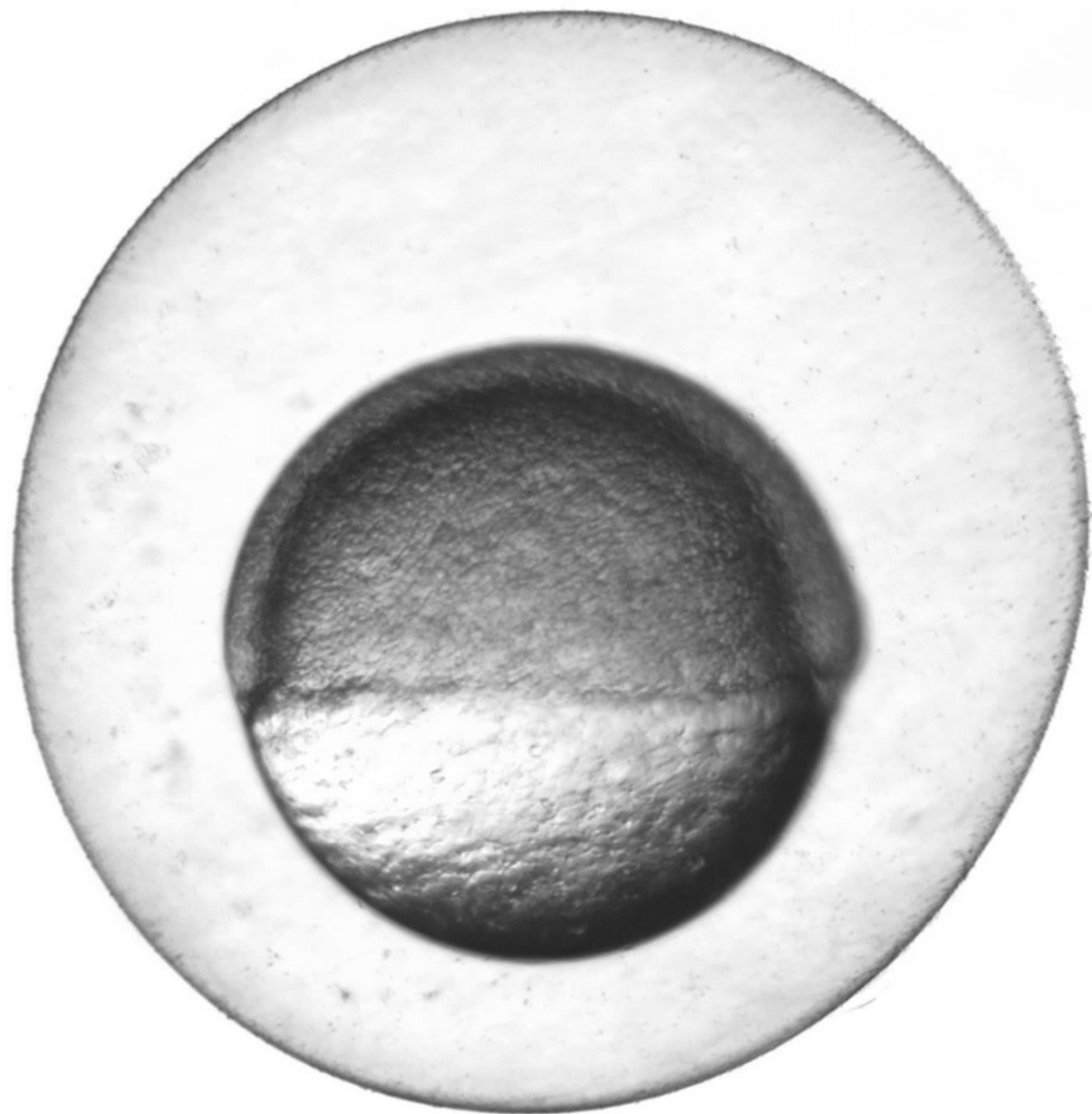

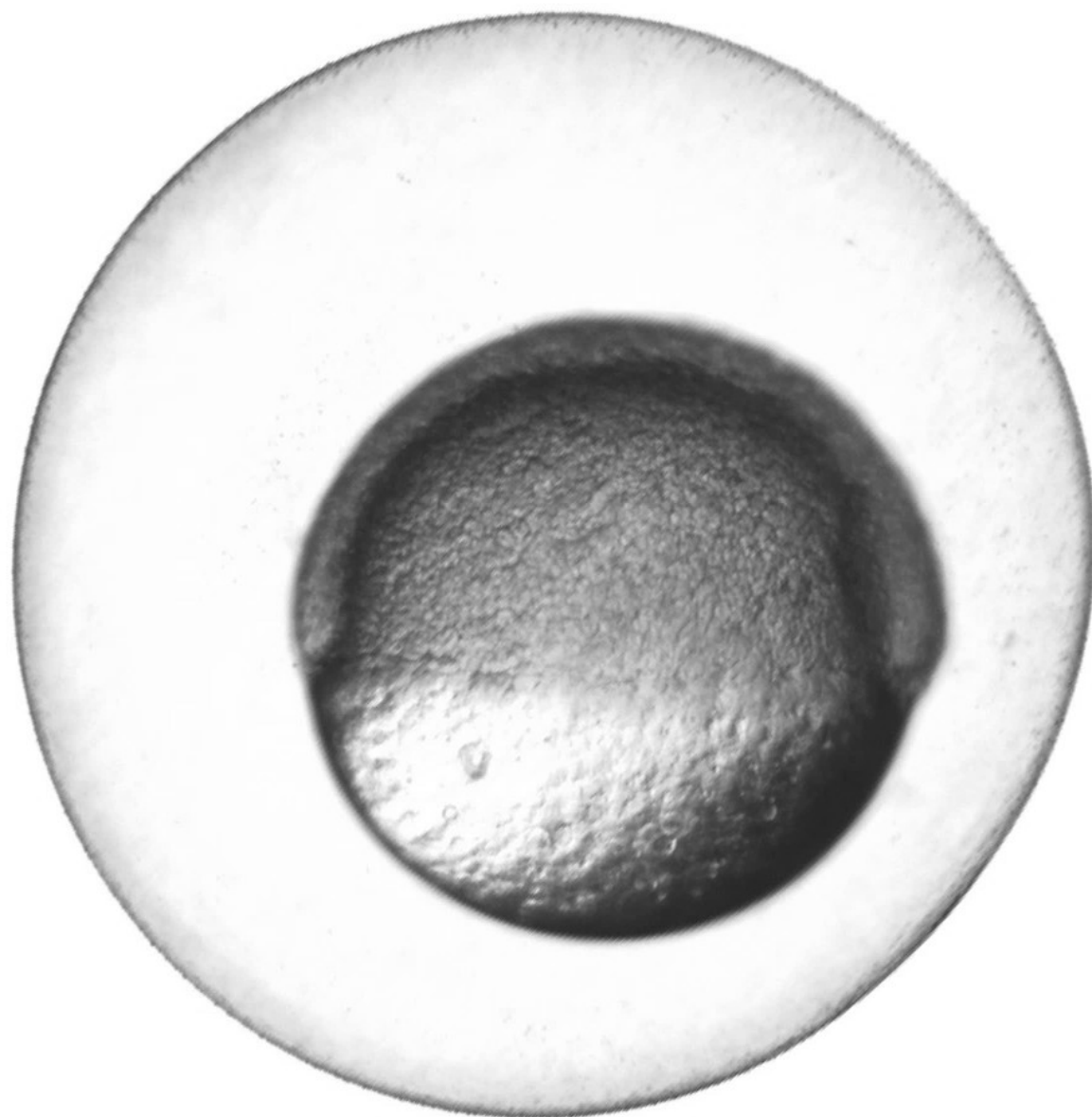

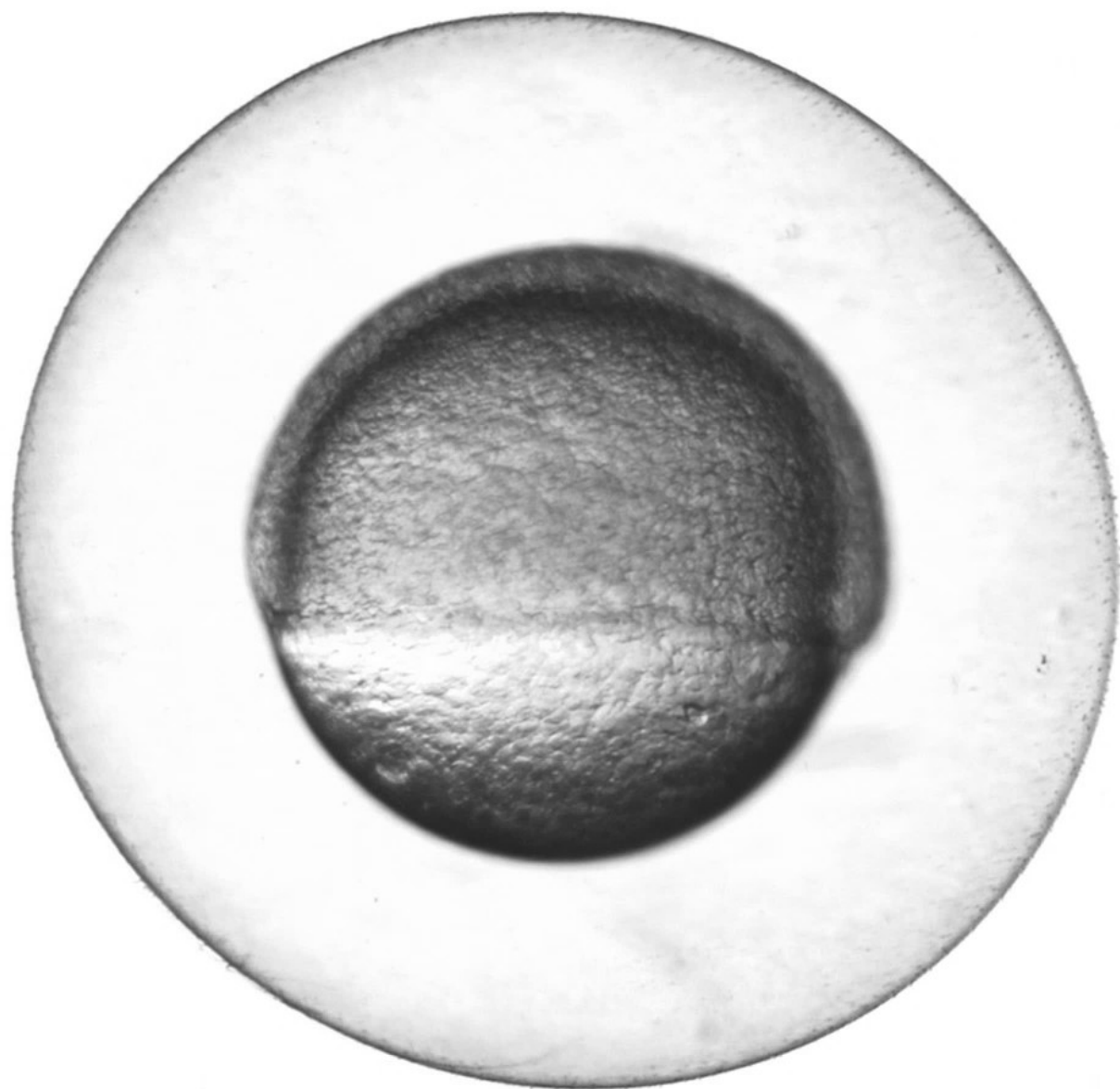

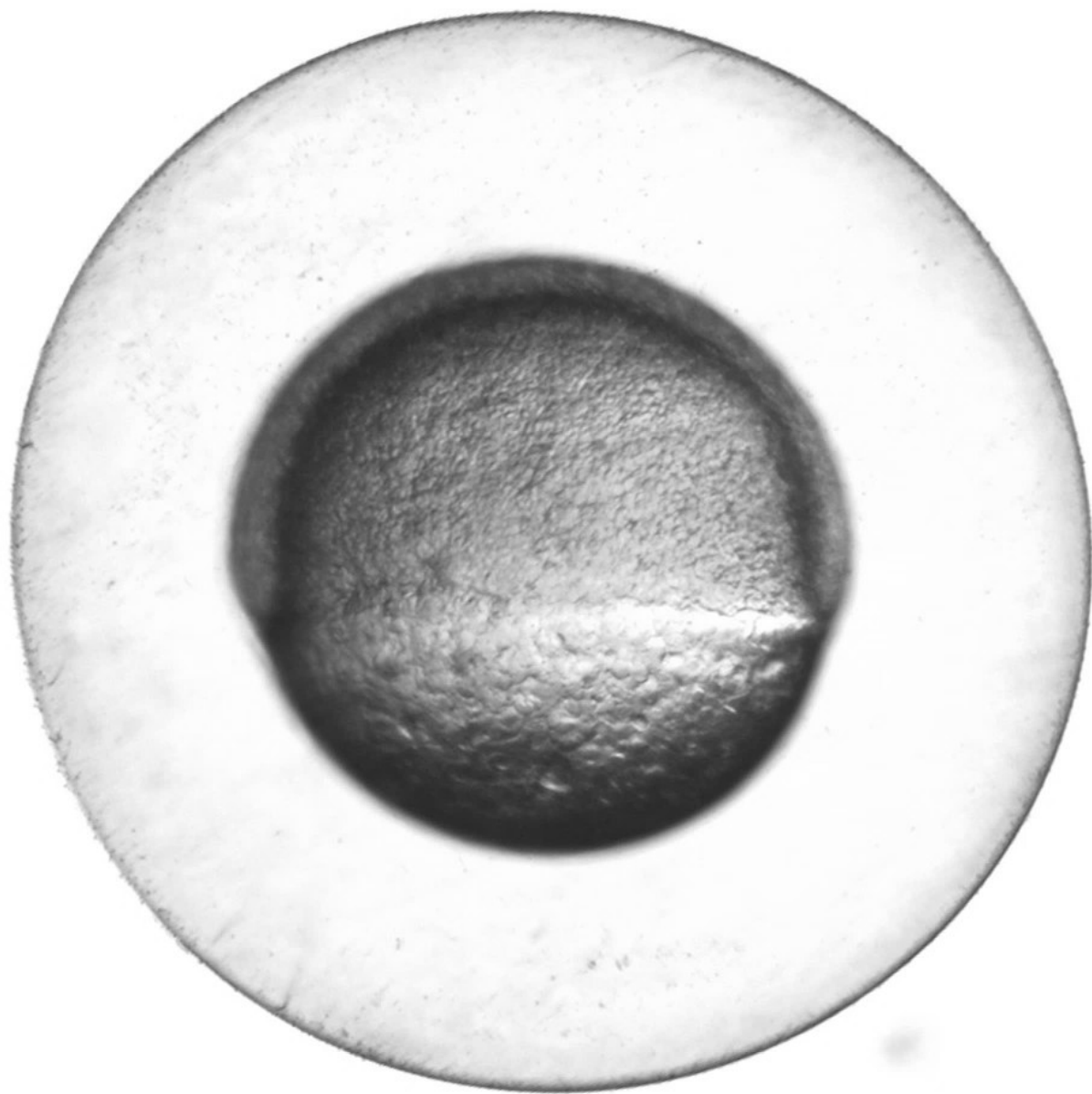

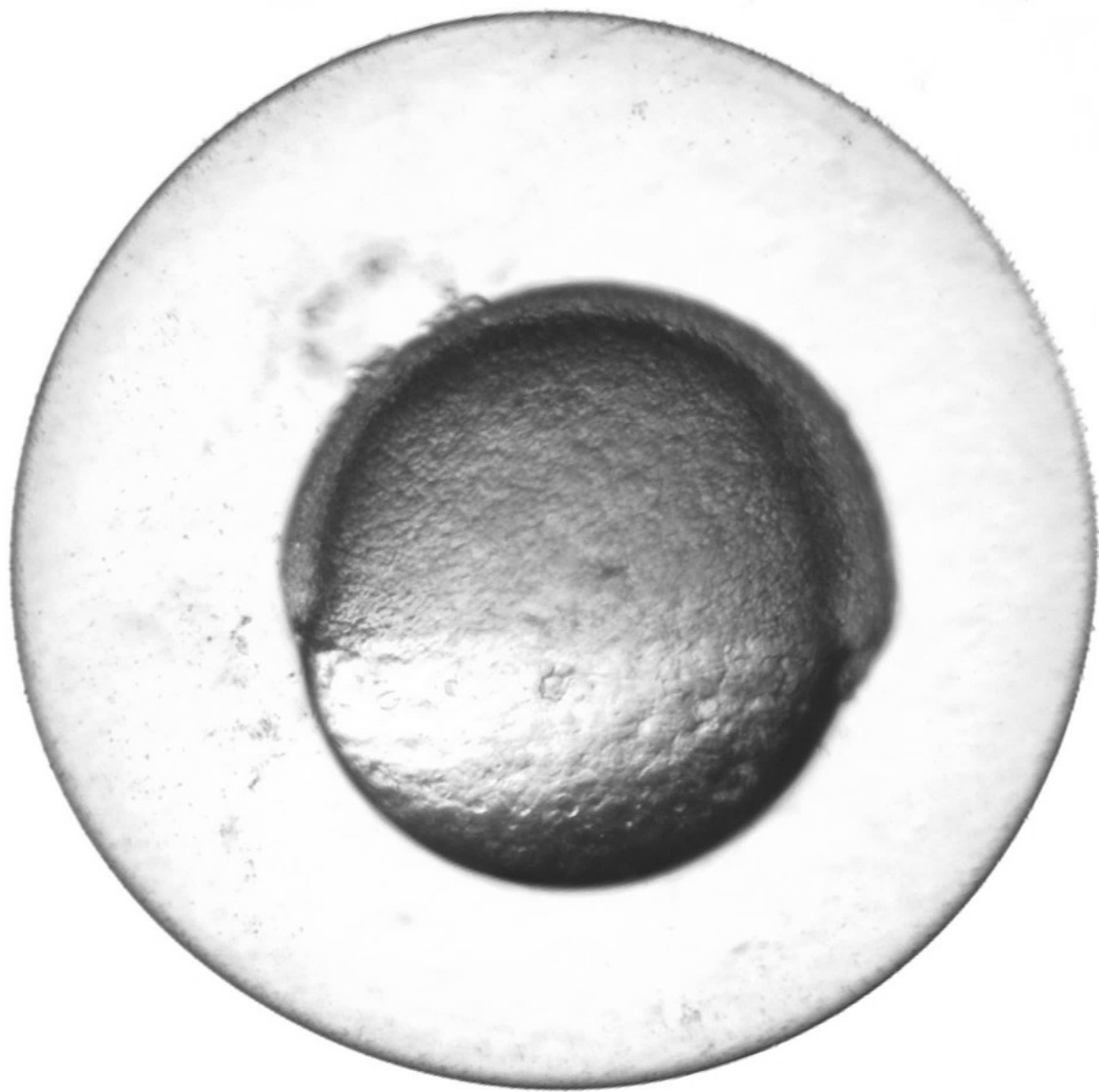

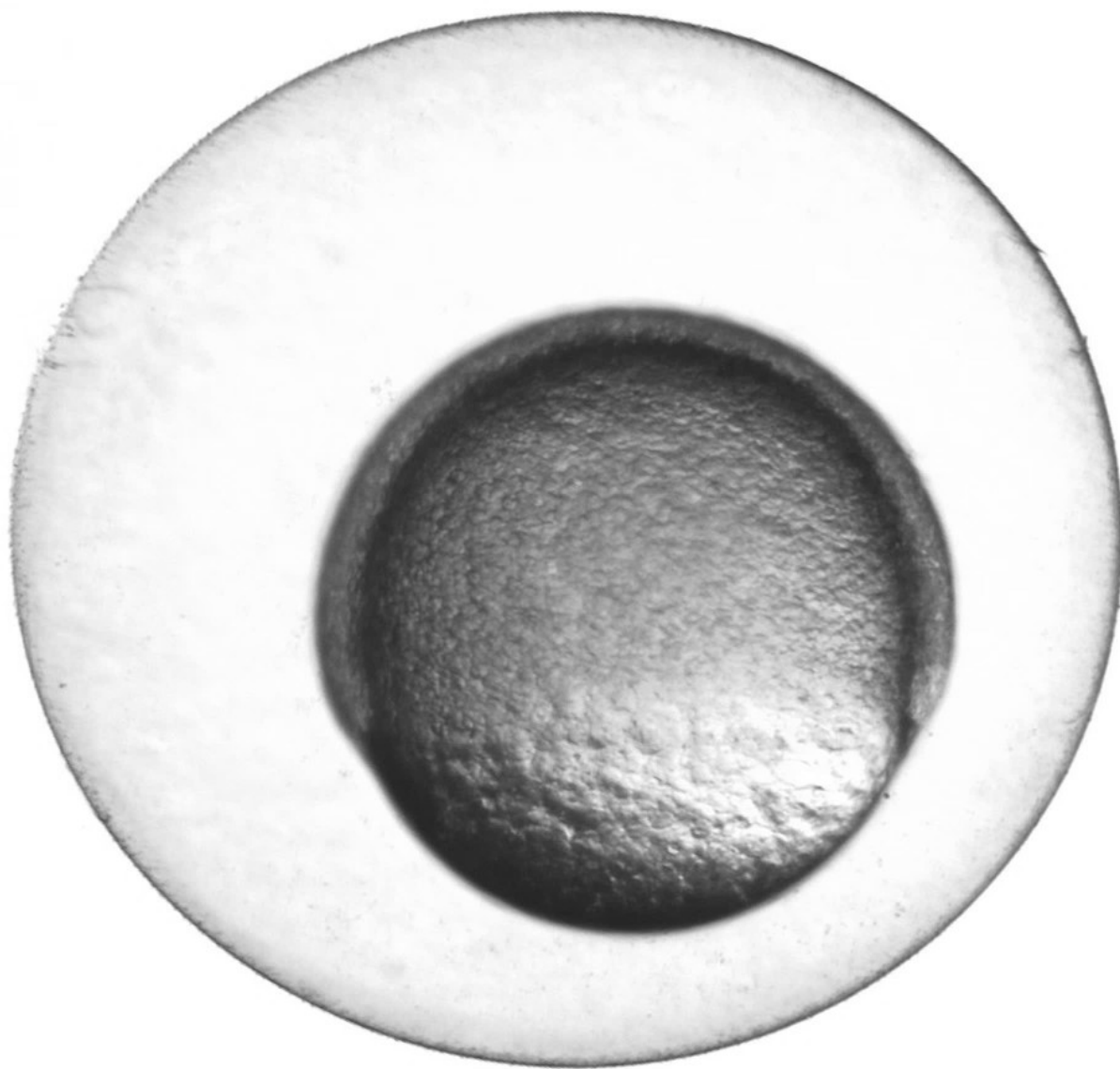

Supplement: Supplementary file 2 — Images of all 179 embryos positioned in the animal pole - vegetal pole direction. (PDF 19069 kb) [file 12864_2017_3672_MOESM2_ESM.pdf]

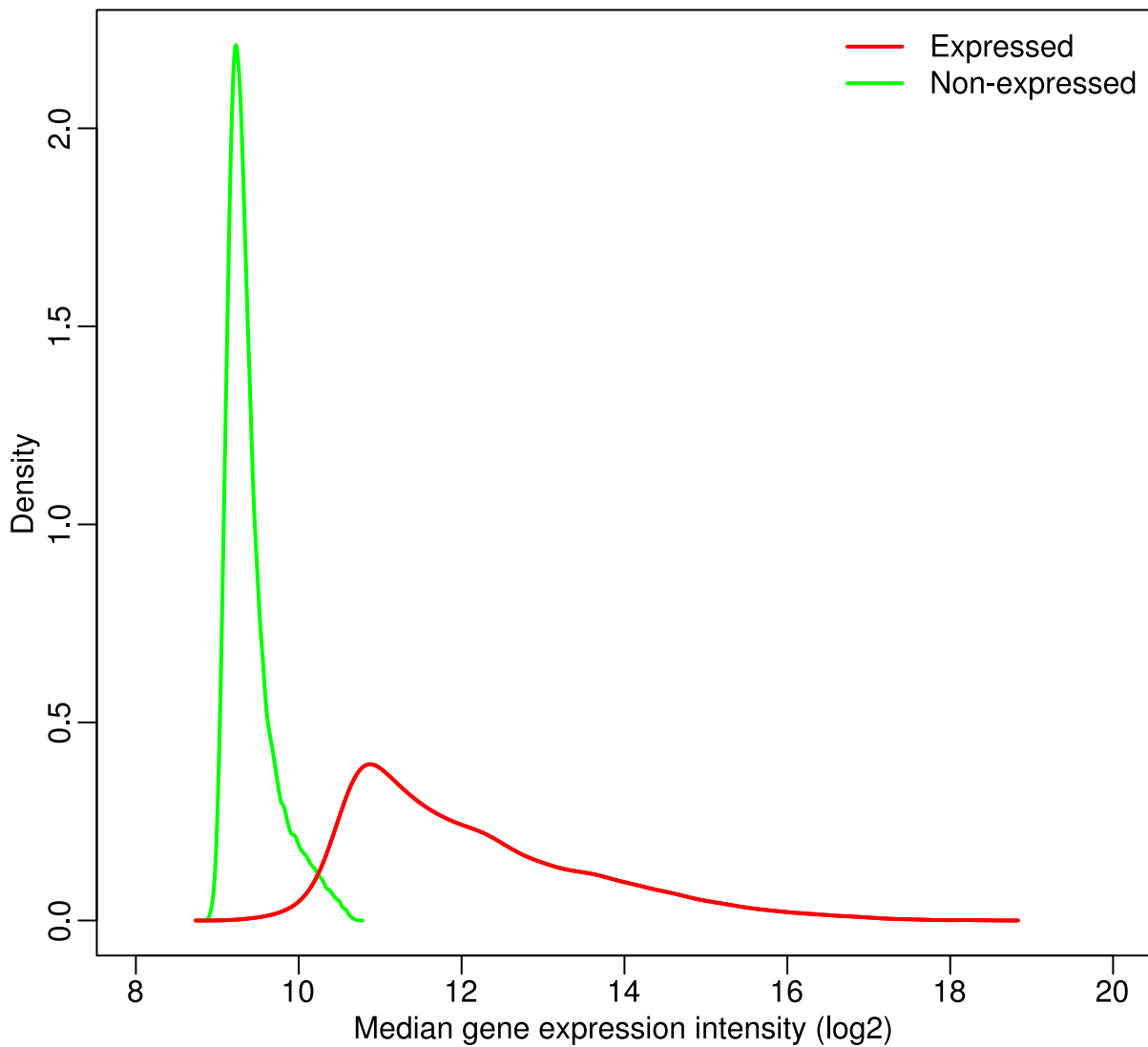

Supplement: Supplementary file 5 — Intensity distribution of the 6,734 expressed (red) and 15,938 non-expressed (green) unique Ensembl defined genes. (PDF 23 kb) [file 12864_2017_3672_MOESM5_ESM.pdf]

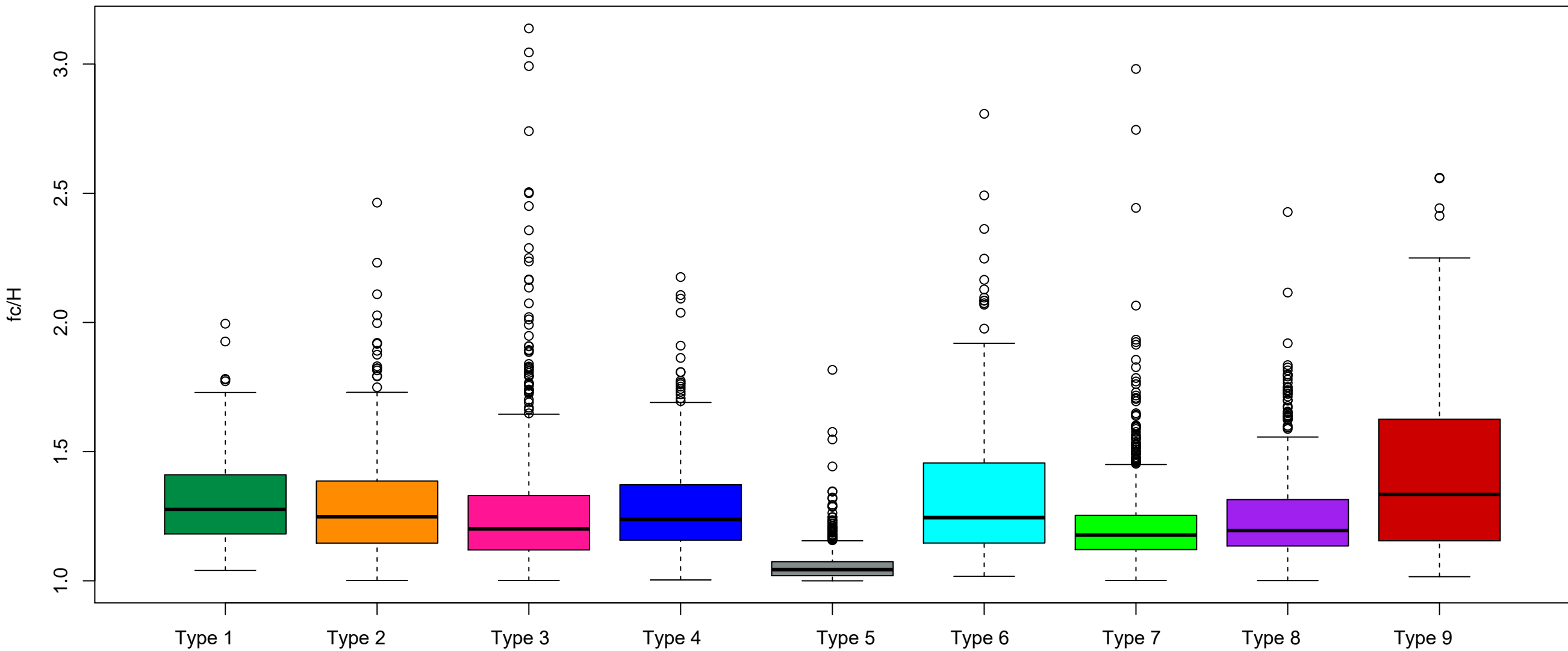

Supplement: Supplementary file 8 — Boxplots per type of absolute expression changes of genes over time, expressed as fold changes per hour. (PDF 37 kb) [file 12864_2017_3672_MOESM8_ESM.pdf]

A

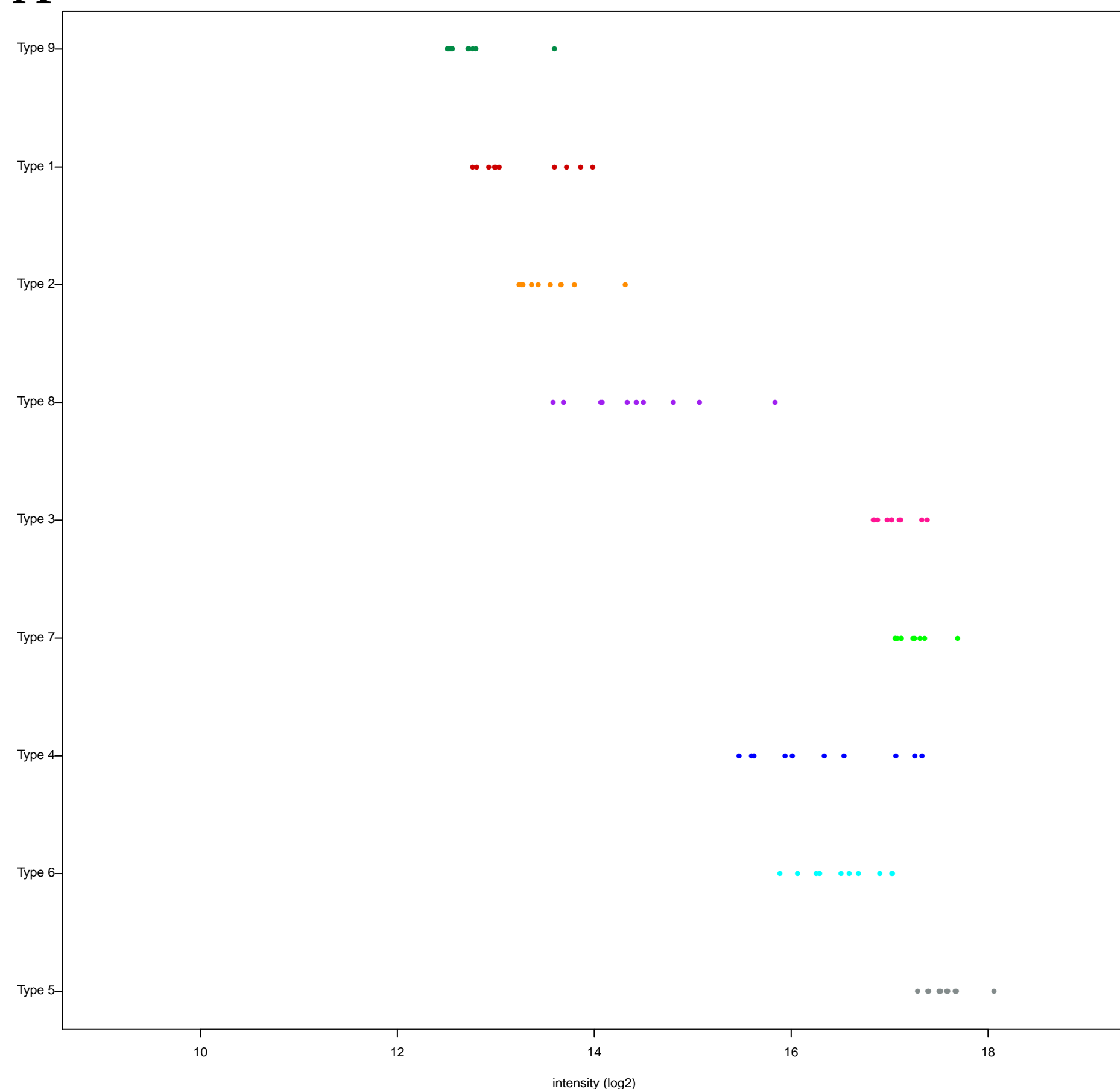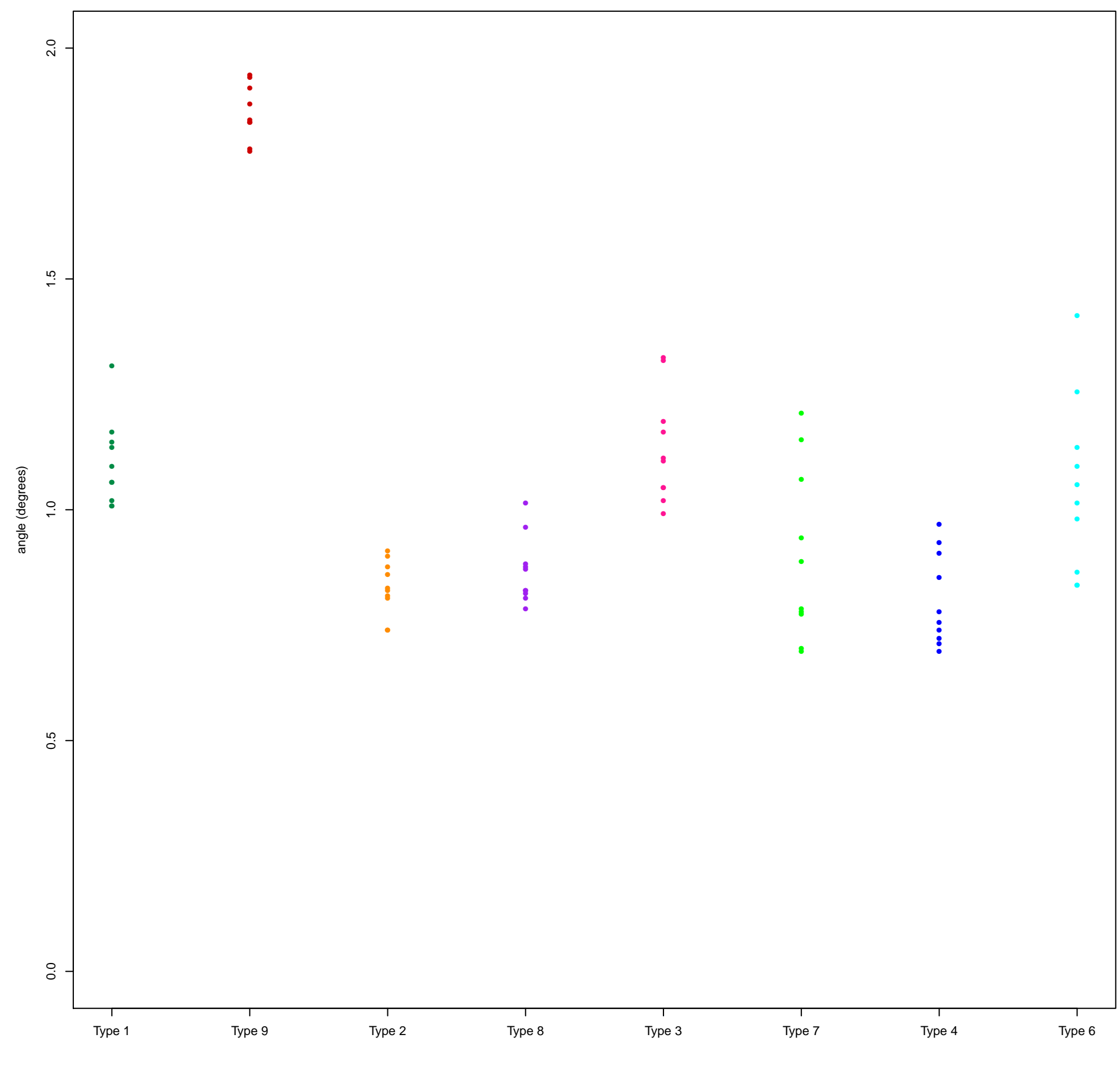

B

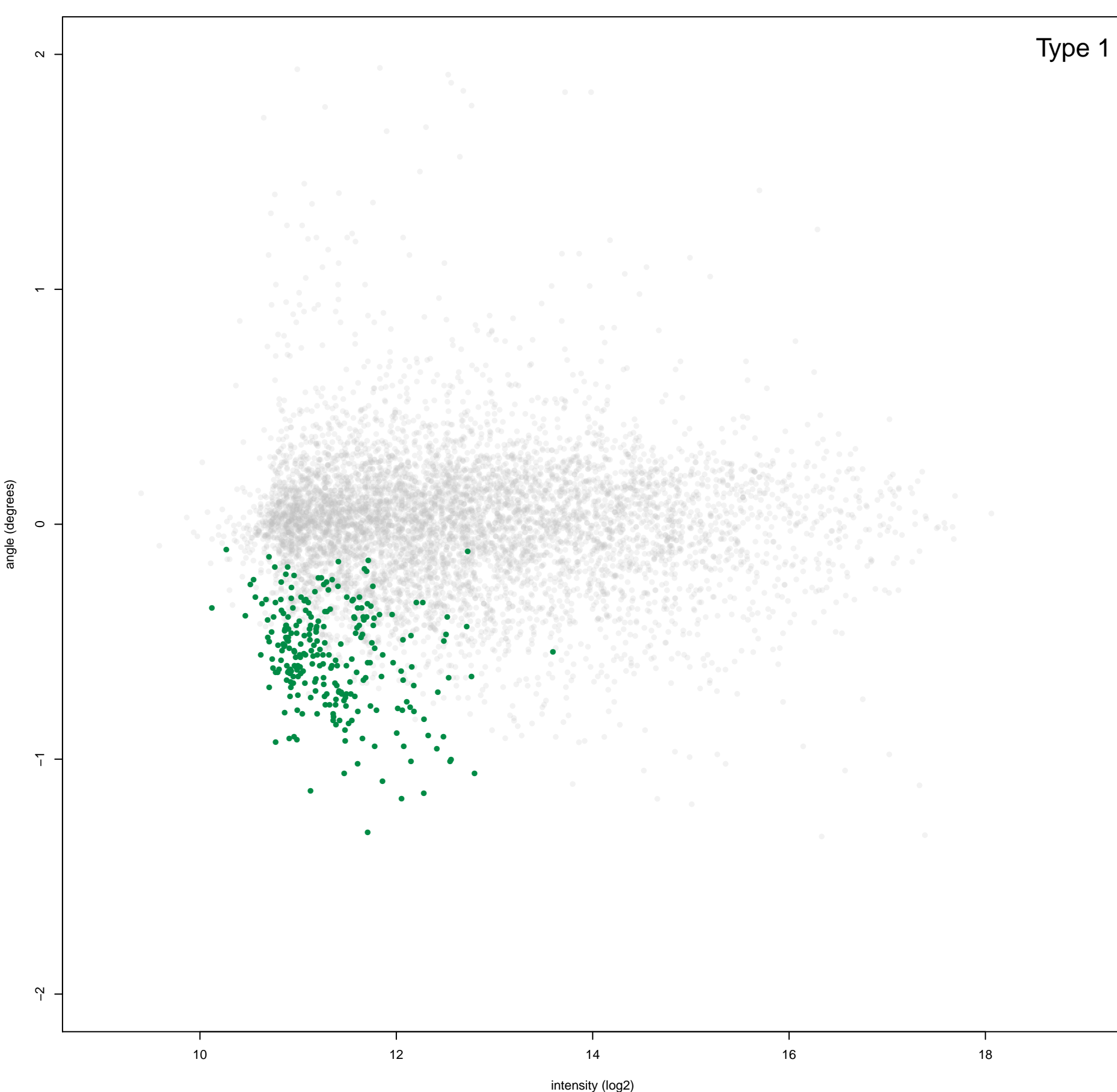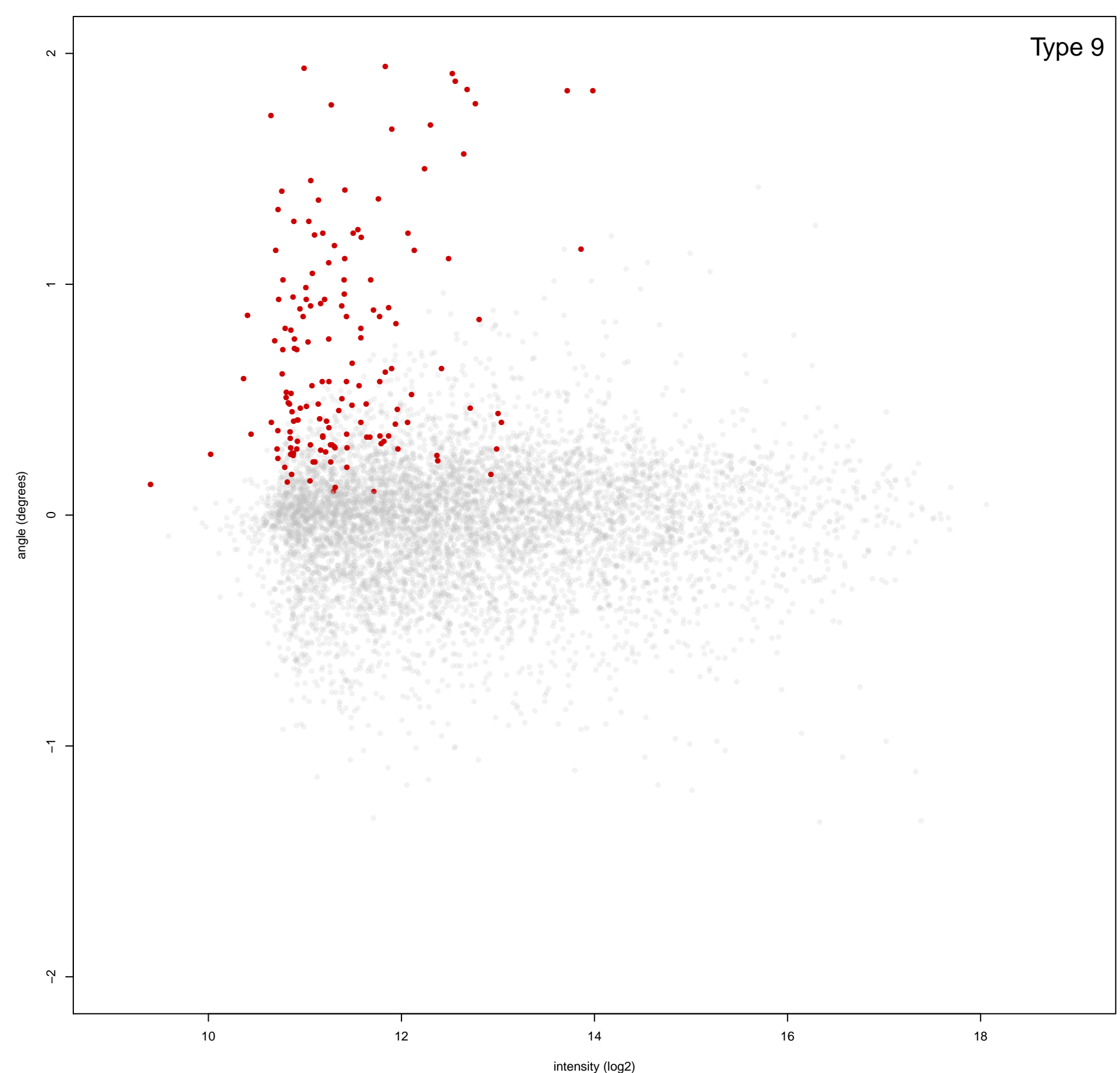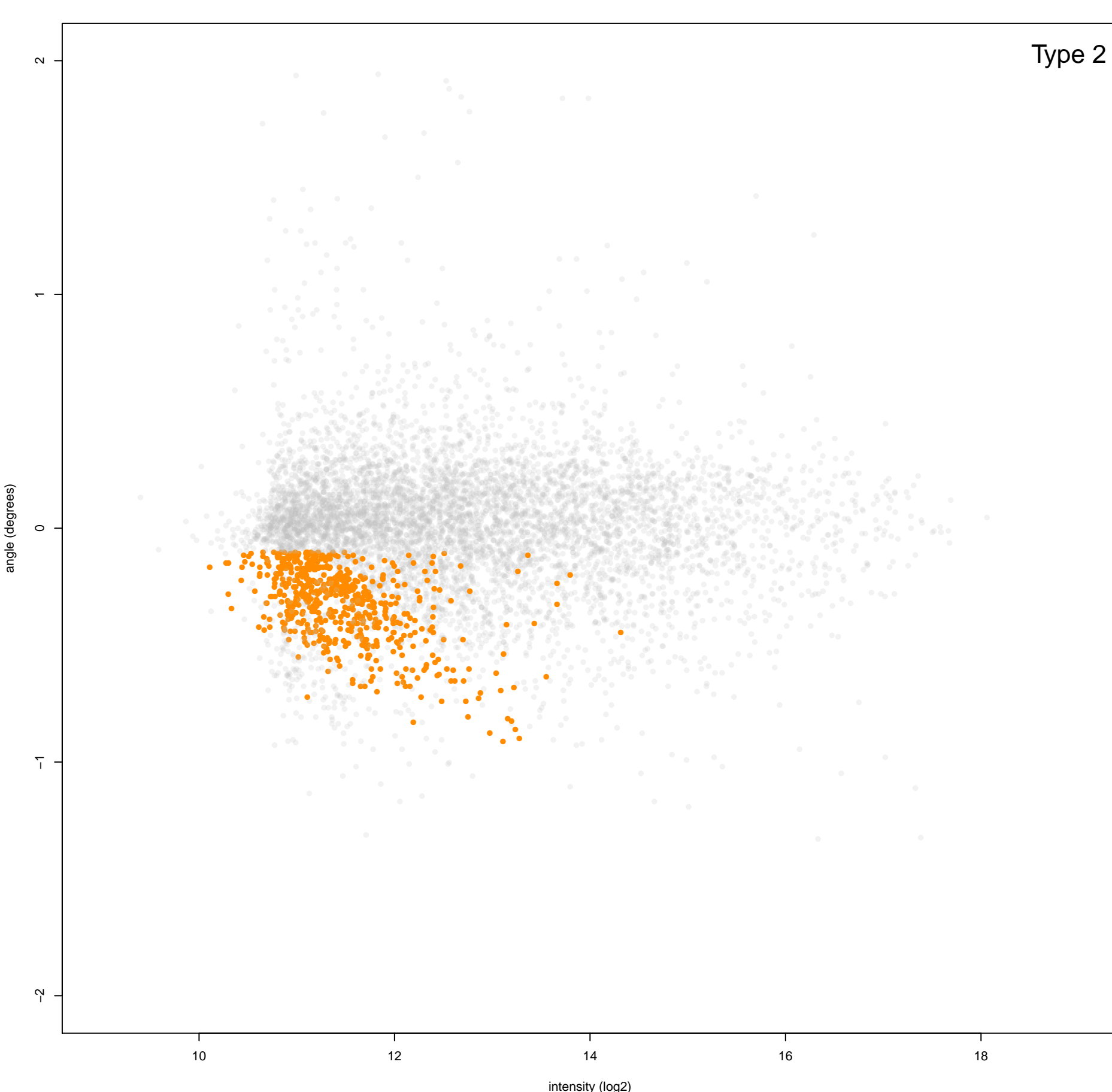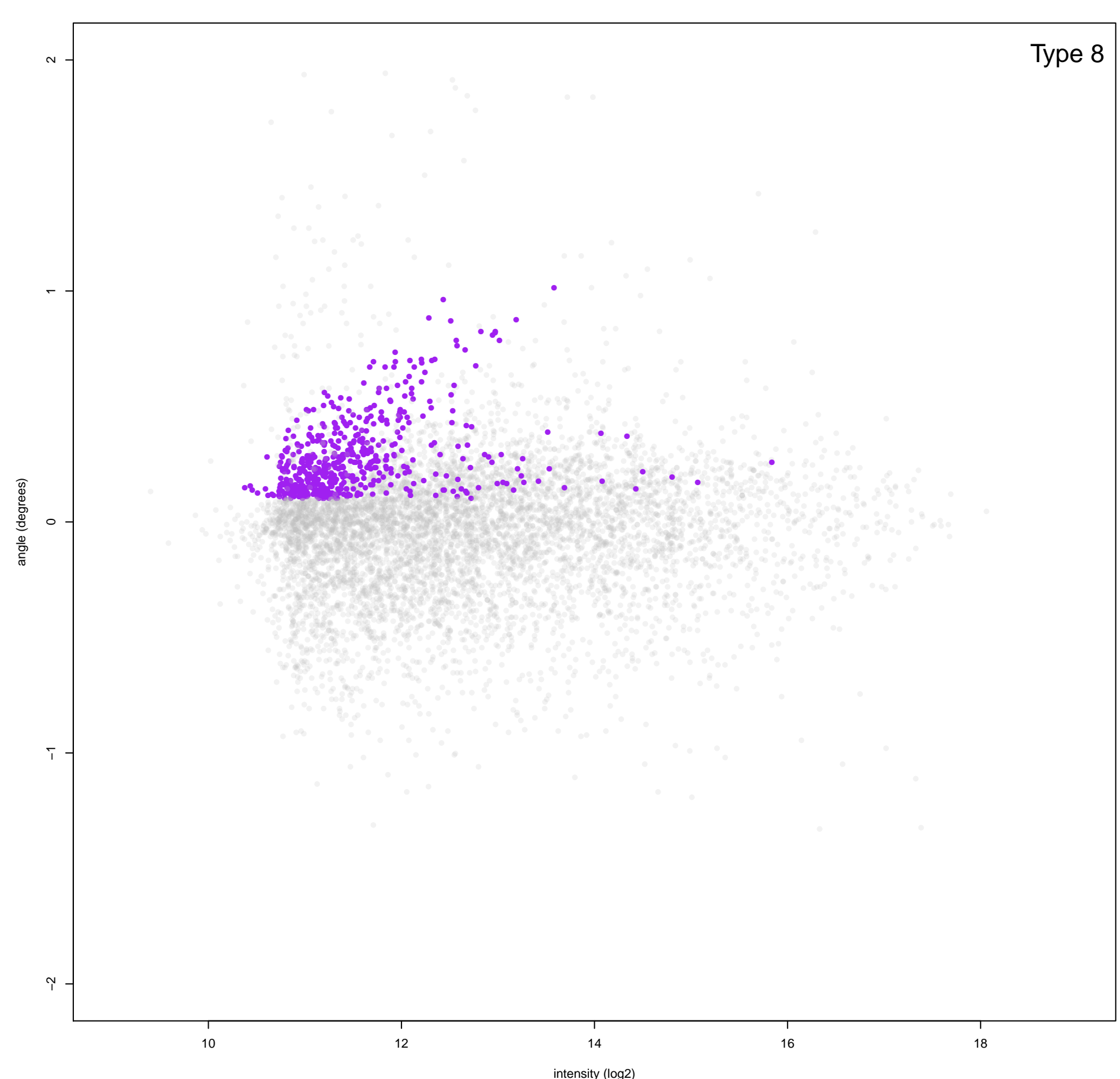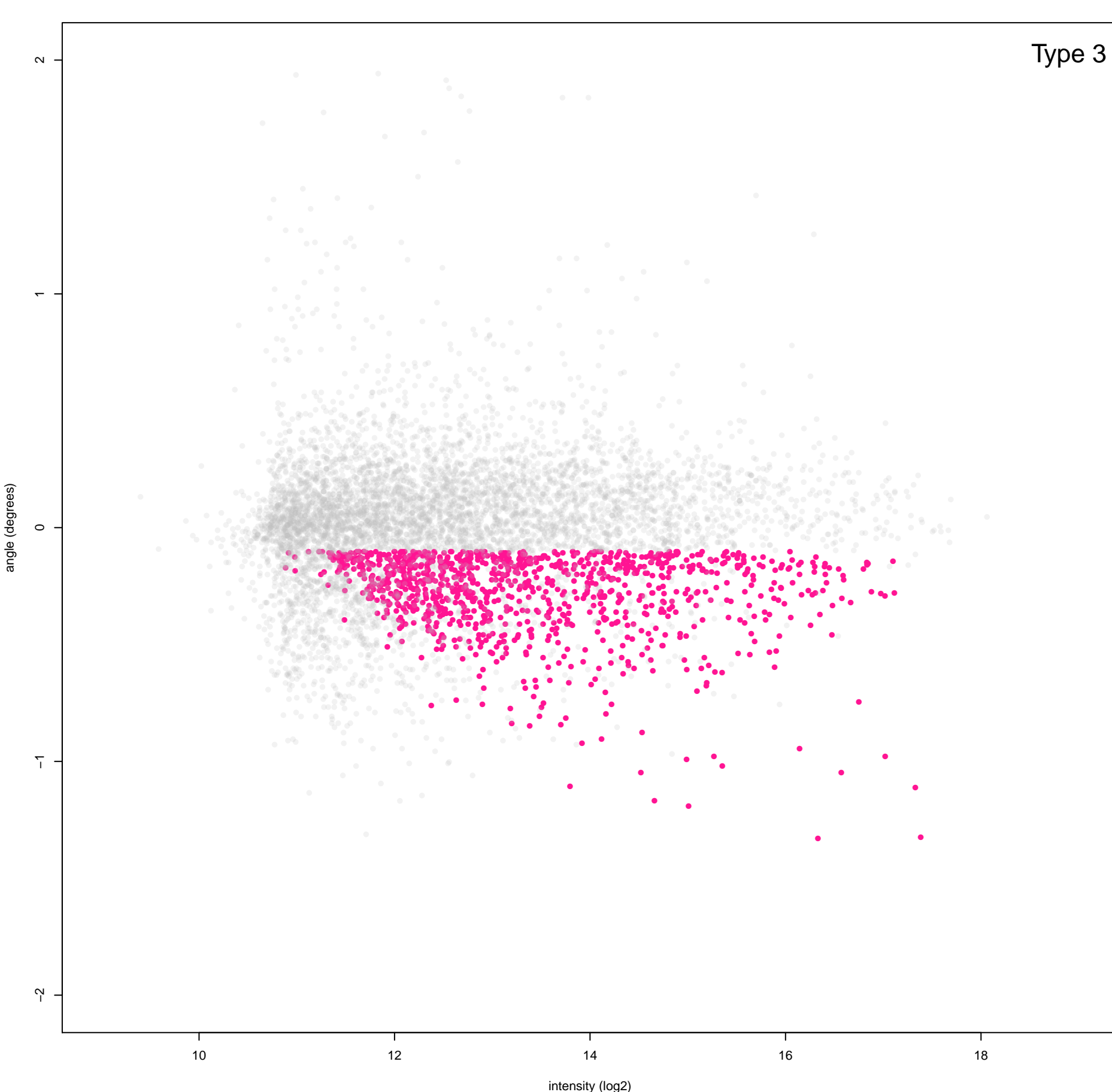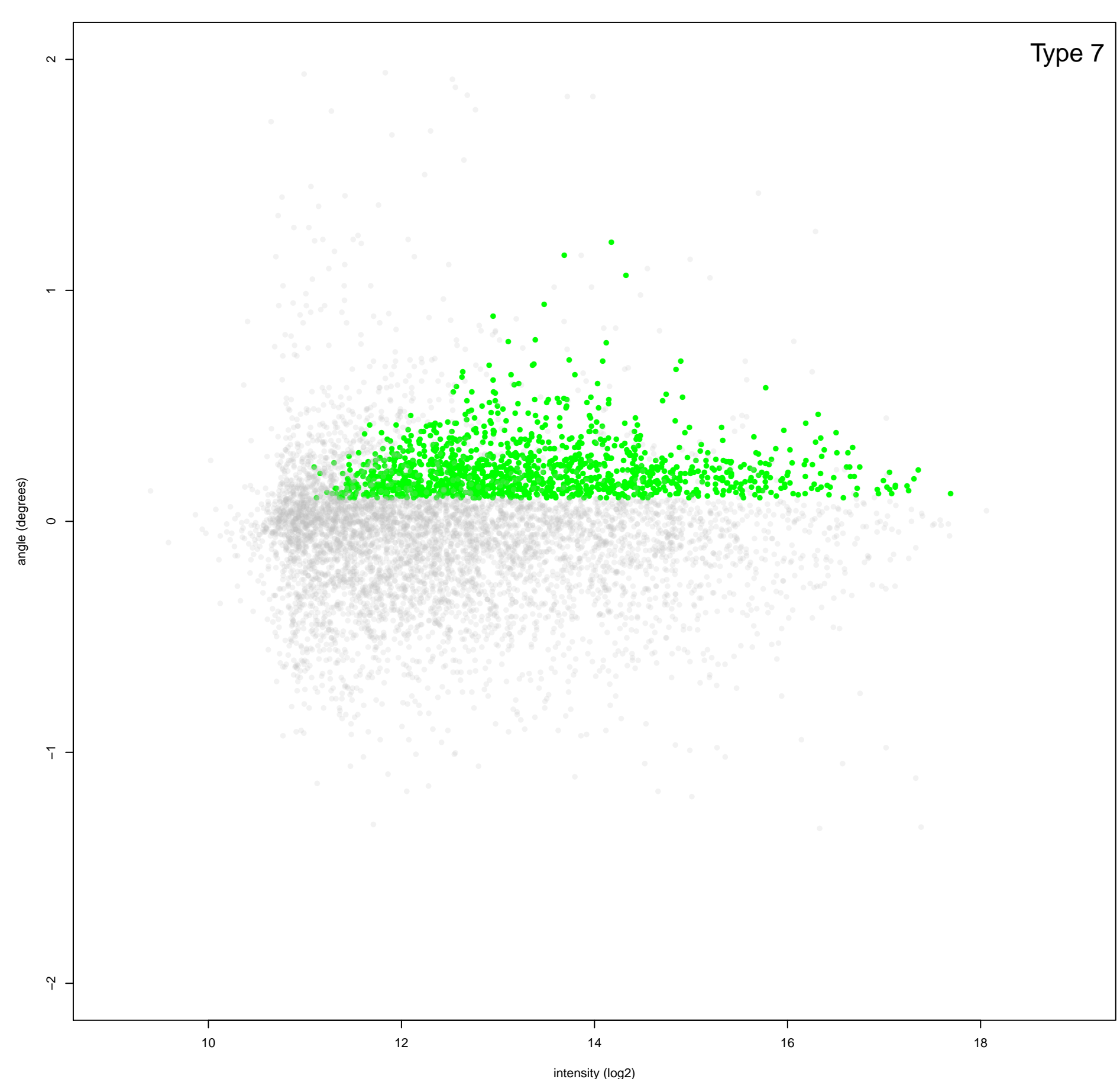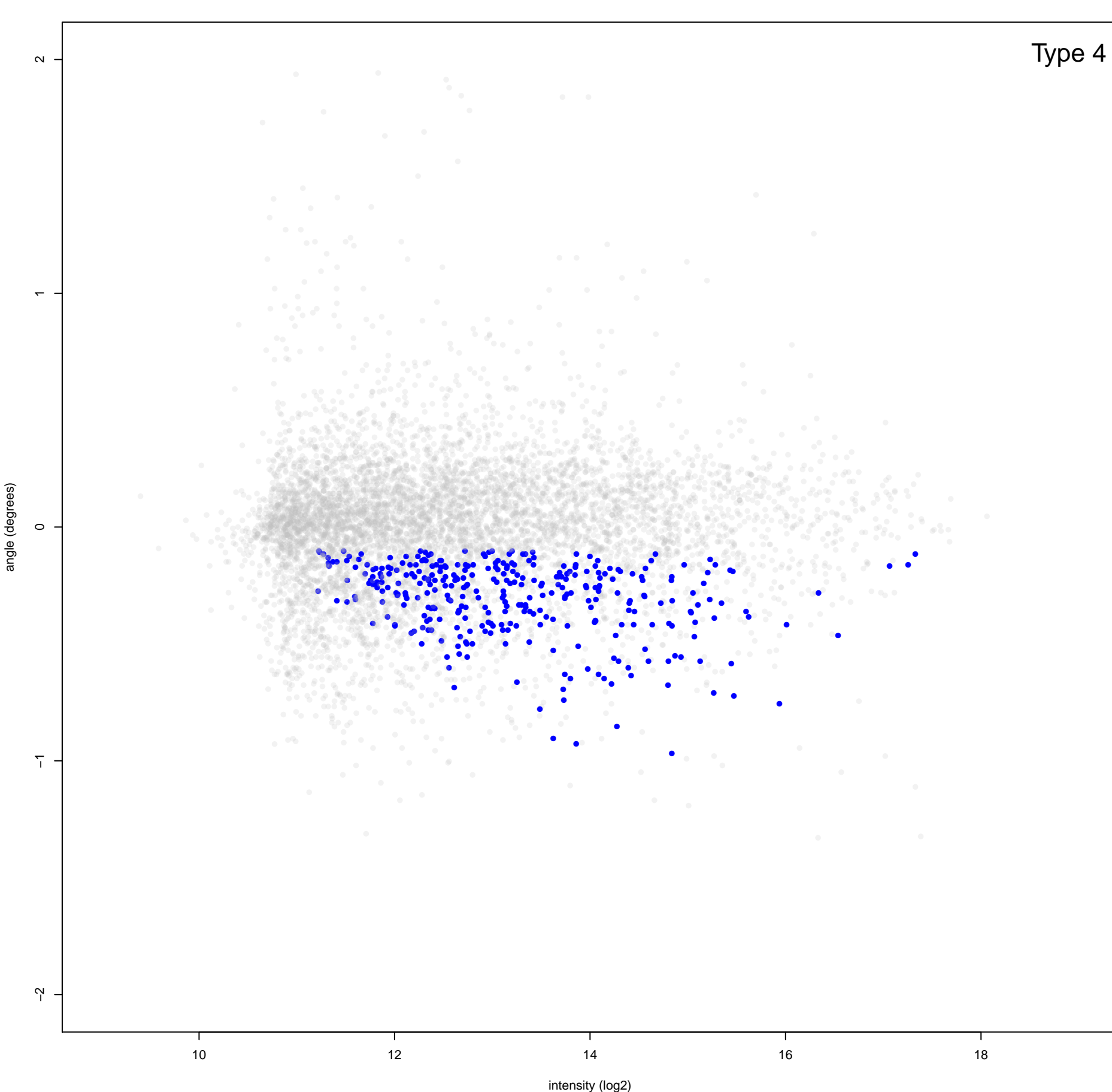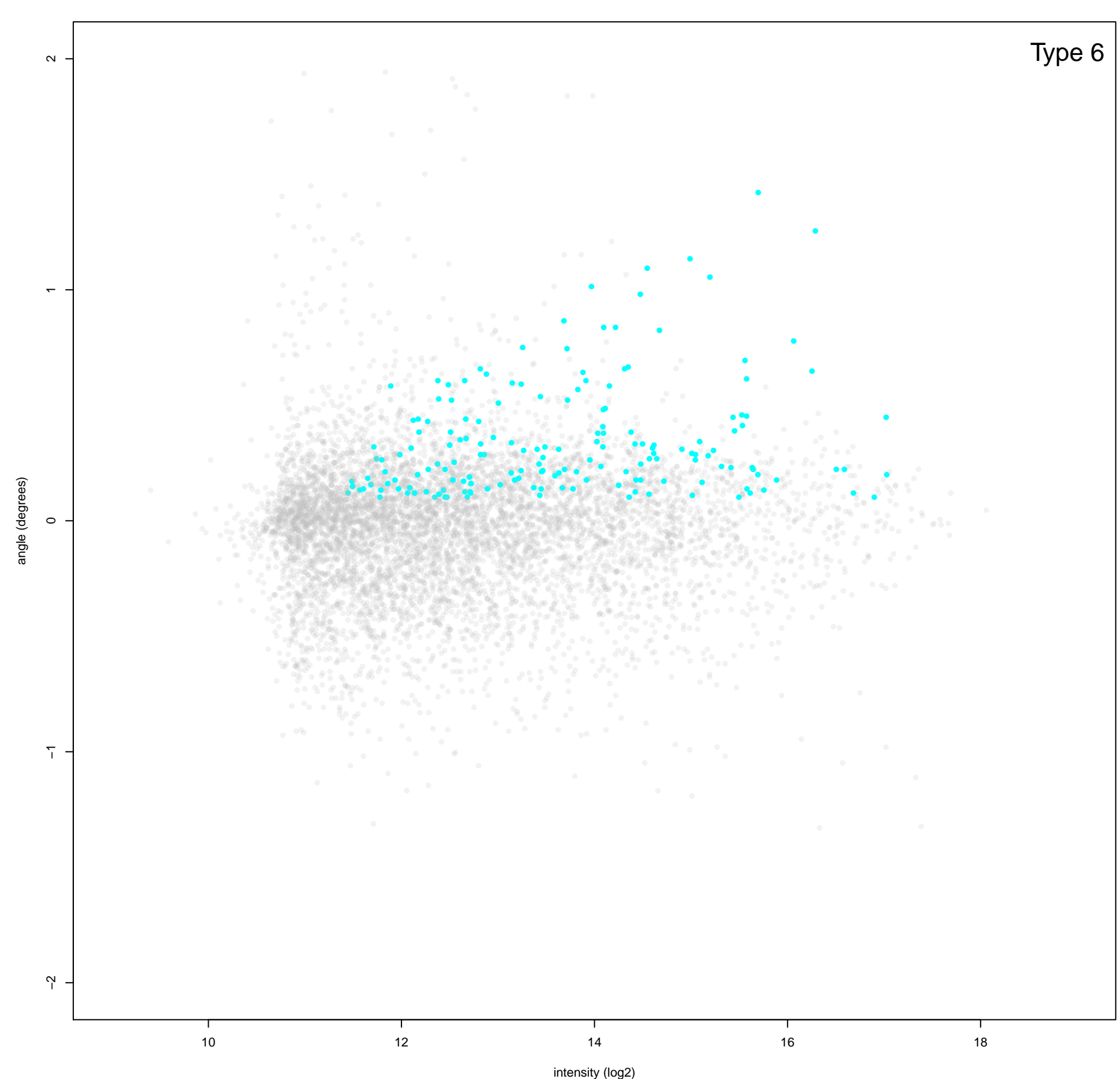

Supplement: Supplementary file 9 — Intensity distributions per type. A) Left panel: top 10 highest gene expression intensities per type. Right panel: top 10 genes with the largest gene expression rates (expressed in angles) type. B) Scatterplot of gene expression rates, expressed as angles versus intensity. In each plot the genes belonging to the type are highlighted. (PDF 520 kb) [file 12864_2017_3672_MOESM9_ESM.pdf]

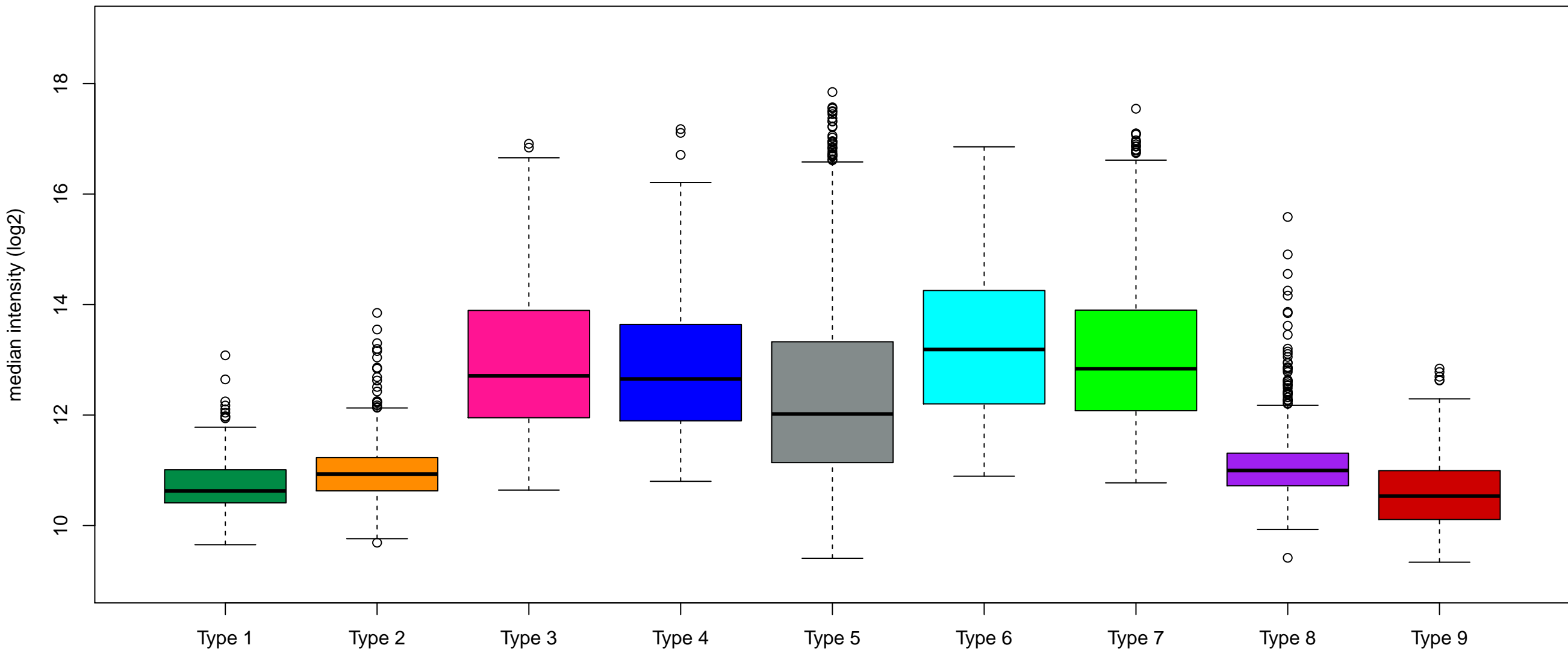

Supplement: Supplementary file 10 — Boxplots of median expression intensities per type. (PDF 11 kb) [file 12864_2017_3672_MOESM10_ESM.pdf]

A

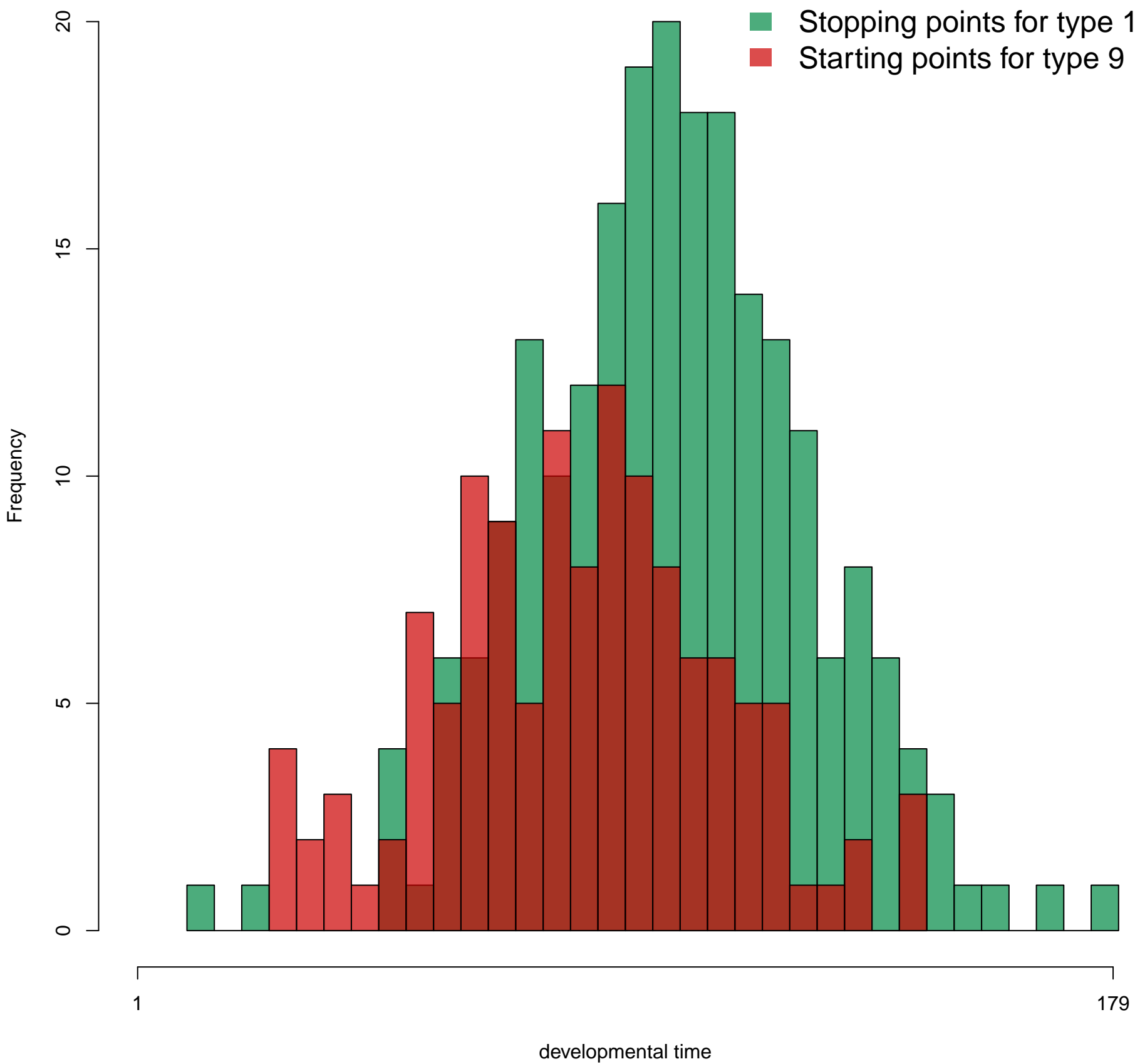

B

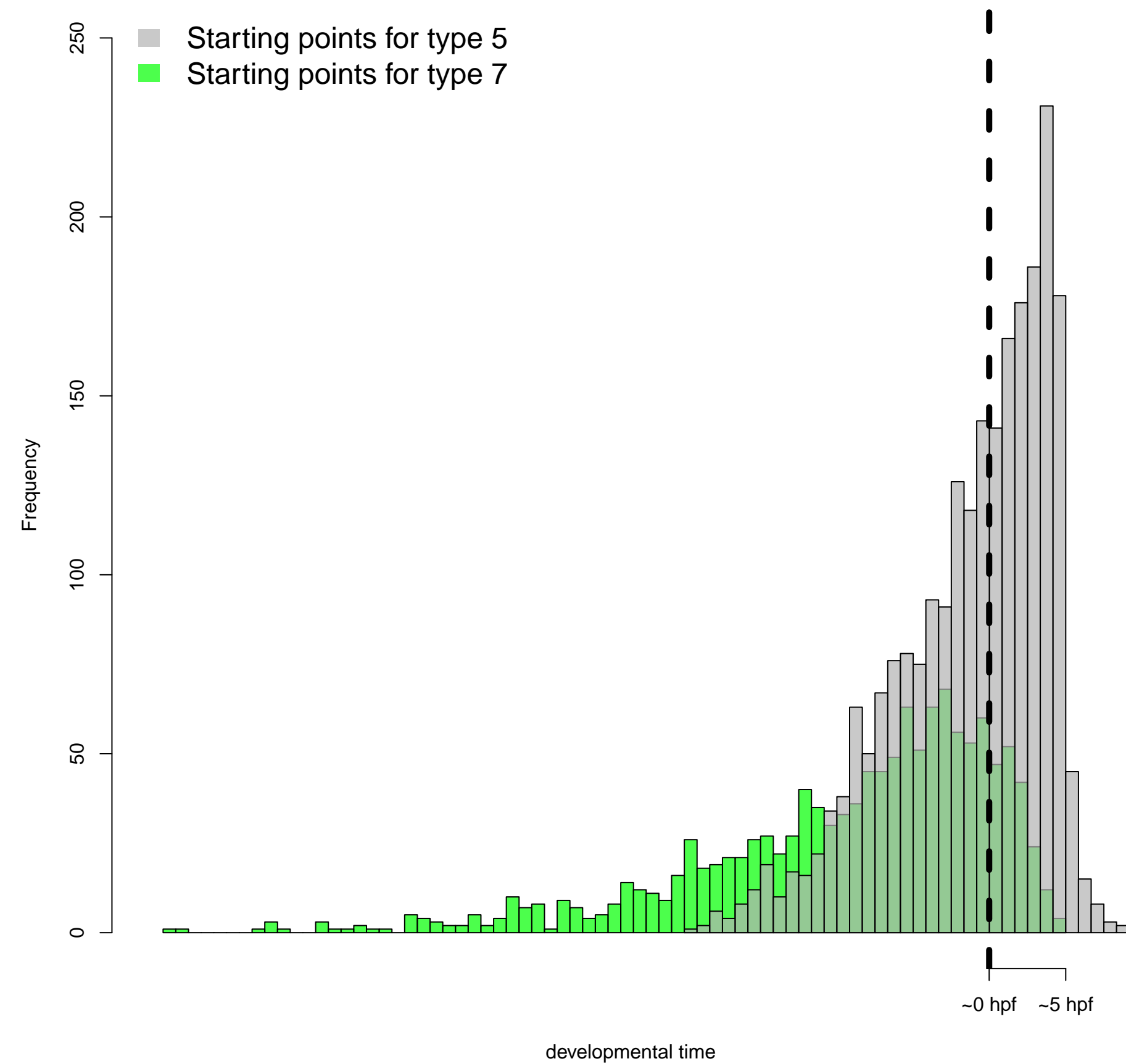

Supplement: Supplementary file 11 — Starting and stopping points. A) Histogram of starting and stopping points in developmental time for genes belonging to type 1 and 9. B) Histogram of extrapolated starting points for type 5 and 7 genes. For type 7 genes the starting point was calculated from the observed expression rate for each gene. For type 5 genes we took the maximum positive expression rate we found in this experiment (2.98 FC/hr for ENSDARG00000095866) to calculate the starting point. The approximate time of spawning is indicated by a dashed line, the start of the time course in this experiment lies to the right of 5 hpf. (PDF 24 kb) [file 12864_2017_3672_MOESM11_ESM.pdf]

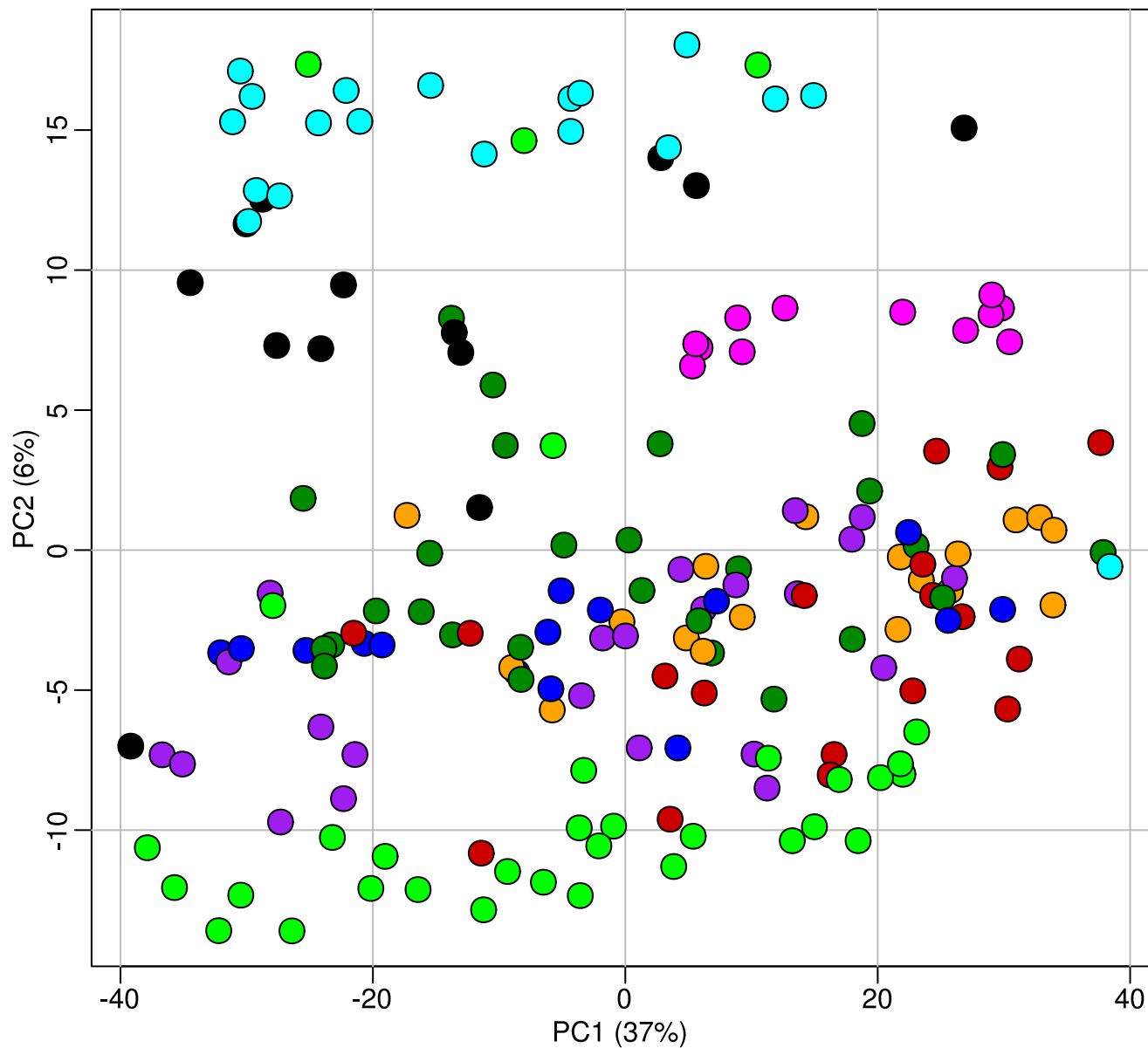

Supplement: Supplementary file 14 — High-variance genes of type five with a log2 variance > −1. On each page the left panel displays the expression intensities of a gene in developmental order, the right panel displays the same gene plotted in developmental order ordered per spawn. (PDF 181 kb) [file 12864_2017_3672_MOESM14_ESM.pdf]

## Amplification controls

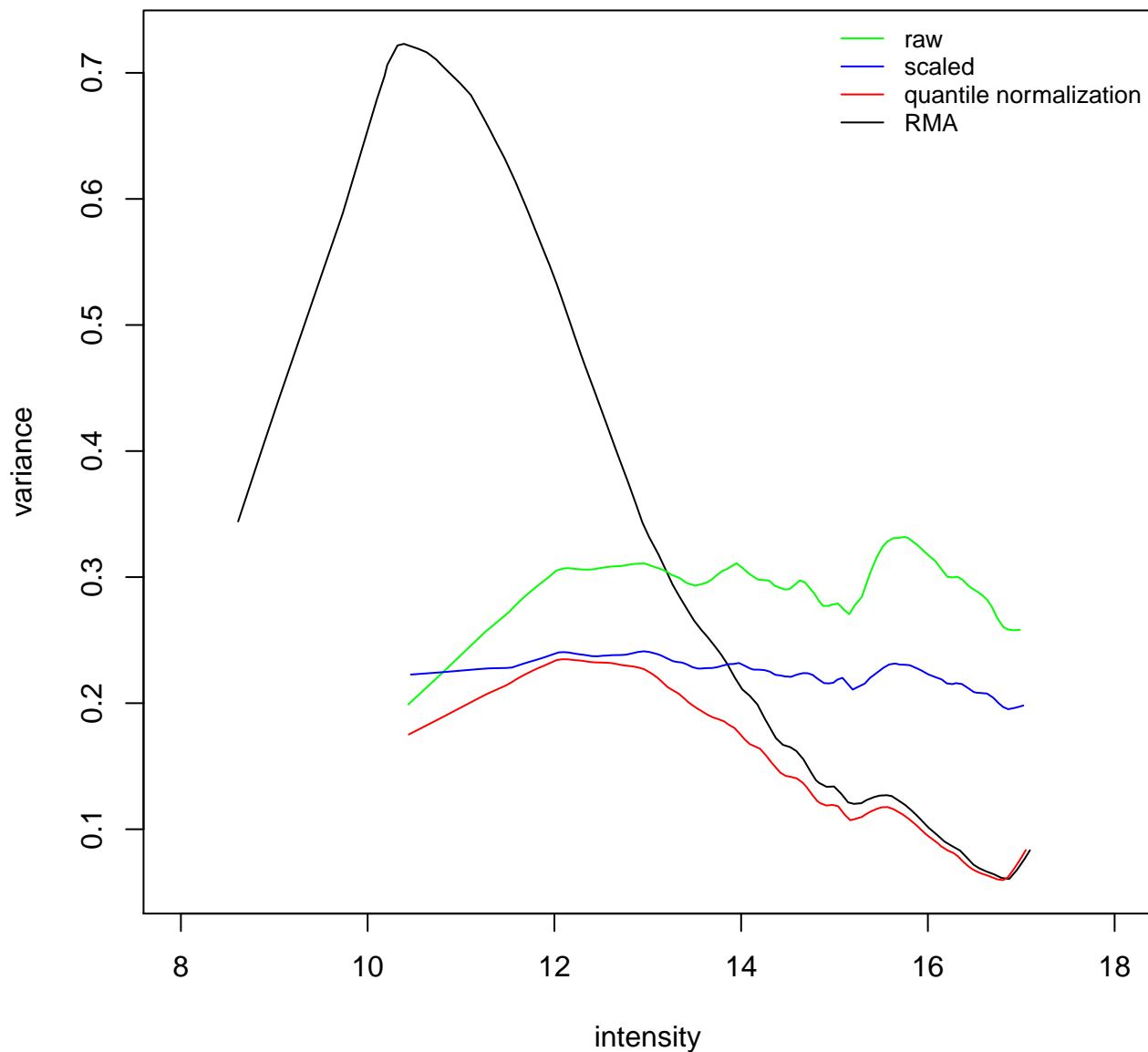

Supplement: Supplementary file 17 — Spike in controls were added to the samples and were hybridized to specific probes on the array. Based on the application of 3 widely used normalization methods and the resulting variance we decided to apply quantile normalization in this analysis. (PDF 13 kb) [file 12864_2017_3672_MOESM17_ESM.pdf]
