# Supplementary material for: Transcriptome dynamics in early zebrafish embryogenesis determined by high-resolution time course analysis of 180 successive, individual zebrafish embryos
Source: BMC Genomics. 2017 Apr 11;18:287. doi: 10.1186/s12864-017-3672-z (PMC5387192; doi:10.1186/s12864-017-3672-z)
Supplement: Supplementary file 6 — Using two different training sets to establish developmental order. Page 1, A - Samples ordered by a training set of genes with continuously increasing gene-expression vs. samples ordered by a training set of genes with continuously decreasing gene-expression. Samples are colored by epiboly. B - ENSDARG00000016725 ordered according to epiboly (bottom) and in developmental order (top); Page 2–123: test set of 121 continuously decreasing genes ordered by a training set of 97 continuously increasing genes. Page 124–221: test set of 97 continuously increasing genes ordered by a training set of 97 continuously decreasing genes. (PDF 1403 kb) [file 12864_2017_3672_MOESM6_ESM.pdf]

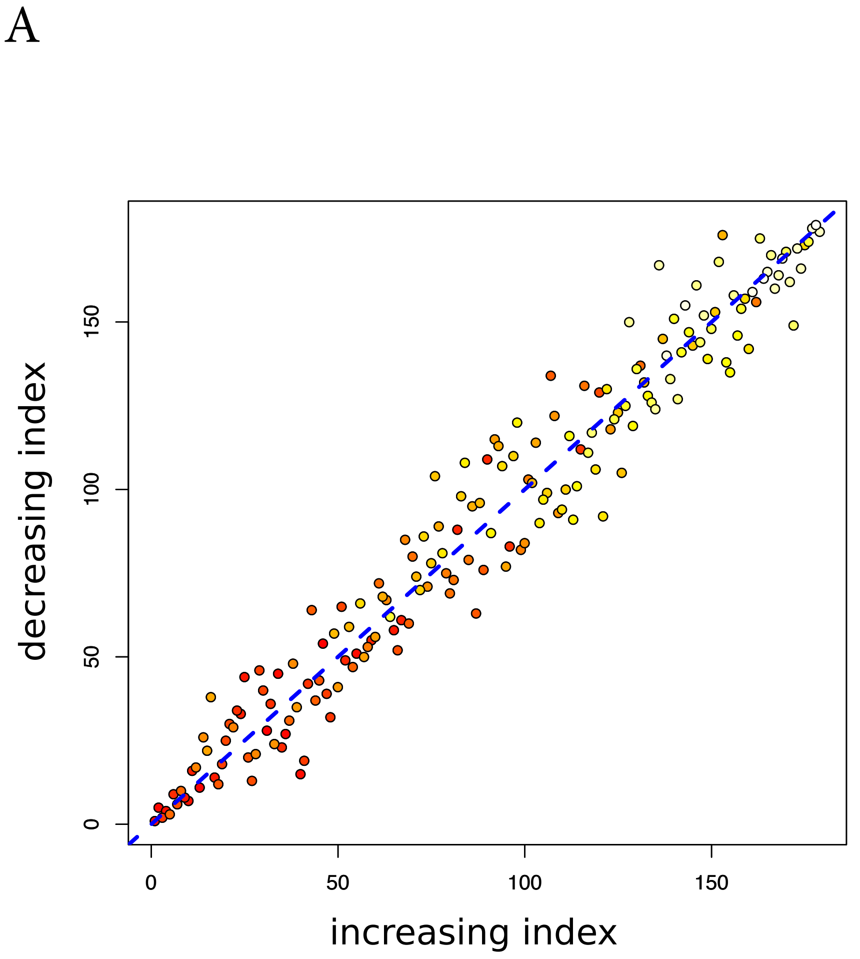

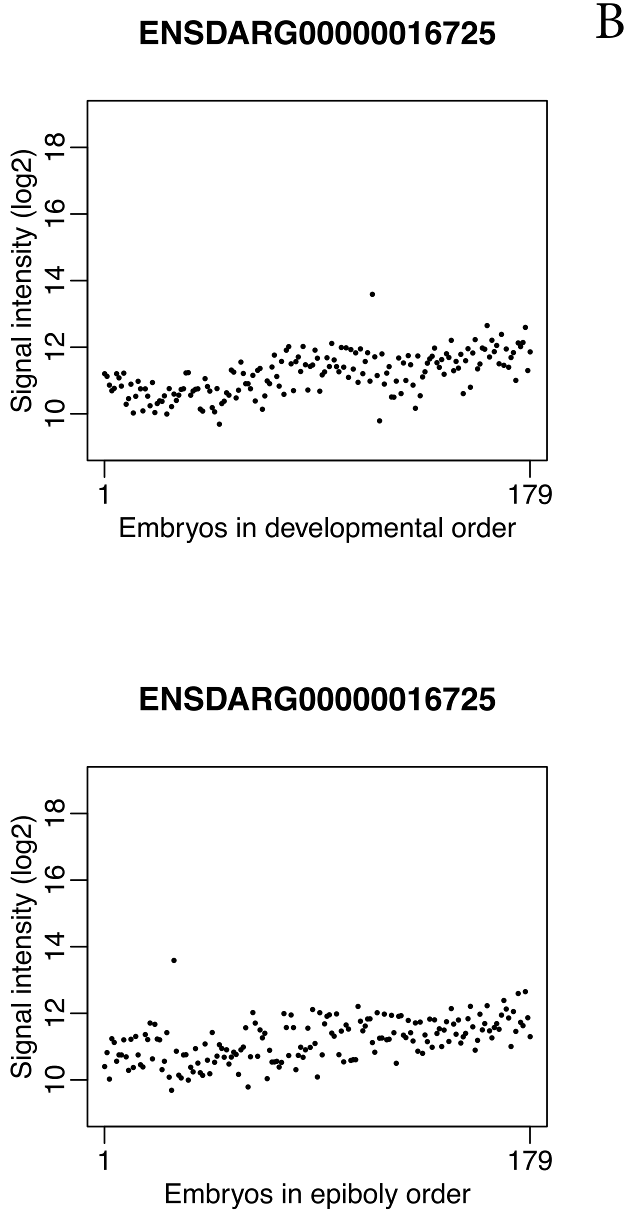

Increasing order test gene profiles from page 3 to 123
Decreasing order test gene profiles from page 125 to 221

Comparison between the gene expression profiles orderings using:

97 continuously **increasing** genes to ***train*** the ordering

121 continuously **decreasing** genes to ***test*** the ordering.

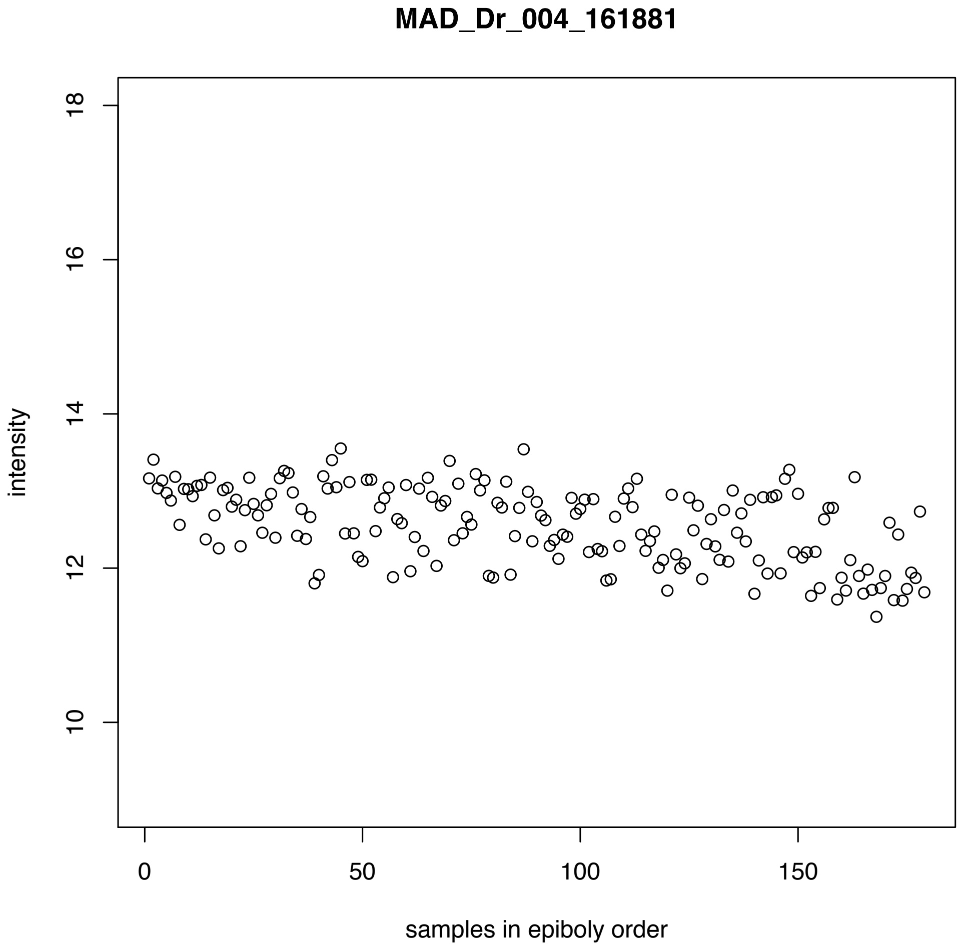
MAD_Dr_004_161881
intensity
18
16
14
12
10
0
50
100
150
samples in epiboly order

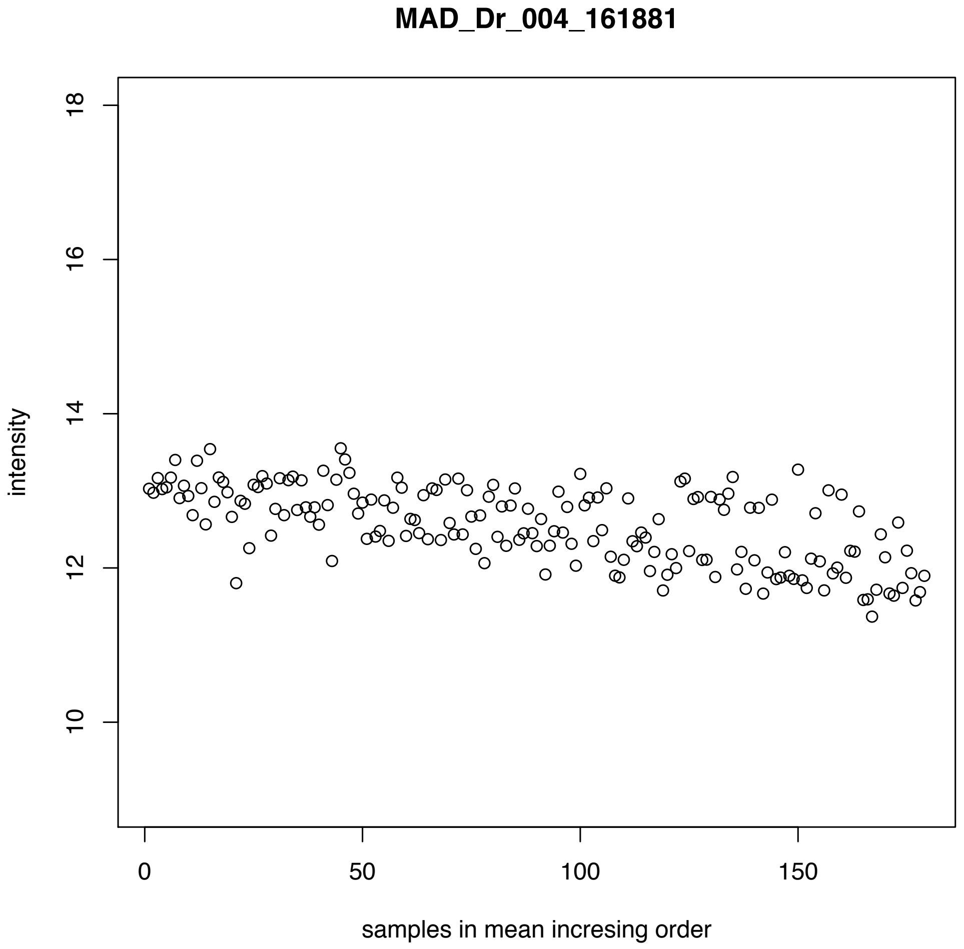
MAD_Dr_004_161881
intensity
18
16
14
12
10
0
50
100
150
samples in mean incresing order

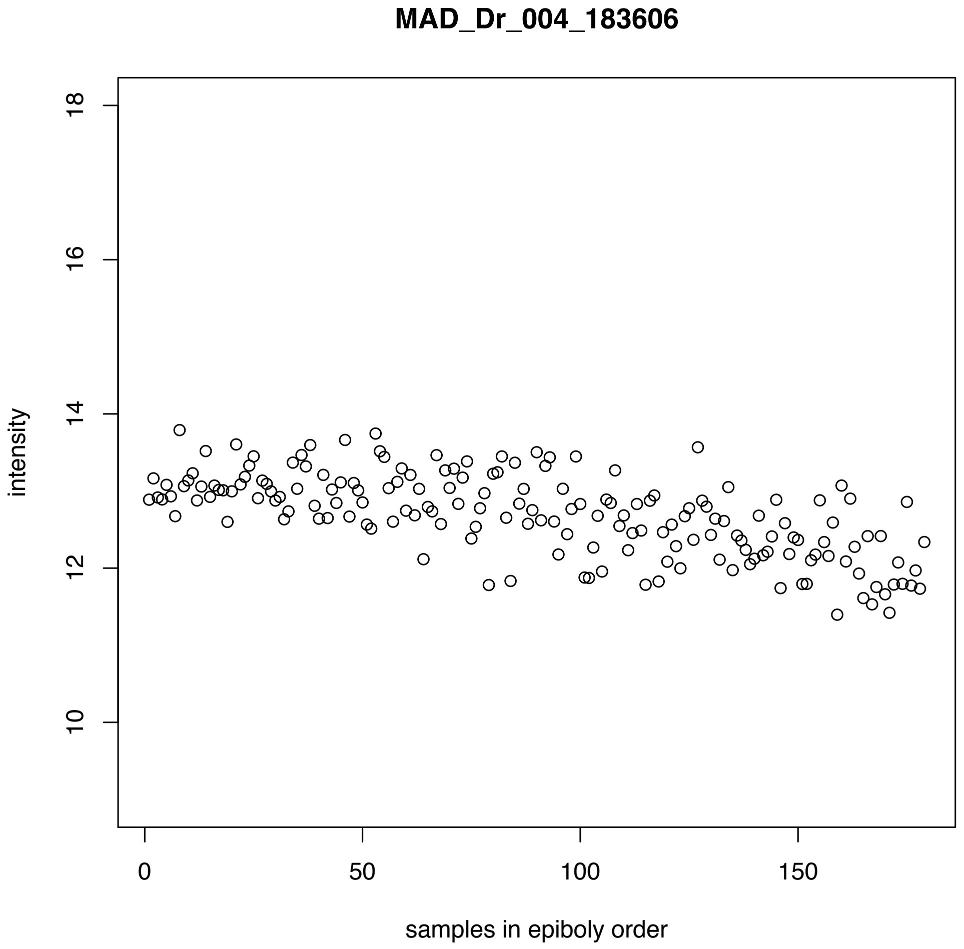
MAD_Dr_004_183606
intensity
10
12
14
16
18
0
50
100
150
samples in epiboly order

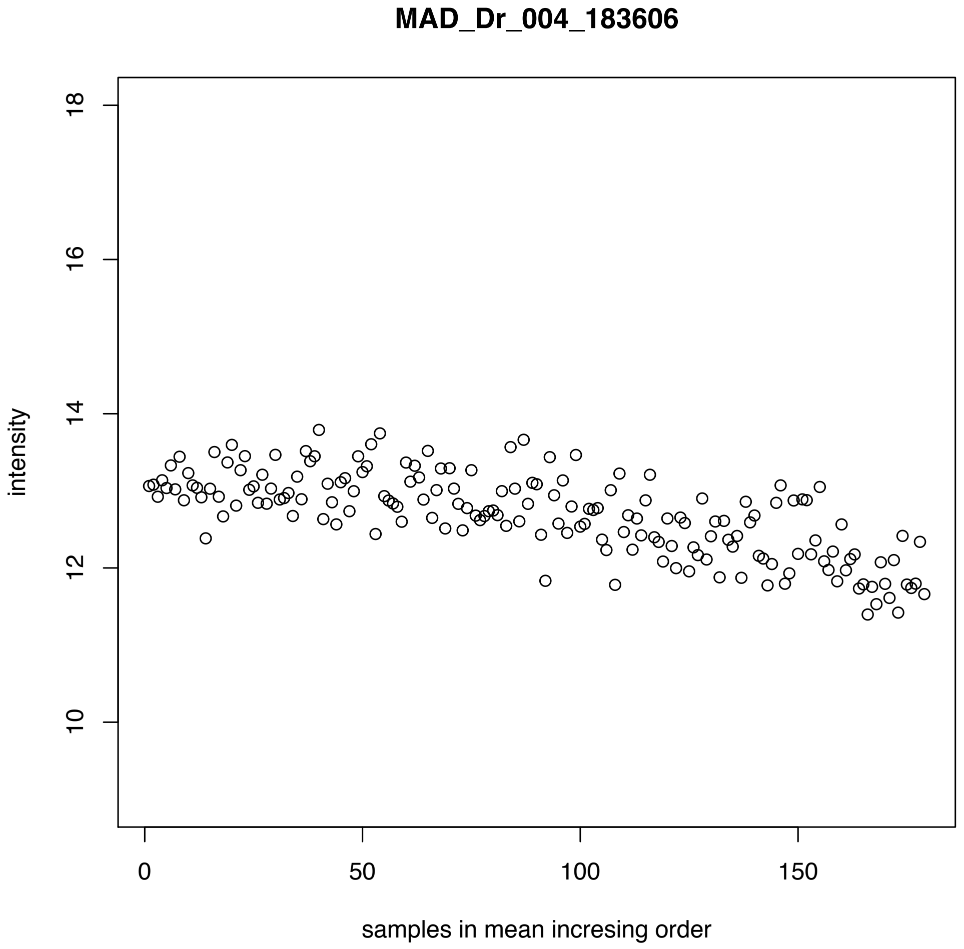
MAD_Dr_004_183606
intensity
10
12
14
16
18
0
50
100
150
samples in mean incresing order

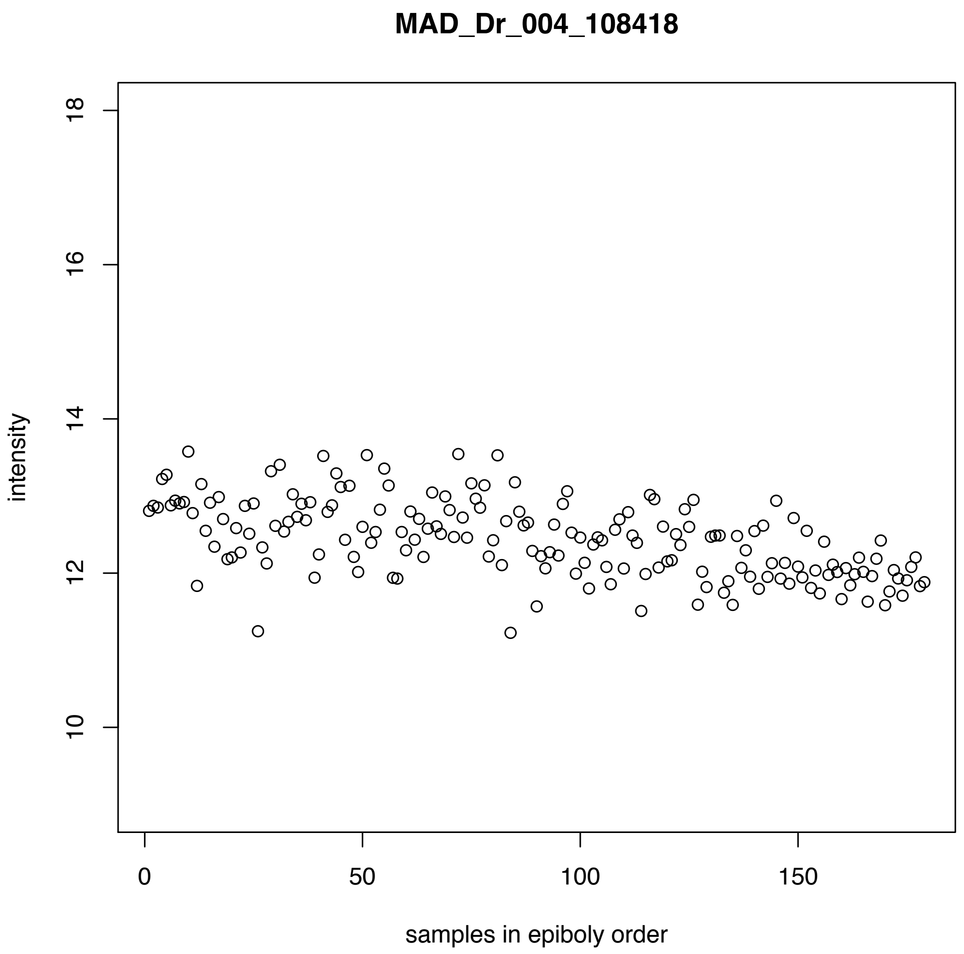
MAD_Dr_004_108418
intensity
10
12
14
16
18
0
50
100
150
samples in epiboly order

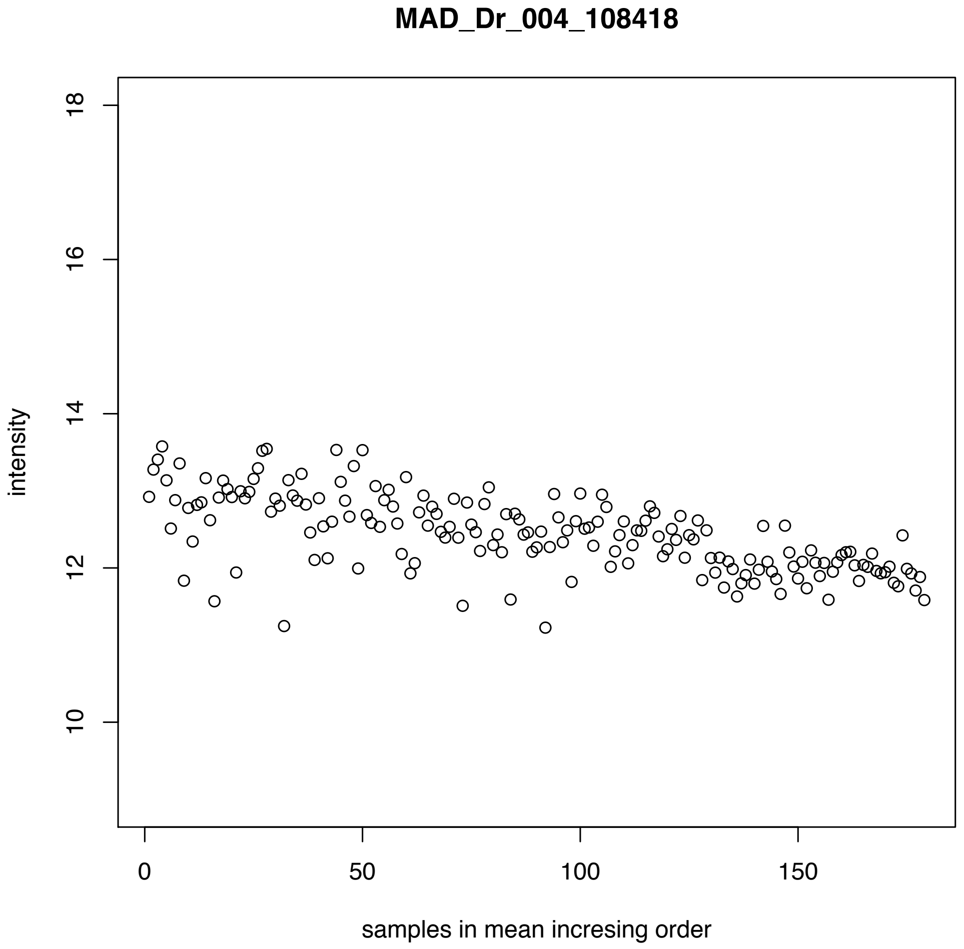
MAD_Dr_004_108418
intensity
10
12
14
16
18
0
50
100
150
samples in mean incresing order

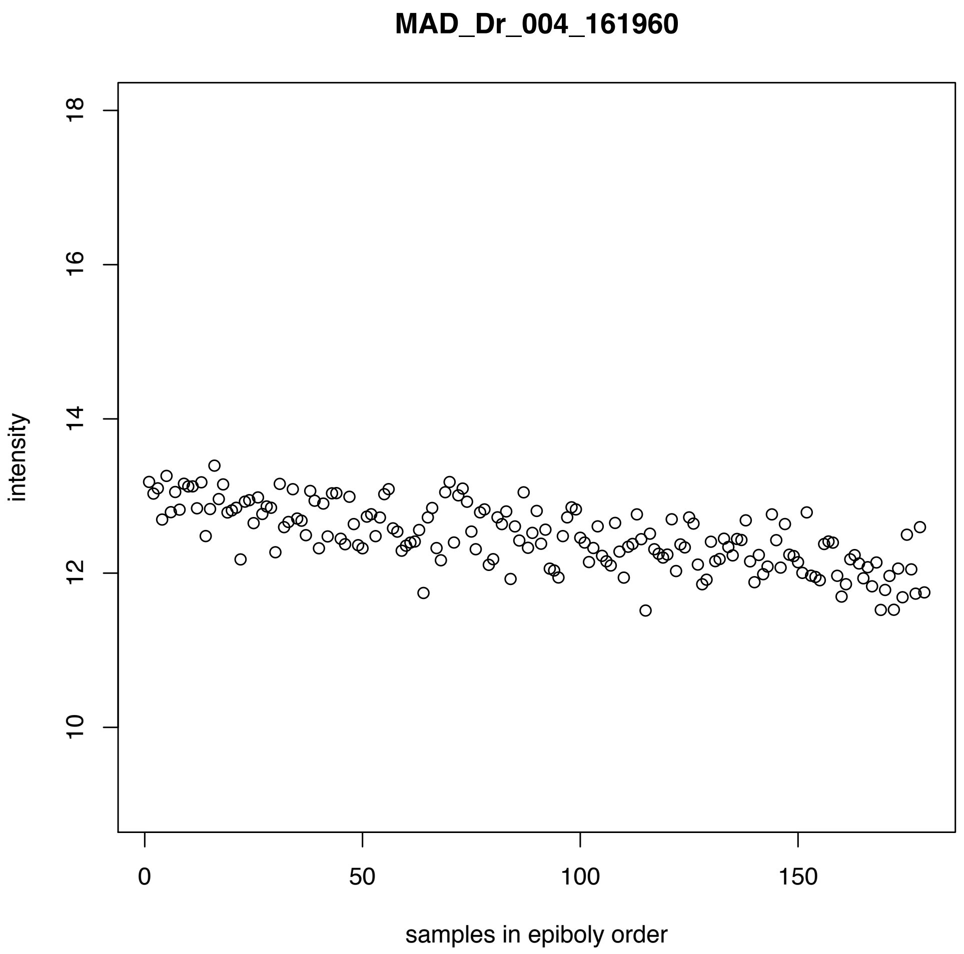
MAD_Dr_004_161960
intensity
10
12
14
16
18
0
50
100
150
samples in epiboly order

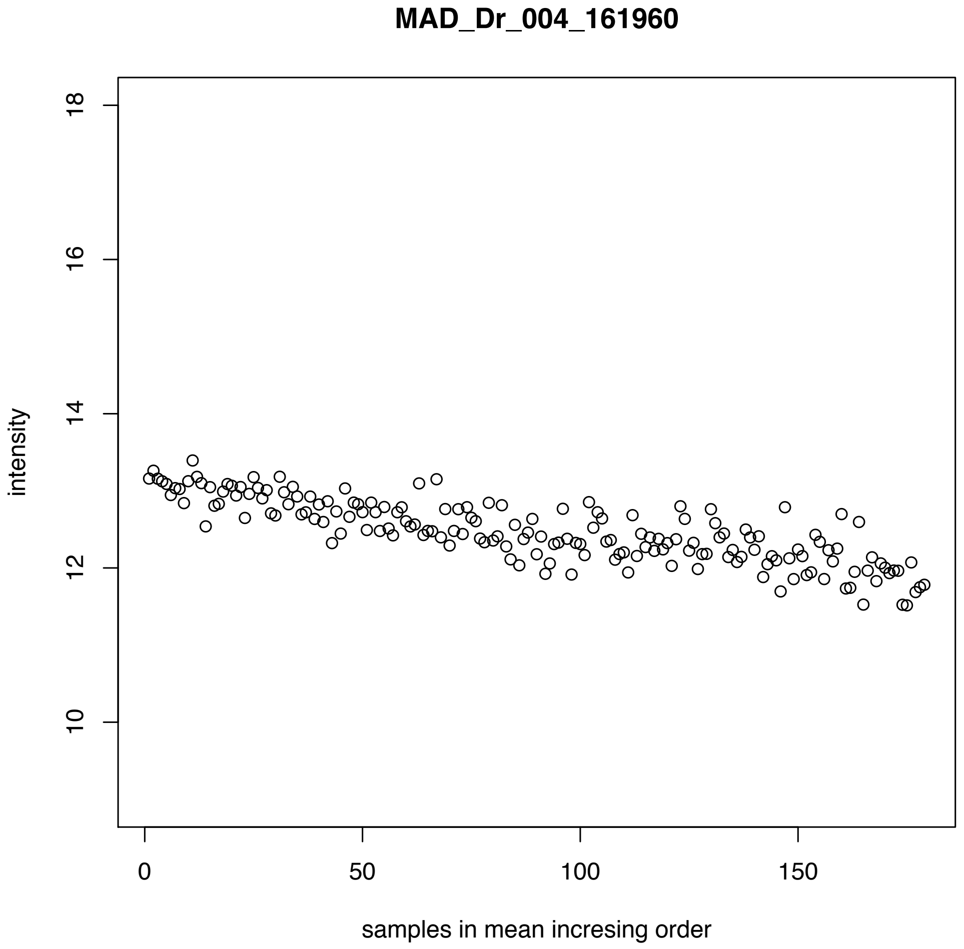
MAD_Dr_004_161960
intensity
10
12
14
16
18
0
50
100
150
samples in mean incresing order

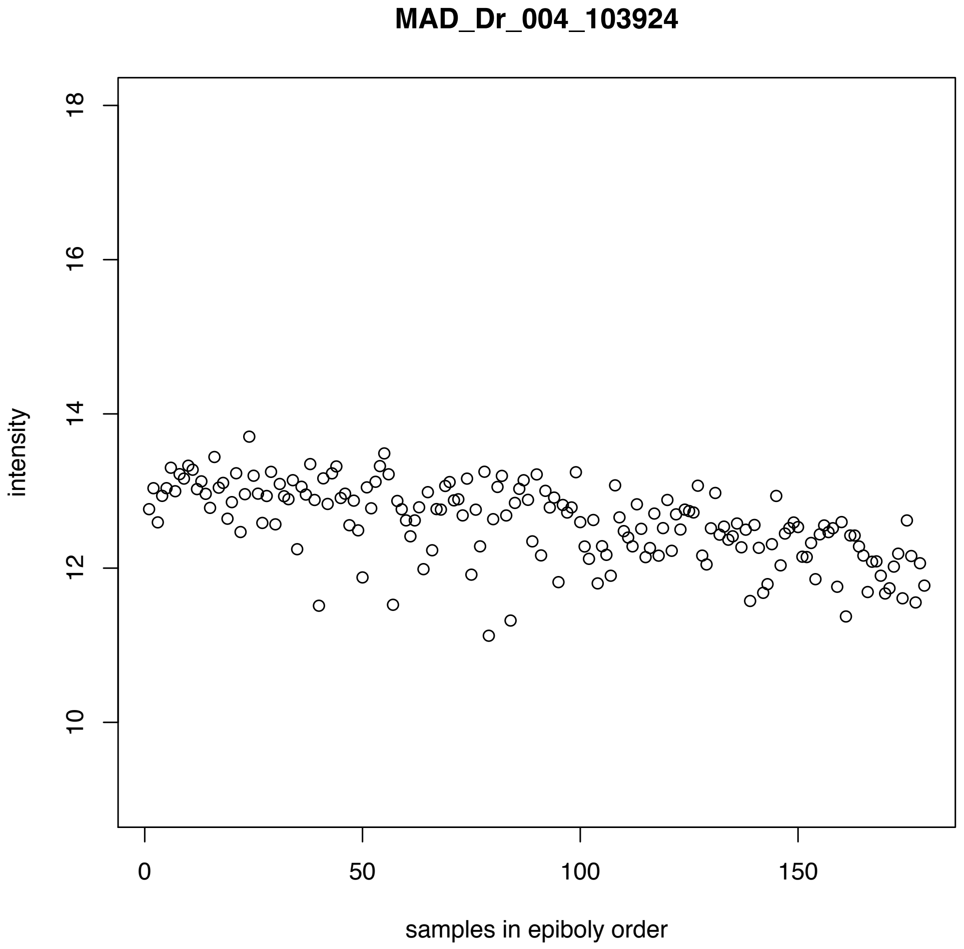
MAD_Dr_004_103924
intensity
samples in epiboly order
10
12
14
16
18
0
50
100
150

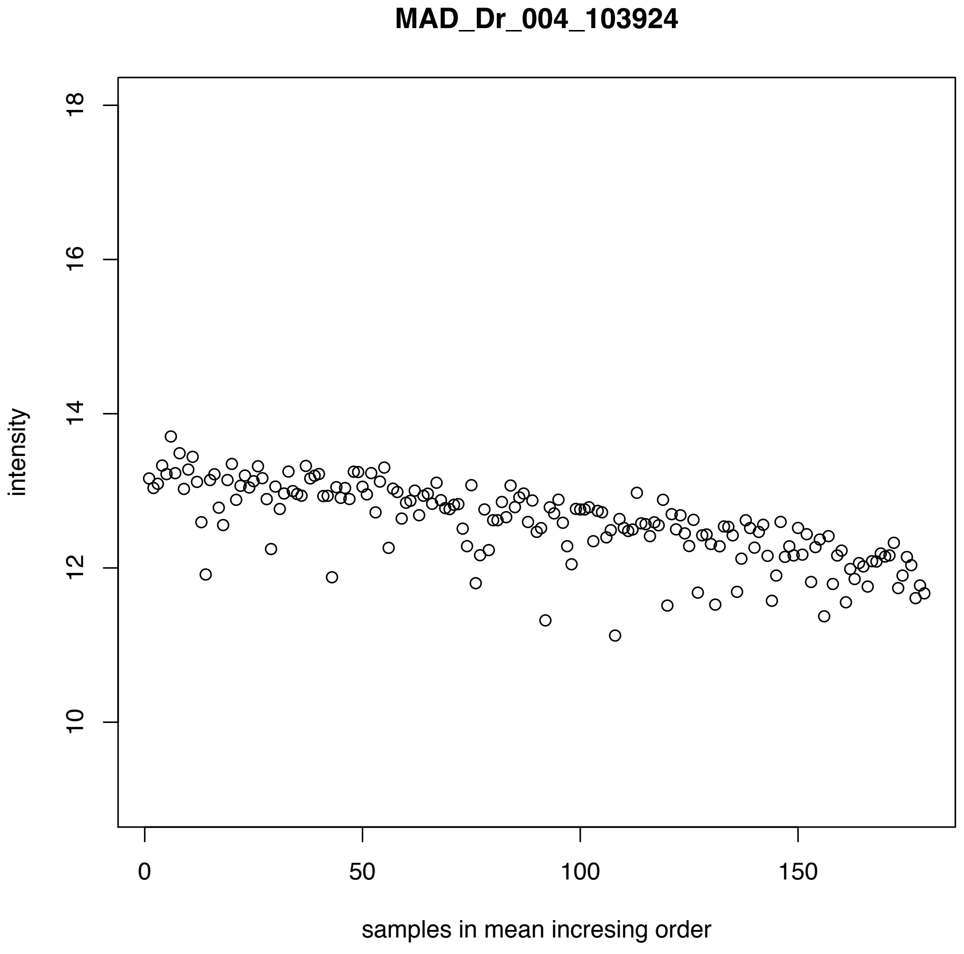
MAD_Dr_004_103924
intensity
samples in mean incresing order
10
12
14
16
18
0
50
100
150

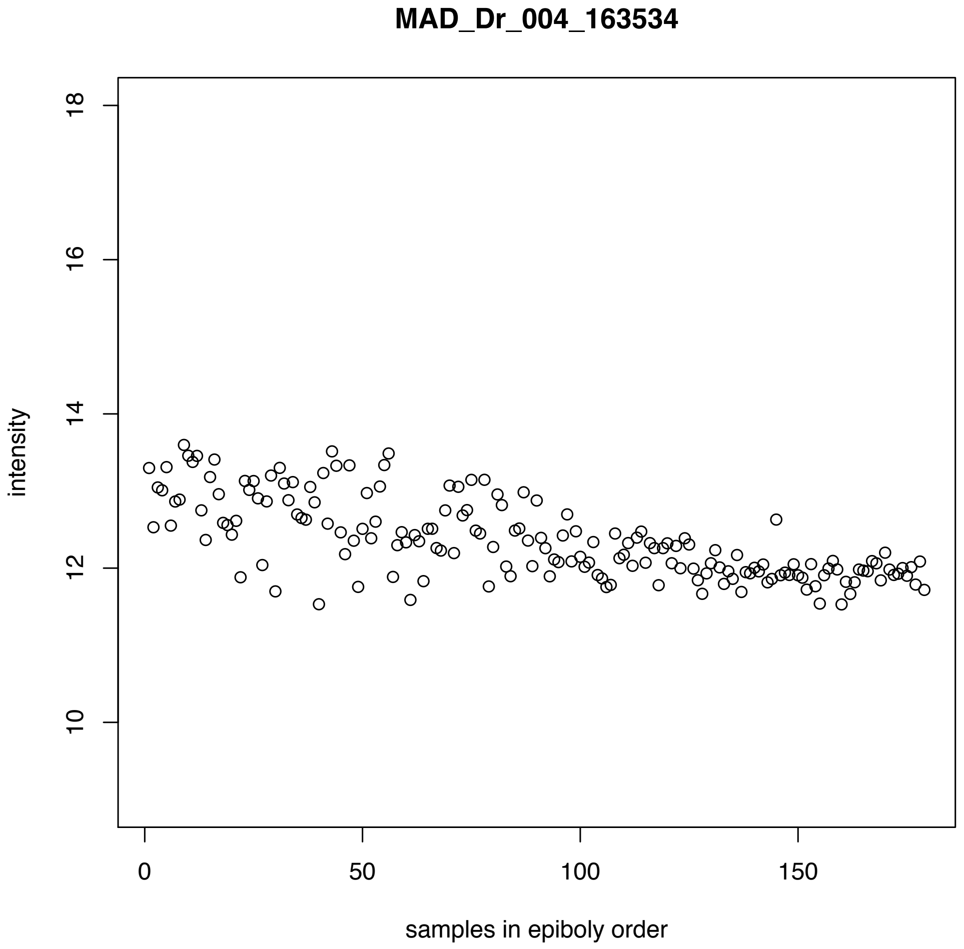
MAD_Dr_004_163534
intensity
10
12
14
16
18
0
50
100
150
samples in epiboly order

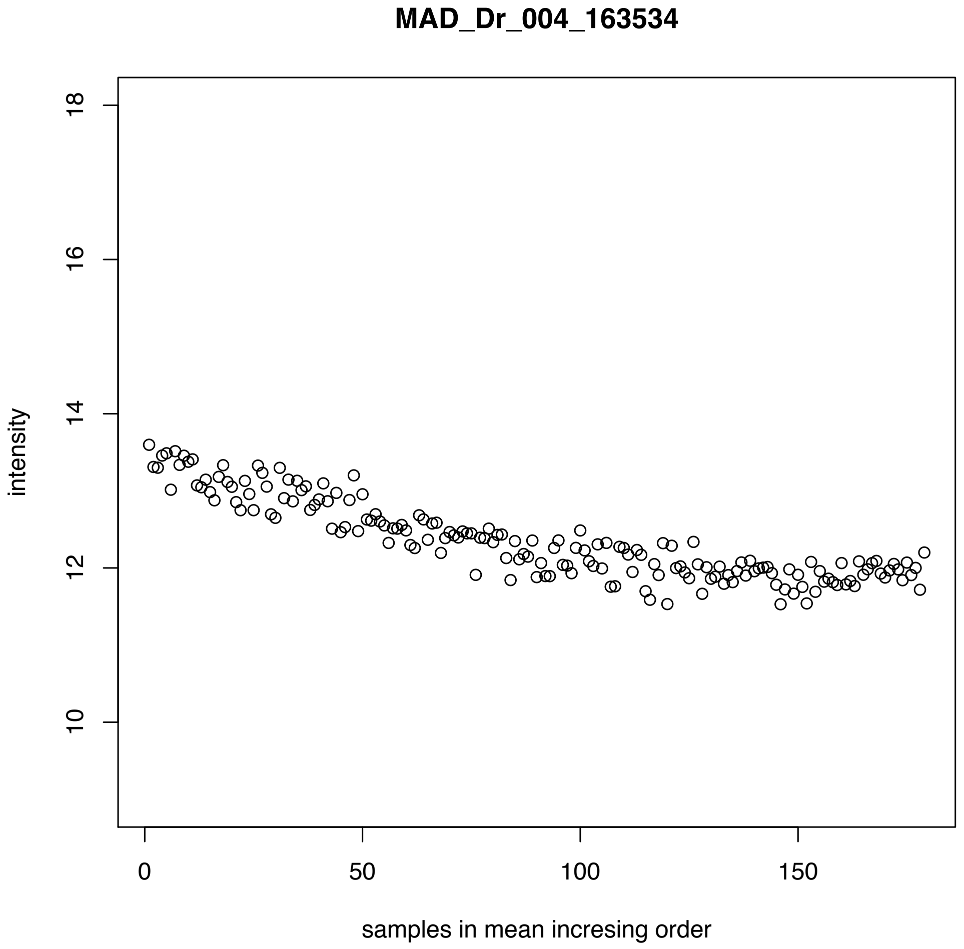
MAD_Dr_004_163534
intensity
10
12
14
16
18
0
50
100
150
samples in mean incresing order

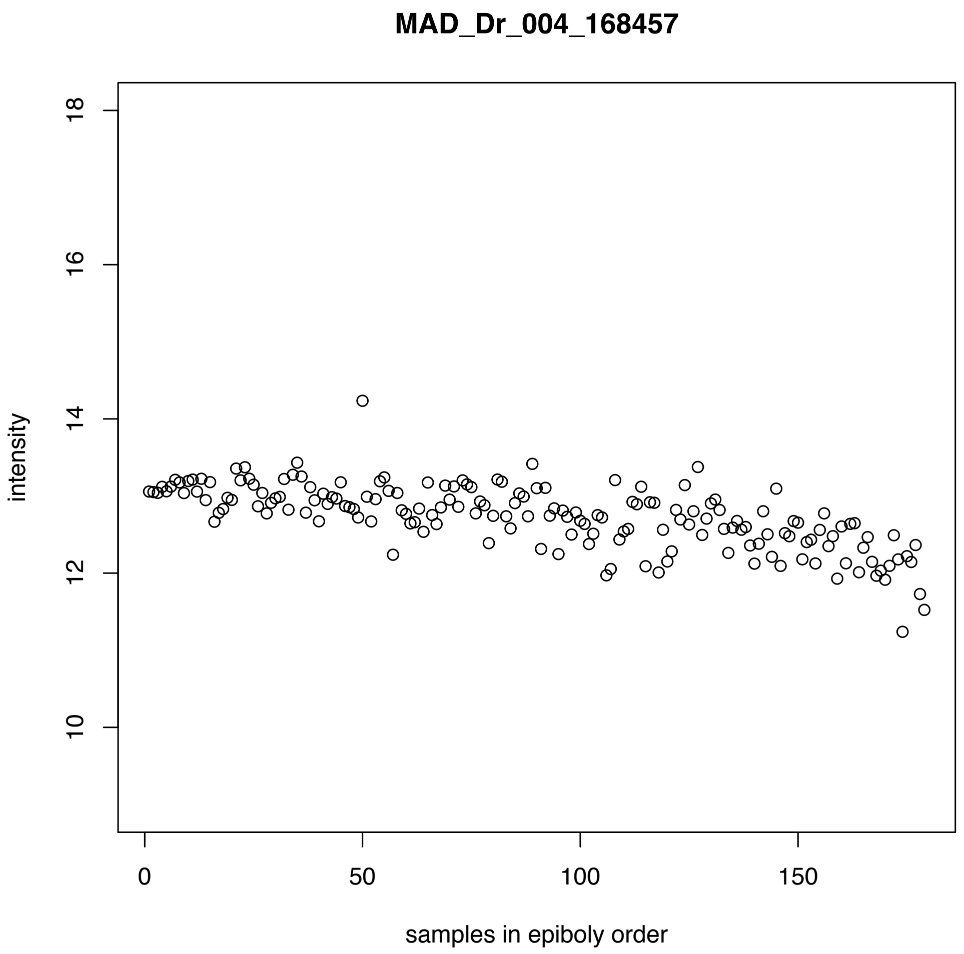

MAD_Dr_004_168457
intensity
10
12
14
16
18
0
50
100
150
samples in epiboly order

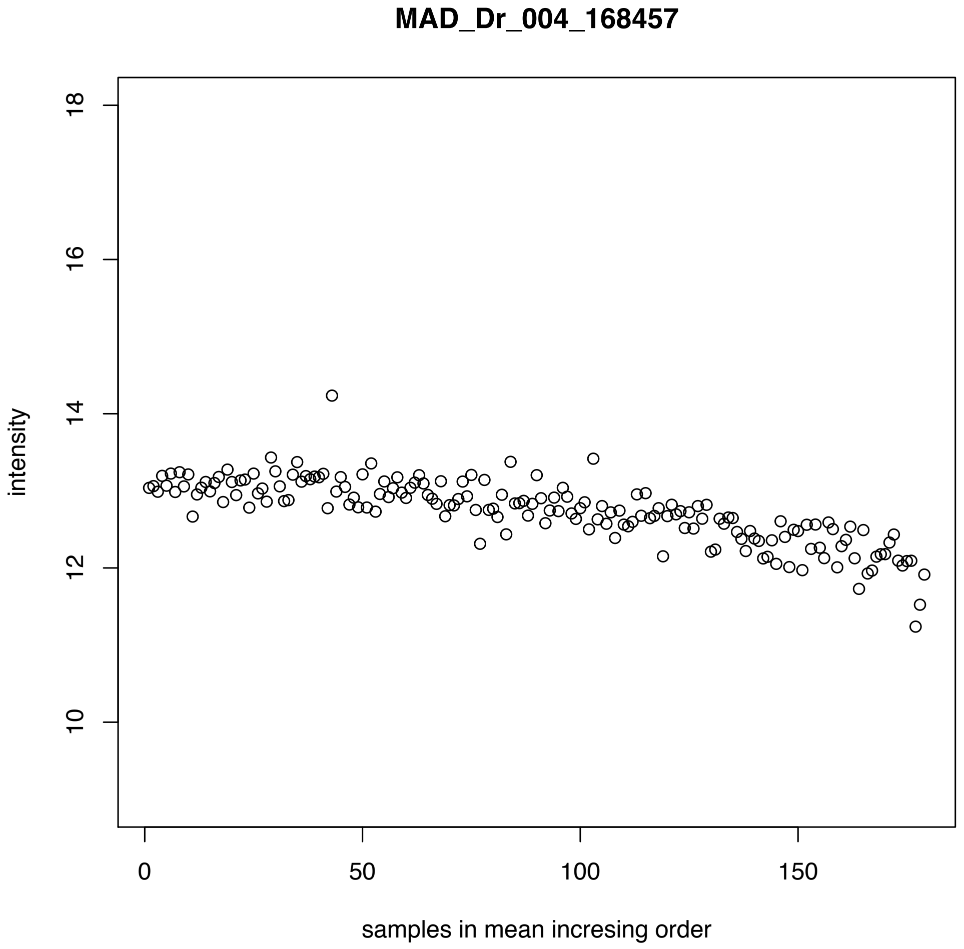

MAD_Dr_004_168457
intensity
10
12
14
16
18
0
50
100
150
samples in mean incresing order

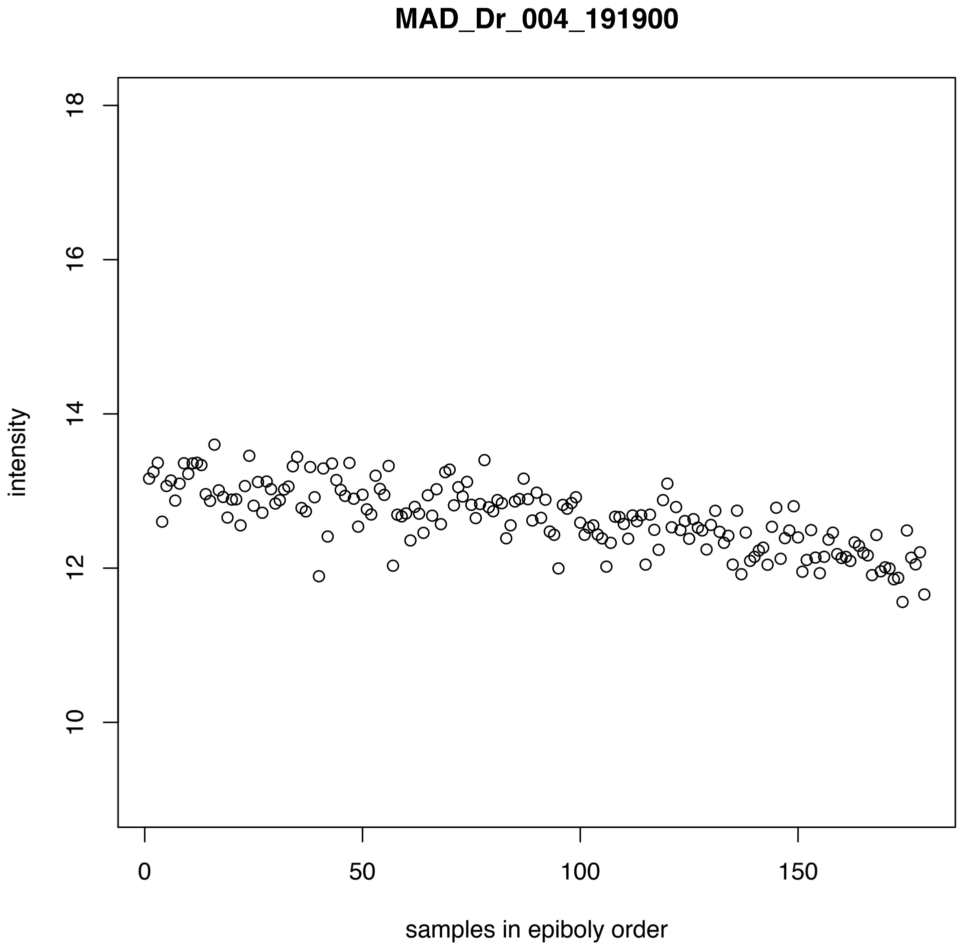
MAD_Dr_004_191900
intensity
samples in epiboly order
10
12
14
16
18
0
50
100
150

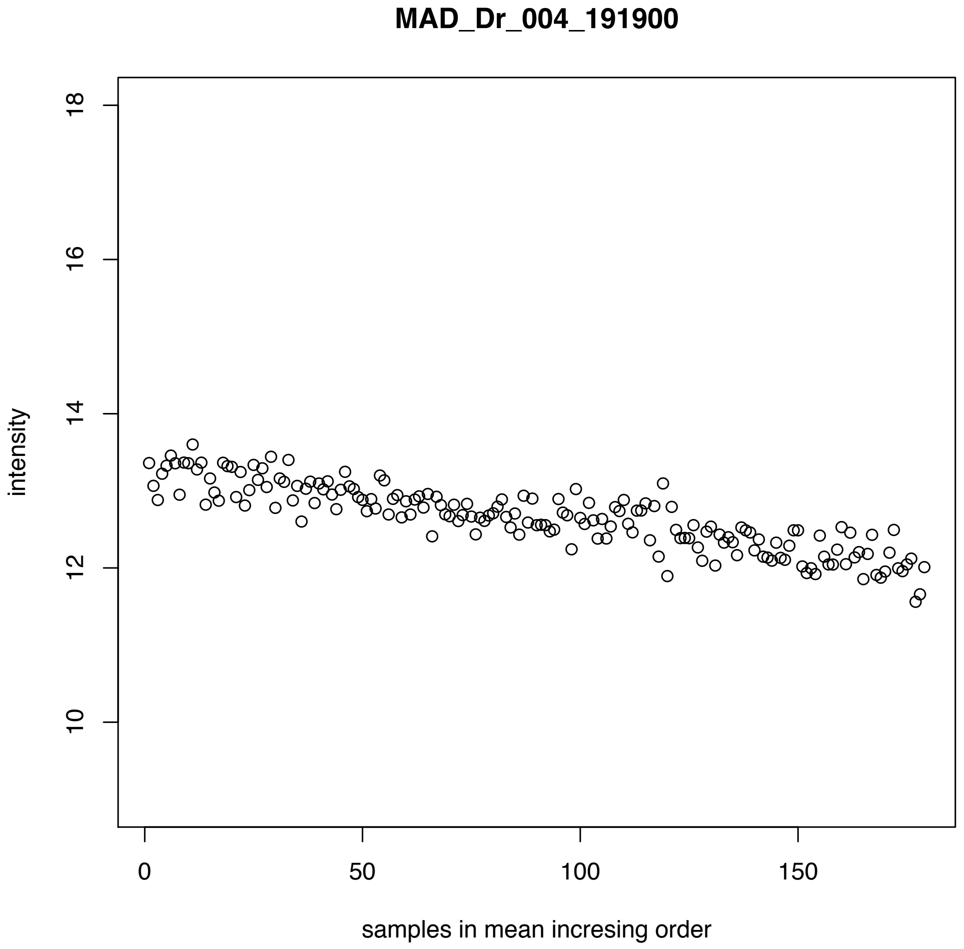
MAD_Dr_004_191900
intensity
samples in mean incresing order
10
12
14
16
18
0
50
100
150

MAD_Dr_004_180556

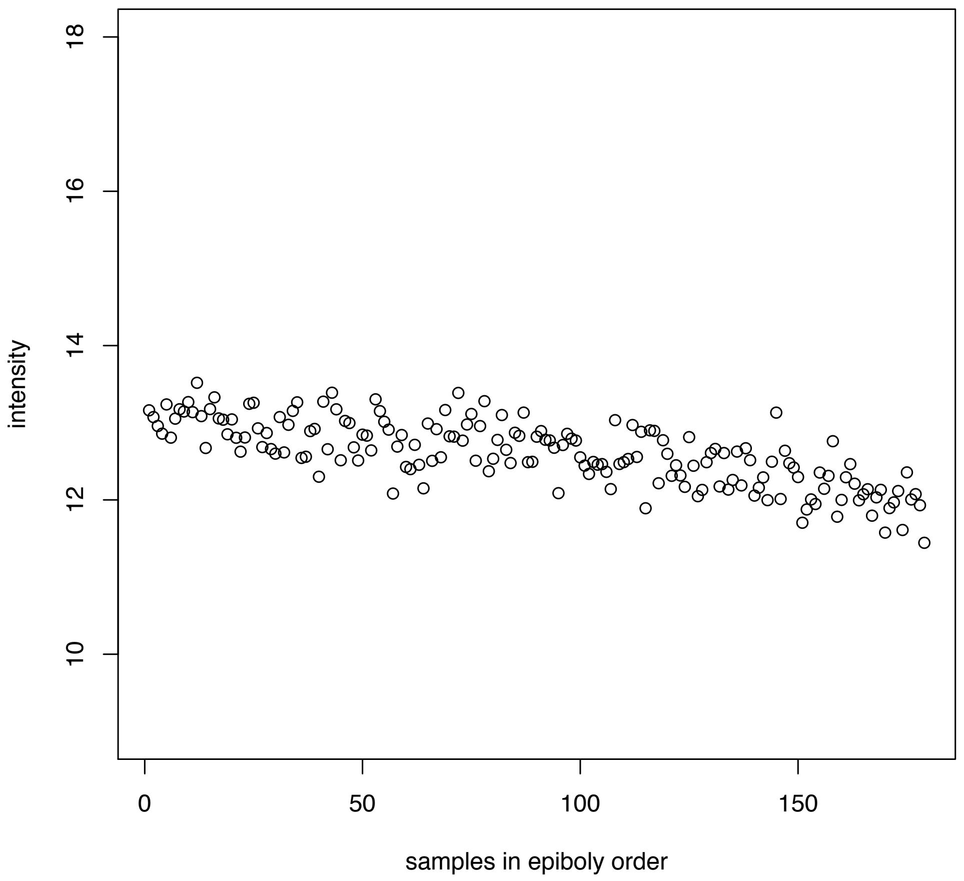

MAD_Dr_004_180556

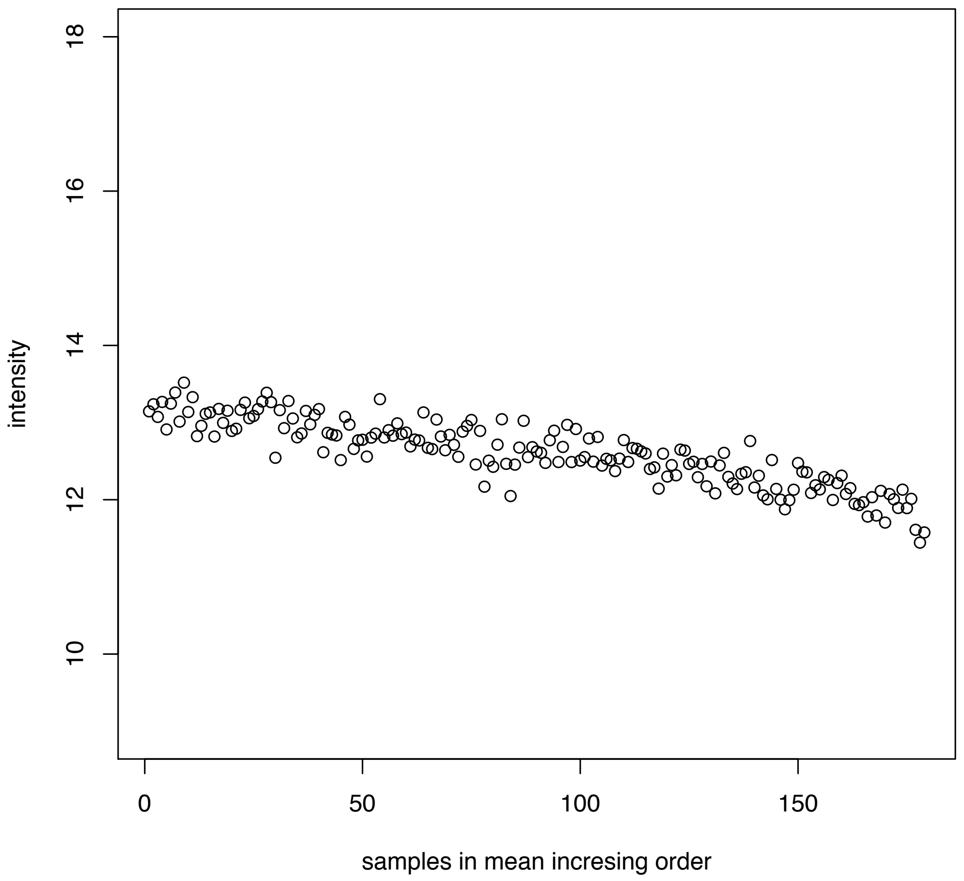

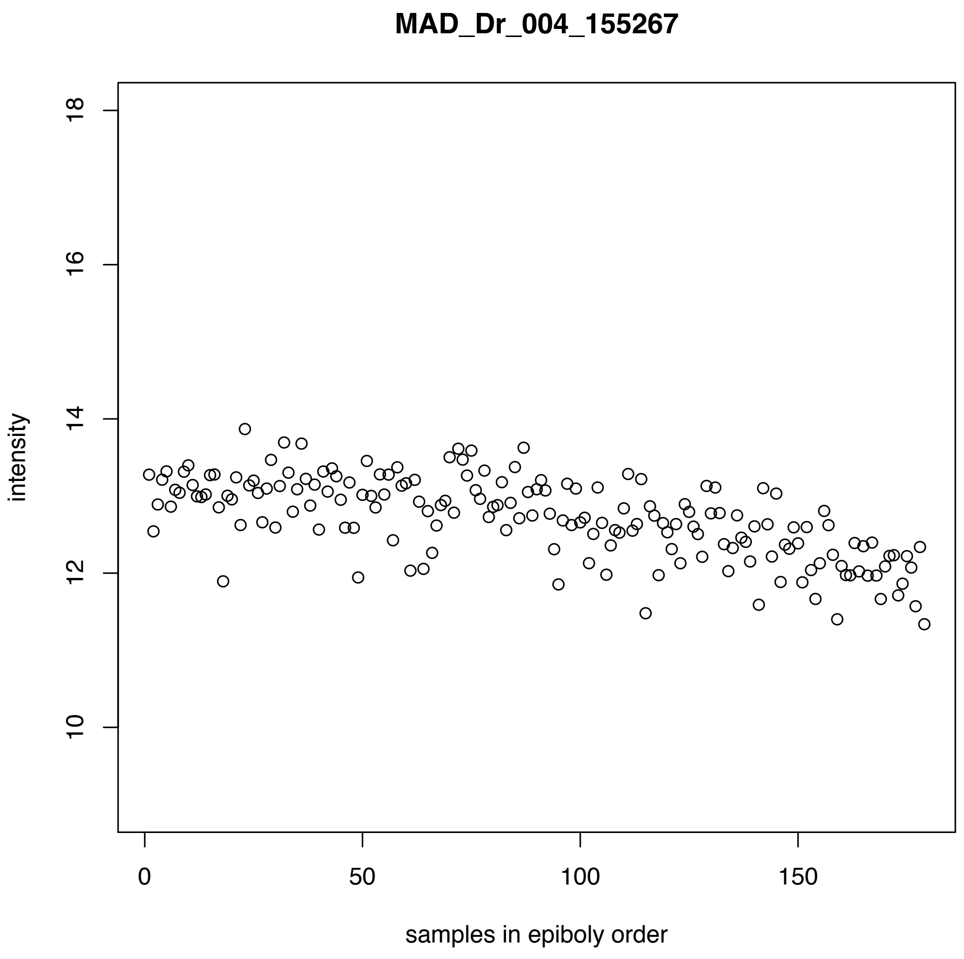
MAD_Dr_004_155267
intensity
18
16
14
12
10
0
50
100
150
samples in epiboly order

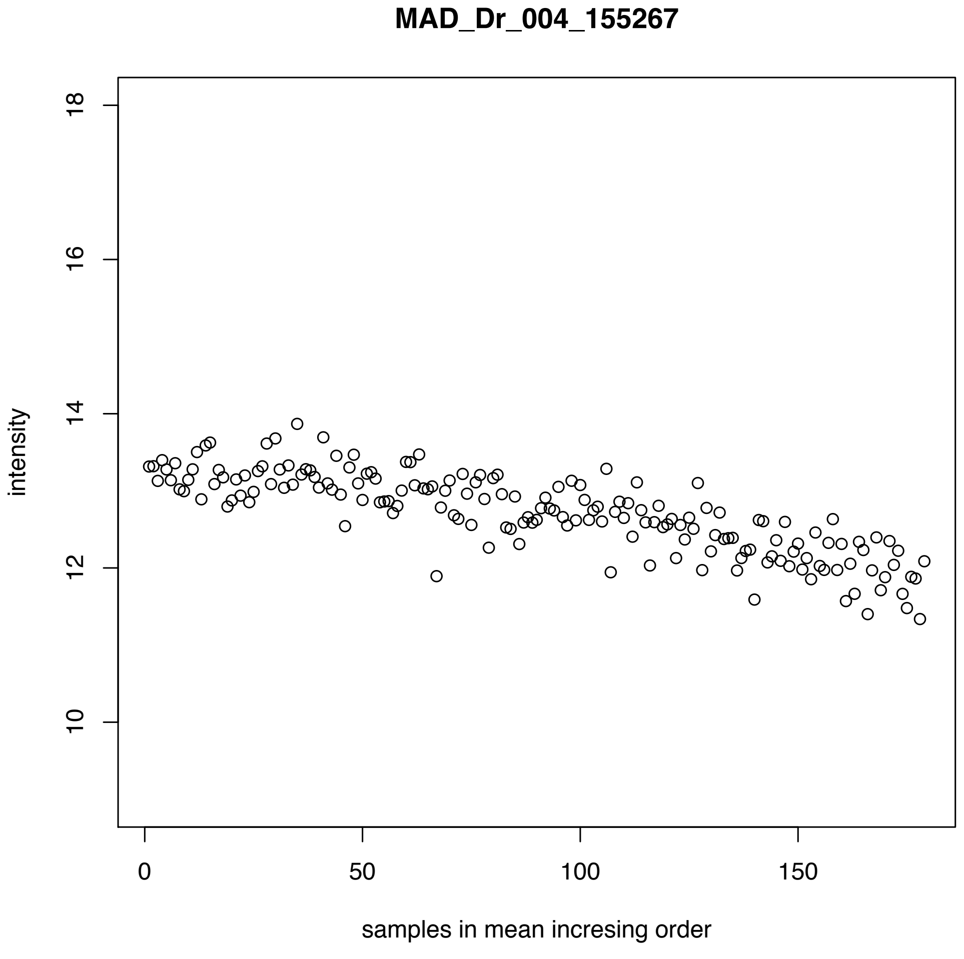
MAD_Dr_004_155267
intensity
18
16
14
12
10
0
50
100
150
samples in mean incresing order

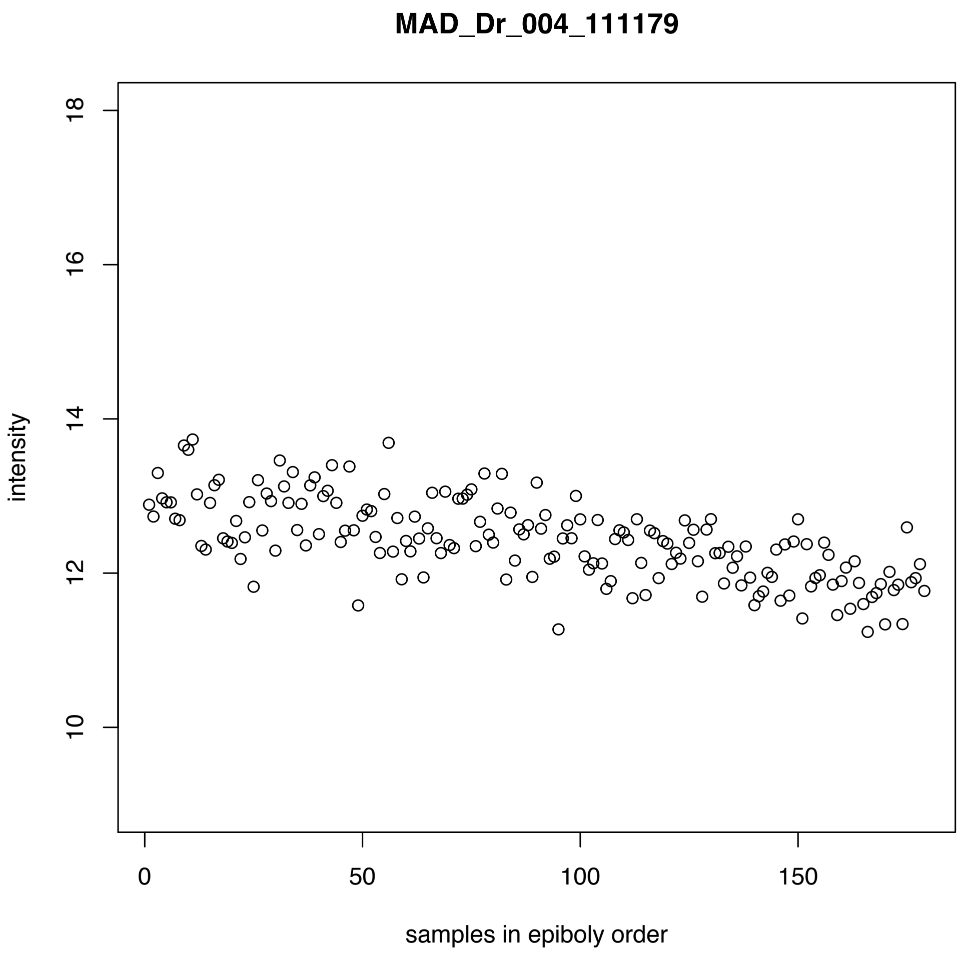
MAD_Dr_004_111179
intensity
18
16
14
12
10
0
50
100
150
samples in epiboly order

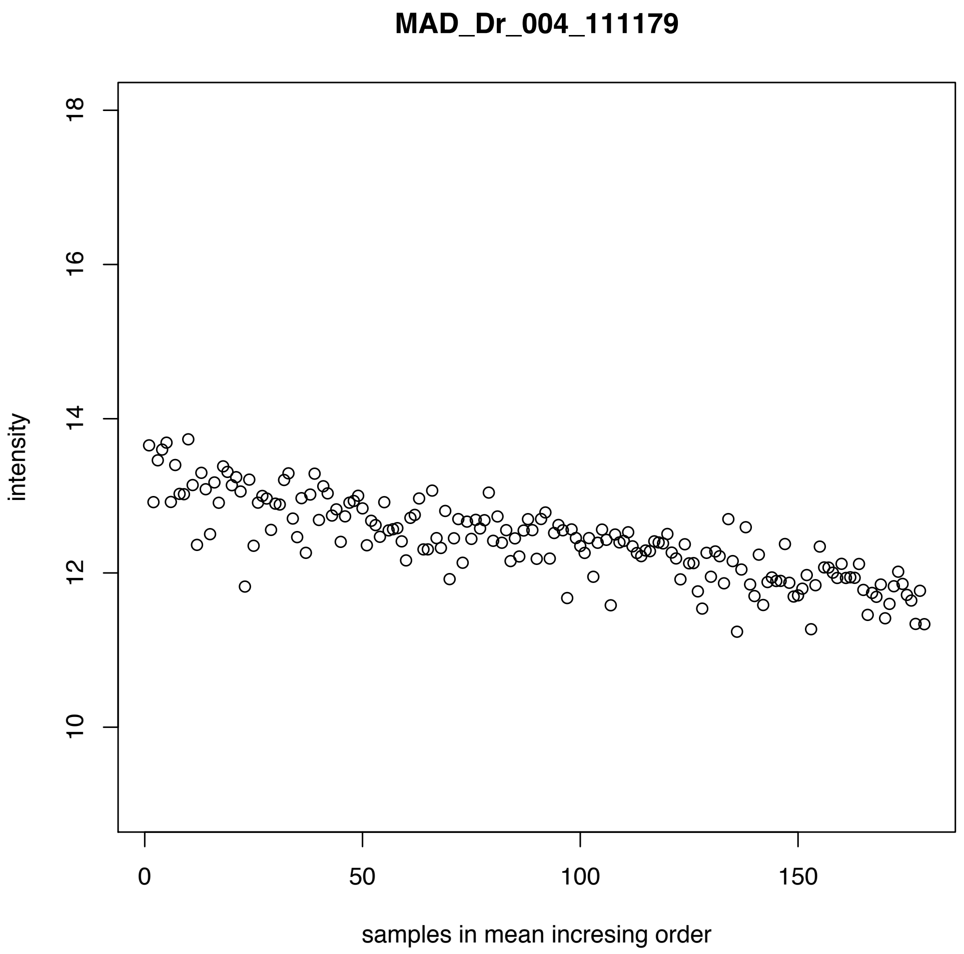
MAD_Dr_004_111179
intensity
18
16
14
12
10
0
50
100
150
samples in mean incresing order

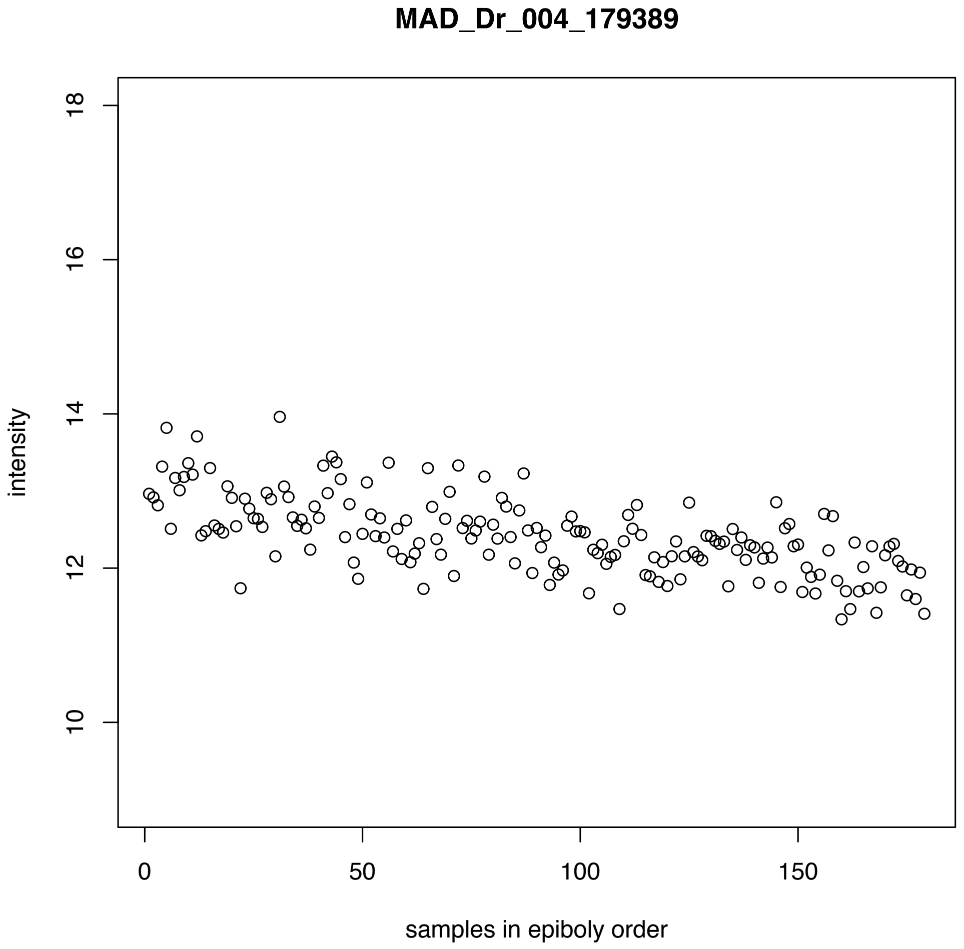
MAD_Dr_004_179389
intensity
10
12
14
16
18
0
50
100
150
samples in epiboly order

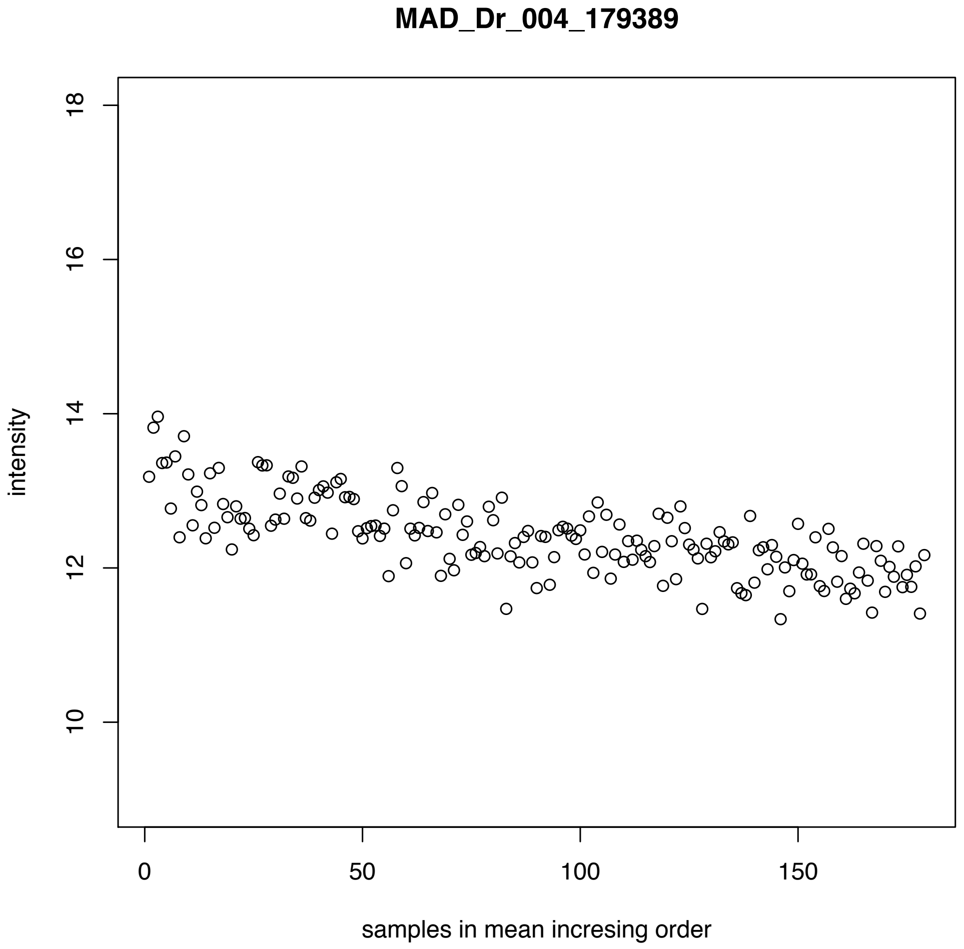
MAD_Dr_004_179389
intensity
10
12
14
16
18
0
50
100
150
samples in mean incresing order

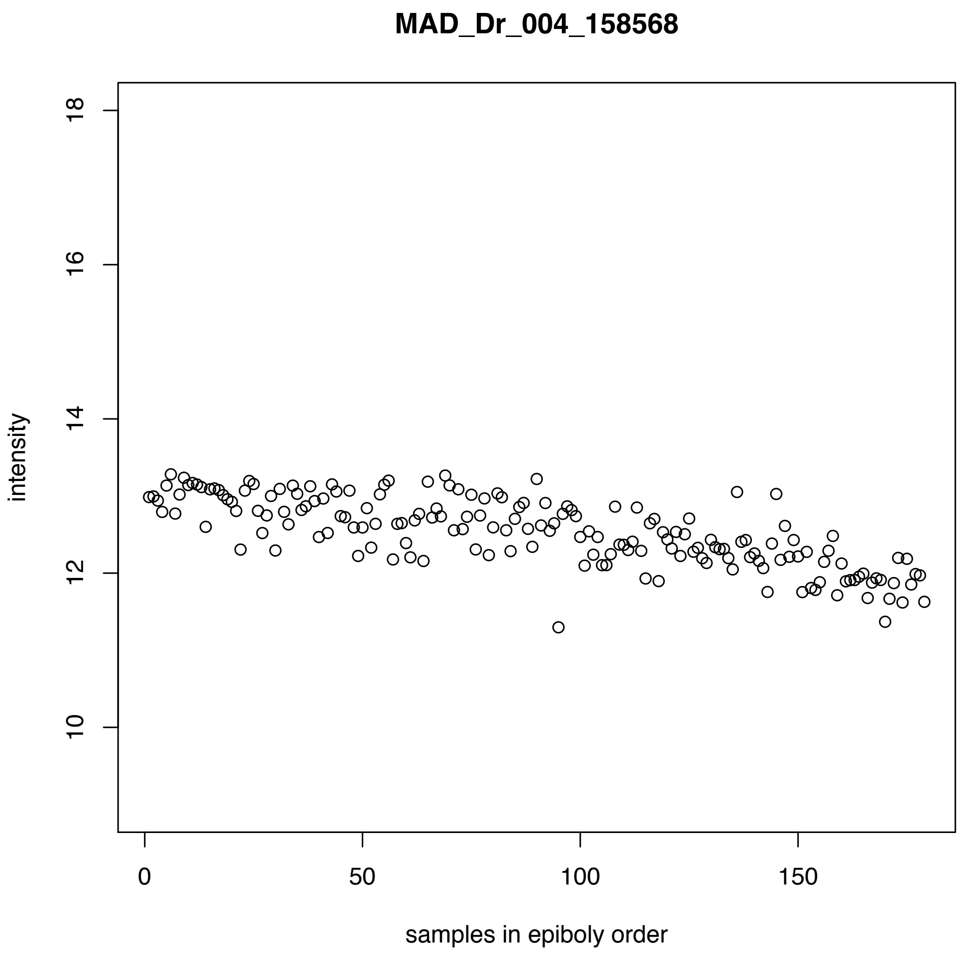

MAD_Dr_004_158568
intensity
10
12
14
16
18
0
50
100
150
samples in epiboly order

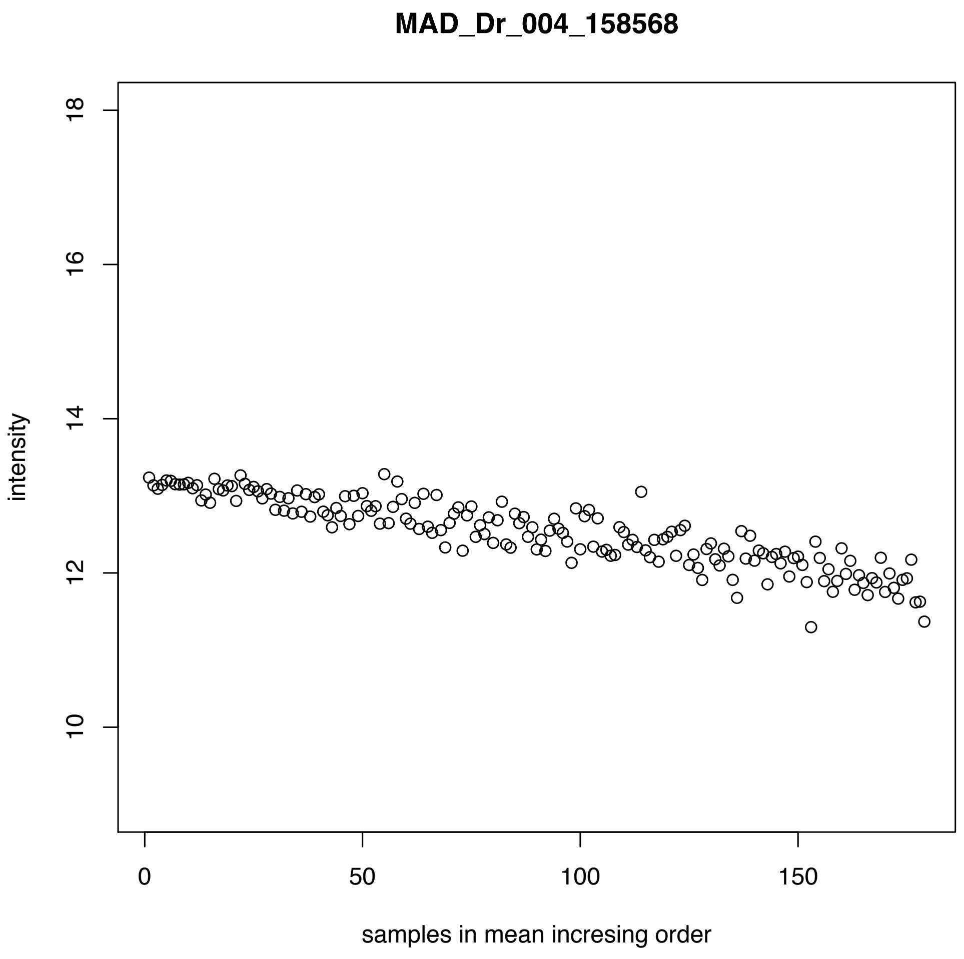

MAD_Dr_004_158568
intensity
10
12
14
16
18
0
50
100
150
samples in mean incresing order

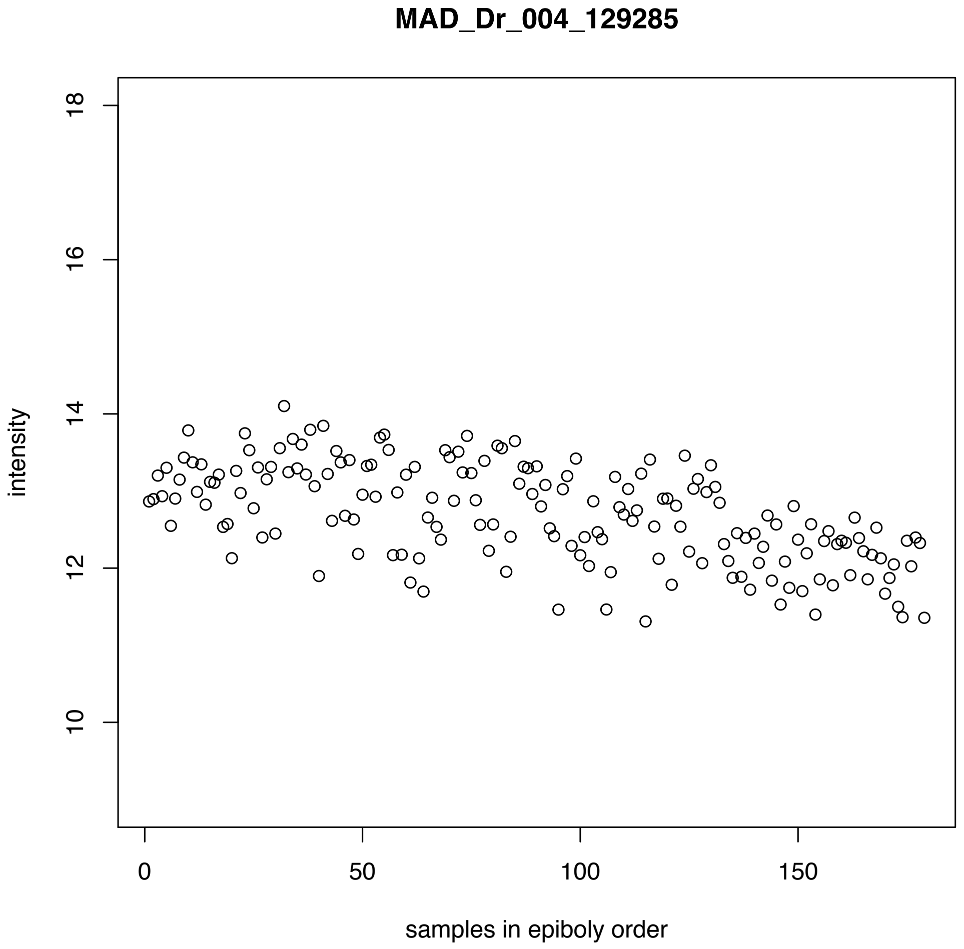
MAD_Dr_004_129285
intensity
10
12
14
16
18
0
50
100
150
samples in epiboly order

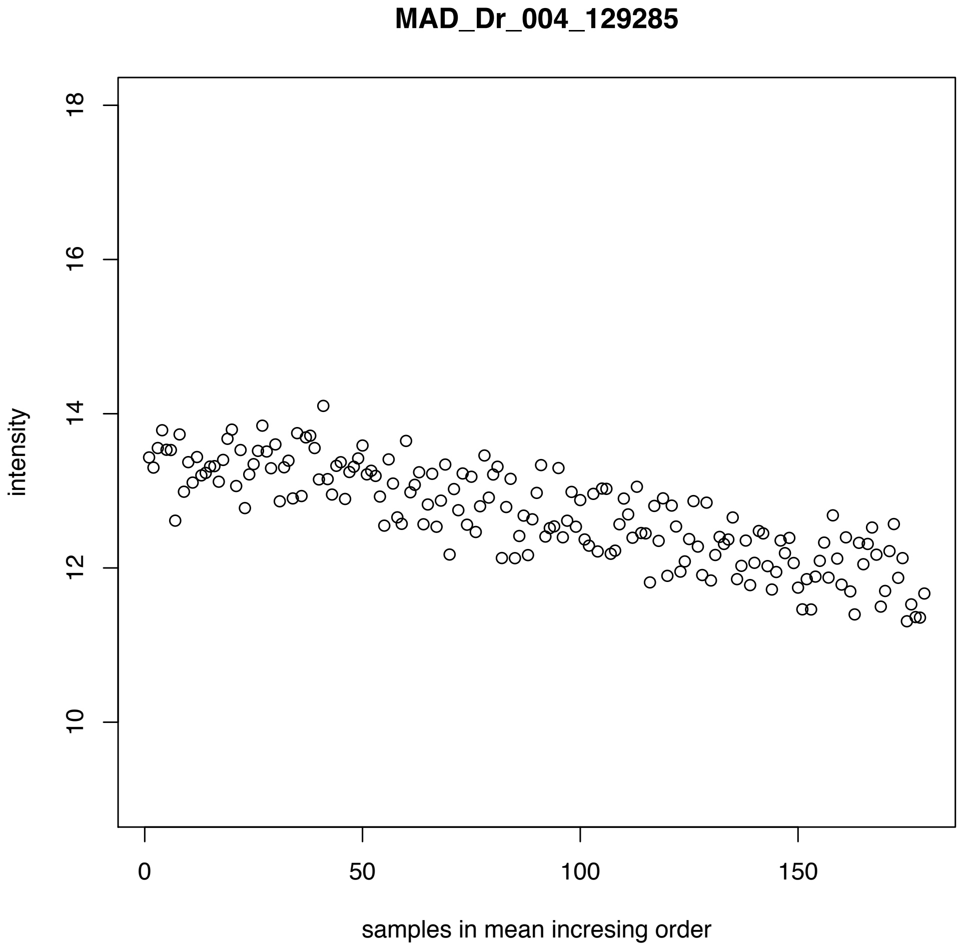
MAD_Dr_004_129285
intensity
10
12
14
16
18
0
50
100
150
samples in mean incresing order

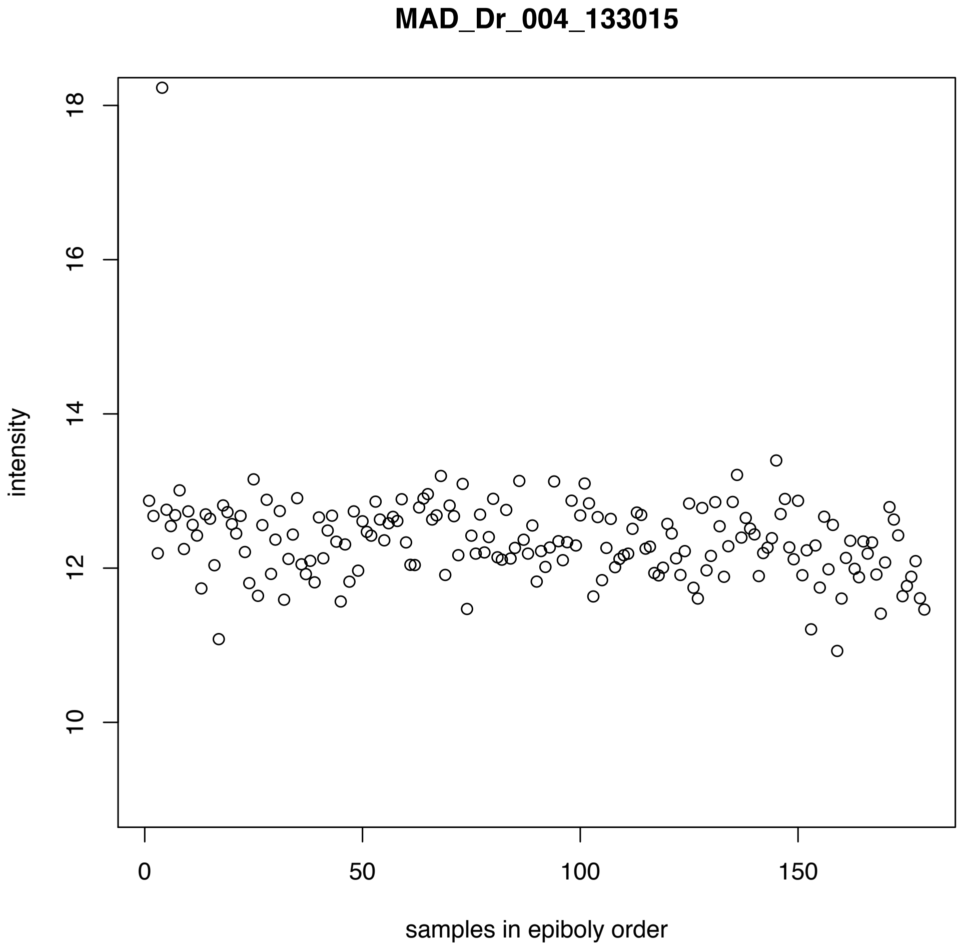
MAD_Dr_004_133015
intensity
10
12
14
16
18
0
50
100
150
samples in epiboly order

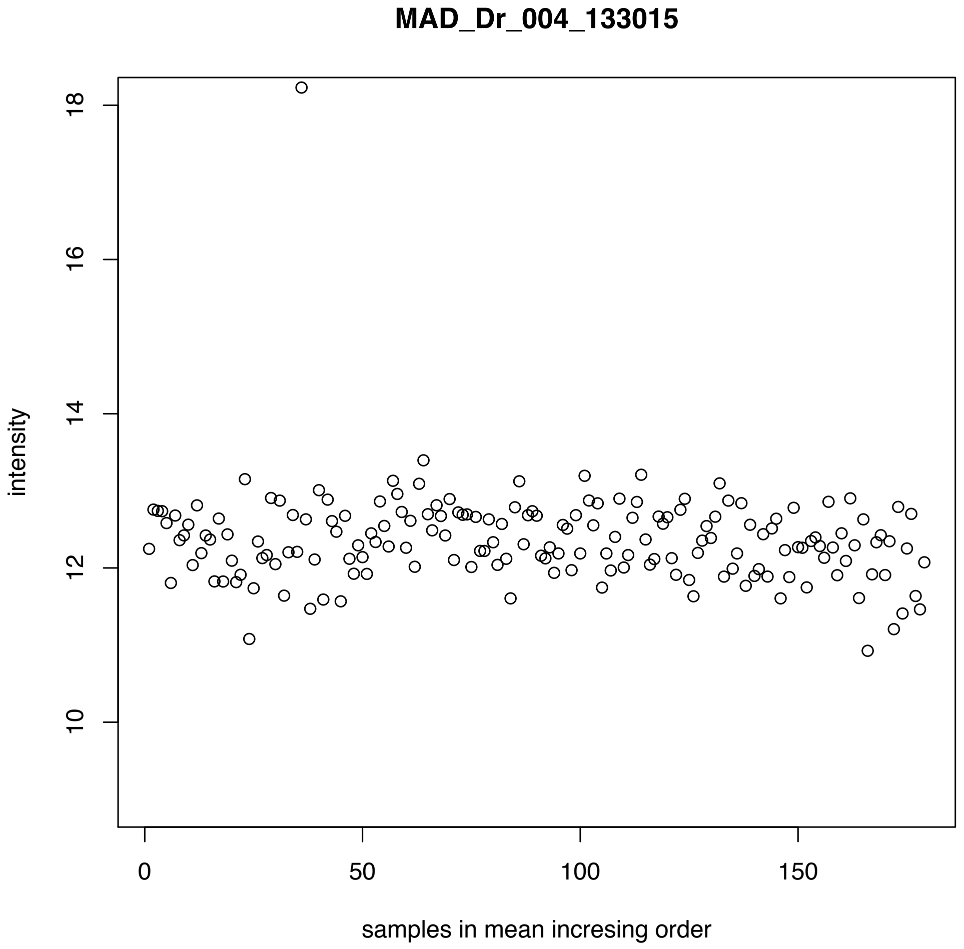
MAD_Dr_004_133015
intensity
10
12
14
16
18
0
50
100
150
samples in mean incresing order

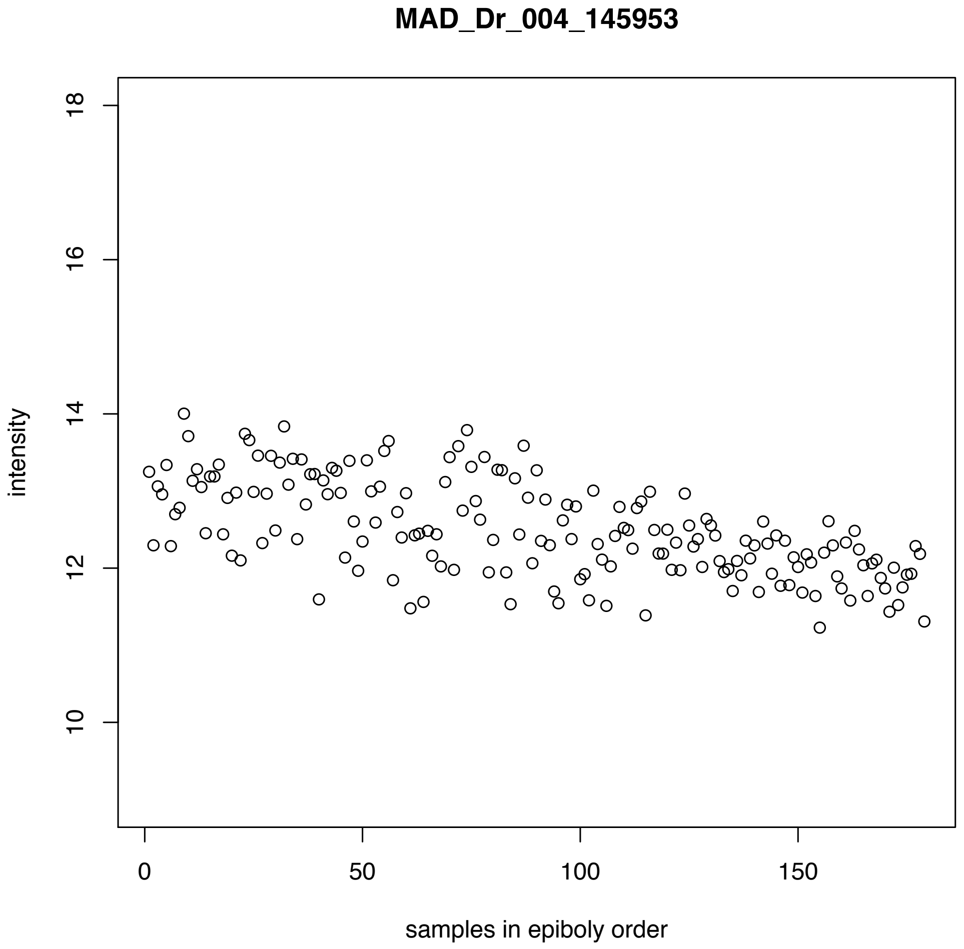
MAD_Dr_004_145953
intensity
18
16
14
12
10
0
50
100
150
samples in epiboly order

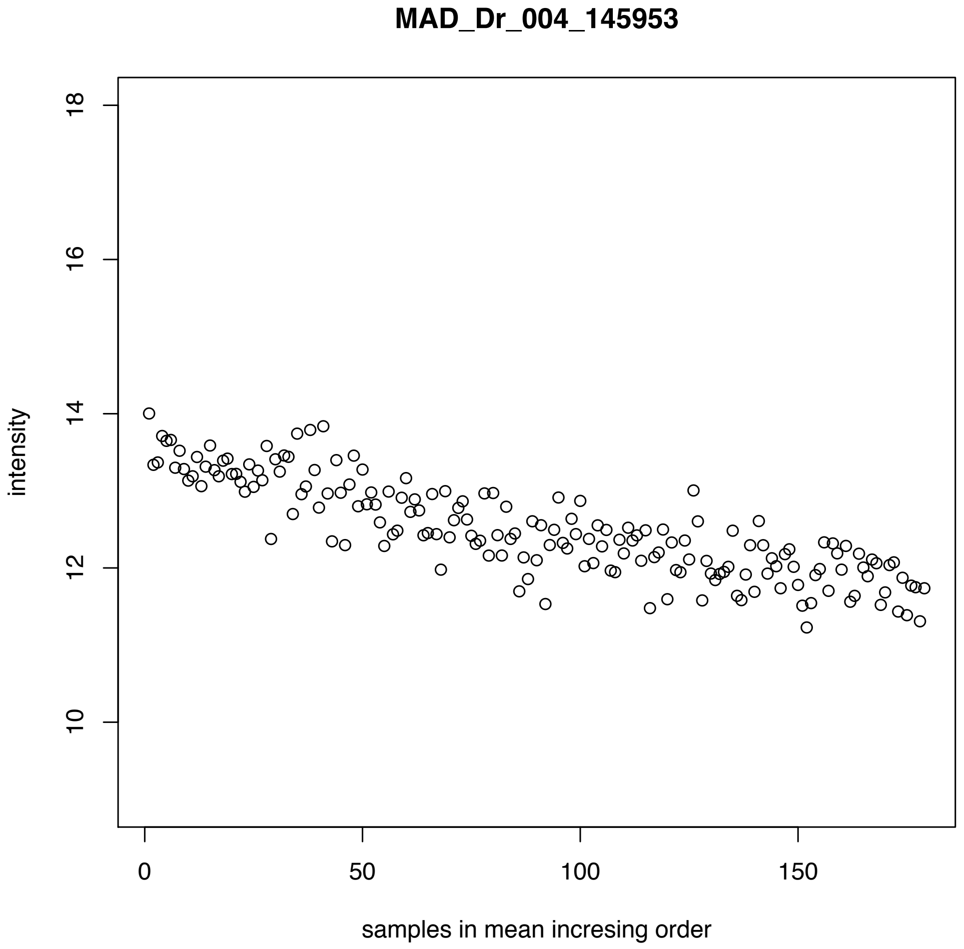
MAD_Dr_004_145953
intensity
18
16
14
12
10
0
50
100
150
samples in mean incresing order

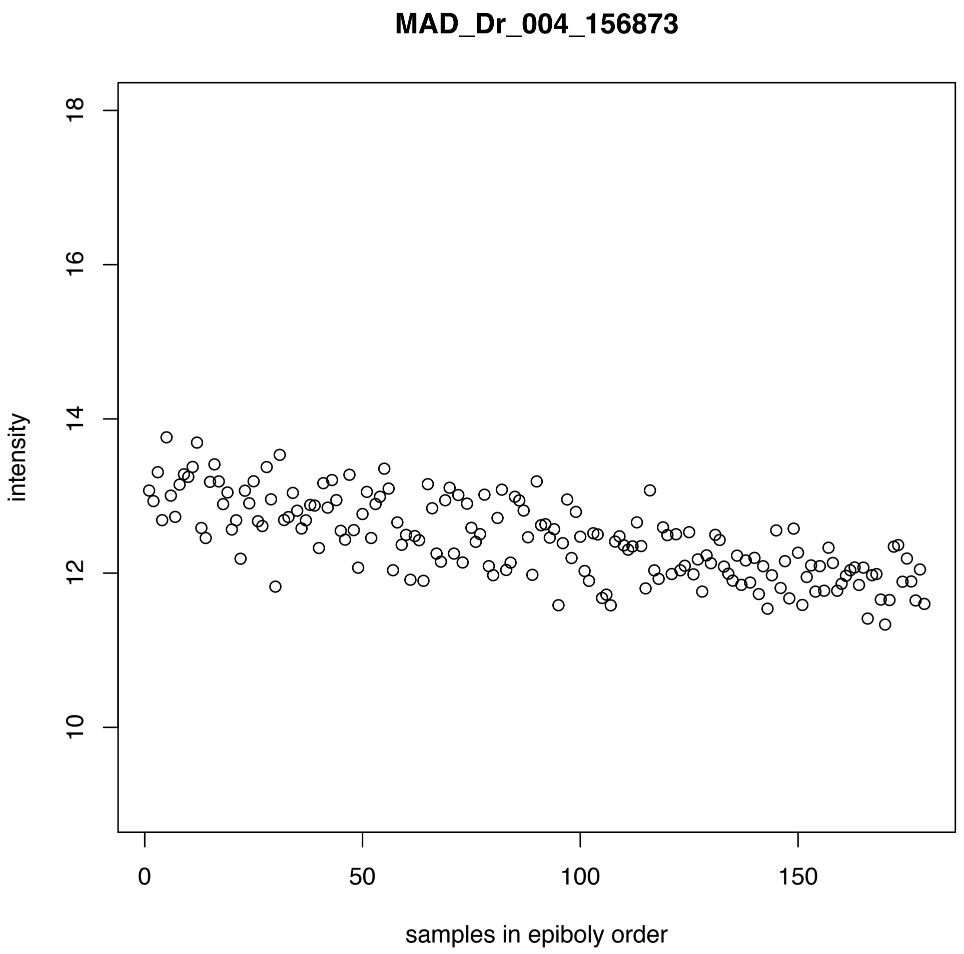
MAD_Dr_004_156873
intensity
10
12
14
16
18
0
50
100
150
samples in epiboly order

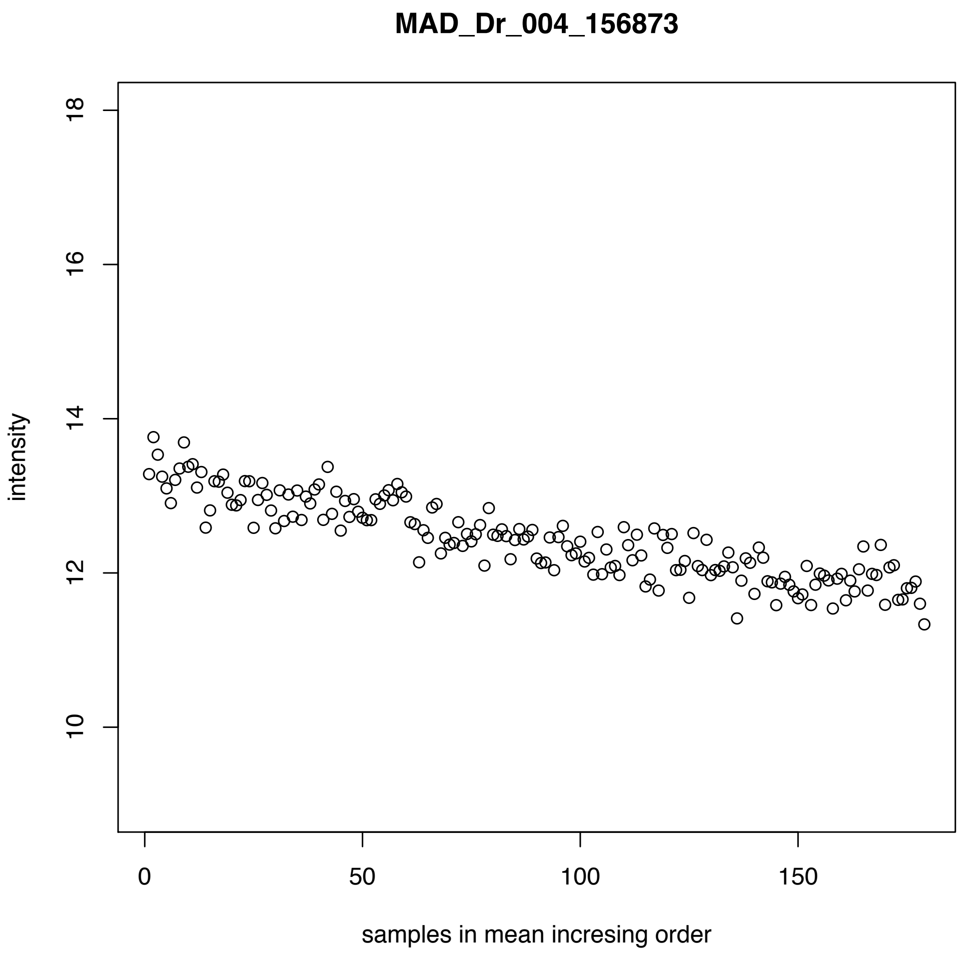
MAD_Dr_004_156873
intensity
10
12
14
16
18
0
50
100
150
samples in mean incresing order

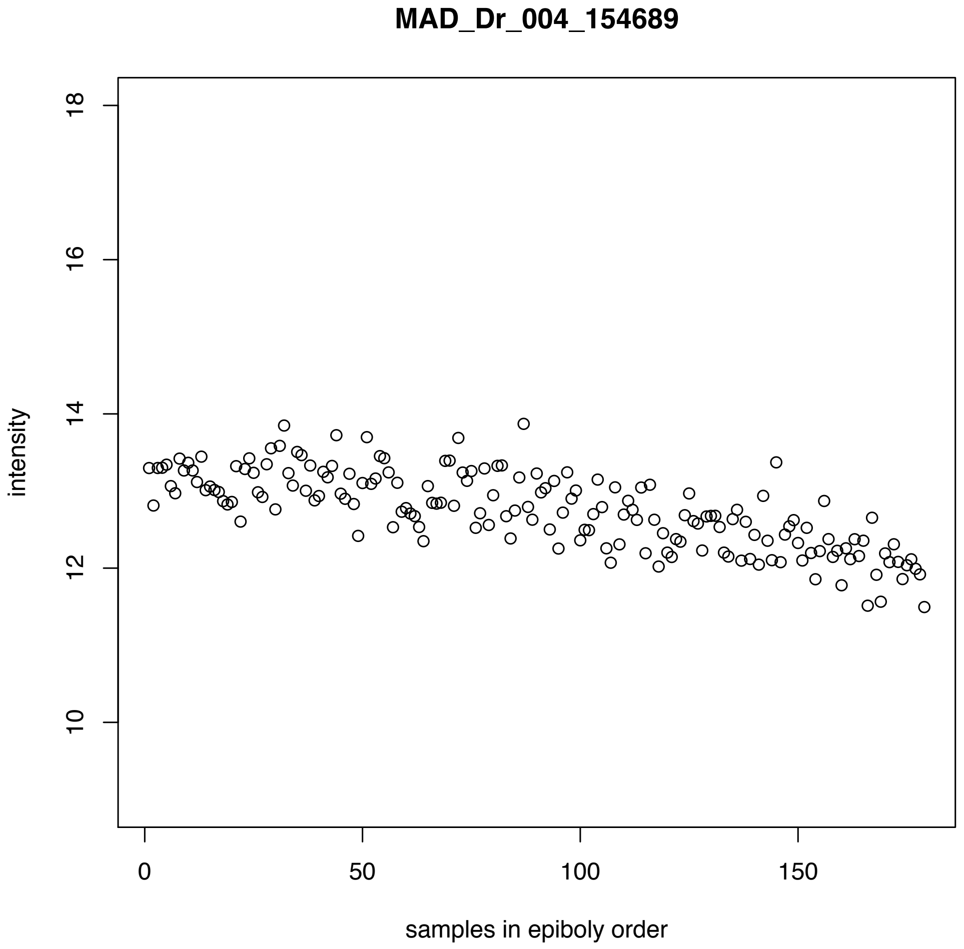

MAD_Dr_004_154689
intensity
10
12
14
16
18
0
50
100
150
samples in epiboly order

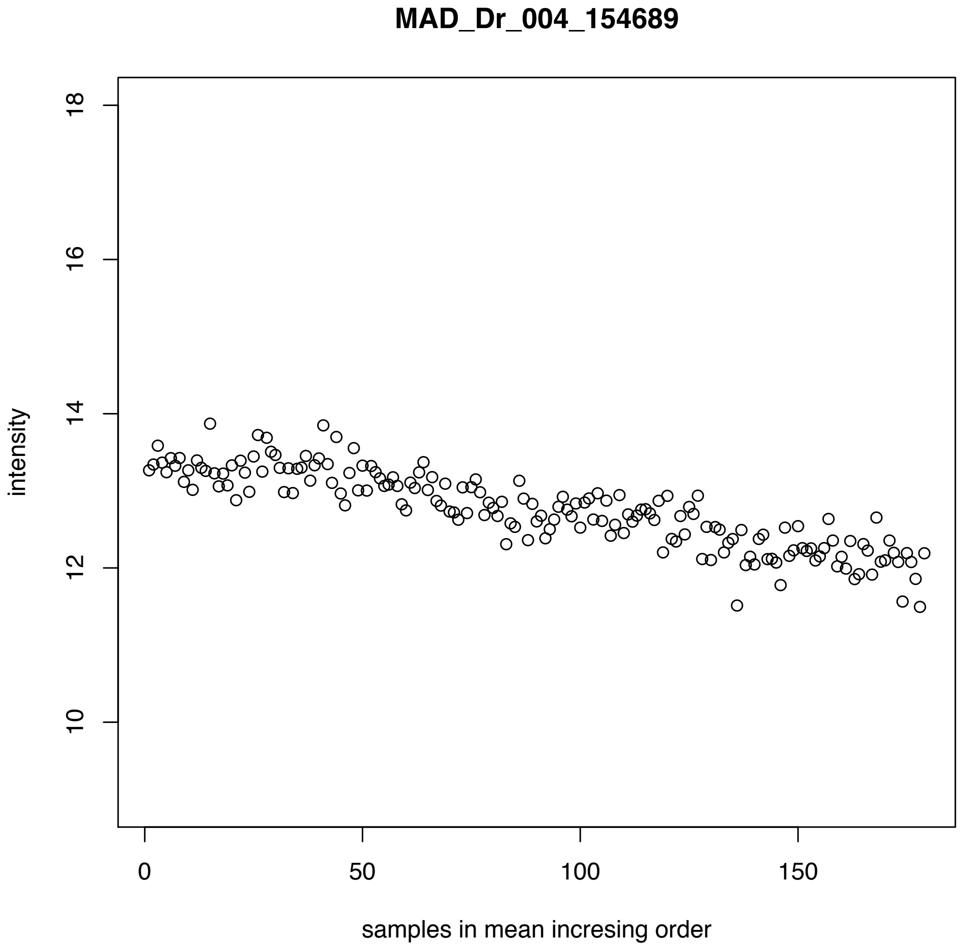

MAD_Dr_004_154689
intensity
10
12
14
16
18
0
50
100
150
samples in mean incresing order

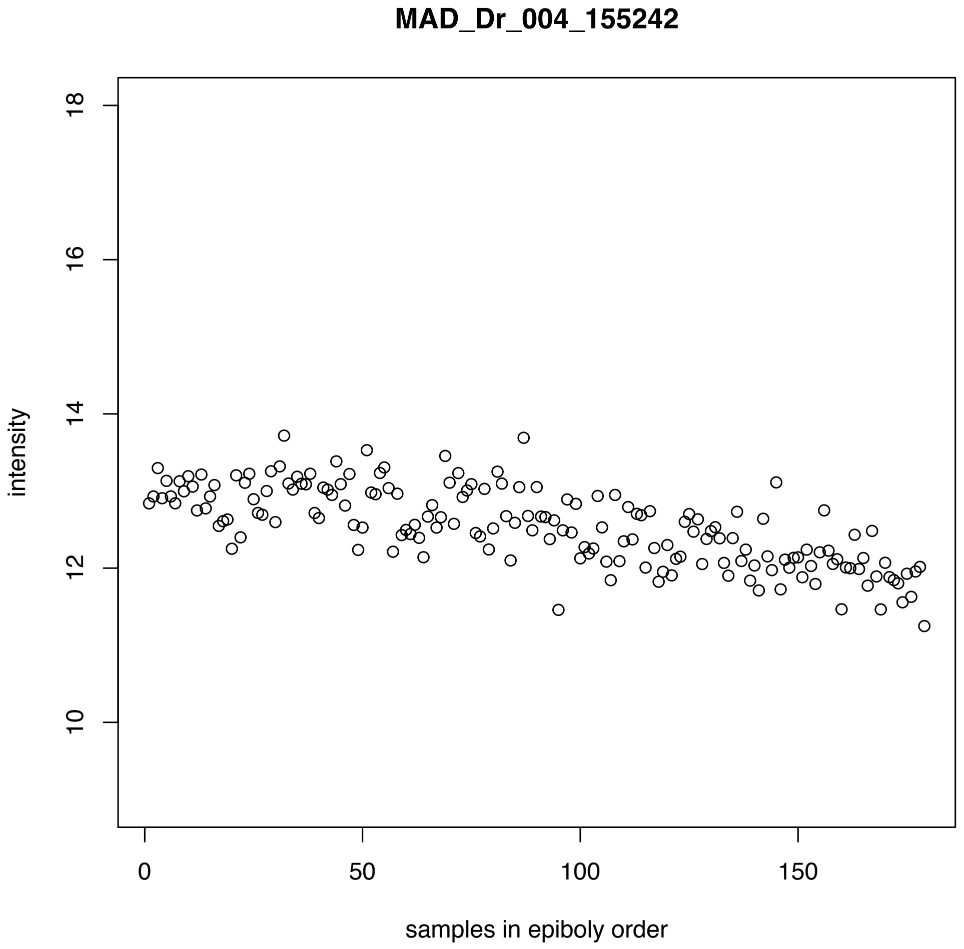
MAD_Dr_004_155242
intensity
18
16
14
12
10
0
50
100
150
samples in epiboly order

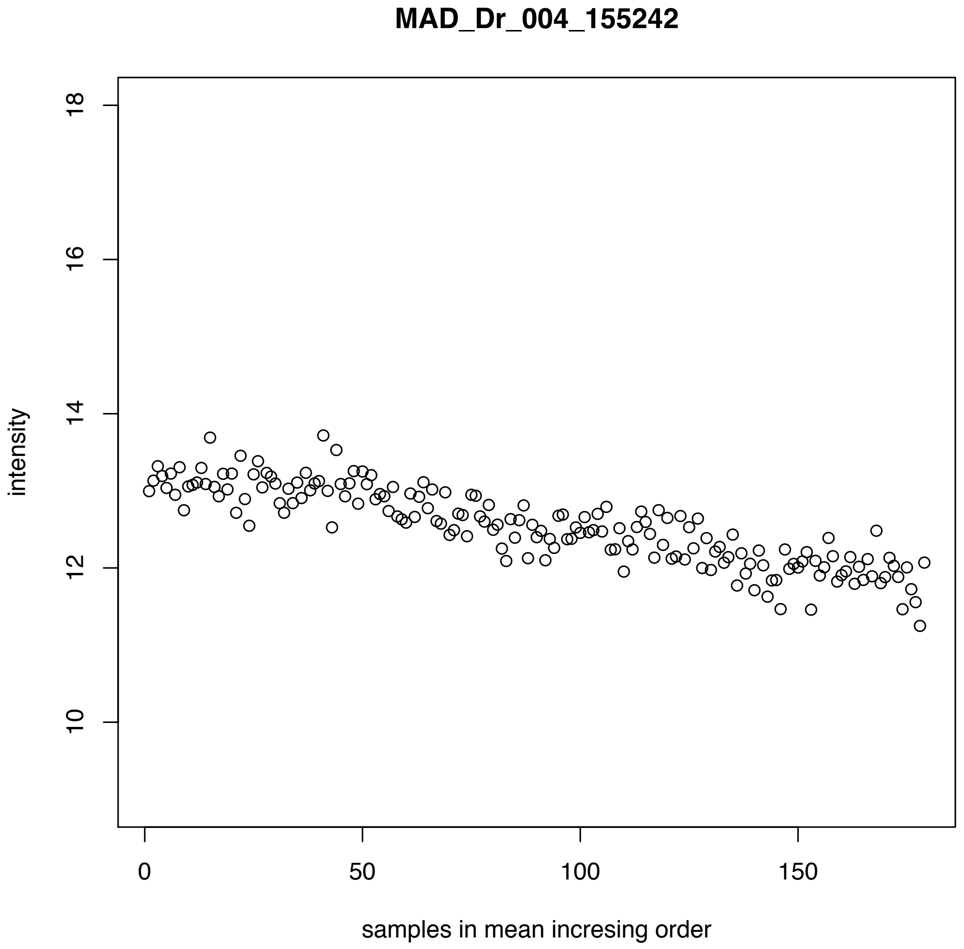
MAD_Dr_004_155242
intensity
18
16
14
12
10
0
50
100
150
samples in mean incresing order

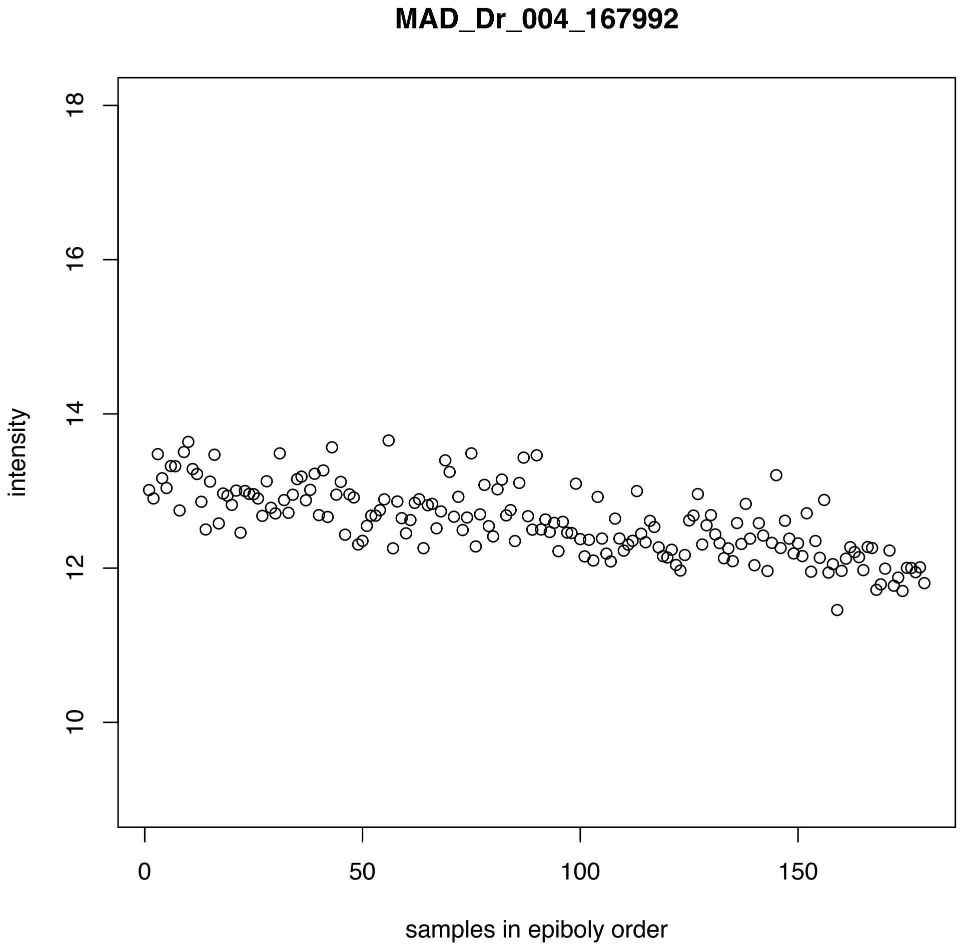
MAD_Dr_004_167992
intensity
10
12
14
16
18
0
50
100
150
samples in epiboly order

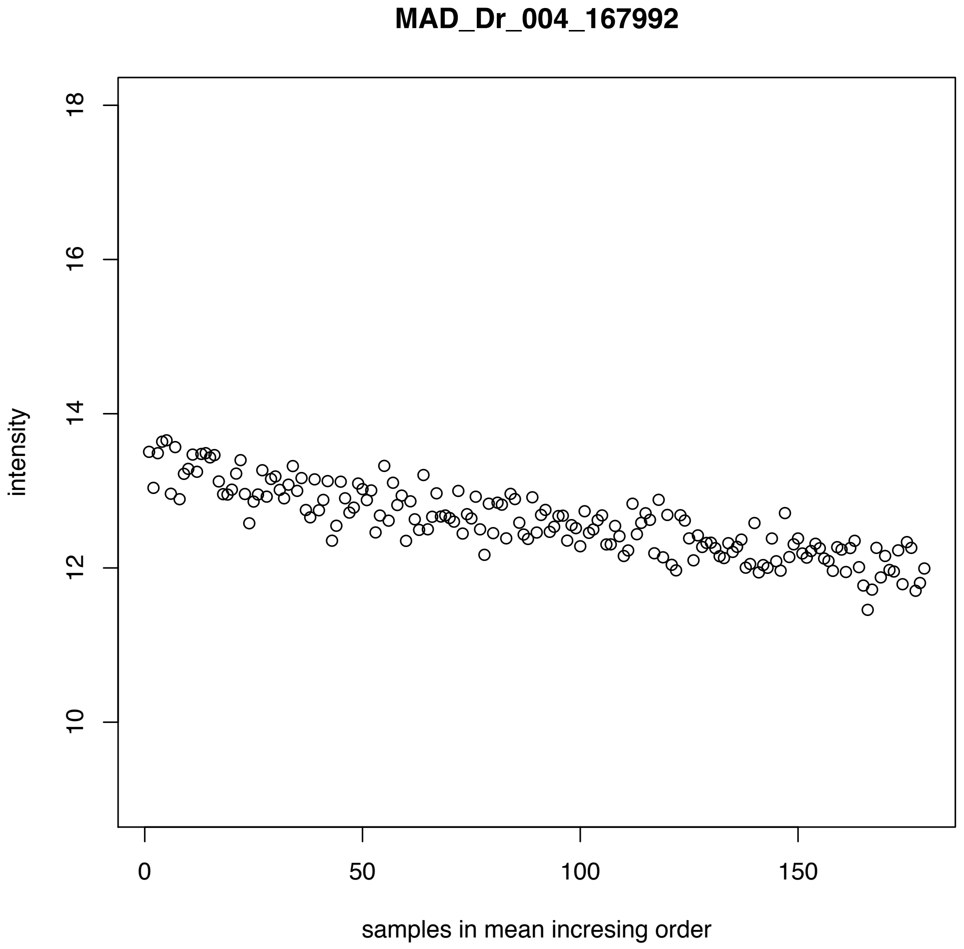
MAD_Dr_004_167992
intensity
10
12
14
16
18
0
50
100
150
samples in mean incresing order

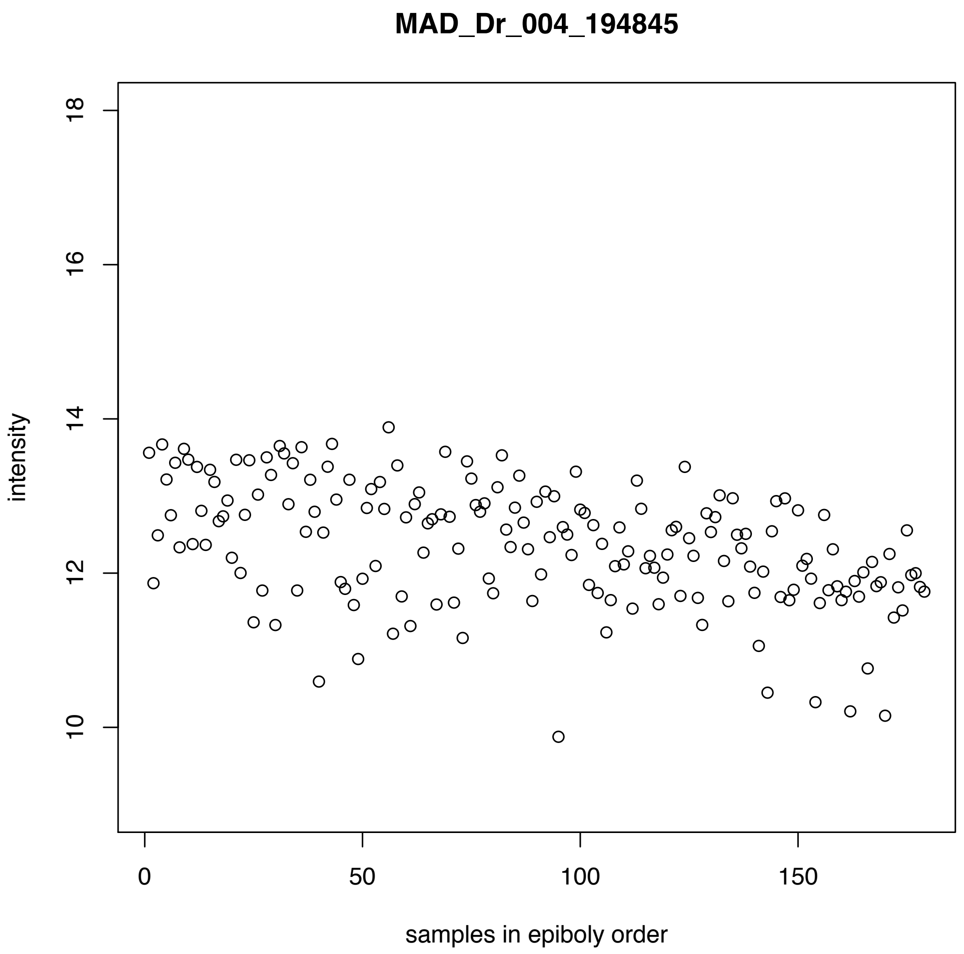
MAD_Dr_004_194845
intensity
10
12
14
16
18
0
50
100
150
samples in epiboly order

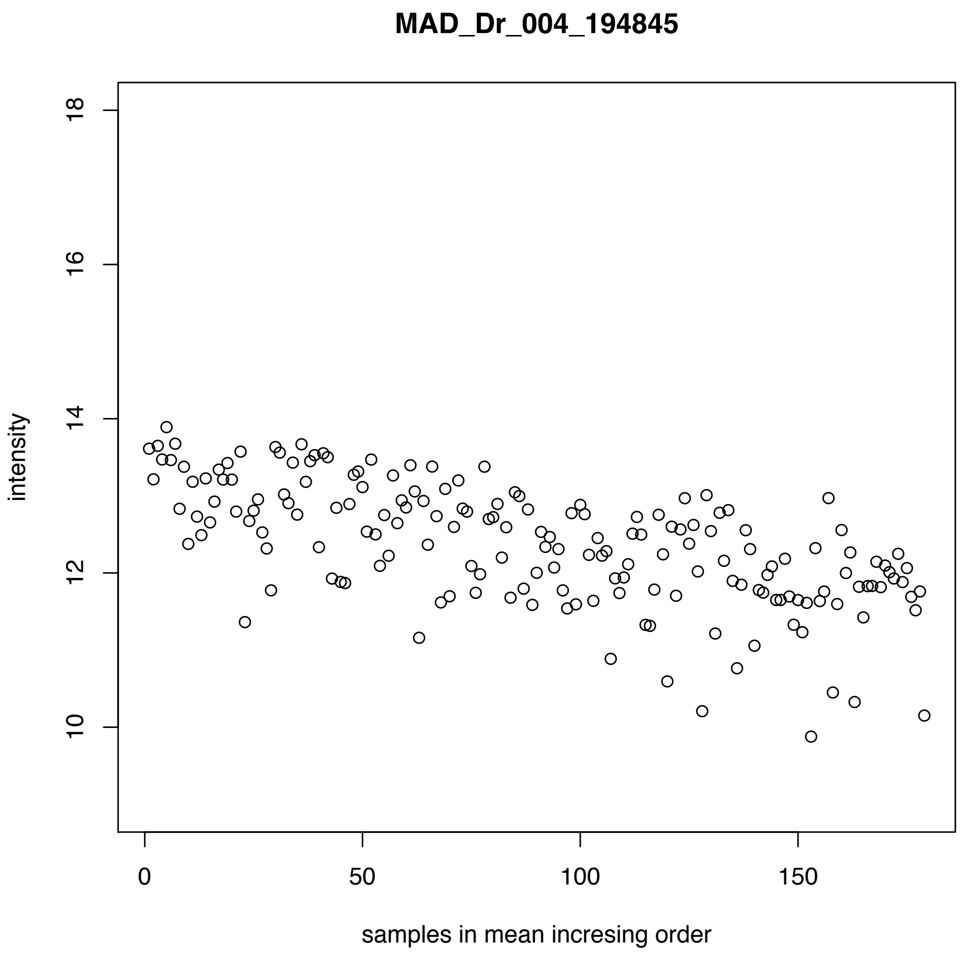
MAD_Dr_004_194845
intensity
10
12
14
16
18
0
50
100
150
samples in mean incresing order

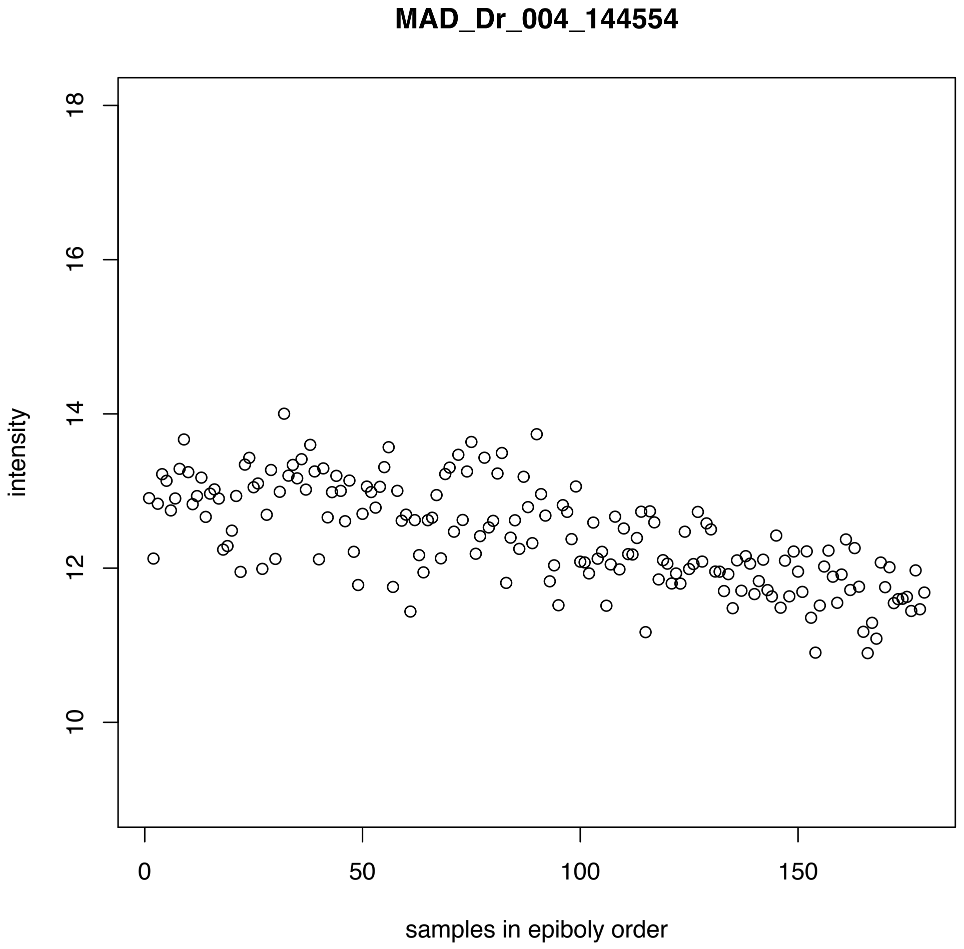
MAD_Dr_004_144554
intensity
10
12
14
16
18
0
50
100
150
samples in epiboly order

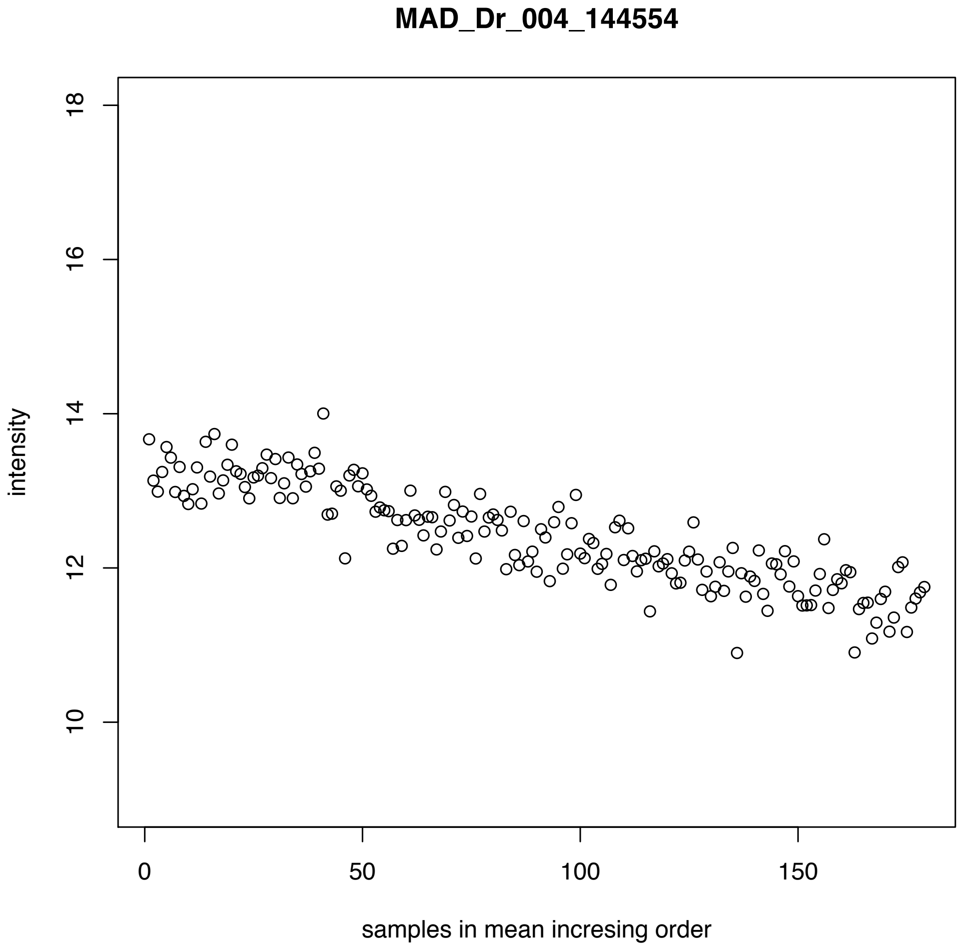
MAD_Dr_004_144554
intensity
10
12
14
16
18
0
50
100
150
samples in mean incresing order

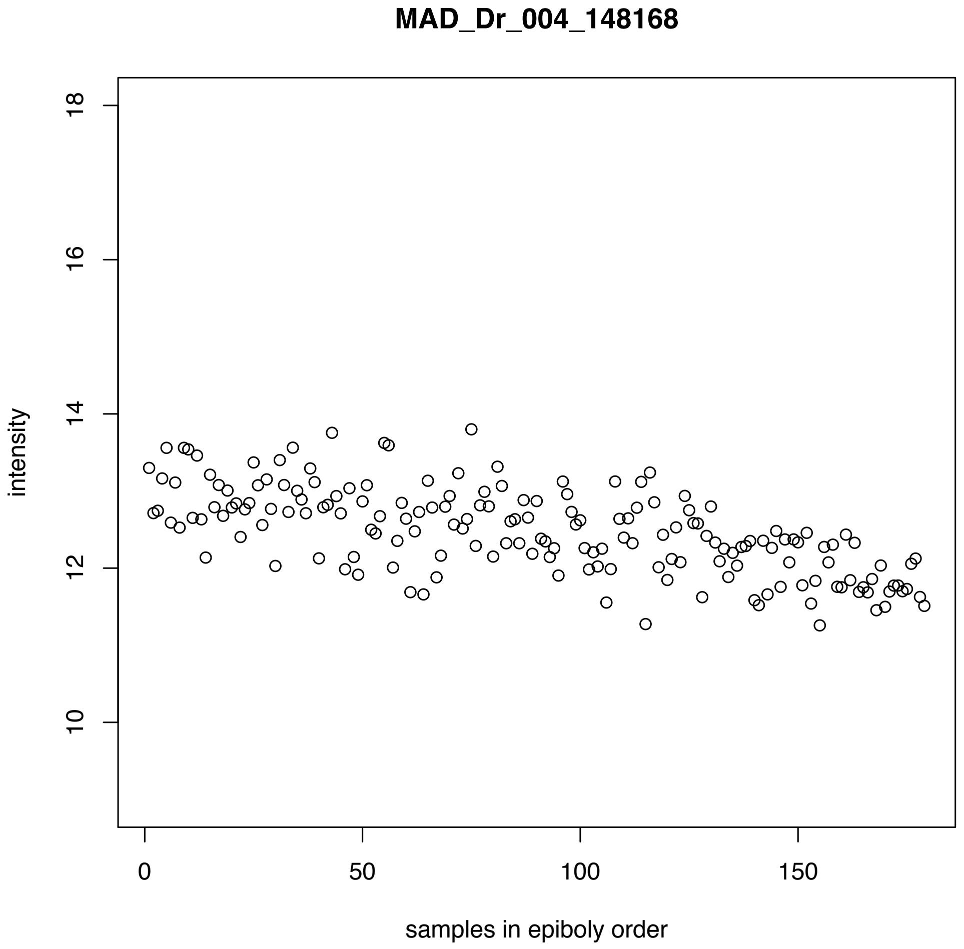
MAD_Dr_004_148168
intensity
samples in epiboly order
10
12
14
16
18
0
50
100
150

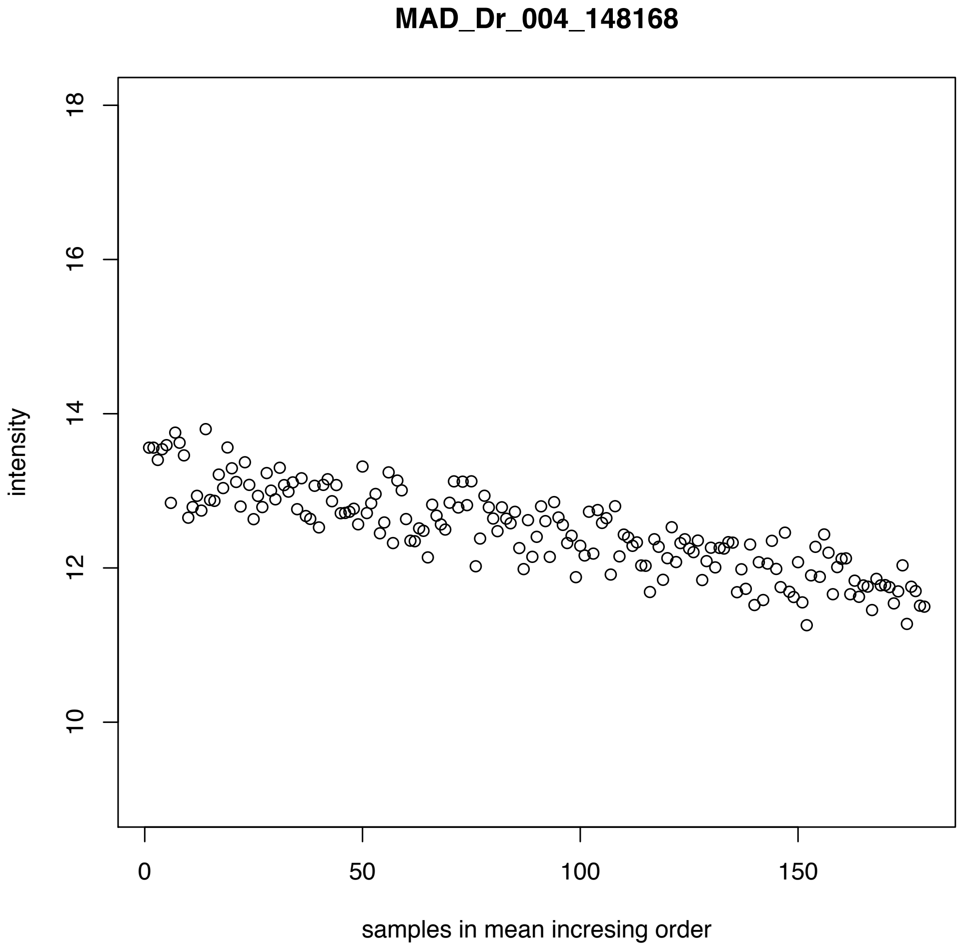
MAD_Dr_004_148168
intensity
samples in mean incresing order
10
12
14
16
18
0
50
100
150

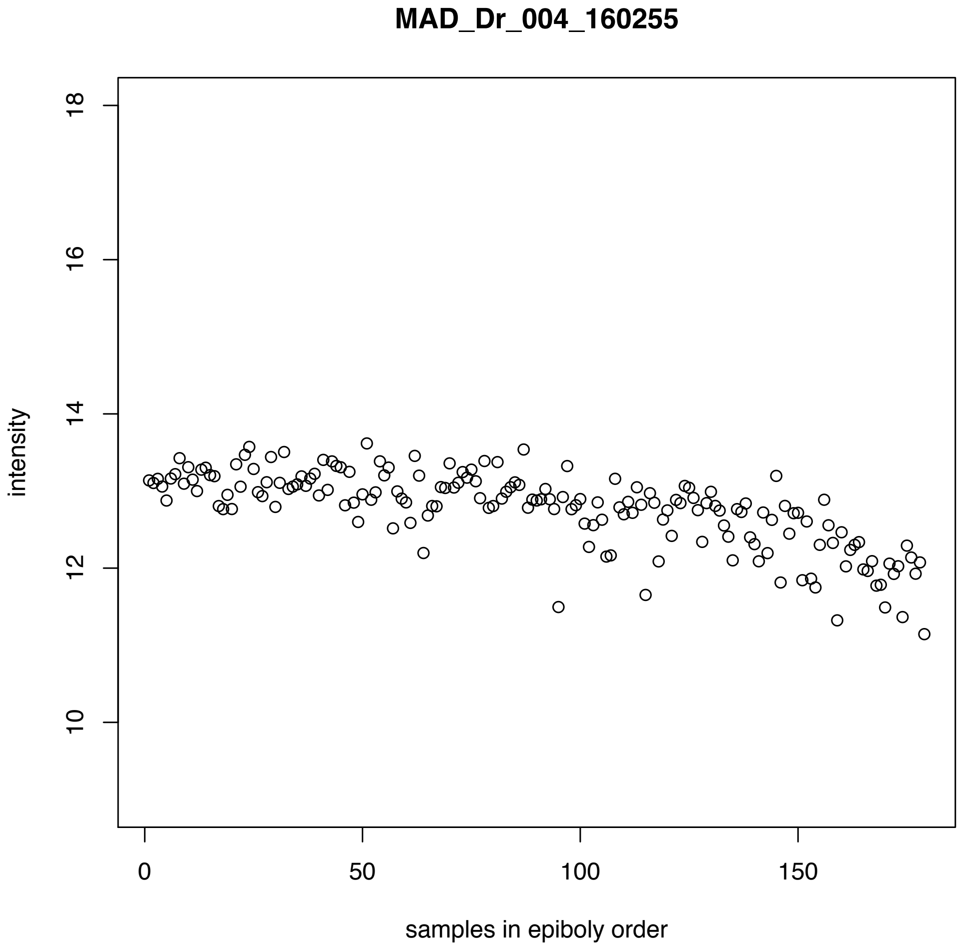
MAD_Dr_004_160255
intensity
samples in epiboly order
10
12
14
16
18
0
50
100
150

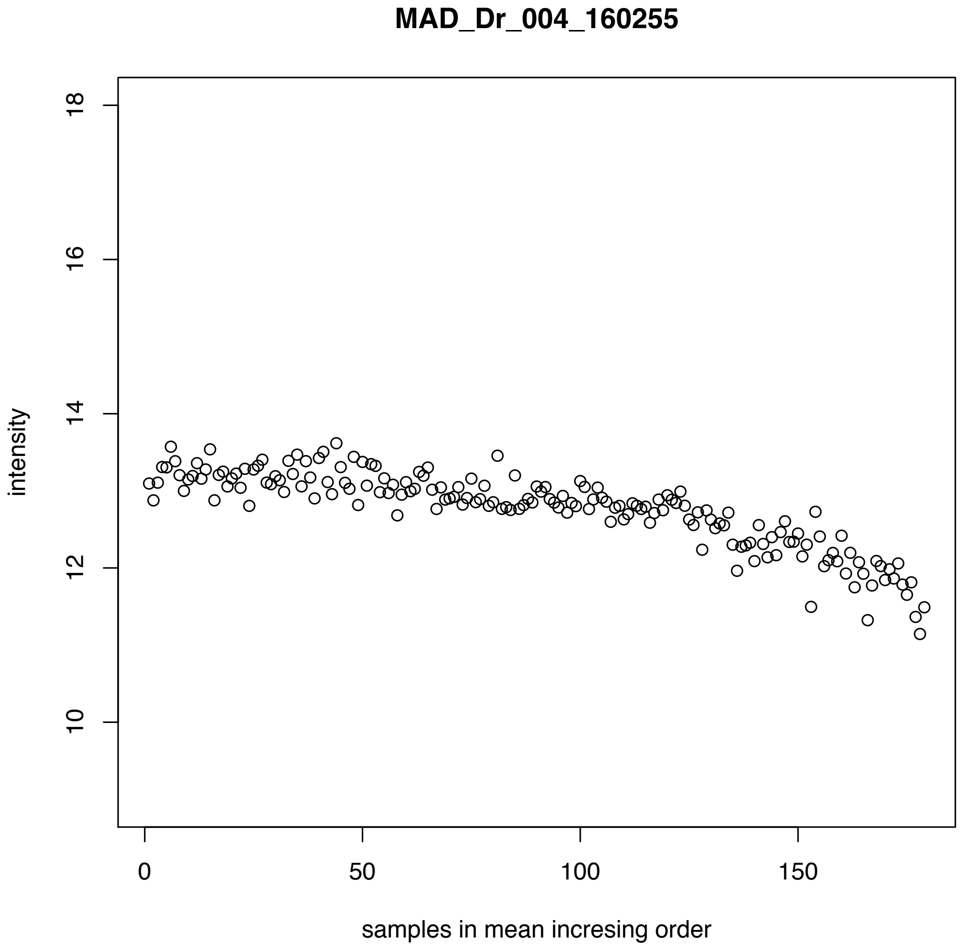
MAD_Dr_004_160255
intensity
samples in mean incresing order
10
12
14
16
18
0
50
100
150

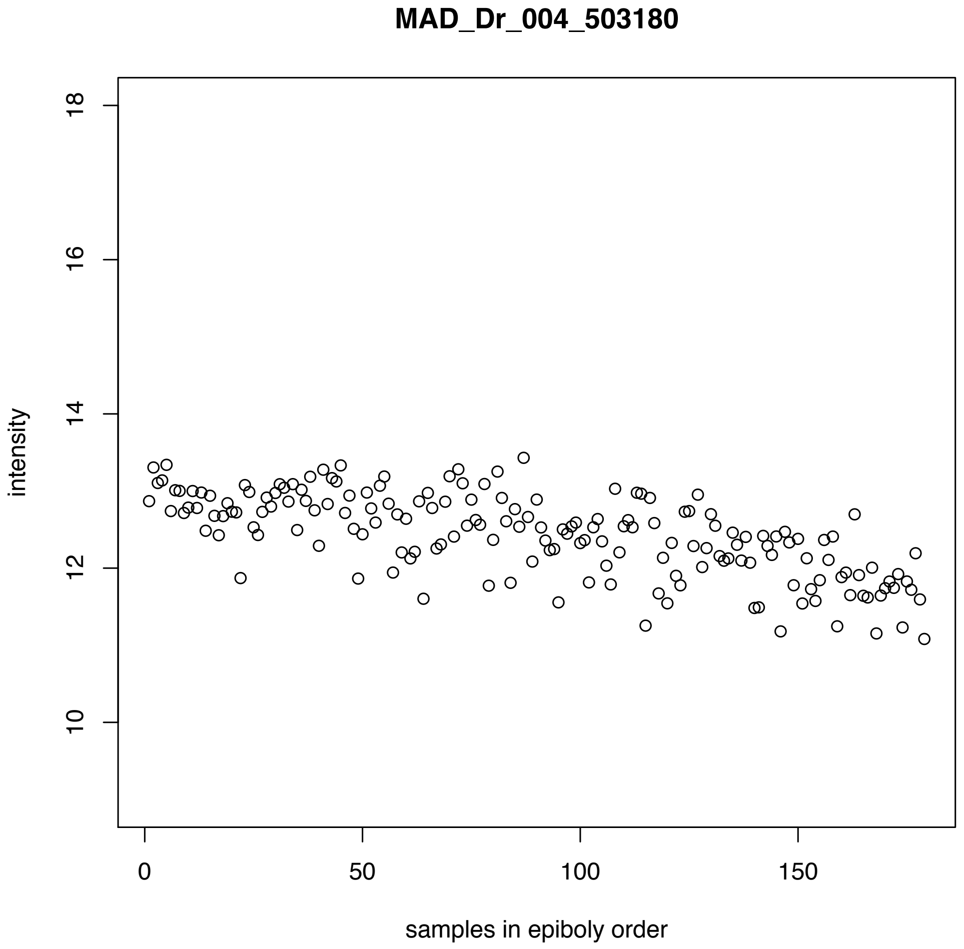
MAD_Dr_004_503180
intensity
10
12
14
16
18
0
50
100
150
samples in epiboly order

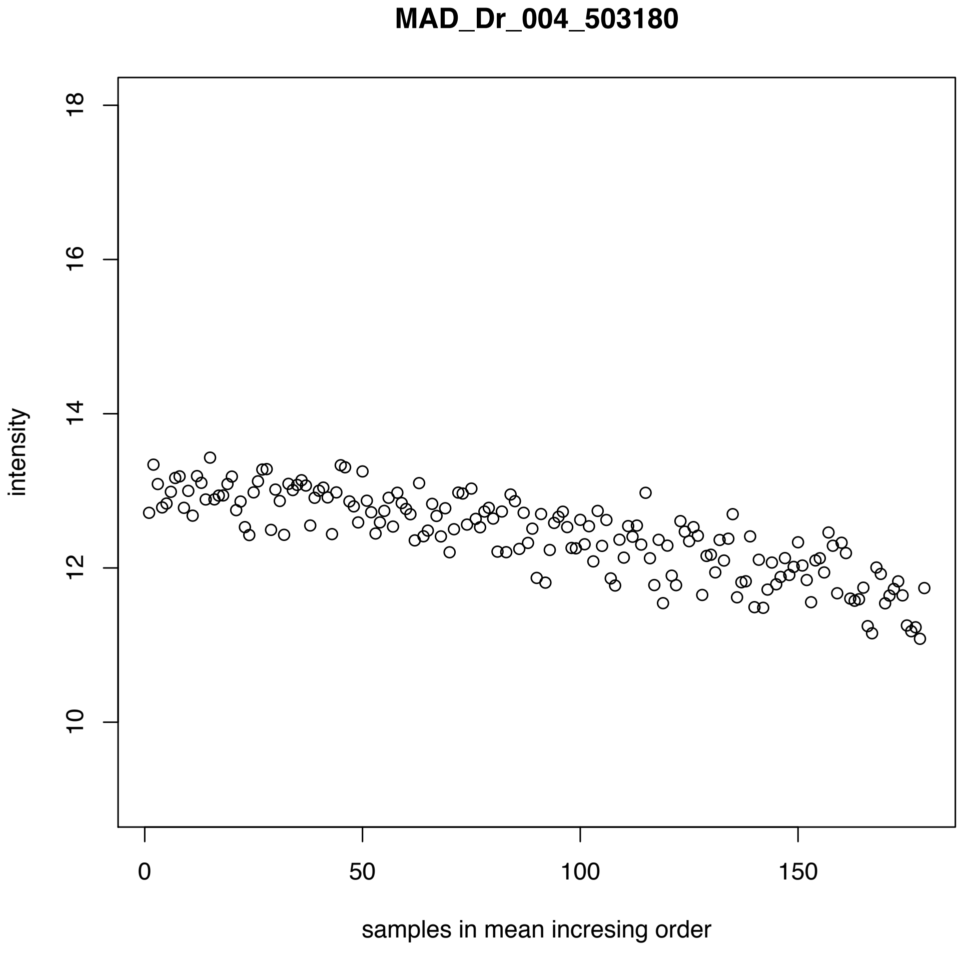
MAD_Dr_004_503180
intensity
10
12
14
16
18
0
50
100
150
samples in mean incresing order

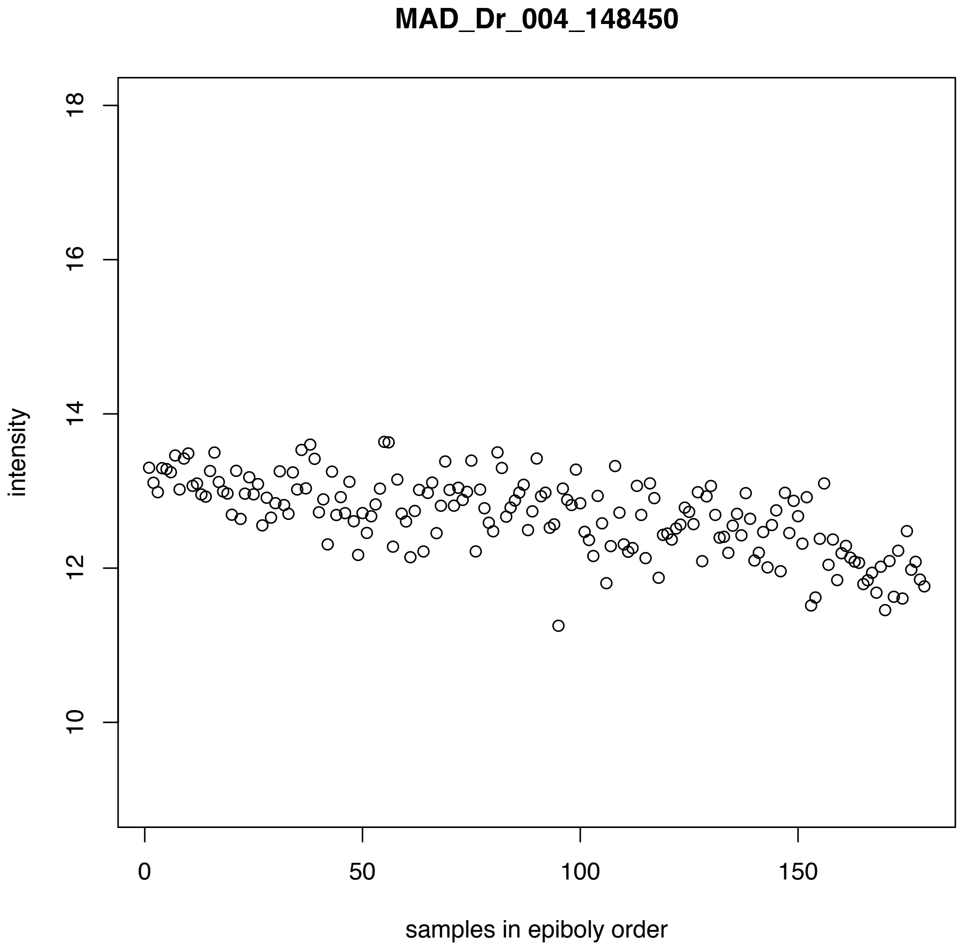
MAD_Dr_004_148450
intensity
10
12
14
16
18
0
50
100
150
samples in epiboly order

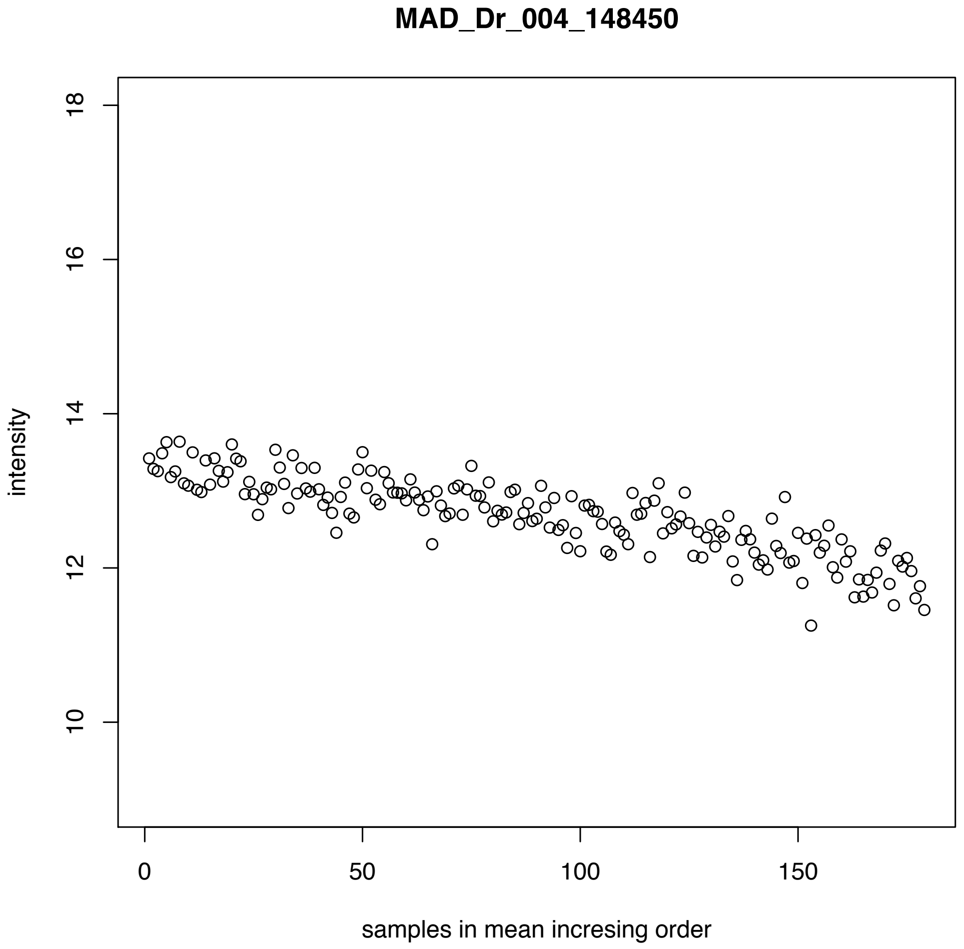
MAD_Dr_004_148450
intensity
10
12
14
16
18
0
50
100
150
samples in mean incresing order

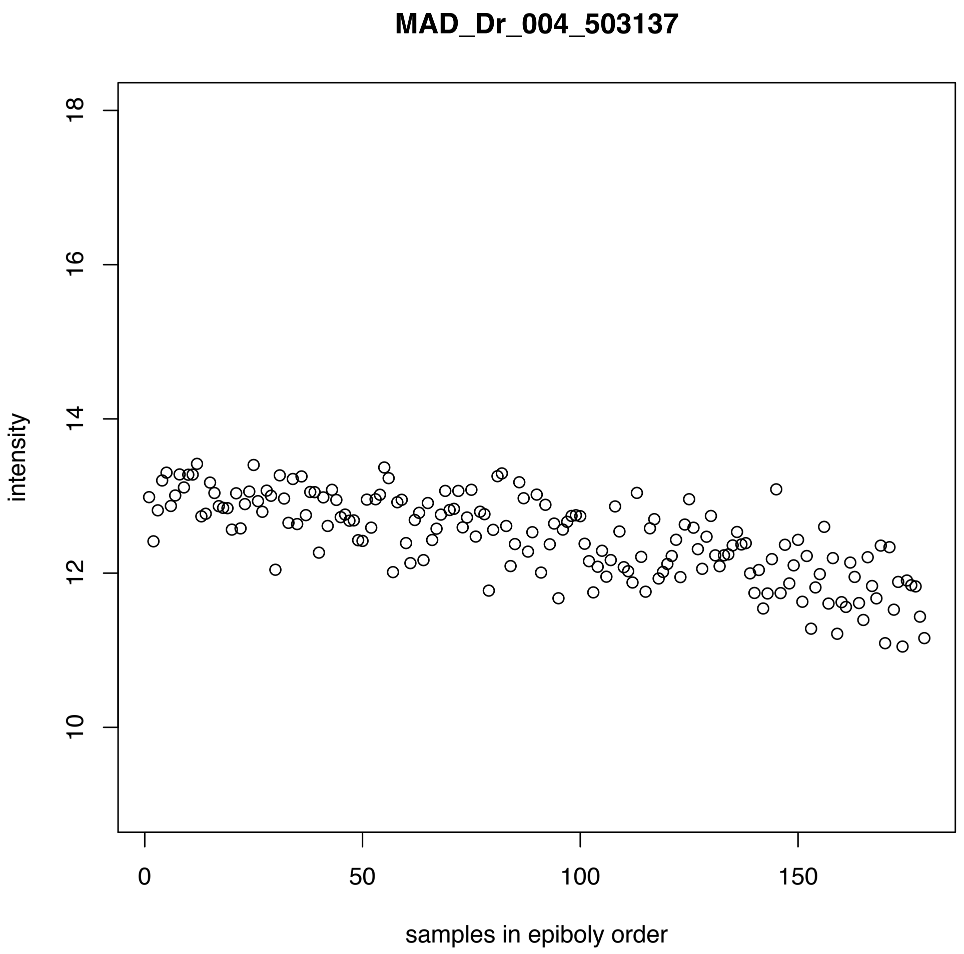
MAD_Dr_004_503137
intensity
18
16
14
12
10
0
50
100
150
samples in epiboly order

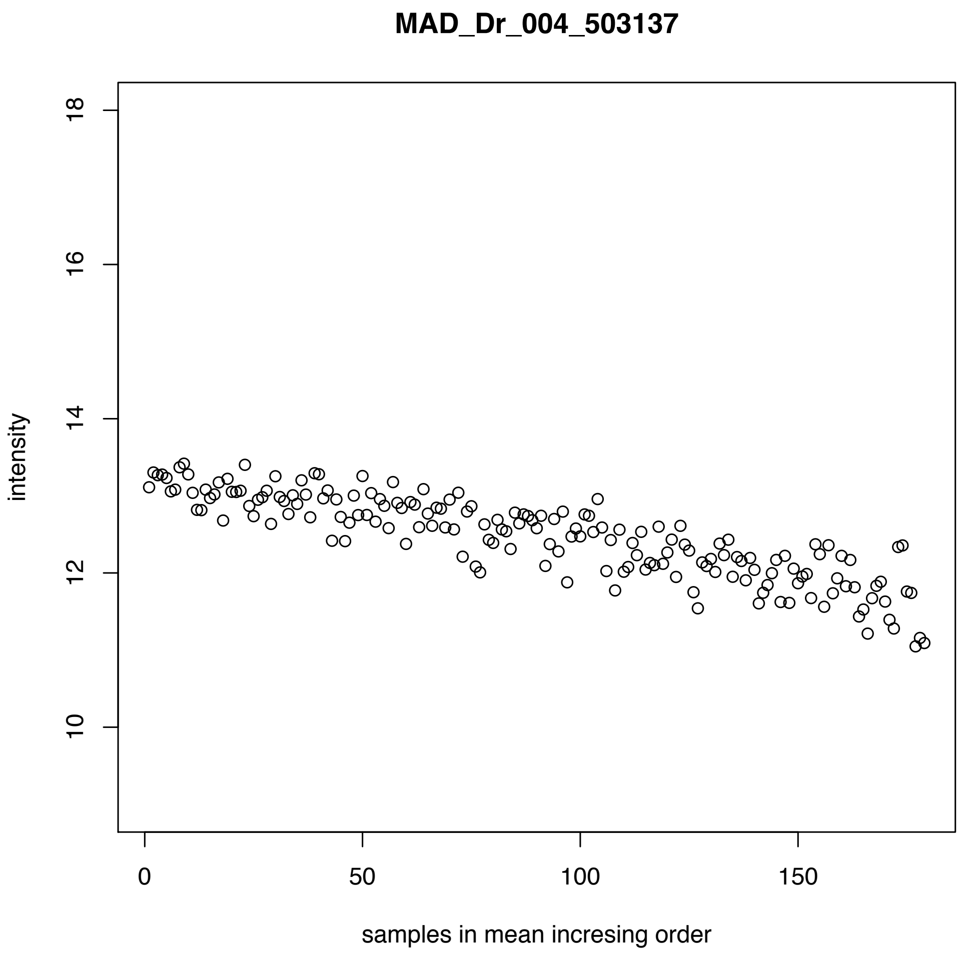
MAD_Dr_004_503137
intensity
18
16
14
12
10
0
50
100
150
samples in mean incresing order

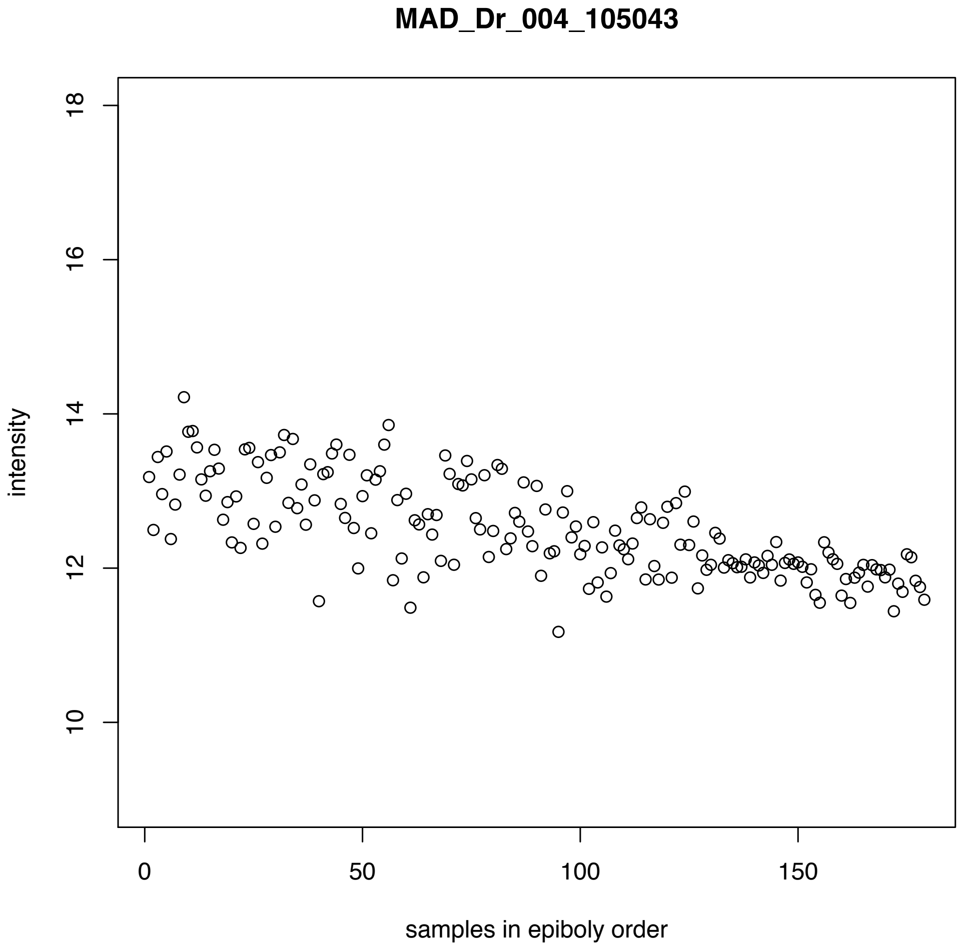
MAD_Dr_004_105043
intensity
18
16
14
12
10
0
50
100
150
samples in epiboly order

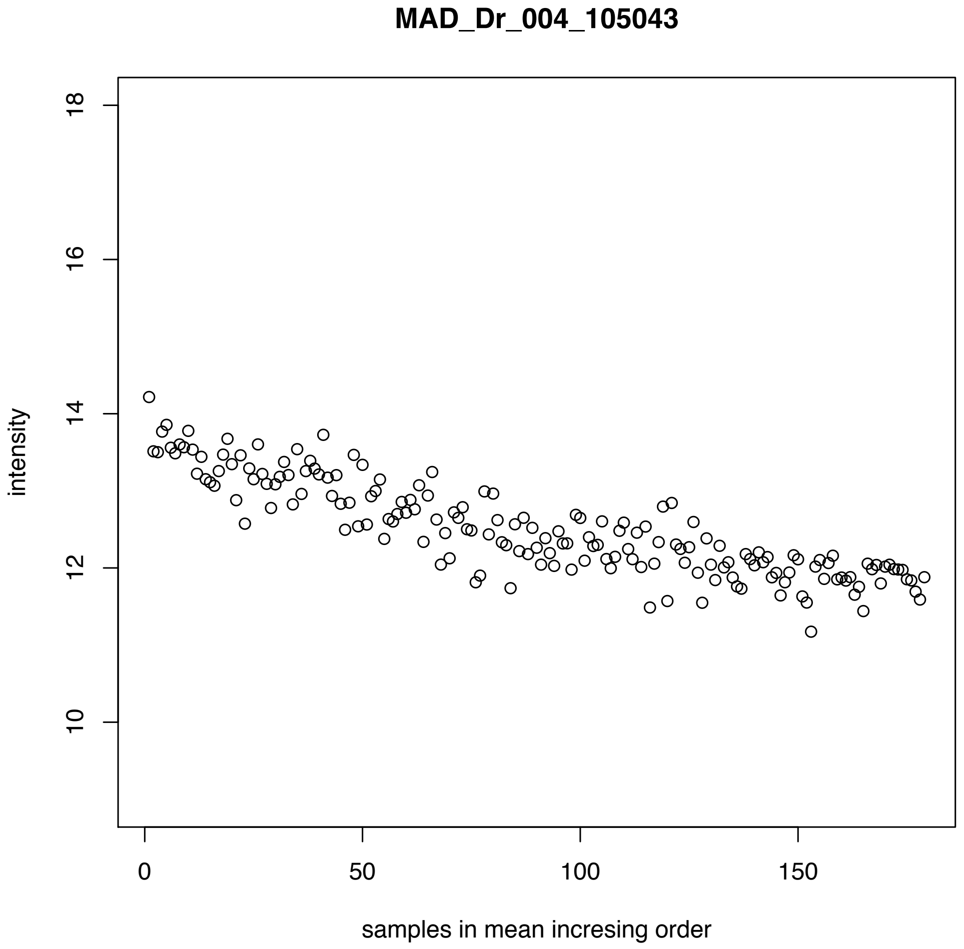
MAD_Dr_004_105043
intensity
18
16
14
12
10
0
50
100
150
samples in mean incresing order

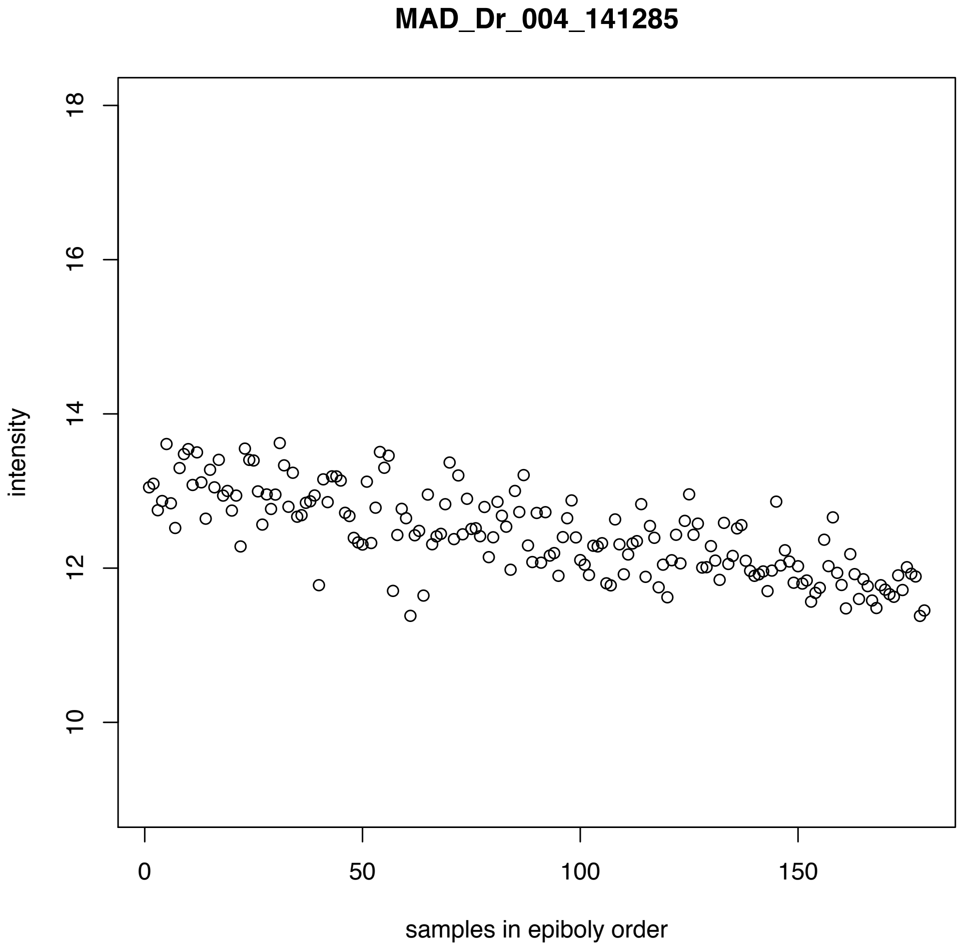
MAD_Dr_004_141285
intensity
18
16
14
12
10
0
50
100
150
samples in epiboly order

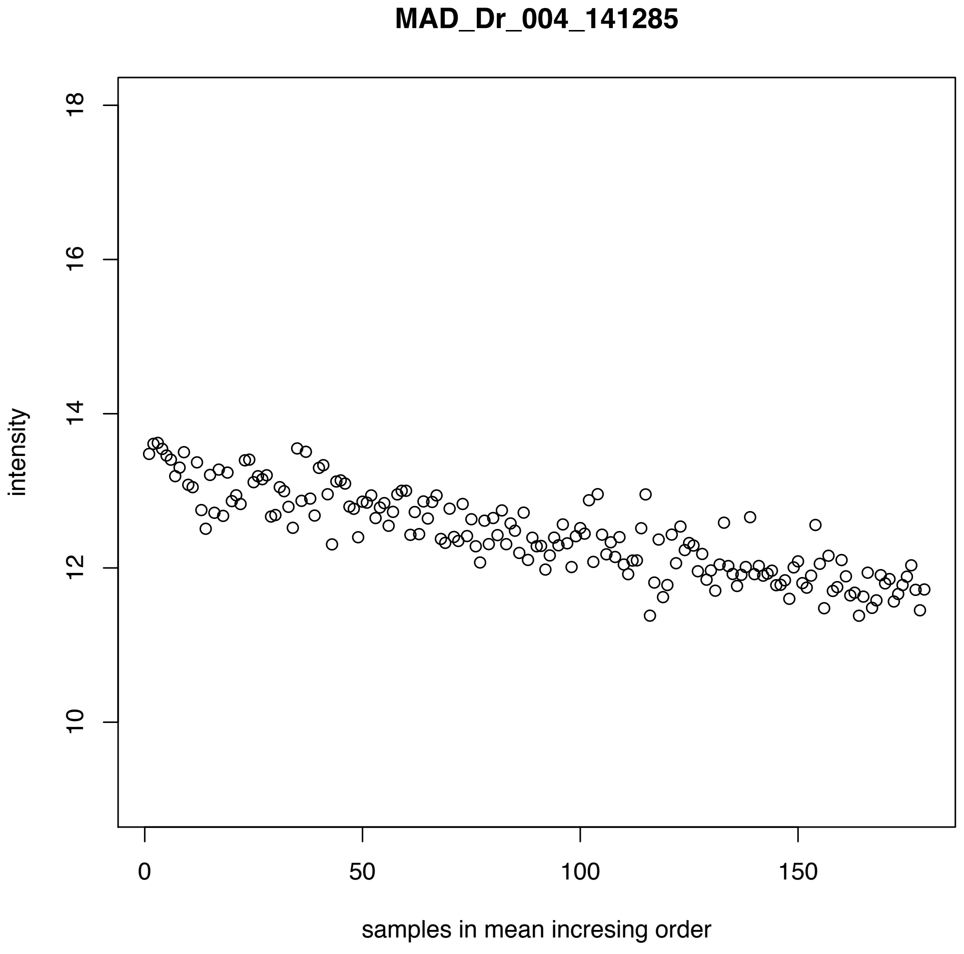
MAD_Dr_004_141285
intensity
18
16
14
12
10
0
50
100
150
samples in mean incresing order

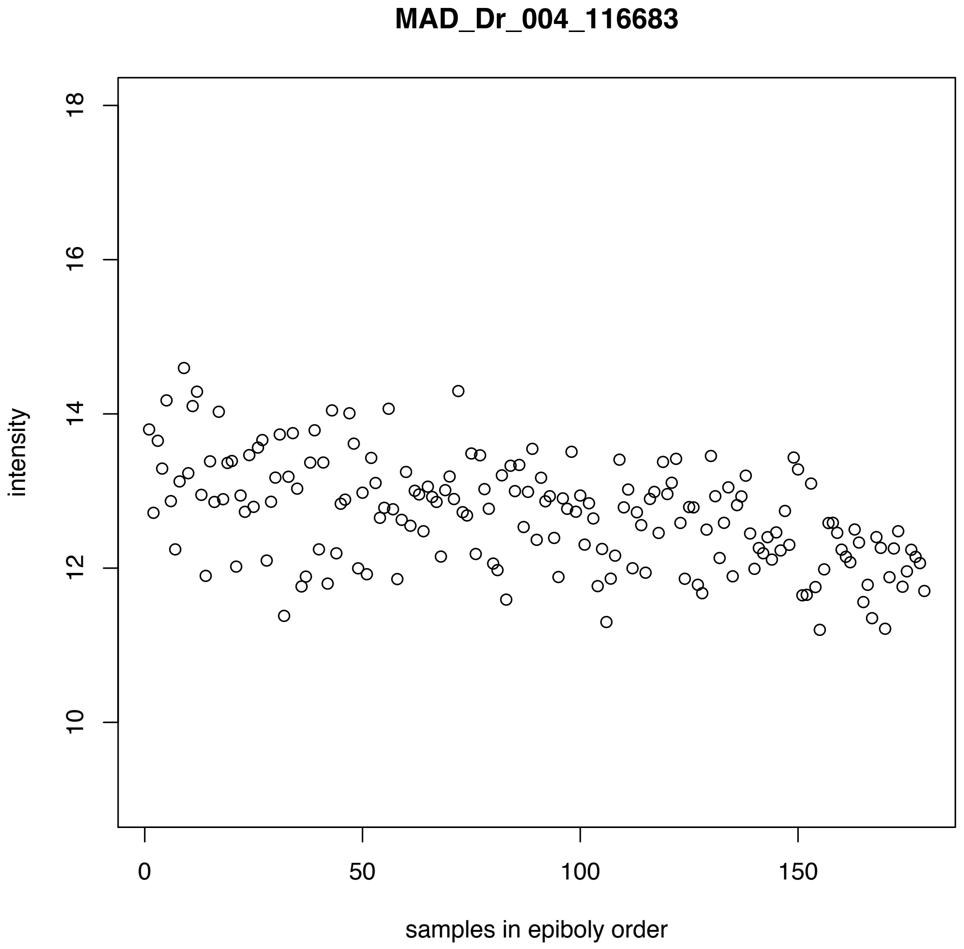
MAD_Dr_004_116683
intensity
10
12
14
16
18
0
50
100
150
samples in epiboly order

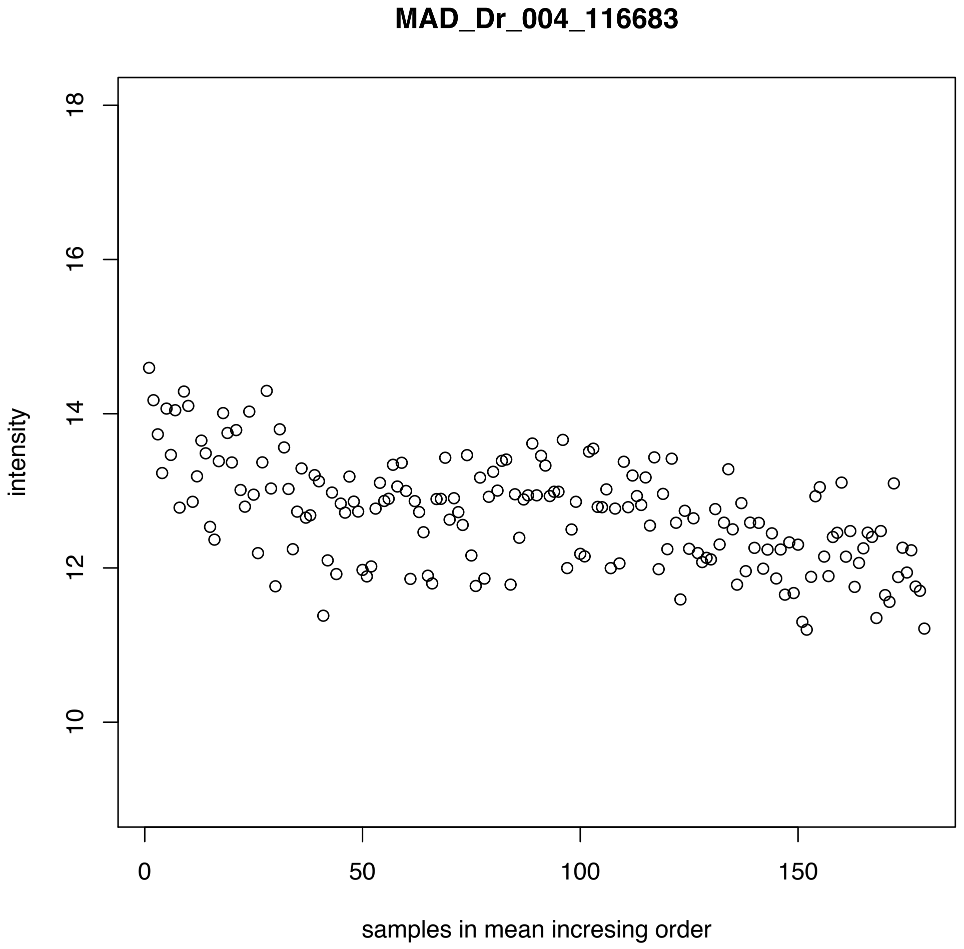
MAD_Dr_004_116683
intensity
10
12
14
16
18
0
50
100
150
samples in mean incresing order

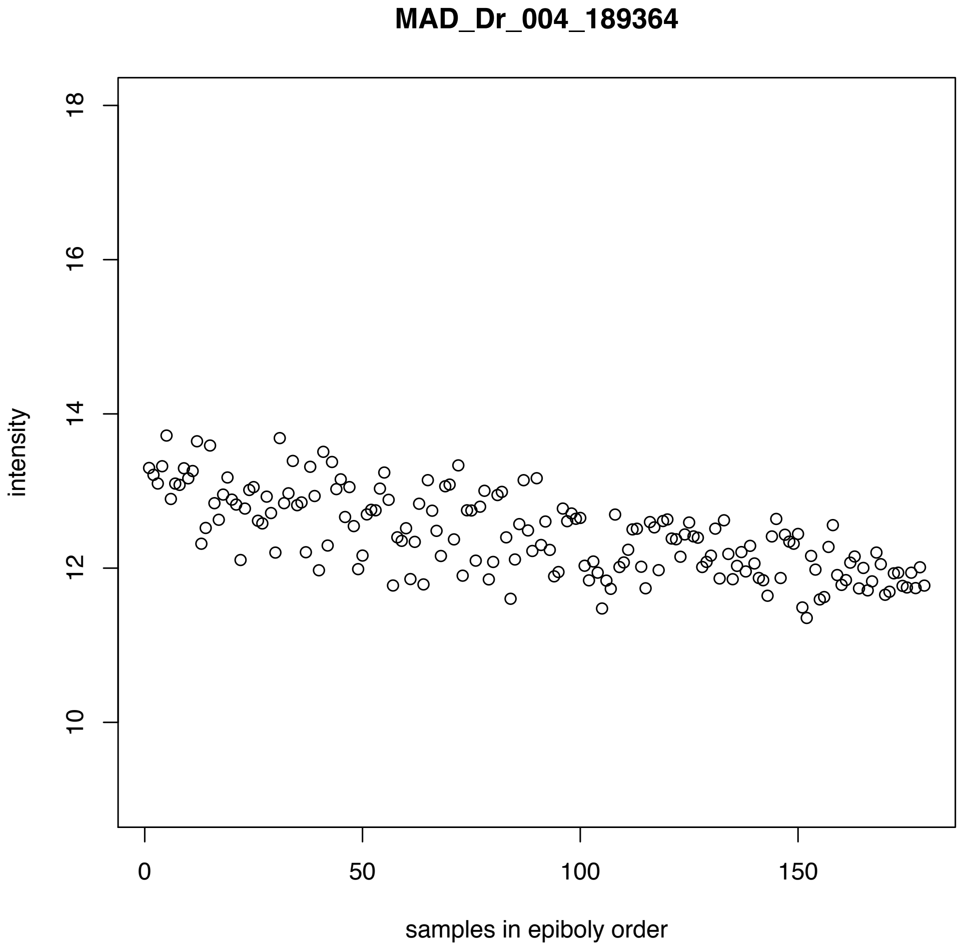
MAD_Dr_004_189364
intensity
10
12
14
16
18
0
50
100
150
samples in epiboly order

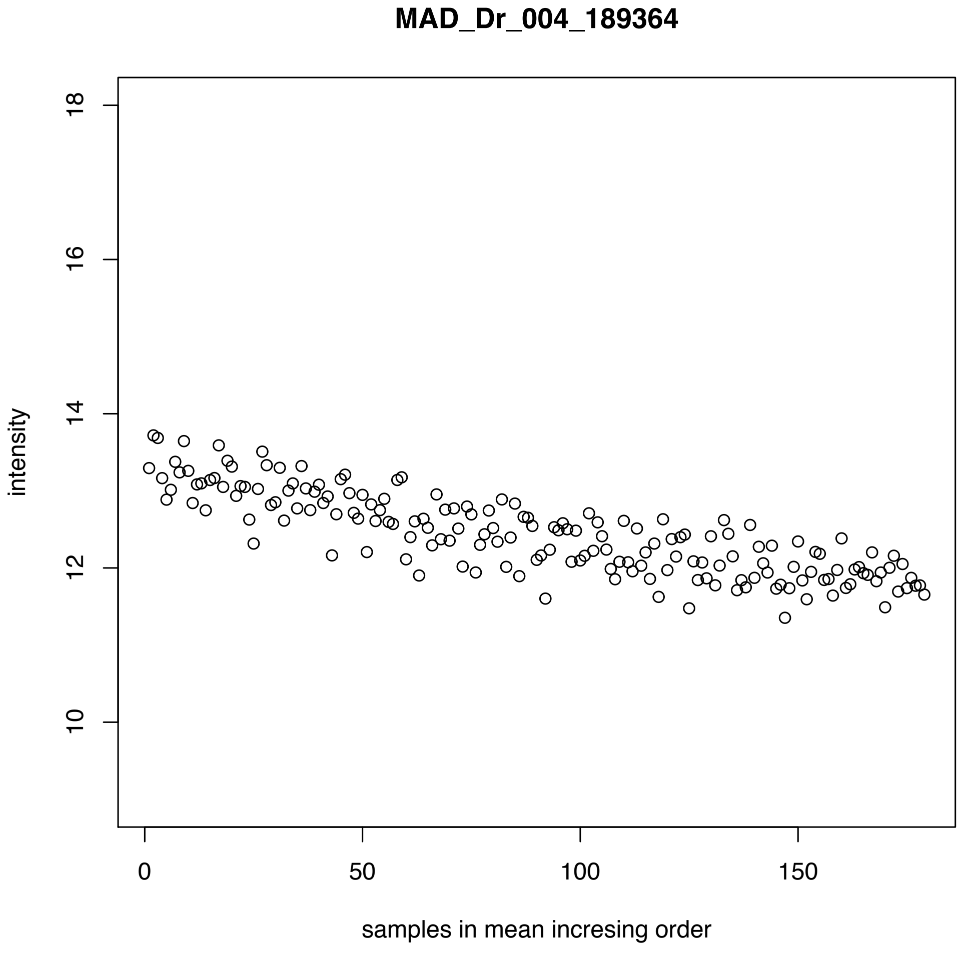
MAD_Dr_004_189364
intensity
10
12
14
16
18
0
50
100
150
samples in mean incresing order

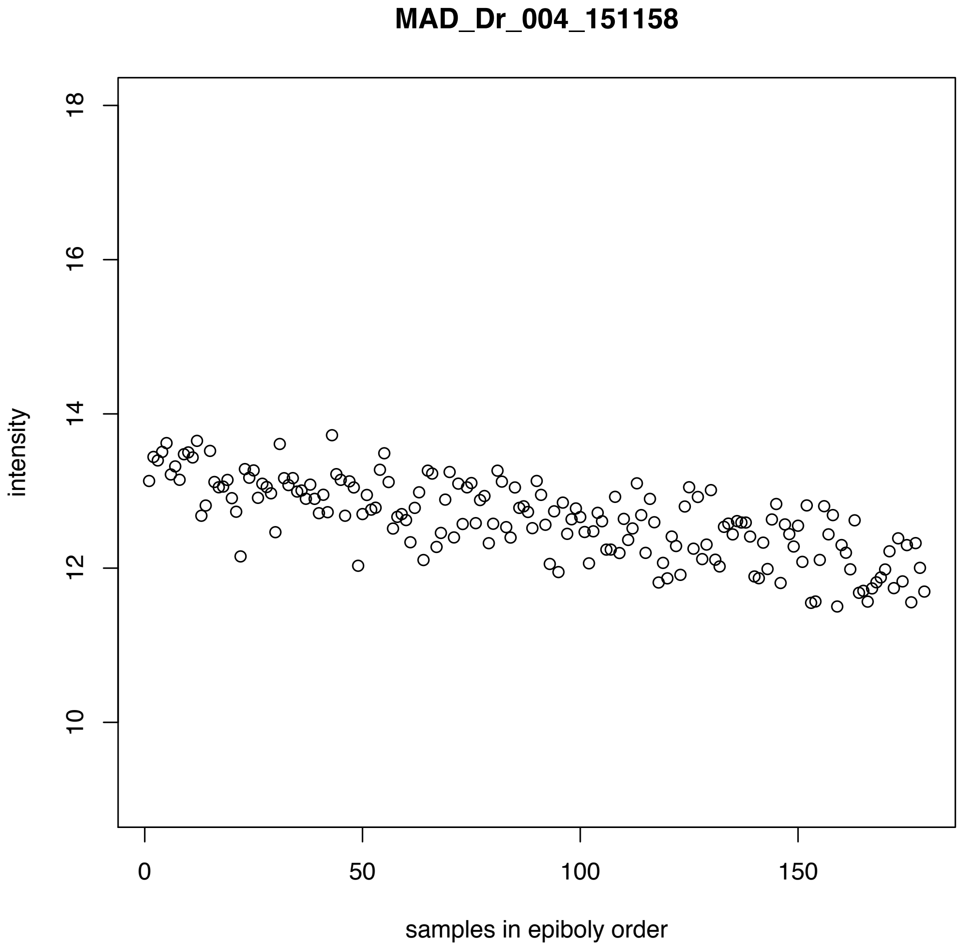
MAD_Dr_004_151158
intensity
10
12
14
16
18
0
50
100
150
samples in epiboly order

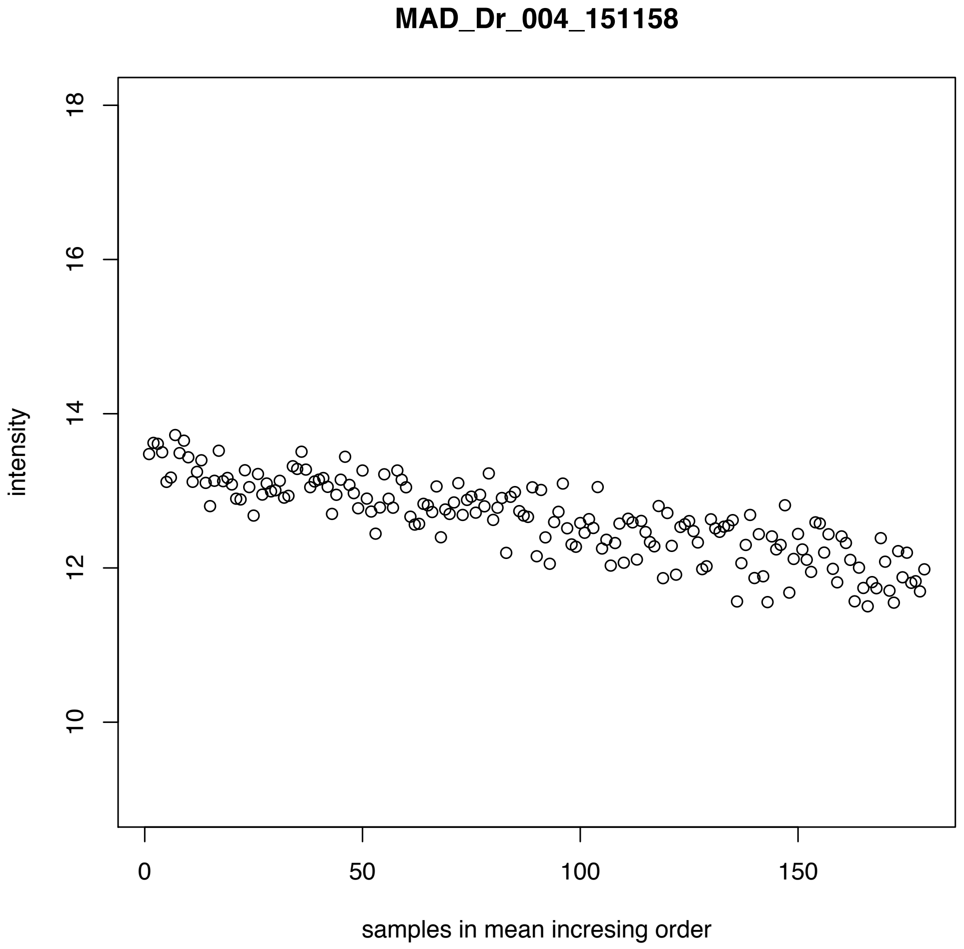
MAD_Dr_004_151158
intensity
10
12
14
16
18
0
50
100
150
samples in mean incresing order

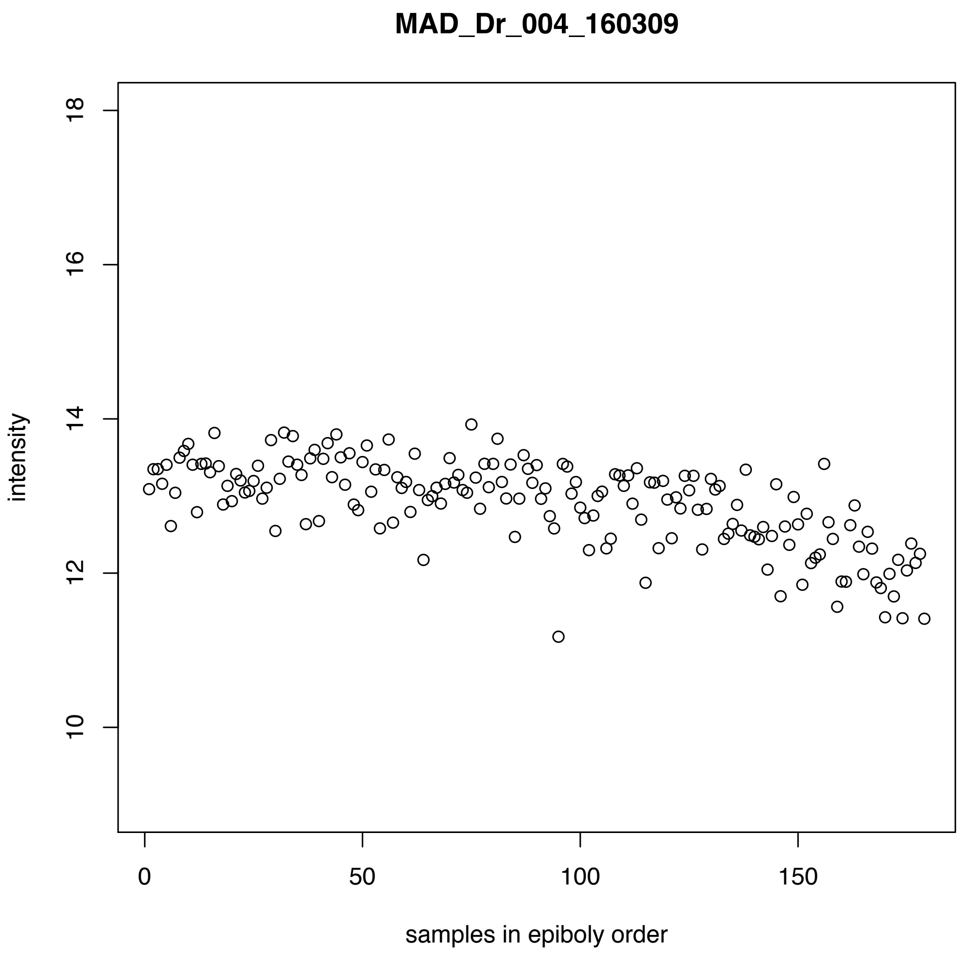
MAD_Dr_004_160309
intensity
samples in epiboly order
10
12
14
16
18
0
50
100
150

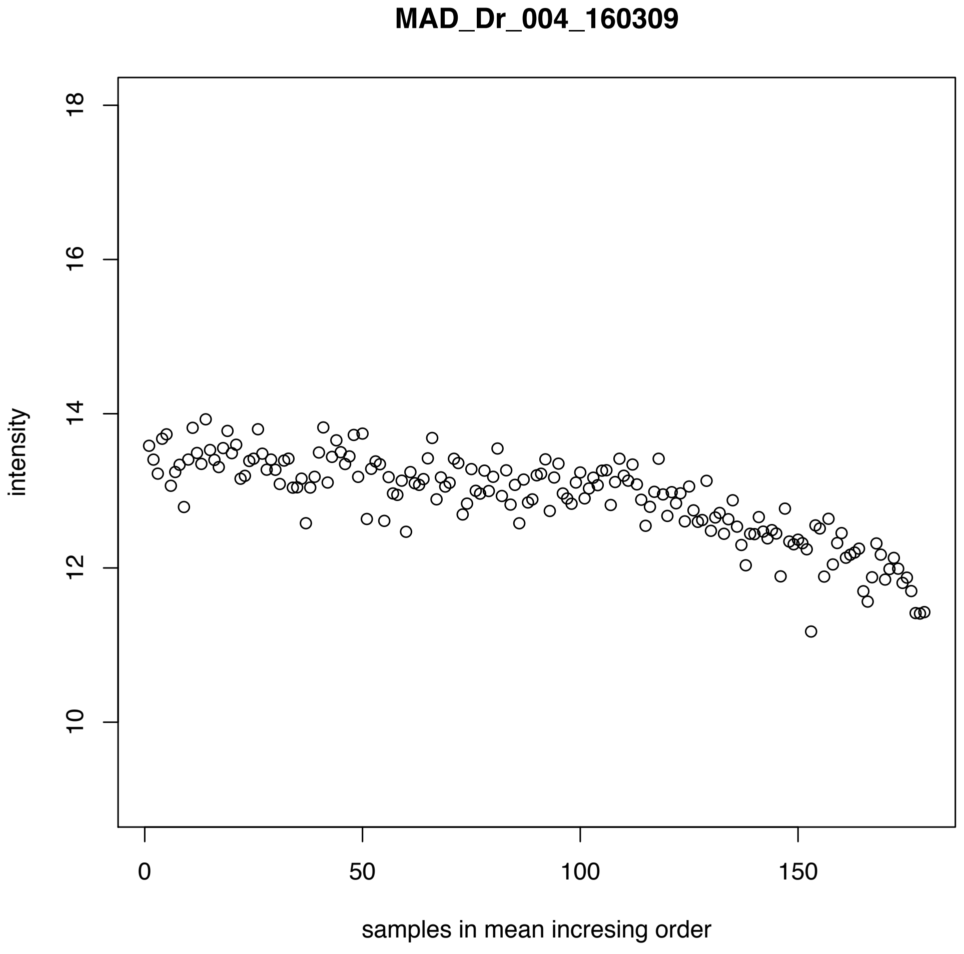
MAD_Dr_004_160309
intensity
samples in mean incresing order
10
12
14
16
18
0
50
100
150

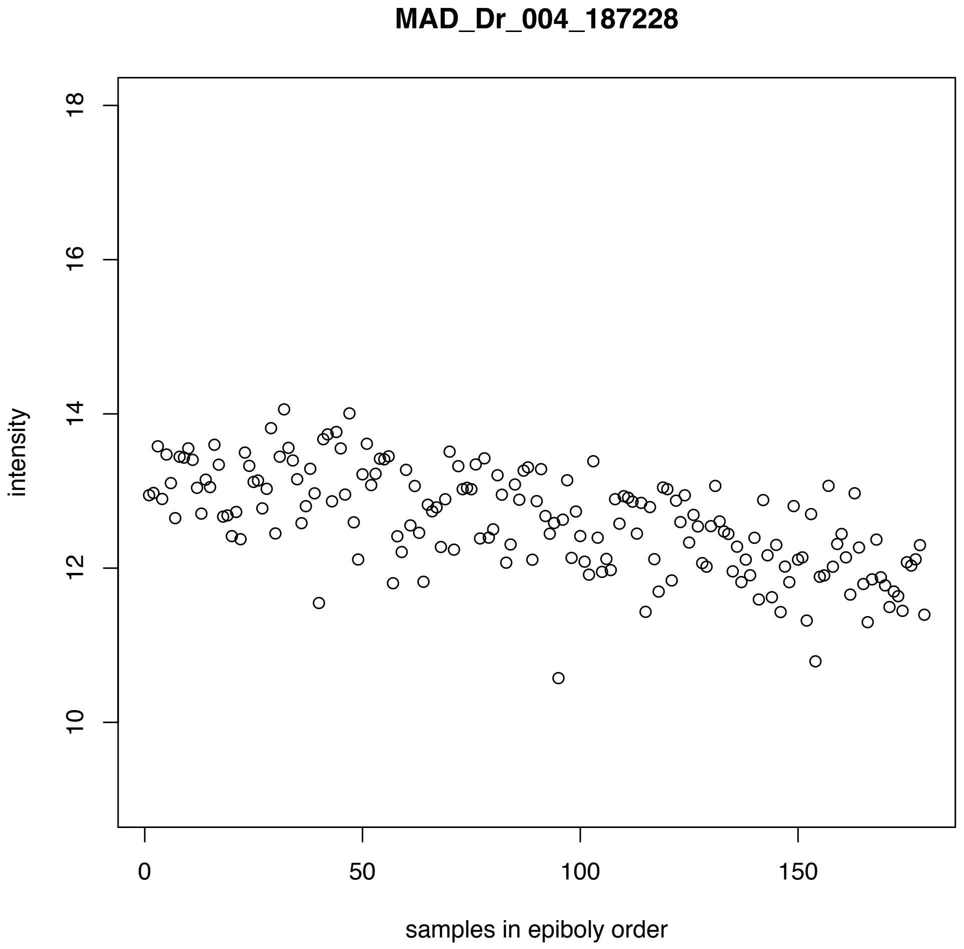
MAD_Dr_004_187228
intensity
samples in epiboly order
18
16
14
12
10
0
50
100
150

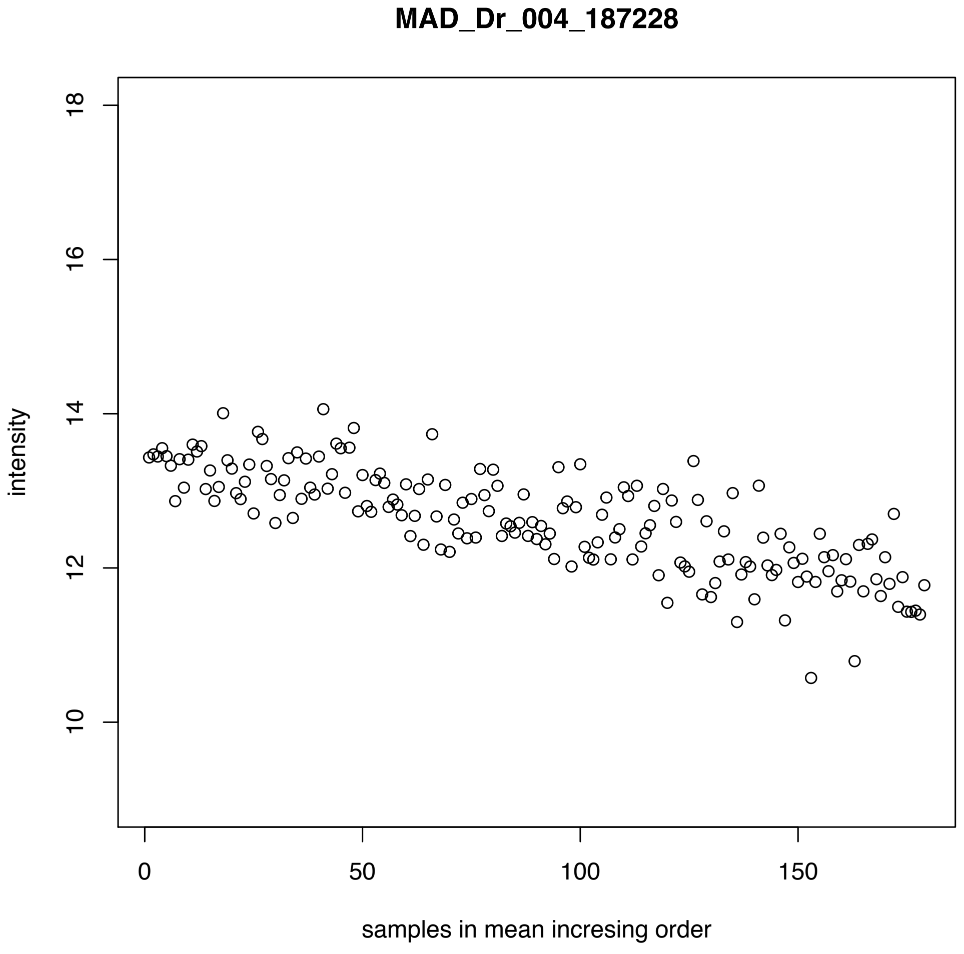
MAD_Dr_004_187228
intensity
samples in mean incresing order
18
16
14
12
10
0
50
100
150

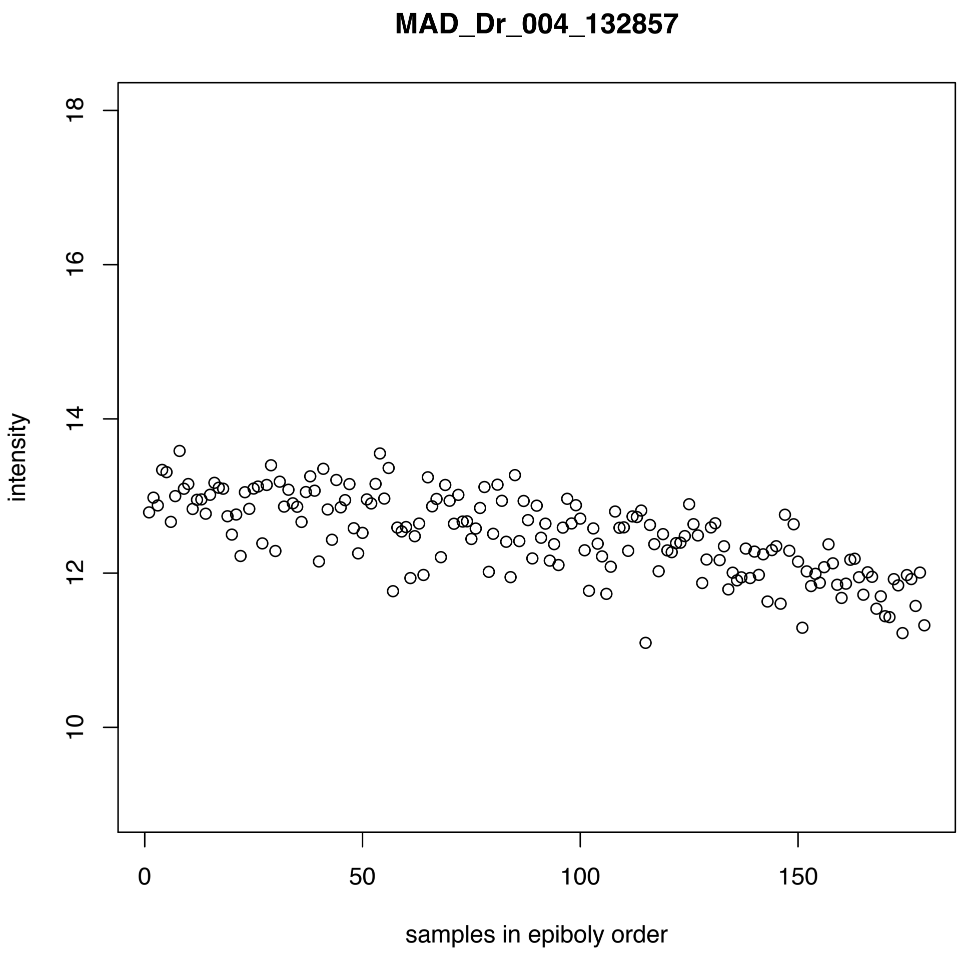
MAD_Dr_004_132857
intensity
18
16
14
12
10
0
50
100
150
samples in epiboly order

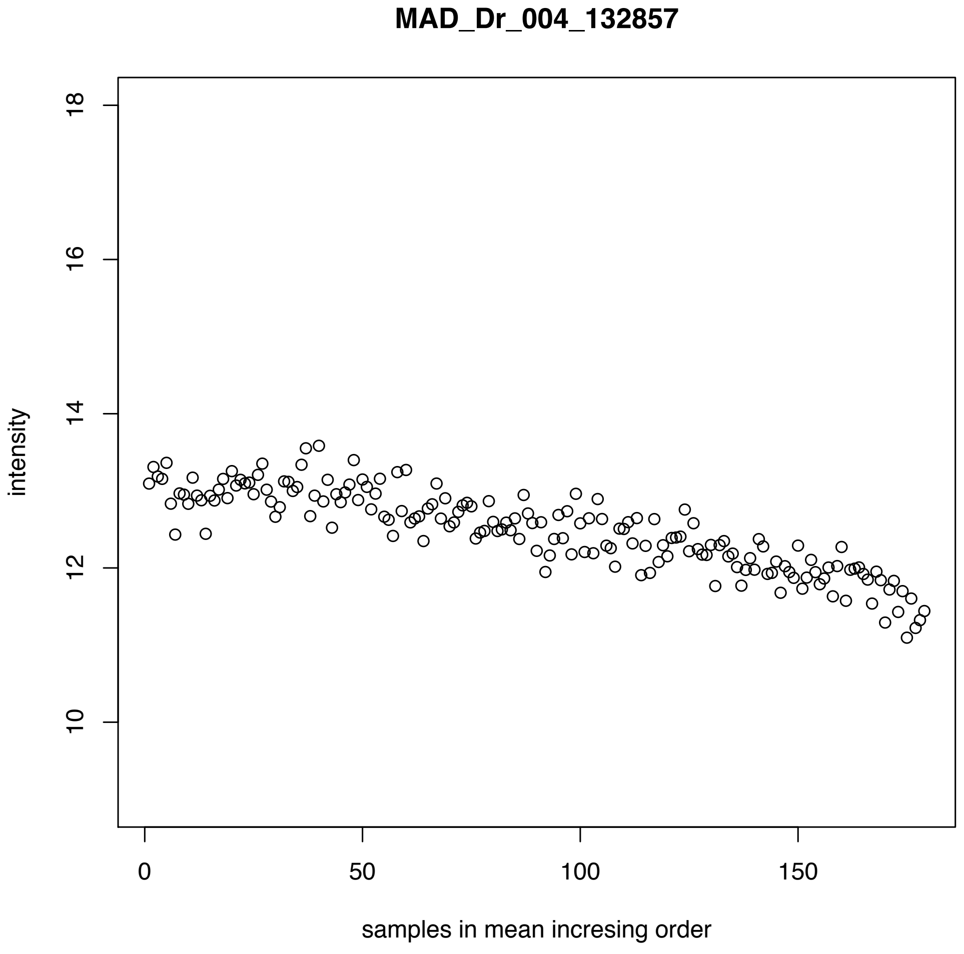
MAD_Dr_004_132857
intensity
18
16
14
12
10
0
50
100
150
samples in mean incresing order

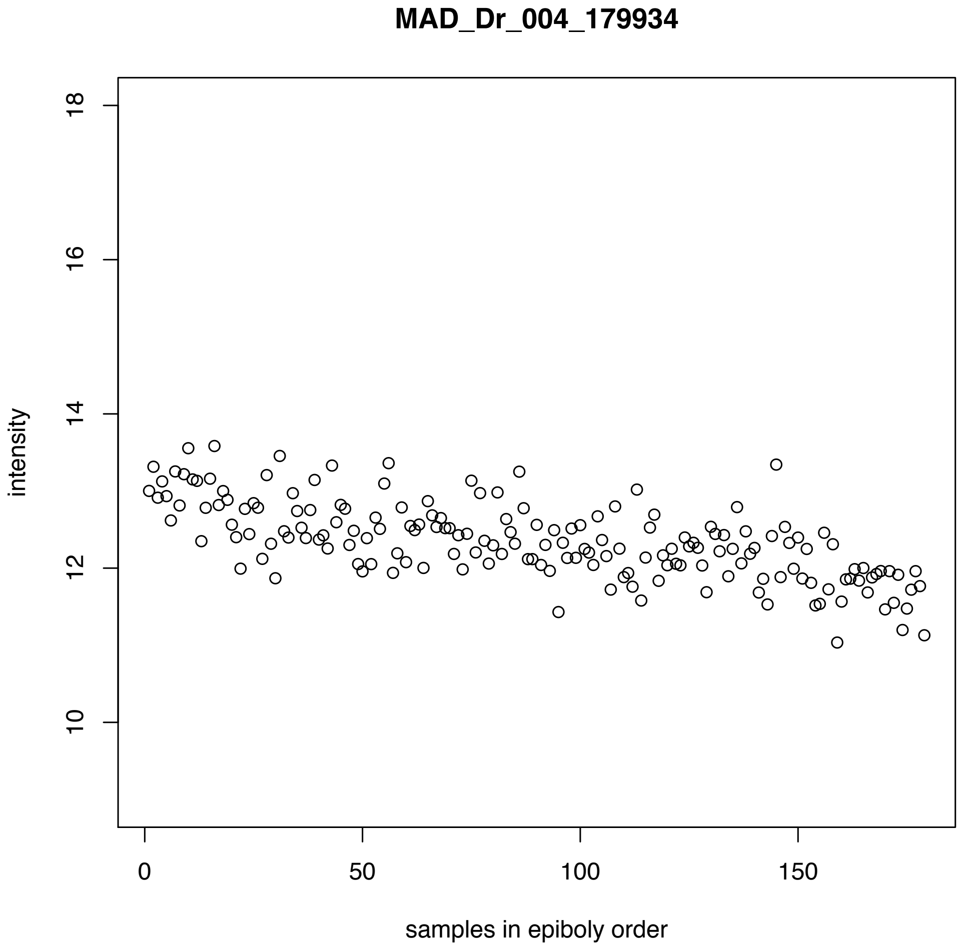

MAD_Dr_004_179934
intensity
10
12
14
16
18
0
50
100
150
samples in epiboly order

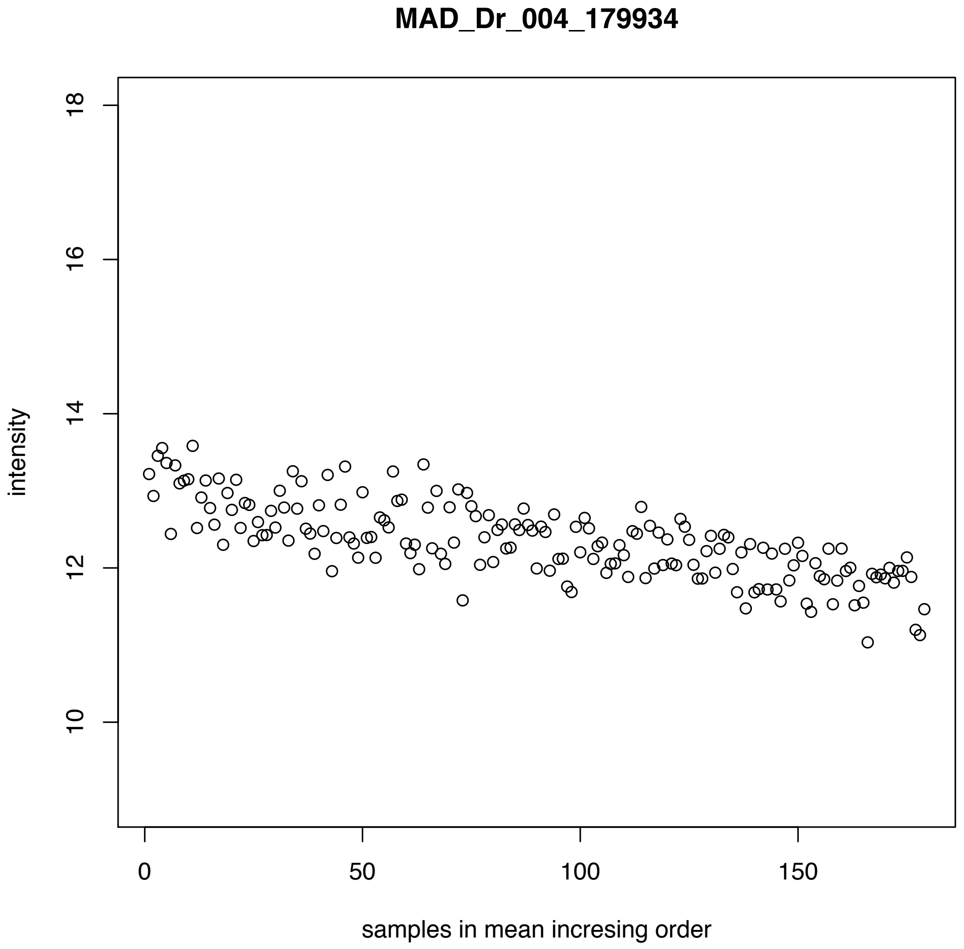

MAD_Dr_004_179934
intensity
10
12
14
16
18
0
50
100
150
samples in mean incresing order

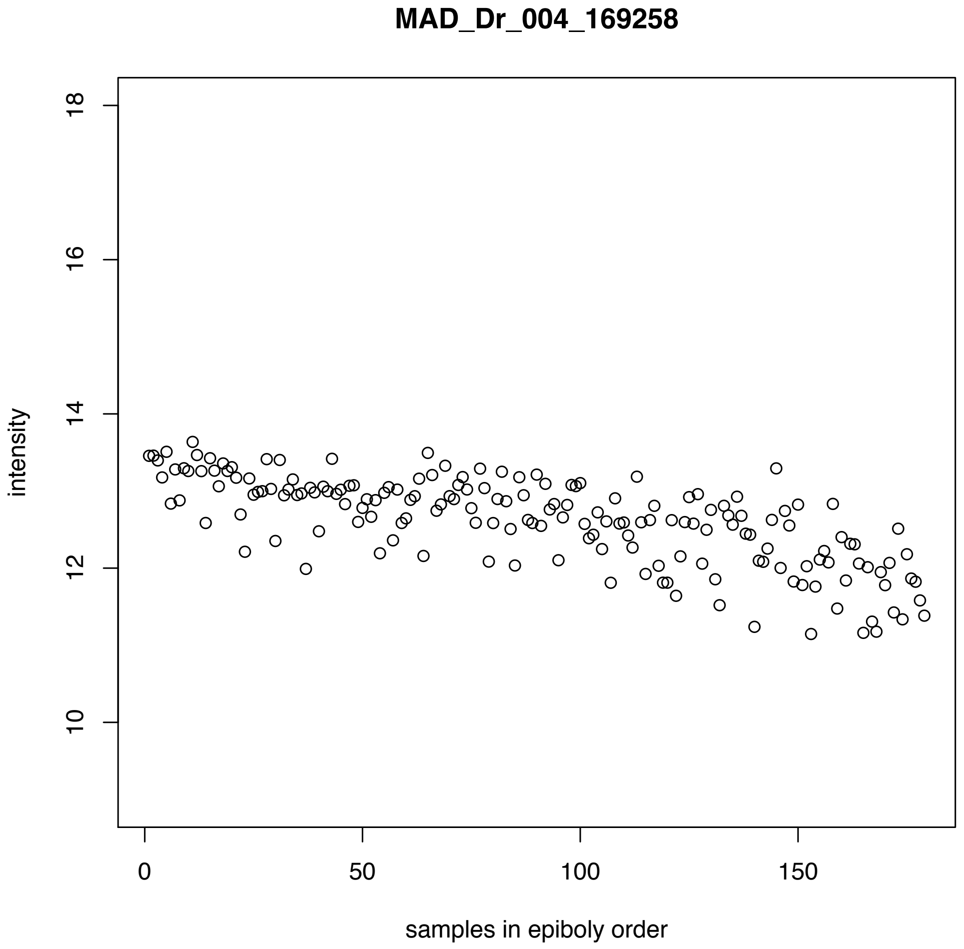
MAD_Dr_004_169258
intensity
10
12
14
16
18
0
50
100
150
samples in epiboly order

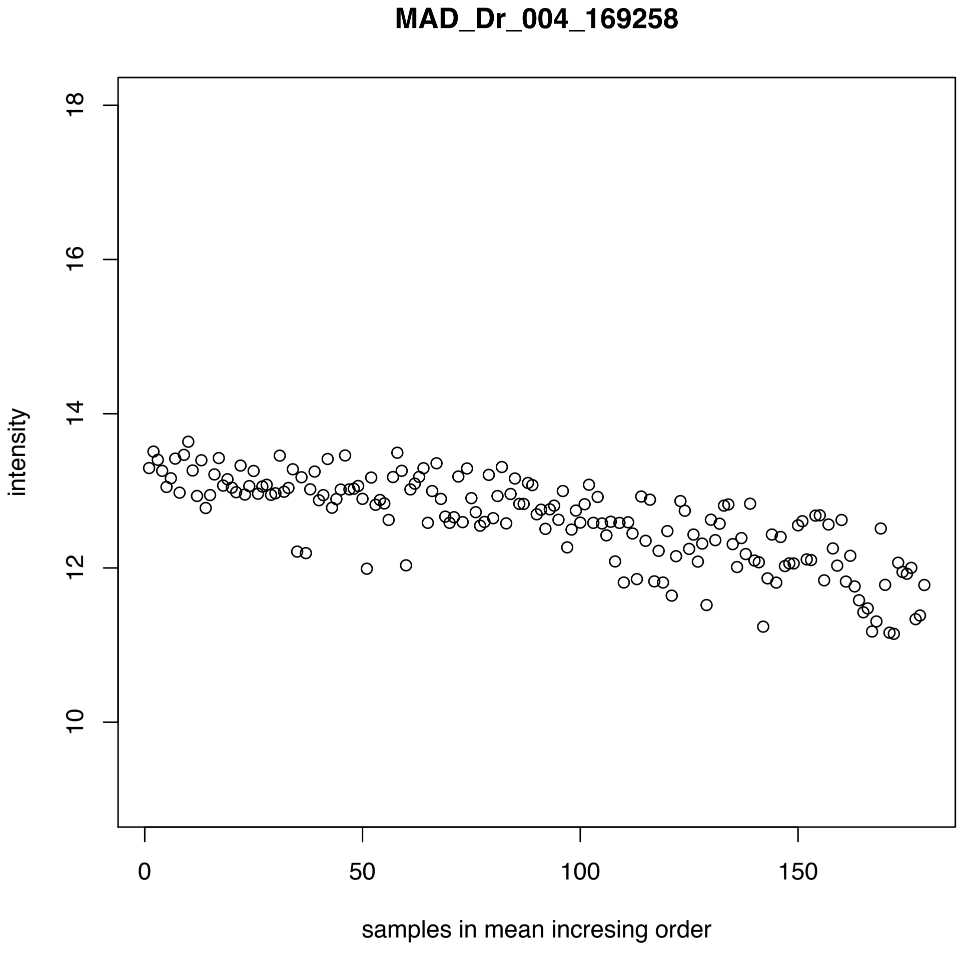
MAD_Dr_004_169258
intensity
10
12
14
16
18
0
50
100
150
samples in mean incresing order

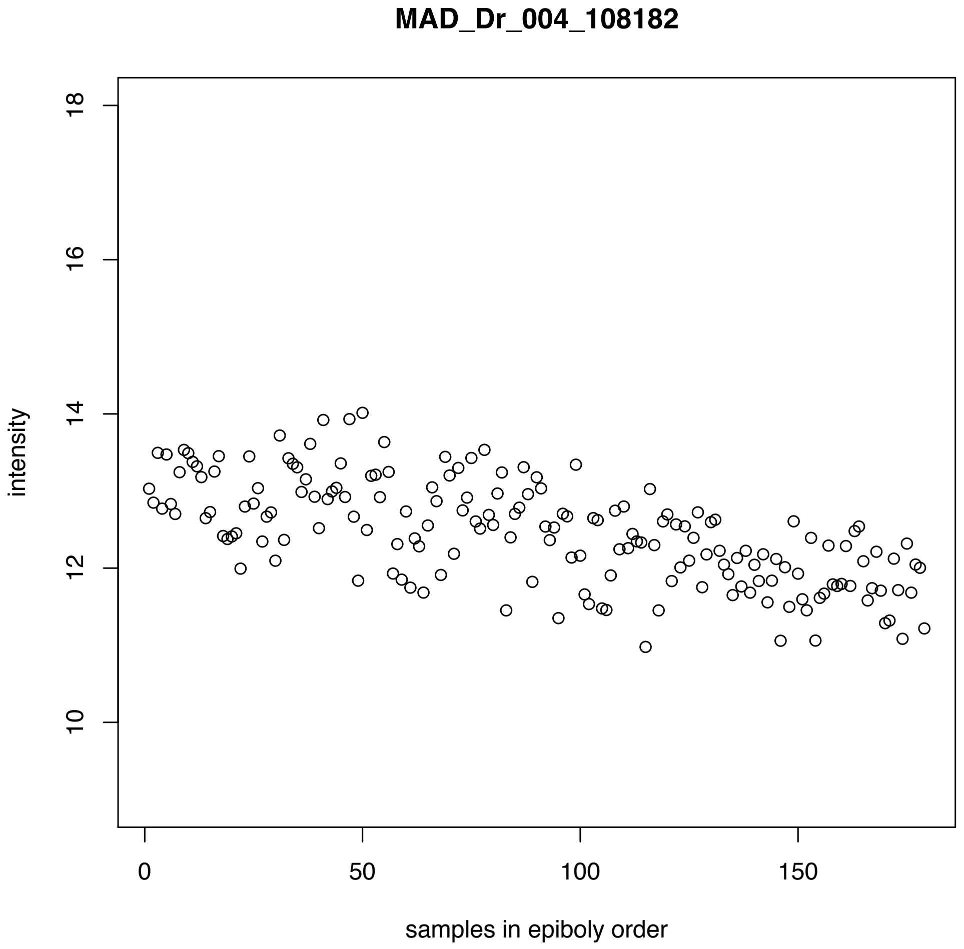
MAD_Dr_004_108182
intensity
10
12
14
16
18
0
50
100
150
samples in epiboly order

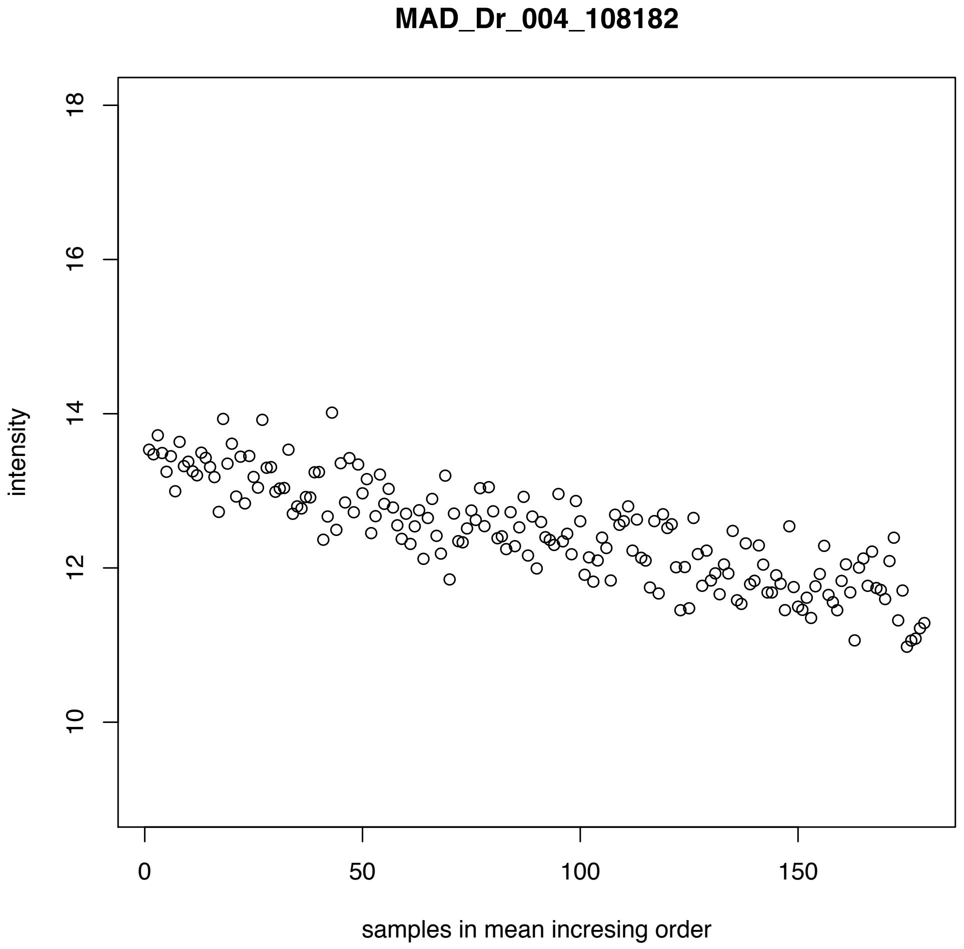
MAD_Dr_004_108182
intensity
10
12
14
16
18
0
50
100
150
samples in mean incresing order

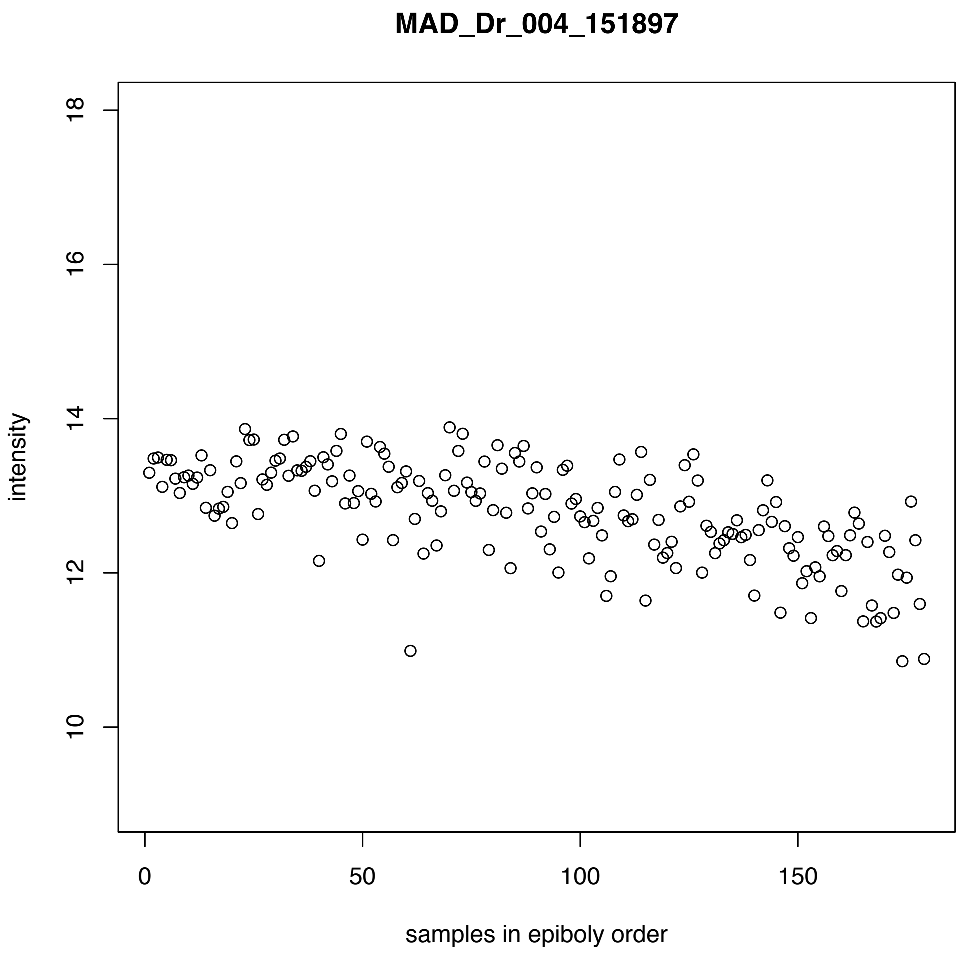
MAD_Dr_004_151897
intensity
18
16
14
12
10
0
50
100
150
samples in epiboly order

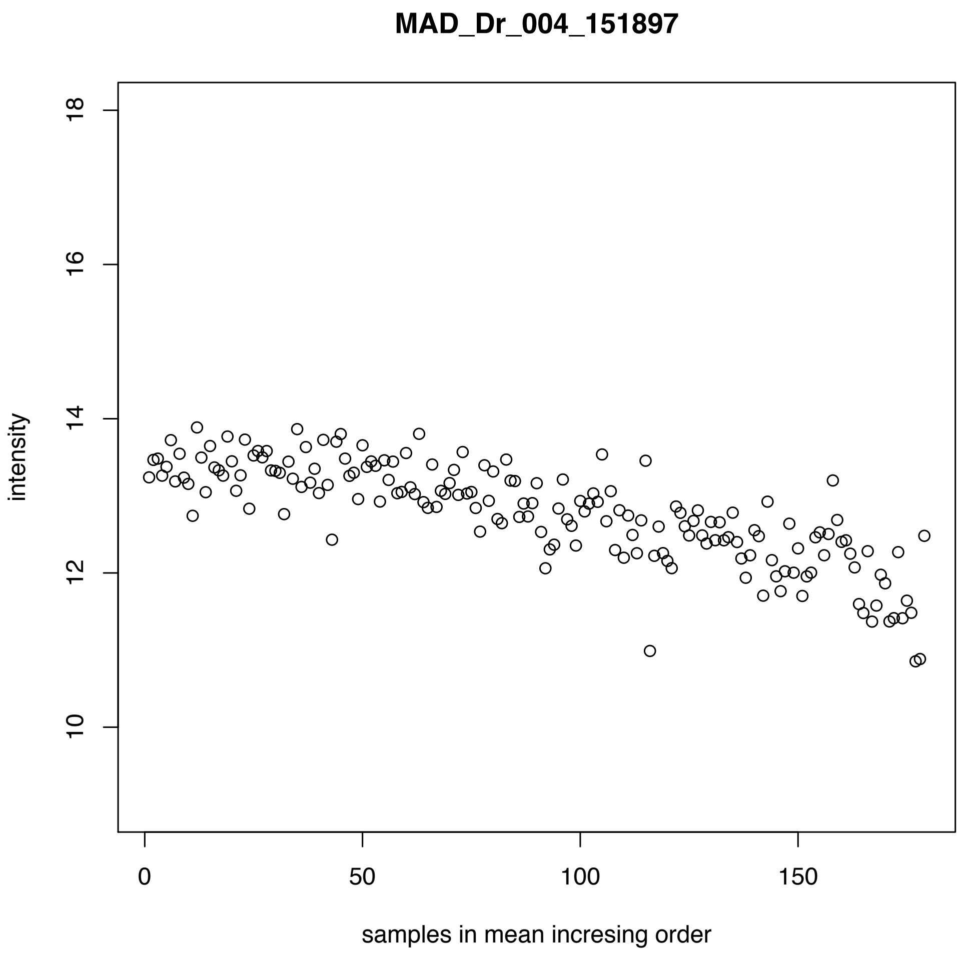
MAD_Dr_004_151897
intensity
18
16
14
12
10
0
50
100
150
samples in mean incresing order

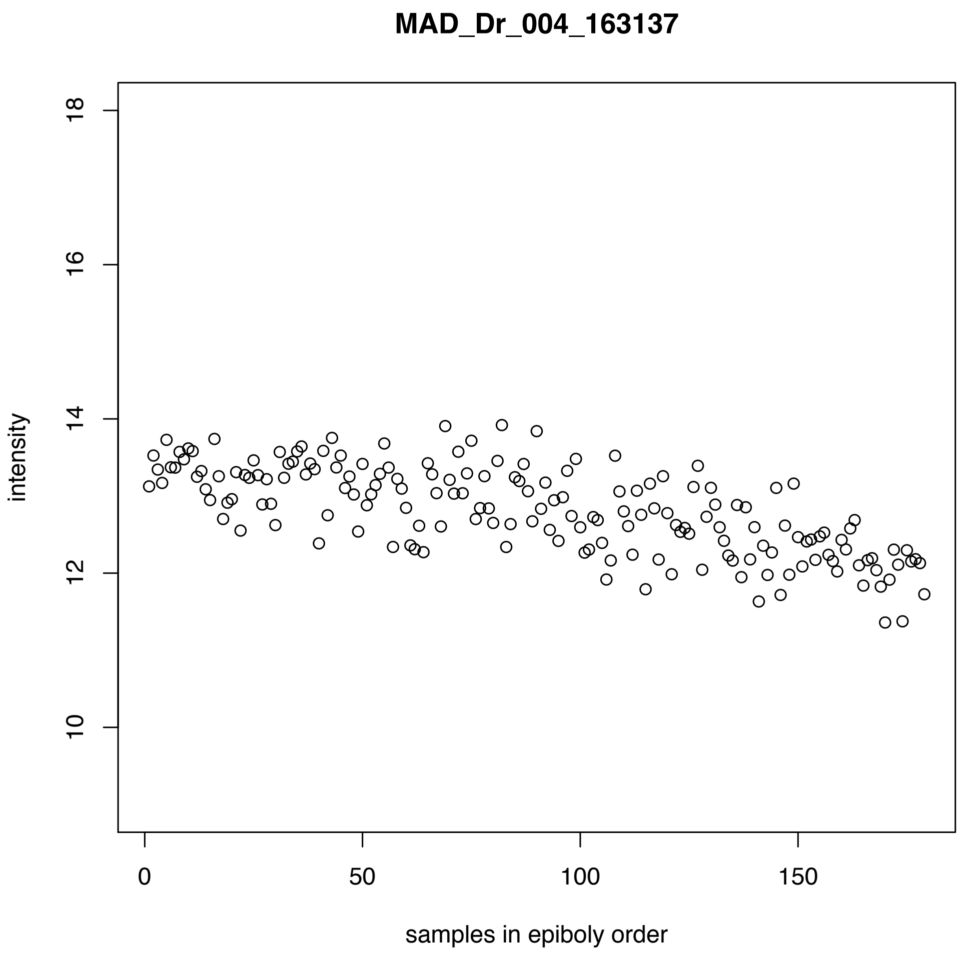
MAD_Dr_004_163137
intensity
10
12
14
16
18
0
50
100
150
samples in epiboly order

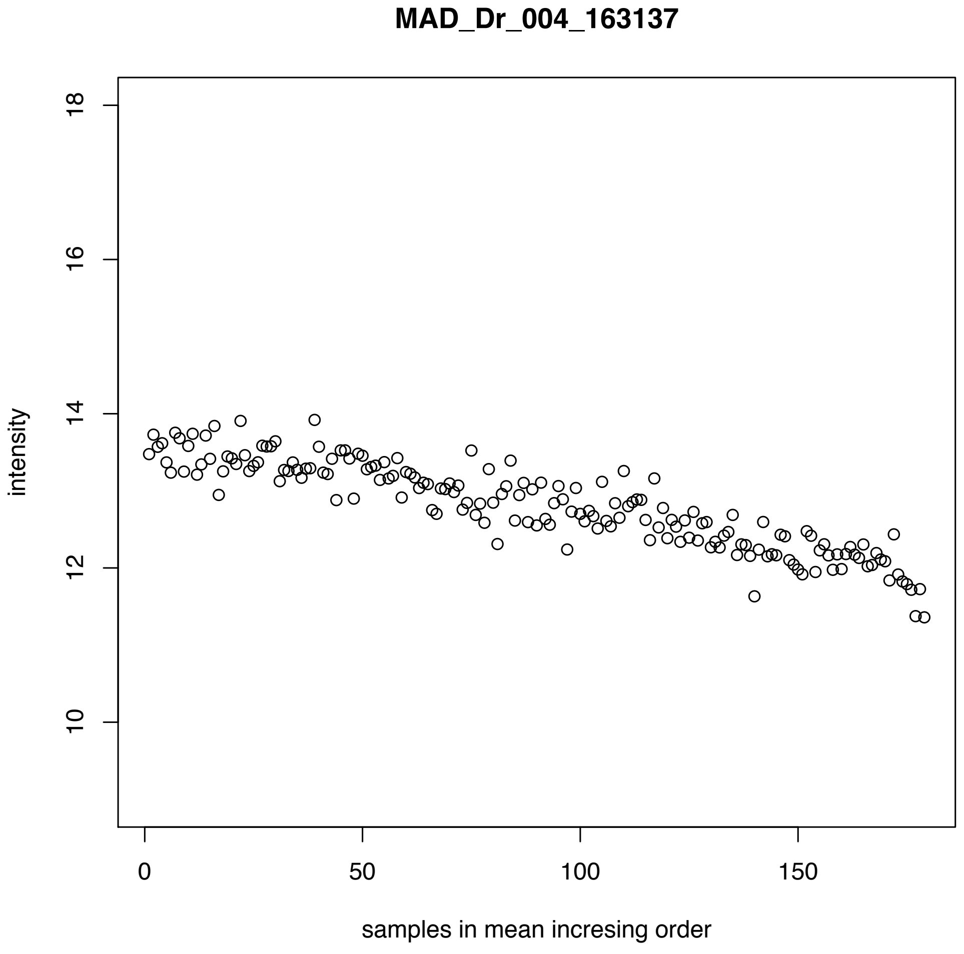
MAD_Dr_004_163137
intensity
10
12
14
16
18
0
50
100
150
samples in mean incresing order

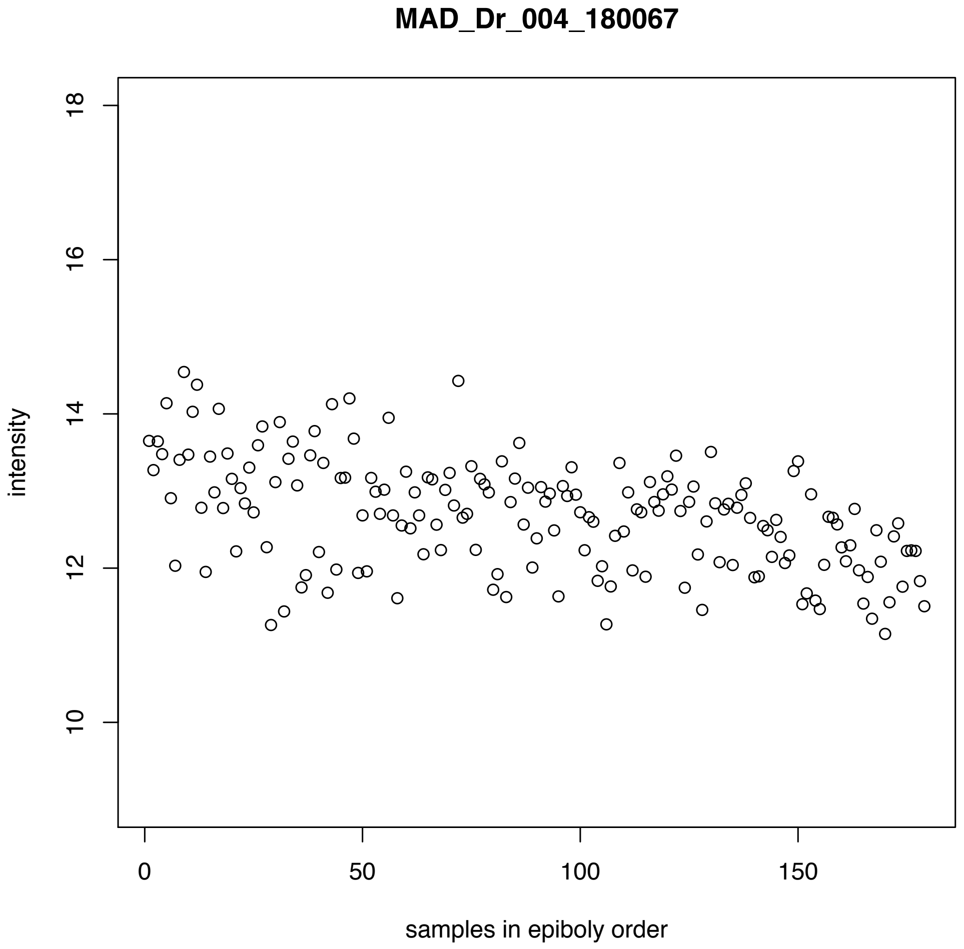
MAD_Dr_004_180067
intensity
10
12
14
16
18
0
50
100
150
samples in epiboly order

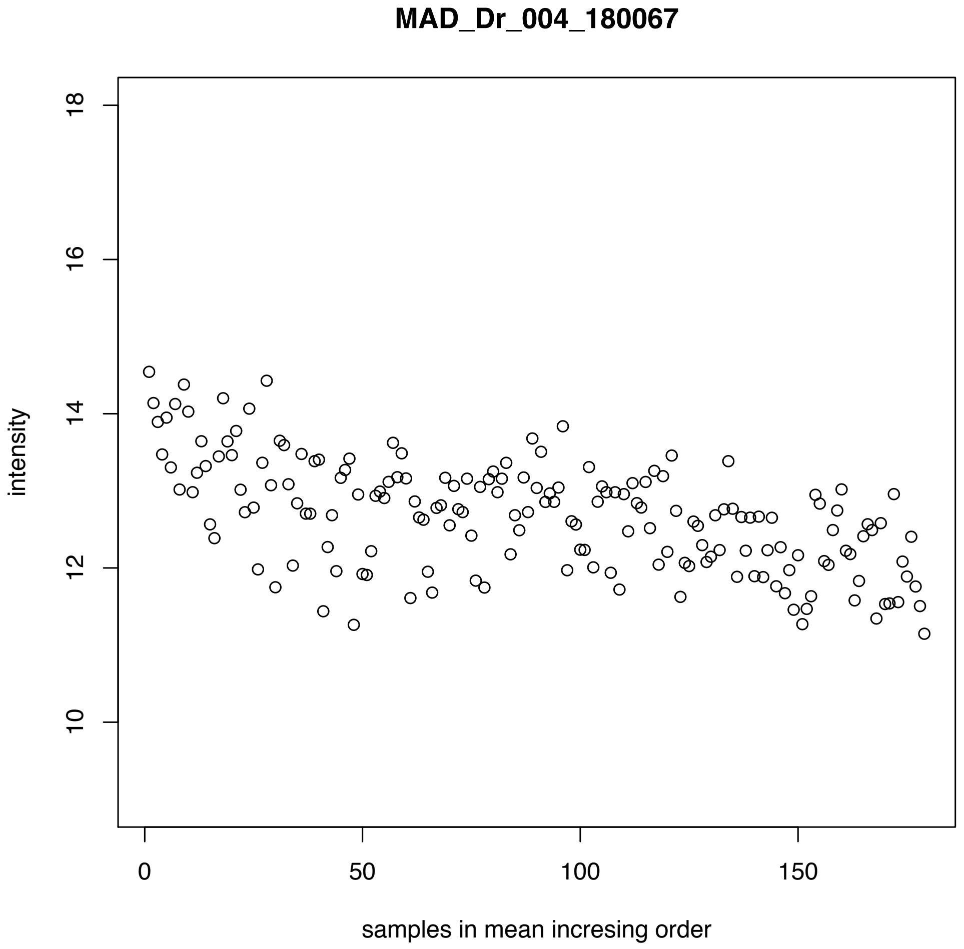
MAD_Dr_004_180067
intensity
10
12
14
16
18
0
50
100
150
samples in mean incresing order

MAD_Dr_004_161431

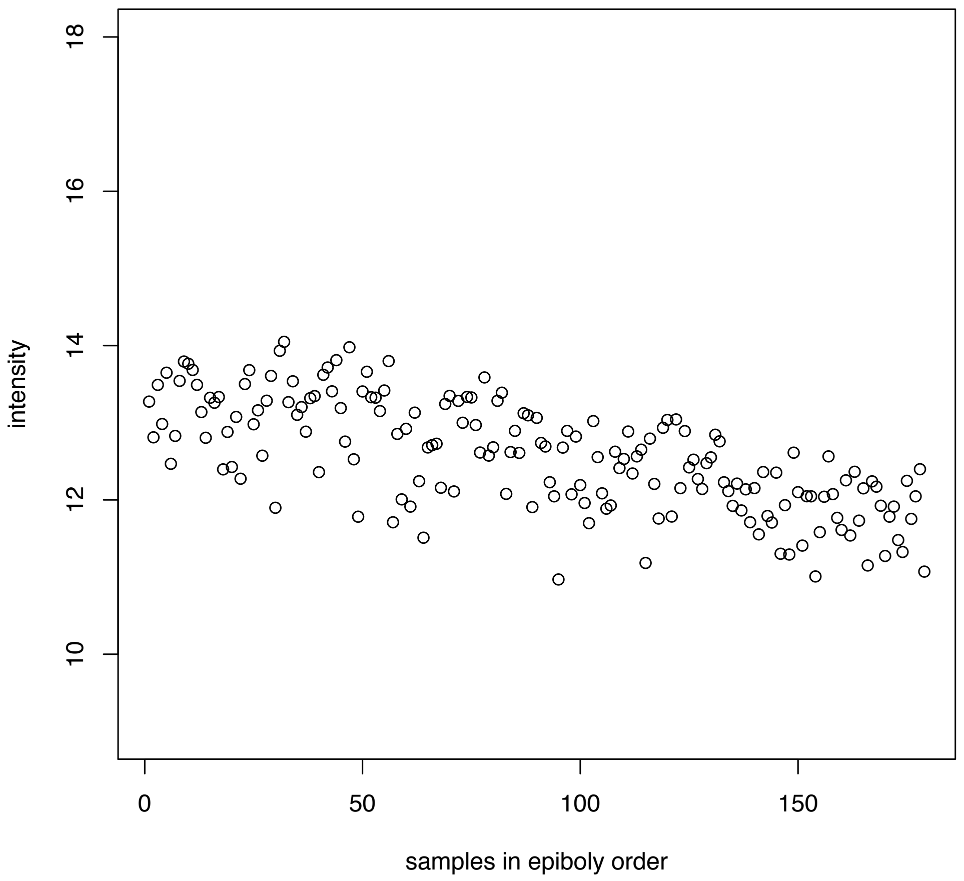

MAD_Dr_004_161431

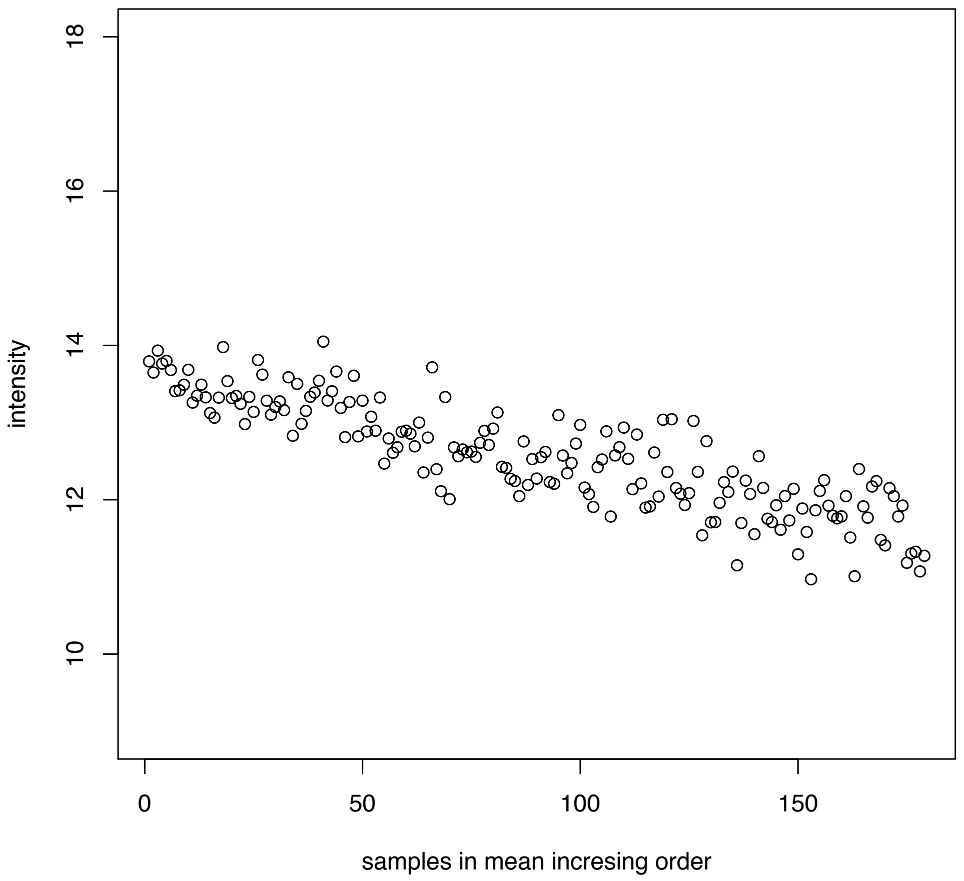

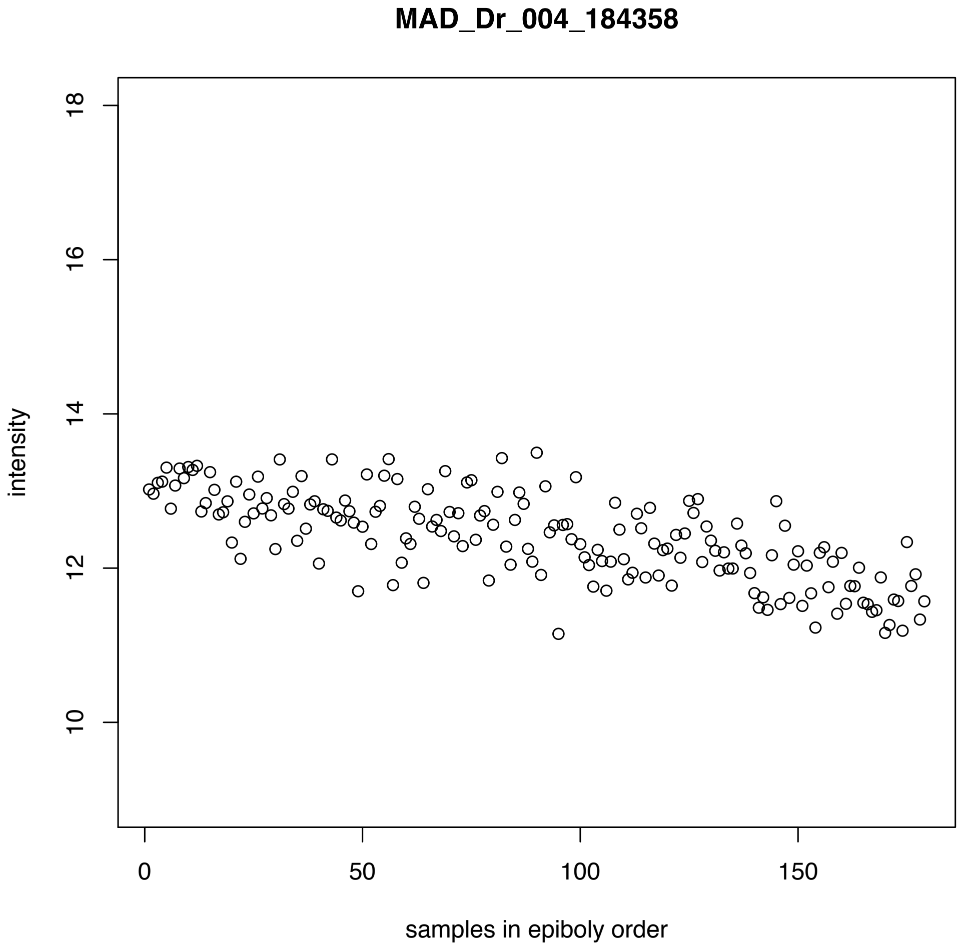
MAD_Dr_004_184358
intensity
18
16
14
12
10
0
50
100
150
samples in epiboly order

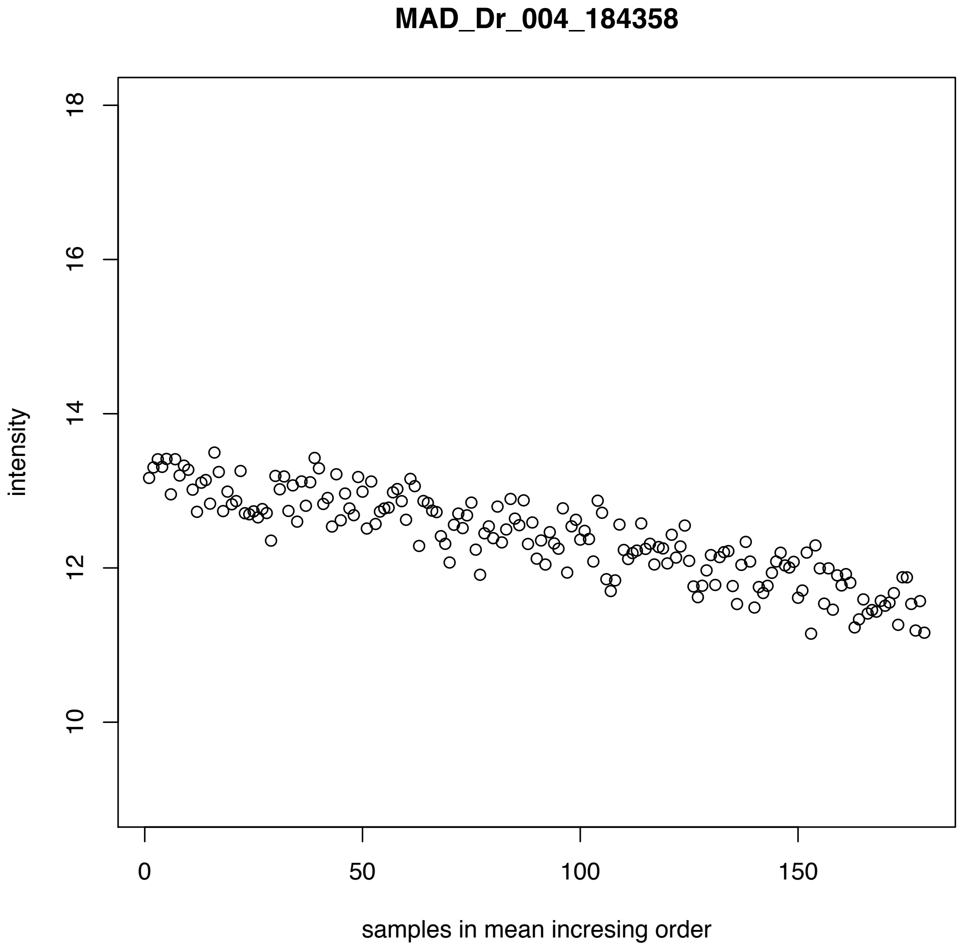
MAD_Dr_004_184358
intensity
18
16
14
12
10
0
50
100
150
samples in mean incresing order

MAD_Dr_004_175739

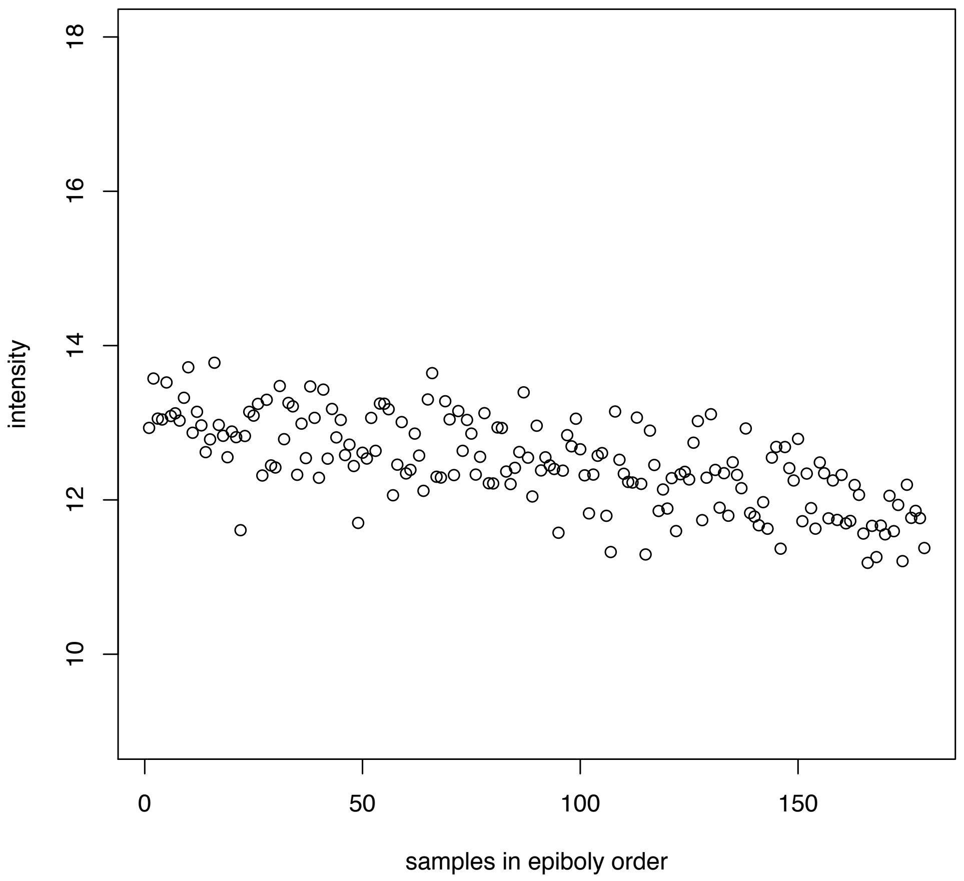

MAD_Dr_004_175739

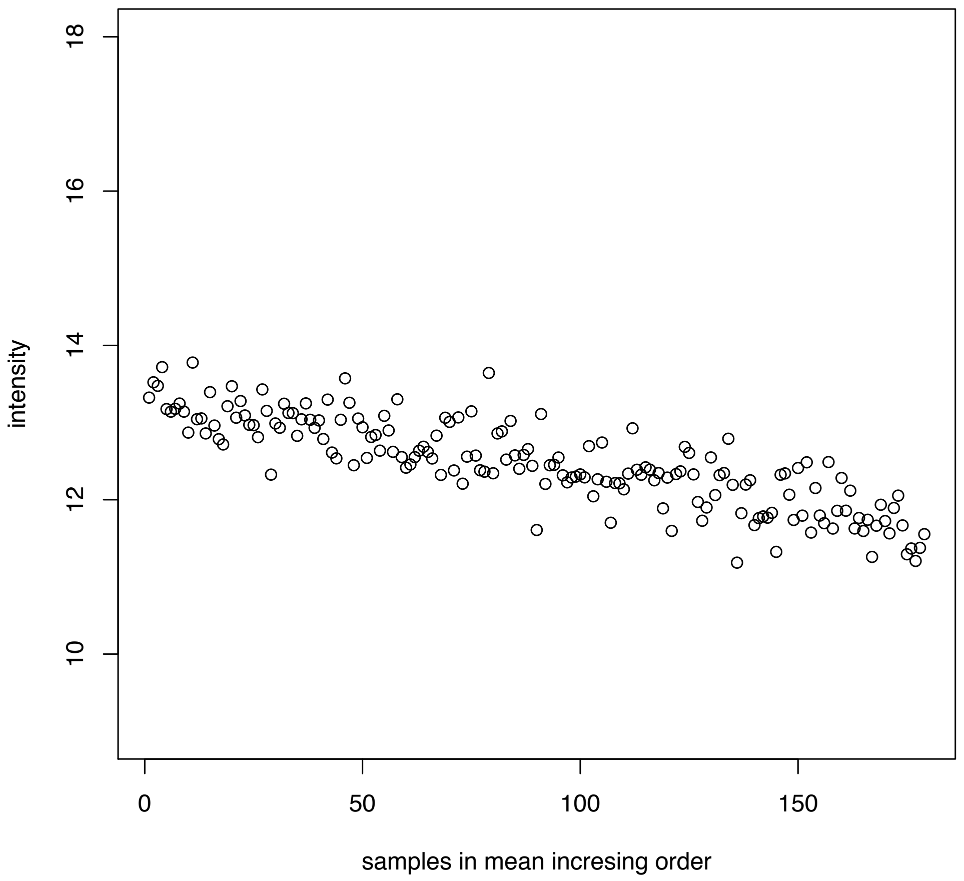

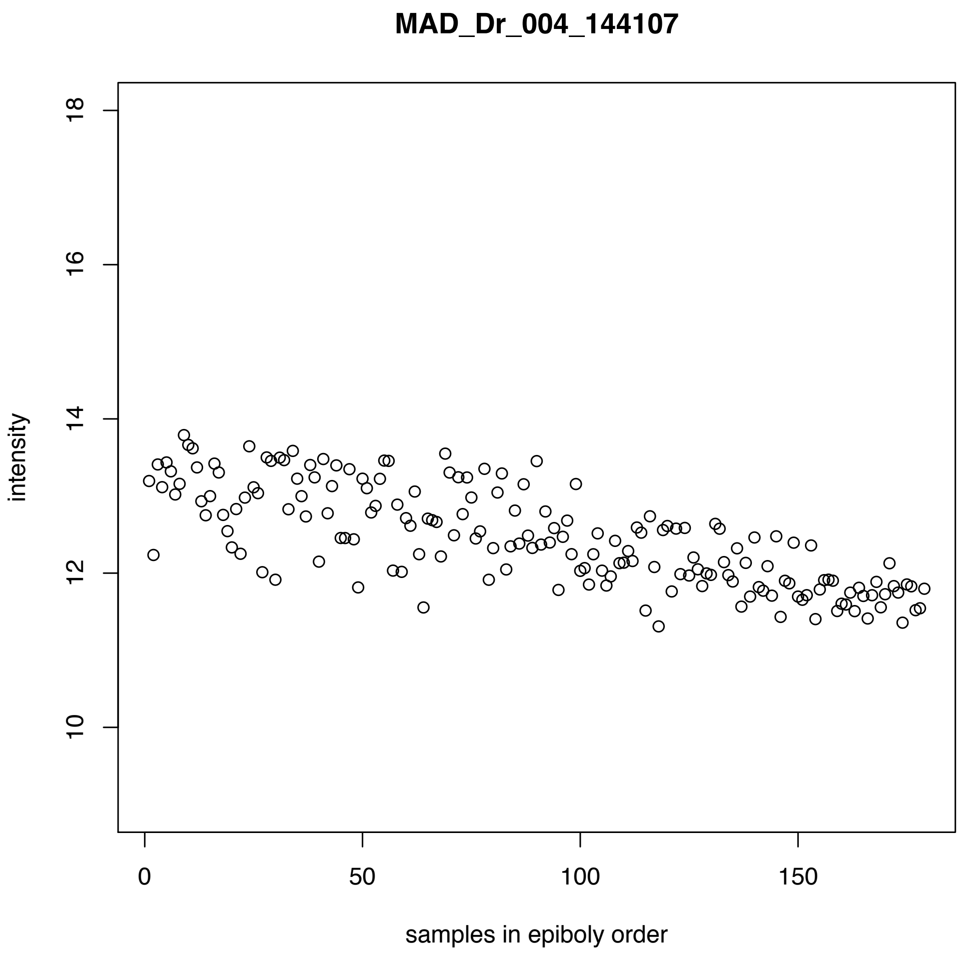

MAD_Dr_004_144107
intensity
10
12
14
16
18
0
50
100
150
samples in epiboly order

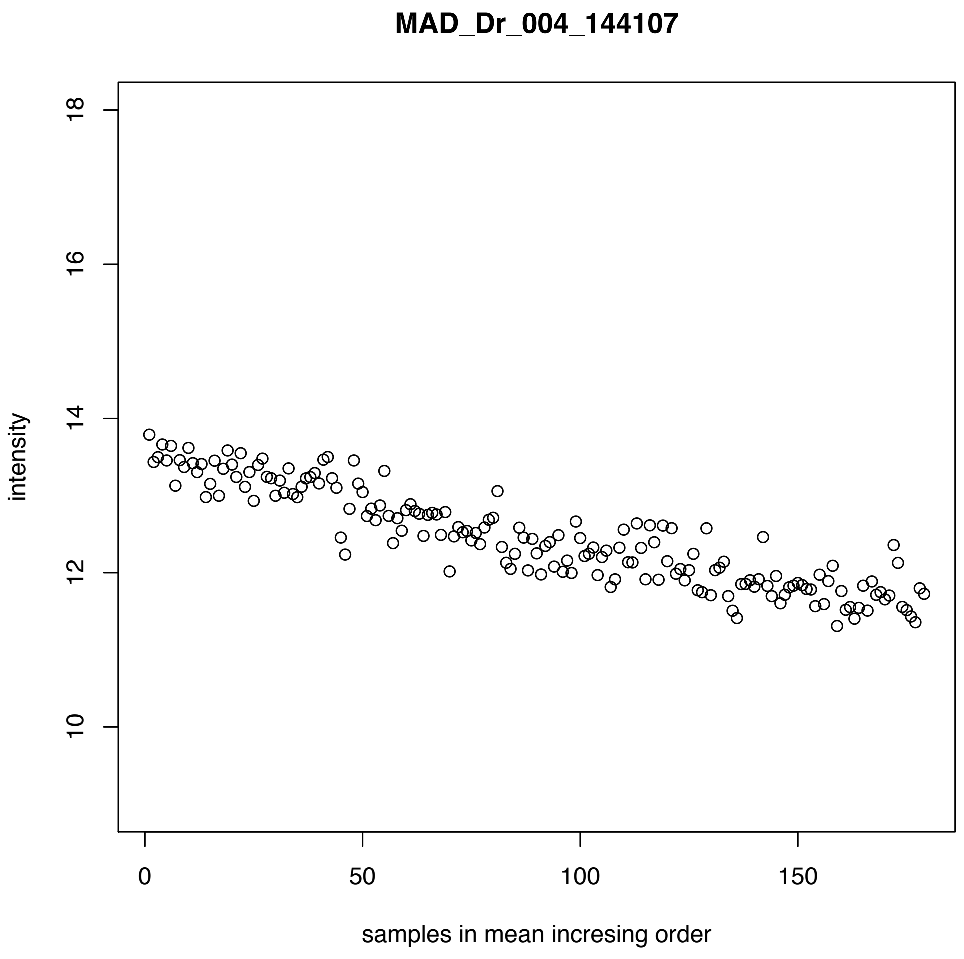

MAD_Dr_004_144107
intensity
10
12
14
16
18
0
50
100
150
samples in mean incresing order

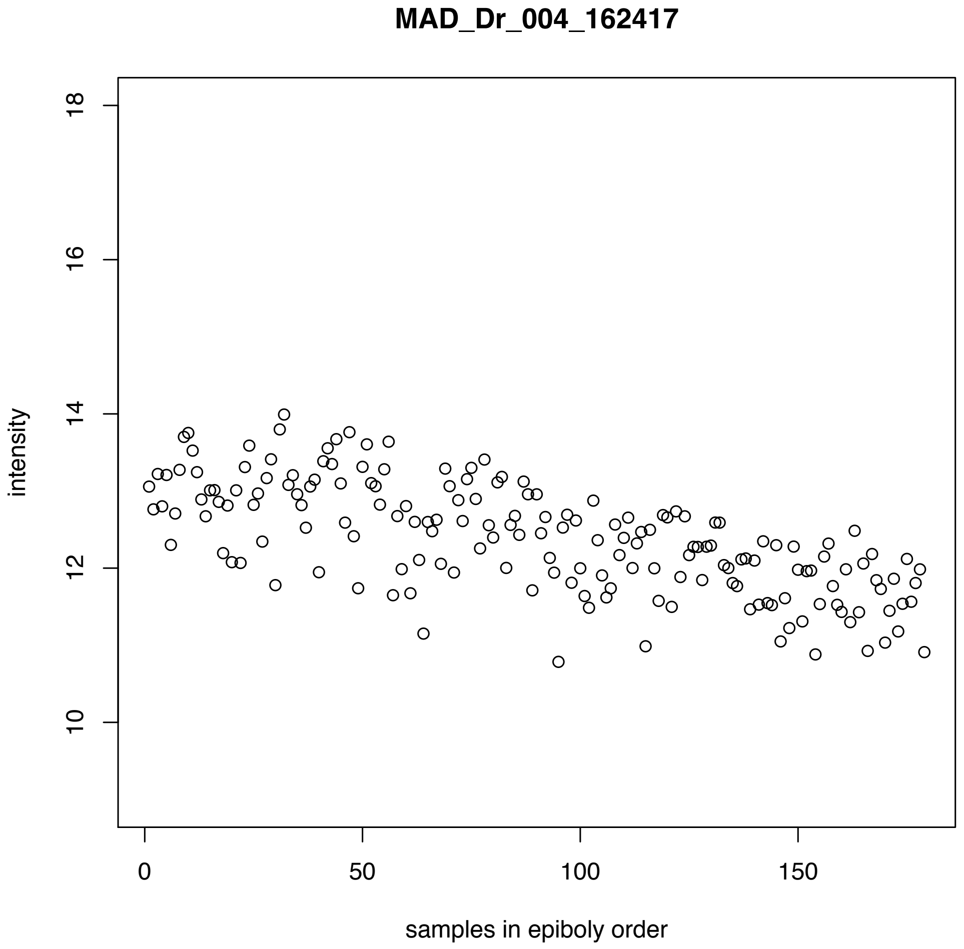
MAD_Dr_004_162417
intensity
samples in epiboly order
10
12
14
16
18
0
50
100
150

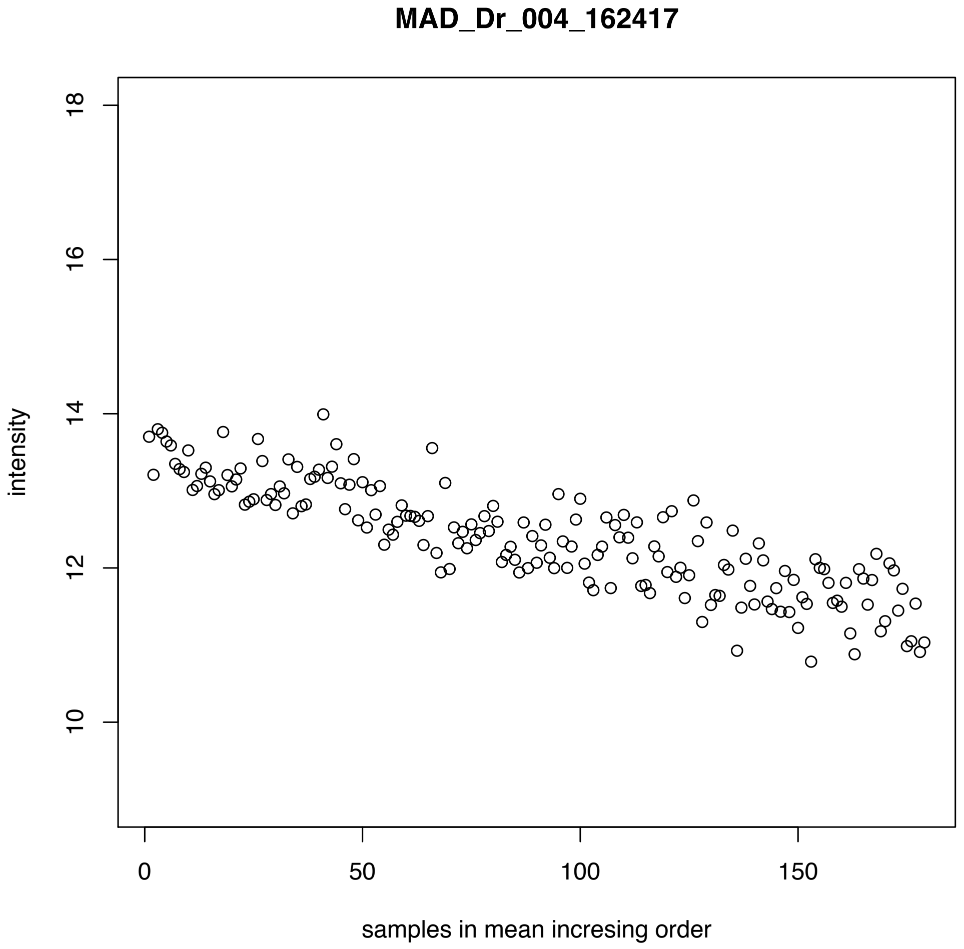
MAD_Dr_004_162417
intensity
samples in mean incresing order
10
12
14
16
18
0
50
100
150

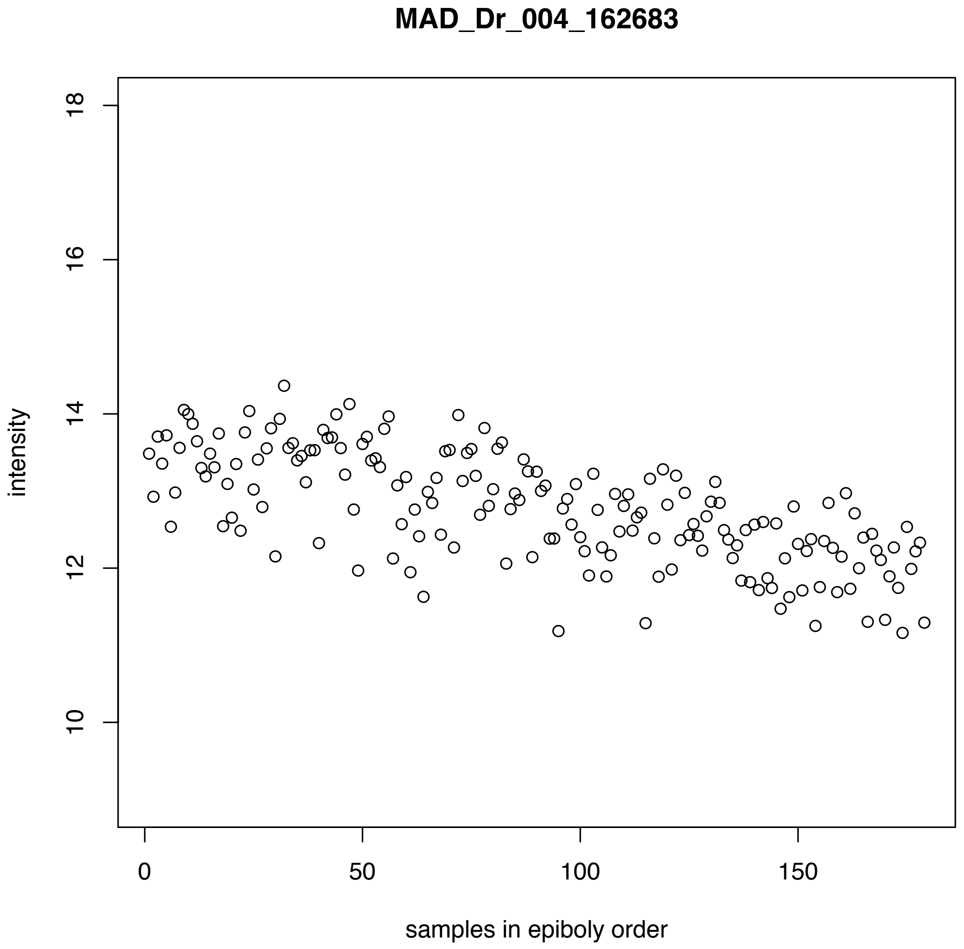
MAD_Dr_004_162683
intensity
samples in epiboly order
10
12
14
16
18
0
50
100
150

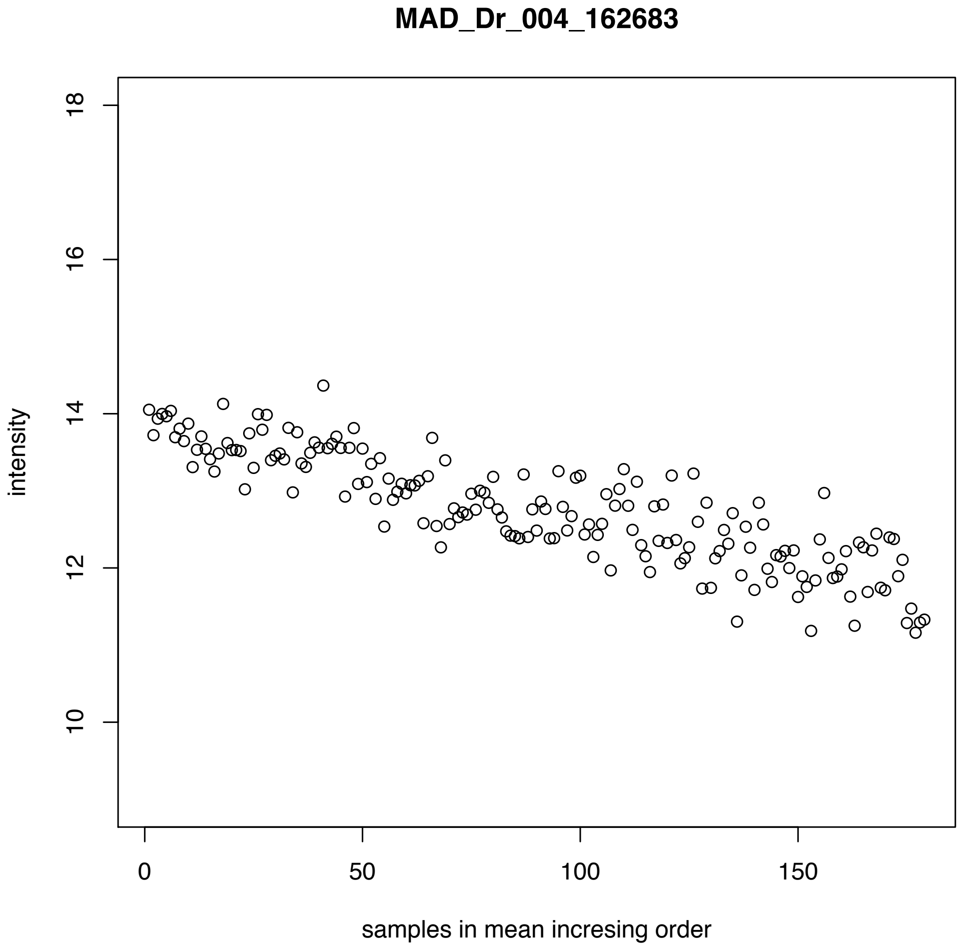
MAD_Dr_004_162683
intensity
samples in mean incresing order
10
12
14
16
18
0
50
100
150

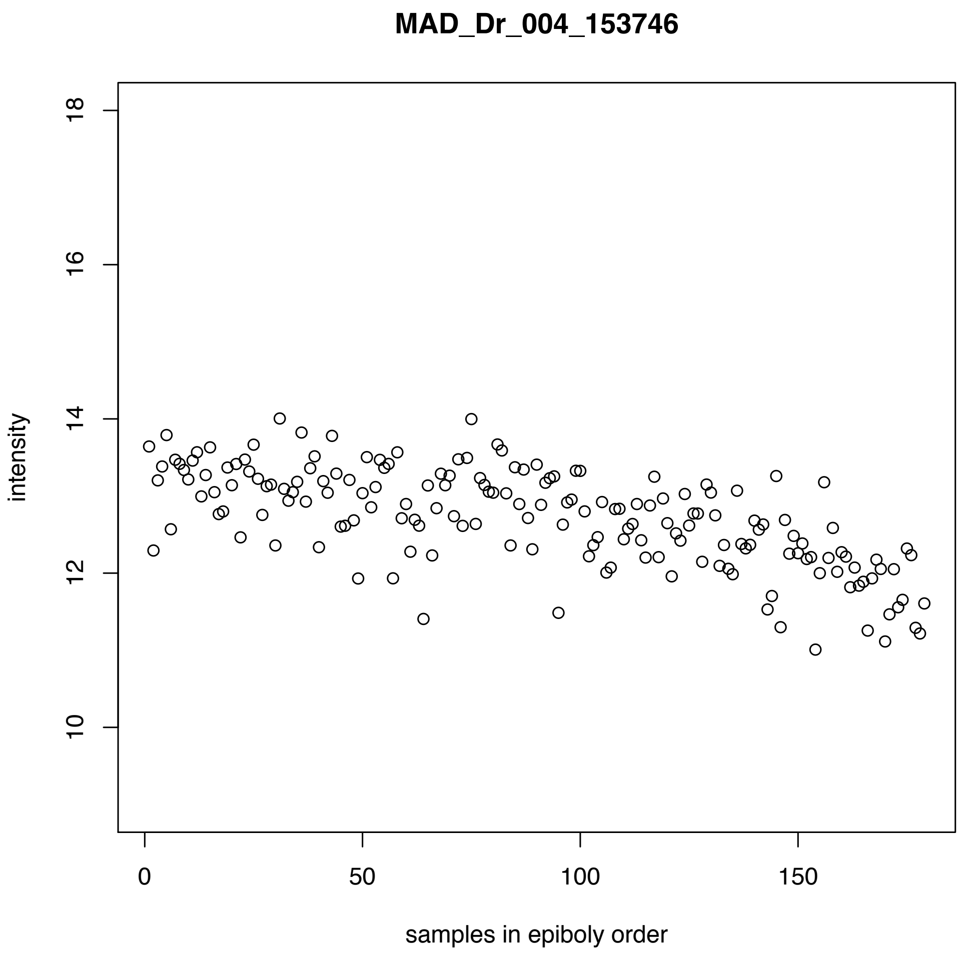

MAD_Dr_004_153746
intensity
10
12
14
16
18
0
50
100
150
samples in epiboly order

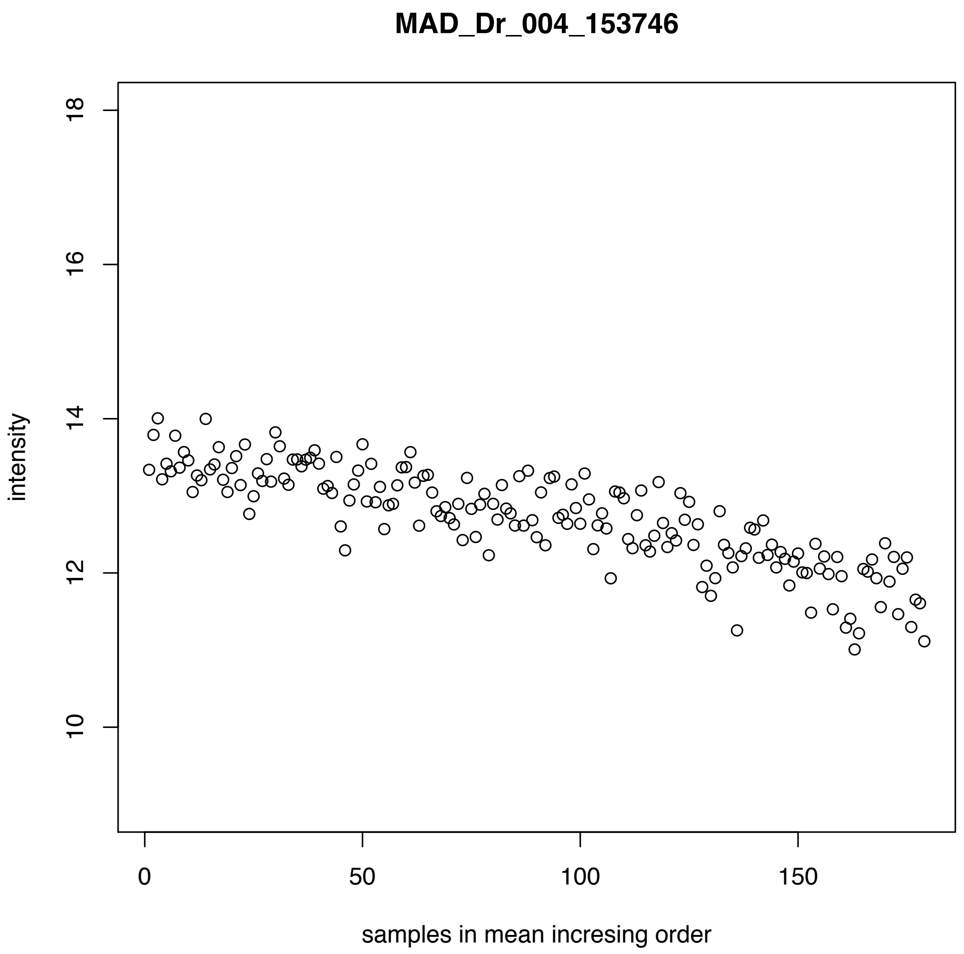

MAD_Dr_004_153746
intensity
10
12
14
16
18
0
50
100
150
samples in mean incresing order

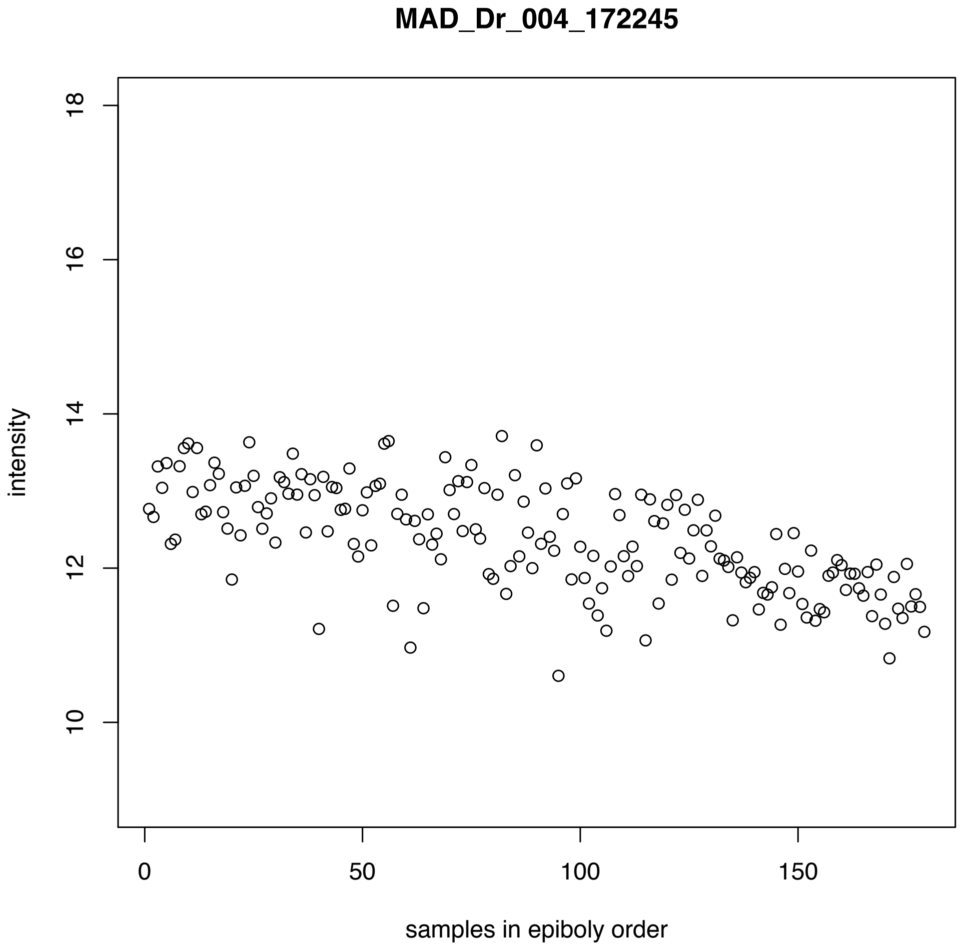
MAD_Dr_004_172245
intensity
10
12
14
16
18
0
50
100
150
samples in epiboly order

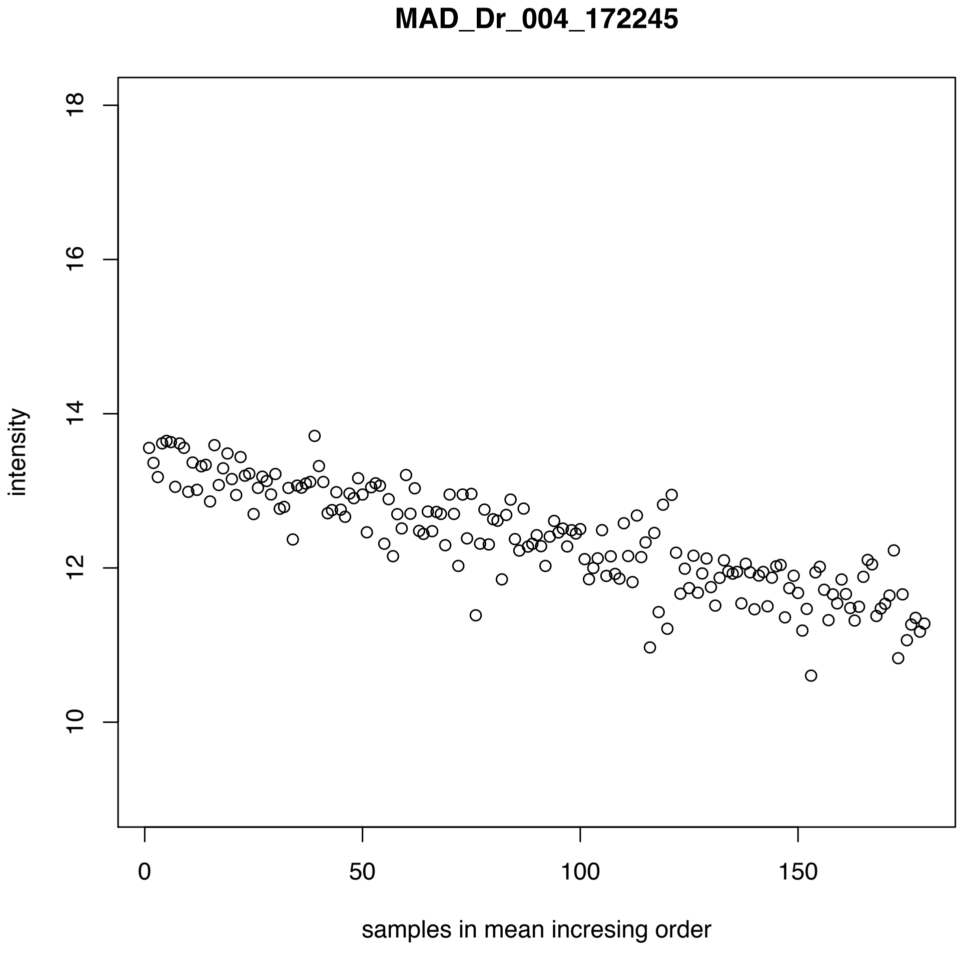
MAD_Dr_004_172245
intensity
10
12
14
16
18
0
50
100
150
samples in mean incresing order

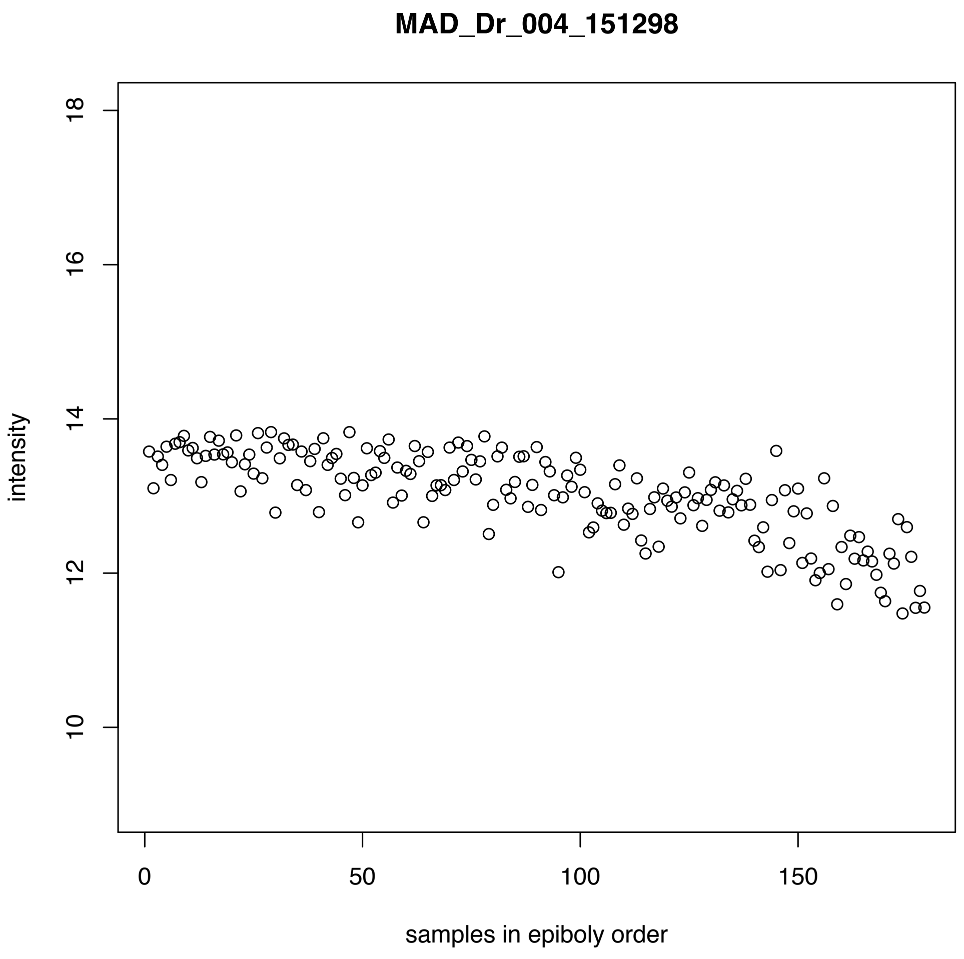
MAD_Dr_004_151298
intensity
10
12
14
16
18
0
50
100
150
samples in epiboly order

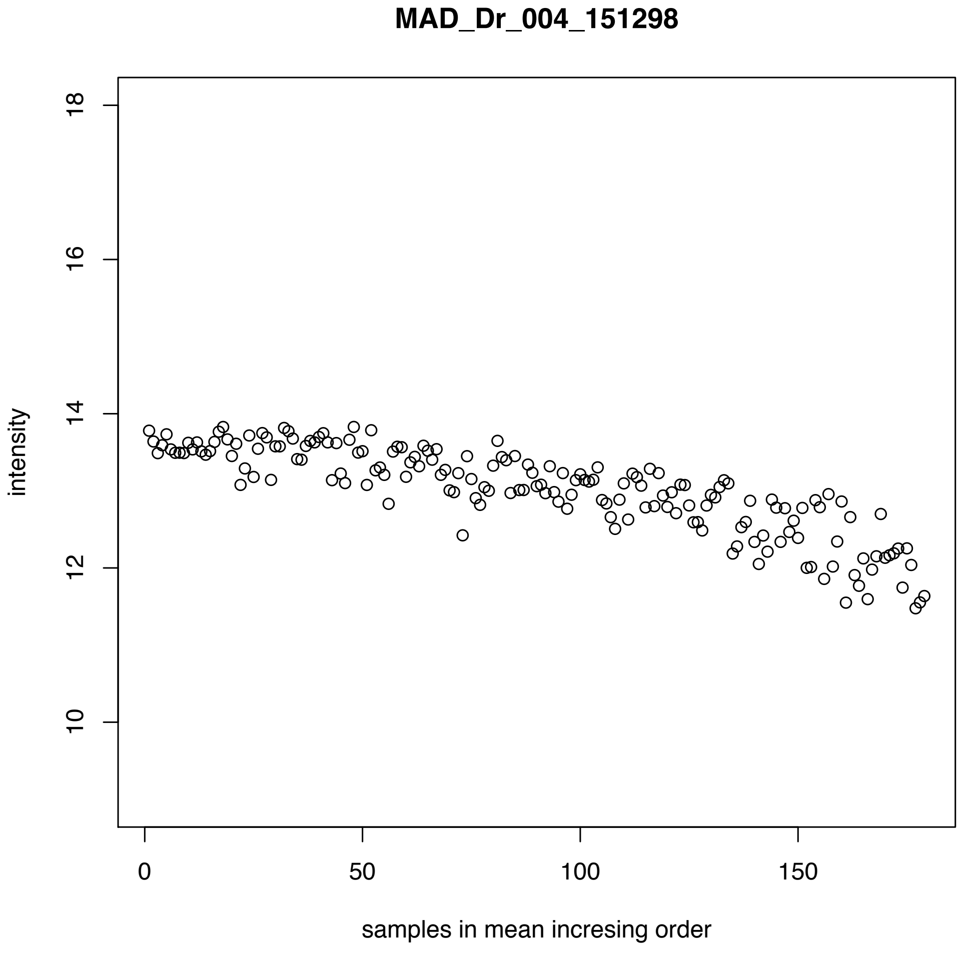
MAD_Dr_004_151298
intensity
10
12
14
16
18
0
50
100
150
samples in mean incresing order

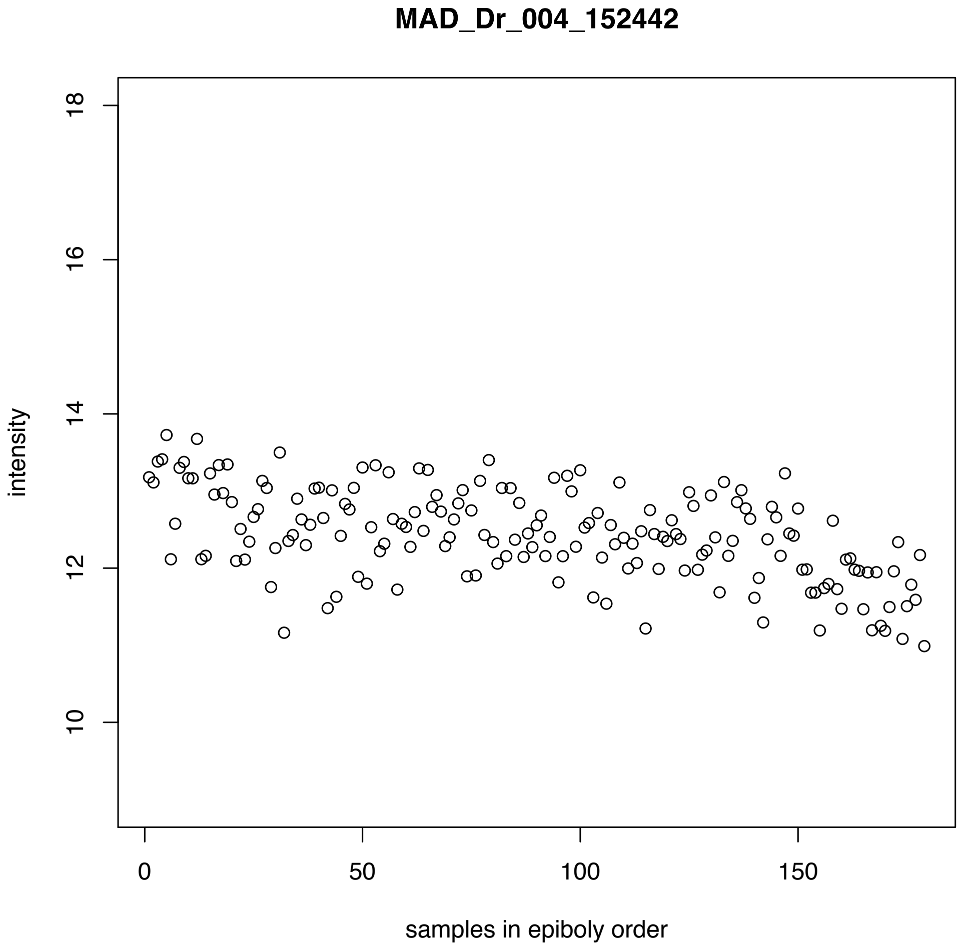
MAD_Dr_004_152442
intensity
10
12
14
16
18
0
50
100
150
samples in epiboly order

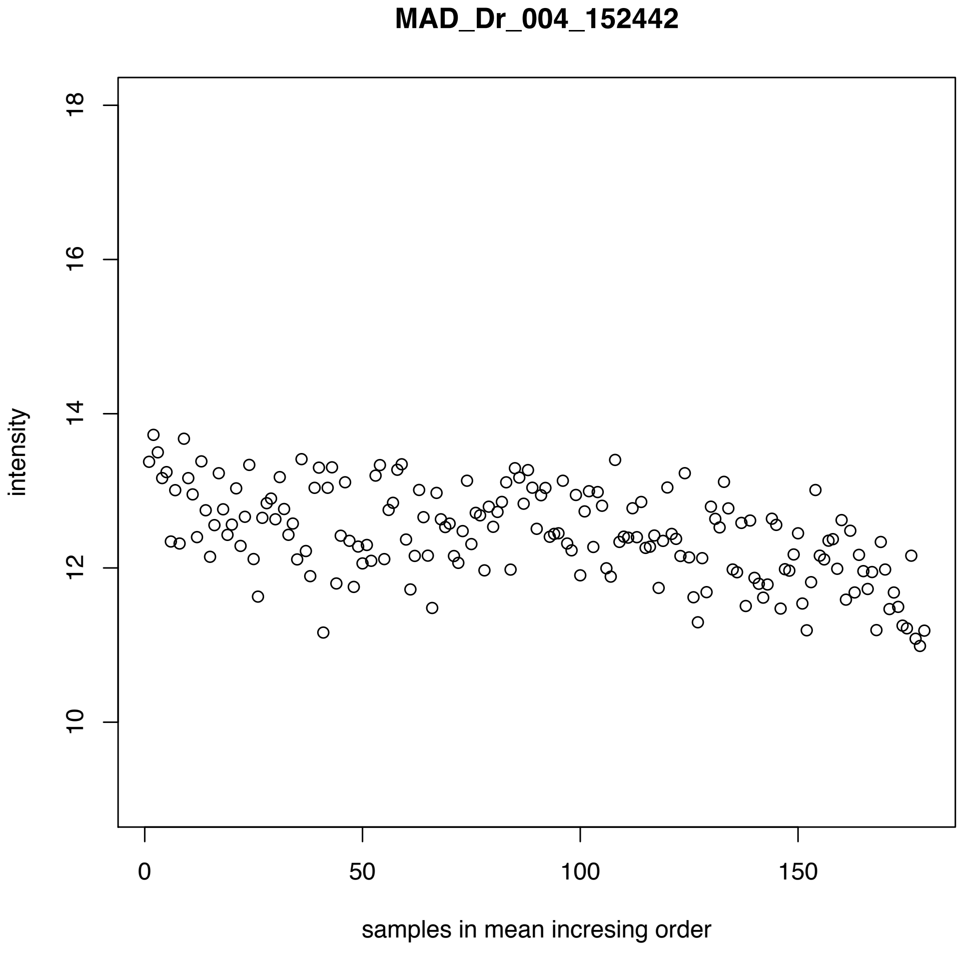
MAD_Dr_004_152442
intensity
10
12
14
16
18
0
50
100
150
samples in mean incresing order

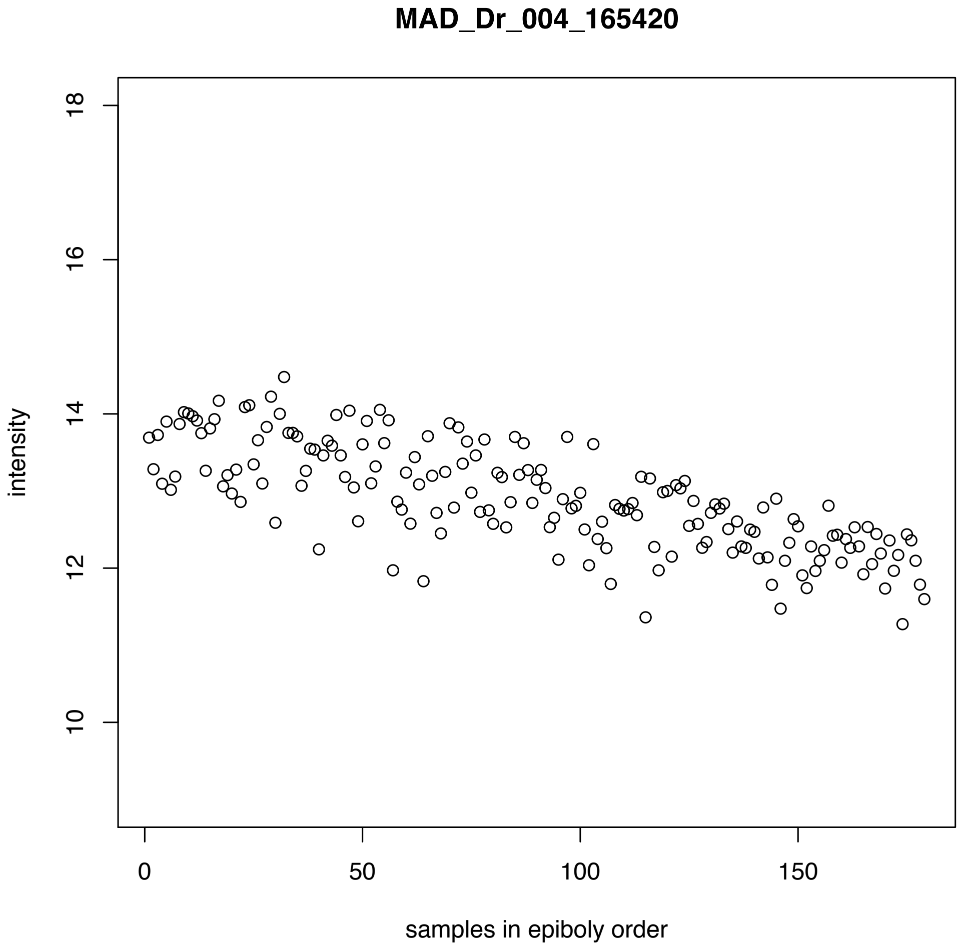
MAD_Dr_004_165420
intensity
samples in epiboly order
10
12
14
16
18
0
50
100
150

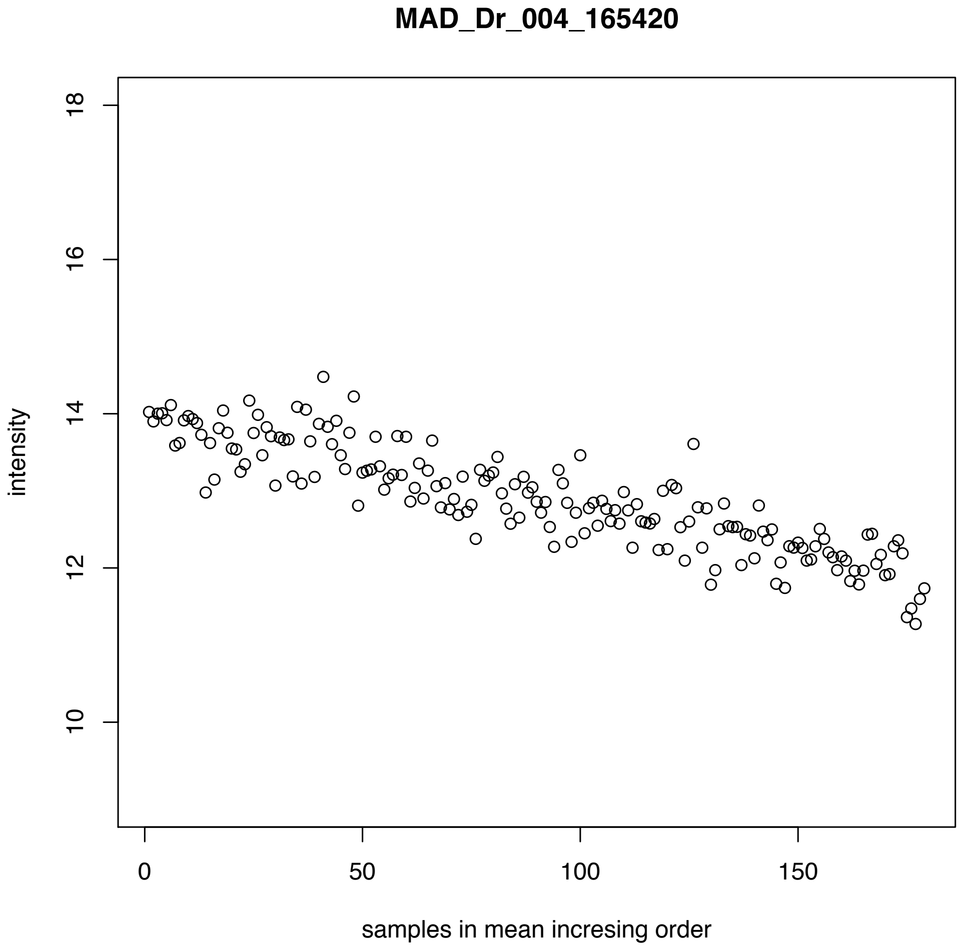
MAD_Dr_004_165420
intensity
samples in mean incresing order
10
12
14
16
18
0
50
100
150

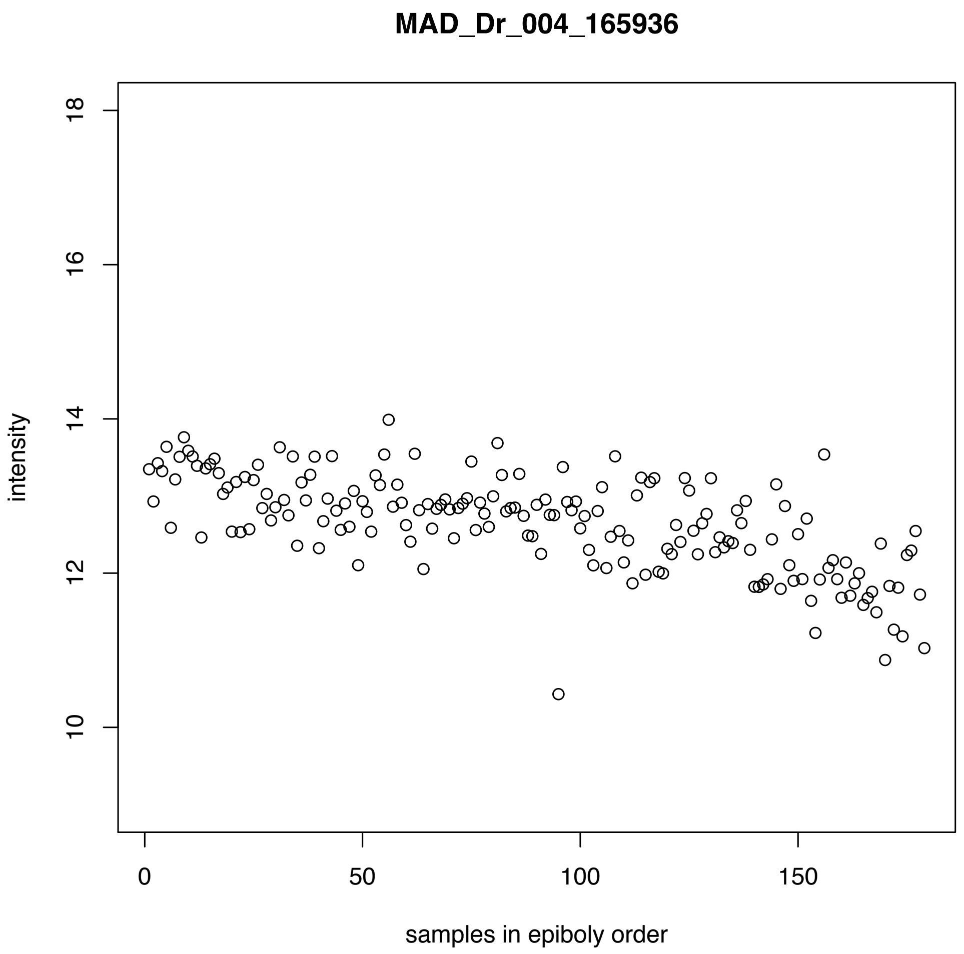
MAD_Dr_004_165936
intensity
10
12
14
16
18
0
50
100
150
samples in epiboly order

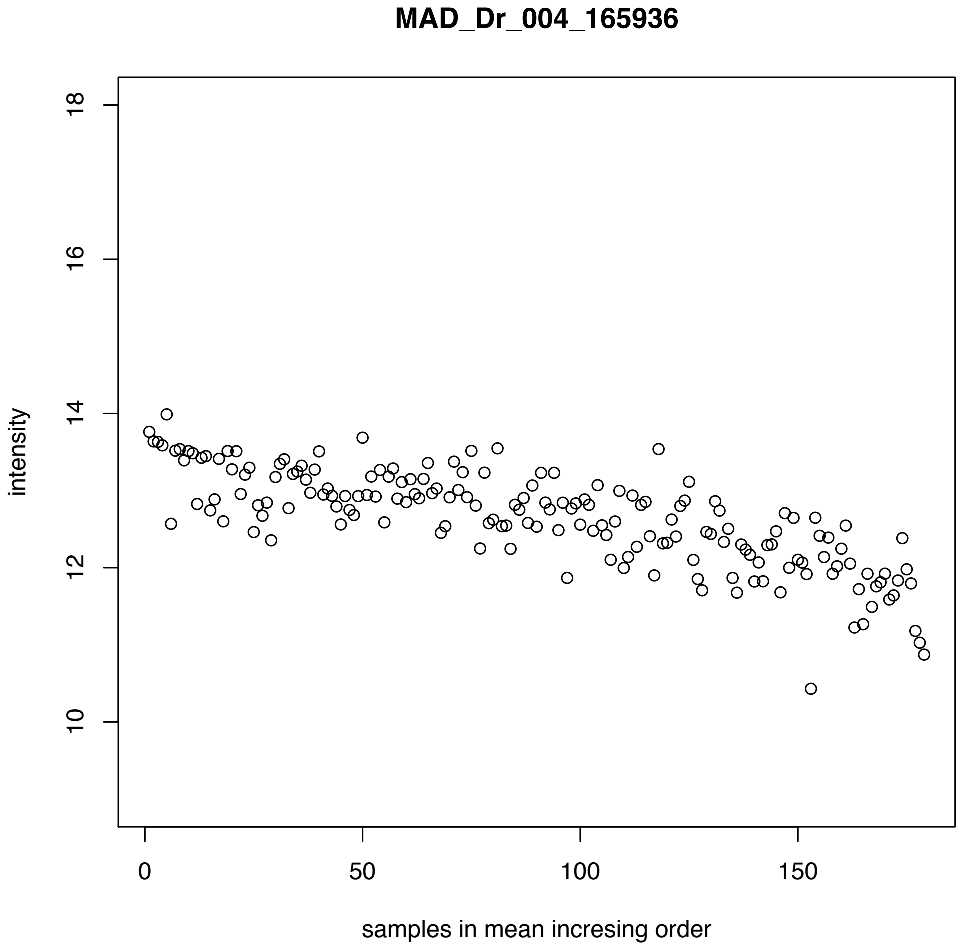
MAD_Dr_004_165936
intensity
10
12
14
16
18
0
50
100
150
samples in mean incresing order

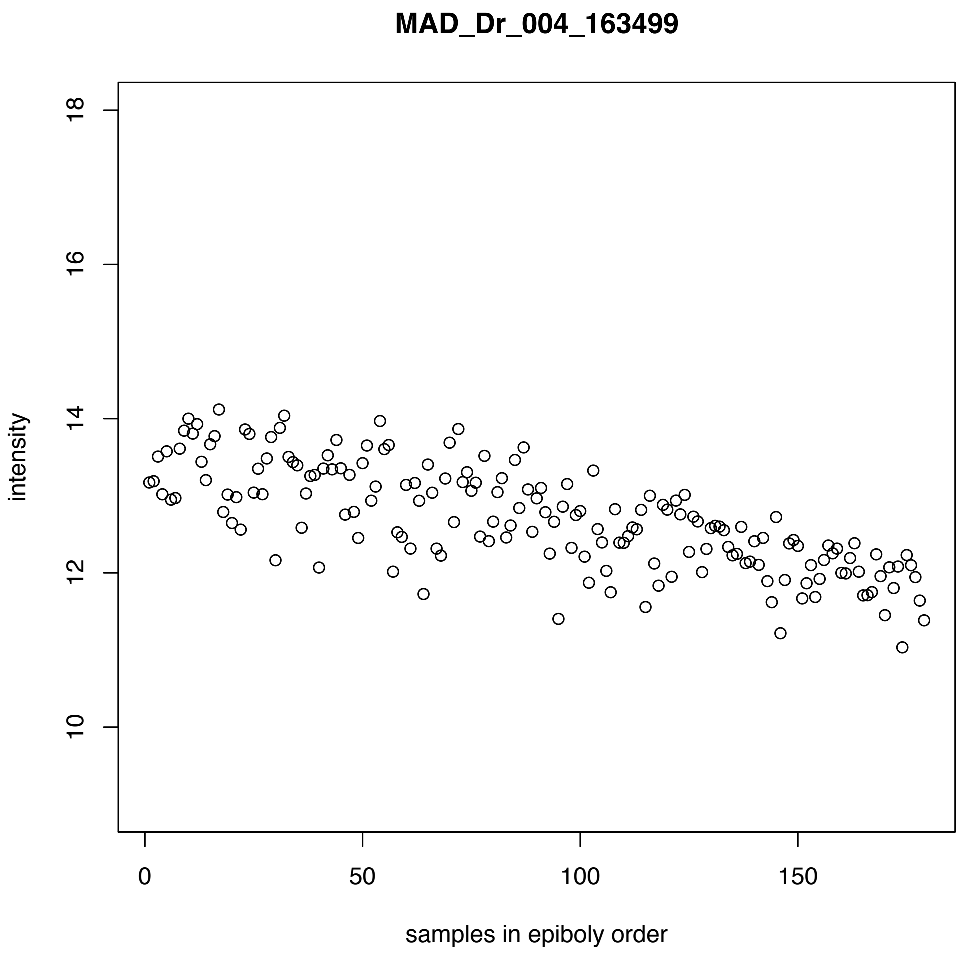
MAD_Dr_004_163499
intensity
10
12
14
16
18
0
50
100
150
samples in epiboly order

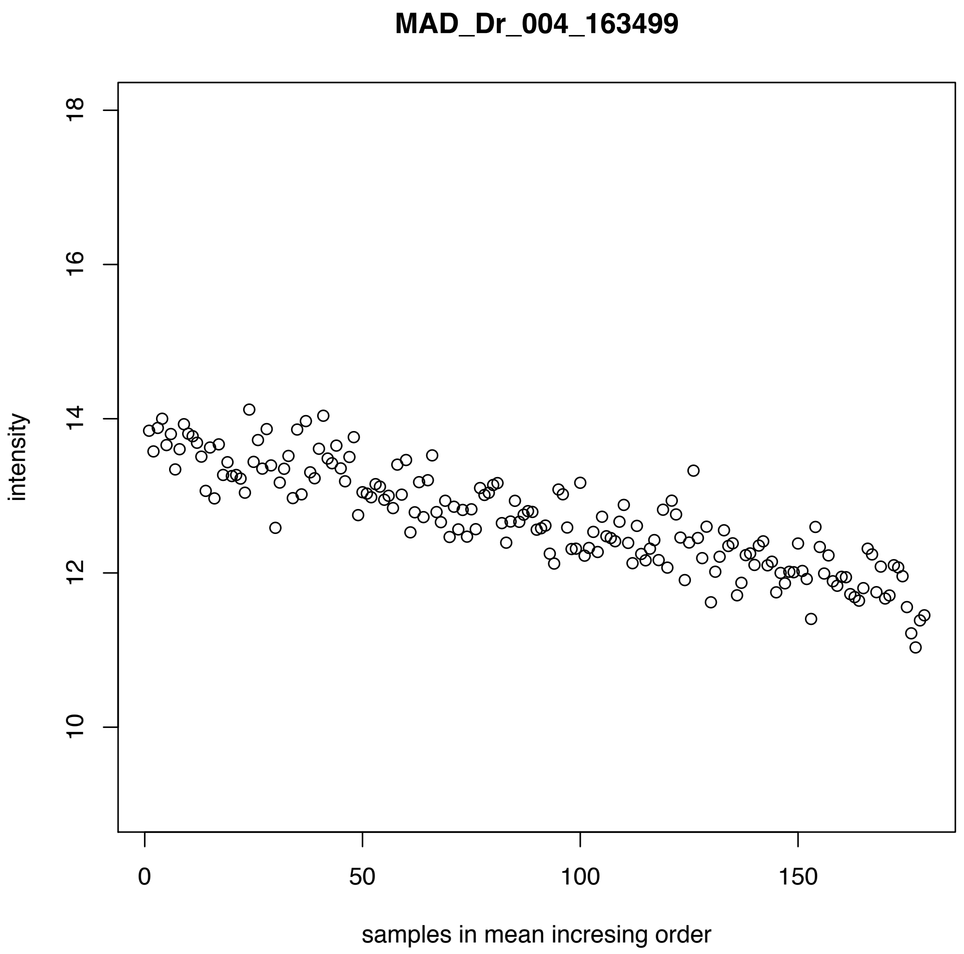
MAD_Dr_004_163499
intensity
10
12
14
16
18
0
50
100
150
samples in mean incresing order

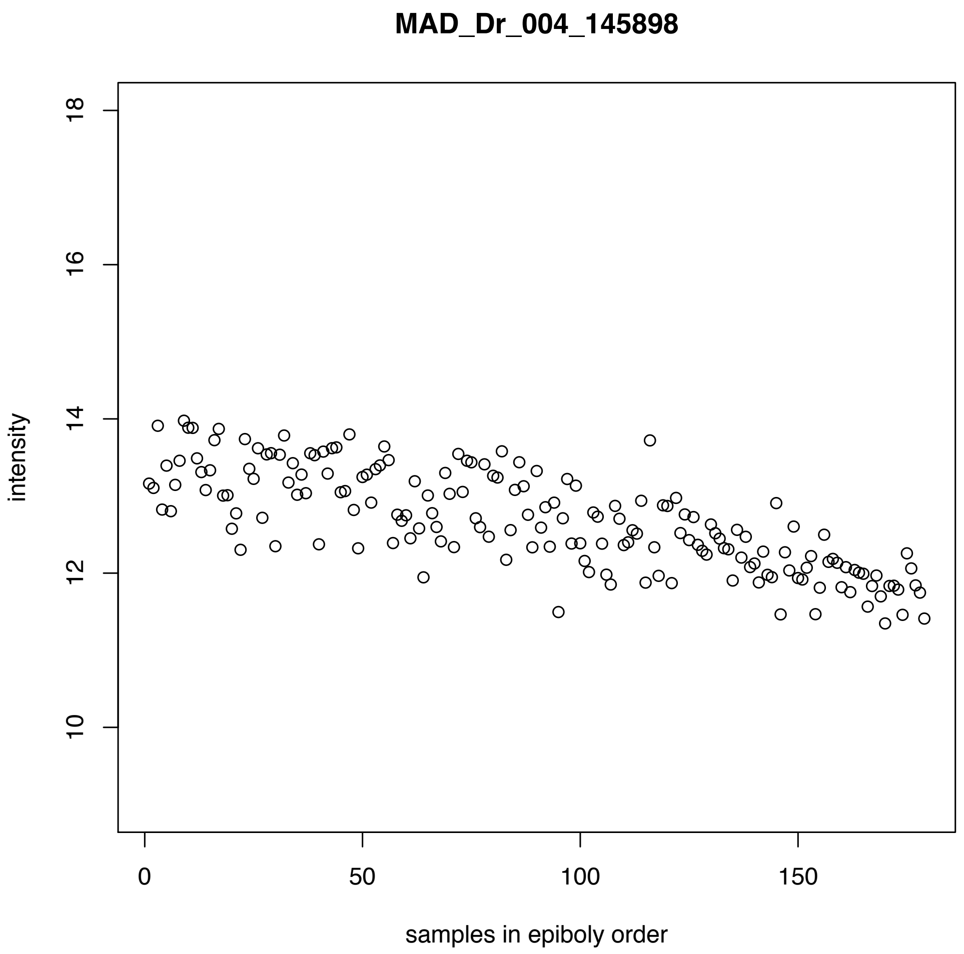
MAD_Dr_004_145898
intensity
10
12
14
16
18
0
50
100
150
samples in epiboly order

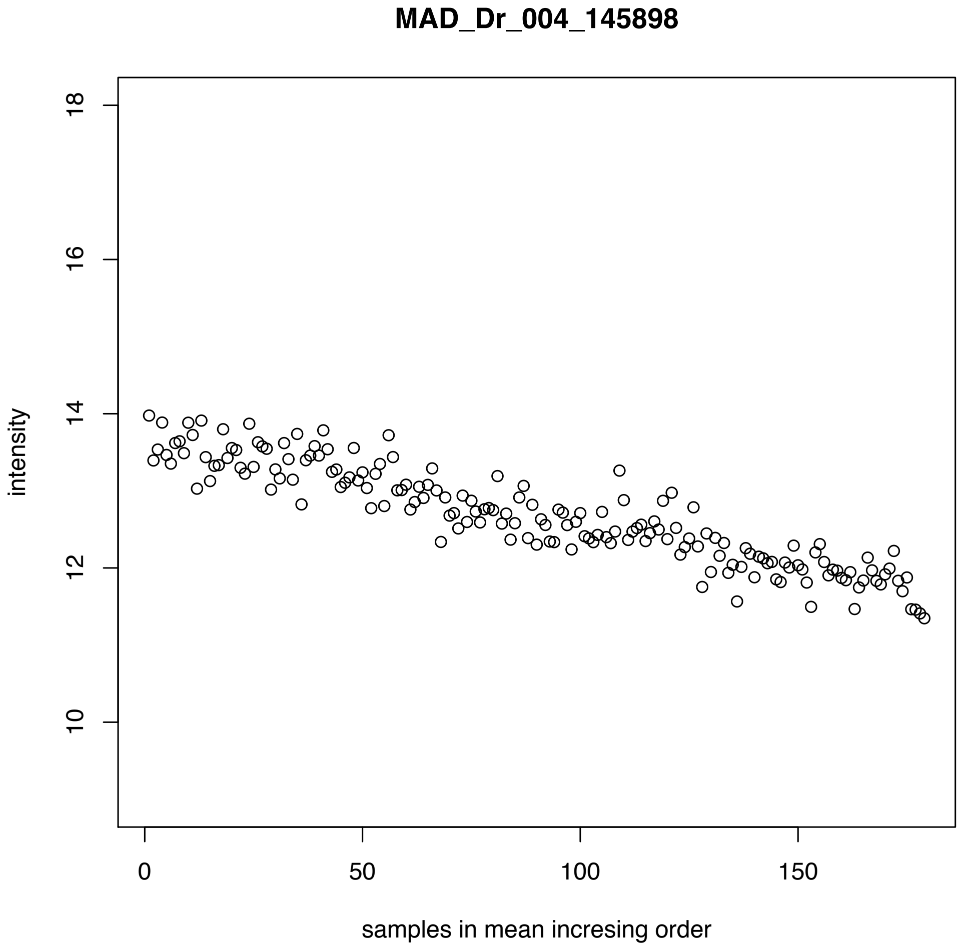
MAD_Dr_004_145898
intensity
10
12
14
16
18
0
50
100
150
samples in mean incresing order

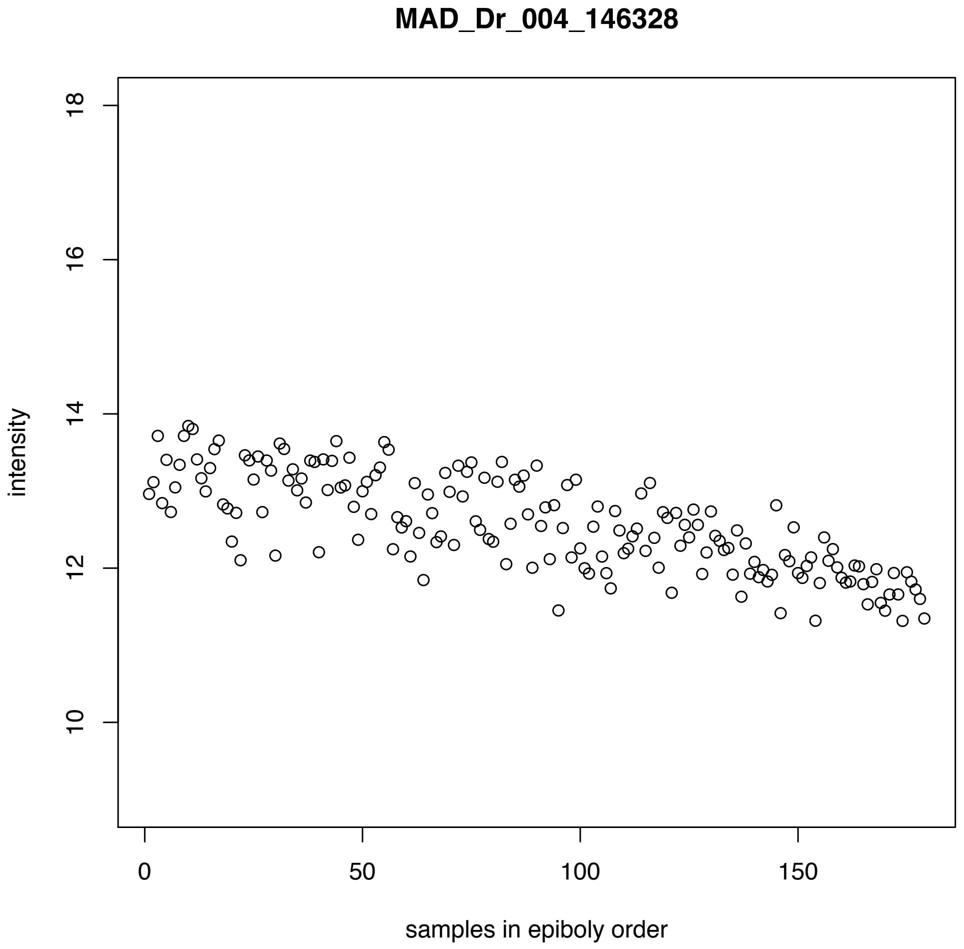

MAD_Dr_004_146328
intensity
10
12
14
16
18
0
50
100
150
samples in epiboly order

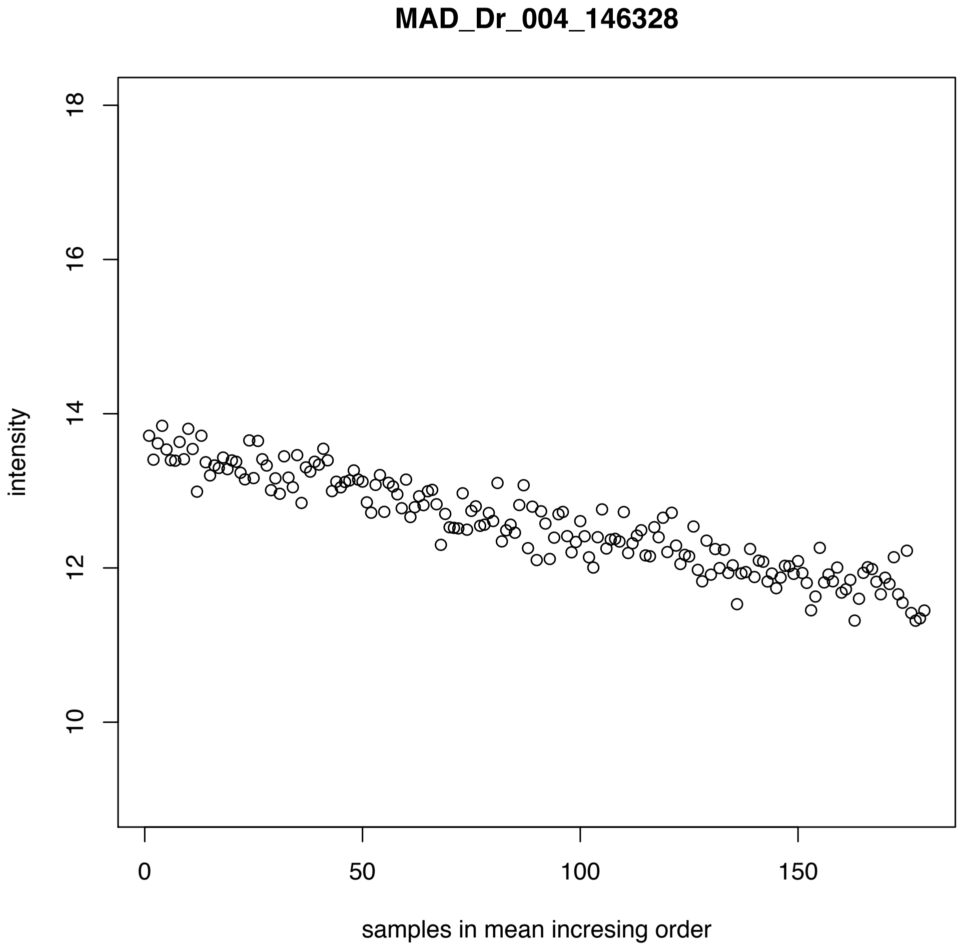

MAD_Dr_004_146328
intensity
10
12
14
16
18
0
50
100
150
samples in mean incresing order

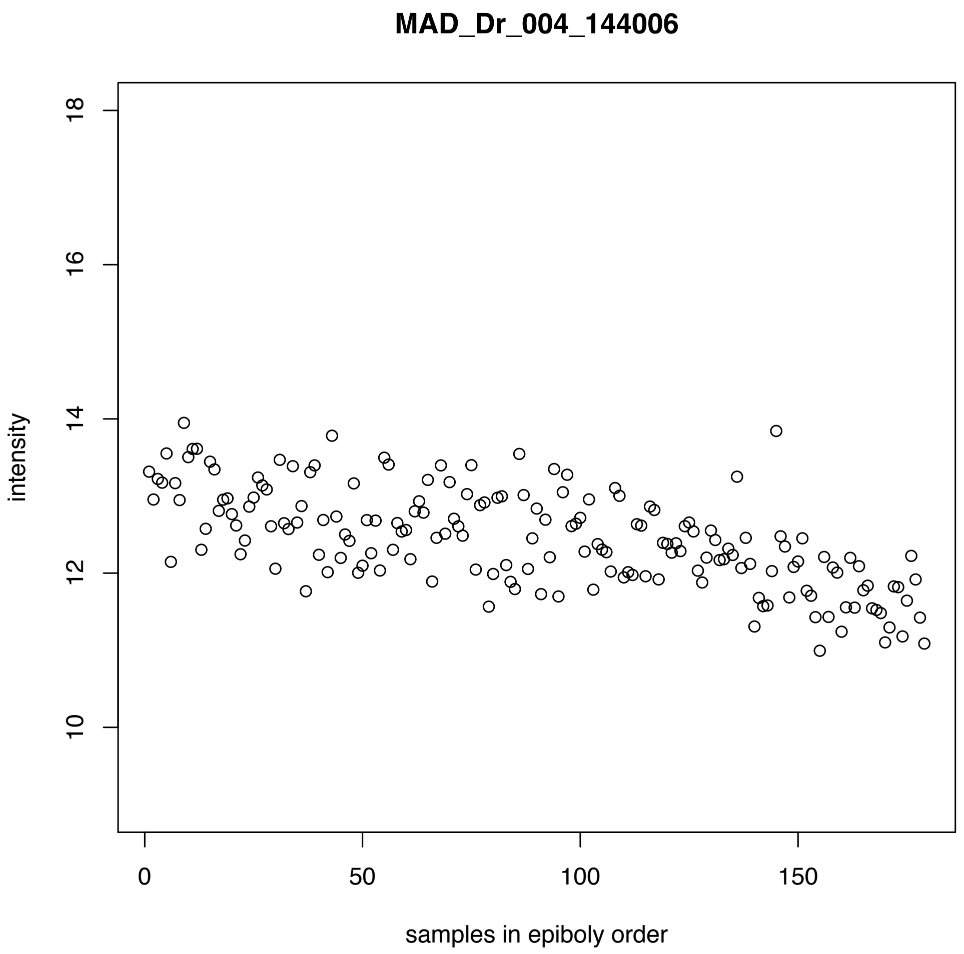
MAD_Dr_004_144006
intensity
samples in epiboly order
10
12
14
16
18
0
50
100
150

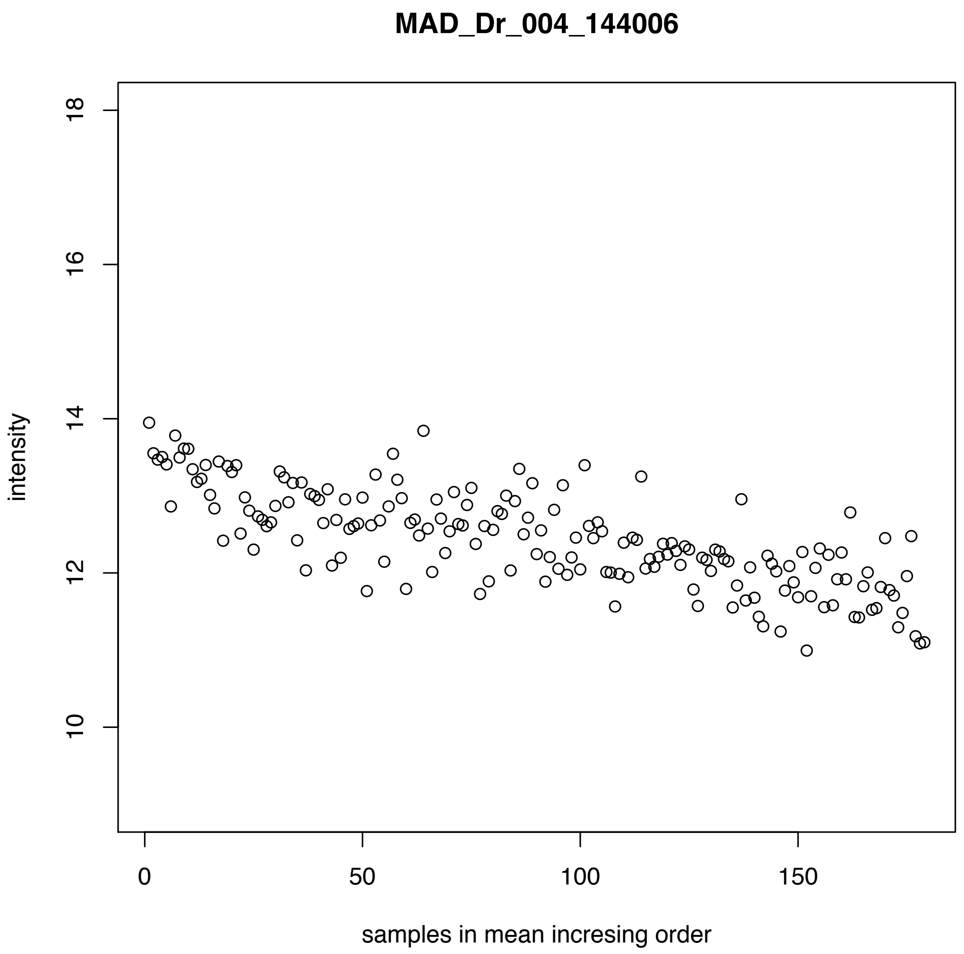
MAD_Dr_004_144006
intensity
samples in mean incresing order
10
12
14
16
18
0
50
100
150

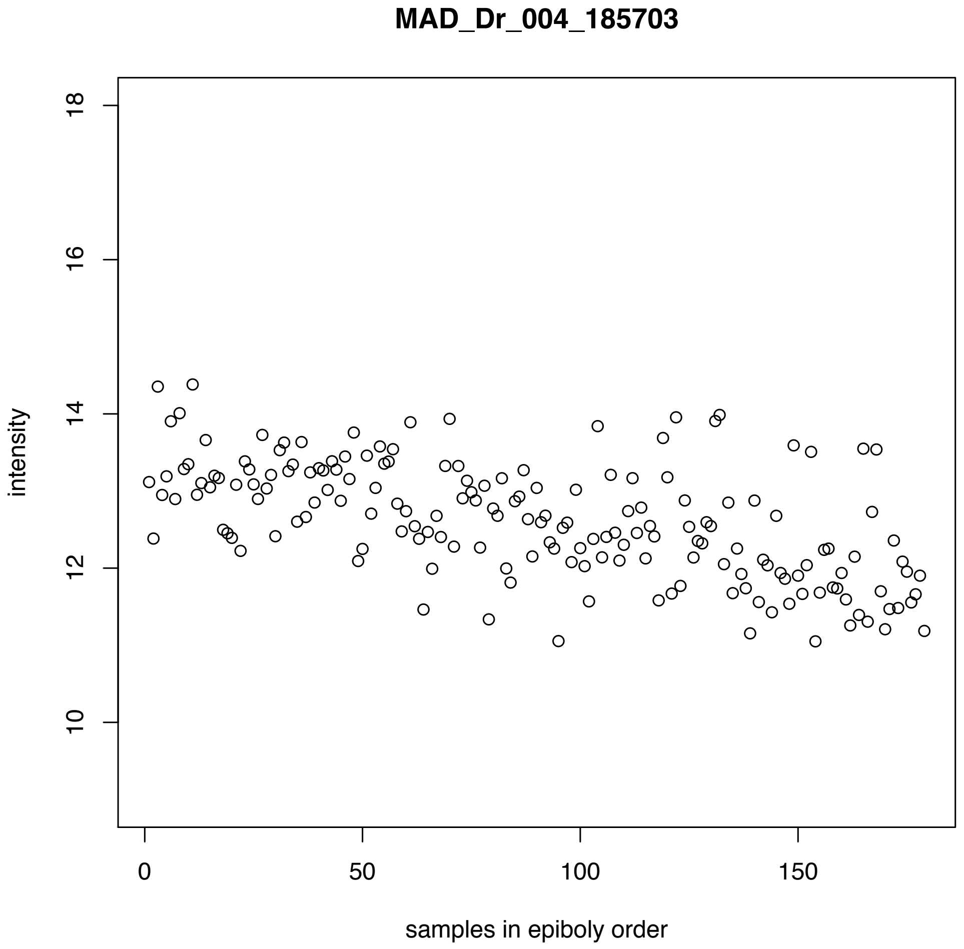
MAD_Dr_004_185703
intensity
samples in epiboly order
10
12
14
16
18
0
50
100
150

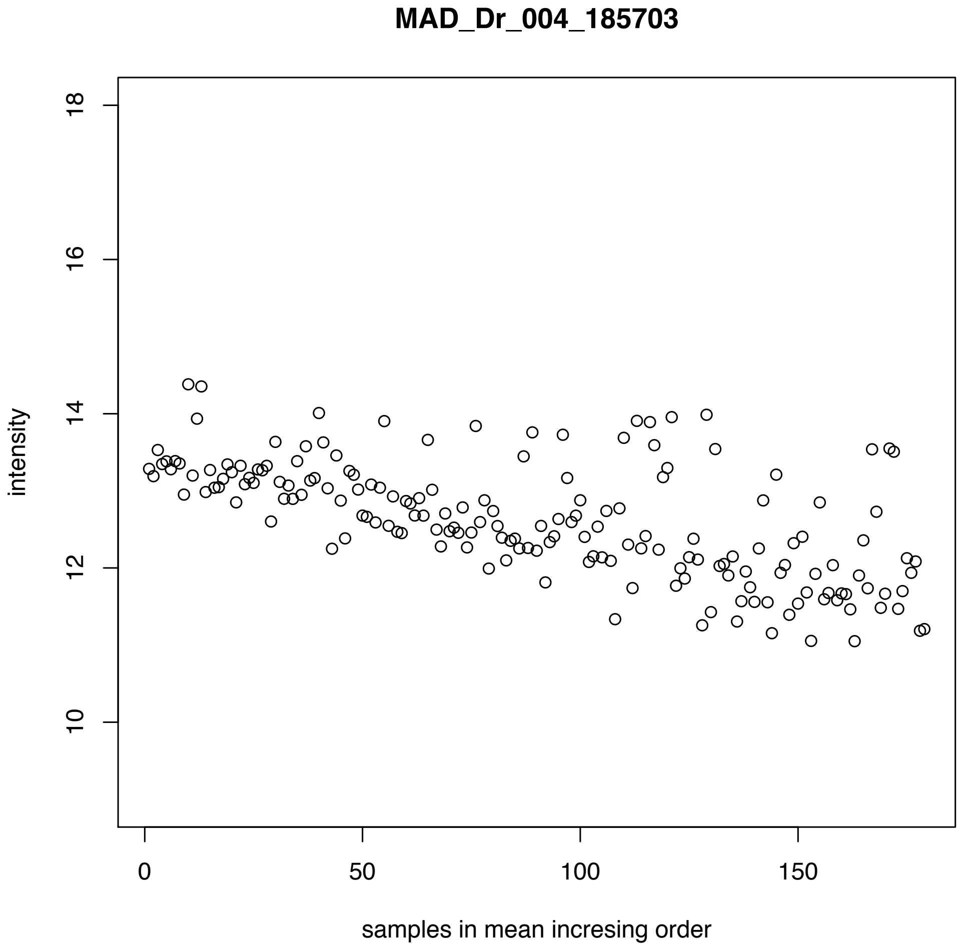
MAD_Dr_004_185703
intensity
samples in mean incresing order
10
12
14
16
18
0
50
100
150

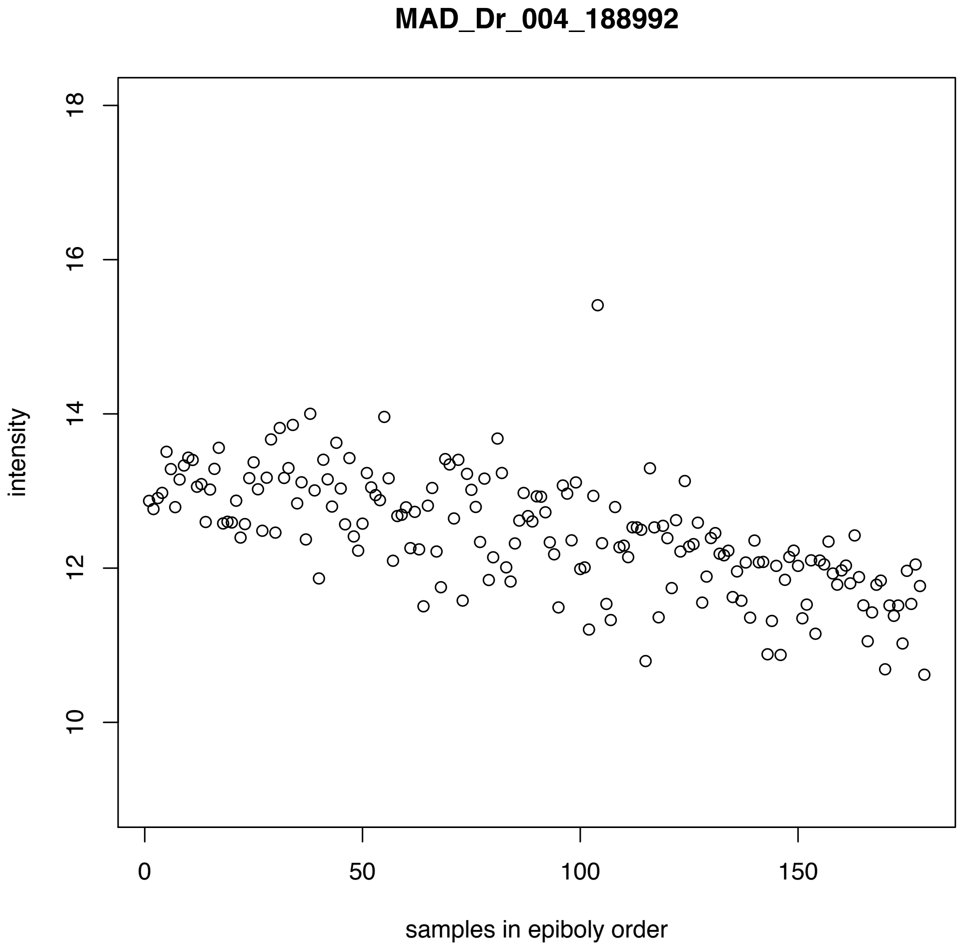

MAD_Dr_004_188992
intensity
10
12
14
16
18
0
50
100
150
samples in epiboly order

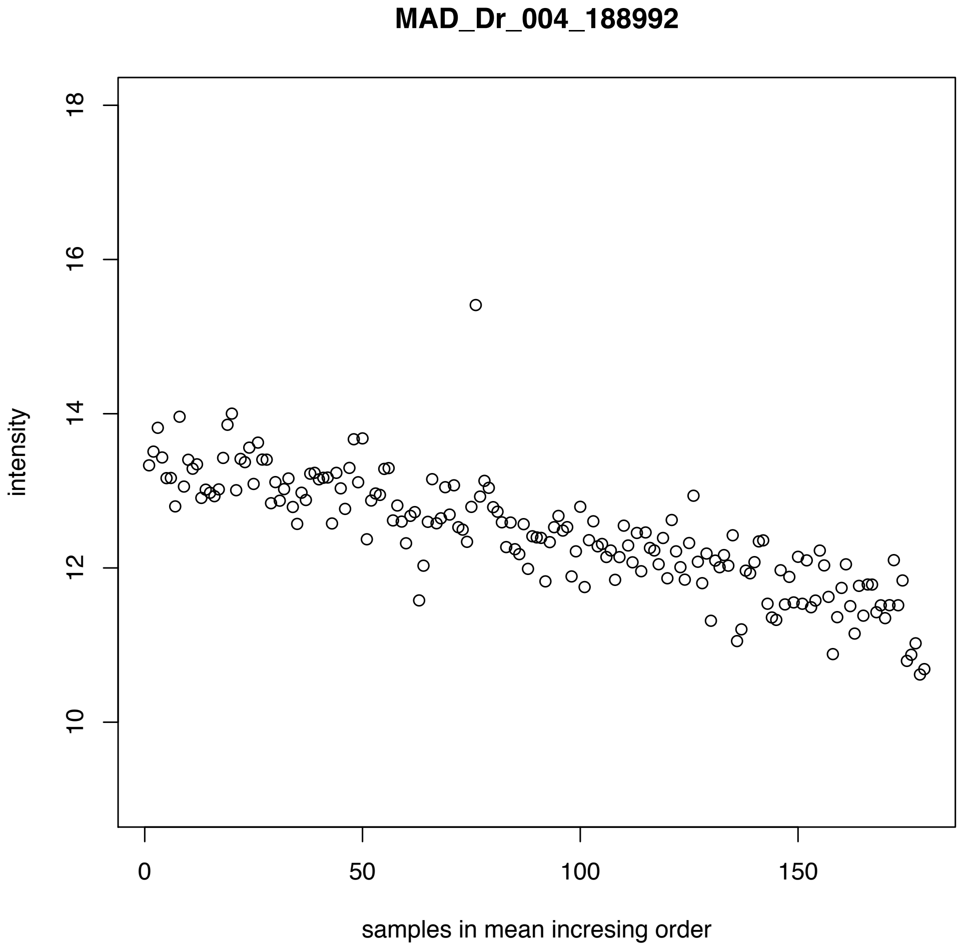

MAD_Dr_004_188992
intensity
10
12
14
16
18
0
50
100
150
samples in mean incresing order

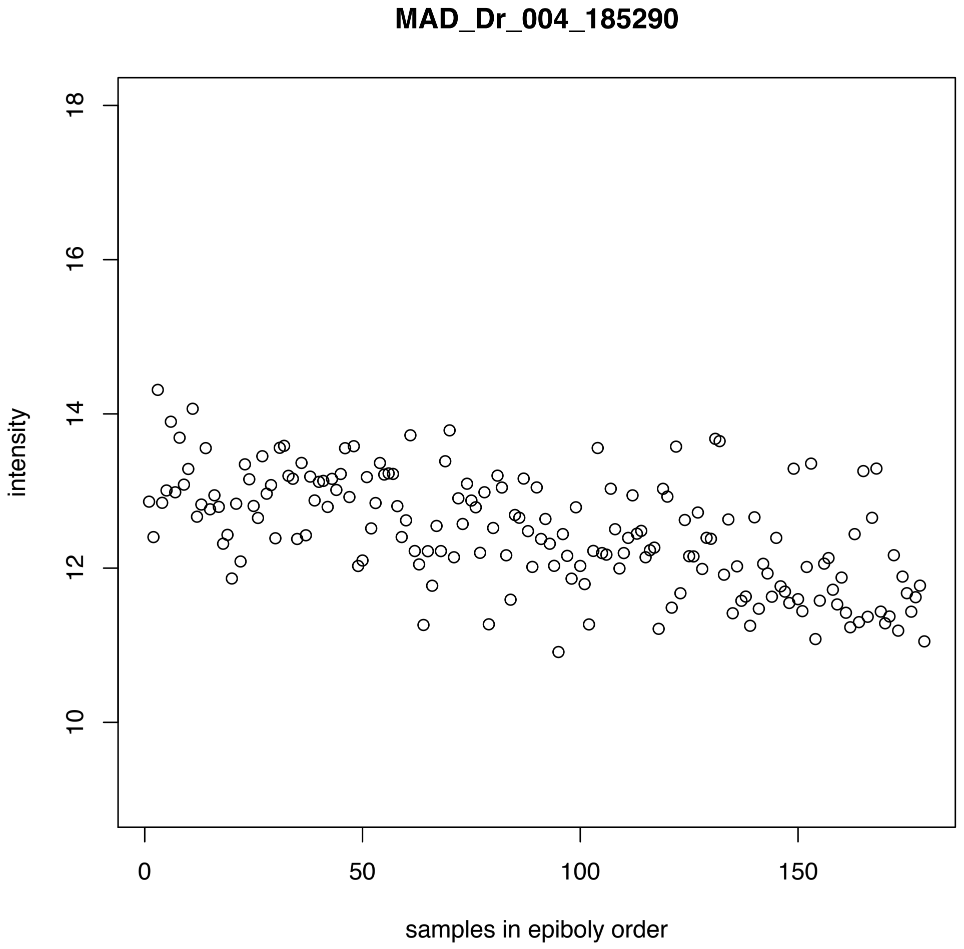
MAD_Dr_004_185290
intensity
10
12
14
16
18
0
50
100
150
samples in epiboly order

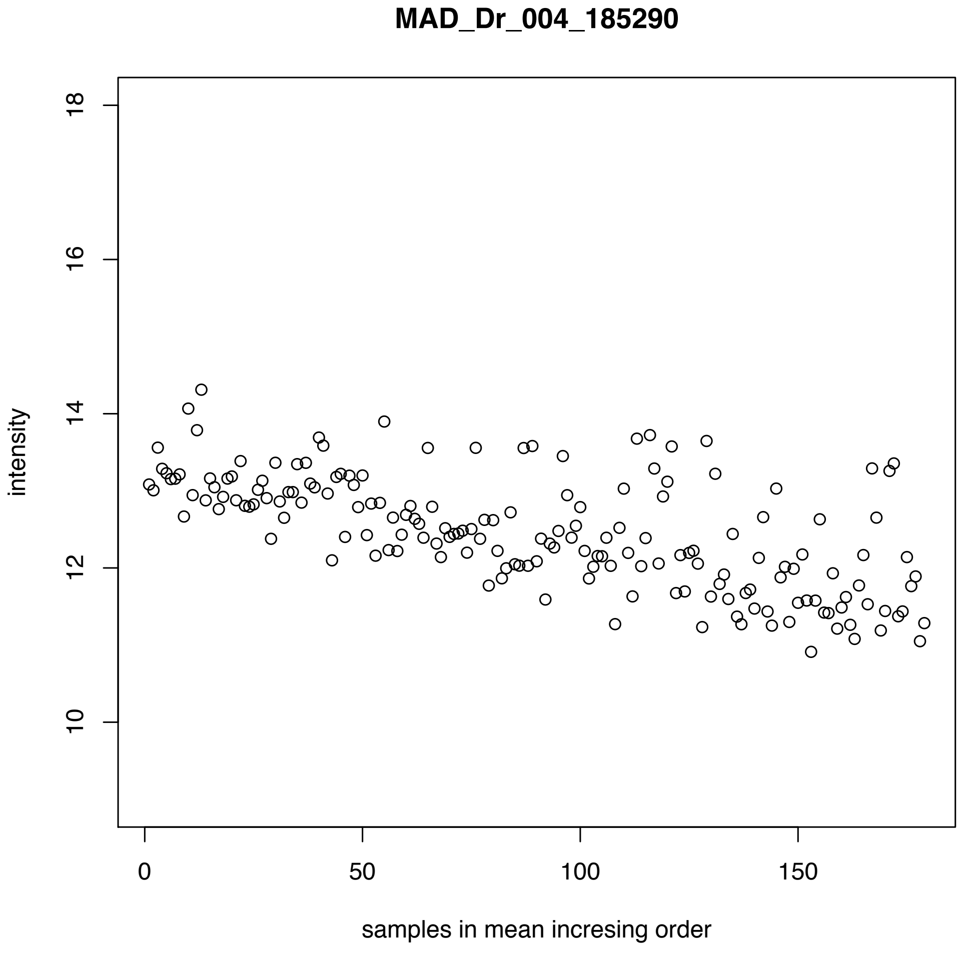
MAD_Dr_004_185290
intensity
10
12
14
16
18
0
50
100
150
samples in mean incresing order

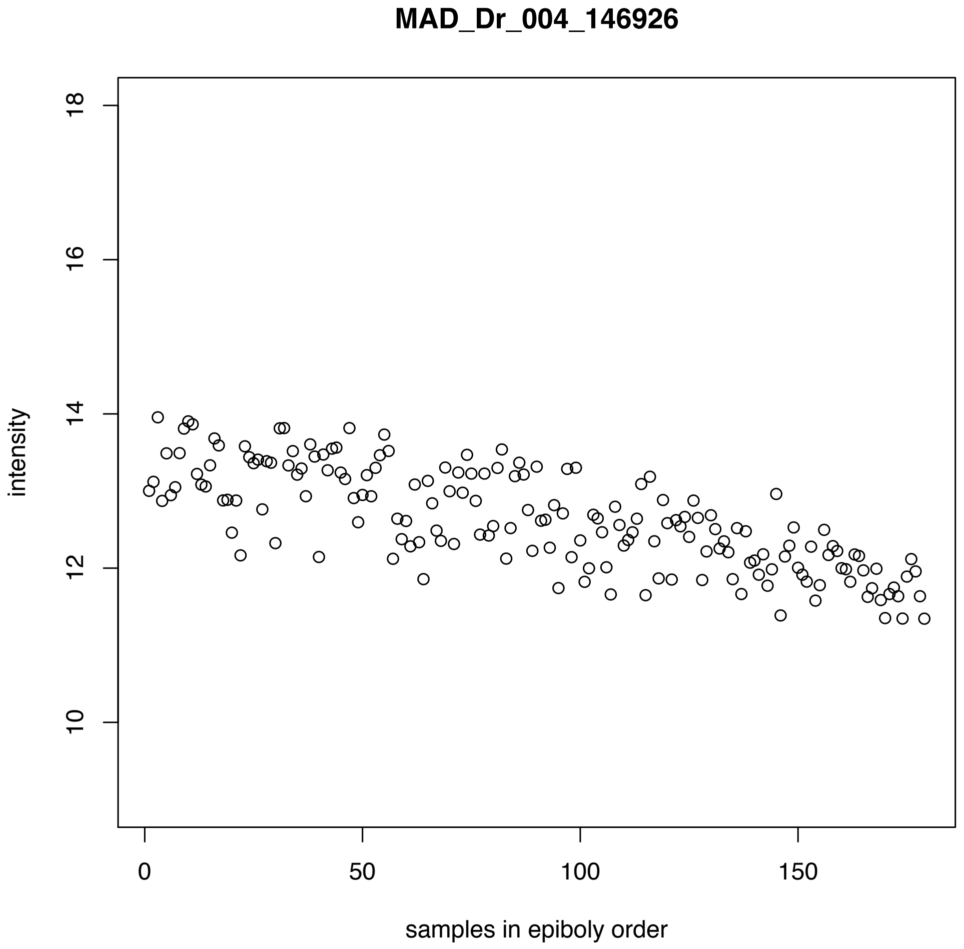
MAD_Dr_004_146926
intensity
10
12
14
16
18
0
50
100
150
samples in epiboly order

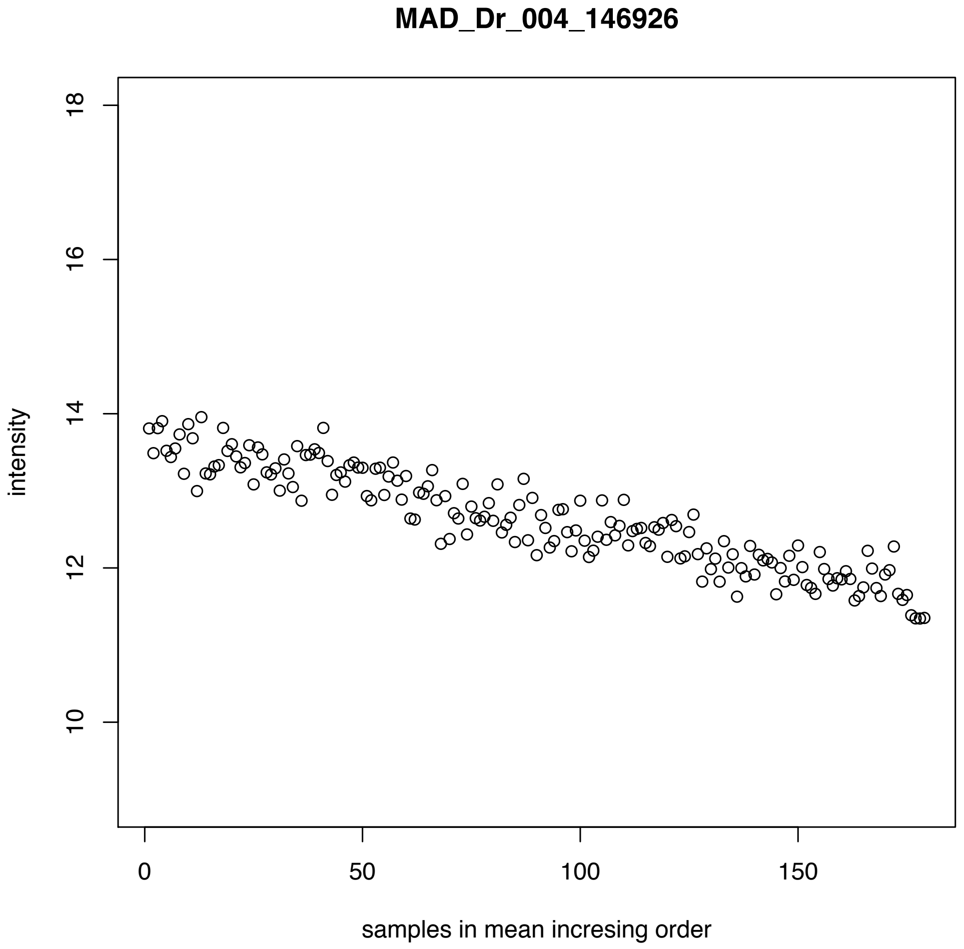
MAD_Dr_004_146926
intensity
10
12
14
16
18
0
50
100
150
samples in mean incresing order

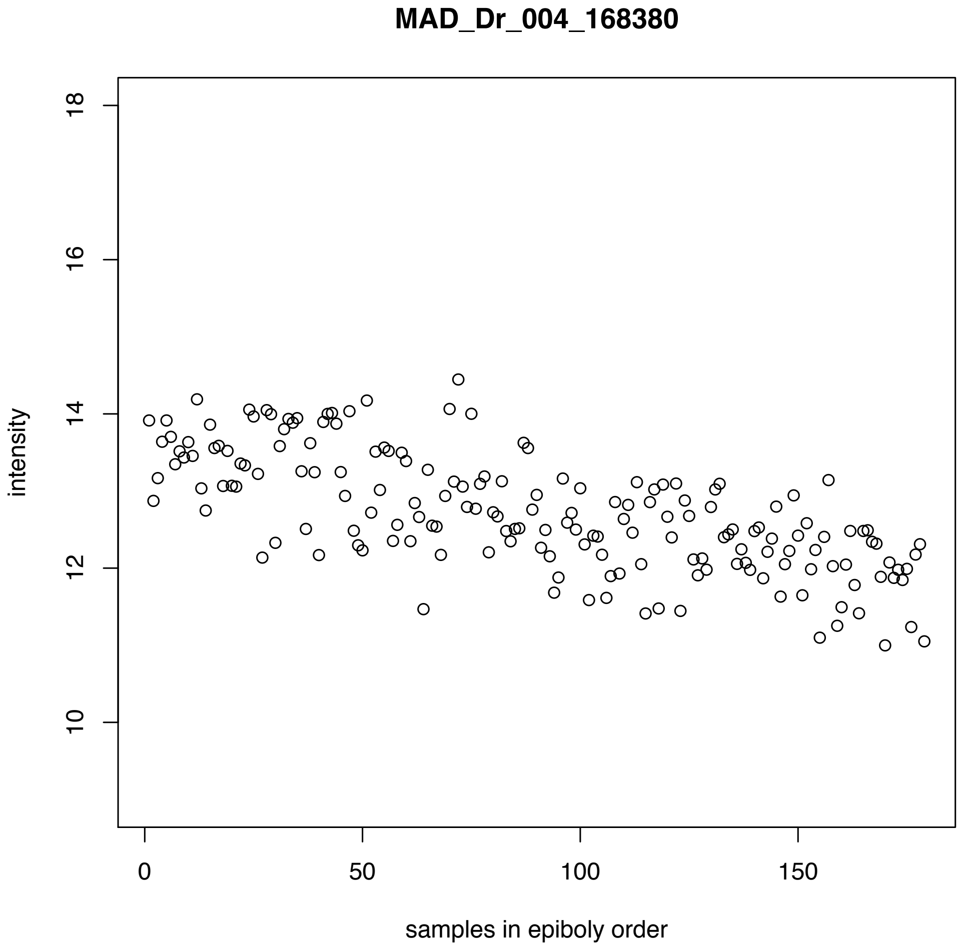
MAD_Dr_004_168380
intensity
18
16
14
12
10
0
50
100
150
samples in epiboly order

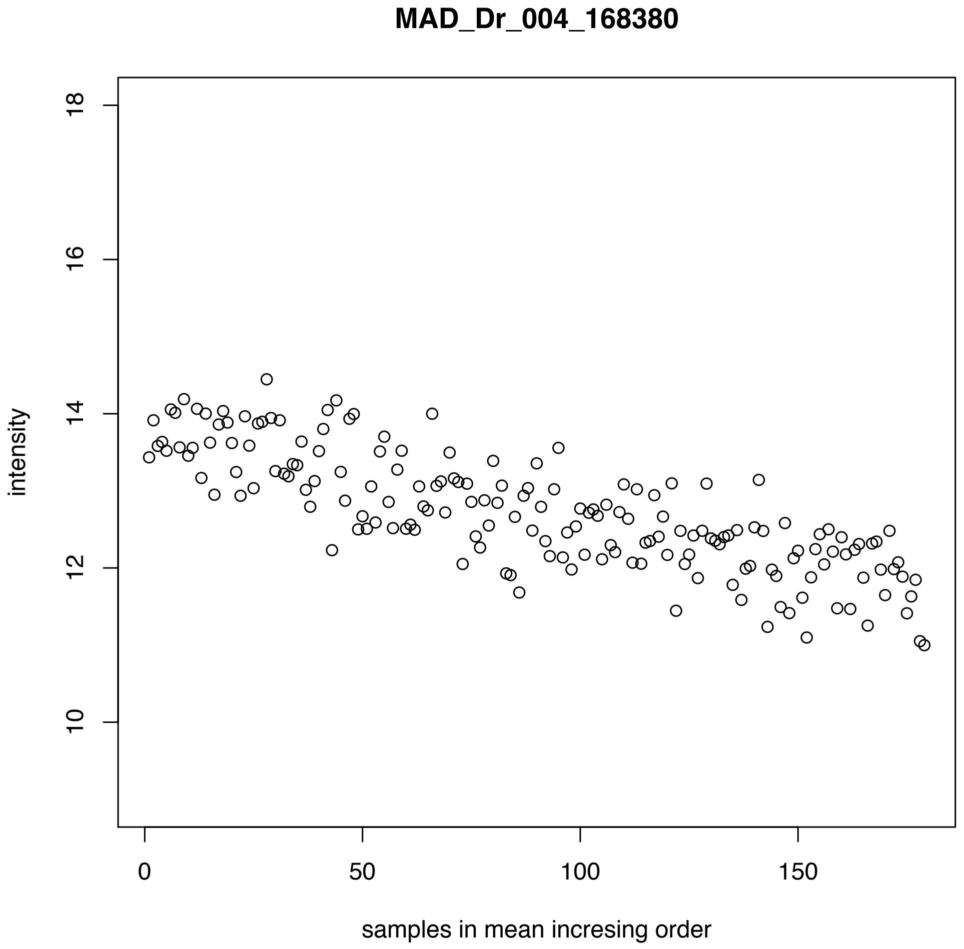
MAD_Dr_004_168380
intensity
18
16
14
12
10
0
50
100
150
samples in mean incresing order

MAD_Dr_004_168641

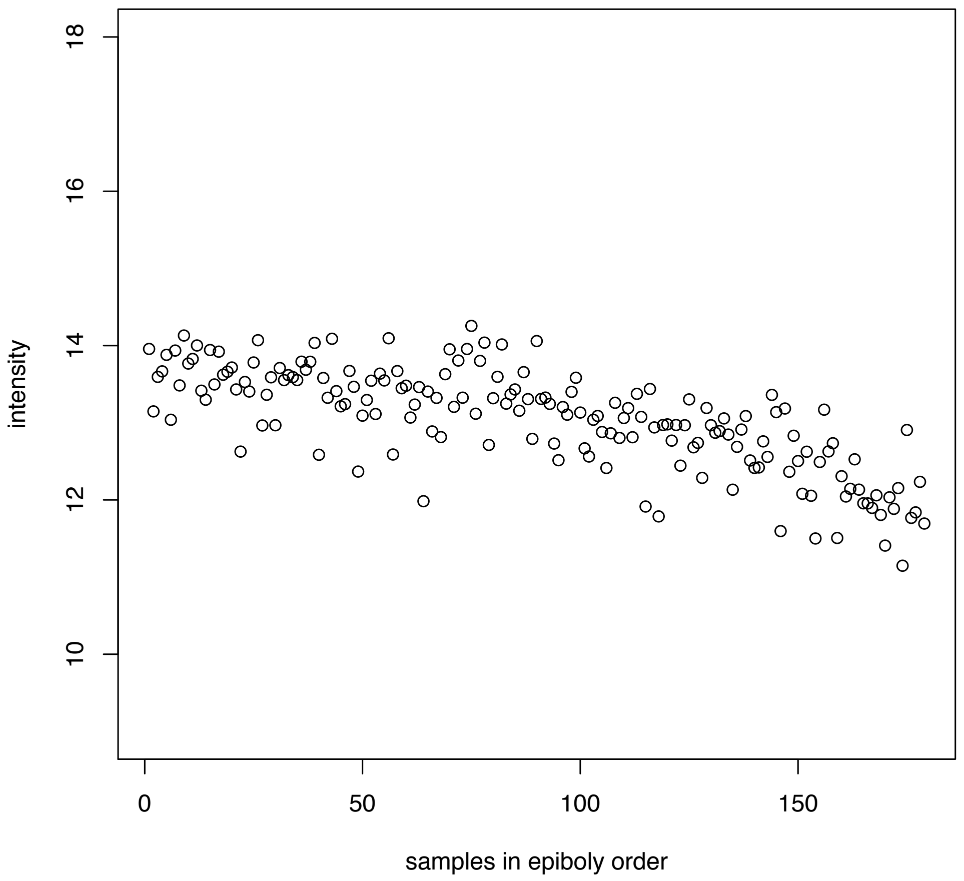

MAD_Dr_004_168641

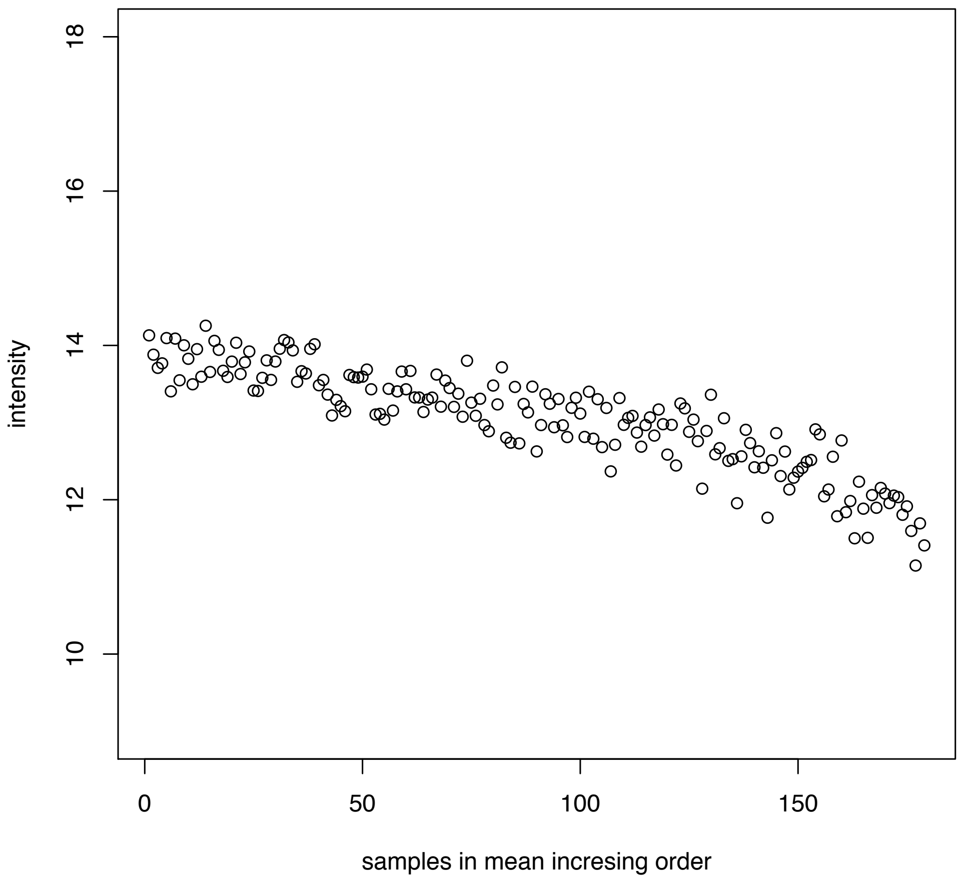

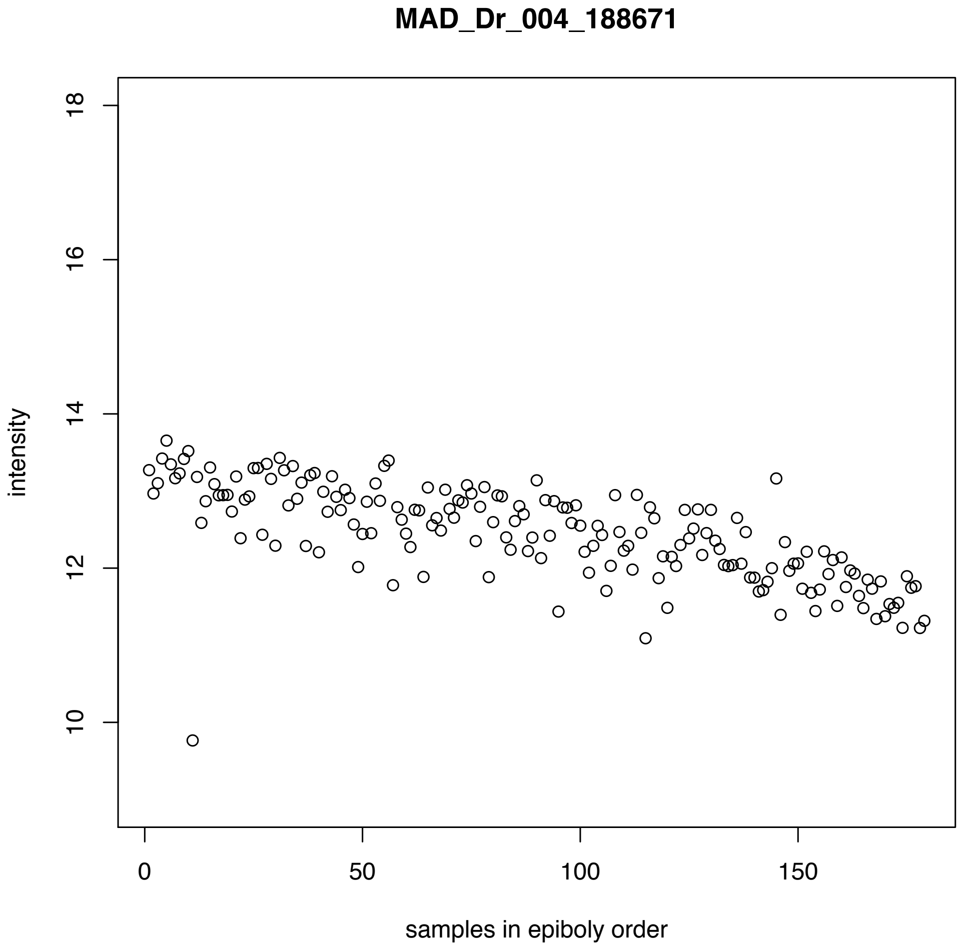
MAD_Dr_004_188671
intensity
10
12
14
16
18
0
50
100
150
samples in epiboly order

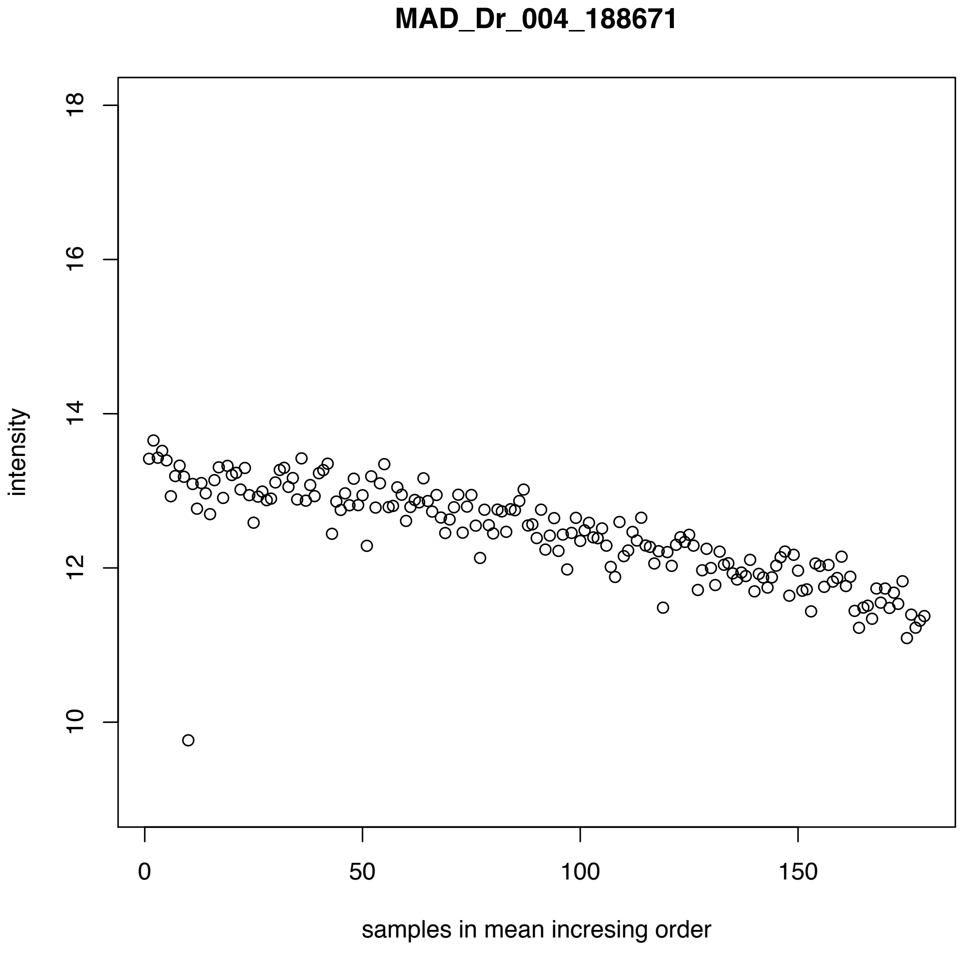
MAD_Dr_004_188671
intensity
10
12
14
16
18
0
50
100
150
samples in mean incresing order

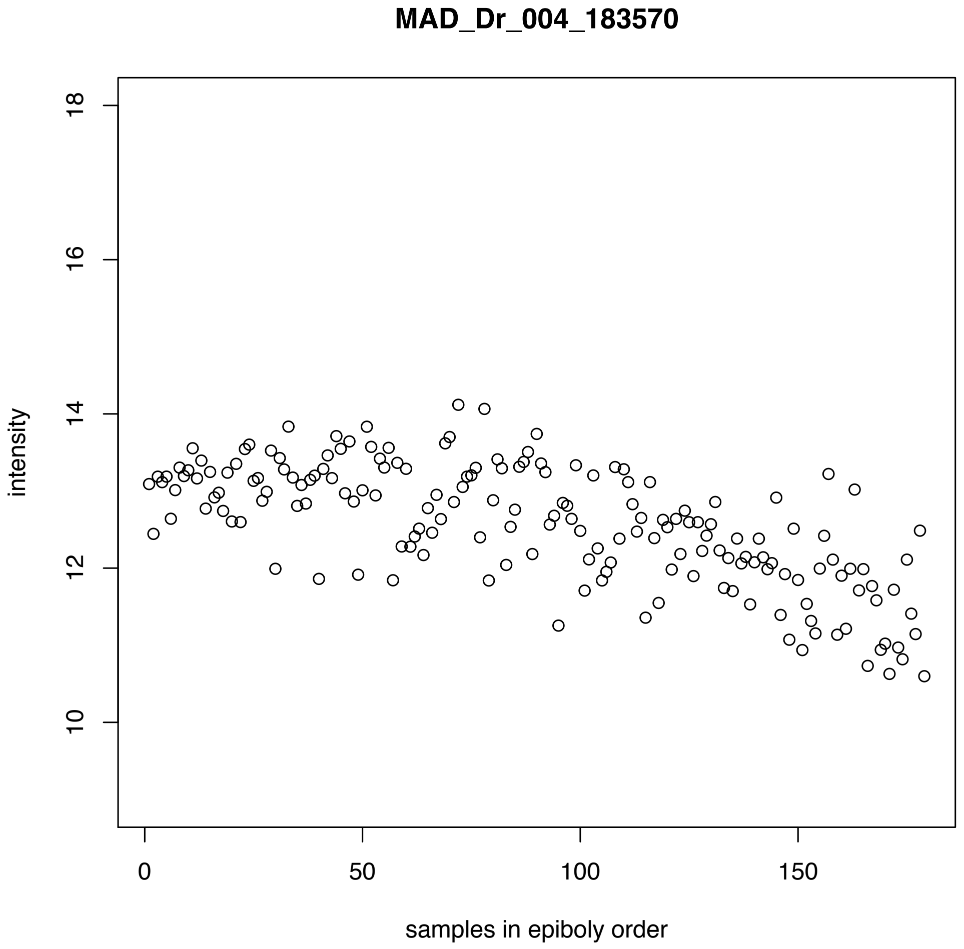
MAD_Dr_004_183570
intensity
18
16
14
12
10
0
50
100
150
samples in epiboly order

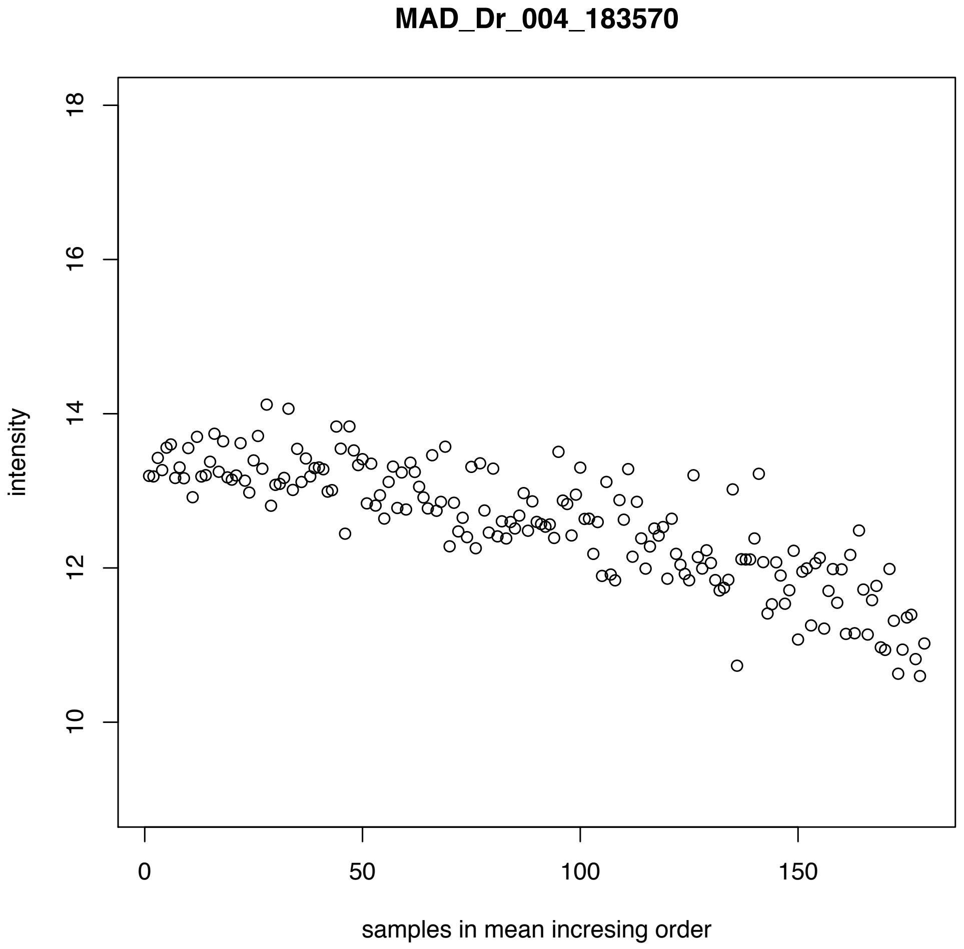
MAD_Dr_004_183570
intensity
18
16
14
12
10
0
50
100
150
samples in mean incresing order

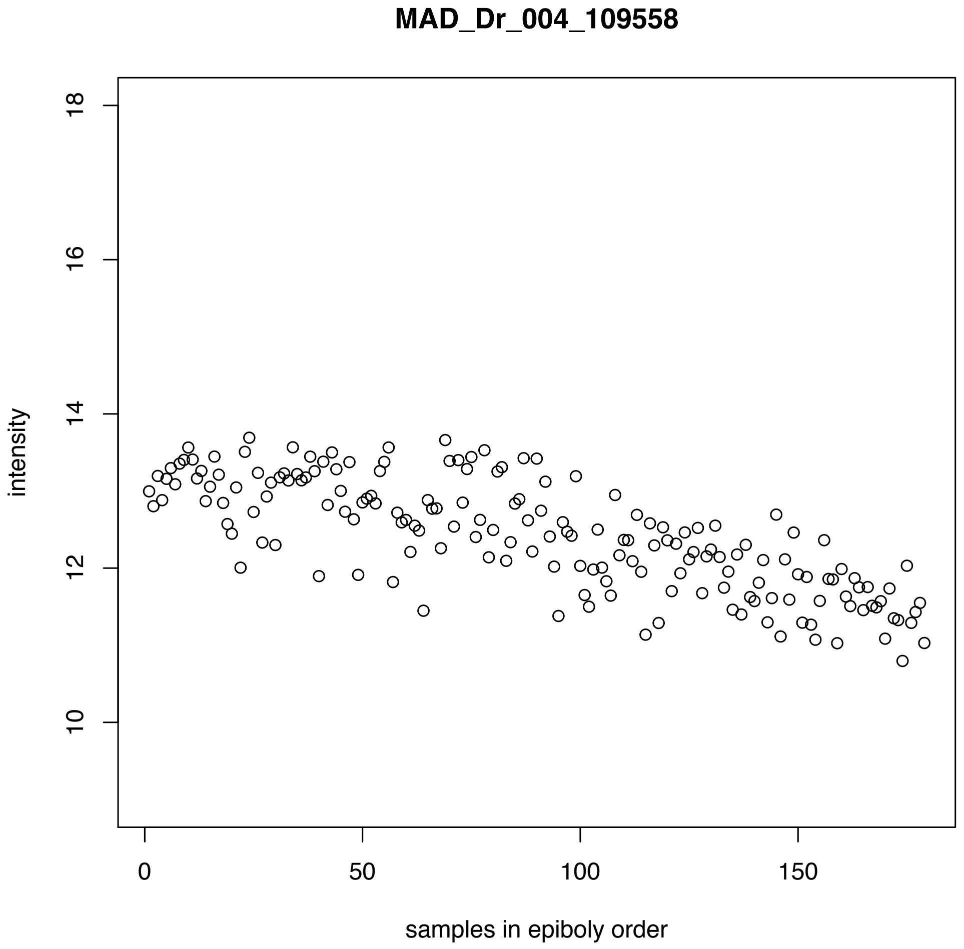
MAD_Dr_004_109558
intensity
10
12
14
16
18
0
50
100
150
samples in epiboly order

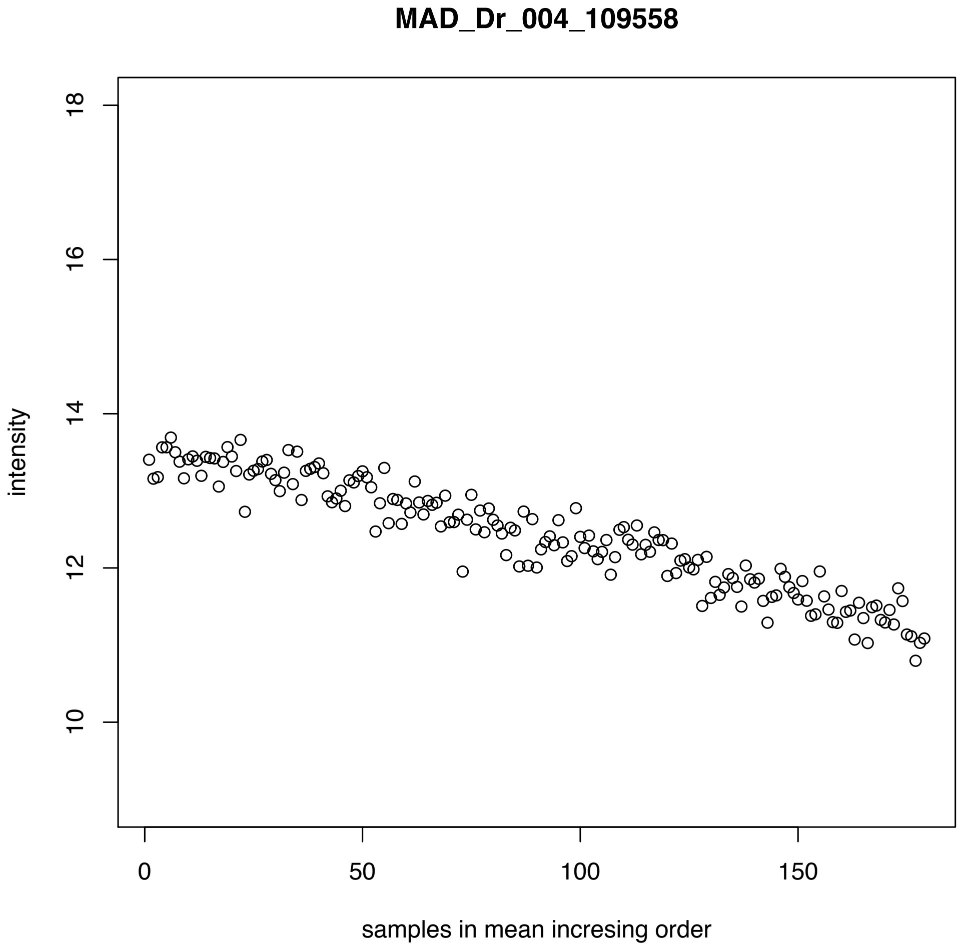
MAD_Dr_004_109558
intensity
10
12
14
16
18
0
50
100
150
samples in mean incresing order

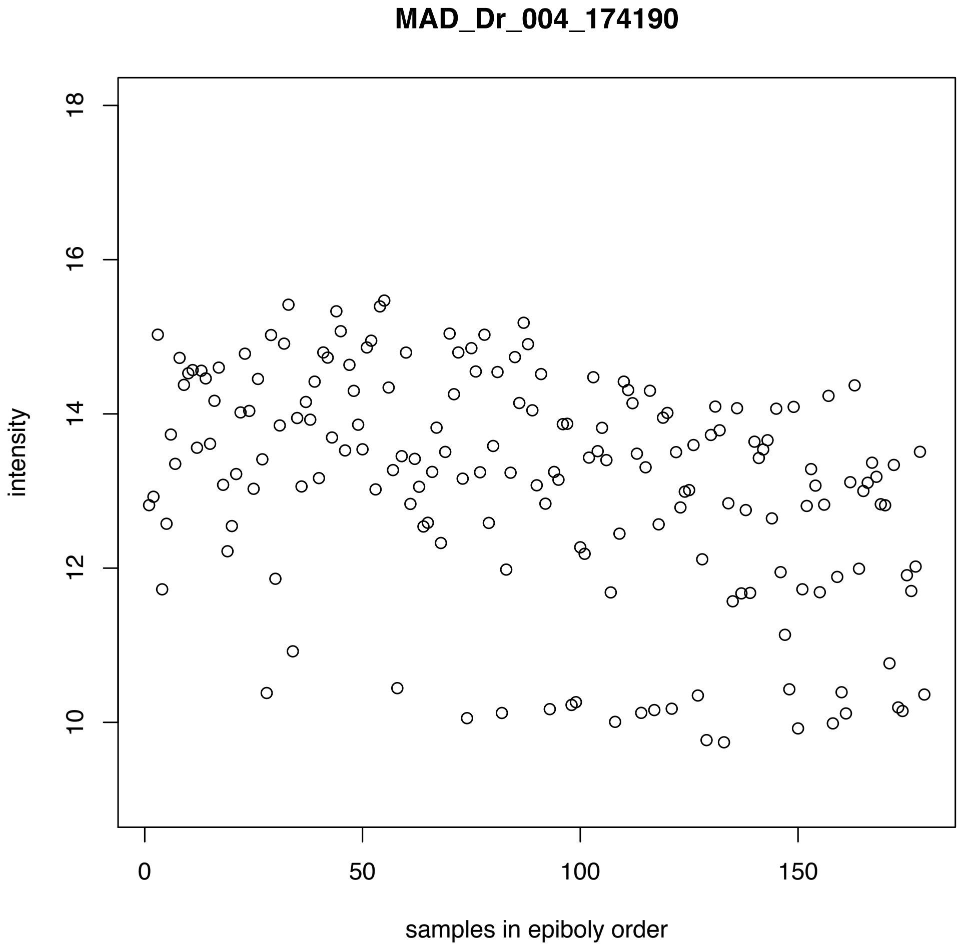
MAD_Dr_004_174190
intensity
samples in epiboly order
0
50
100
150
10
12
14
16
18

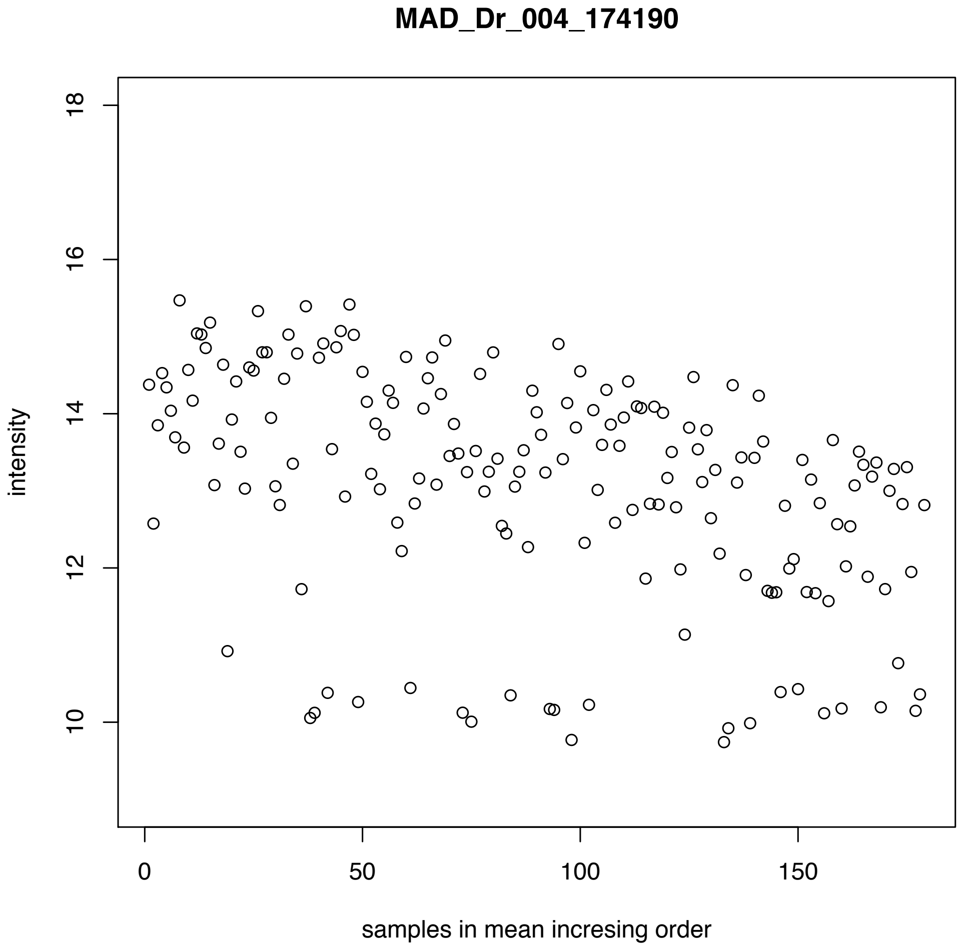
MAD_Dr_004_174190
intensity
samples in mean incresing order
0
50
100
150
10
12
14
16
18

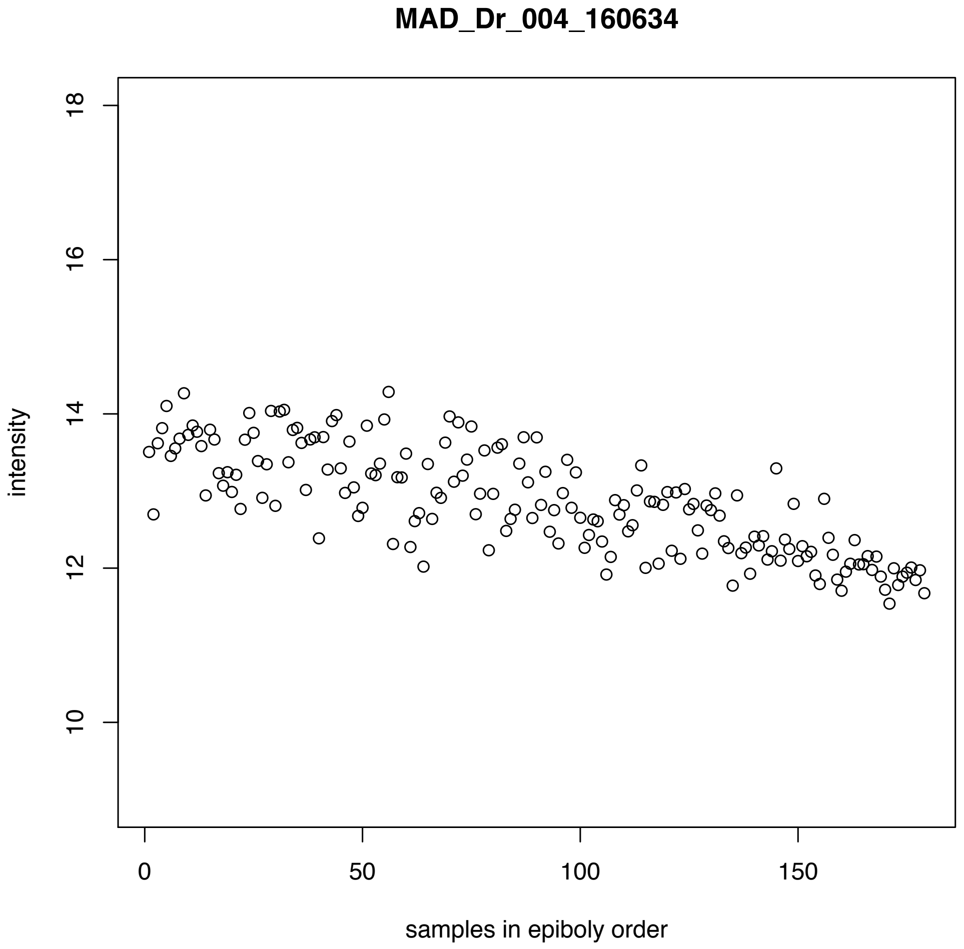
MAD_Dr_004_160634
intensity
10
12
14
16
18
0
50
100
150
samples in epiboly order

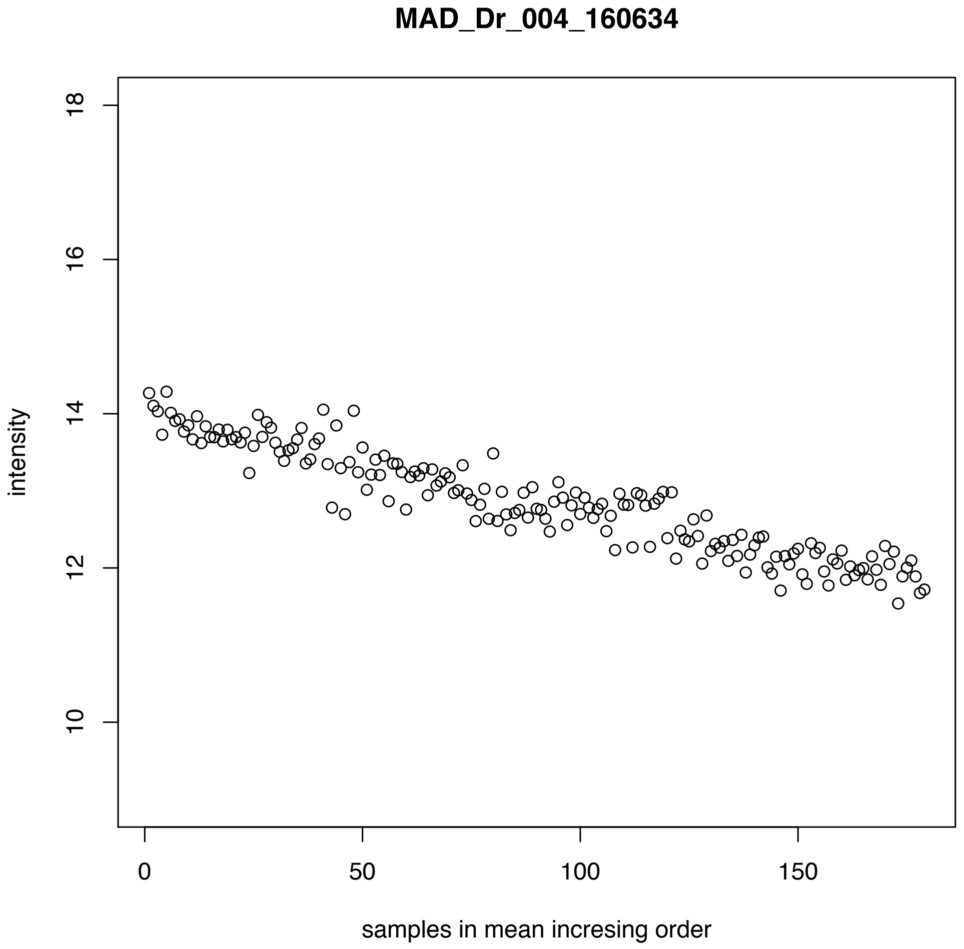
MAD_Dr_004_160634
intensity
10
12
14
16
18
0
50
100
150
samples in mean incresing order

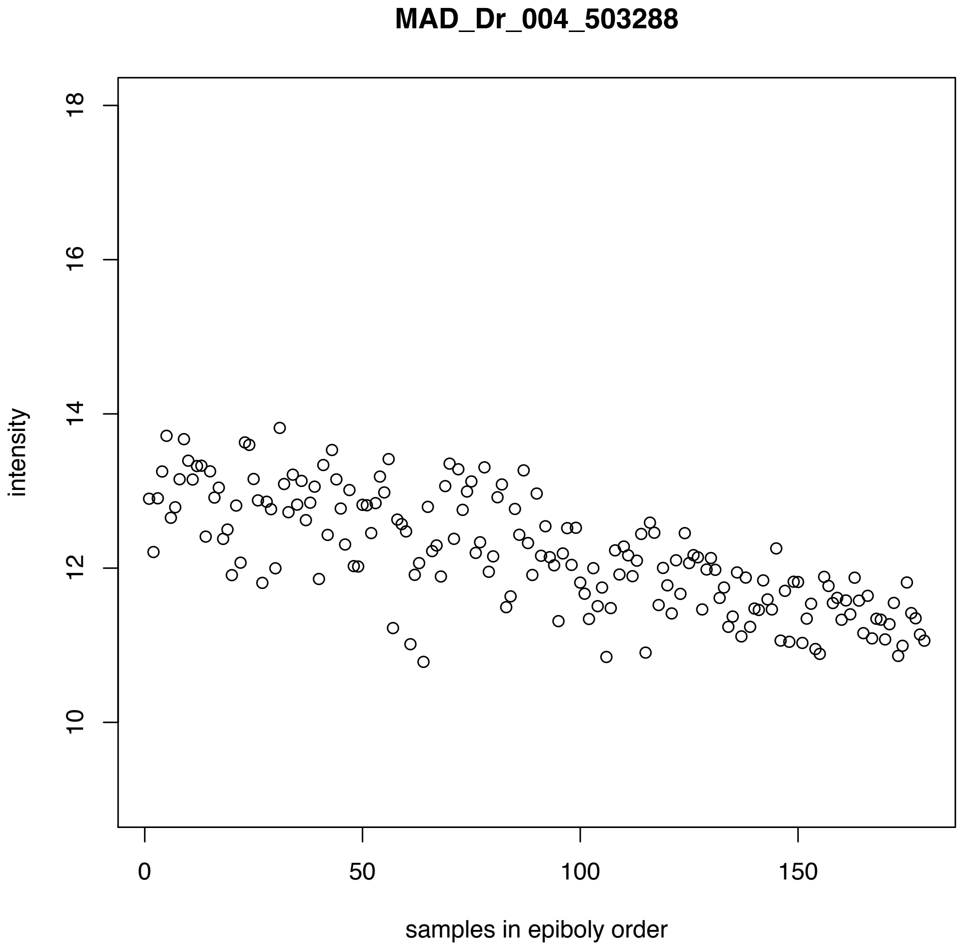

MAD_Dr_004_503288
intensity
10
12
14
16
18
0
50
100
150
samples in epiboly order

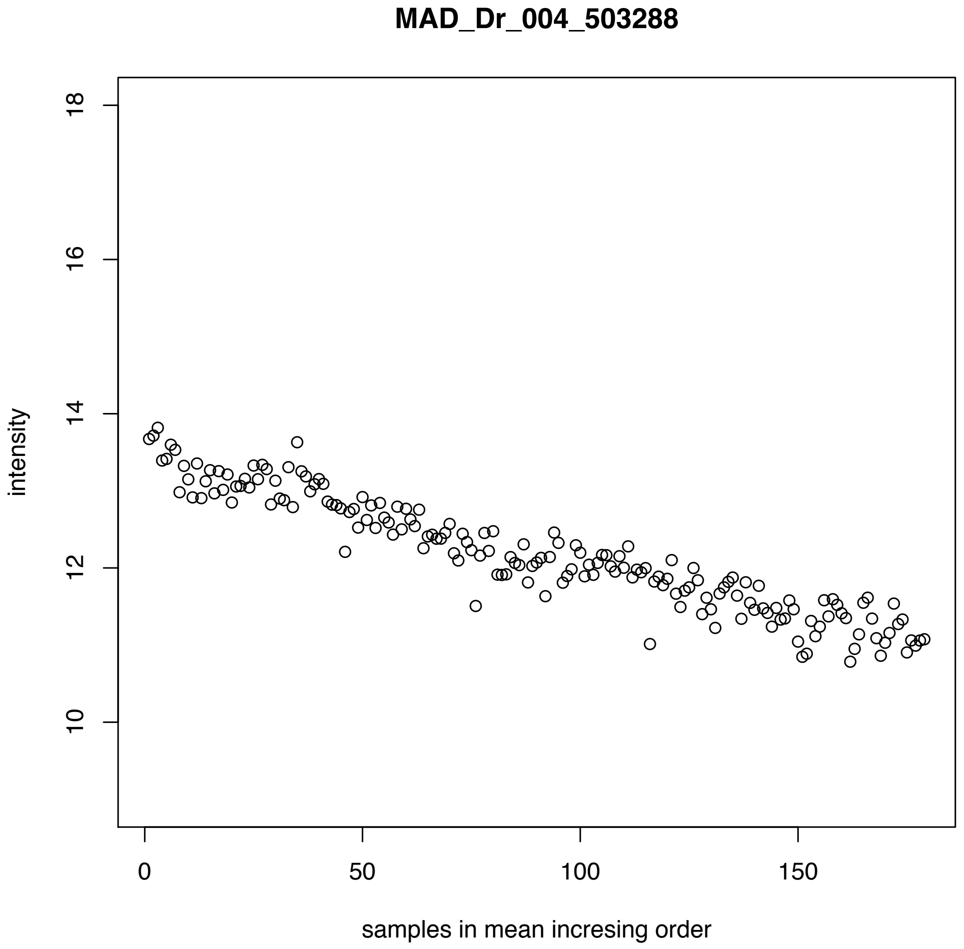

MAD_Dr_004_503288
intensity
10
12
14
16
18
0
50
100
150
samples in mean incresing order

MAD_Dr_004_157691

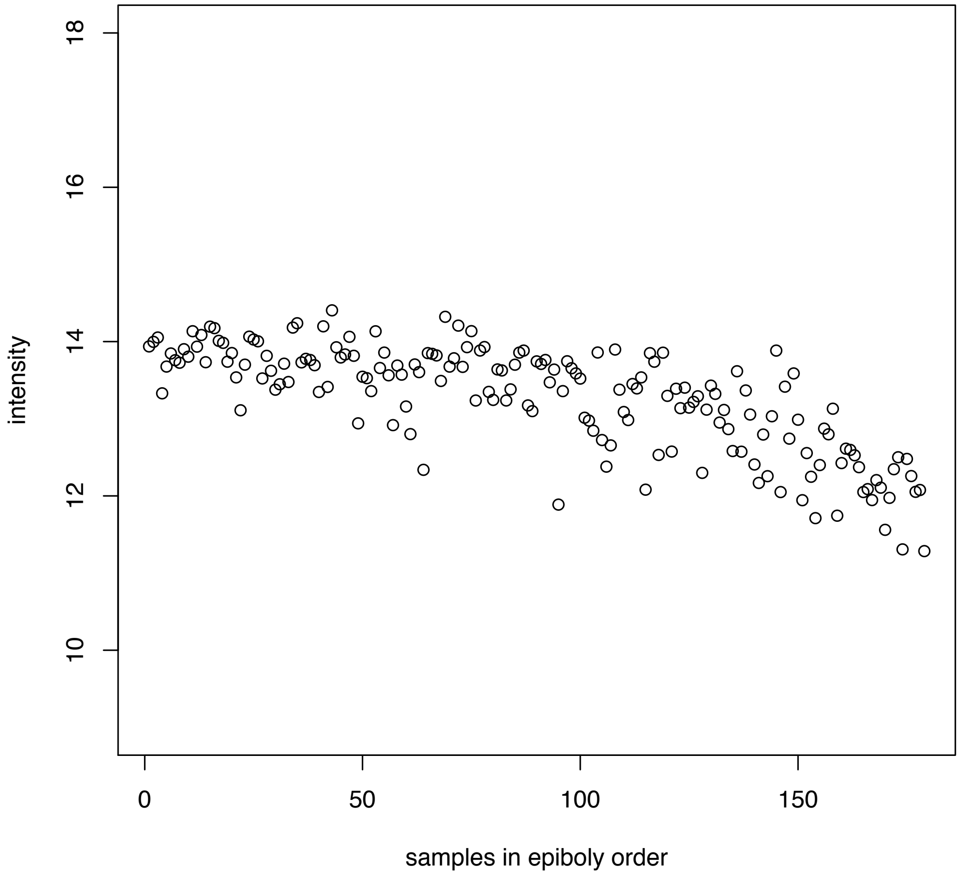

MAD_Dr_004_157691

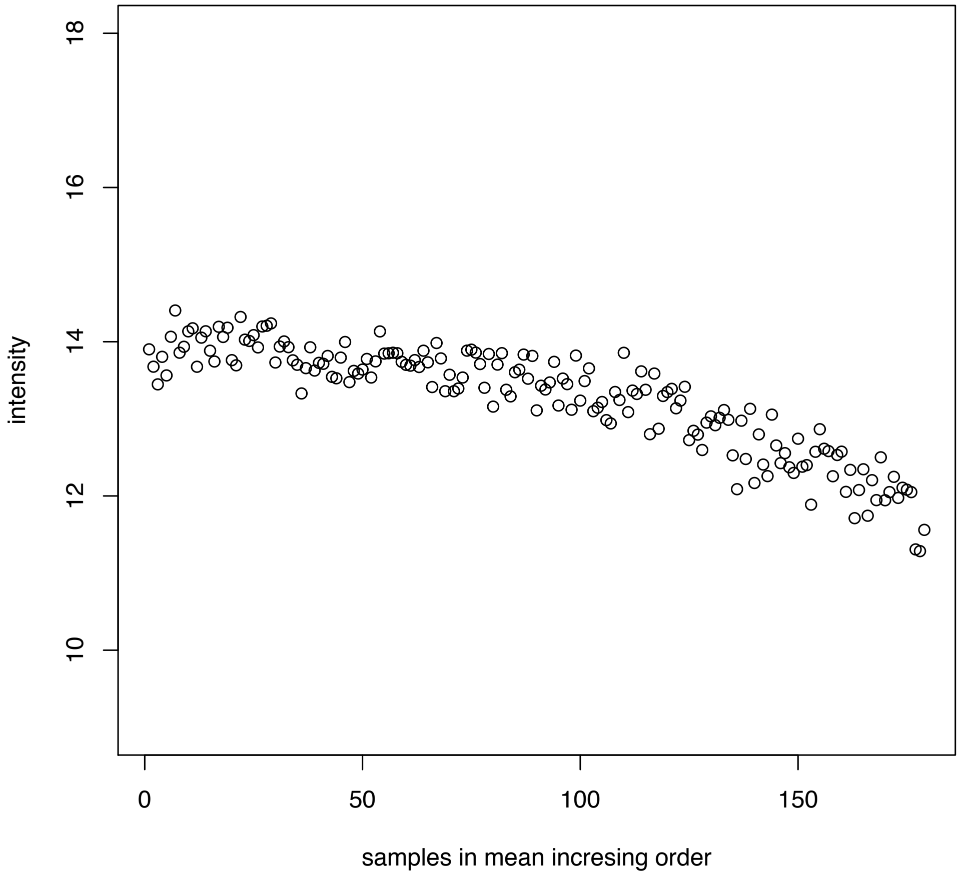

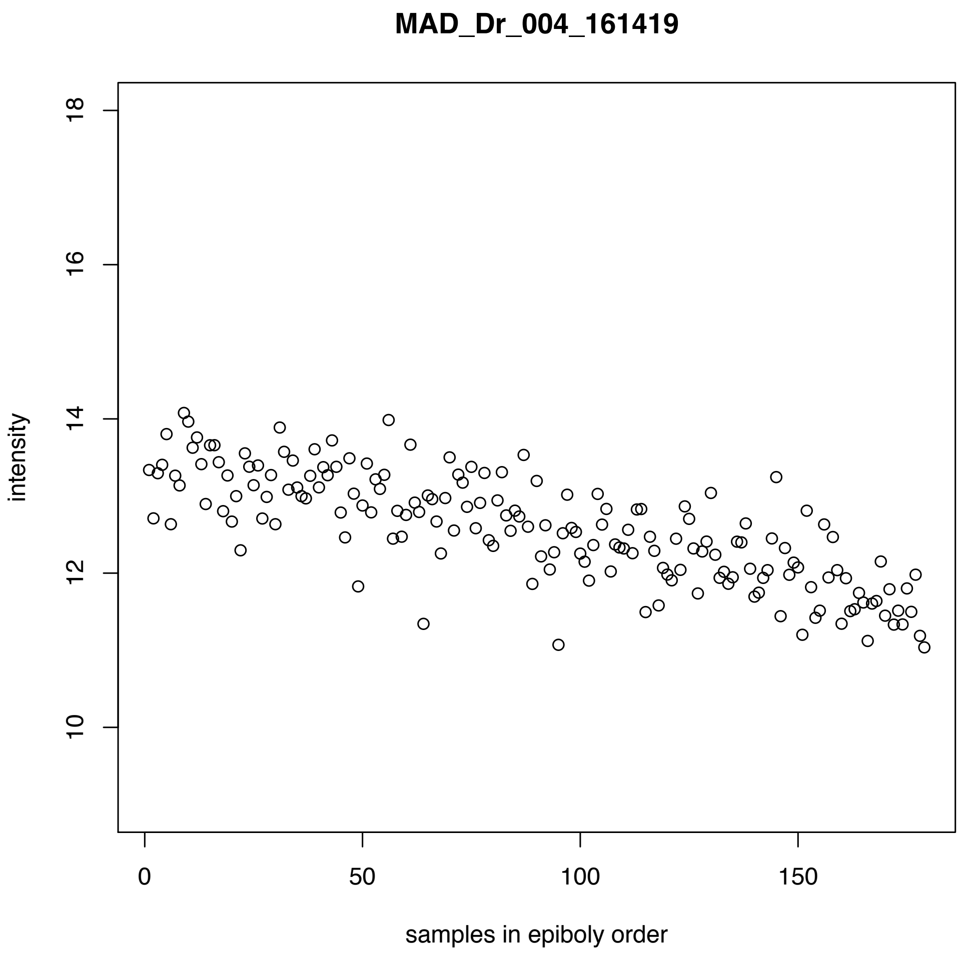
MAD_Dr_004_161419
intensity
samples in epiboly order
18
16
14
12
10
0
50
100
150

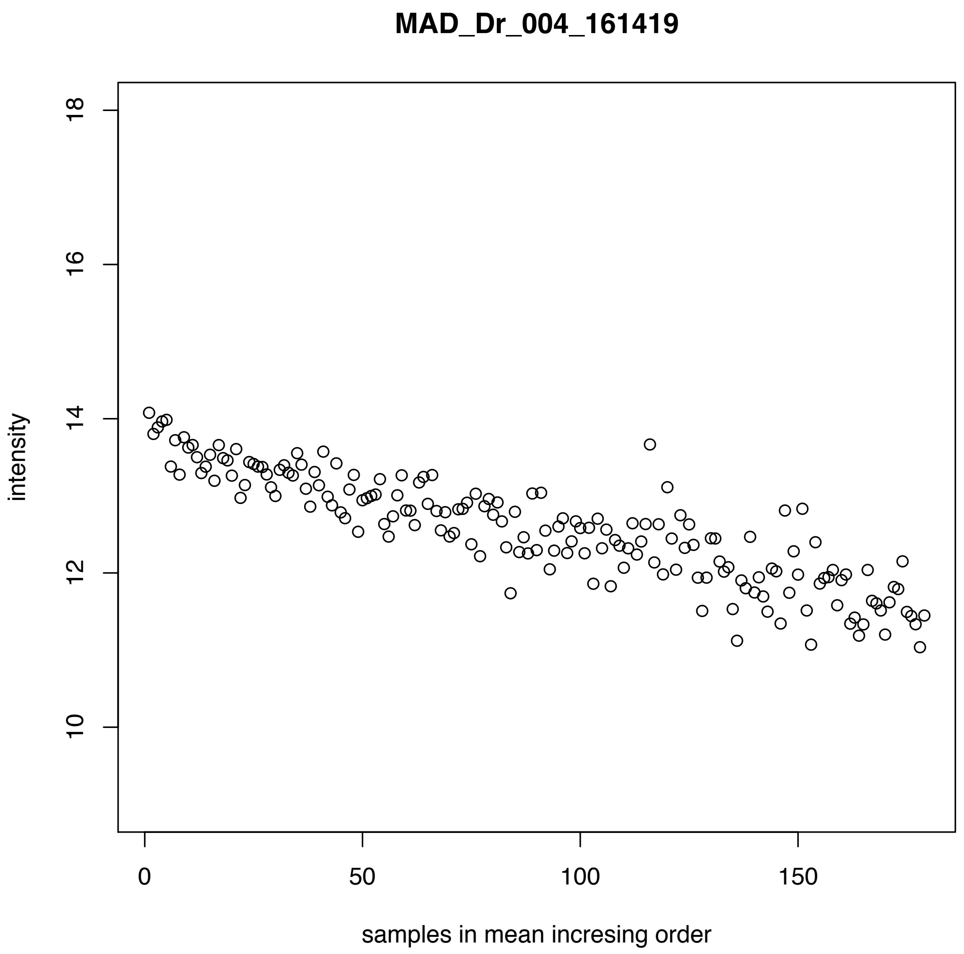
MAD_Dr_004_161419
intensity
samples in mean incresing order
18
16
14
12
10
0
50
100
150

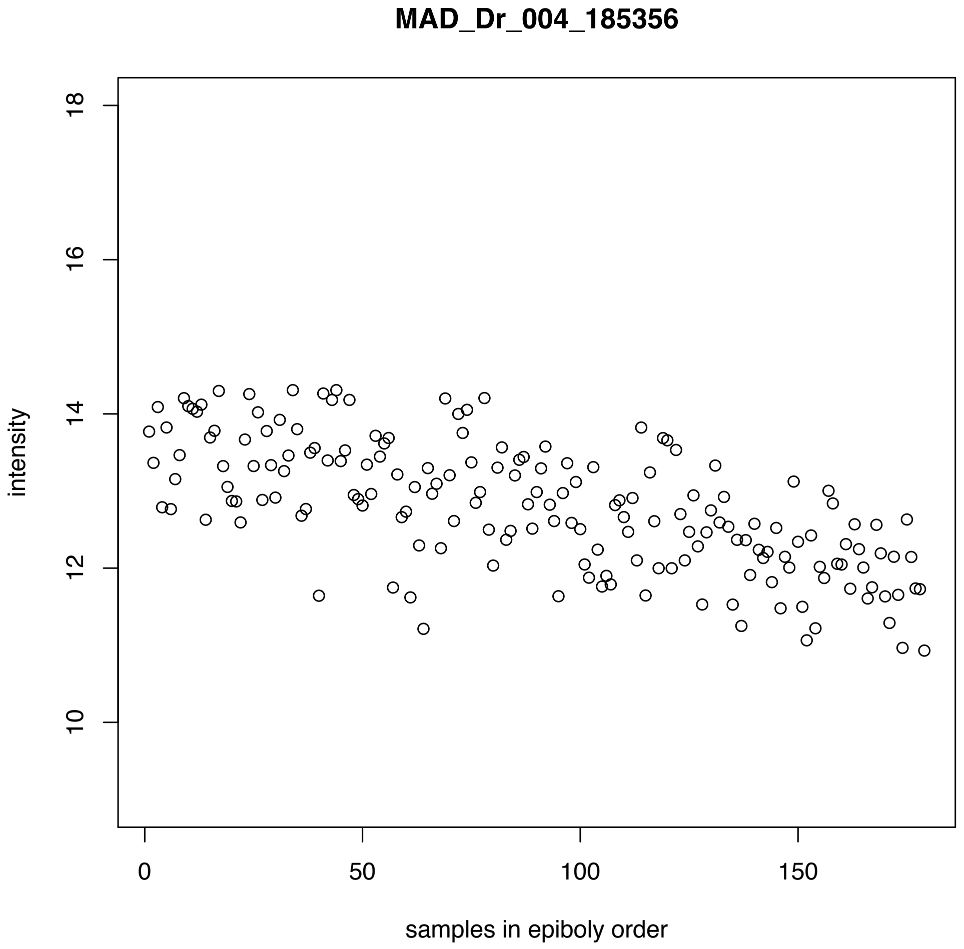
MAD_Dr_004_185356
intensity
10
12
14
16
18
0
50
100
150
samples in epiboly order

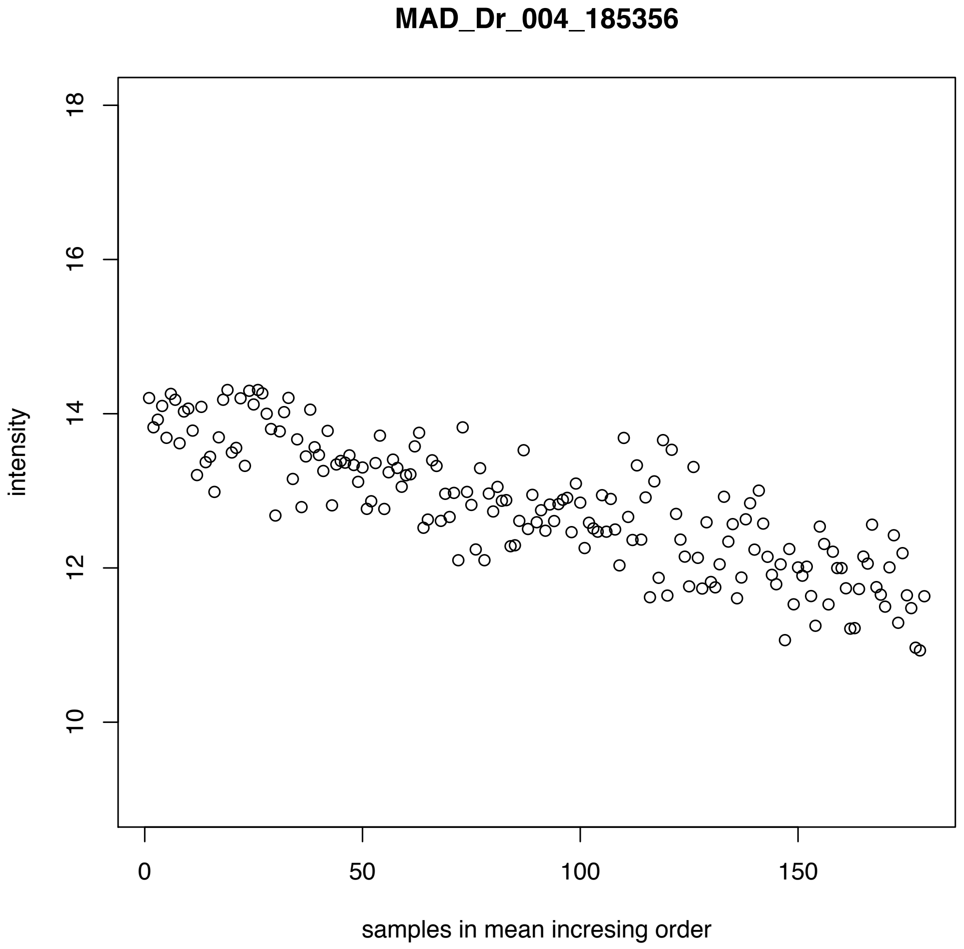
MAD_Dr_004_185356
intensity
10
12
14
16
18
0
50
100
150
samples in mean incresing order

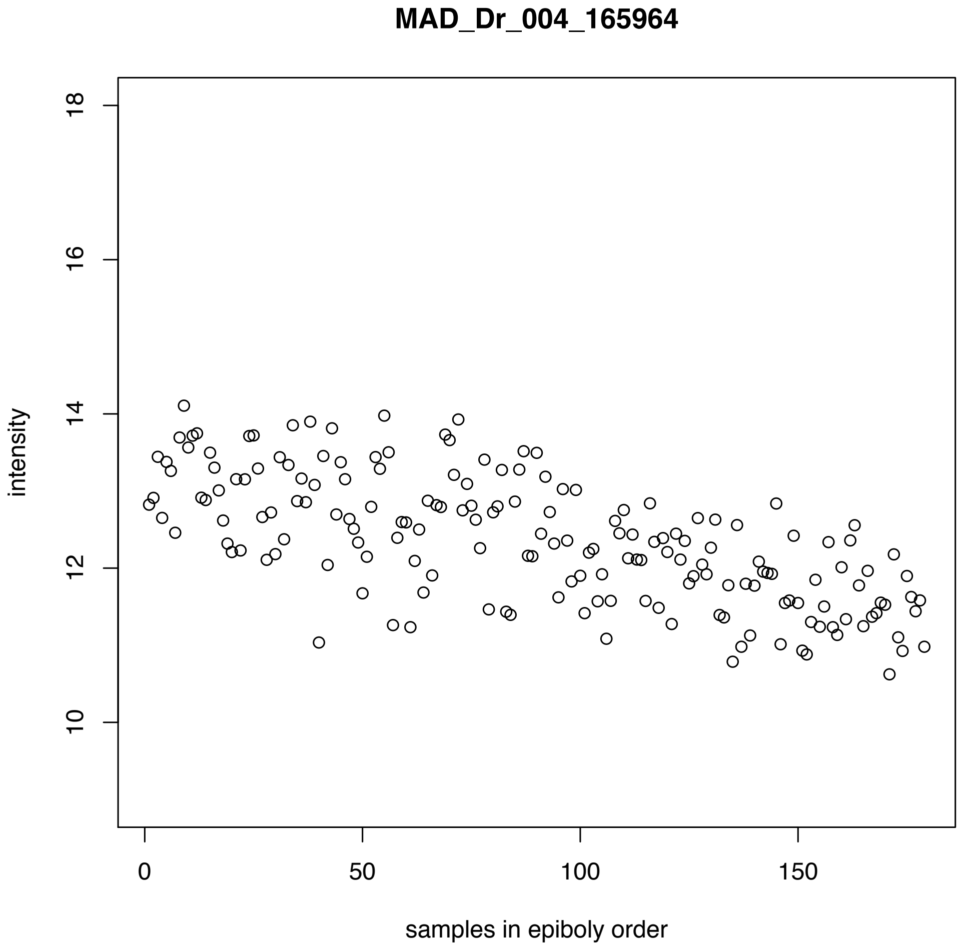
MAD_Dr_004_165964
intensity
10
12
14
16
18
0
50
100
150
samples in epiboly order

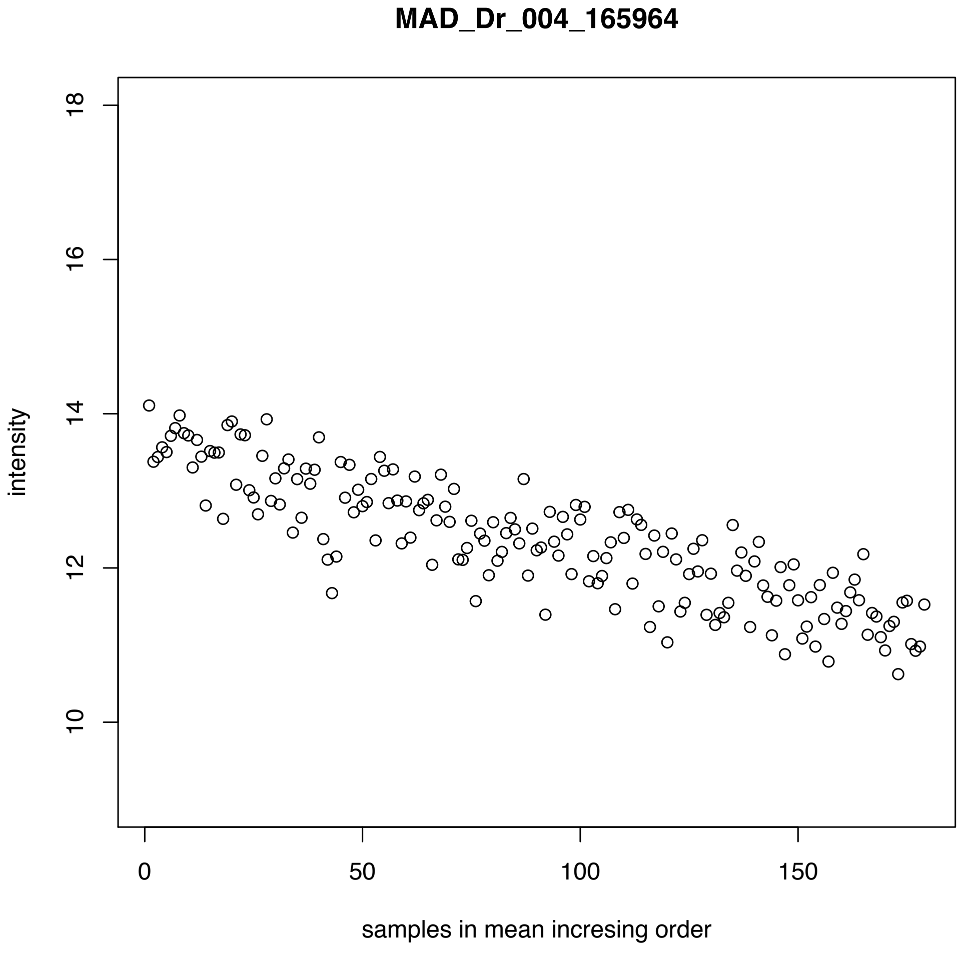
MAD_Dr_004_165964
intensity
10
12
14
16
18
0
50
100
150
samples in mean incresing order

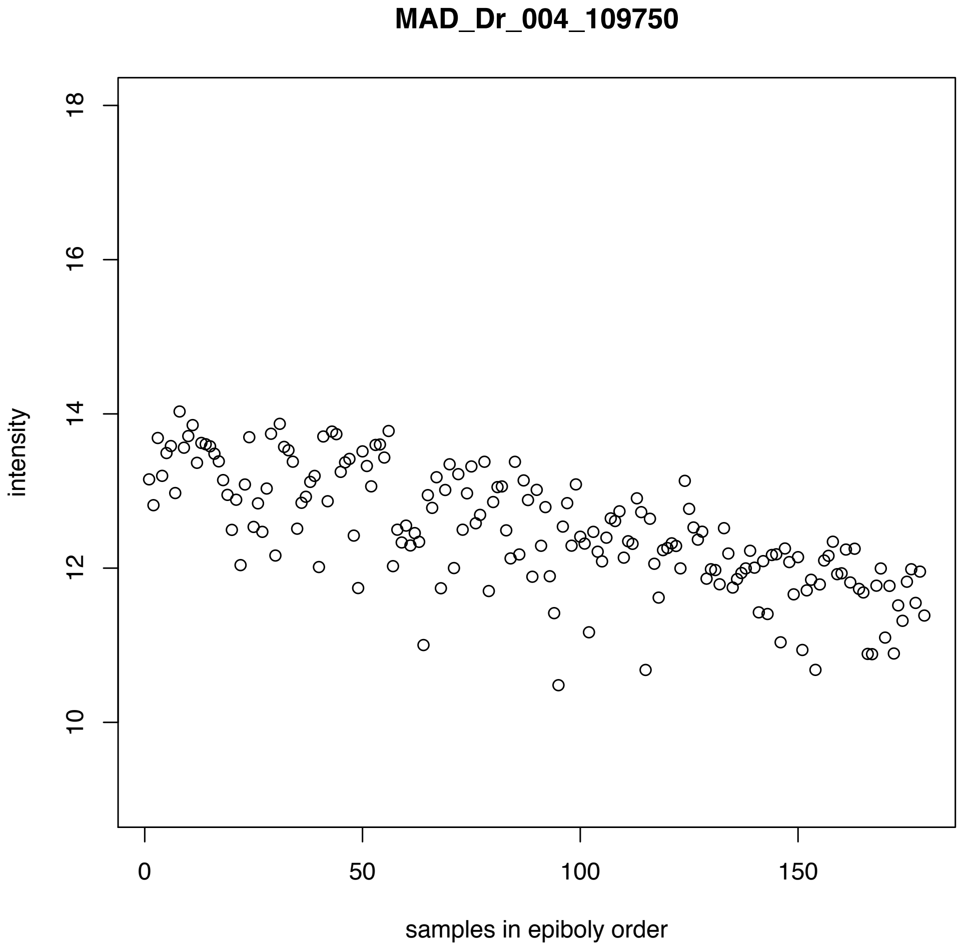
MAD_Dr_004_109750
intensity
samples in epiboly order
10
12
14
16
18
0
50
100
150

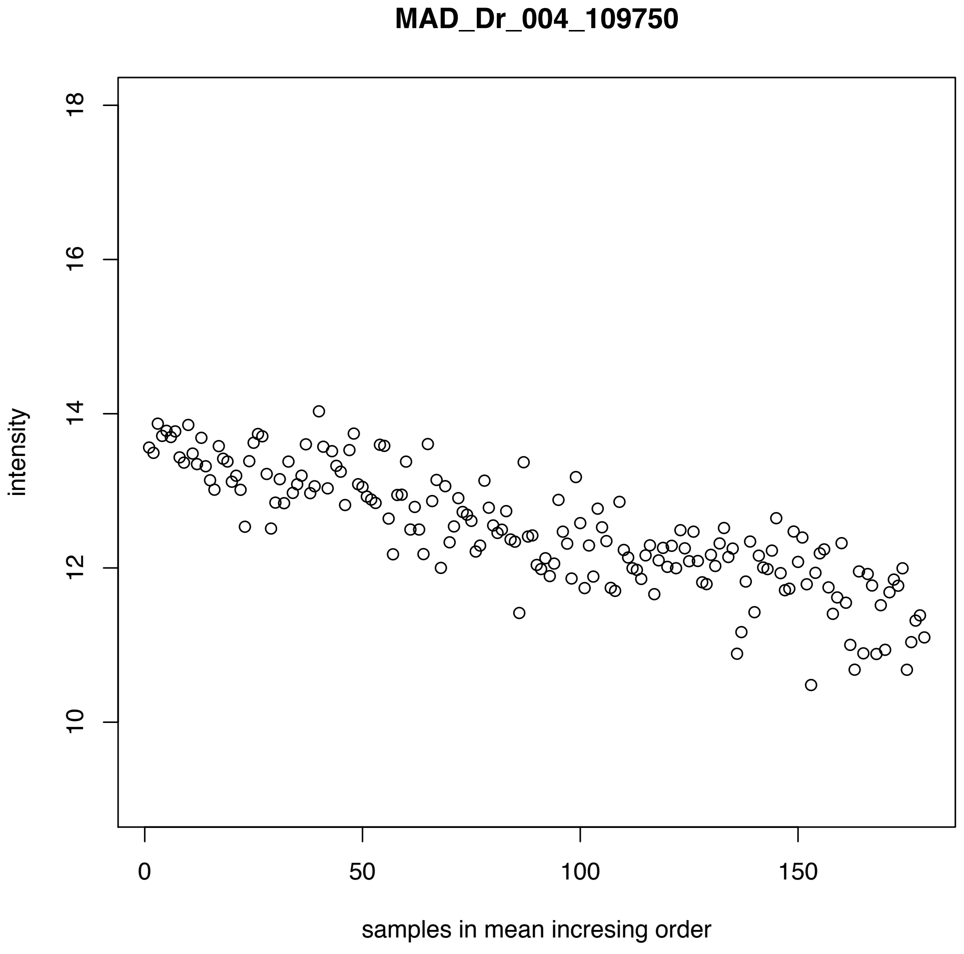
MAD_Dr_004_109750
intensity
samples in mean incresing order
10
12
14
16
18
0
50
100
150

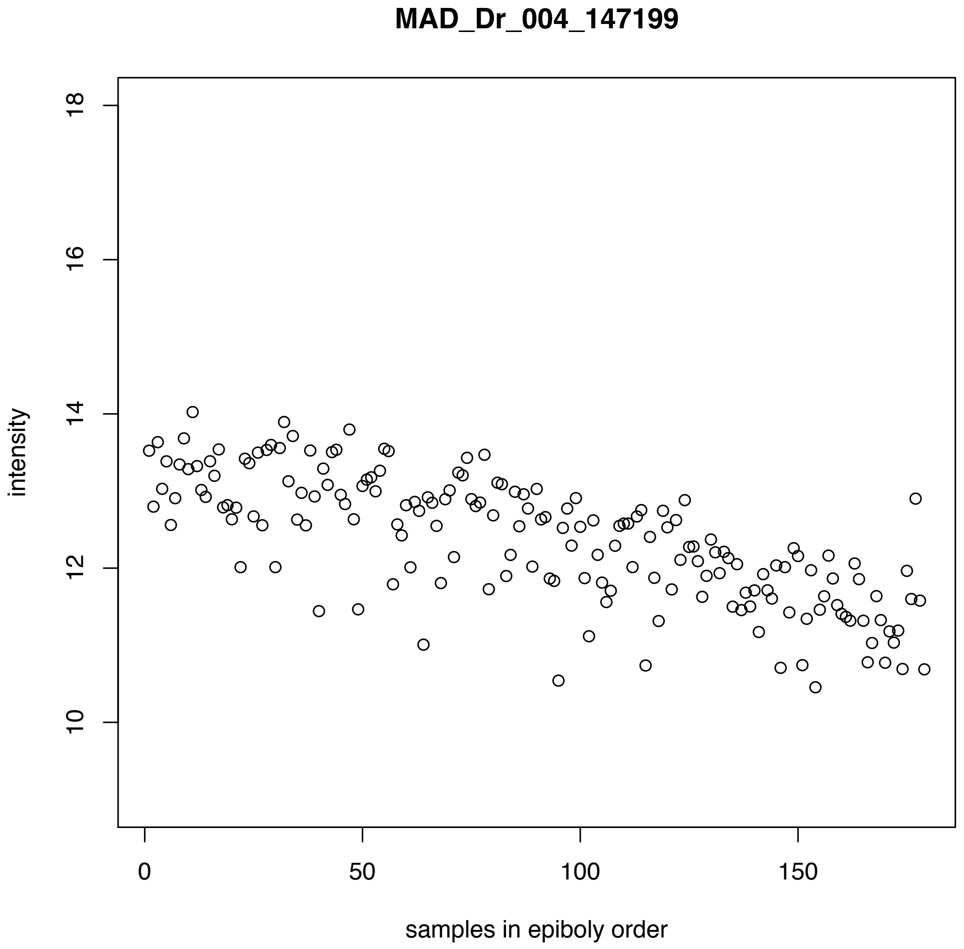
MAD_Dr_004_147199
intensity
10
12
14
16
18
0
50
100
150
samples in epiboly order

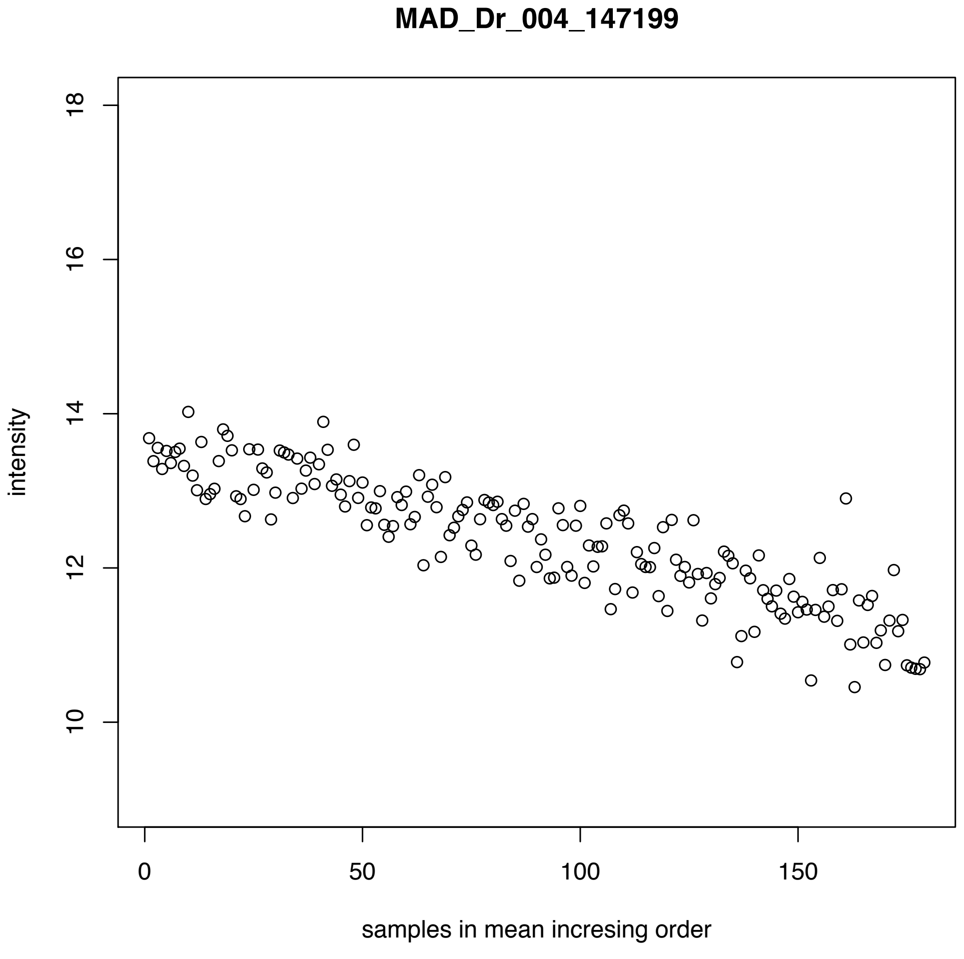
MAD_Dr_004_147199
intensity
10
12
14
16
18
0
50
100
150
samples in mean incresing order

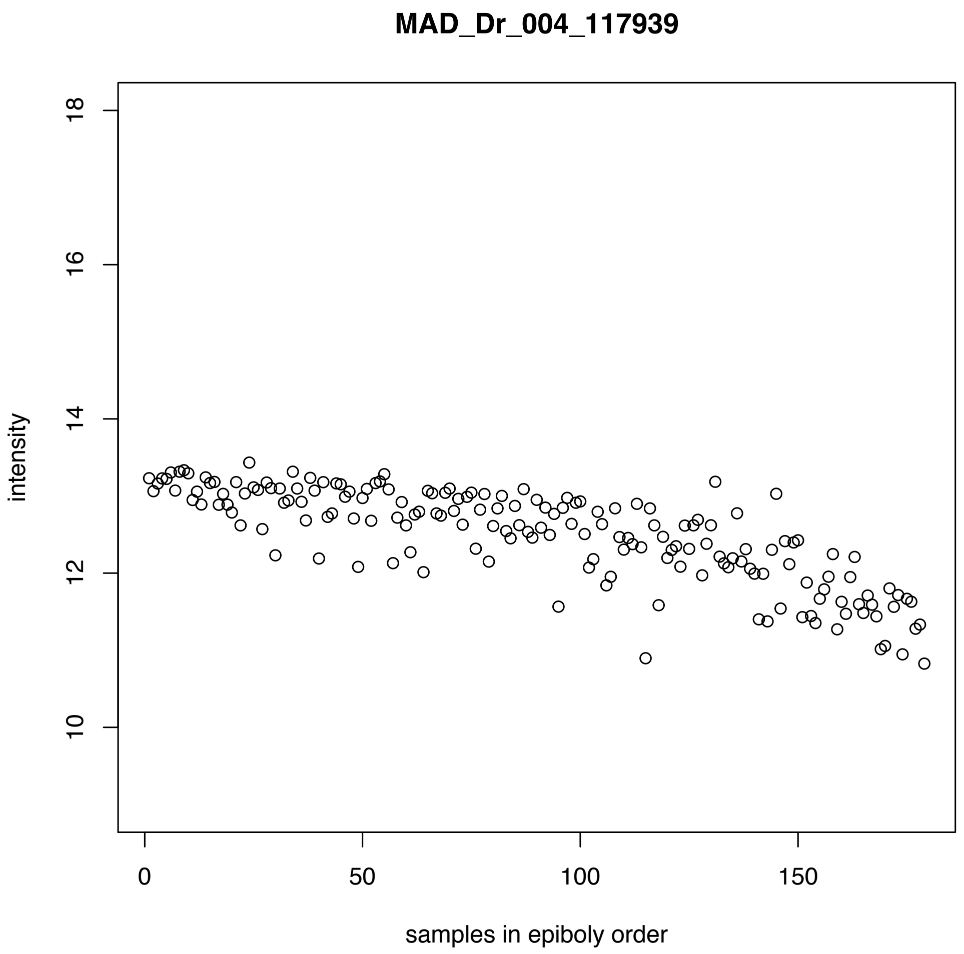
MAD_Dr_004_117939
intensity
10
12
14
16
18
0
50
100
150
samples in epiboly order

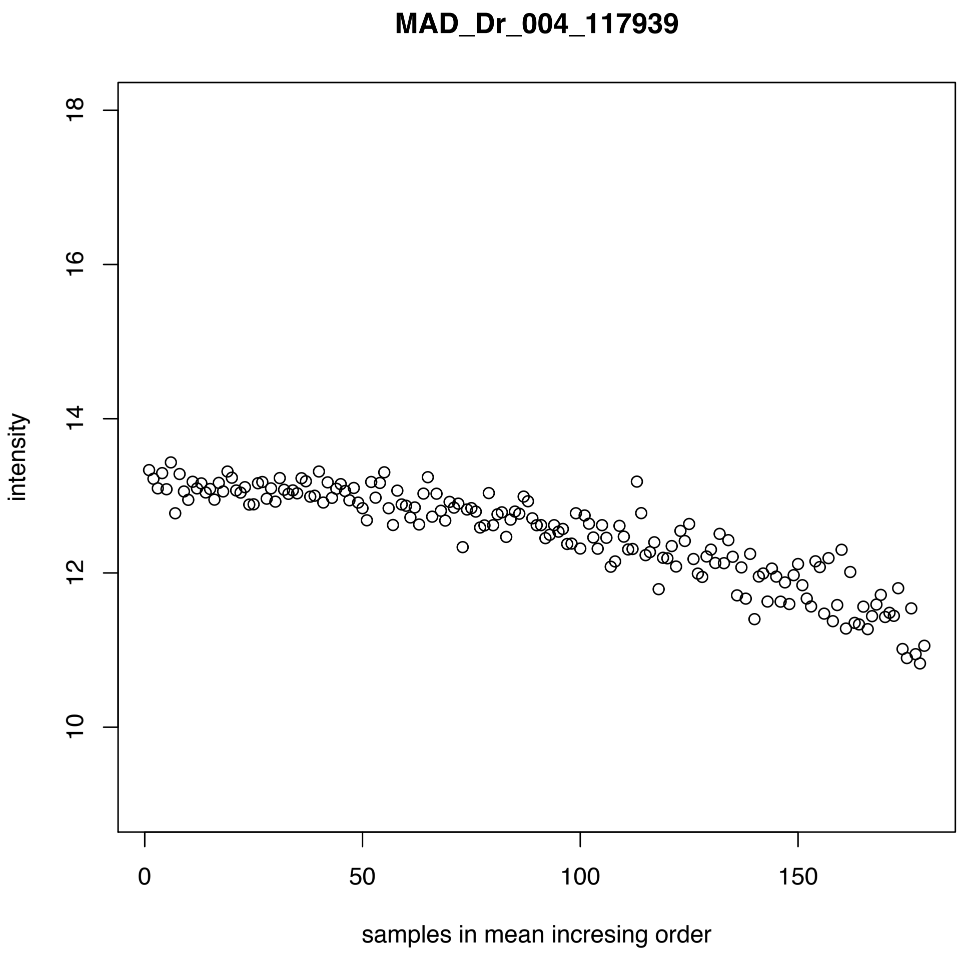
MAD_Dr_004_117939
intensity
10
12
14
16
18
0
50
100
150
samples in mean incresing order

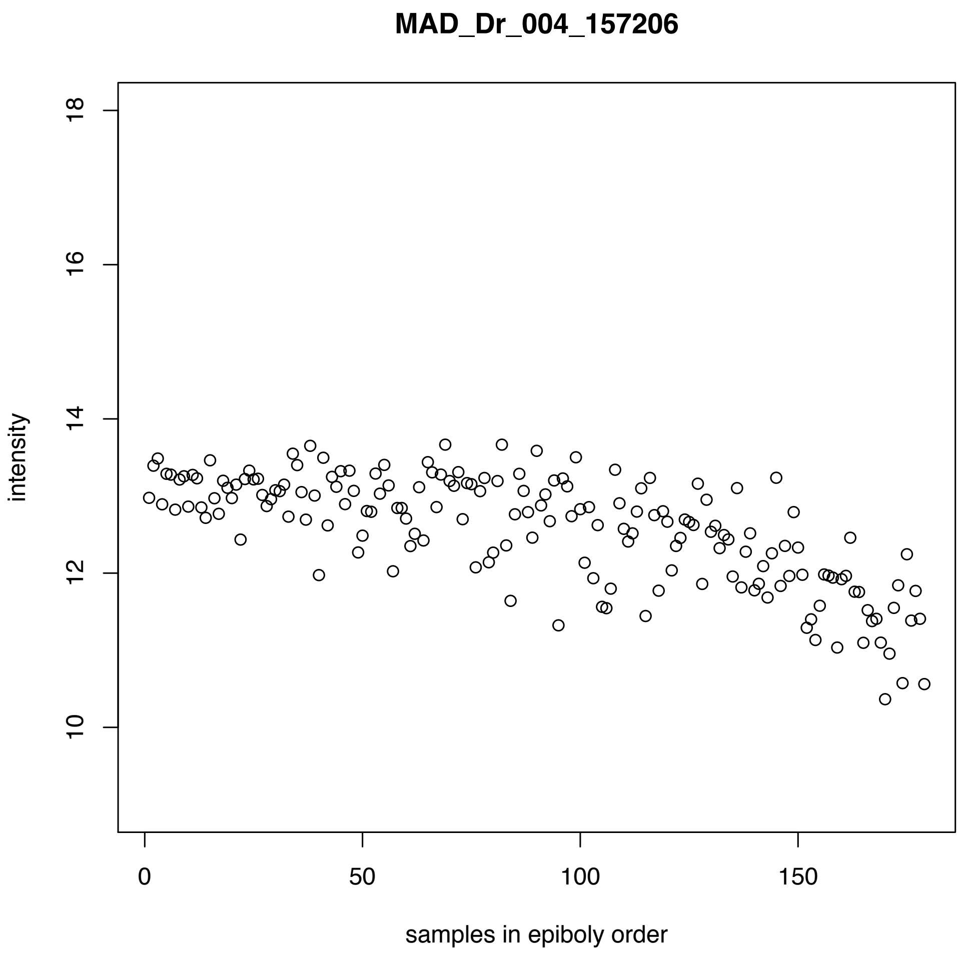
MAD_Dr_004_157206
intensity
10
12
14
16
18
0
50
100
150
samples in epiboly order

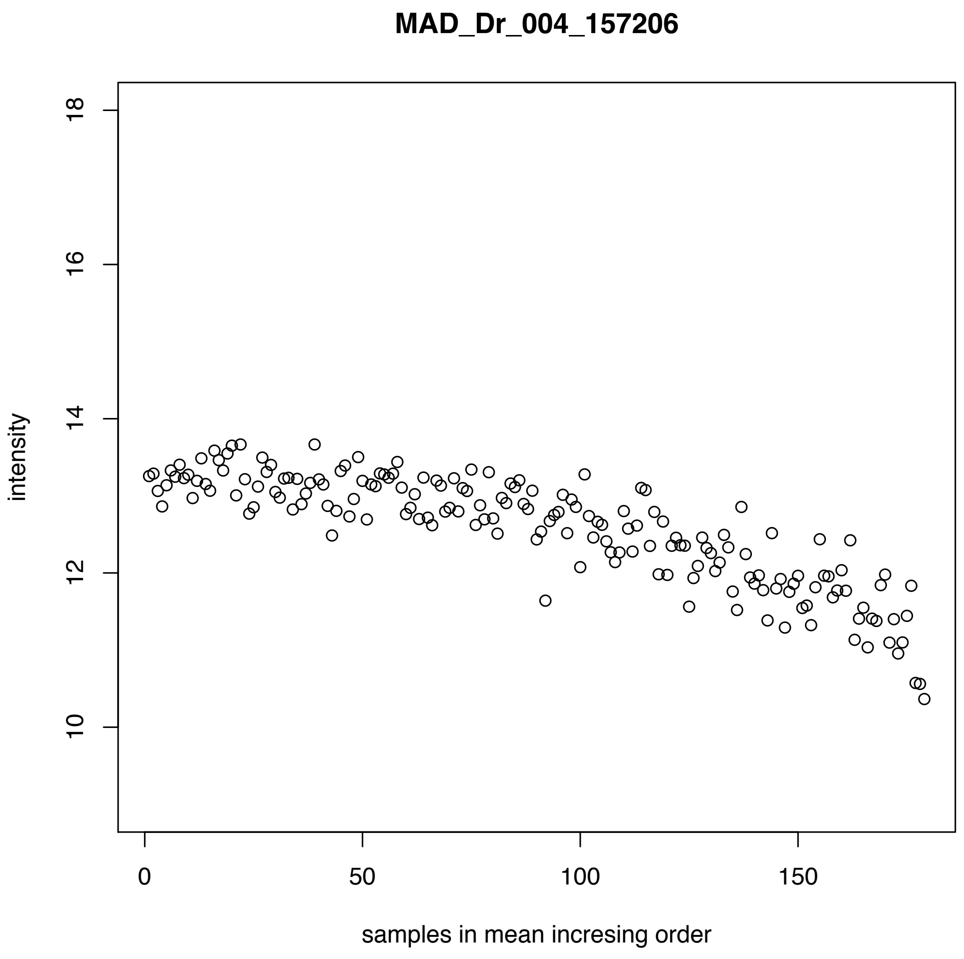
MAD_Dr_004_157206
intensity
10
12
14
16
18
0
50
100
150
samples in mean incresing order

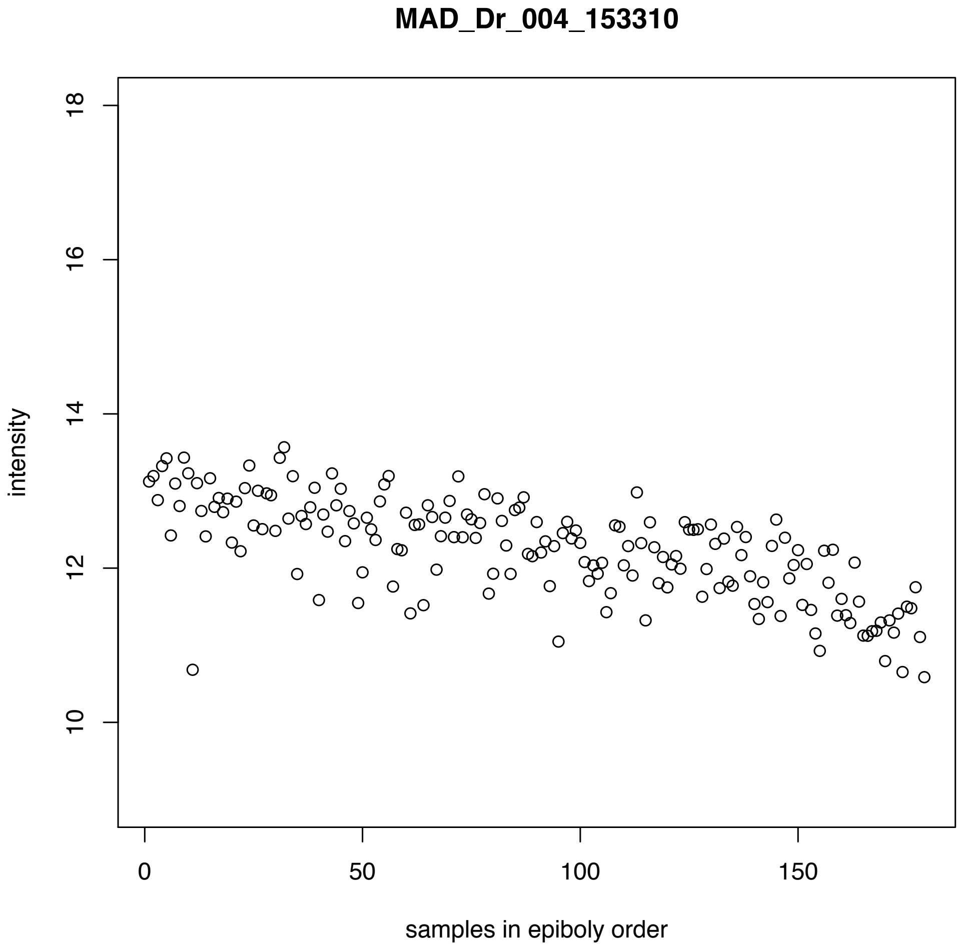
MAD_Dr_004_153310
intensity
samples in epiboly order
10
12
14
16
18
0
50
100
150

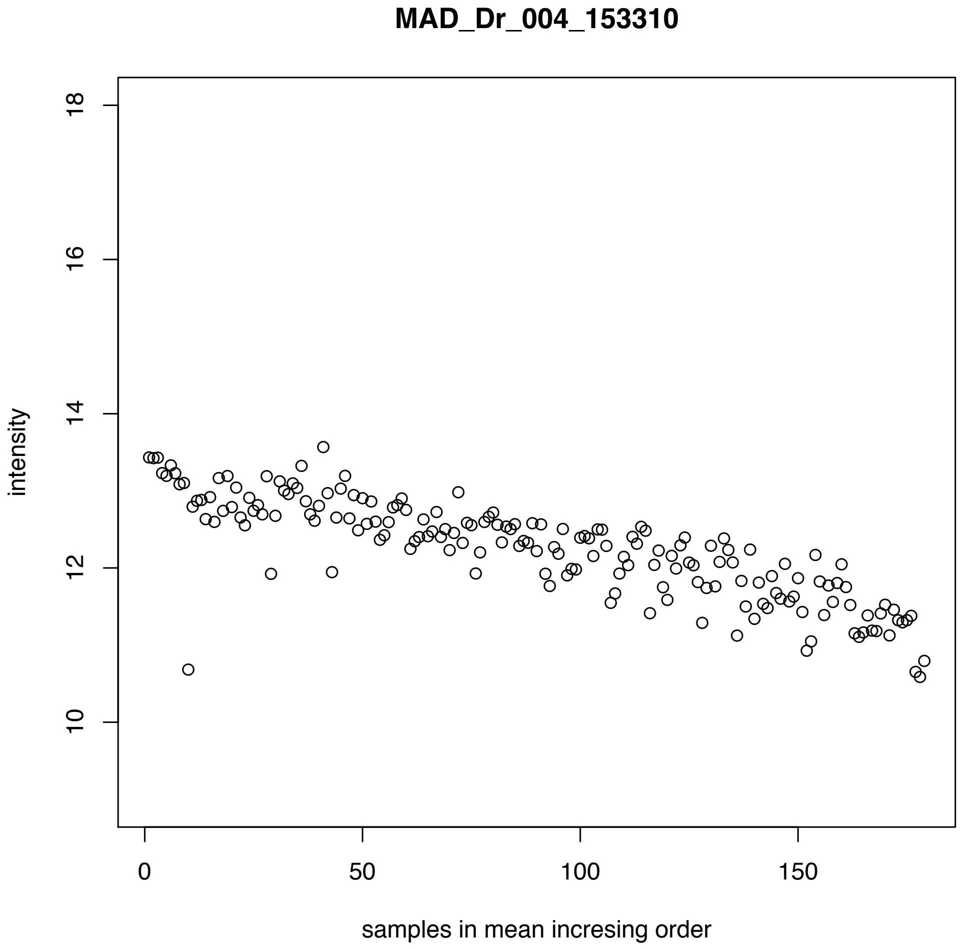
MAD_Dr_004_153310
intensity
samples in mean incresing order
10
12
14
16
18
0
50
100
150

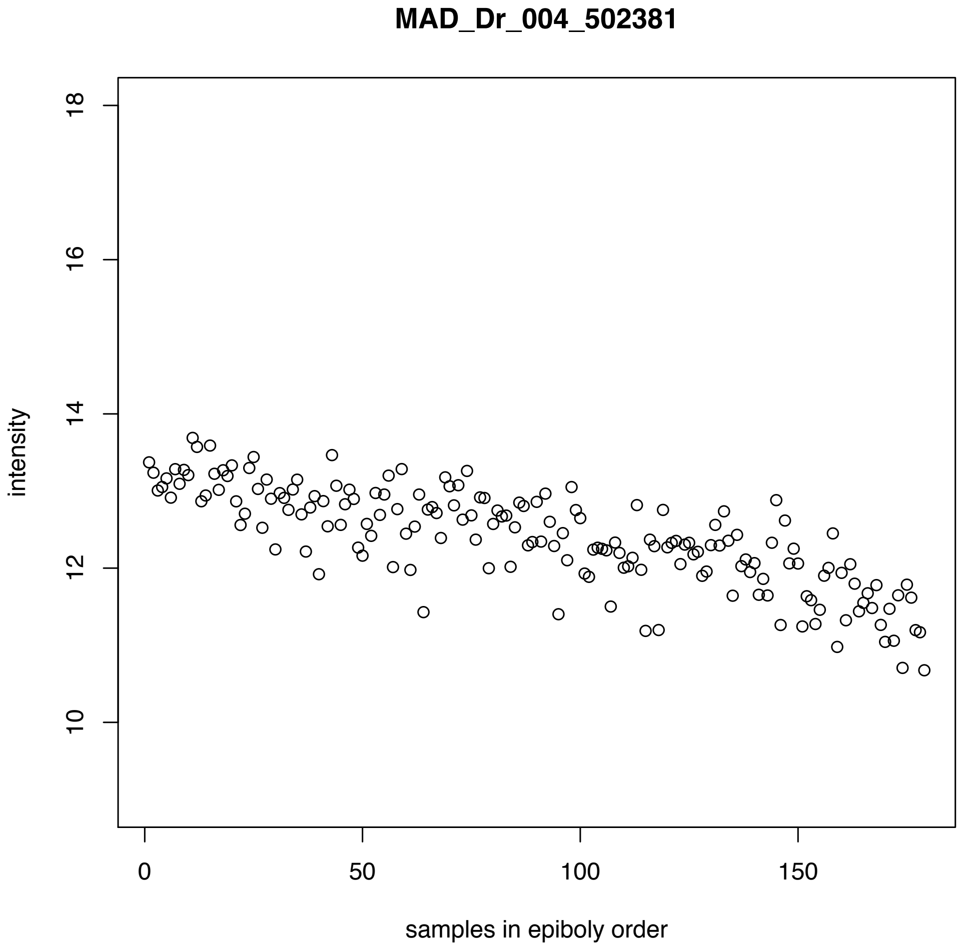
MAD_Dr_004_502381
intensity
samples in epiboly order
10
12
14
16
18
0
50
100
150

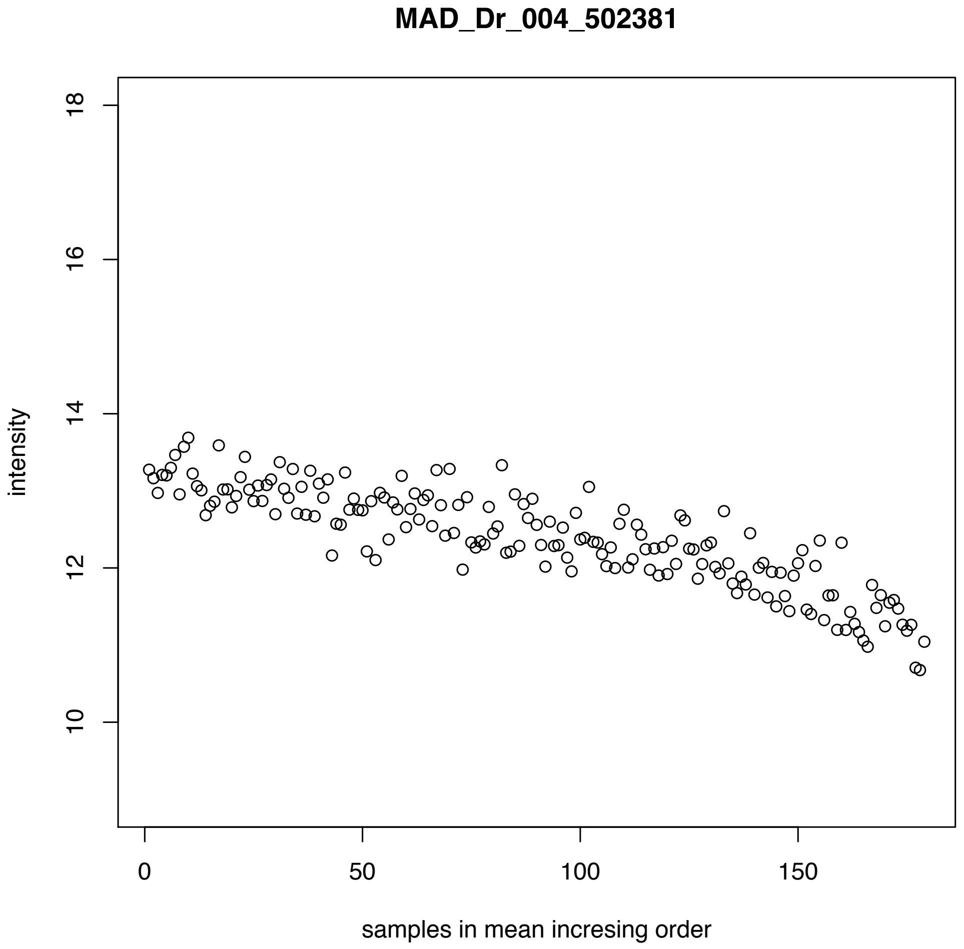
MAD_Dr_004_502381
intensity
samples in mean incresing order
10
12
14
16
18
0
50
100
150

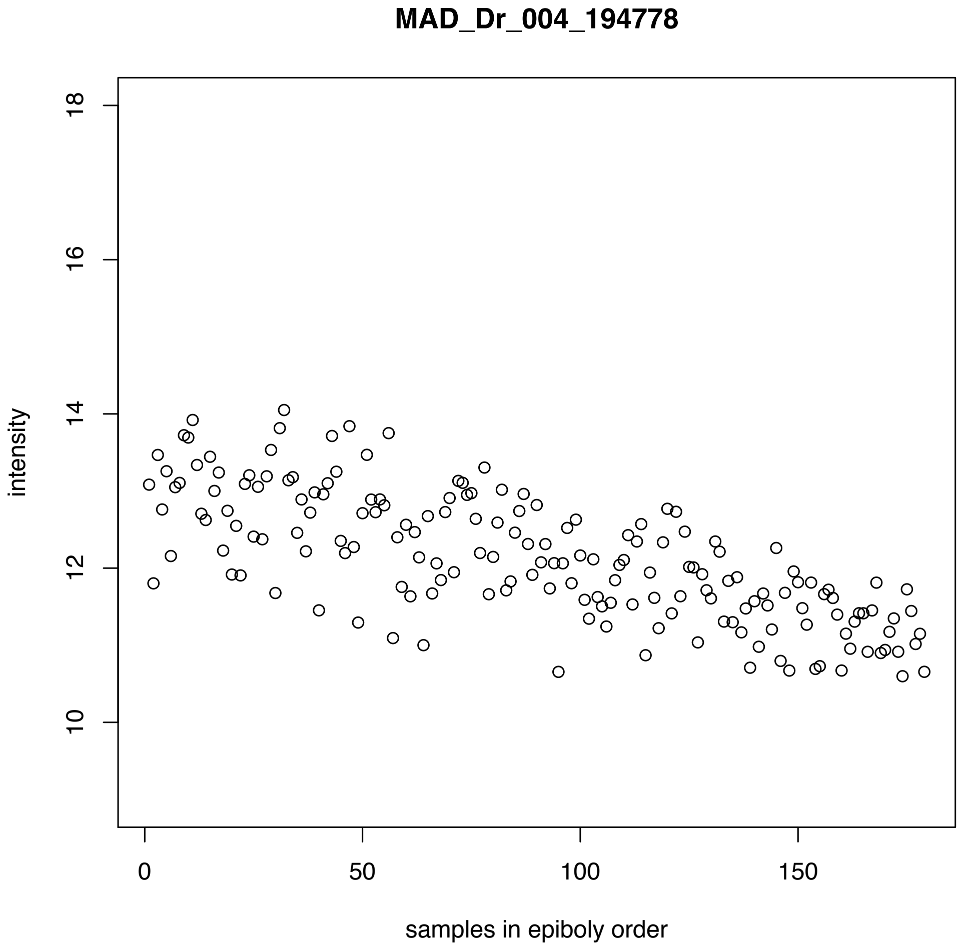
MAD_Dr_004_194778
intensity
10
12
14
16
18
0
50
100
150
samples in epiboly order

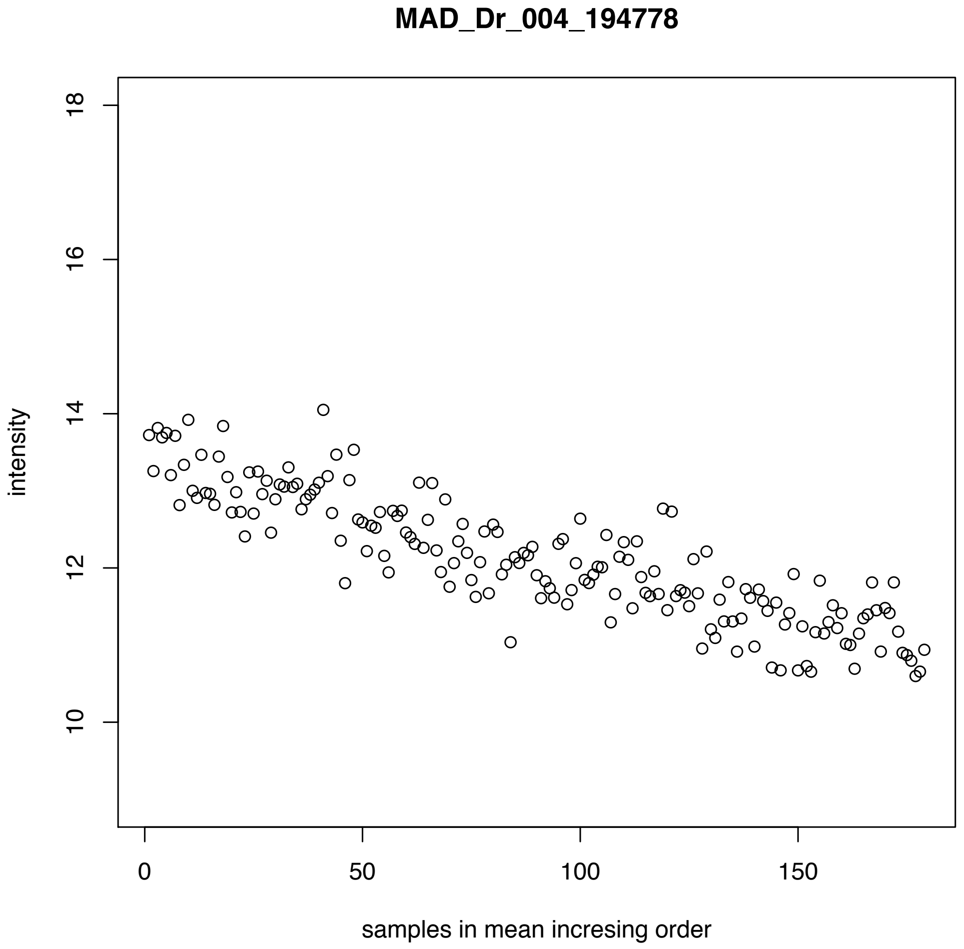
MAD_Dr_004_194778
intensity
10
12
14
16
18
0
50
100
150
samples in mean incresing order

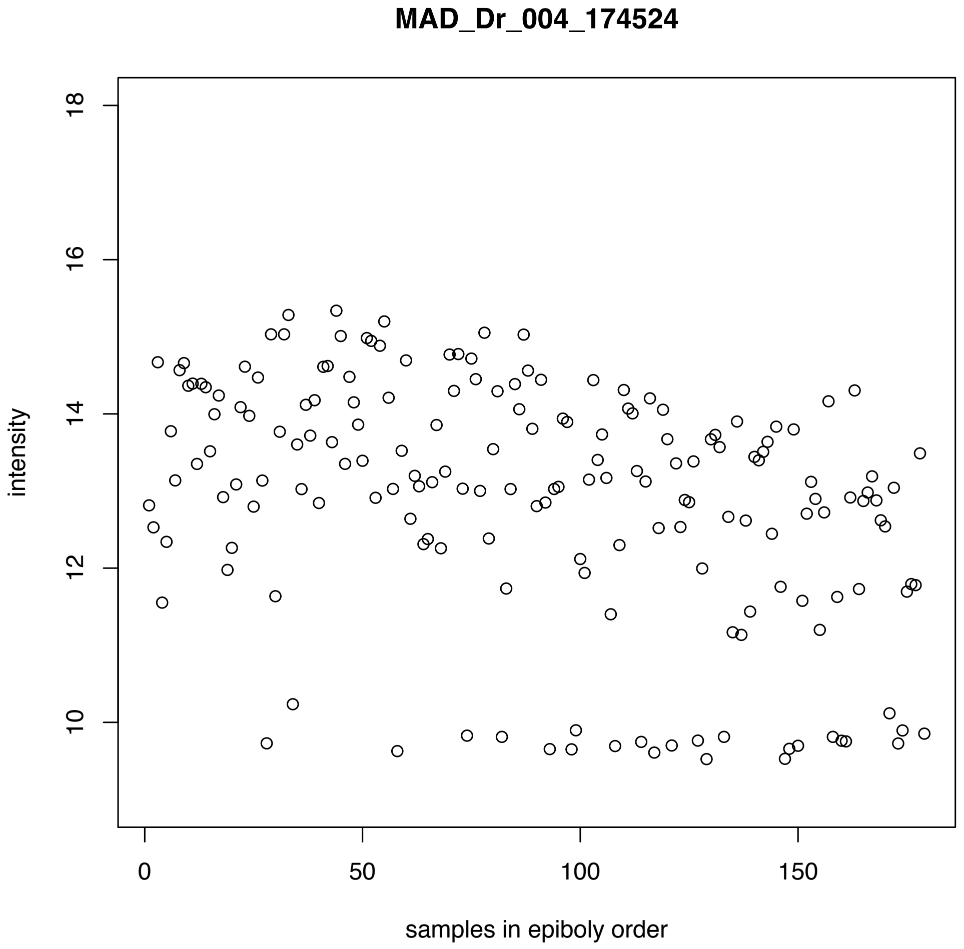
MAD_Dr_004_174524
intensity
samples in epiboly order
0
50
100
150
10
12
14
16
18

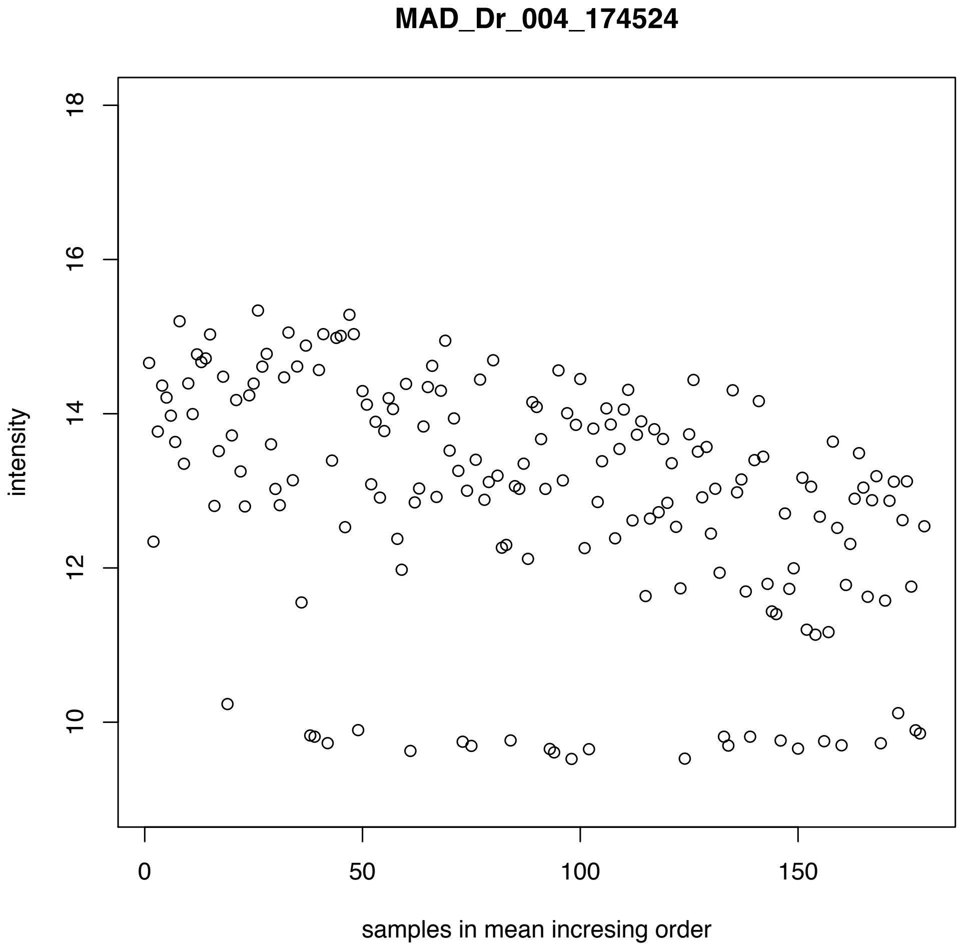
MAD_Dr_004_174524
intensity
samples in mean incresing order
0
50
100
150
10
12
14
16
18

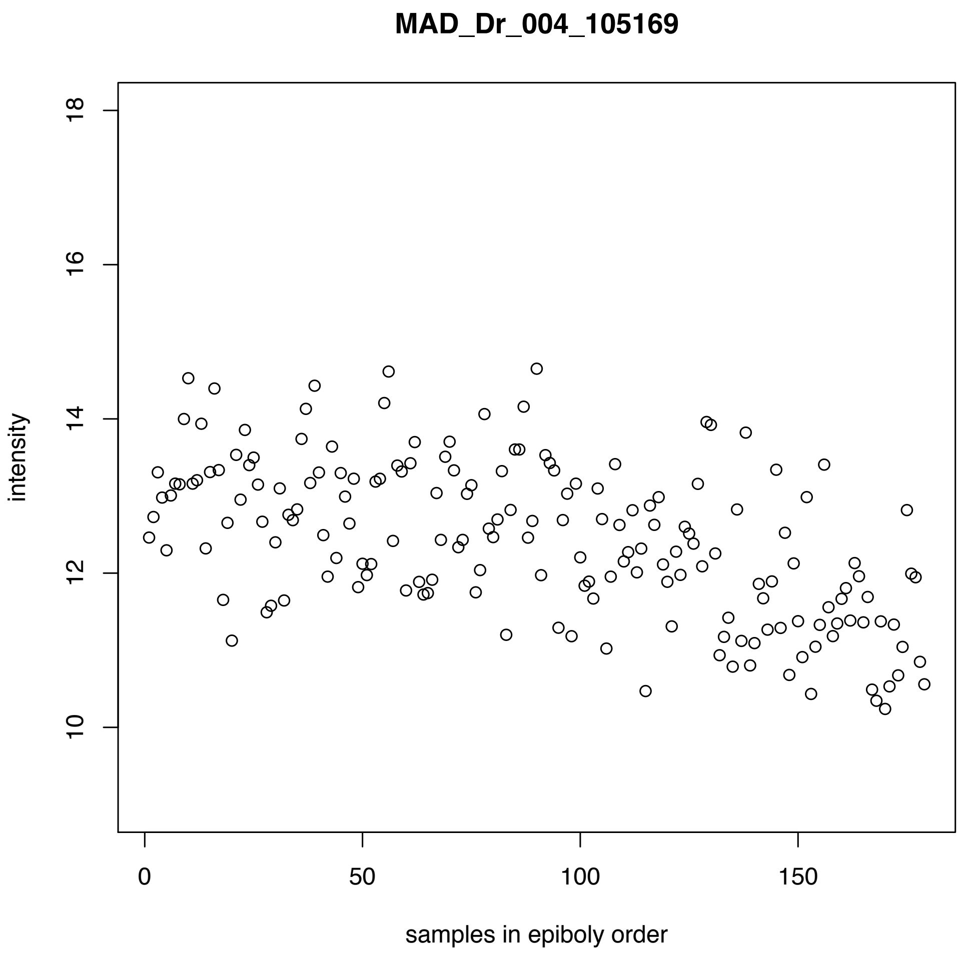
MAD_Dr_004_105169
intensity
18
16
14
12
10
0
50
100
150
samples in epiboly order

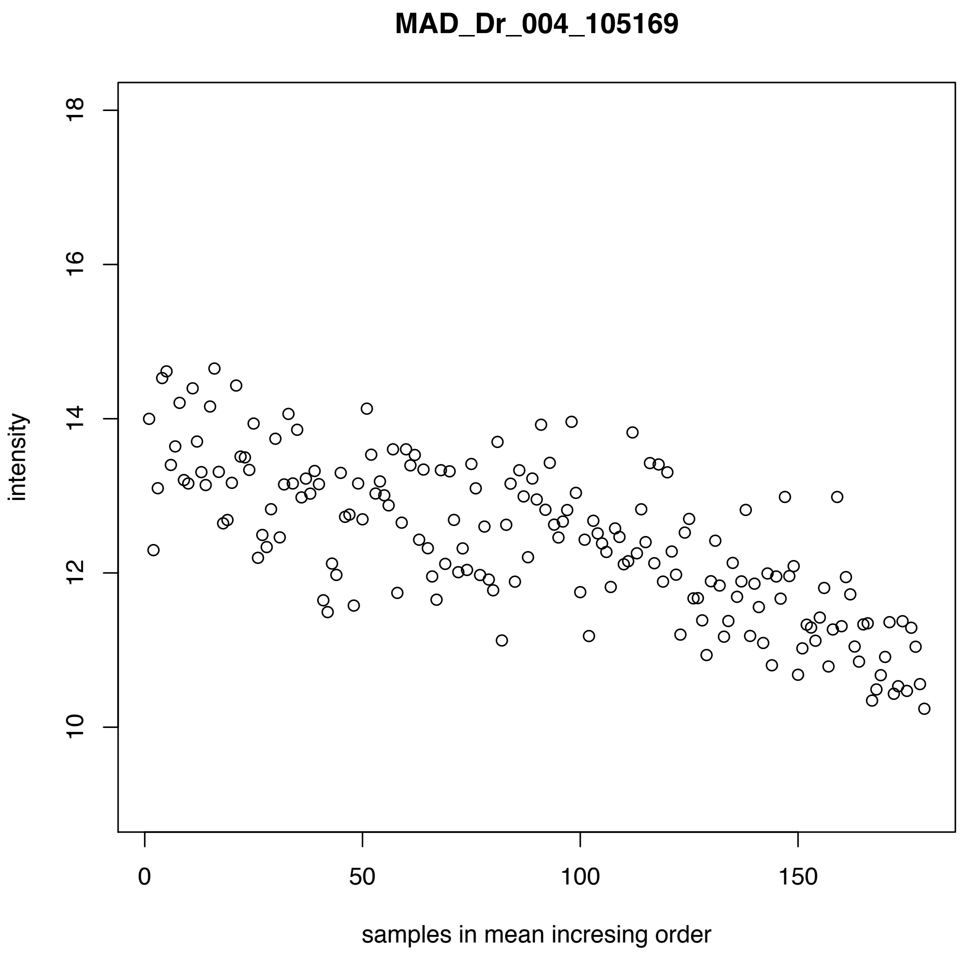
MAD_Dr_004_105169
intensity
18
16
14
12
10
0
50
100
150
samples in mean incresing order

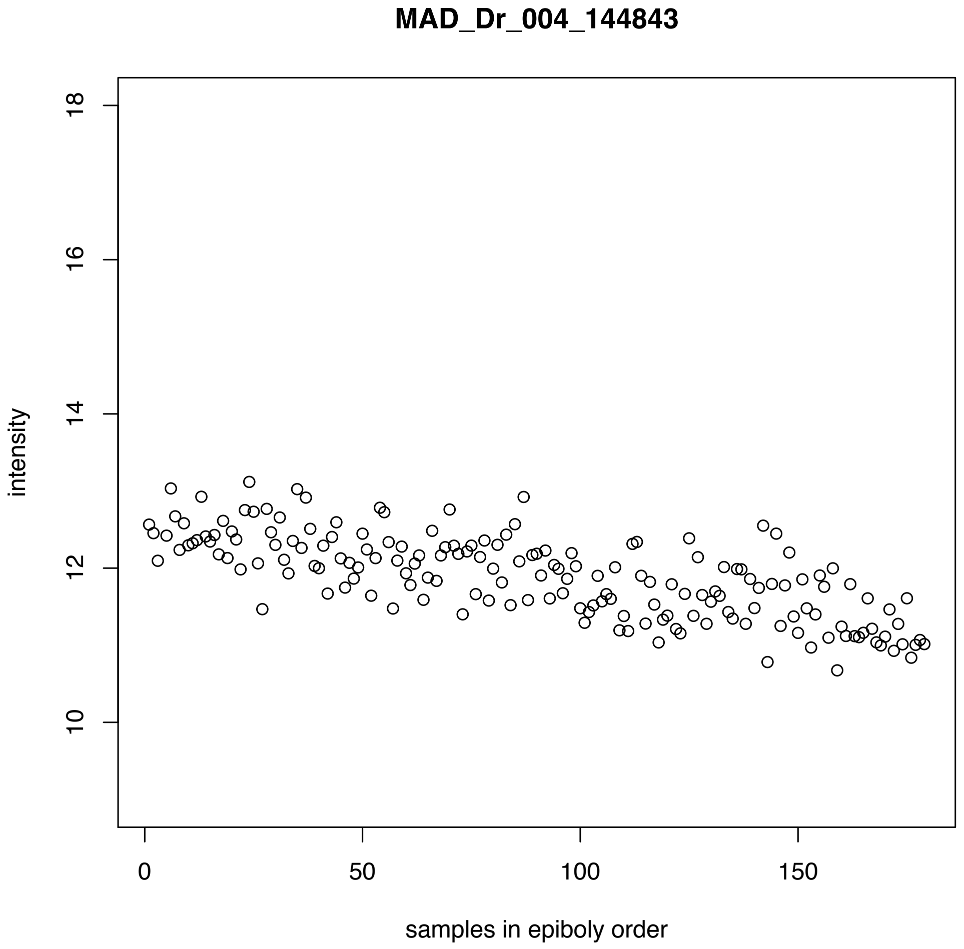
MAD_Dr_004_144843
intensity
samples in epiboly order
10
12
14
16
18
0
50
100
150

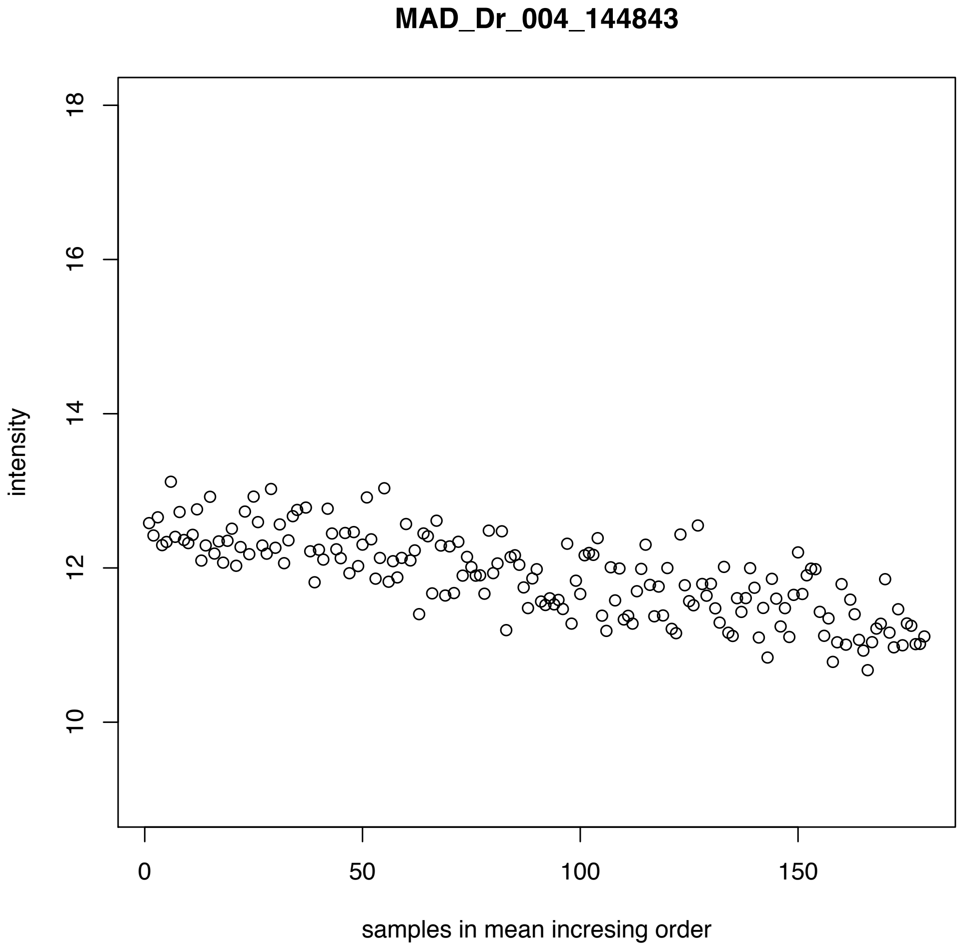
MAD_Dr_004_144843
intensity
samples in mean incresing order
10
12
14
16
18
0
50
100
150

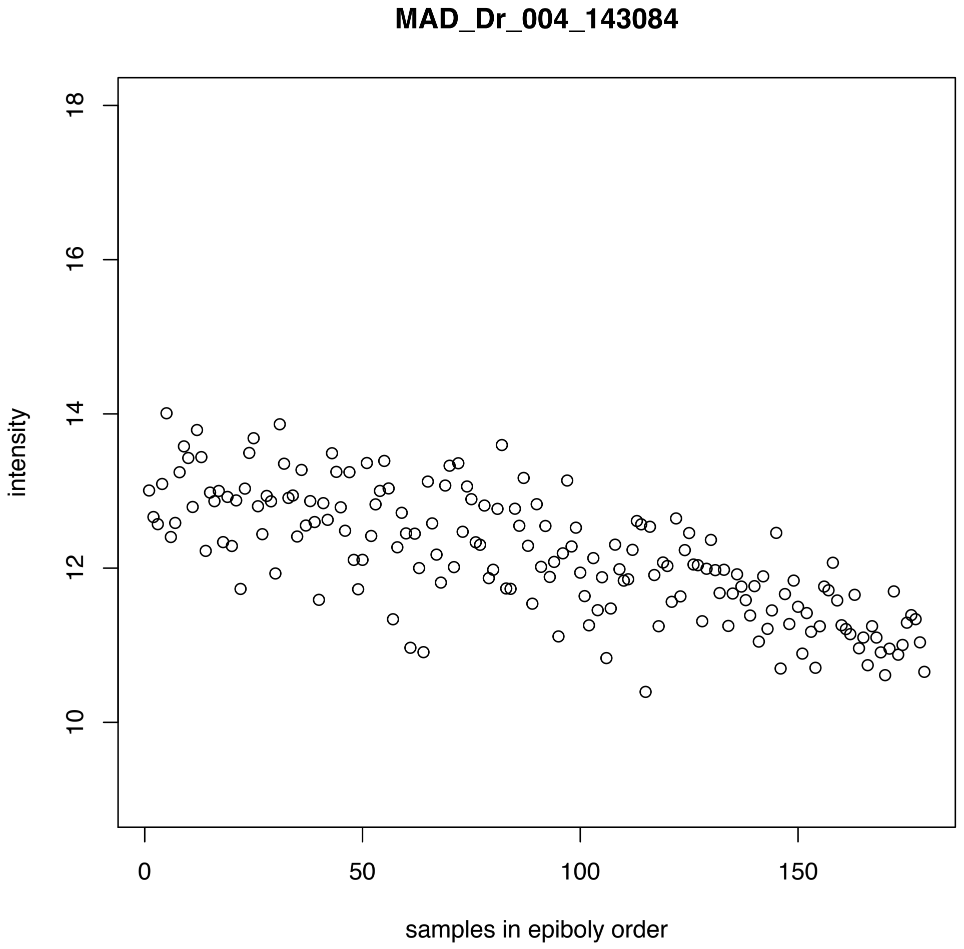
MAD_Dr_004_143084
intensity
10
12
14
16
18
0
50
100
150
samples in epiboly order

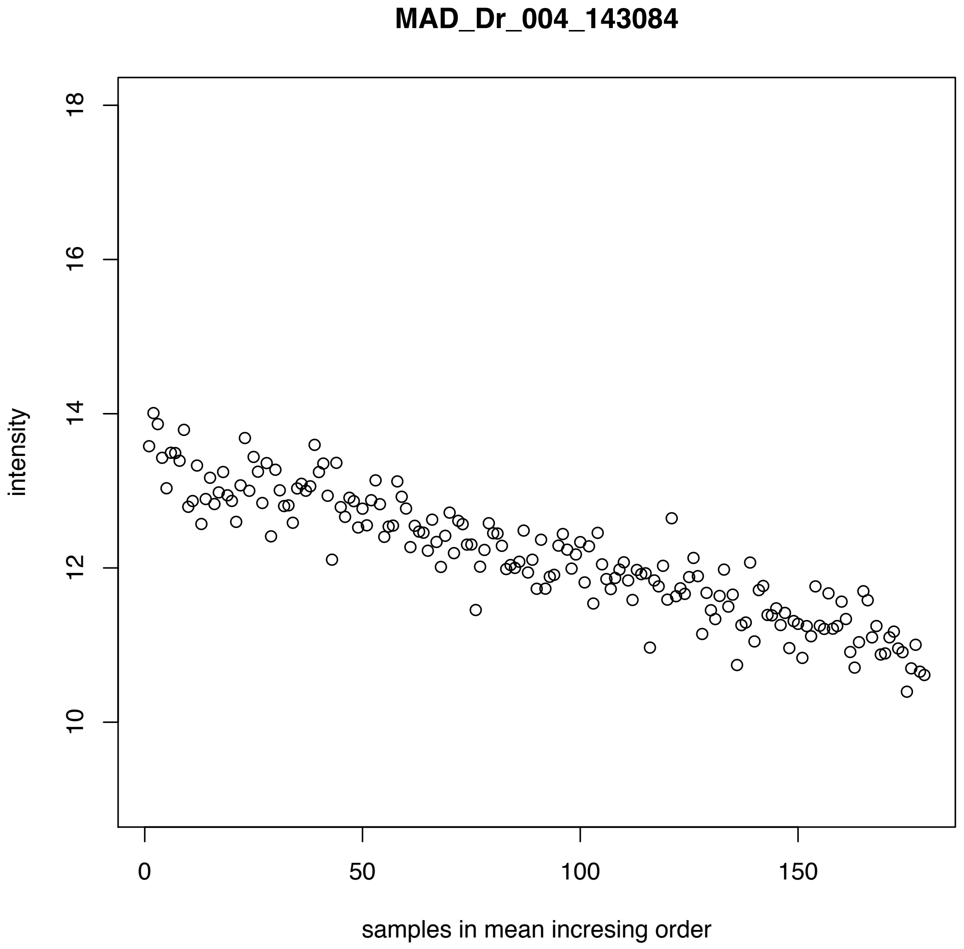
MAD_Dr_004_143084
intensity
10
12
14
16
18
0
50
100
150
samples in mean incresing order

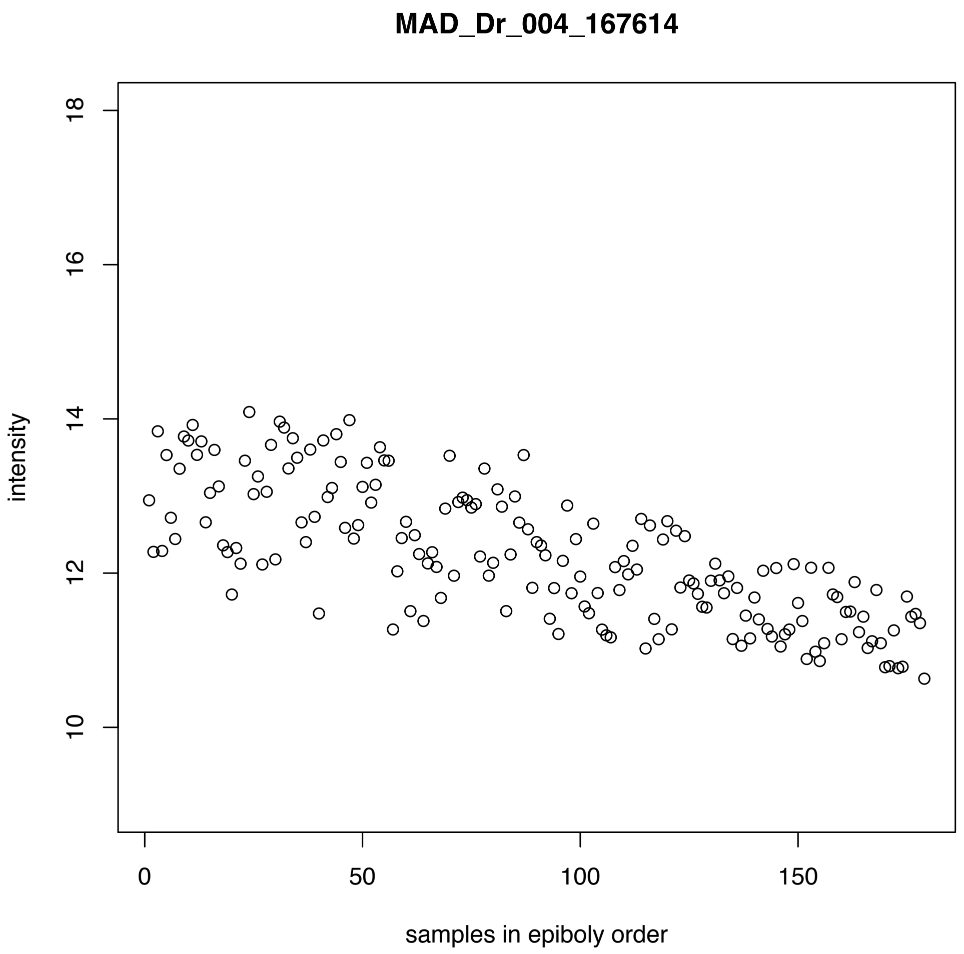
MAD_Dr_004_167614
intensity
18
16
14
12
10
0
50
100
150
samples in epiboly order

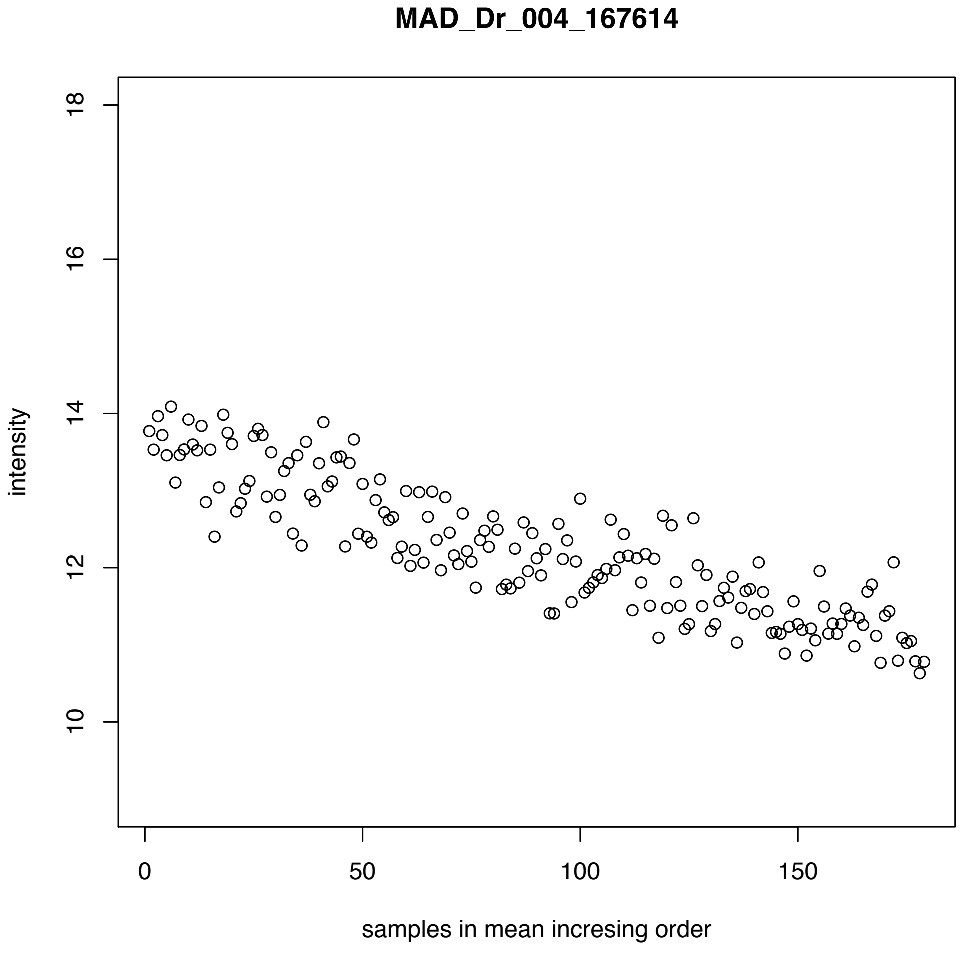
MAD_Dr_004_167614
intensity
18
16
14
12
10
0
50
100
150
samples in mean incresing order

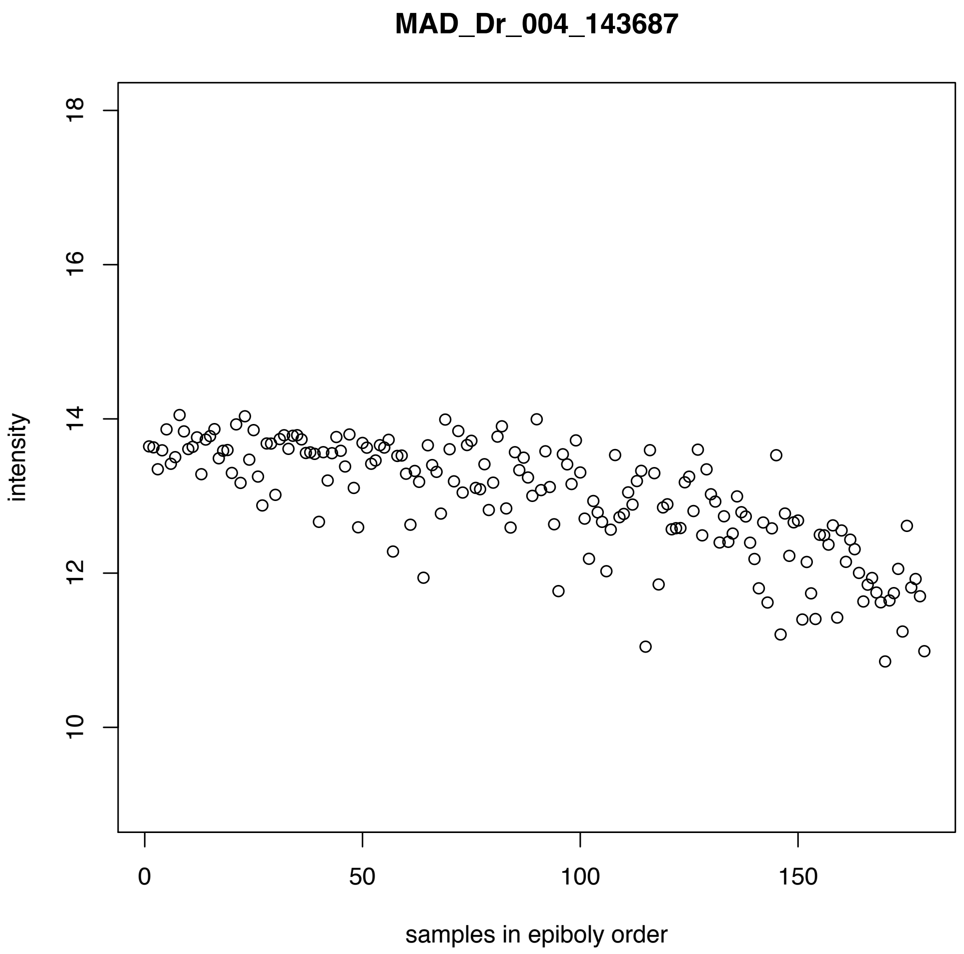
MAD_Dr_004_143687
intensity
10
12
14
16
18
0
50
100
150
samples in epiboly order

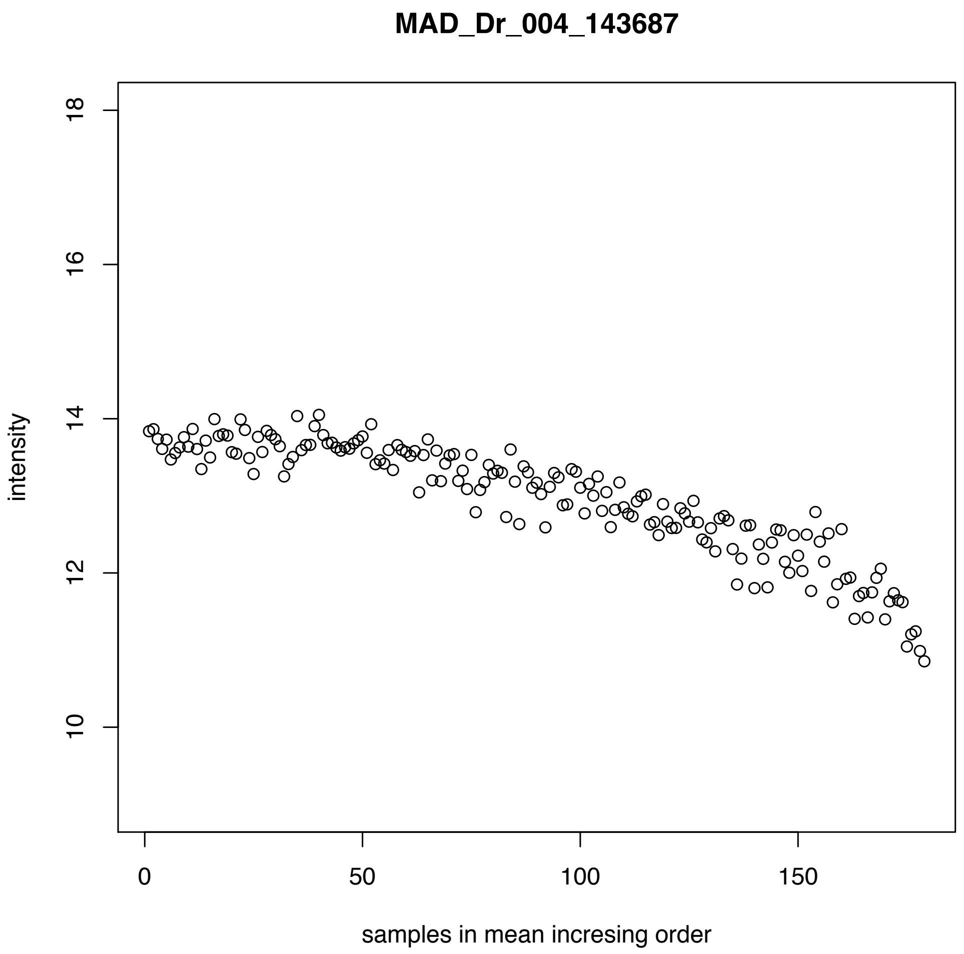
MAD_Dr_004_143687
intensity
10
12
14
16
18
0
50
100
150
samples in mean incresing order

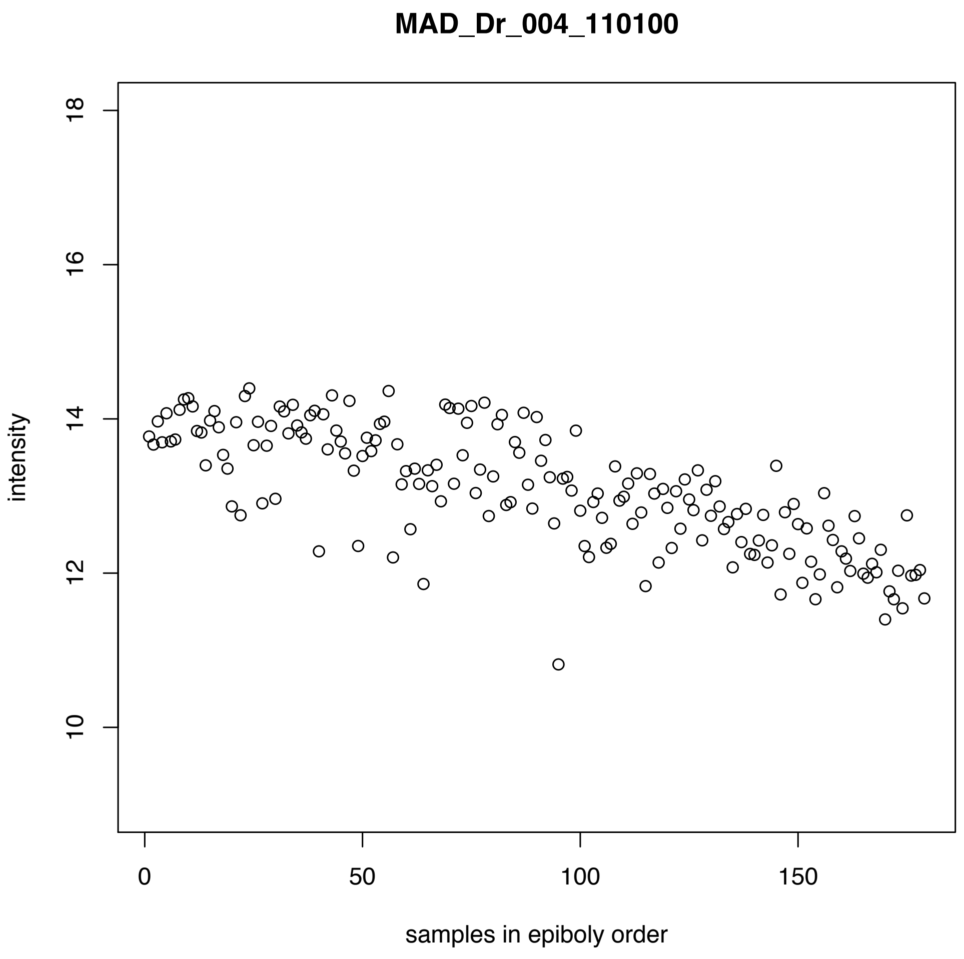
MAD_Dr_004_110100
intensity
10
12
14
16
18
0
50
100
150
samples in epiboly order

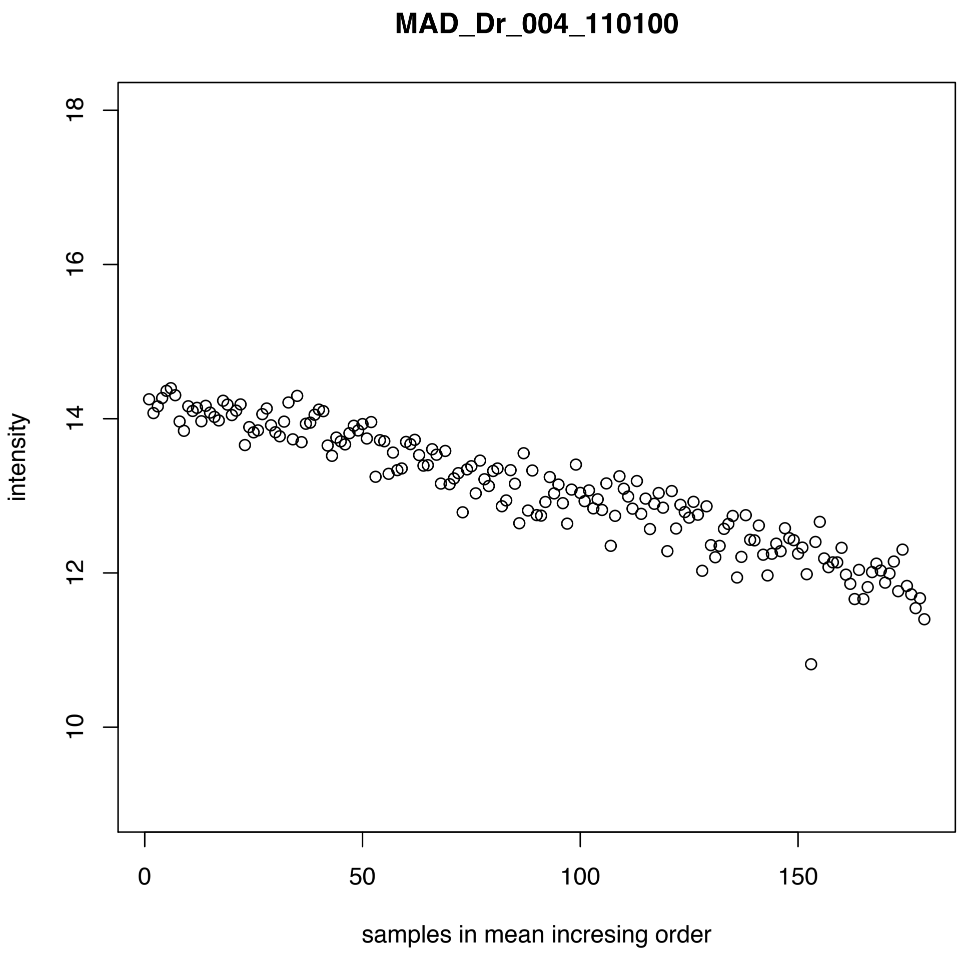
MAD_Dr_004_110100
intensity
10
12
14
16
18
0
50
100
150
samples in mean incresing order

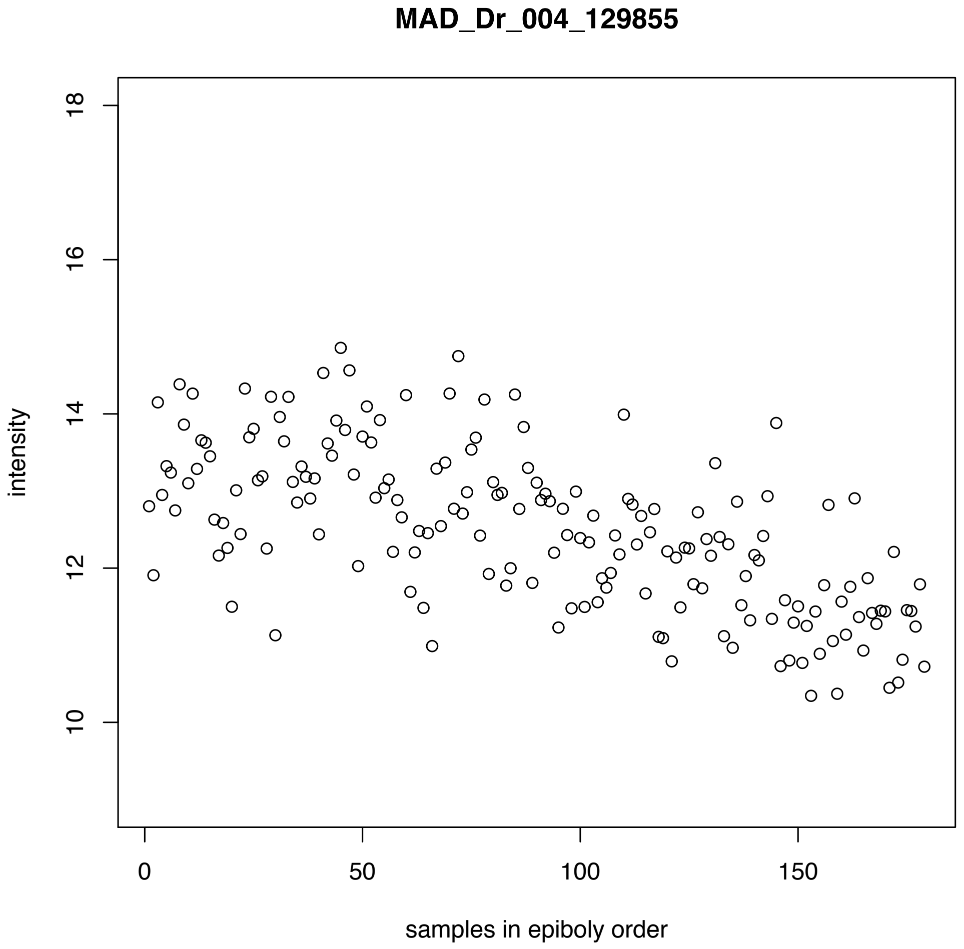
MAD_Dr_004_129855
intensity
samples in epiboly order
10
12
14
16
18
0
50
100
150

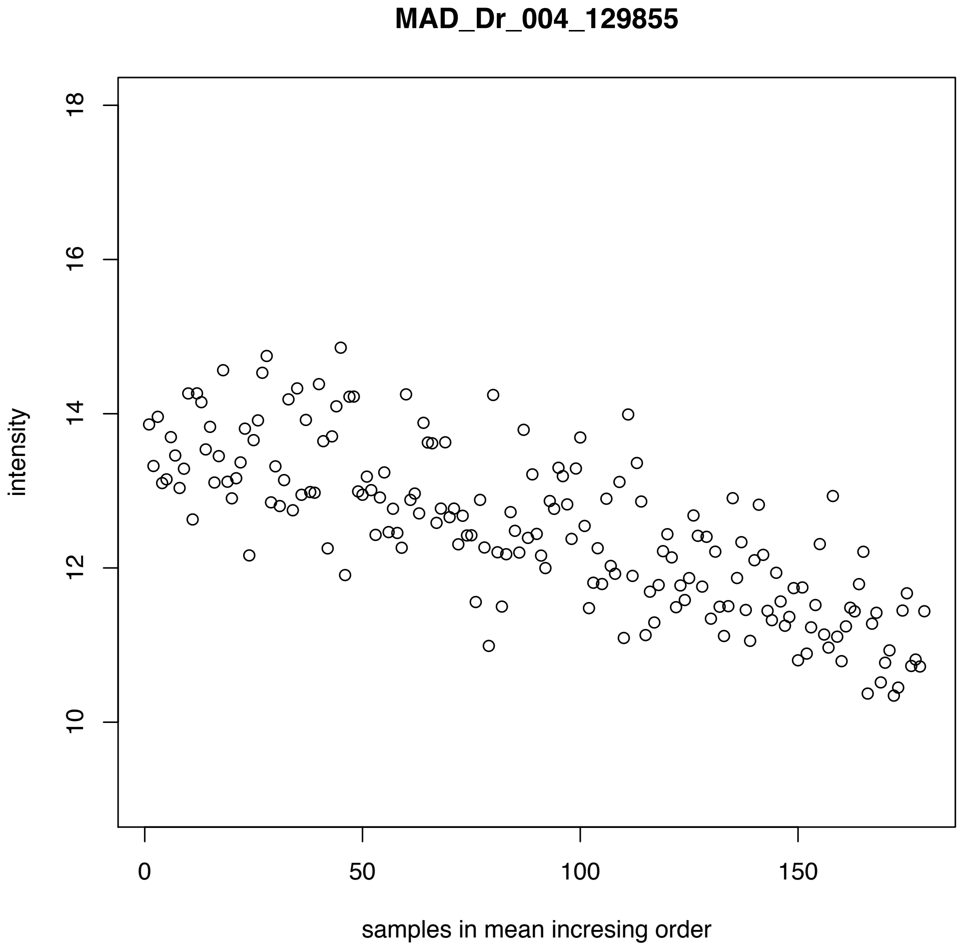
MAD_Dr_004_129855
intensity
samples in mean incresing order
10
12
14
16
18
0
50
100
150

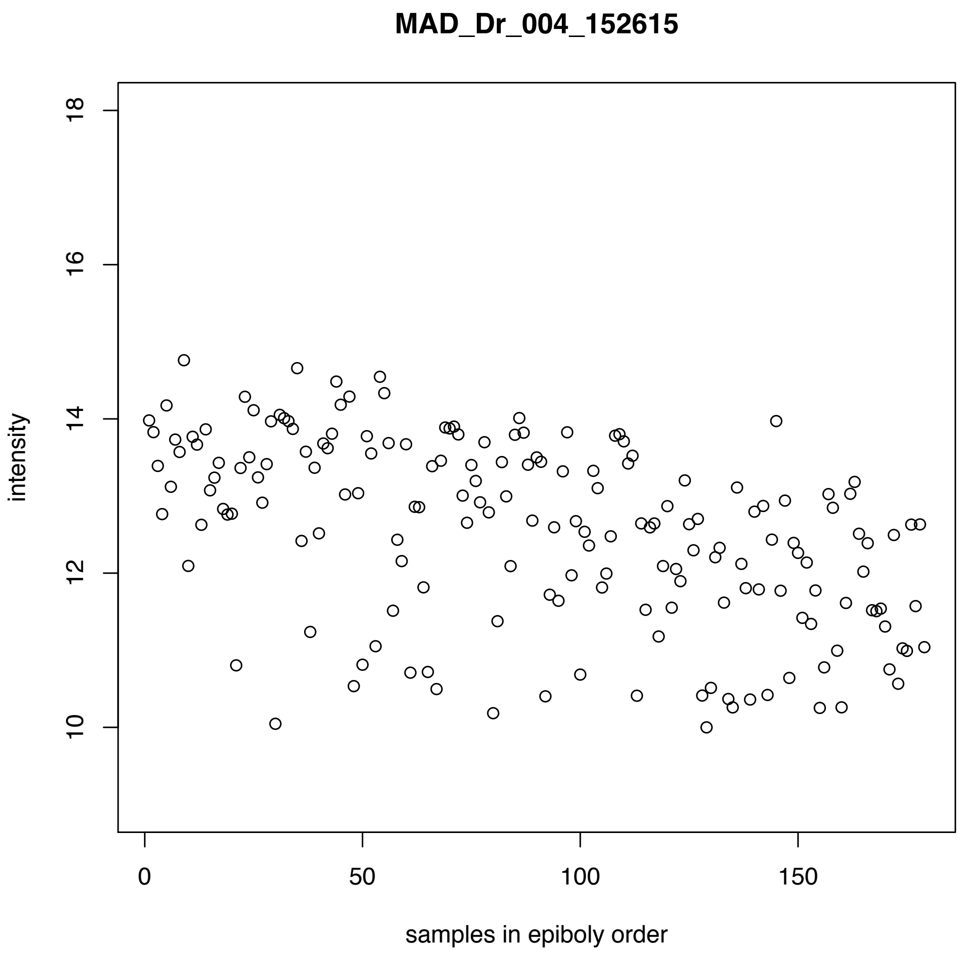
MAD_Dr_004_152615
intensity
samples in epiboly order
10
12
14
16
18
0
50
100
150

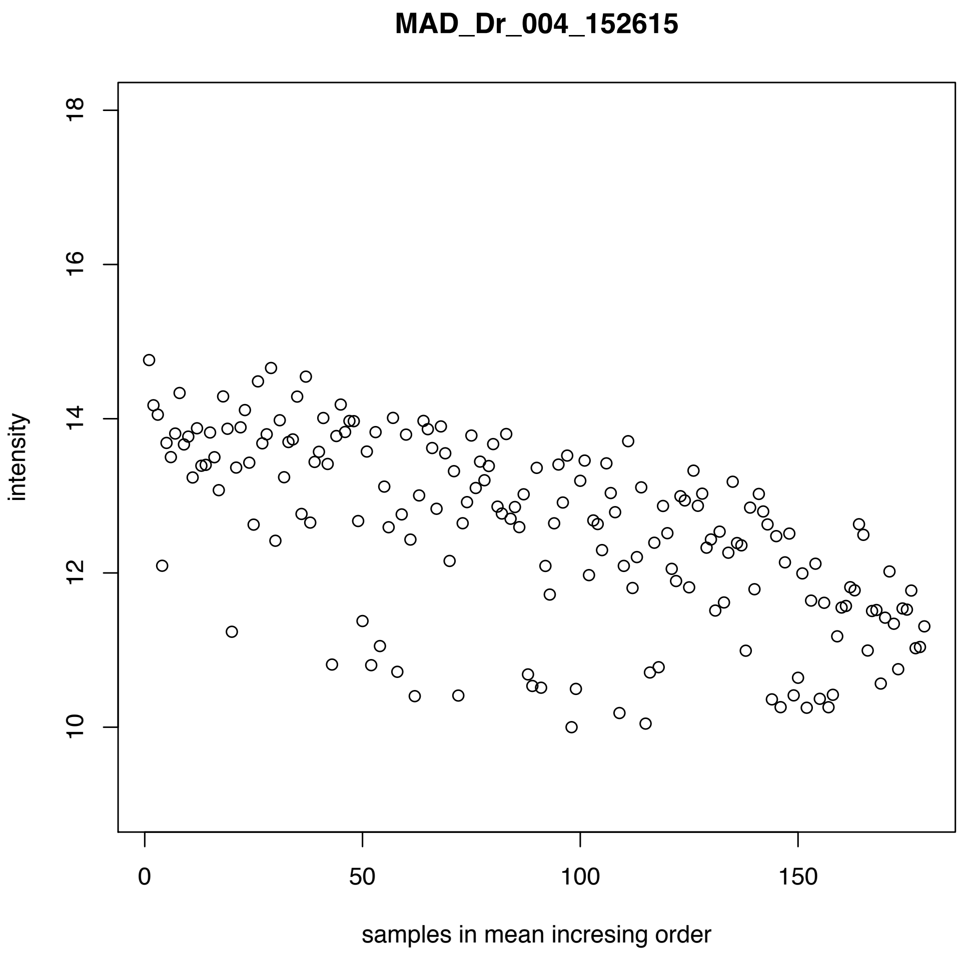
MAD_Dr_004_152615
intensity
samples in mean incresing order
10
12
14
16
18
0
50
100
150

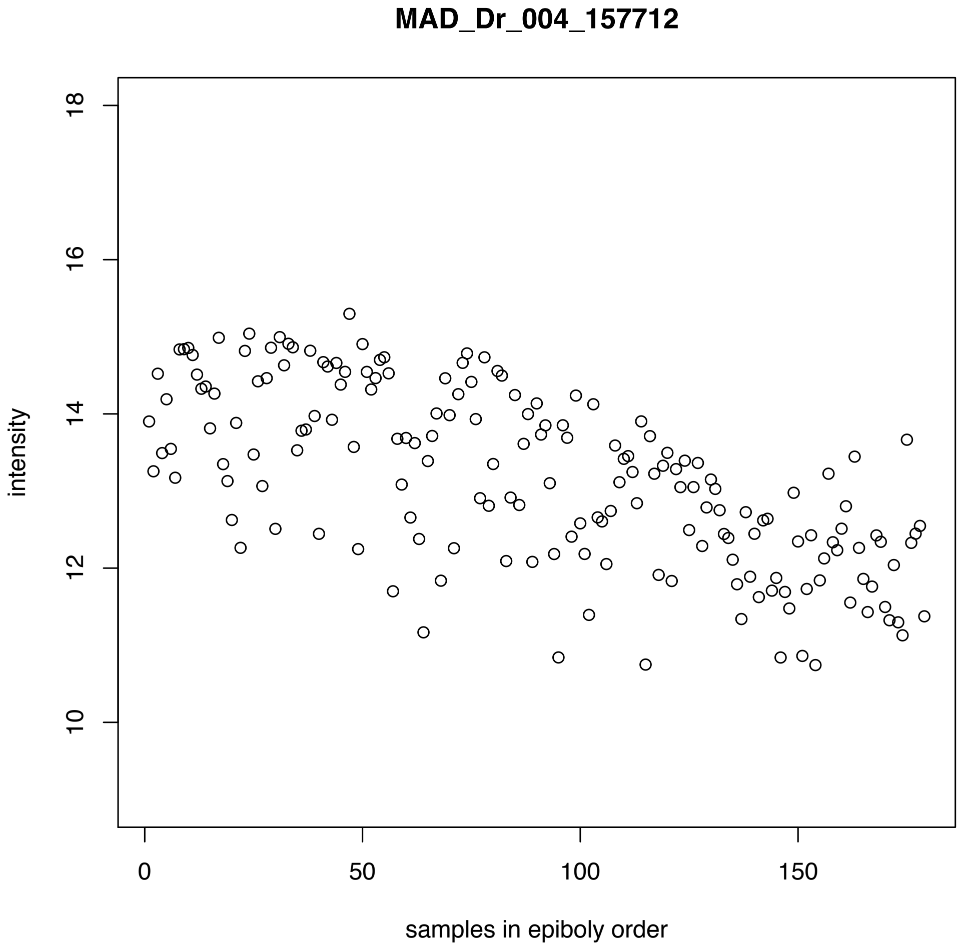

MAD_Dr_004_157712
intensity
samples in epiboly order

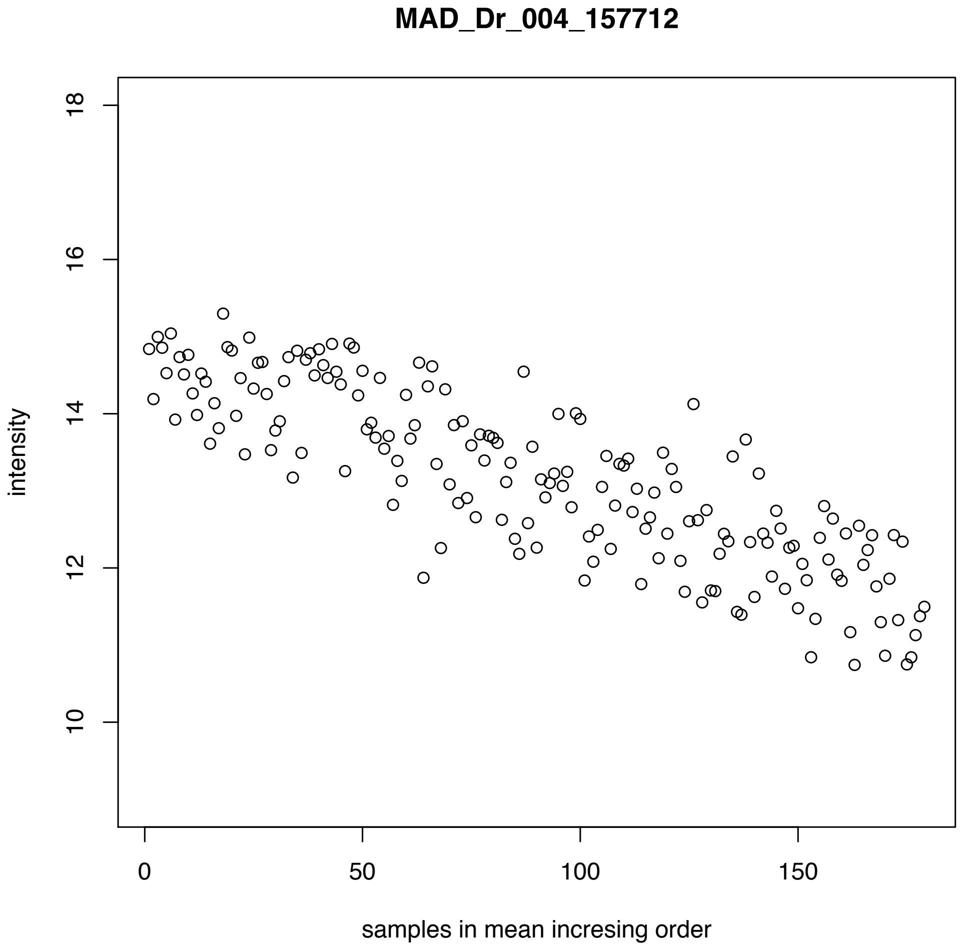

MAD_Dr_004_157712
intensity
samples in mean incresing order

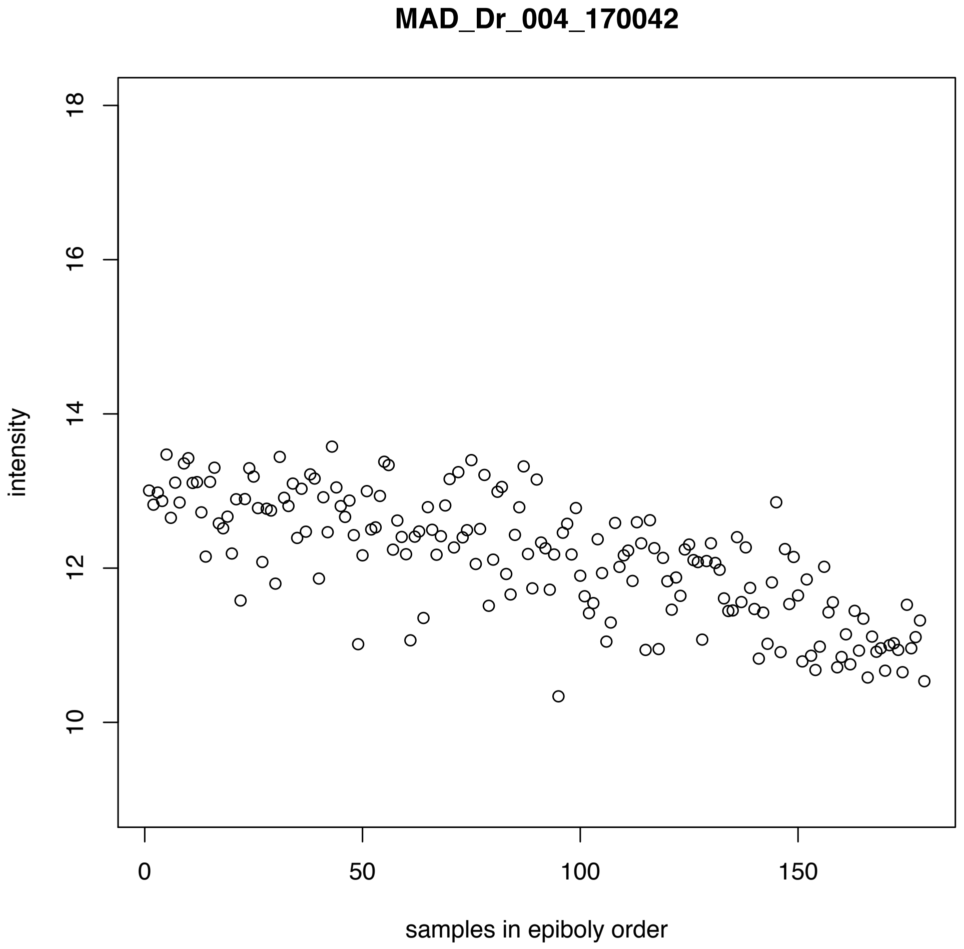
MAD_Dr_004_170042
intensity
10
12
14
16
18
0
50
100
150
samples in epiboly order

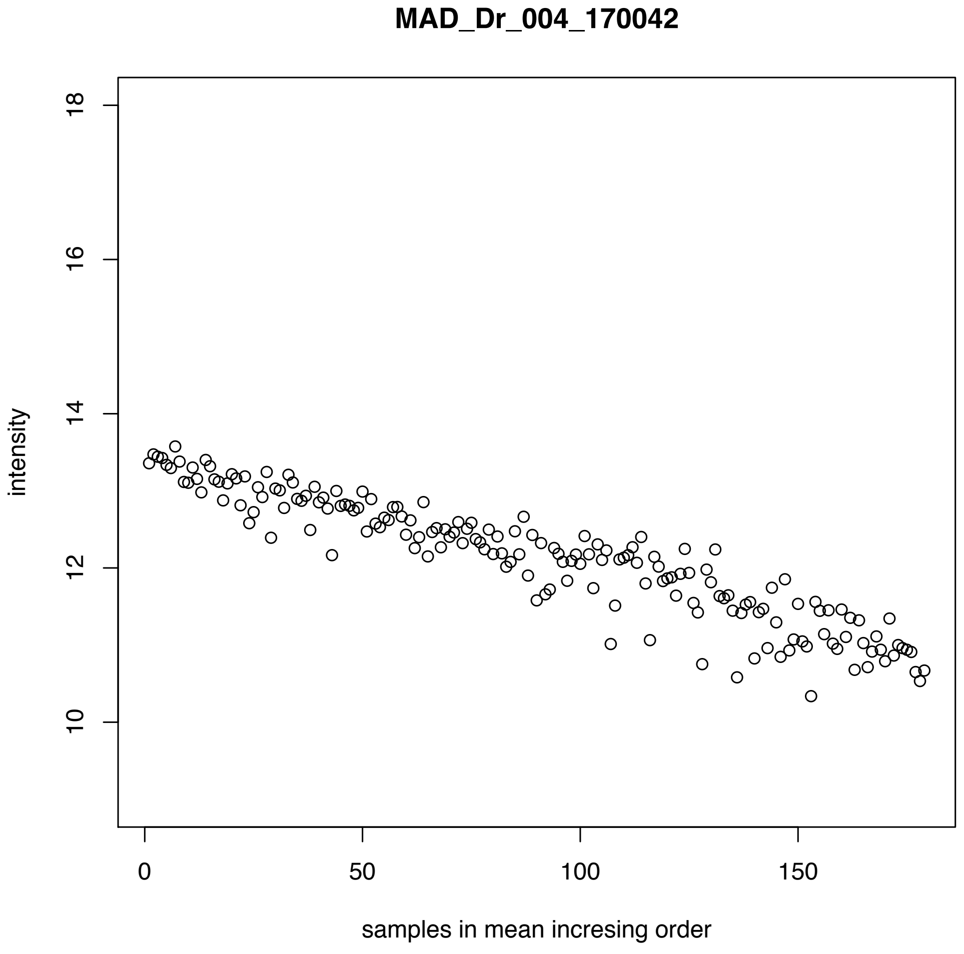
MAD_Dr_004_170042
intensity
10
12
14
16
18
0
50
100
150
samples in mean incresing order

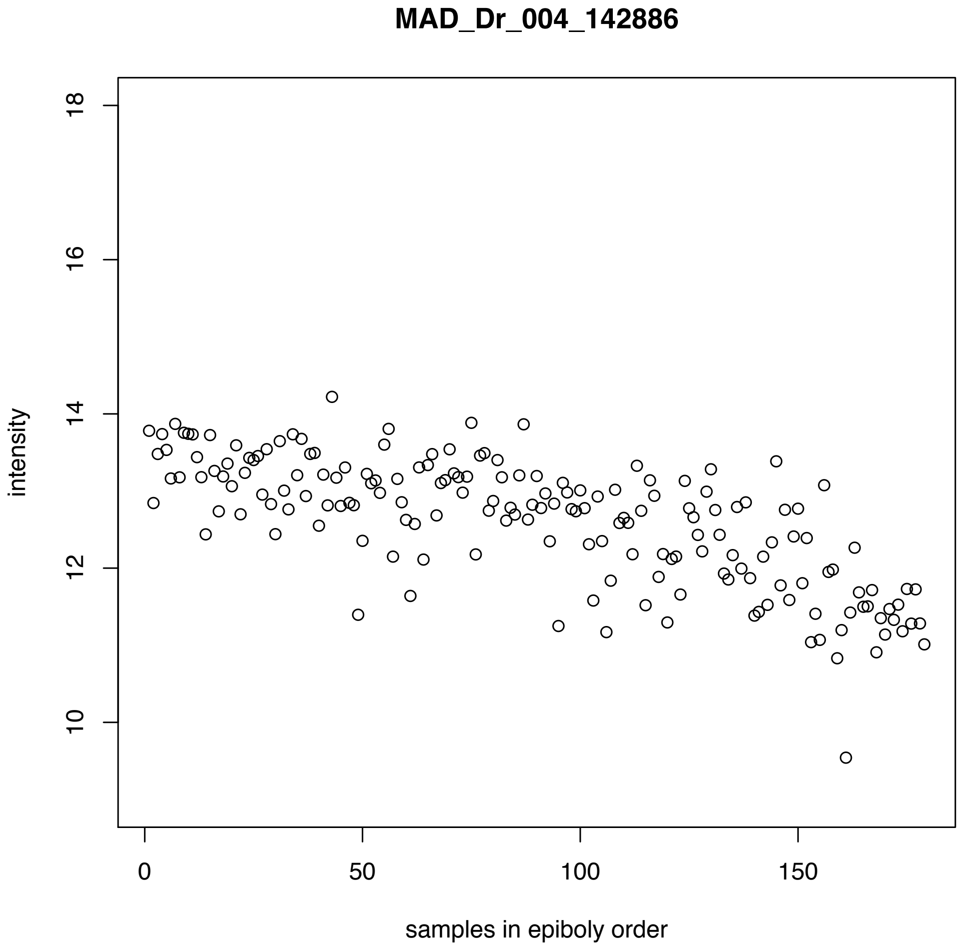
MAD_Dr_004_142886
intensity
samples in epiboly order
0
50
100
150
10
12
14
16
18

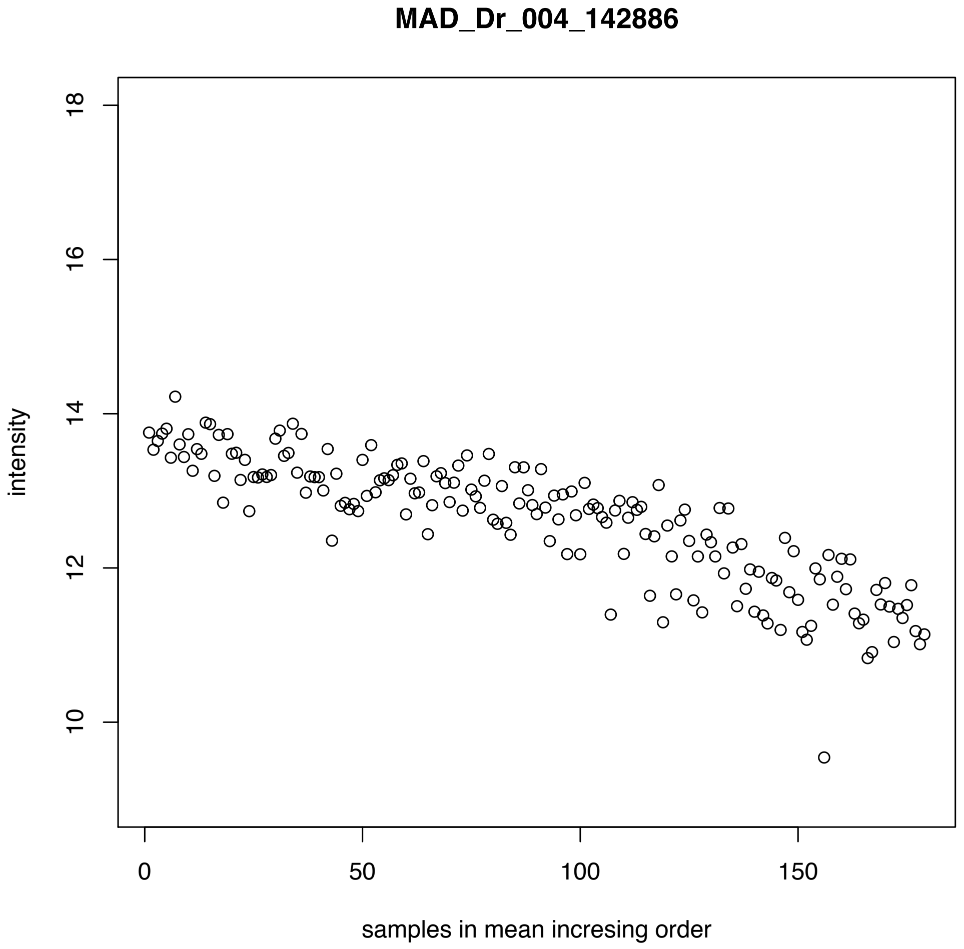
MAD_Dr_004_142886
intensity
samples in mean incresing order
0
50
100
150
10
12
14
16
18

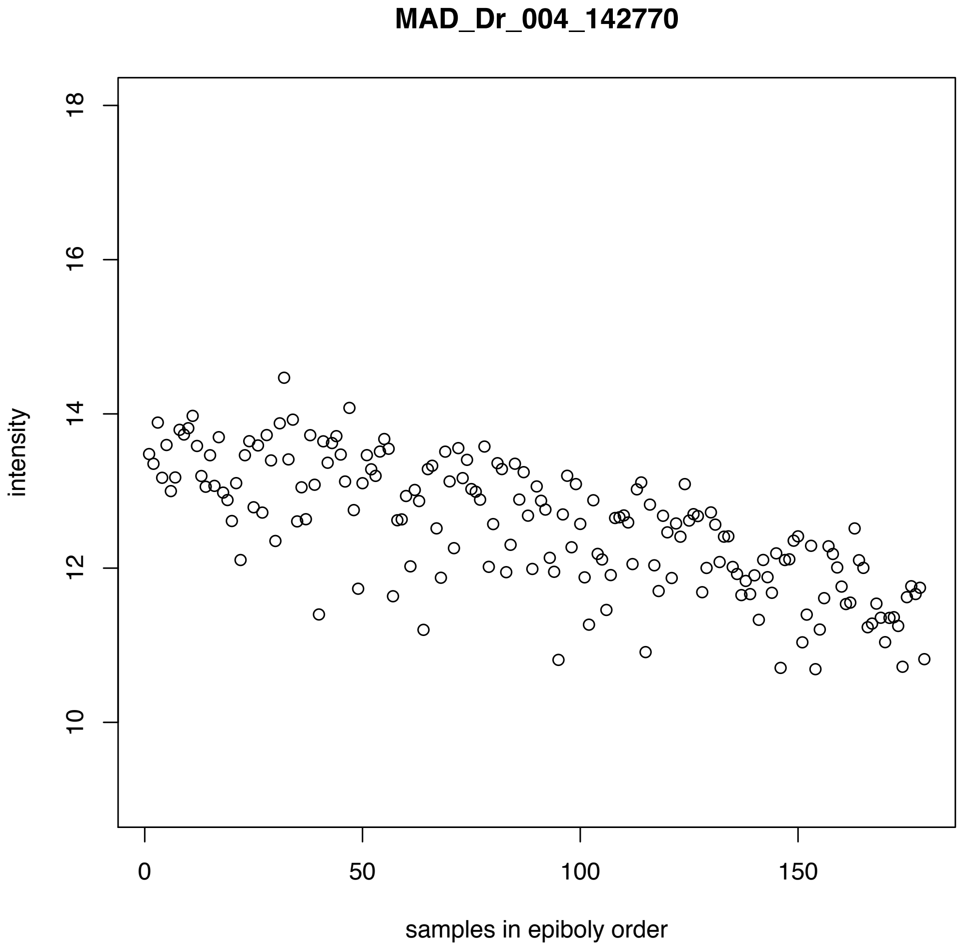
MAD_Dr_004_142770
intensity
10
12
14
16
18
0
50
100
150
samples in epiboly order

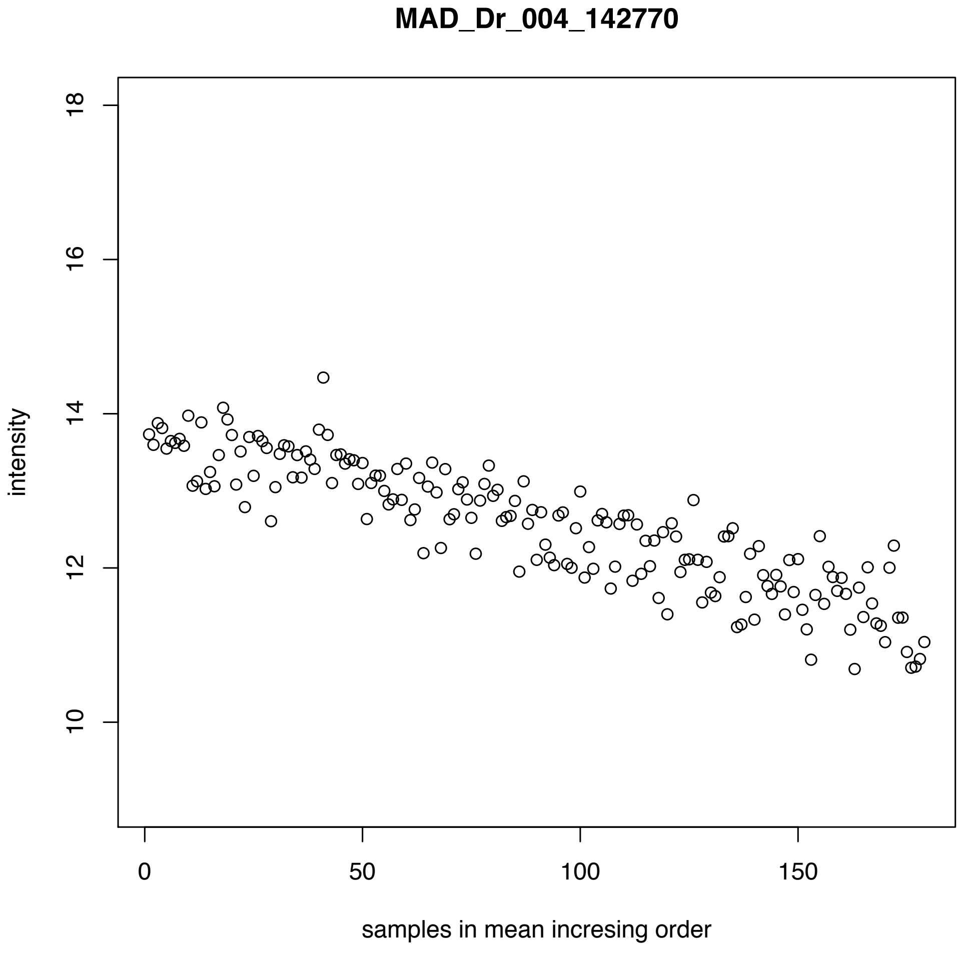
MAD_Dr_004_142770
intensity
10
12
14
16
18
0
50
100
150
samples in mean incresing order

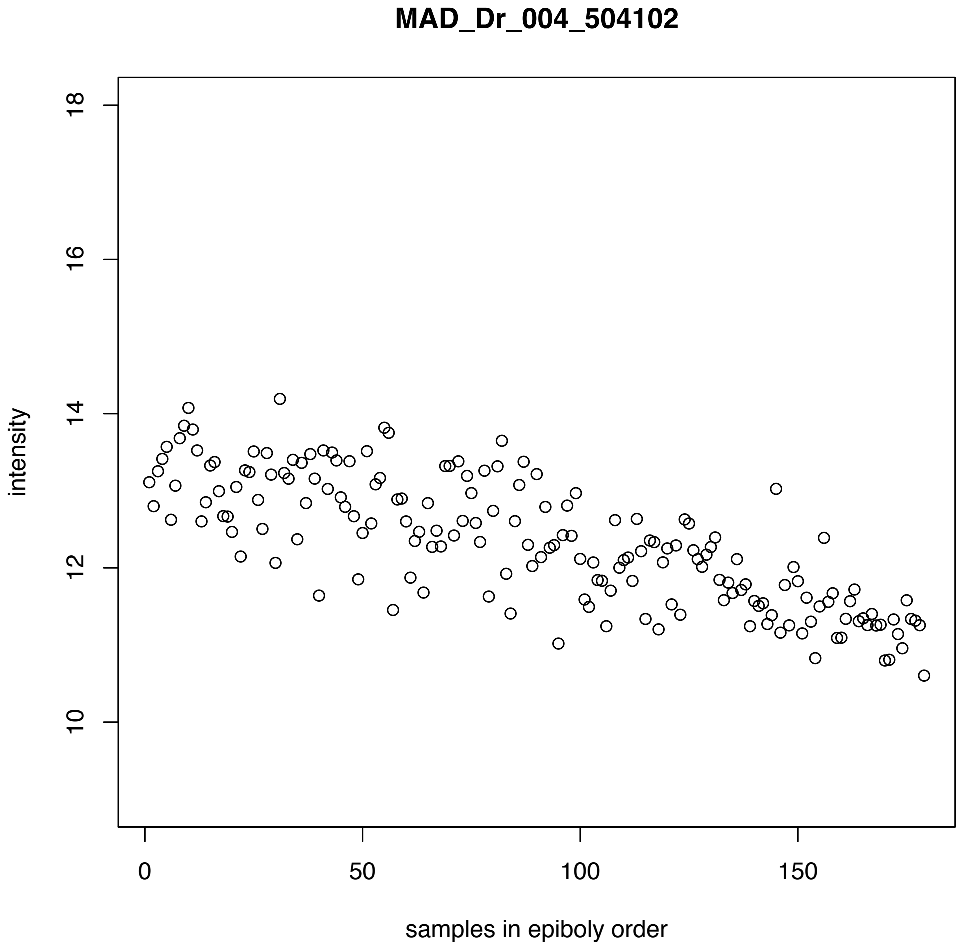

MAD_Dr_004_504102
intensity
10
12
14
16
18
0
50
100
150
samples in epiboly order

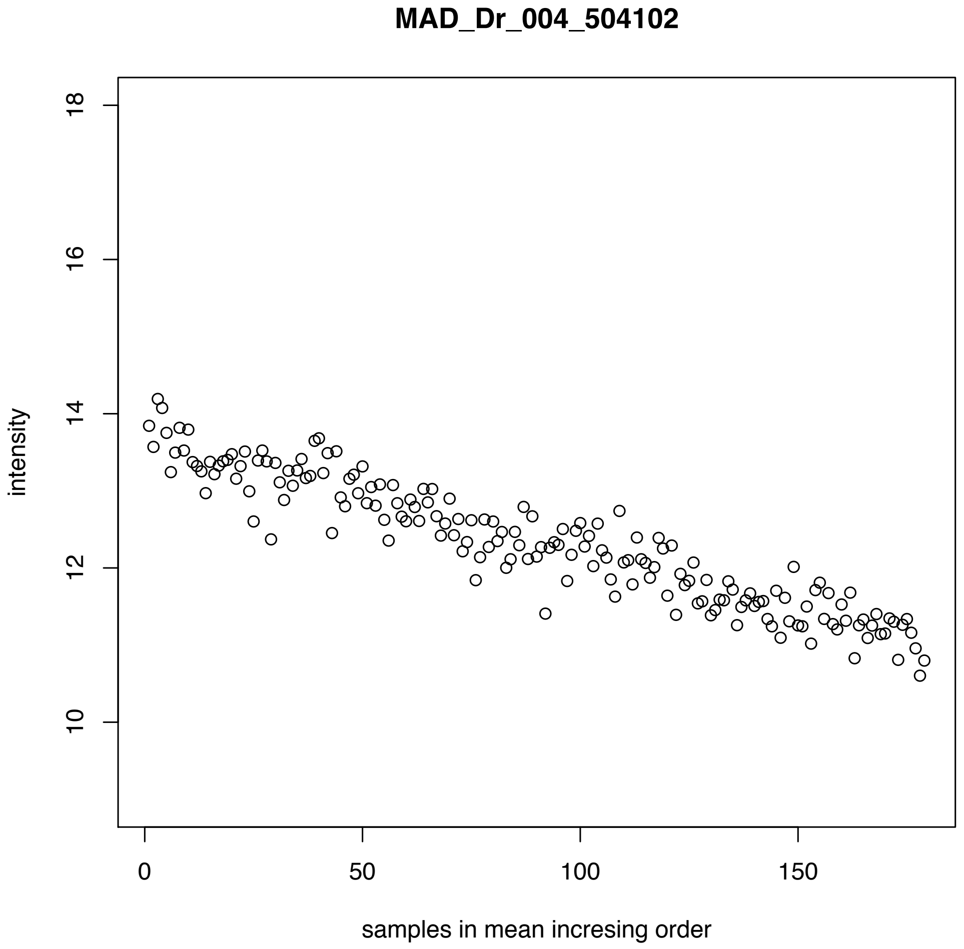

MAD_Dr_004_504102
intensity
10
12
14
16
18
0
50
100
150
samples in mean incresing order

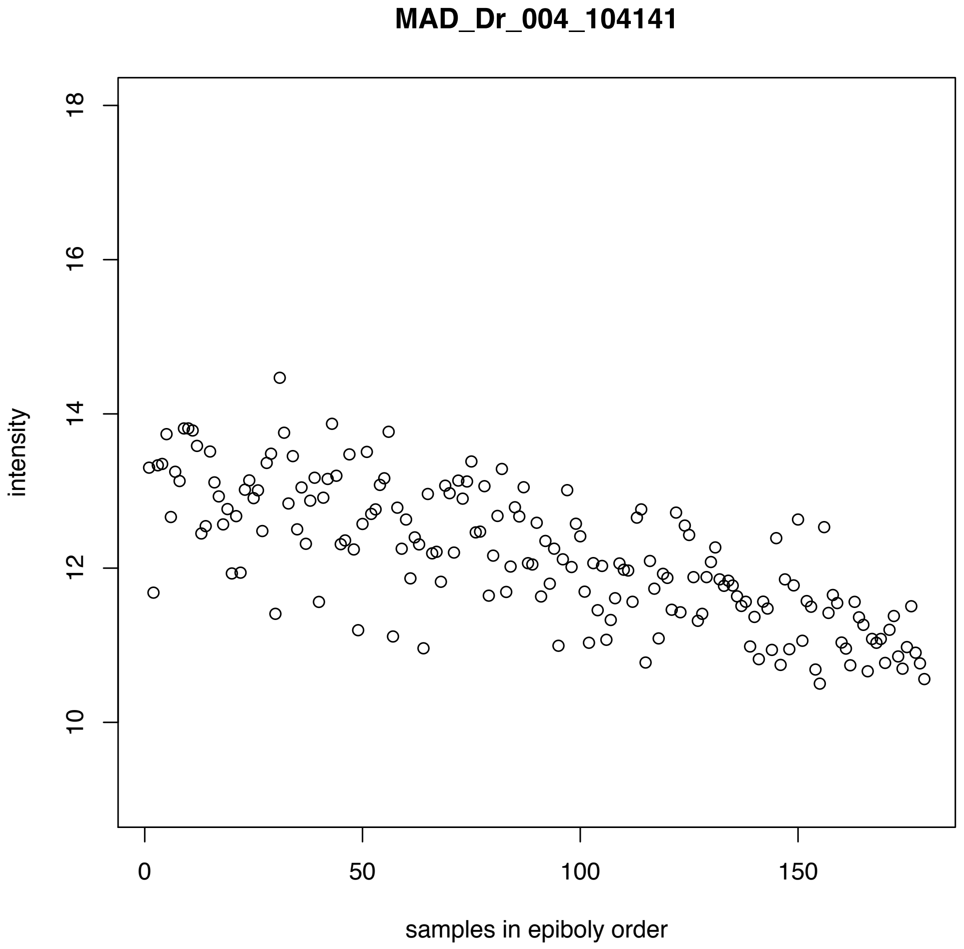
MAD_Dr_004_104141
intensity
samples in epiboly order
10
12
14
16
18
0
50
100
150

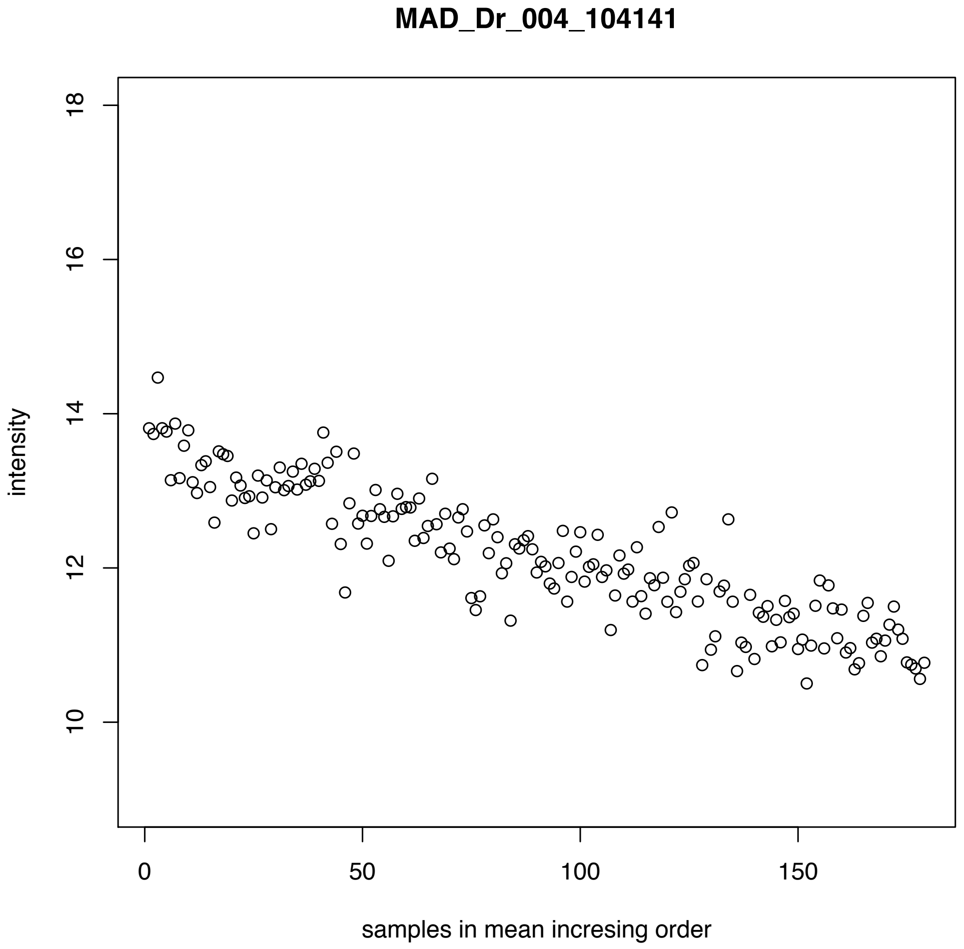
MAD_Dr_004_104141
intensity
samples in mean incresing order
10
12
14
16
18
0
50
100
150

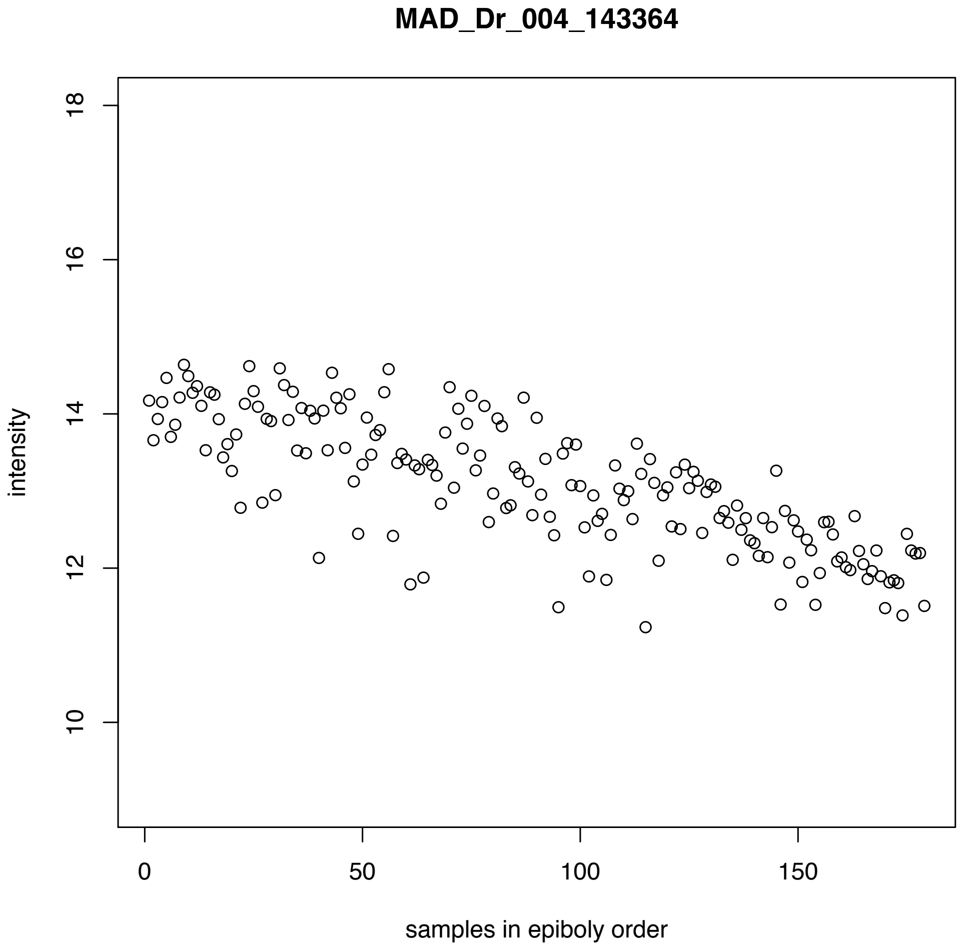
MAD_Dr_004_143364
intensity
18
16
14
12
10
0
50
100
150
samples in epiboly order

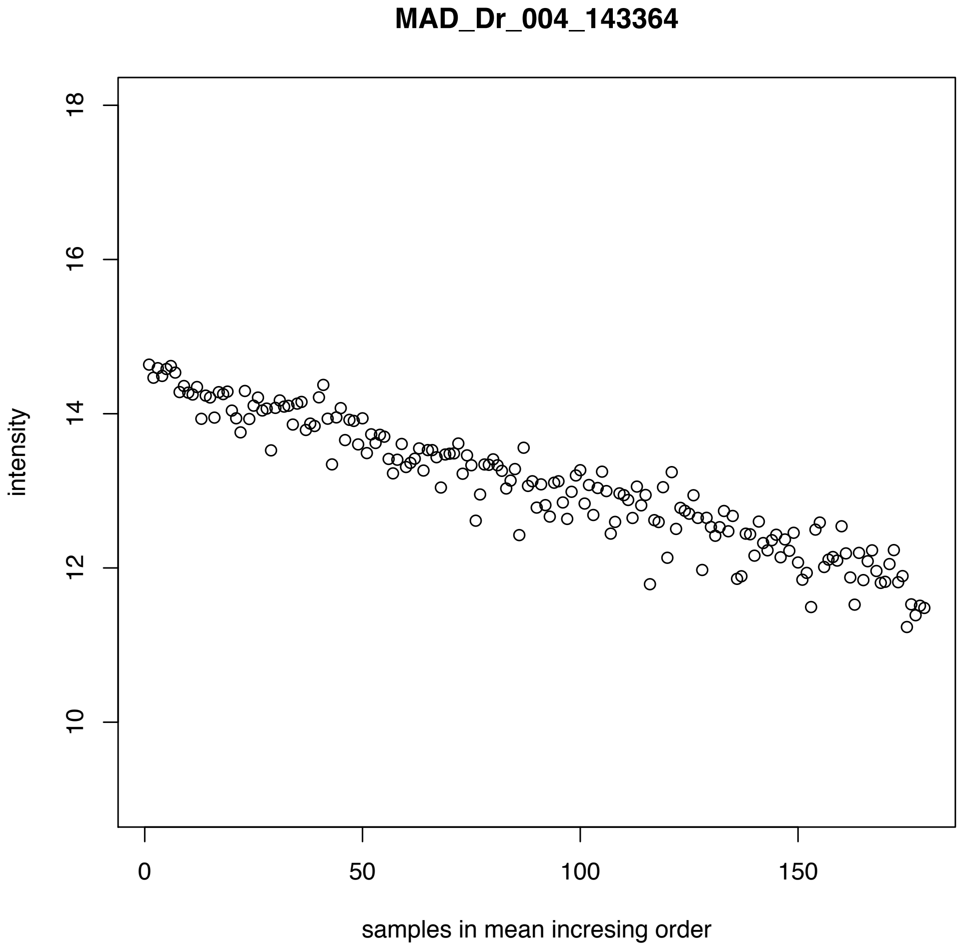
MAD_Dr_004_143364
intensity
18
16
14
12
10
0
50
100
150
samples in mean incresing order

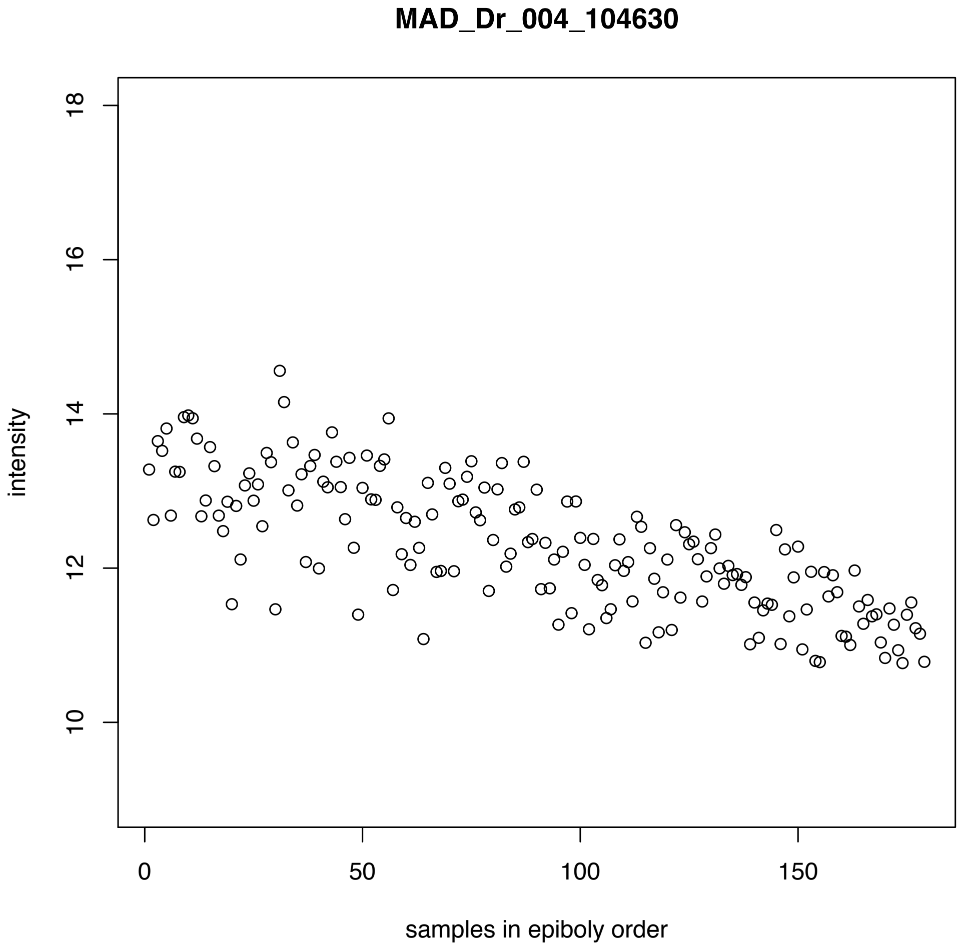
MAD_Dr_004_104630
intensity
samples in epiboly order
10
12
14
16
18
0
50
100
150

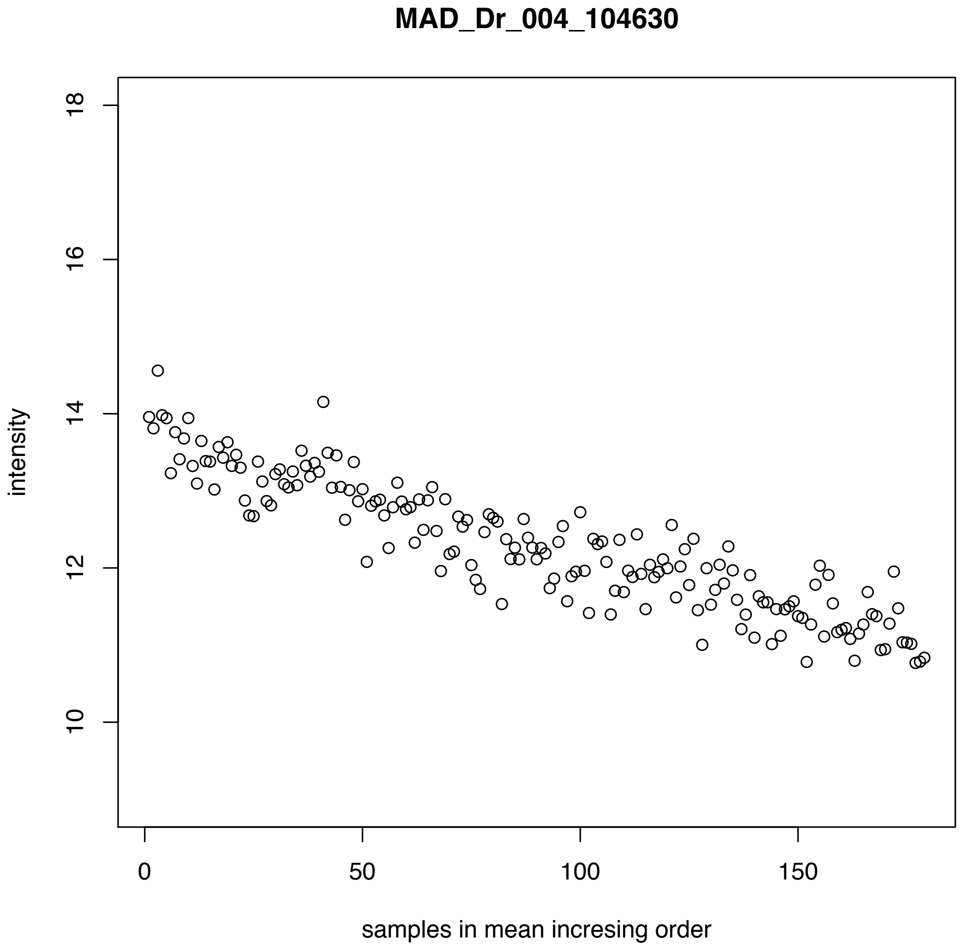
MAD_Dr_004_104630
intensity
samples in mean incresing order
10
12
14
16
18
0
50
100
150

MAD_Dr_004_162396

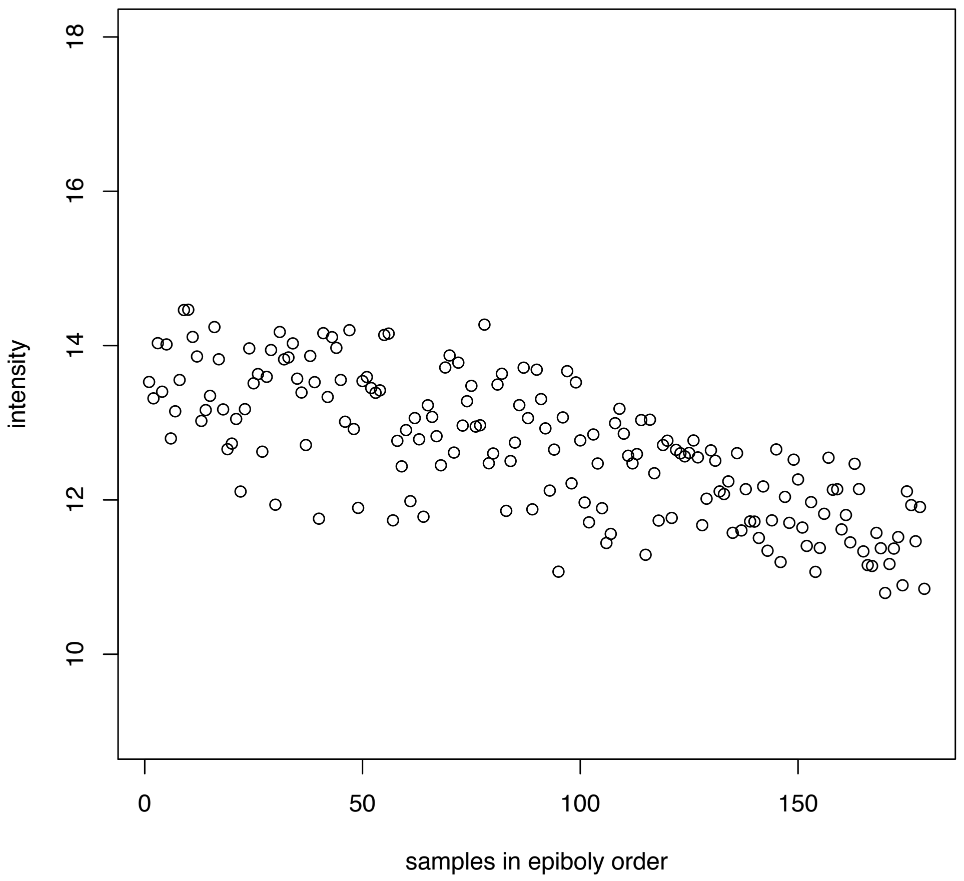

MAD_Dr_004_162396

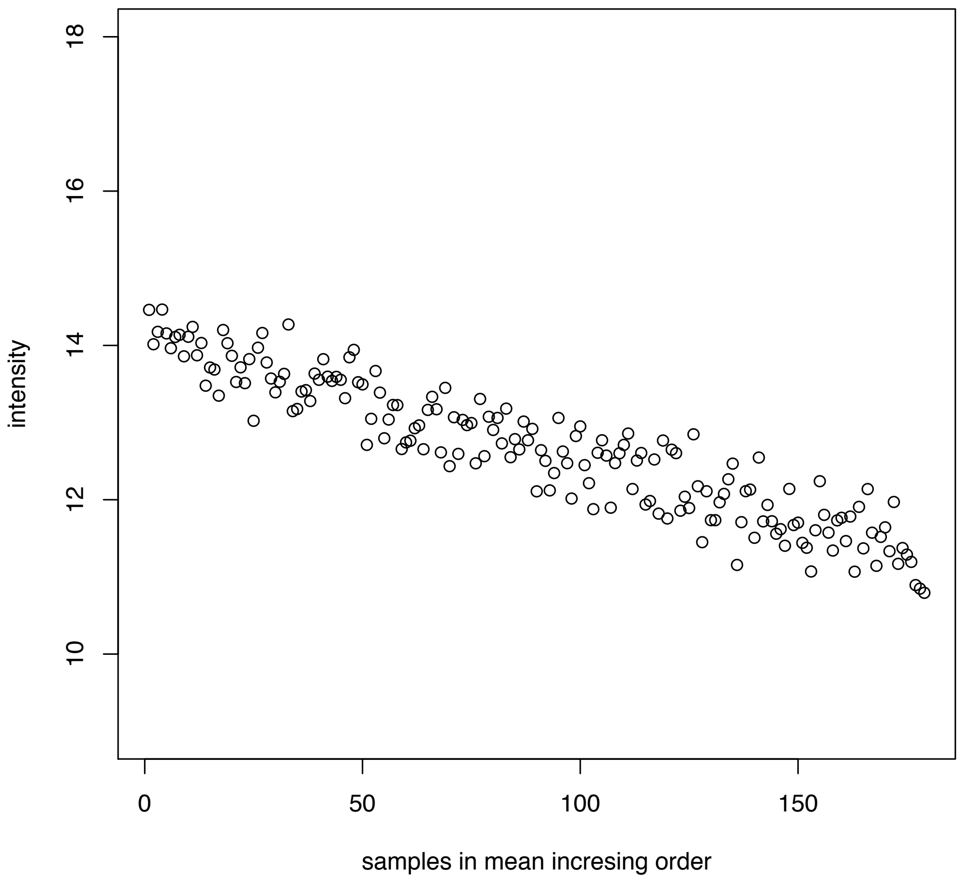

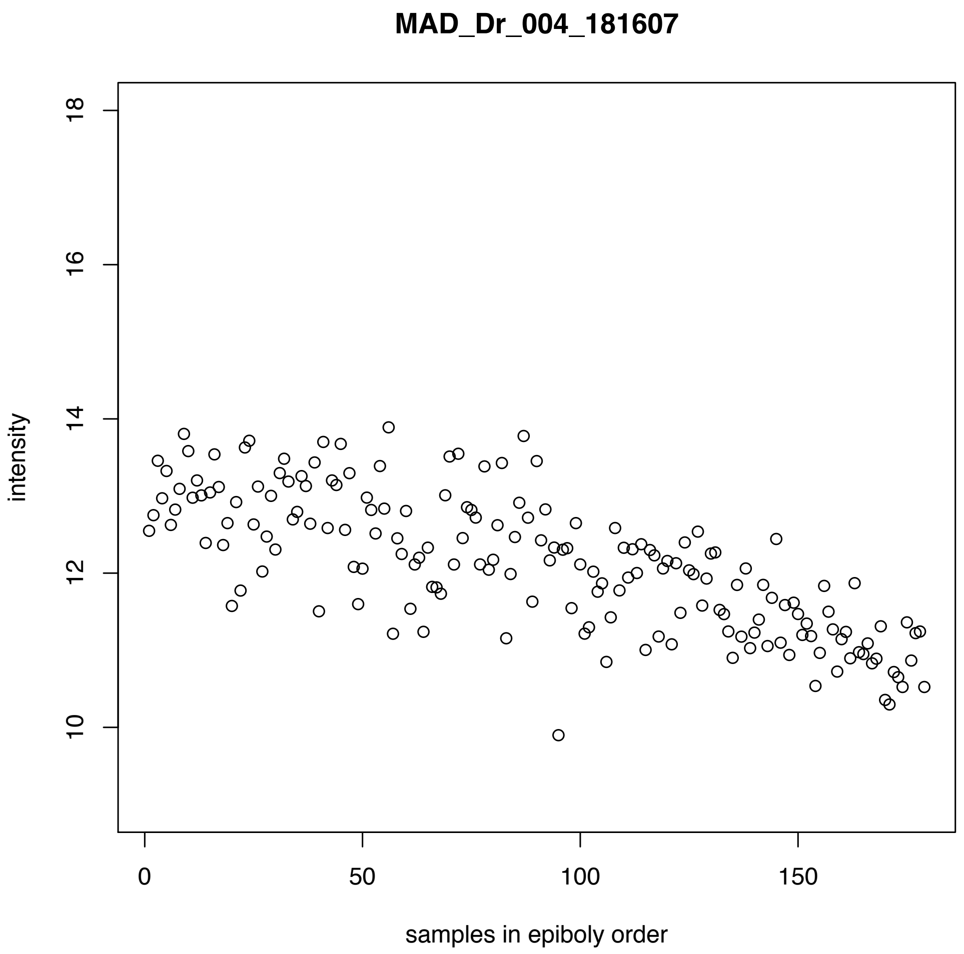
MAD_Dr_004_181607
intensity
samples in epiboly order
10
12
14
16
18
0
50
100
150

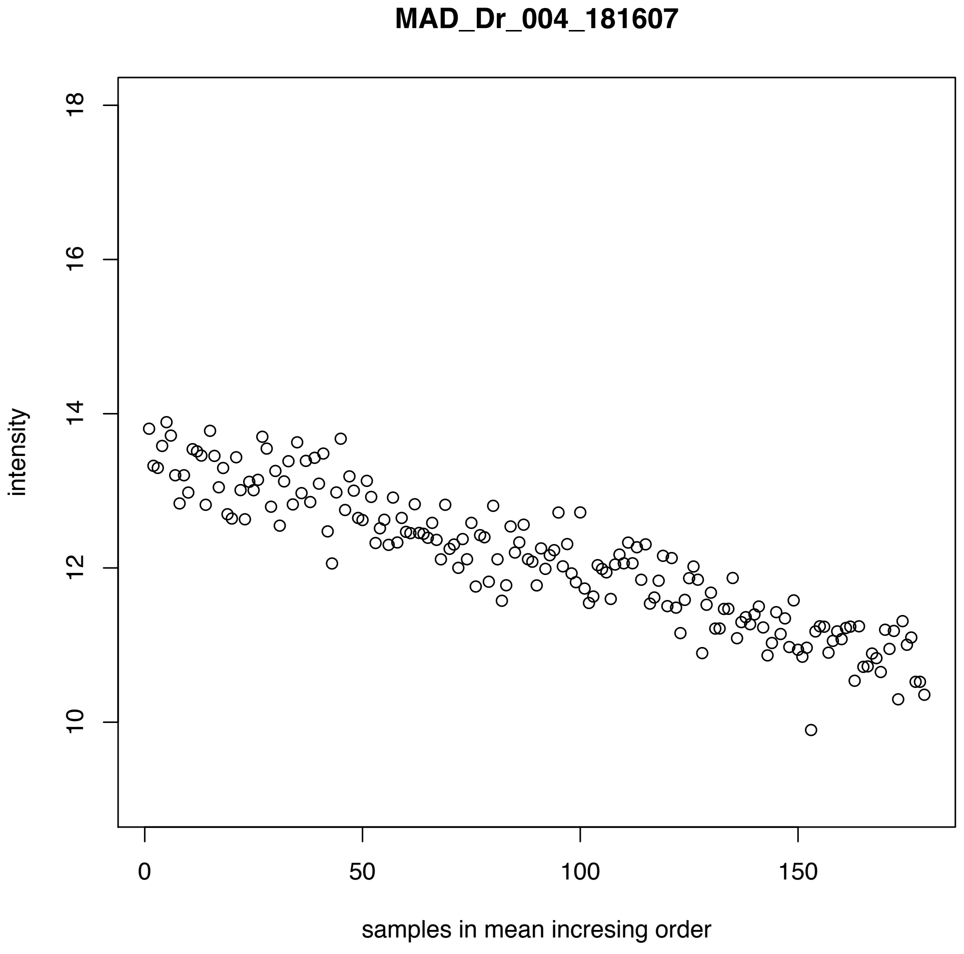
MAD_Dr_004_181607
intensity
samples in mean incresing order
10
12
14
16
18
0
50
100
150

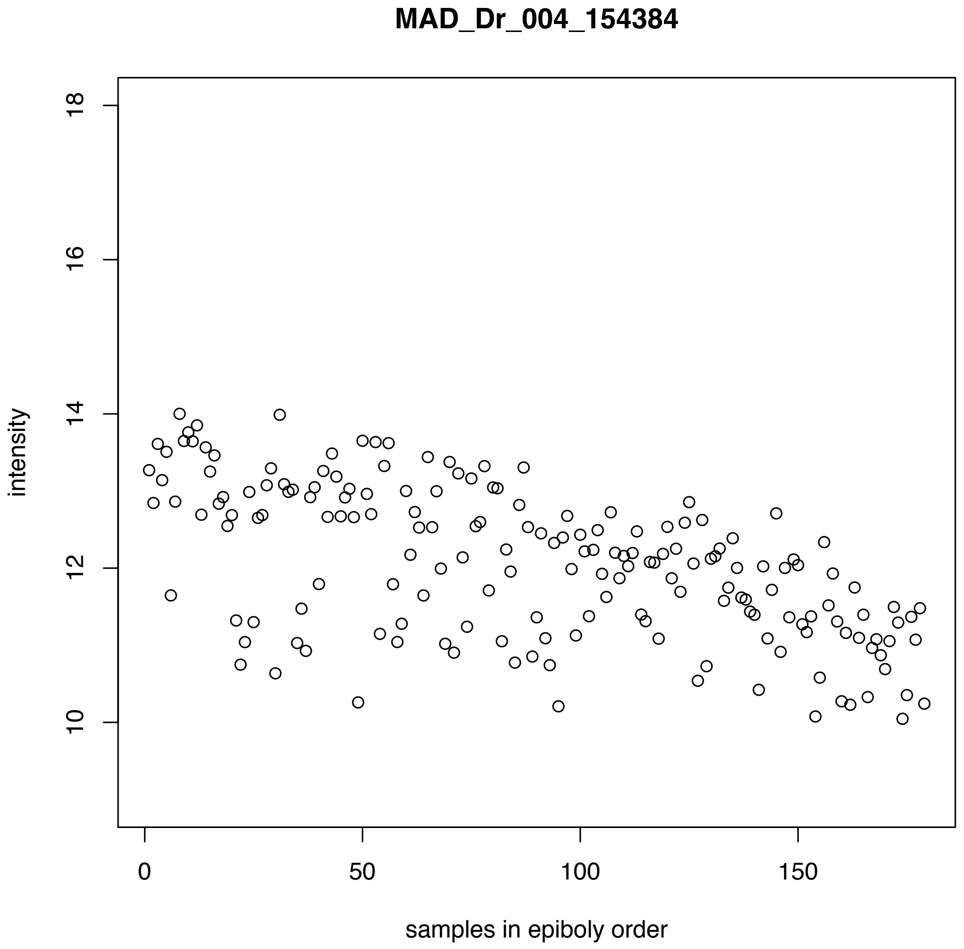
MAD_Dr_004_154384
intensity
10
12
14
16
18
0
50
100
150
samples in epiboly order

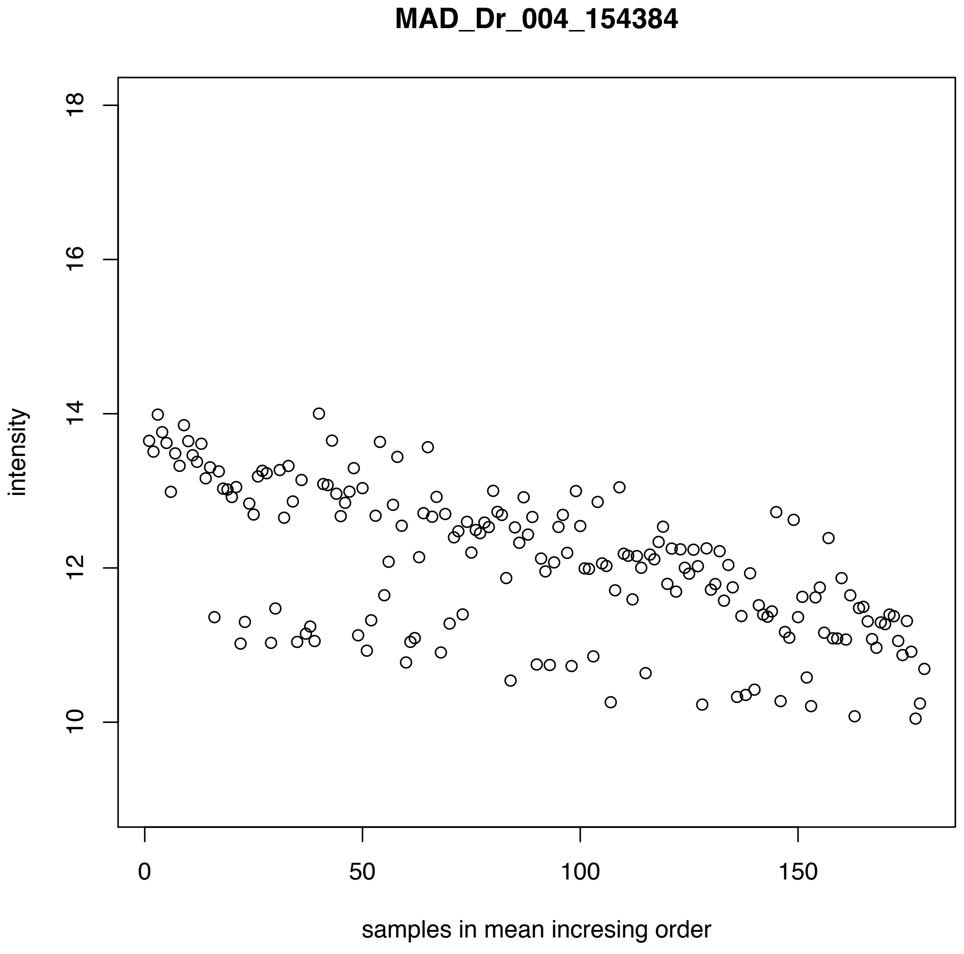
MAD_Dr_004_154384
intensity
10
12
14
16
18
0
50
100
150
samples in mean incresing order

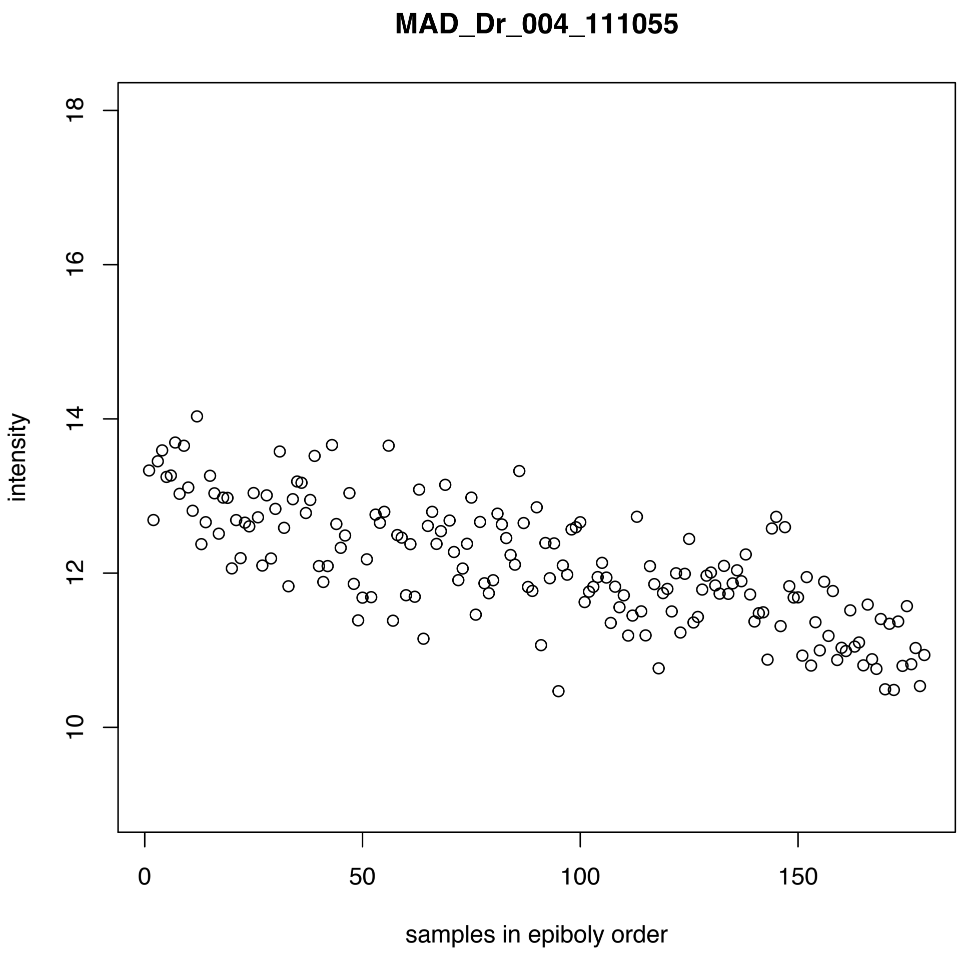
MAD_Dr_004_111055
intensity
10
12
14
16
18
0
50
100
150
samples in epiboly order

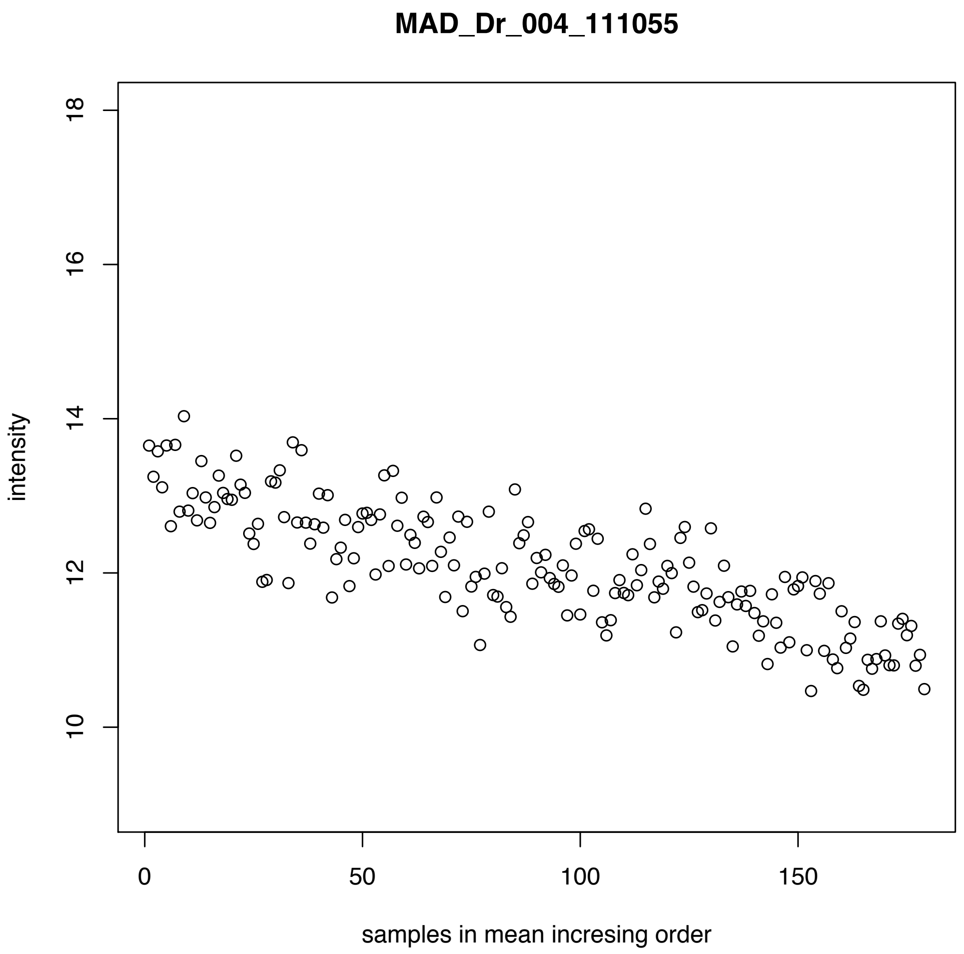
MAD_Dr_004_111055
intensity
10
12
14
16
18
0
50
100
150
samples in mean incresing order

MAD_Dr_004_186764

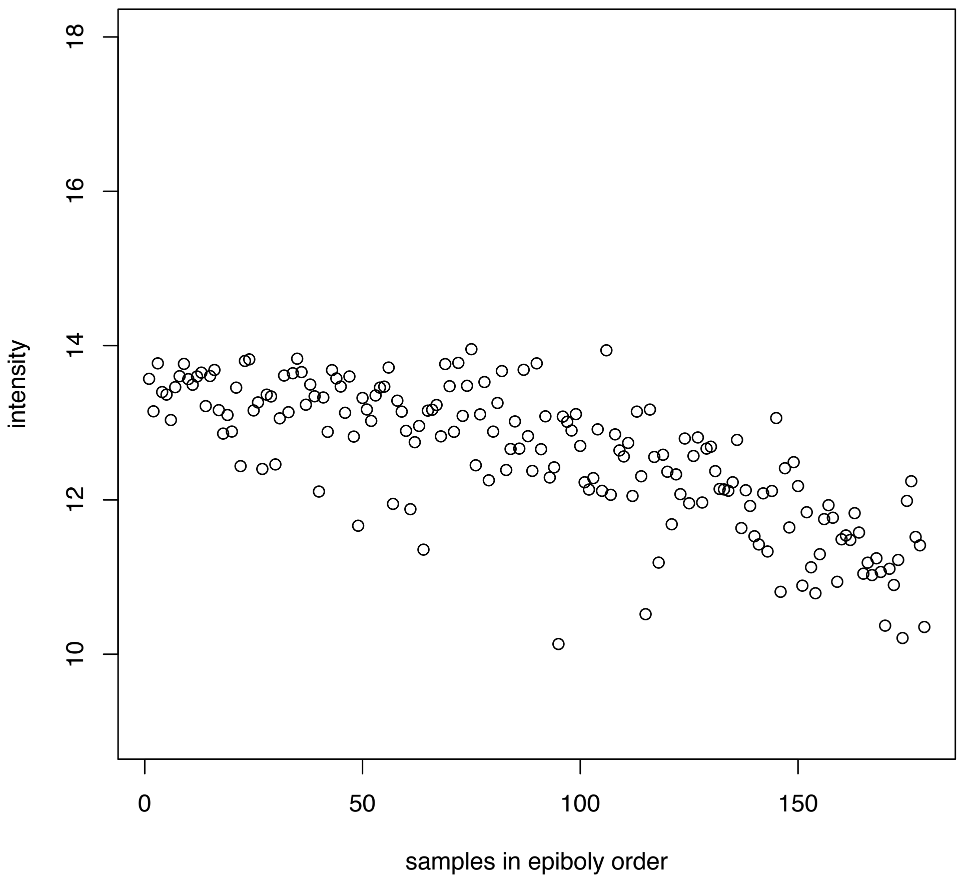

MAD_Dr_004_186764

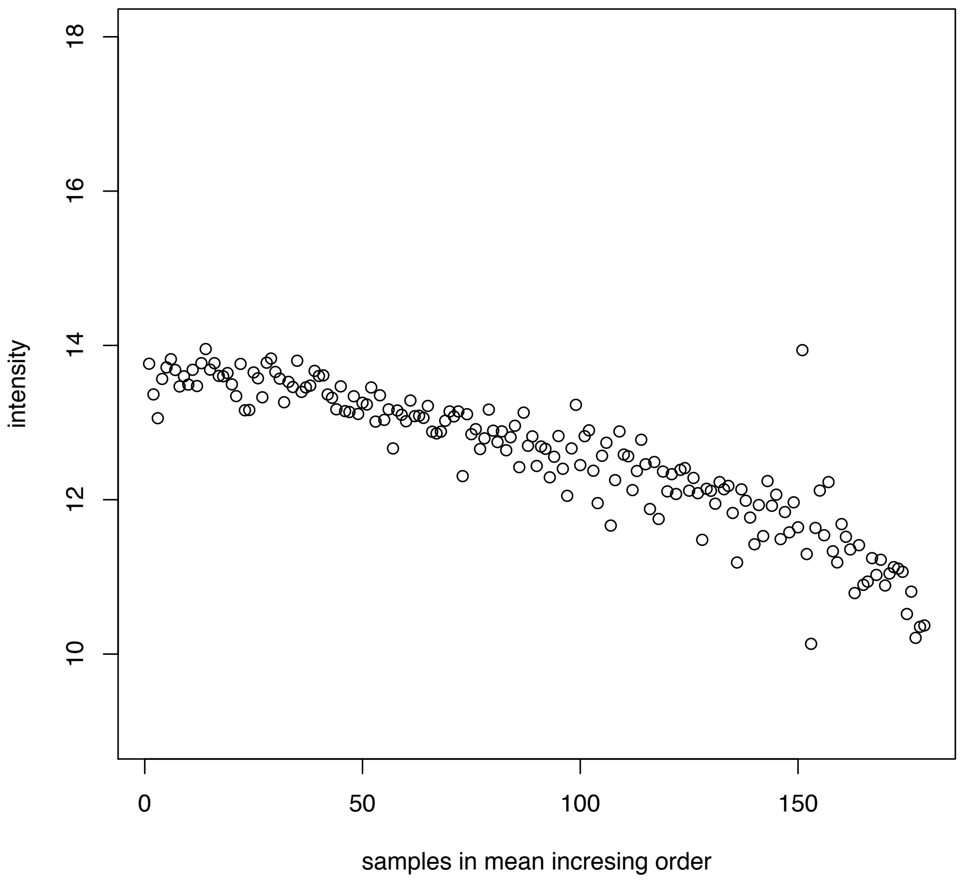

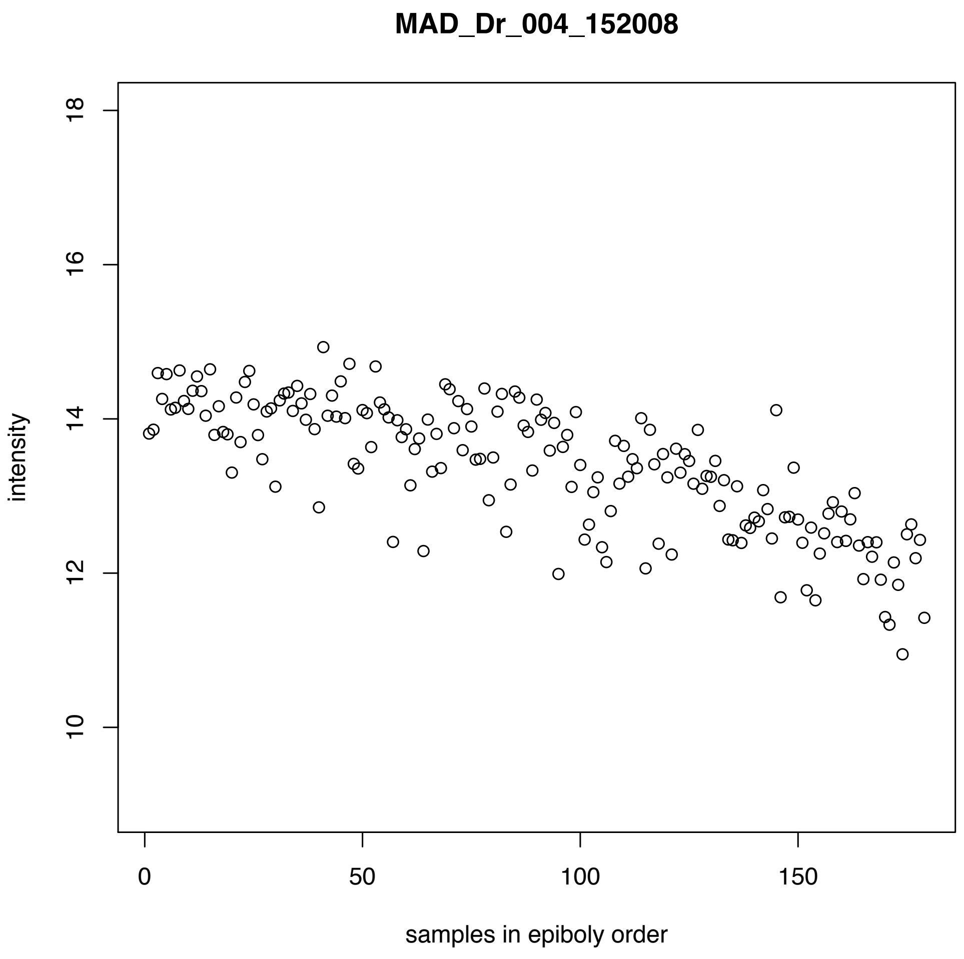
MAD_Dr_004_152008
intensity
10
12
14
16
18
0
50
100
150
samples in epiboly order

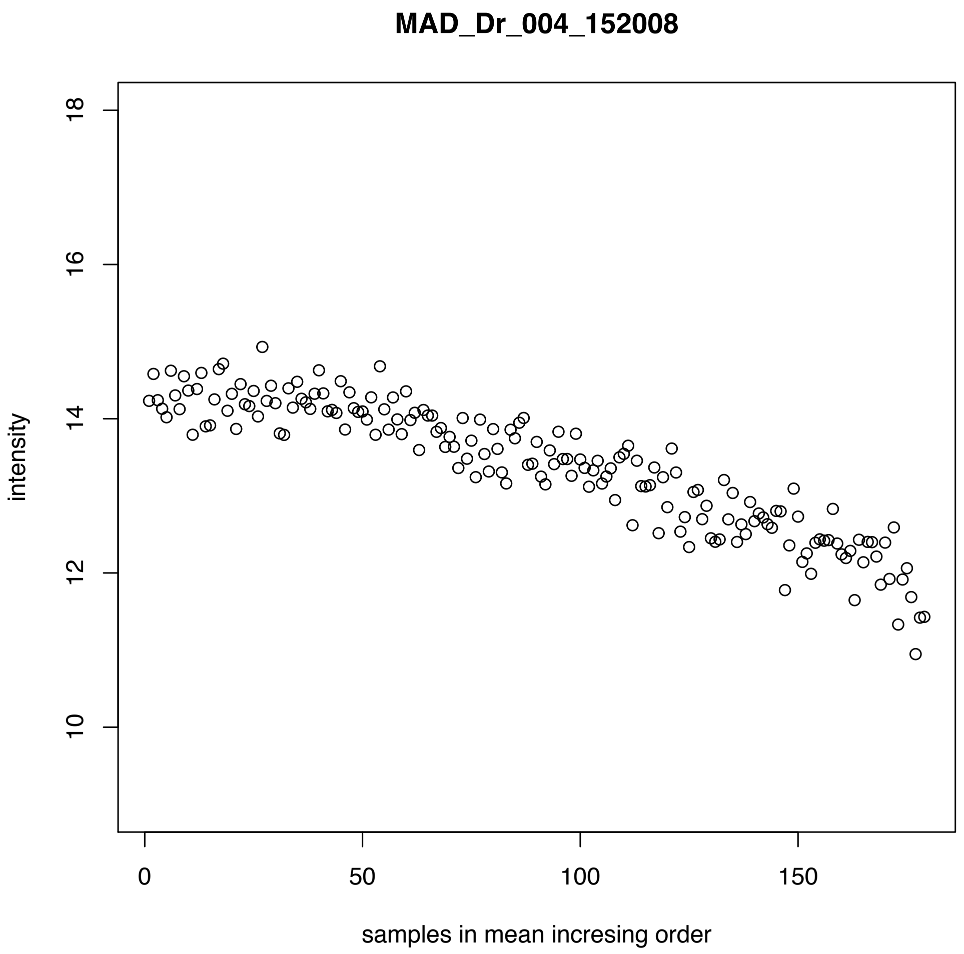
MAD_Dr_004_152008
intensity
10
12
14
16
18
0
50
100
150
samples in mean incresing order

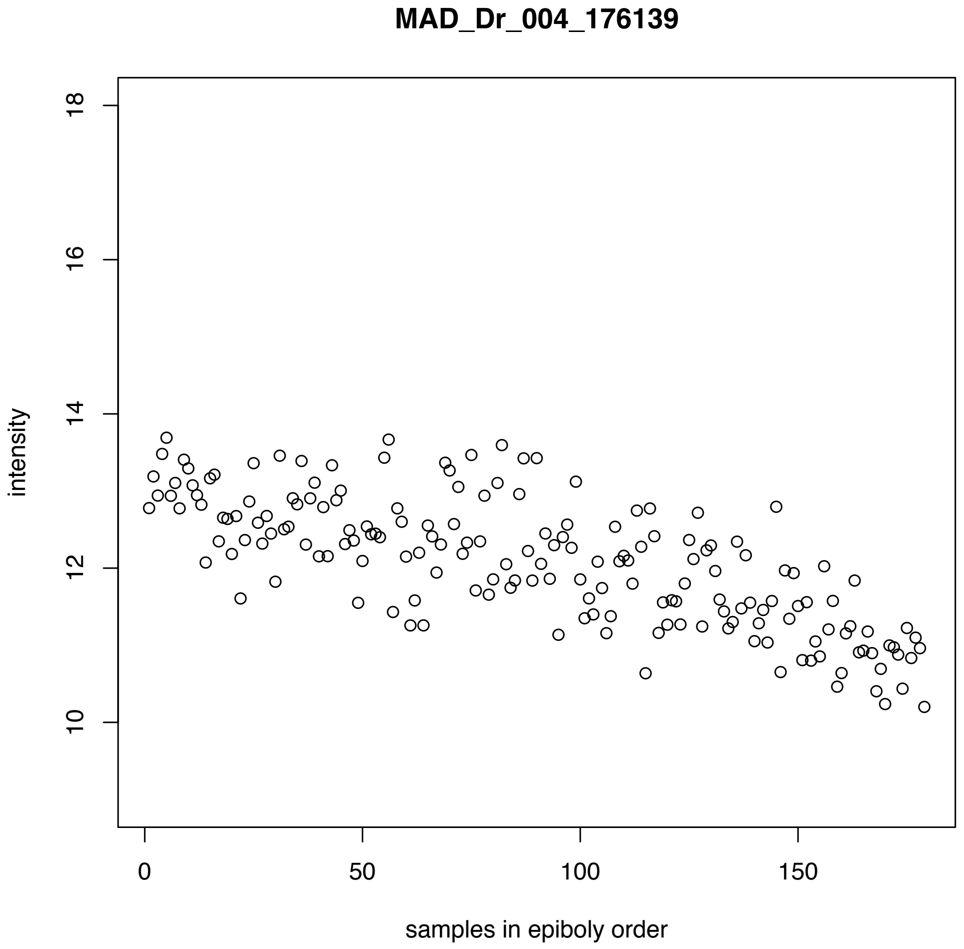
MAD_Dr_004_176139
intensity
samples in epiboly order
10
12
14
16
18
0
50
100
150

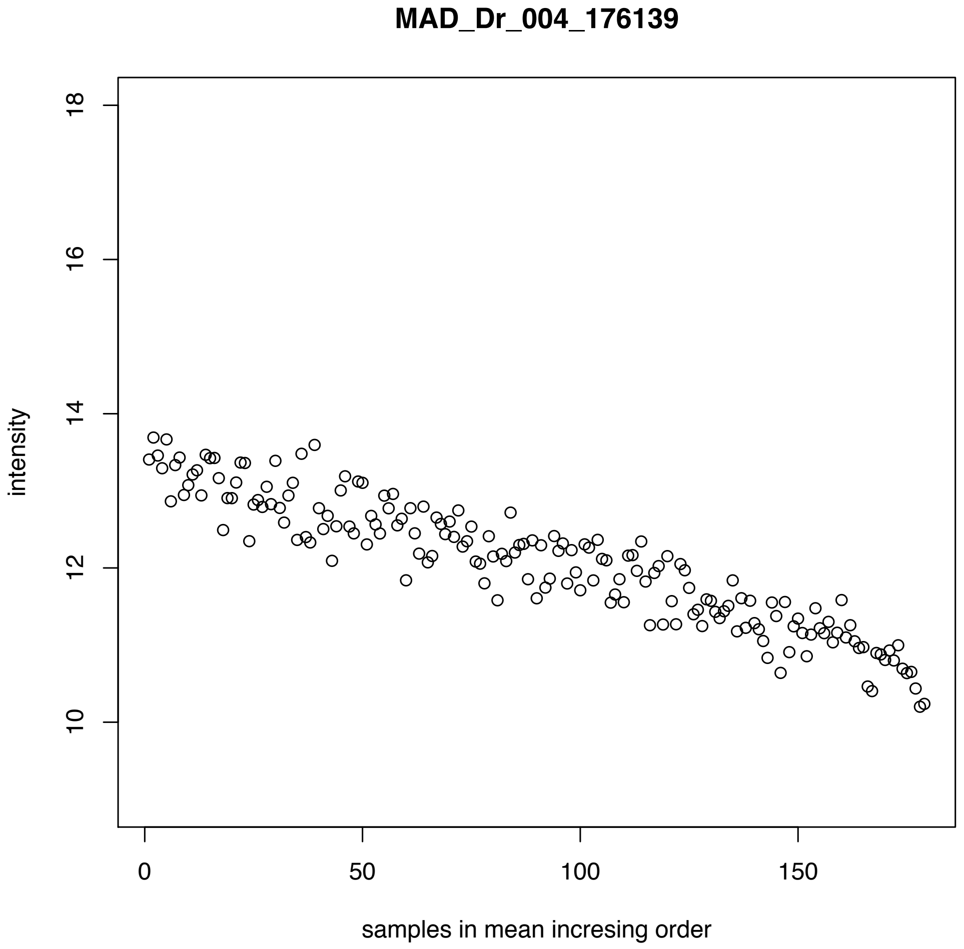
MAD_Dr_004_176139
intensity
samples in mean incresing order
10
12
14
16
18
0
50
100
150

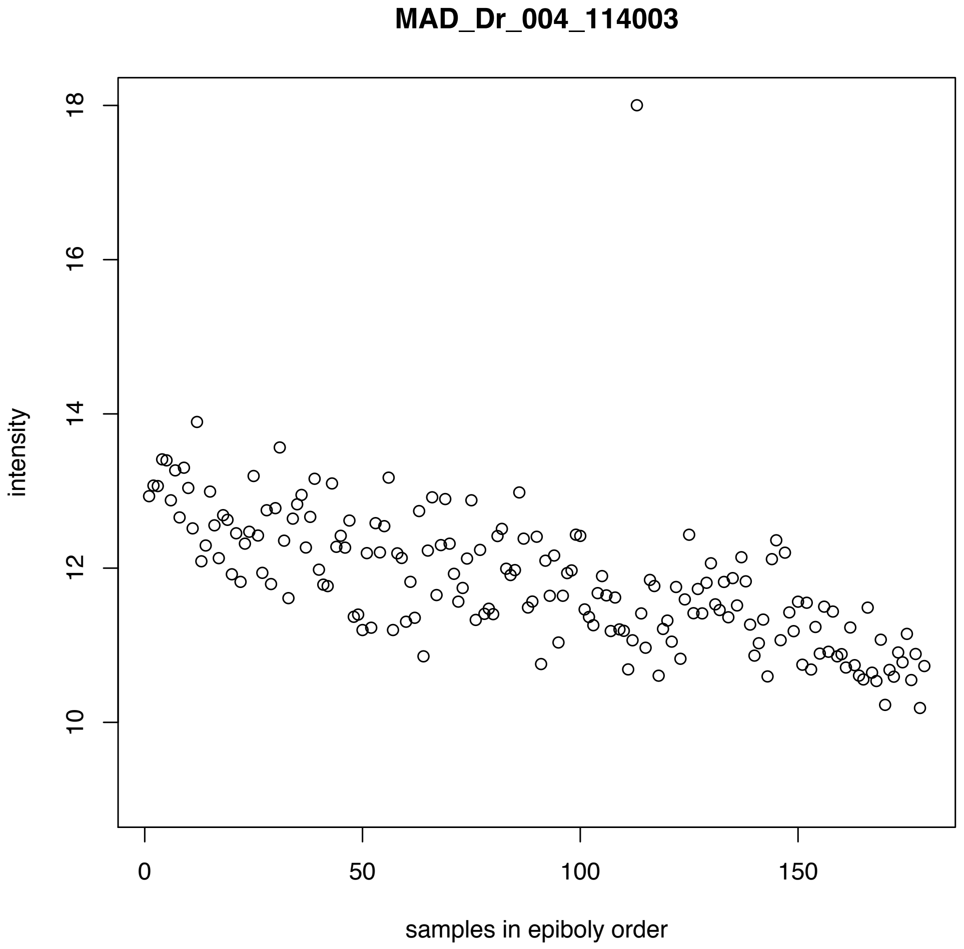
MAD_Dr_004_114003
intensity
10
12
14
16
18
0
50
100
150
samples in epiboly order

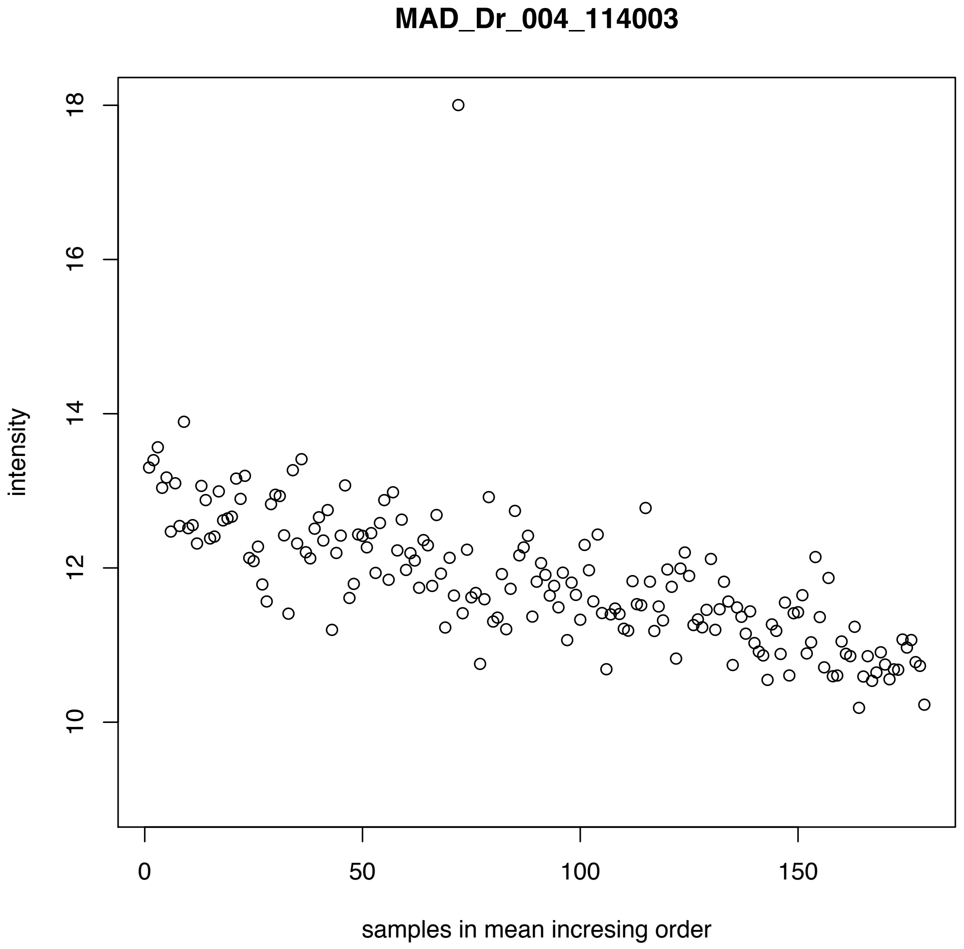
MAD_Dr_004_114003
intensity
10
12
14
16
18
0
50
100
150
samples in mean incresing order

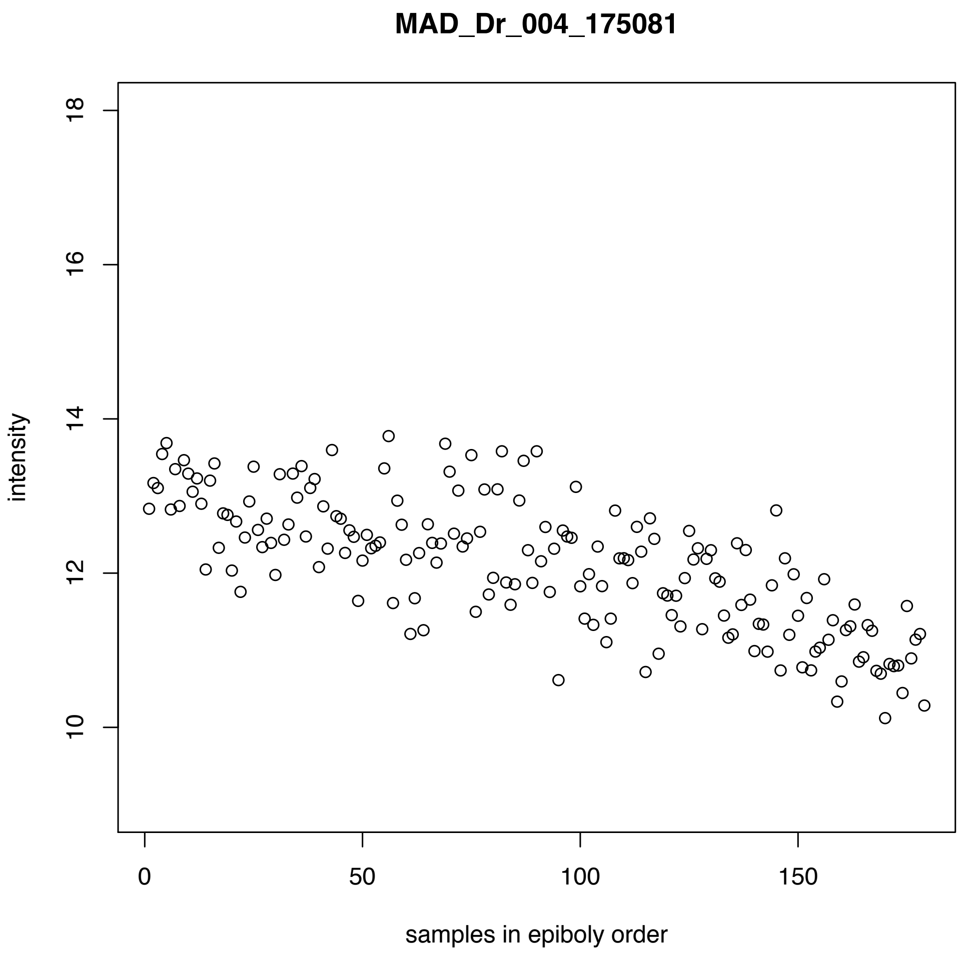
MAD_Dr_004_175081
intensity
samples in epiboly order
10
12
14
16
18
0
50
100
150

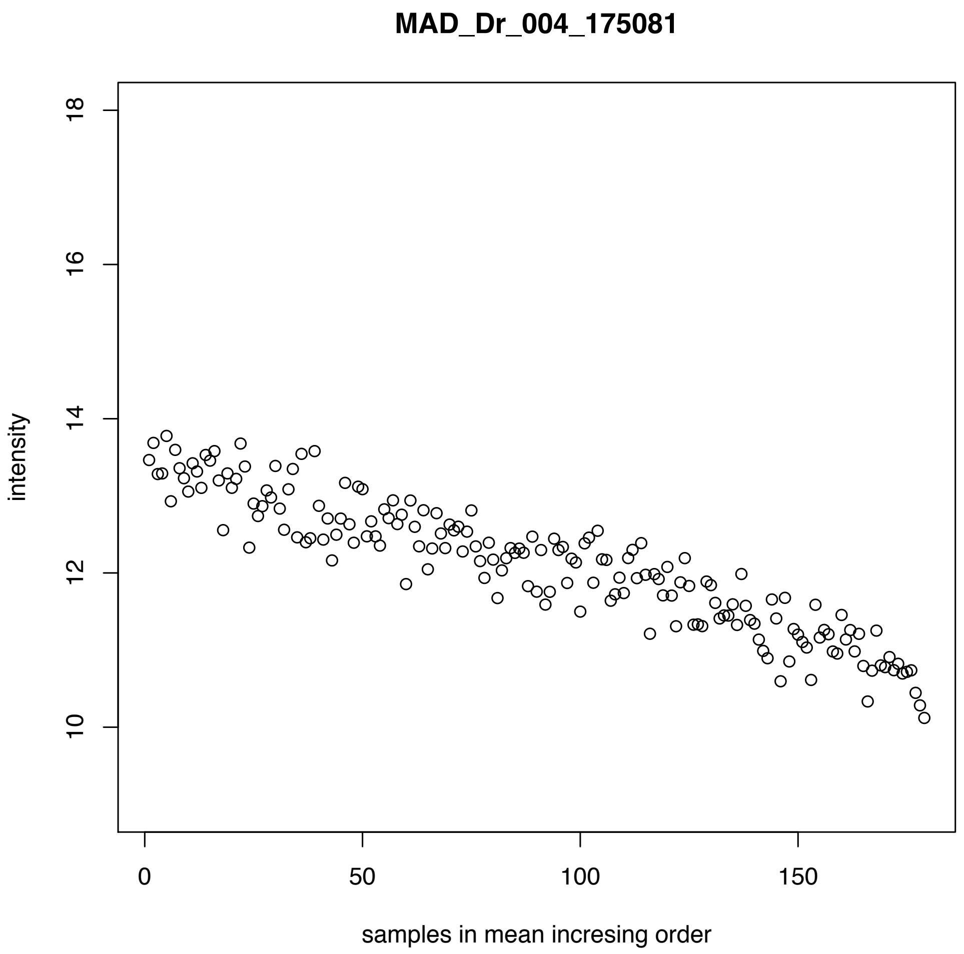
MAD_Dr_004_175081
intensity
samples in mean incresing order
10
12
14
16
18
0
50
100
150

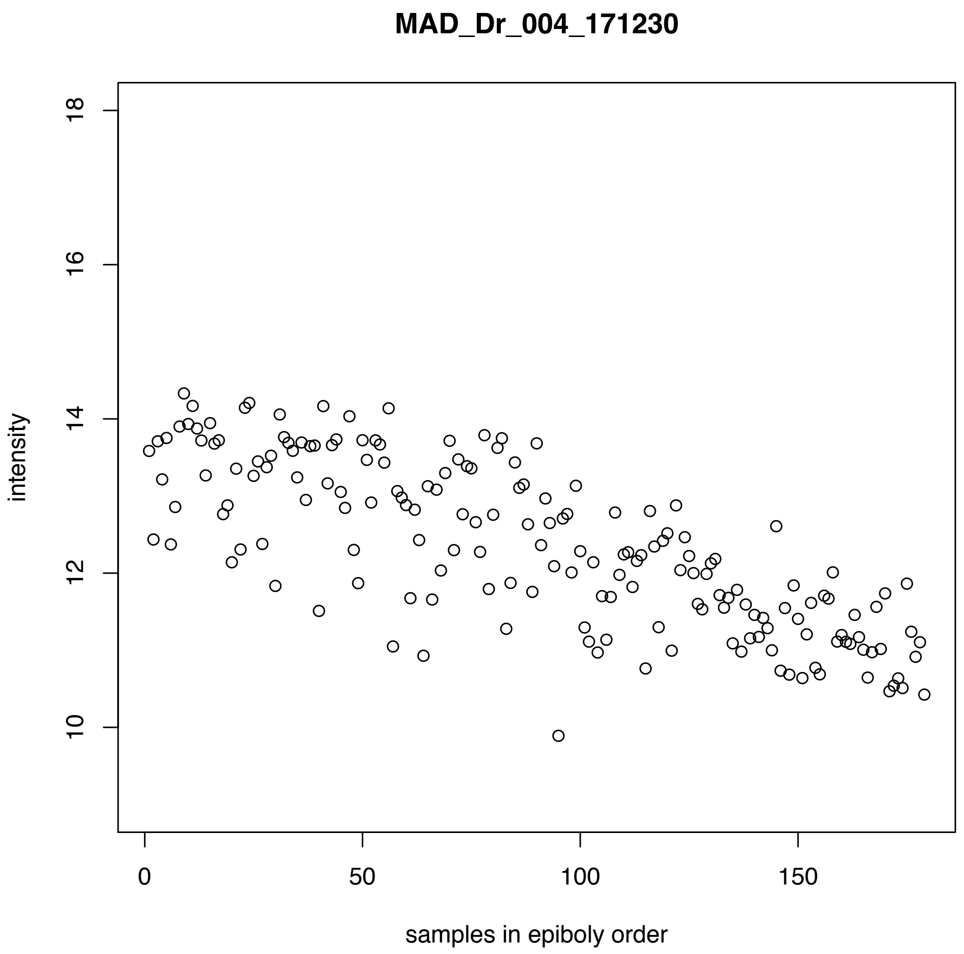
MAD_Dr_004_171230
intensity
samples in epiboly order
10
12
14
16
18
0
50
100
150

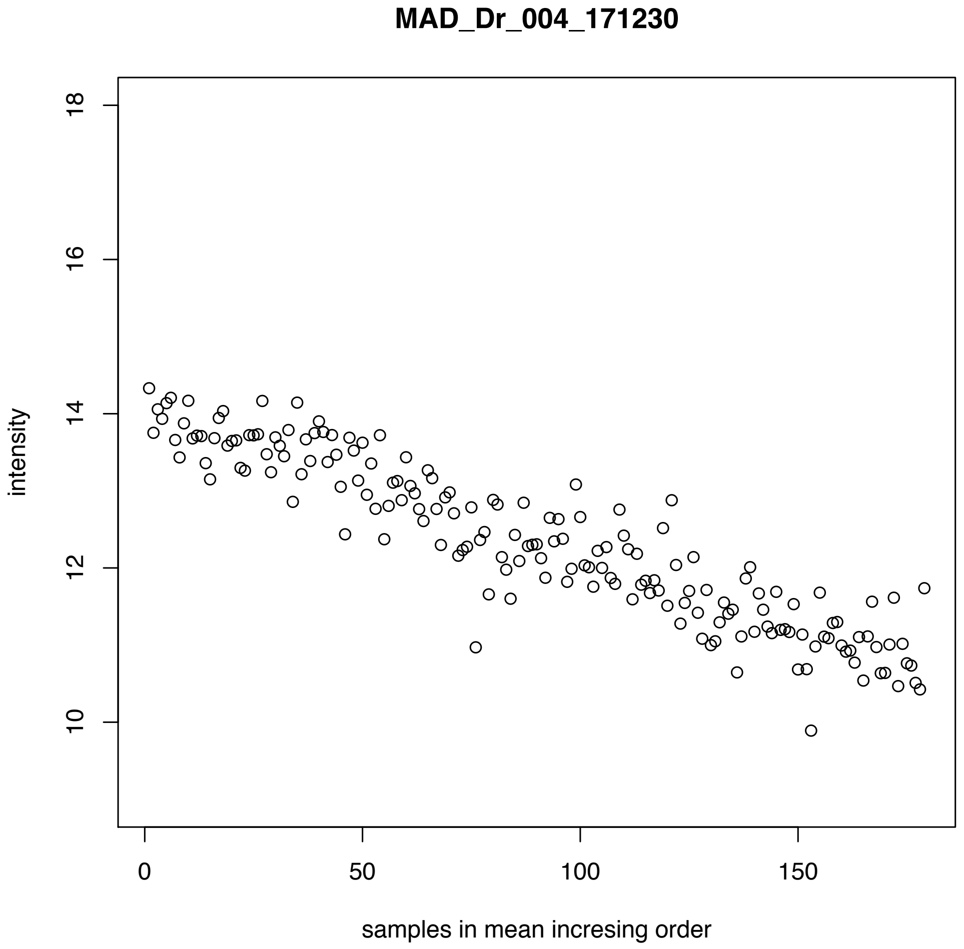
MAD_Dr_004_171230
intensity
samples in mean incresing order
10
12
14
16
18
0
50
100
150

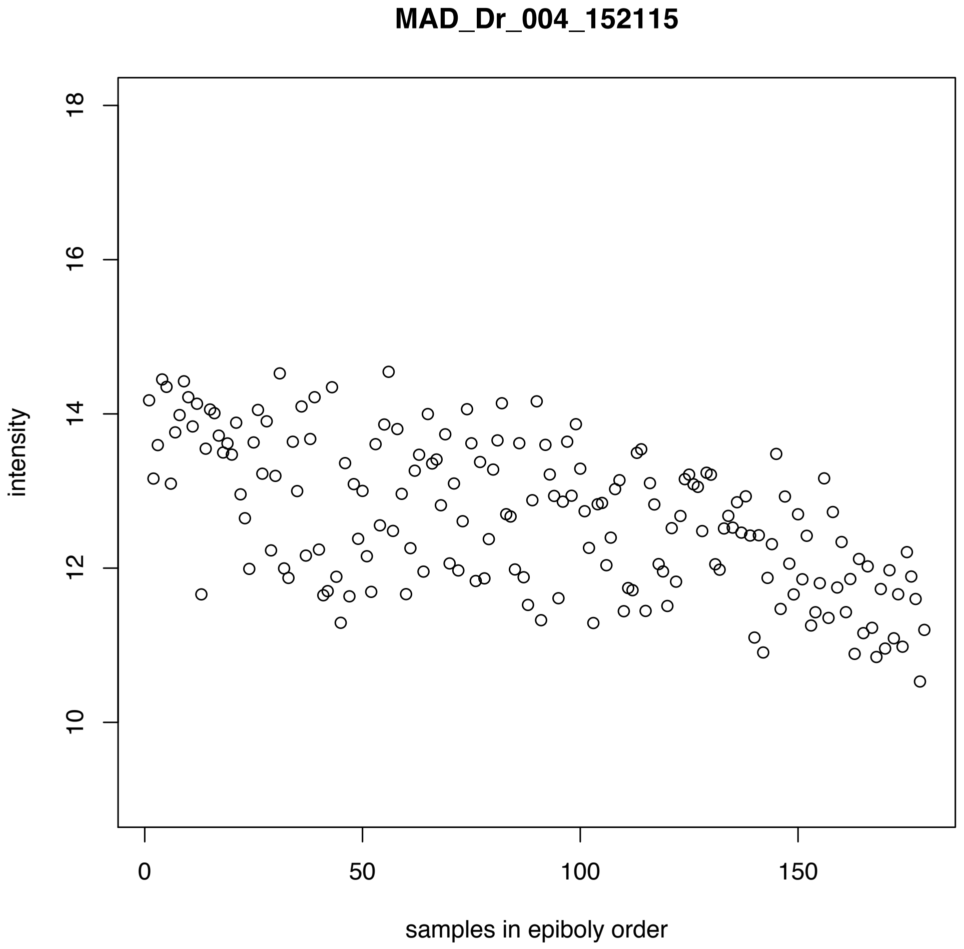
MAD_Dr_004_152115
intensity
10
12
14
16
18
0
50
100
150
samples in epiboly order

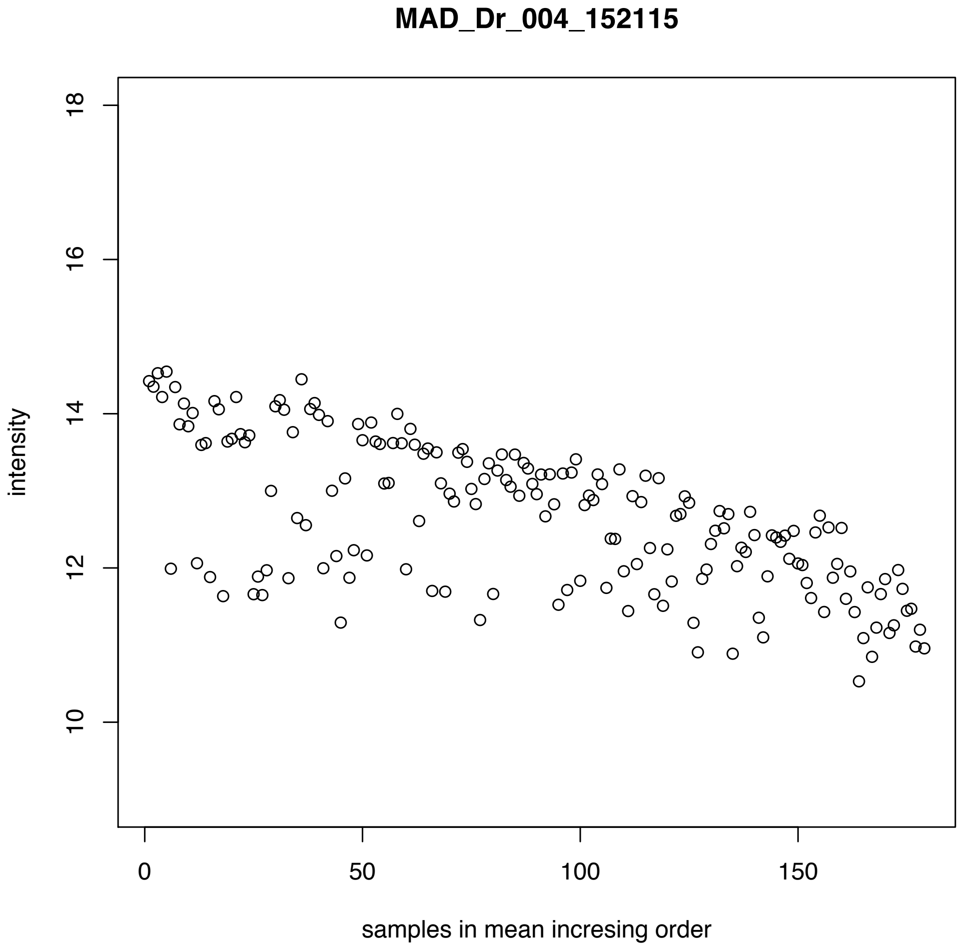
MAD_Dr_004_152115
intensity
10
12
14
16
18
0
50
100
150
samples in mean incresing order

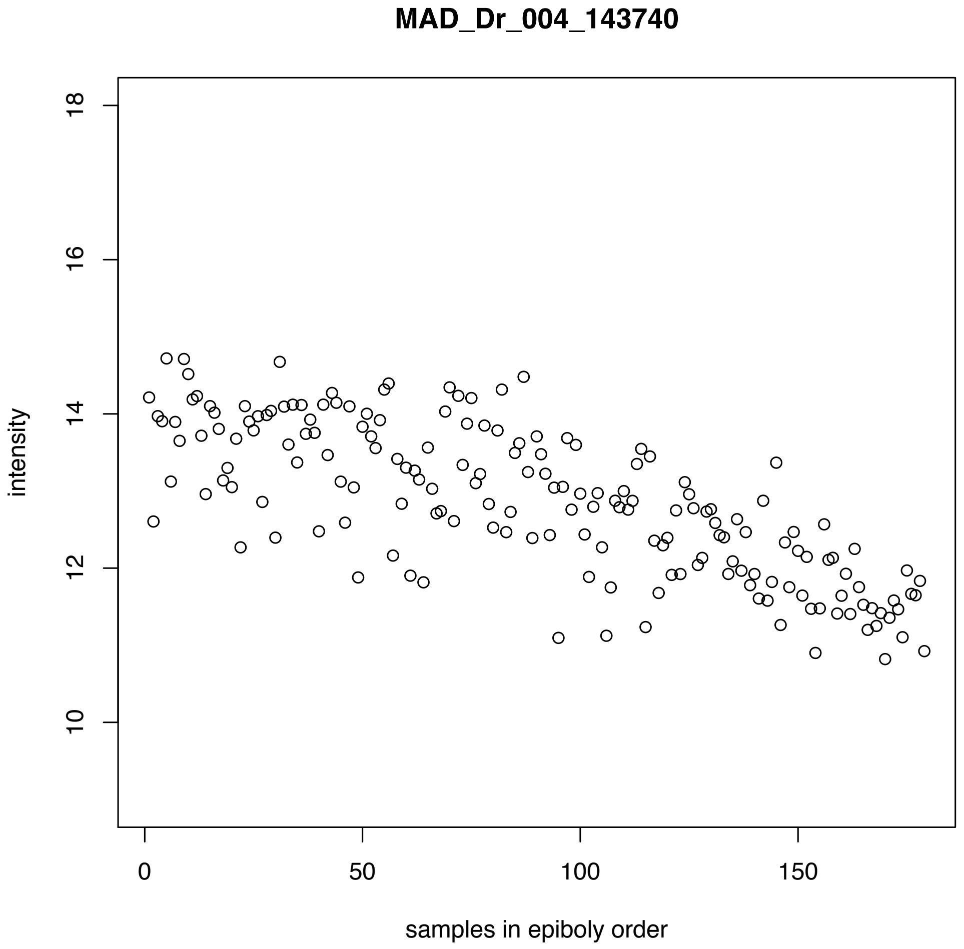
MAD_Dr_004_143740
intensity
samples in epiboly order
0
50
100
150
10
12
14
16
18

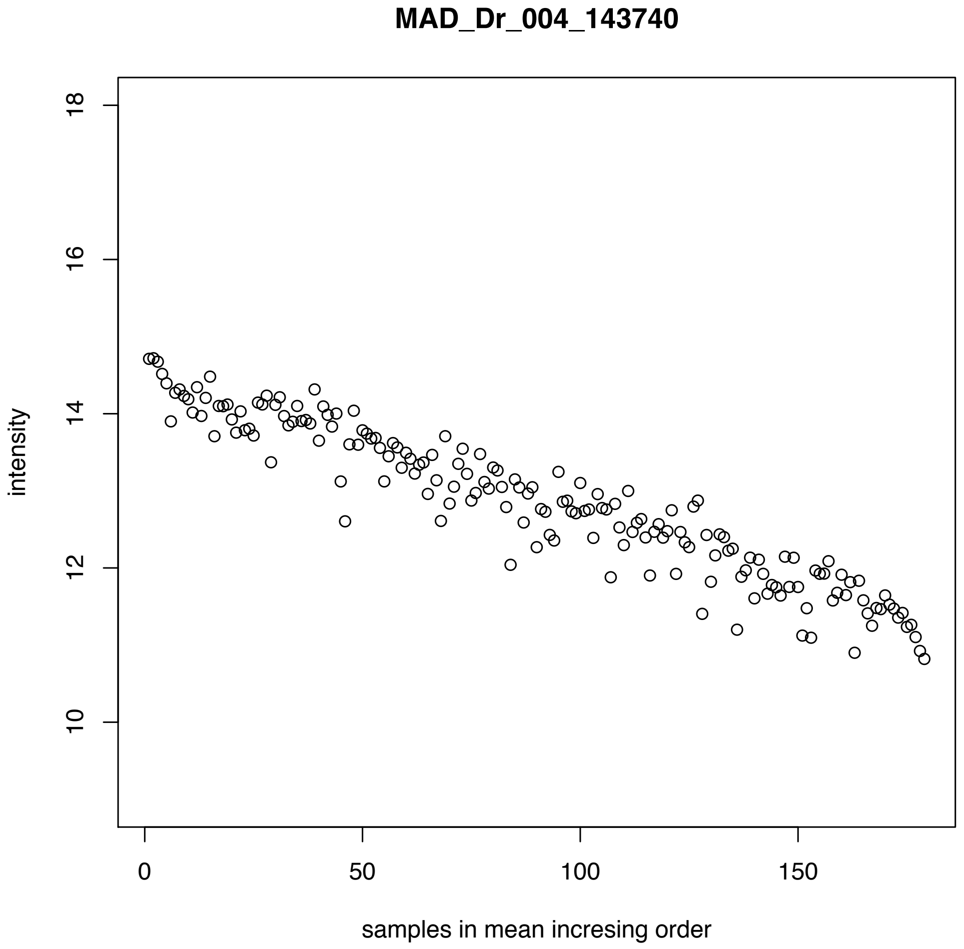
MAD_Dr_004_143740
intensity
samples in mean incresing order
0
50
100
150
10
12
14
16
18

MAD_Dr_004_161893

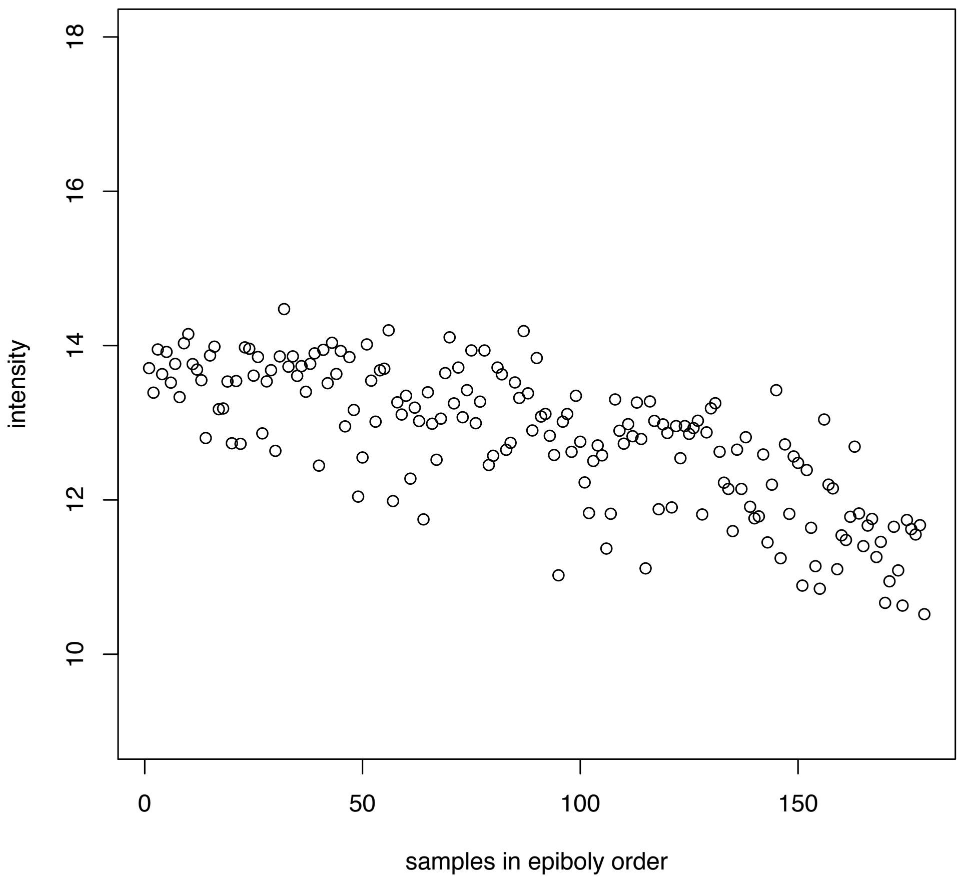

MAD_Dr_004_161893

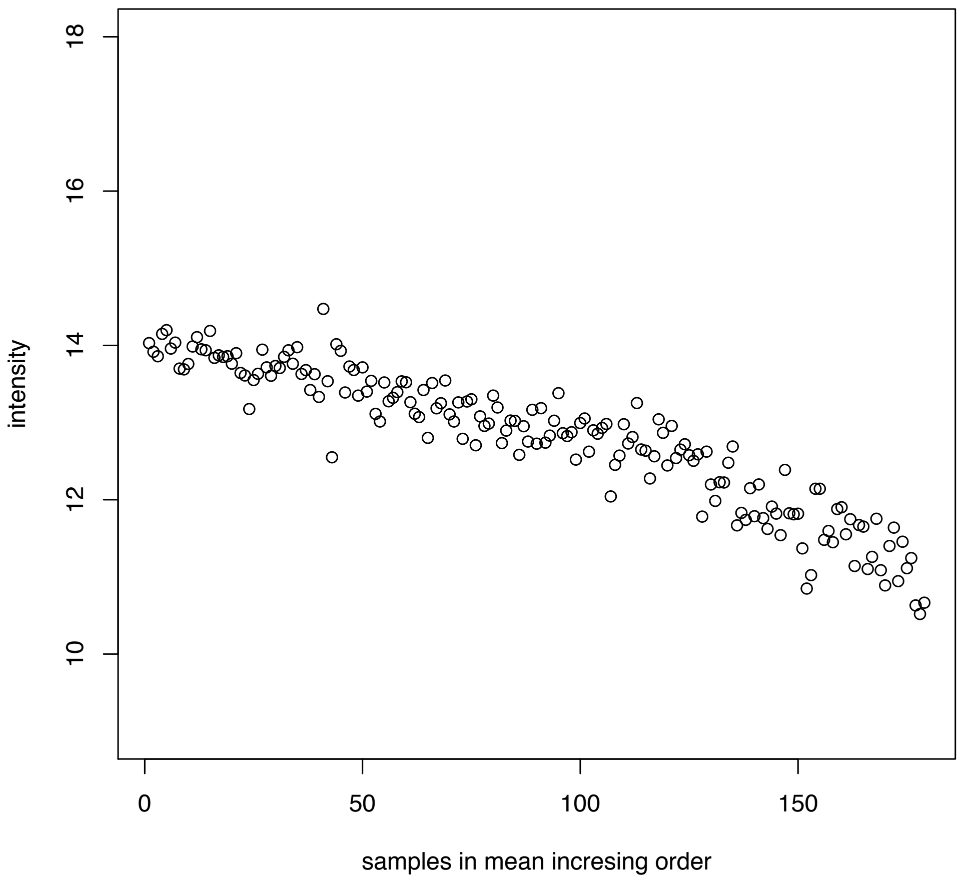

MAD_Dr_004_161878

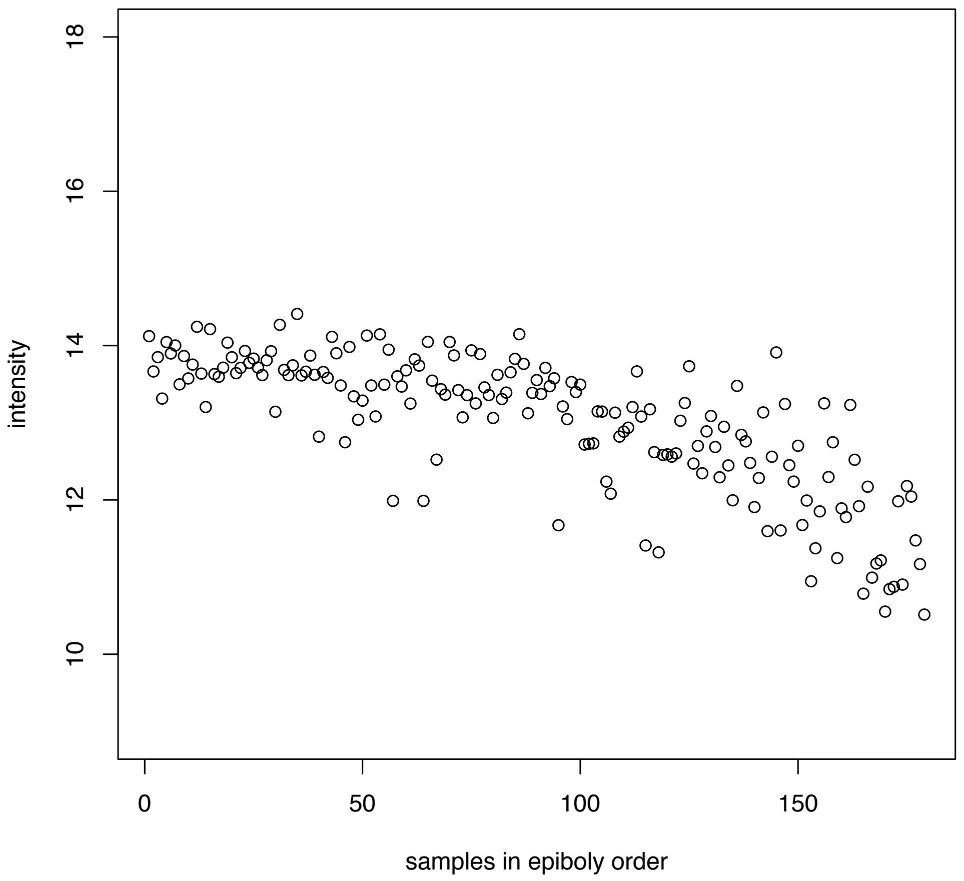

MAD_Dr_004_161878

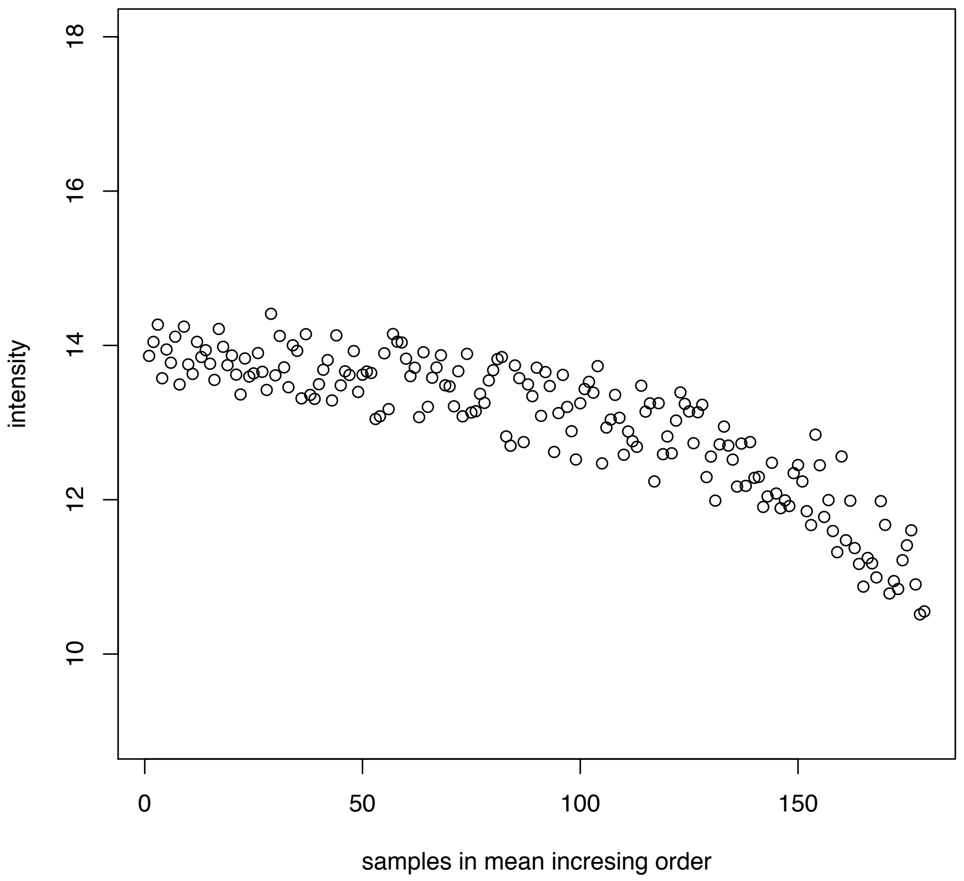

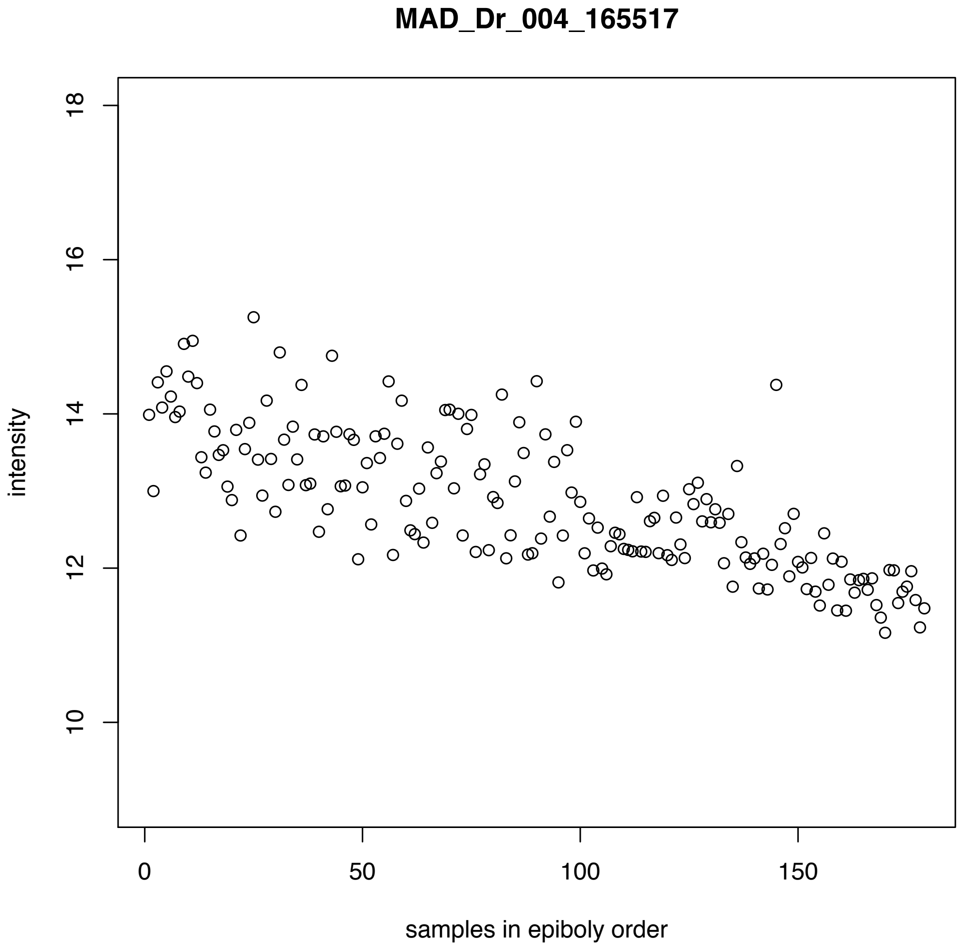
MAD_Dr_004_165517
intensity
10
12
14
16
18
0
50
100
150
samples in epiboly order

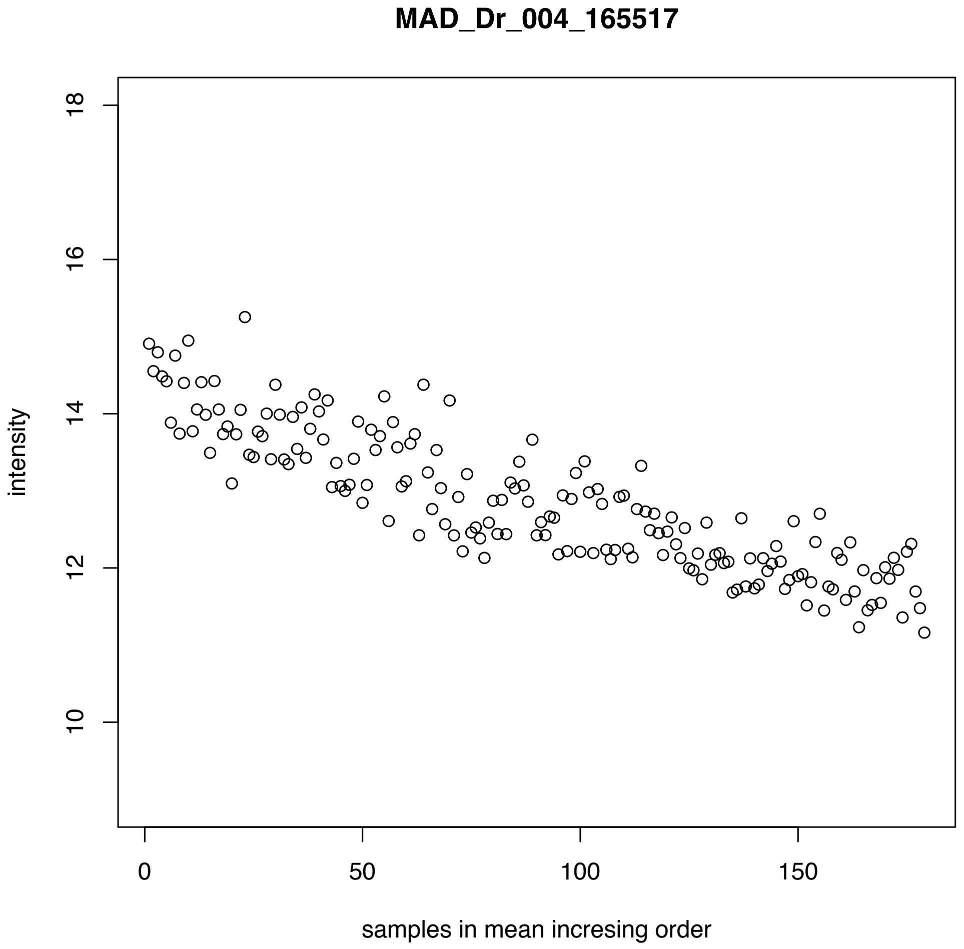
MAD_Dr_004_165517
intensity
10
12
14
16
18
0
50
100
150
samples in mean incresing order

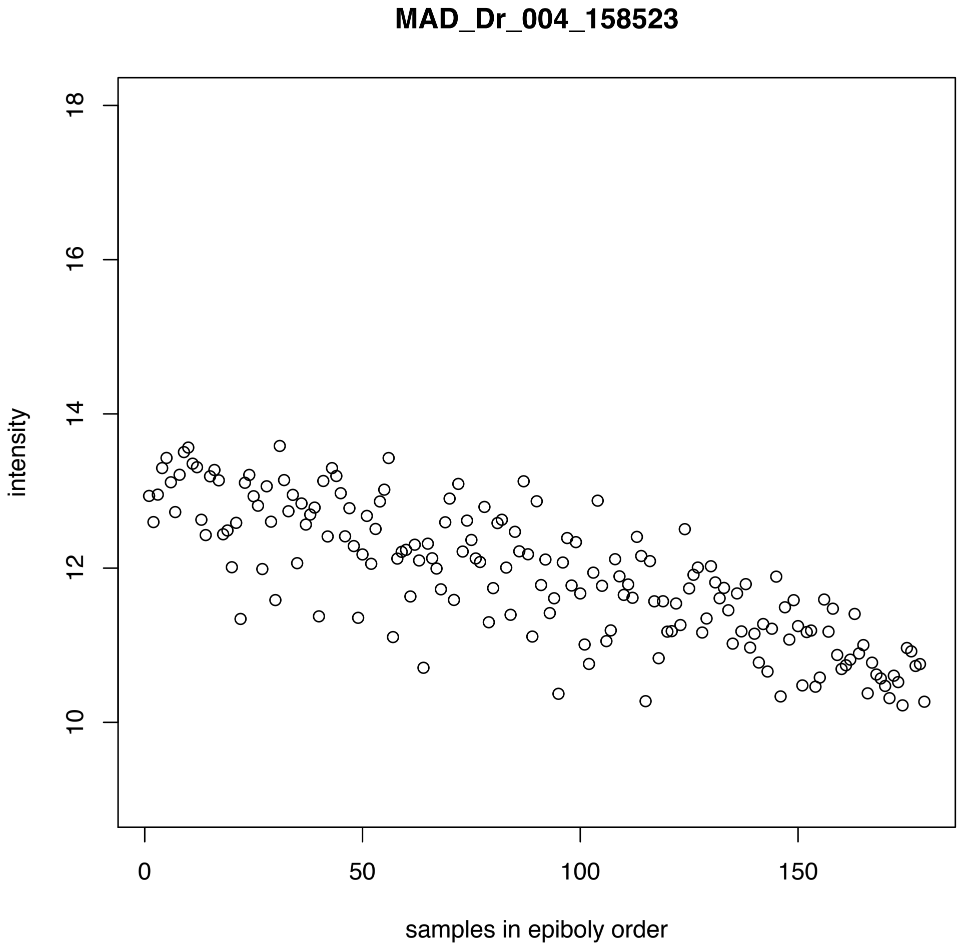
MAD_Dr_004_158523
intensity
samples in epiboly order
10
12
14
16
18
0
50
100
150

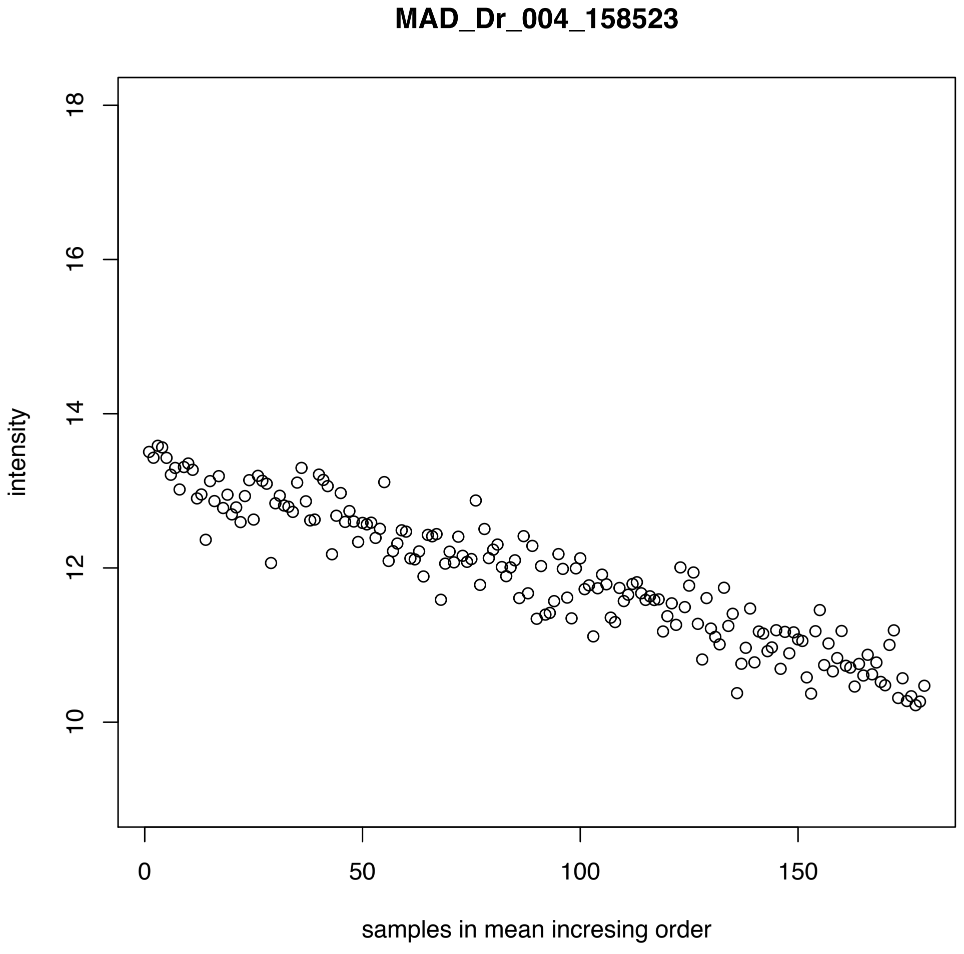
MAD_Dr_004_158523
intensity
samples in mean incresing order
10
12
14
16
18
0
50
100
150

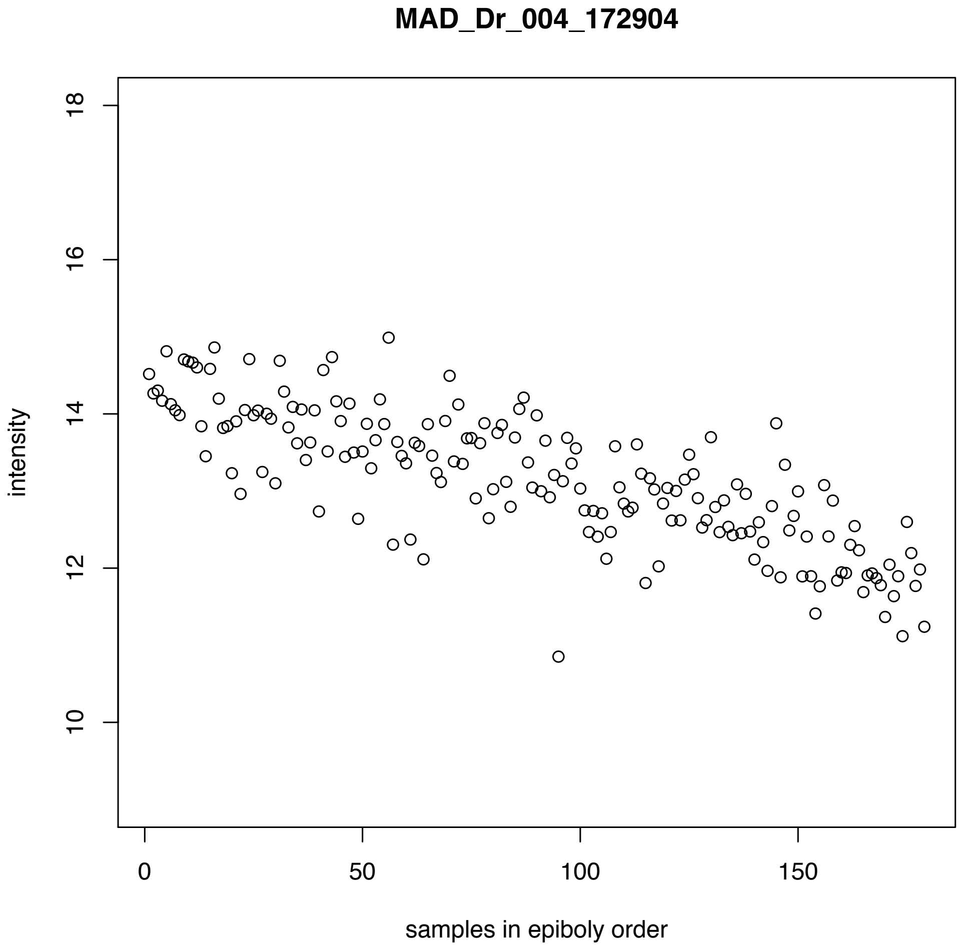
MAD_Dr_004_172904
intensity
10
12
14
16
18
0
50
100
150
samples in epiboly order

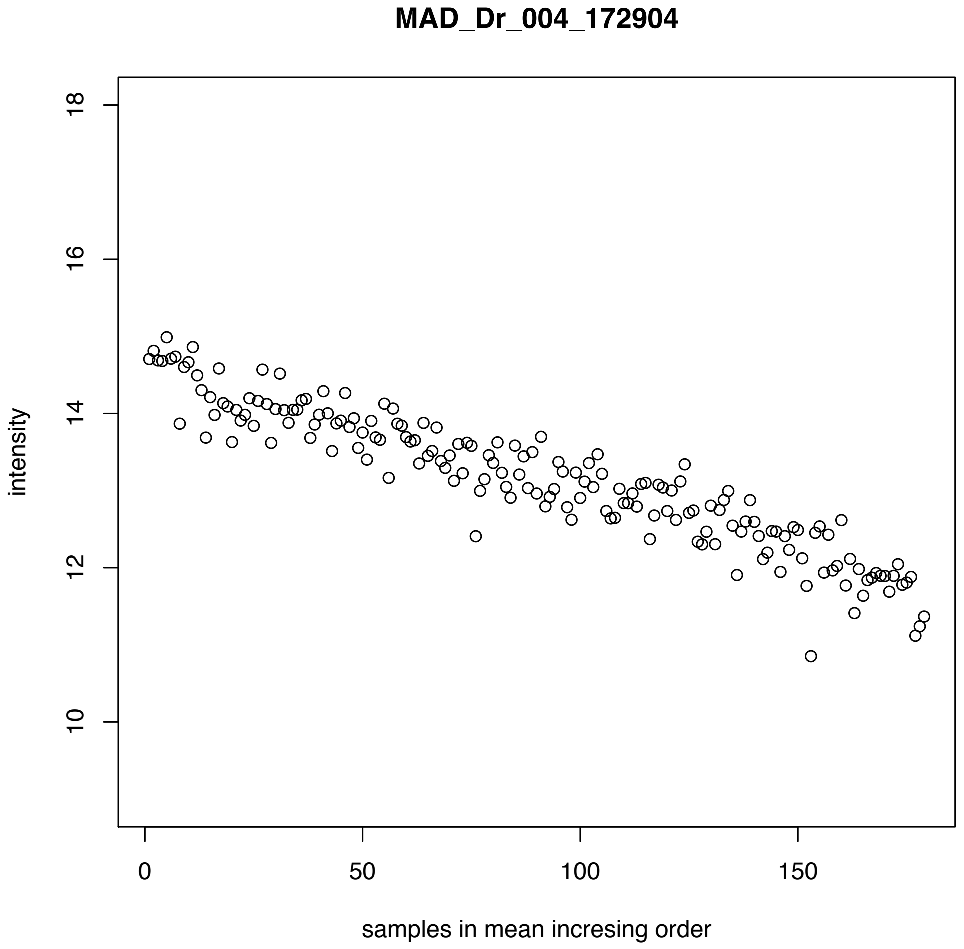
MAD_Dr_004_172904
intensity
10
12
14
16
18
0
50
100
150
samples in mean incresing order

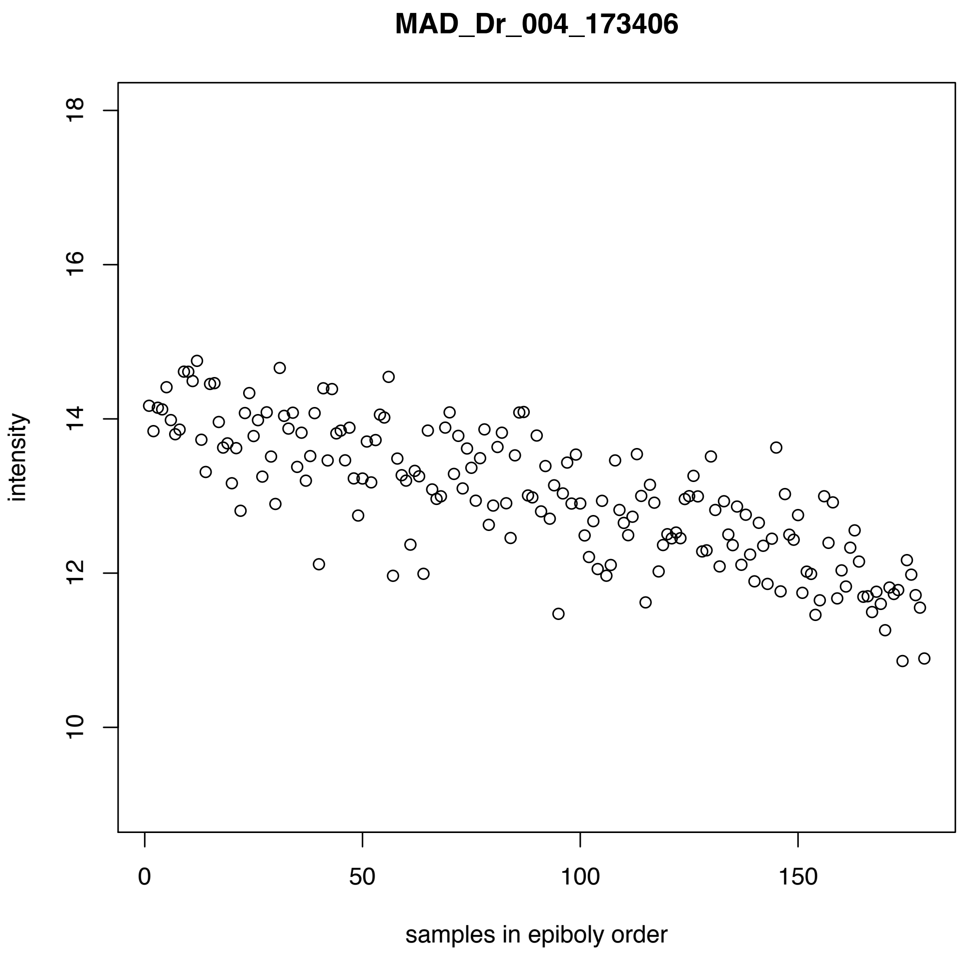

MAD_Dr_004_173406
intensity
10
12
14
16
18
0
50
100
150
samples in epiboly order

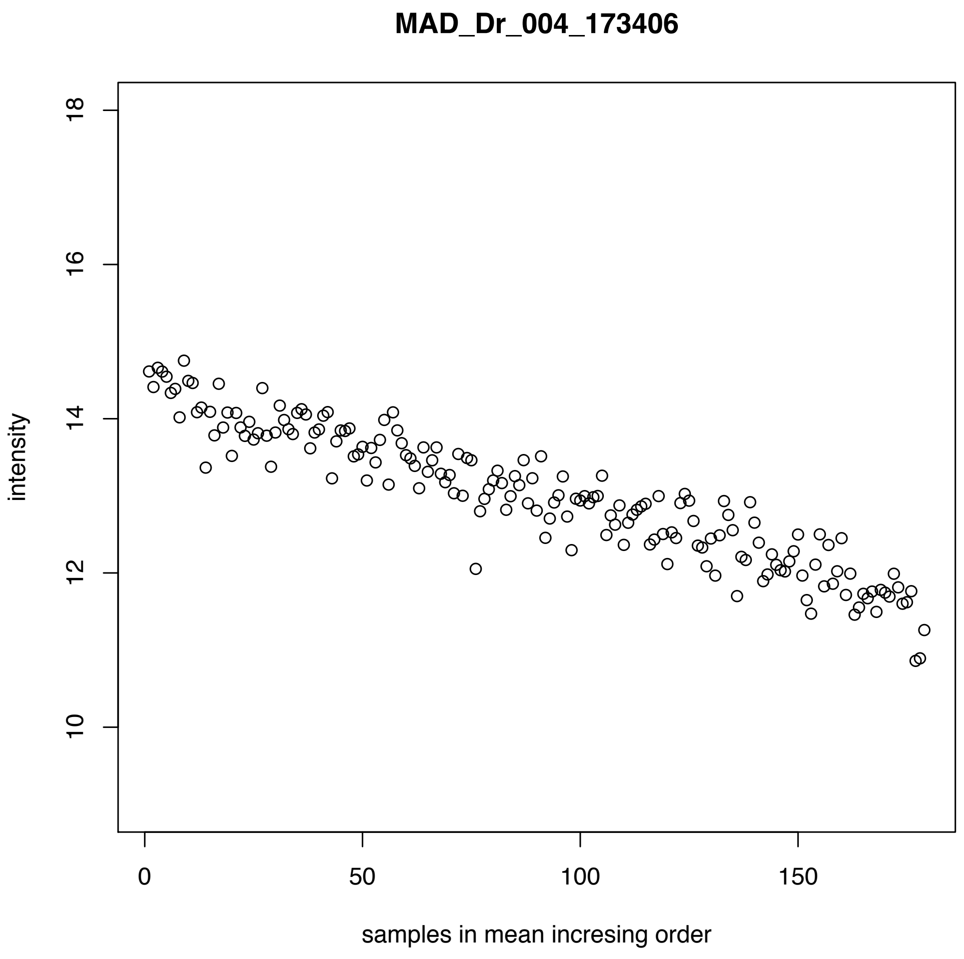

MAD_Dr_004_173406
intensity
10
12
14
16
18
0
50
100
150
samples in mean incresing order

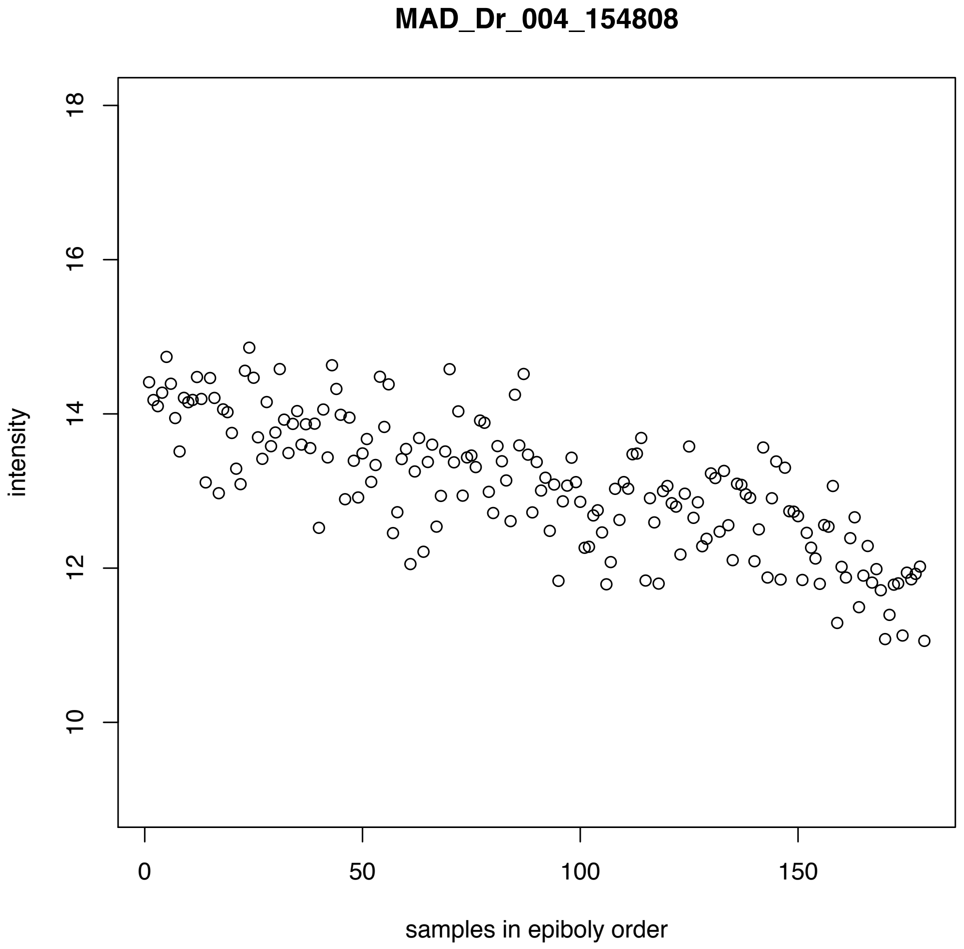
MAD_Dr_004_154808
intensity
10
12
14
16
18
0
50
100
150
samples in epiboly order

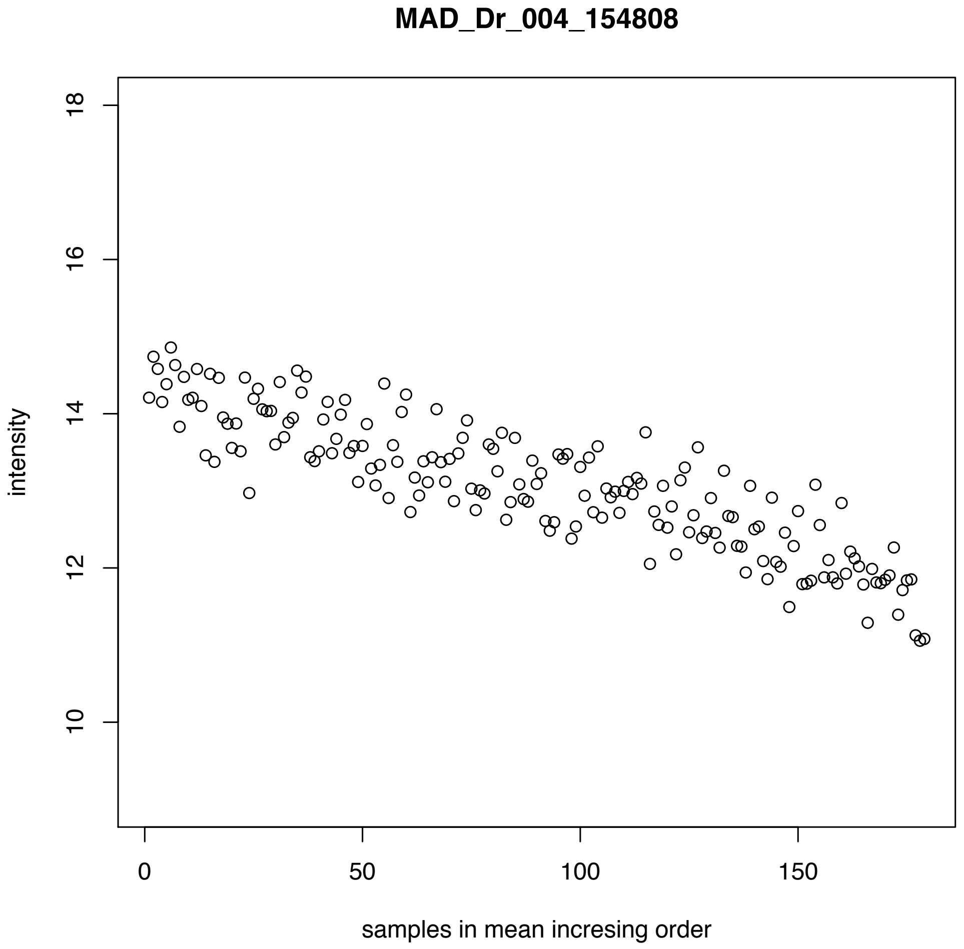
MAD_Dr_004_154808
intensity
10
12
14
16
18
0
50
100
150
samples in mean incresing order

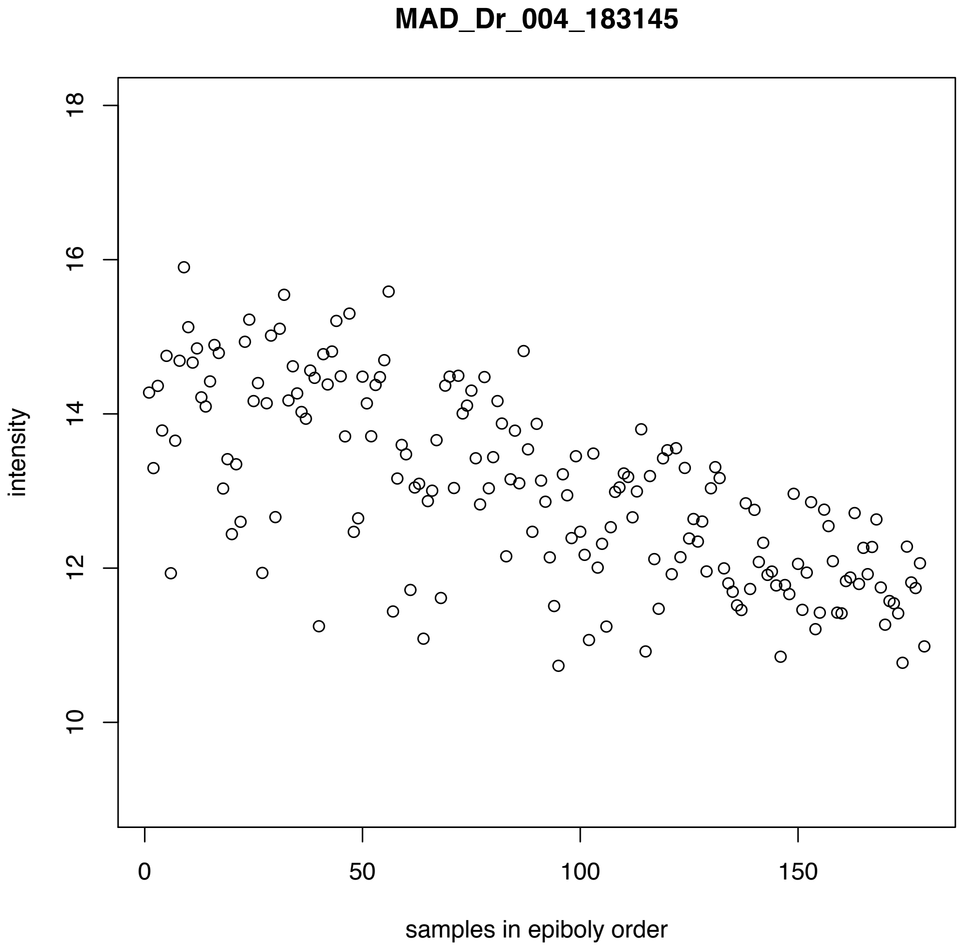
MAD_Dr_004_183145
intensity
samples in epiboly order
10
12
14
16
18
0
50
100
150

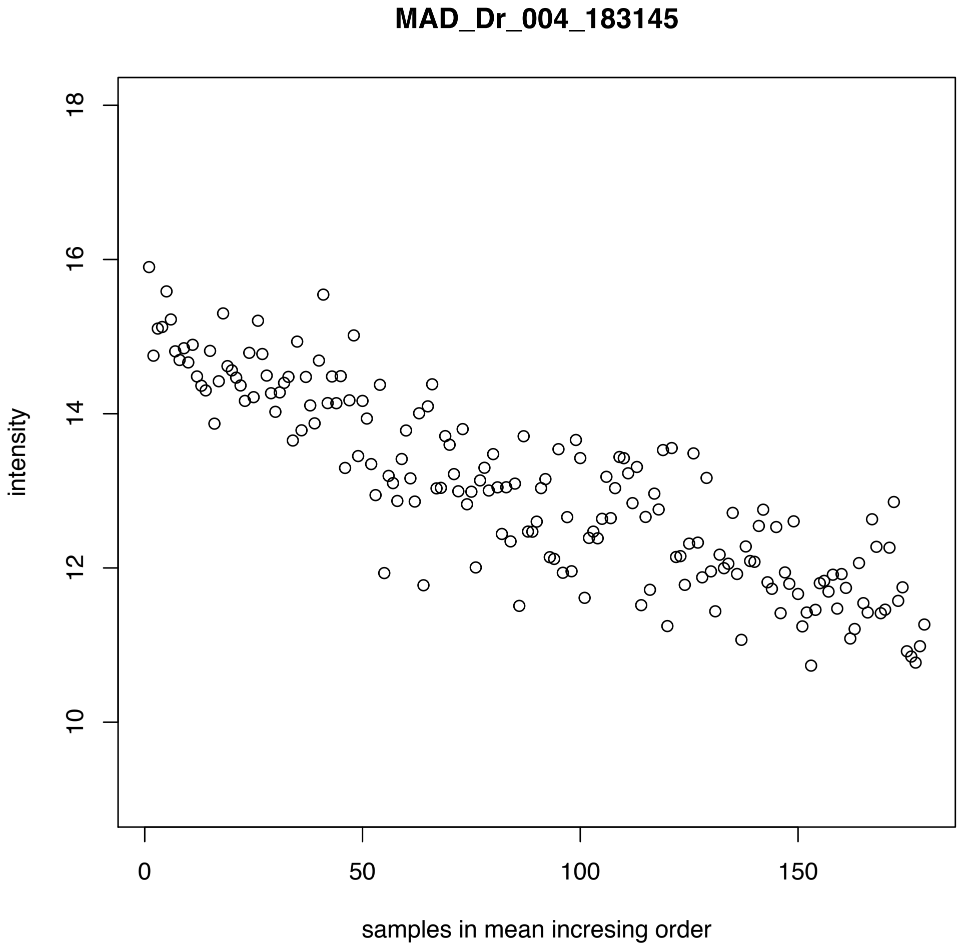
MAD_Dr_004_183145
intensity
samples in mean incresing order
10
12
14
16
18
0
50
100
150

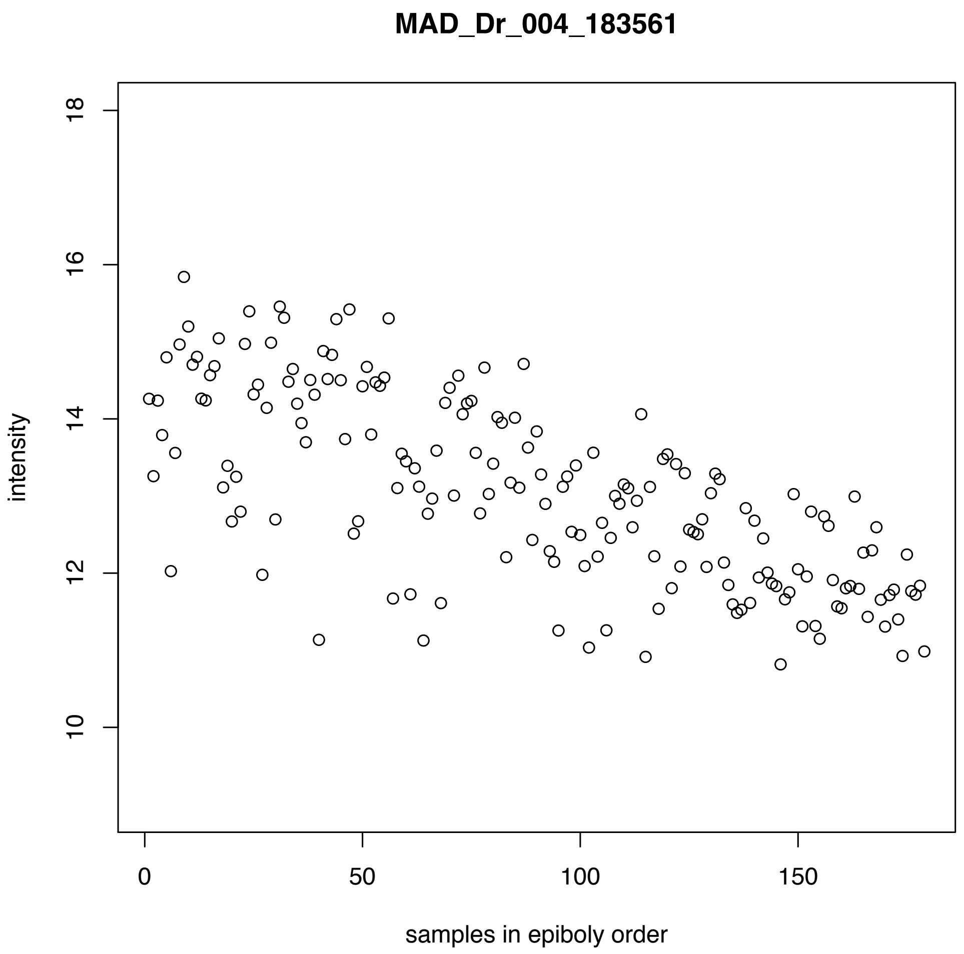
MAD_Dr_004_183561
intensity
10
12
14
16
18
0
50
100
150
samples in epiboly order

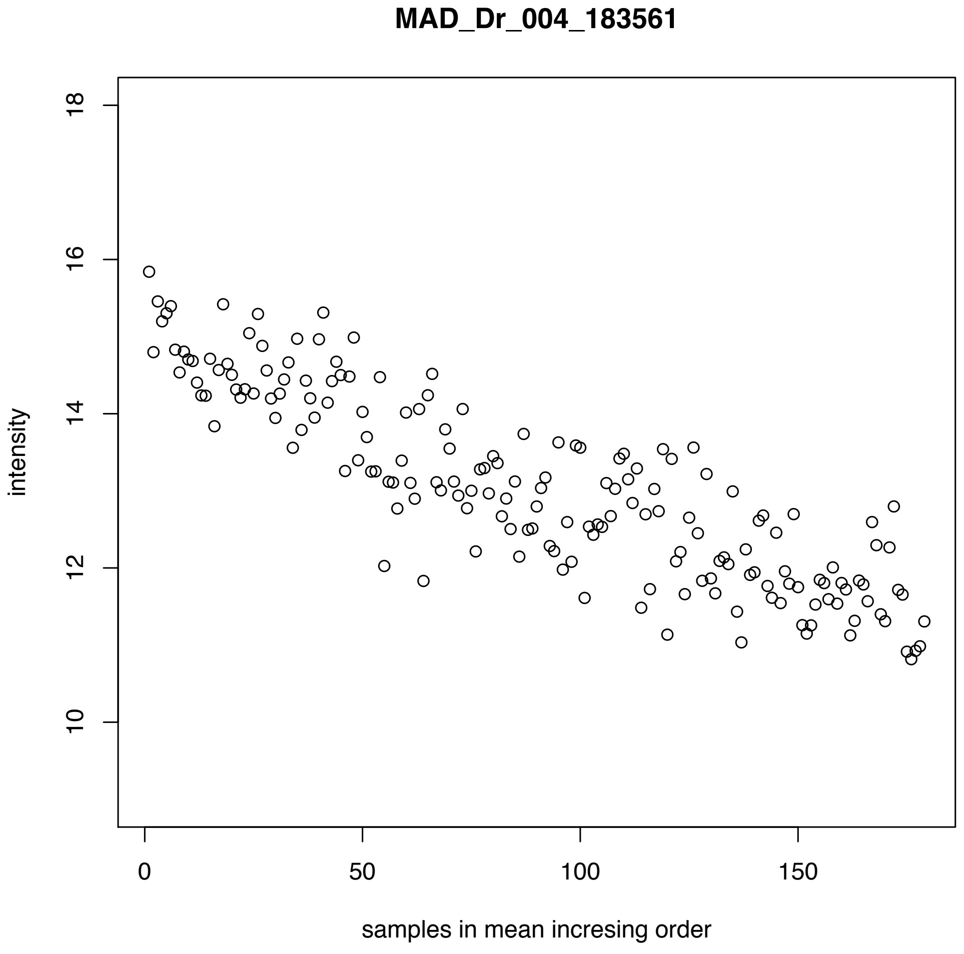
MAD_Dr_004_183561
intensity
10
12
14
16
18
0
50
100
150
samples in mean incresing order

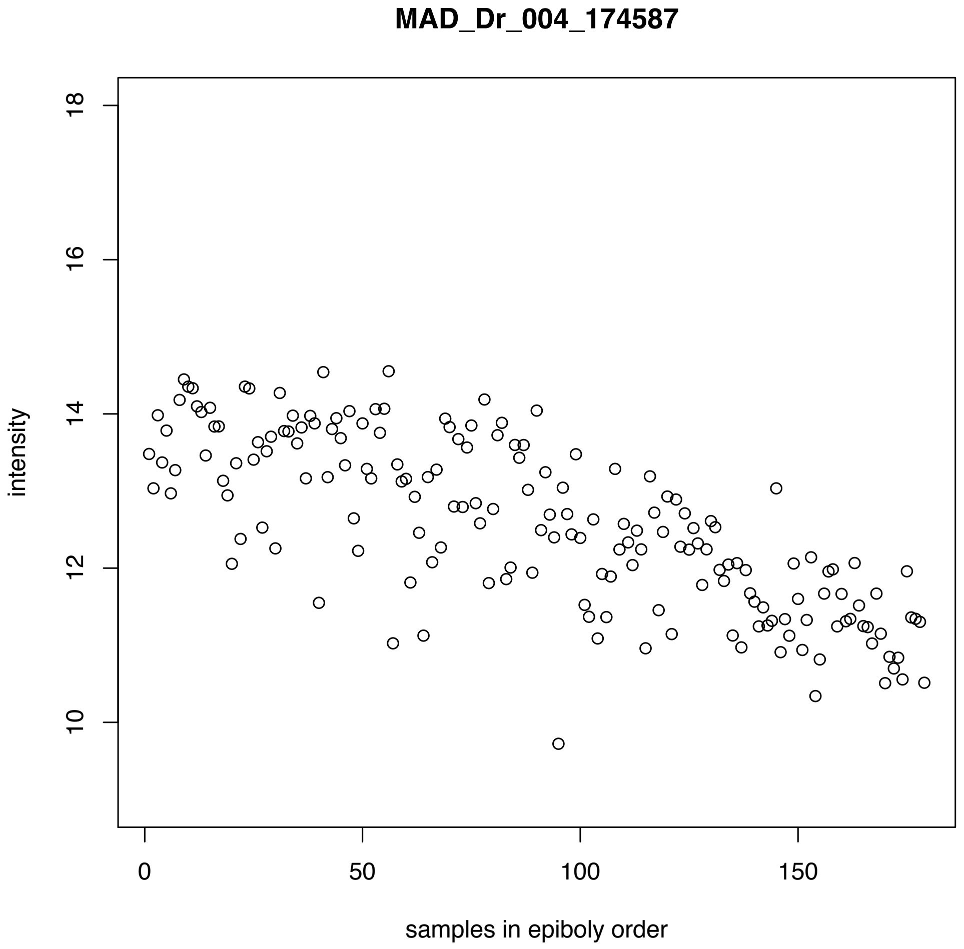
MAD_Dr_004_174587
intensity
10
12
14
16
18
0
50
100
150
samples in epiboly order

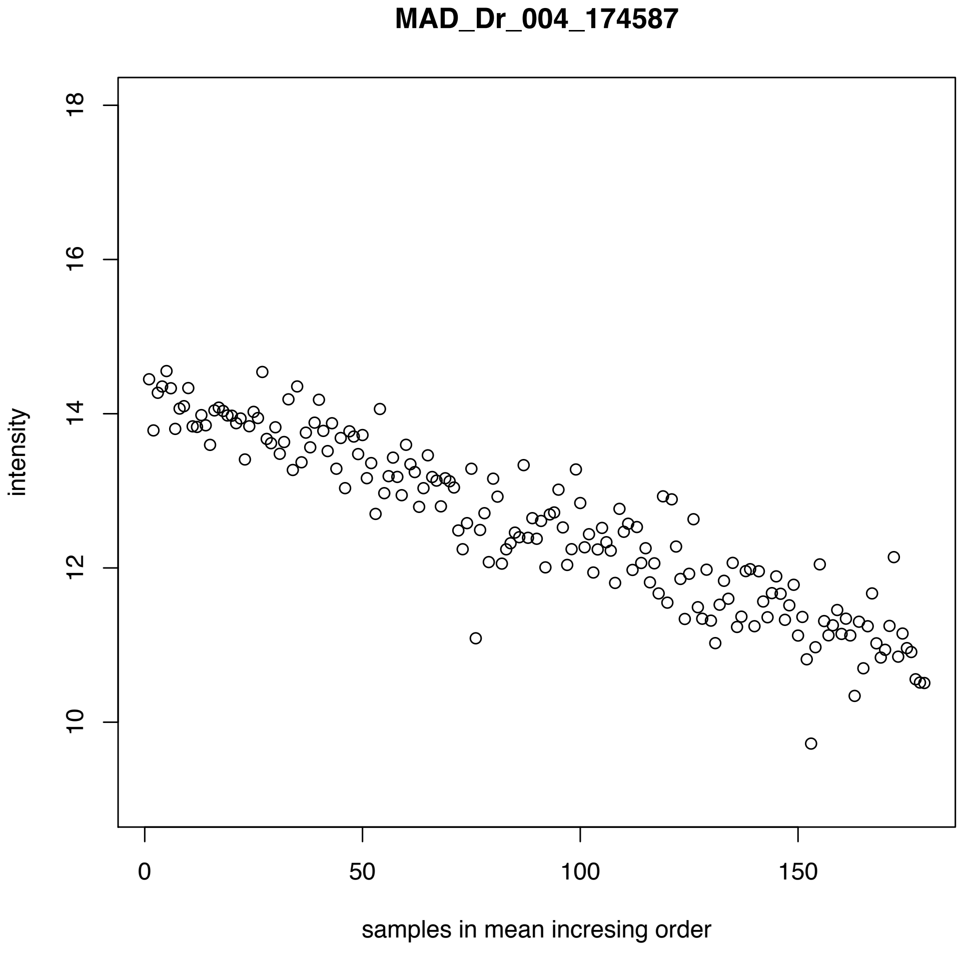
MAD_Dr_004_174587
intensity
10
12
14
16
18
0
50
100
150
samples in mean incresing order

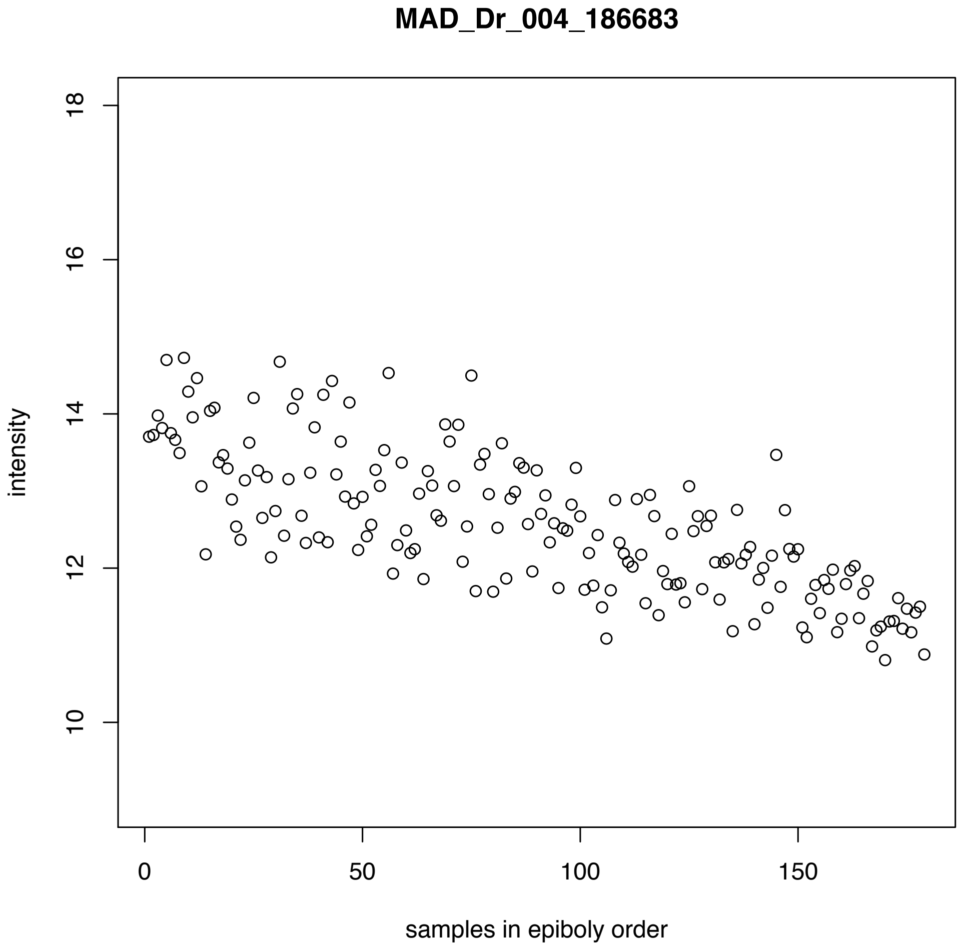
MAD_Dr_004_186683
intensity
10
12
14
16
18
0
50
100
150
samples in epiboly order

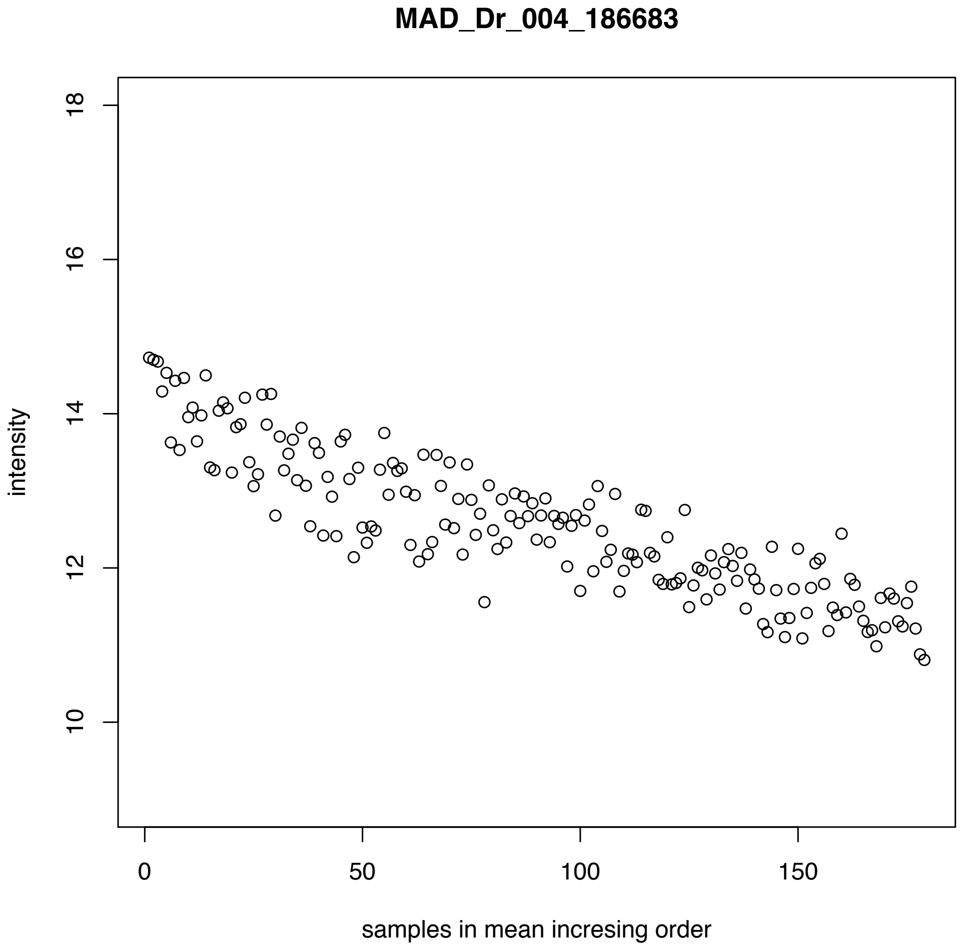
MAD_Dr_004_186683
intensity
10
12
14
16
18
0
50
100
150
samples in mean incresing order

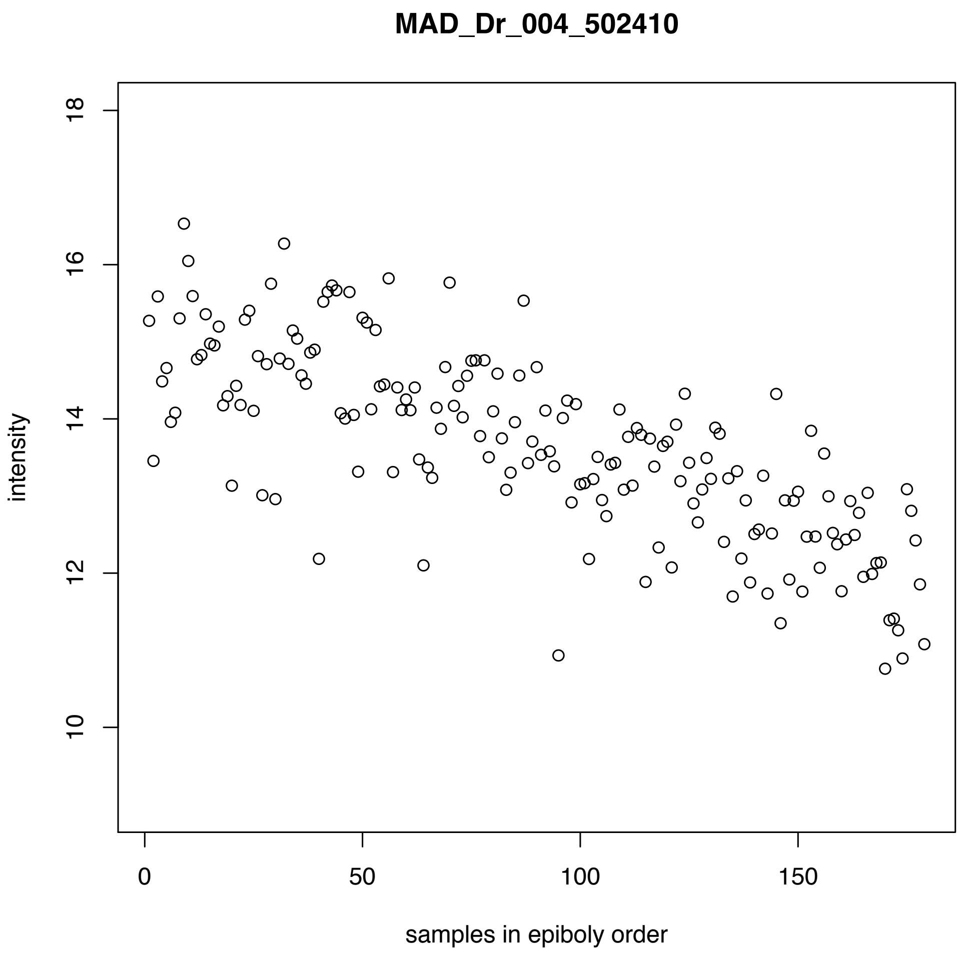
MAD_Dr_004_502410
intensity
samples in epiboly order
10
12
14
16
18
0
50
100
150

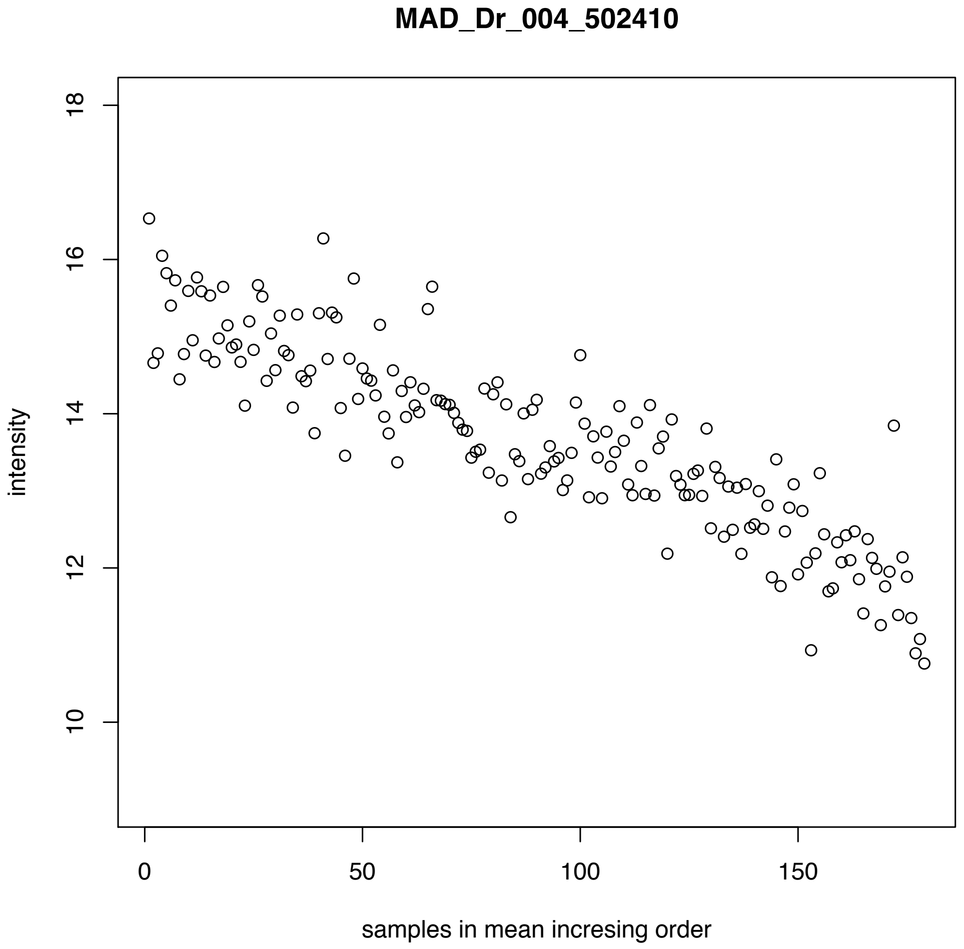
MAD_Dr_004_502410
intensity
samples in mean incresing order
10
12
14
16
18
0
50
100
150

Comparison between the gene expression profiles orderings using:

121 continuously **decreasing** genes to ***train*** the ordering

97 continuously **increasing** genes to ***test*** the ordering.

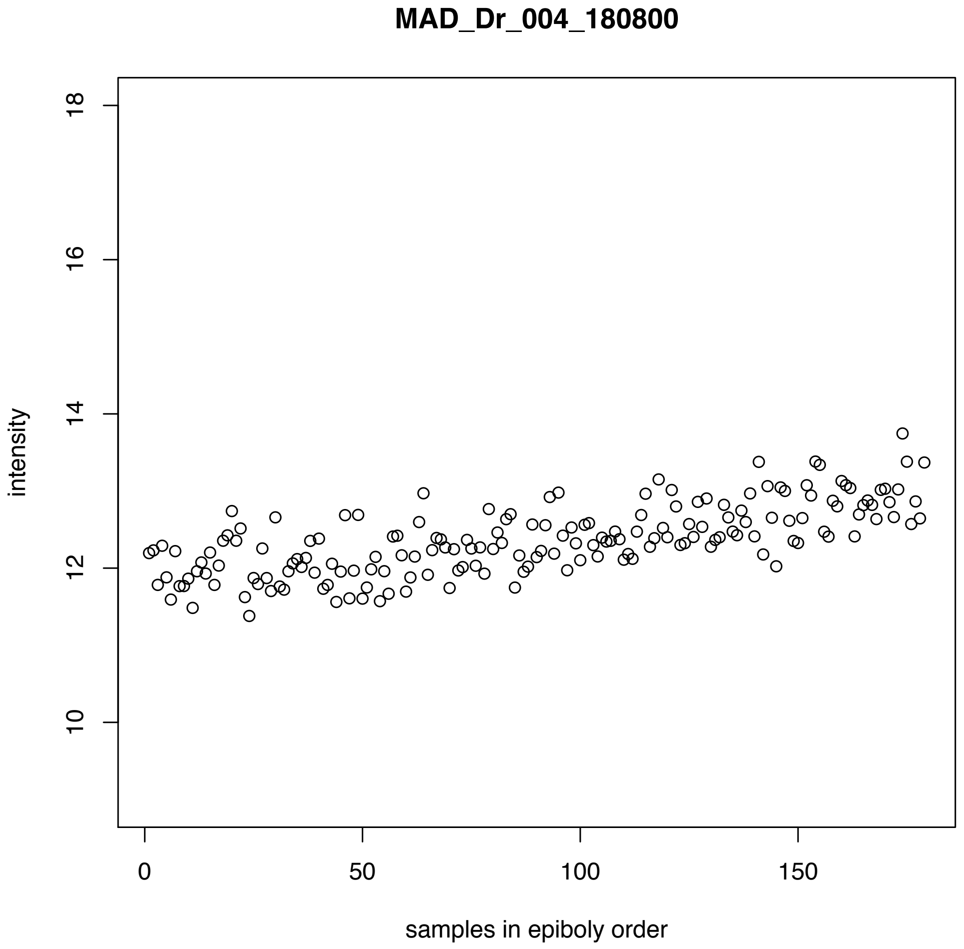
MAD_Dr_004_180800
intensity
18
16
14
12
10
0
50
100
150
samples in epiboly order

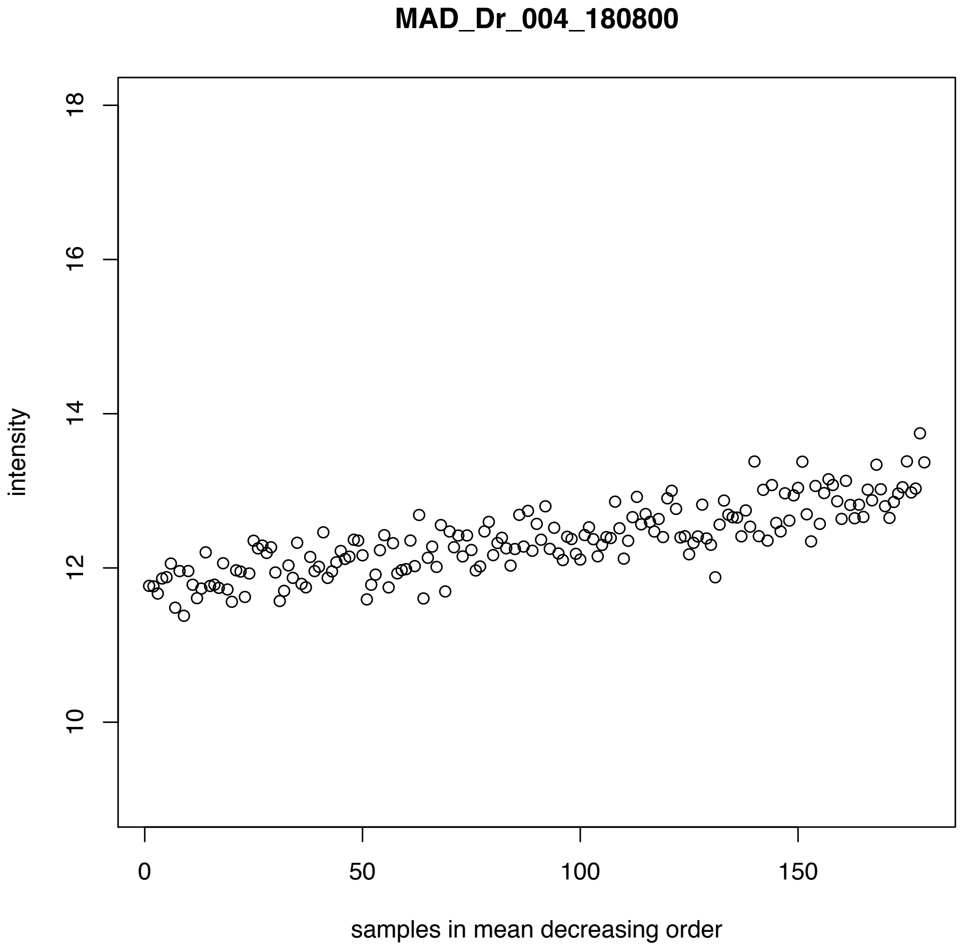
MAD_Dr_004_180800
intensity
18
16
14
12
10
0
50
100
150
samples in mean decreasing order

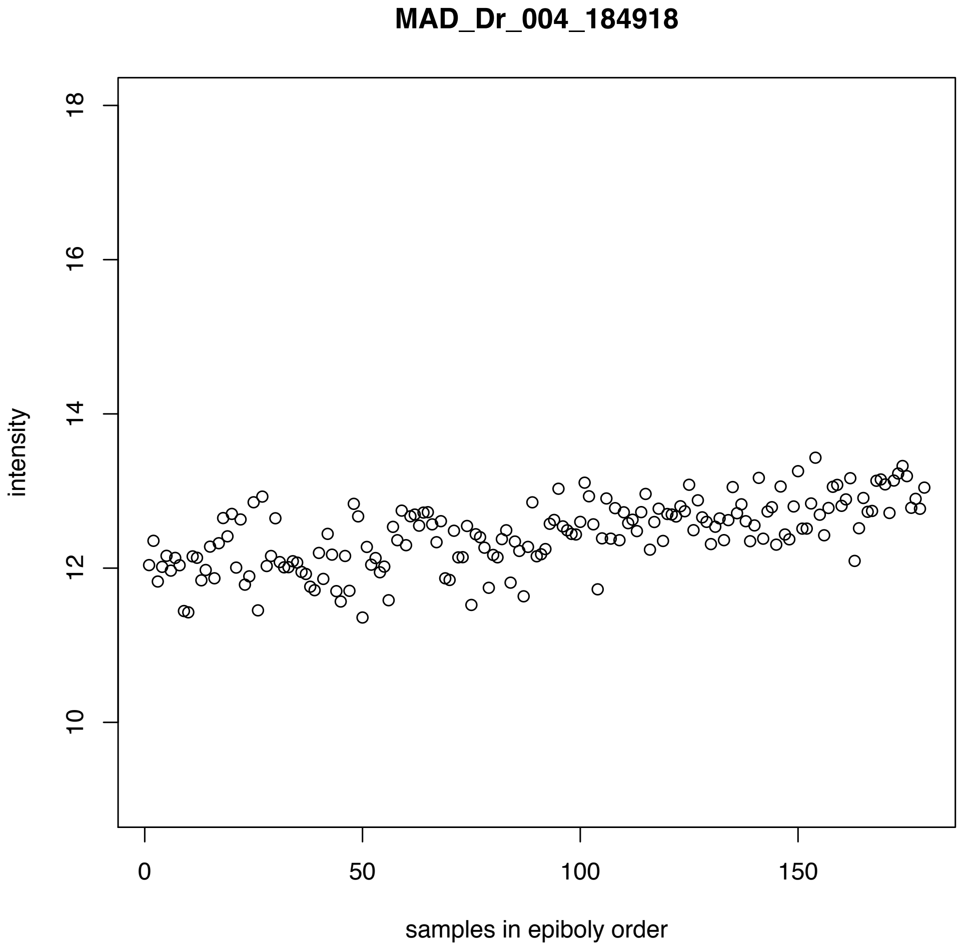
MAD_Dr_004_184918
intensity
10
12
14
16
18
0
50
100
150
samples in epiboly order

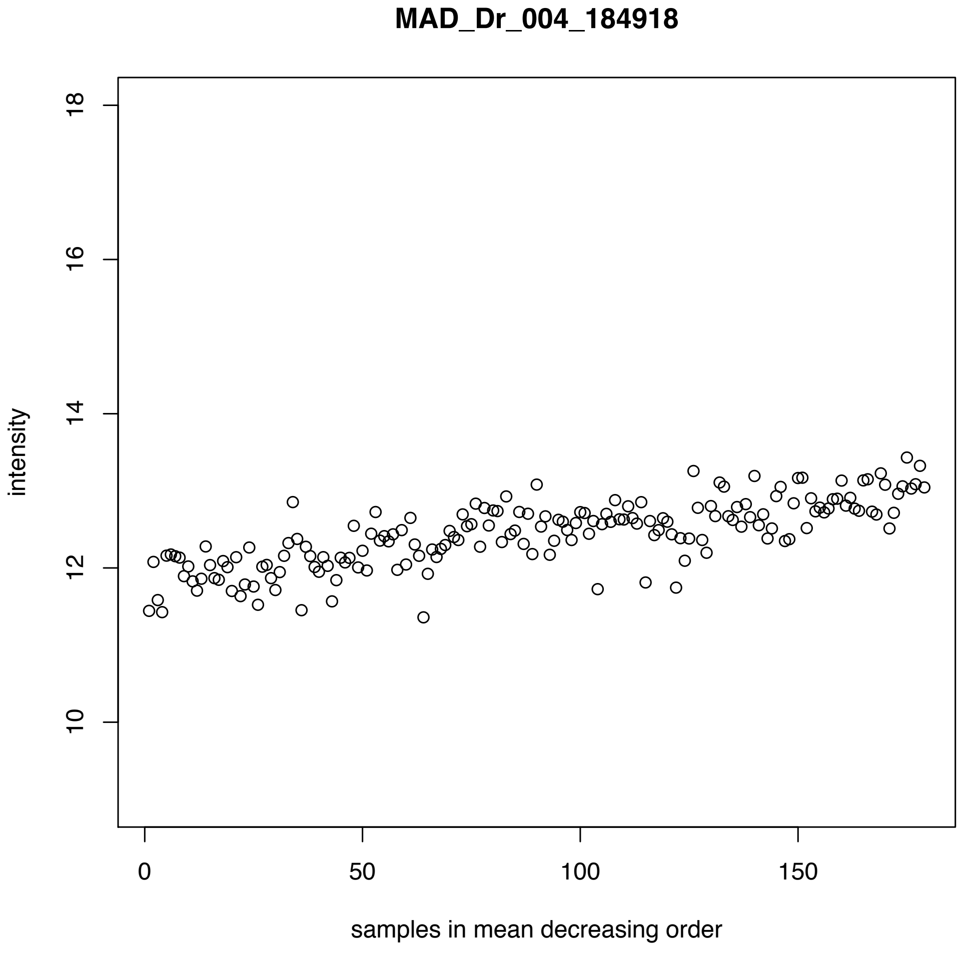
MAD_Dr_004_184918
intensity
10
12
14
16
18
0
50
100
150
samples in mean decreasing order

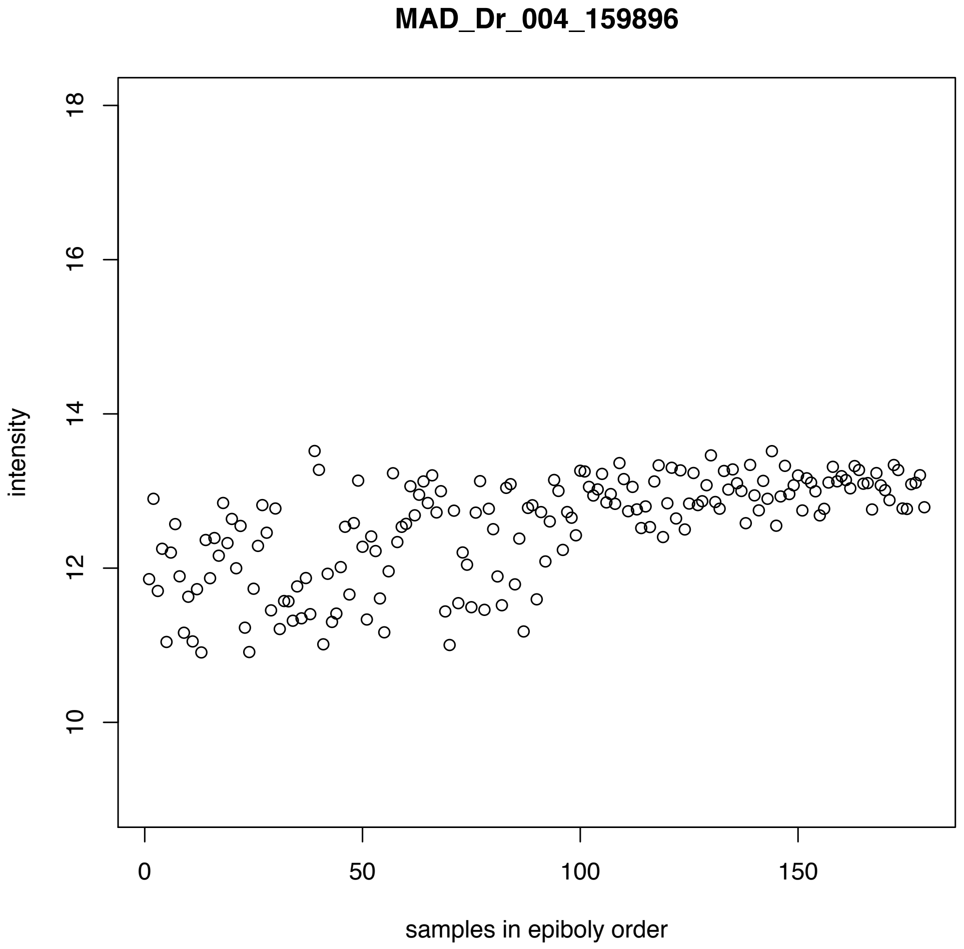

MAD_Dr_004_159896
intensity
10
12
14
16
18
0
50
100
150
samples in epiboly order

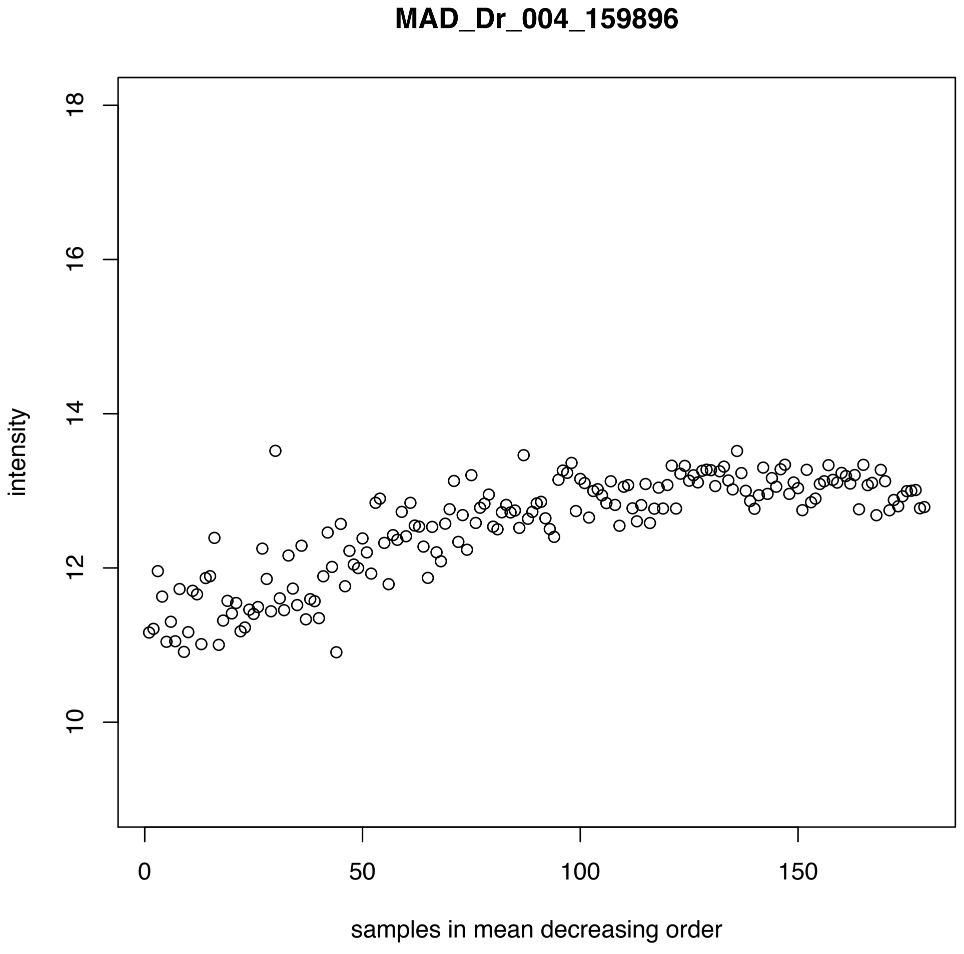

MAD_Dr_004_159896
intensity
10
12
14
16
18
0
50
100
150
samples in mean decreasing order

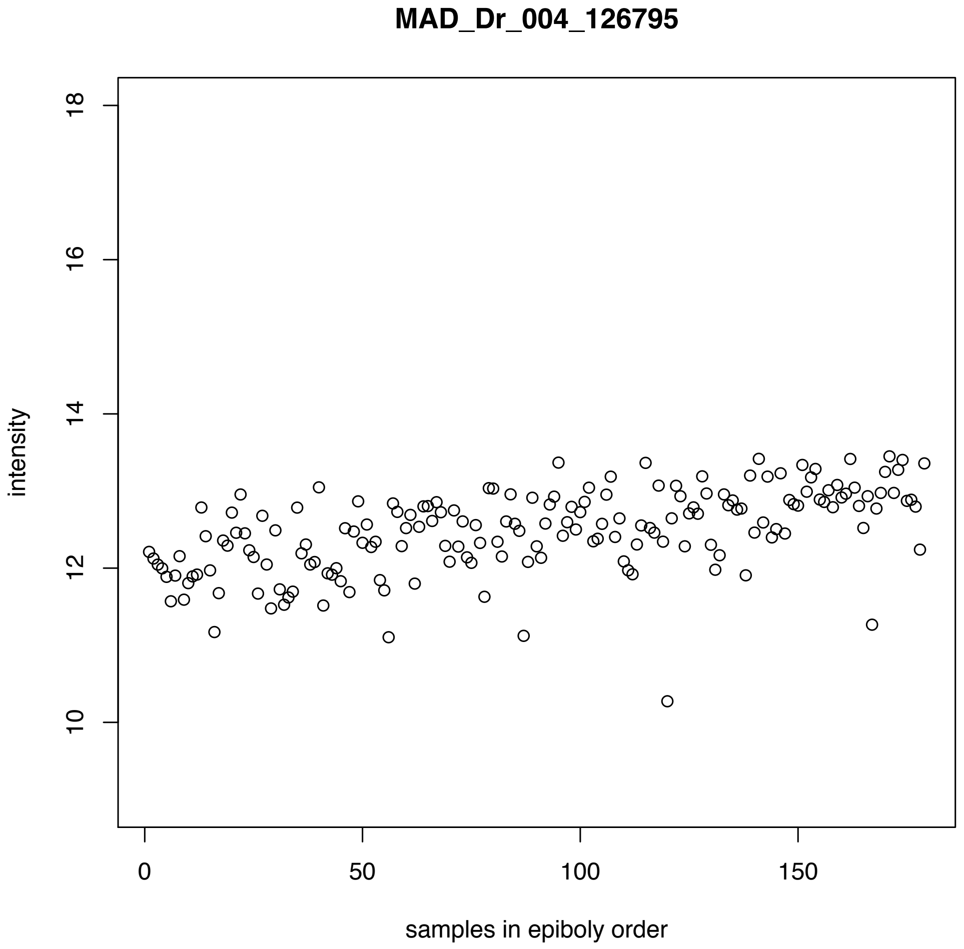
MAD_Dr_004_126795
intensity
samples in epiboly order
10
12
14
16
18
0
50
100
150

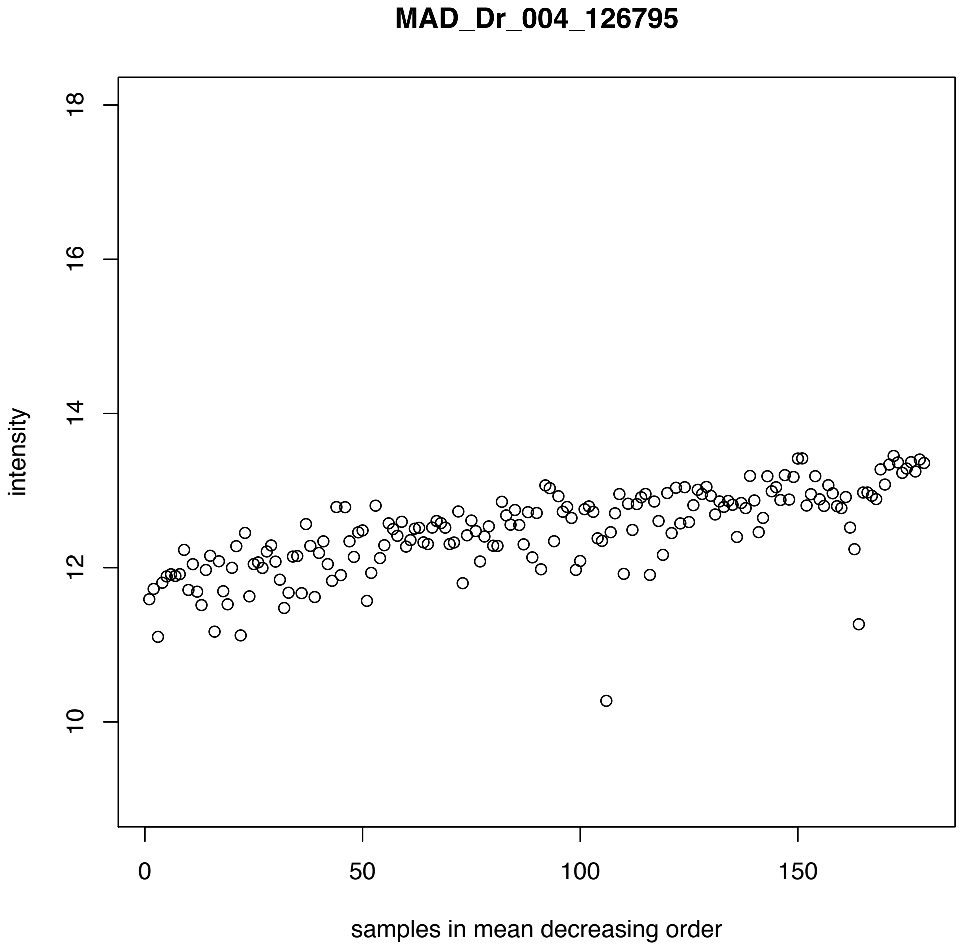
MAD_Dr_004_126795
intensity
samples in mean decreasing order
10
12
14
16
18
0
50
100
150

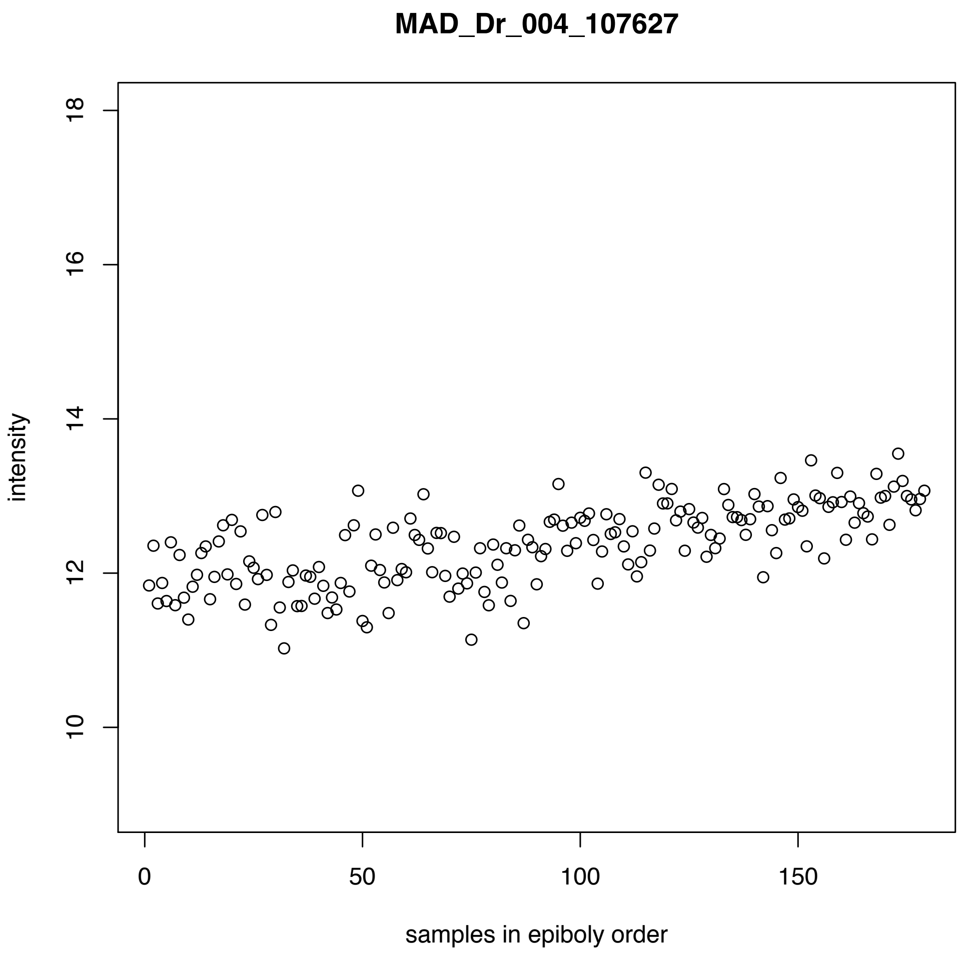
MAD_Dr_004_107627
intensity
10
12
14
16
18
0
50
100
150
samples in epiboly order

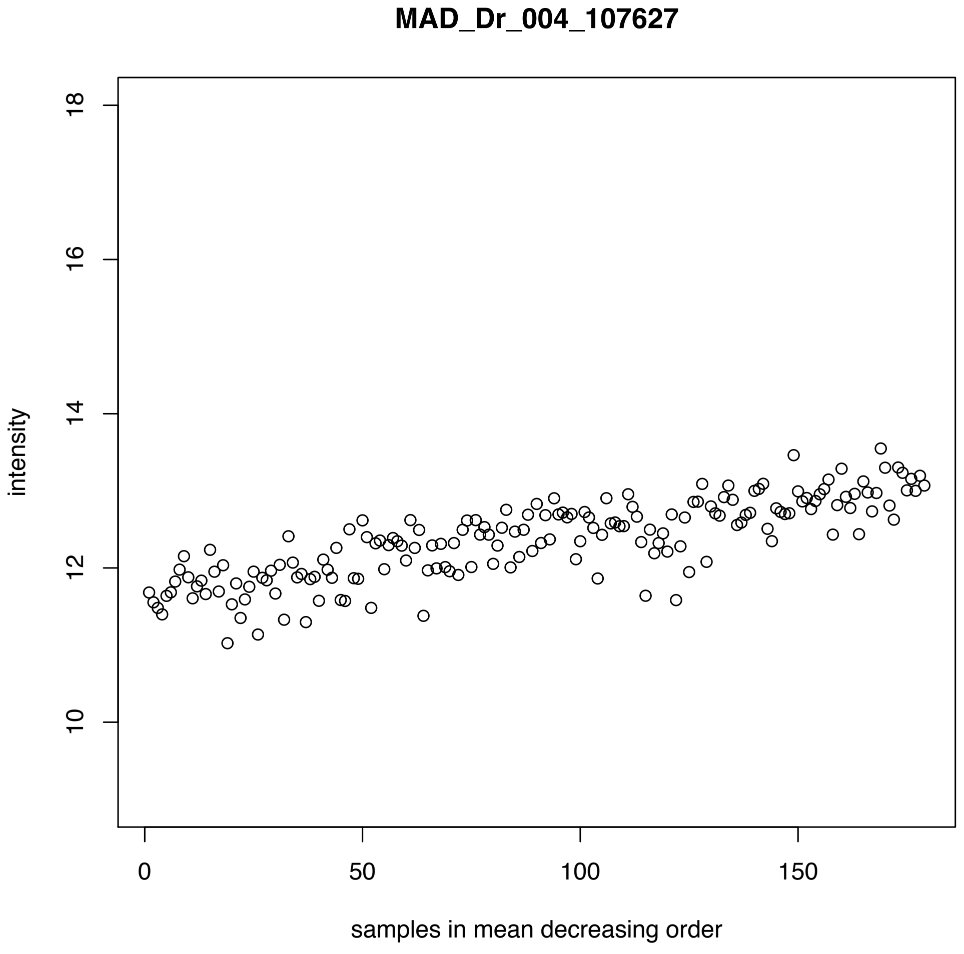
MAD_Dr_004_107627
intensity
10
12
14
16
18
0
50
100
150
samples in mean decreasing order

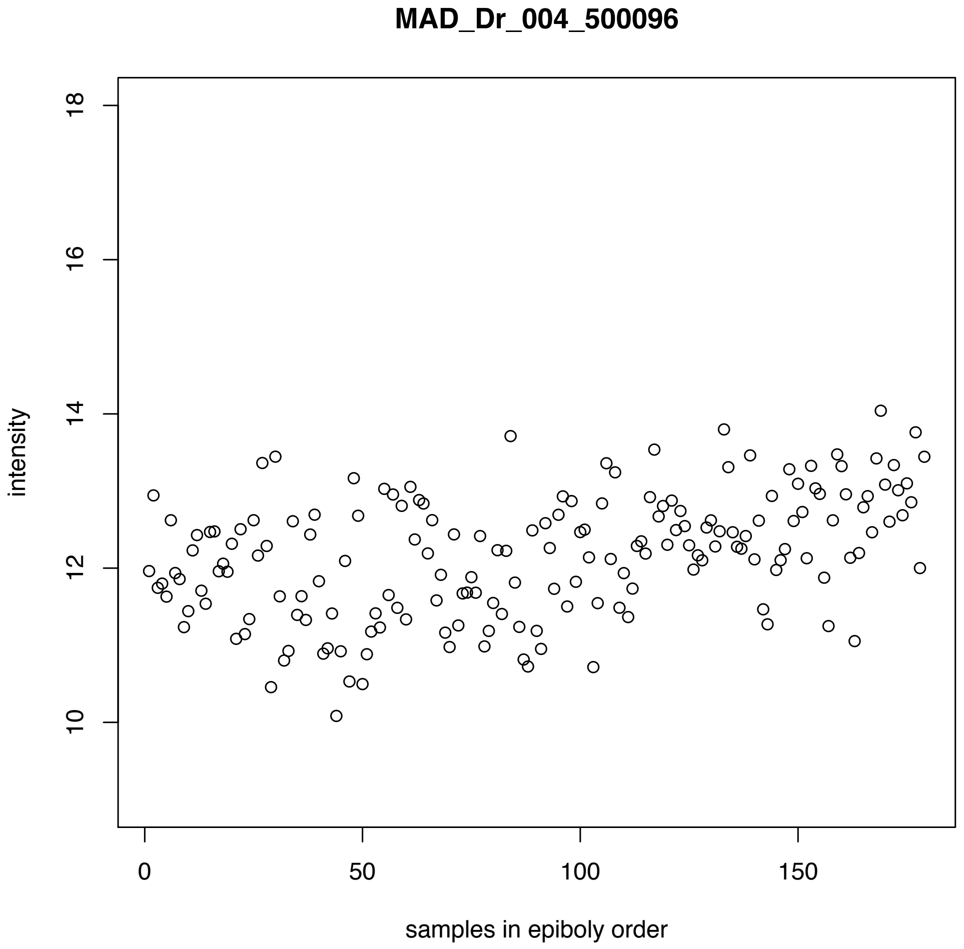
MAD_Dr_004_500096
intensity
18
16
14
12
10
0
50
100
150
samples in epiboly order

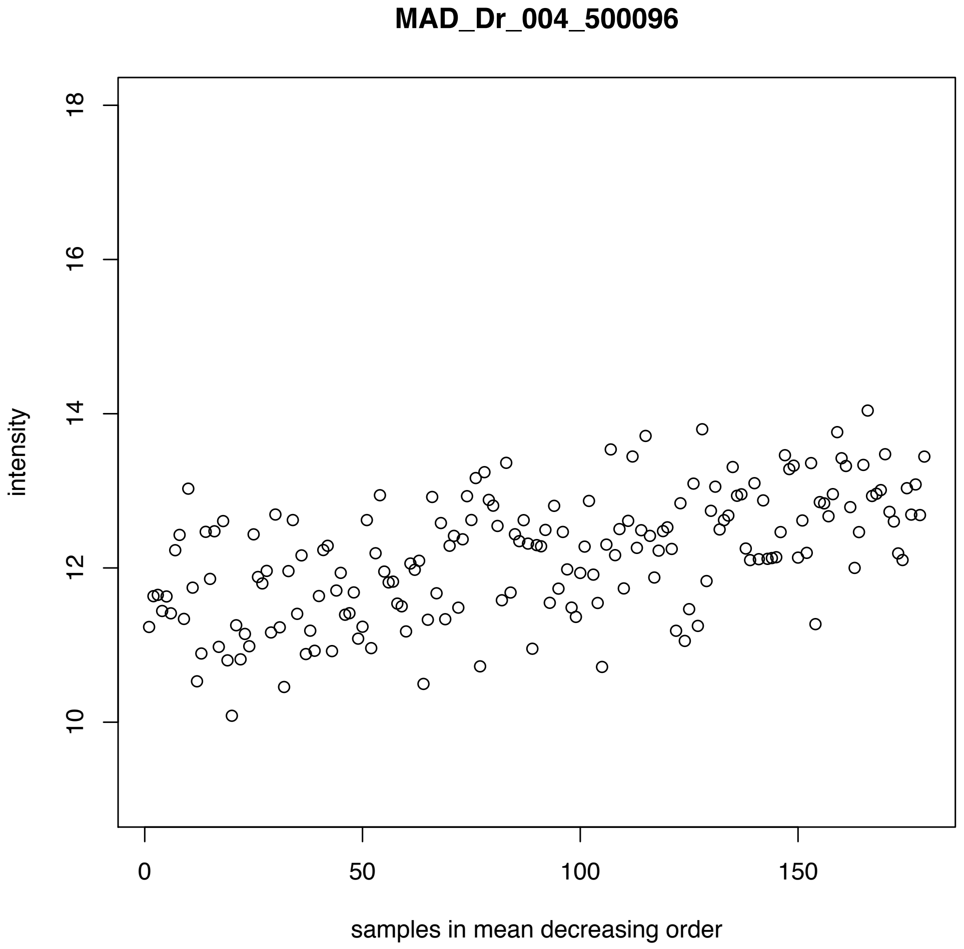
MAD_Dr_004_500096
intensity
18
16
14
12
10
0
50
100
150
samples in mean decreasing order

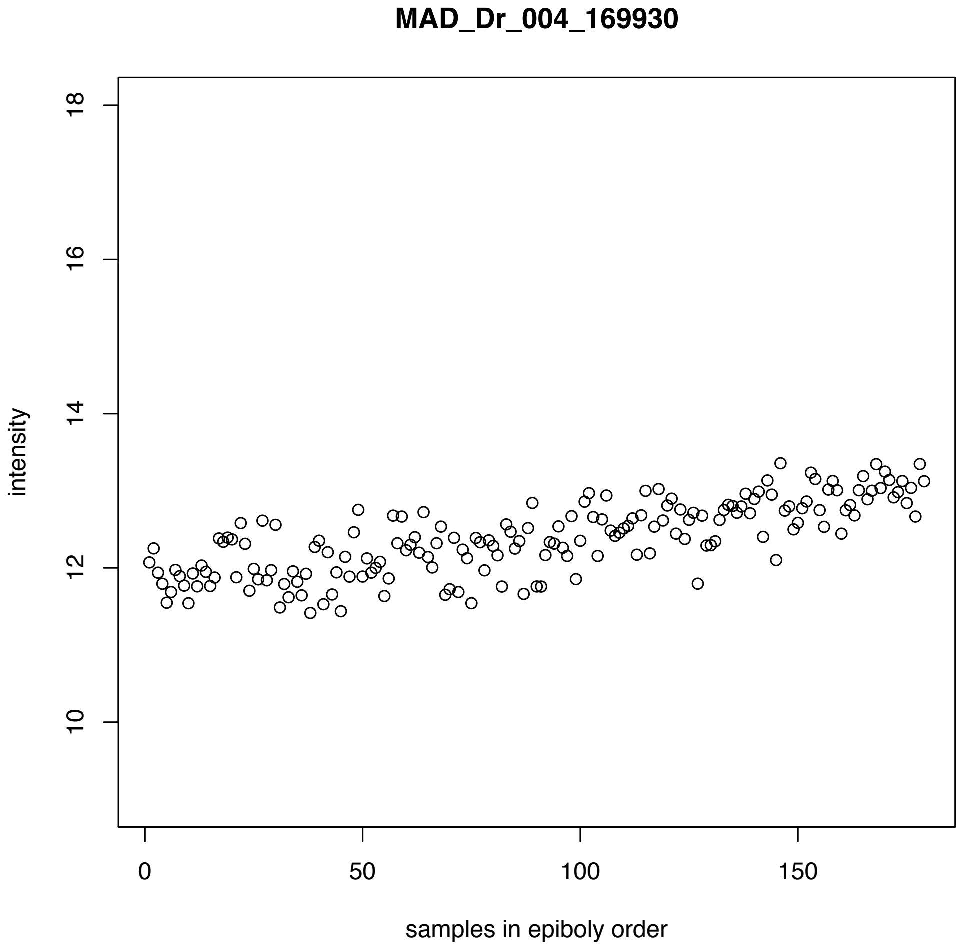
MAD_Dr_004_169930
intensity
18
16
14
12
10
0
50
100
150
samples in epiboly order

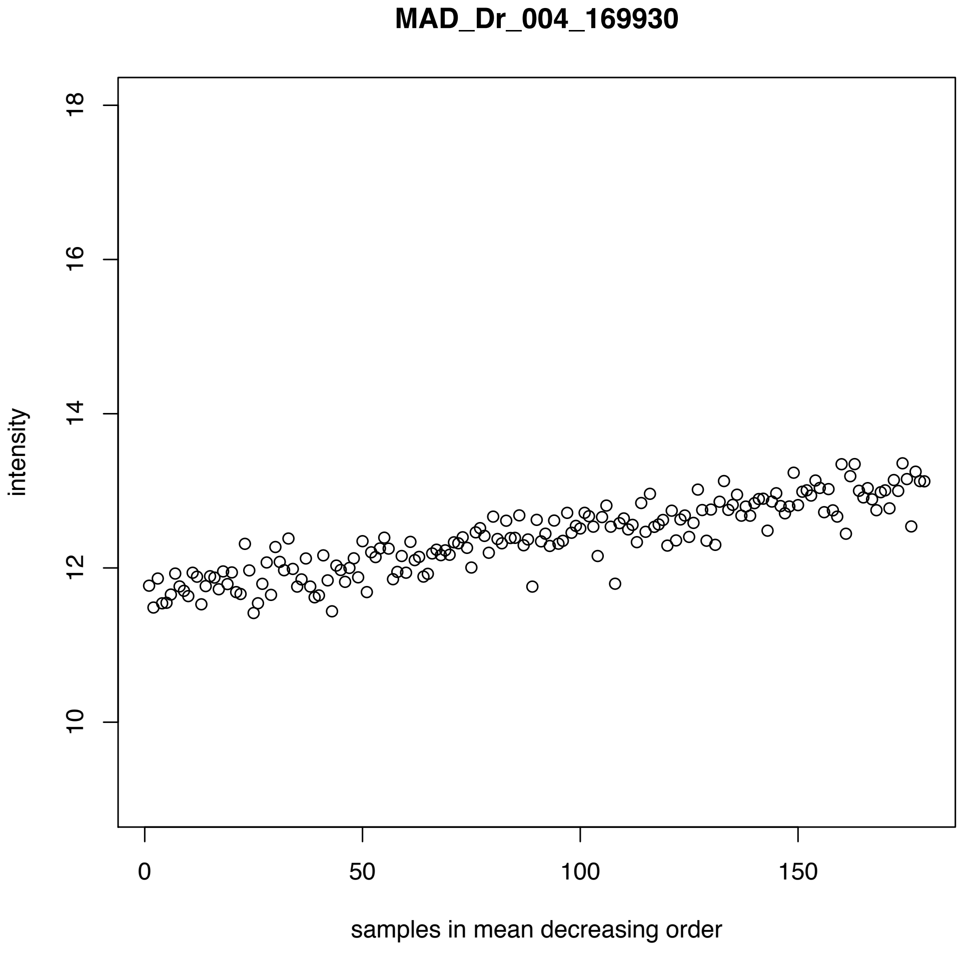
MAD_Dr_004_169930
intensity
18
16
14
12
10
0
50
100
150
samples in mean decreasing order

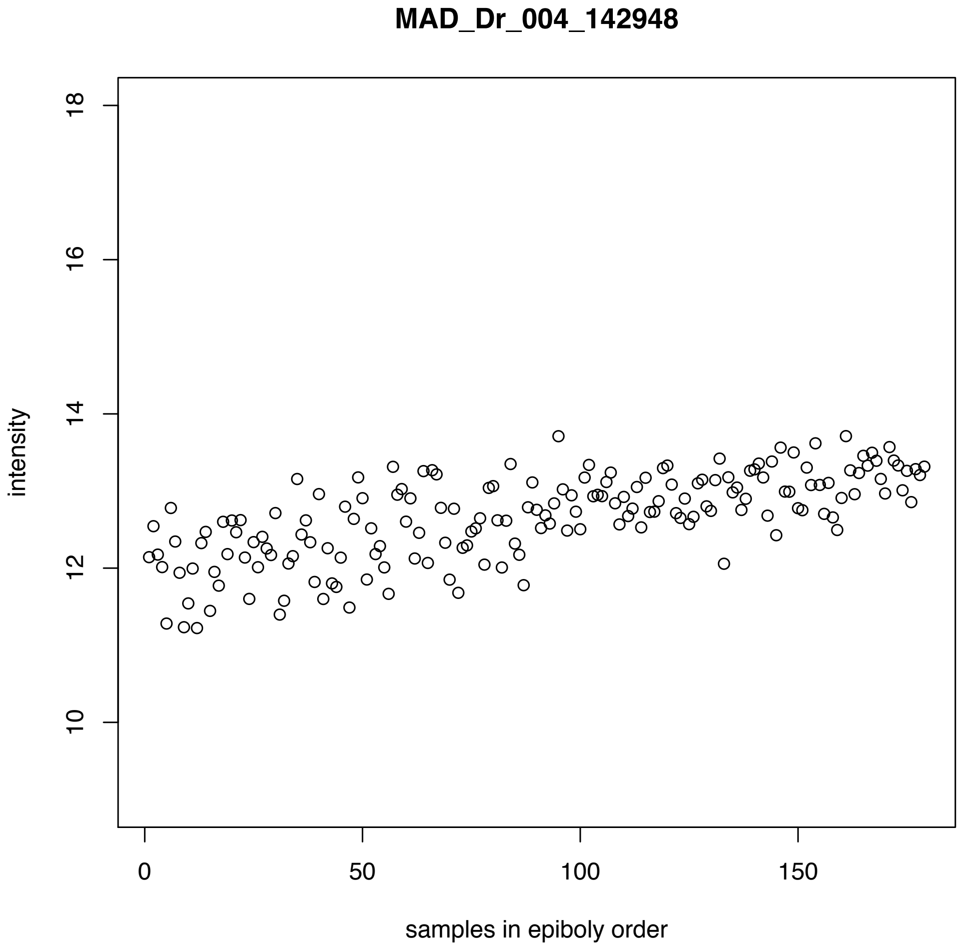
MAD_Dr_004_142948
intensity
18
16
14
12
10
0
50
100
150
samples in epiboly order

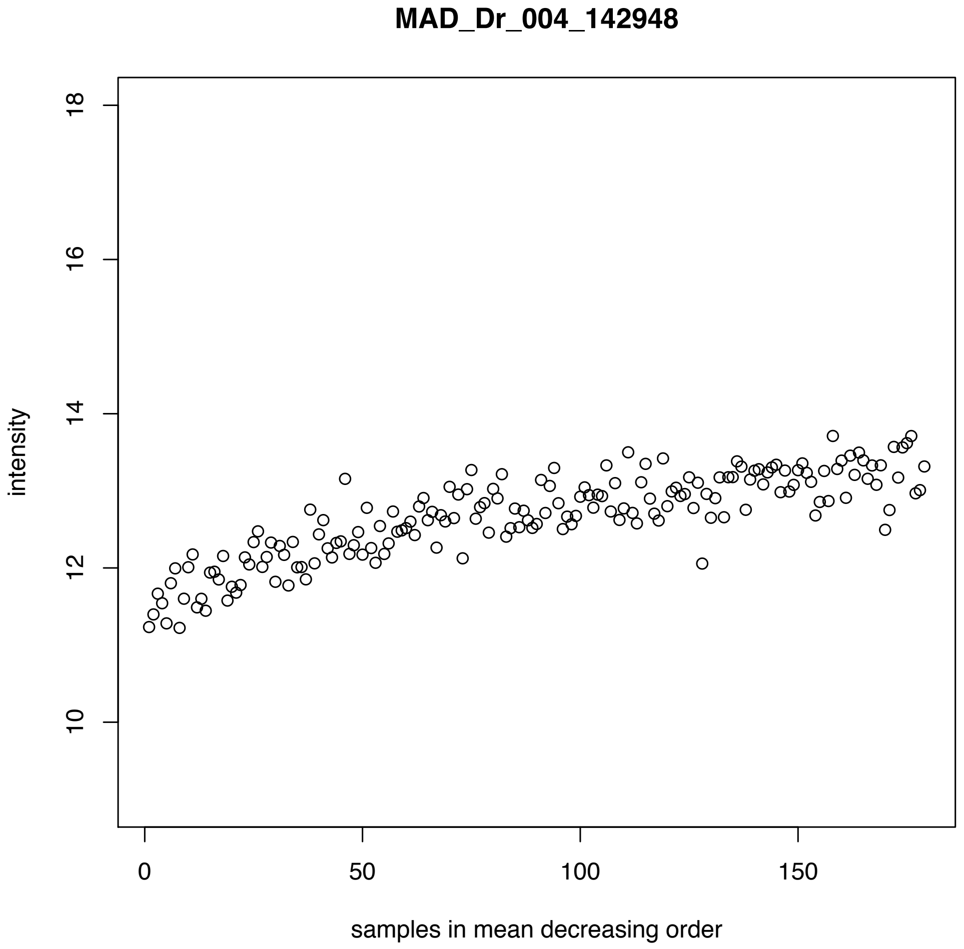
MAD_Dr_004_142948
intensity
18
16
14
12
10
0
50
100
150
samples in mean decreasing order

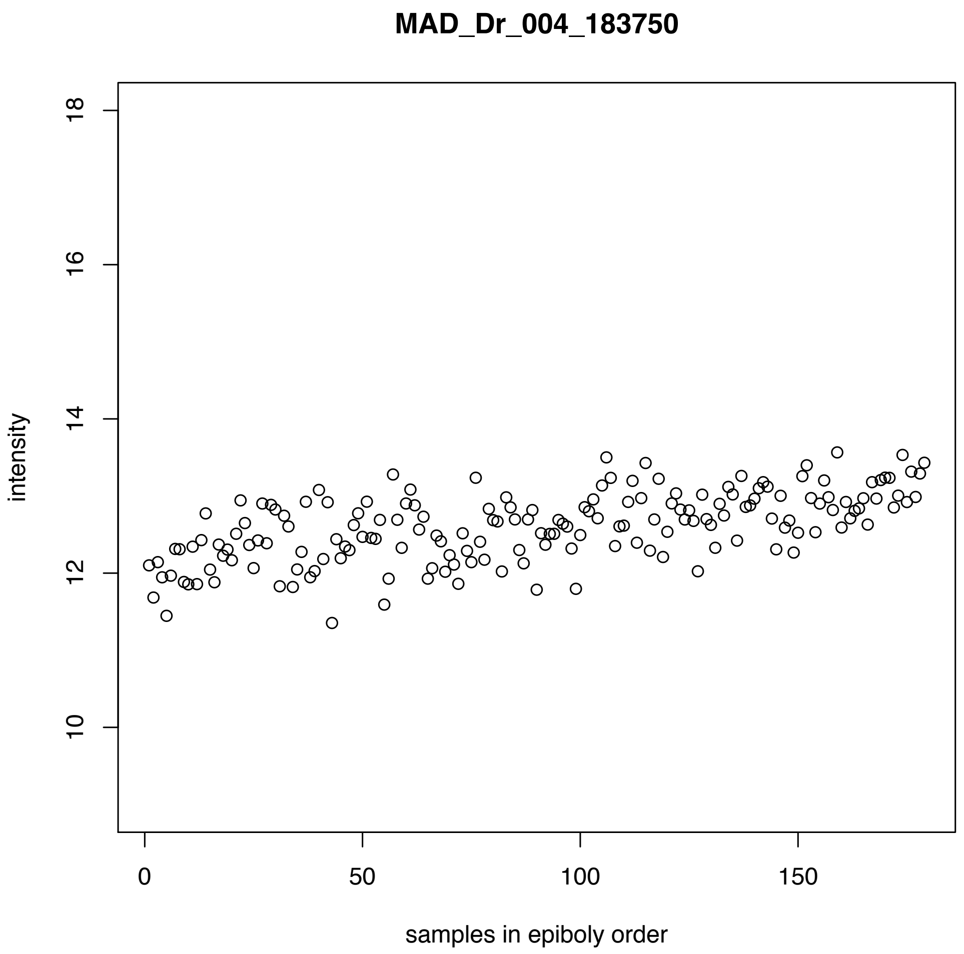

MAD_Dr_004_183750
intensity
10
12
14
16
18
0
50
100
150
samples in epiboly order

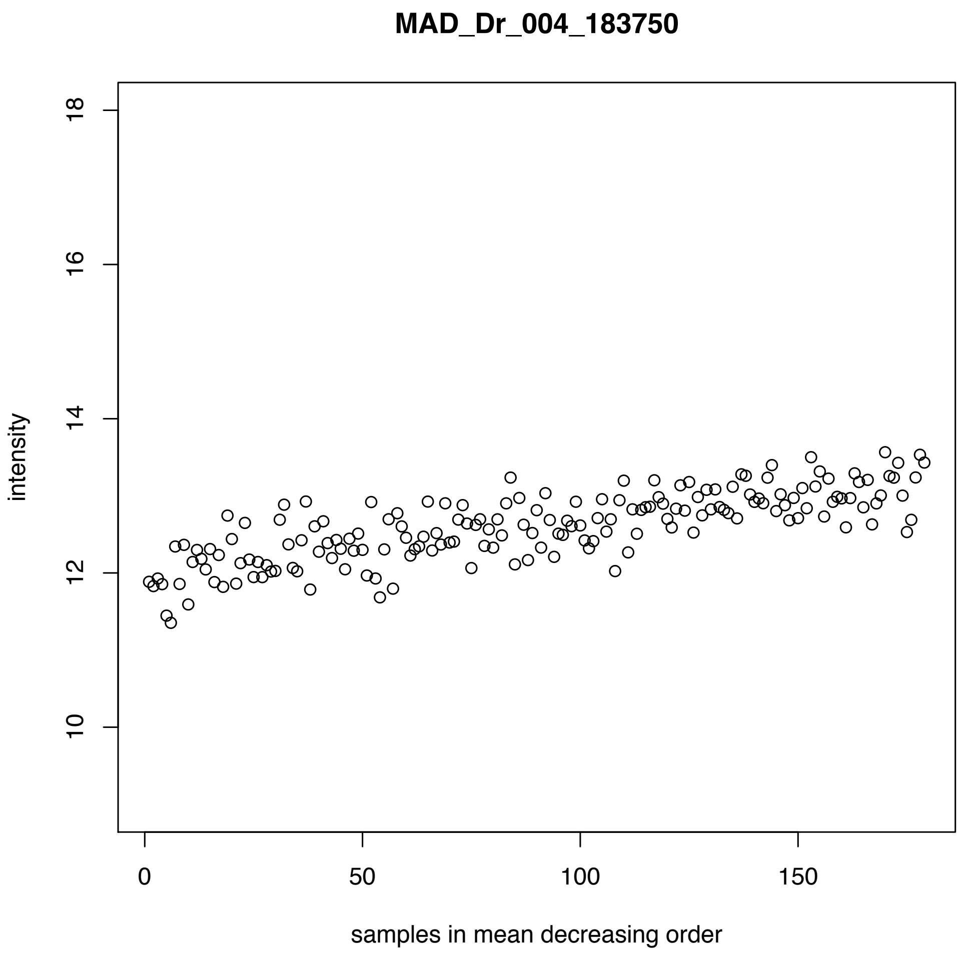

MAD_Dr_004_183750
intensity
10
12
14
16
18
0
50
100
150
samples in mean decreasing order

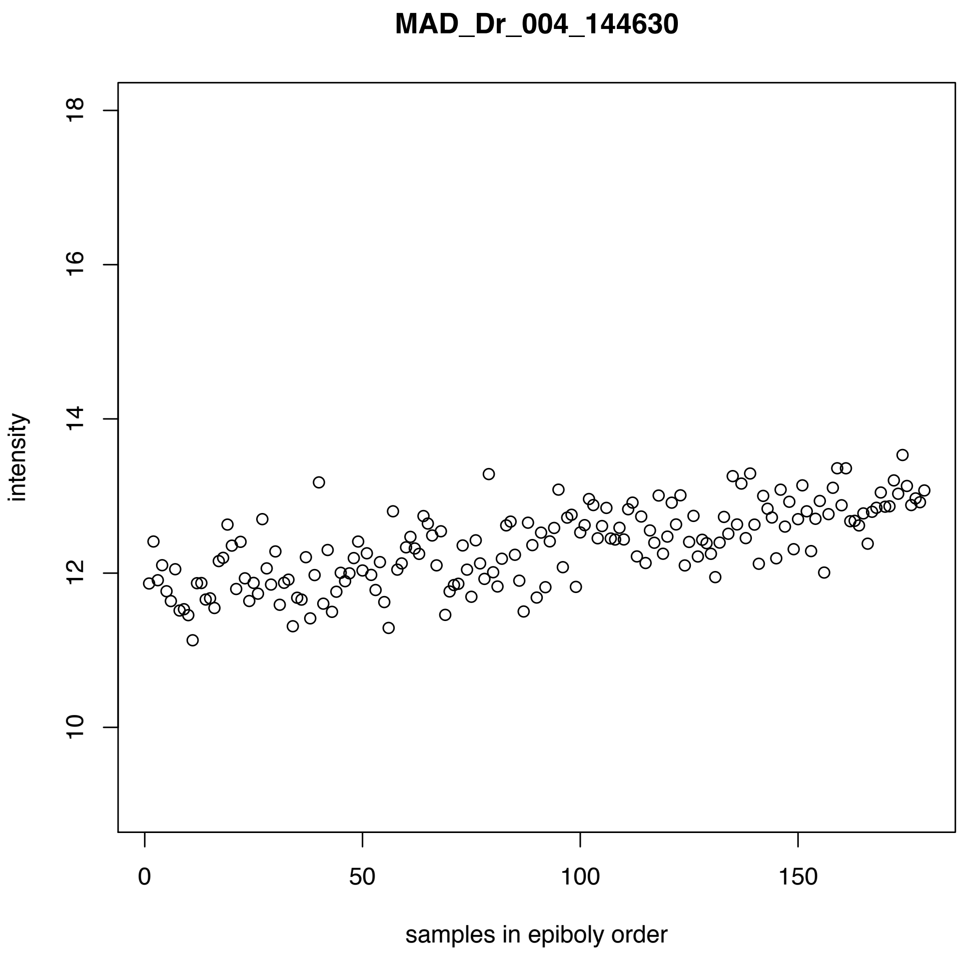
MAD_Dr_004_144630
intensity
10
12
14
16
18
0
50
100
150
samples in epiboly order

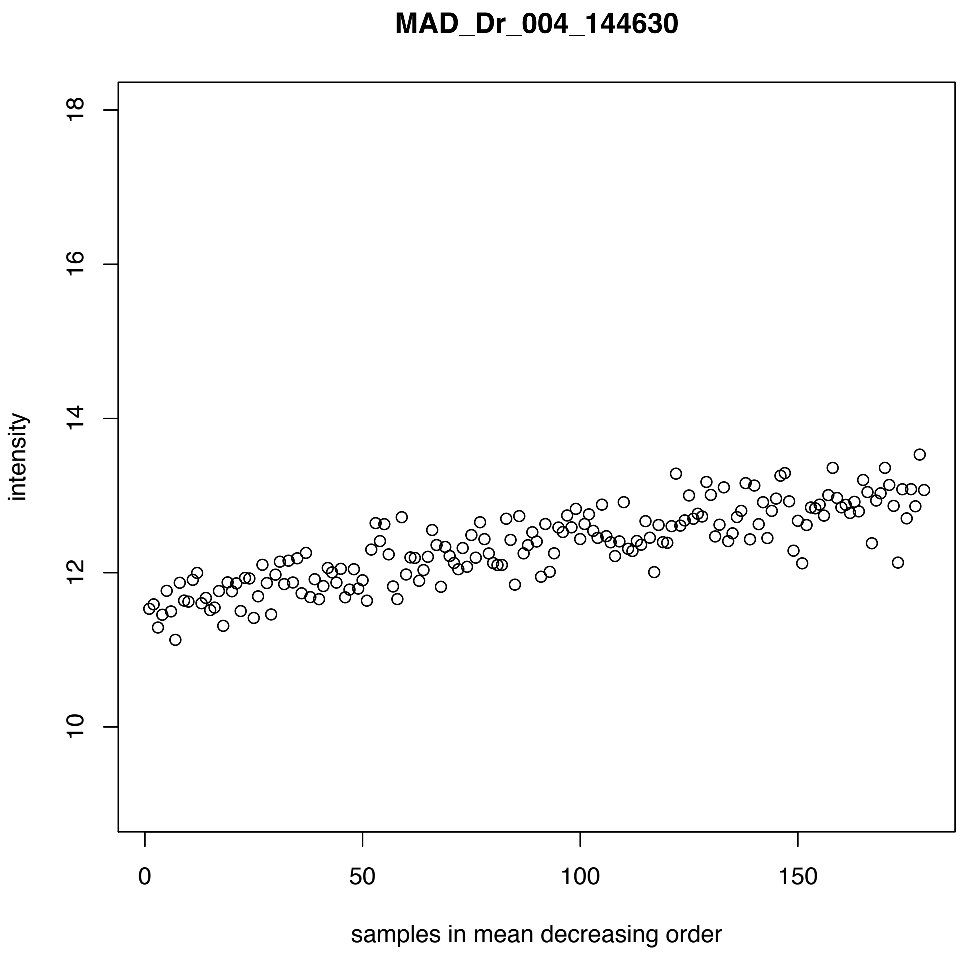
MAD_Dr_004_144630
intensity
10
12
14
16
18
0
50
100
150
samples in mean decreasing order

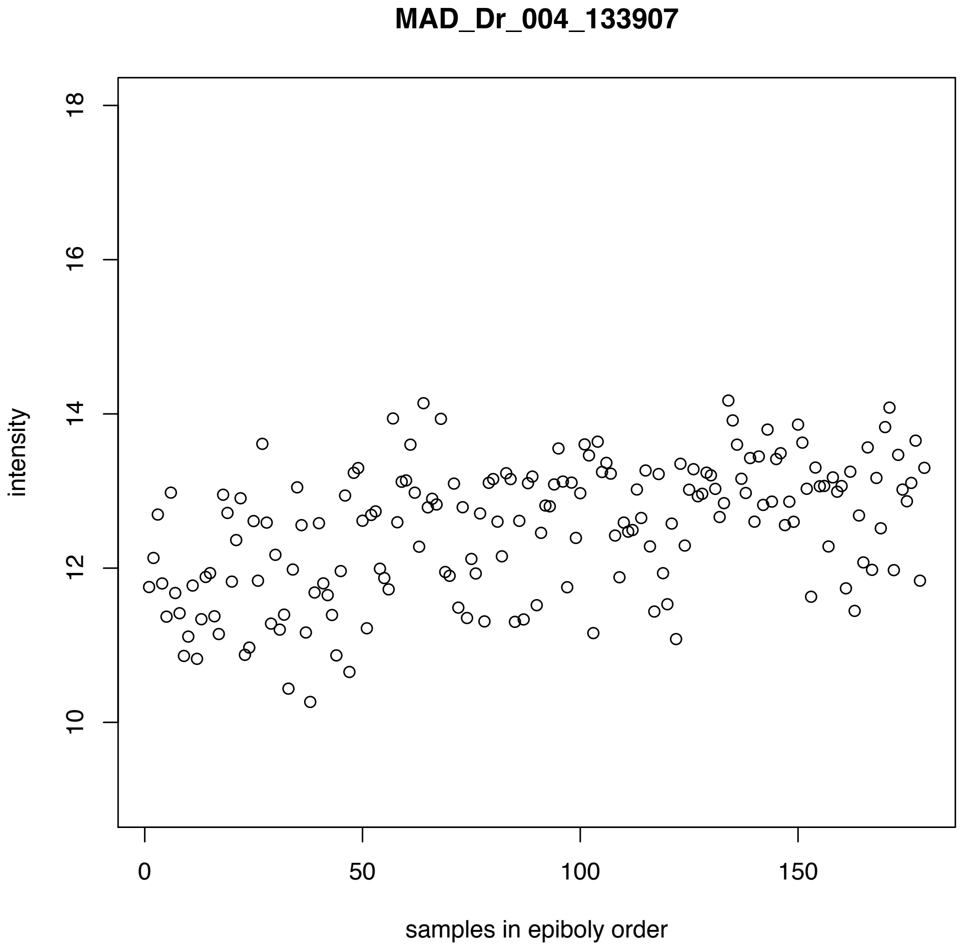
MAD_Dr_004_133907
intensity
10
12
14
16
18
0
50
100
150
samples in epiboly order

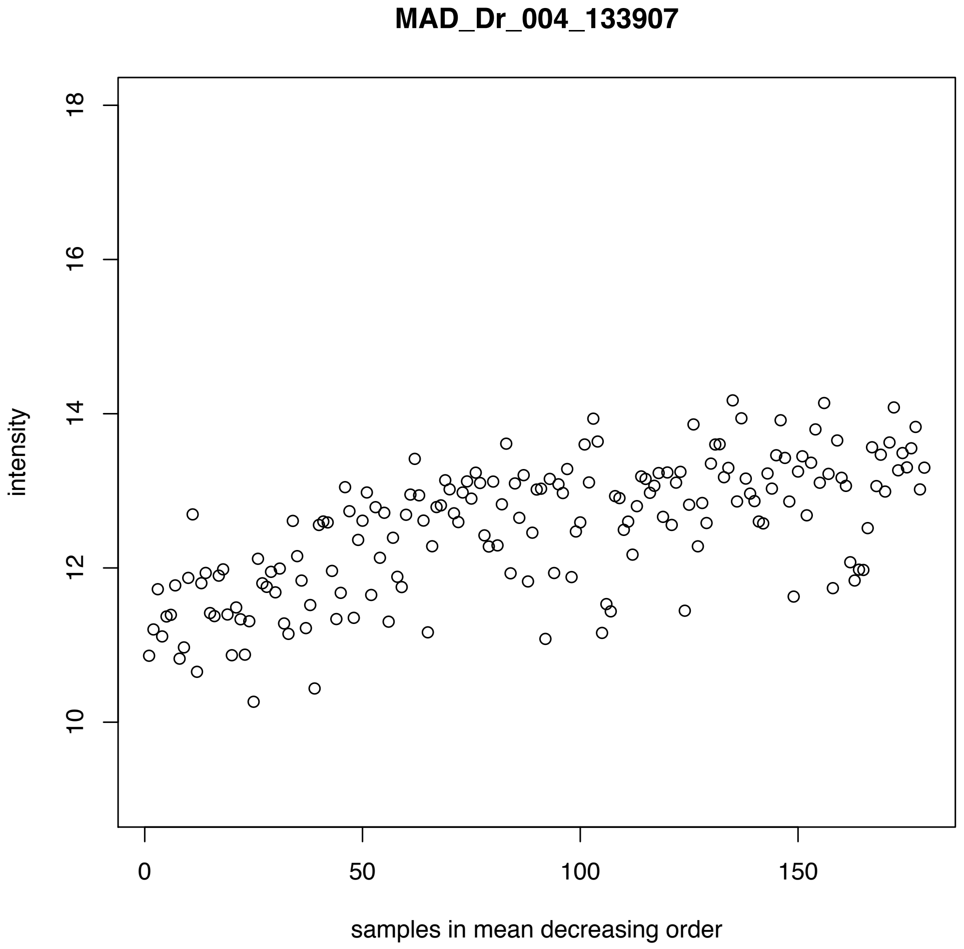
MAD_Dr_004_133907
intensity
10
12
14
16
18
0
50
100
150
samples in mean decreasing order

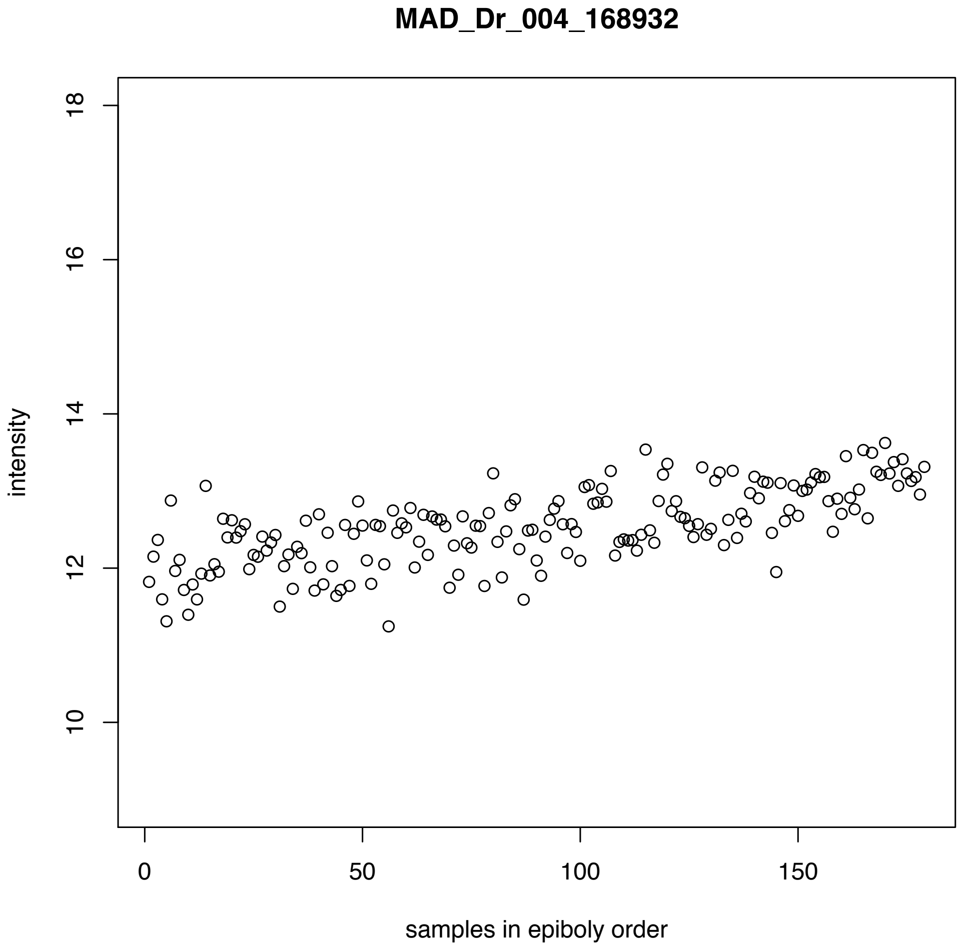
MAD_Dr_004_168932
intensity
samples in epiboly order
10
12
14
16
18
0
50
100
150

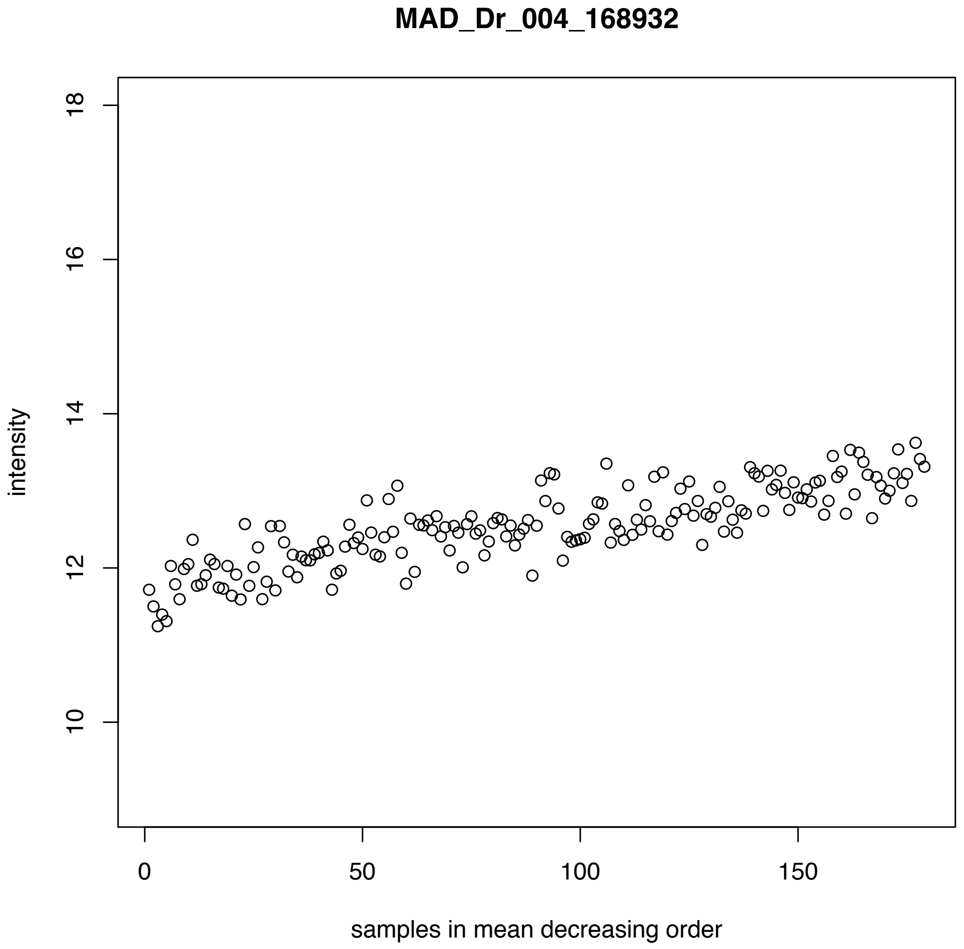
MAD_Dr_004_168932
intensity
samples in mean decreasing order
10
12
14
16
18
0
50
100
150

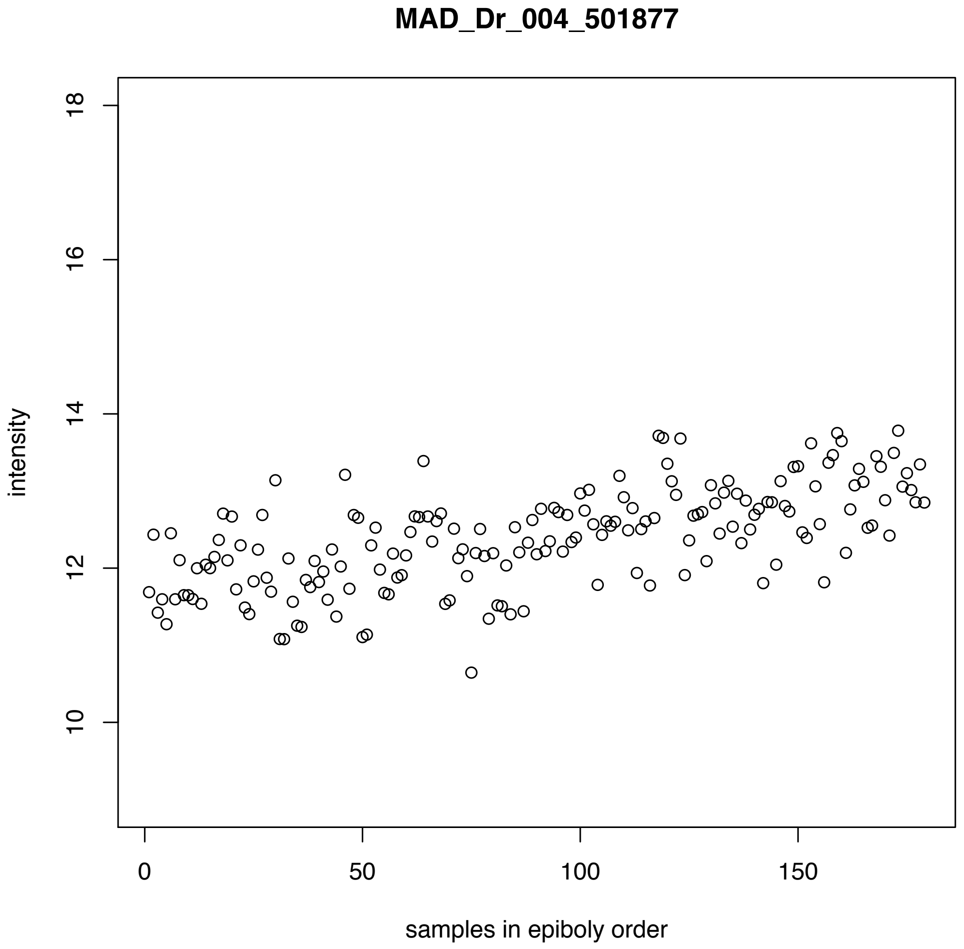
MAD_Dr_004_501877
intensity
samples in epiboly order
10
12
14
16
18
0
50
100
150

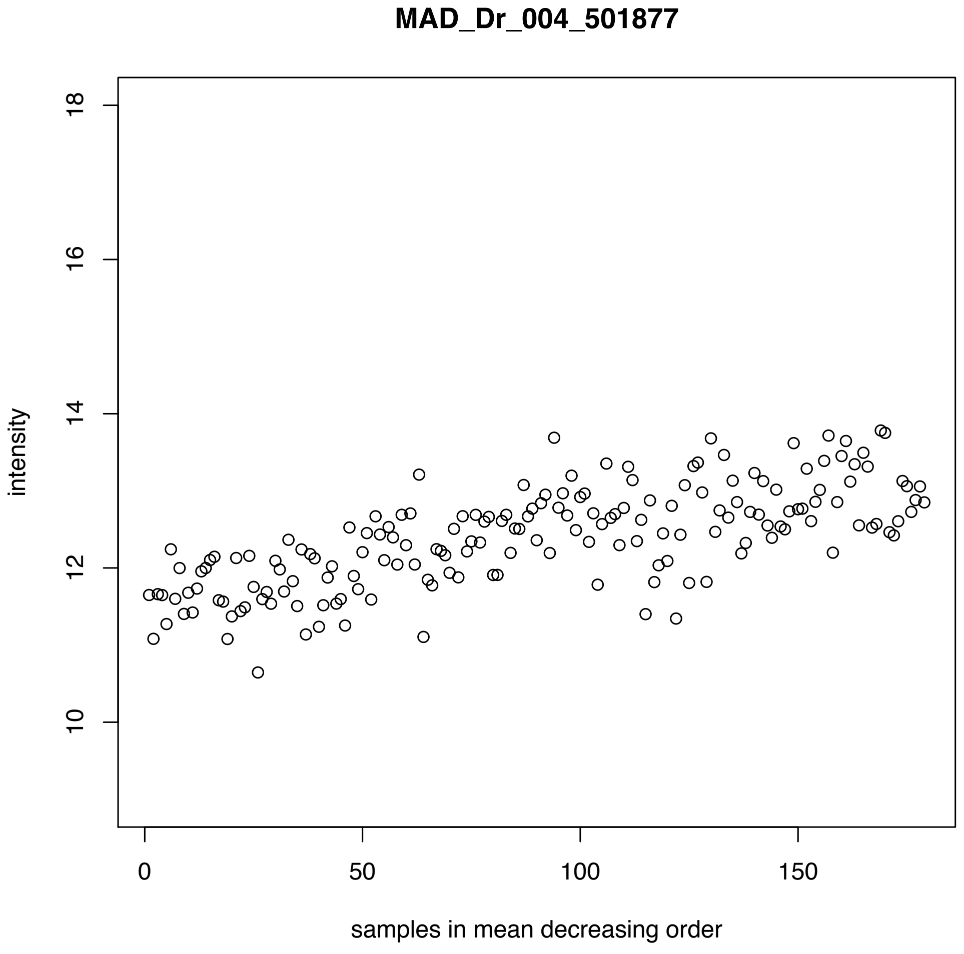
MAD_Dr_004_501877
intensity
samples in mean decreasing order
10
12
14
16
18
0
50
100
150

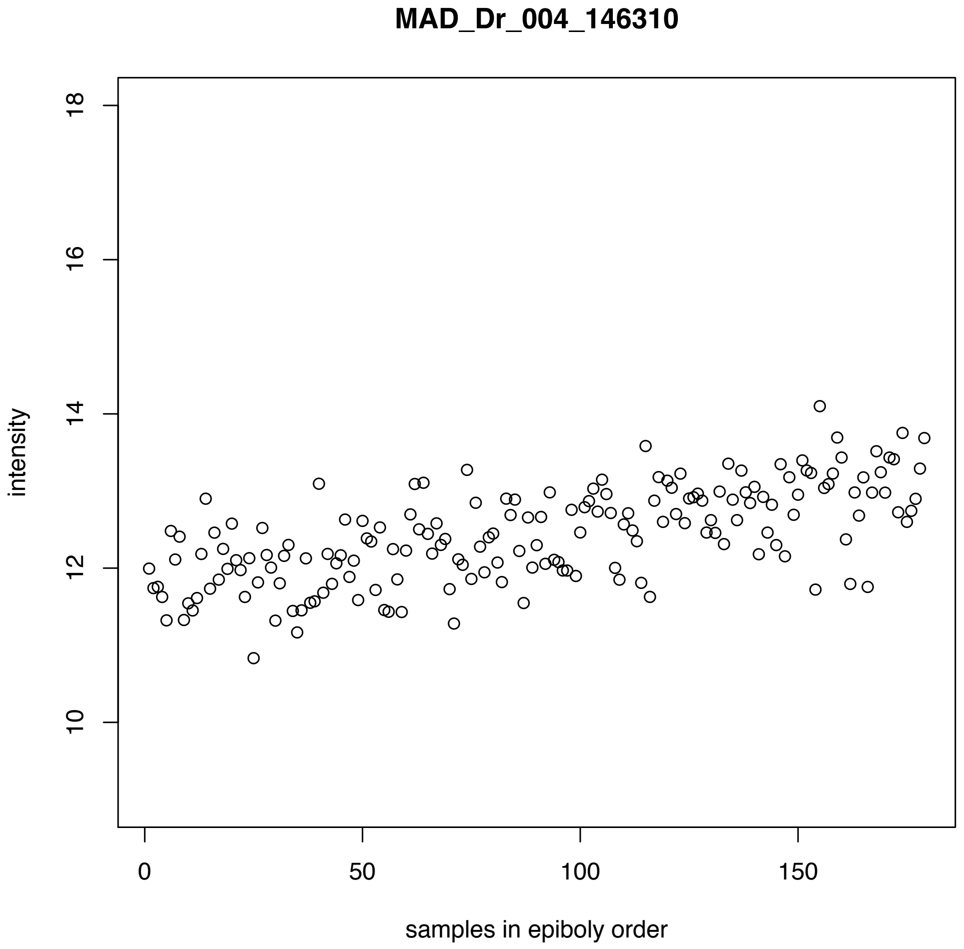
MAD_Dr_004_146310
intensity
10
12
14
16
18
0
50
100
150
samples in epiboly order

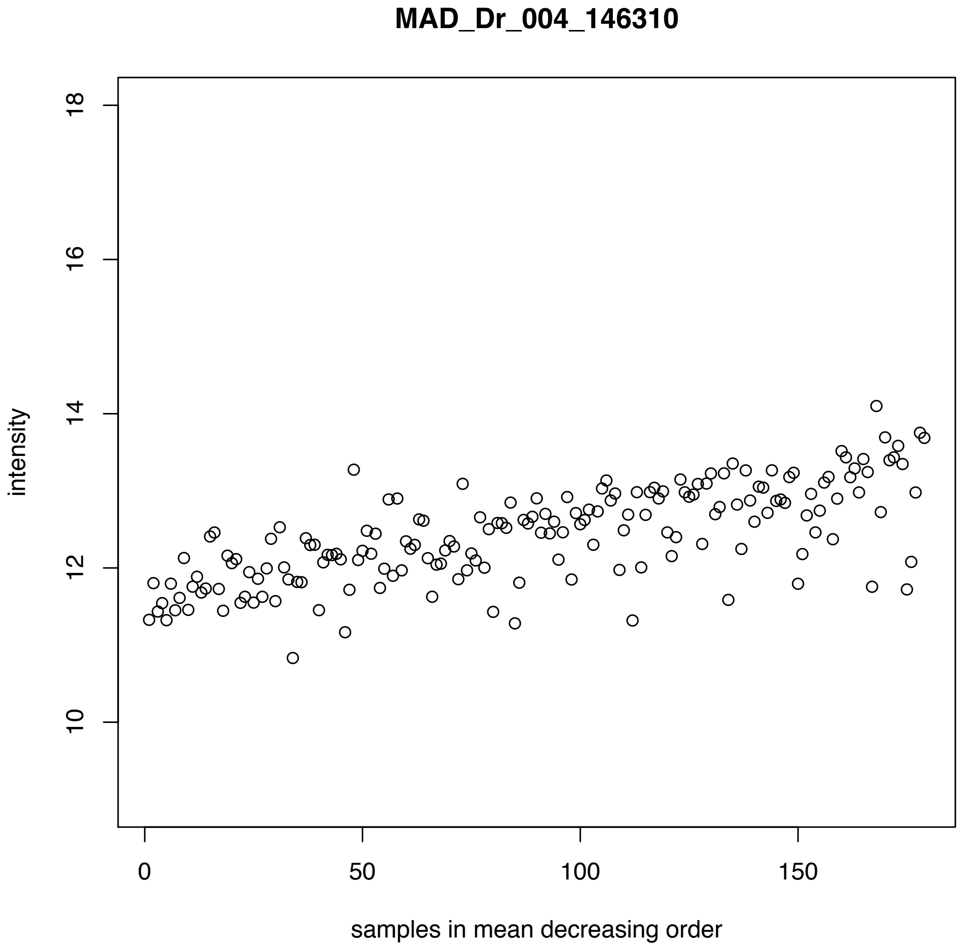
MAD_Dr_004_146310
intensity
10
12
14
16
18
0
50
100
150
samples in mean decreasing order

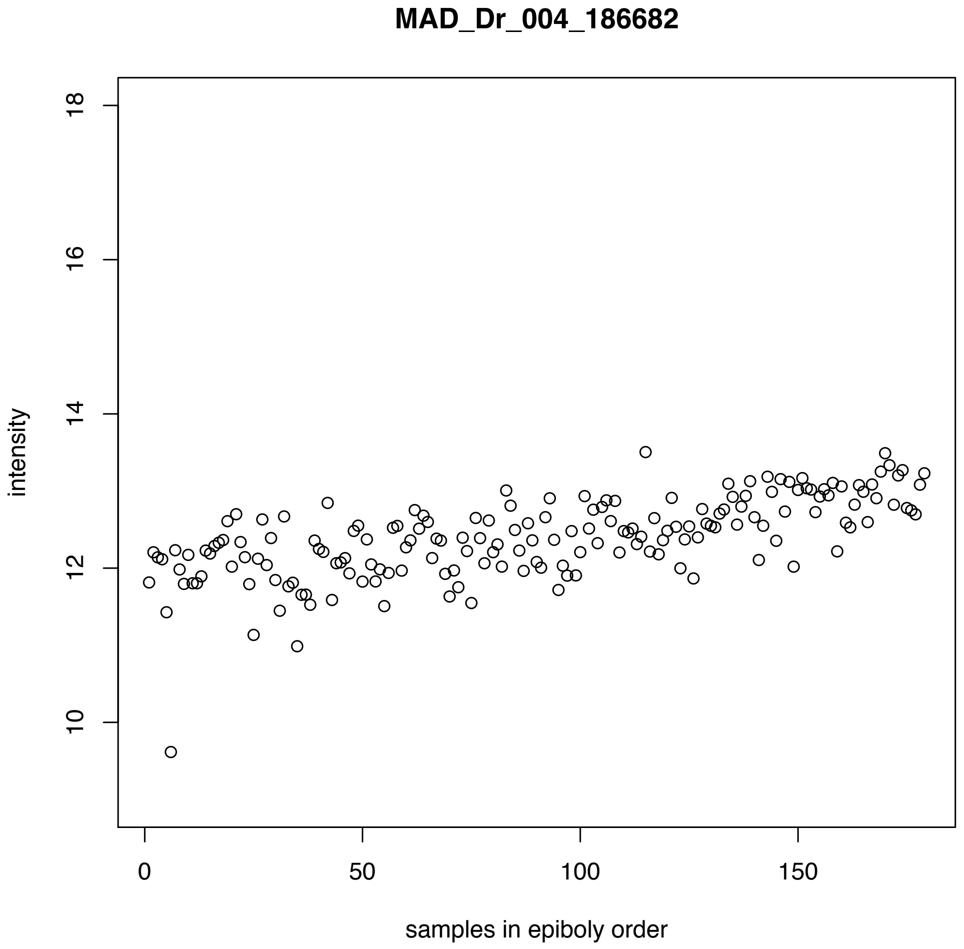
MAD_Dr_004_186682
intensity
samples in epiboly order
10
12
14
16
18
0
50
100
150

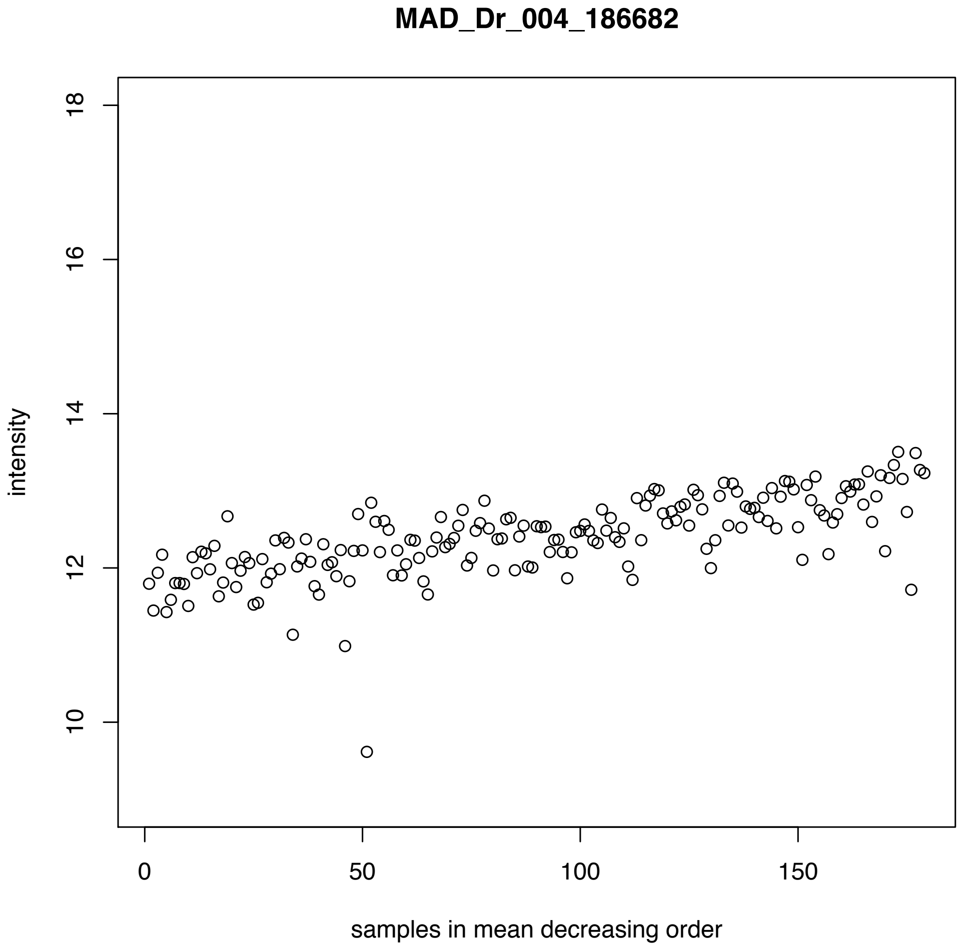
MAD_Dr_004_186682
intensity
samples in mean decreasing order
10
12
14
16
18
0
50
100
150

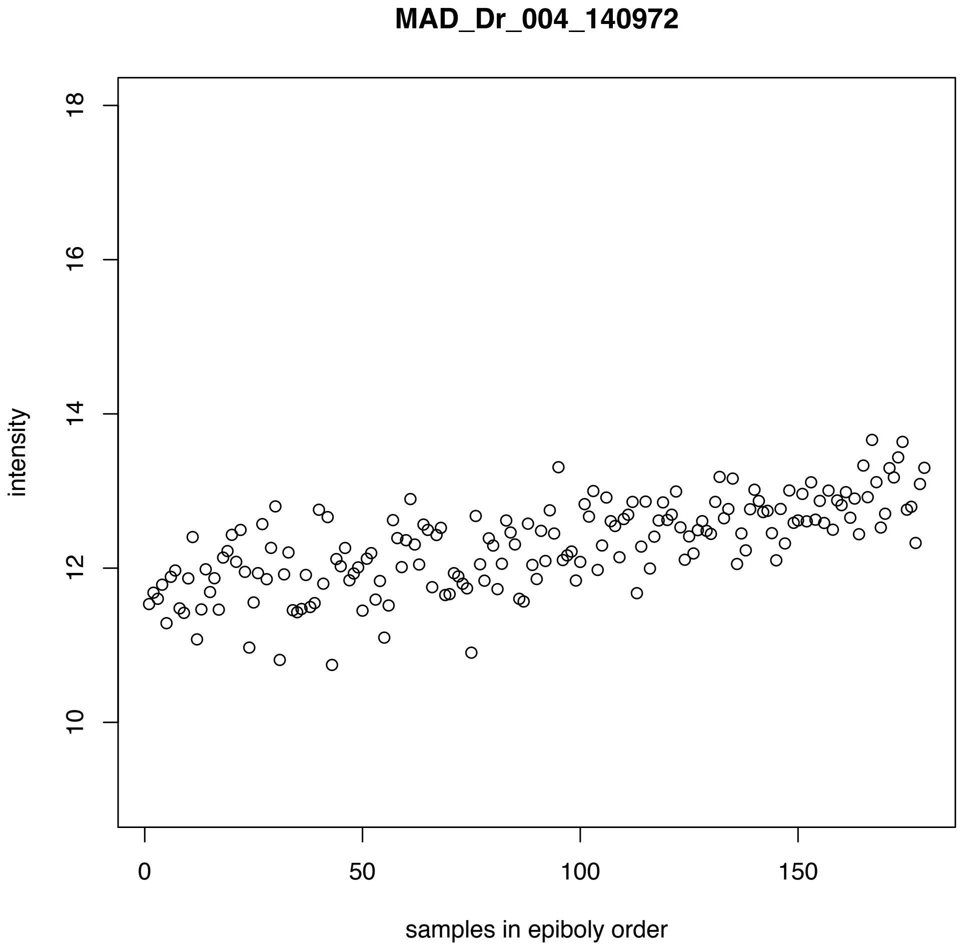

MAD_Dr_004_140972
intensity
samples in epiboly order

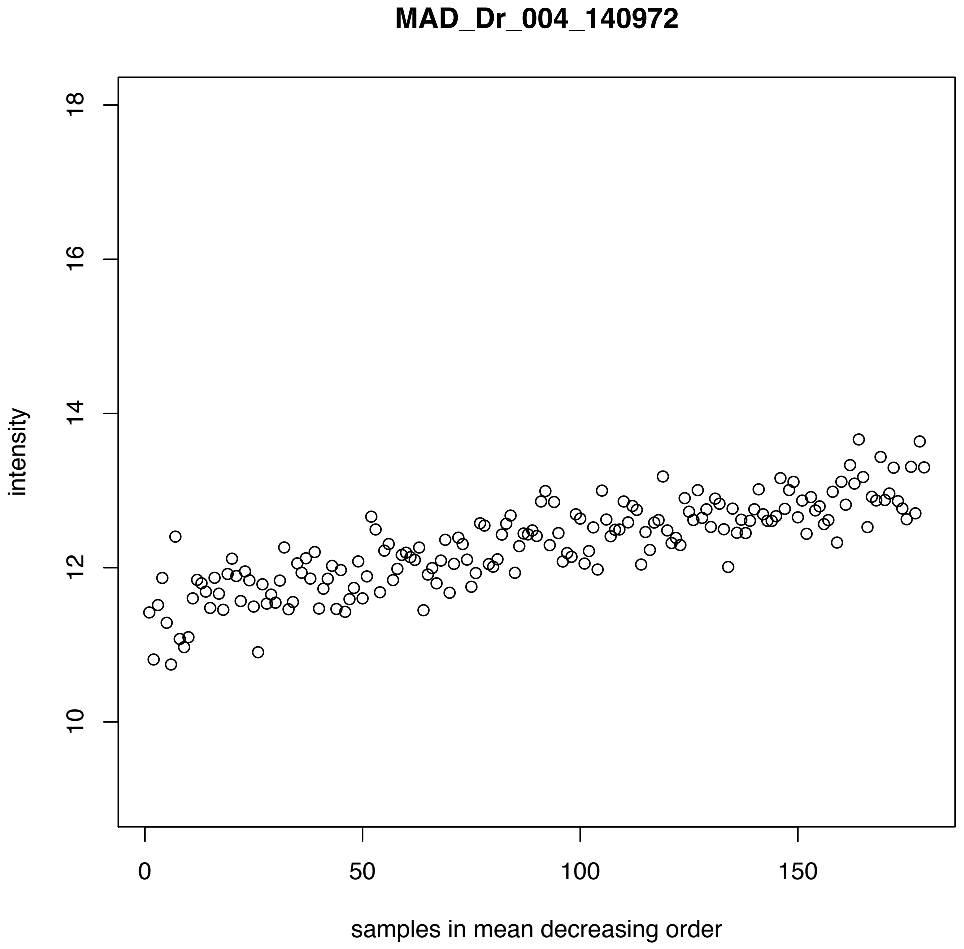

MAD_Dr_004_140972
intensity
samples in mean decreasing order

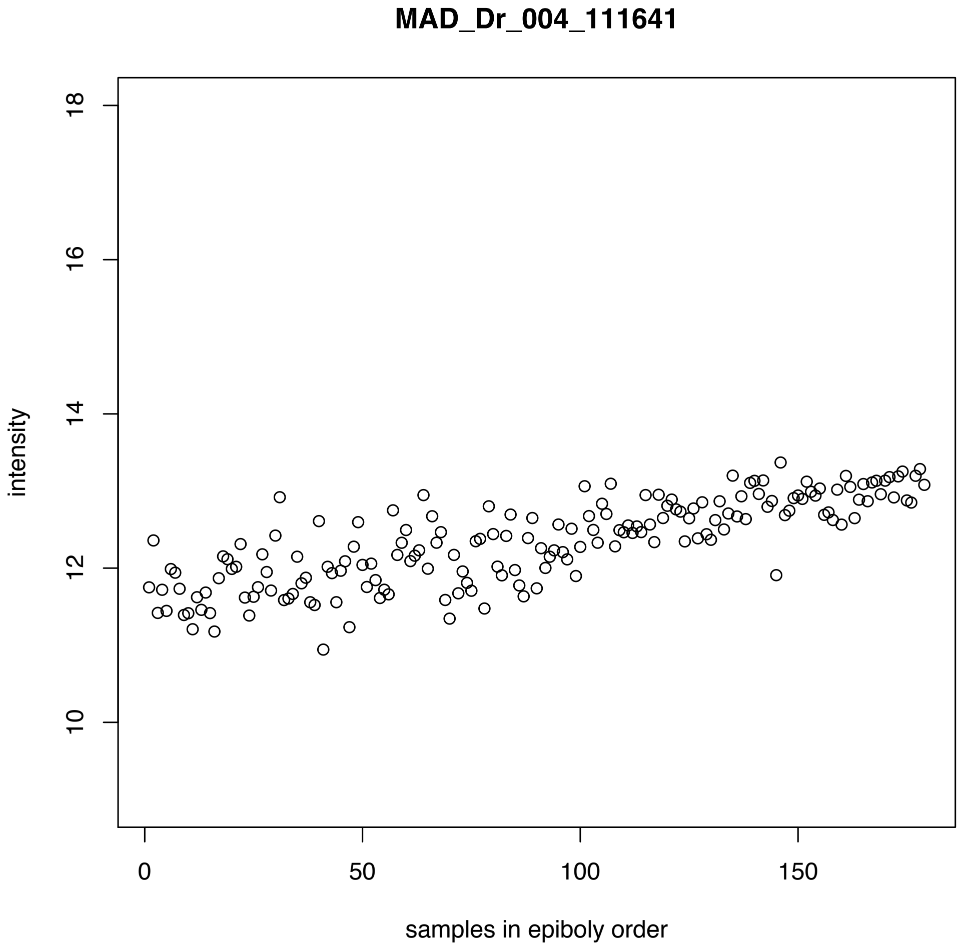
MAD_Dr_004_111641
intensity
10
12
14
16
18
0
50
100
150
samples in epiboly order

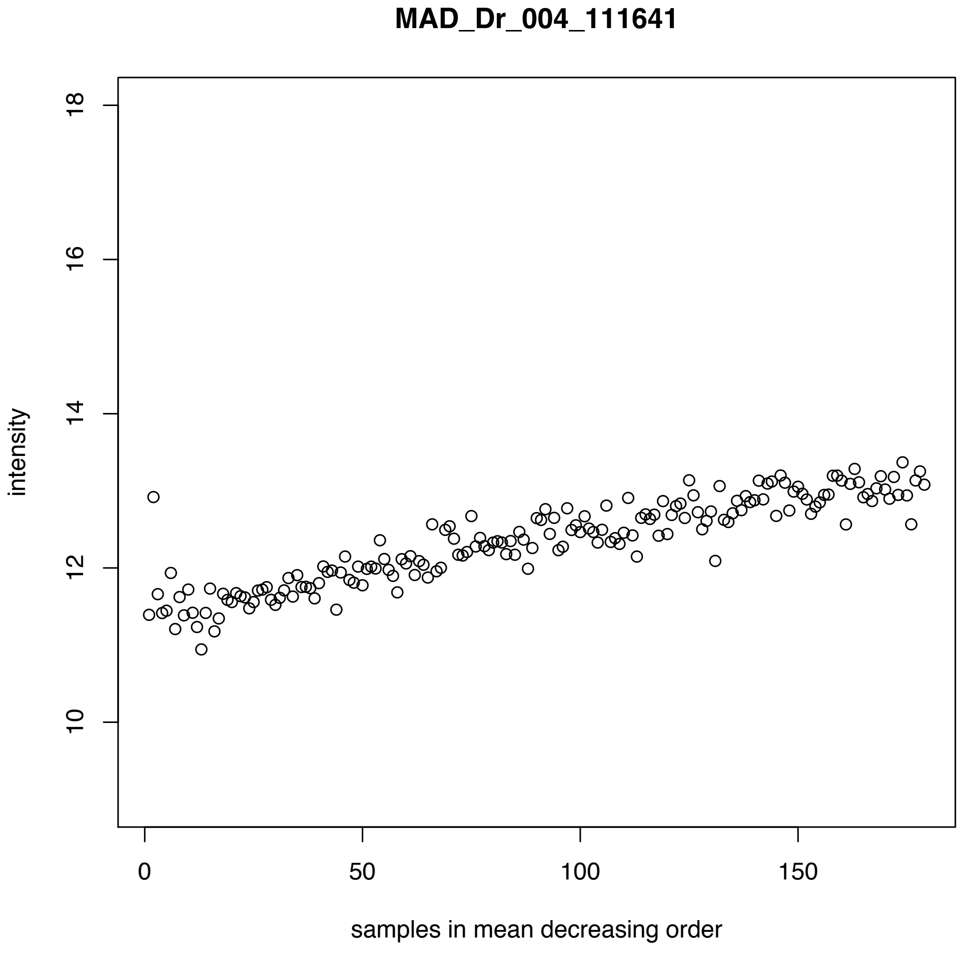
MAD_Dr_004_111641
intensity
10
12
14
16
18
0
50
100
150
samples in mean decreasing order

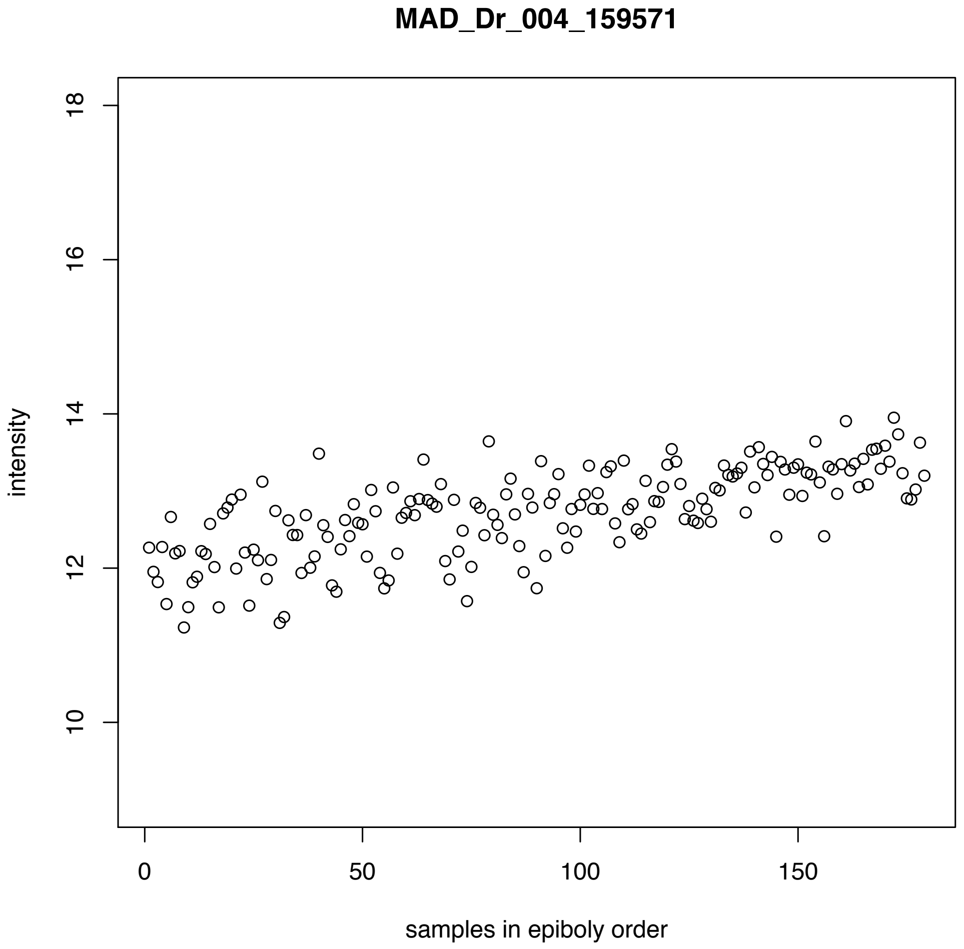
MAD_Dr_004_159571
intensity
10
12
14
16
18
0
50
100
150
samples in epiboly order

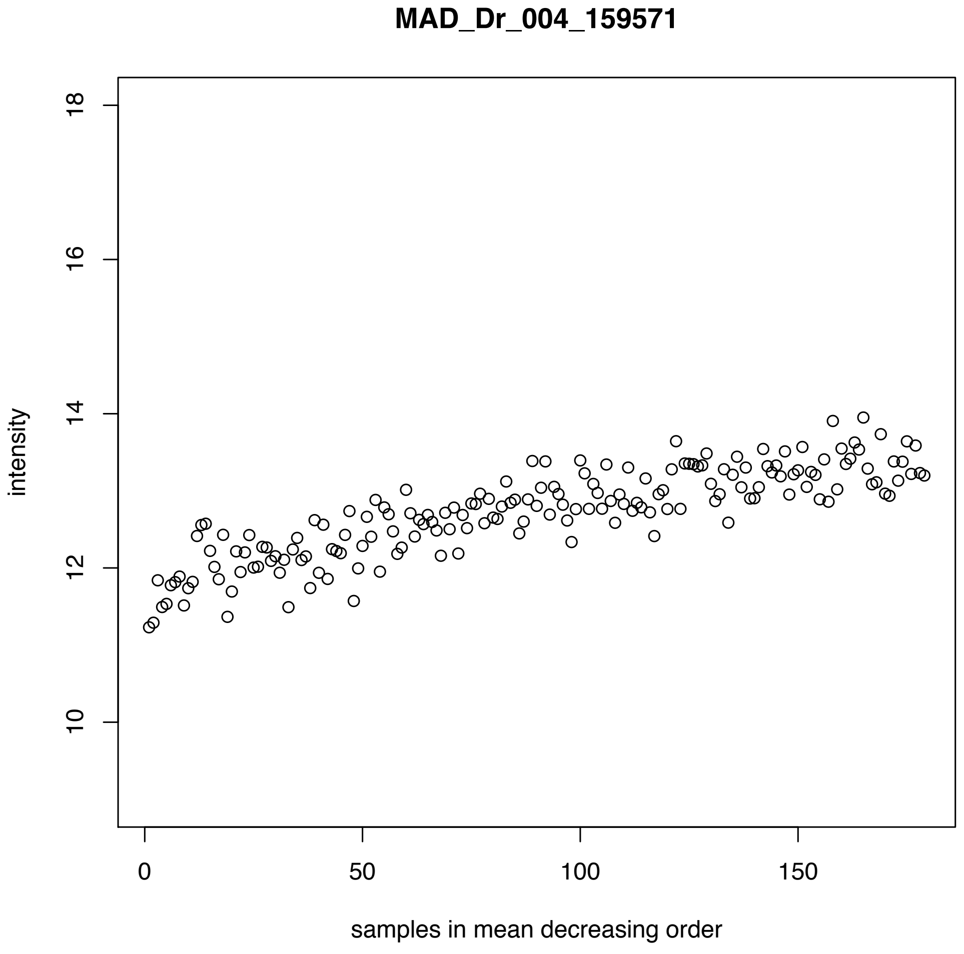
MAD_Dr_004_159571
intensity
10
12
14
16
18
0
50
100
150
samples in mean decreasing order

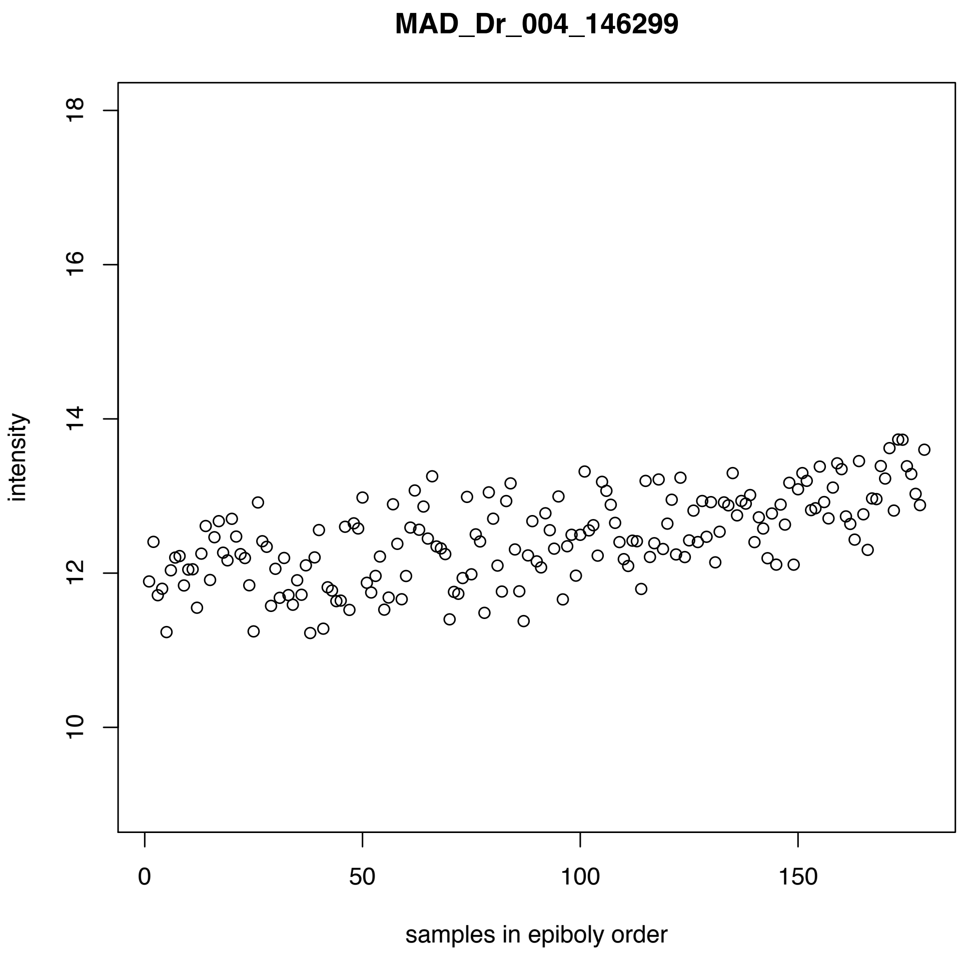
MAD_Dr_004_146299
intensity
samples in epiboly order
10
12
14
16
18
0
50
100
150

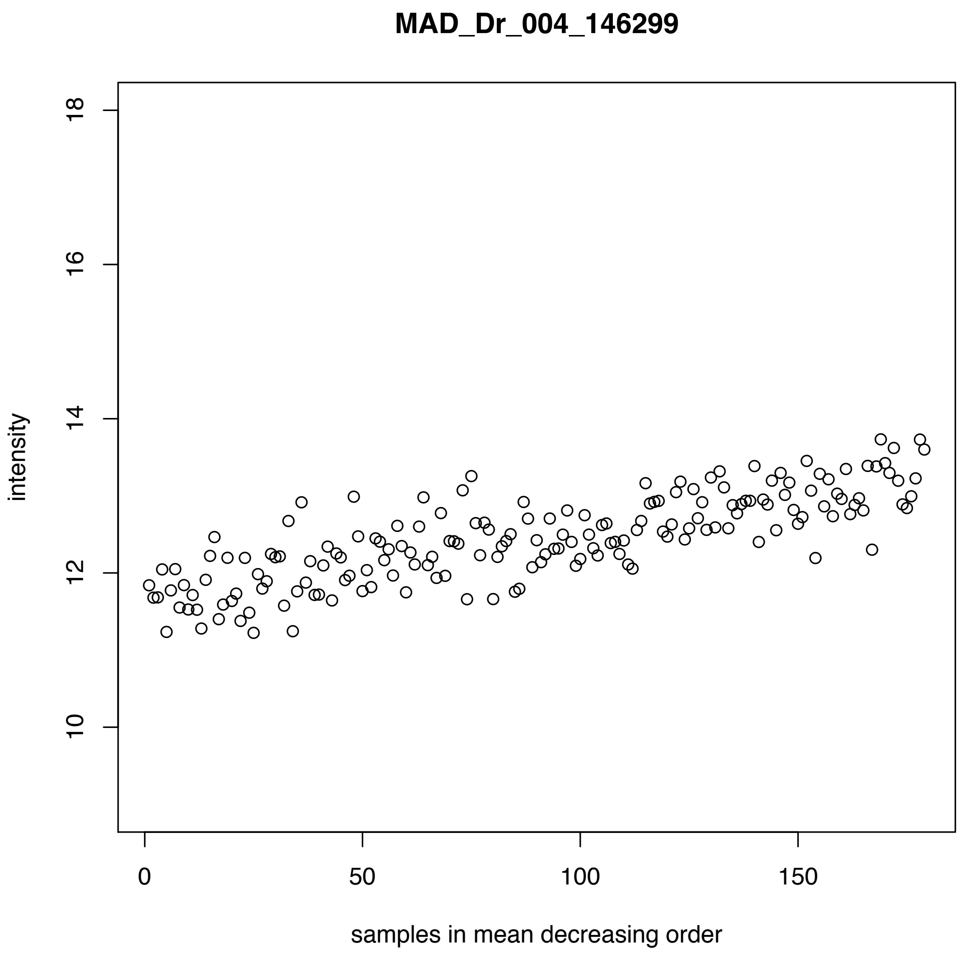
MAD_Dr_004_146299
intensity
samples in mean decreasing order
10
12
14
16
18
0
50
100
150

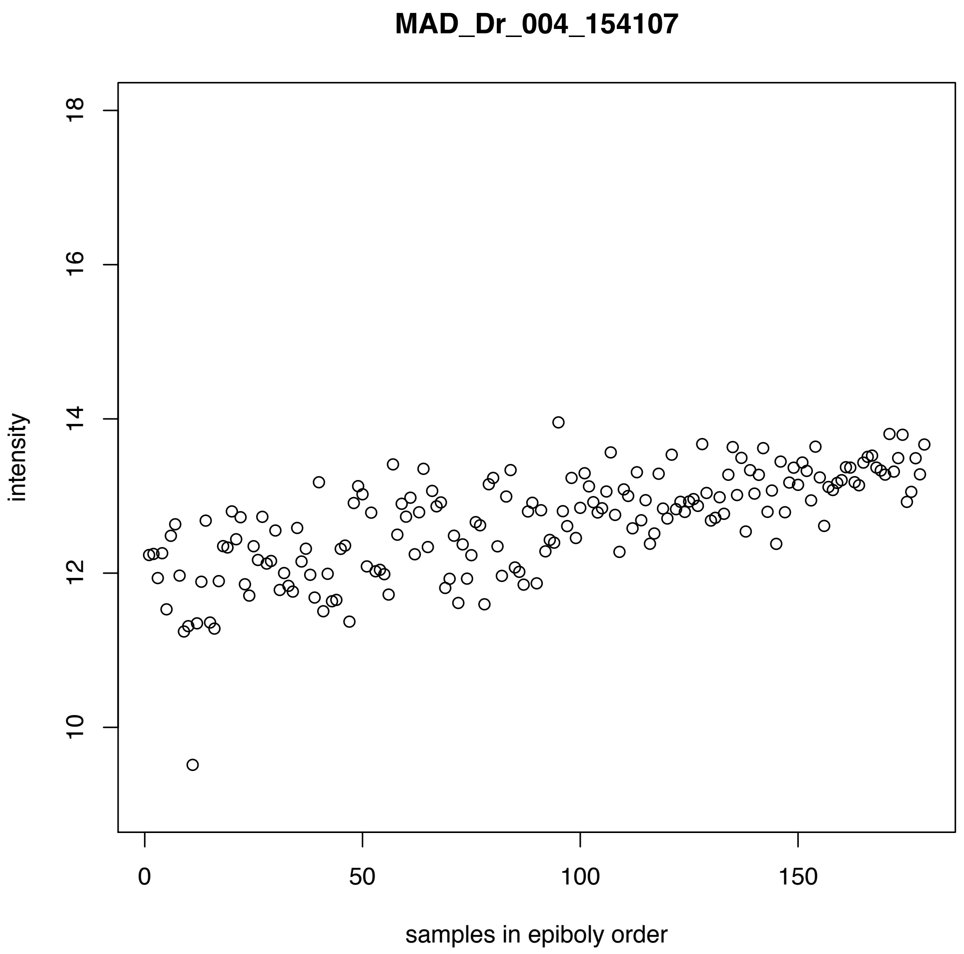
MAD_Dr_004_154107
intensity
10
12
14
16
18
0
50
100
150
samples in epiboly order

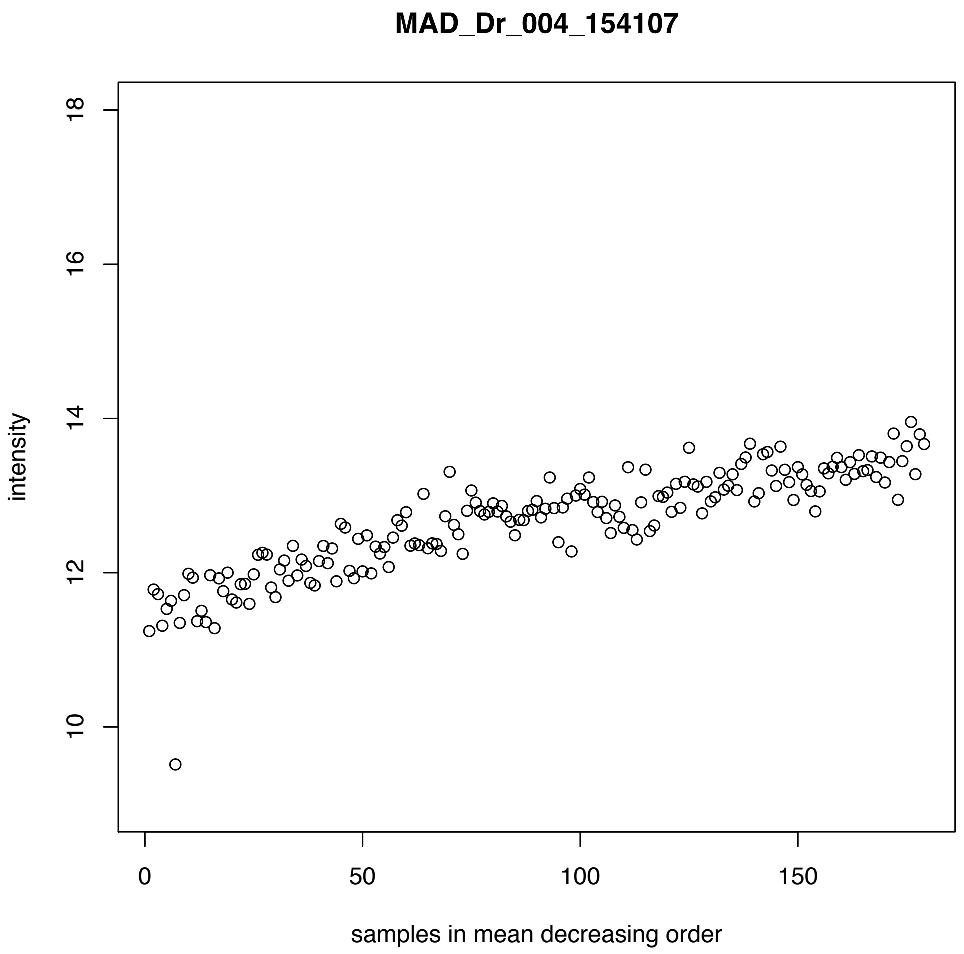
MAD_Dr_004_154107
intensity
10
12
14
16
18
0
50
100
150
samples in mean decreasing order

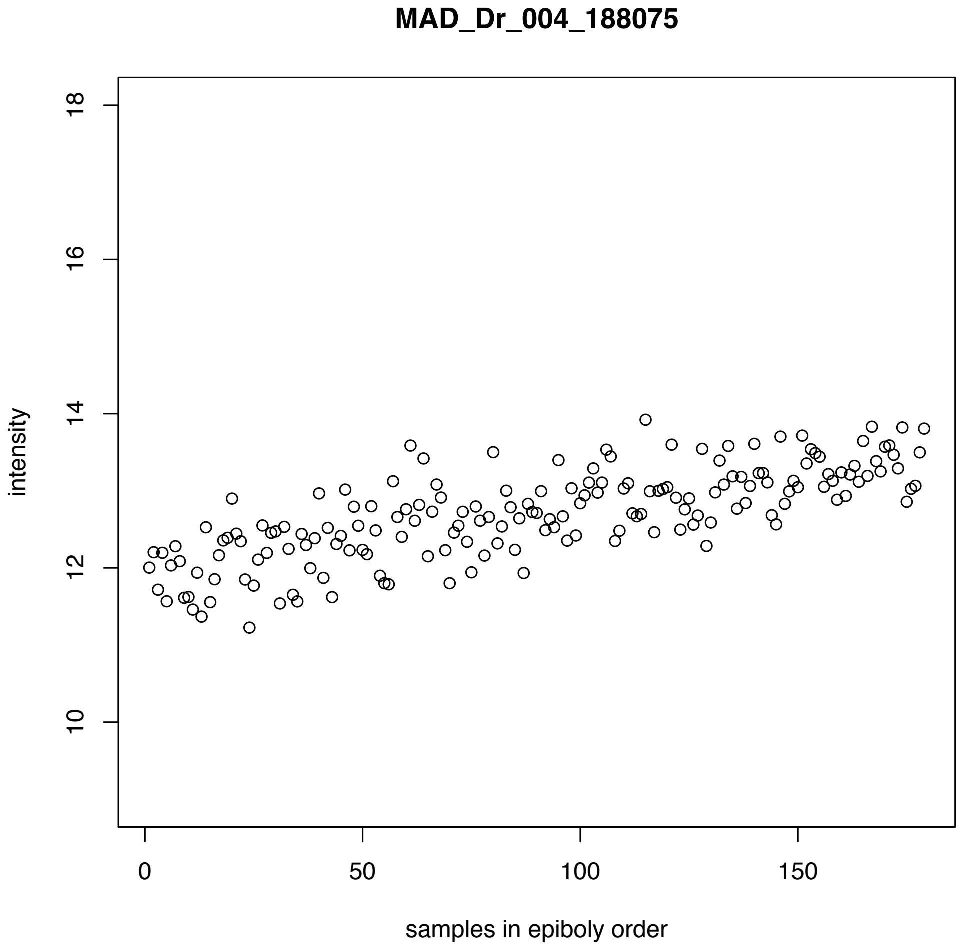
MAD_Dr_004_188075
intensity
samples in epiboly order
10
12
14
16
18
0
50
100
150

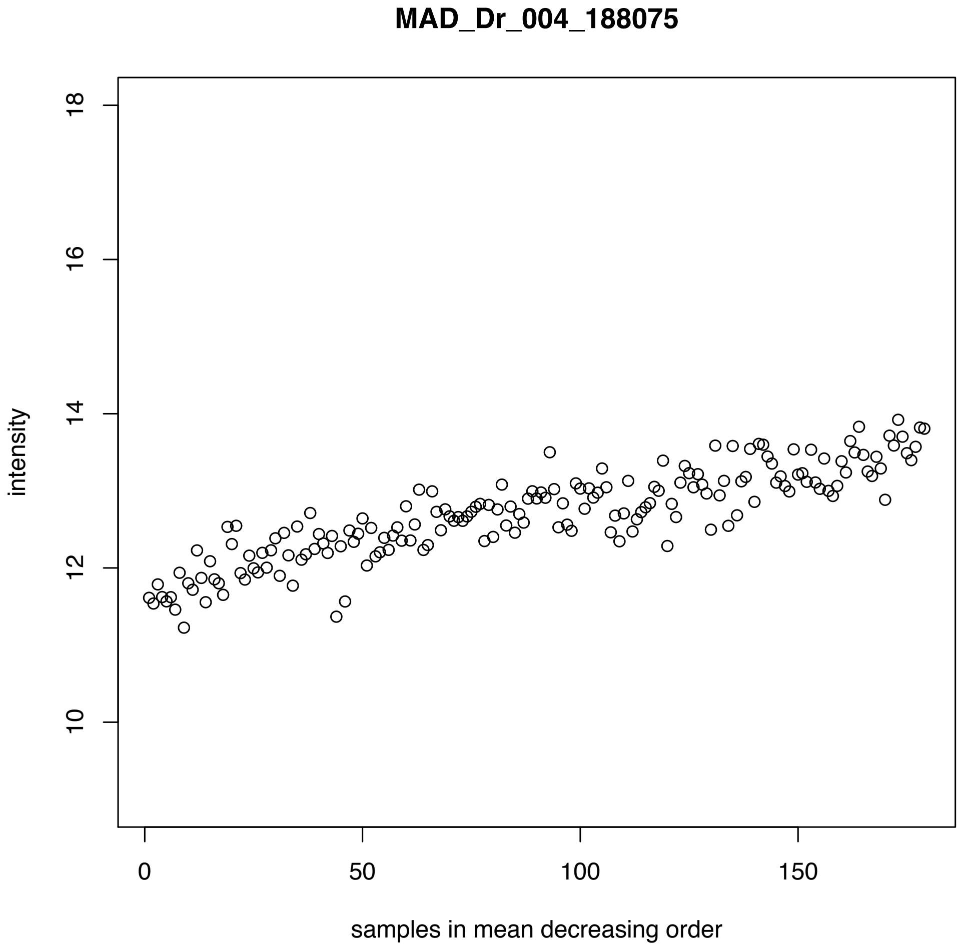
MAD_Dr_004_188075
intensity
samples in mean decreasing order
10
12
14
16
18
0
50
100
150

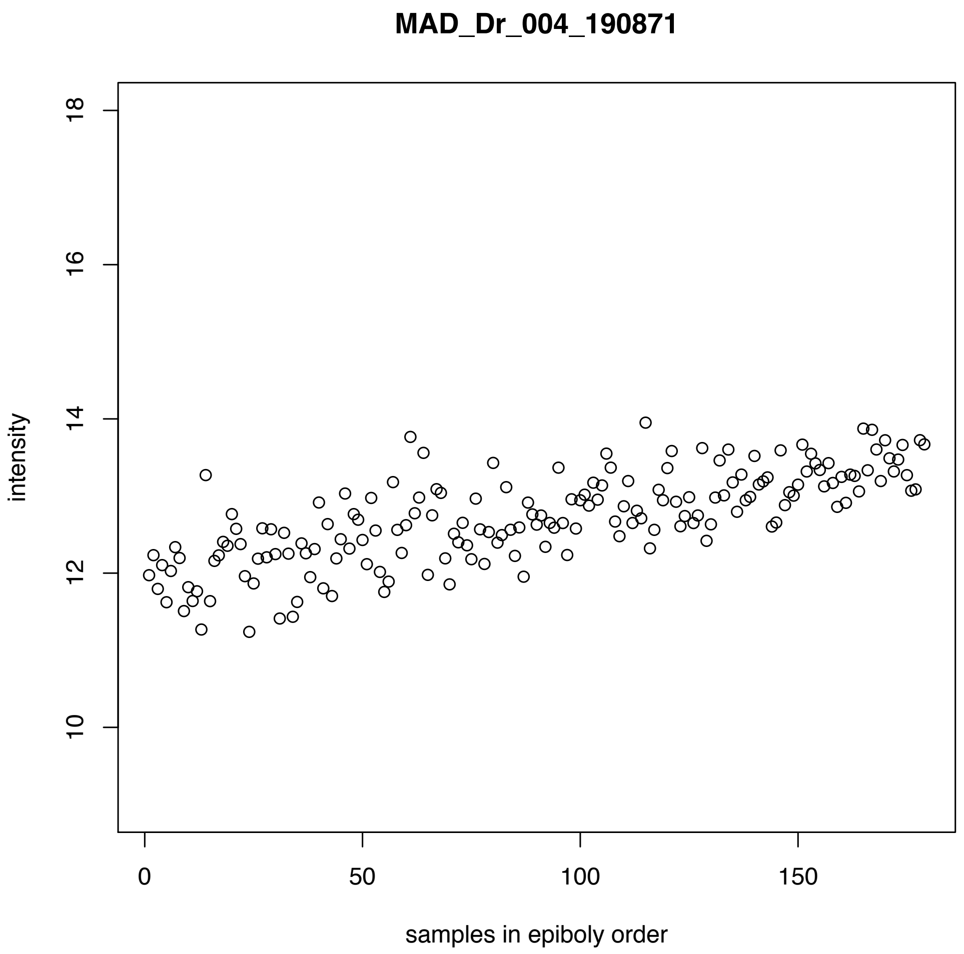
MAD_Dr_004_190871
intensity
samples in epiboly order
10
12
14
16
18
0
50
100
150

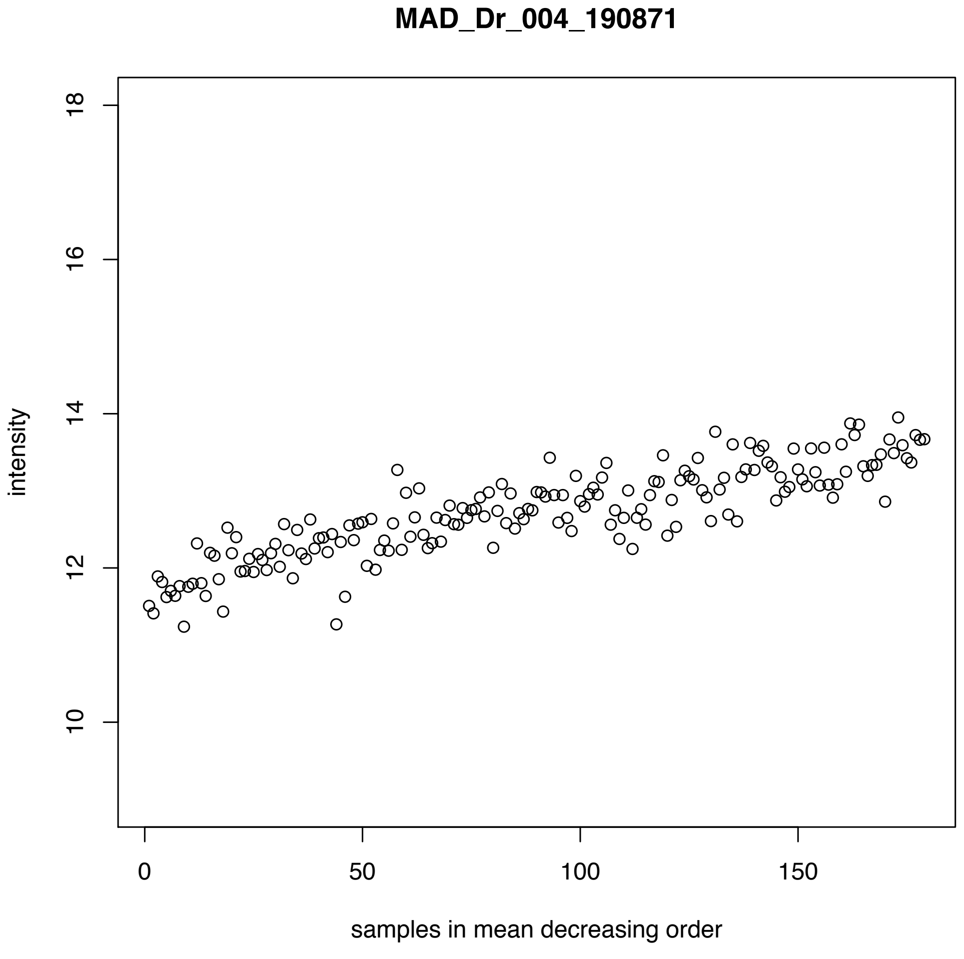
MAD_Dr_004_190871
intensity
samples in mean decreasing order
10
12
14
16
18
0
50
100
150

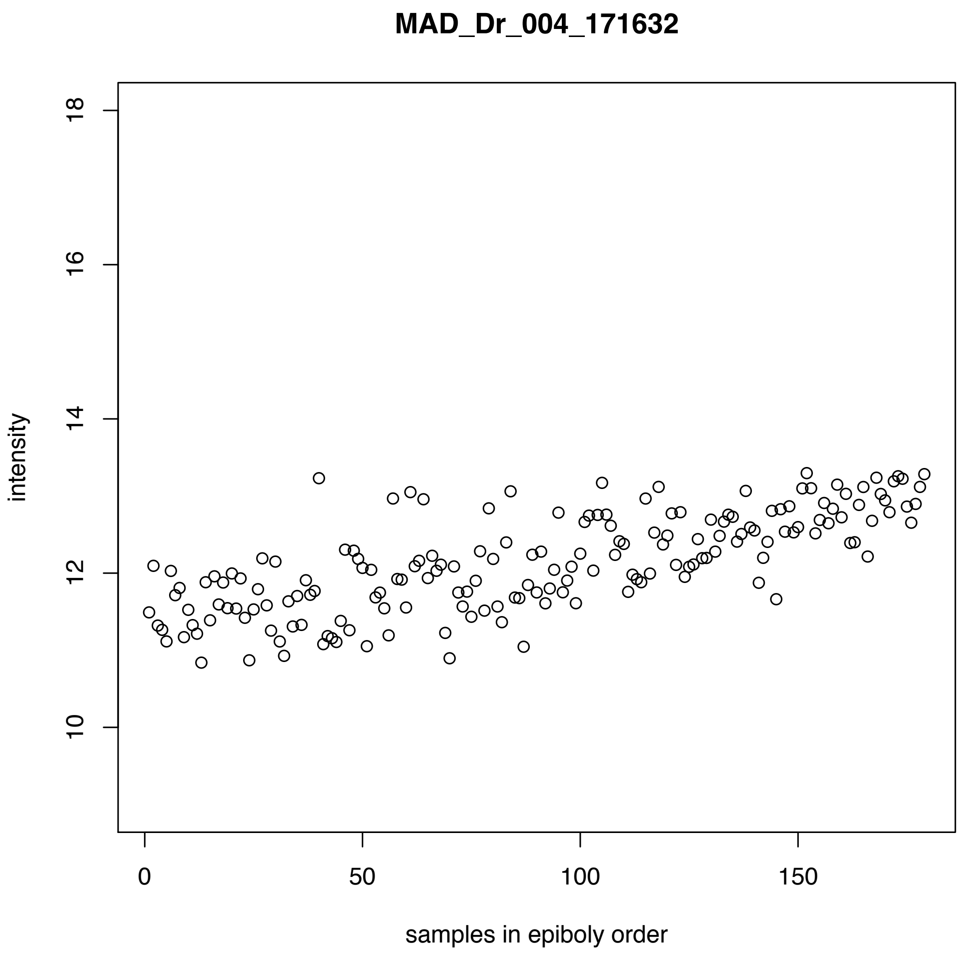
MAD_Dr_004_171632
intensity
10
12
14
16
18
0
50
100
150
samples in epiboly order

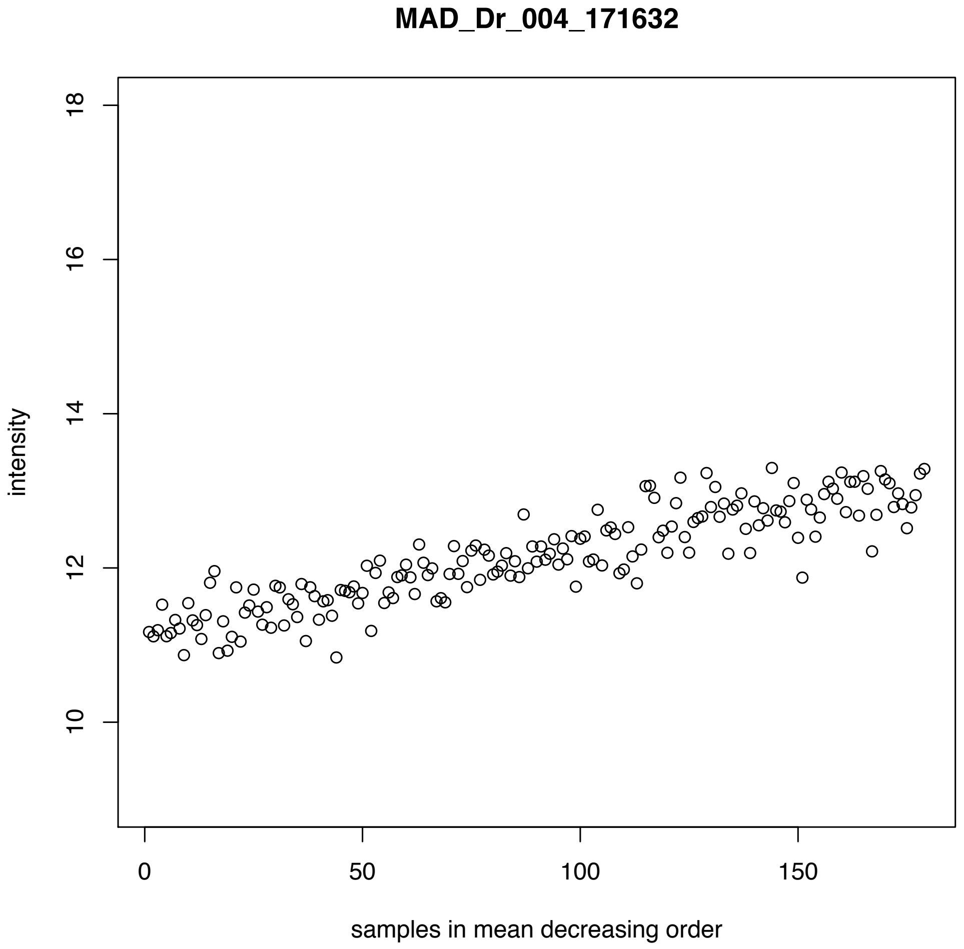
MAD_Dr_004_171632
intensity
10
12
14
16
18
0
50
100
150
samples in mean decreasing order

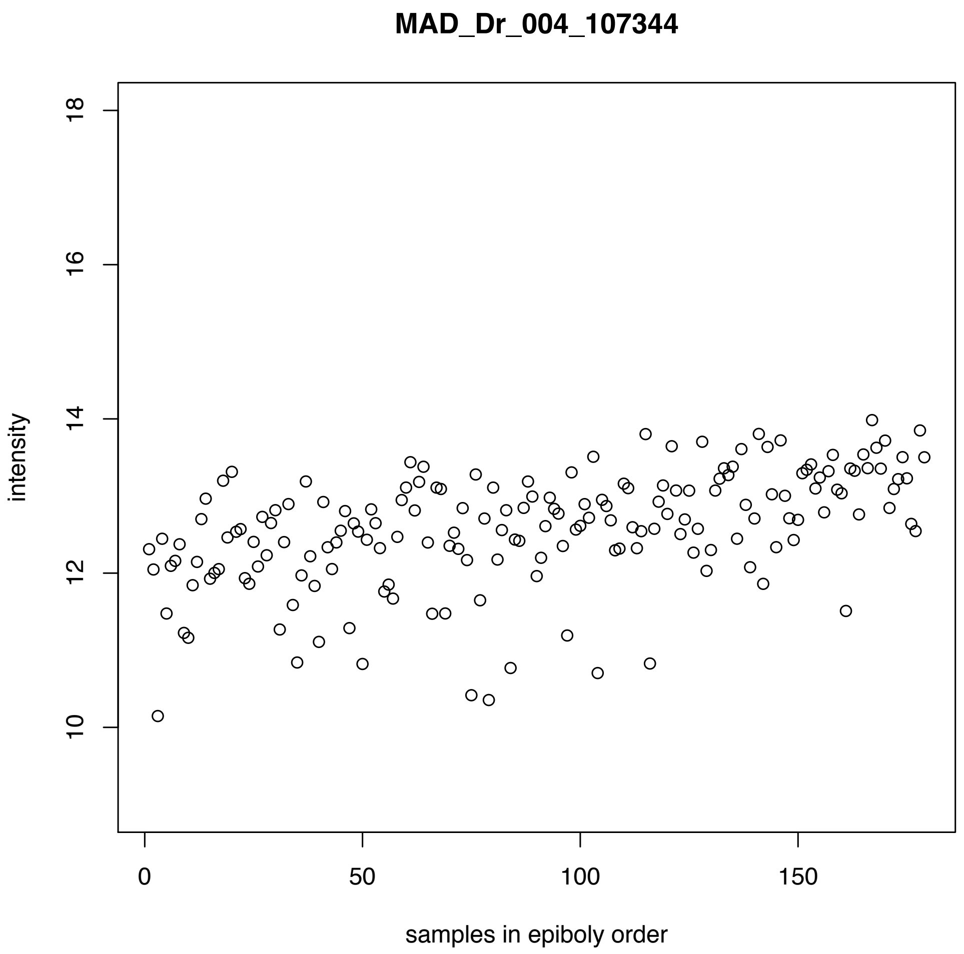

MAD_Dr_004_107344
intensity
samples in epiboly order

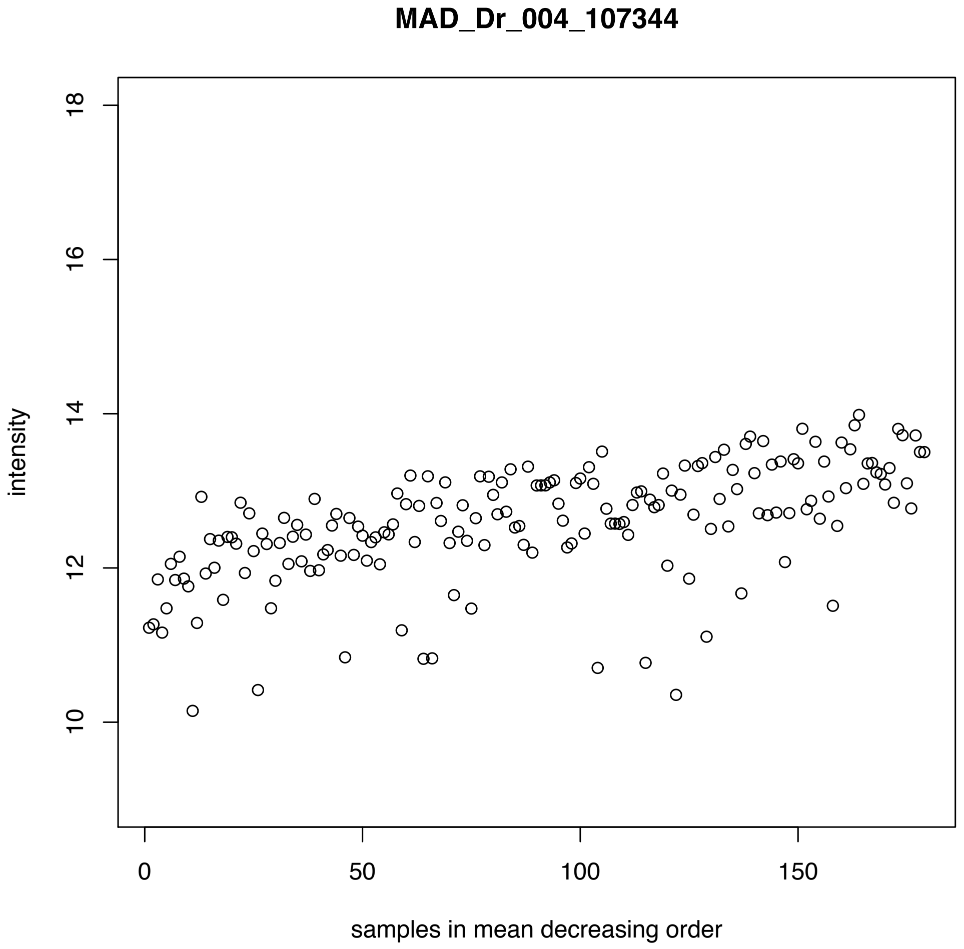

MAD_Dr_004_107344
intensity
samples in mean decreasing order

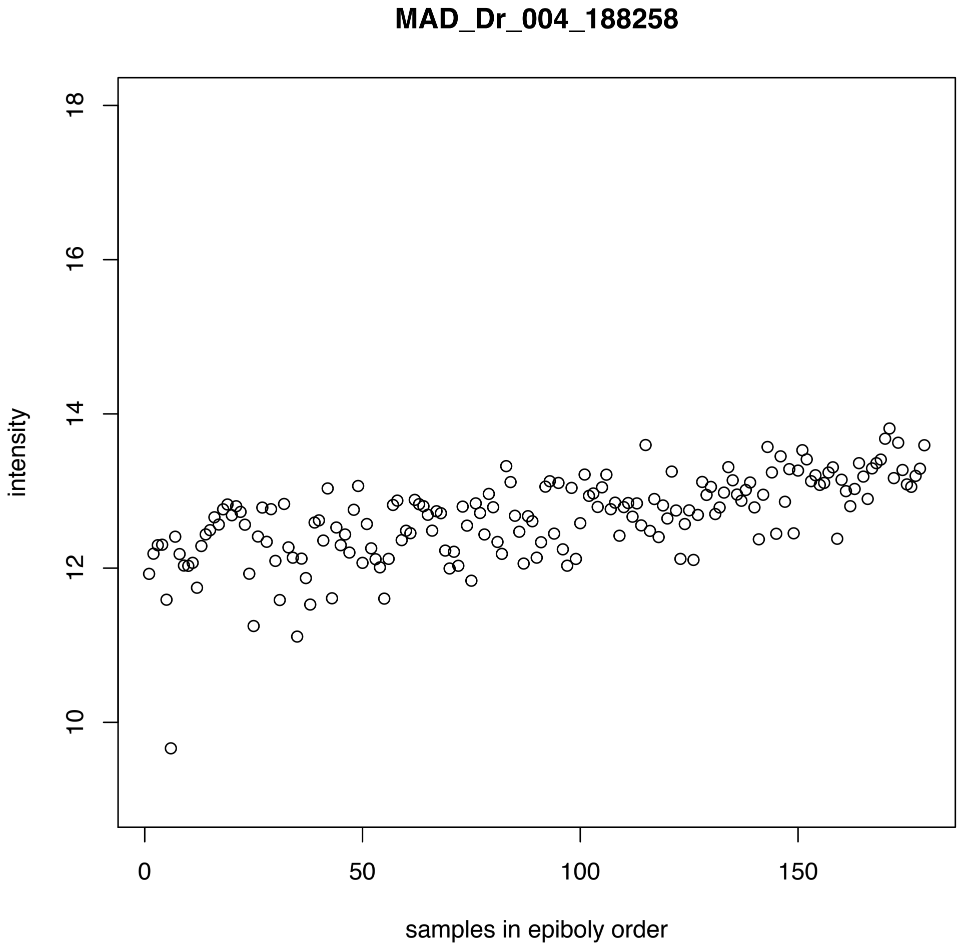
MAD_Dr_004_188258
intensity
18
16
14
12
10
0
50
100
150
samples in epiboly order

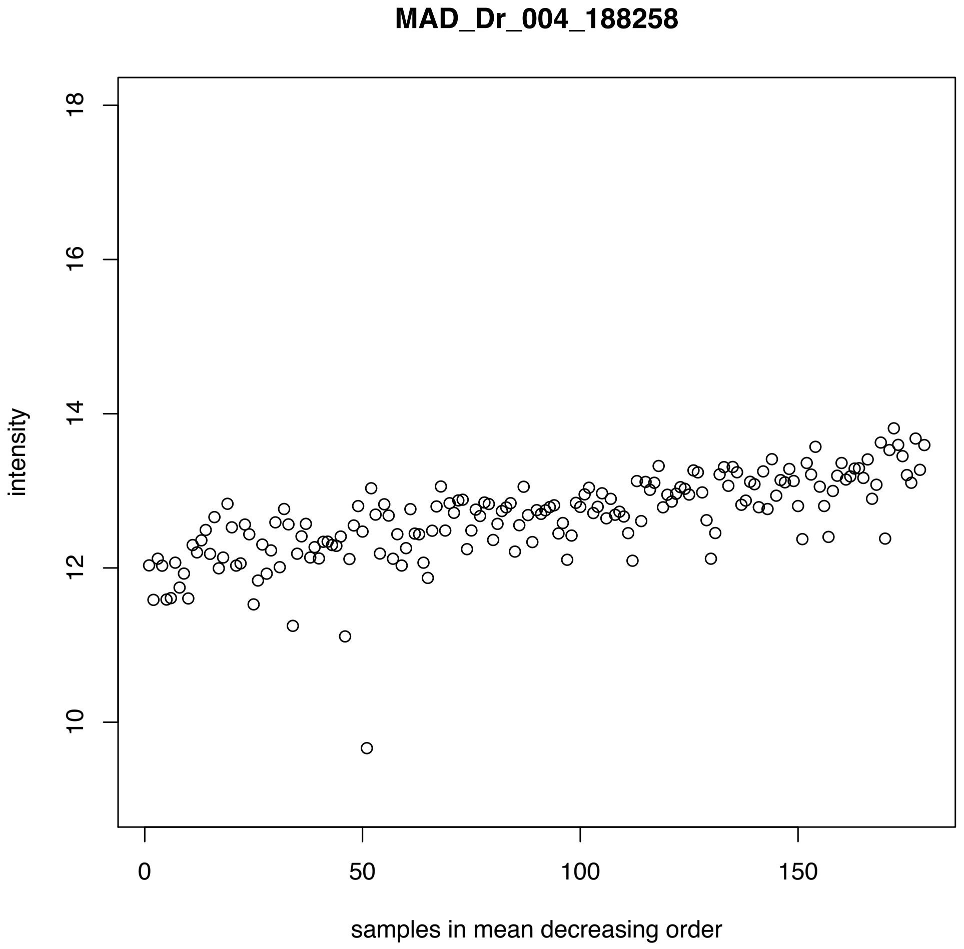
MAD_Dr_004_188258
intensity
18
16
14
12
10
0
50
100
150
samples in mean decreasing order

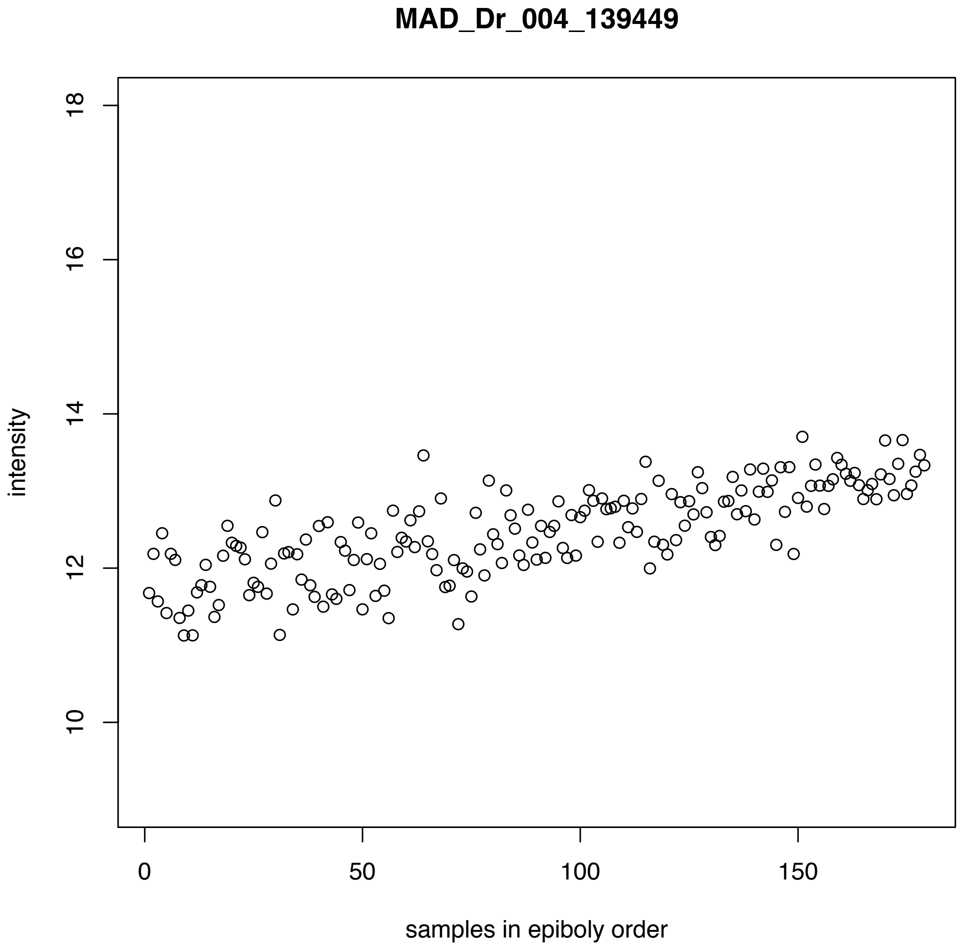
MAD_Dr_004_139449
intensity
10
12
14
16
18
0
50
100
150
samples in epiboly order

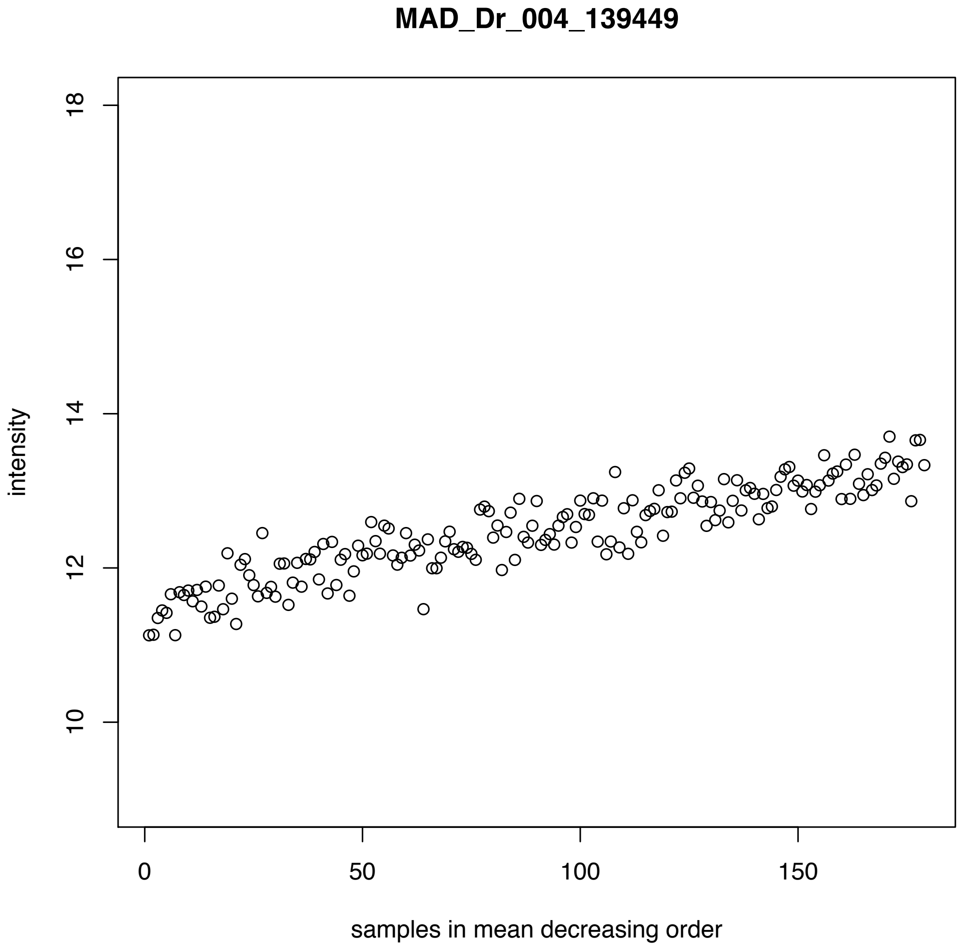
MAD_Dr_004_139449
intensity
10
12
14
16
18
0
50
100
150
samples in mean decreasing order

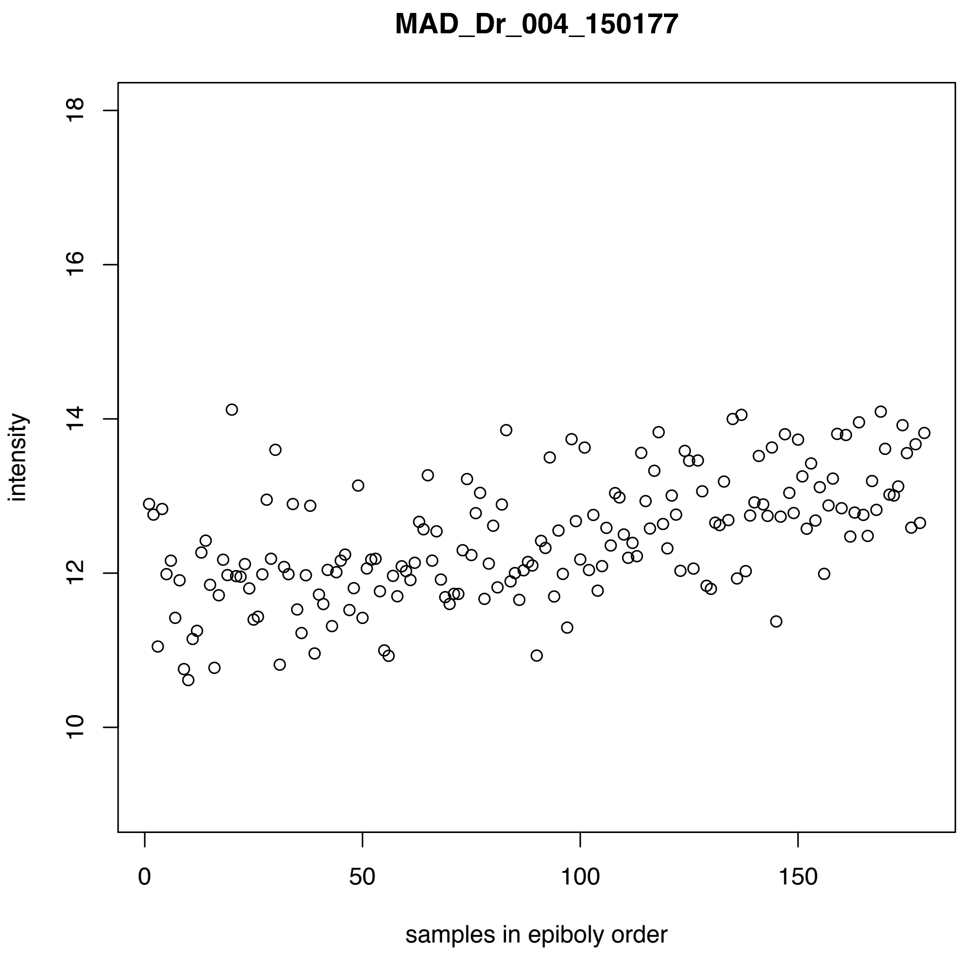
MAD_Dr_004_150177
intensity
10
12
14
16
18
0
50
100
150
samples in epiboly order

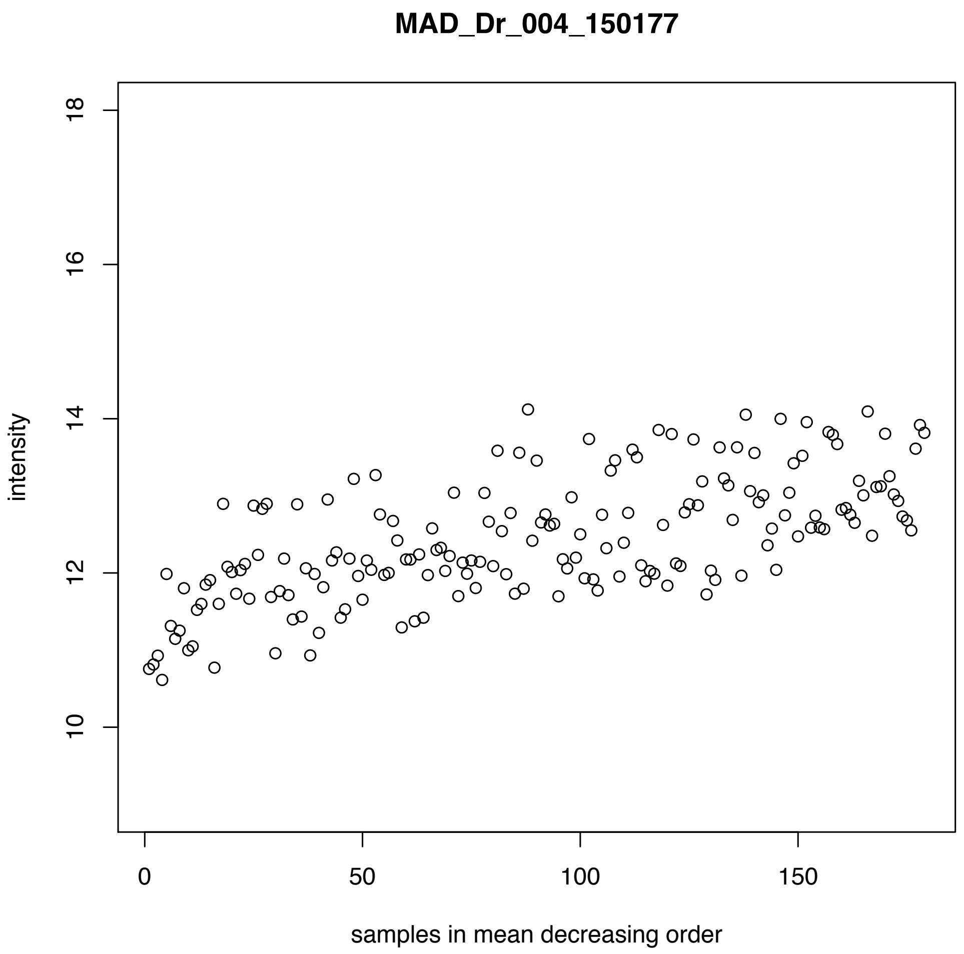
MAD_Dr_004_150177
intensity
10
12
14
16
18
0
50
100
150
samples in mean decreasing order

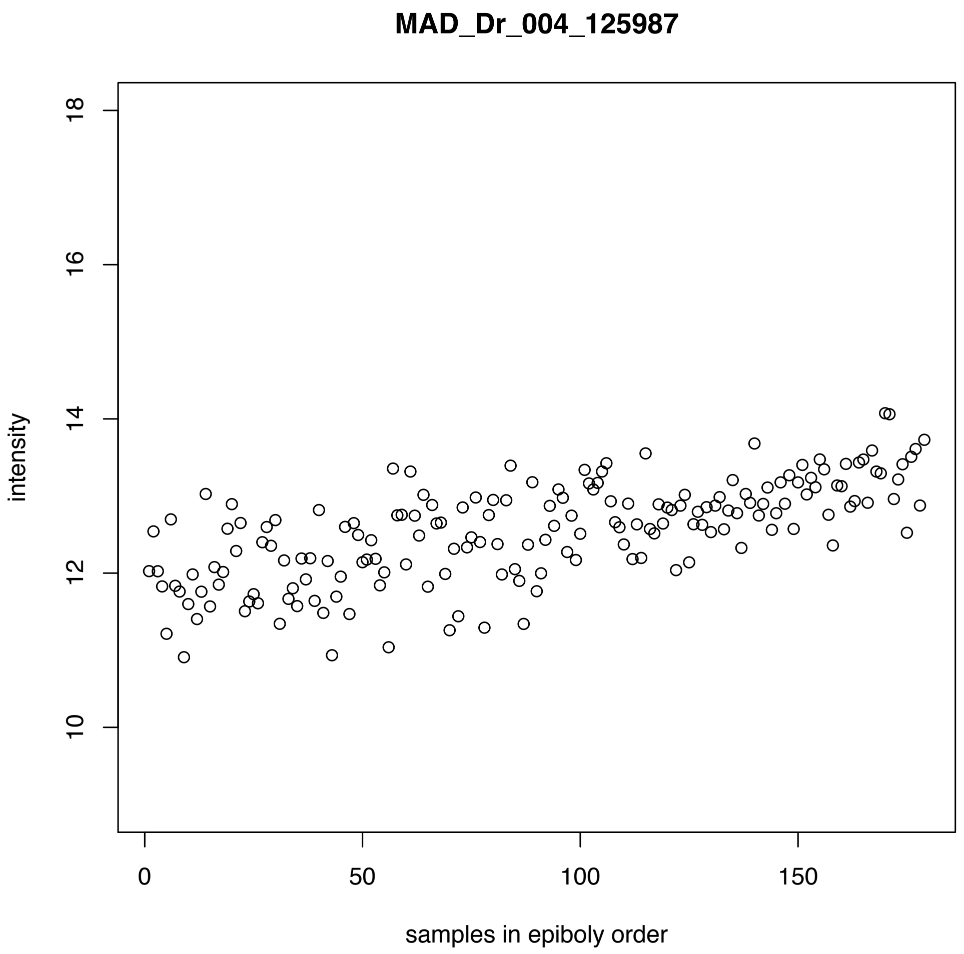
MAD_Dr_004_125987
intensity
samples in epiboly order
0
50
100
150
10
12
14
16
18

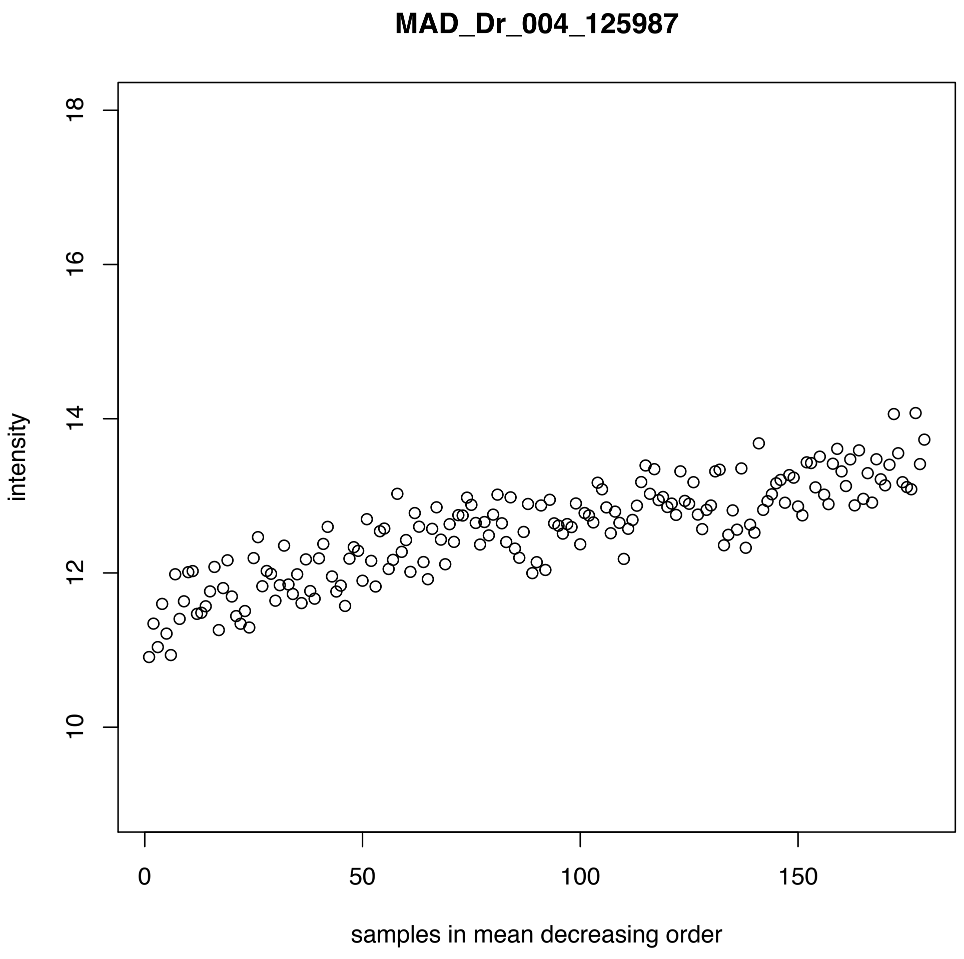
MAD_Dr_004_125987
intensity
samples in mean decreasing order
0
50
100
150
10
12
14
16
18

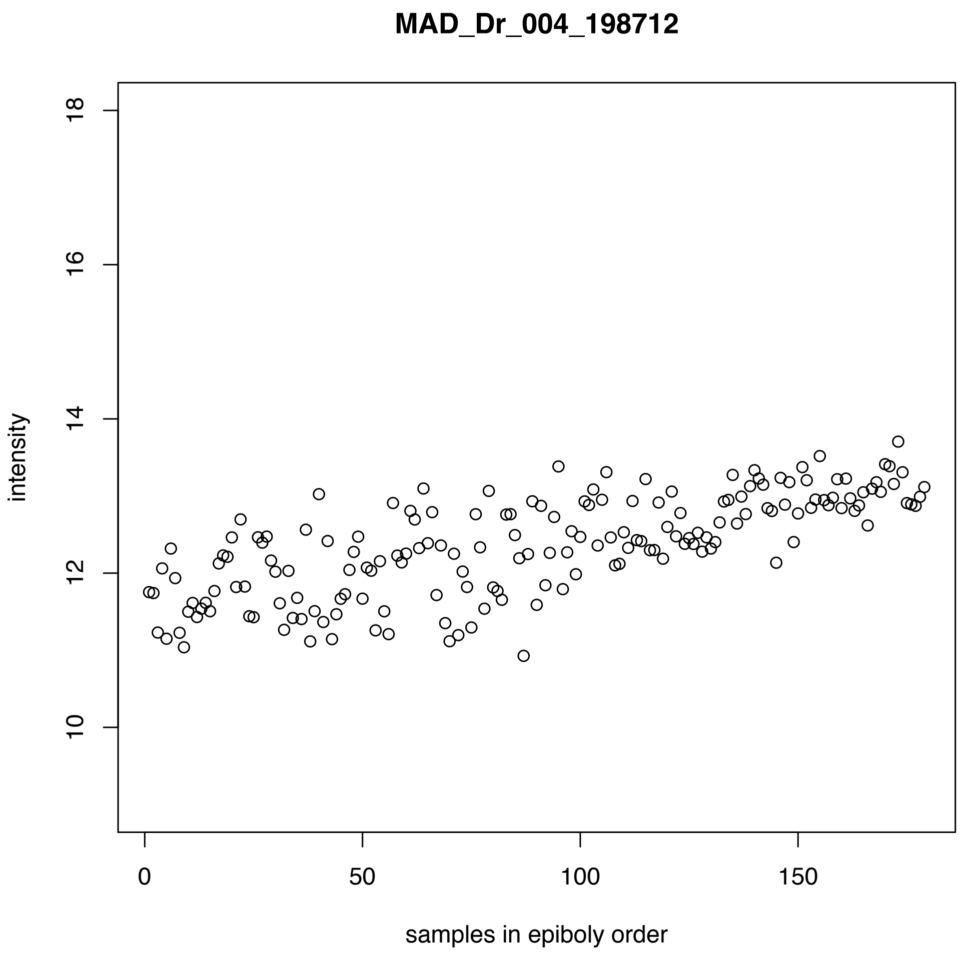
MAD_Dr_004_198712
intensity
10
12
14
16
18
0
50
100
150
samples in epiboly order

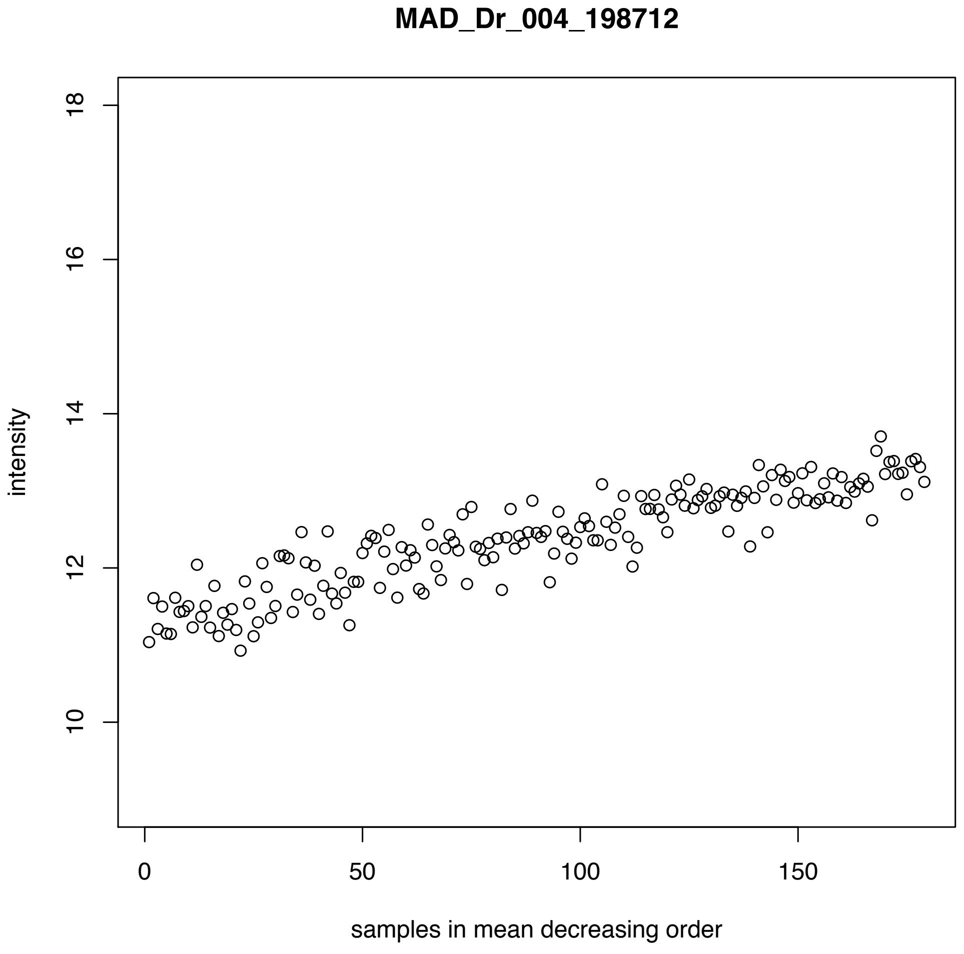
MAD_Dr_004_198712
intensity
10
12
14
16
18
0
50
100
150
samples in mean decreasing order

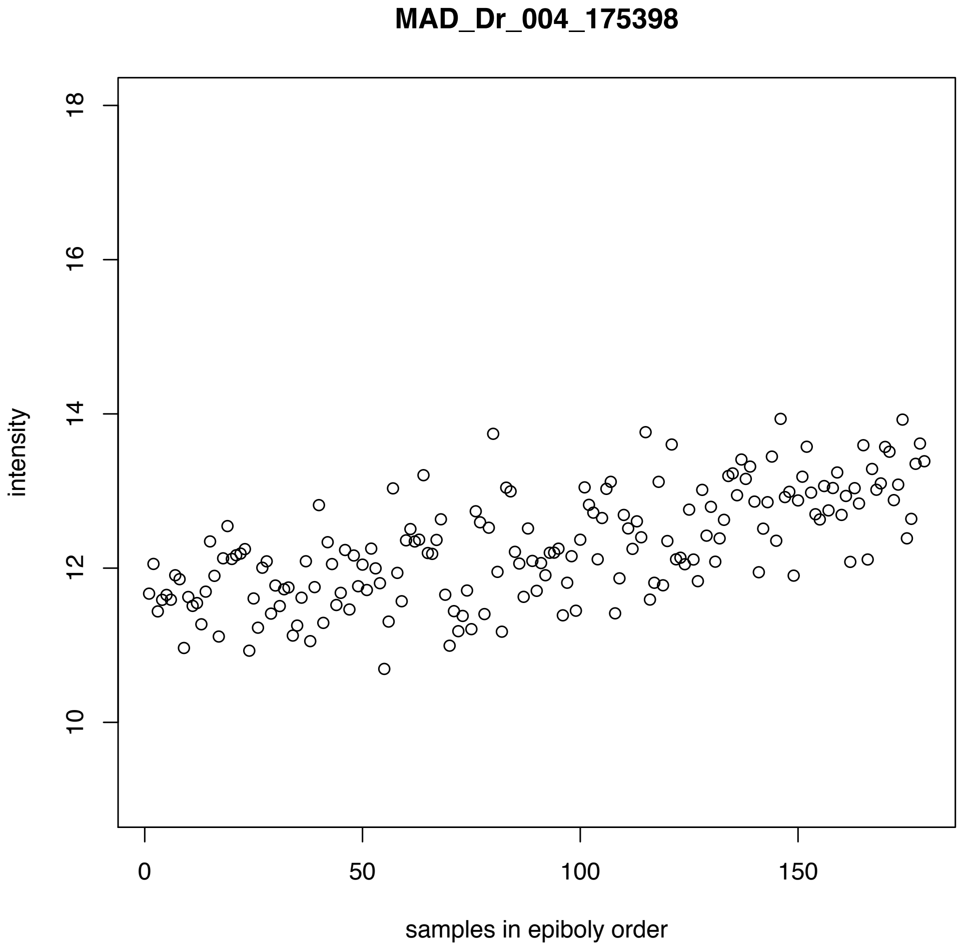

MAD_Dr_004_175398
intensity
10
12
14
16
18
0
50
100
150
samples in epiboly order

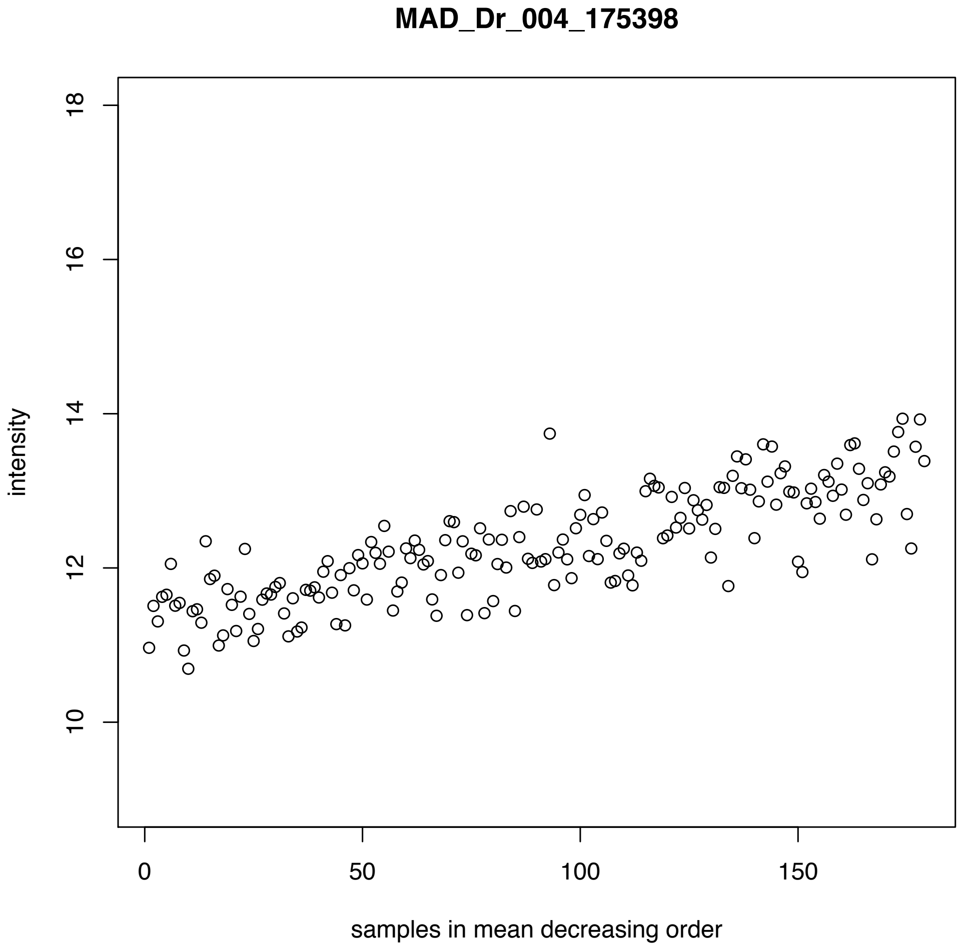

MAD_Dr_004_175398
intensity
10
12
14
16
18
0
50
100
150
samples in mean decreasing order

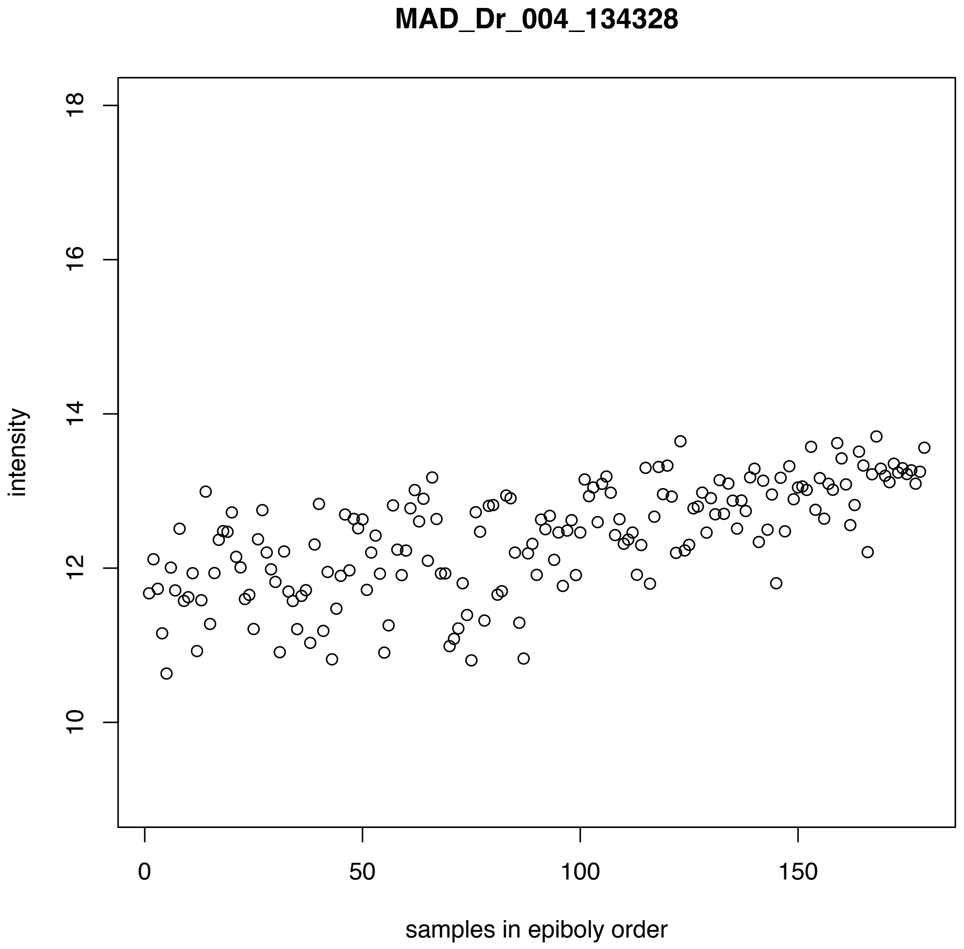
MAD_Dr_004_134328
intensity
18
16
14
12
10
0
50
100
150
samples in epiboly order

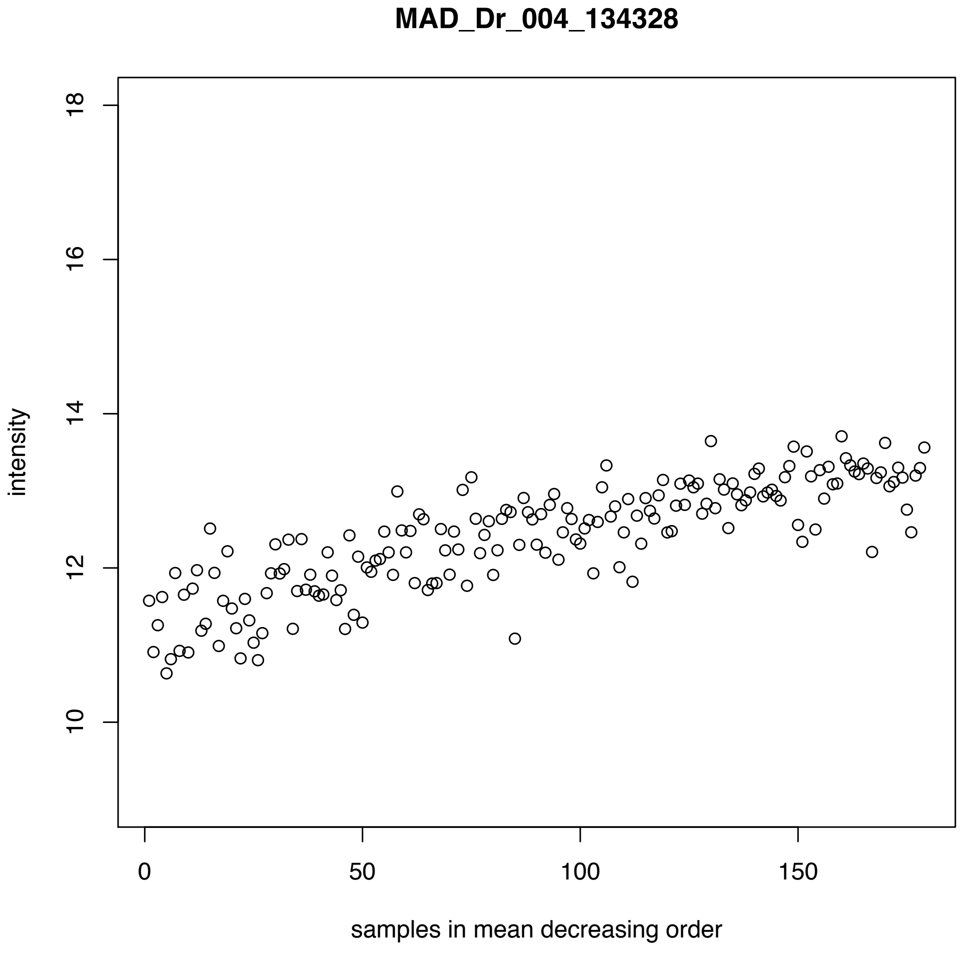
MAD_Dr_004_134328
intensity
18
16
14
12
10
0
50
100
150
samples in mean decreasing order

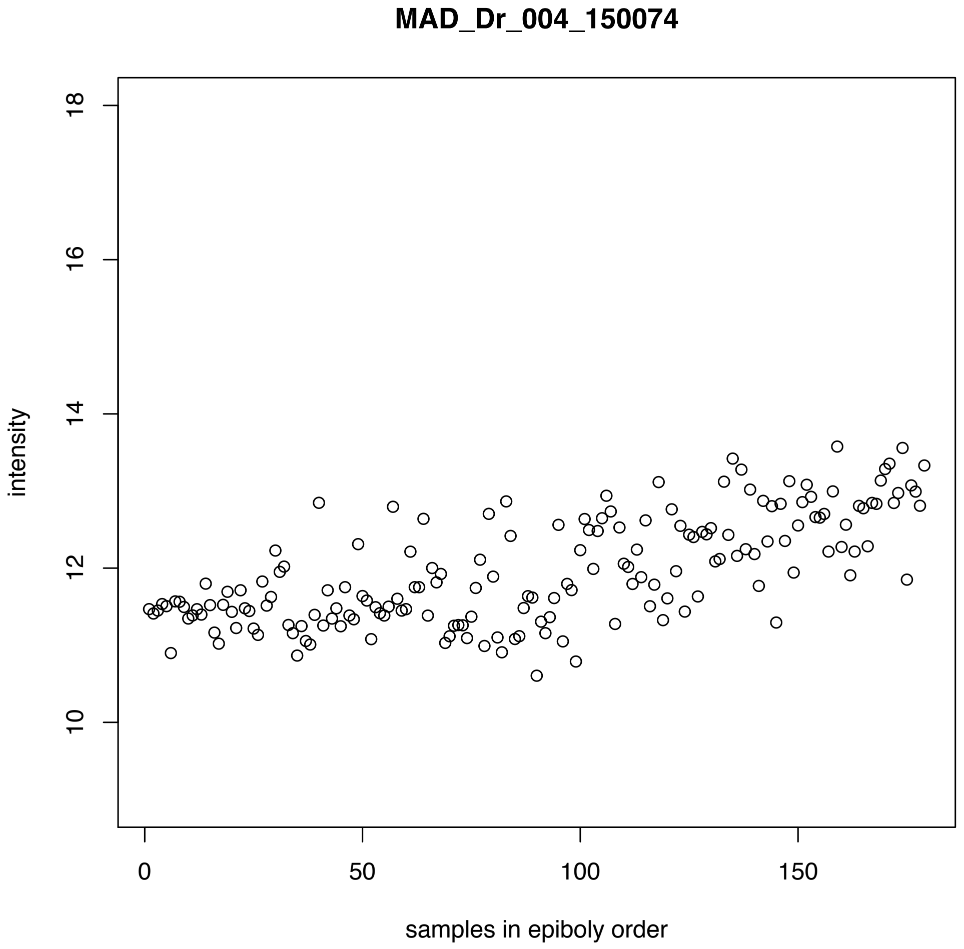
MAD_Dr_004_150074
intensity
samples in epiboly order
10
12
14
16
18
0
50
100
150

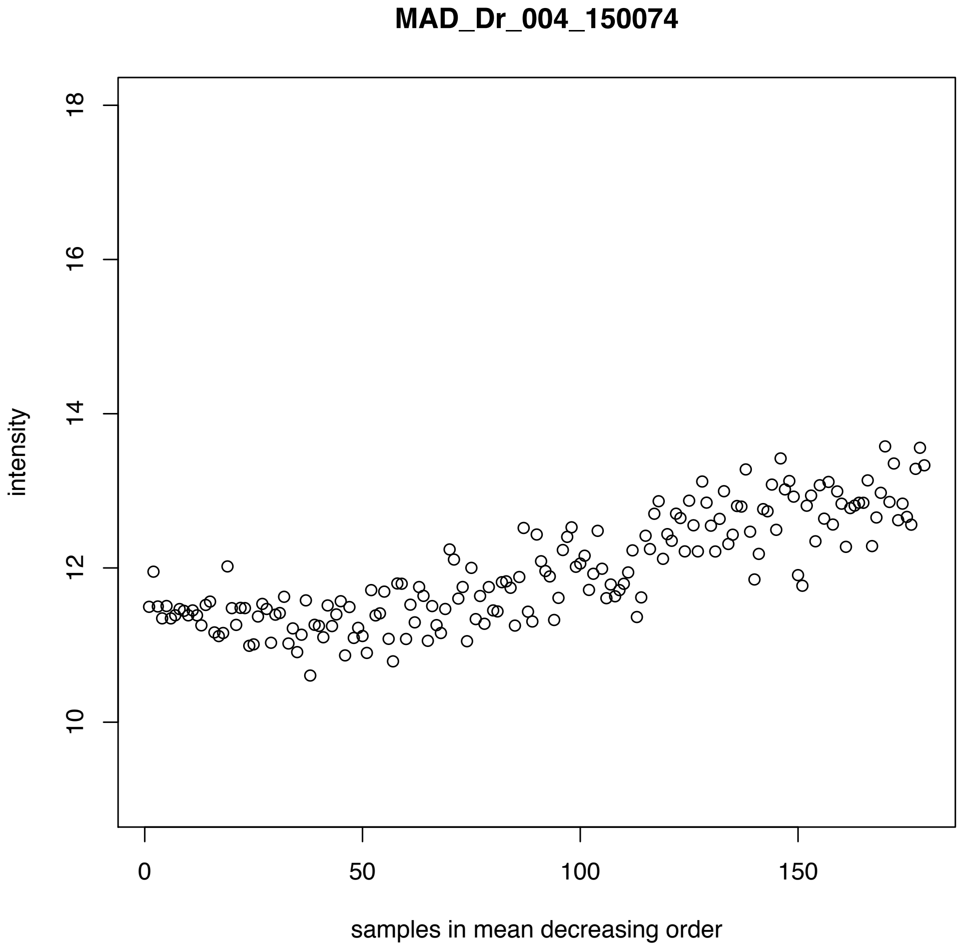
MAD_Dr_004_150074
intensity
samples in mean decreasing order
10
12
14
16
18
0
50
100
150

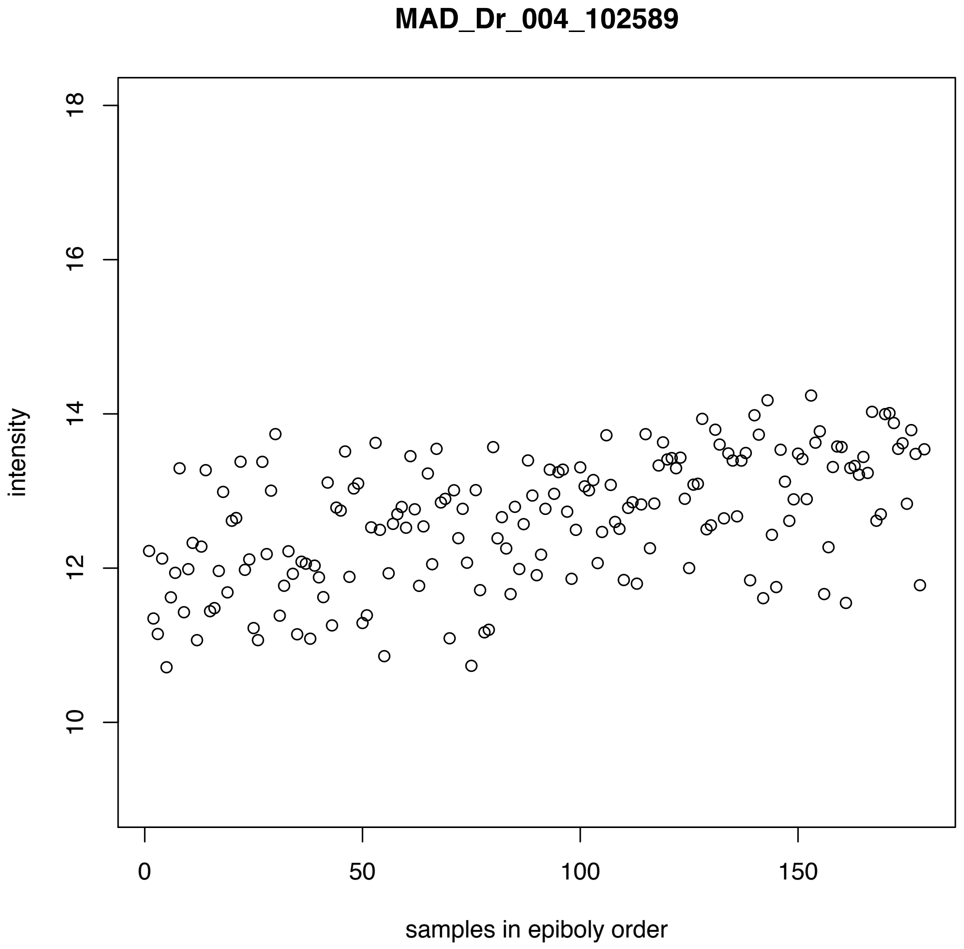

MAD_Dr_004_102589
intensity
10
12
14
16
18
0
50
100
150
samples in epiboly order

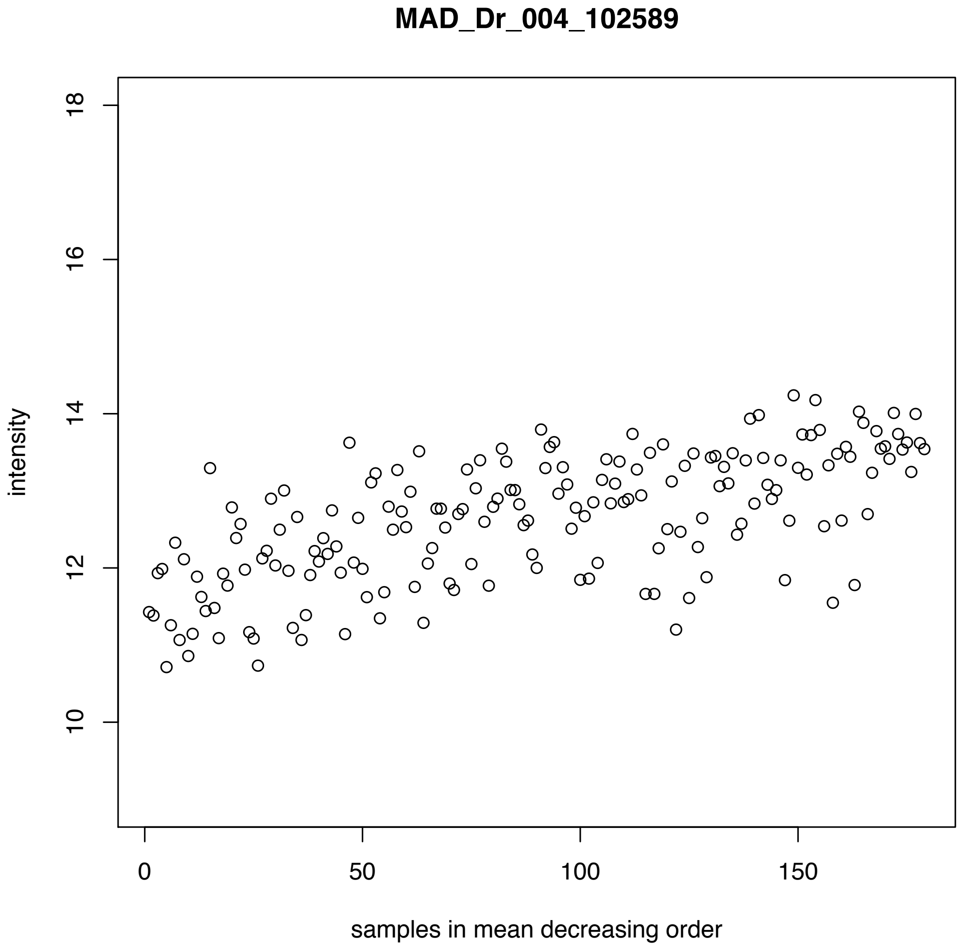

MAD_Dr_004_102589
intensity
10
12
14
16
18
0
50
100
150
samples in mean decreasing order

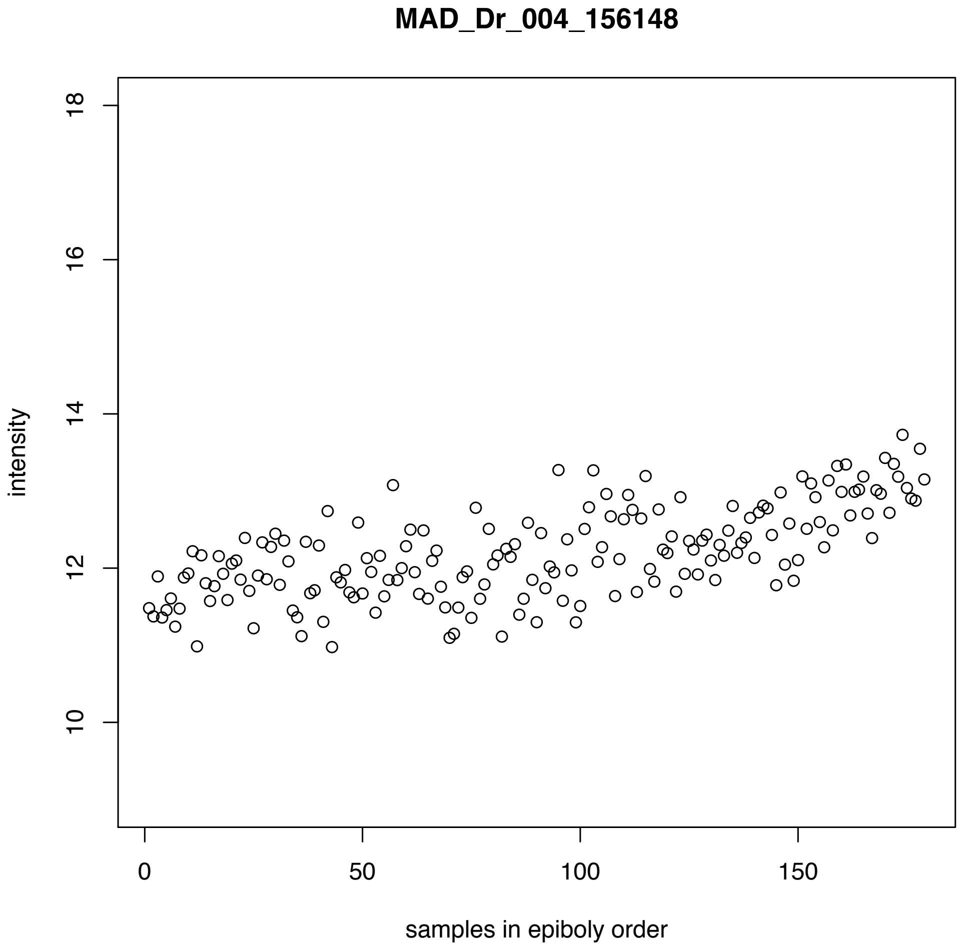
MAD_Dr_004_156148
intensity
10
12
14
16
18
0
50
100
150
samples in epiboly order

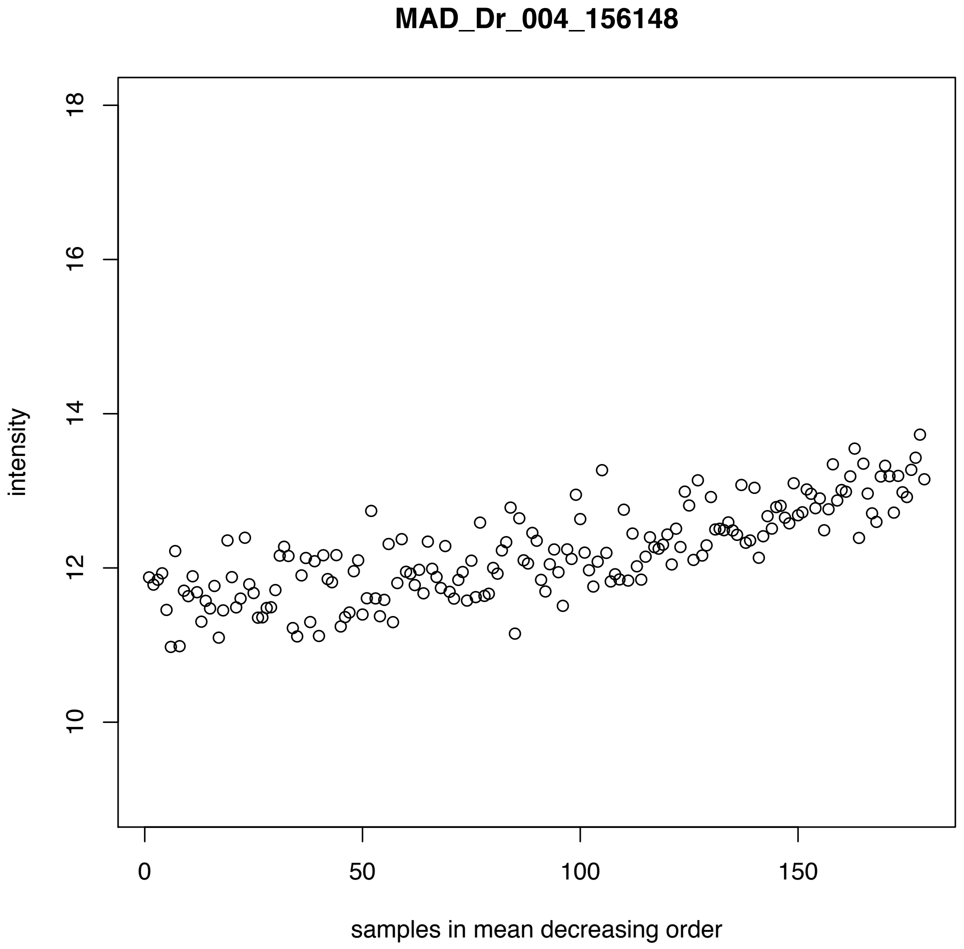
MAD_Dr_004_156148
intensity
10
12
14
16
18
0
50
100
150
samples in mean decreasing order

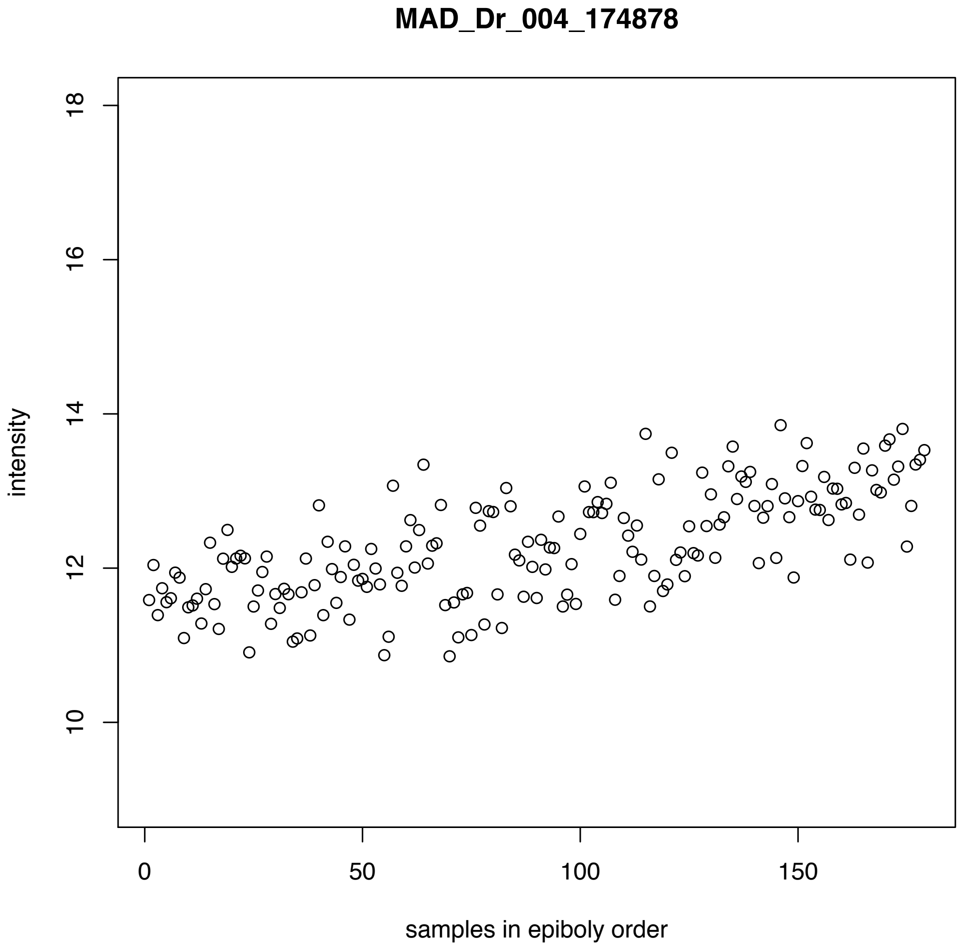

MAD_Dr_004_174878
intensity
samples in epiboly order

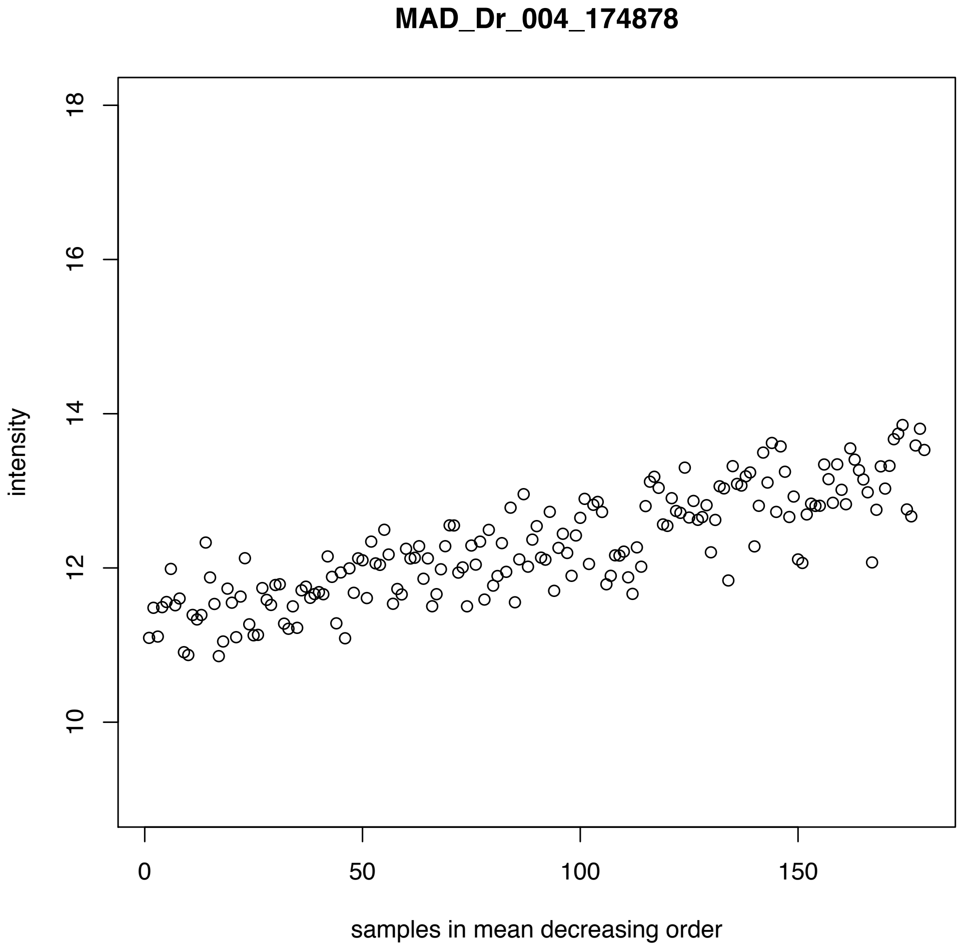

MAD_Dr_004_174878
intensity
samples in mean decreasing order

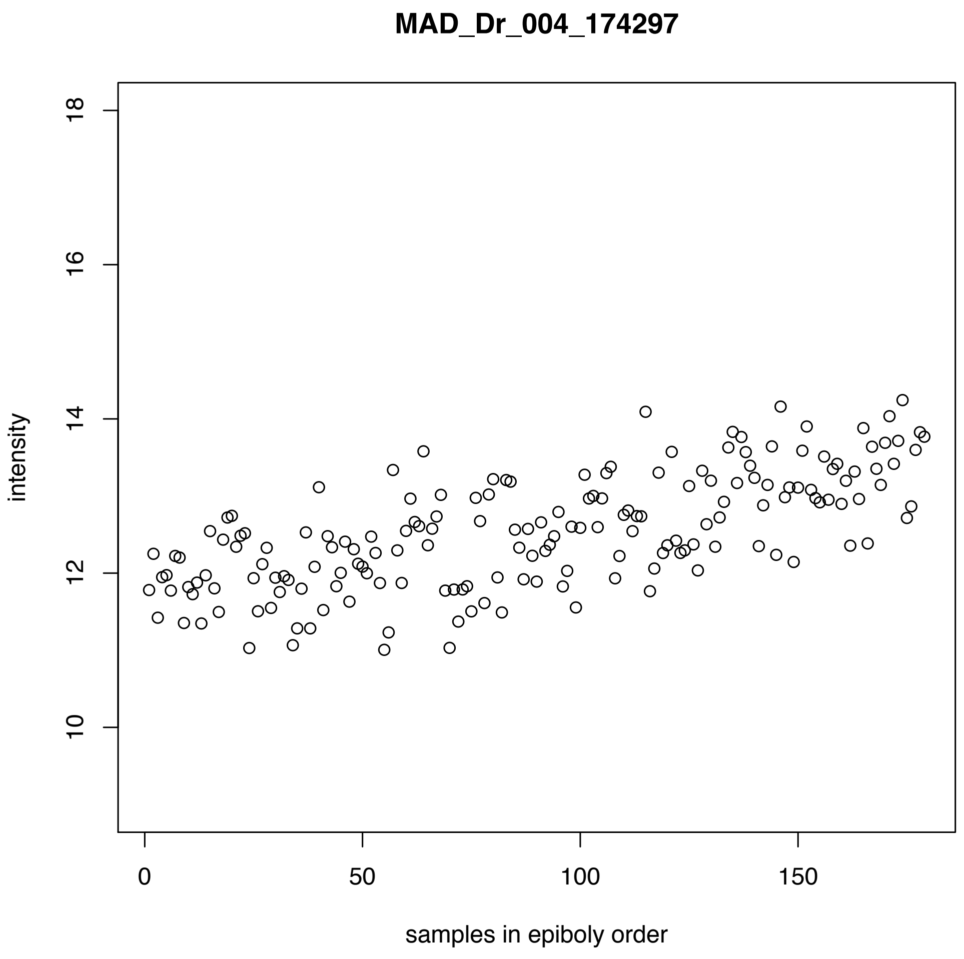
MAD_Dr_004_174297
intensity
10
12
14
16
18
0
50
100
150
samples in epiboly order

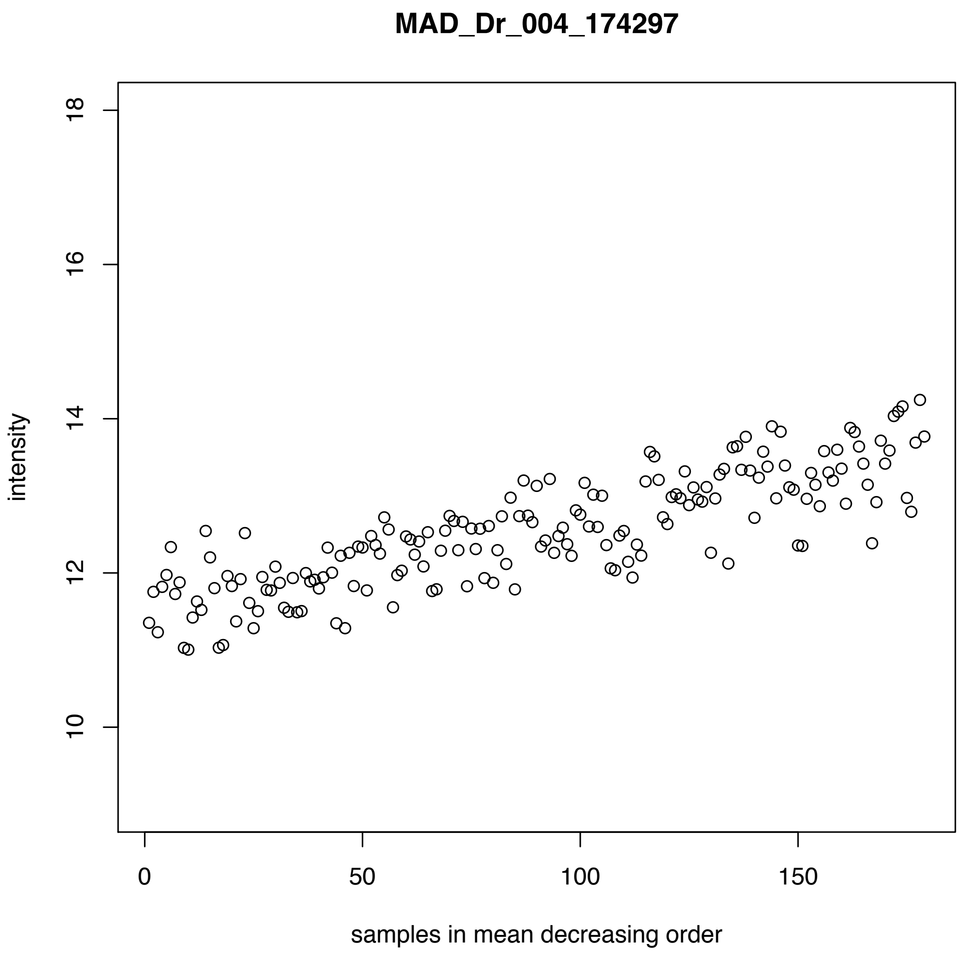
MAD_Dr_004_174297
intensity
10
12
14
16
18
0
50
100
150
samples in mean decreasing order

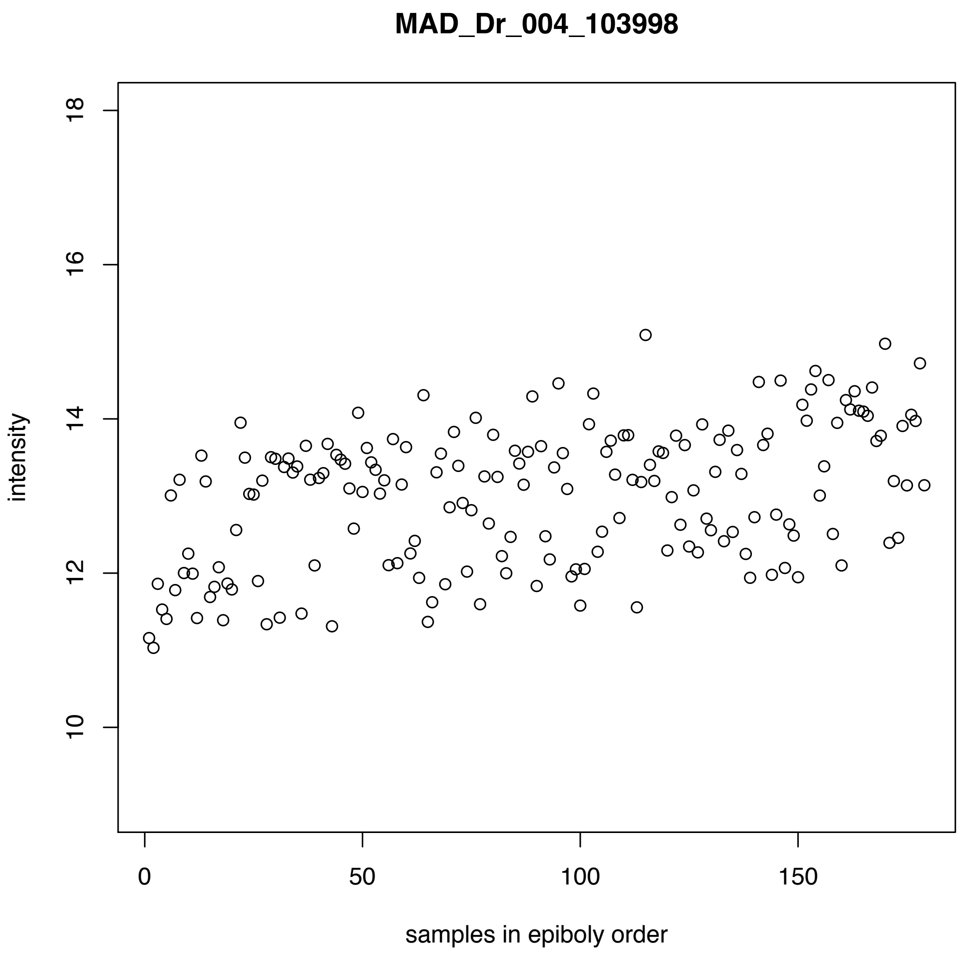
MAD_Dr_004_103998
intensity
10
12
14
16
18
0
50
100
150
samples in epiboly order

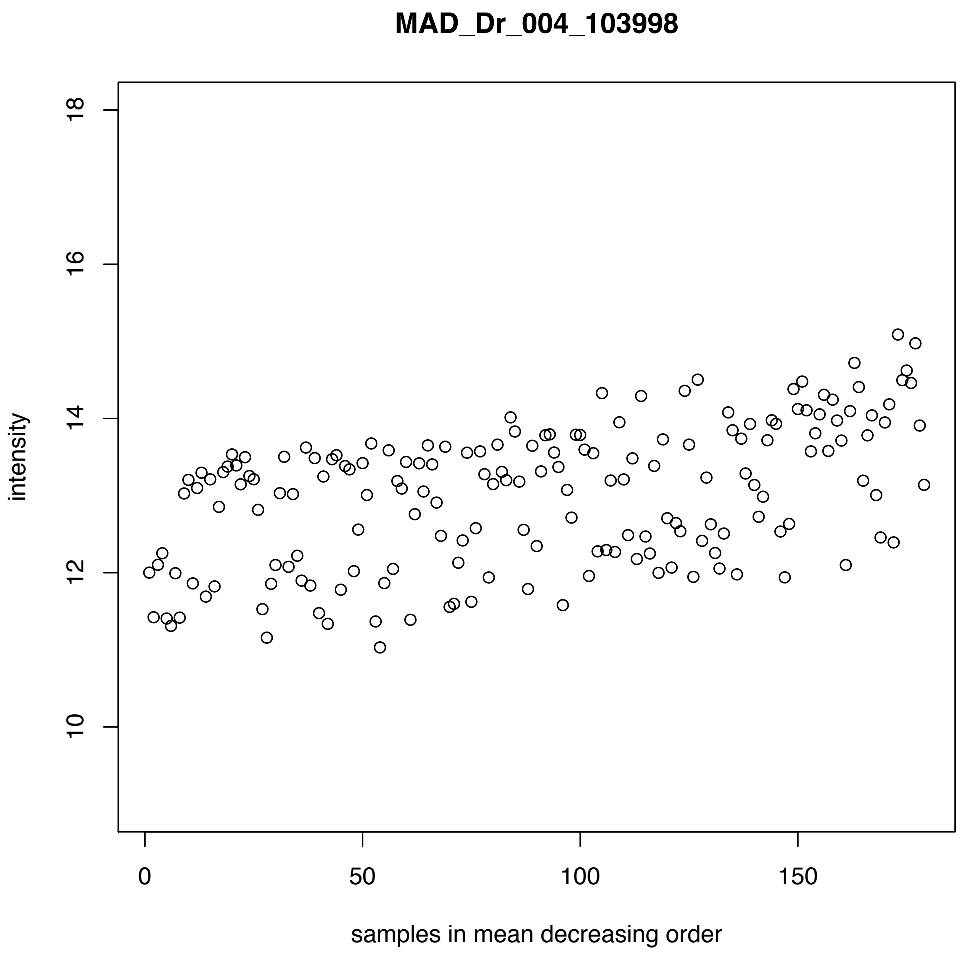
MAD_Dr_004_103998
intensity
10
12
14
16
18
0
50
100
150
samples in mean decreasing order

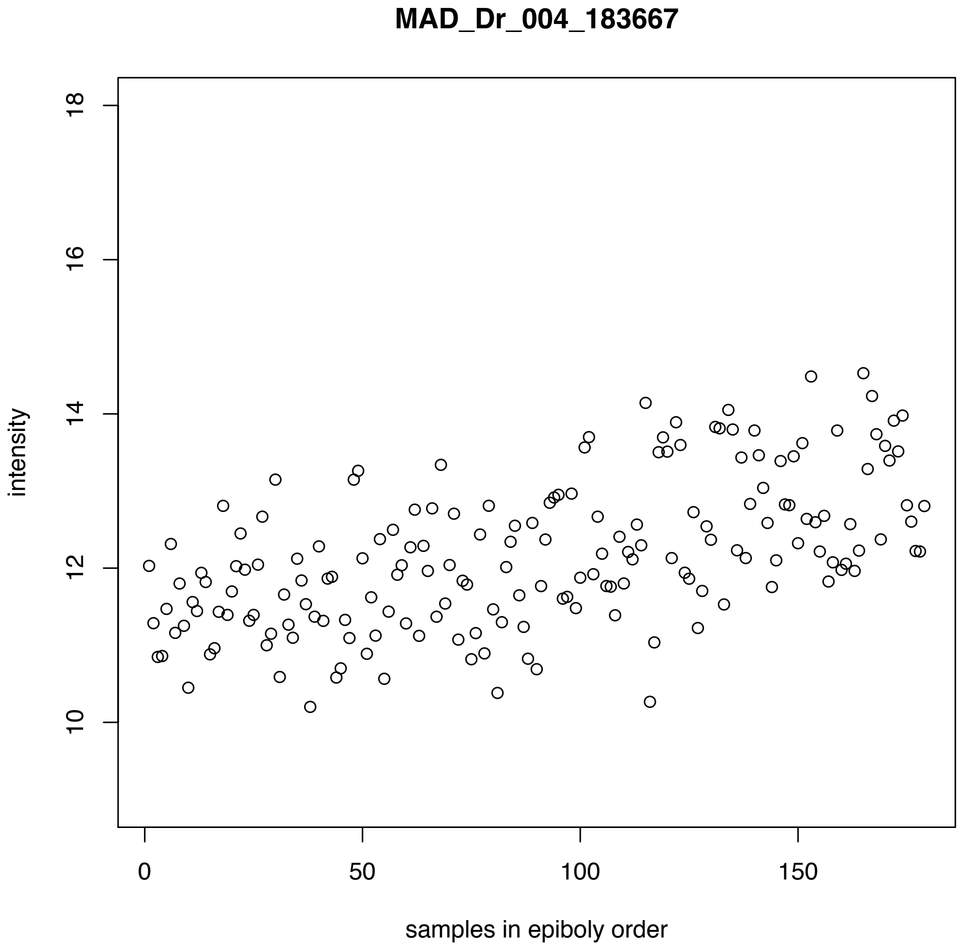
MAD_Dr_004_183667
intensity
10
12
14
16
18
0
50
100
150
samples in epiboly order

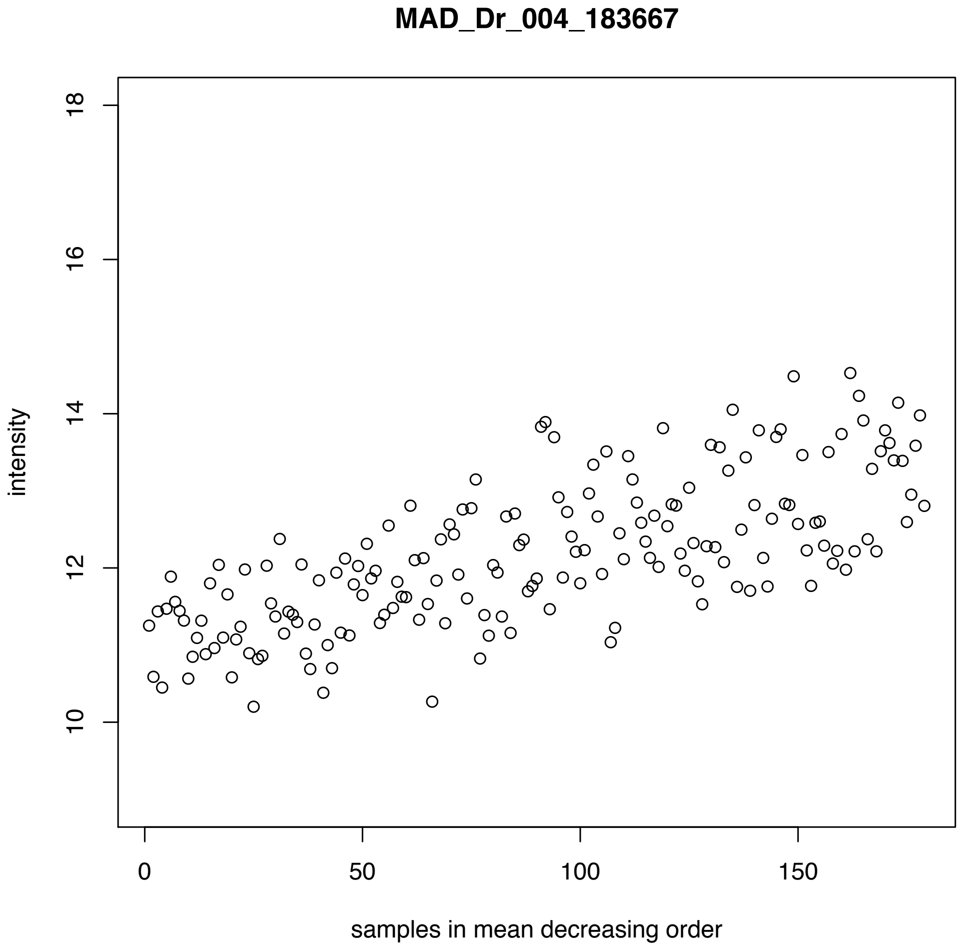
MAD_Dr_004_183667
intensity
10
12
14
16
18
0
50
100
150
samples in mean decreasing order

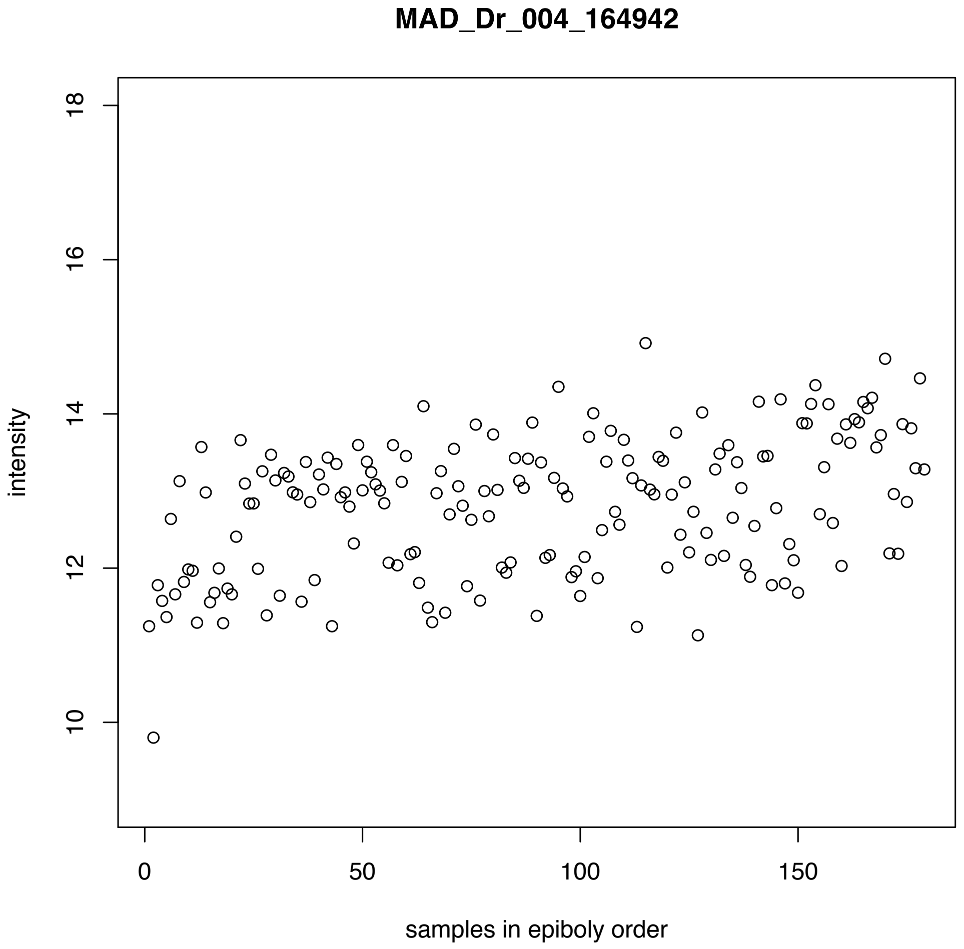
MAD_Dr_004_164942
intensity
samples in epiboly order
10
12
14
16
18
0
50
100
150

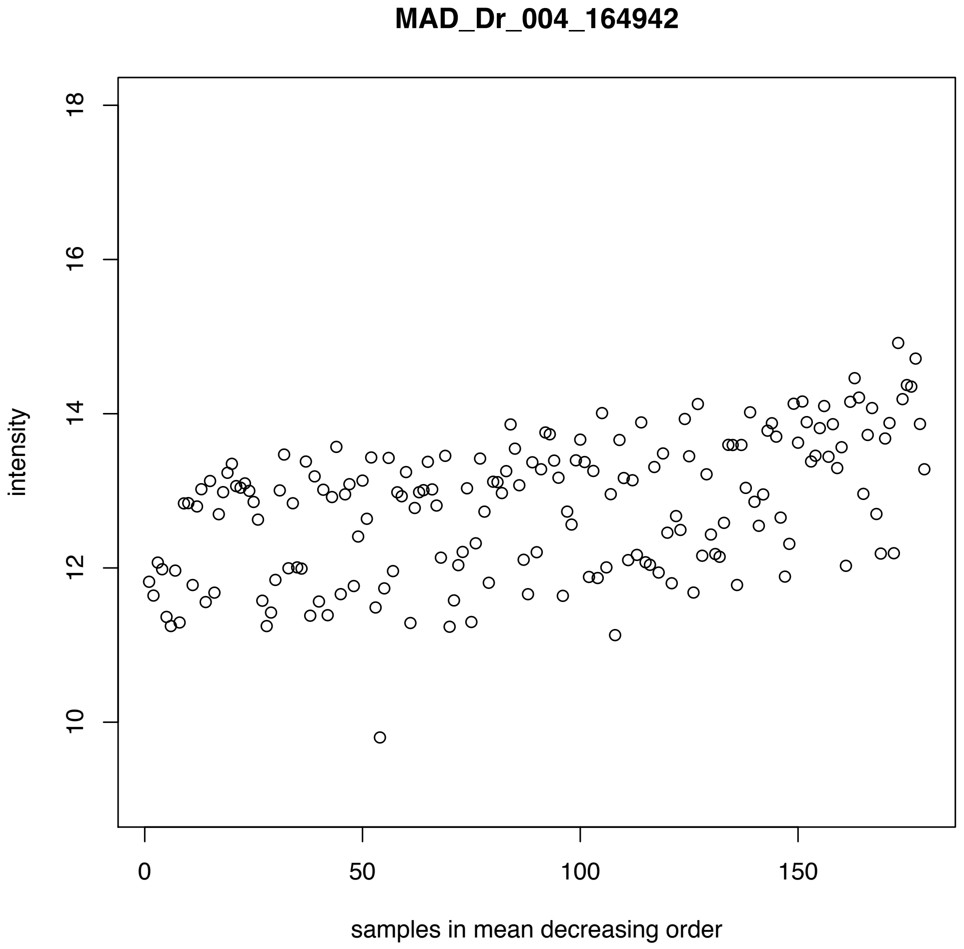
MAD_Dr_004_164942
intensity
samples in mean decreasing order
10
12
14
16
18
0
50
100
150

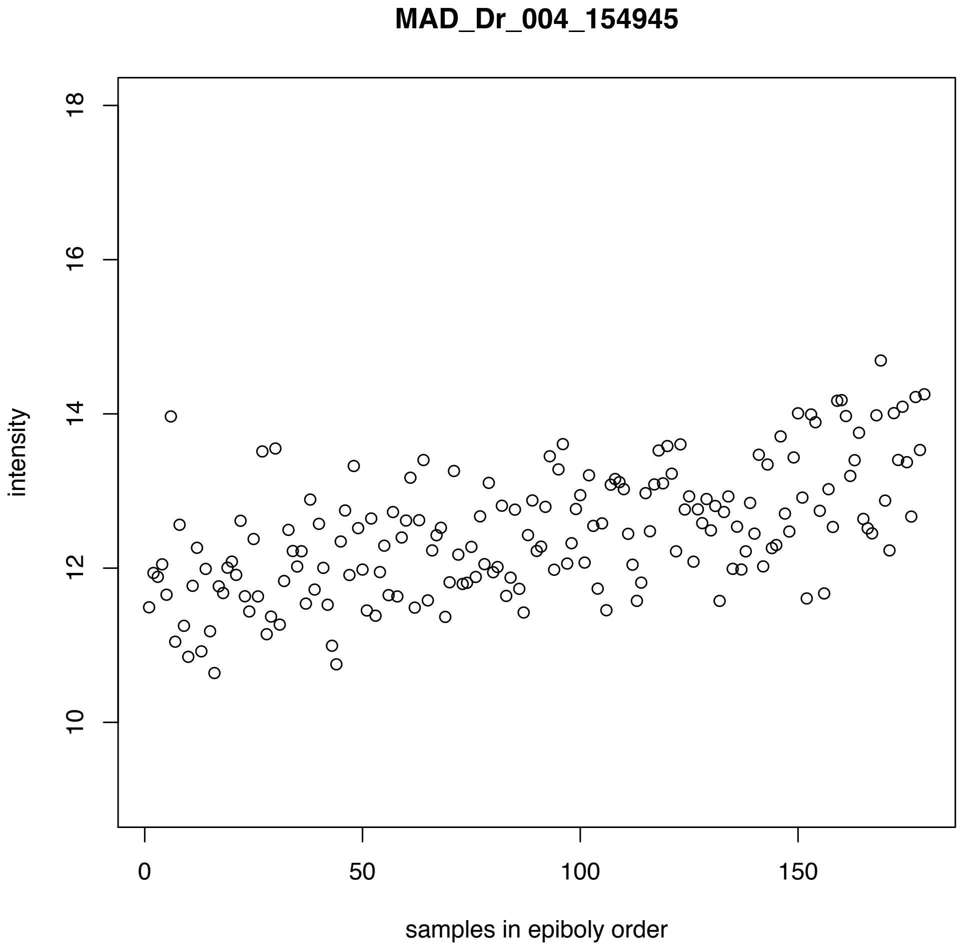
MAD_Dr_004_154945
intensity
samples in epiboly order
10
12
14
16
18
0
50
100
150

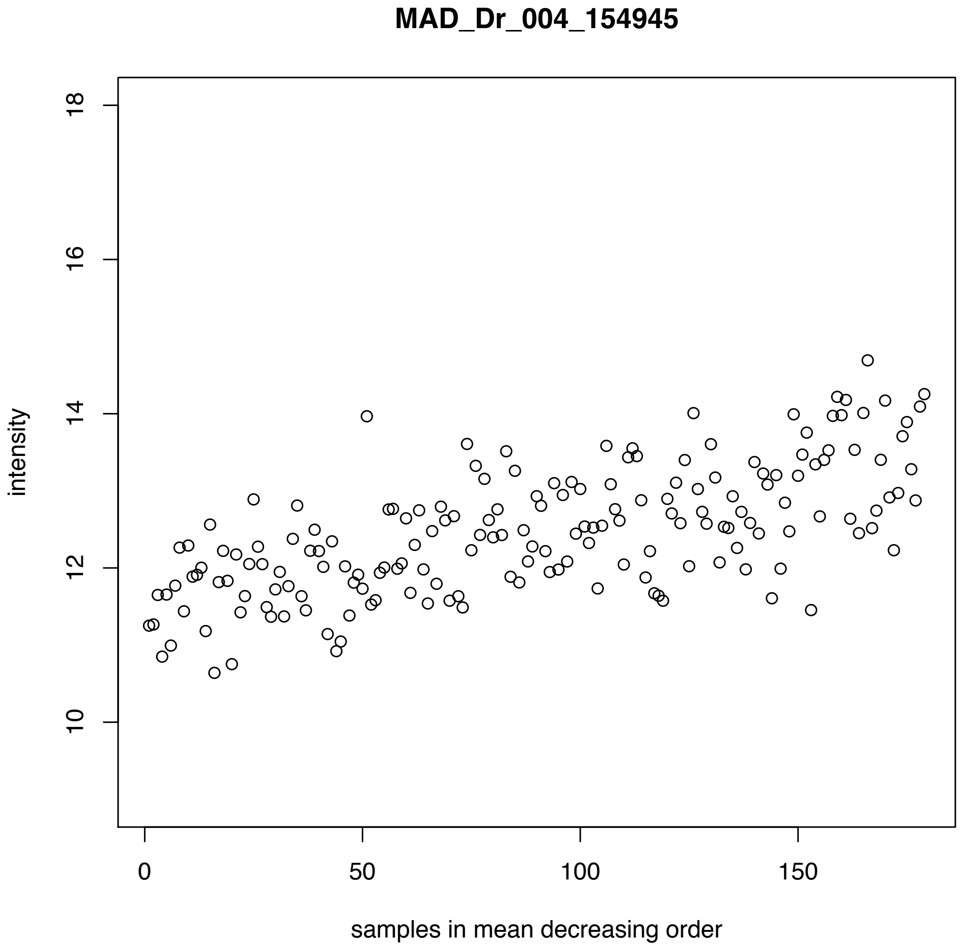
MAD_Dr_004_154945
intensity
samples in mean decreasing order
10
12
14
16
18
0
50
100
150

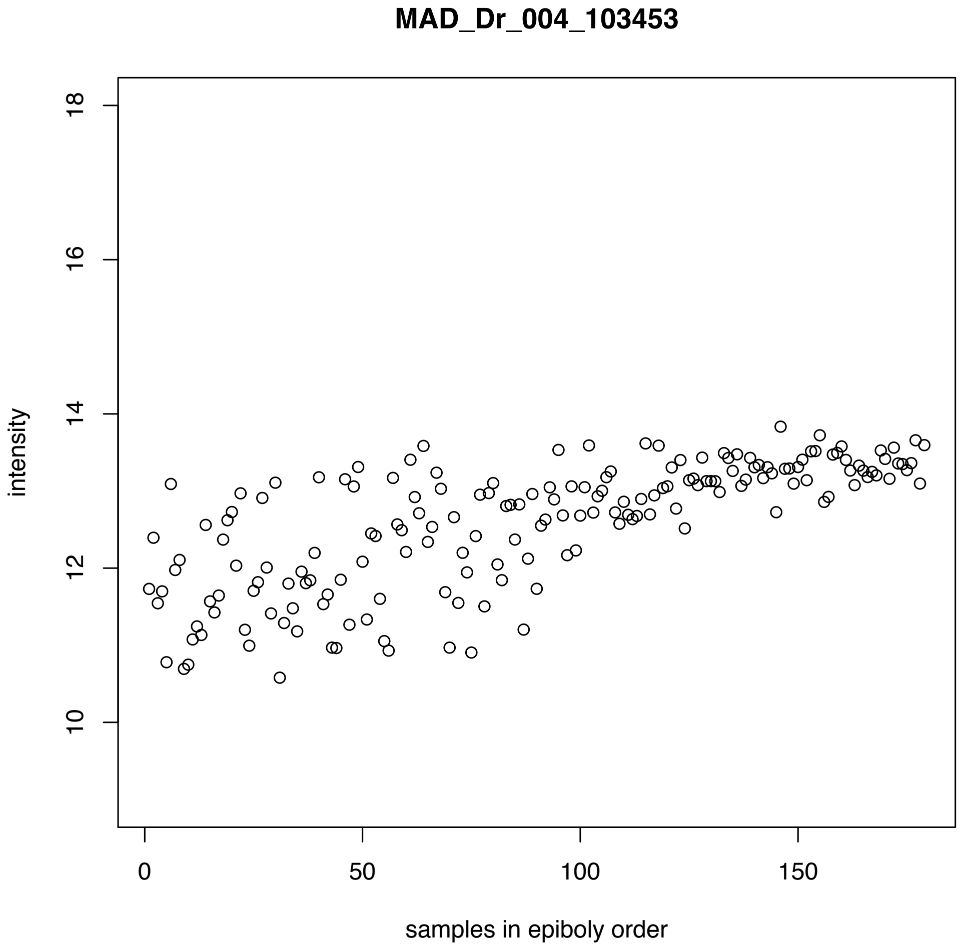
MAD_Dr_004_103453
intensity
10
12
14
16
18
0
50
100
150
samples in epiboly order

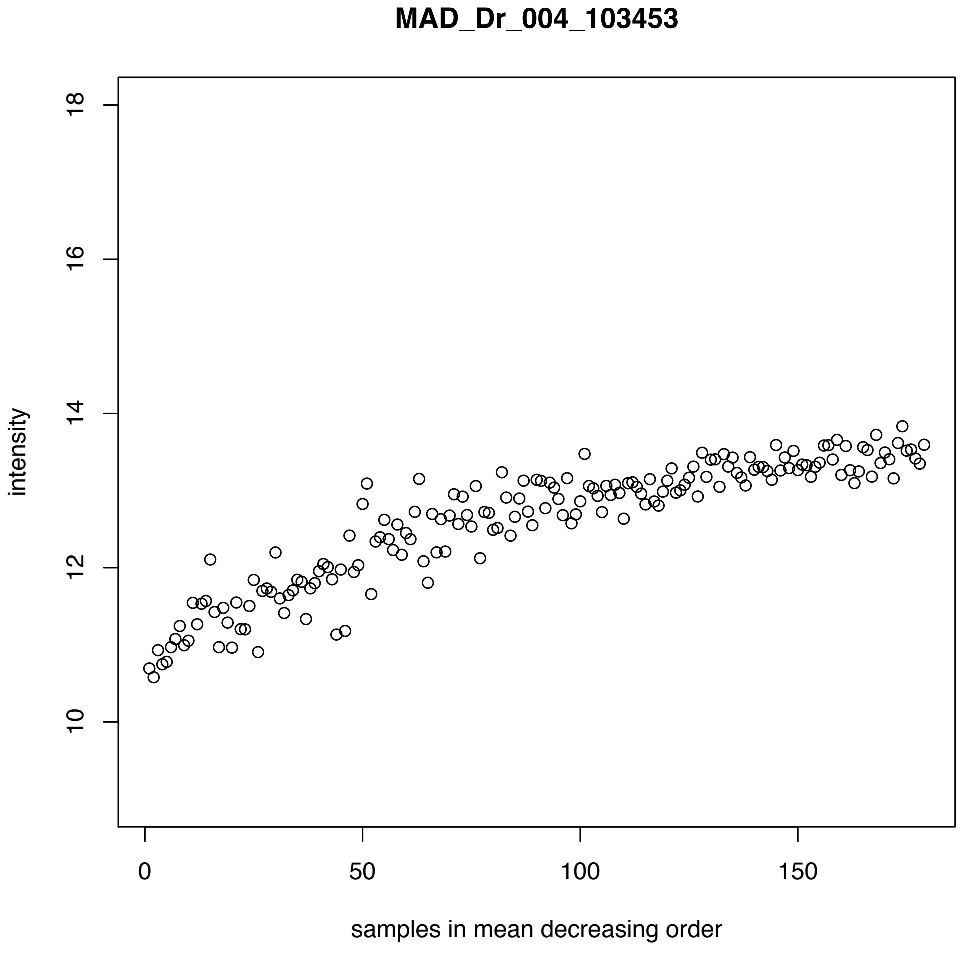
MAD_Dr_004_103453
intensity
10
12
14
16
18
0
50
100
150
samples in mean decreasing order

MAD_Dr_004_107549

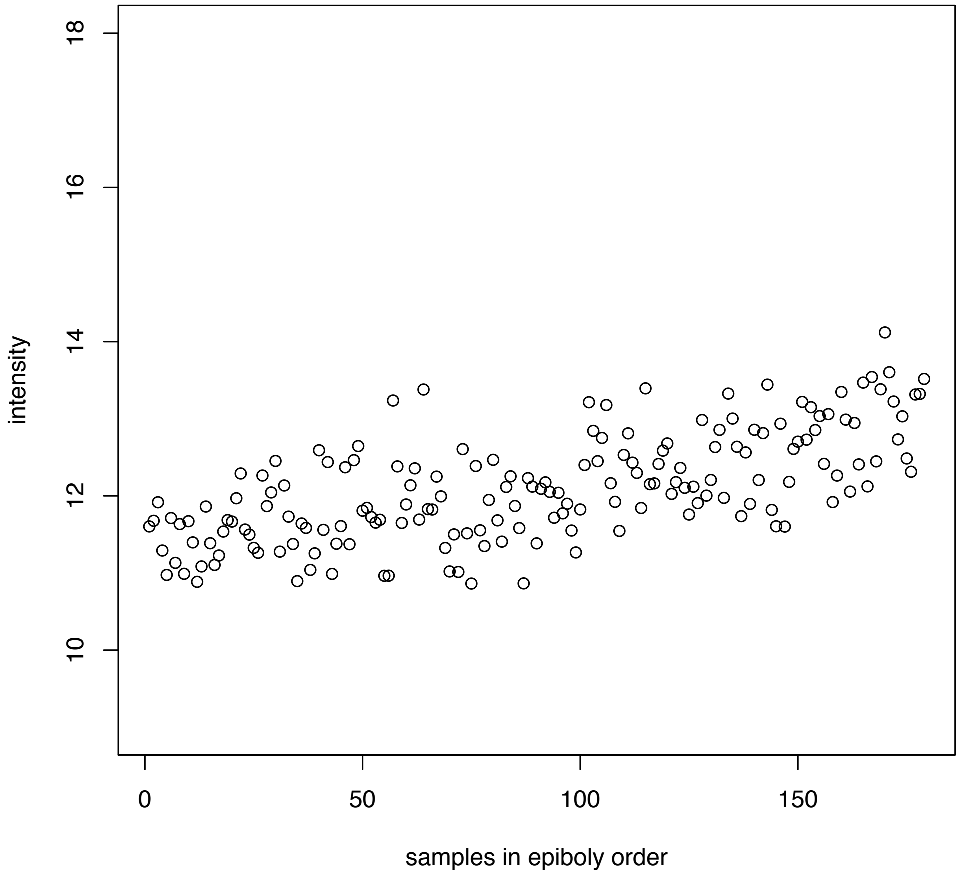

MAD_Dr_004_107549

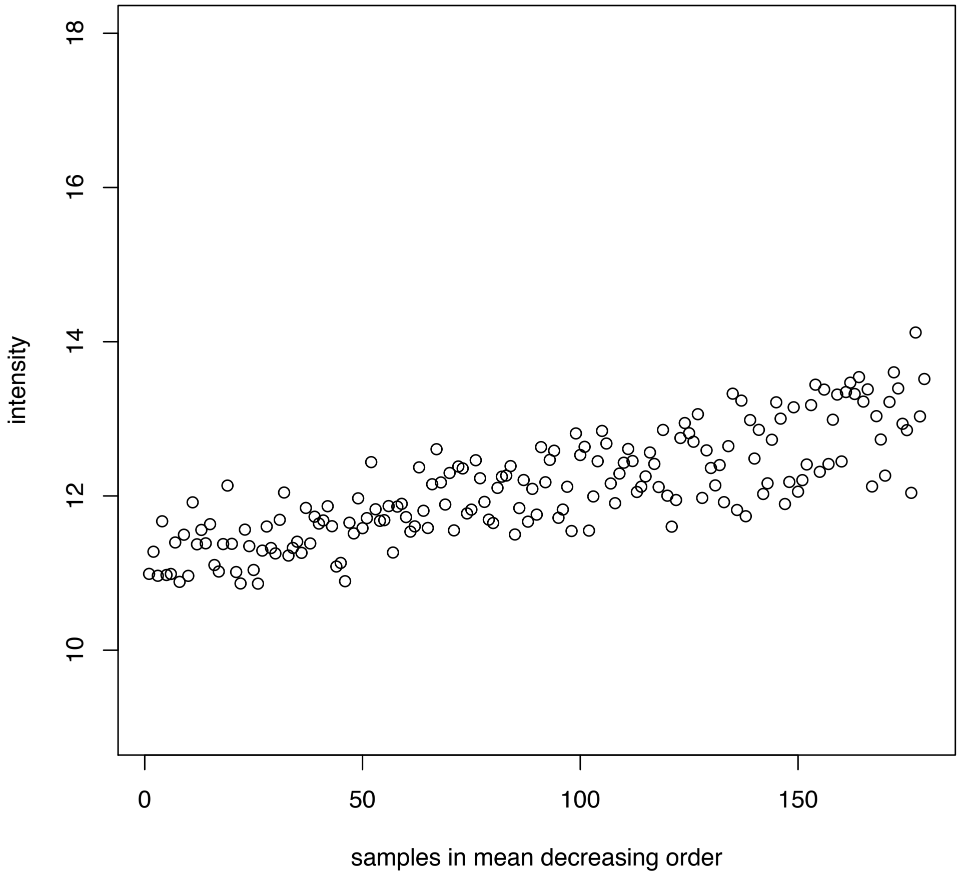

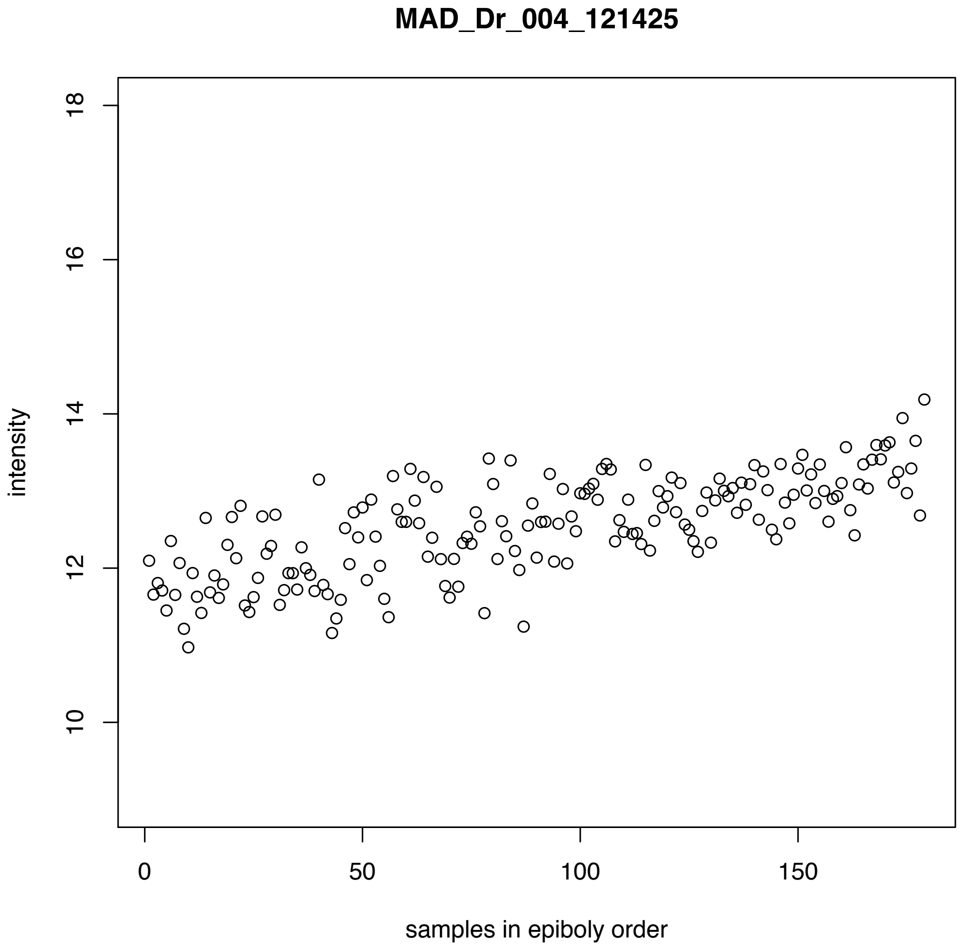
MAD_Dr_004_121425
intensity
10
12
14
16
18
0
50
100
150
samples in epiboly order

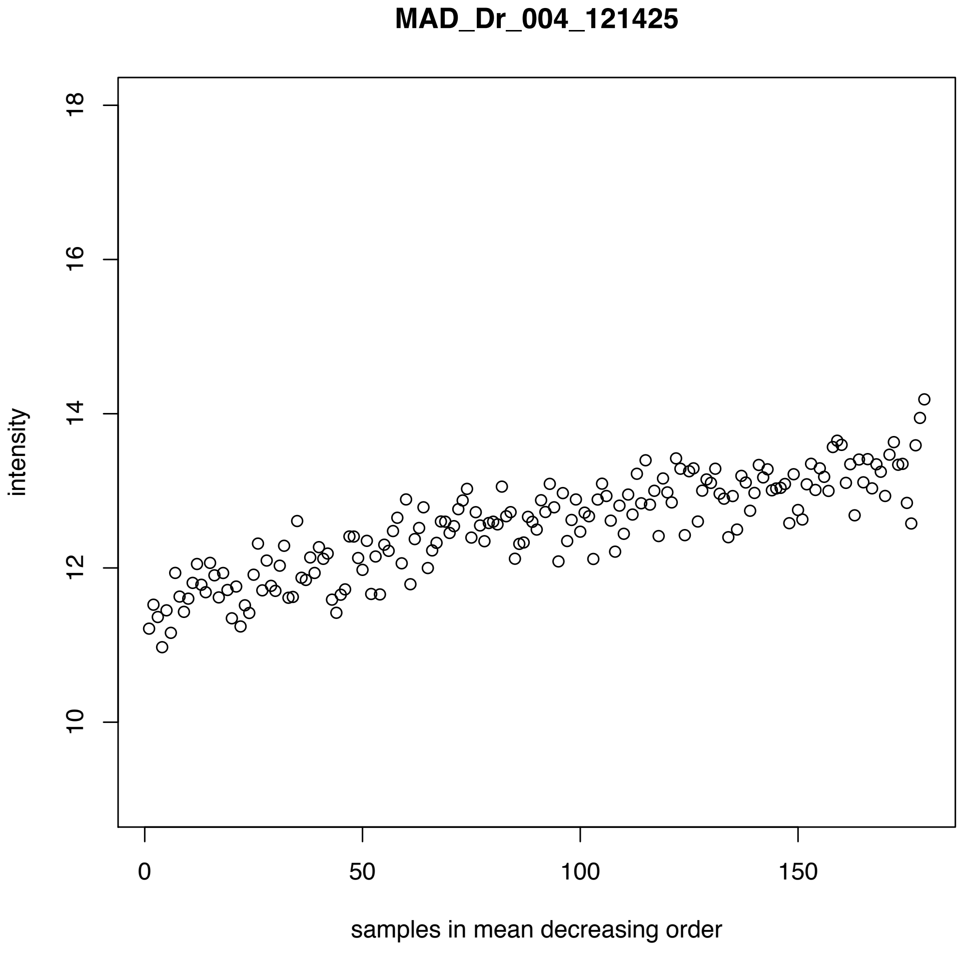
MAD_Dr_004_121425
intensity
10
12
14
16
18
0
50
100
150
samples in mean decreasing order

MAD_Dr_004_103081

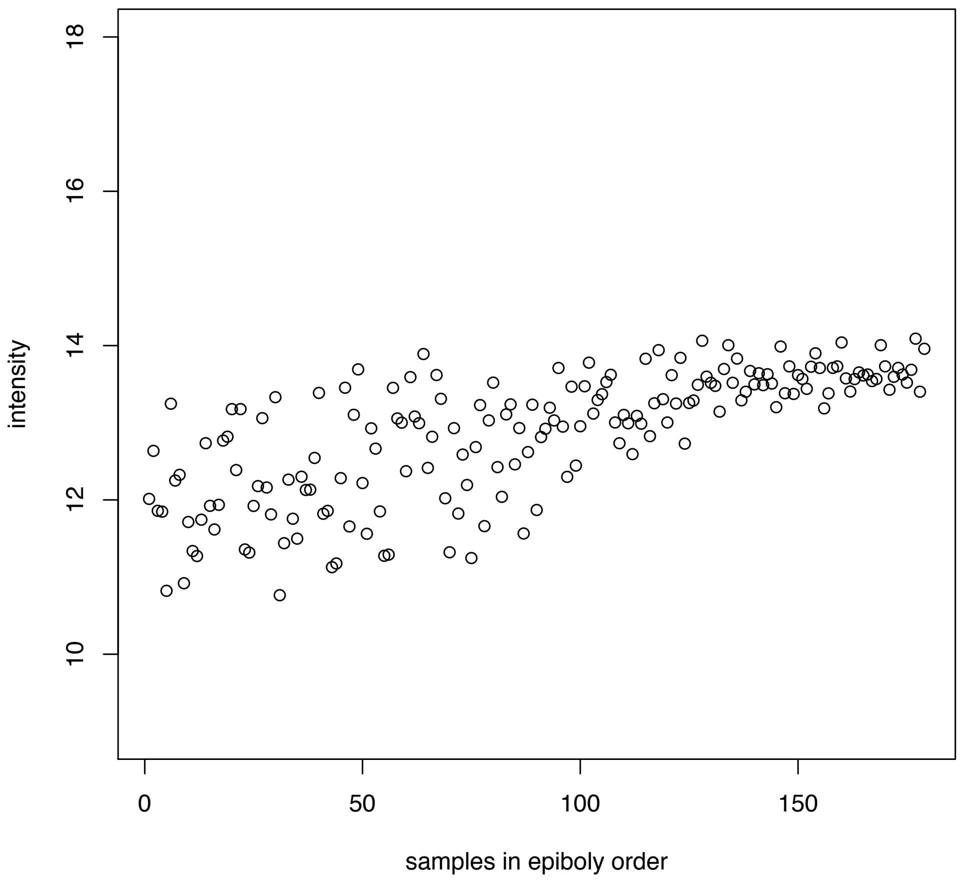

MAD_Dr_004_103081

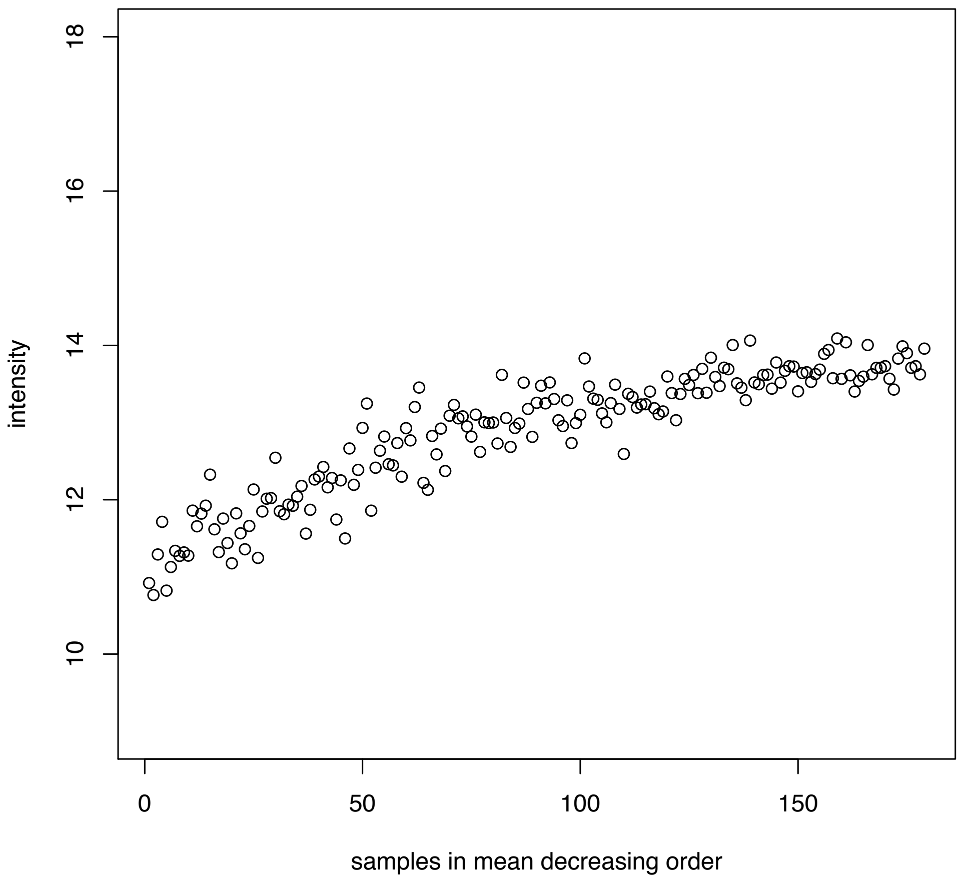

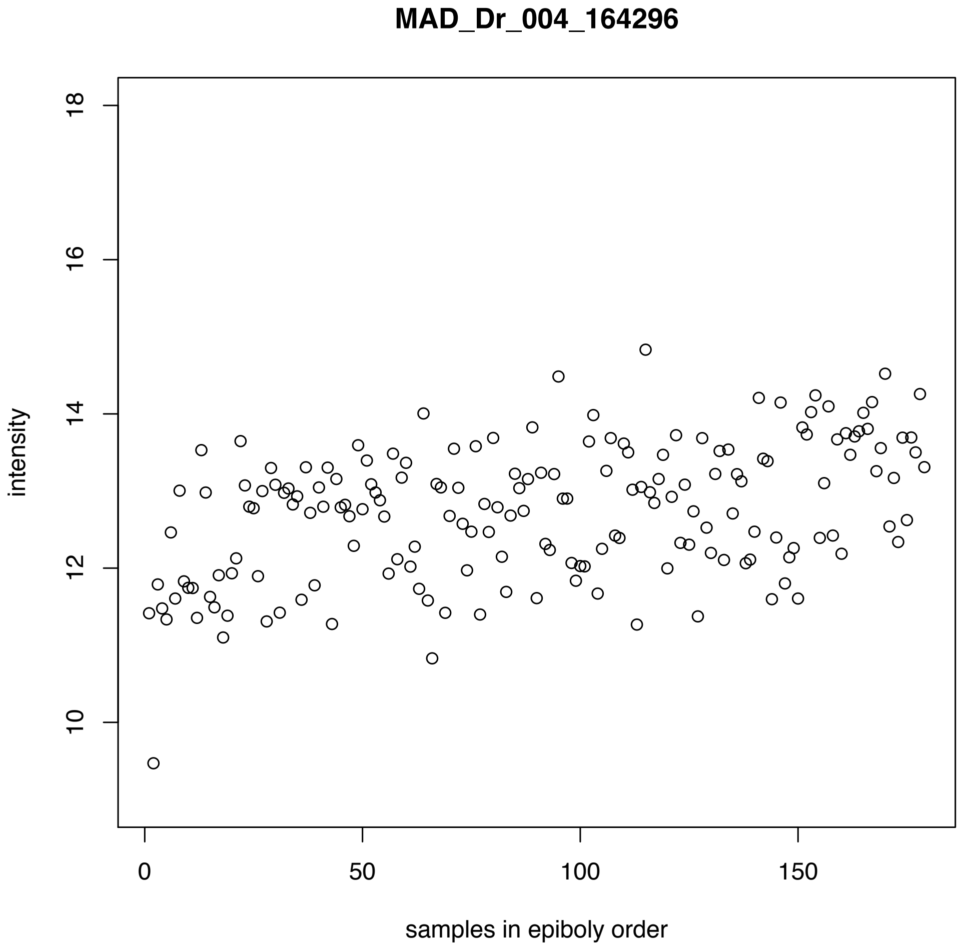

MAD_Dr_004_164296
intensity
samples in epiboly order

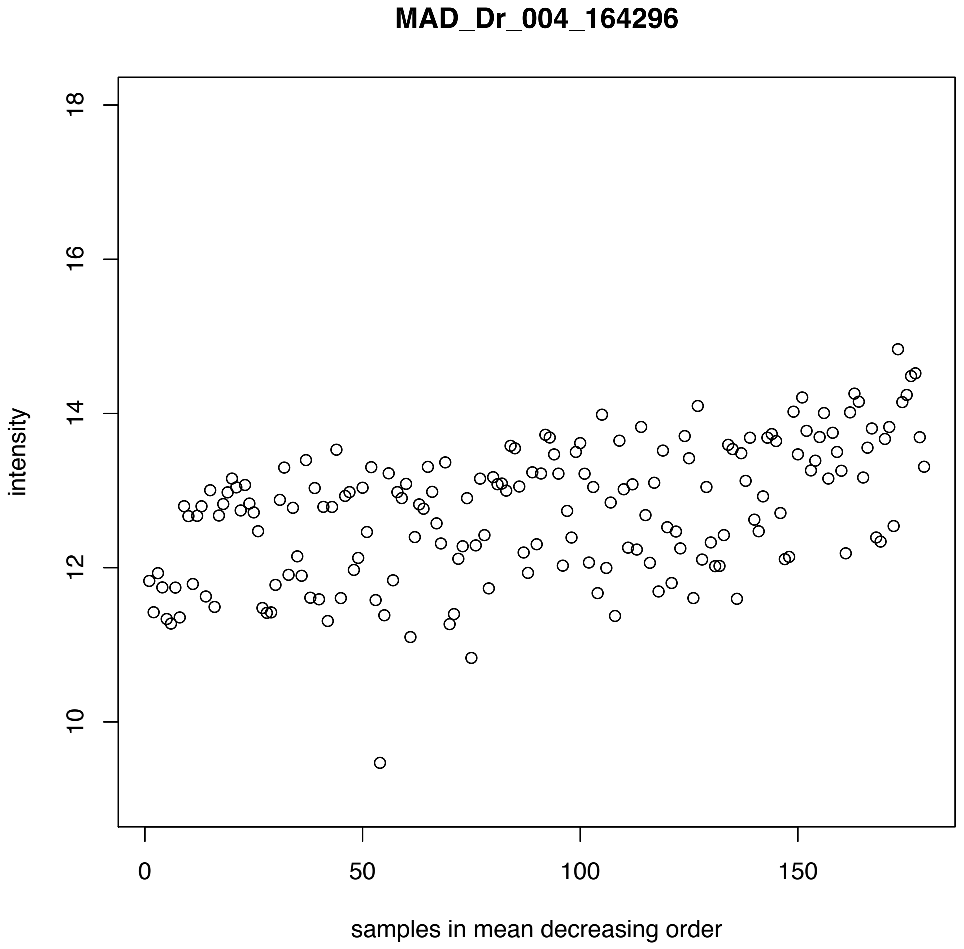

MAD_Dr_004_164296
intensity
samples in mean decreasing order

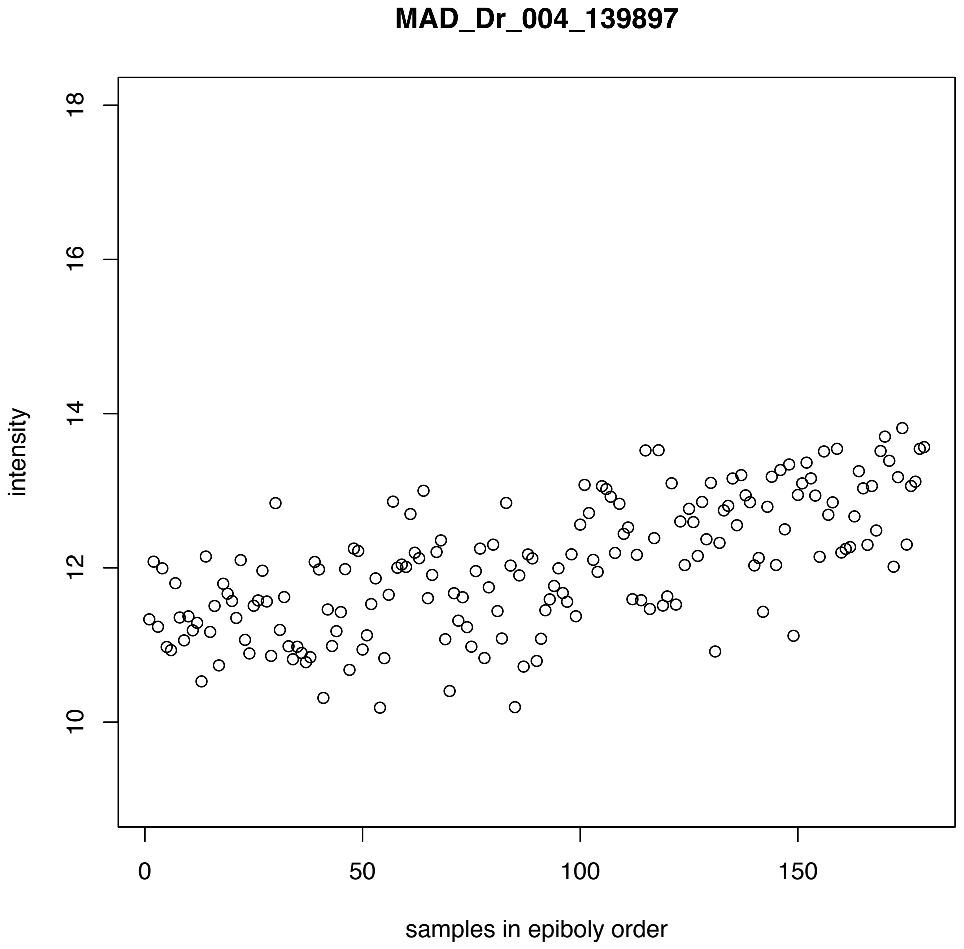

MAD_Dr_004_139897
intensity
10
12
14
16
18
0
50
100
150
samples in epiboly order

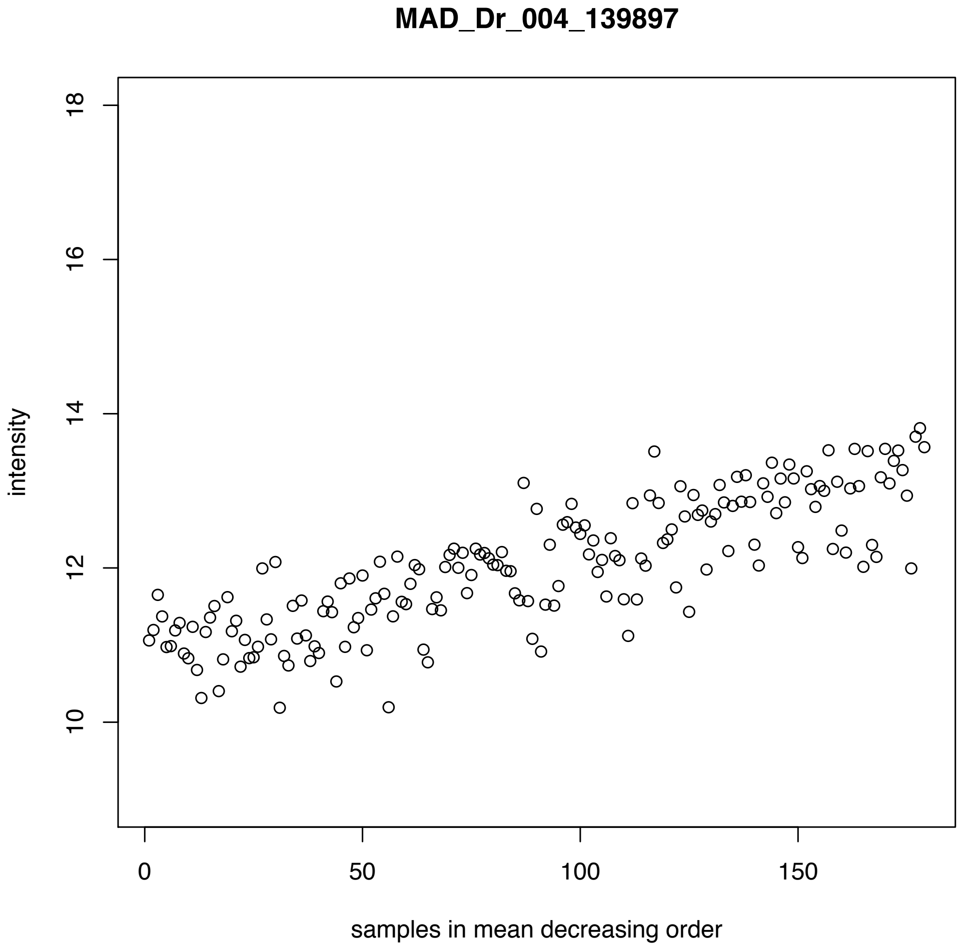

MAD_Dr_004_139897
intensity
10
12
14
16
18
0
50
100
150
samples in mean decreasing order

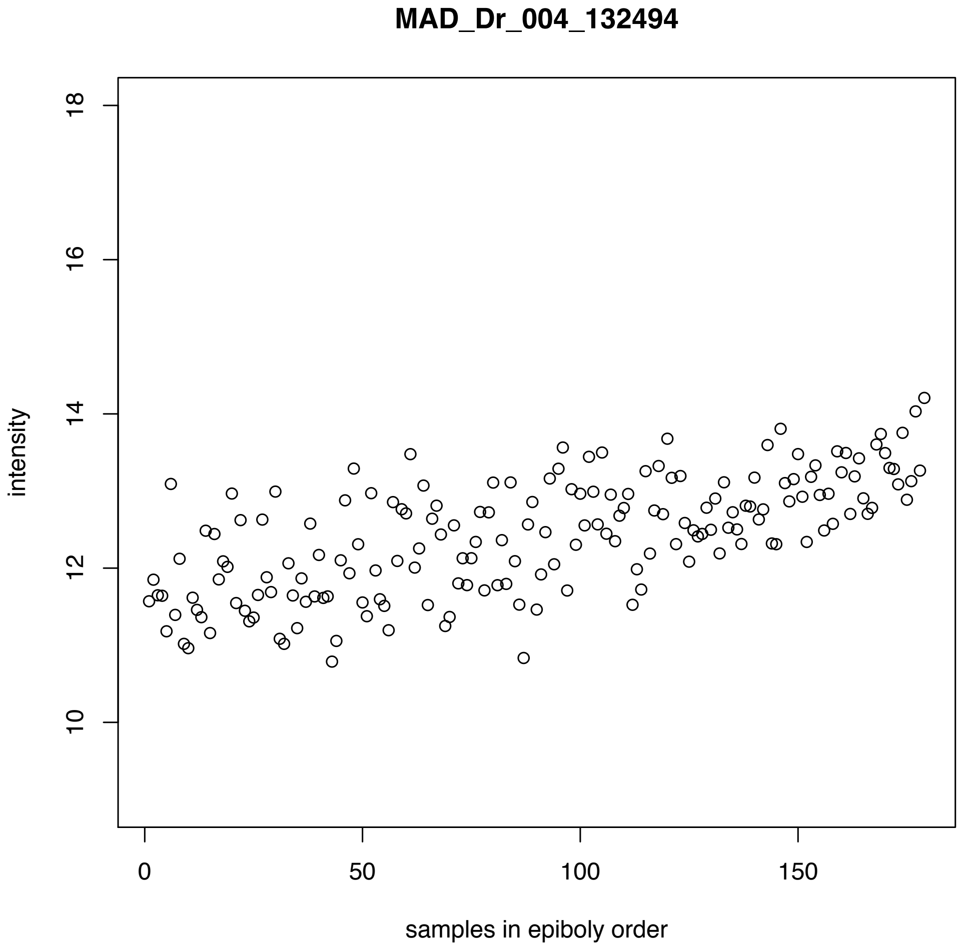
MAD_Dr_004_132494
intensity
samples in epiboly order
10
12
14
16
18
0
50
100
150

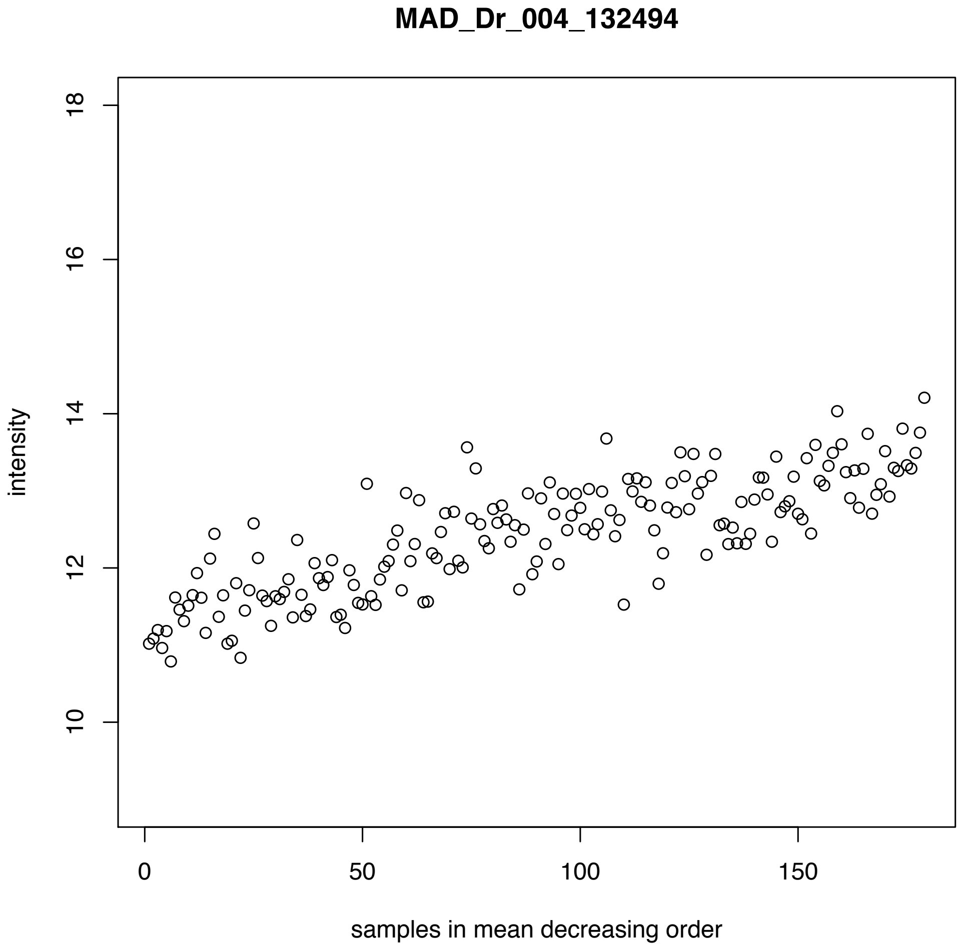
MAD_Dr_004_132494
intensity
samples in mean decreasing order
10
12
14
16
18
0
50
100
150

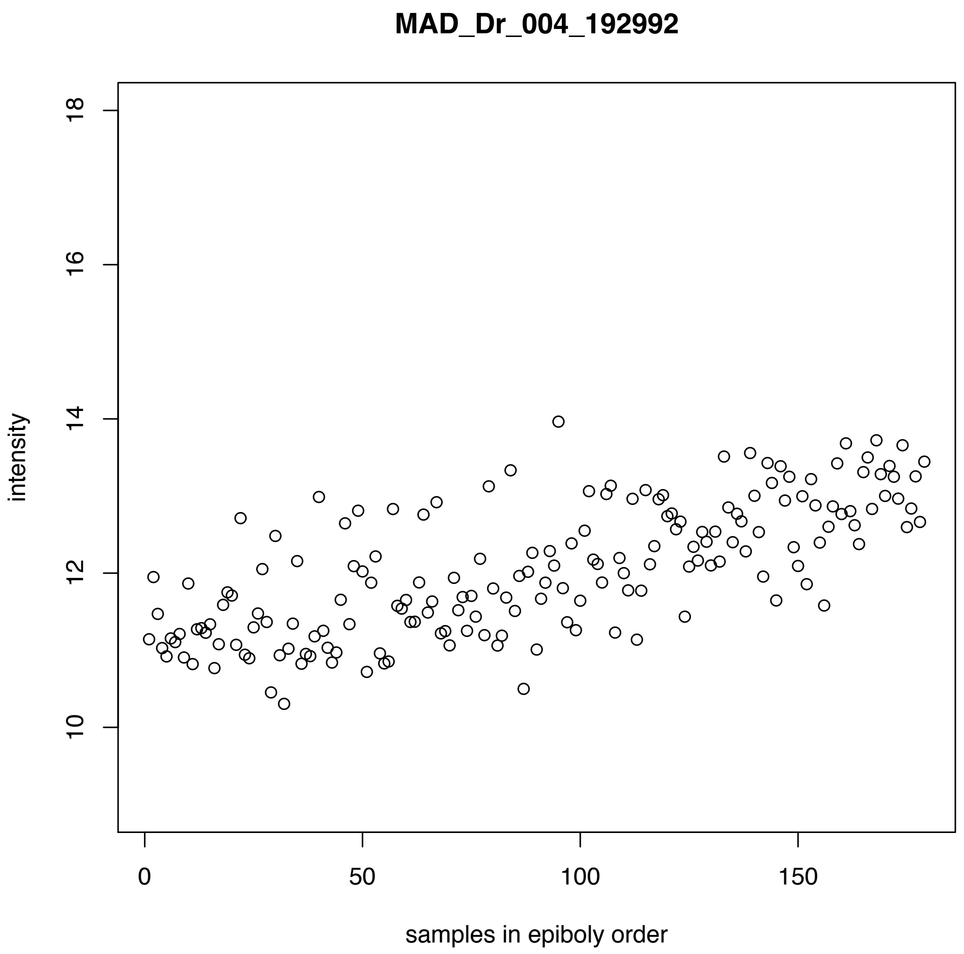
MAD_Dr_004_192992
intensity
10
12
14
16
18
0
50
100
150
samples in epiboly order

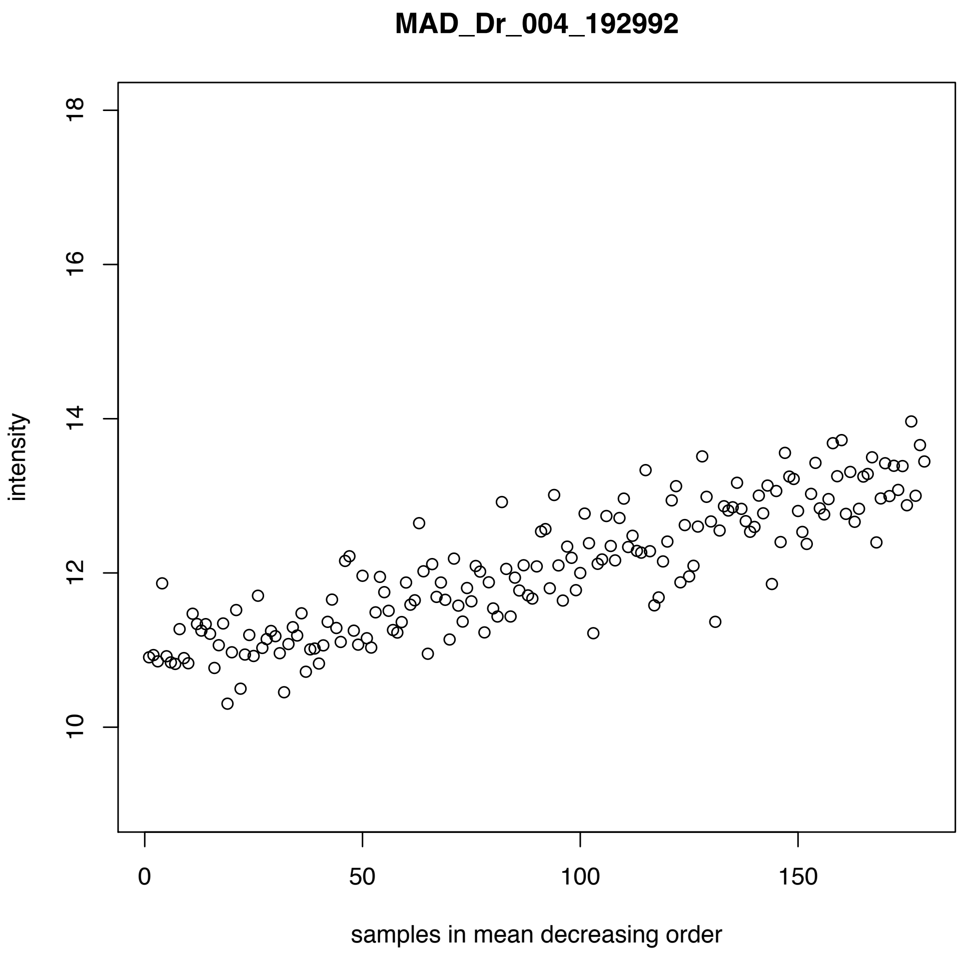
MAD_Dr_004_192992
intensity
10
12
14
16
18
0
50
100
150
samples in mean decreasing order

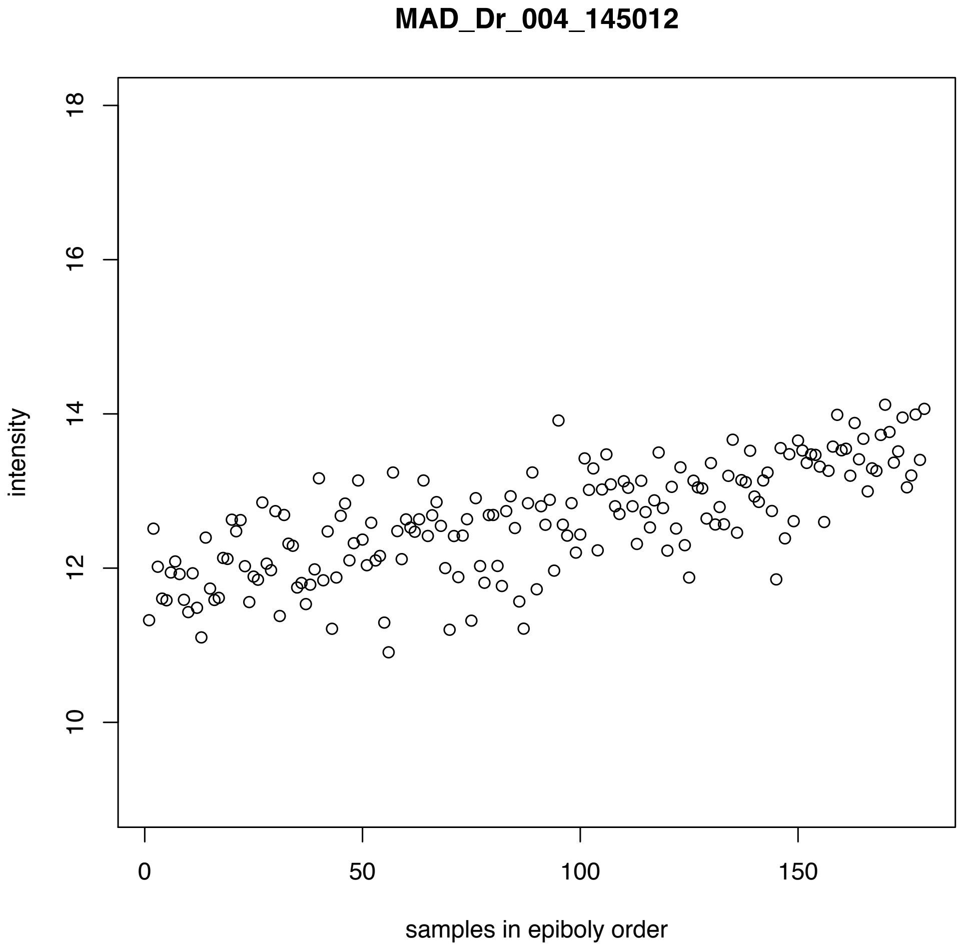
MAD_Dr_004_145012
intensity
18
16
14
12
10
0
50
100
150
samples in epiboly order

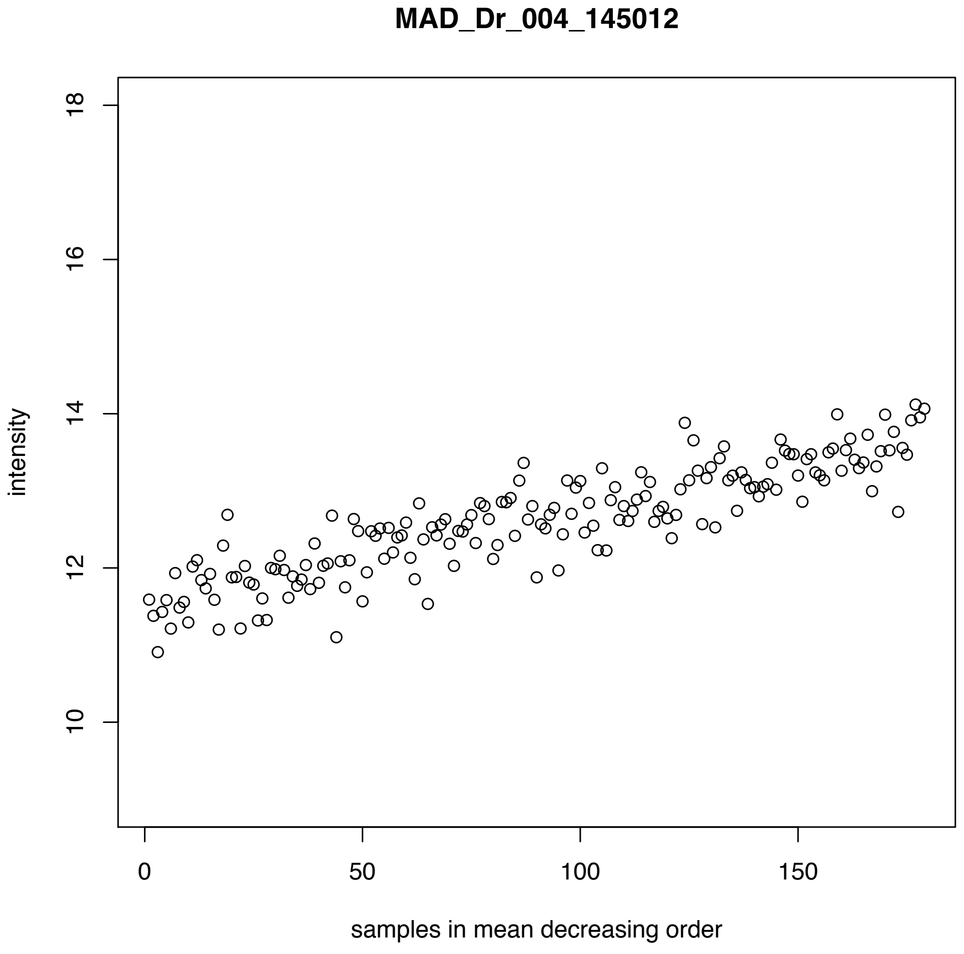
MAD_Dr_004_145012
intensity
18
16
14
12
10
0
50
100
150
samples in mean decreasing order

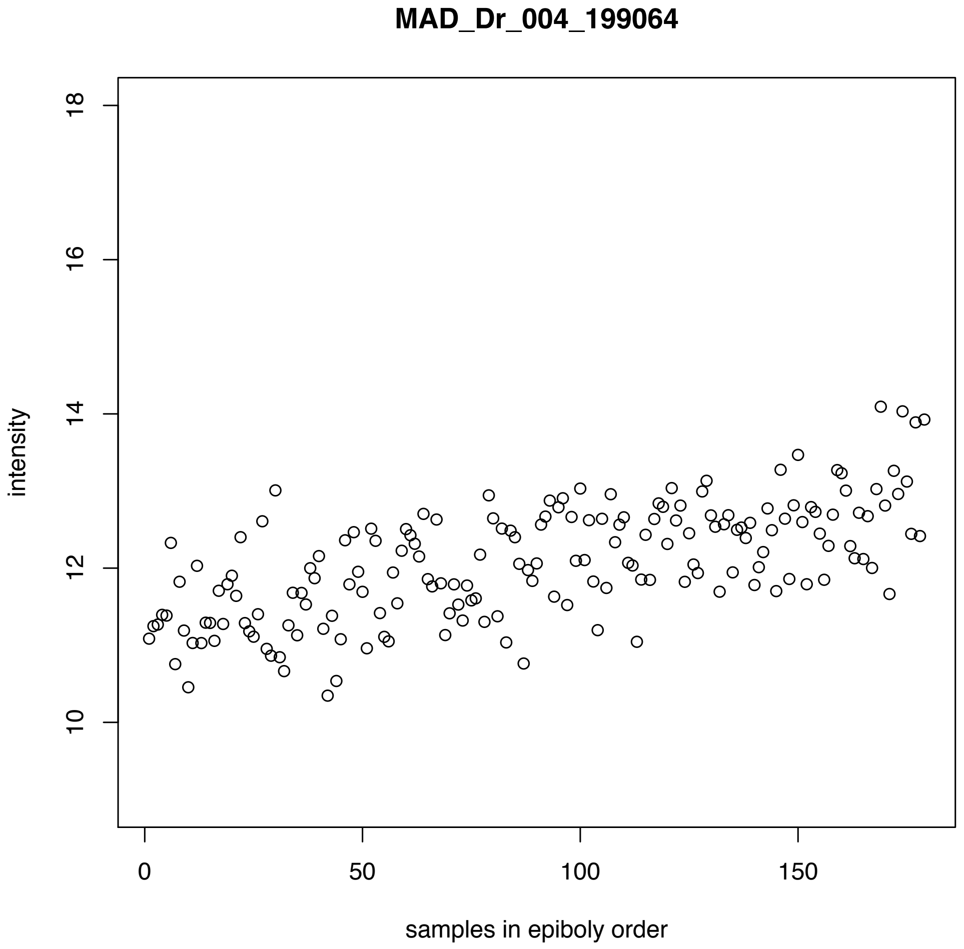
MAD_Dr_004_199064
intensity
samples in epiboly order
10
12
14
16
18
0
50
100
150

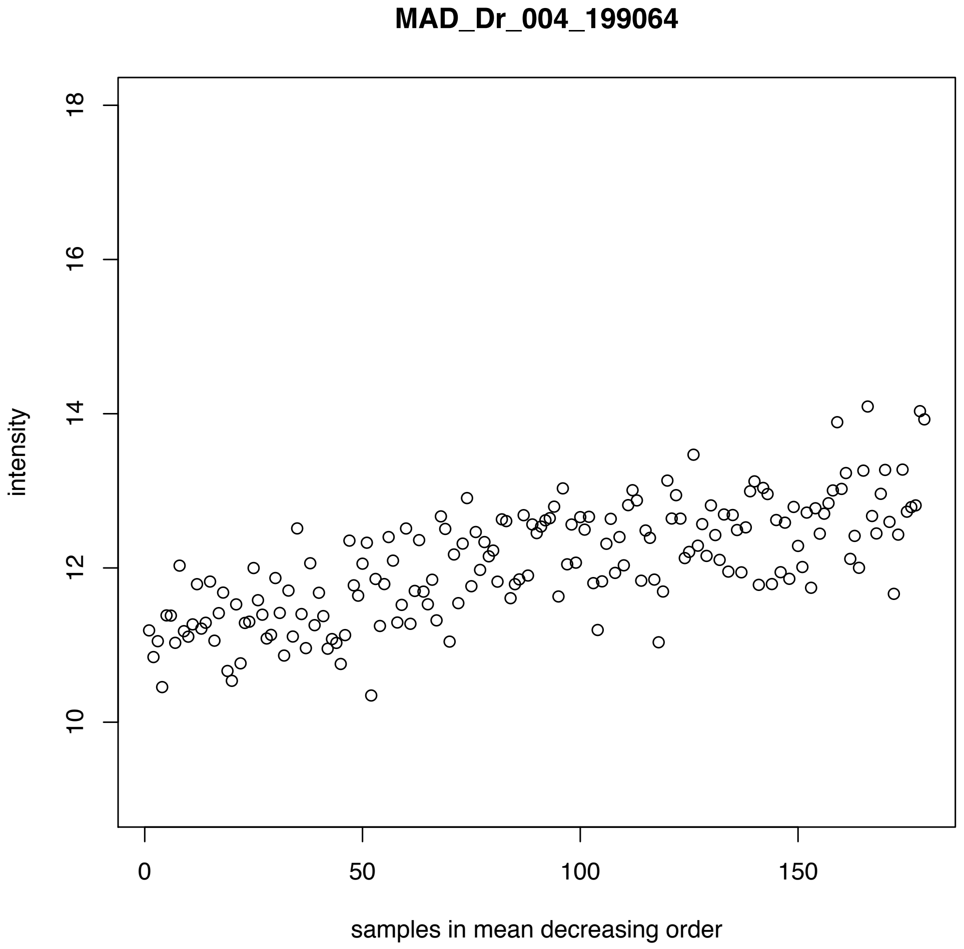
MAD_Dr_004_199064
intensity
samples in mean decreasing order
10
12
14
16
18
0
50
100
150

MAD_Dr_004_112669

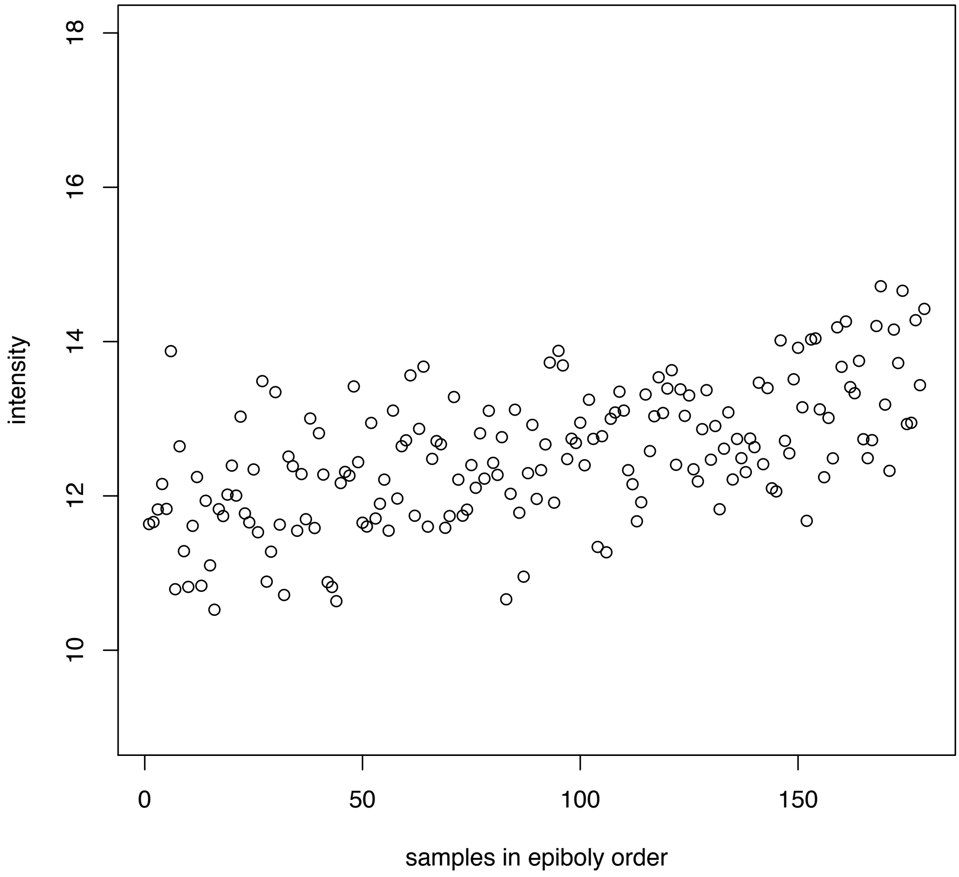

MAD_Dr_004_112669

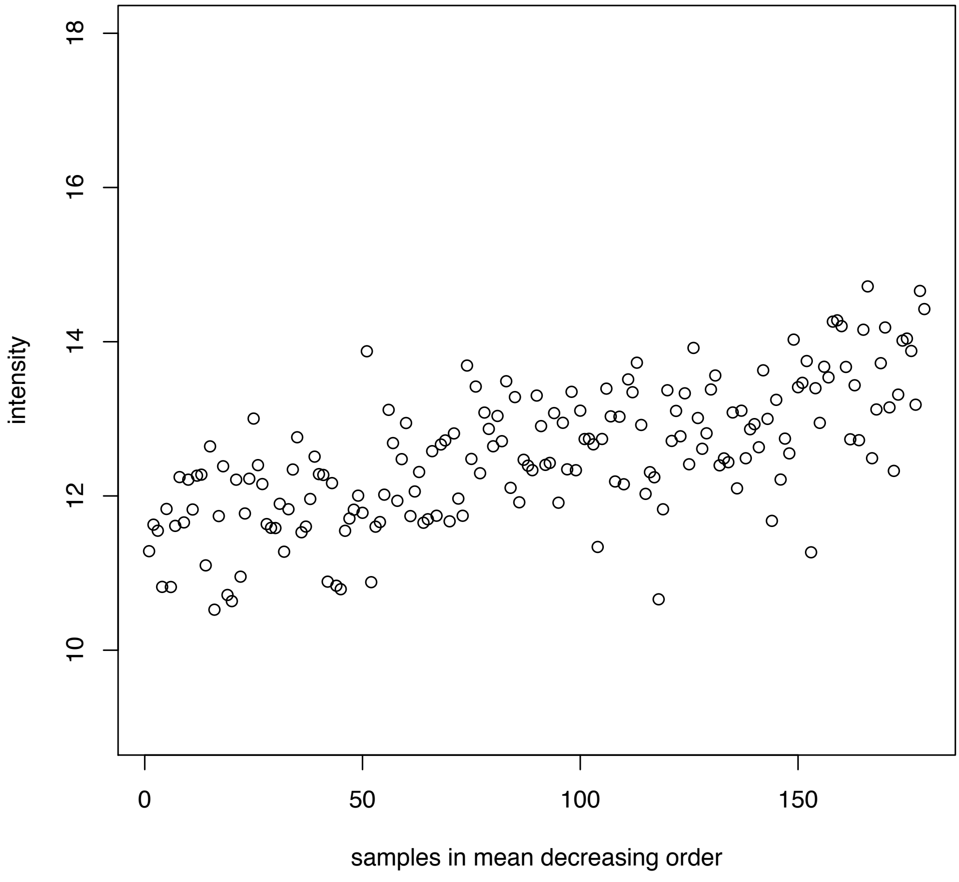

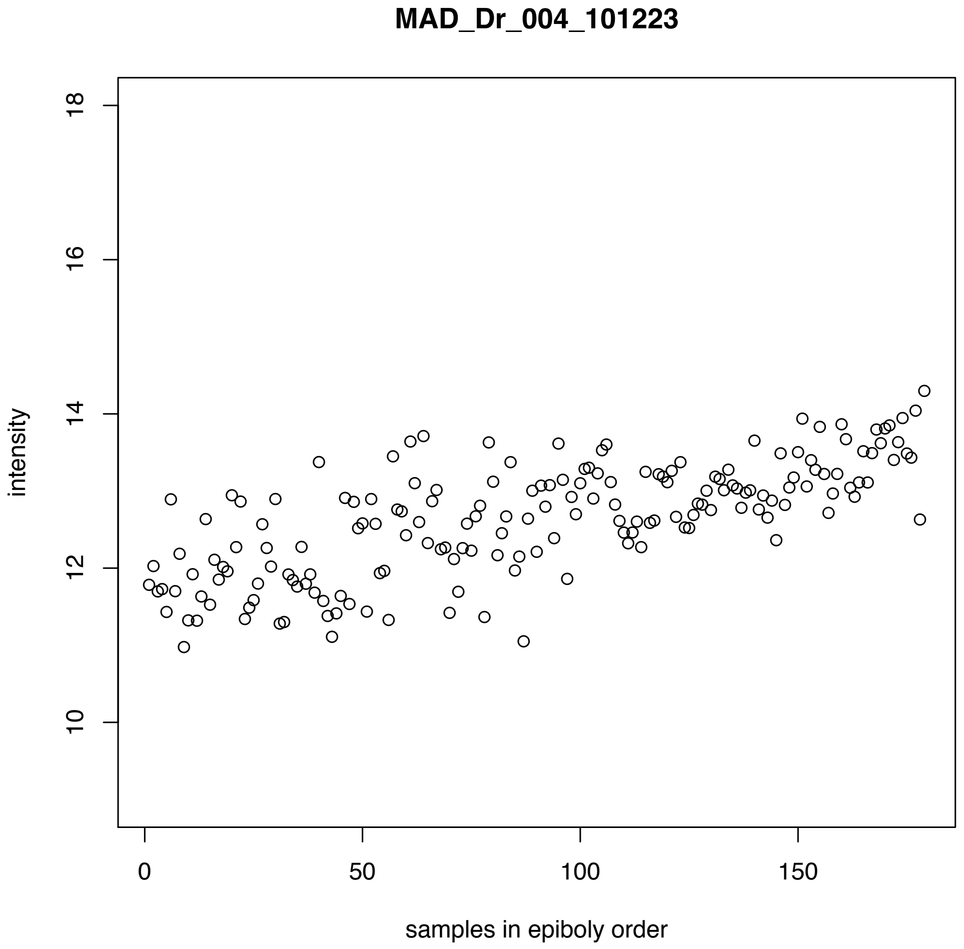
MAD_Dr_004_101223
intensity
10
12
14
16
18
0
50
100
150
samples in epiboly order

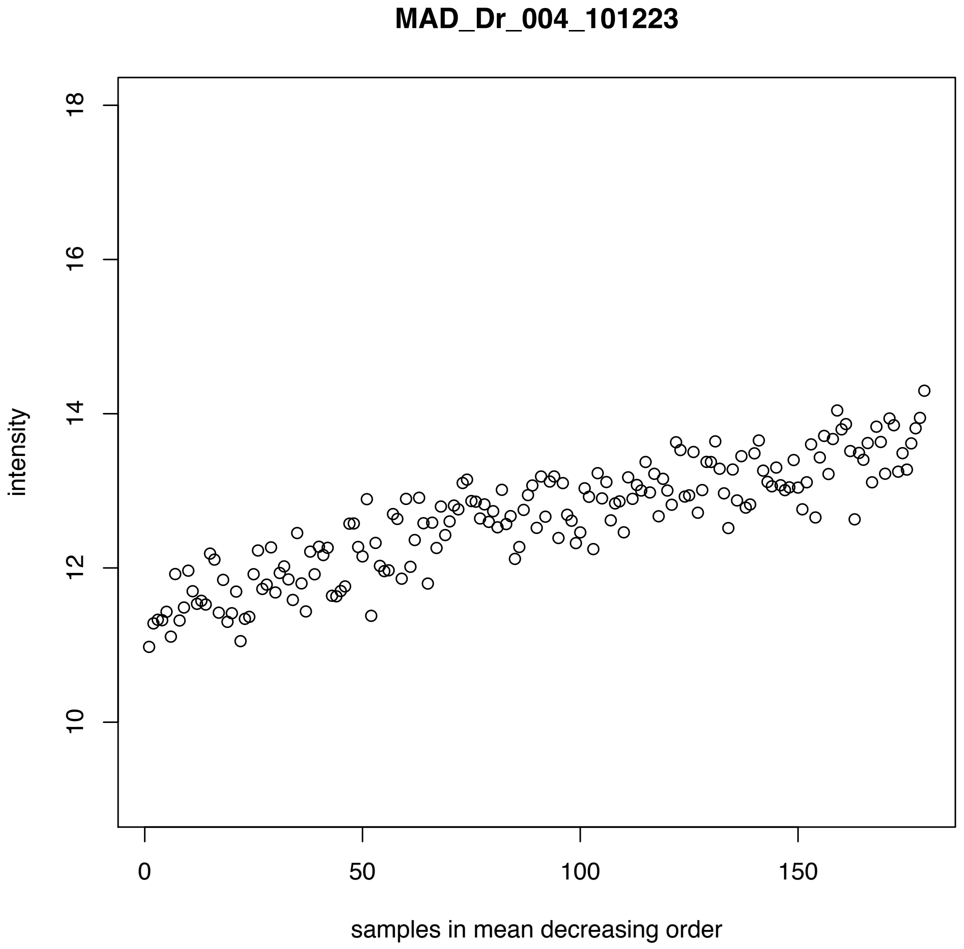
MAD_Dr_004_101223
intensity
10
12
14
16
18
0
50
100
150
samples in mean decreasing order

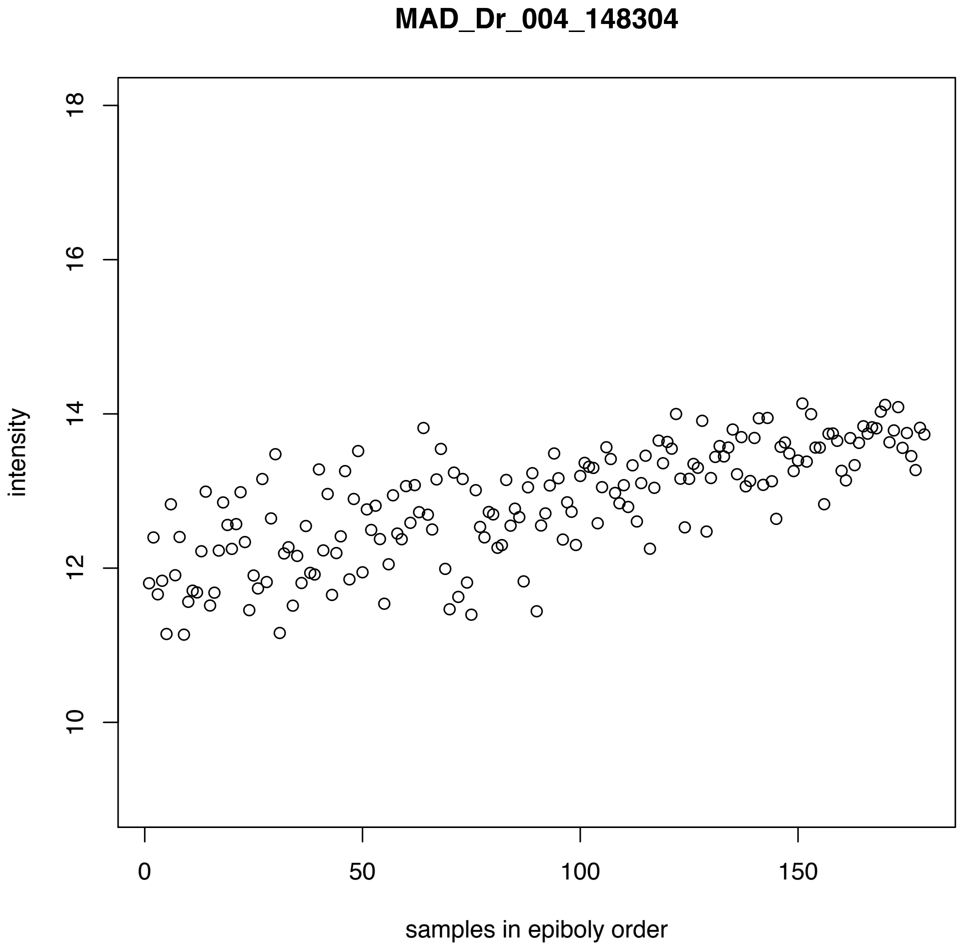
MAD_Dr_004_148304
intensity
10
12
14
16
18
0
50
100
150
samples in epiboly order

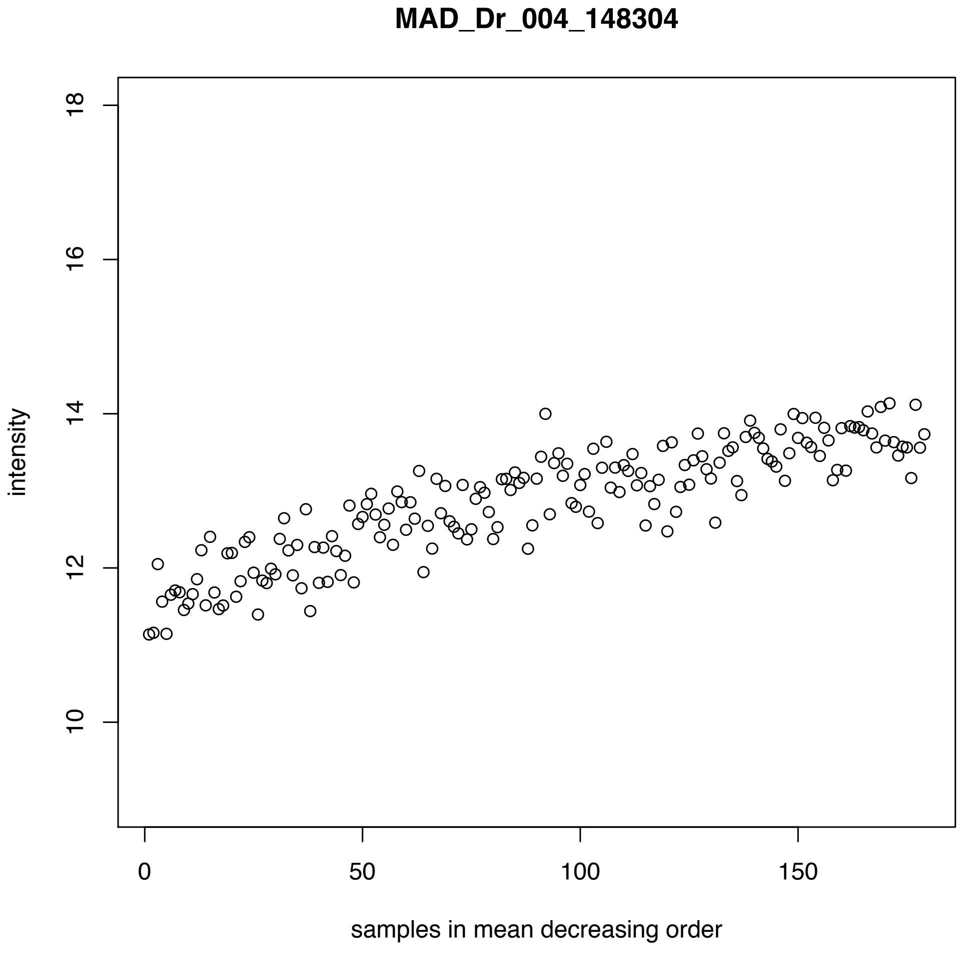
MAD_Dr_004_148304
intensity
10
12
14
16
18
0
50
100
150
samples in mean decreasing order

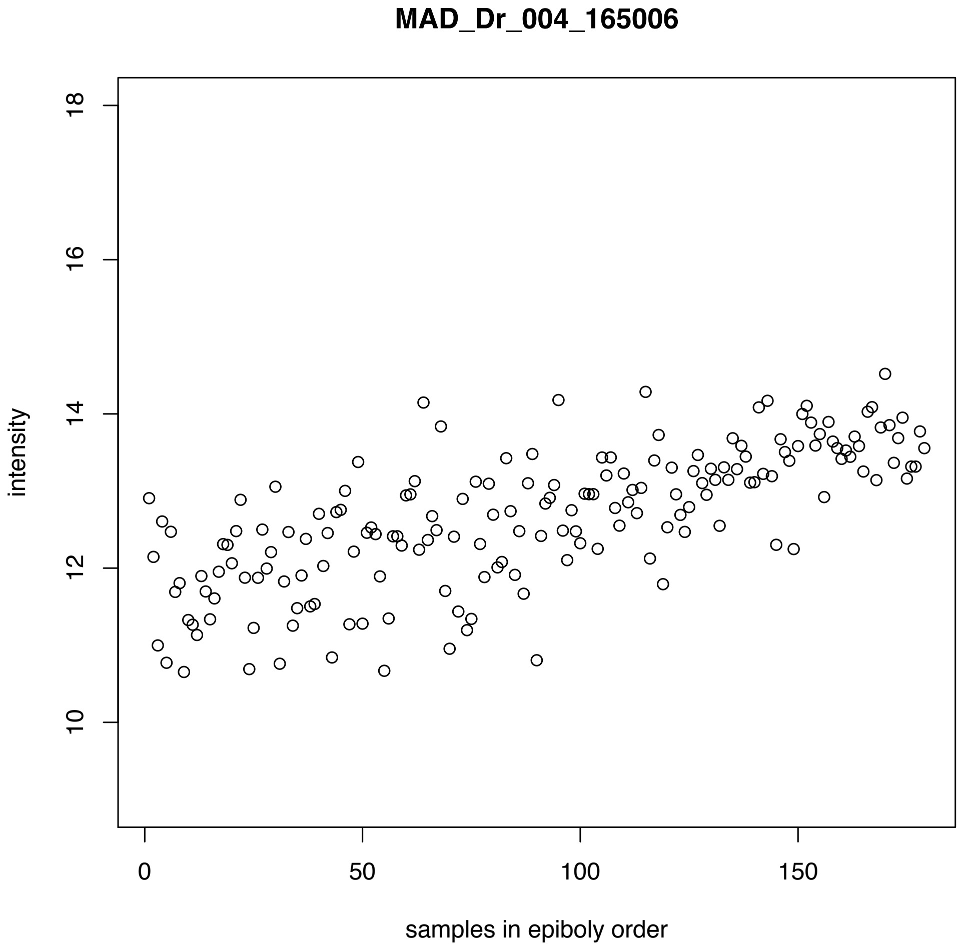
MAD_Dr_004_165006
intensity
10
12
14
16
18
0
50
100
150
samples in epiboly order

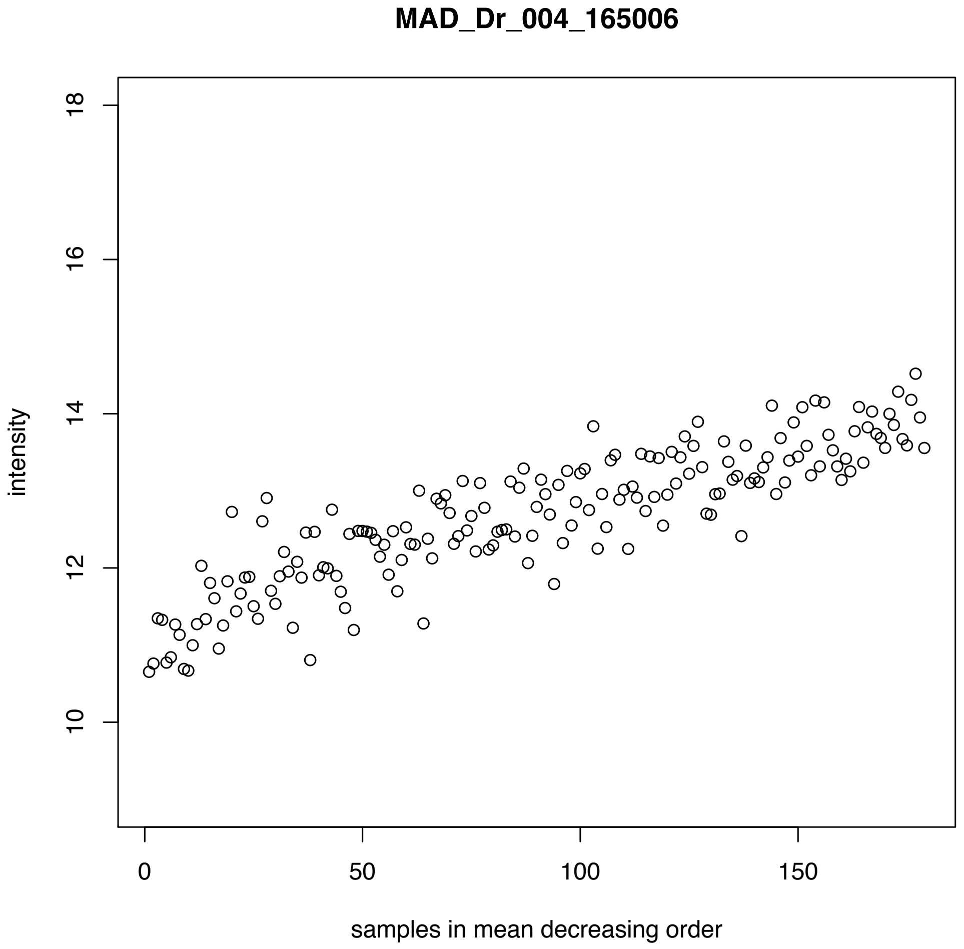
MAD_Dr_004_165006
intensity
10
12
14
16
18
0
50
100
150
samples in mean decreasing order

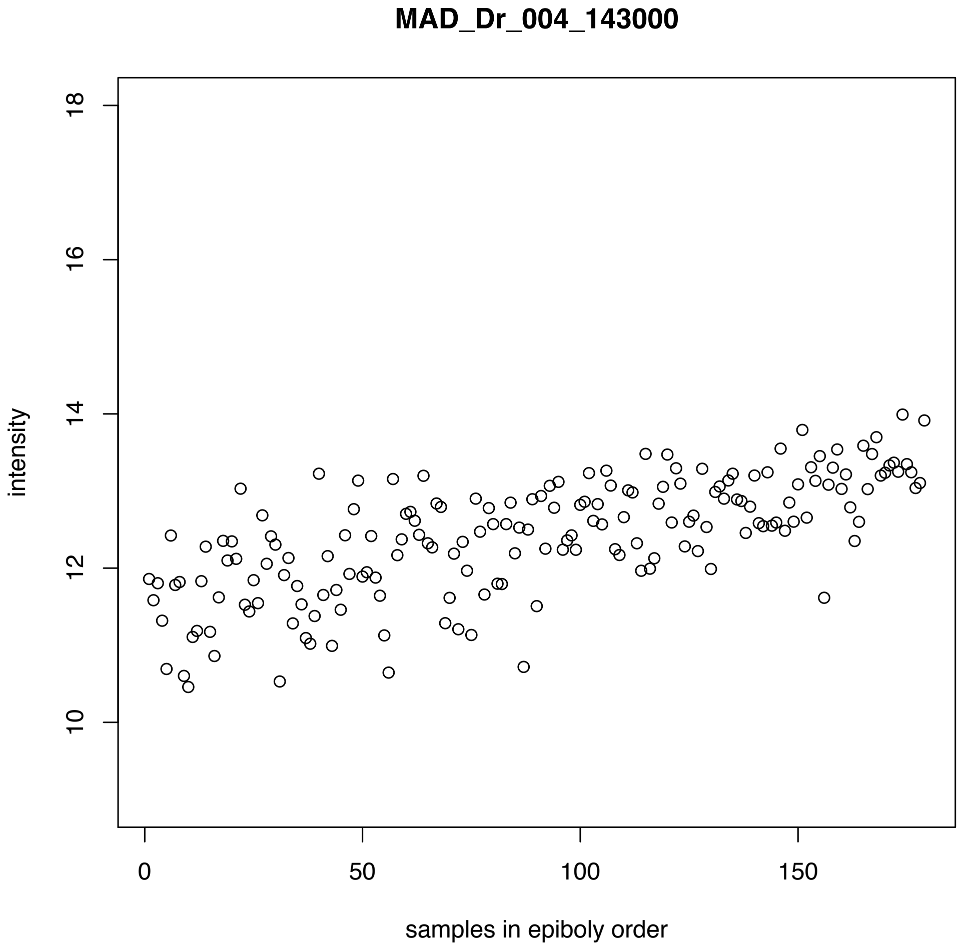

MAD_Dr_004_143000
intensity
samples in epiboly order
0
50
100
150
10
12
14
16
18

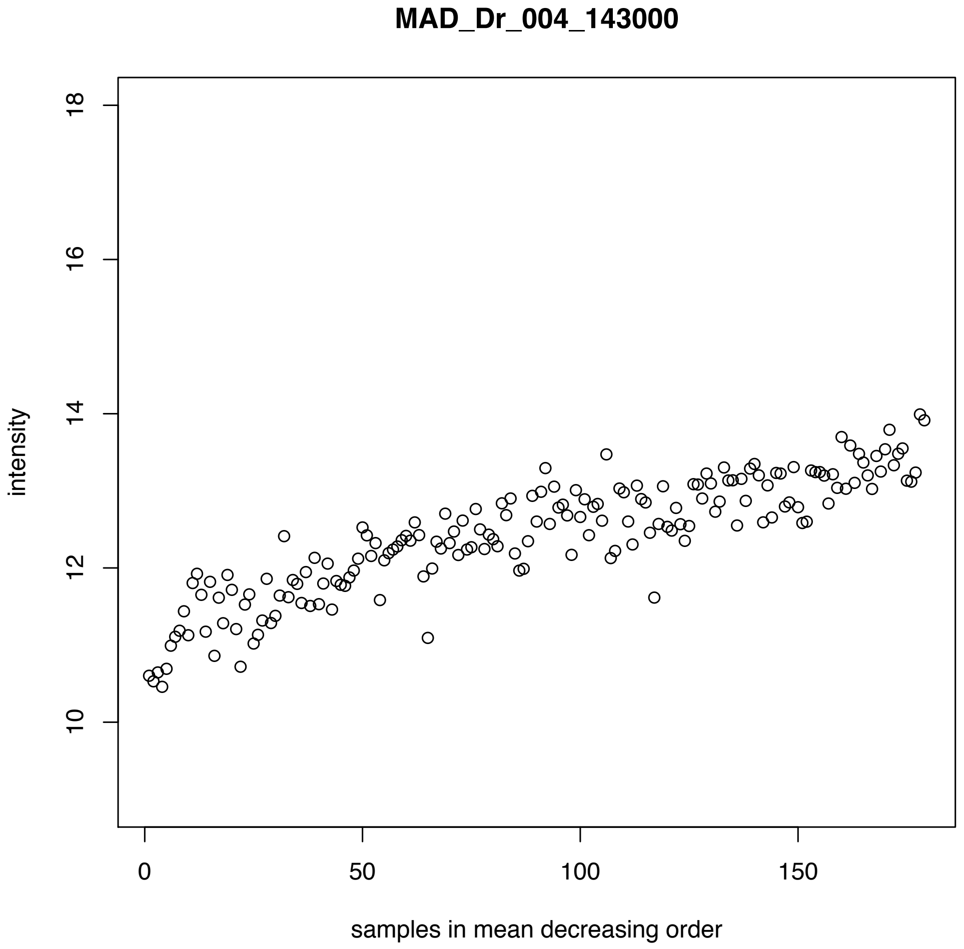

MAD_Dr_004_143000
intensity
samples in mean decreasing order
0
50
100
150
10
12
14
16
18

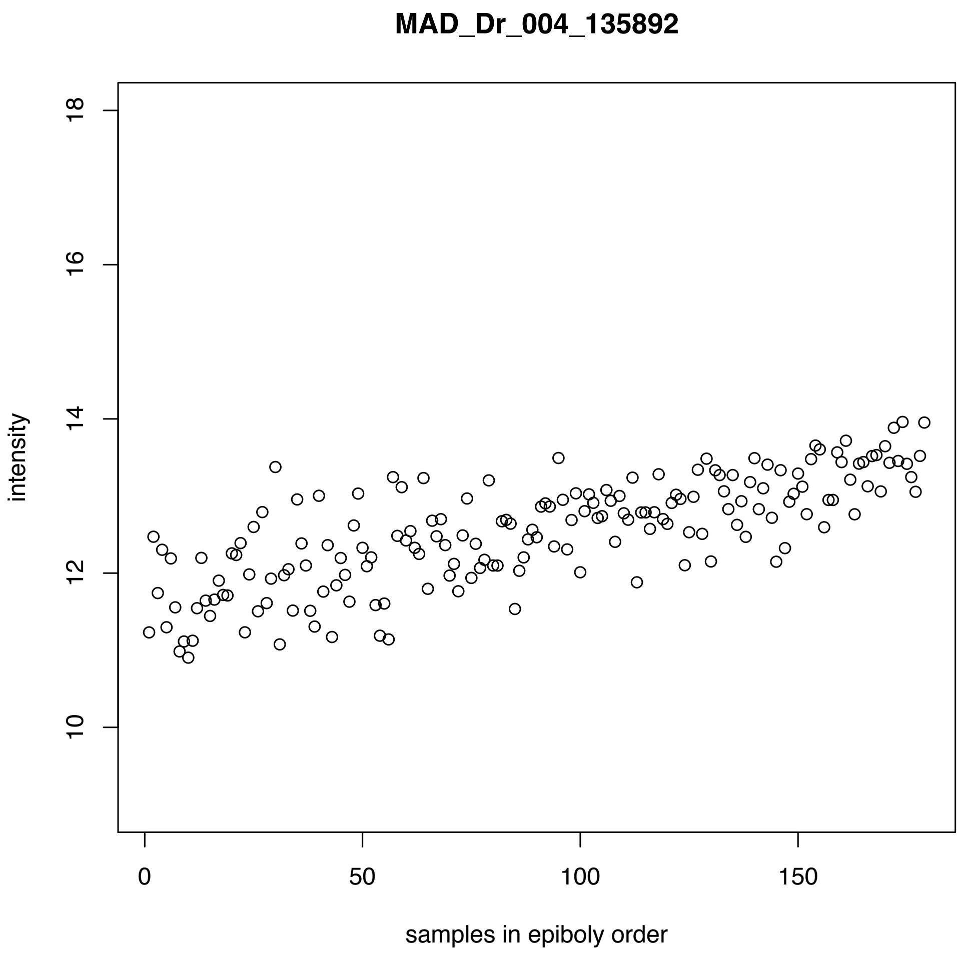
MAD_Dr_004_135892
intensity
10
12
14
16
18
0
50
100
150
samples in epiboly order

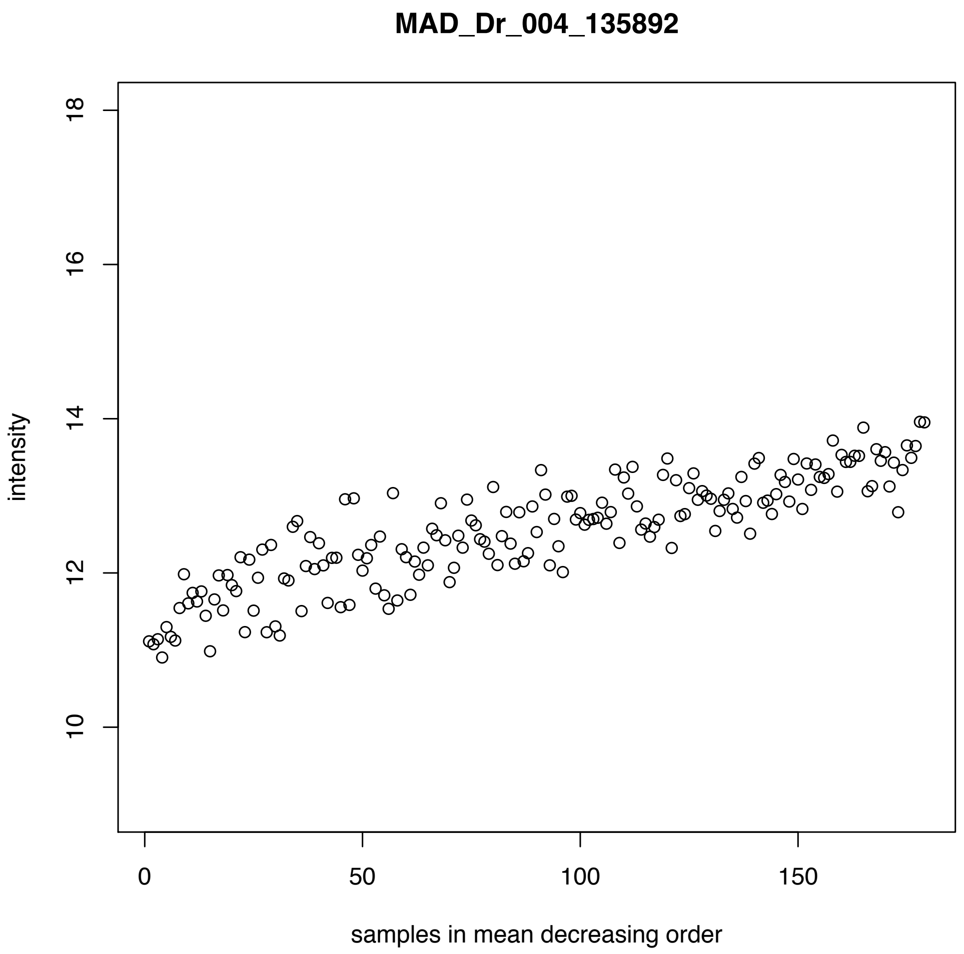
MAD_Dr_004_135892
intensity
10
12
14
16
18
0
50
100
150
samples in mean decreasing order

MAD_Dr_004_141775

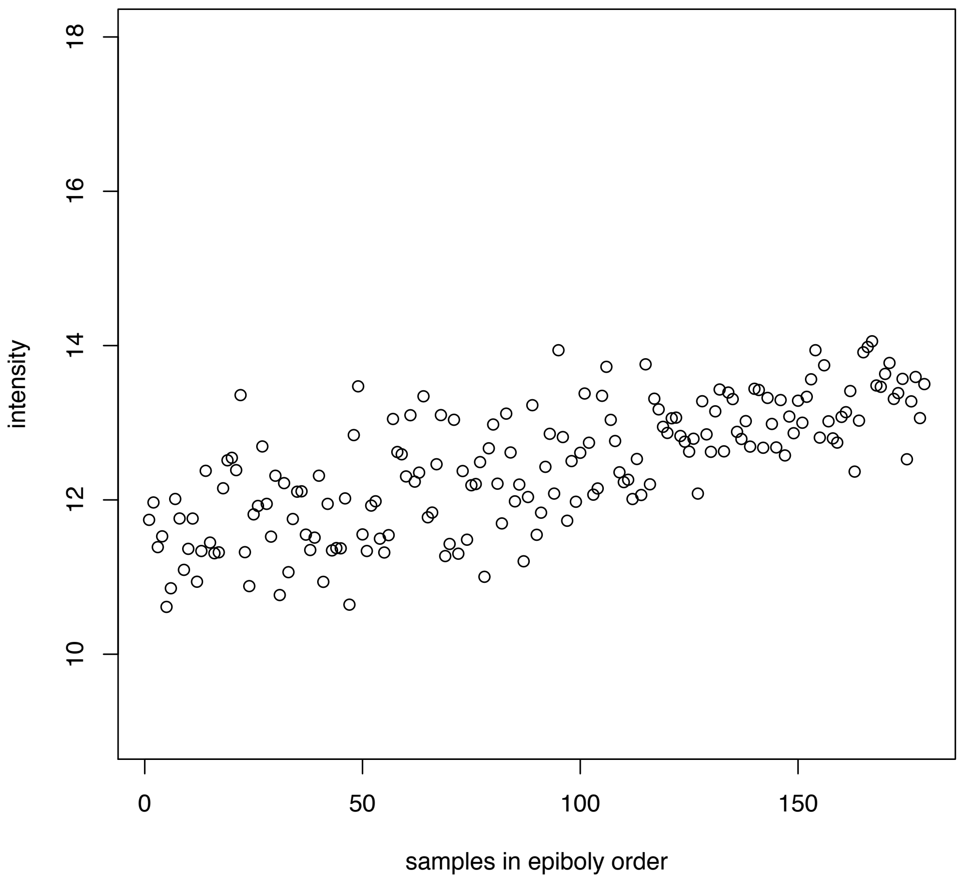

MAD_Dr_004_141775

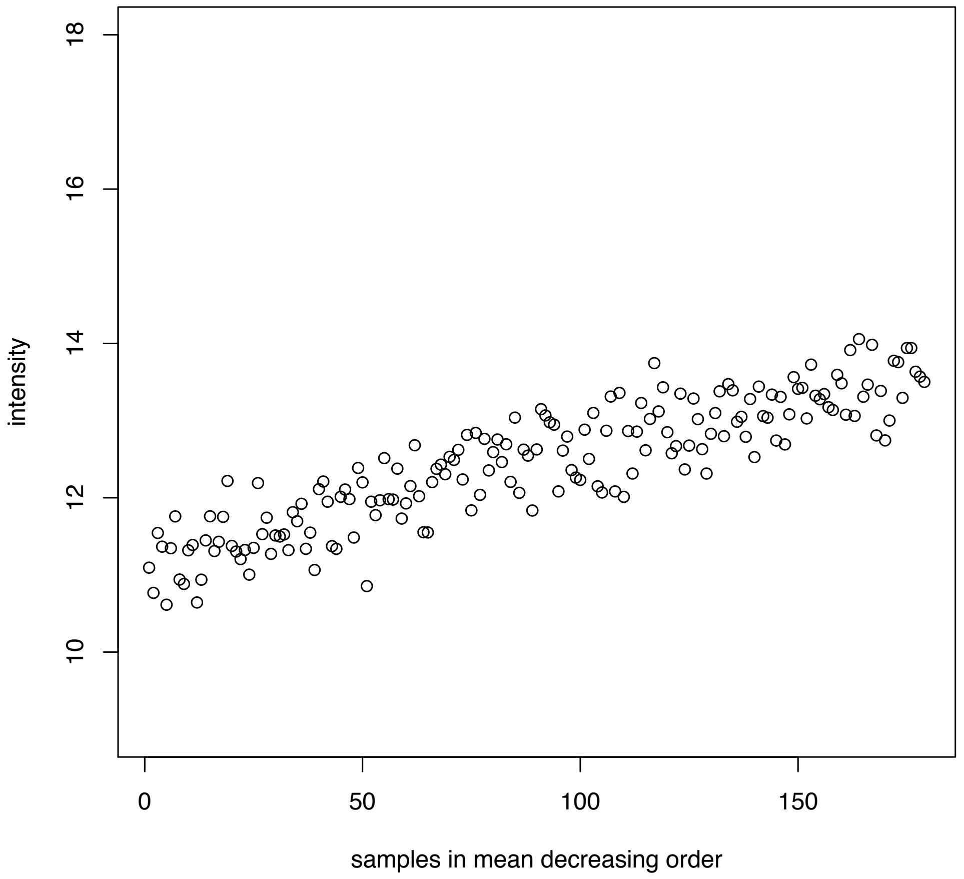

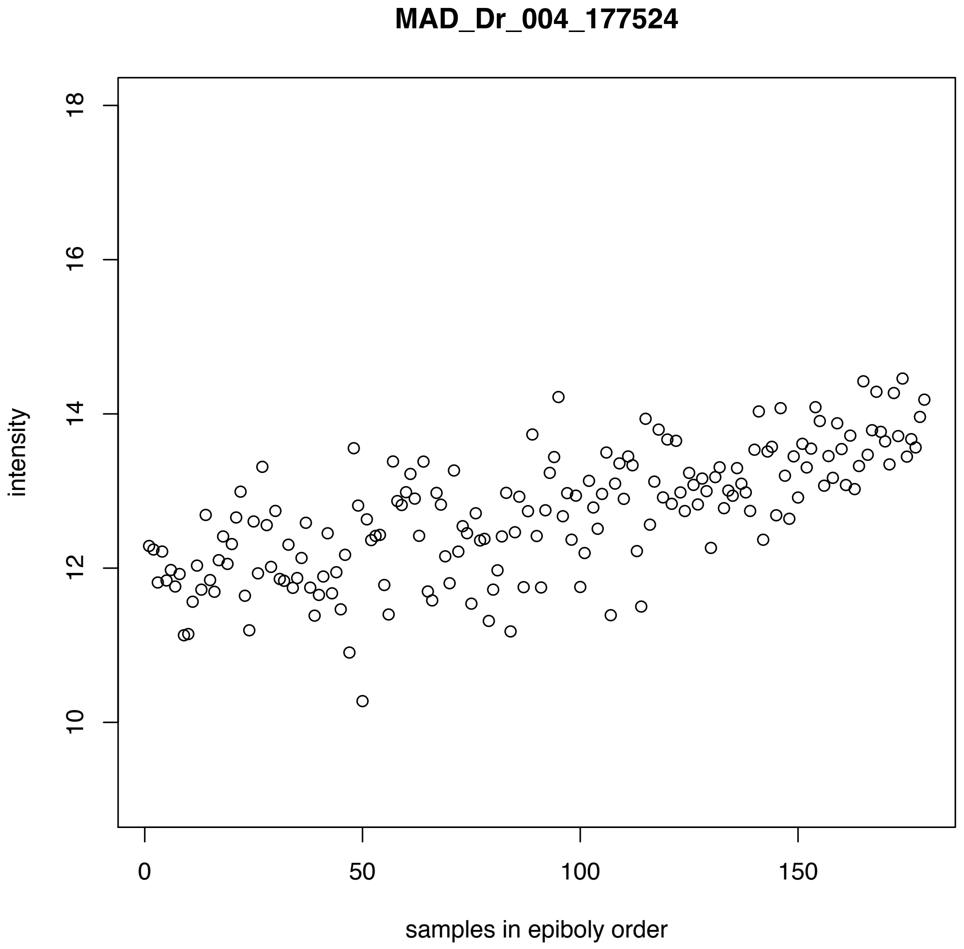
MAD_Dr_004_177524
intensity
10
12
14
16
18
0
50
100
150
samples in epiboly order

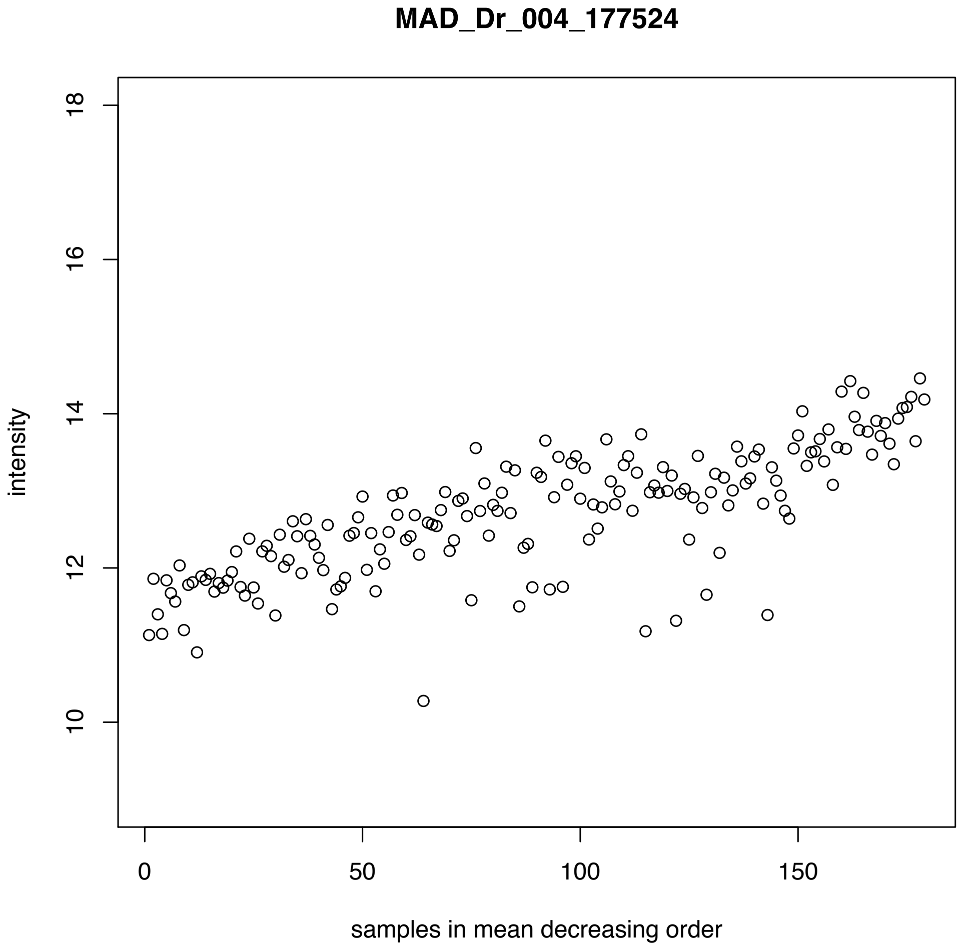
MAD_Dr_004_177524
intensity
10
12
14
16
18
0
50
100
150
samples in mean decreasing order

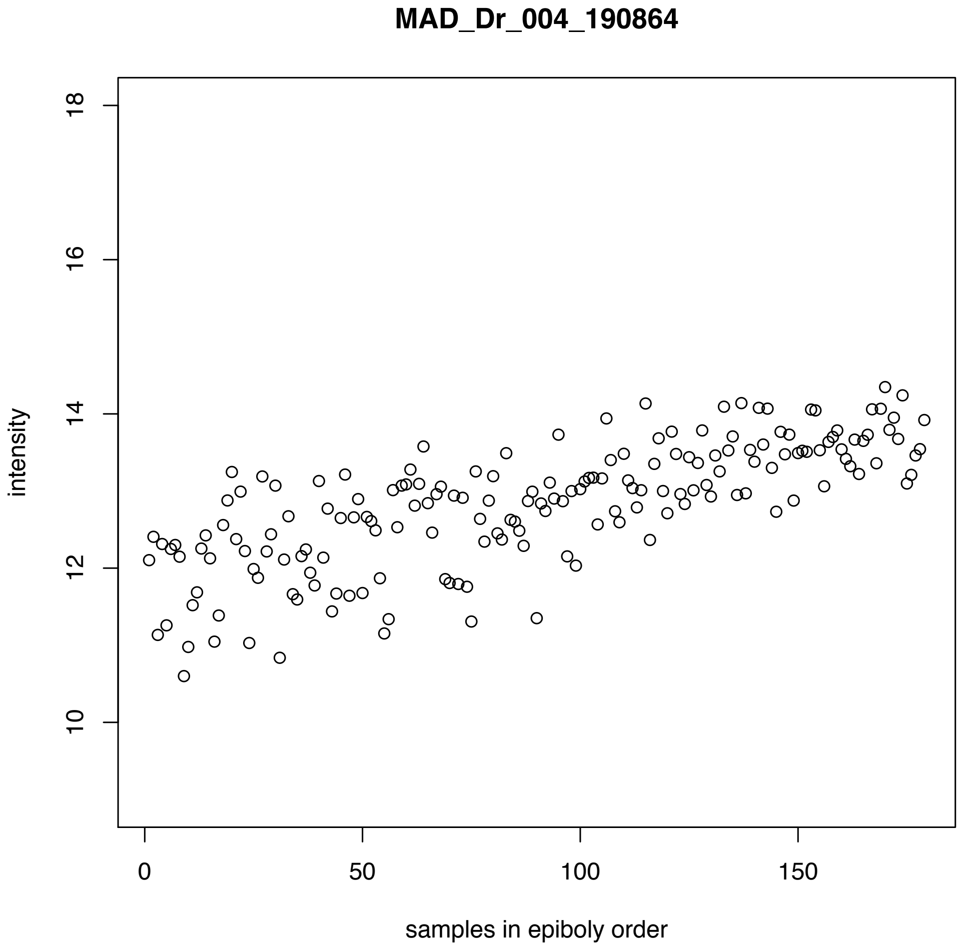
MAD_Dr_004_190864
intensity
samples in epiboly order
0
50
100
150
10
12
14
16
18

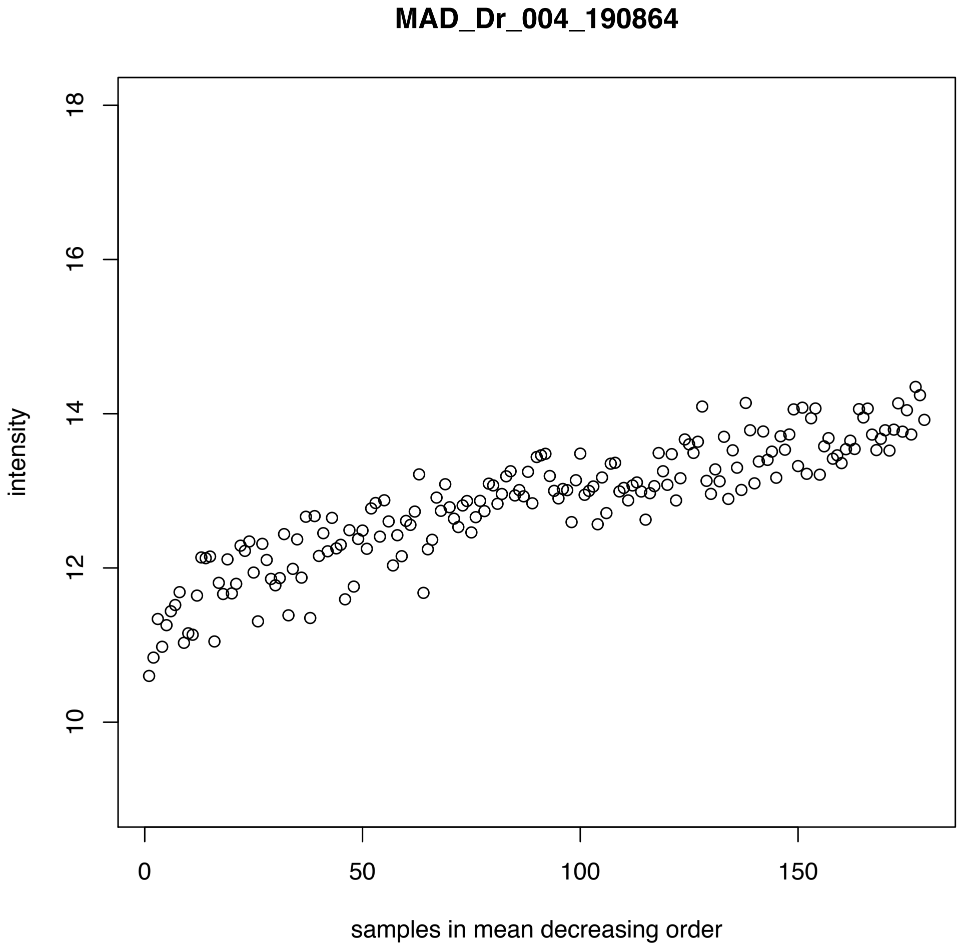
MAD_Dr_004_190864
intensity
samples in mean decreasing order
0
50
100
150
10
12
14
16
18

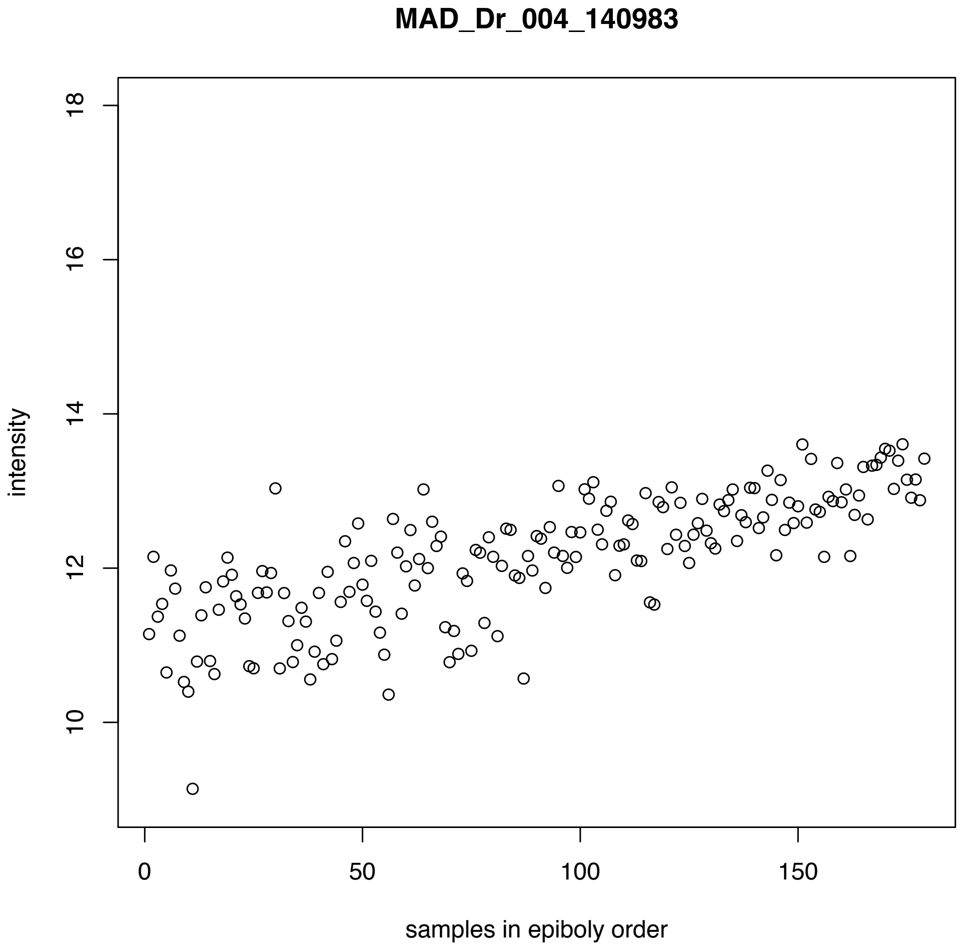
MAD_Dr_004_140983
intensity
10
12
14
16
18
0
50
100
150
samples in epiboly order

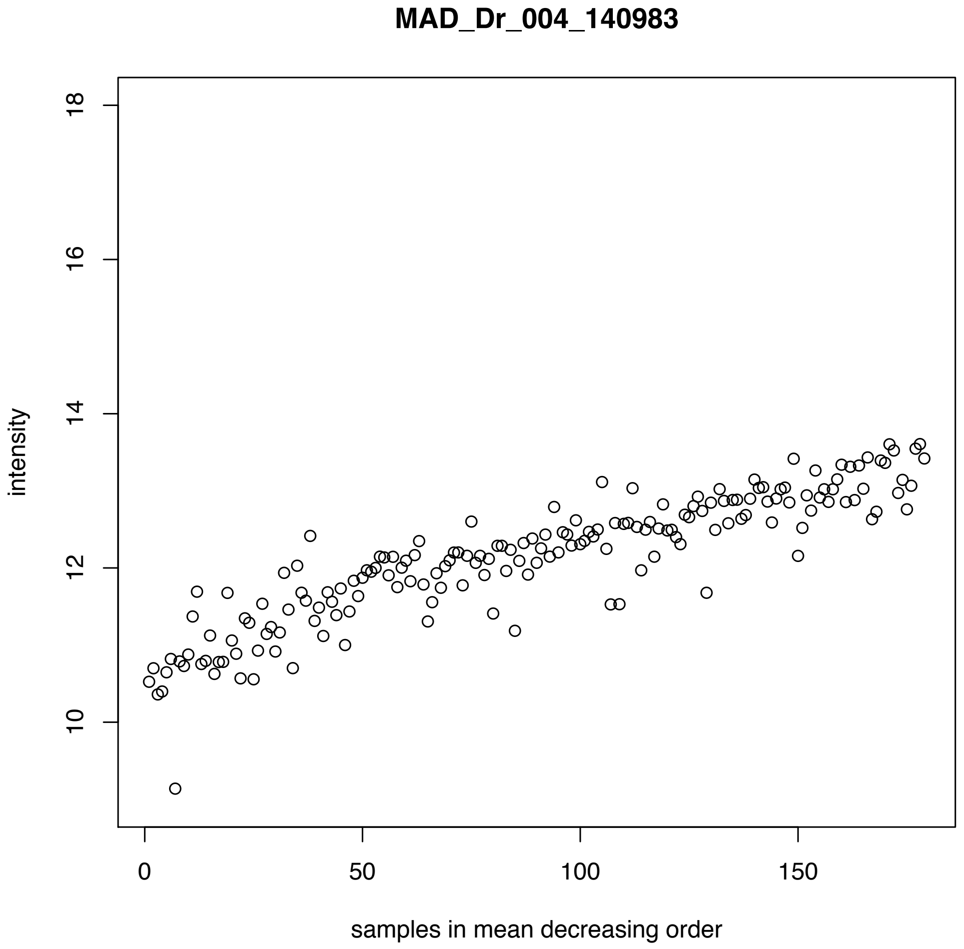
MAD_Dr_004_140983
intensity
10
12
14
16
18
0
50
100
150
samples in mean decreasing order

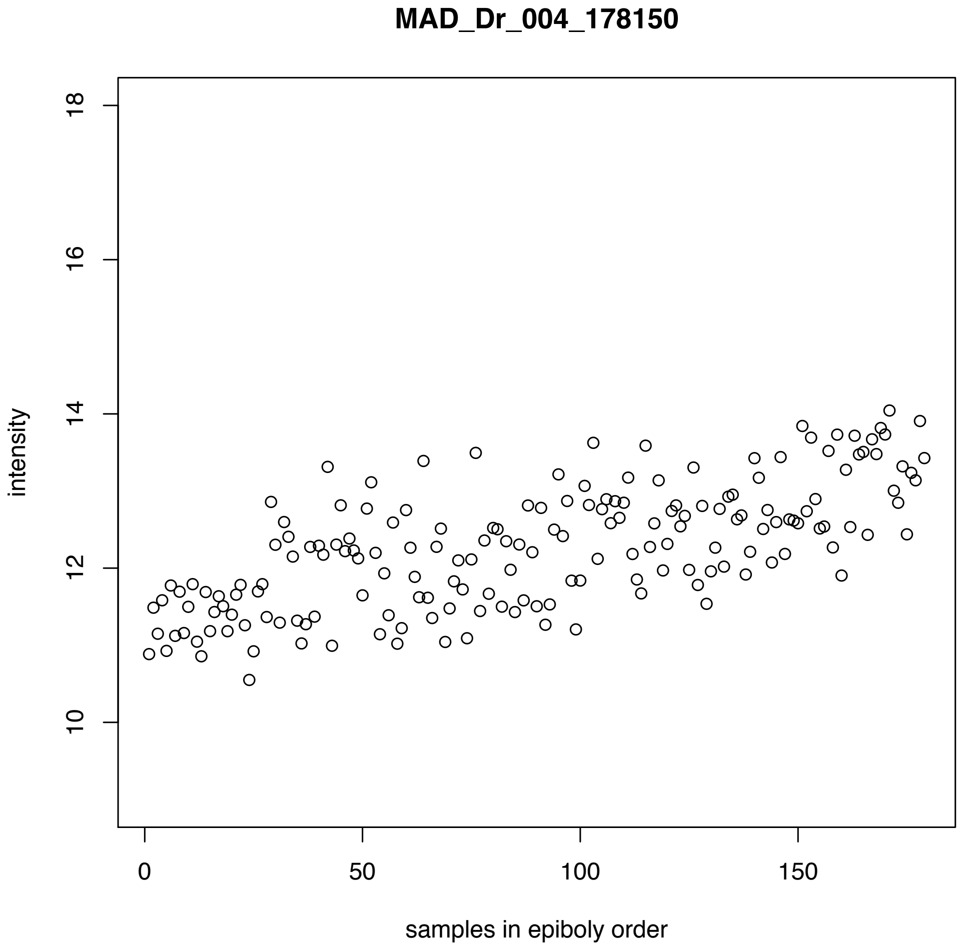

MAD_Dr_004_178150
intensity
samples in epiboly order

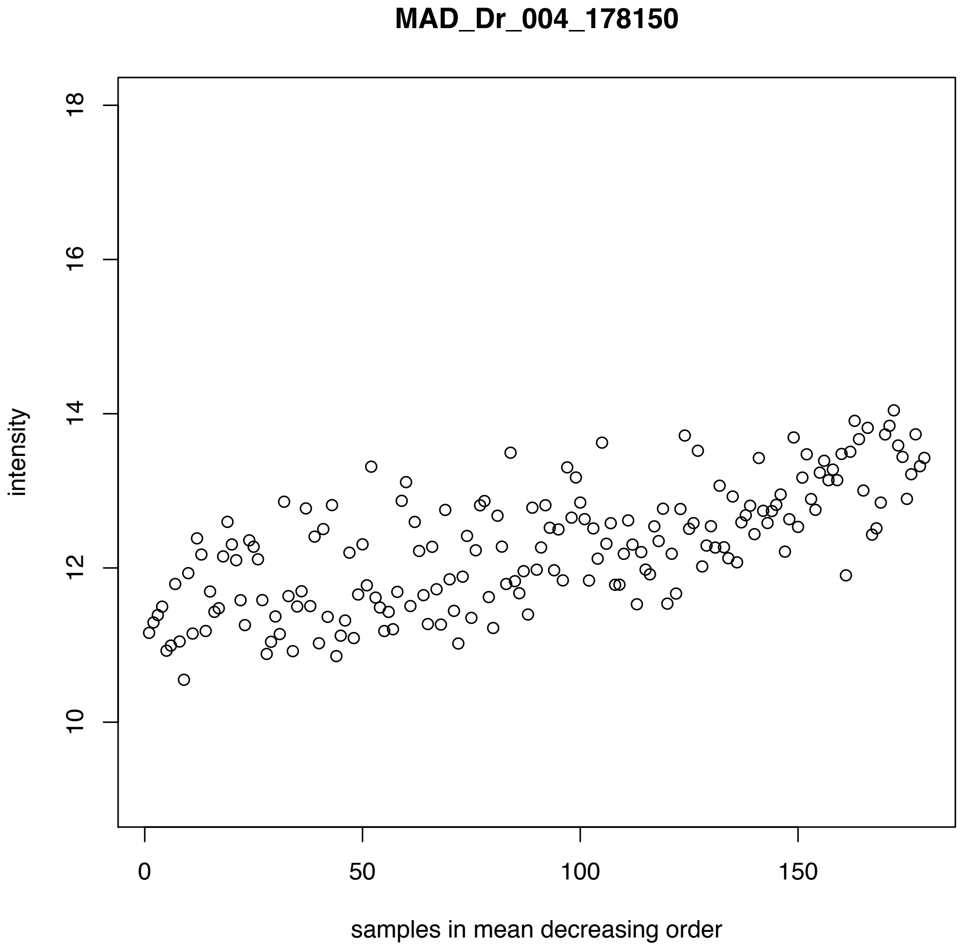

MAD_Dr_004_178150
intensity
samples in mean decreasing order

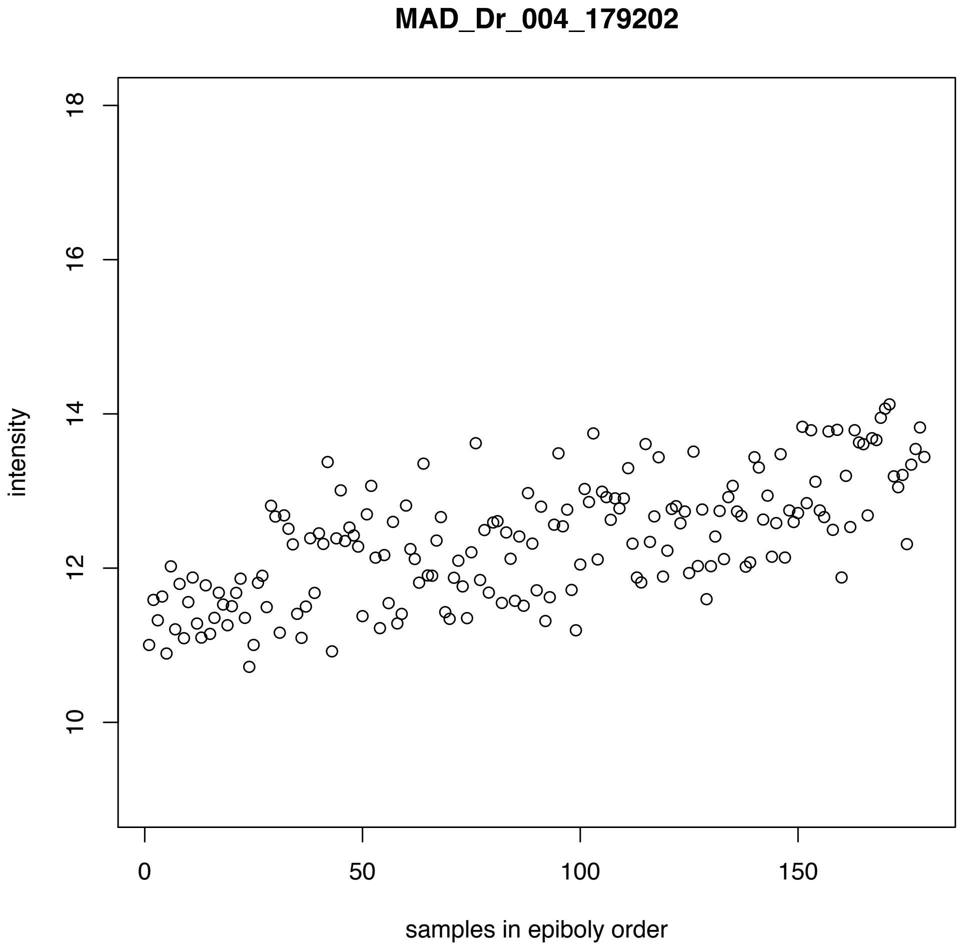

MAD_Dr_004_179202
intensity
18
16
14
12
10
0
50
100
150
samples in epiboly order

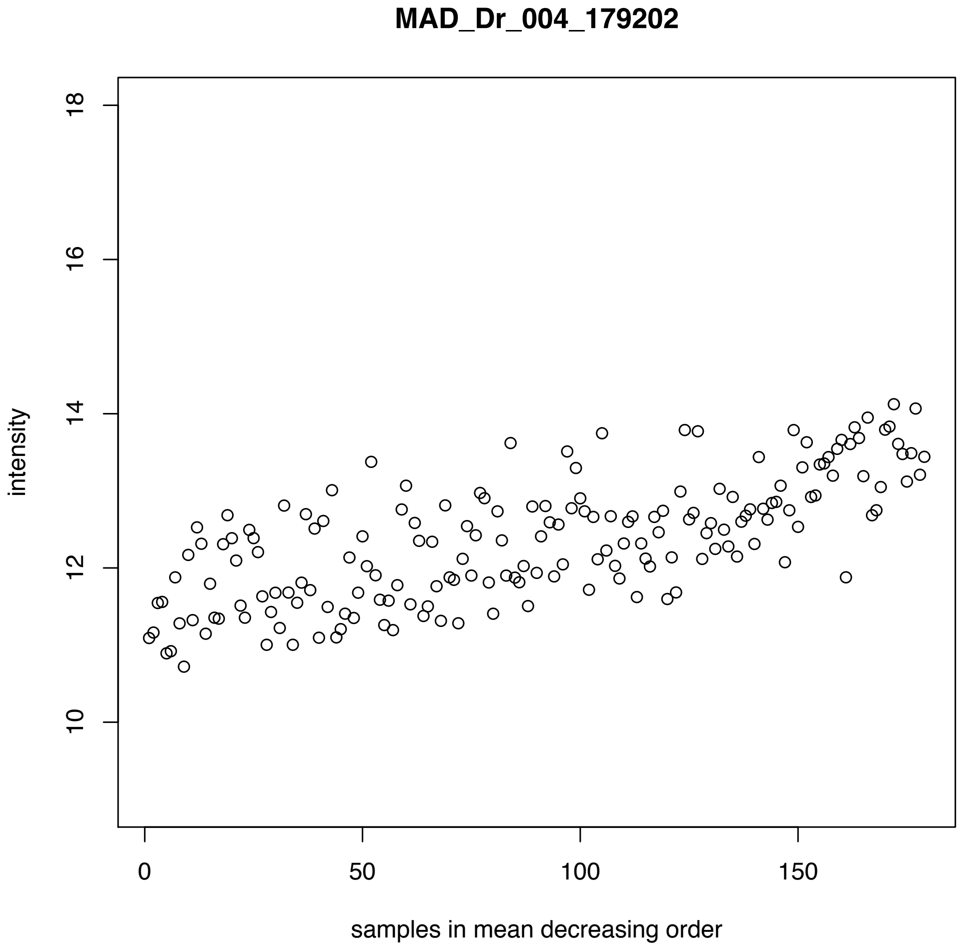

MAD_Dr_004_179202
intensity
18
16
14
12
10
0
50
100
150
samples in mean decreasing order

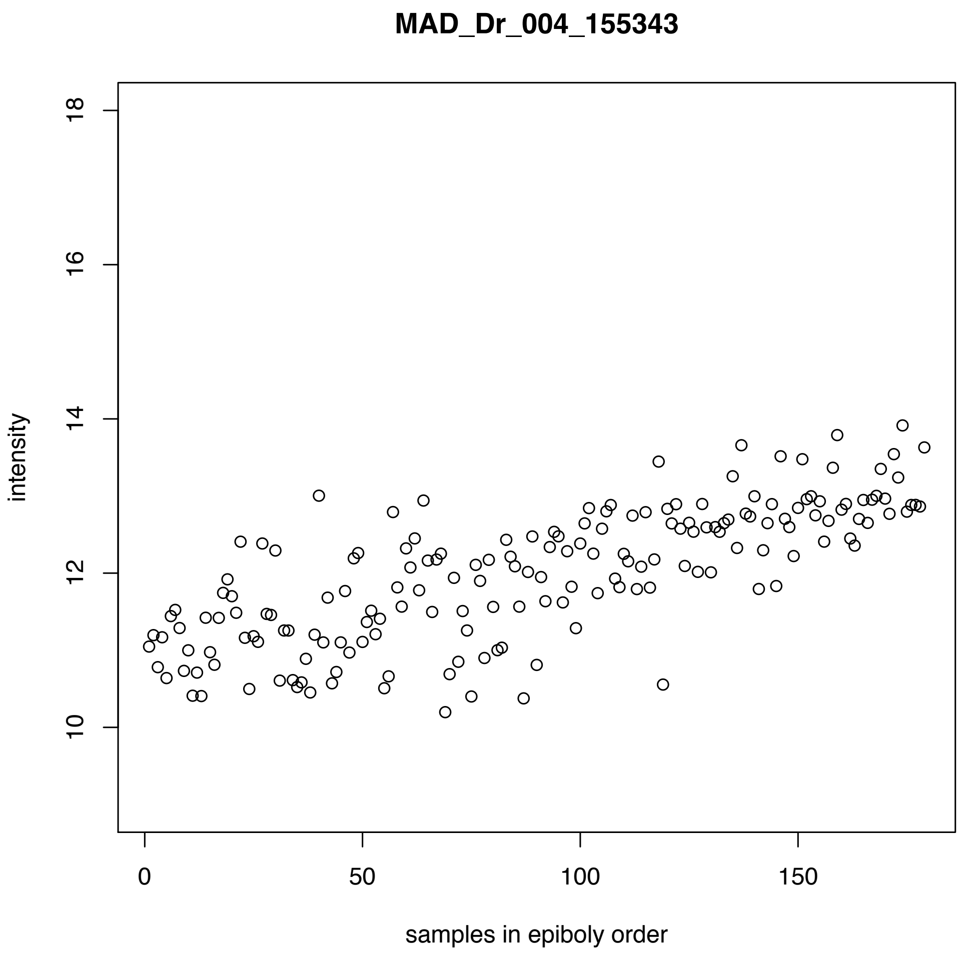
MAD_Dr_004_155343
intensity
10
12
14
16
18
0
50
100
150
samples in epiboly order

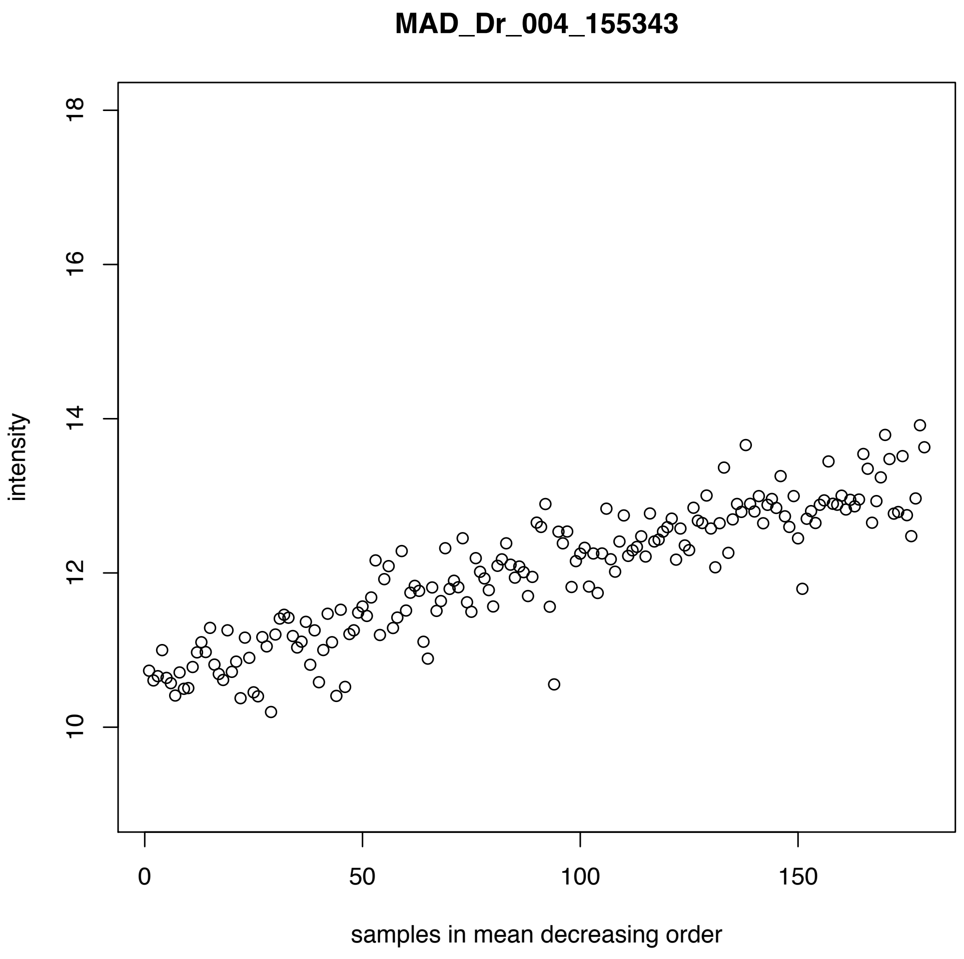
MAD_Dr_004_155343
intensity
10
12
14
16
18
0
50
100
150
samples in mean decreasing order

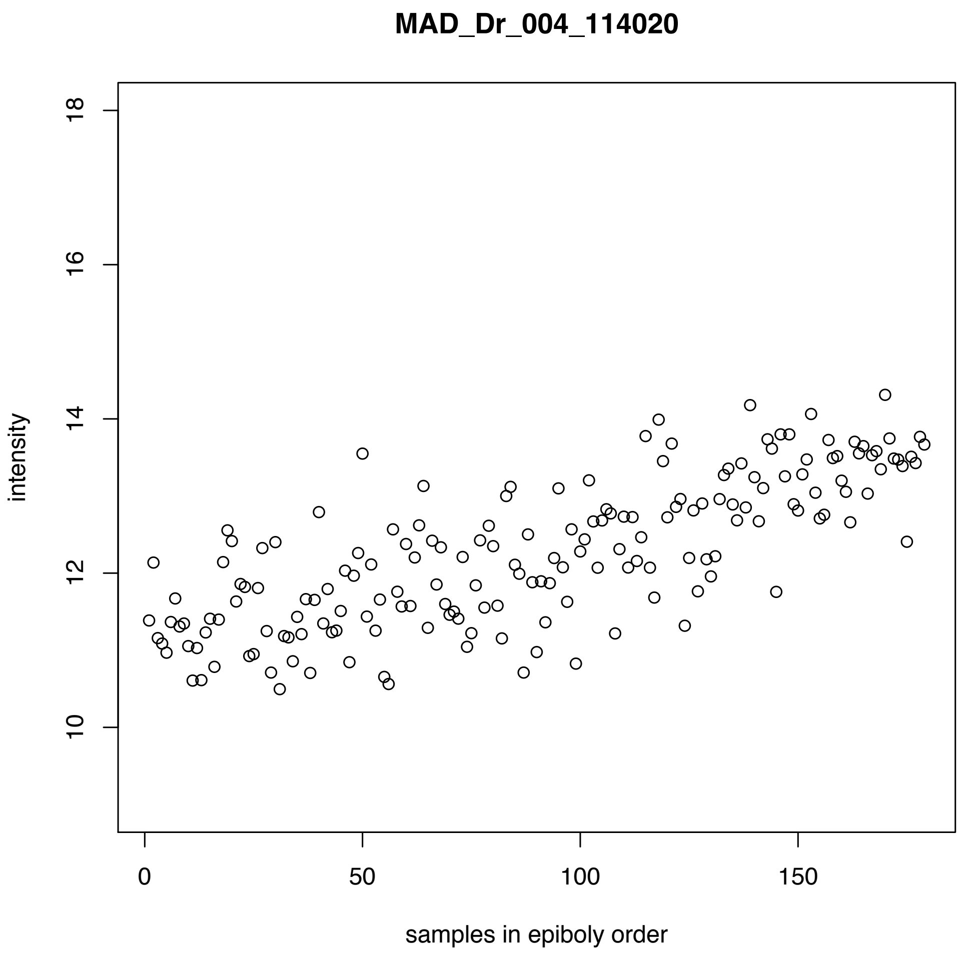
MAD_Dr_004_114020
intensity
samples in epiboly order
10
12
14
16
18
0
50
100
150

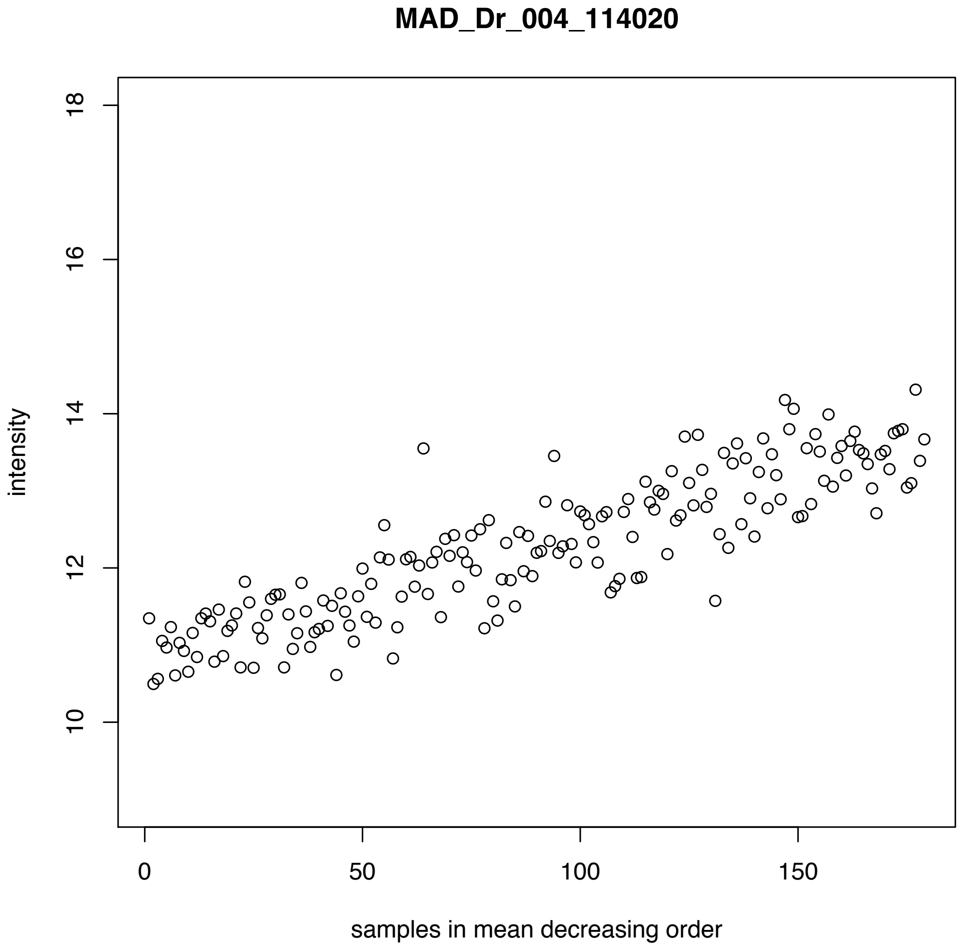
MAD_Dr_004_114020
intensity
samples in mean decreasing order
10
12
14
16
18
0
50
100
150

MAD_Dr_004_179180

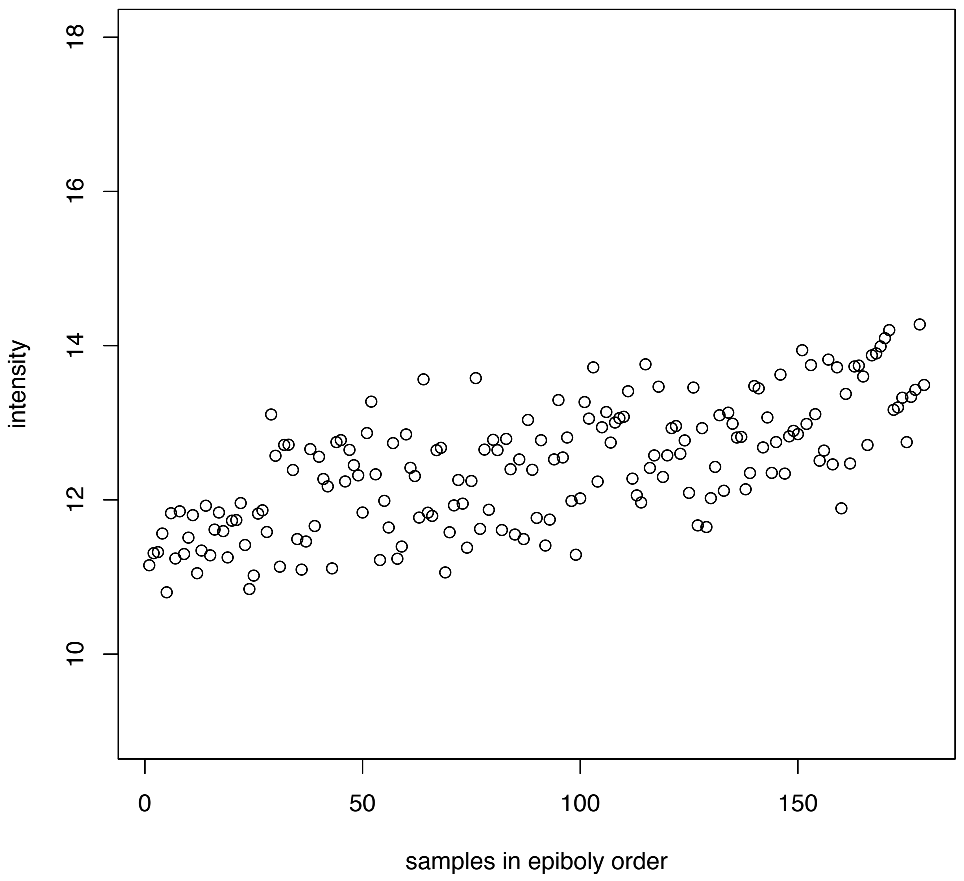

MAD_Dr_004_179180

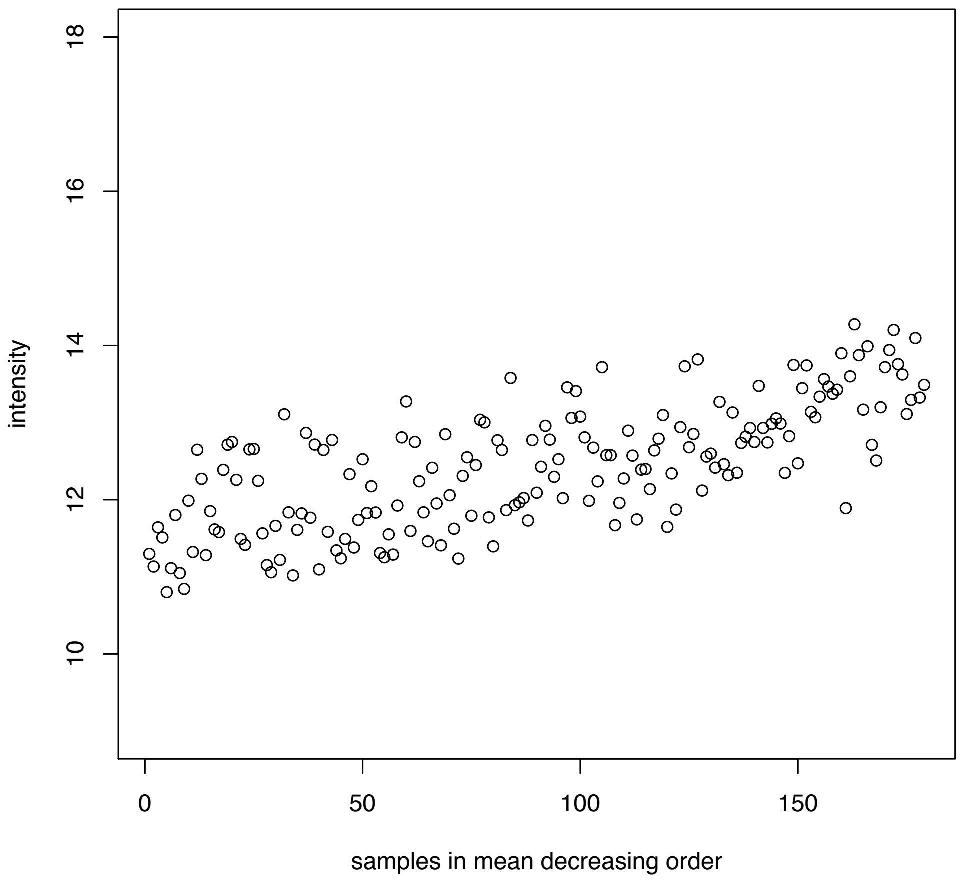

MAD_Dr_004_125005

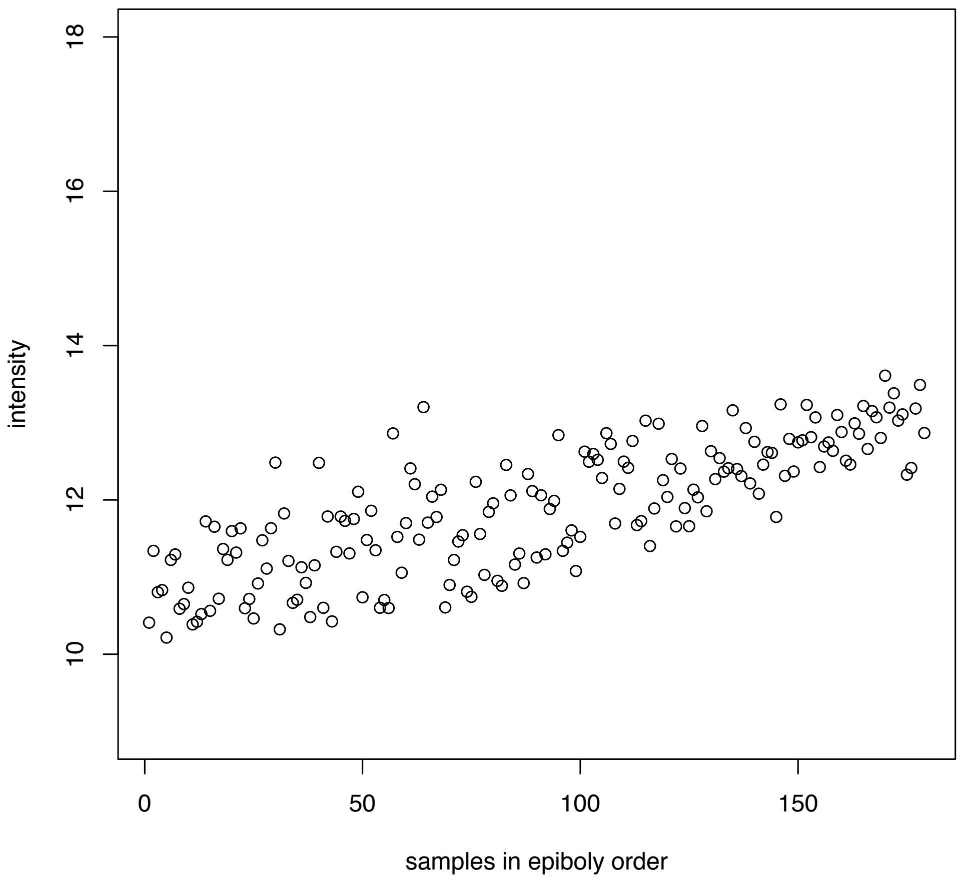

MAD_Dr_004_125005

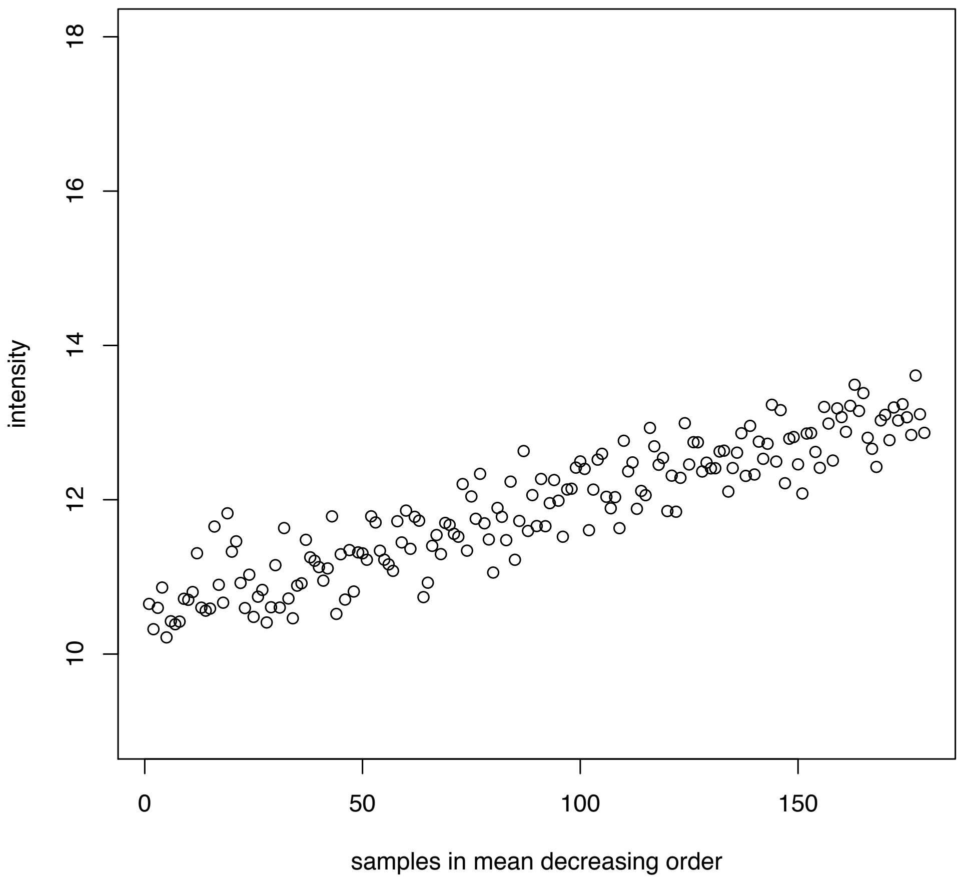

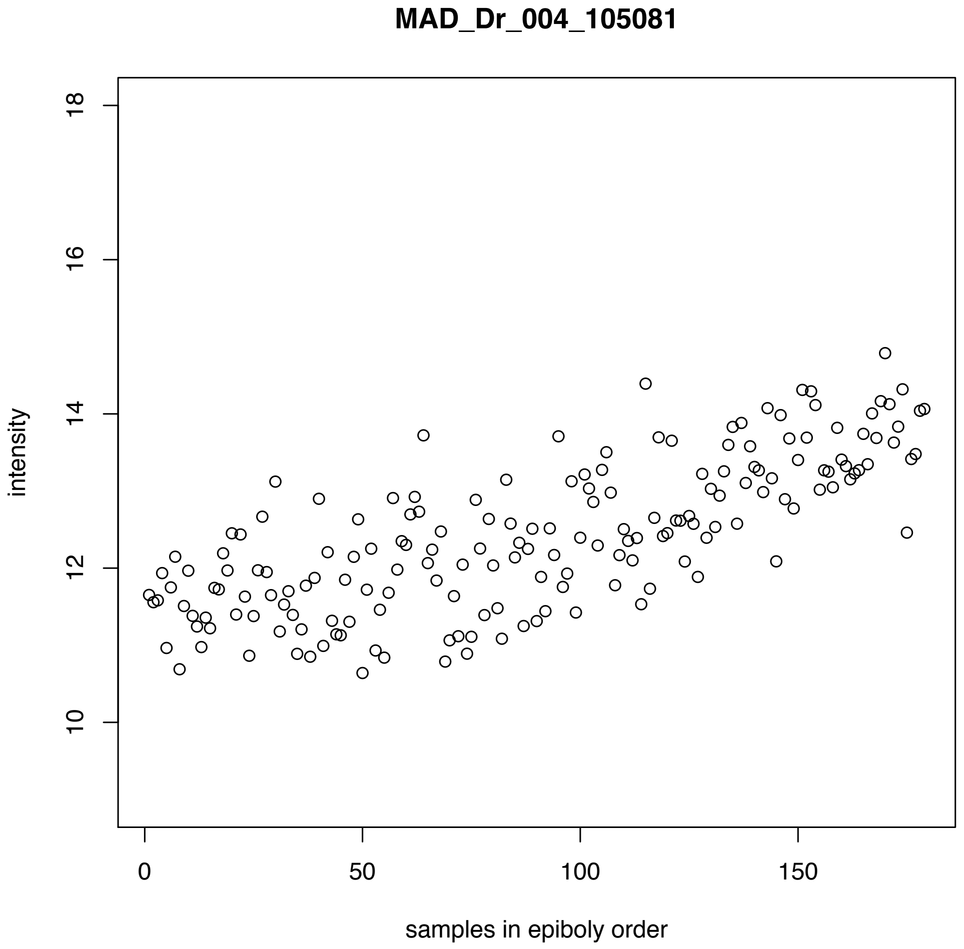
MAD_Dr_004_105081
intensity
10
12
14
16
18
0
50
100
150
samples in epiboly order

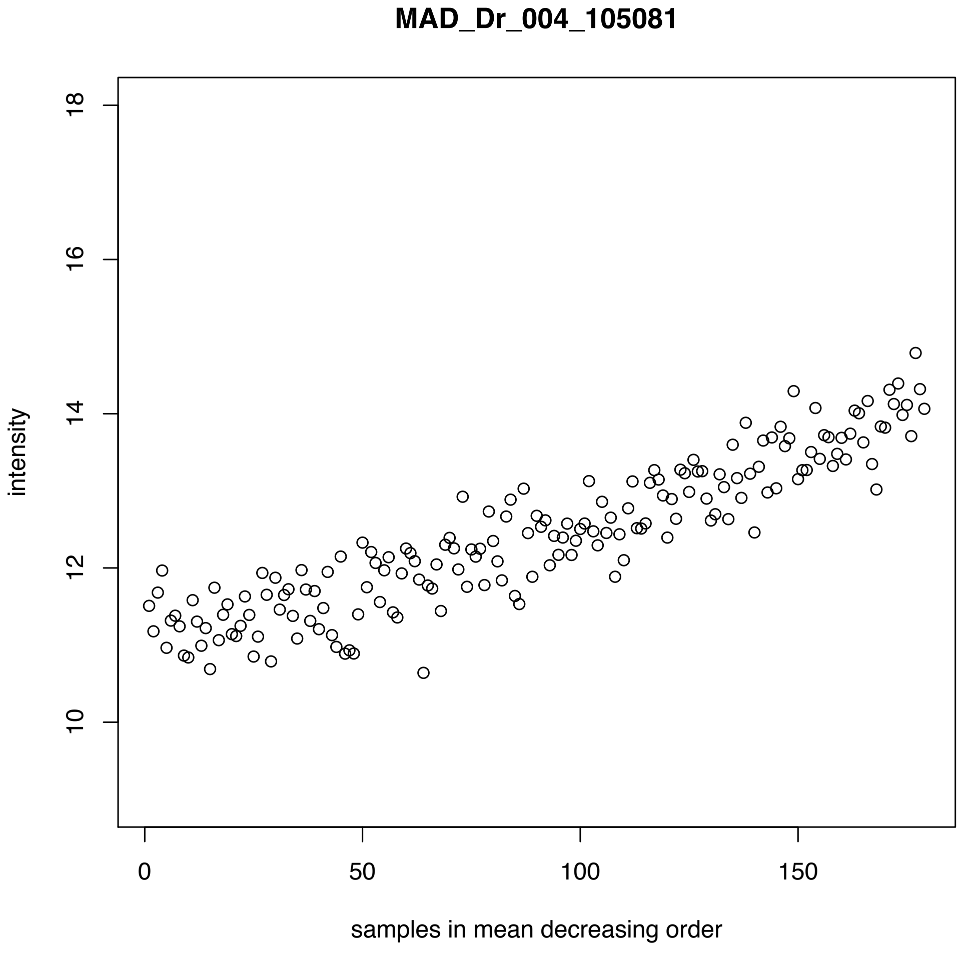
MAD_Dr_004_105081
intensity
10
12
14
16
18
0
50
100
150
samples in mean decreasing order

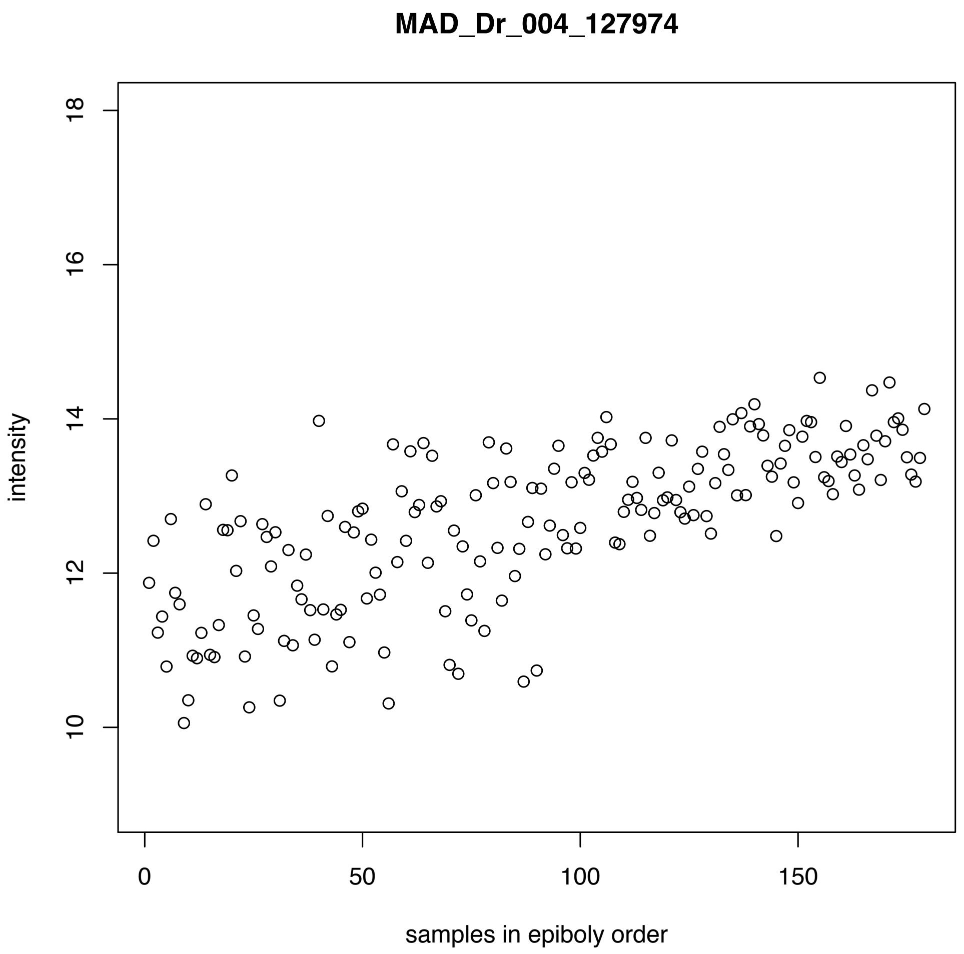
MAD_Dr_004_127974
intensity
10
12
14
16
18
0
50
100
150
samples in epiboly order

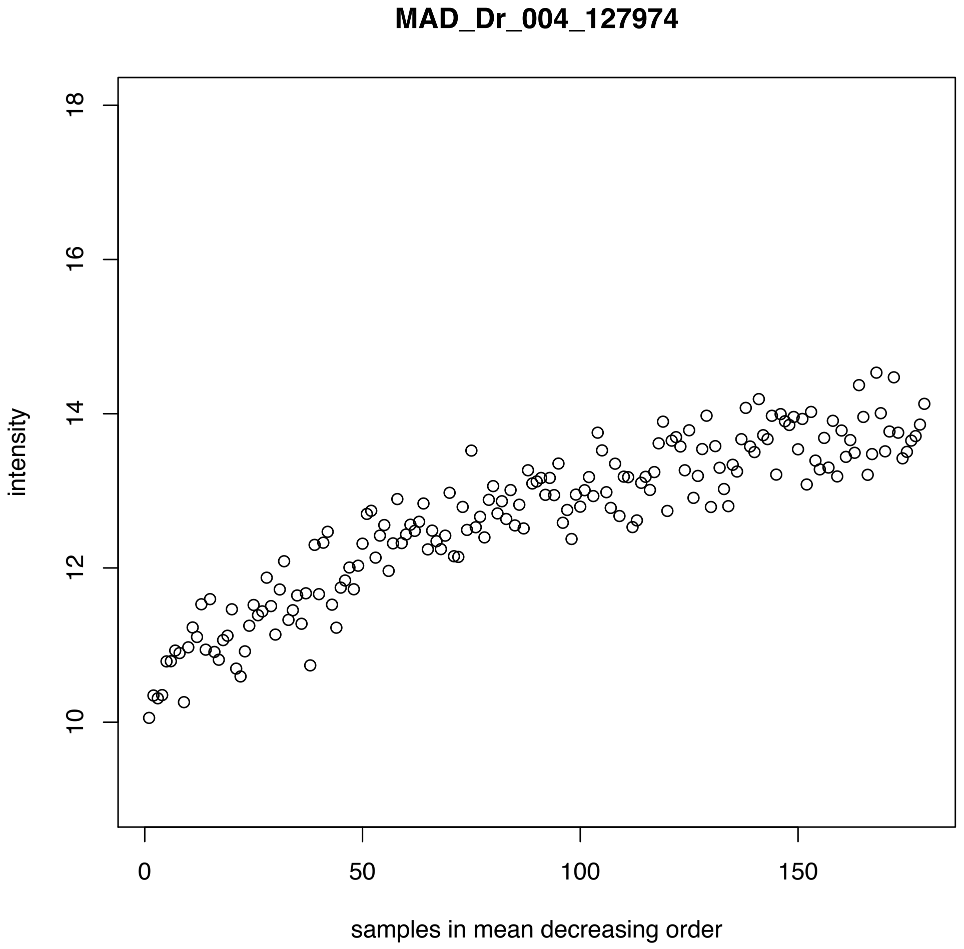
MAD_Dr_004_127974
intensity
10
12
14
16
18
0
50
100
150
samples in mean decreasing order

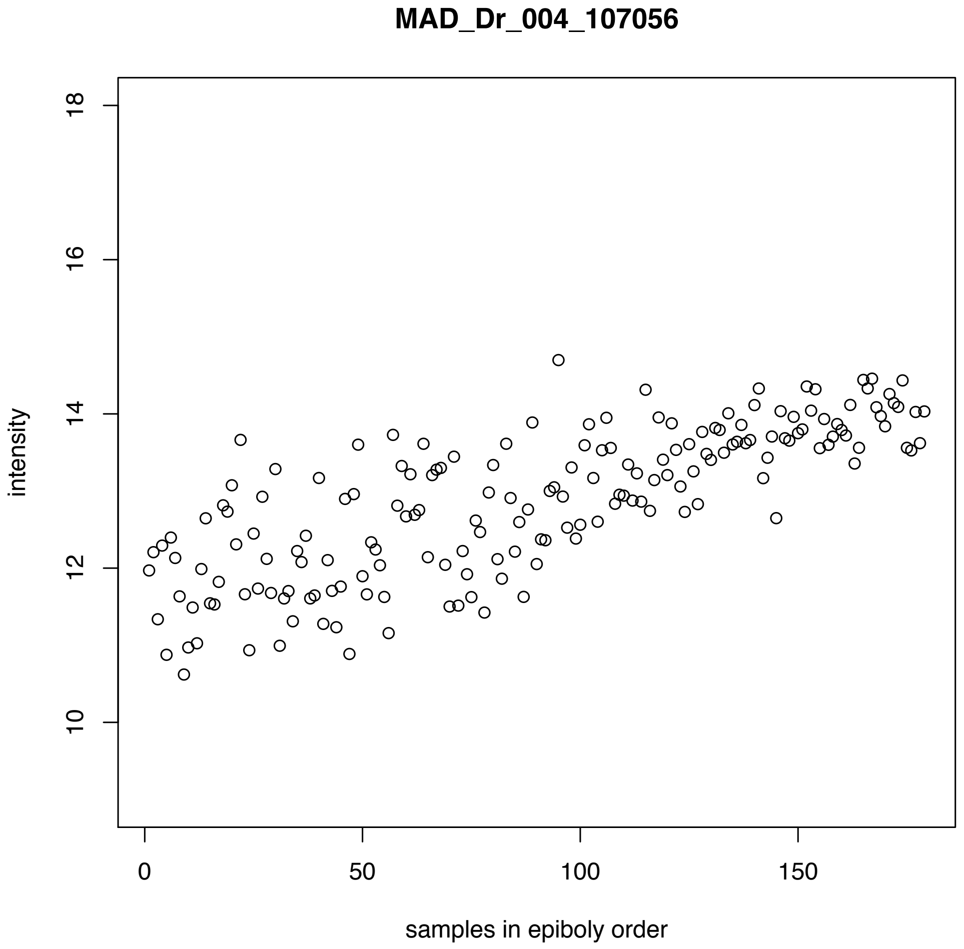

MAD_Dr_004_107056
intensity
10
12
14
16
18
0
50
100
150
samples in epiboly order

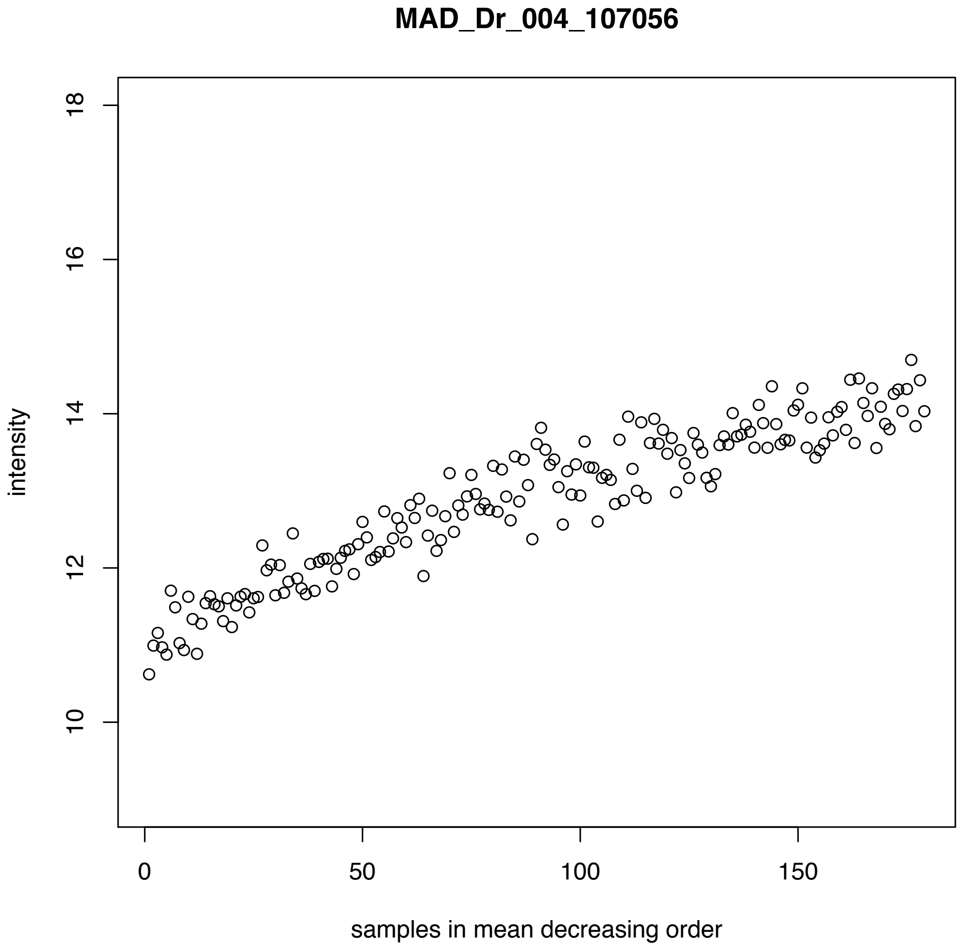

MAD_Dr_004_107056
intensity
10
12
14
16
18
0
50
100
150
samples in mean decreasing order

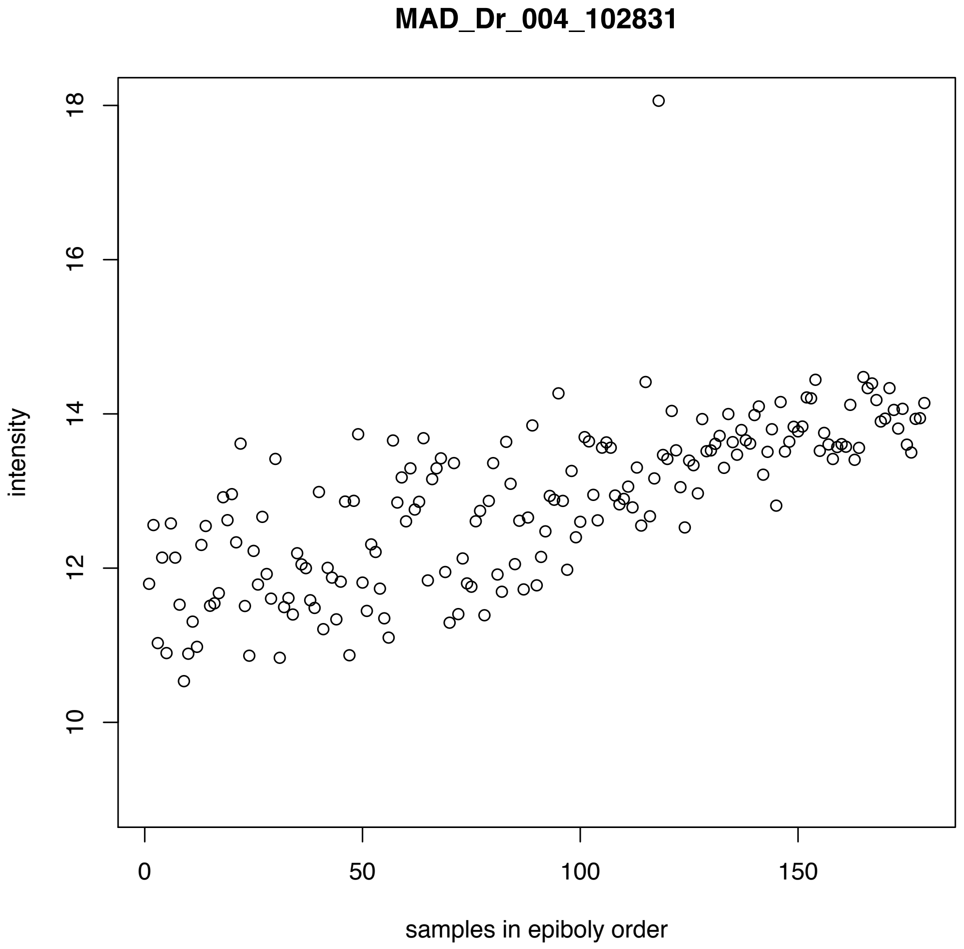
MAD_Dr_004_102831
intensity
10
12
14
16
18
0
50
100
150
samples in epiboly order

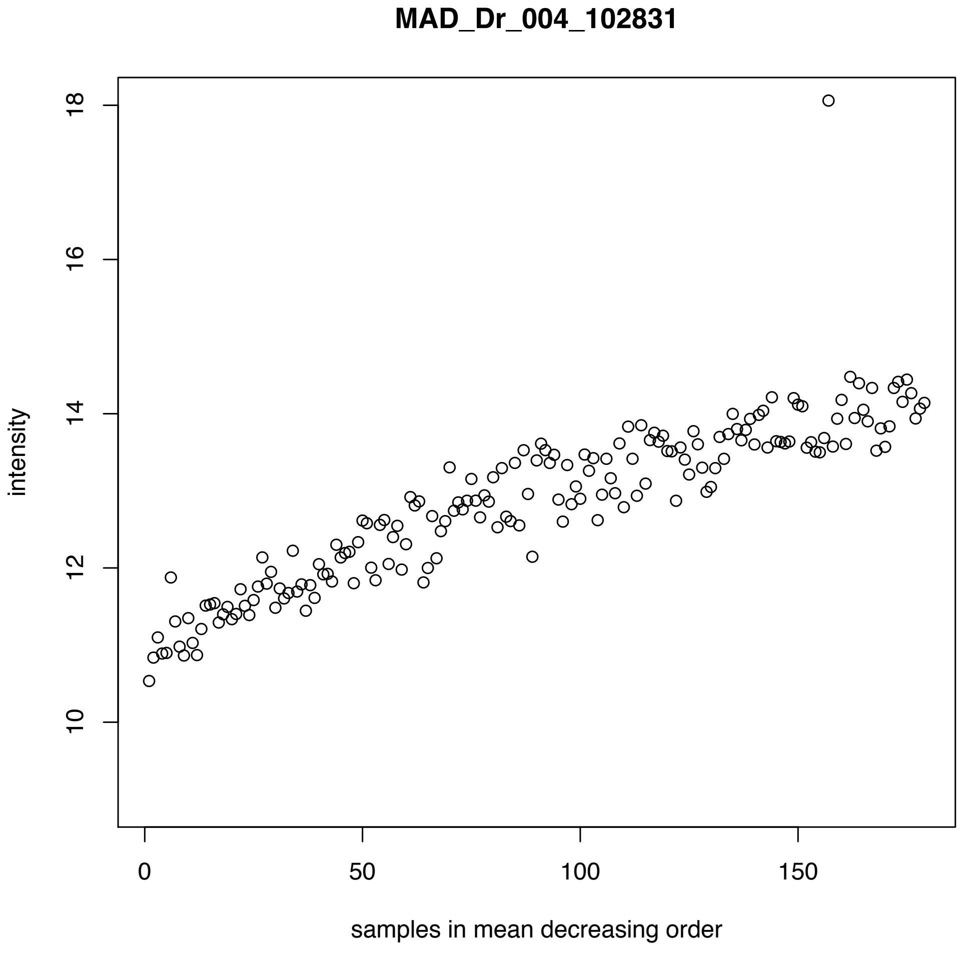
MAD_Dr_004_102831
intensity
10
12
14
16
18
0
50
100
150
samples in mean decreasing order

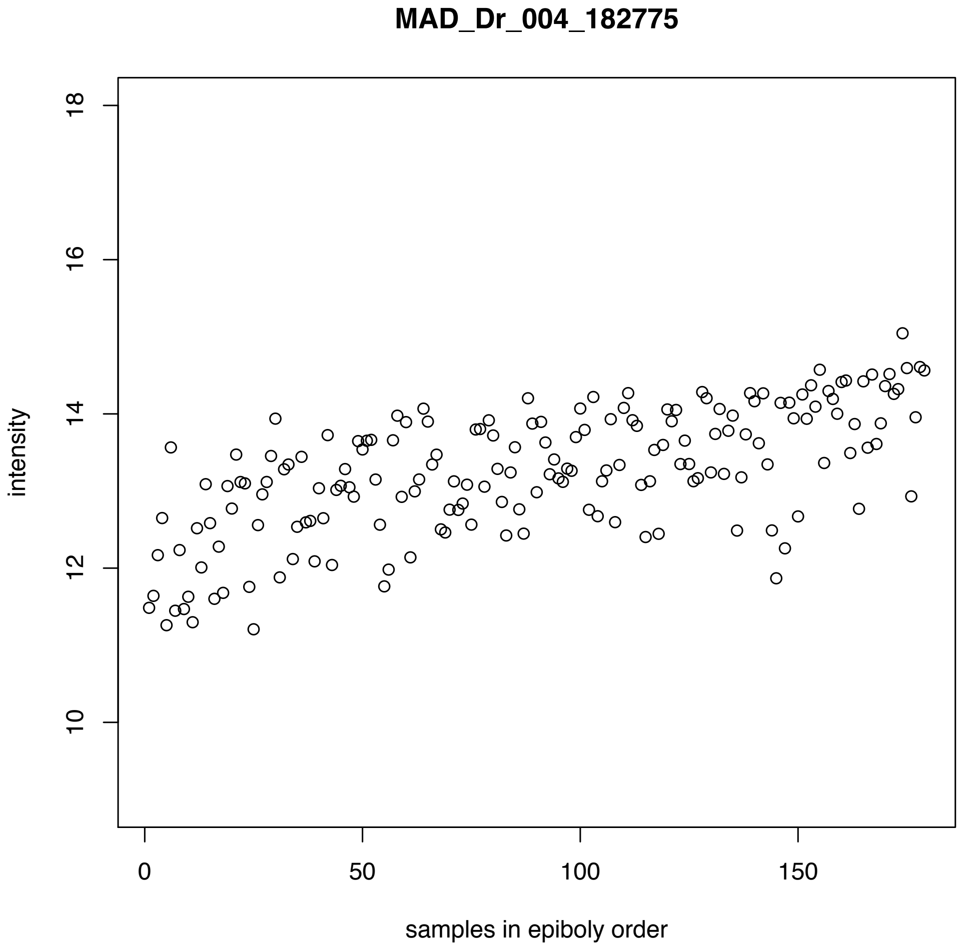
MAD_Dr_004_182775
intensity
samples in epiboly order
10
12
14
16
18
0
50
100
150

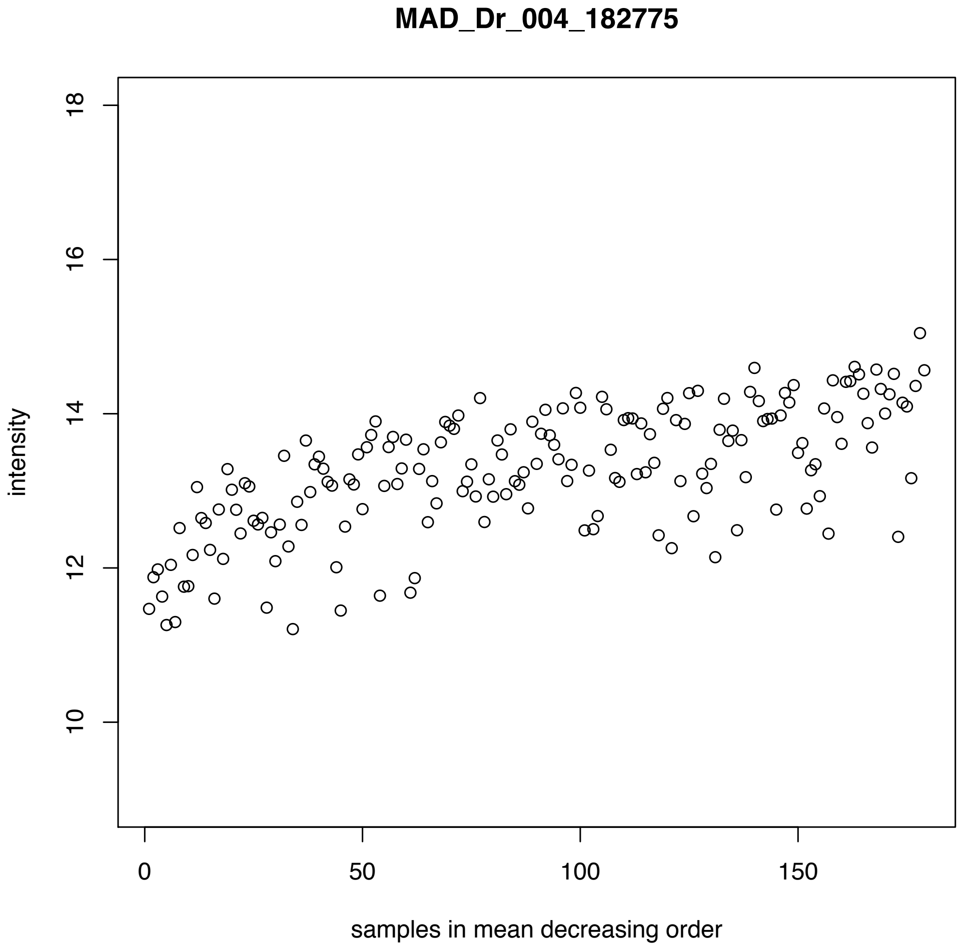
MAD_Dr_004_182775
intensity
samples in mean decreasing order
10
12
14
16
18
0
50
100
150

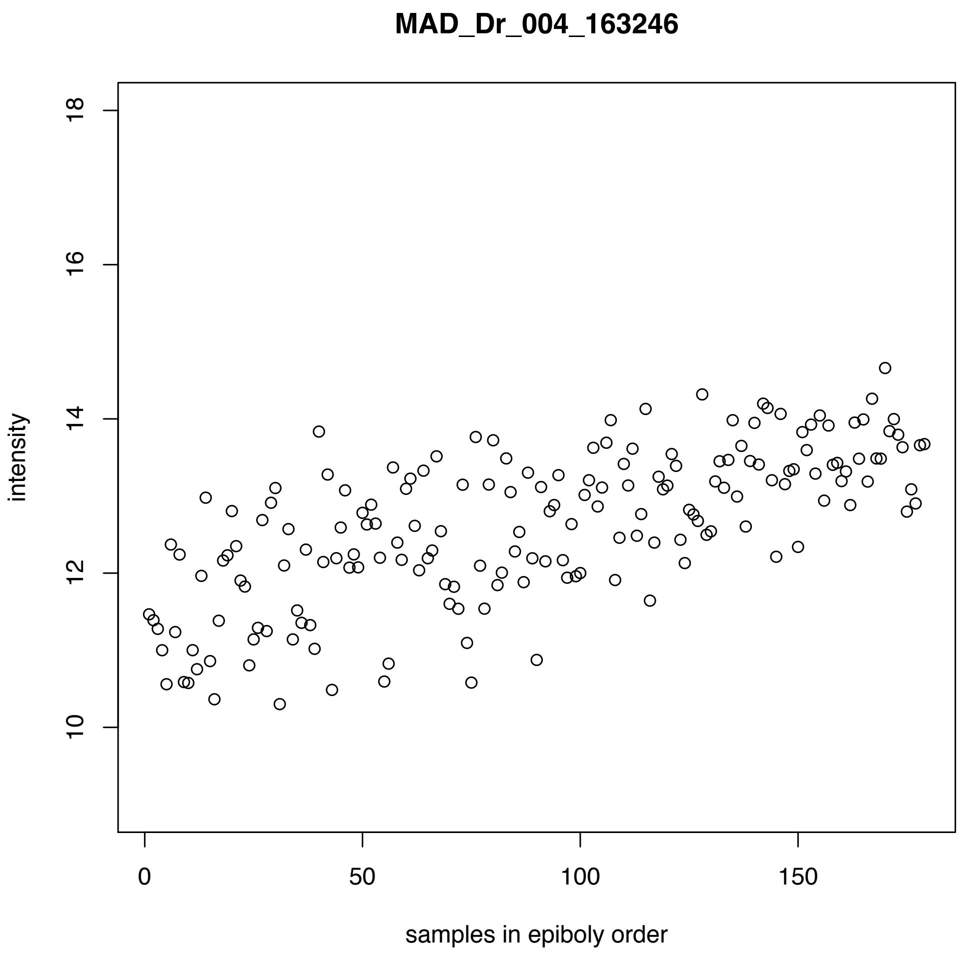
MAD_Dr_004_163246
intensity
samples in epiboly order
10
12
14
16
18
0
50
100
150

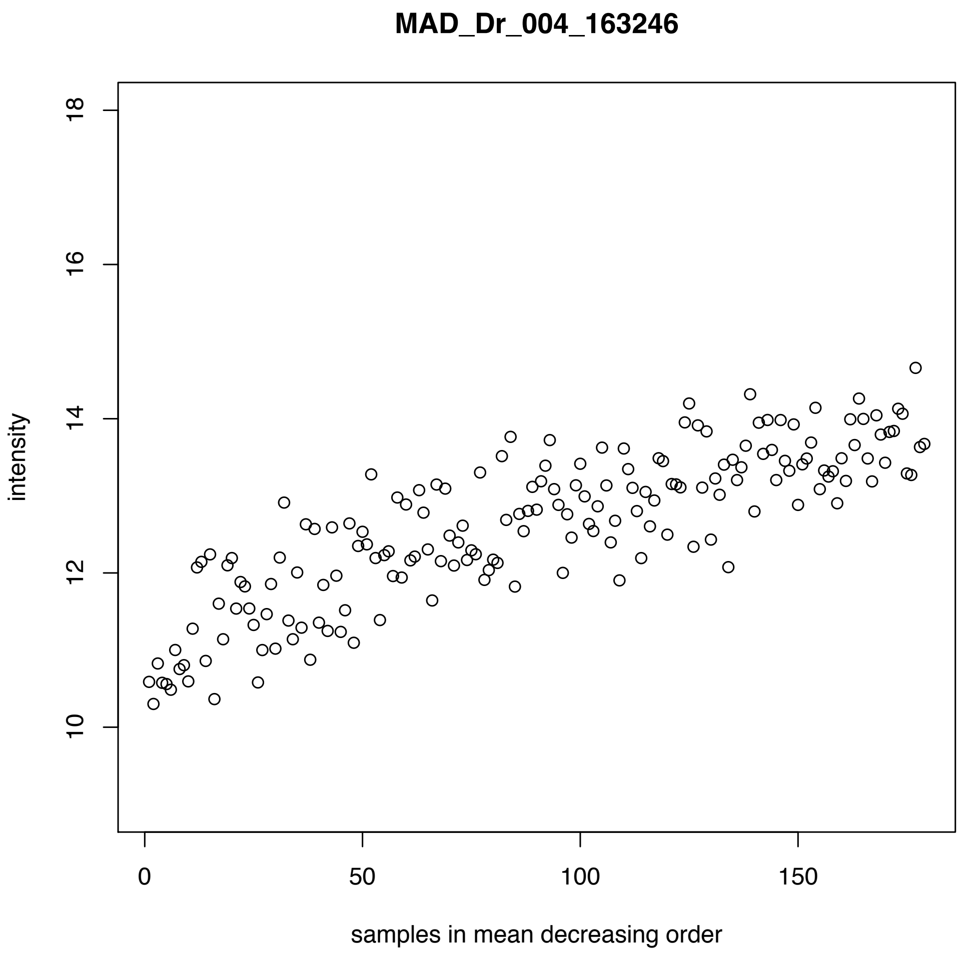
MAD_Dr_004_163246
intensity
samples in mean decreasing order
10
12
14
16
18
0
50
100
150

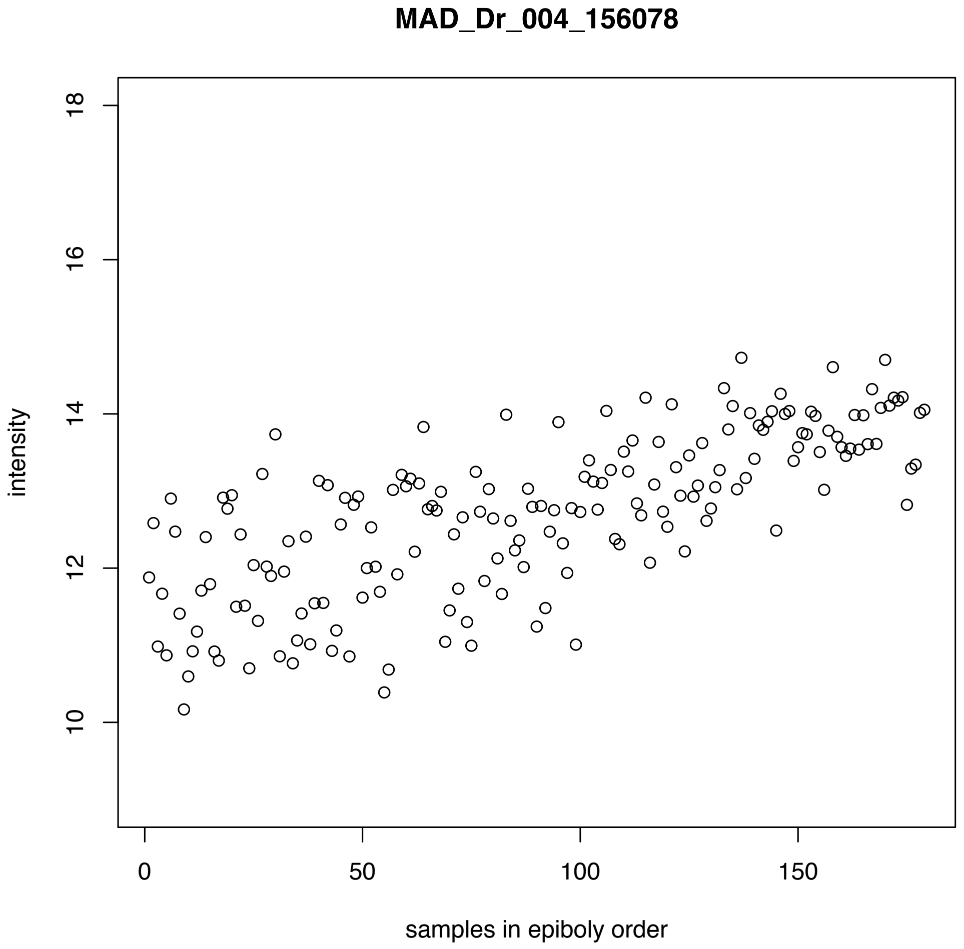
MAD_Dr_004_156078
intensity
10
12
14
16
18
0
50
100
150
samples in epiboly order

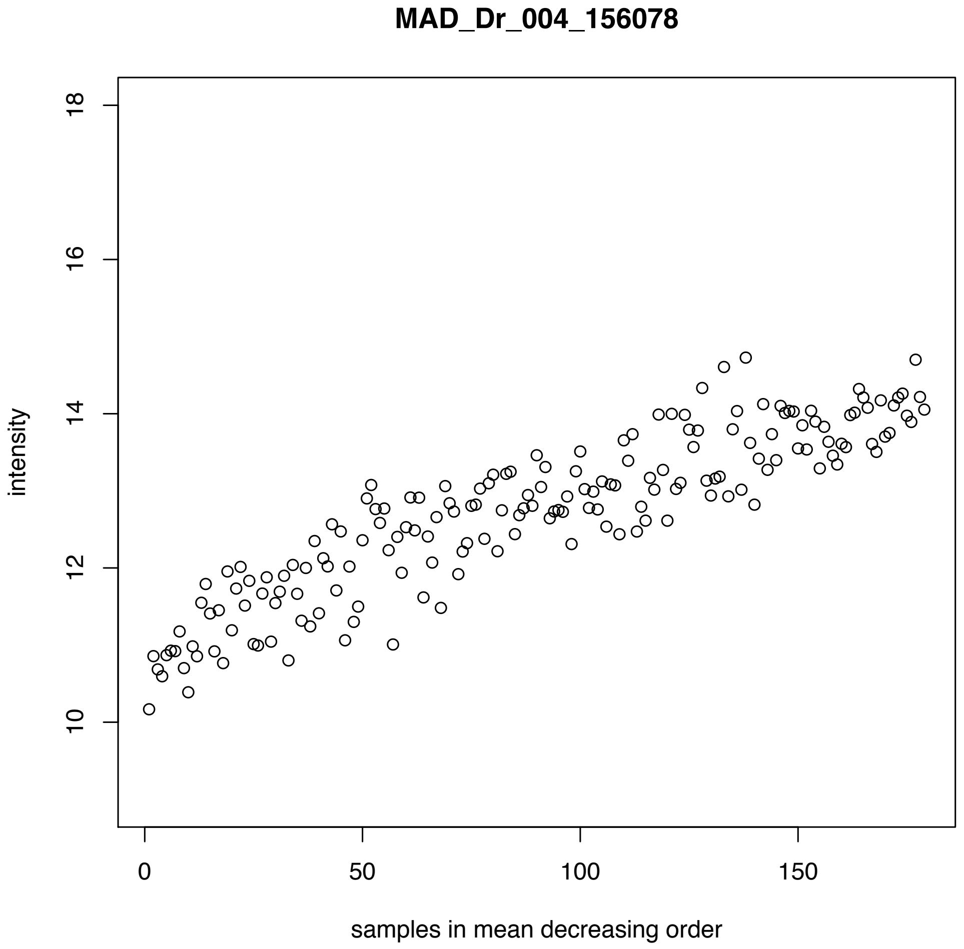
MAD_Dr_004_156078
intensity
10
12
14
16
18
0
50
100
150
samples in mean decreasing order

MAD_Dr_004_164192

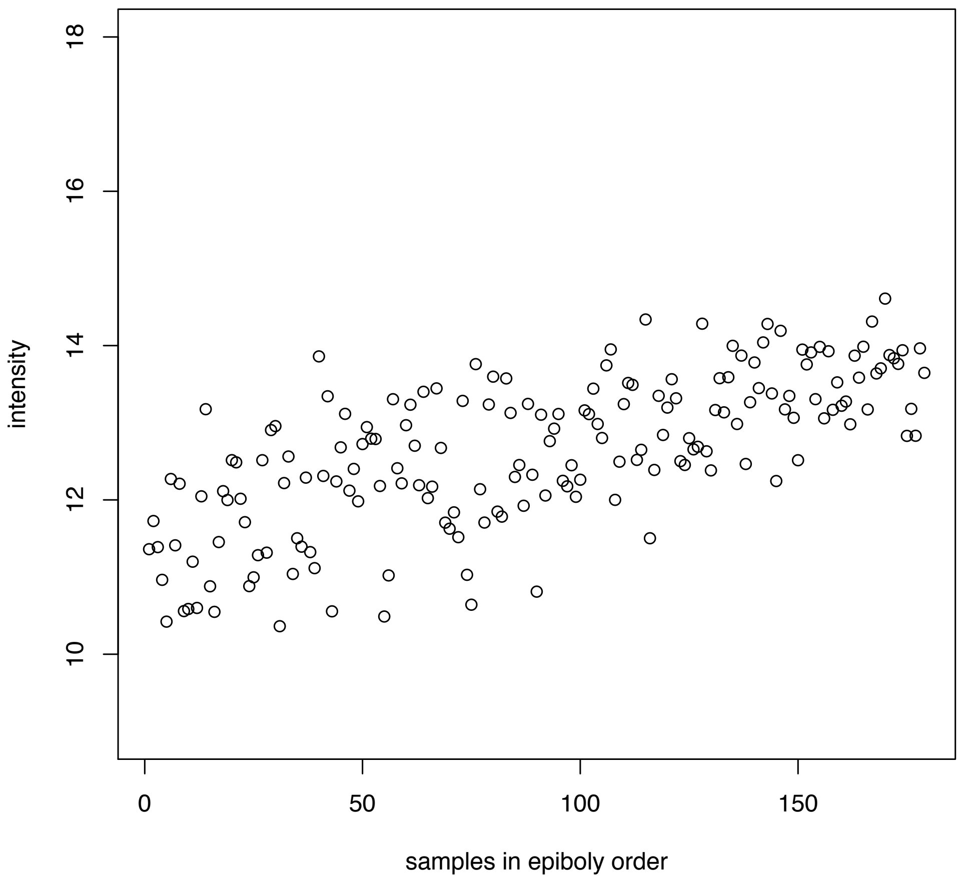

MAD_Dr_004_164192

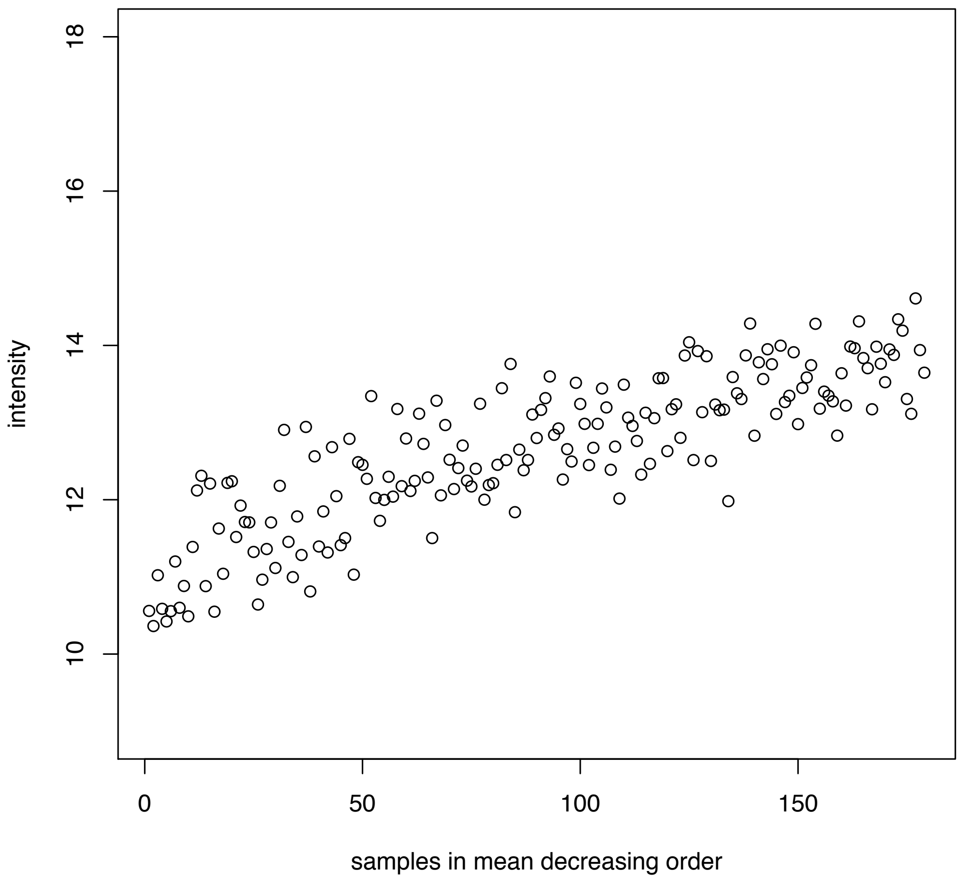

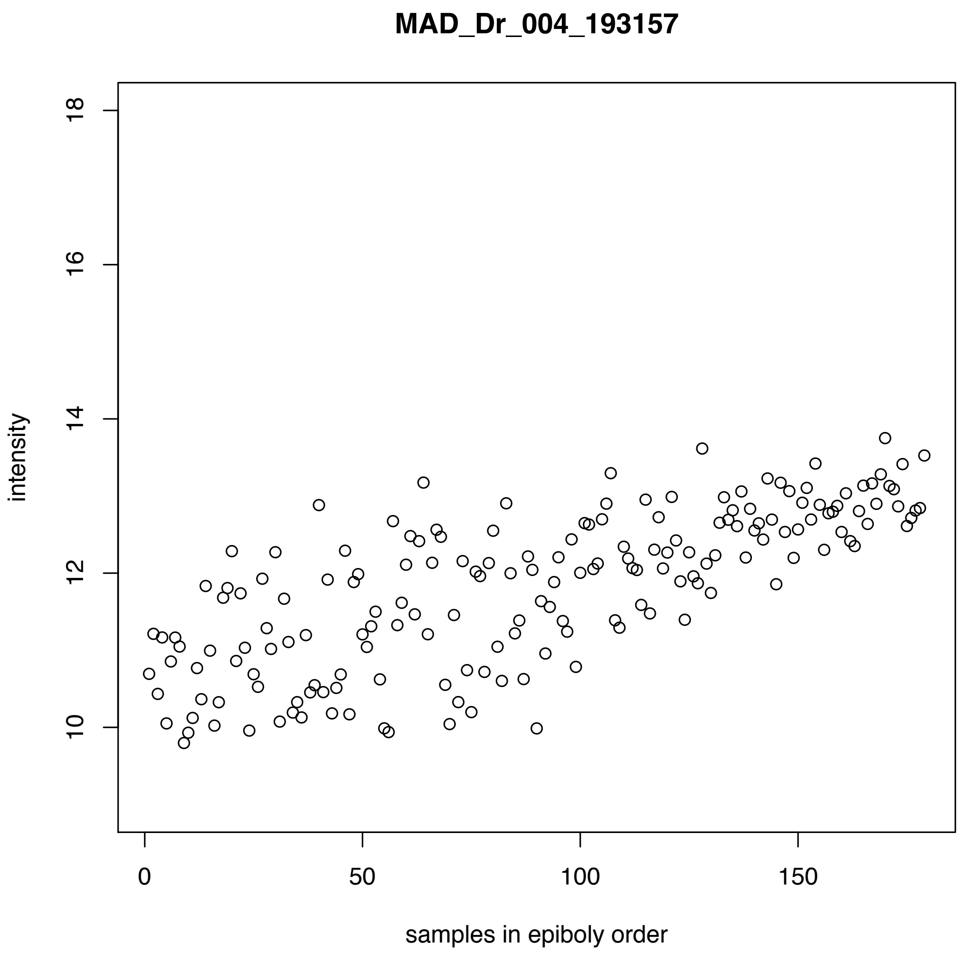
MAD_Dr_004_193157
intensity
10
12
14
16
18
0
50
100
150
samples in epiboly order

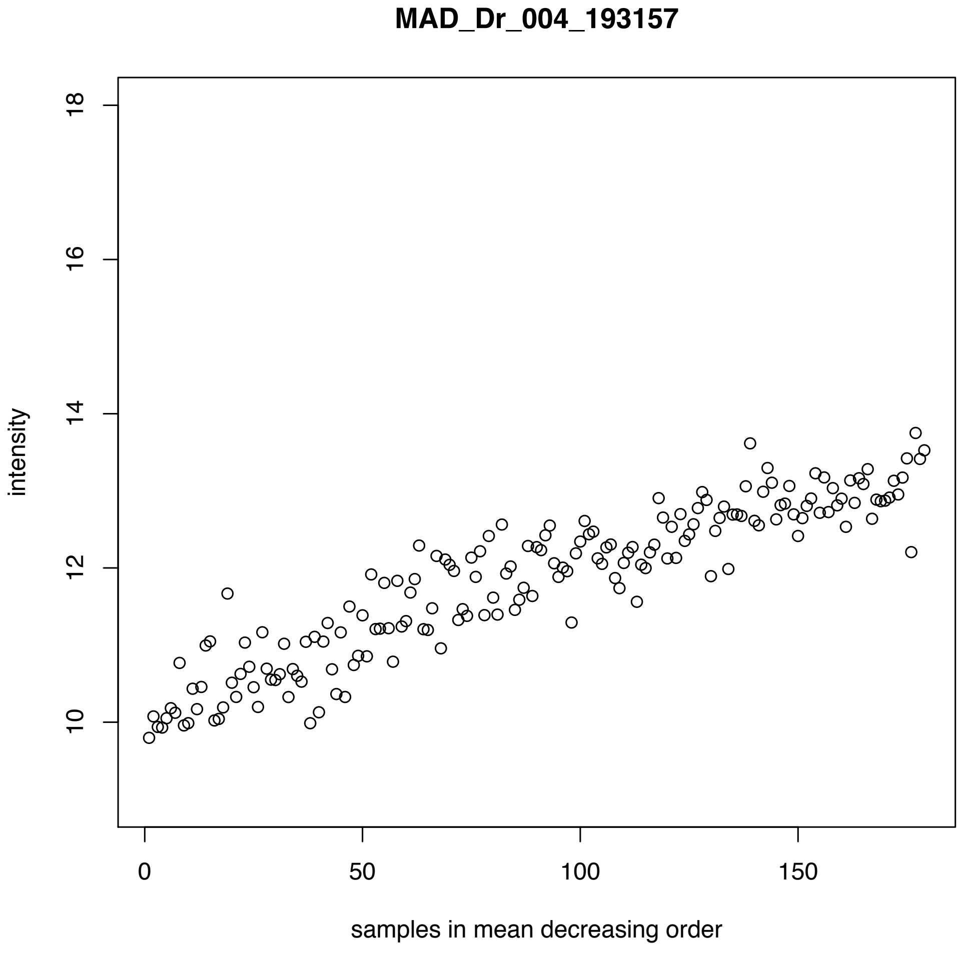
MAD_Dr_004_193157
intensity
10
12
14
16
18
0
50
100
150
samples in mean decreasing order

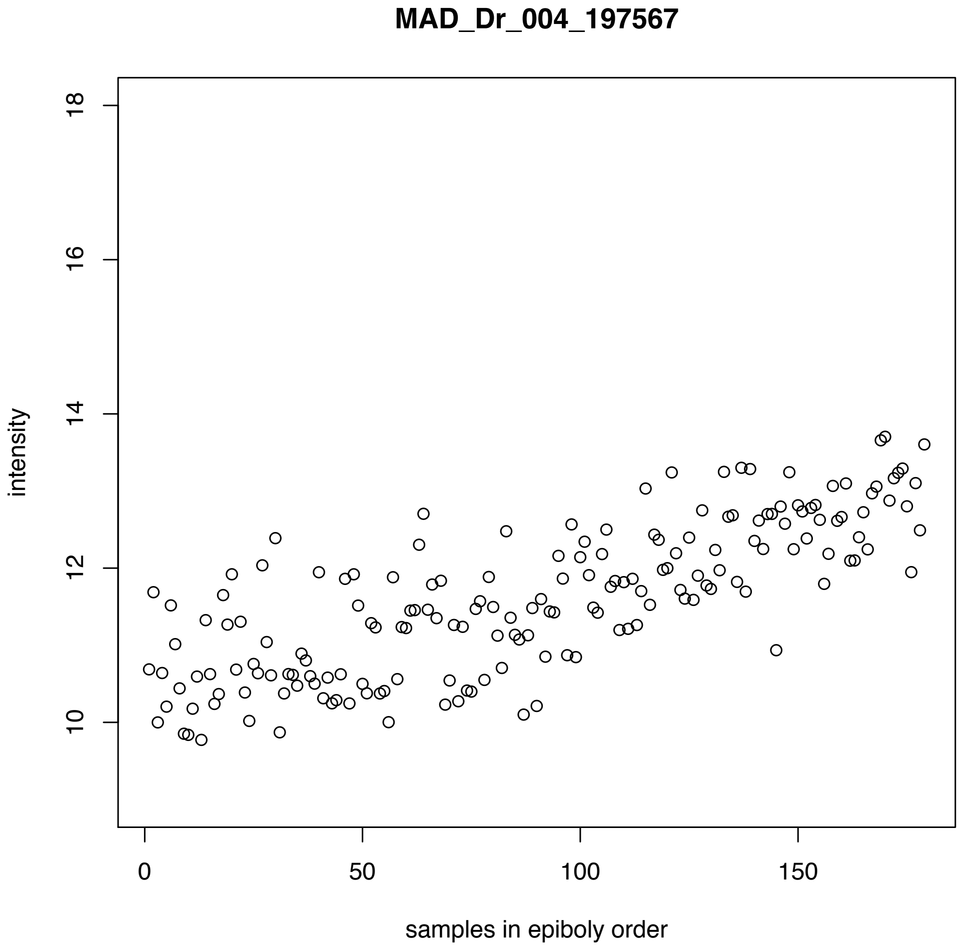
MAD_Dr_004_197567
intensity
samples in epiboly order
0
50
100
150
10
12
14
16
18

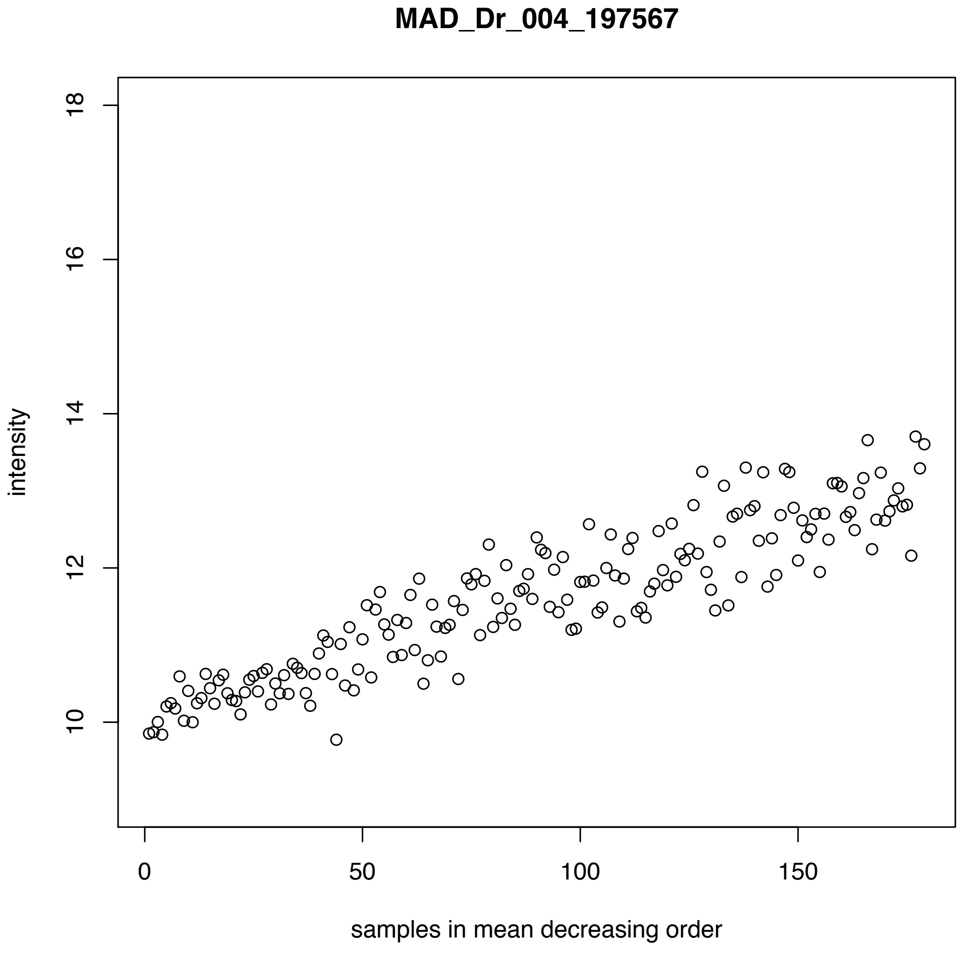
MAD_Dr_004_197567
intensity
samples in mean decreasing order
0
50
100
150
10
12
14
16
18

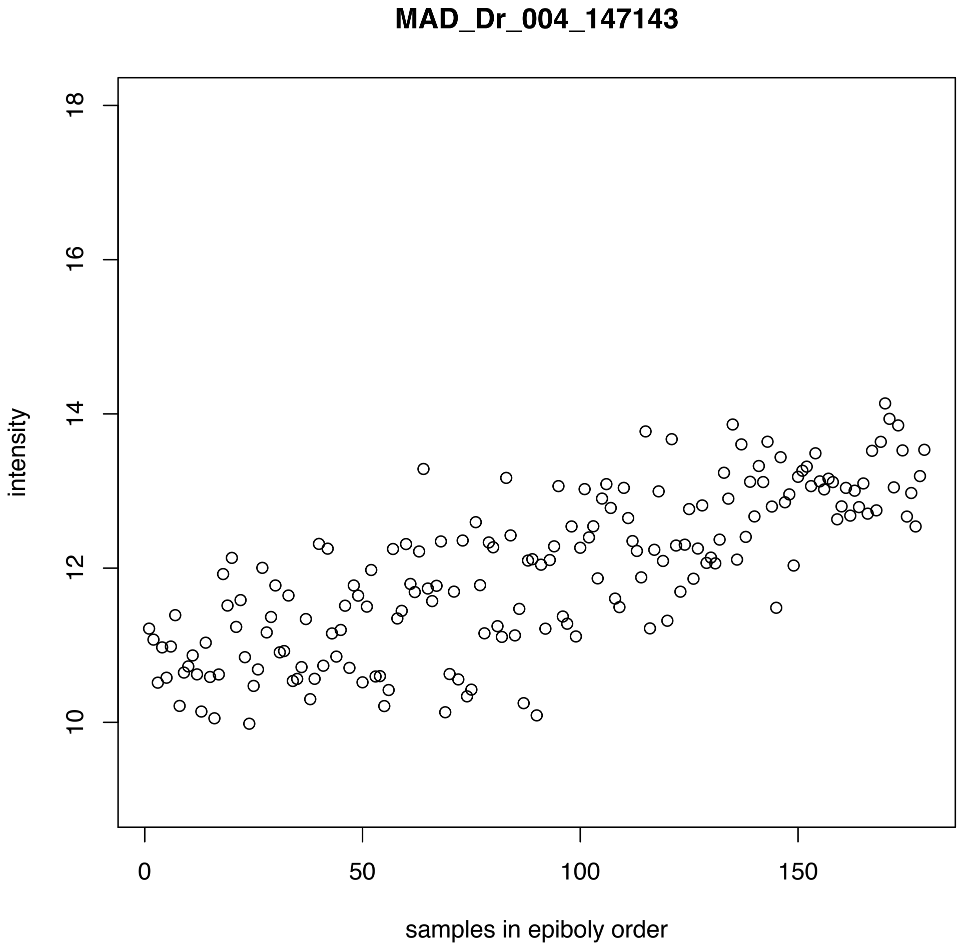
MAD_Dr_004_147143
intensity
10
12
14
16
18
0
50
100
150
samples in epiboly order

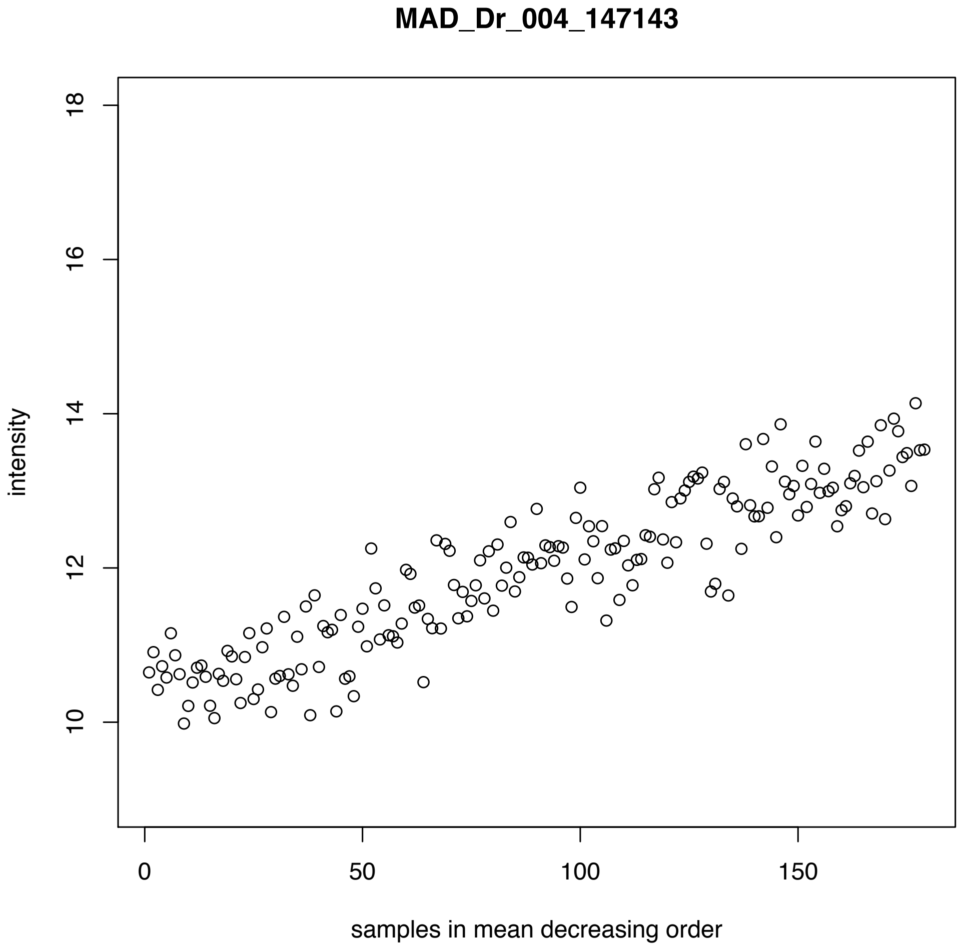
MAD_Dr_004_147143
intensity
10
12
14
16
18
0
50
100
150
samples in mean decreasing order

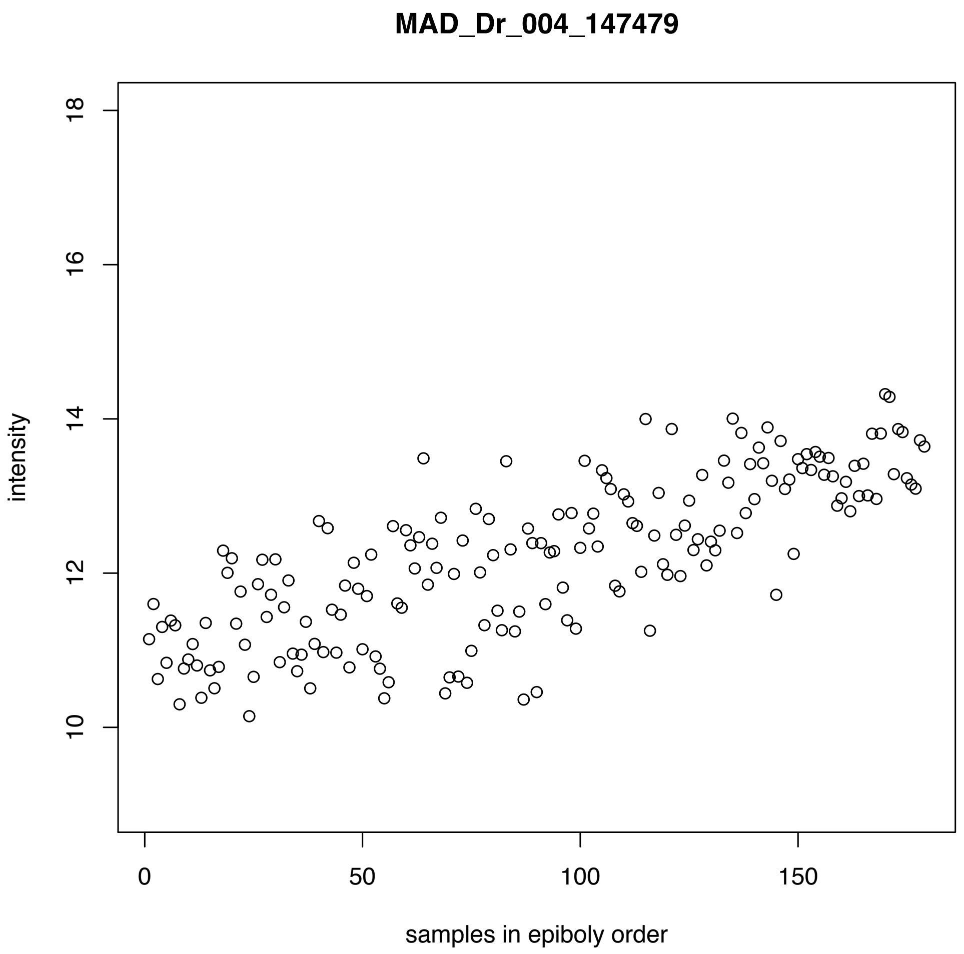
MAD_Dr_004_147479
intensity
10
12
14
16
18
0
50
100
150
samples in epiboly order

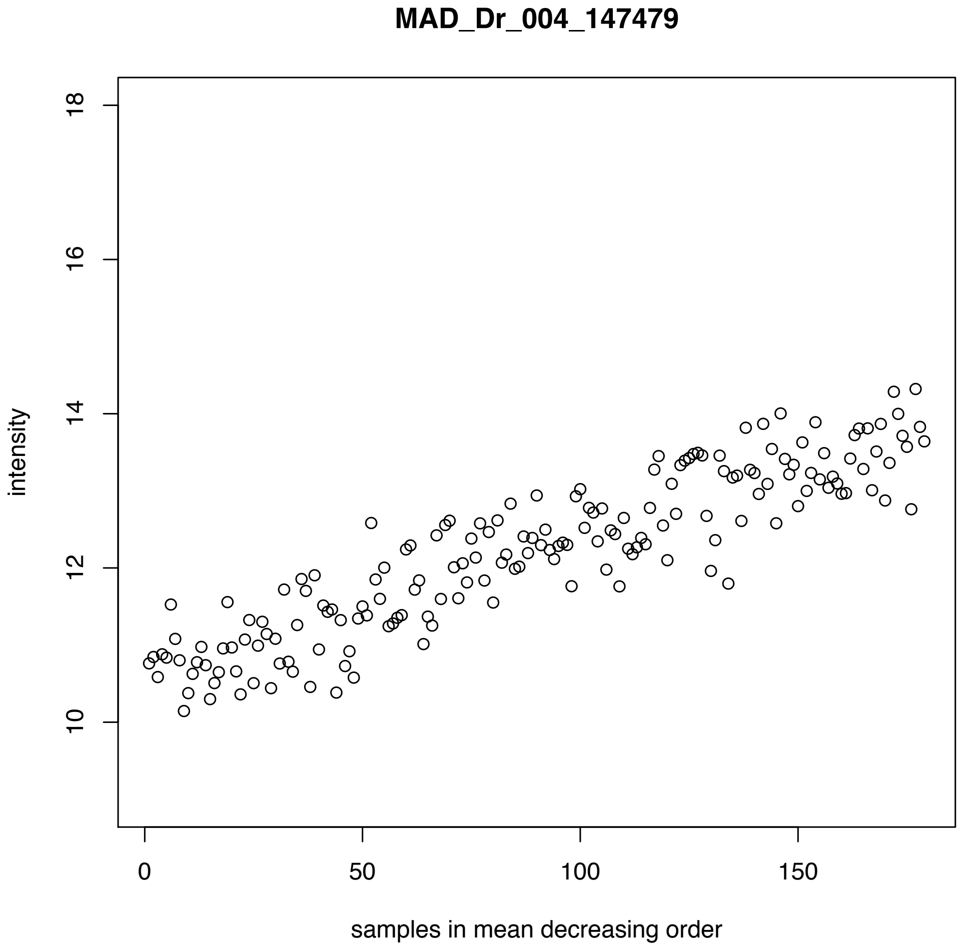
MAD_Dr_004_147479
intensity
10
12
14
16
18
0
50
100
150
samples in mean decreasing order

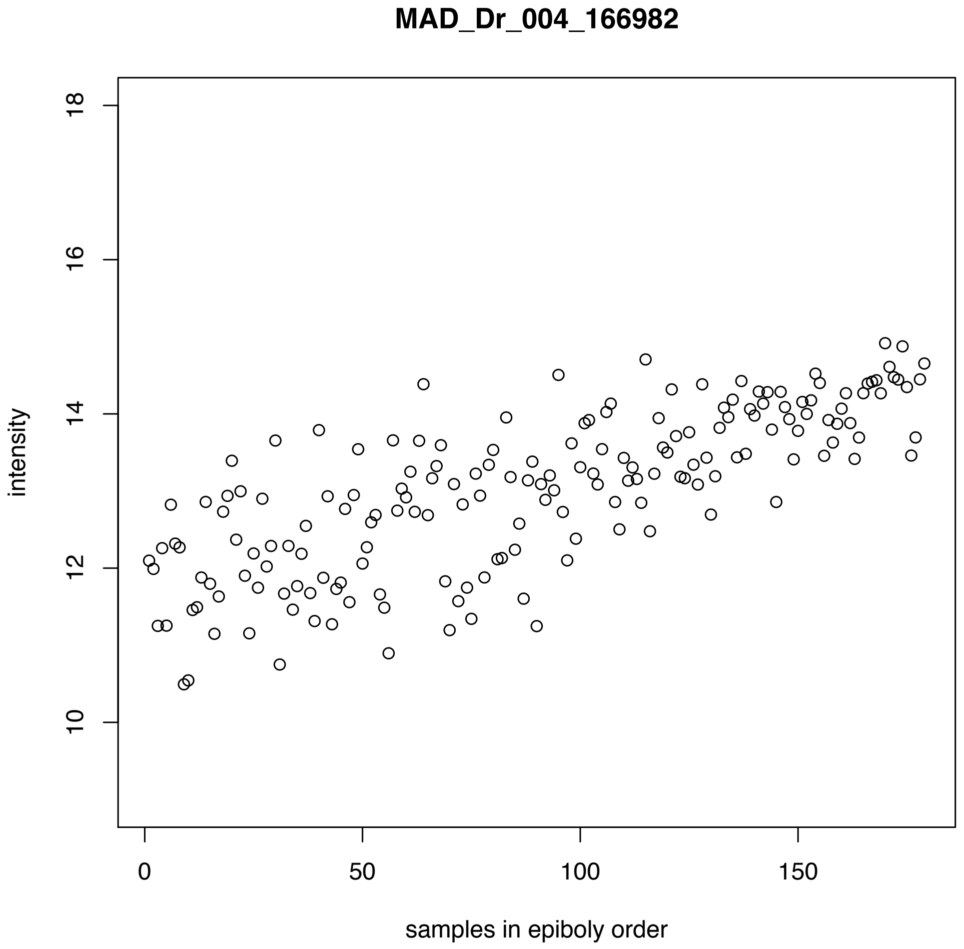
MAD_Dr_004_166982
intensity
samples in epiboly order
10
12
14
16
18
0
50
100
150

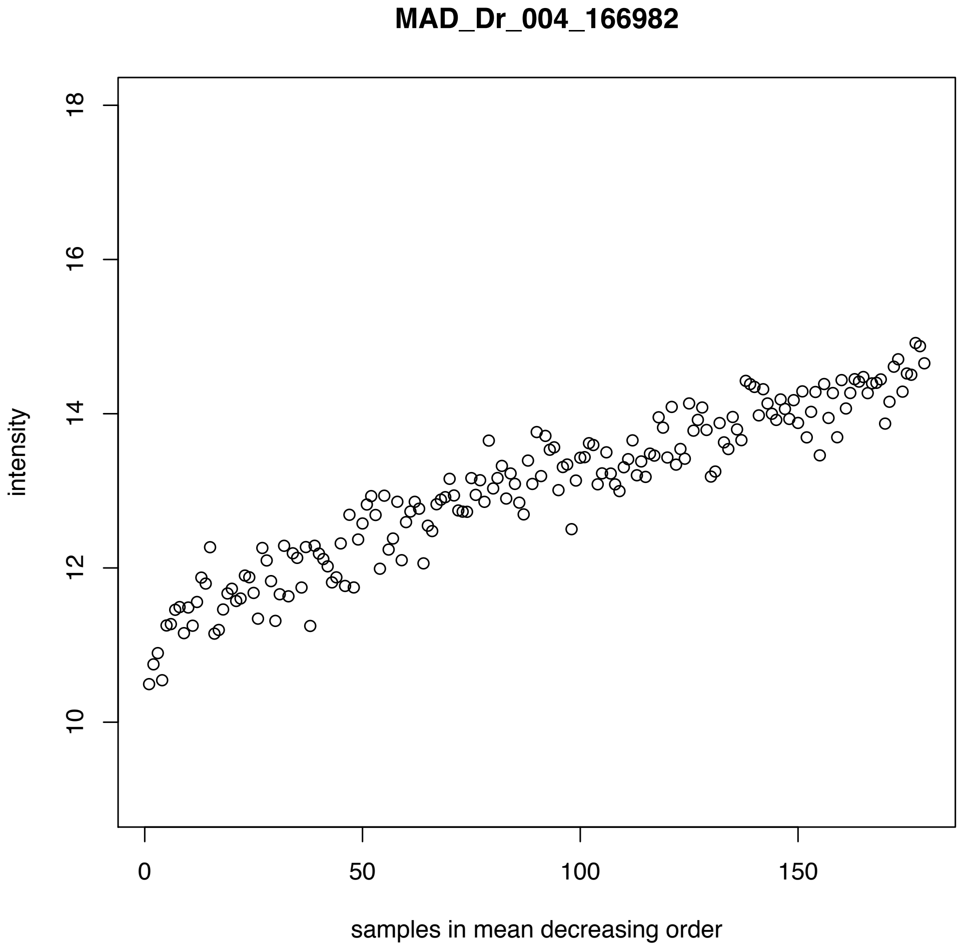
MAD_Dr_004_166982
intensity
samples in mean decreasing order
10
12
14
16
18
0
50
100
150

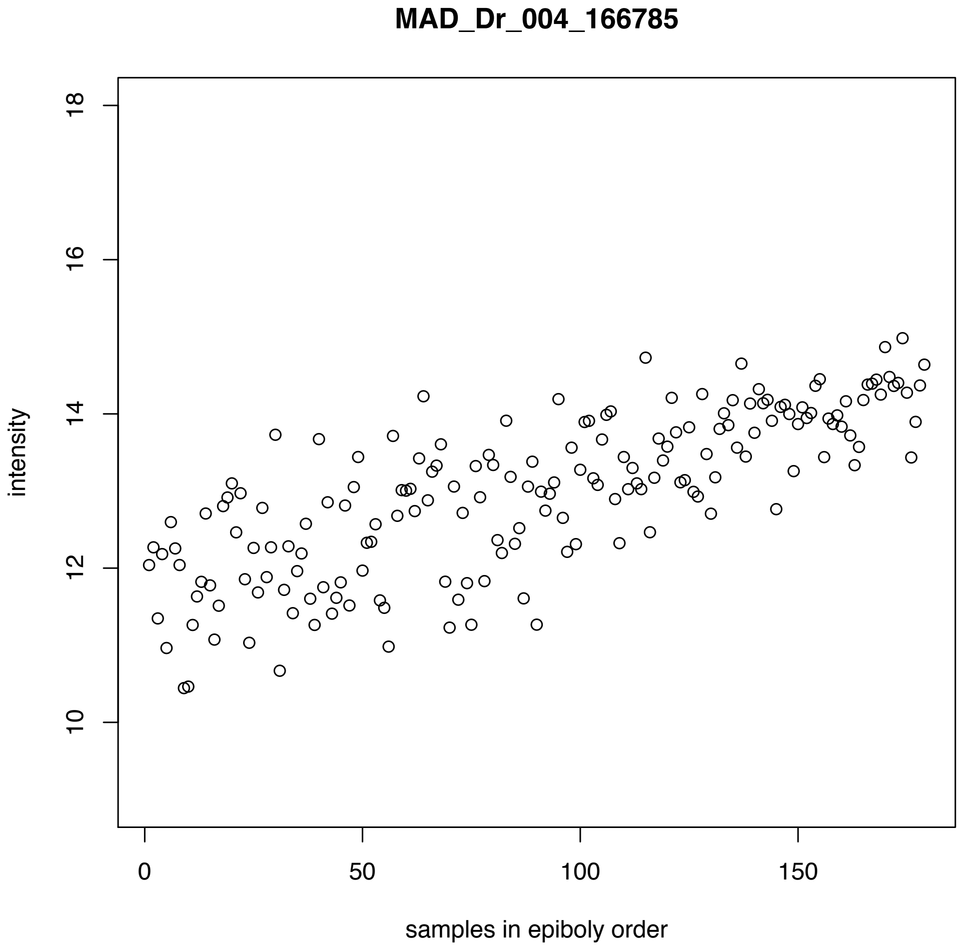
MAD_Dr_004_166785
intensity
10
12
14
16
18
0
50
100
150
samples in epiboly order

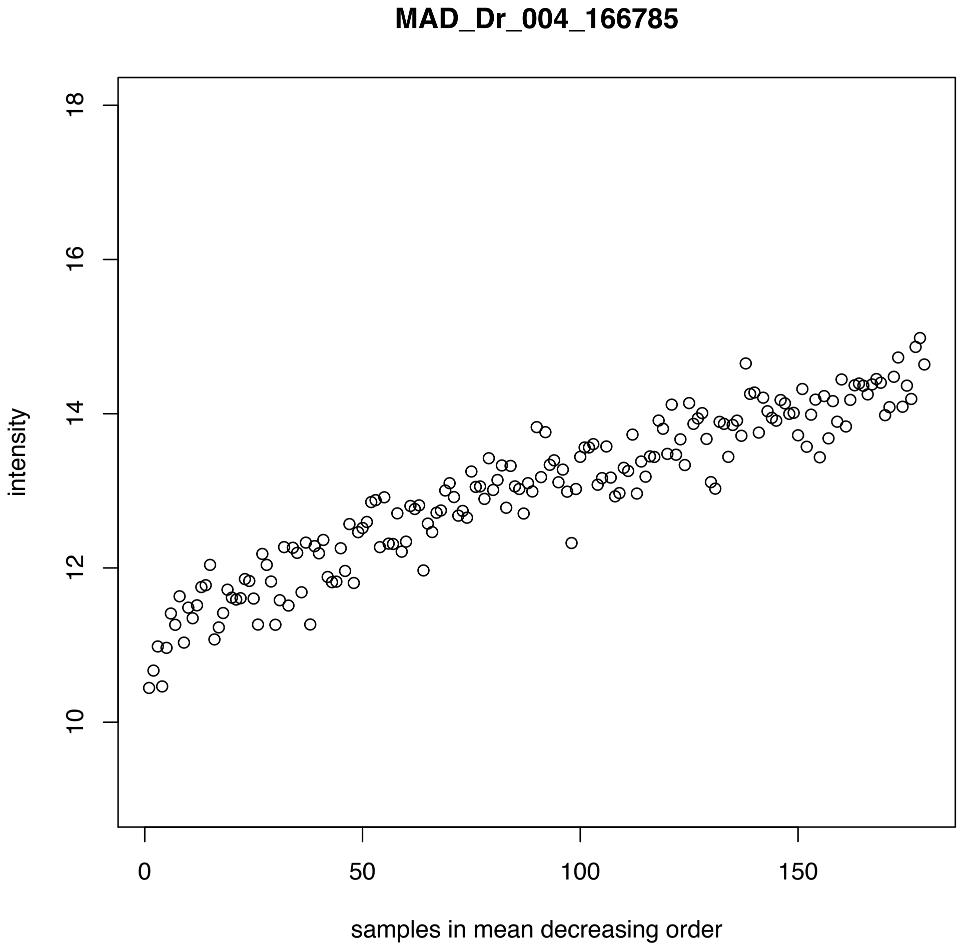
MAD_Dr_004_166785
intensity
10
12
14
16
18
0
50
100
150
samples in mean decreasing order

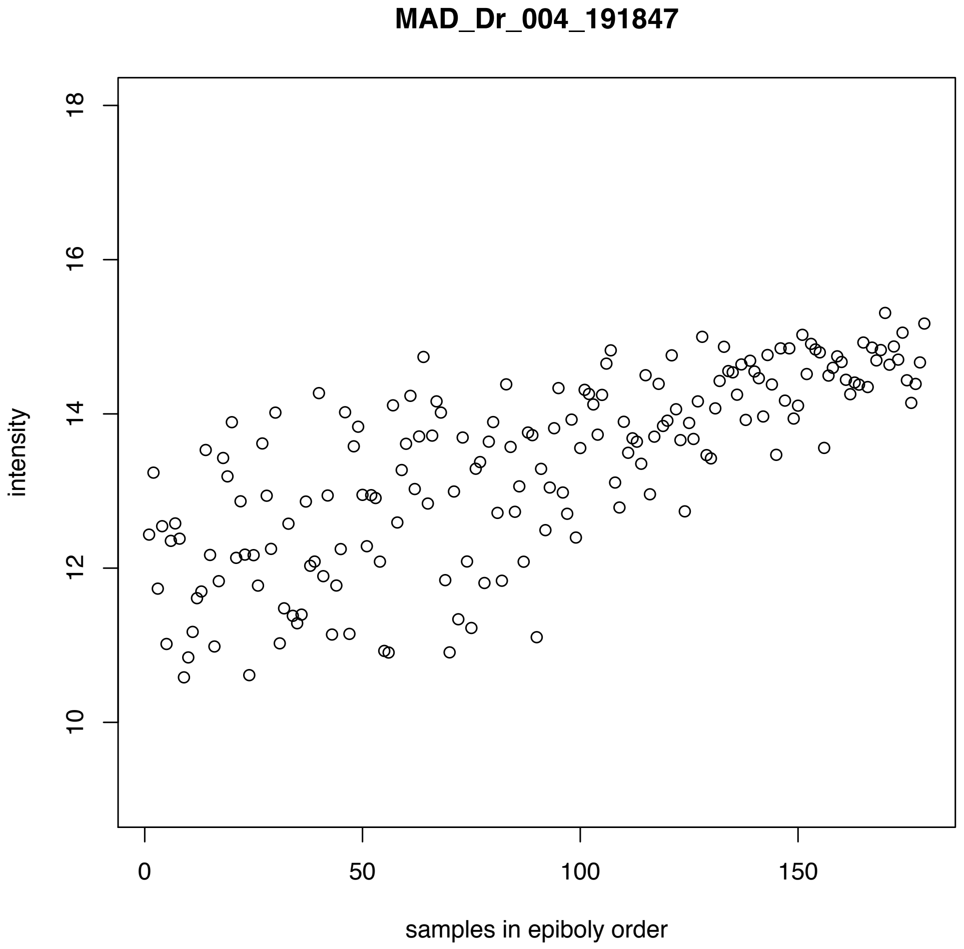
MAD_Dr_004_191847
intensity
samples in epiboly order
10
12
14
16
18
0
50
100
150

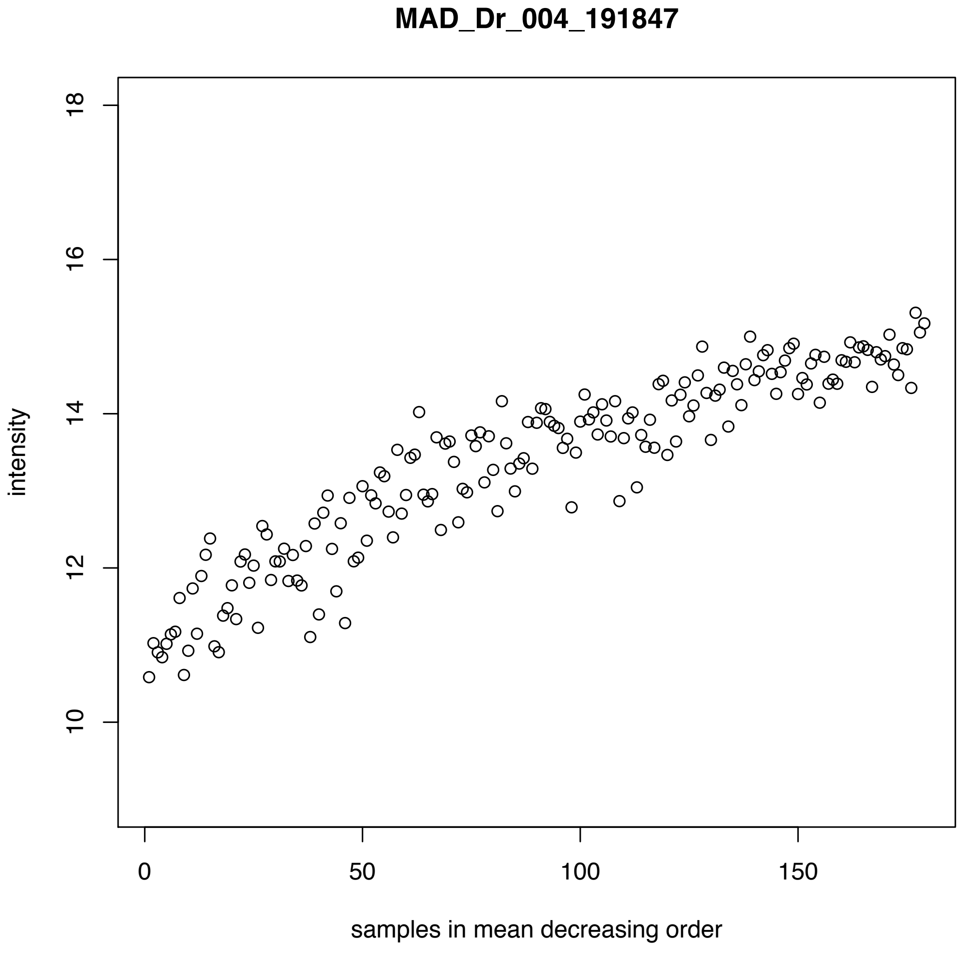
MAD_Dr_004_191847
intensity
samples in mean decreasing order
10
12
14
16
18
0
50
100
150

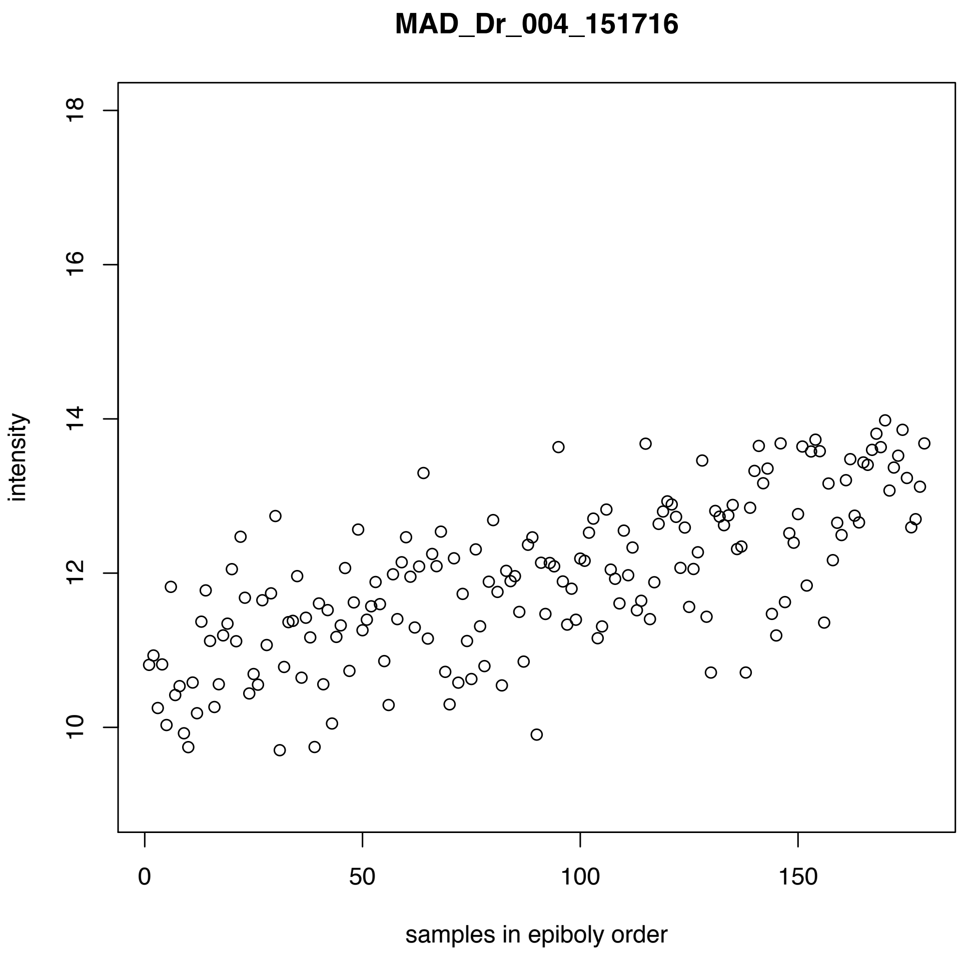
MAD_Dr_004_151716
intensity
10
12
14
16
18
0
50
100
150
samples in epiboly order

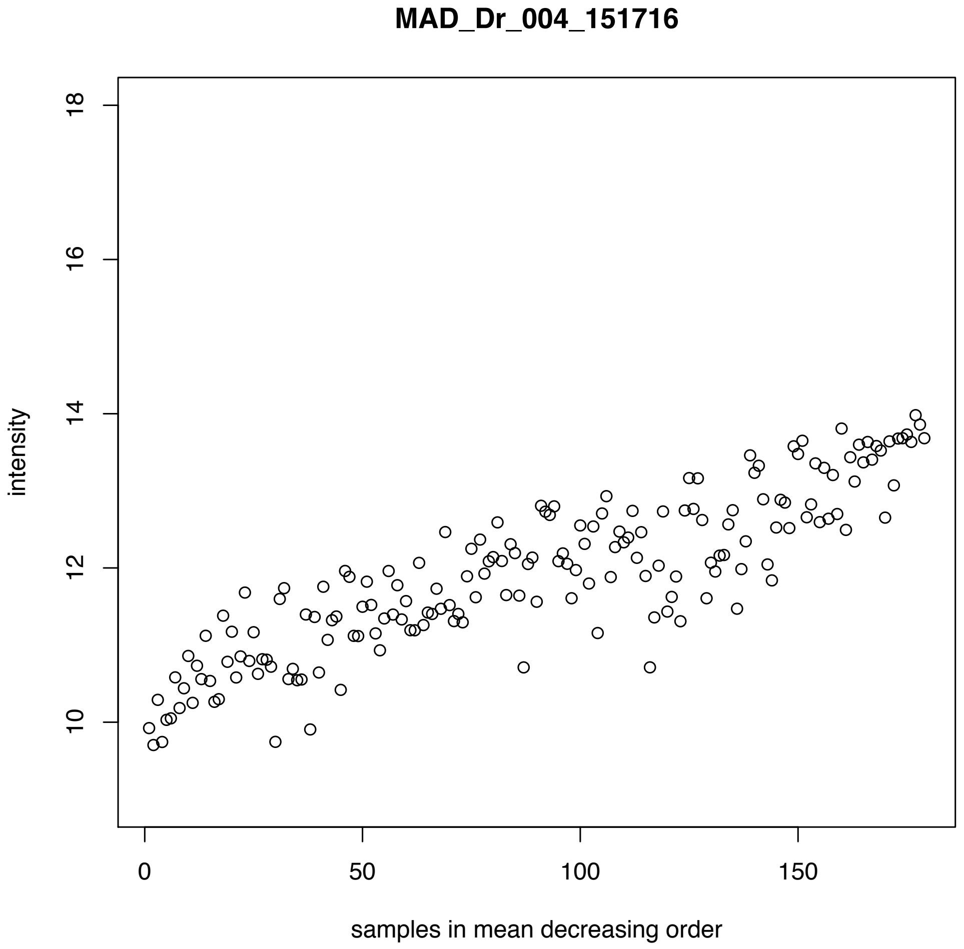
MAD_Dr_004_151716
intensity
10
12
14
16
18
0
50
100
150
samples in mean decreasing order

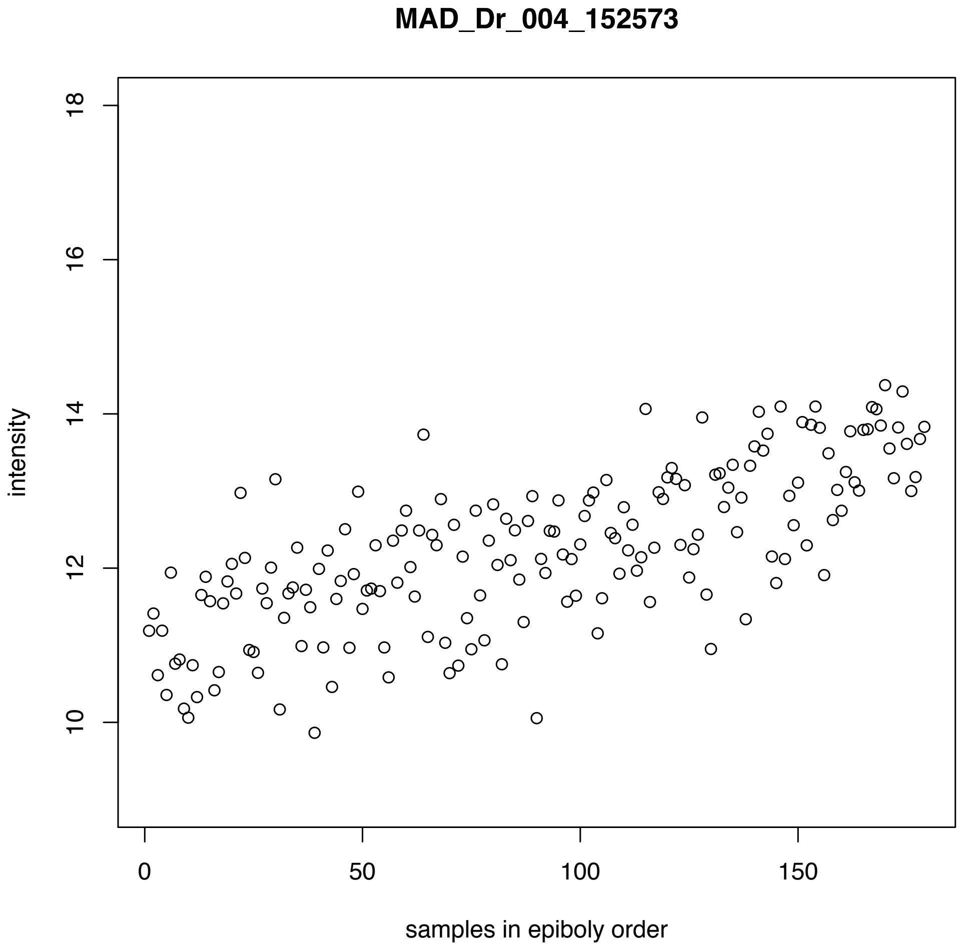
MAD_Dr_004_152573
intensity
10
12
14
16
18
0
50
100
150
samples in epiboly order

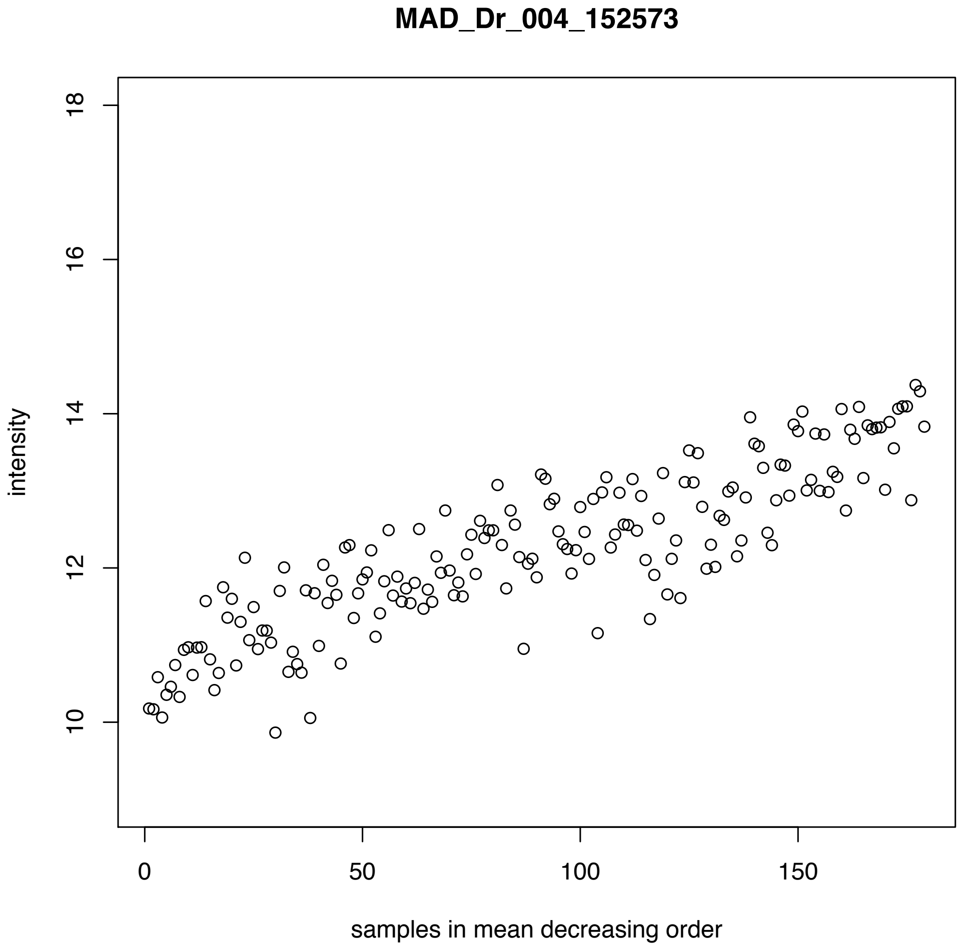
MAD_Dr_004_152573
intensity
10
12
14
16
18
0
50
100
150
samples in mean decreasing order

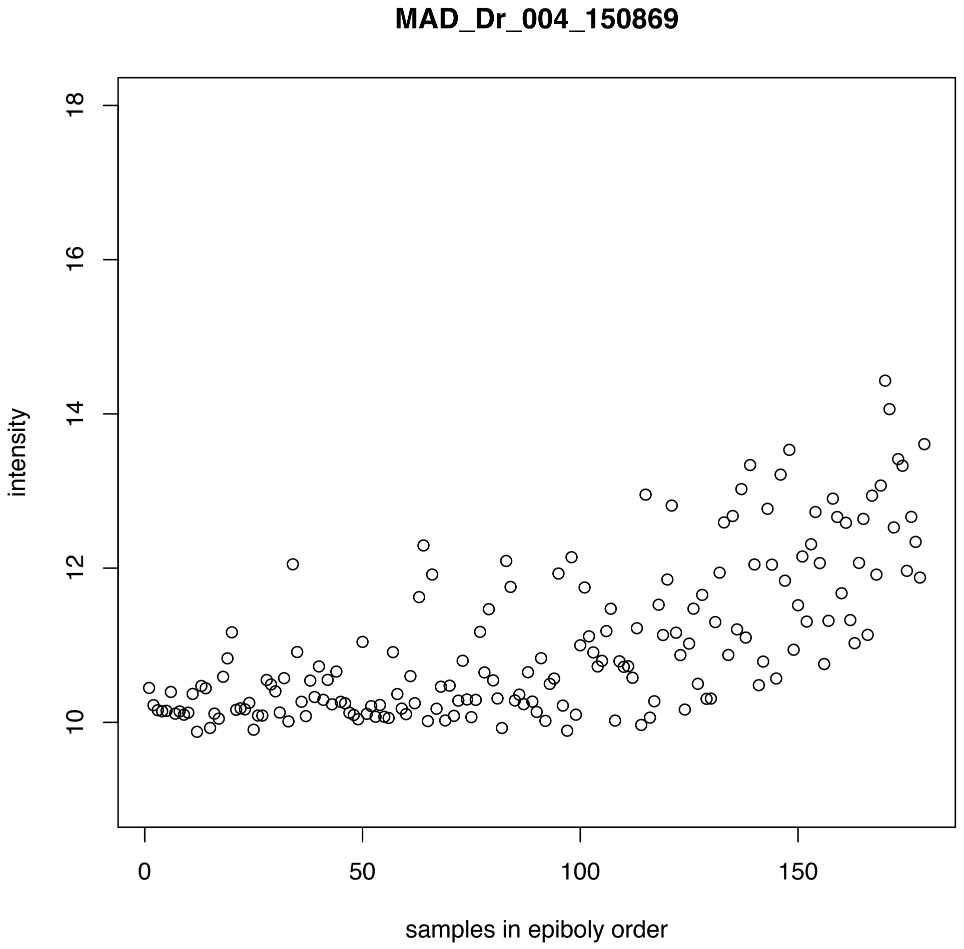
MAD_Dr_004_150869
intensity
samples in epiboly order
10
12
14
16
18
0
50
100
150

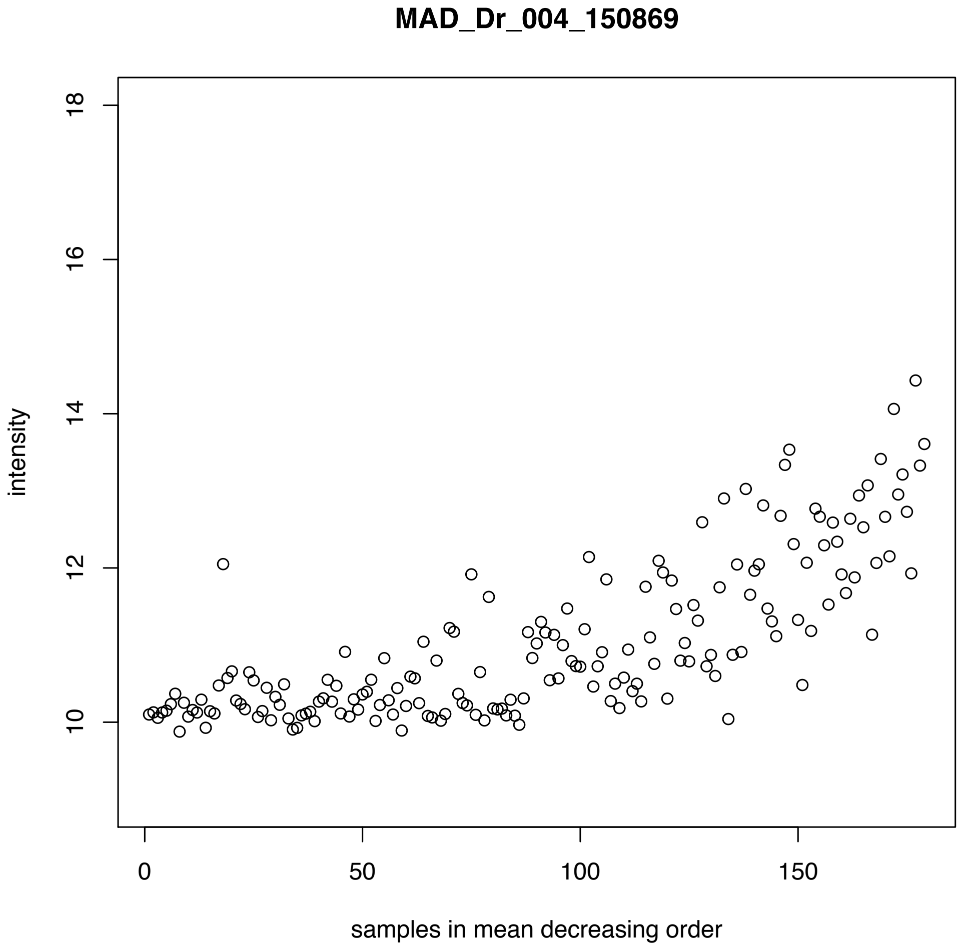
MAD_Dr_004_150869
intensity
samples in mean decreasing order
10
12
14
16
18
0
50
100
150

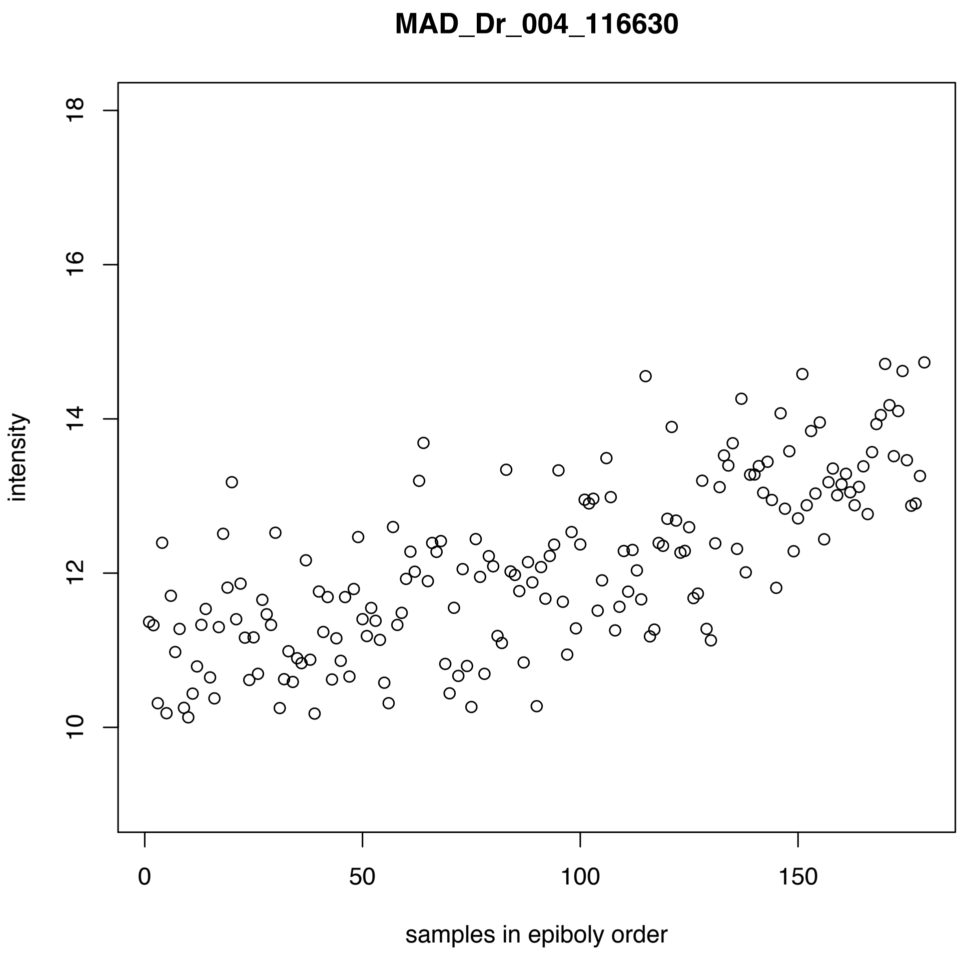
MAD_Dr_004_116630
intensity
10
12
14
16
18
0
50
100
150
samples in epiboly order

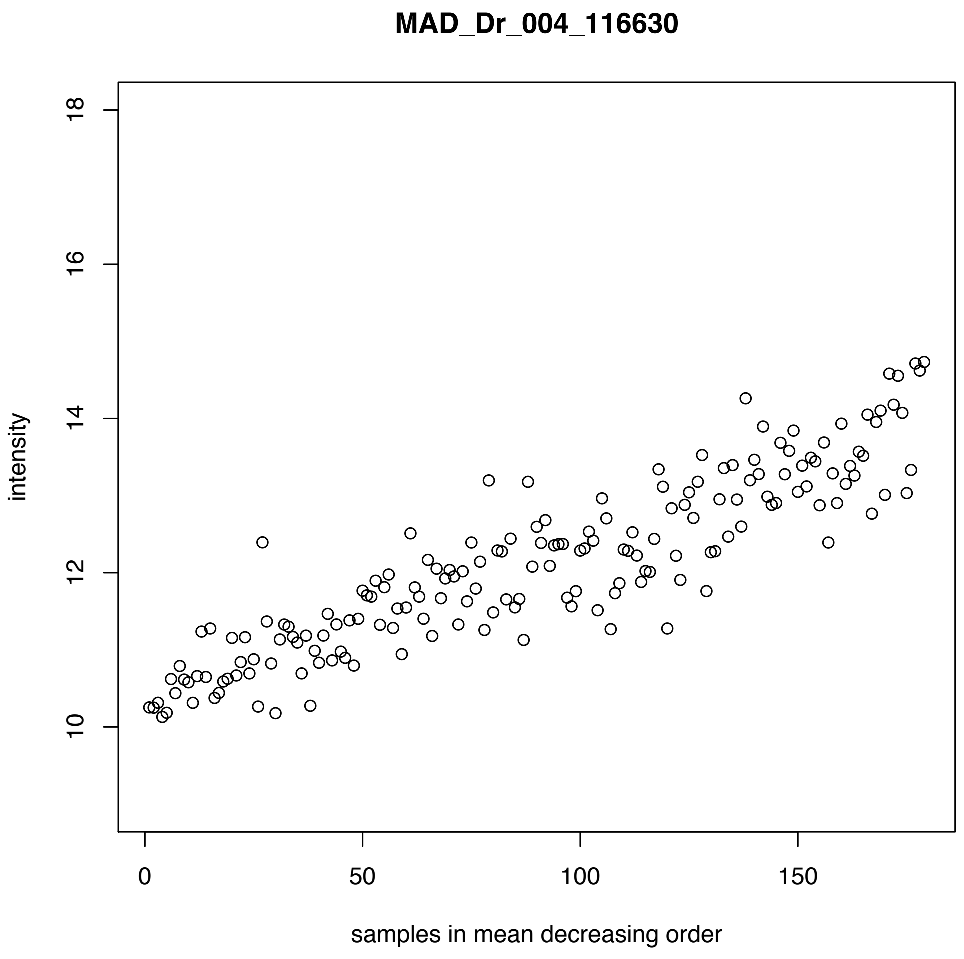
MAD_Dr_004_116630
intensity
10
12
14
16
18
0
50
100
150
samples in mean decreasing order

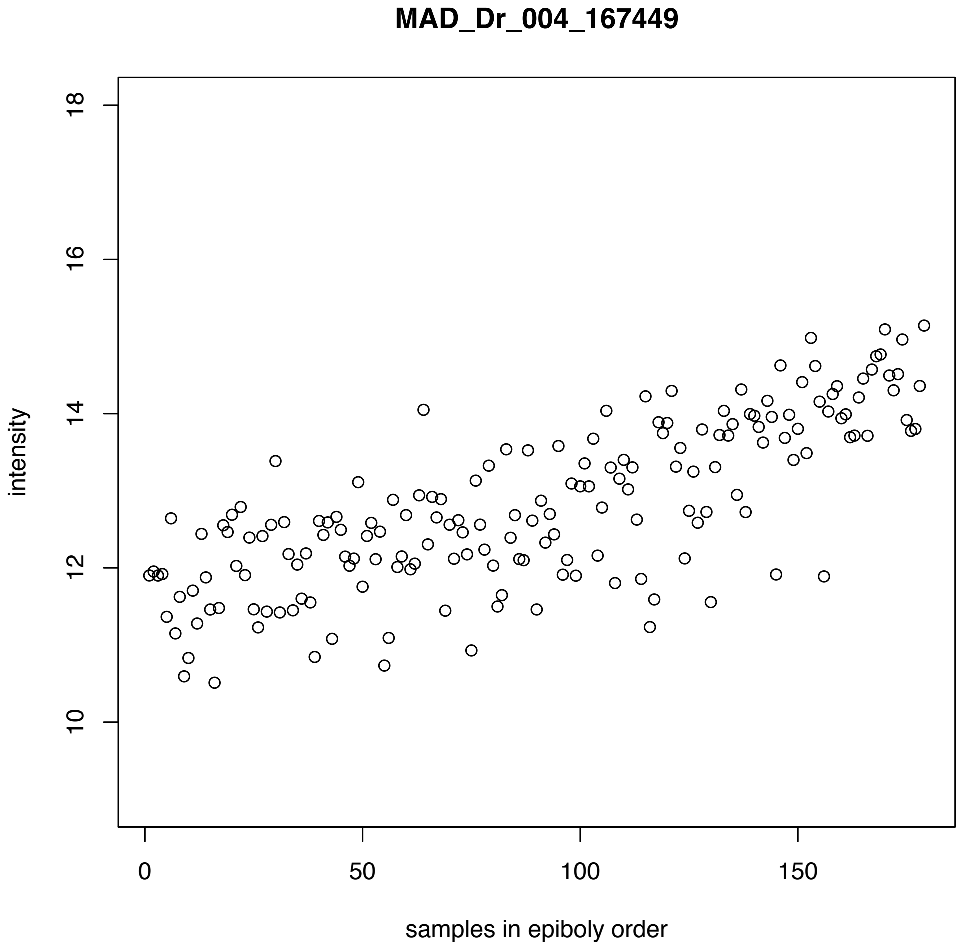
MAD_Dr_004_167449
intensity
samples in epiboly order
10
12
14
16
18
0
50
100
150

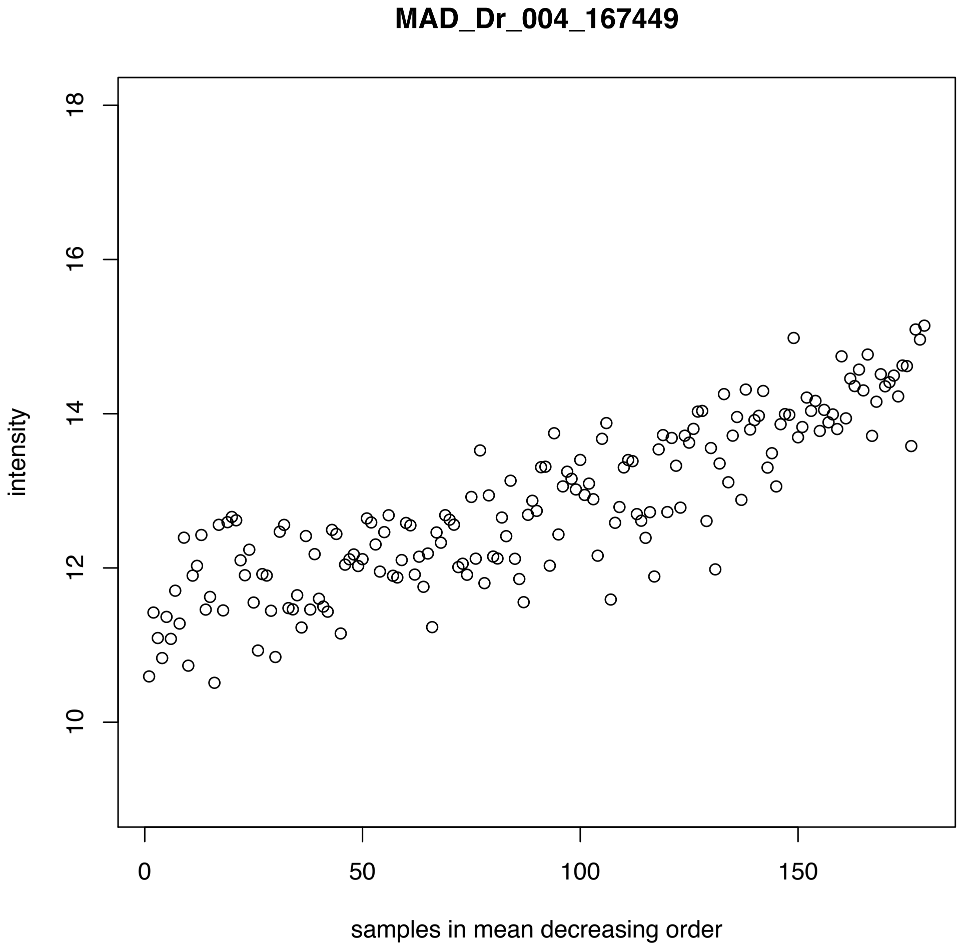
MAD_Dr_004_167449
intensity
samples in mean decreasing order
10
12
14
16
18
0
50
100
150

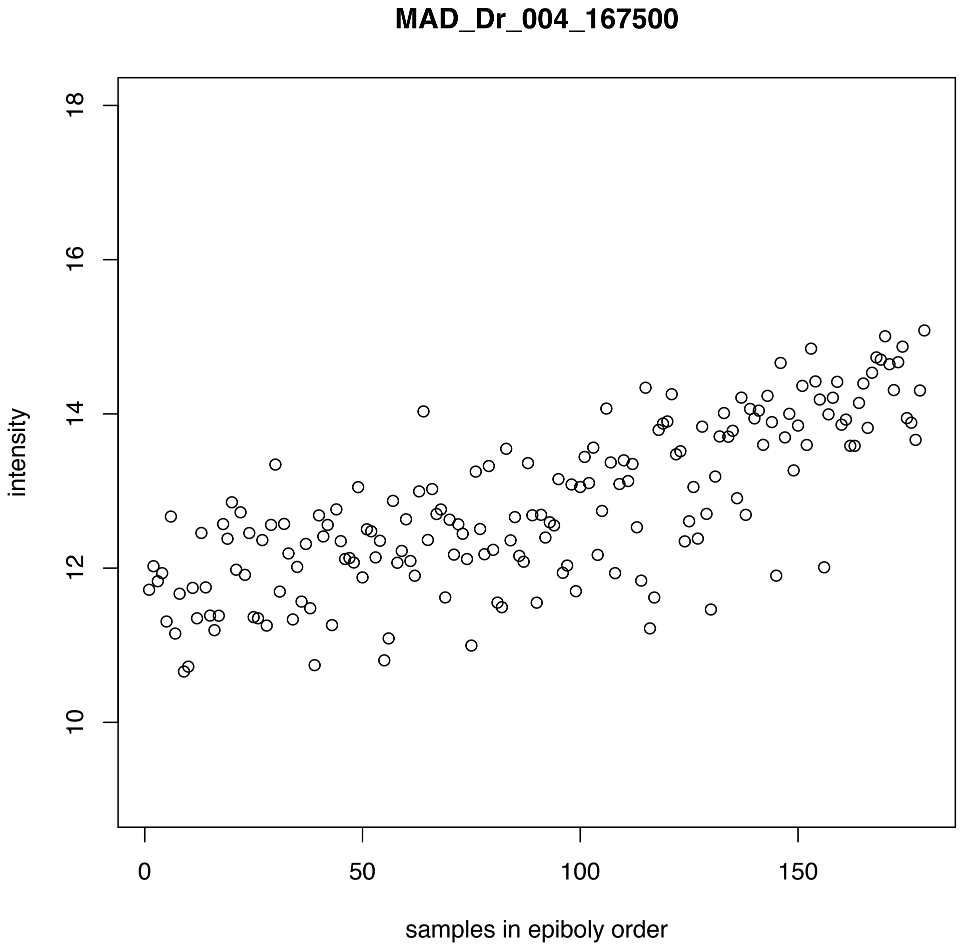

MAD_Dr_004_167500
intensity
10
12
14
16
18
0
50
100
150
samples in epiboly order

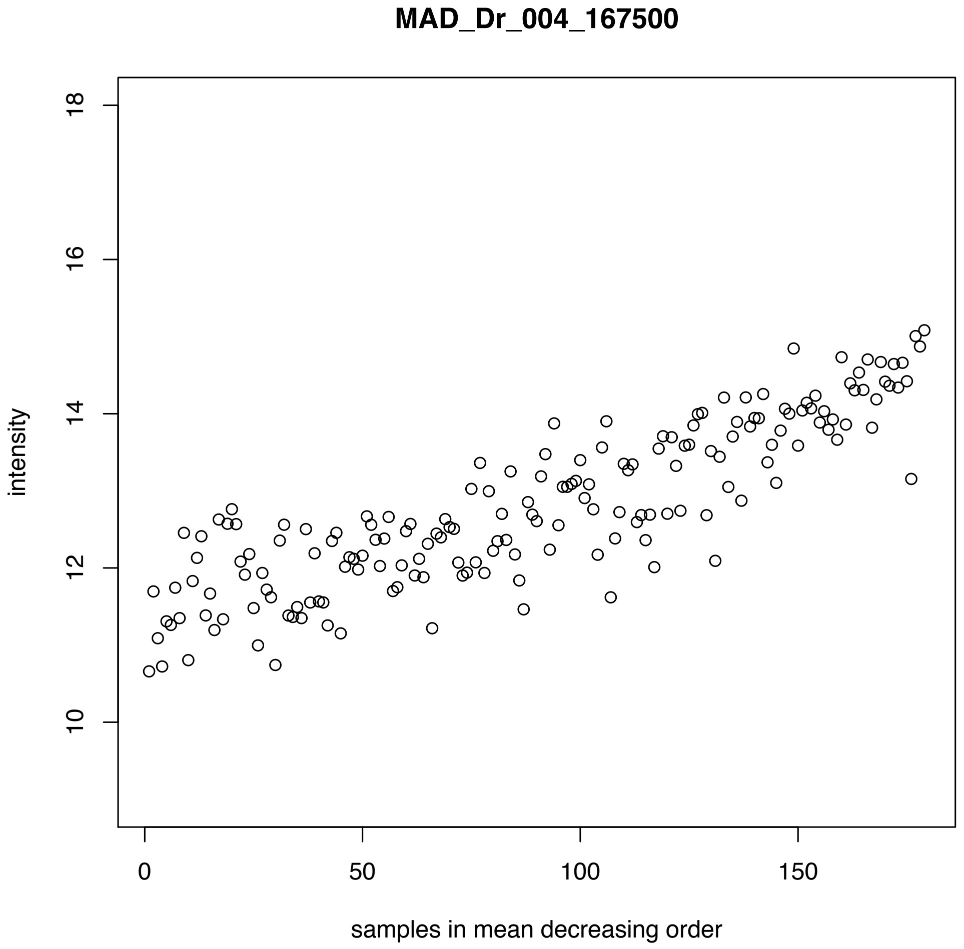

MAD_Dr_004_167500
intensity
10
12
14
16
18
0
50
100
150
samples in mean decreasing order

MAD_Dr_004_168114

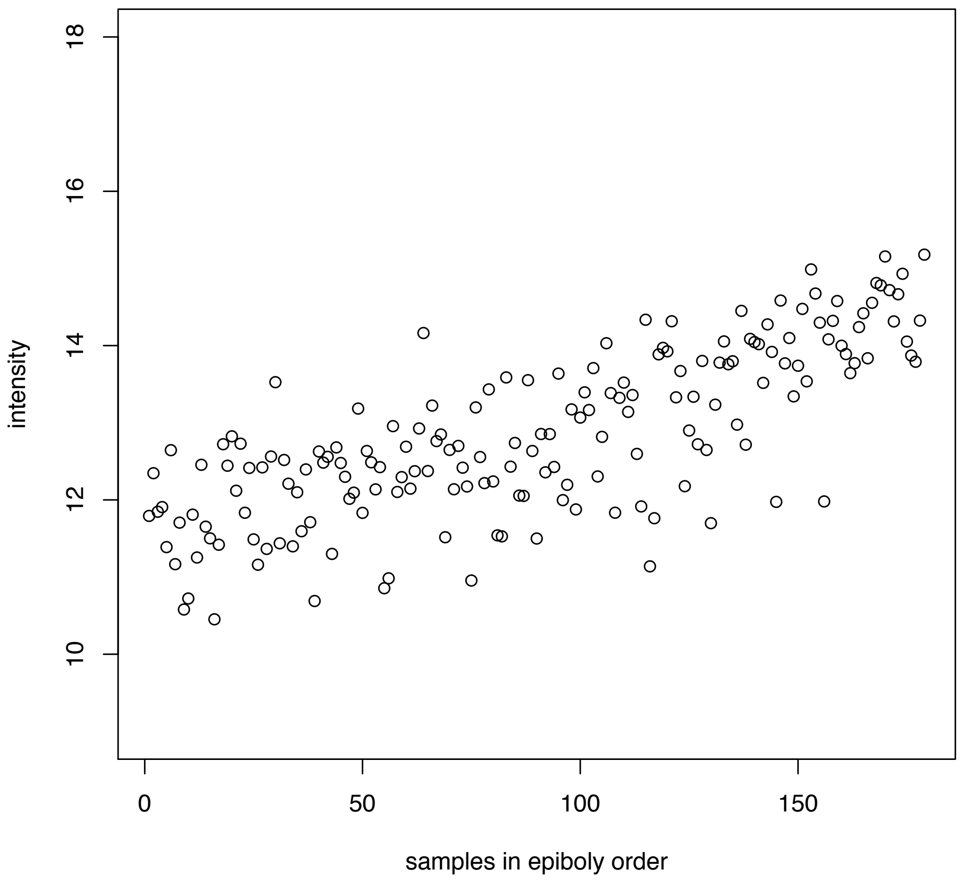

MAD_Dr_004_168114

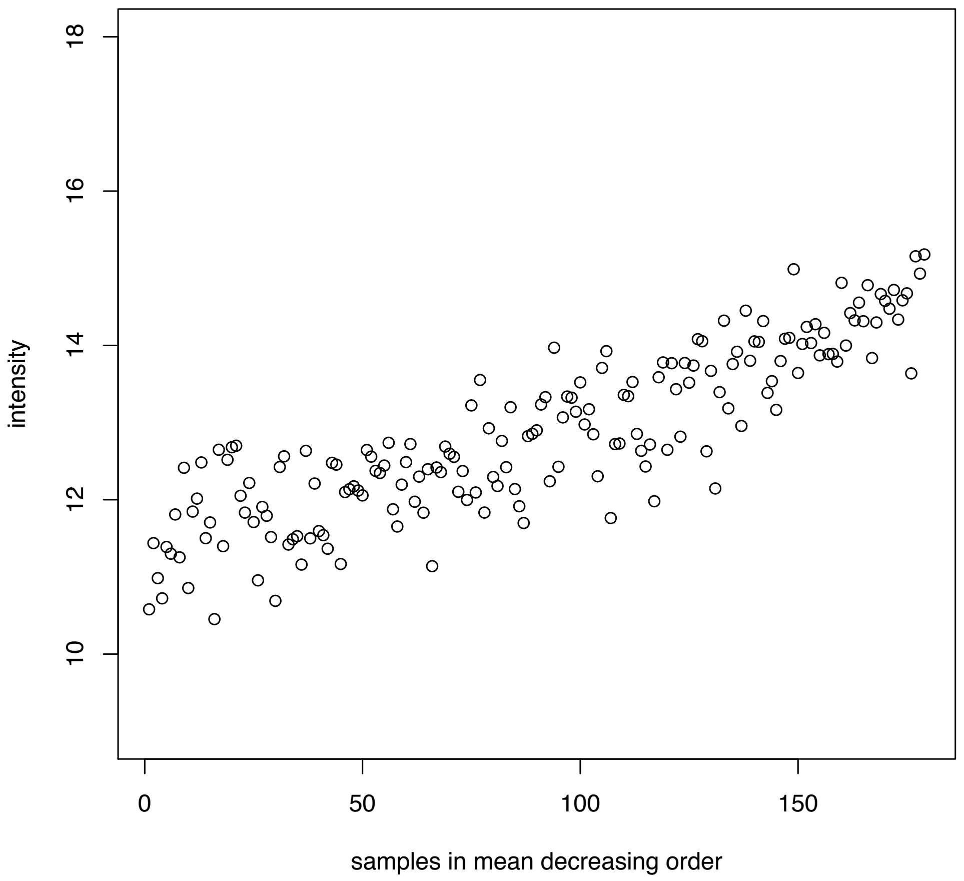

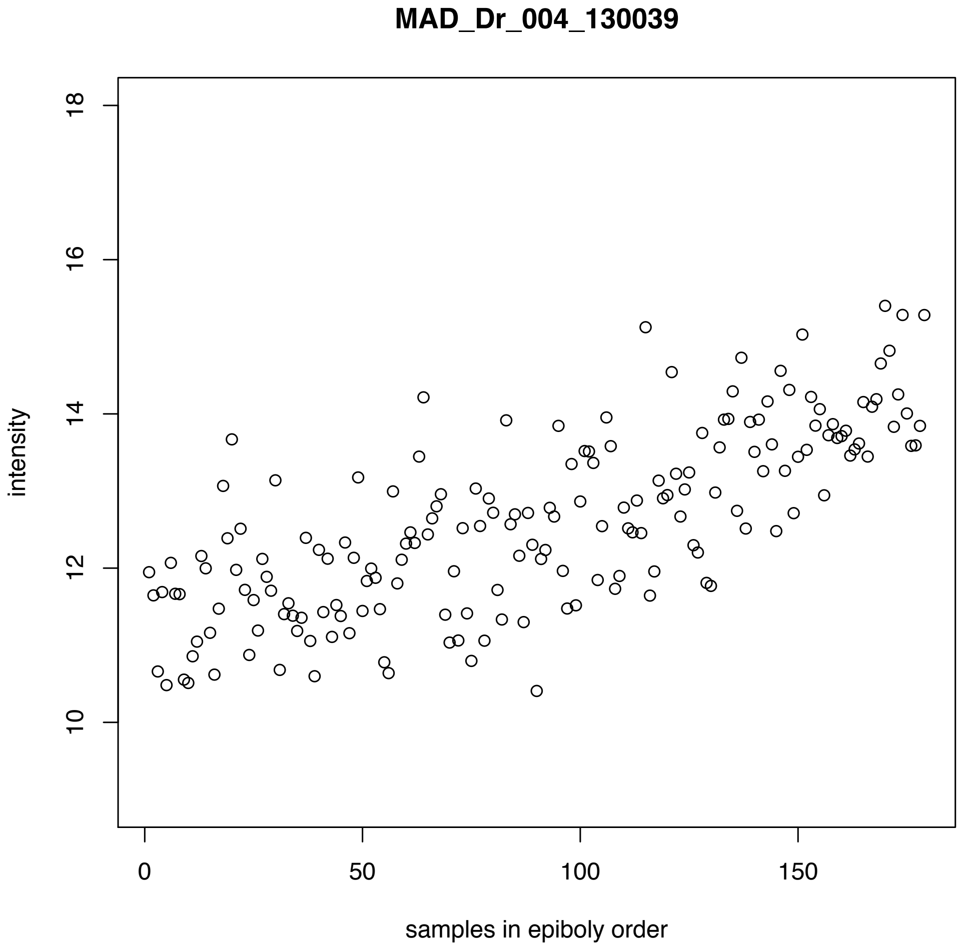
MAD_Dr_004_130039
intensity
samples in epiboly order
0
50
100
150
10
12
14
16
18

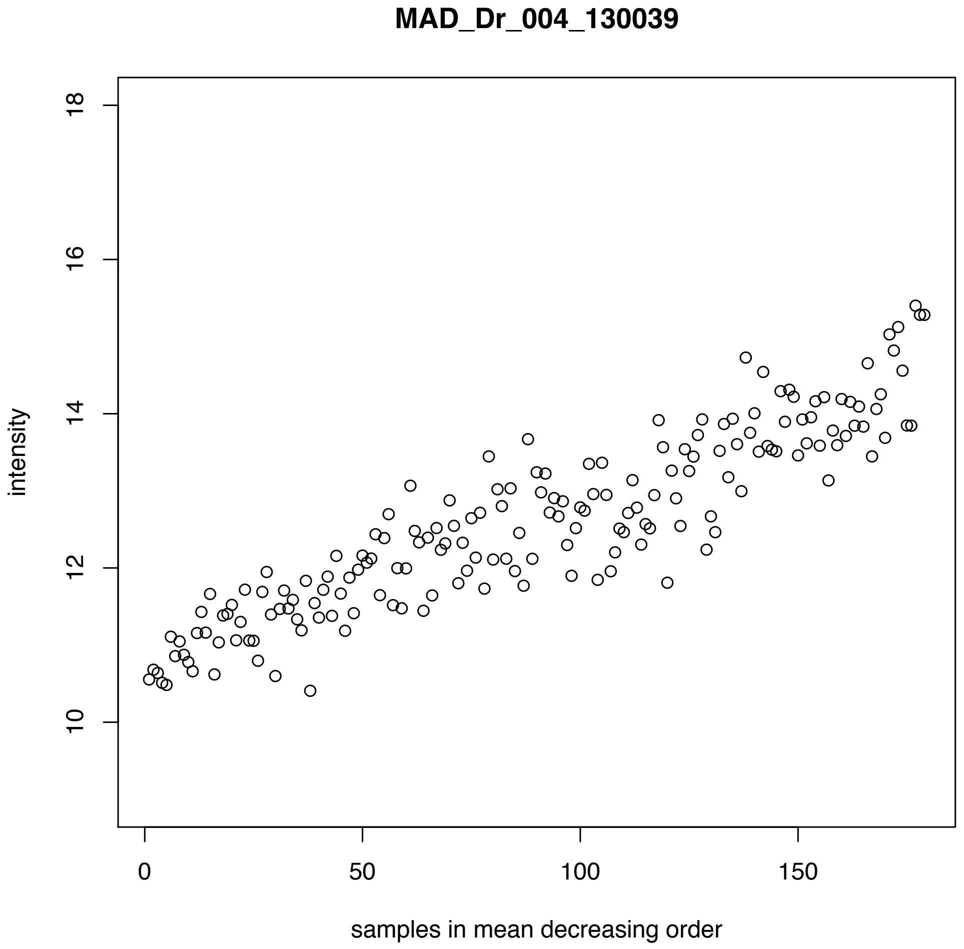
MAD_Dr_004_130039
intensity
samples in mean decreasing order
0
50
100
150
10
12
14
16
18

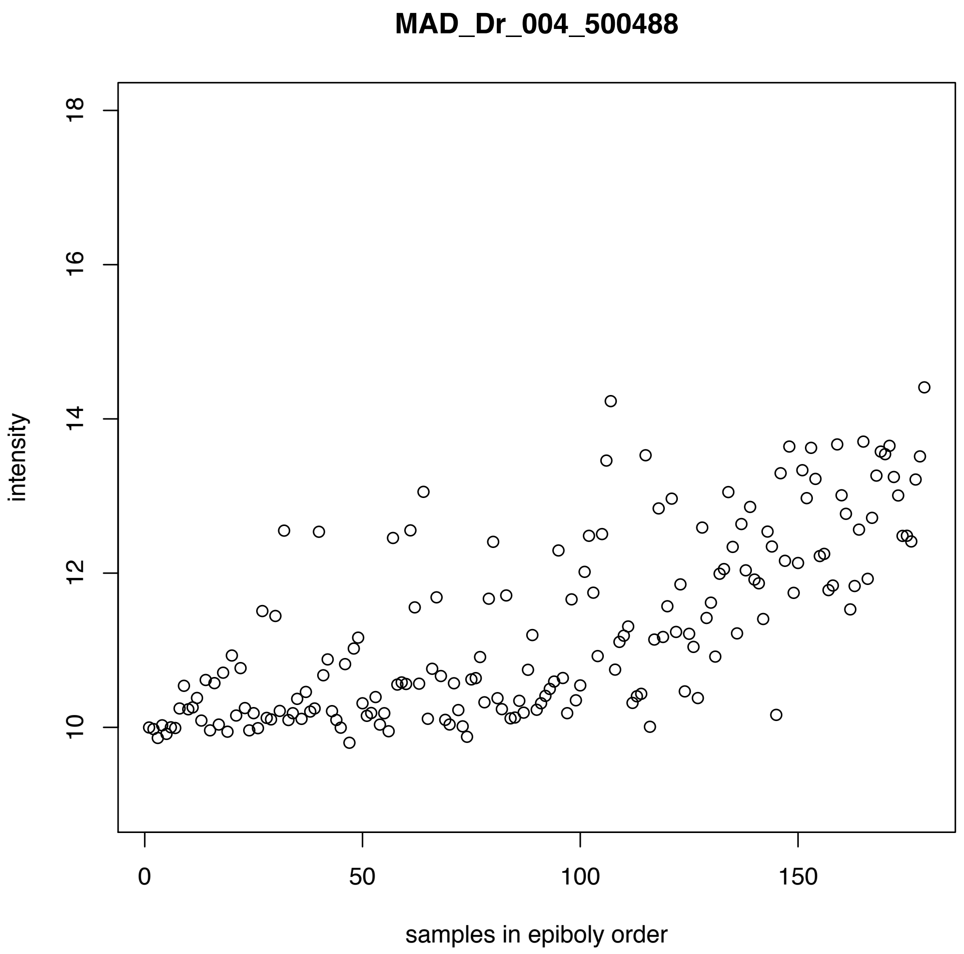
MAD_Dr_004_500488
intensity
10
12
14
16
18
0
50
100
150
samples in epiboly order

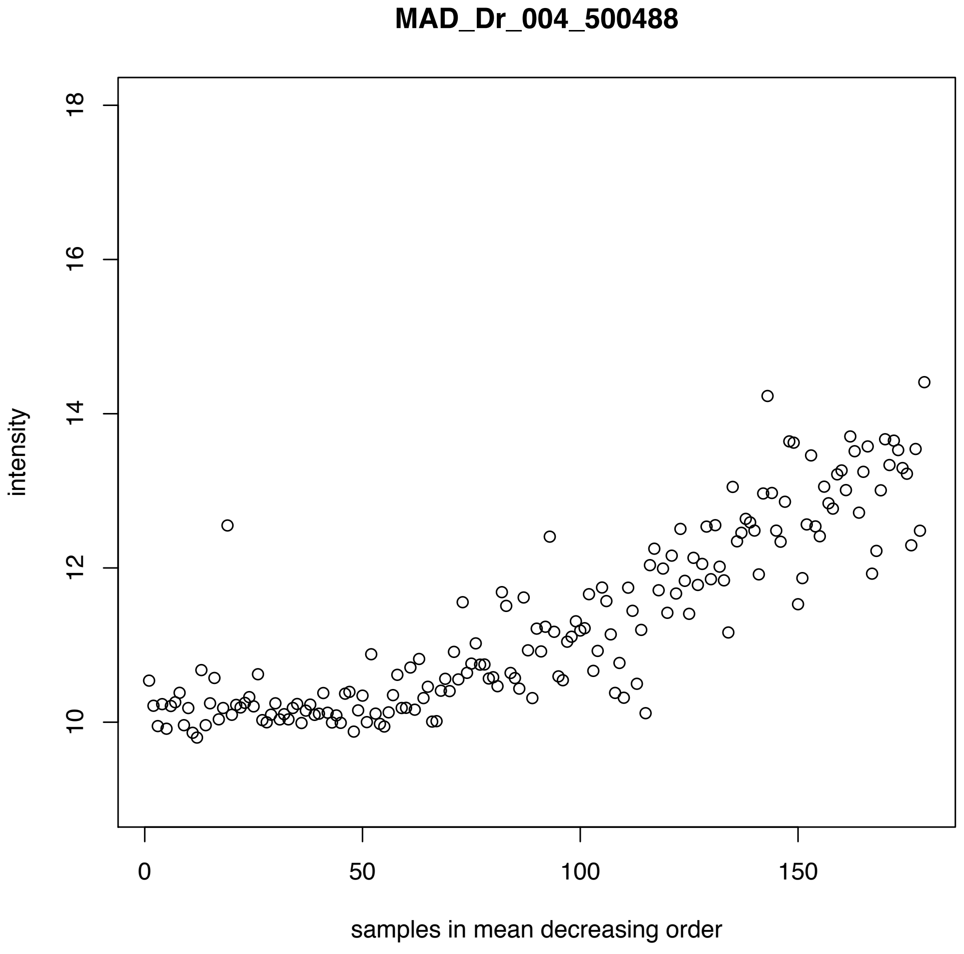
MAD_Dr_004_500488
intensity
10
12
14
16
18
0
50
100
150
samples in mean decreasing order

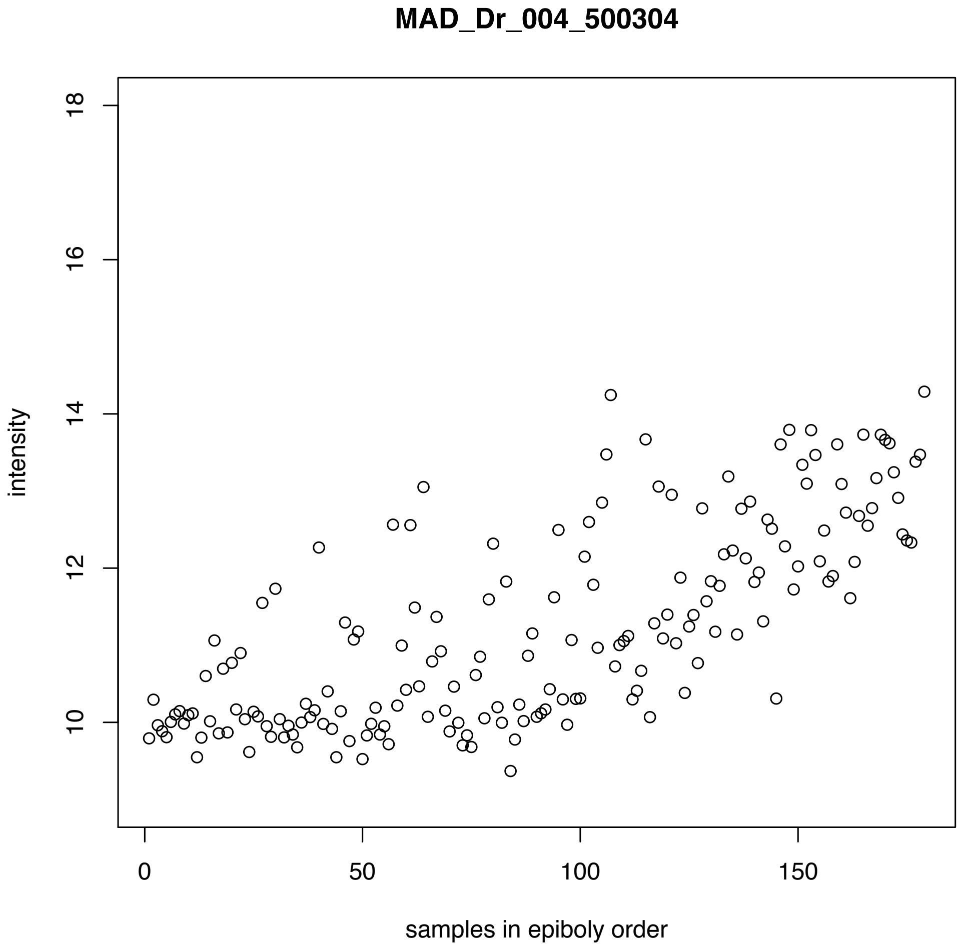

MAD_Dr_004_500304
intensity
10
12
14
16
18
0
50
100
150
samples in epiboly order

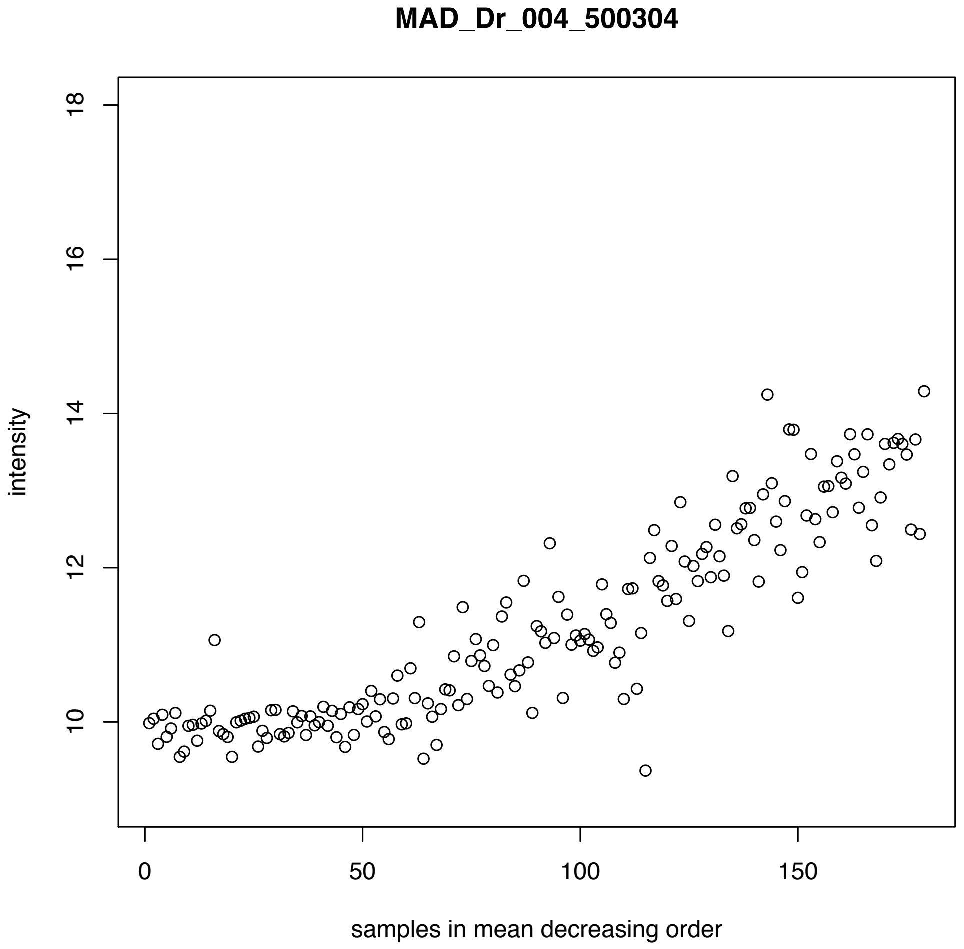

MAD_Dr_004_500304
intensity
10
12
14
16
18
0
50
100
150
samples in mean decreasing order

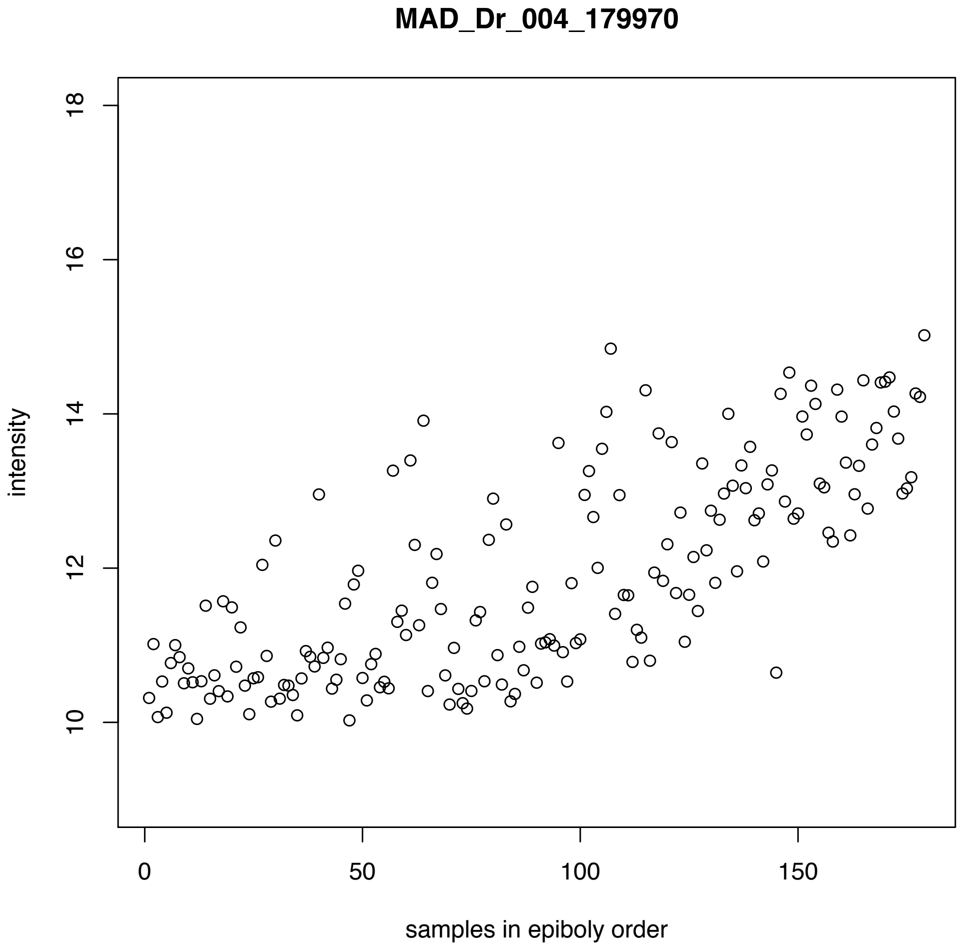
MAD_Dr_004_179970
intensity
10
12
14
16
18
0
50
100
150
samples in epiboly order

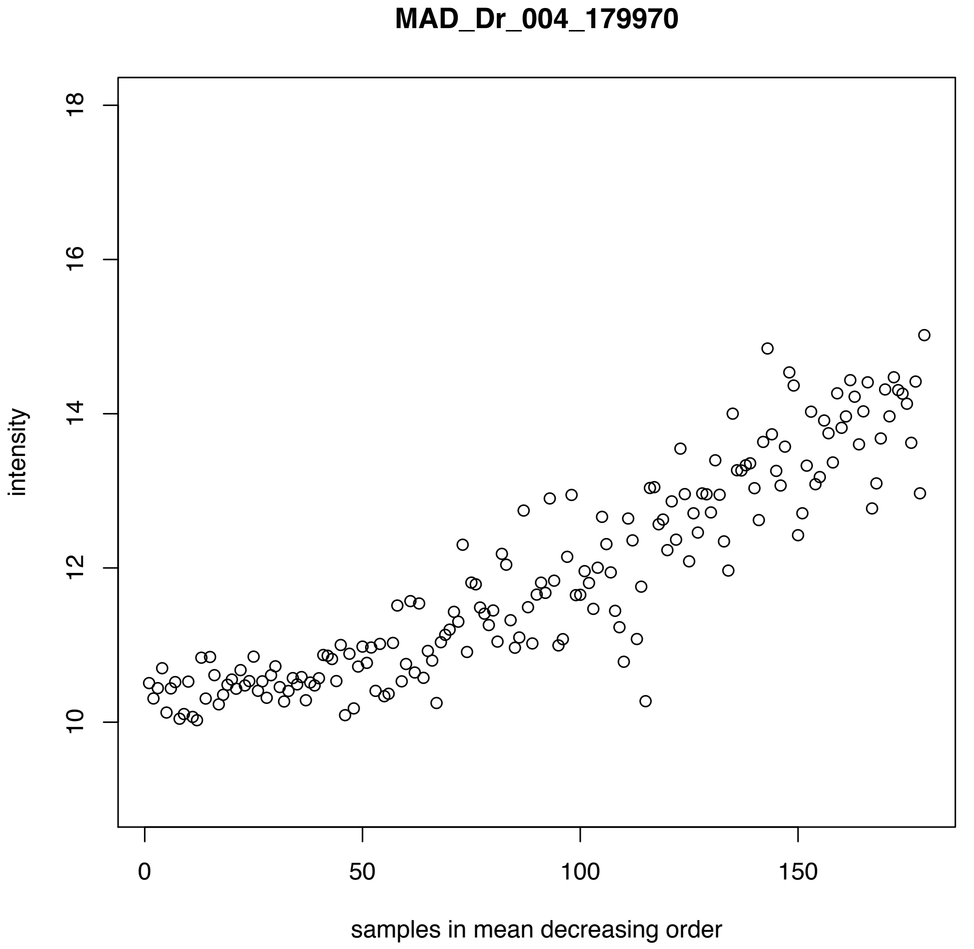
MAD_Dr_004_179970
intensity
10
12
14
16
18
0
50
100
150
samples in mean decreasing order

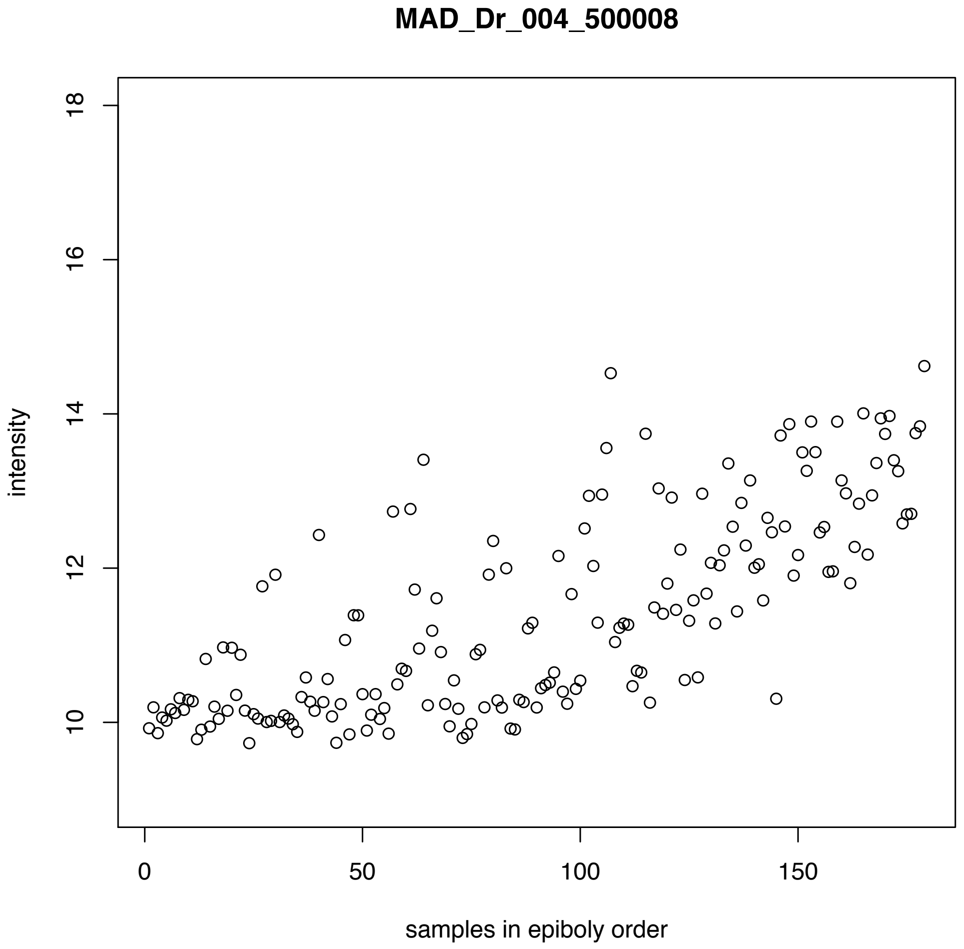
MAD_Dr_004_500008
intensity
10
12
14
16
18
0
50
100
150
samples in epiboly order

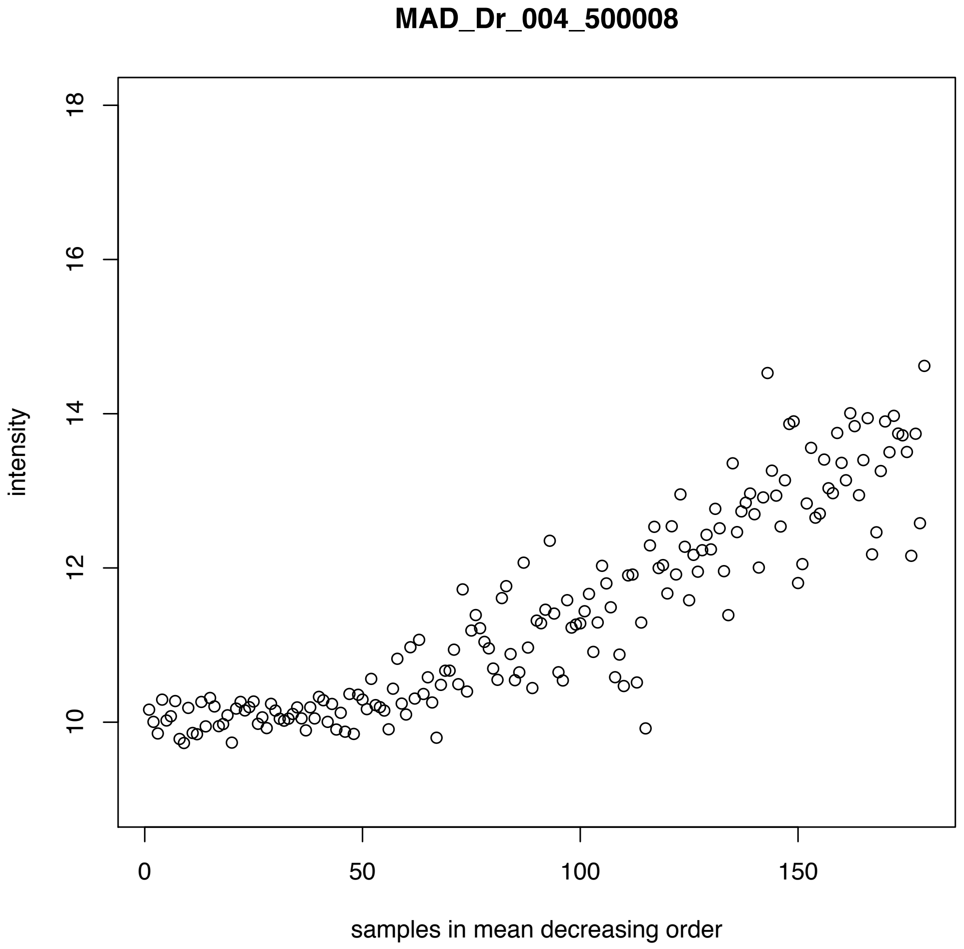
MAD_Dr_004_500008
intensity
10
12
14
16
18
0
50
100
150
samples in mean decreasing order

MAD_Dr_004_171212

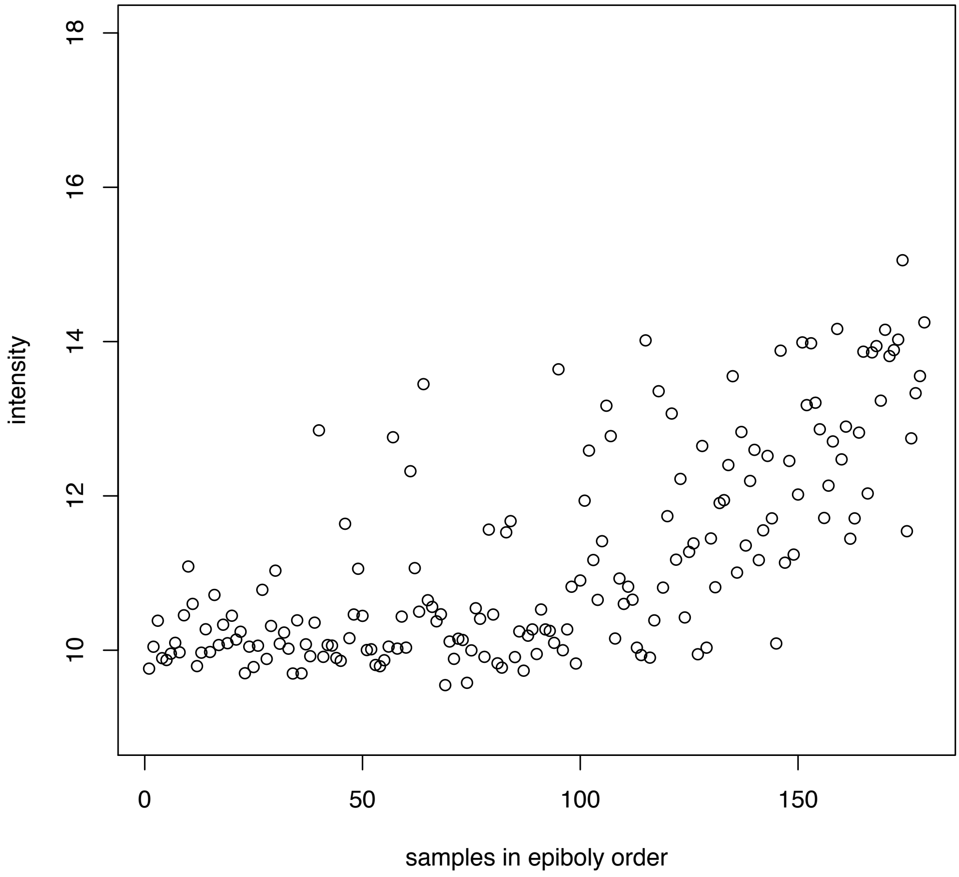

MAD_Dr_004_171212

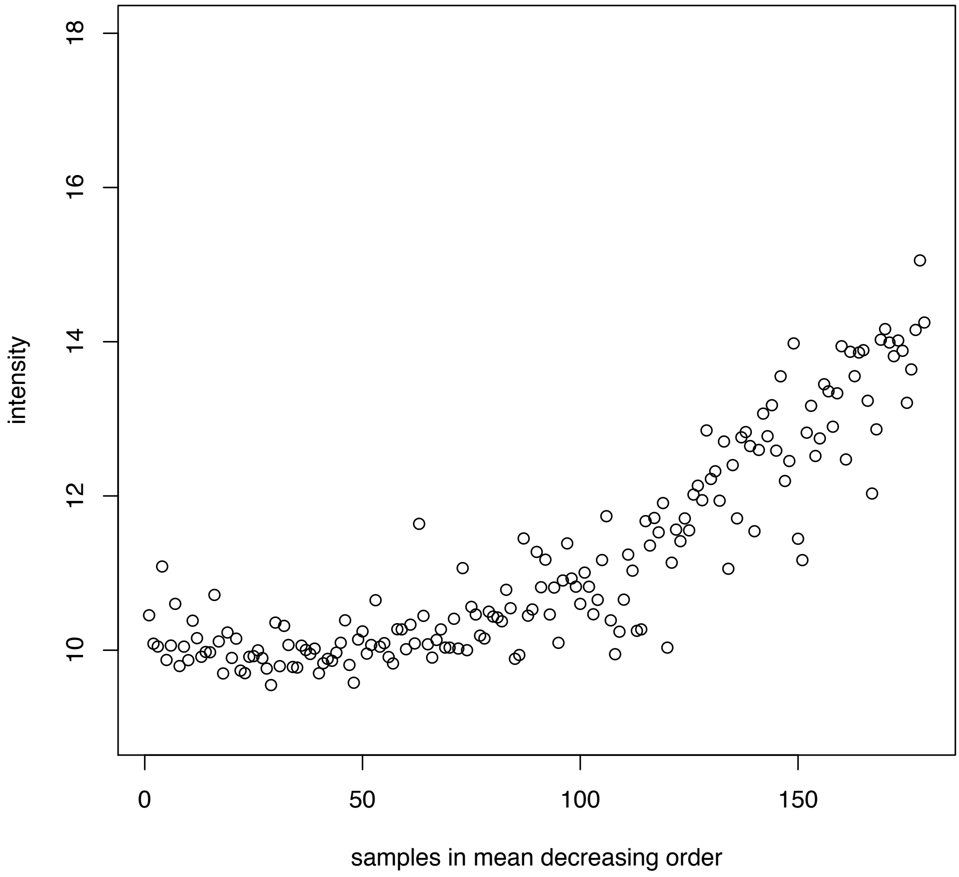

MAD_Dr_004_182196

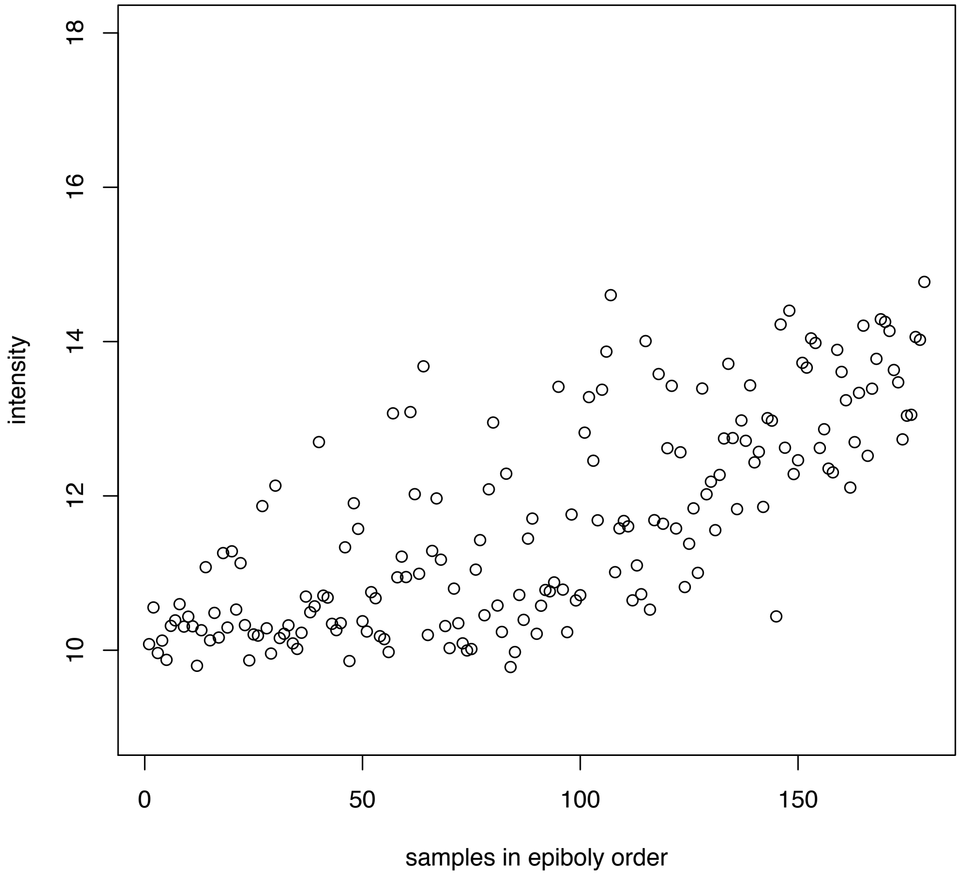

MAD_Dr_004_182196

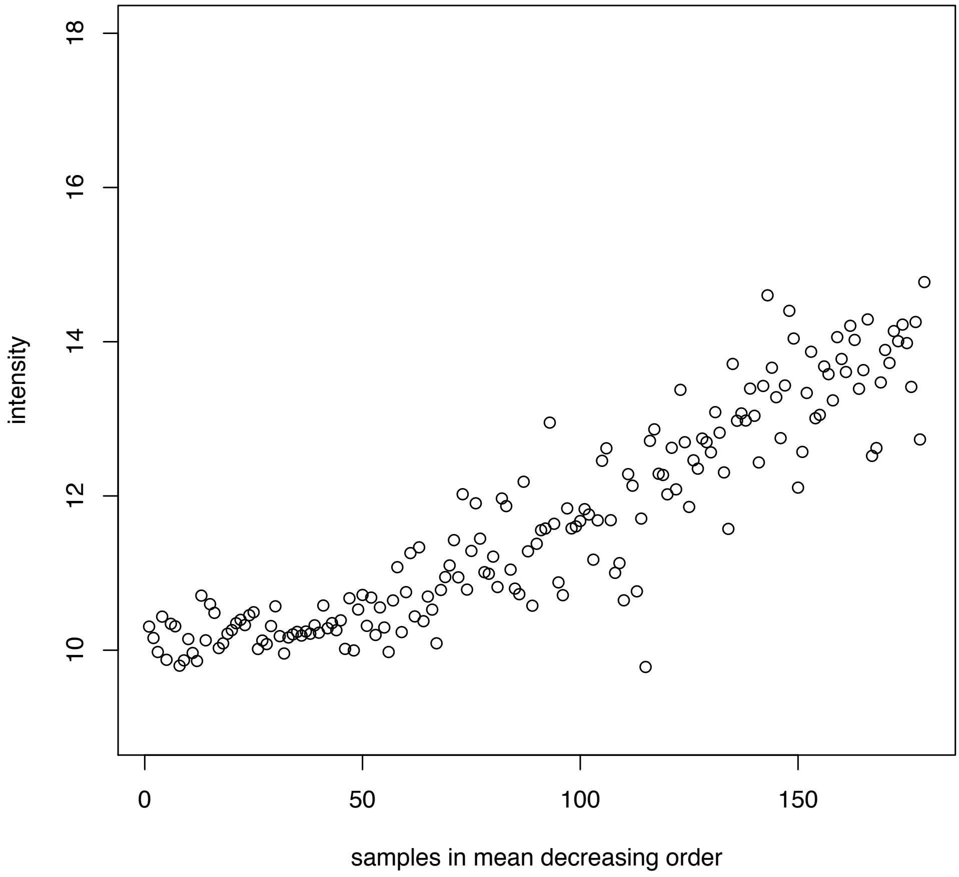

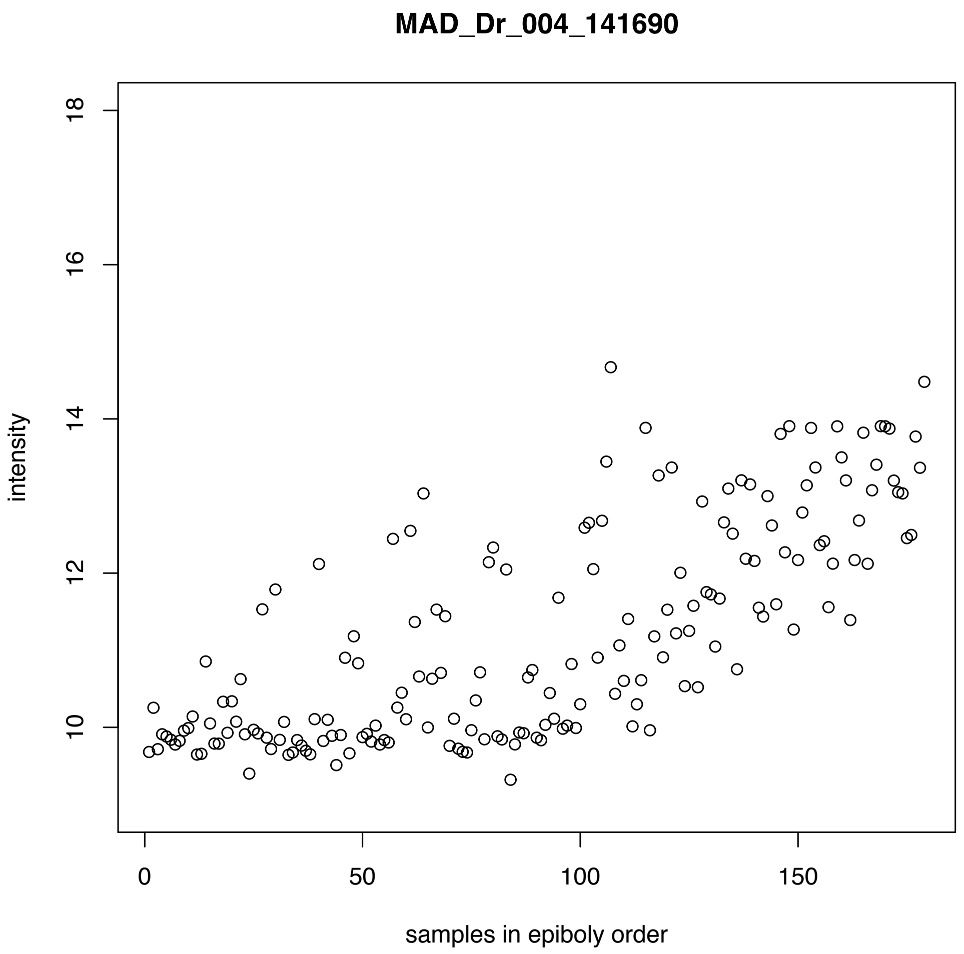
MAD_Dr_004_141690
intensity
samples in epiboly order
10
12
14
16
18
0
50
100
150

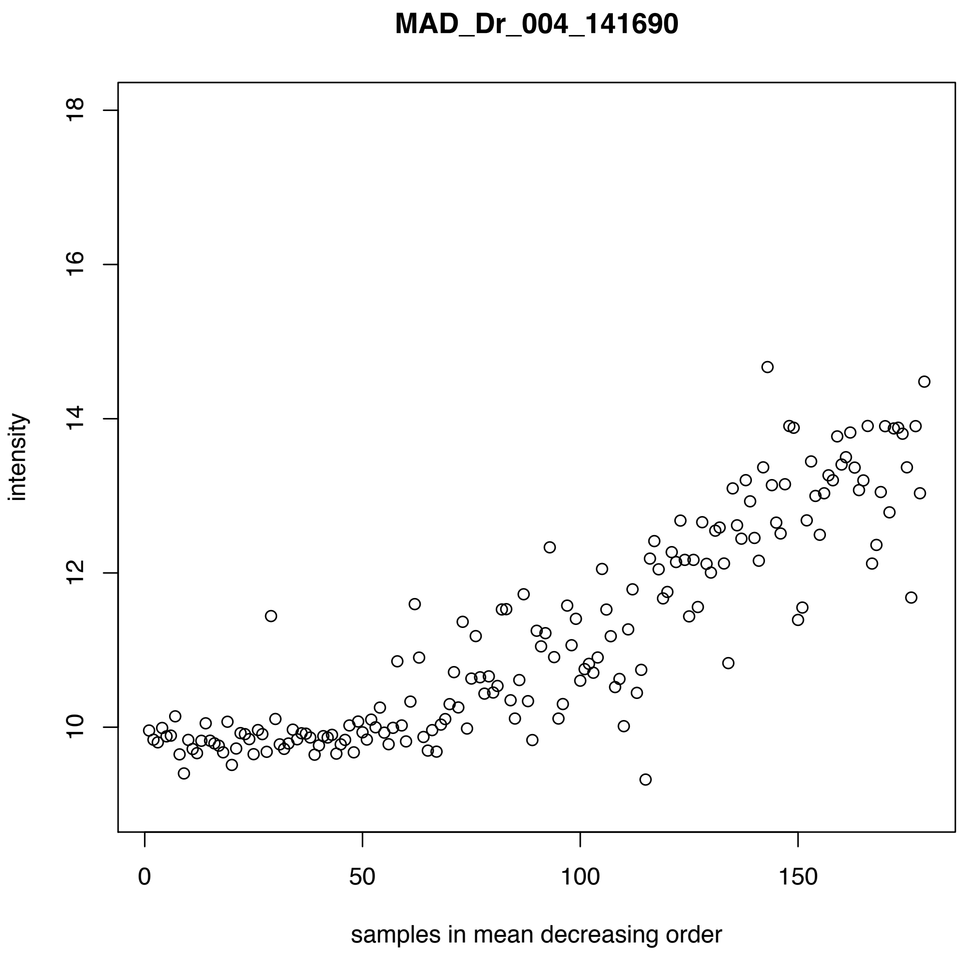
MAD_Dr_004_141690
intensity
samples in mean decreasing order
10
12
14
16
18
0
50
100
150

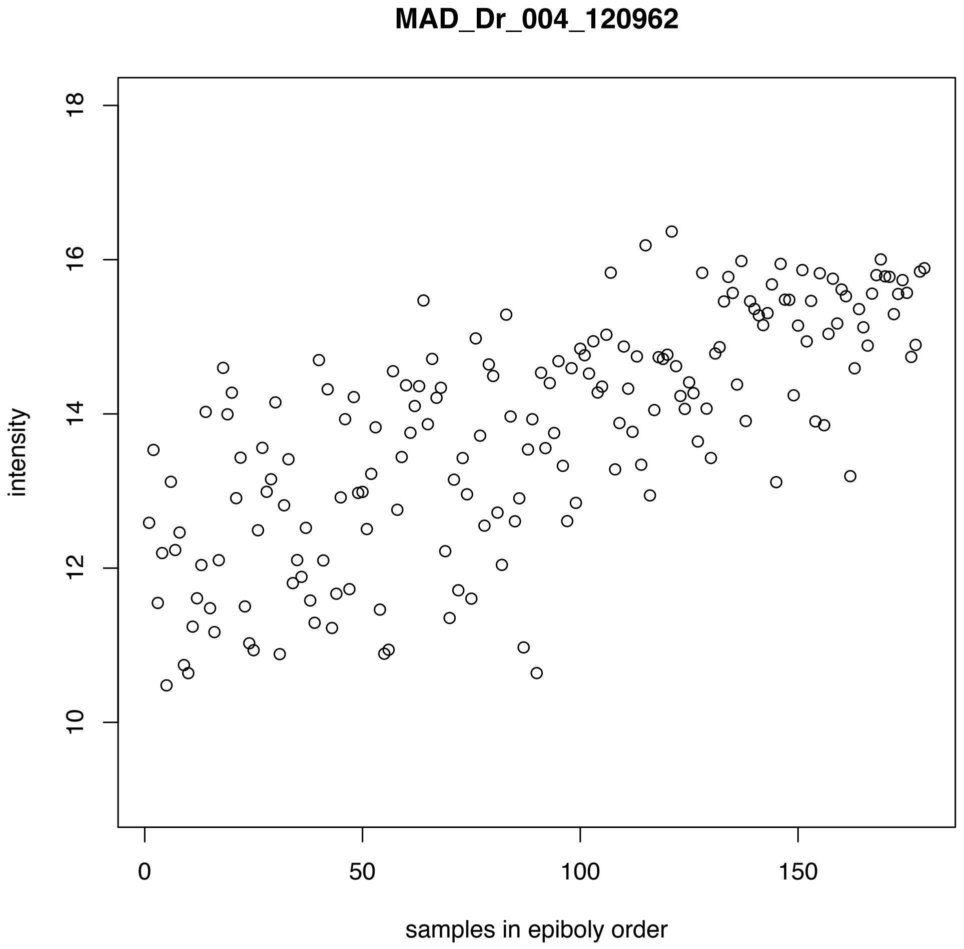
MAD_Dr_004_120962
intensity
10
12
14
16
18
0
50
100
150
samples in epiboly order

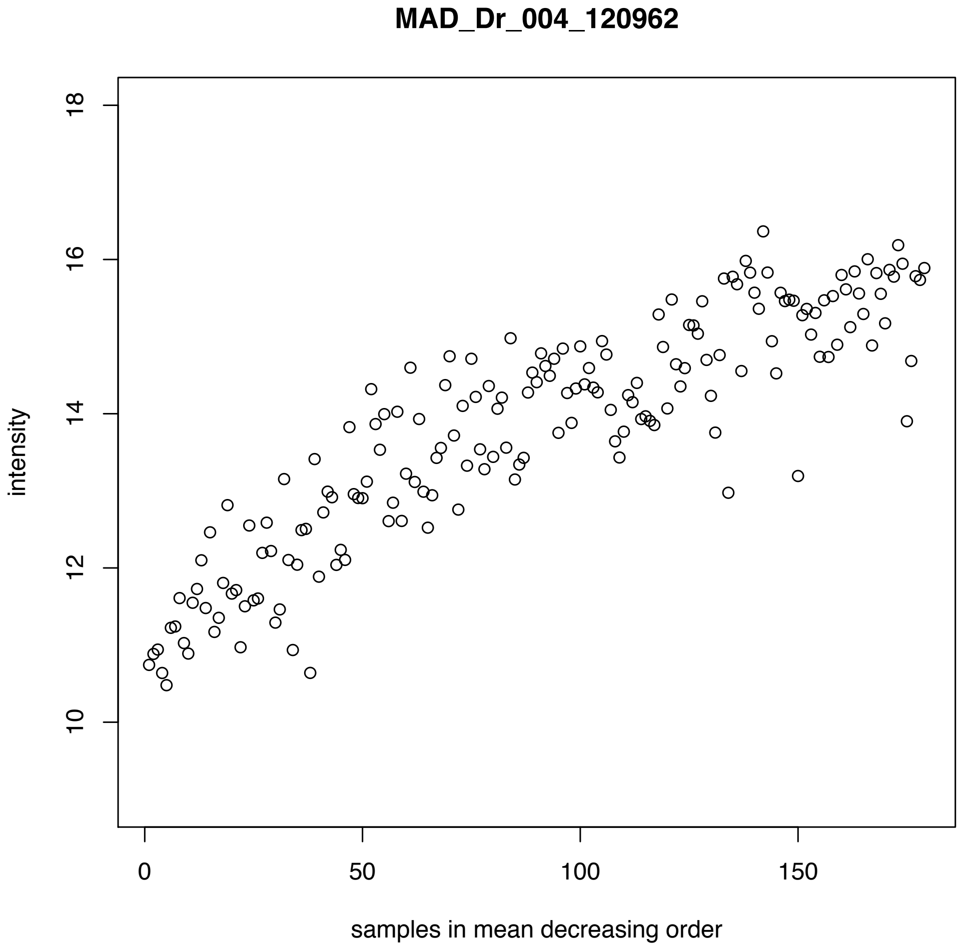
MAD_Dr_004_120962
intensity
10
12
14
16
18
0
50
100
150
samples in mean decreasing order
