# Supplementary material for: Transcriptome dynamics in early zebrafish embryogenesis determined by high-resolution time course analysis of 180 successive, individual zebrafish embryos
Source: BMC Genomics. 2017 Apr 11;18:287. doi: 10.1186/s12864-017-3672-z (PMC5387192; doi:10.1186/s12864-017-3672-z)
Supplement: Supplementary file 12 — Multi-level genes. On each page the left panel displays the expression intensities of a gene in developmental order, the right panel displays the same gene plotted in developmental order ordered per spawn. (PDF 354 kb) [file 12864_2017_3672_MOESM12_ESM.pdf]

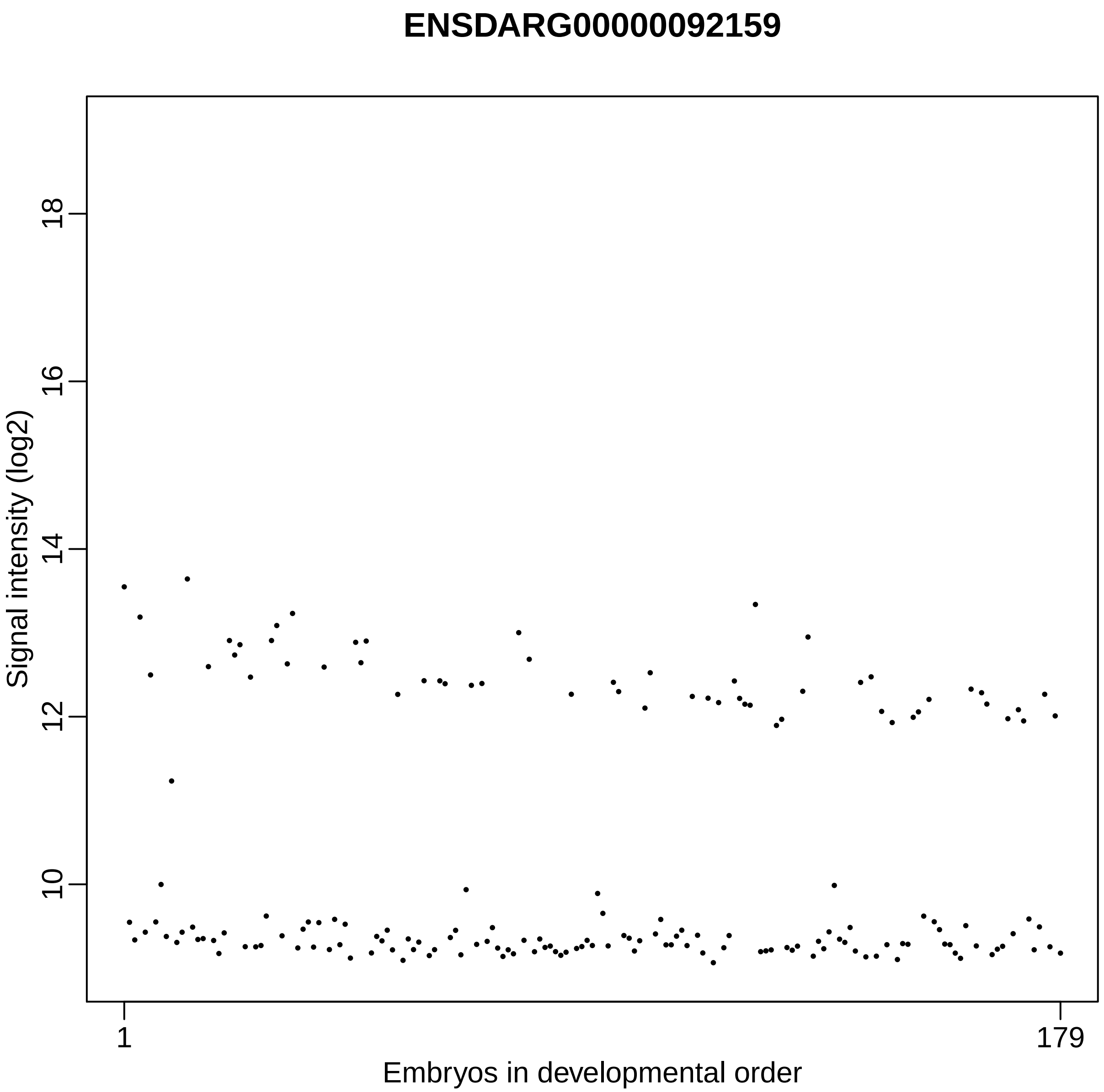
ENSDARG00000092159
Signal intensity (log2)
10
12
14
16
18
1
179
Embryos in developmental order

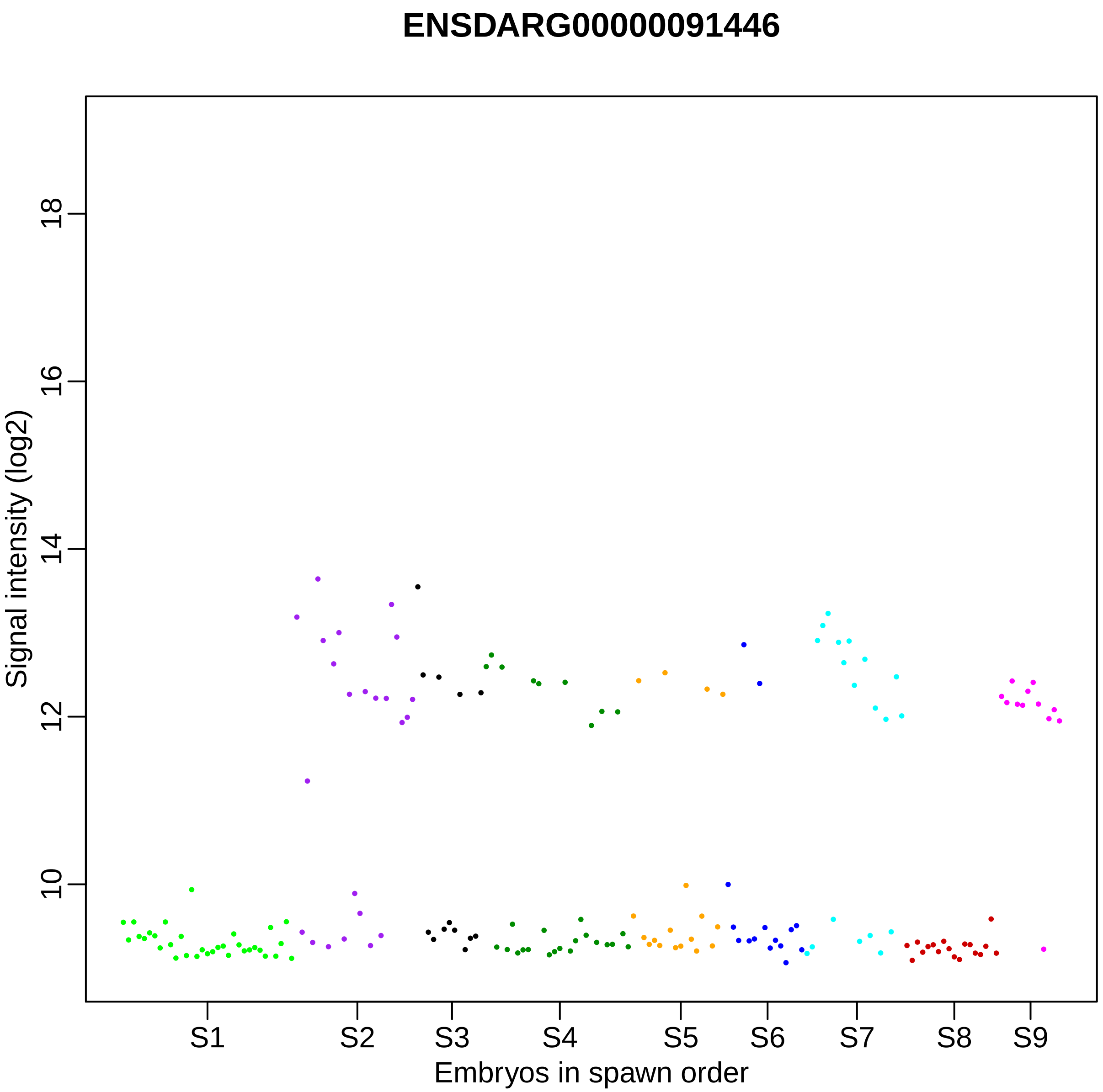
ENSDARG00000091446
Signal intensity (log2)
10
12
14
16
18
S1
S2
S3
S4
S5
S6
S7
S8
S9
Embryos in spawn order

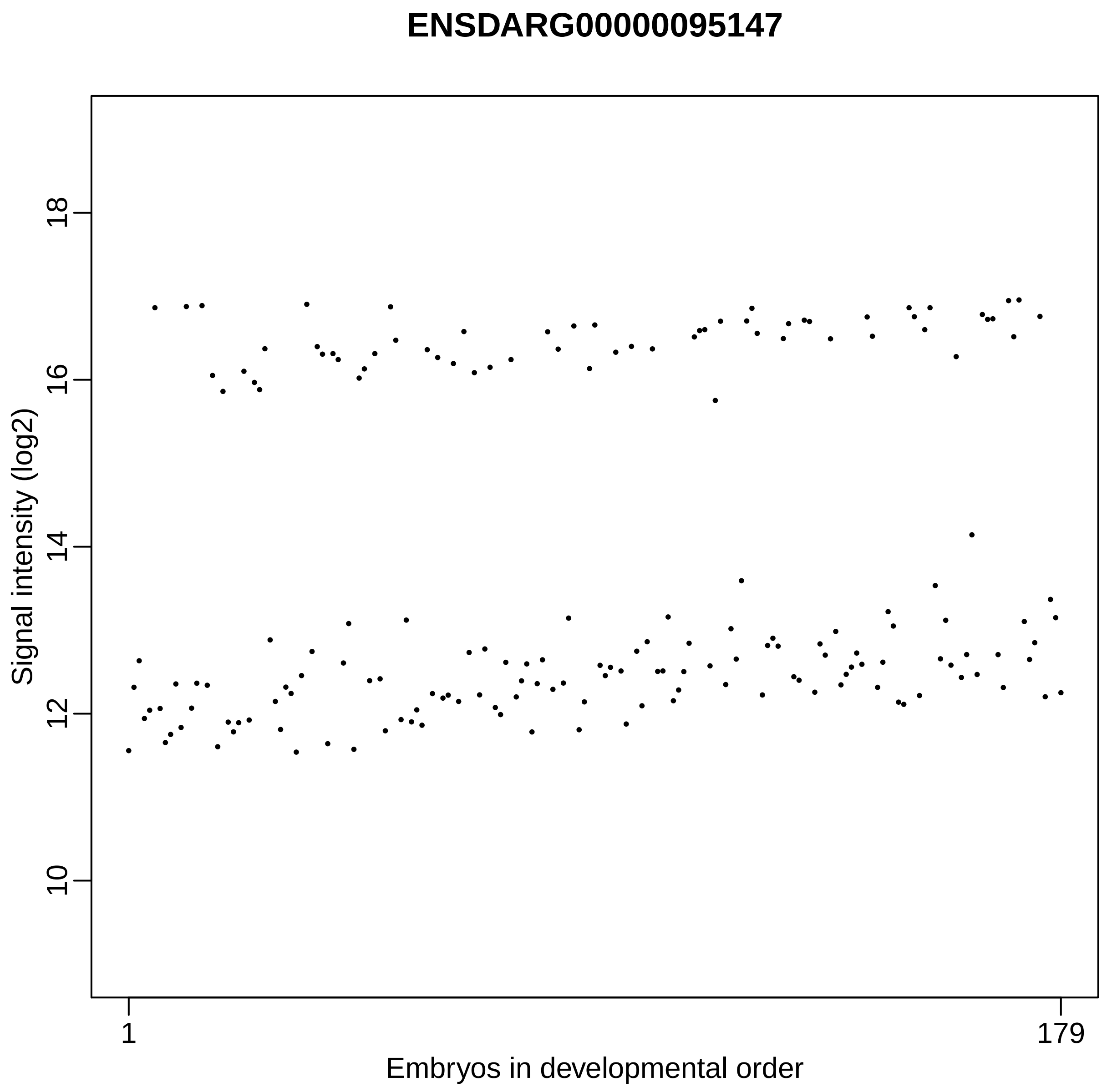
ENSDARG00000095147
Signal intensity (log2)
10
12
14
16
18
1
179
Embryos in developmental order

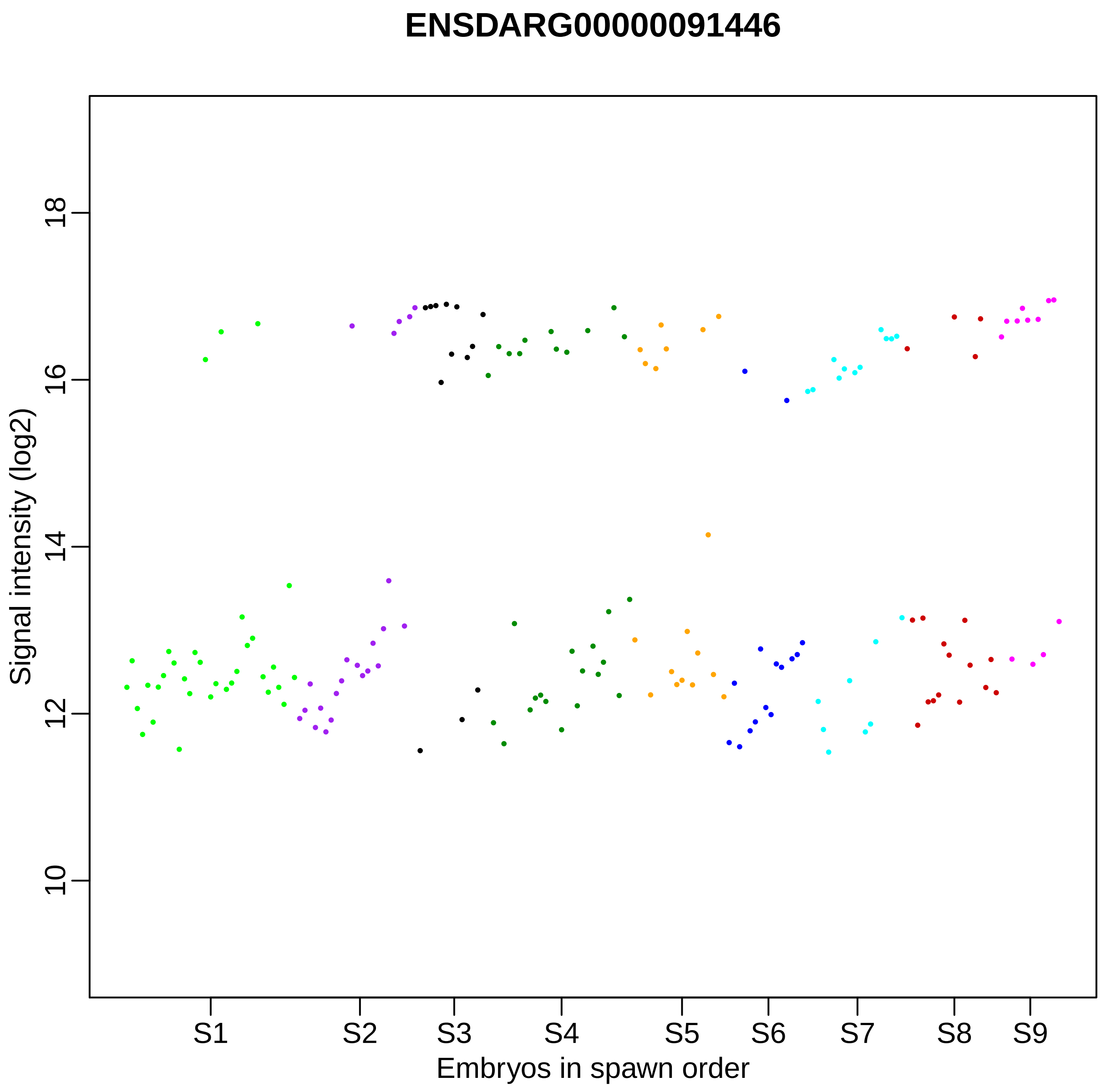
ENSDARG00000091446
Signal intensity (log2)
10
12
14
16
18
S1
S2
S3
S4
S5
S6
S7
S8
S9
Embryos in spawn order

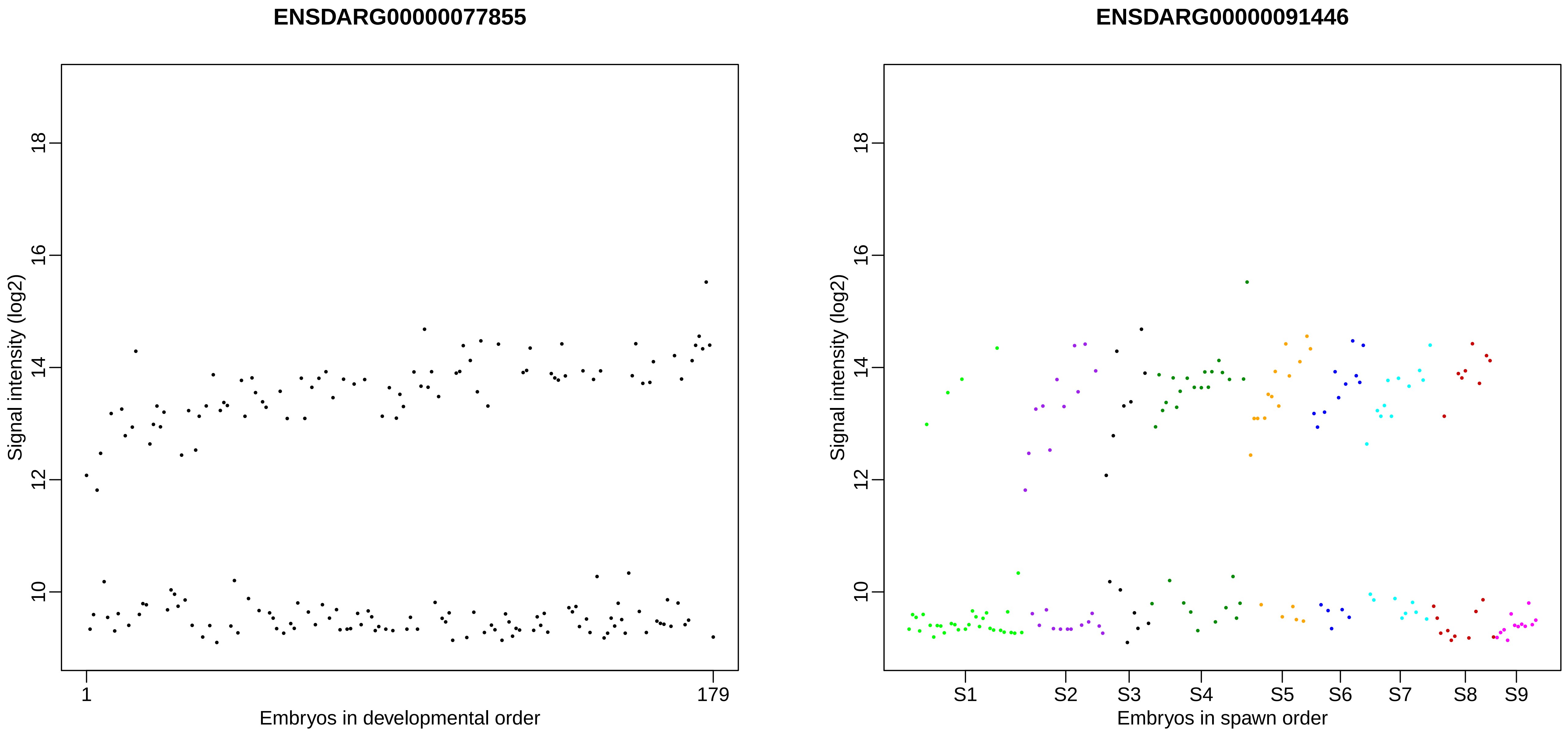
ENSDARG00000077855
Signal intensity (log2)
10
12
14
16
18
1
179
Embryos in developmental order
ENSDARG00000091446
Signal intensity (log2)
10
12
14
16
18
S1
S2
S3
S4
S5
S6
S7
S8
S9
Embryos in spawn order

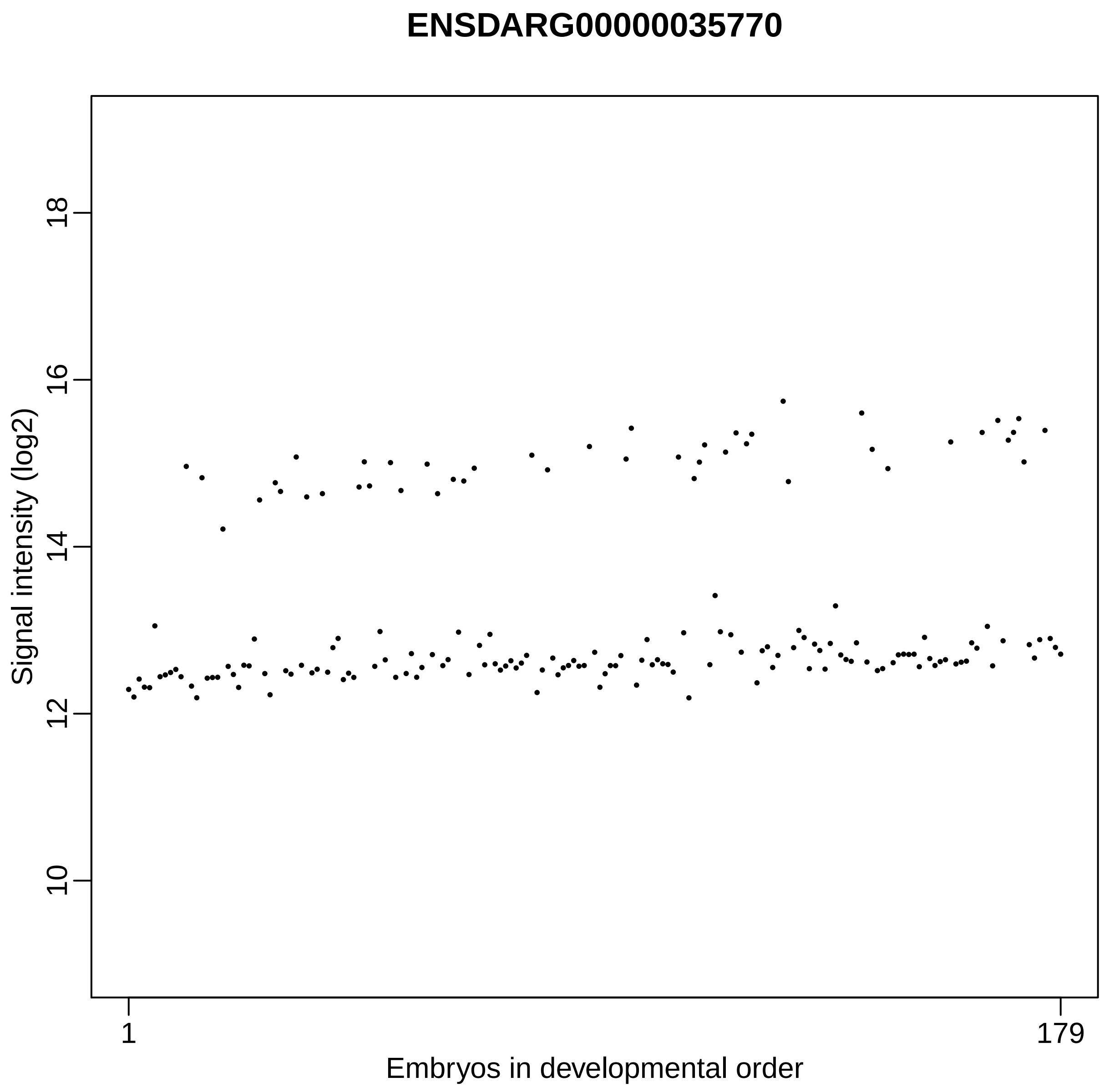
ENSDARG00000035770
Signal intensity (log2)
10
12
14
16
18
1
179
Embryos in developmental order

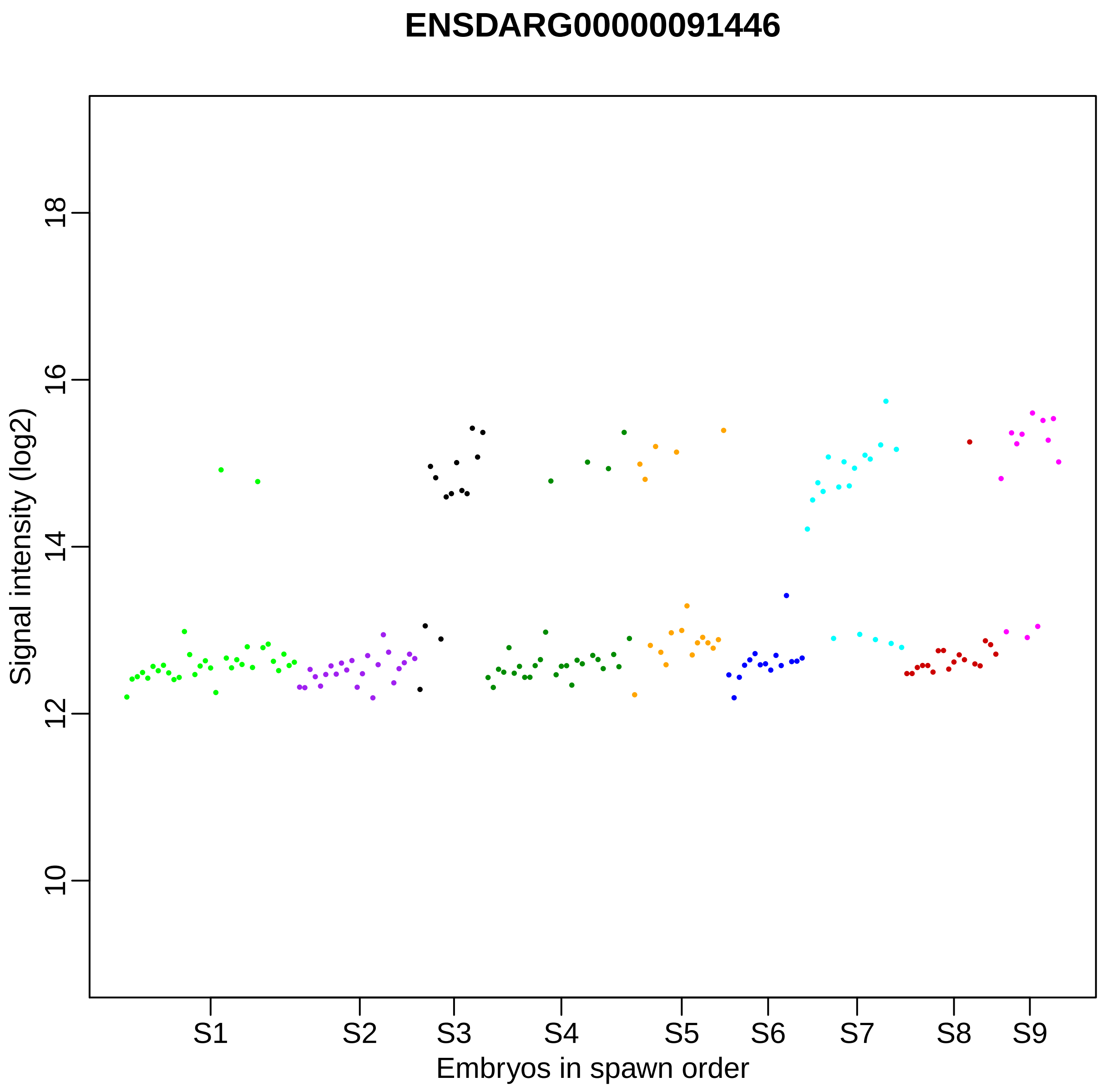
ENSDARG00000091446
Signal intensity (log2)
10
12
14
16
18
S1
S2
S3
S4
S5
S6
S7
S8
S9
Embryos in spawn order

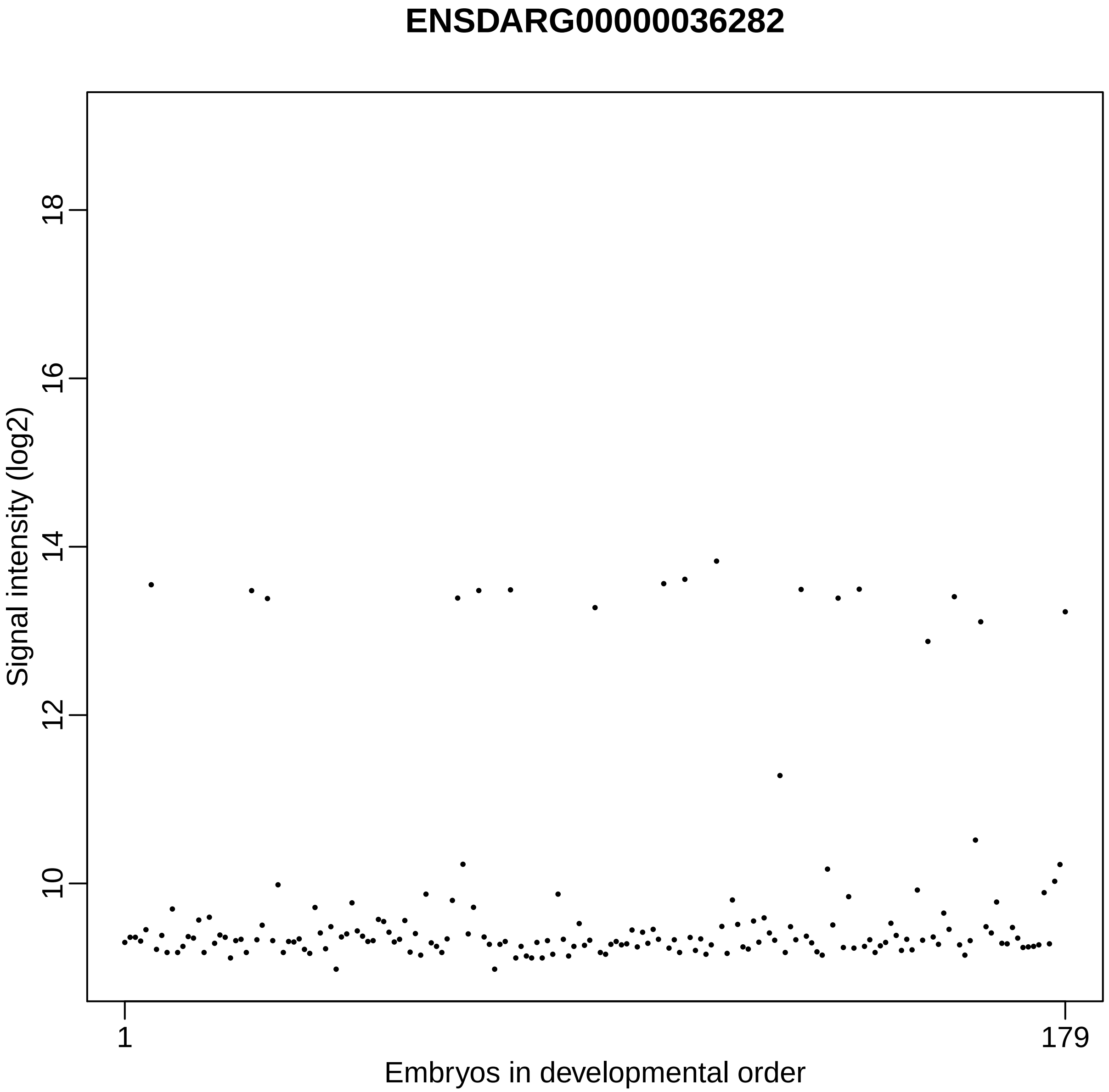
ENSDARG00000036282
Signal intensity (log2)
10
12
14
16
18
1
179
Embryos in developmental order

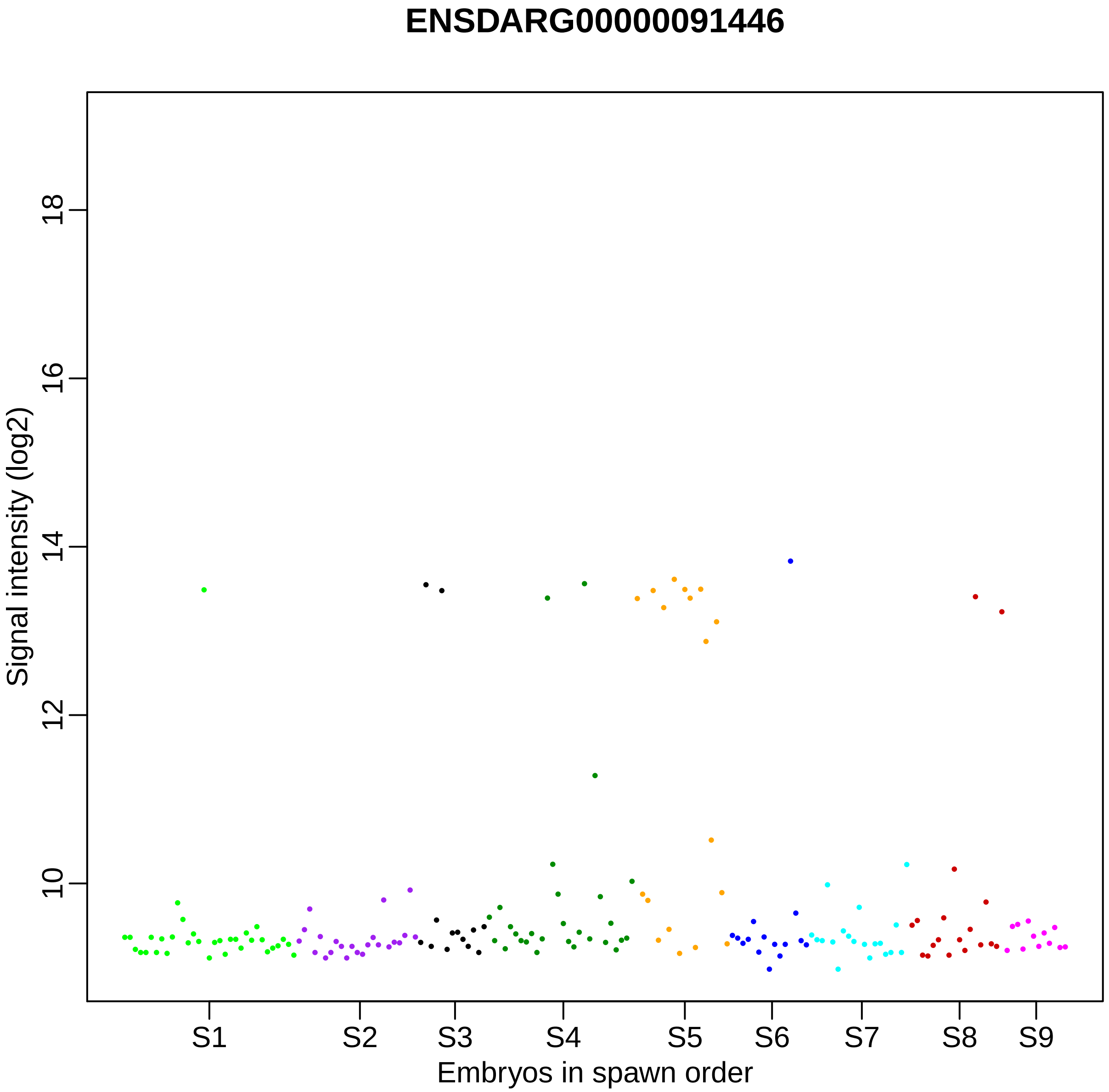
ENSDARG00000091446
Signal intensity (log2)
10
12
14
16
18
S1
S2
S3
S4
S5
S6
S7
S8
S9
Embryos in spawn order

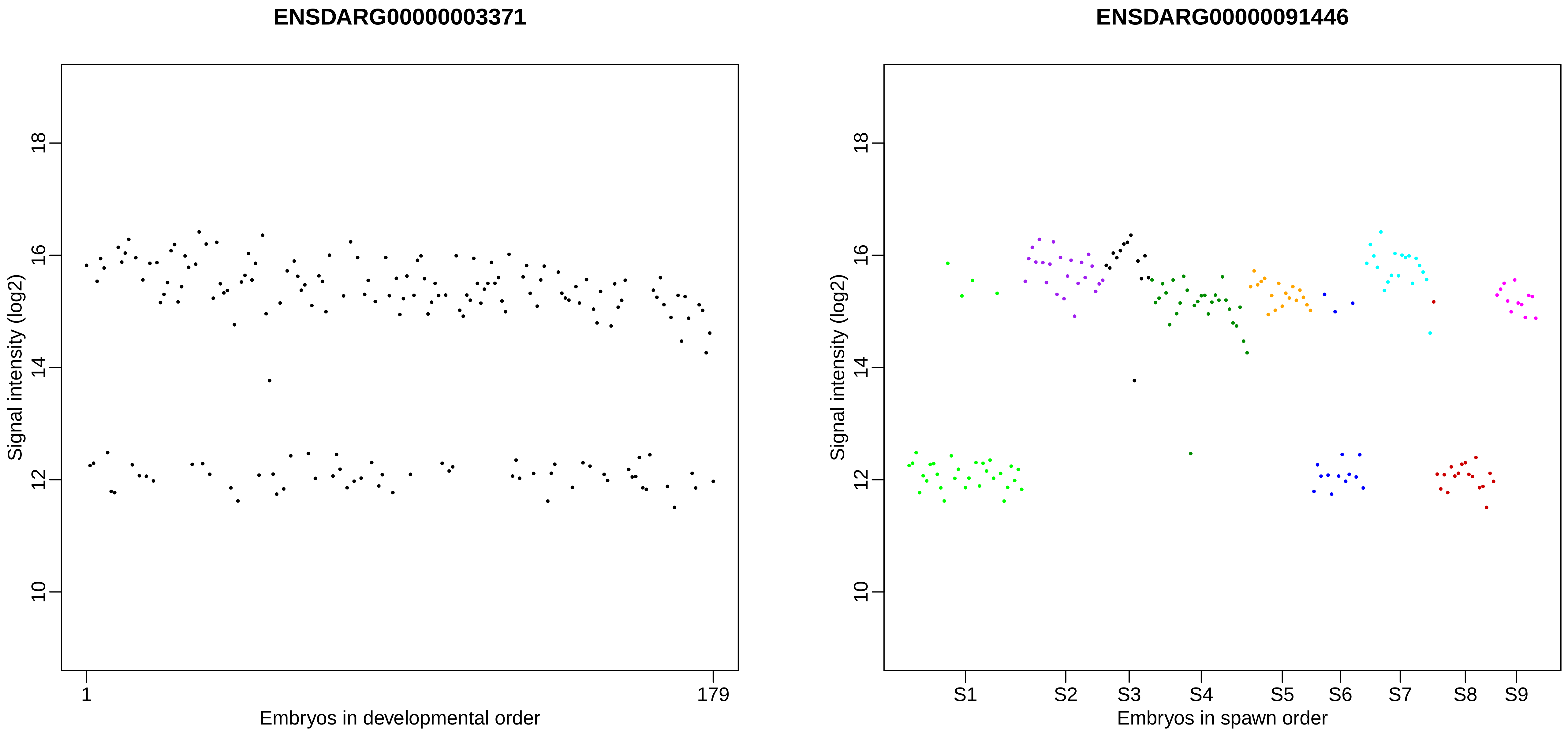

ENSDARG00000003371
Signal intensity (log2)
10
12
14
16
18
1
179
Embryos in developmental order
ENSDARG00000091446
Signal intensity (log2)
10
12
14
16
18
S1
S2
S3
S4
S5
S6
S7
S8
S9
Embryos in spawn order

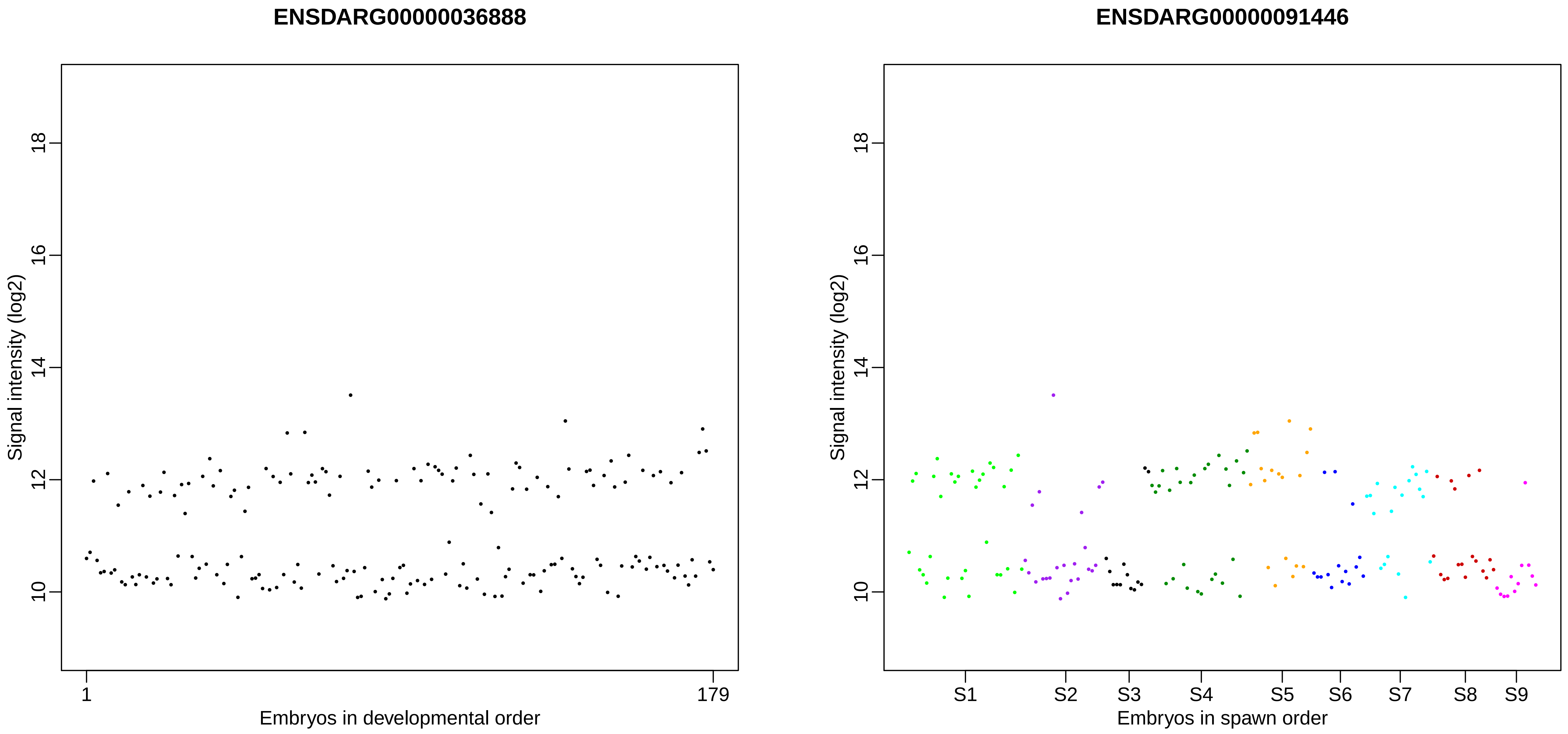
ENSDARG00000036888
Signal intensity (log2)
10
12
14
16
18
1
179
Embryos in developmental order
ENSDARG00000091446
Signal intensity (log2)
10
12
14
16
18
S1
S2
S3
S4
S5
S6
S7
S8
S9
Embryos in spawn order

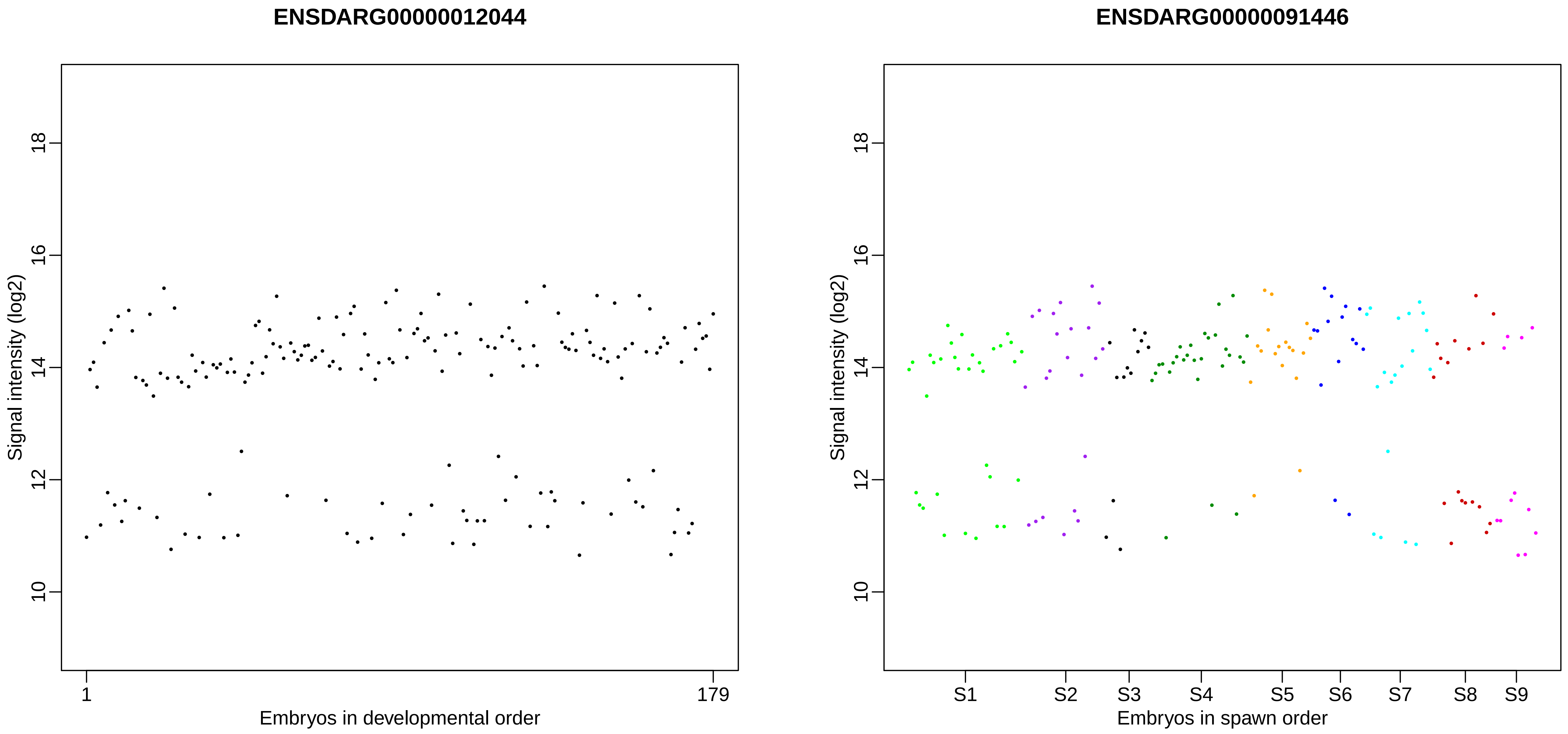

ENSDARG00000012044
Signal intensity (log2)
10
12
14
16
18
1
179
Embryos in developmental order
ENSDARG00000091446
Signal intensity (log2)
10
12
14
16
18
S1
S2
S3
S4
S5
S6
S7
S8
S9
Embryos in spawn order

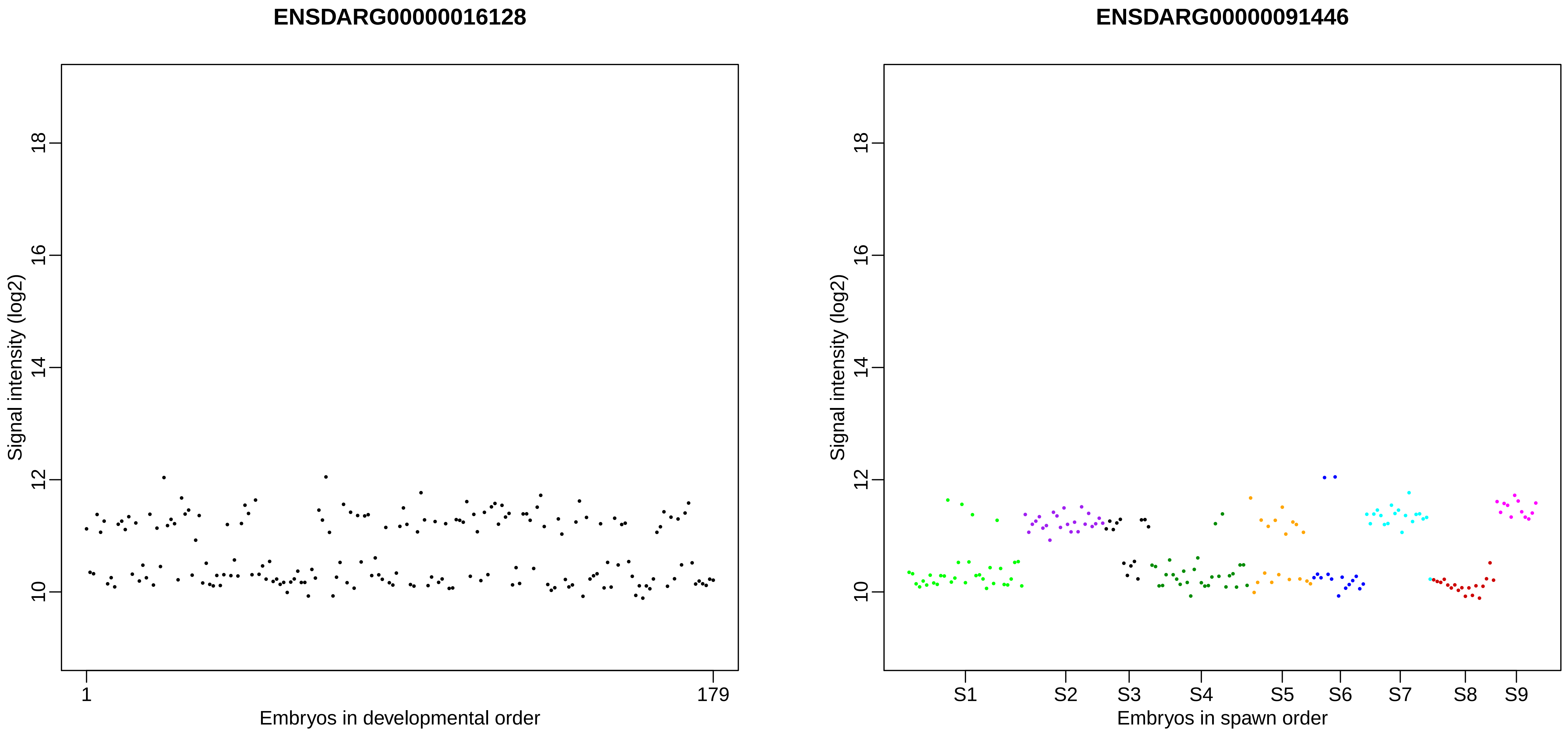
ENSDARG00000016128
Signal intensity (log2)
10
12
14
16
18
1
179
Embryos in developmental order
ENSDARG00000091446
Signal intensity (log2)
10
12
14
16
18
S1
S2
S3
S4
S5
S6
S7
S8
S9
Embryos in spawn order

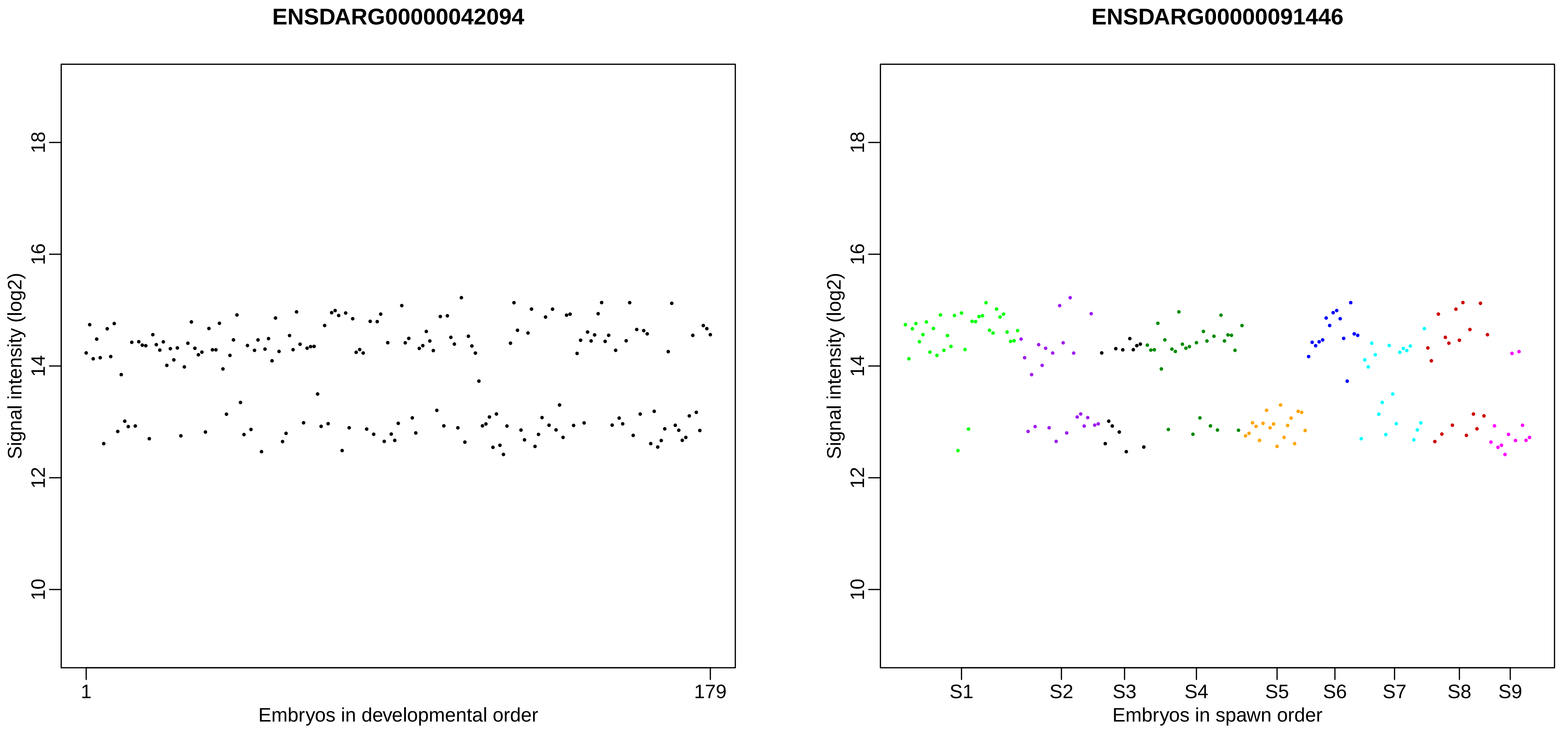
ENSDARG00000042094
Signal intensity (log2)
10
12
14
16
18
1
179
Embryos in developmental order
ENSDARG00000091446
Signal intensity (log2)
10
12
14
16
18
S1
S2
S3
S4
S5
S6
S7
S8
S9
Embryos in spawn order

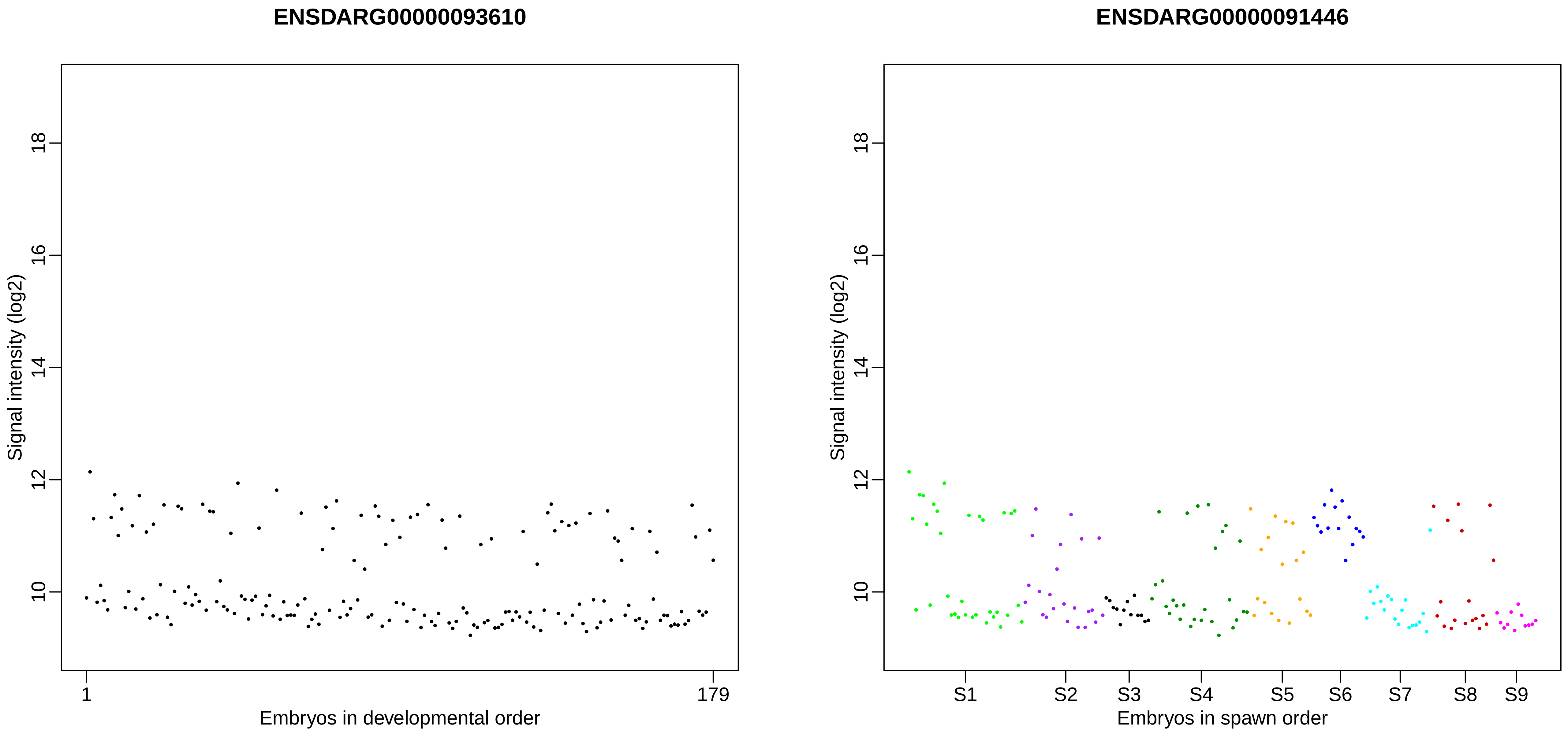
ENSDARG00000093610
Signal intensity (log2)
10
12
14
16
18
1
179
Embryos in developmental order
ENSDARG00000091446
Signal intensity (log2)
10
12
14
16
18
S1
S2
S3
S4
S5
S6
S7
S8
S9
Embryos in spawn order

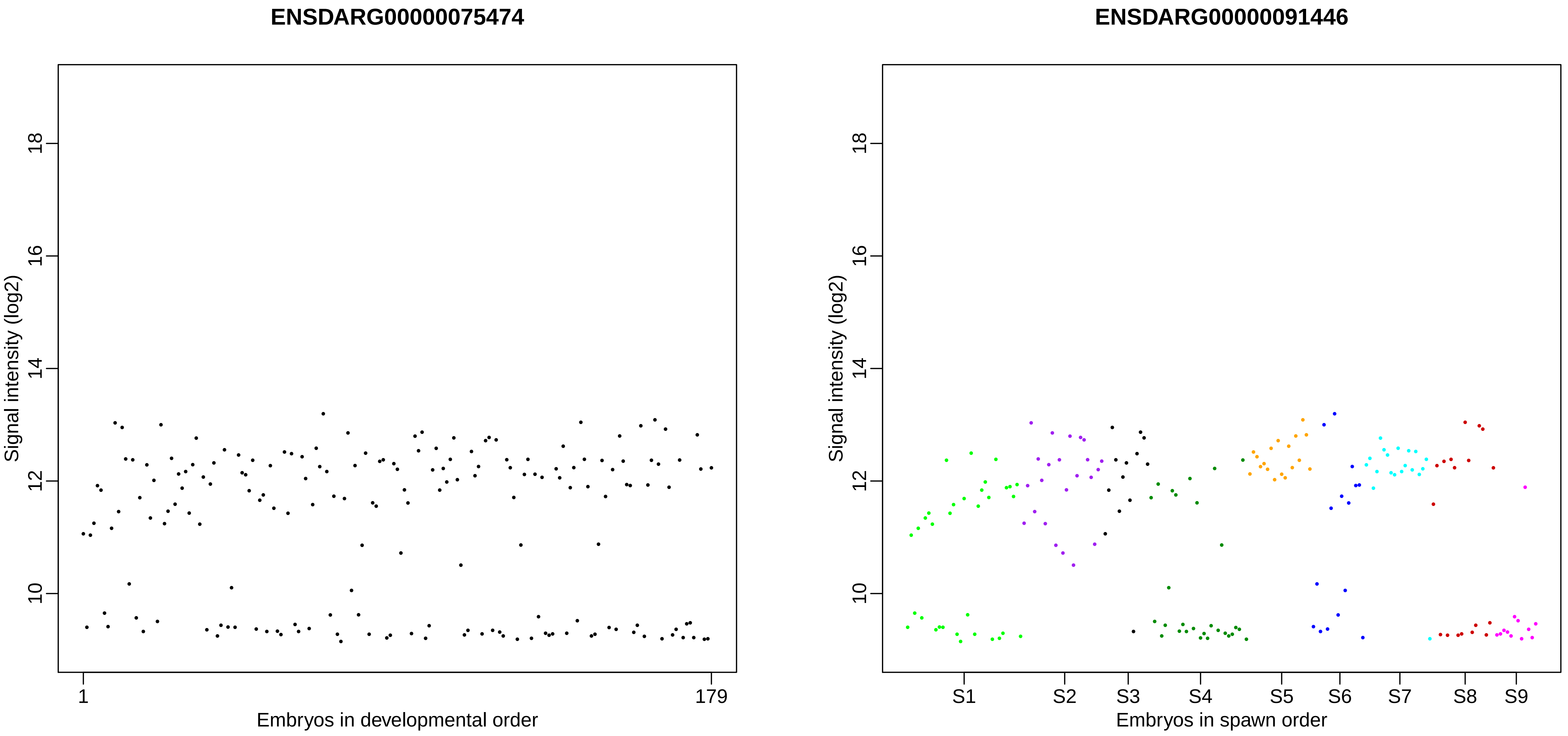
ENSDARG00000075474
ENSDARG00000091446
Signal intensity (log2)
18
16
14
12
10
1
179
Embryos in developmental order
S1
S2
S3
S4
S5
S6
S7
S8
S9
Embryos in spawn order

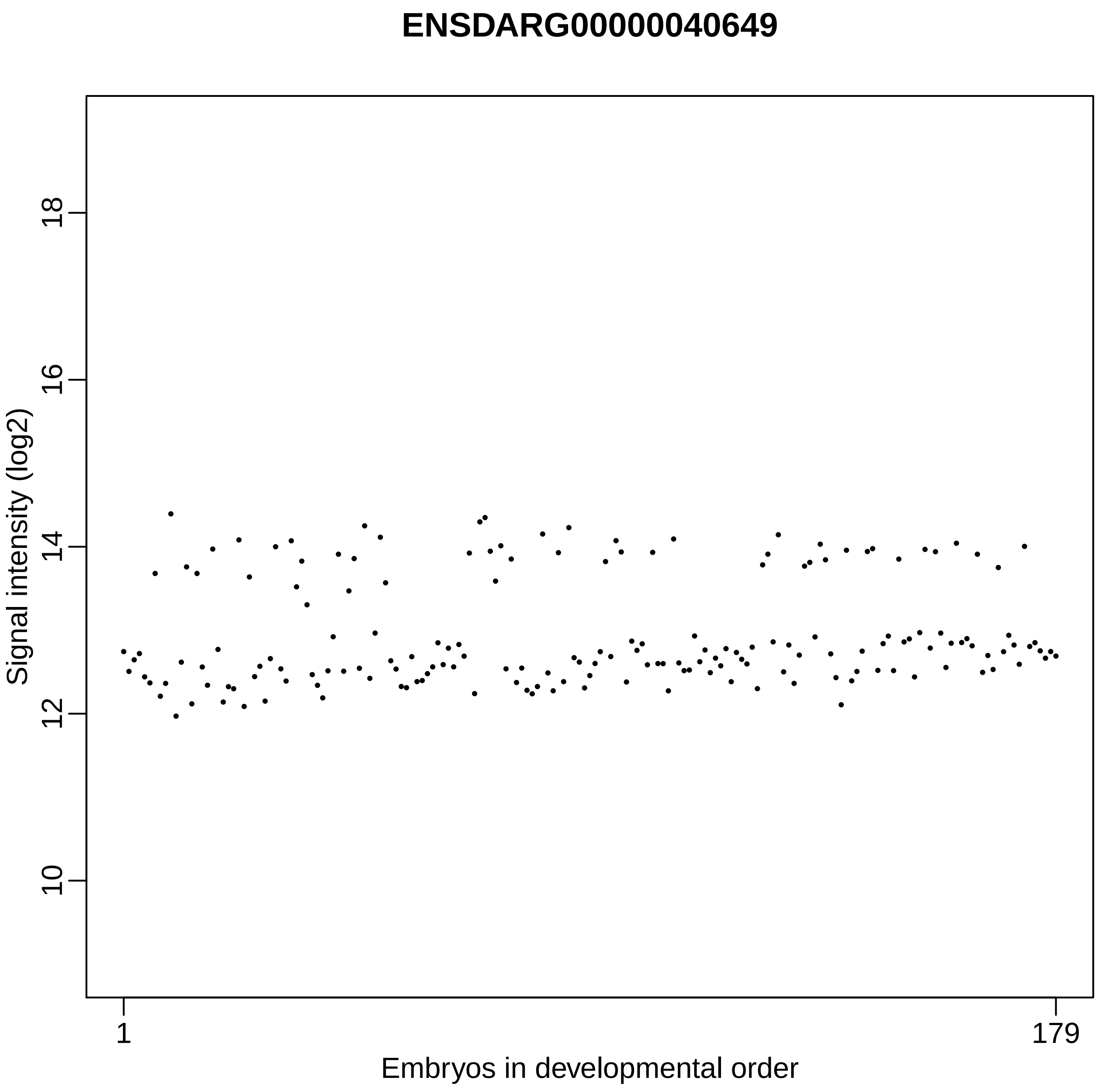
ENSDARG00000040649
Signal intensity (log2)
10
12
14
16
18
1
179
Embryos in developmental order

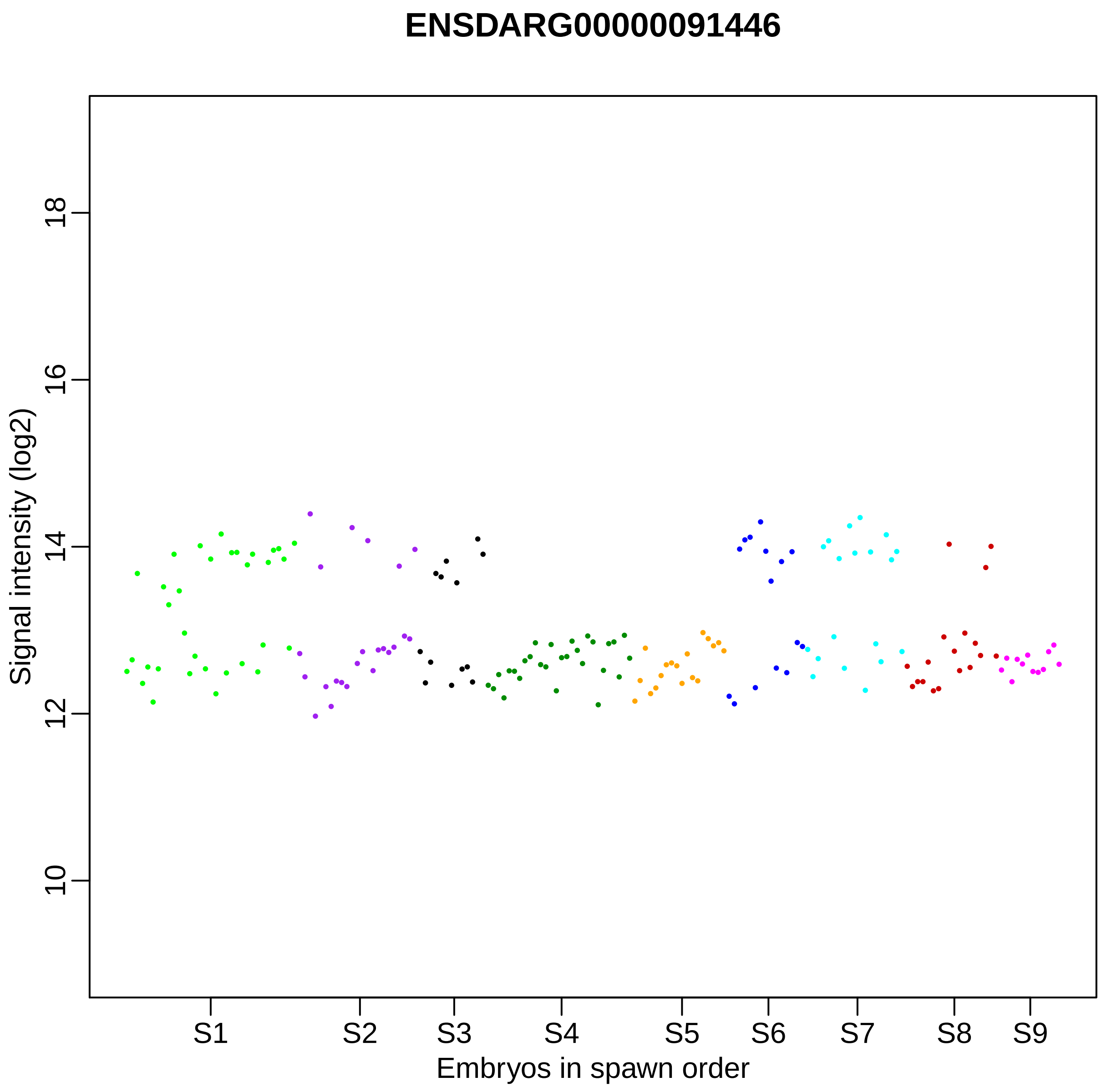
ENSDARG00000091446
Signal intensity (log2)
10
12
14
16
18
S1
S2
S3
S4
S5
S6
S7
S8
S9
Embryos in spawn order

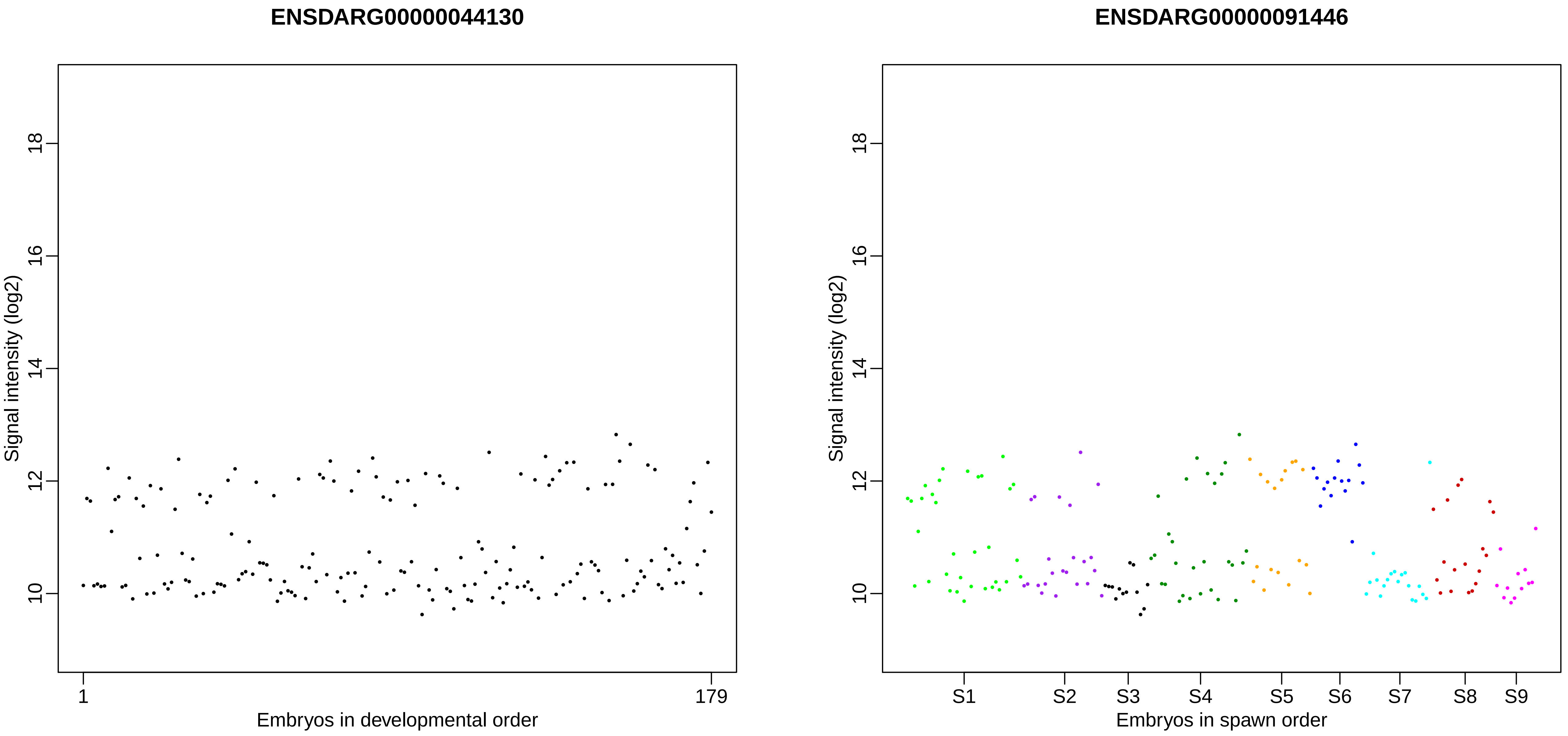

ENSDARG00000044130
Signal intensity (log2)
10
12
14
16
18
1
179
Embryos in developmental order
ENSDARG00000091446
Signal intensity (log2)
10
12
14
16
18
S1
S2
S3
S4
S5
S6
S7
S8
S9
Embryos in spawn order

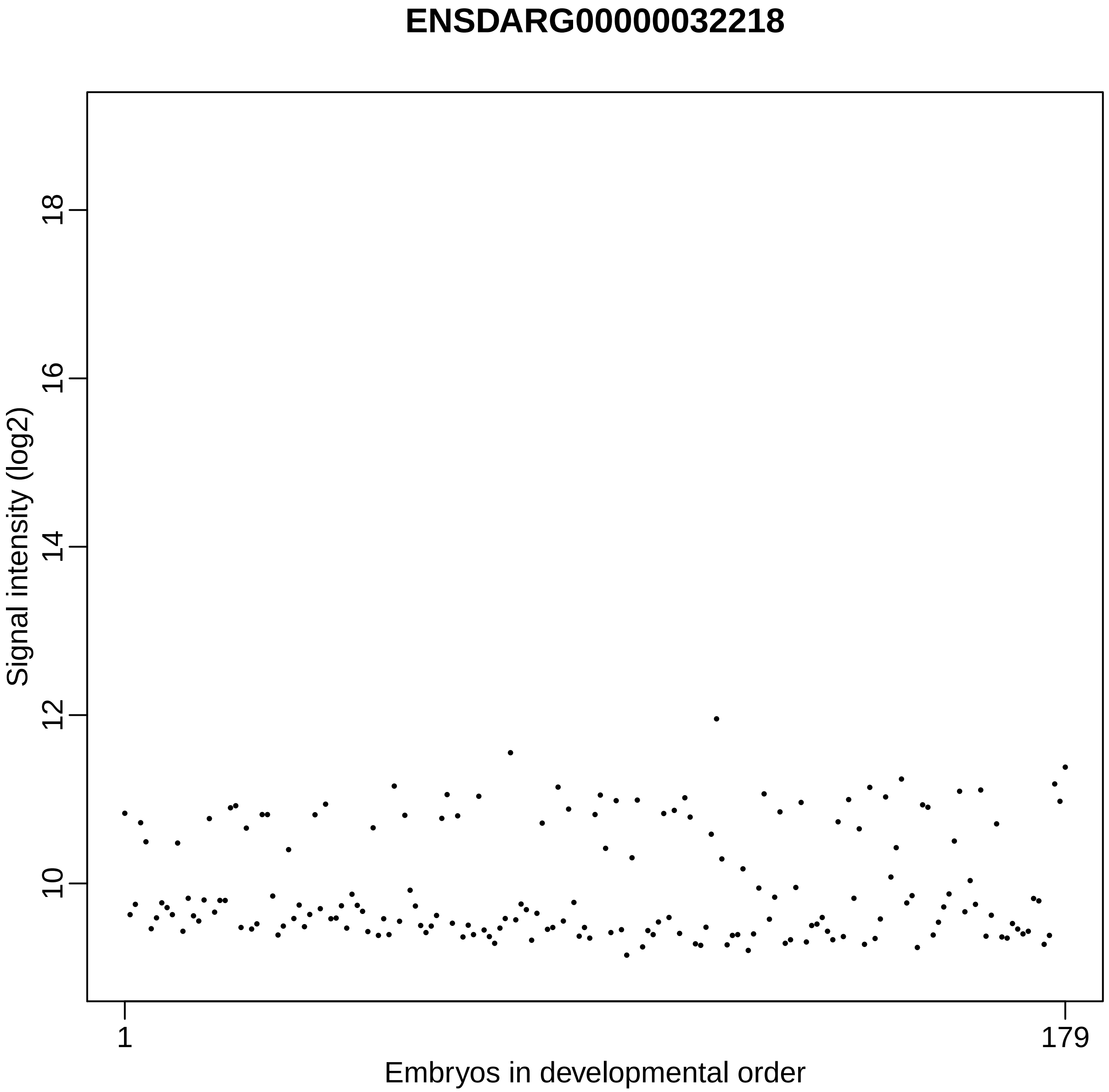
ENSDARG00000032218
Signal intensity (log2)
18
16
14
12
10
1
179
Embryos in developmental order

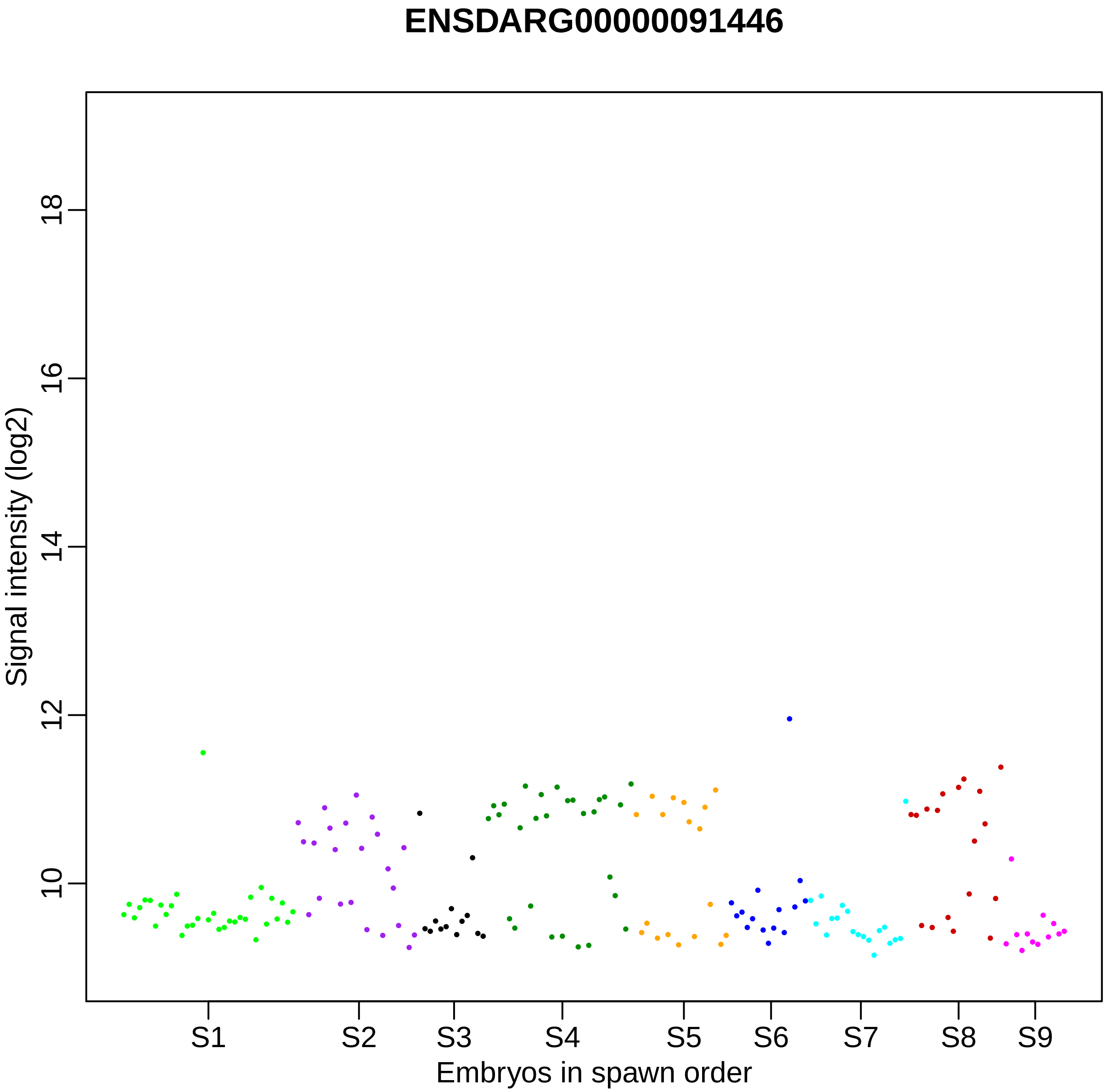
ENSDARG00000091446
Signal intensity (log2)
18
16
14
12
10
S1
S2
S3
S4
S5
S6
S7
S8
S9
Embryos in spawn order

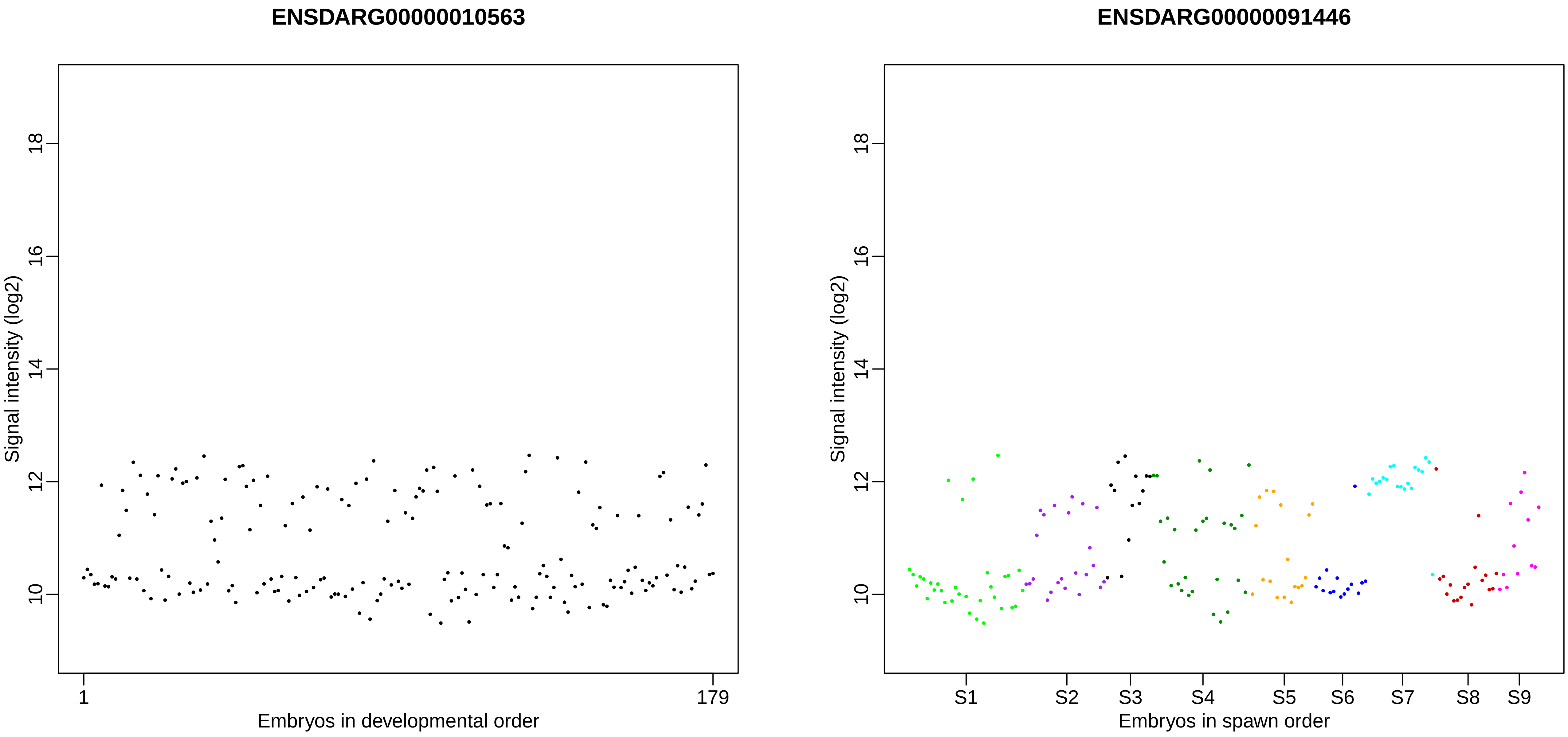
ENSDARG00000010563
Signal intensity (log2)
10
12
14
16
18
1
179
Embryos in developmental order
ENSDARG00000091446
Signal intensity (log2)
10
12
14
16
18
S1
S2
S3
S4
S5
S6
S7
S8
S9
Embryos in spawn order

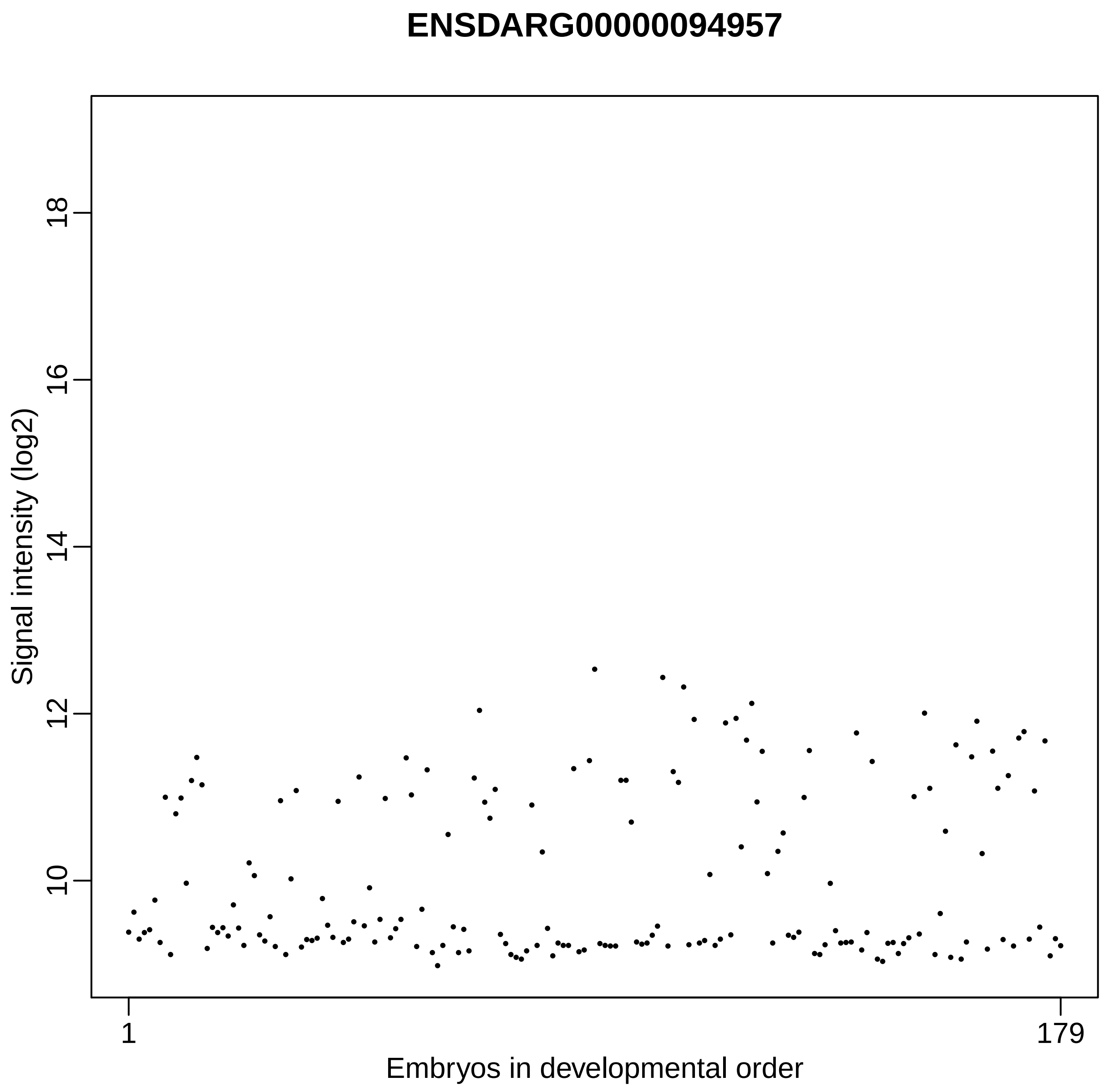
ENSDARG00000094957
Signal intensity (log2)
10
12
14
16
18
1
179
Embryos in developmental order

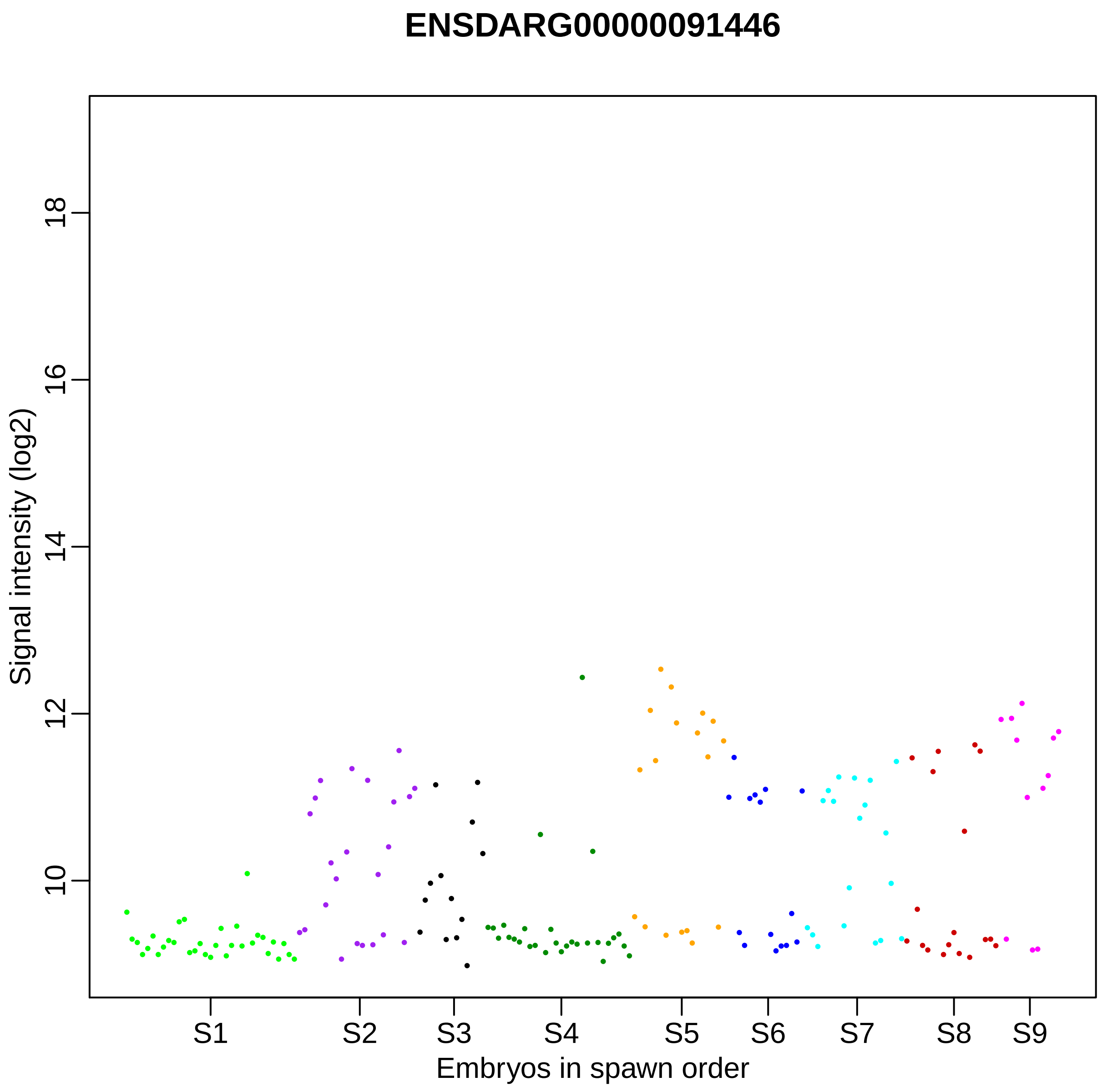
ENSDARG00000091446
Signal intensity (log2)
10
12
14
16
18
S1
S2
S3
S4
S5
S6
S7
S8
S9
Embryos in spawn order

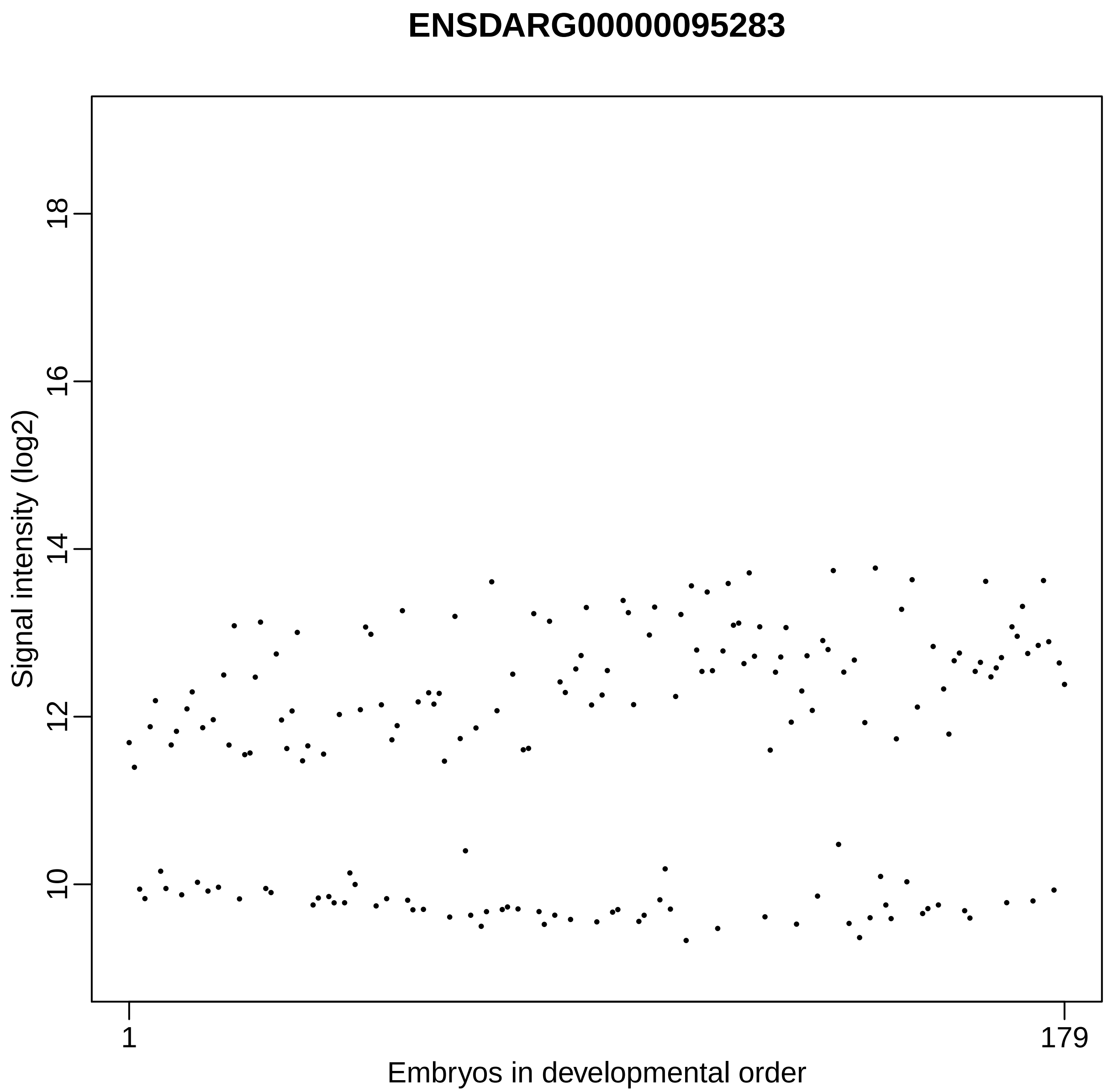
ENSDARG00000095283
Signal intensity (log2)
10
12
14
16
18
1
179
Embryos in developmental order

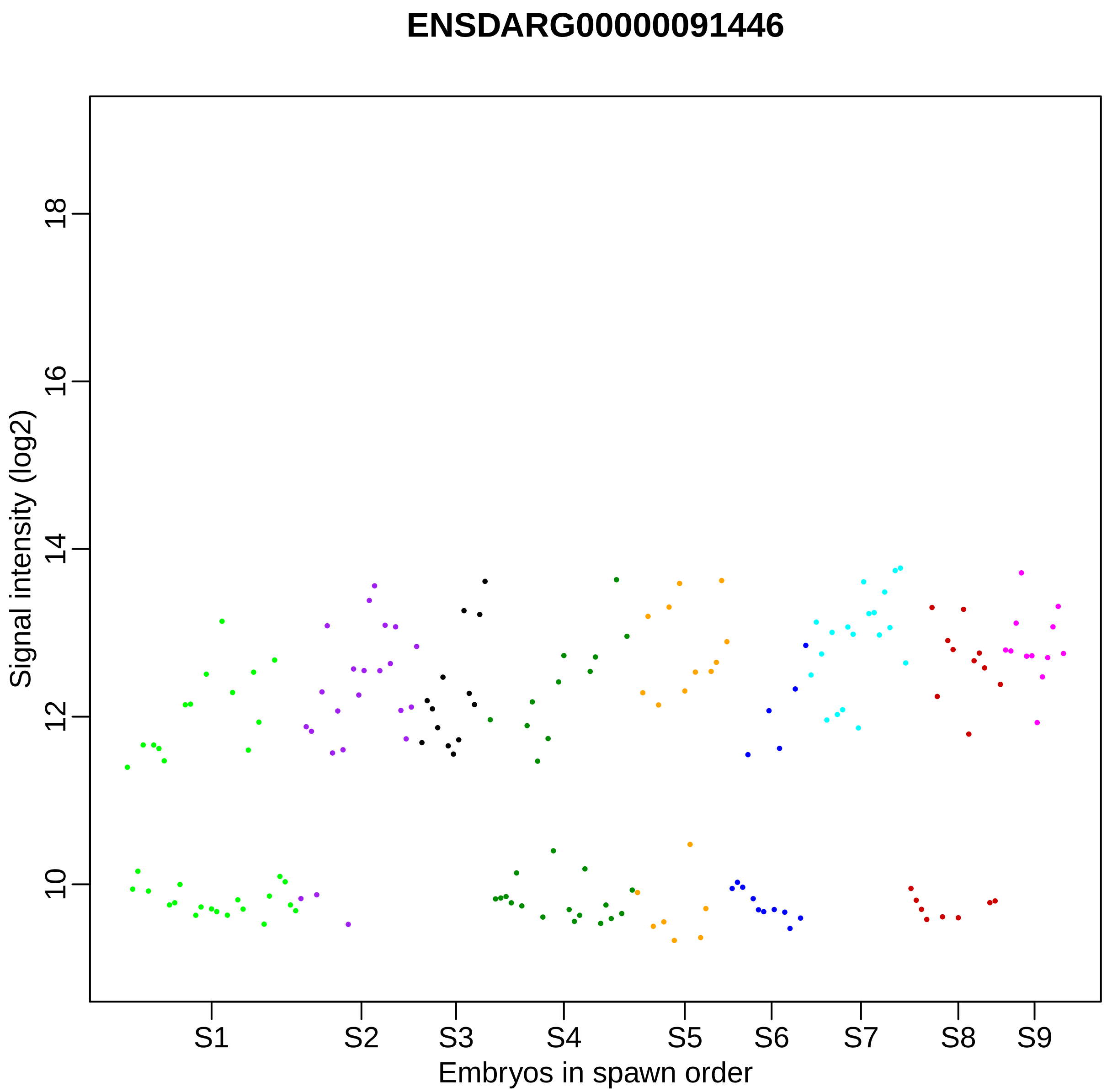
ENSDARG00000091446
Signal intensity (log2)
10
12
14
16
18
S1
S2
S3
S4
S5
S6
S7
S8
S9
Embryos in spawn order

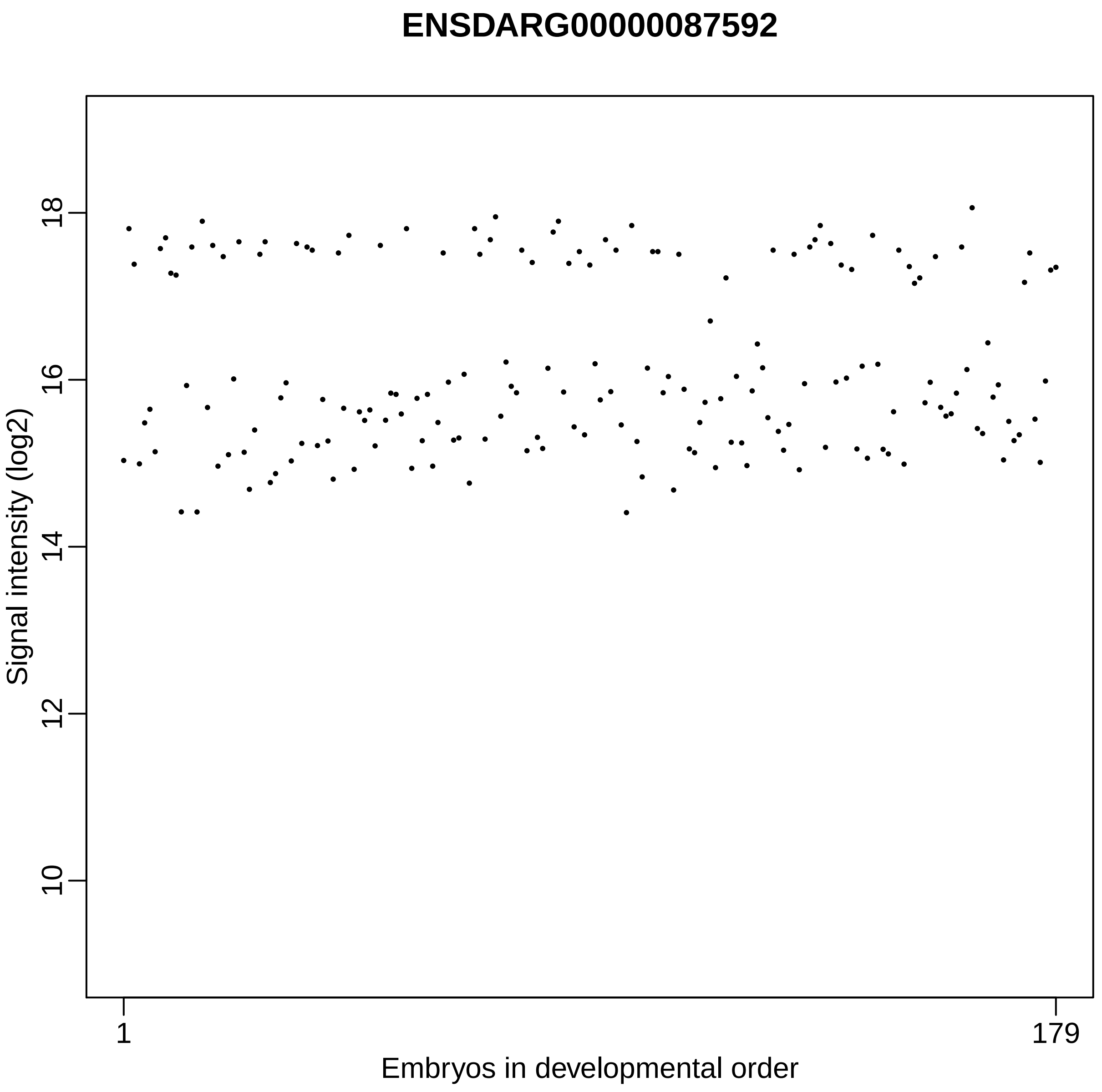

ENSDARG00000087592
Signal intensity (log2)
10
12
14
16
18
1
179
Embryos in developmental order

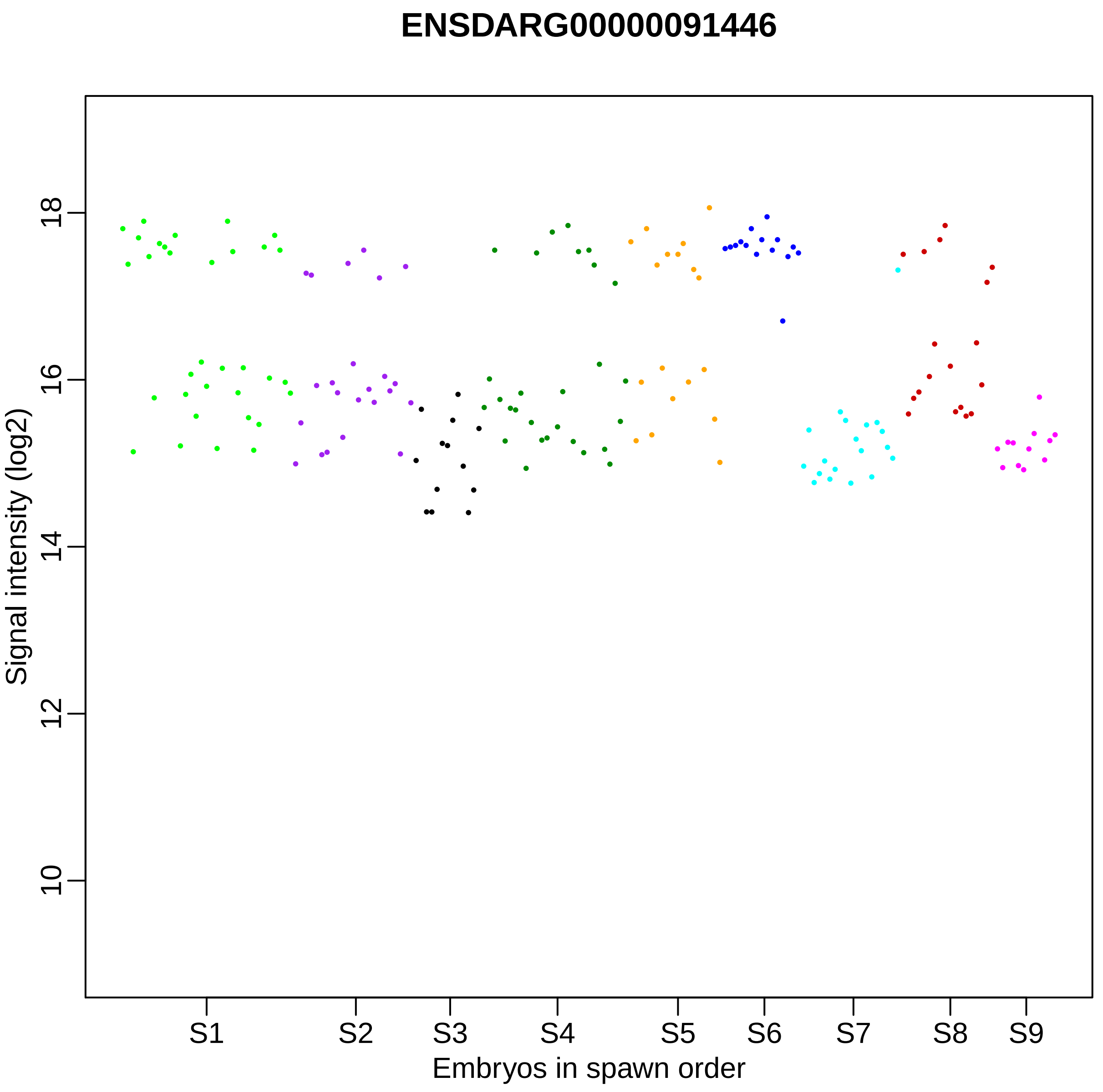

ENSDARG00000091446
Signal intensity (log2)
10
12
14
16
18
S1
S2
S3
S4
S5
S6
S7
S8
S9
Embryos in spawn order

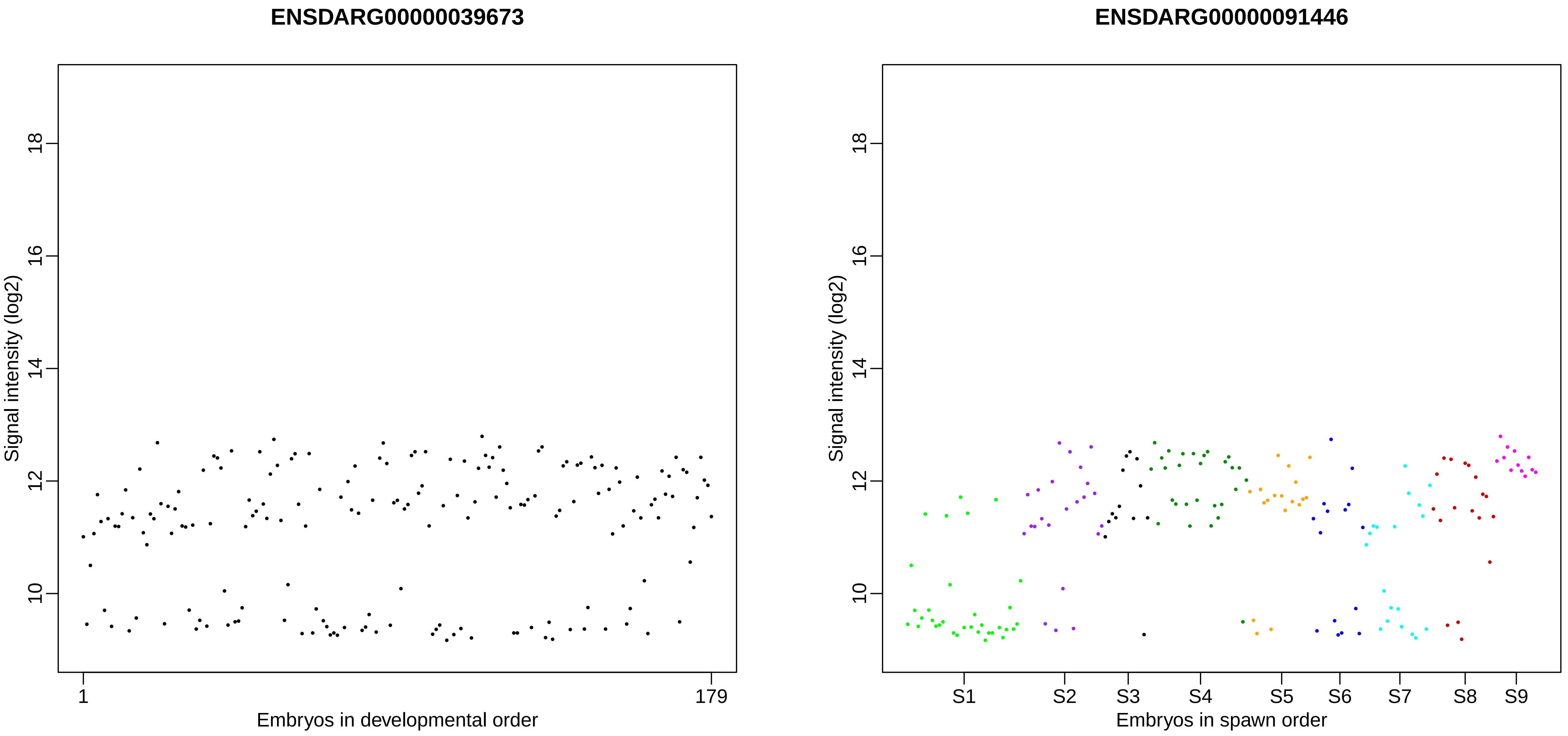

ENSDARG00000039673
Signal intensity (log2)
18
16
14
12
10
1
179
Embryos in developmental order
ENSDARG00000091446
Signal intensity (log2)
18
16
14
12
10
S1
S2
S3
S4
S5
S6
S7
S8
S9
Embryos in spawn order

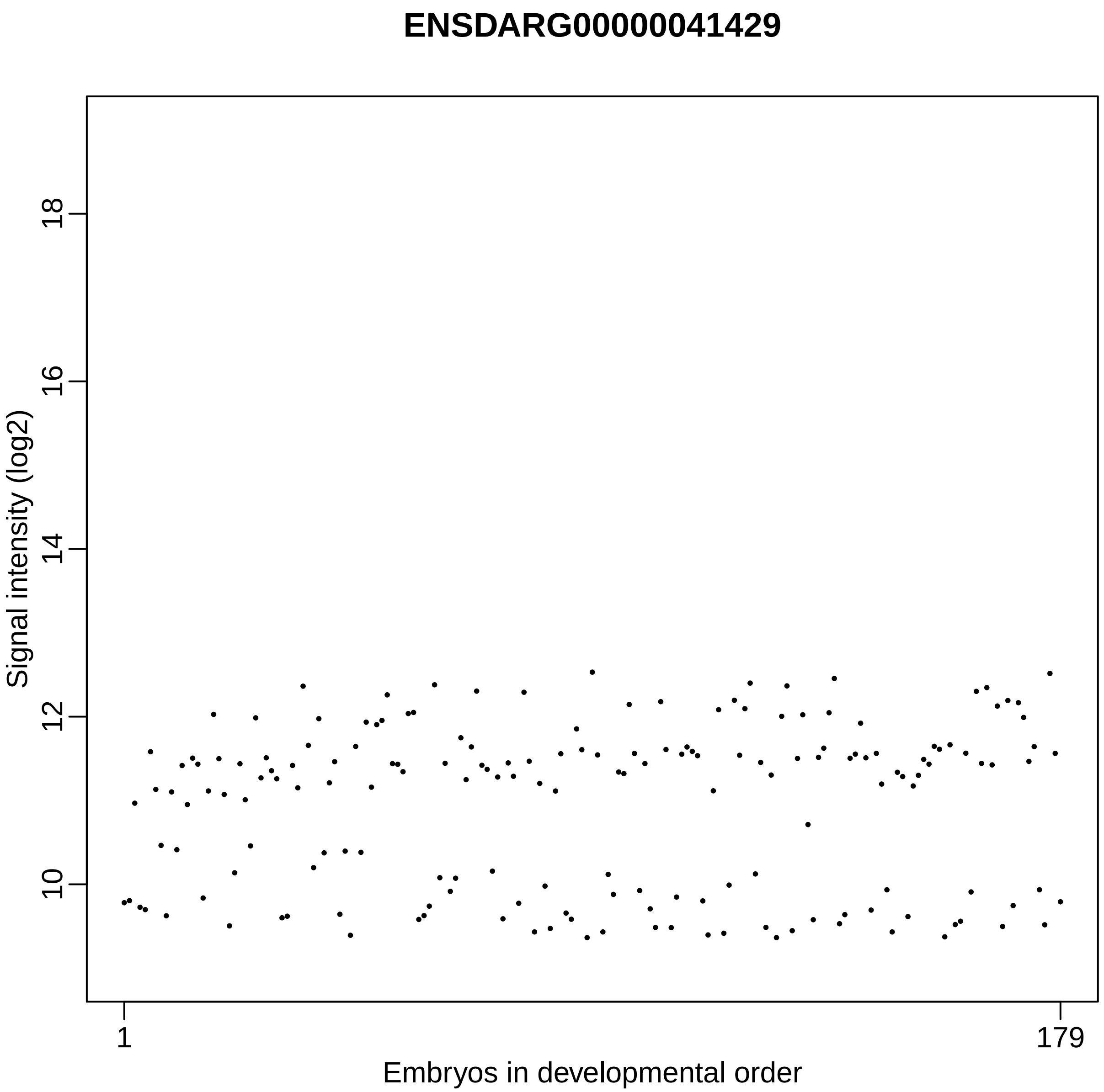

ENSDARG00000041429
Signal intensity (log2)
18
16
14
12
10
1
179
Embryos in developmental order

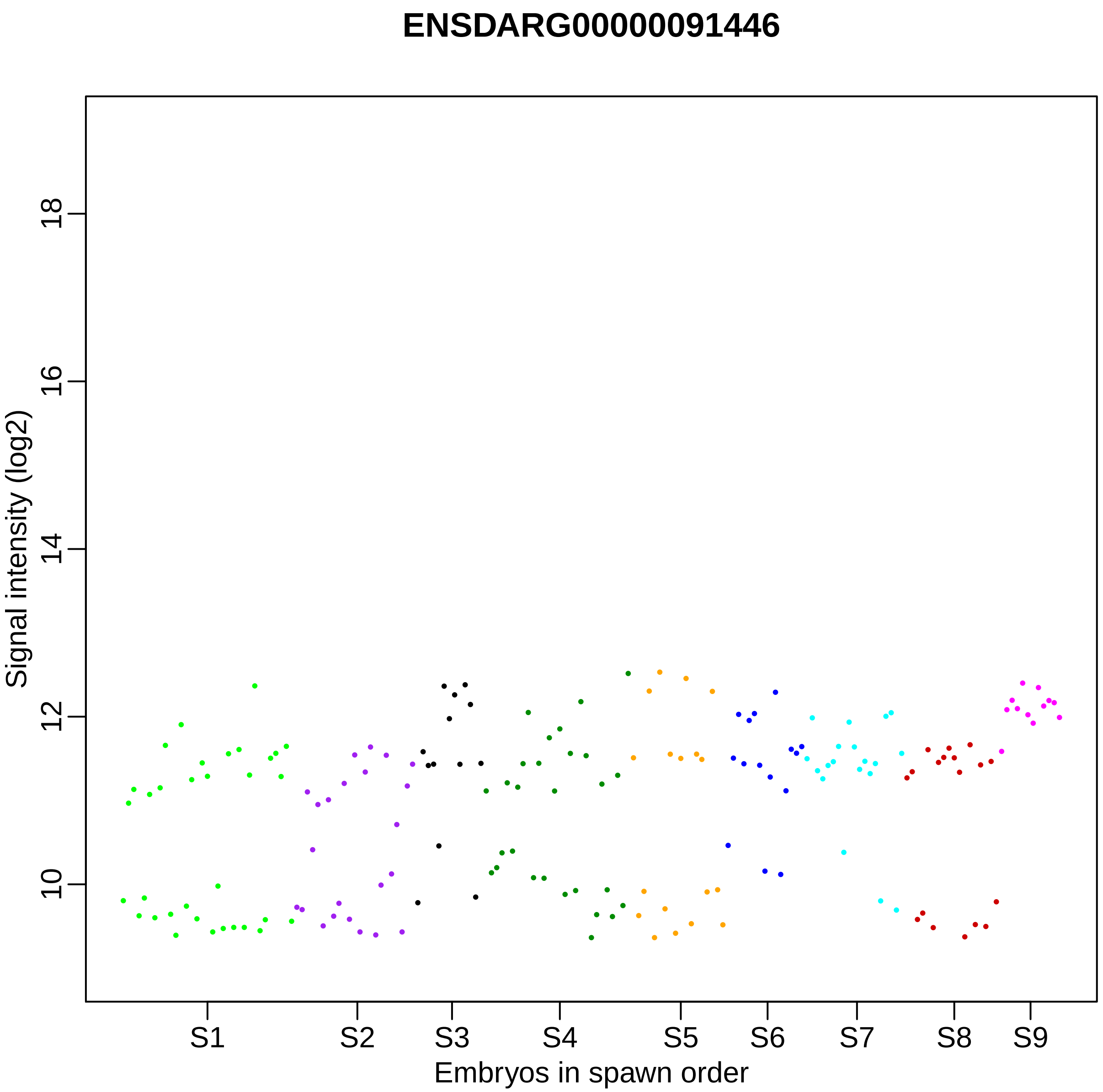

ENSDARG00000091446
Signal intensity (log2)
18
16
14
12
10
S1
S2
S3
S4
S5
S6
S7
S8
S9
Embryos in spawn order

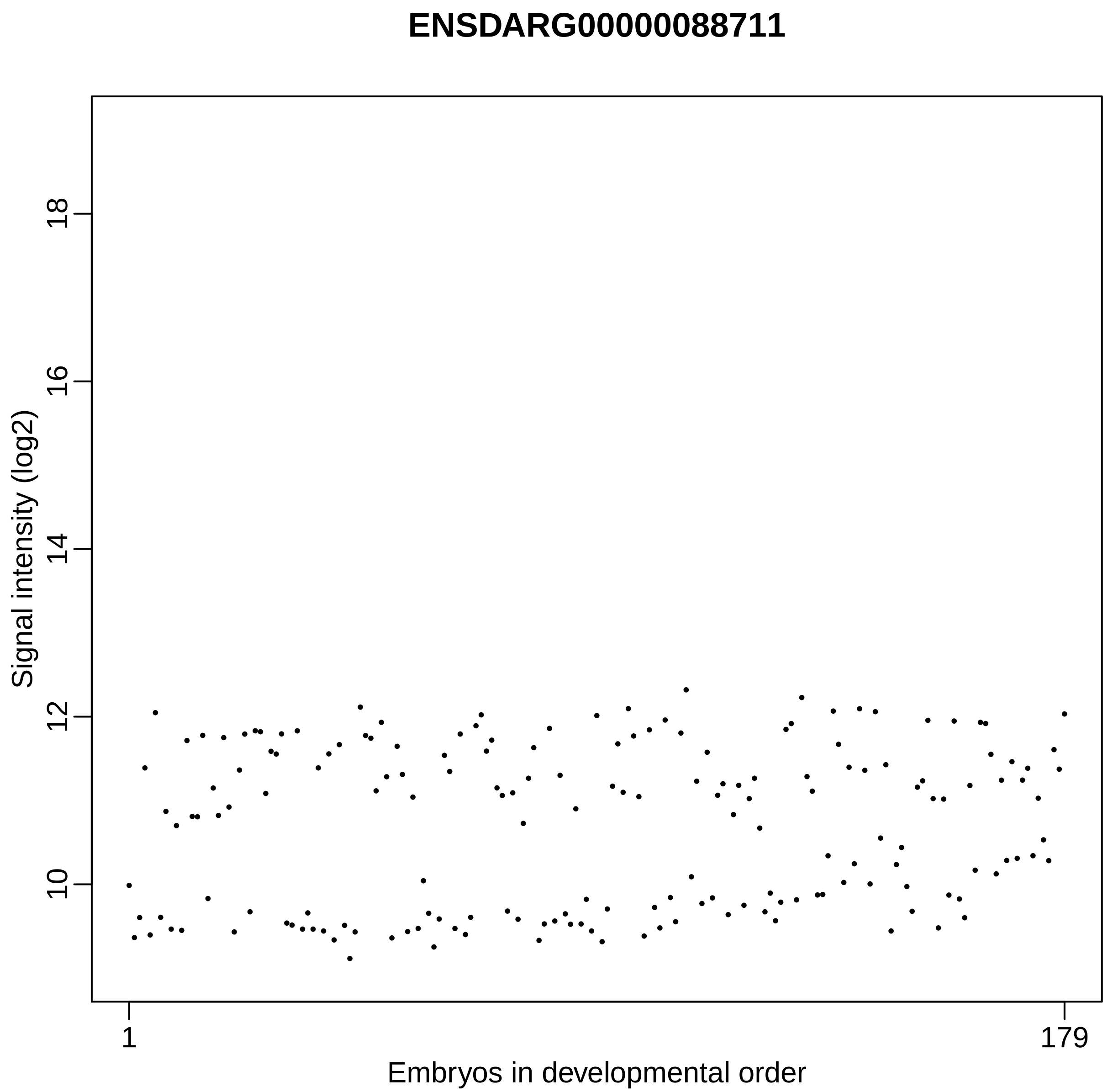
ENSDARG00000088711
Signal intensity (log2)
18
16
14
12
10
1
179
Embryos in developmental order

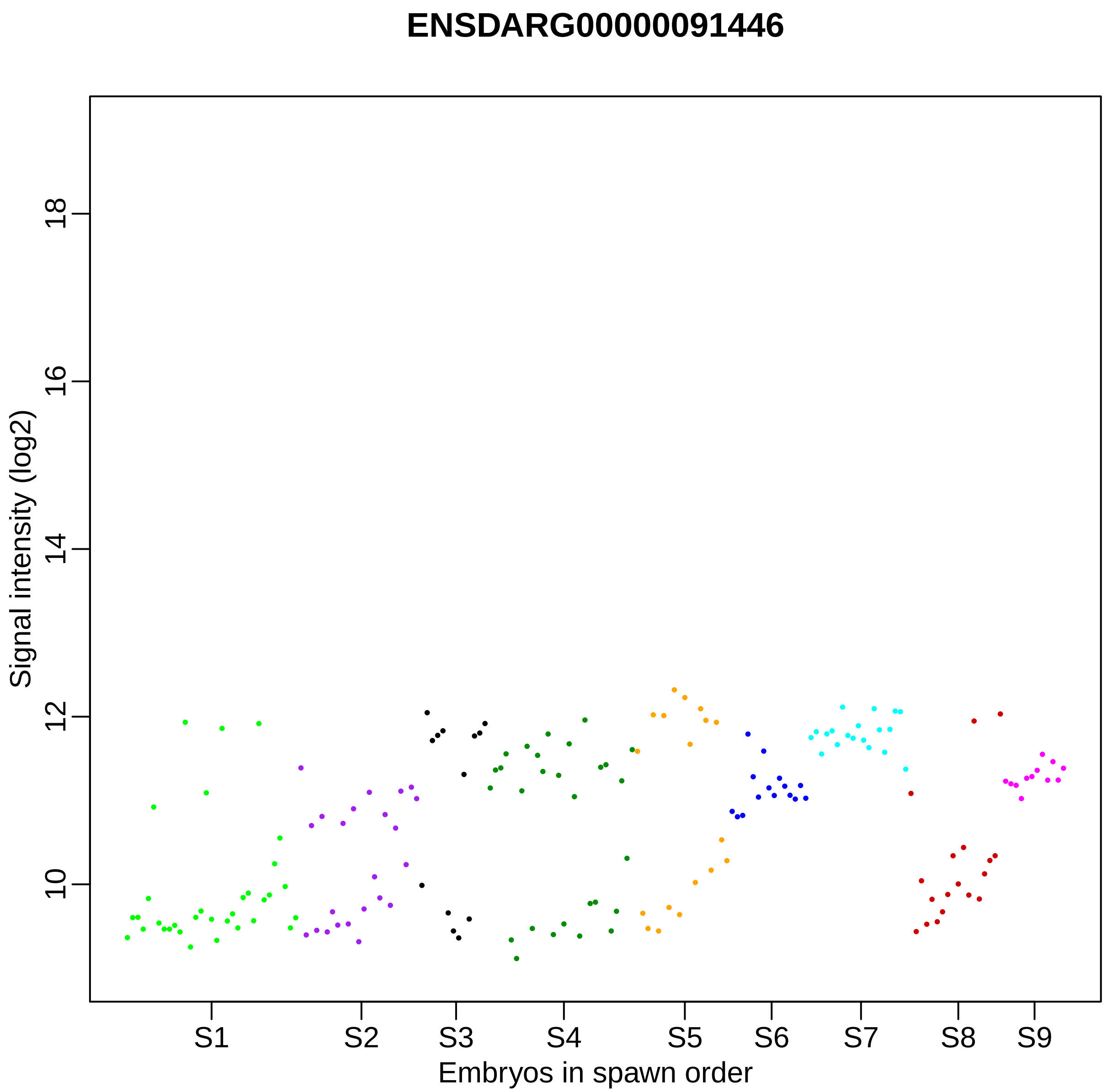
ENSDARG00000091446
Signal intensity (log2)
18
16
14
12
10
S1
S2
S3
S4
S5
S6
S7
S8
S9
Embryos in spawn order

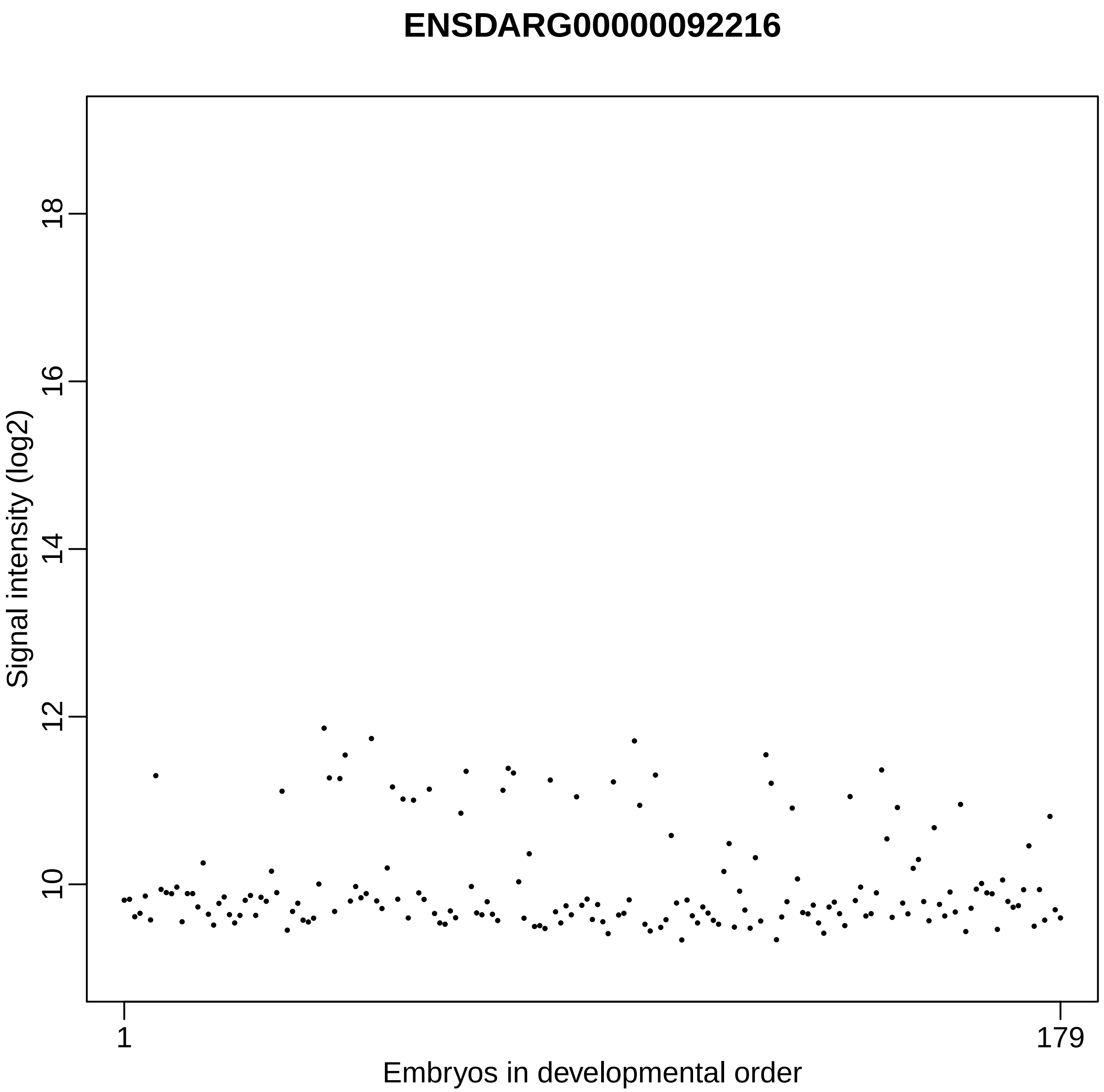
ENSDARG00000092216
Signal intensity (log2)
10
12
14
16
18
1
179
Embryos in developmental order

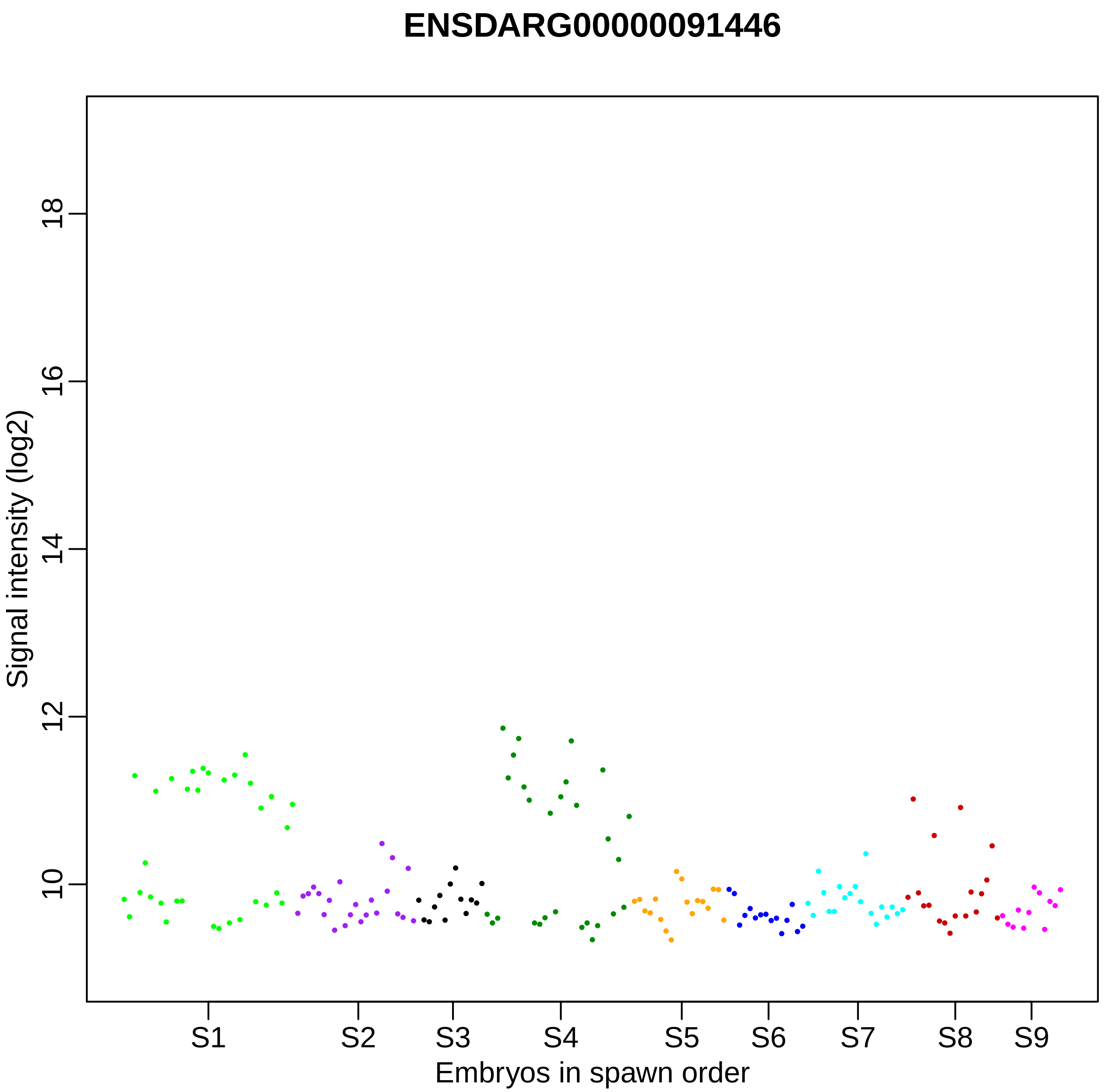
ENSDARG00000091446
Signal intensity (log2)
10
12
14
16
18
S1
S2
S3
S4
S5
S6
S7
S8
S9
Embryos in spawn order

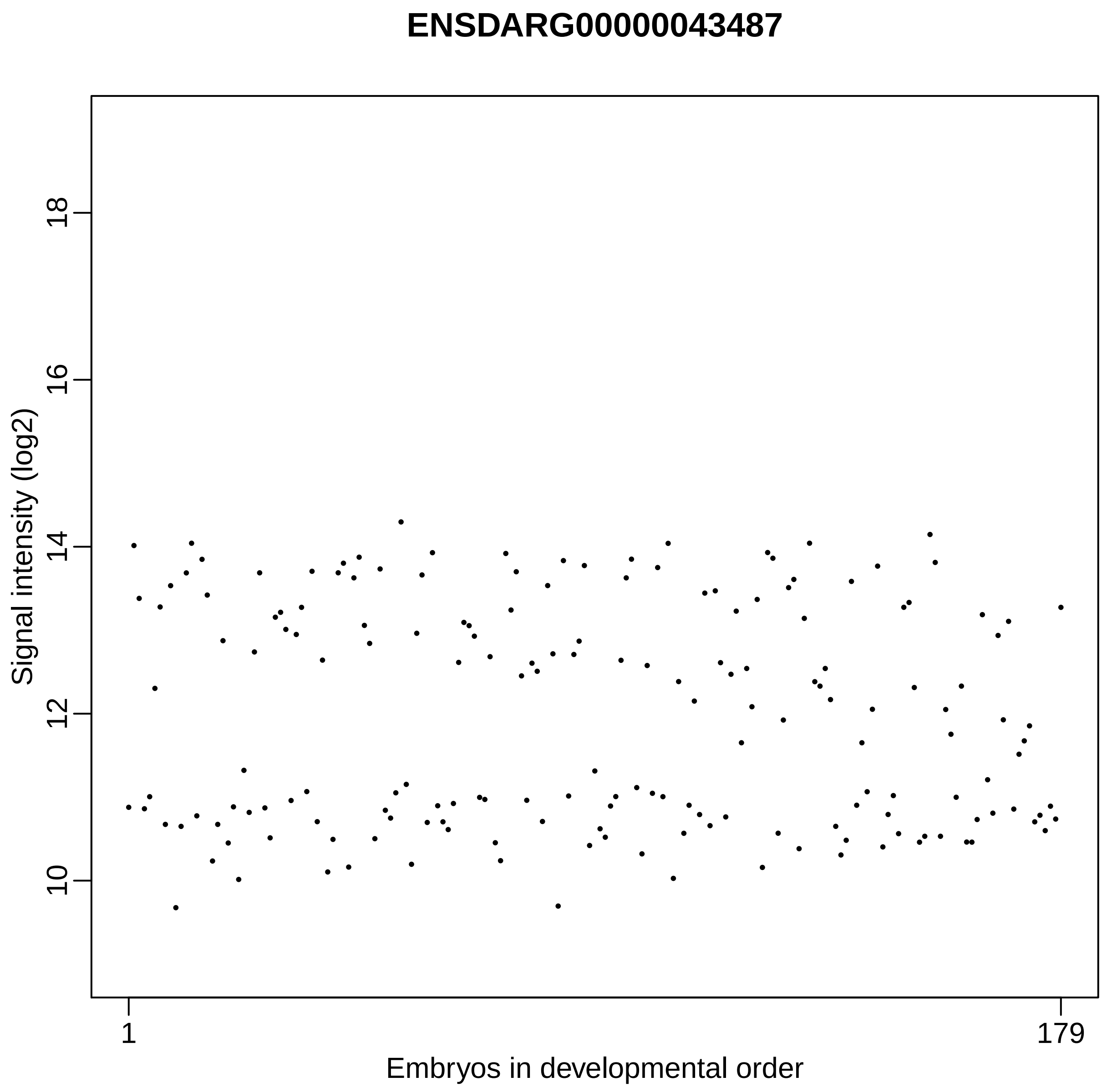
ENSDARG00000043487
Signal intensity (log2)
10
12
14
16
18
1
179
Embryos in developmental order

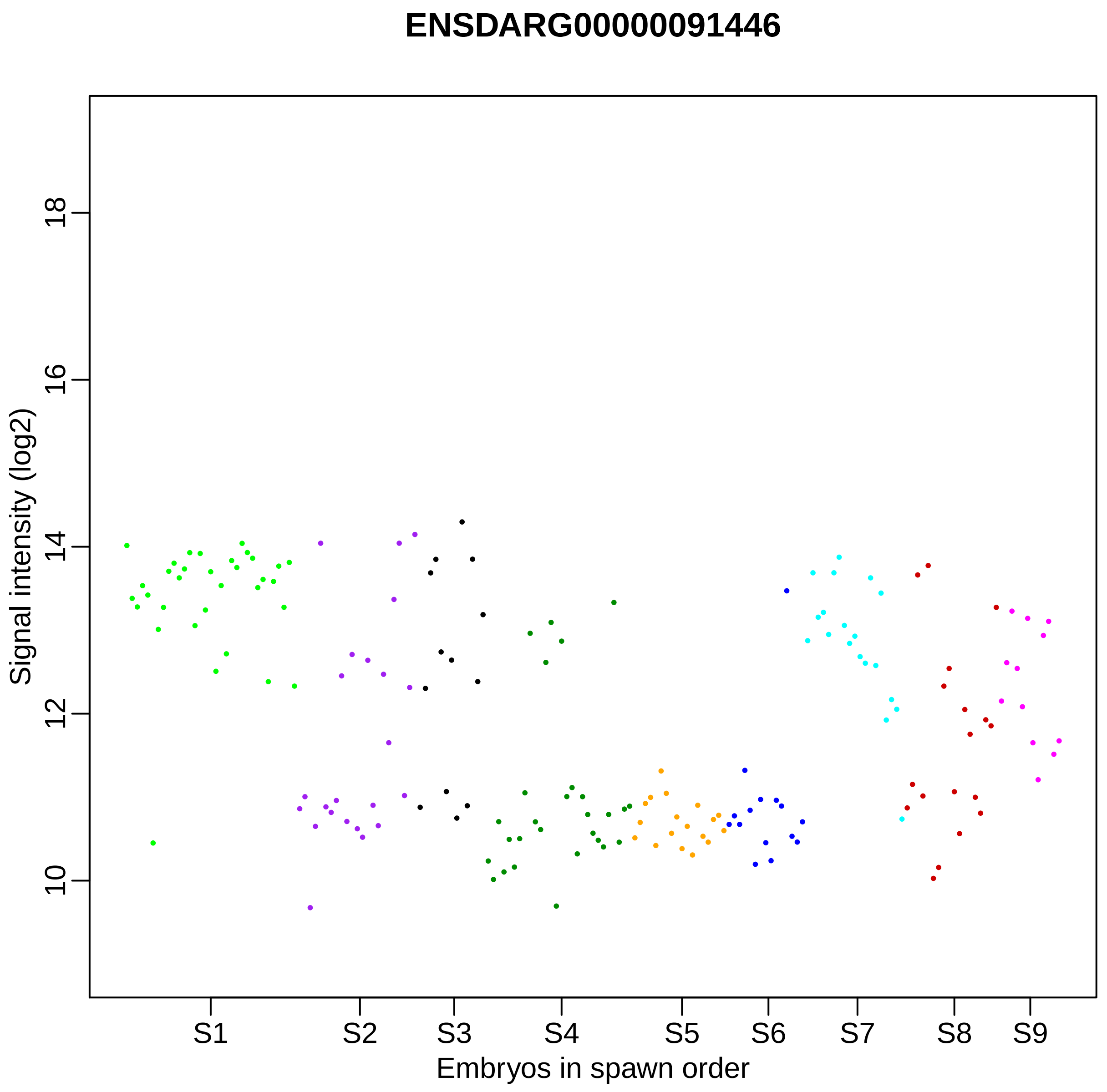
ENSDARG00000091446
Signal intensity (log2)
10
12
14
16
18
S1
S2
S3
S4
S5
S6
S7
S8
S9
Embryos in spawn order

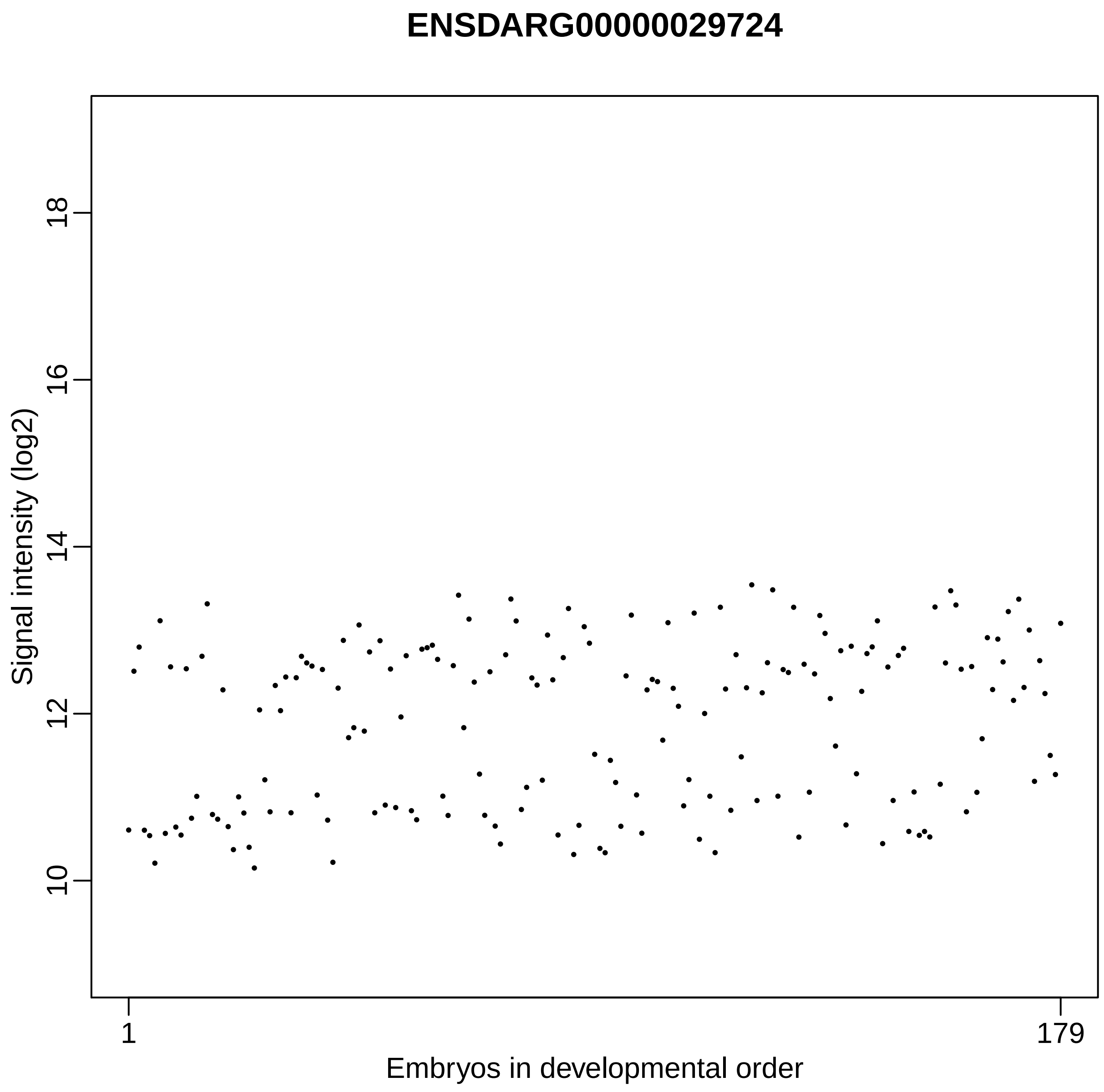

ENSDARG00000029724
Signal intensity (log2)
10
12
14
16
18
1
179
Embryos in developmental order

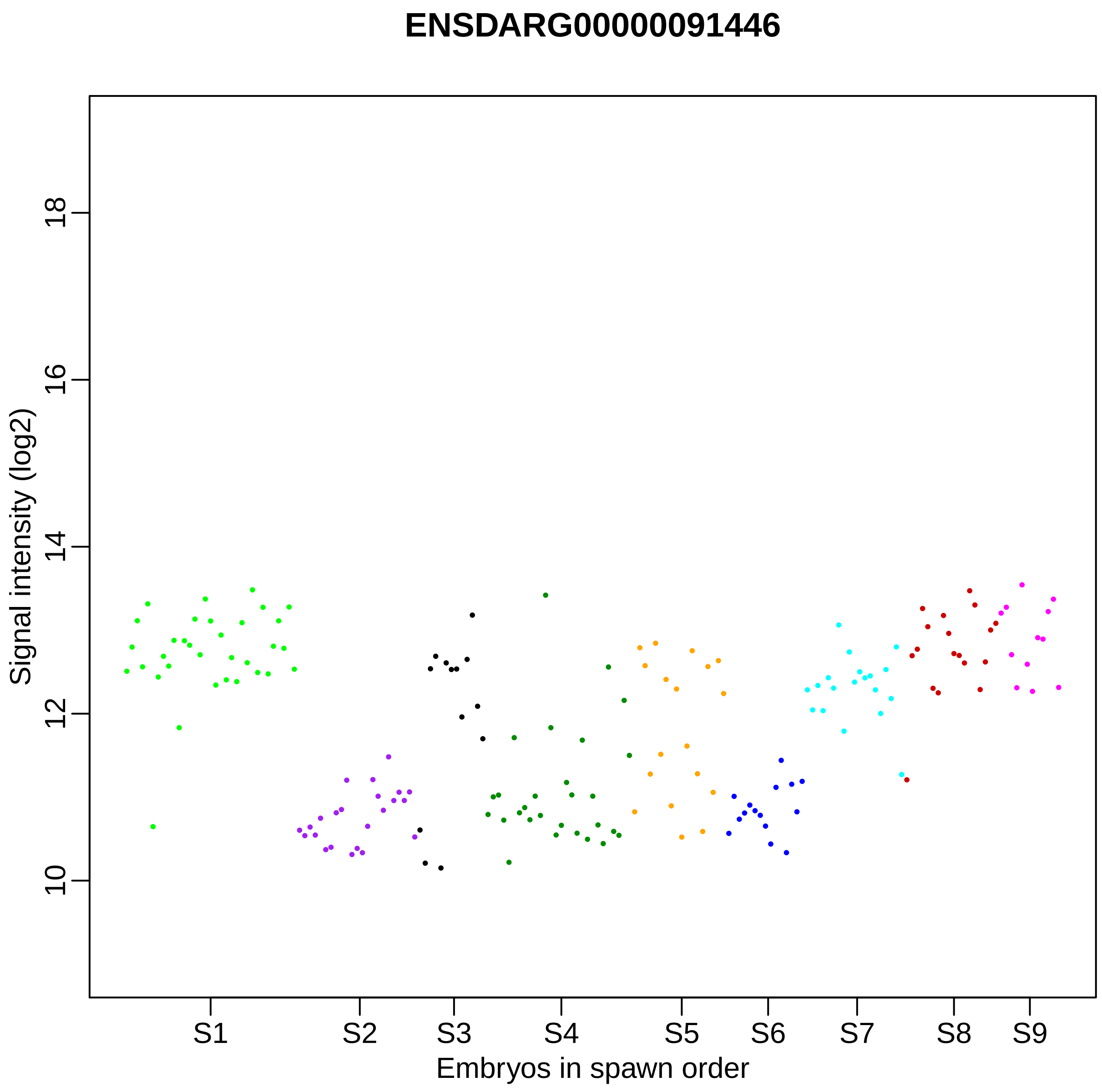

ENSDARG00000091446
Signal intensity (log2)
10
12
14
16
18
S1
S2
S3
S4
S5
S6
S7
S8
S9
Embryos in spawn order

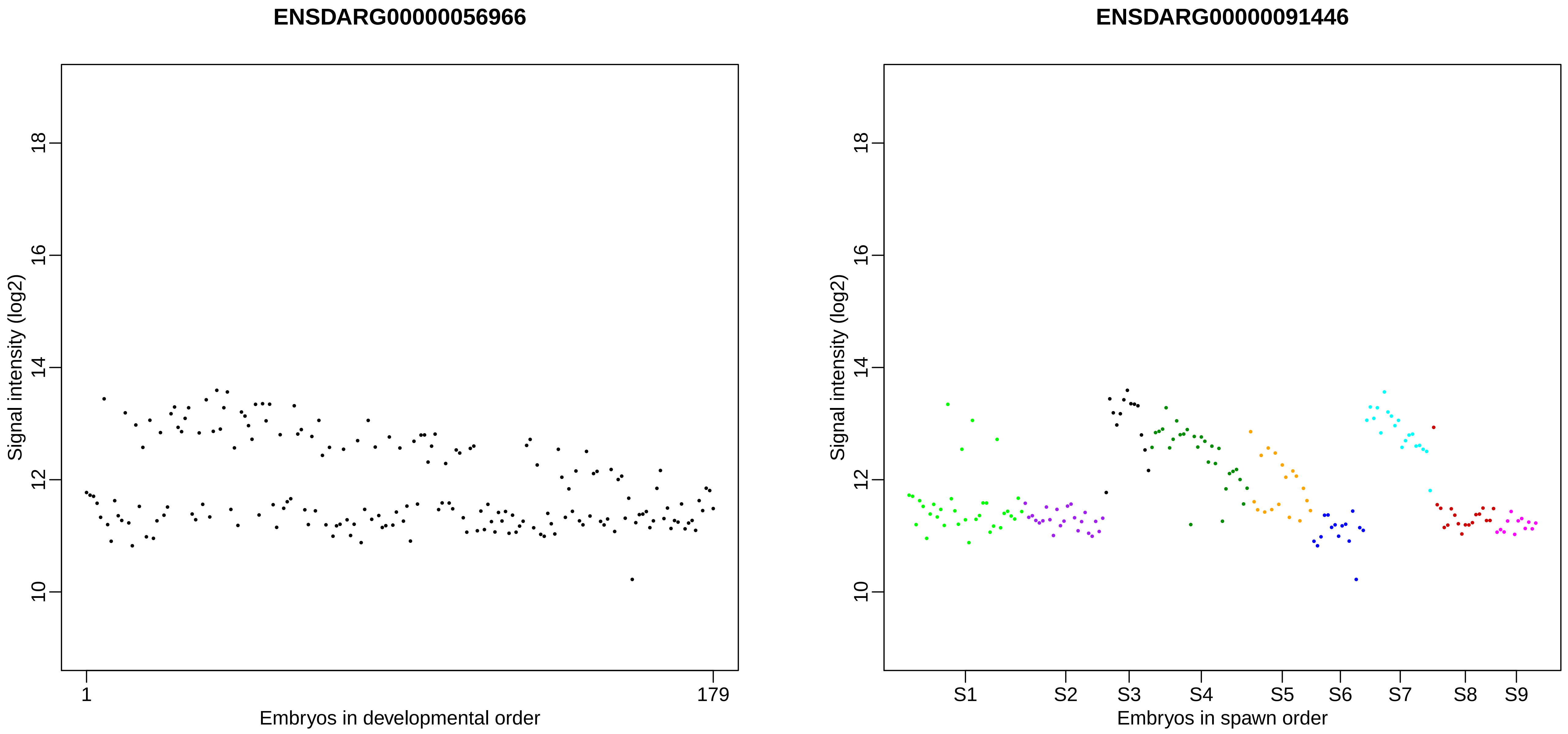

ENSDARG00000056966
Signal intensity (log2)
10
12
14
16
18
1
179
Embryos in developmental order
ENSDARG00000091446
Signal intensity (log2)
10
12
14
16
18
S1
S2
S3
S4
S5
S6
S7
S8
S9
Embryos in spawn order

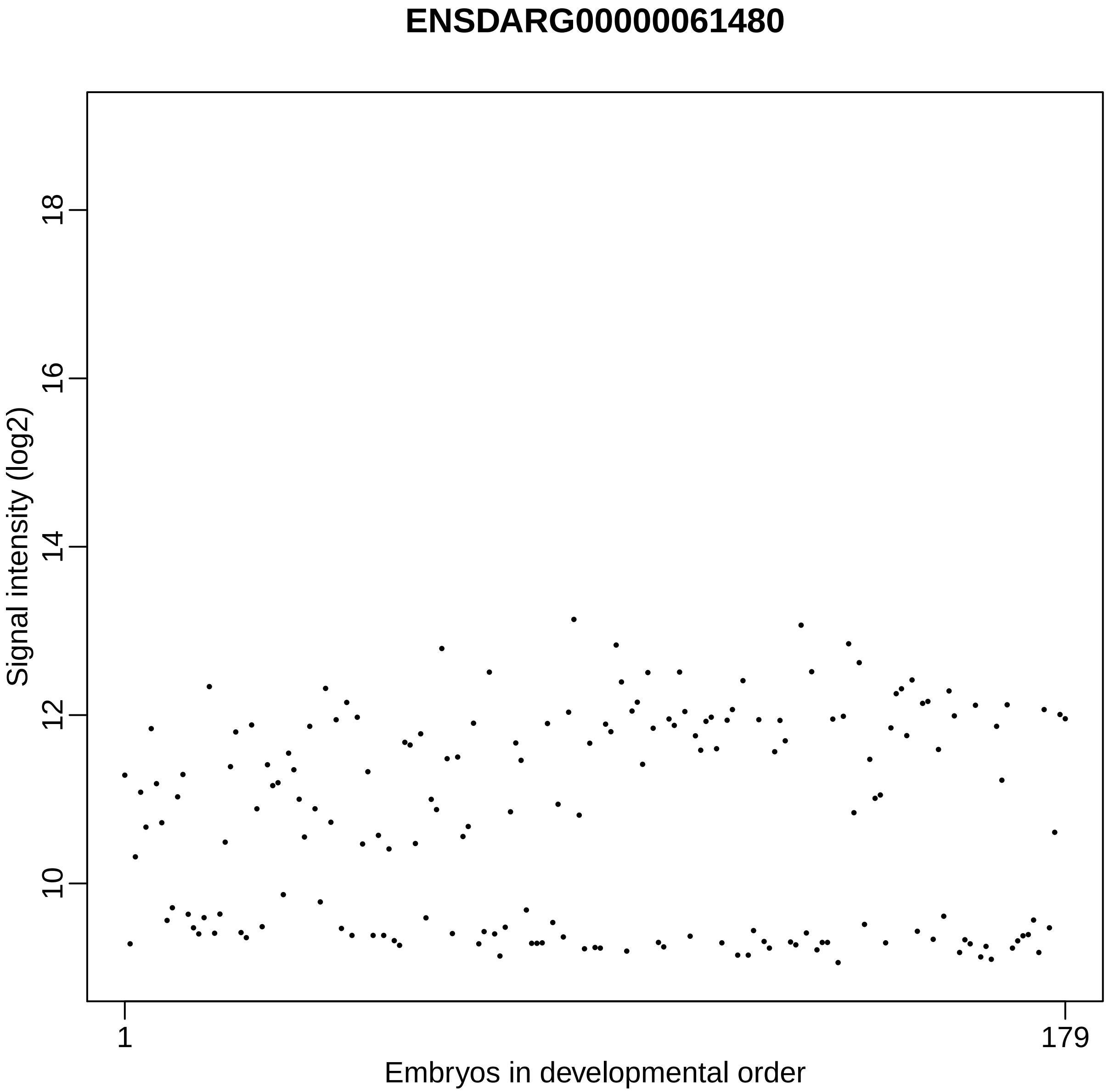

ENSDARG00000061480
Signal intensity (log2)
10
12
14
16
18
1
179
Embryos in developmental order

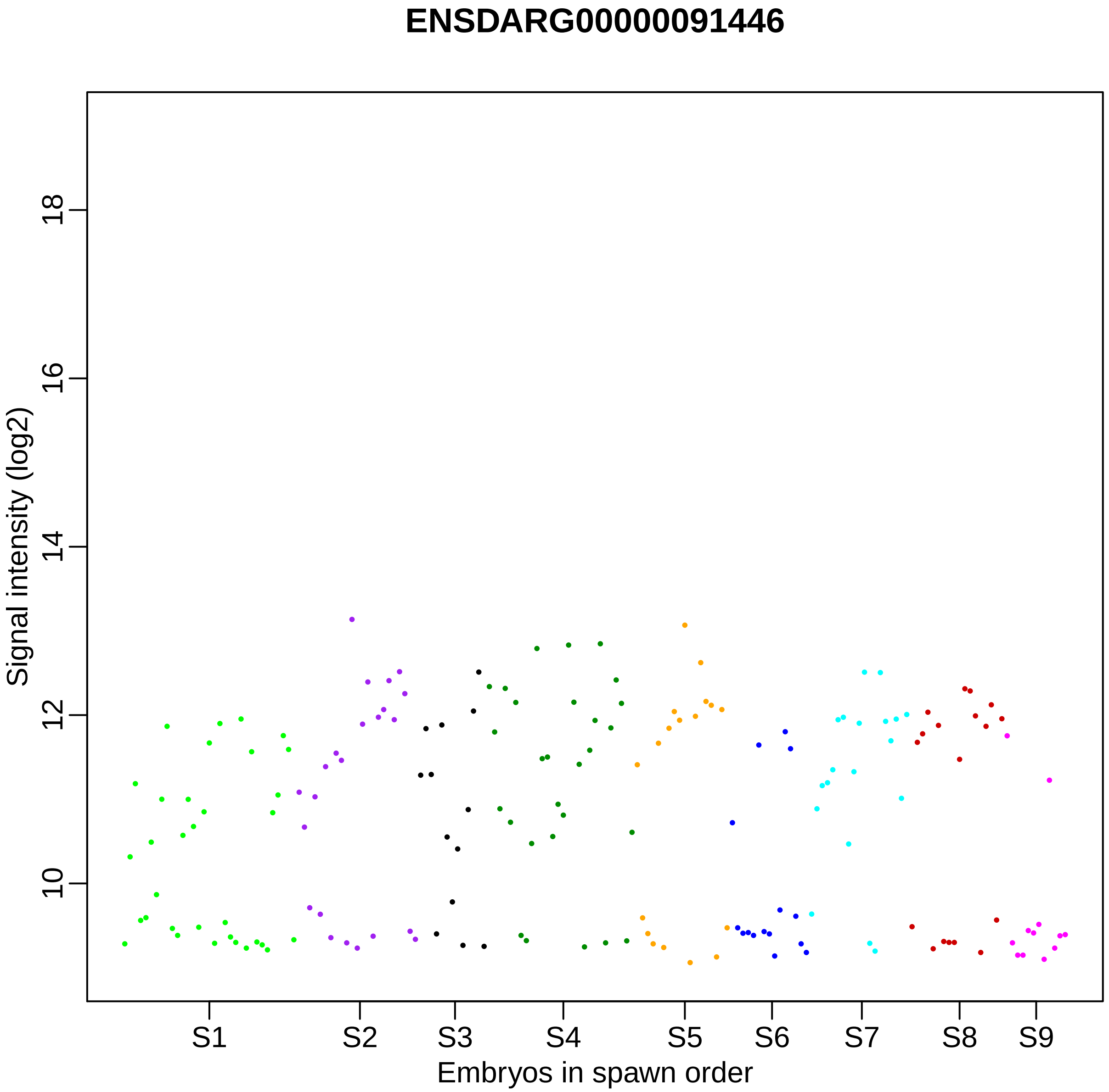

ENSDARG00000091446
Signal intensity (log2)
10
12
14
16
18
S1
S2
S3
S4
S5
S6
S7
S8
S9
Embryos in spawn order

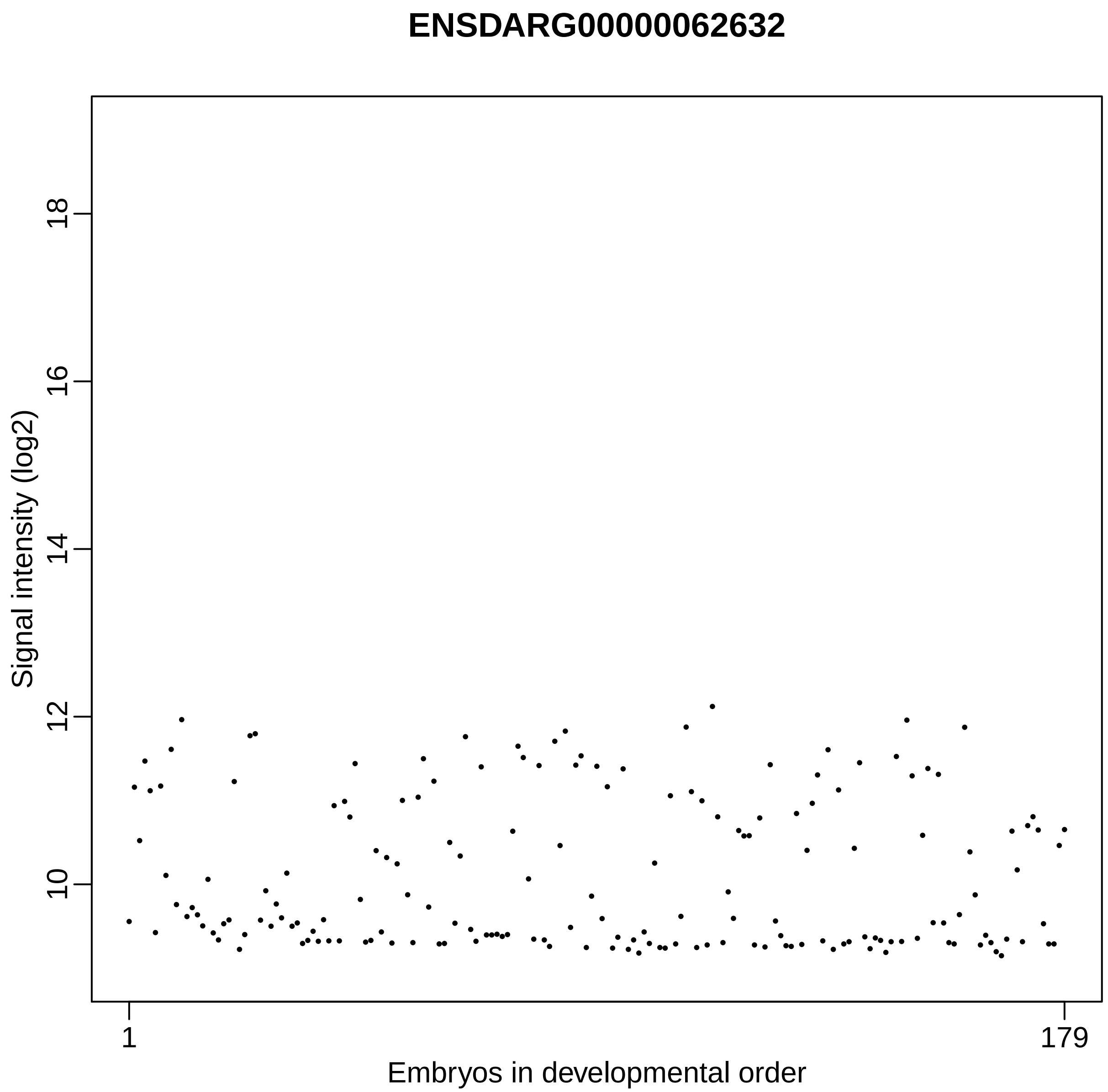

ENSDARG00000062632
Signal intensity (log2)
18
16
14
12
10
1
179
Embryos in developmental order

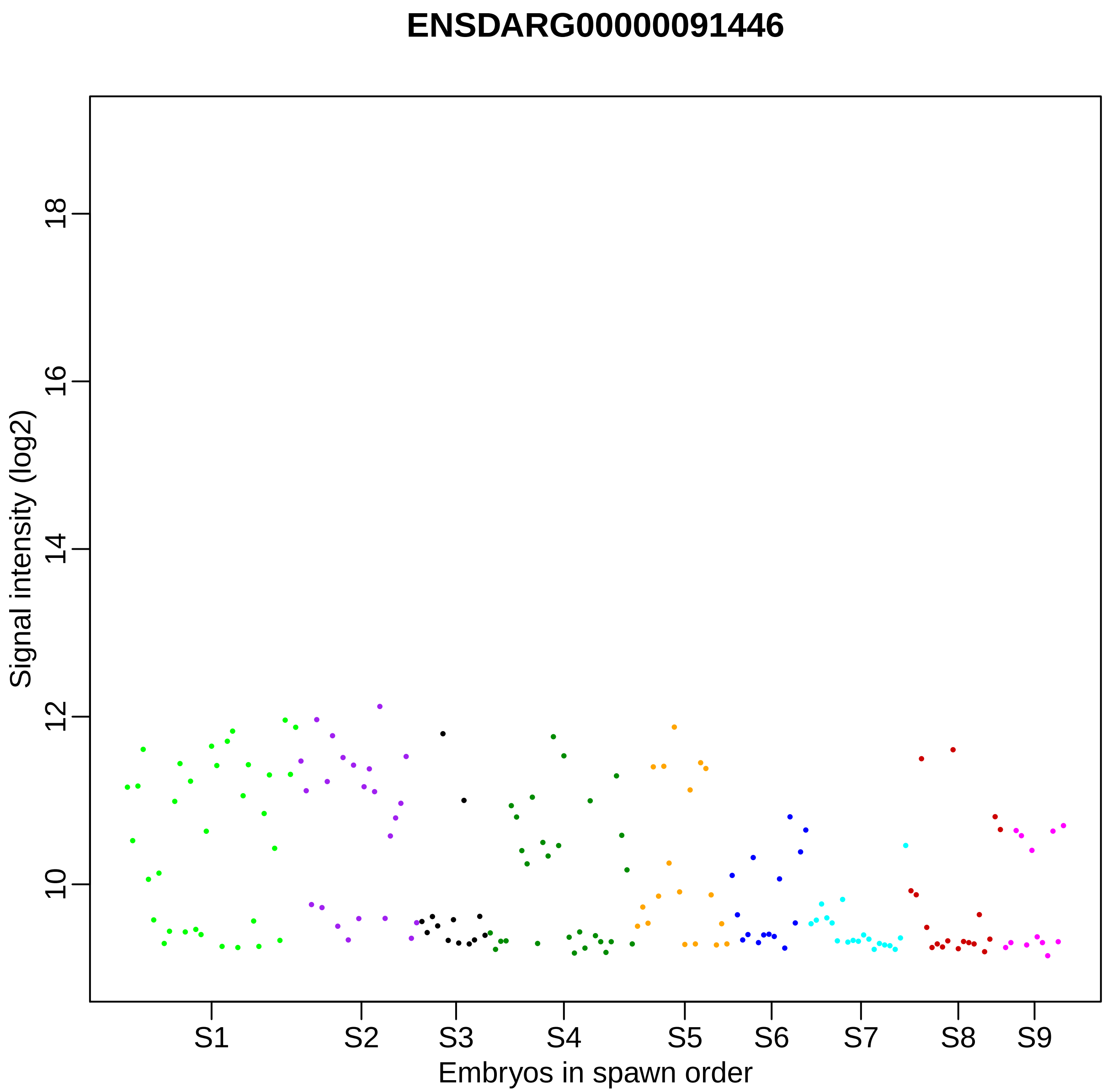

ENSDARG00000091446
Signal intensity (log2)
18
16
14
12
10
S1
S2
S3
S4
S5
S6
S7
S8
S9
Embryos in spawn order

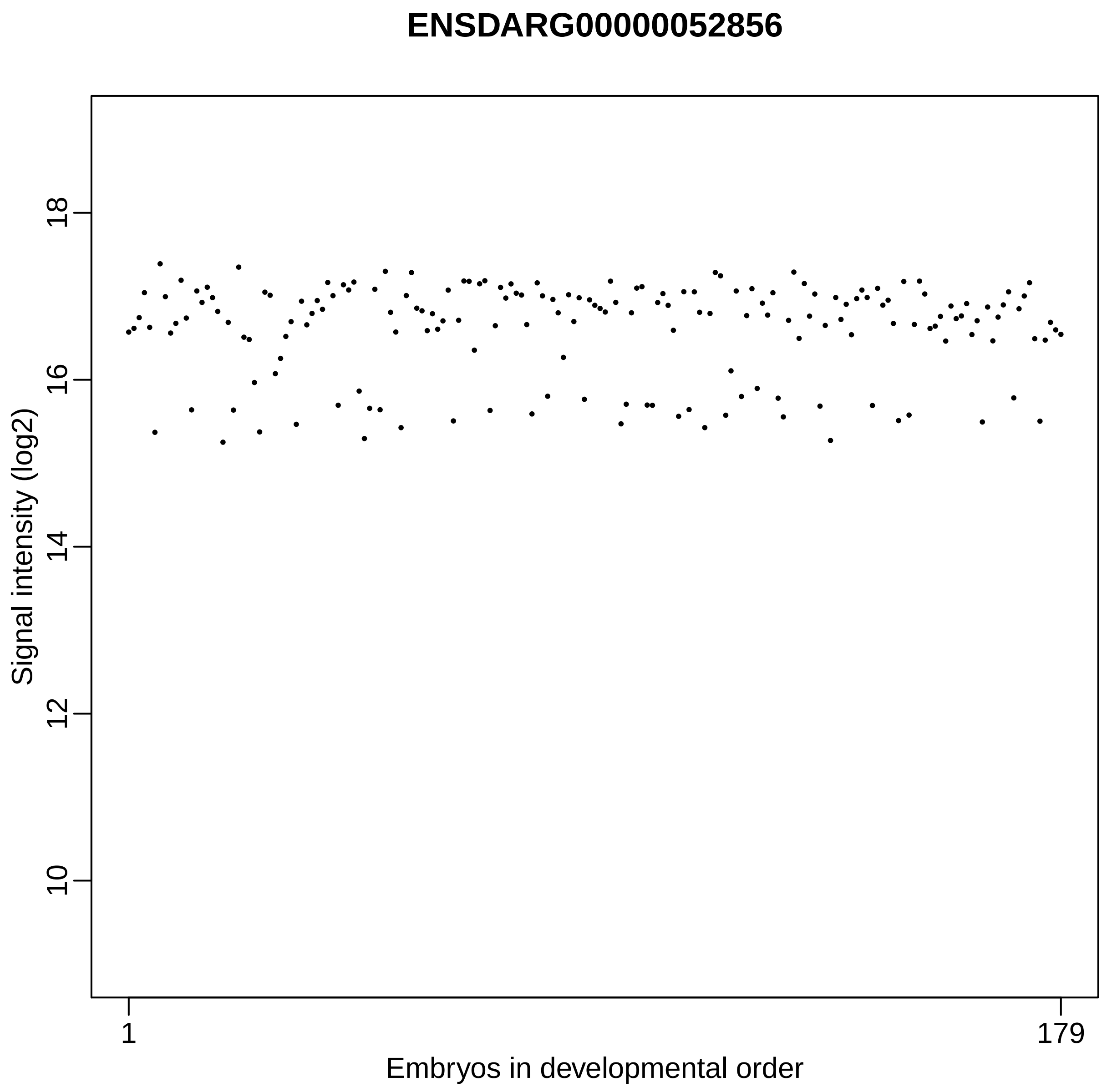
ENSDARG00000052856
Signal intensity (log2)
10
12
14
16
18
1
179
Embryos in developmental order

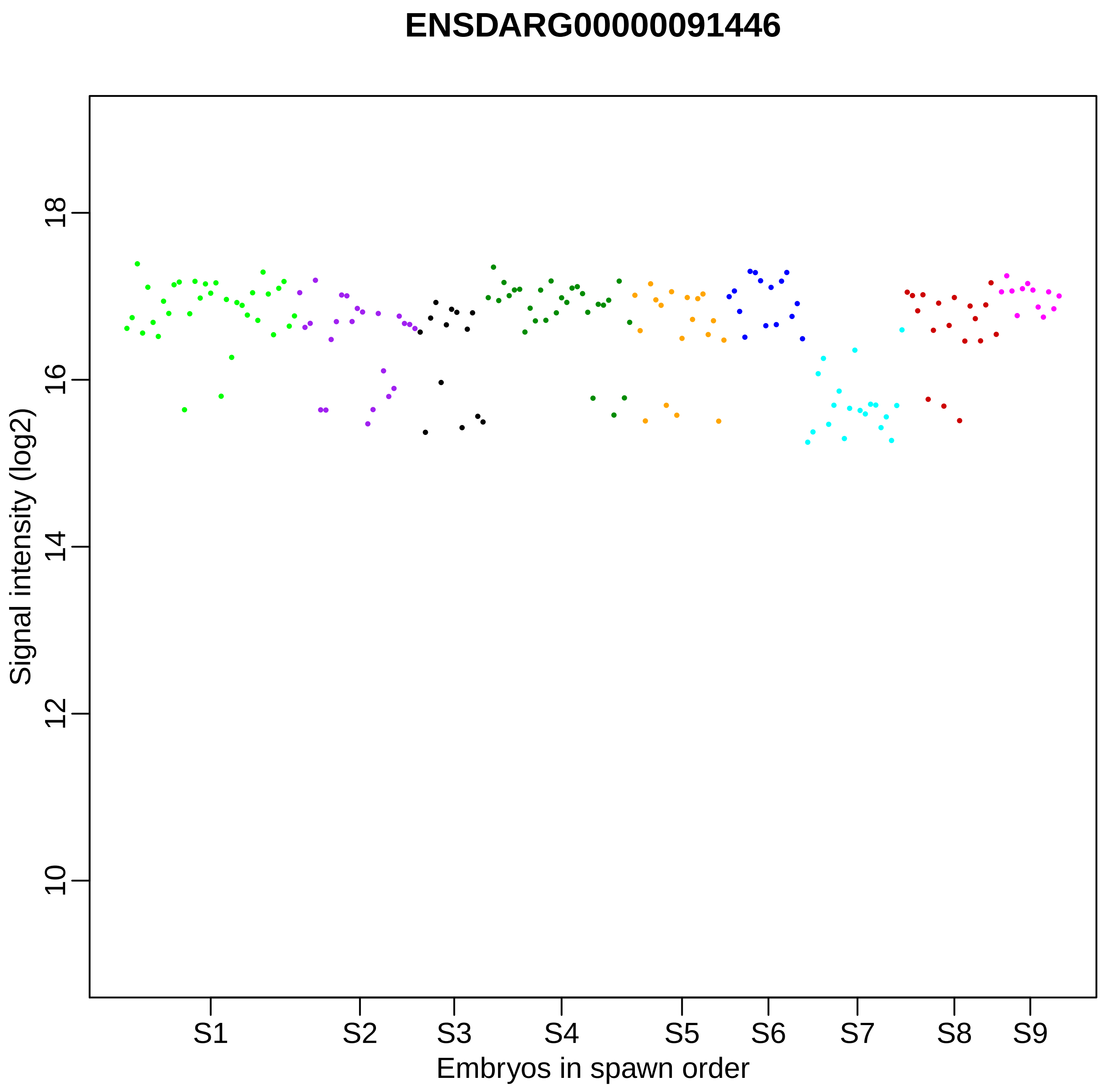
ENSDARG00000091446
Signal intensity (log2)
10
12
14
16
18
S1
S2
S3
S4
S5
S6
S7
S8
S9
Embryos in spawn order

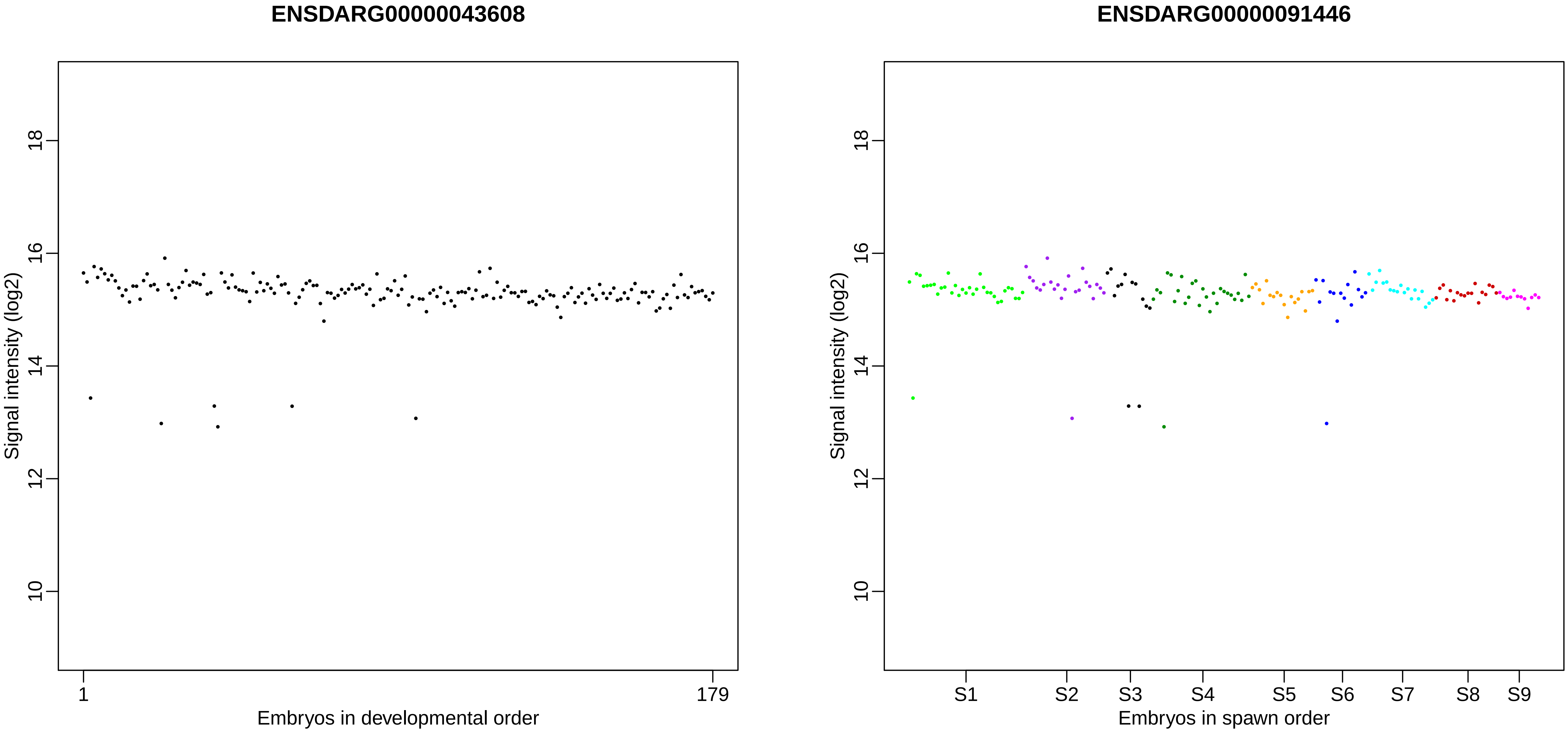

ENSDARG00000043608
Signal intensity (log2)
18
16
14
12
10
1
179
Embryos in developmental order
ENSDARG00000091446
Signal intensity (log2)
18
16
14
12
10
S1
S2
S3
S4
S5
S6
S7
S8
S9
Embryos in spawn order

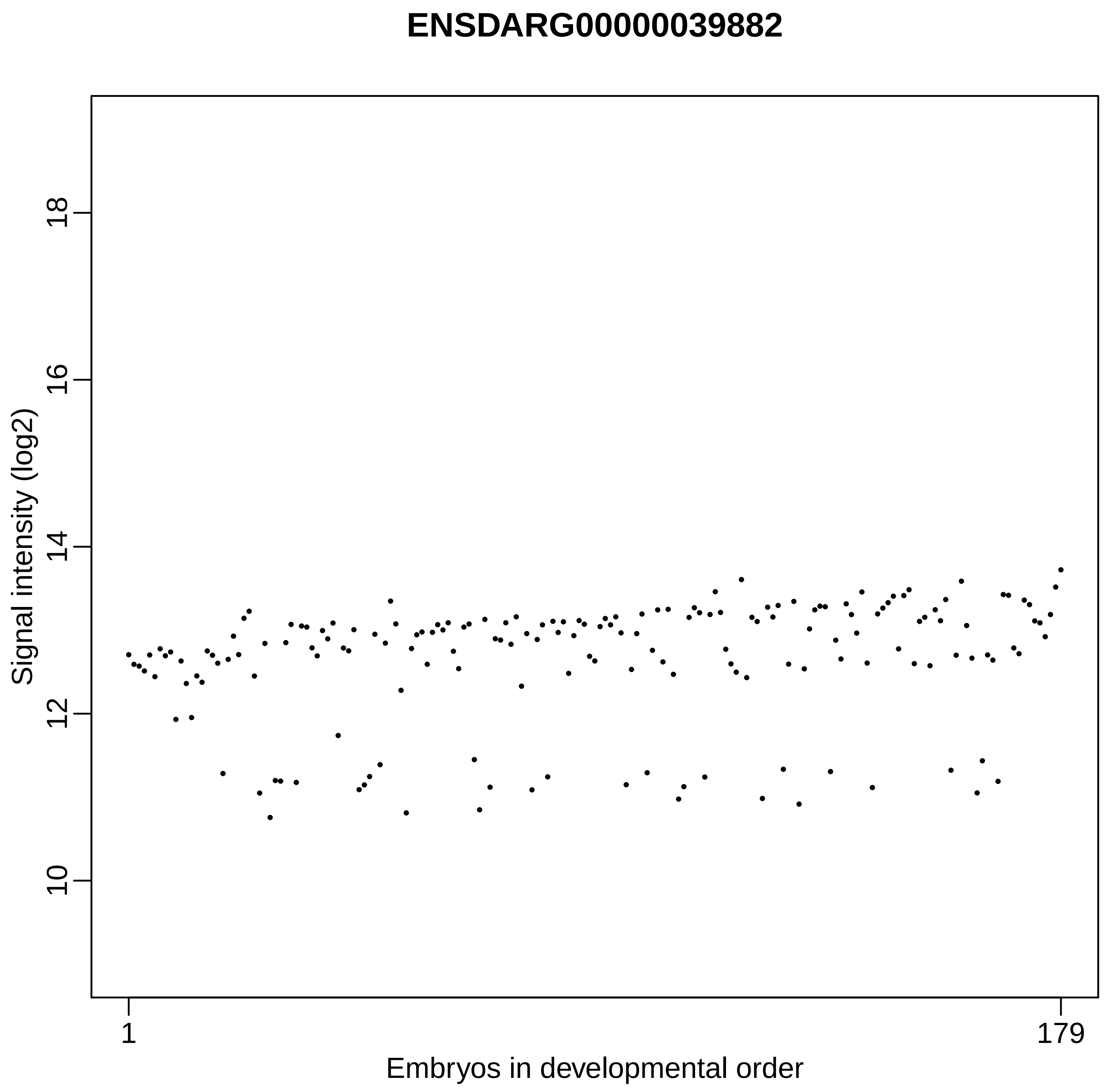

ENSDARG00000039882
Signal intensity (log2)
10
12
14
16
18
1
179
Embryos in developmental order

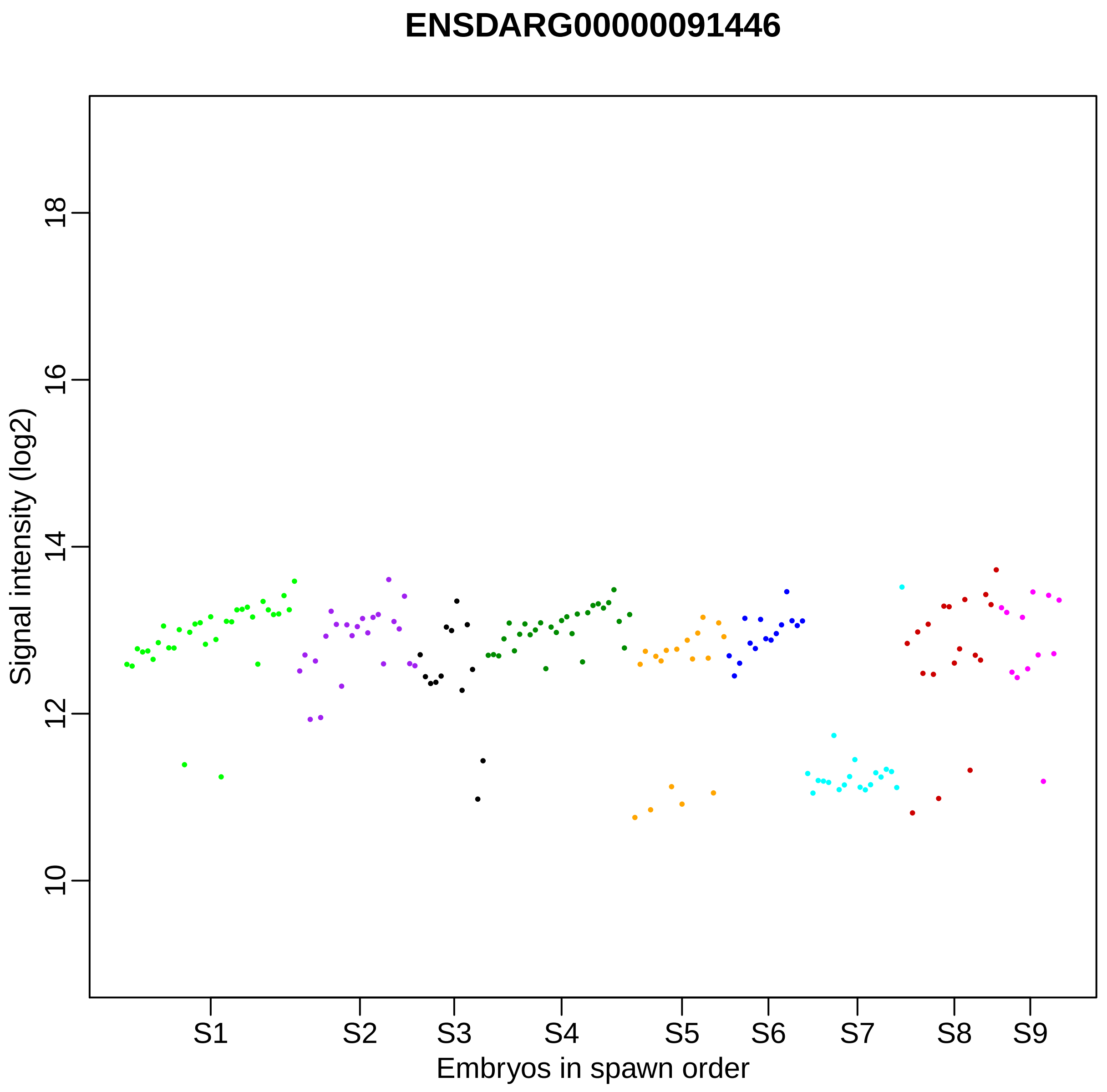

ENSDARG00000091446
Signal intensity (log2)
10
12
14
16
18
S1
S2
S3
S4
S5
S6
S7
S8
S9
Embryos in spawn order

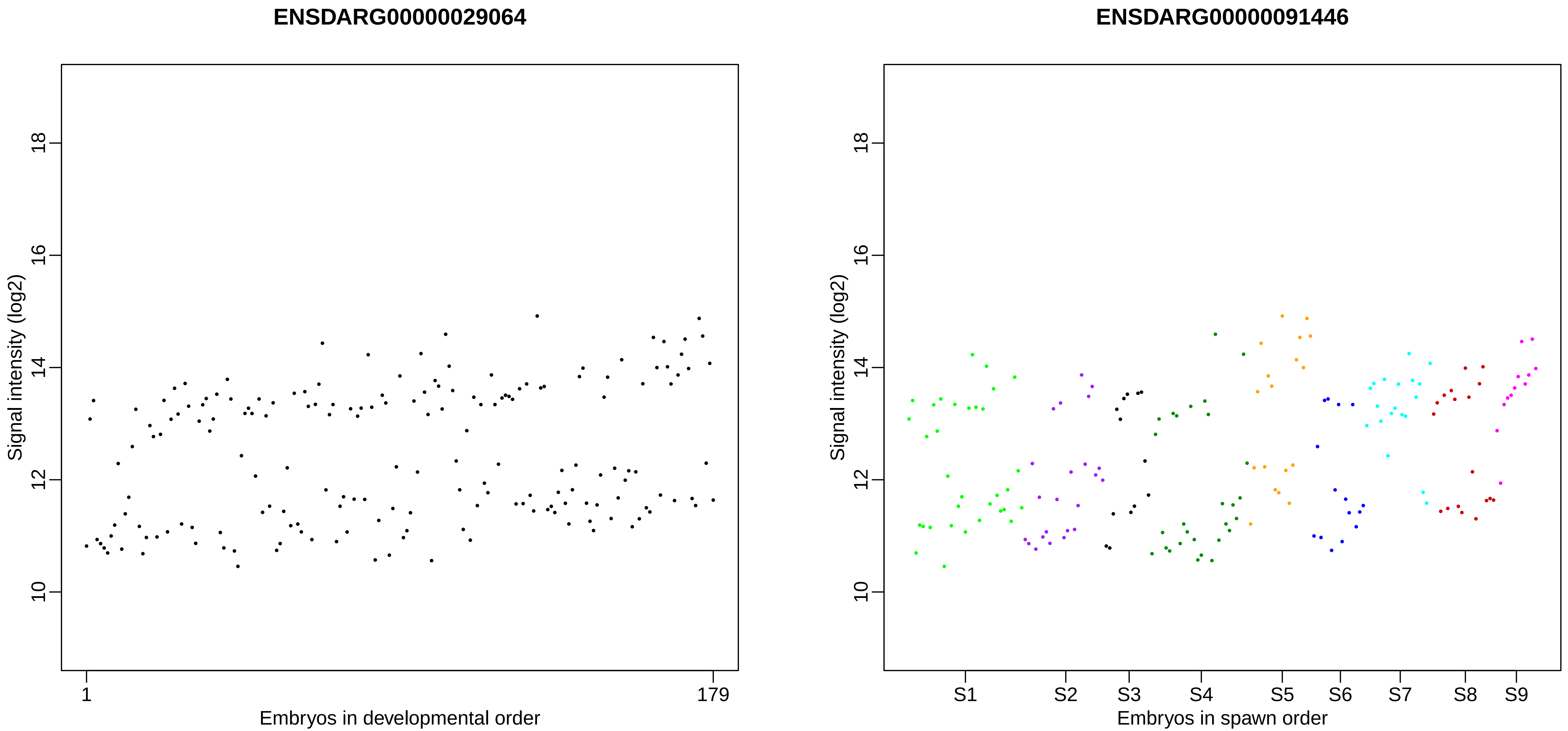

ENSDARG00000029064
Signal intensity (log2)
10
12
14
16
18
1
179
Embryos in developmental order
ENSDARG00000091446
Signal intensity (log2)
10
12
14
16
18
S1
S2
S3
S4
S5
S6
S7
S8
S9
Embryos in spawn order

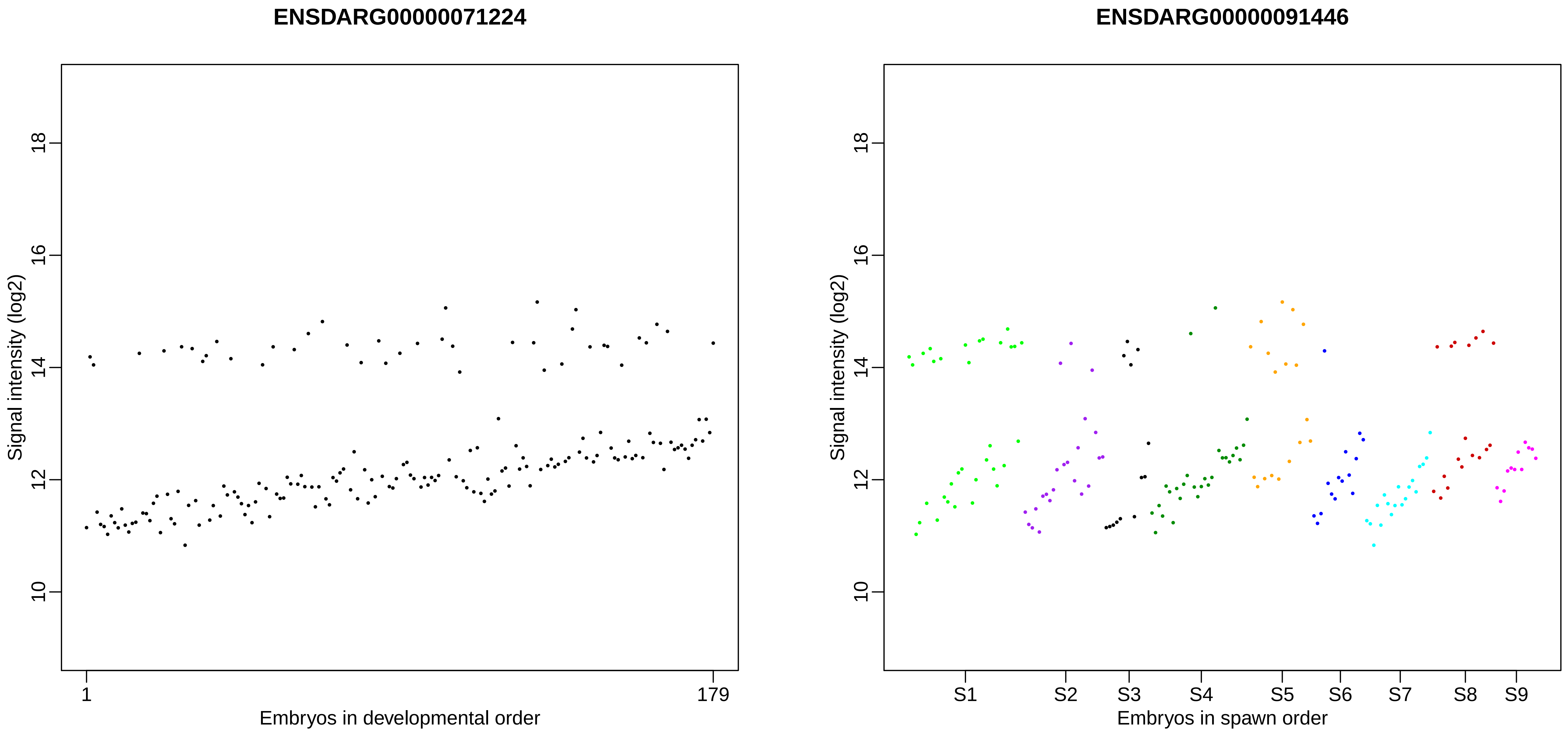
ENSDARG00000071224
Signal intensity (log2)
10
12
14
16
18
1
179
Embryos in developmental order
ENSDARG00000091446
Signal intensity (log2)
10
12
14
16
18
S1
S2
S3
S4
S5
S6
S7
S8
S9
Embryos in spawn order

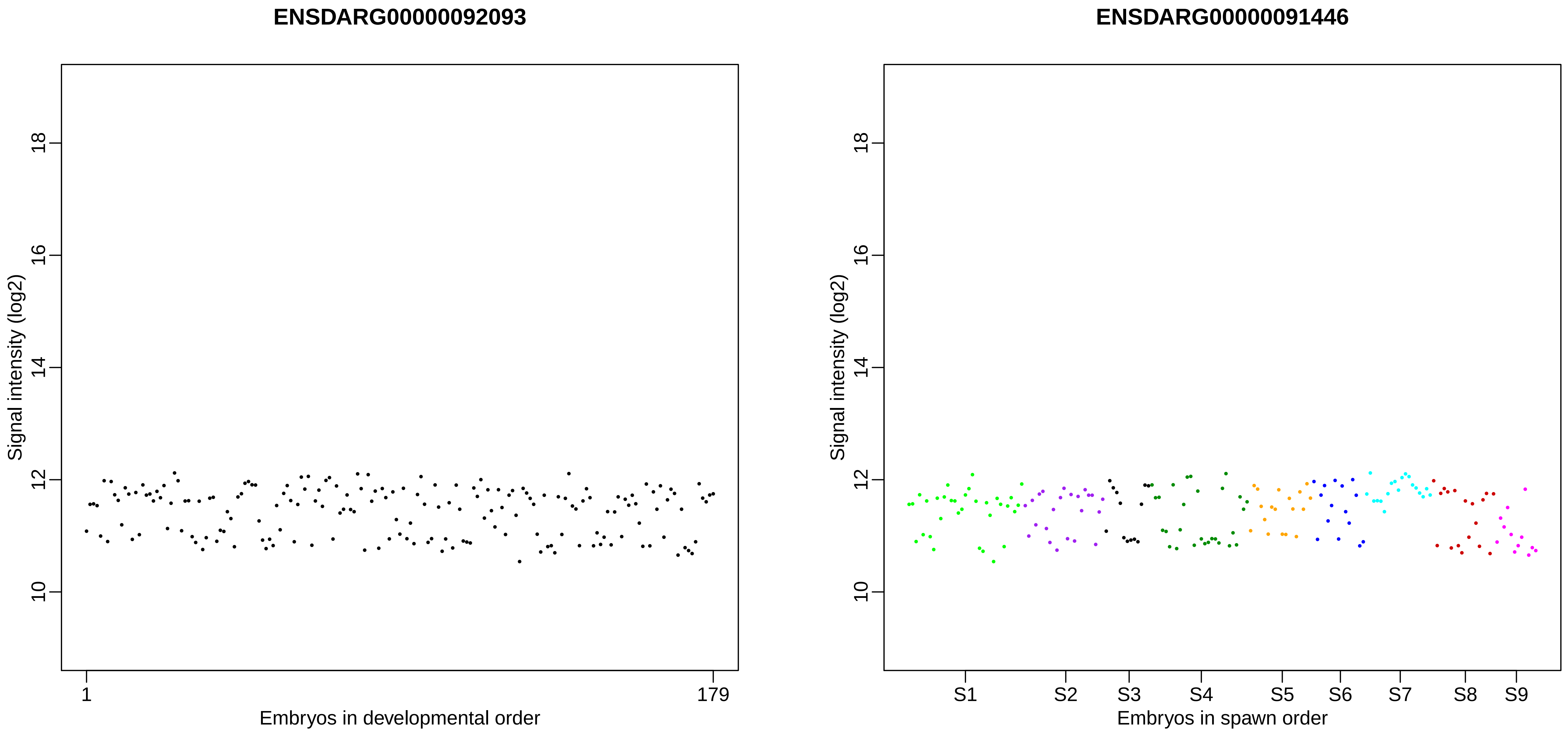
ENSDARG00000092093
Signal intensity (log2)
10
12
14
16
18
1
179
Embryos in developmental order
ENSDARG00000091446
Signal intensity (log2)
10
12
14
16
18
S1
S2
S3
S4
S5
S6
S7
S8
S9
Embryos in spawn order

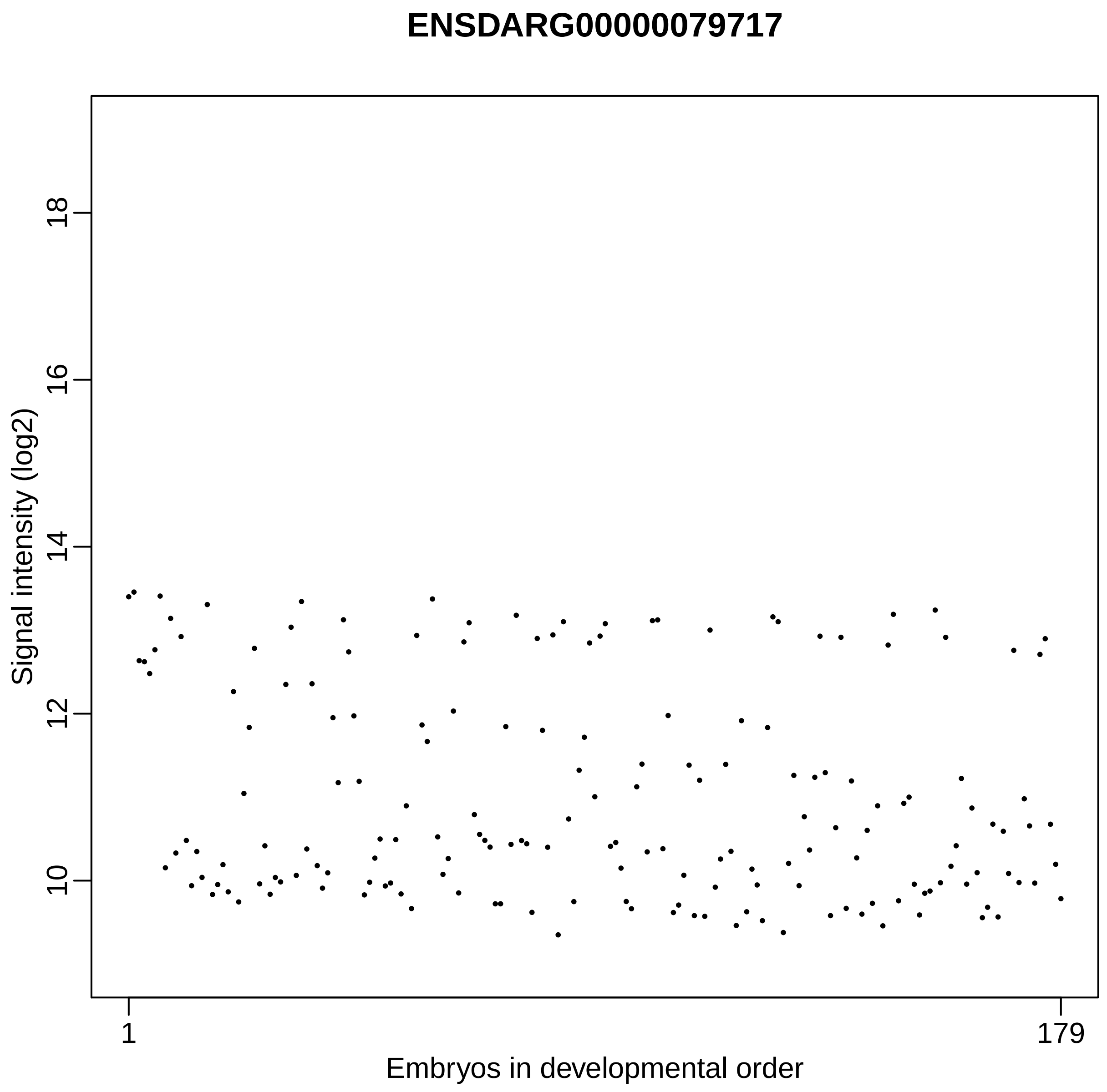
ENSDARG00000079717
Signal intensity (log2)
10
12
14
16
18
1
179
Embryos in developmental order

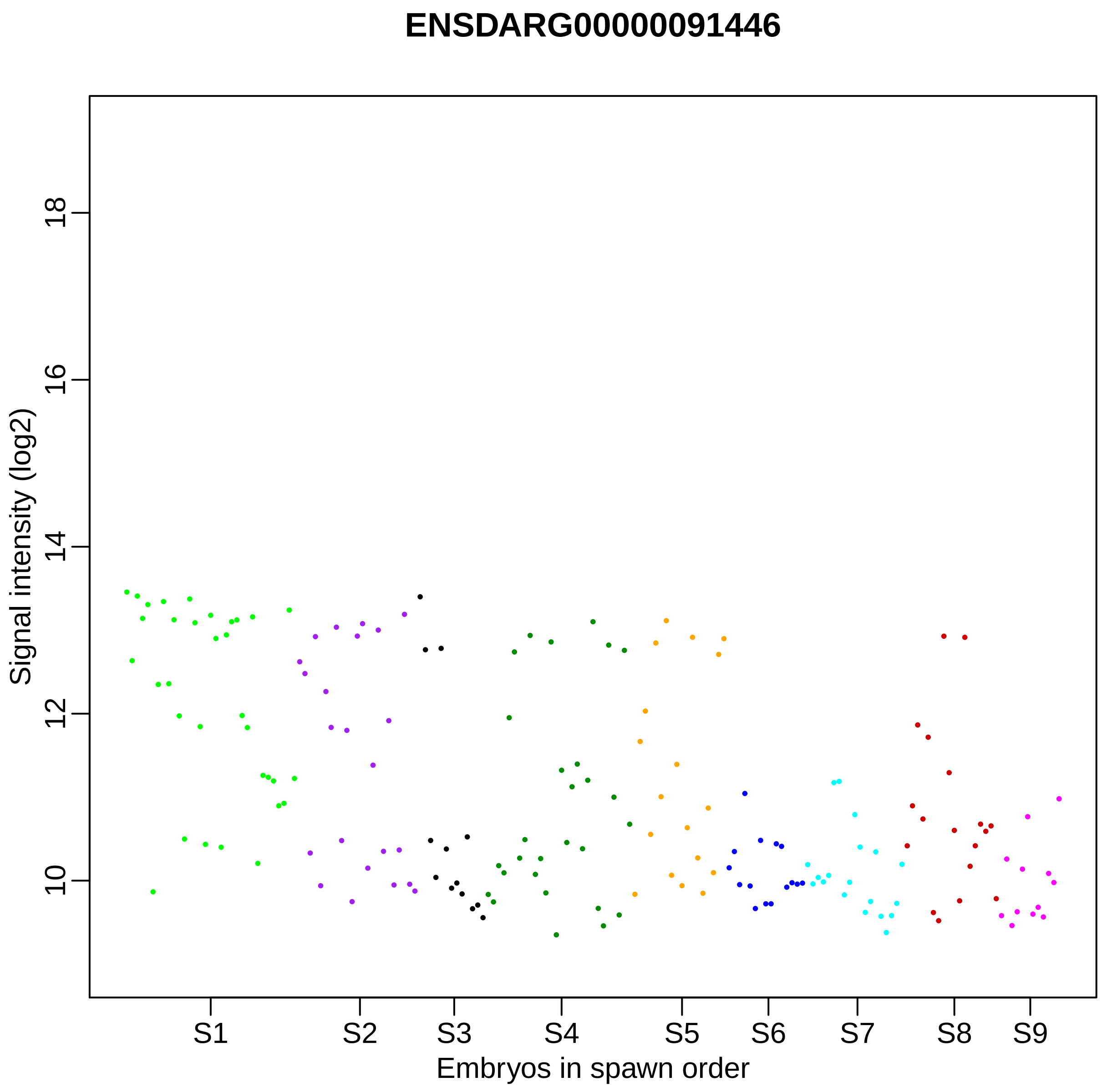
ENSDARG00000091446
Signal intensity (log2)
10
12
14
16
18
S1
S2
S3
S4
S5
S6
S7
S8
S9
Embryos in spawn order

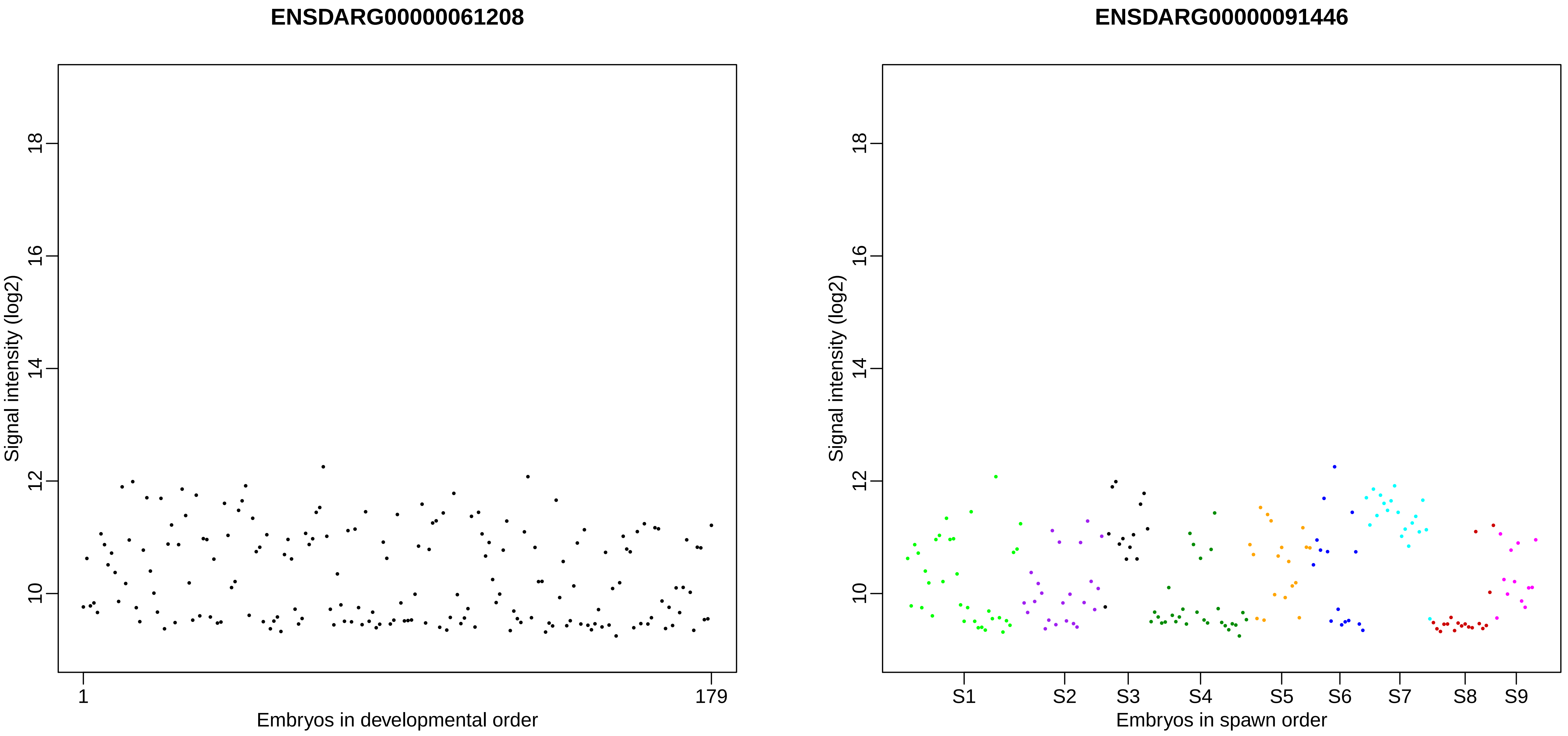

ENSDARG00000061208
Signal intensity (log2)
10
12
14
16
18
1
179
Embryos in developmental order
ENSDARG00000091446
Signal intensity (log2)
10
12
14
16
18
S1
S2
S3
S4
S5
S6
S7
S8
S9
Embryos in spawn order

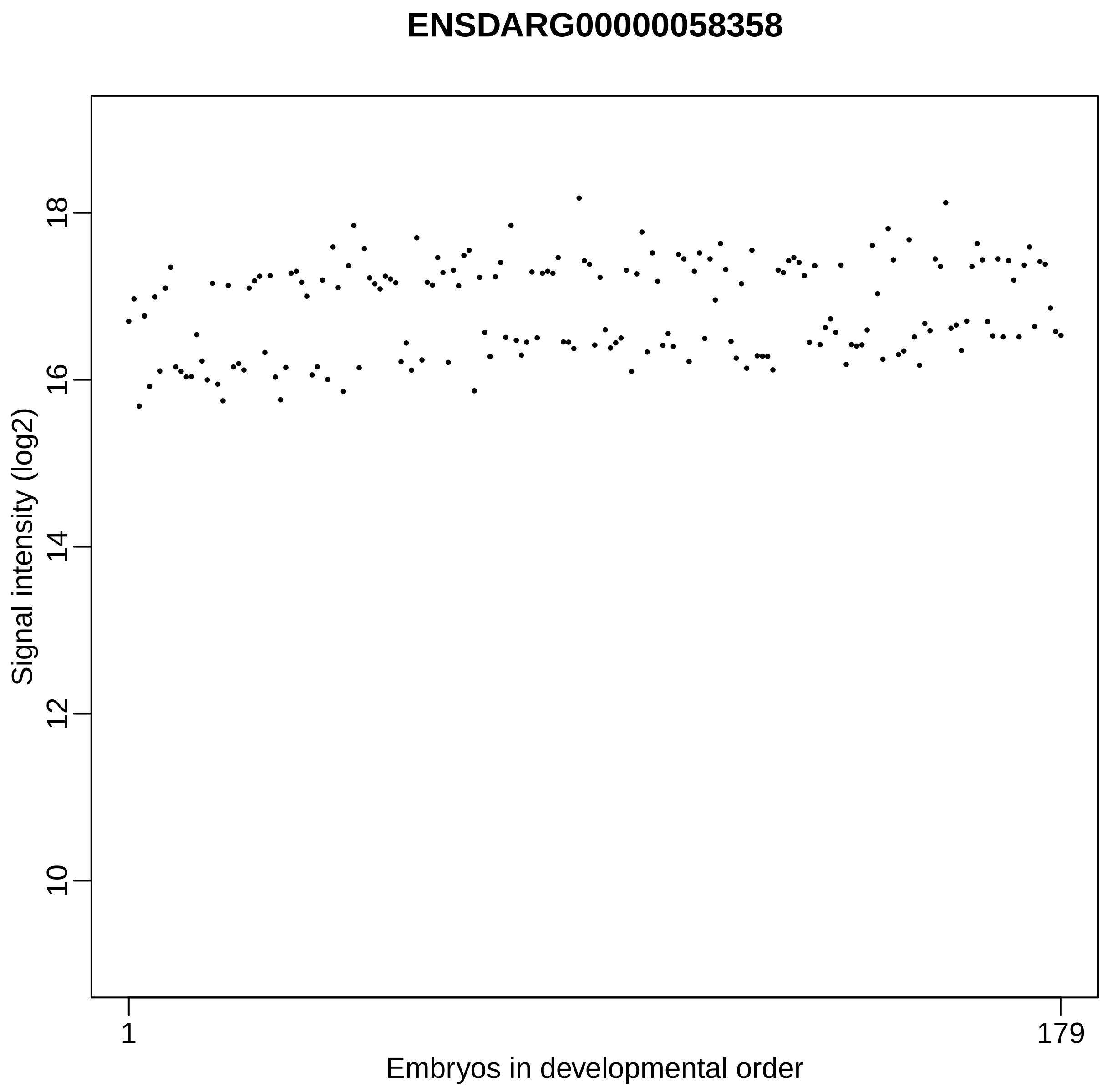
ENSDARG00000058358
Signal intensity (log2)
10
12
14
16
18
1
179
Embryos in developmental order

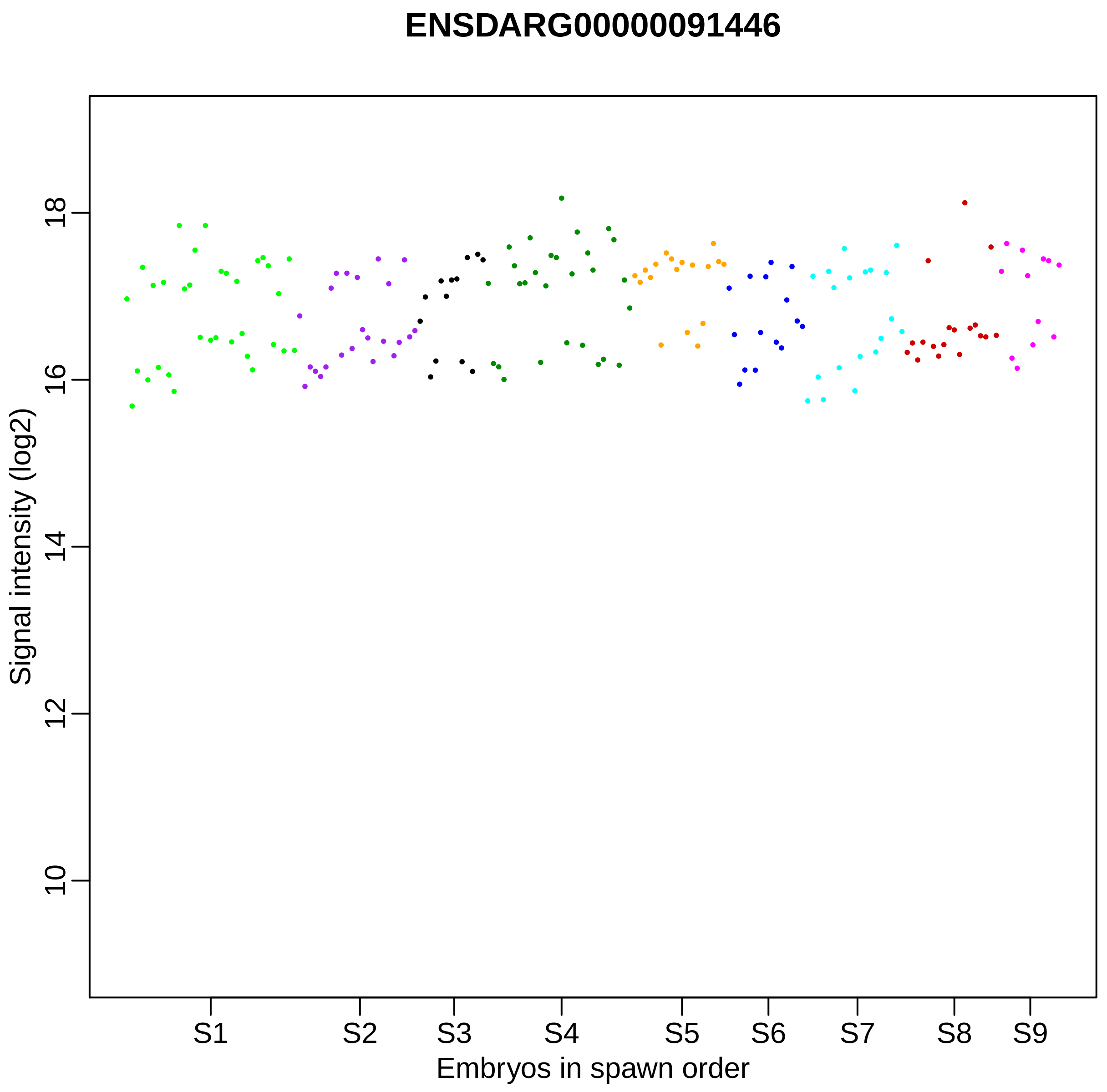
ENSDARG00000091446
Signal intensity (log2)
10
12
14
16
18
S1
S2
S3
S4
S5
S6
S7
S8
S9
Embryos in spawn order

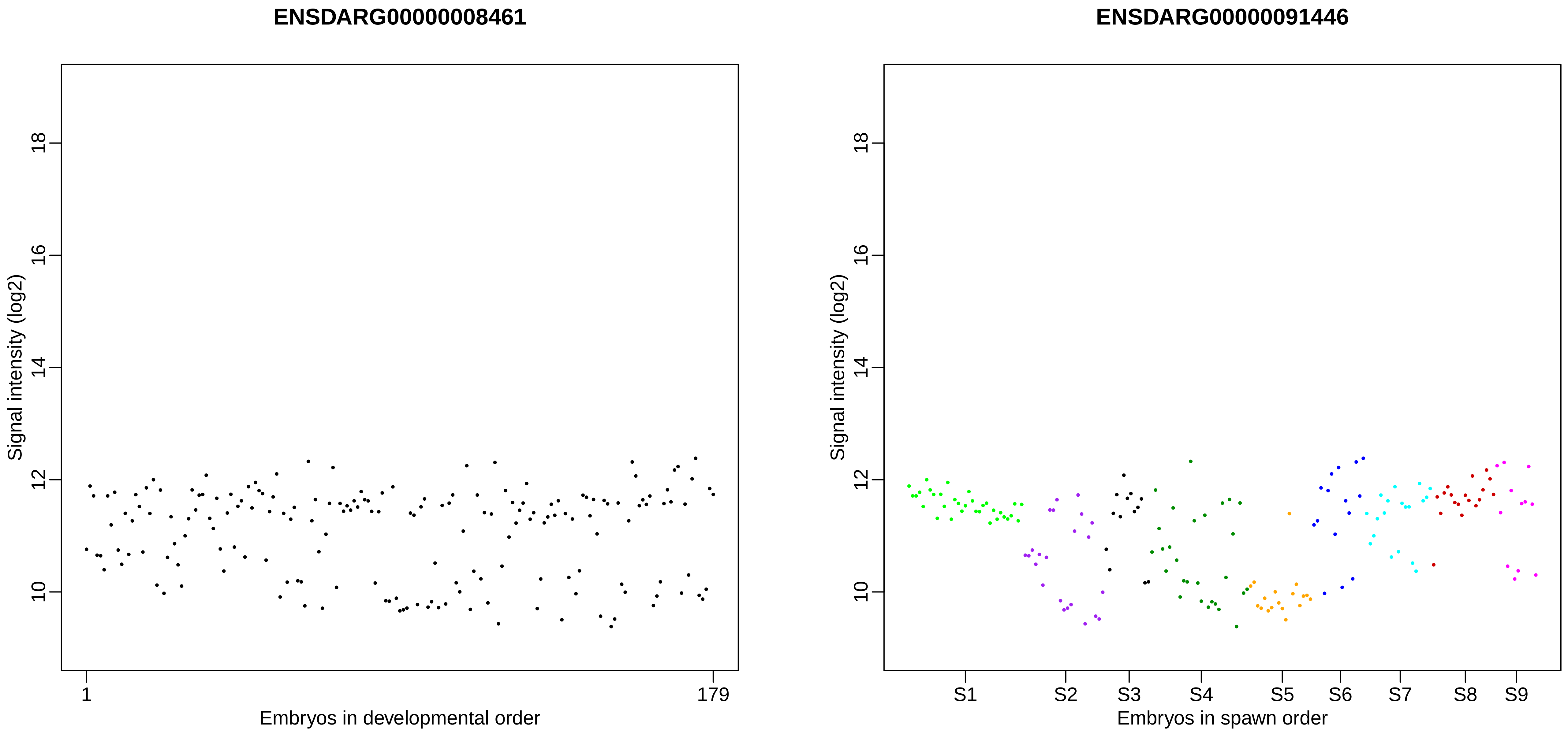

ENSDARG00000008461
Signal intensity (log2)
10
12
14
16
18
1
179
Embryos in developmental order
ENSDARG00000091446
Signal intensity (log2)
10
12
14
16
18
S1
S2
S3
S4
S5
S6
S7
S8
S9
Embryos in spawn order

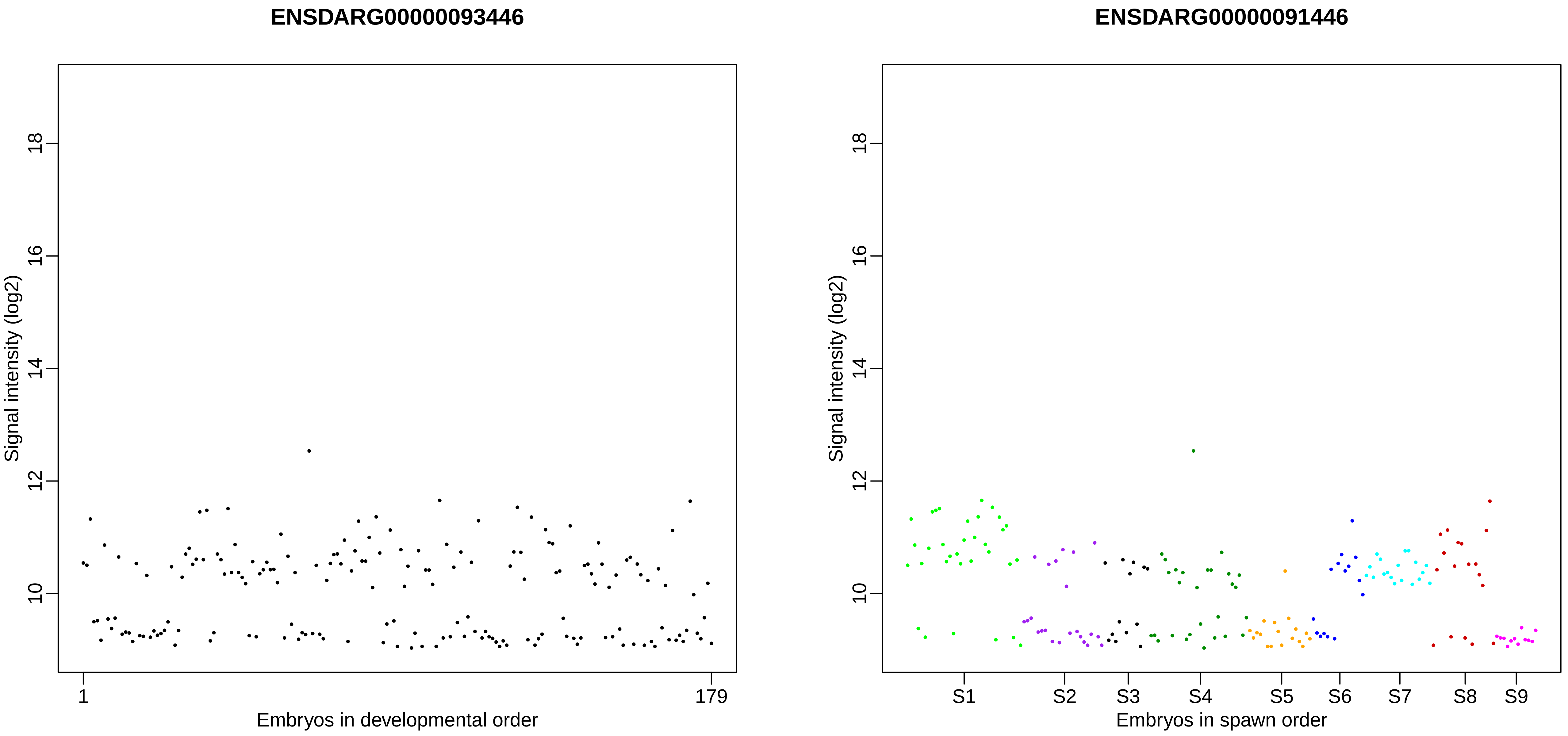

ENSDARG00000093446
Signal intensity (log2)
10
12
14
16
18
1
179
Embryos in developmental order
ENSDARG00000091446
Signal intensity (log2)
10
12
14
16
18
S1
S2
S3
S4
S5
S6
S7
S8
S9
Embryos in spawn order

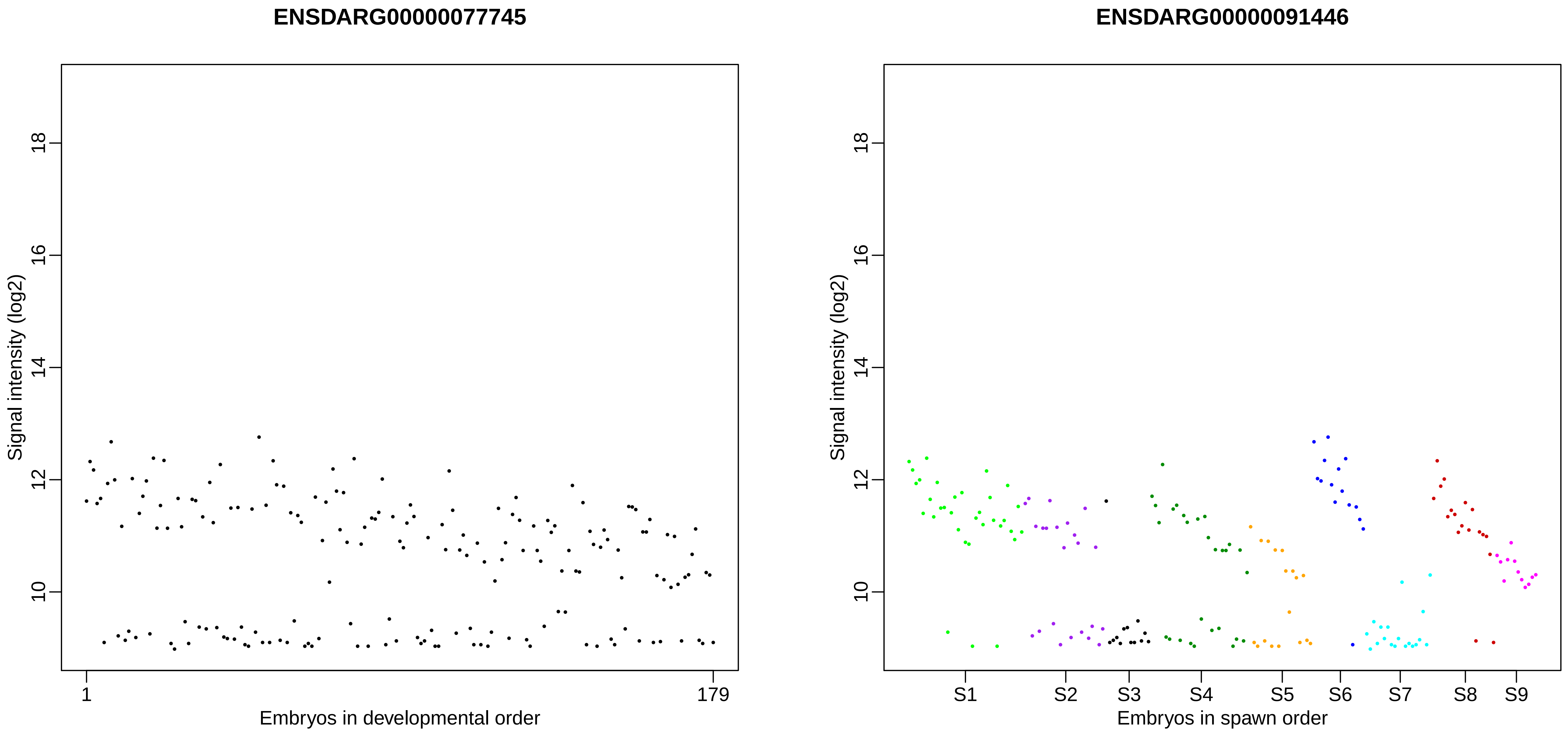

ENSDARG00000077745
Signal intensity (log2)
18
16
14
12
10
1
179
Embryos in developmental order
ENSDARG00000091446
Signal intensity (log2)
18
16
14
12
10
S1
S2
S3
S4
S5
S6
S7
S8
S9
Embryos in spawn order

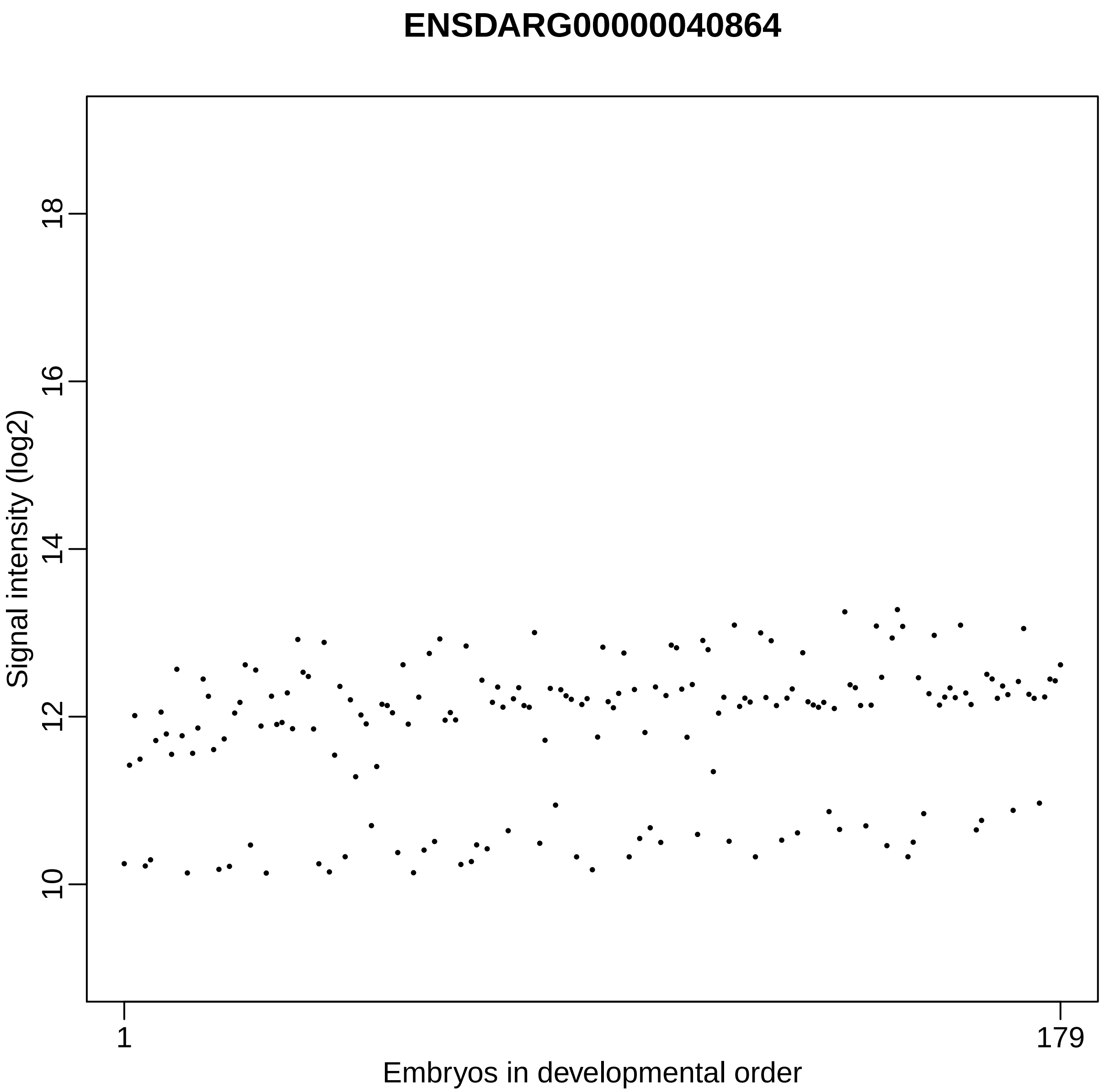
ENSDARG00000040864
Signal intensity (log2)
10
12
14
16
18
1
179
Embryos in developmental order

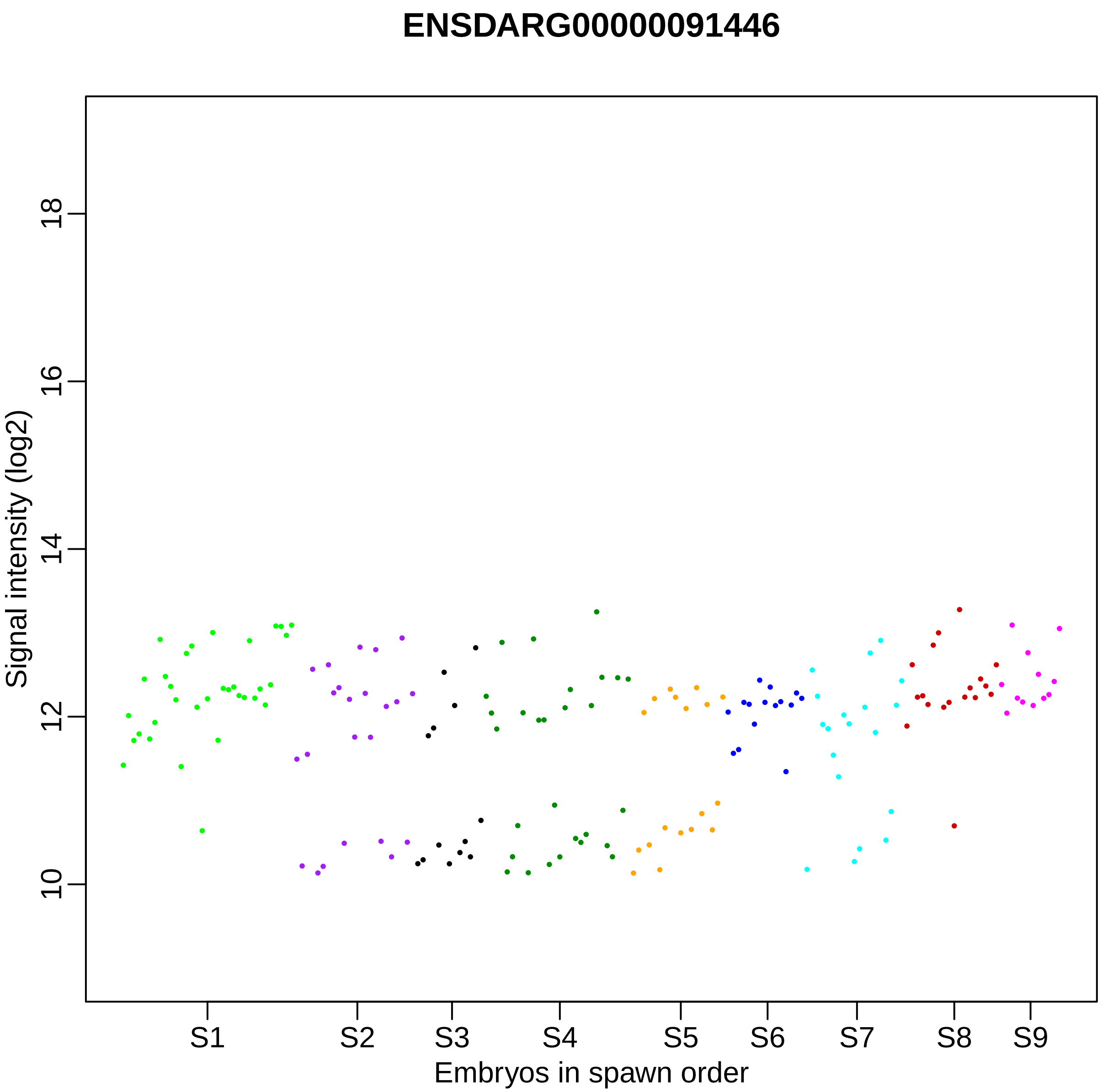
ENSDARG00000091446
Signal intensity (log2)
10
12
14
16
18
S1
S2
S3
S4
S5
S6
S7
S8
S9
Embryos in spawn order

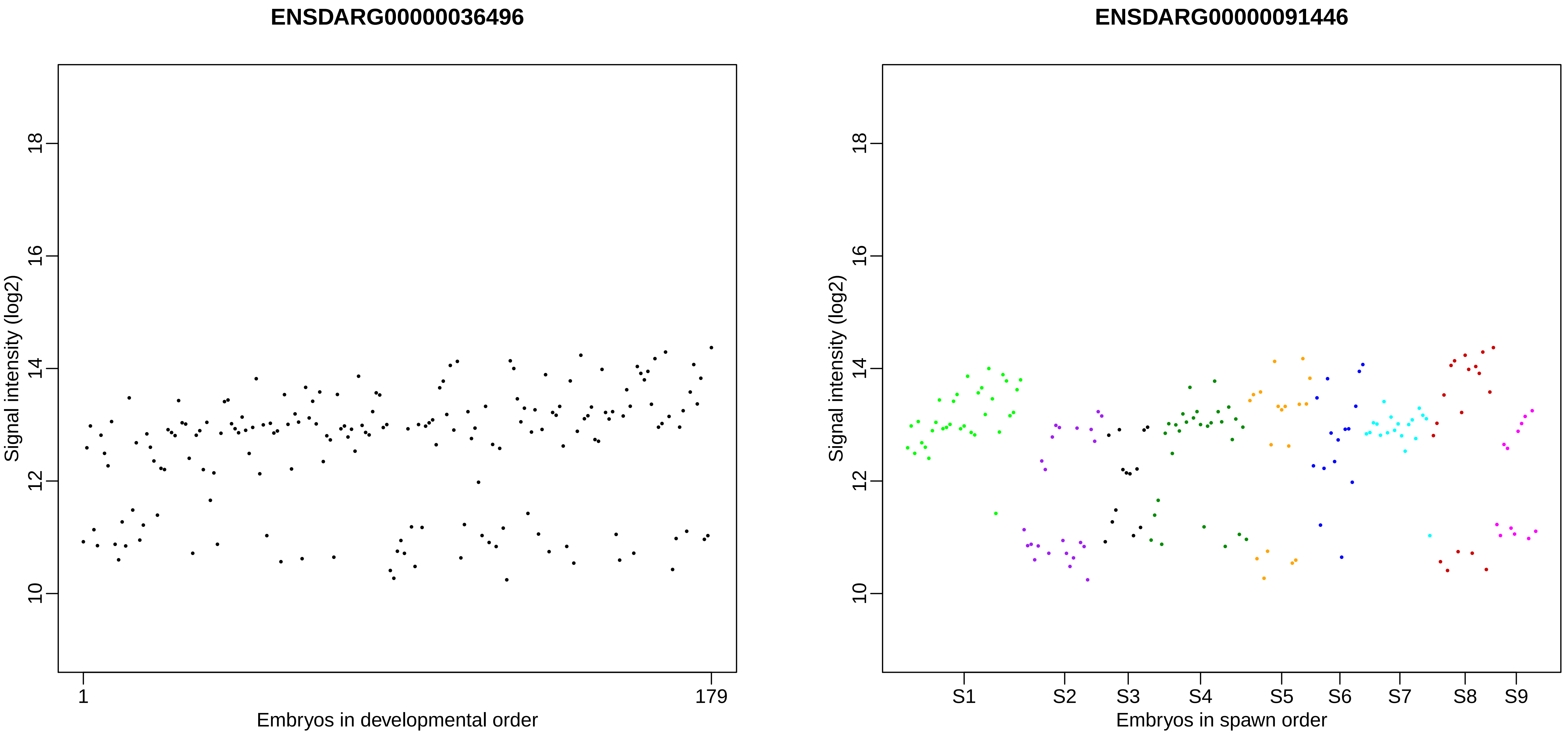
ENSDARG00000036496
Signal intensity (log2)
10
12
14
16
18
1
179
Embryos in developmental order
ENSDARG00000091446
Signal intensity (log2)
10
12
14
16
18
S1
S2
S3
S4
S5
S6
S7
S8
S9
Embryos in spawn order

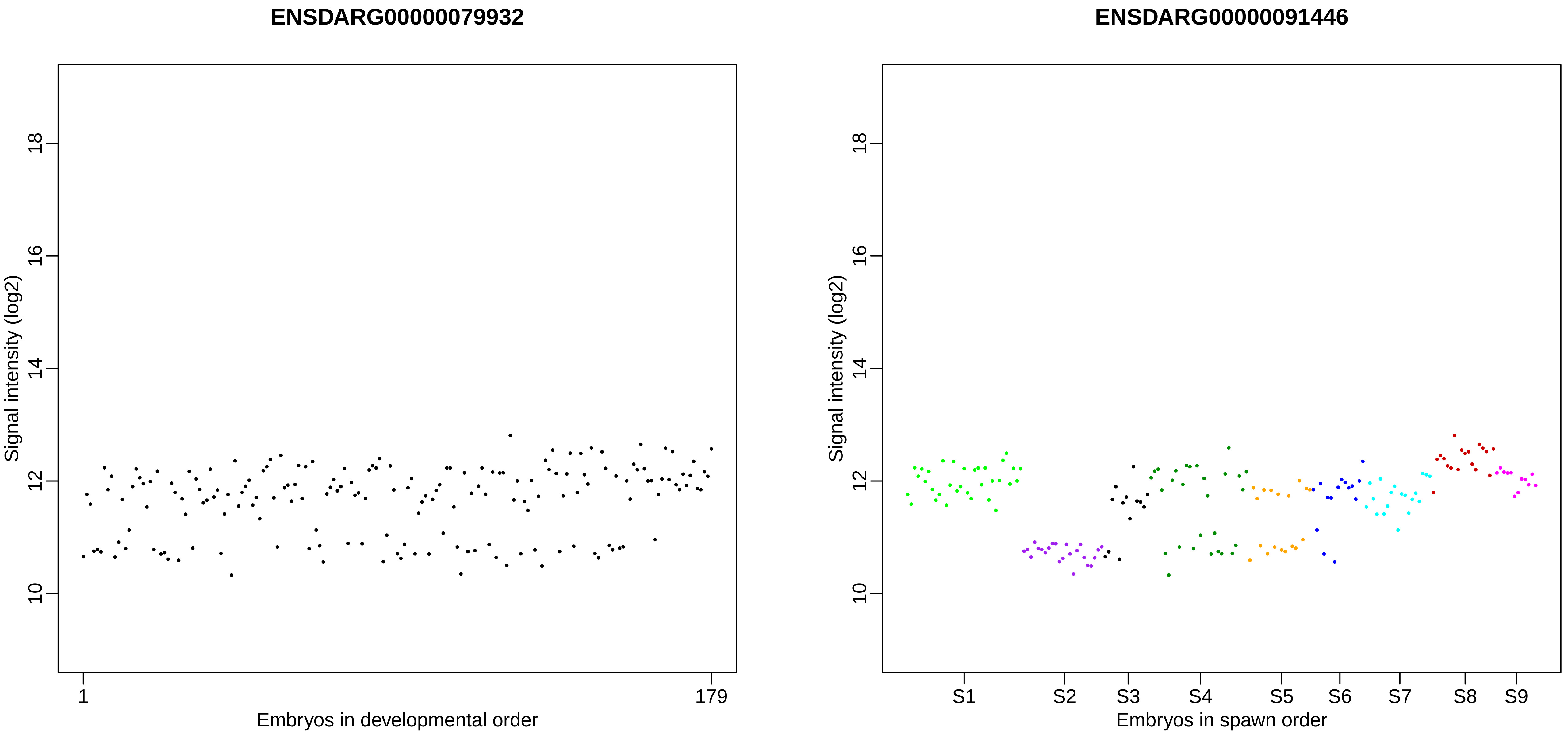
ENSDARG00000079932
Signal intensity (log2)
10
12
14
16
18
1
179
Embryos in developmental order
ENSDARG00000091446
Signal intensity (log2)
10
12
14
16
18
S1
S2
S3
S4
S5
S6
S7
S8
S9
Embryos in spawn order

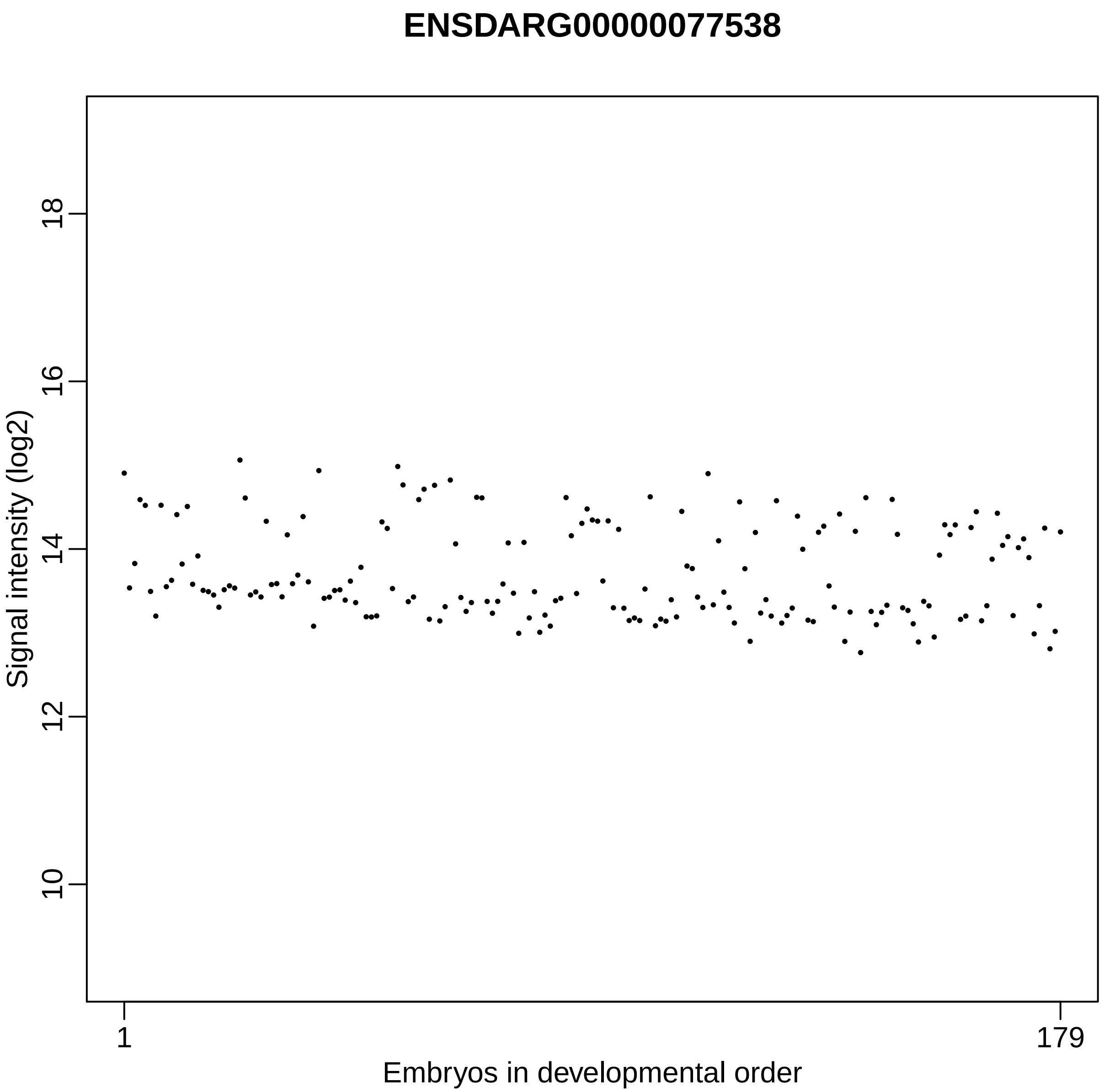

ENSDARG00000077538
Signal intensity (log2)
18
16
14
12
10
1
179
Embryos in developmental order

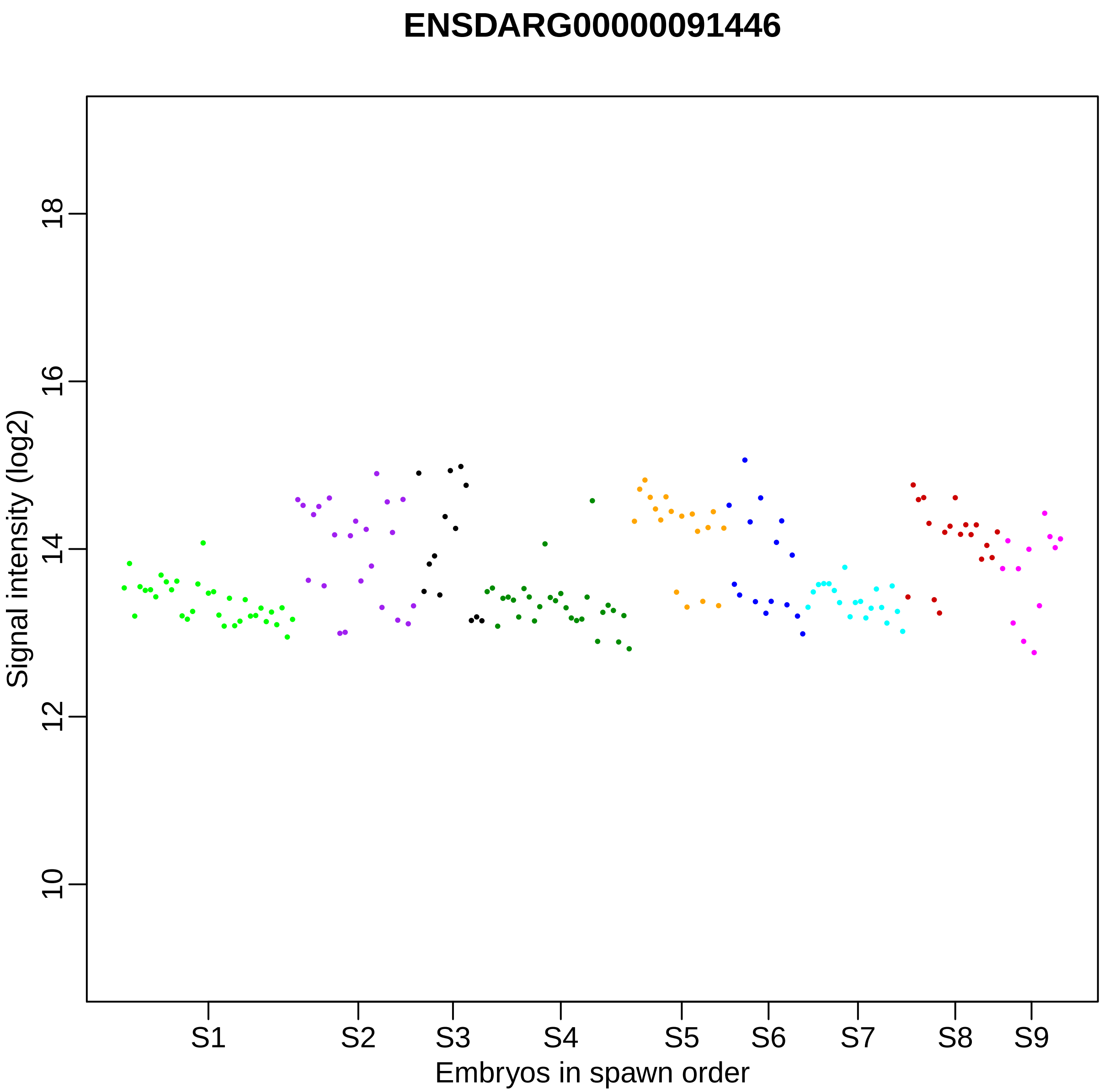

ENSDARG00000091446
Signal intensity (log2)
18
16
14
12
10
S1
S2
S3
S4
S5
S6
S7
S8
S9
Embryos in spawn order

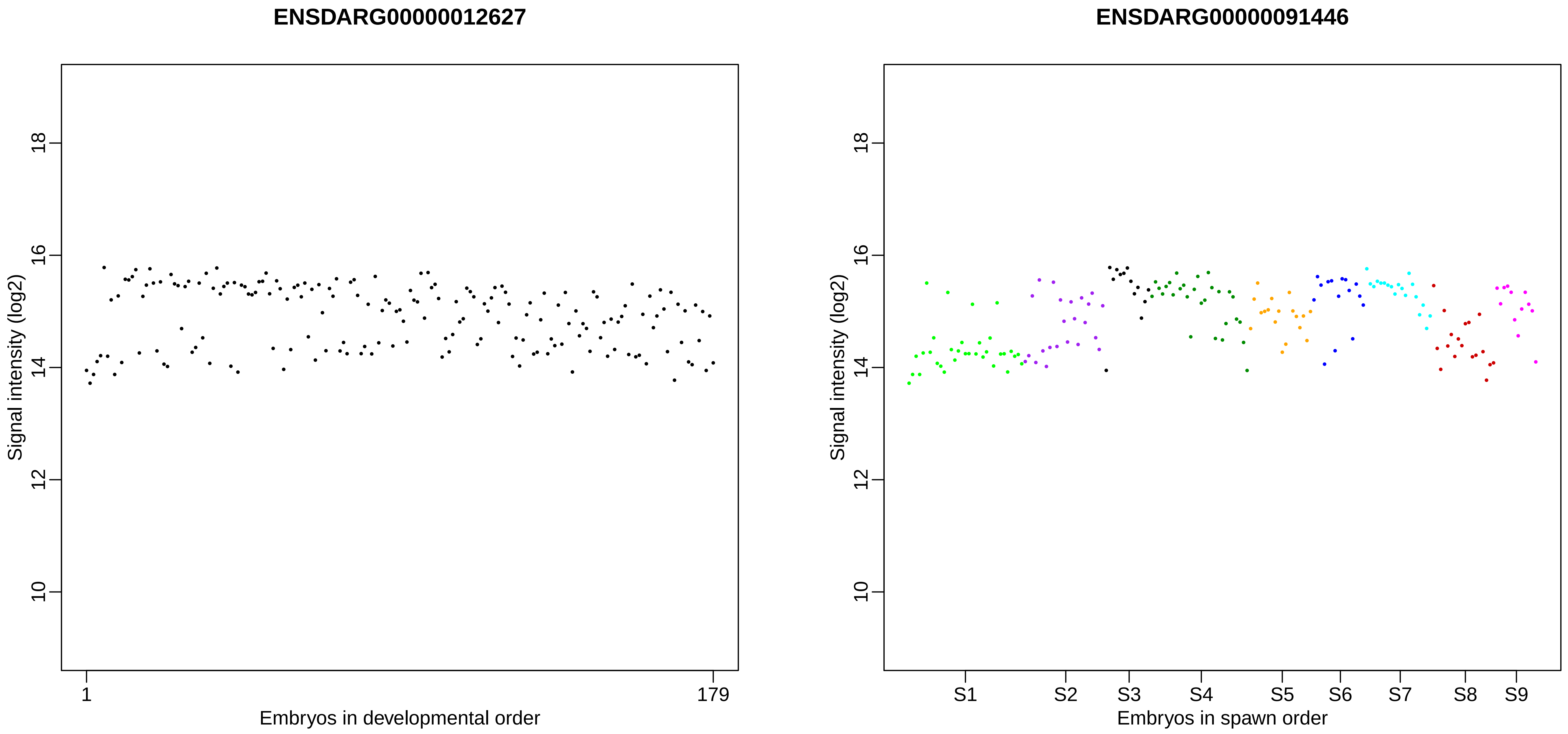
ENSDARG00000012627
ENSDARG00000091446
Signal intensity (log2)
Signal intensity (log2)
18
16
14
12
10
18
16
14
12
10
1
179
Embryos in developmental order
S1
S2
S3
S4
S5
S6
S7
S8
S9
Embryos in spawn order

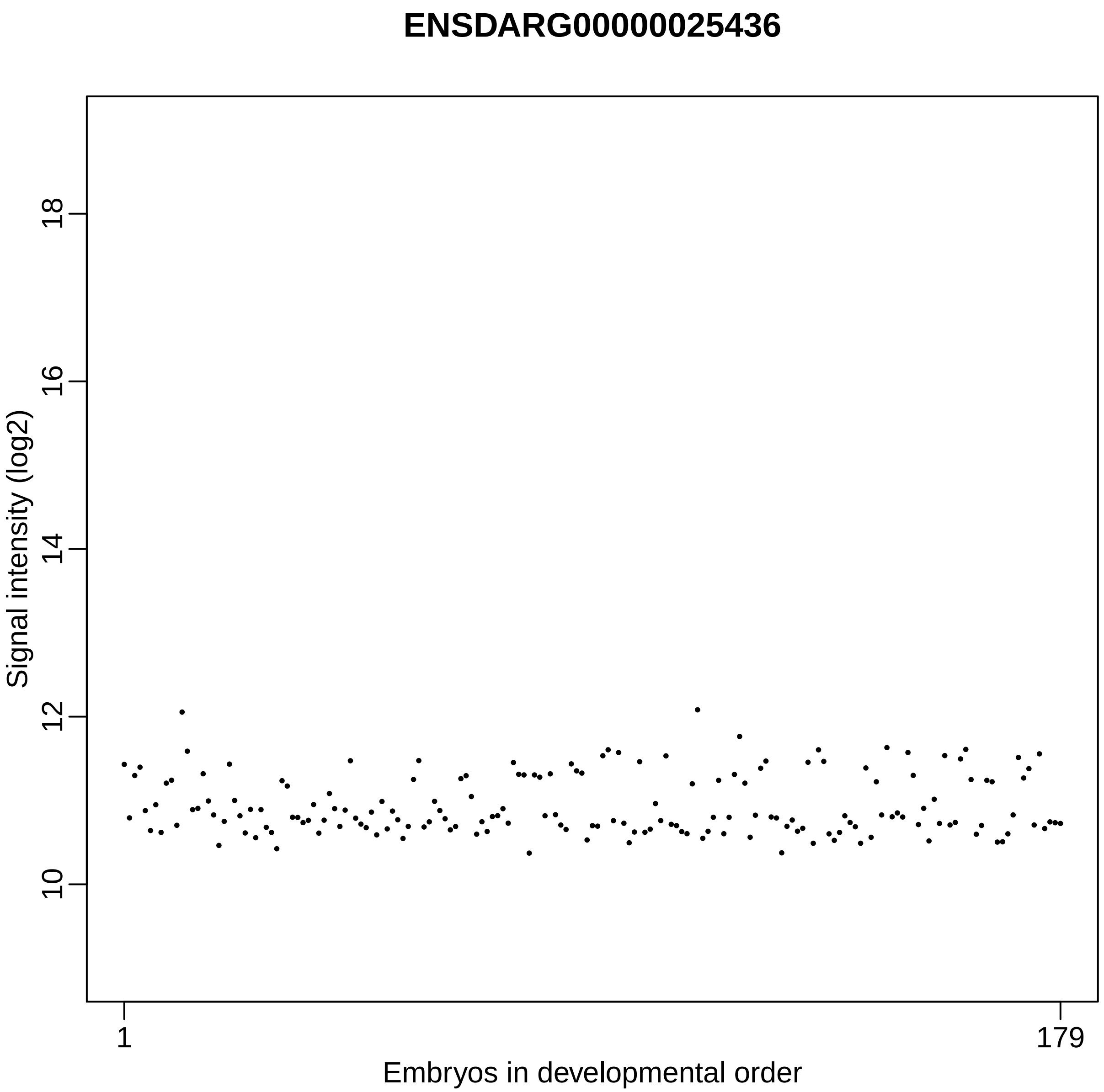
ENSDARG00000025436
Signal intensity (log2)
18
16
14
12
10
1
179
Embryos in developmental order

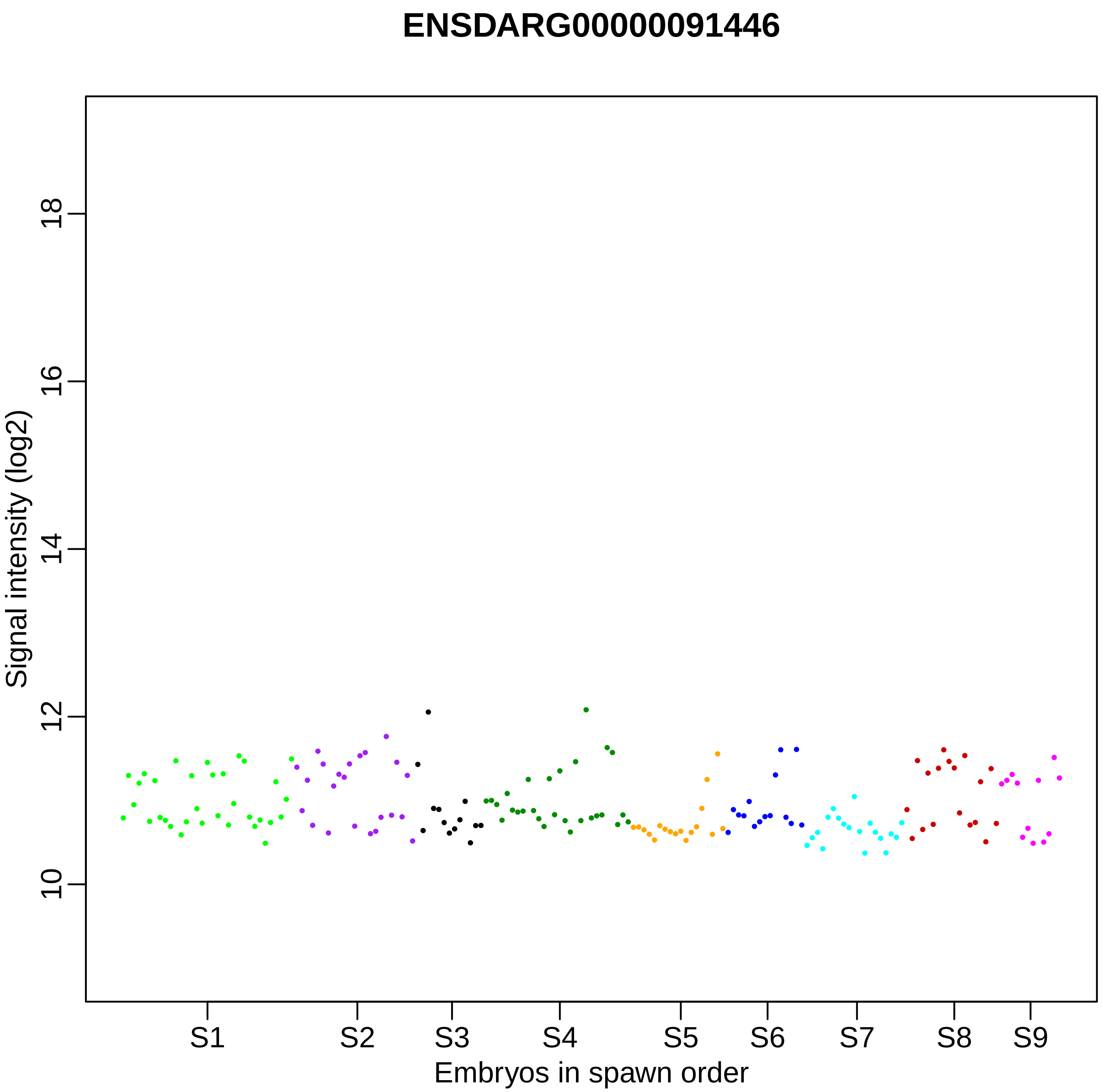
ENSDARG00000091446
Signal intensity (log2)
18
16
14
12
10
S1
S2
S3
S4
S5
S6
S7
S8
S9
Embryos in spawn order

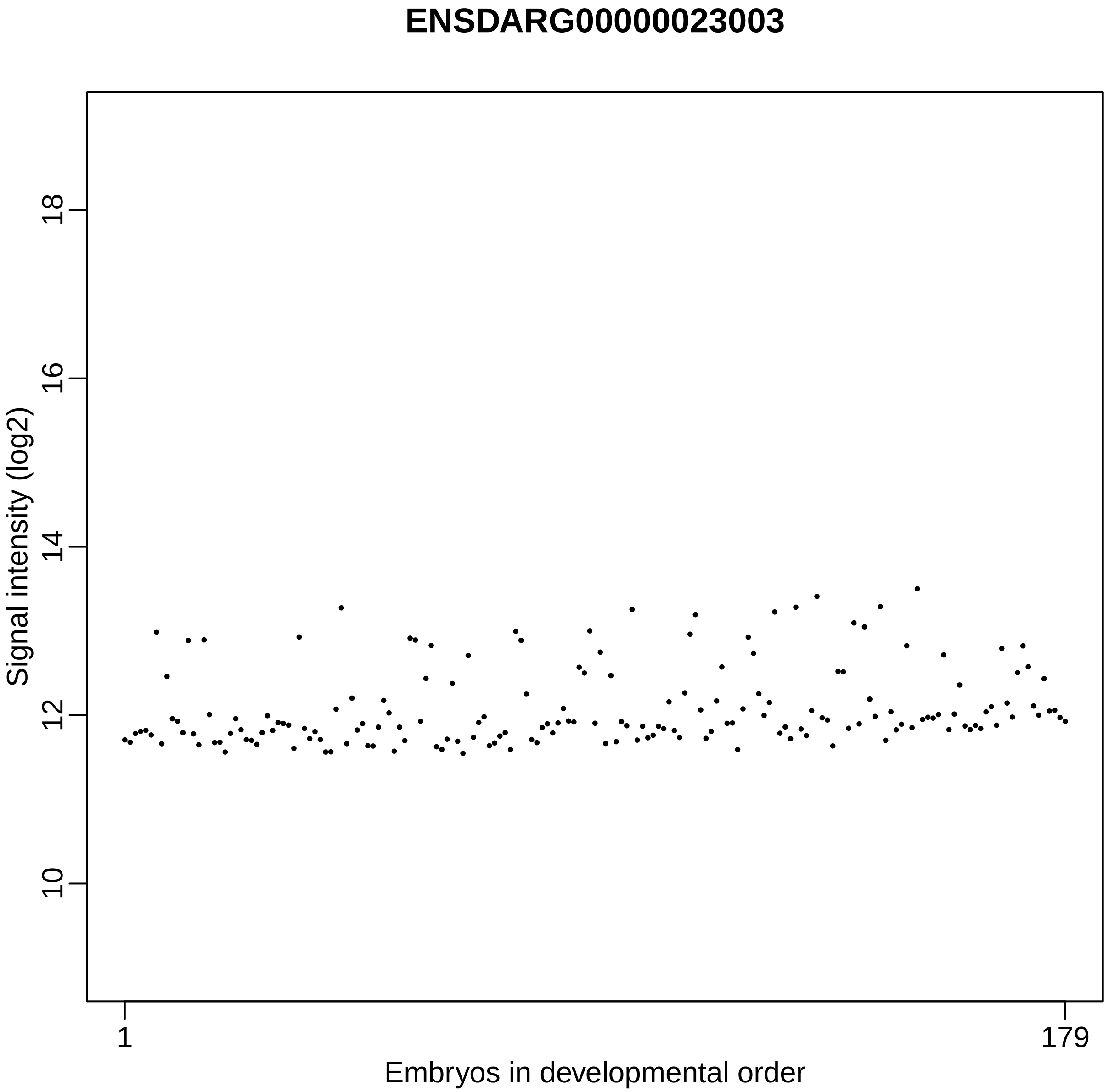
ENSDARG00000023003
Signal intensity (log2)
10
12
14
16
18
1
179
Embryos in developmental order

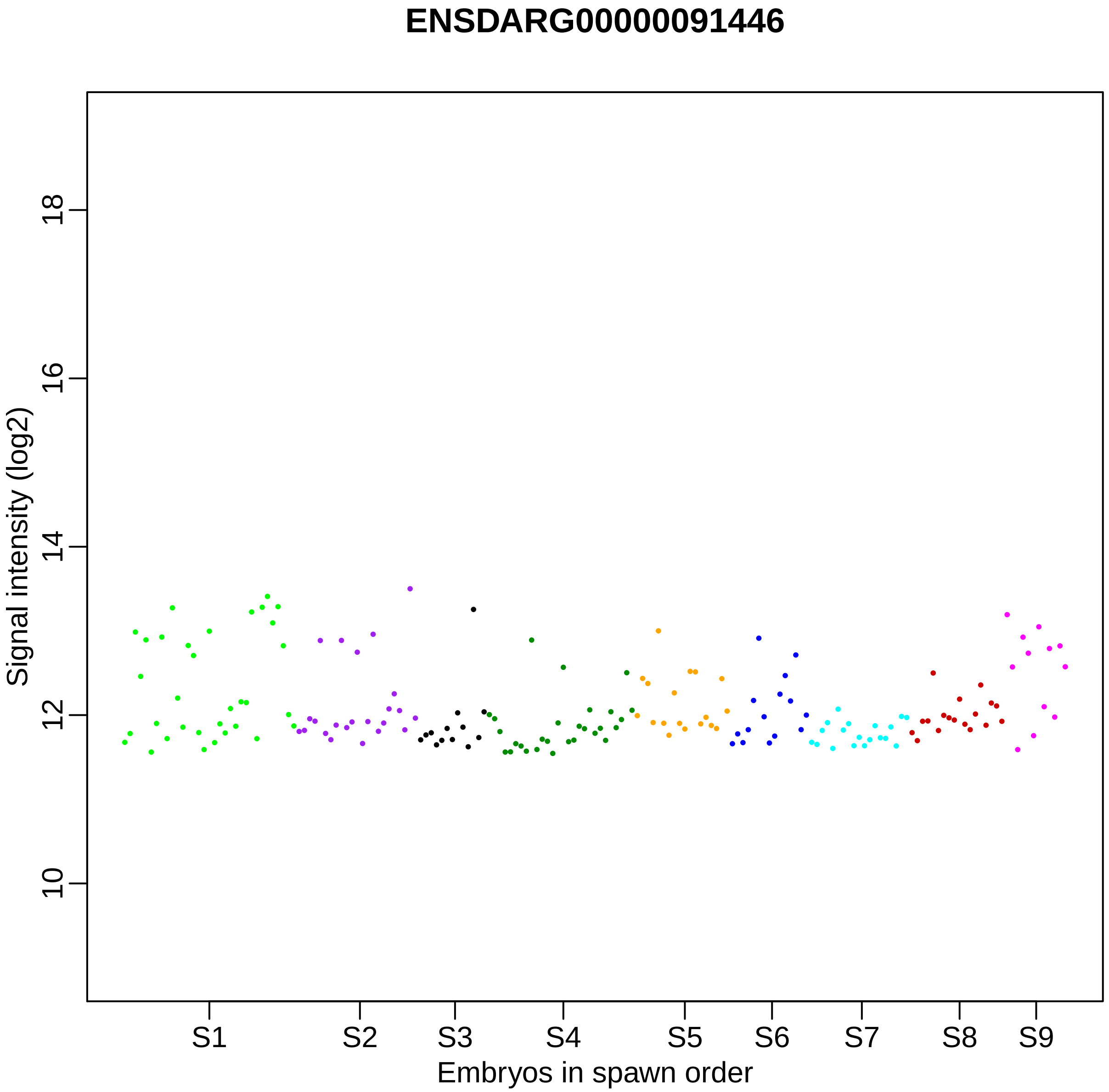
ENSDARG00000091446
Signal intensity (log2)
10
12
14
16
18
S1
S2
S3
S4
S5
S6
S7
S8
S9
Embryos in spawn order

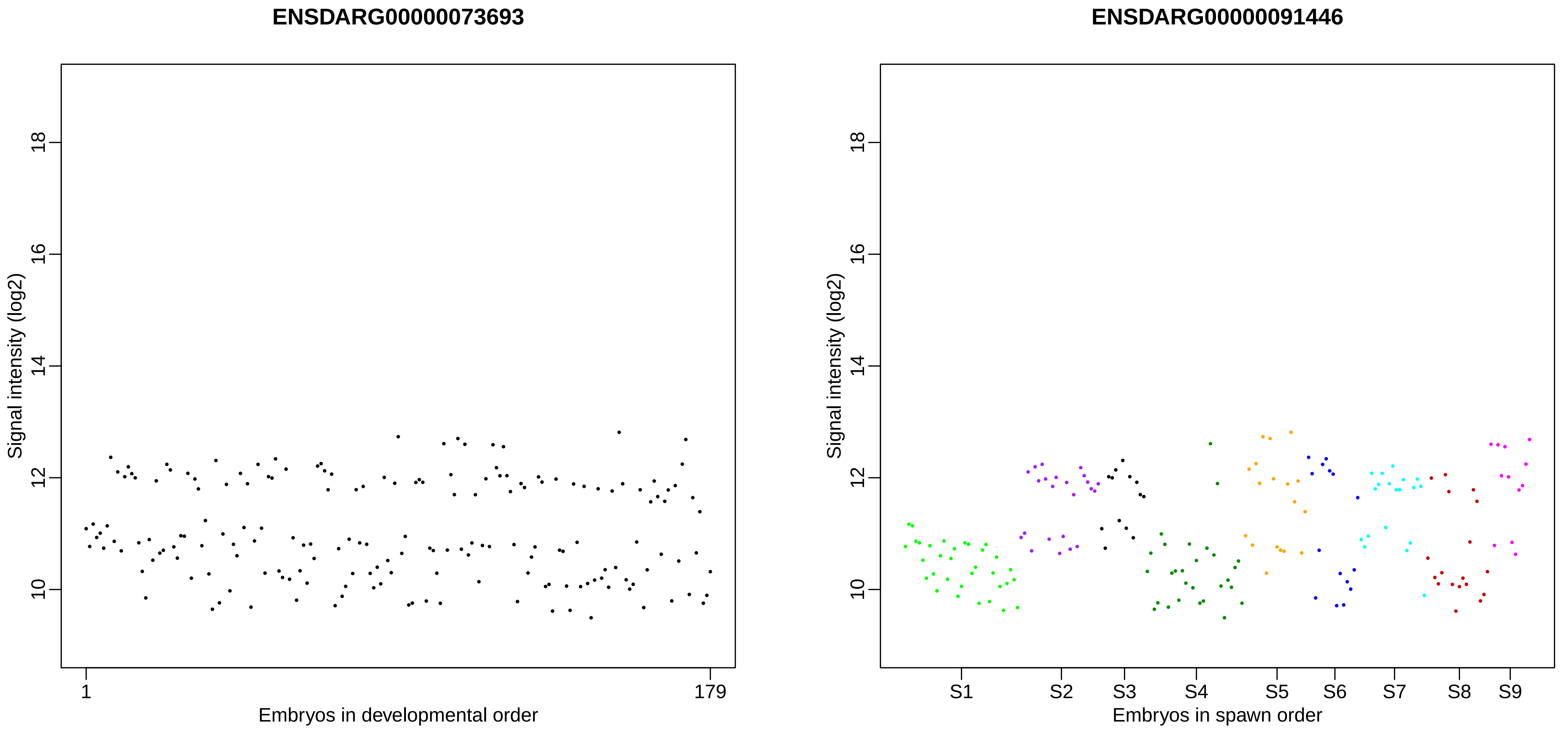
ENSDARG00000073693
Signal intensity (log2)
10
12
14
16
18
1
179
Embryos in developmental order
ENSDARG00000091446
Signal intensity (log2)
10
12
14
16
18
S1
S2
S3
S4
S5
S6
S7
S8
S9
Embryos in spawn order

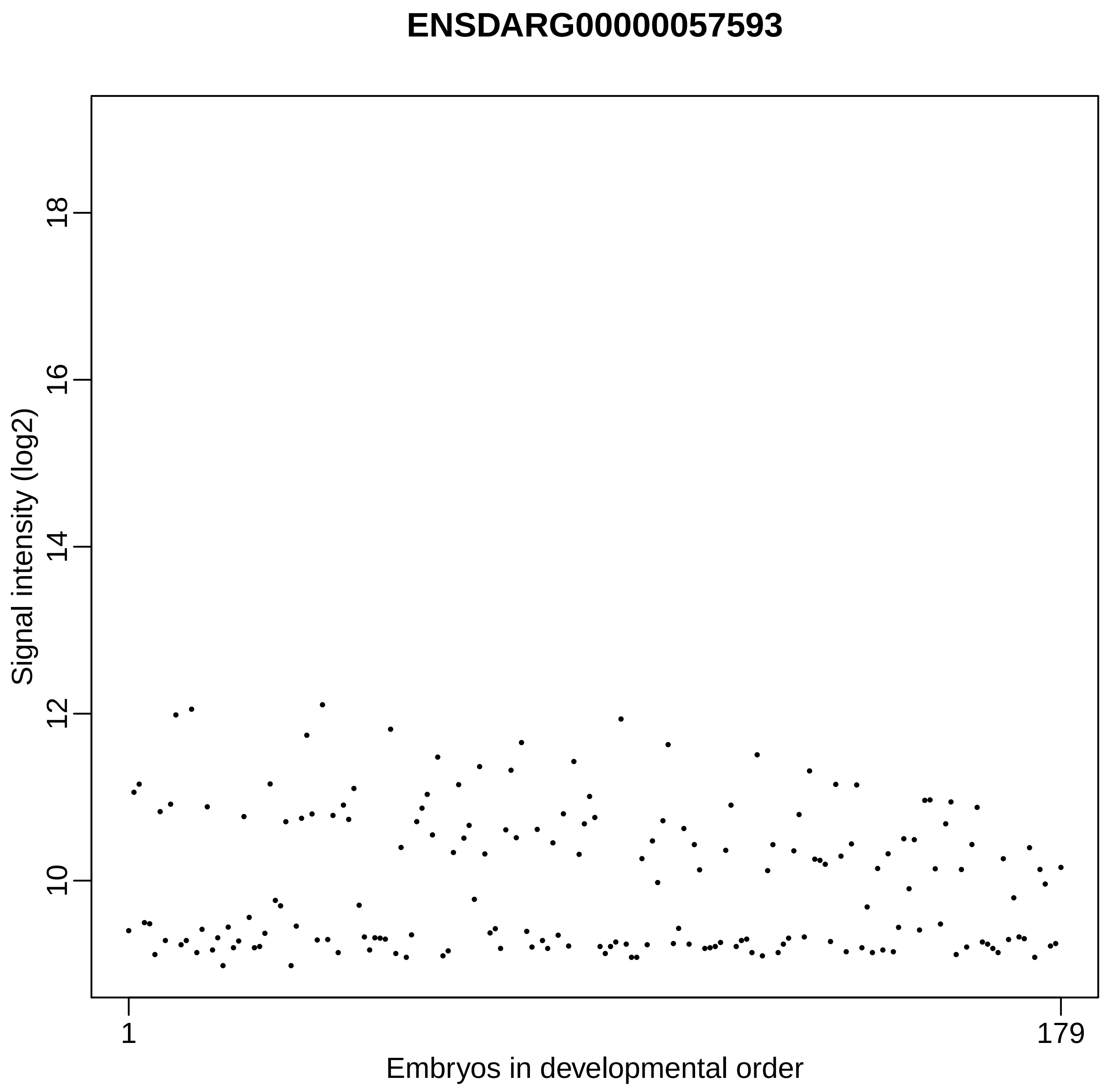
ENSDARG00000057593
Signal intensity (log2)
10
12
14
16
18
1
179
Embryos in developmental order

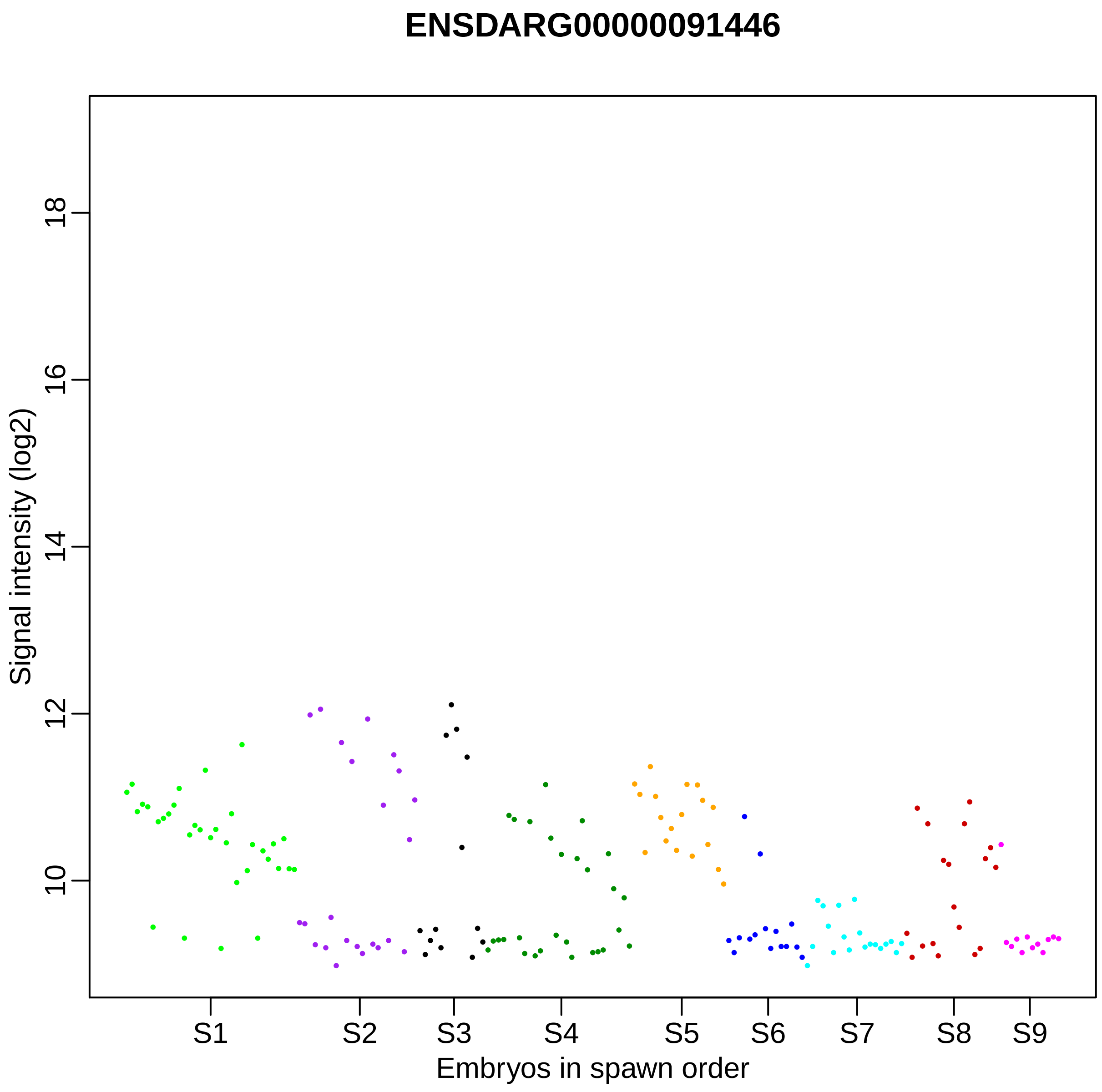
ENSDARG00000091446
Signal intensity (log2)
10
12
14
16
18
S1
S2
S3
S4
S5
S6
S7
S8
S9
Embryos in spawn order

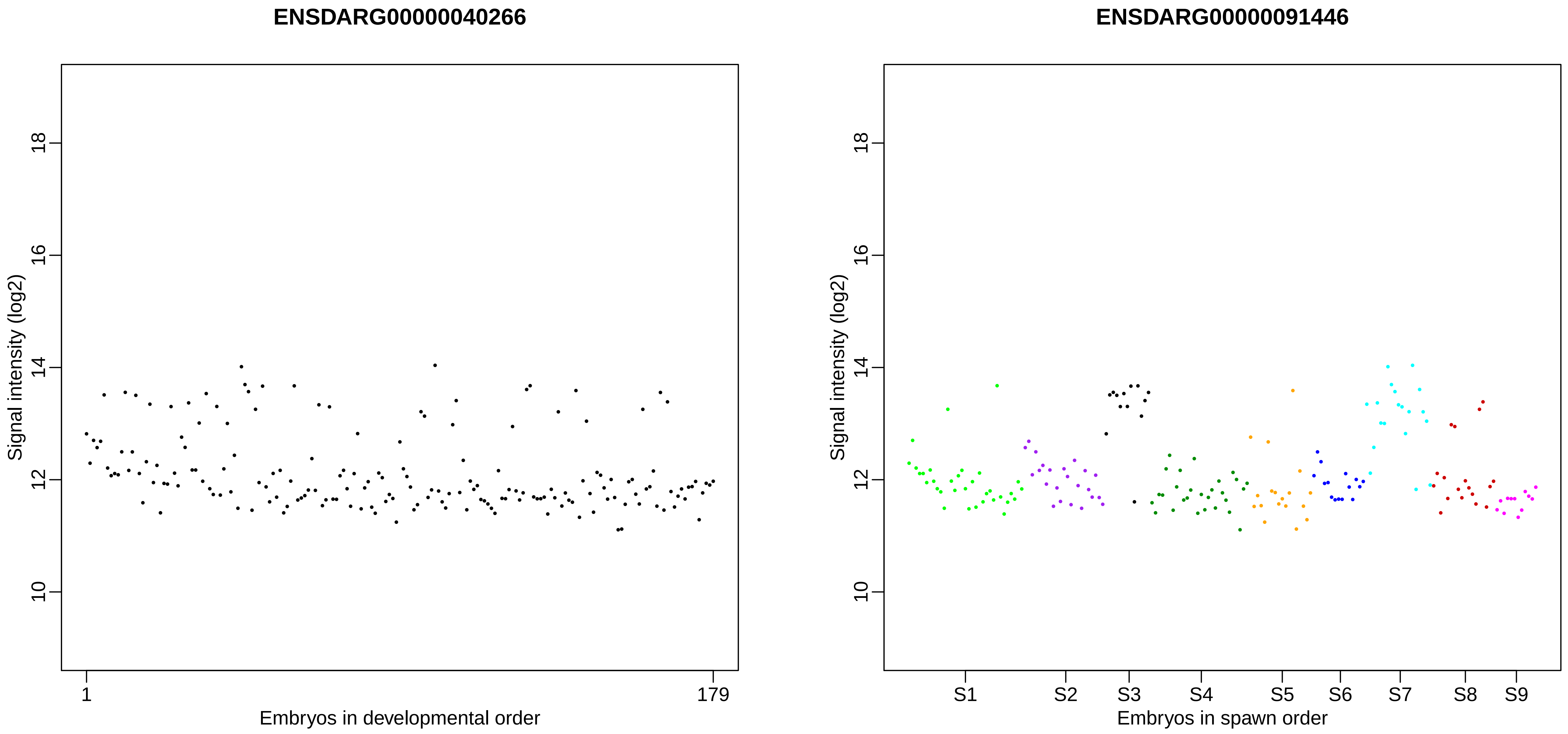
ENSDARG00000040266
Signal intensity (log2)
10
12
14
16
18
1
179
Embryos in developmental order
ENSDARG00000091446
Signal intensity (log2)
10
12
14
16
18
S1
S2
S3
S4
S5
S6
S7
S8
S9
Embryos in spawn order

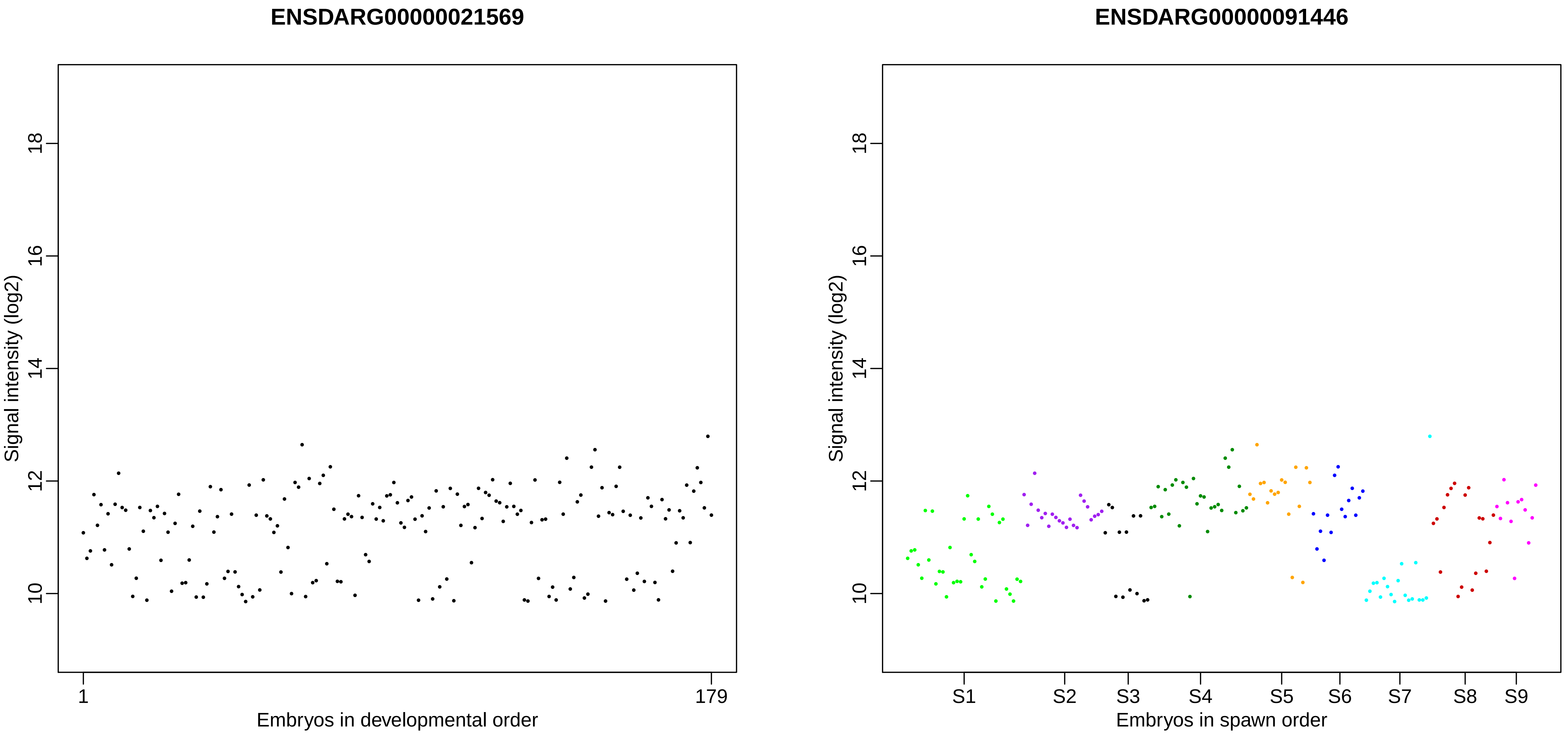

ENSDARG00000021569
Signal intensity (log2)
18
16
14
12
10
1
179
Embryos in developmental order
ENSDARG00000091446
Signal intensity (log2)
18
16
14
12
10
S1
S2
S3
S4
S5
S6
S7
S8
S9
Embryos in spawn order

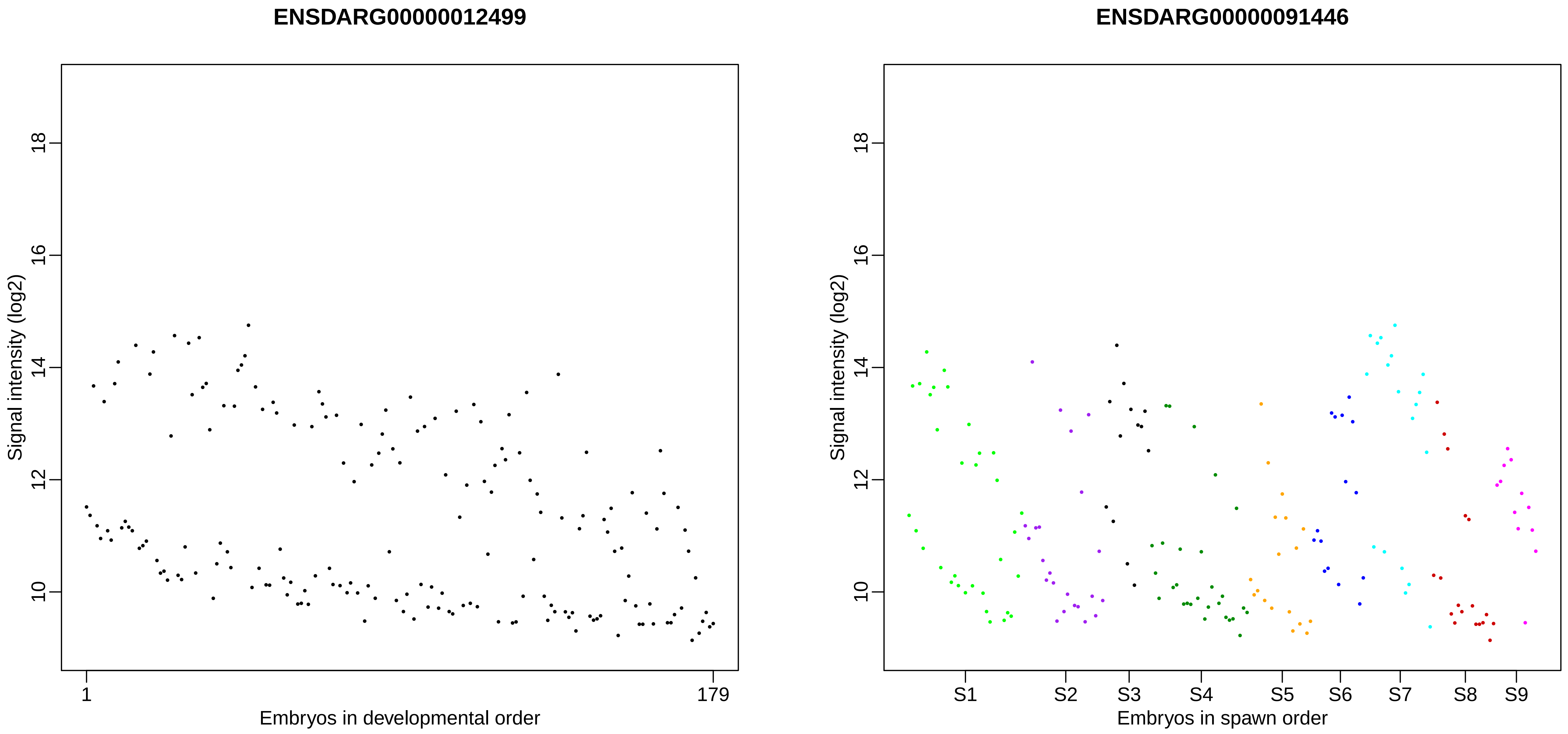
ENSDARG00000012499
Signal intensity (log2)
10
12
14
16
18
1
179
Embryos in developmental order
ENSDARG00000091446
Signal intensity (log2)
10
12
14
16
18
S1
S2
S3
S4
S5
S6
S7
S8
S9
Embryos in spawn order

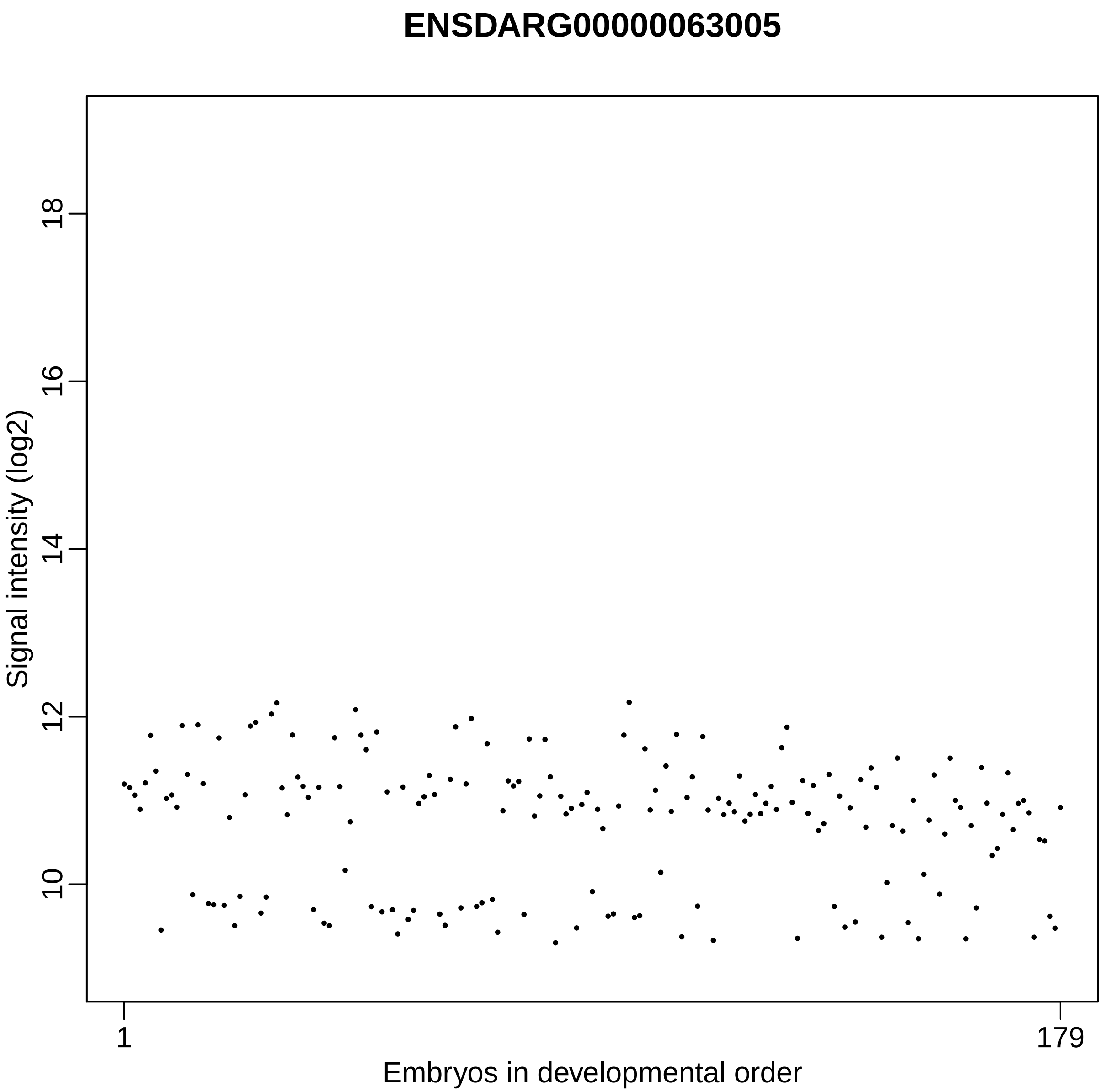
ENSDARG00000063005
Signal intensity (log2)
10
12
14
16
18
1
179
Embryos in developmental order

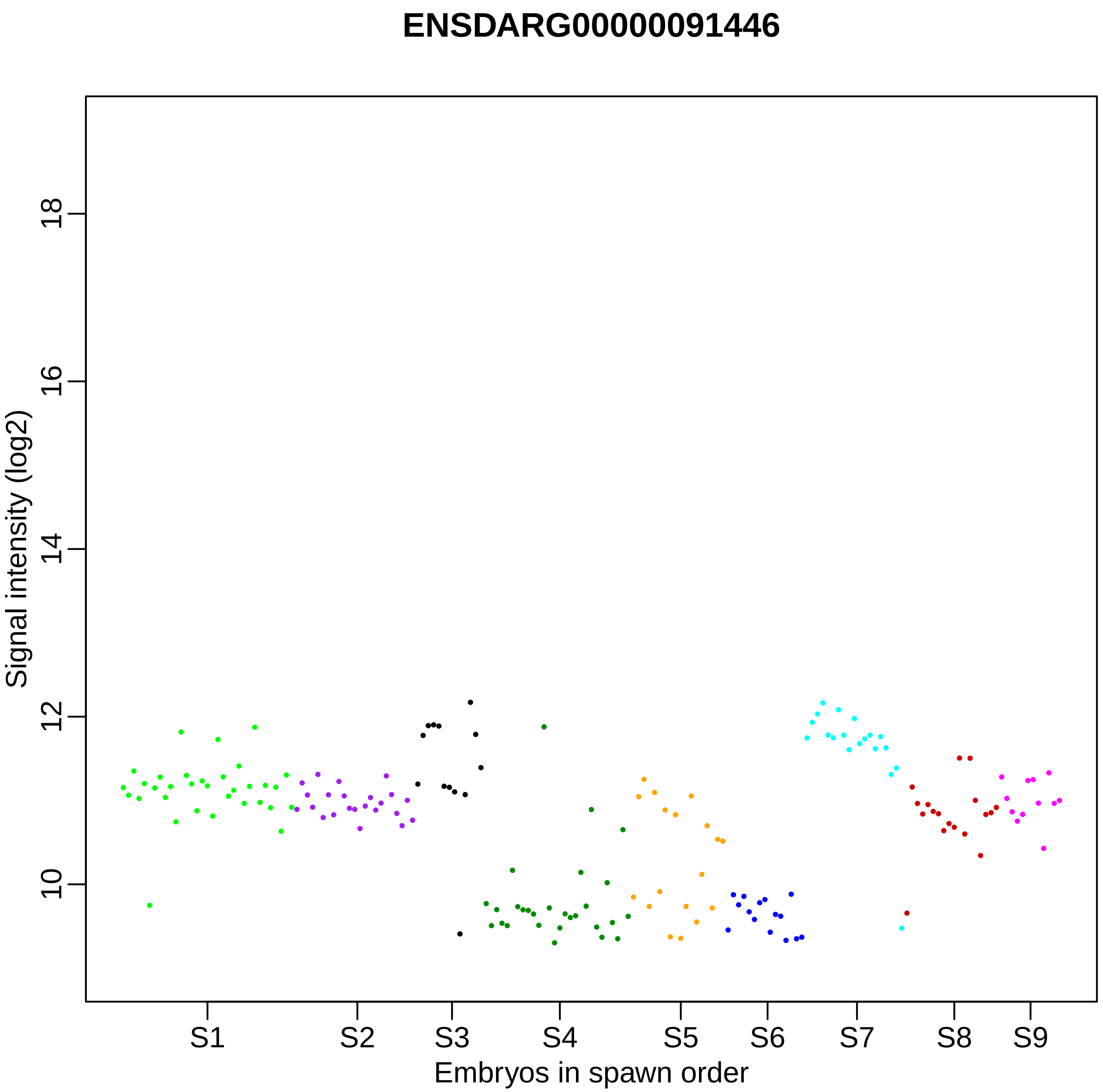
ENSDARG00000091446
Signal intensity (log2)
10
12
14
16
18
S1
S2
S3
S4
S5
S6
S7
S8
S9
Embryos in spawn order

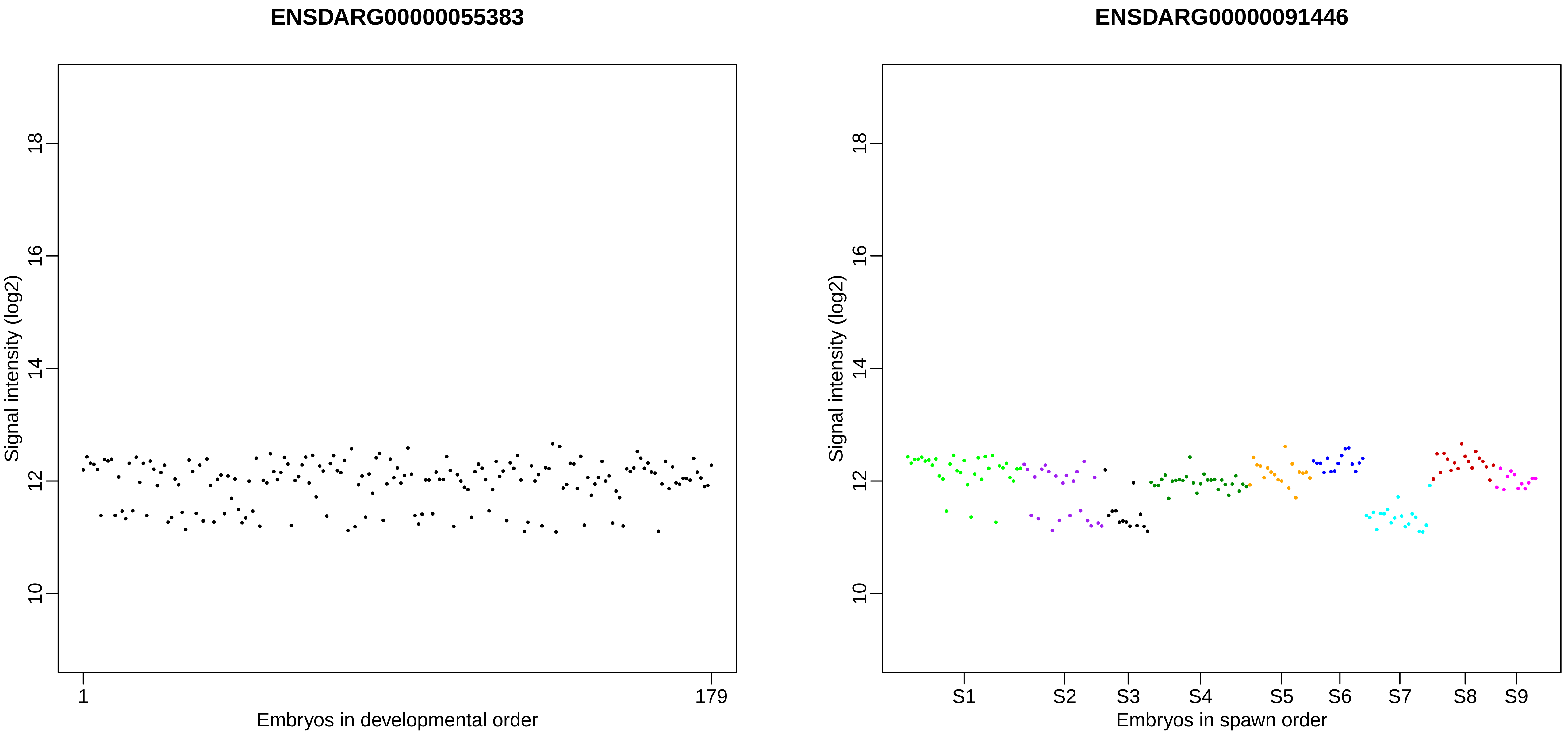

ENSDARG00000055383
Signal intensity (log2)
10
12
14
16
18
1
179
Embryos in developmental order
ENSDARG00000091446
Signal intensity (log2)
10
12
14
16
18
S1
S2
S3
S4
S5
S6
S7
S8
S9
Embryos in spawn order

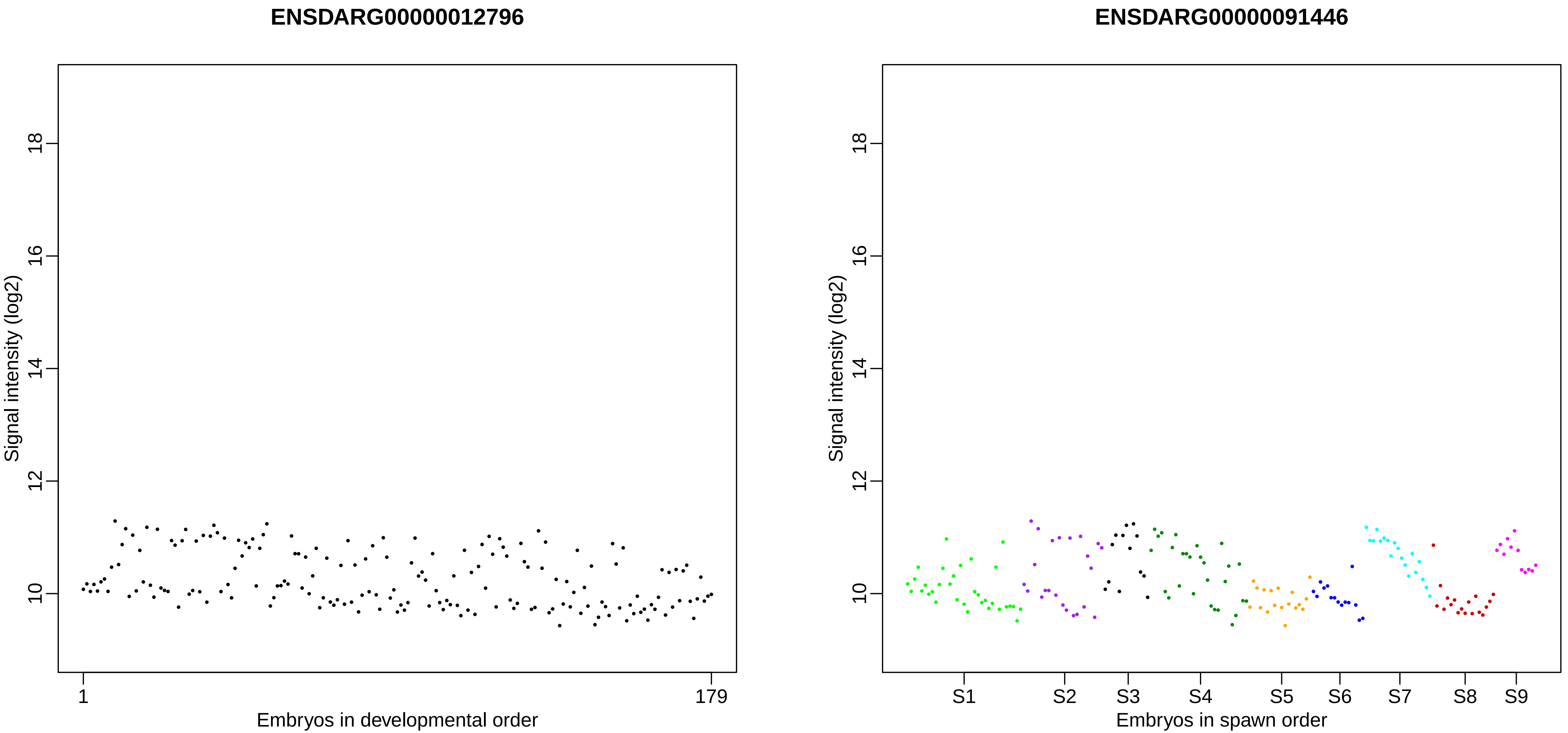
ENSDARG00000012796
Signal intensity (log2)
10
12
14
16
18
1
179
Embryos in developmental order
ENSDARG00000091446
Signal intensity (log2)
10
12
14
16
18
S1
S2
S3
S4
S5
S6
S7
S8
S9
Embryos in spawn order

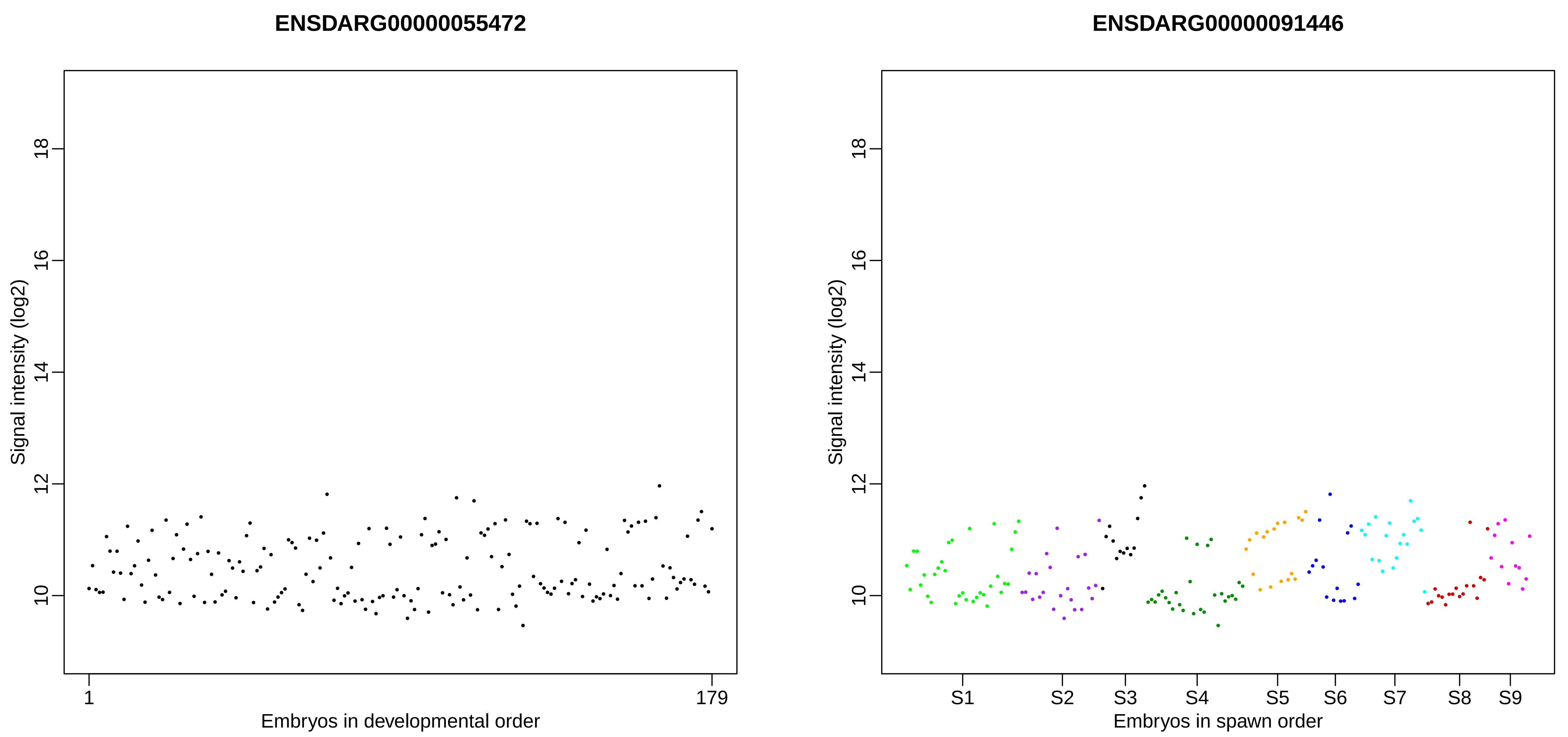

ENSDARG00000055472
Signal intensity (log2)
10
12
14
16
18
1
179
Embryos in developmental order
ENSDARG00000091446
Signal intensity (log2)
10
12
14
16
18
S1
S2
S3
S4
S5
S6
S7
S8
S9
Embryos in spawn order

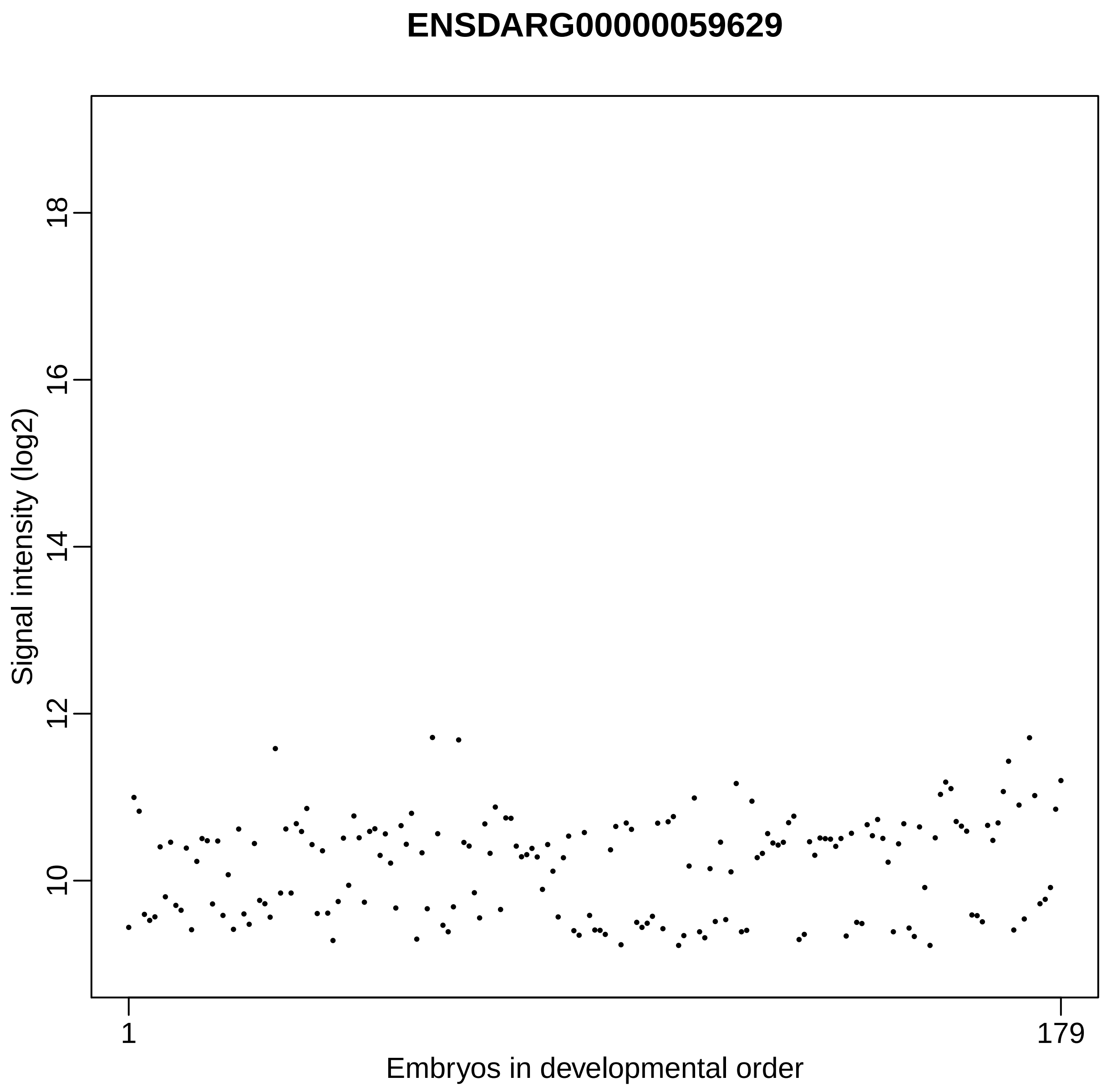
ENSDARG00000059629
Signal intensity (log2)
10
12
14
16
18
1
179
Embryos in developmental order

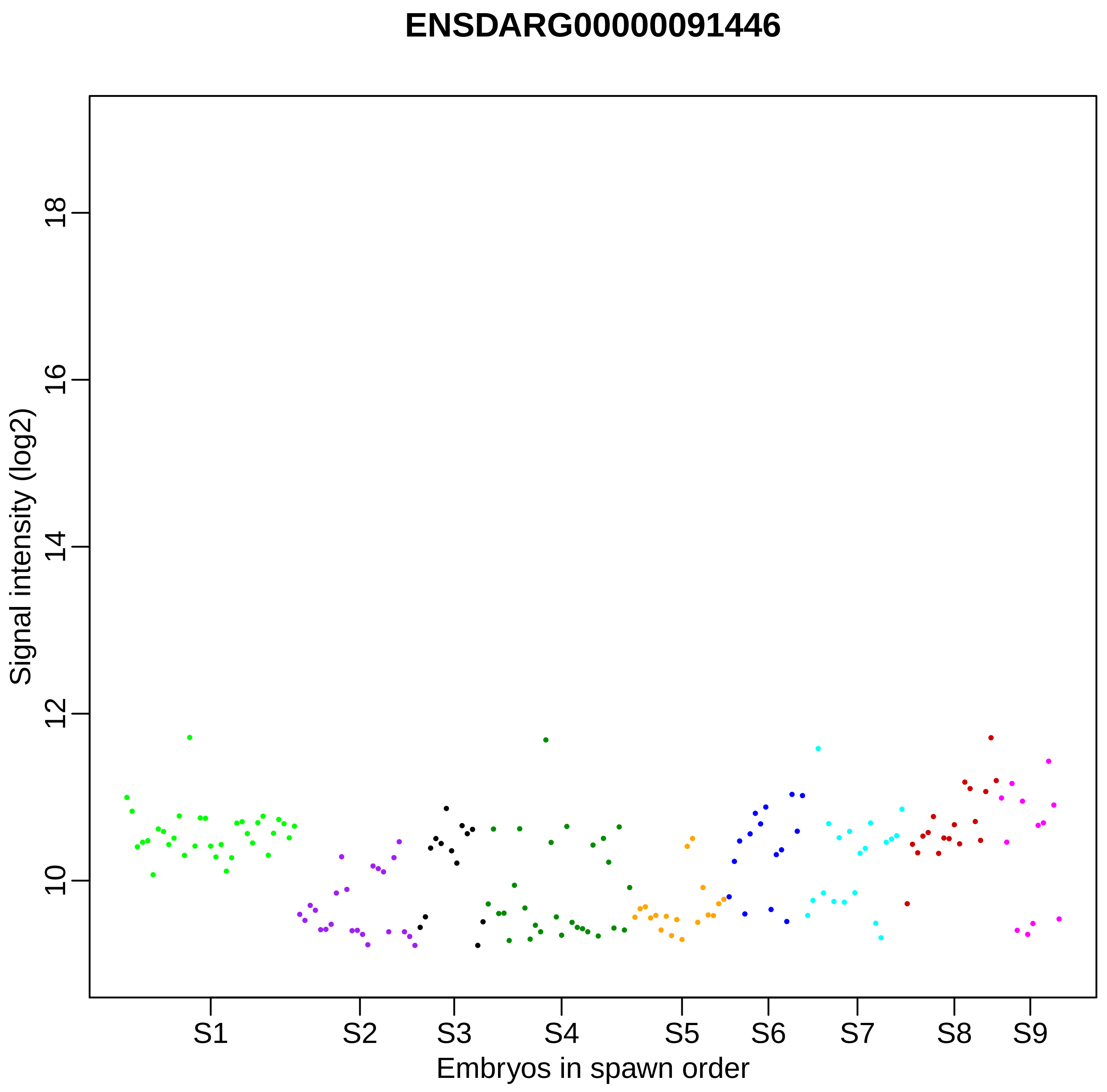
ENSDARG00000091446
Signal intensity (log2)
10
12
14
16
18
S1
S2
S3
S4
S5
S6
S7
S8
S9
Embryos in spawn order

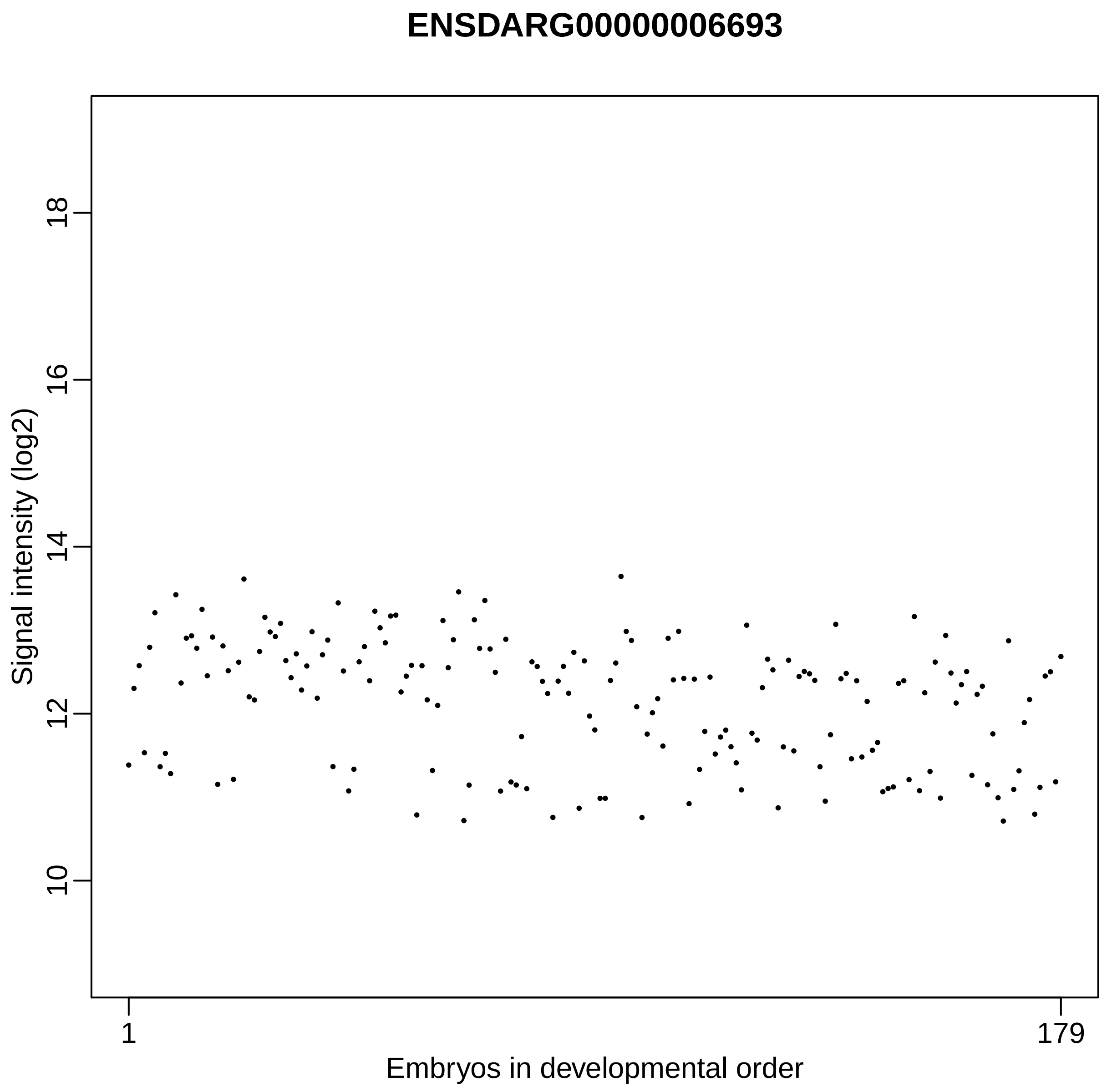

ENSDARG00000006693
Signal intensity (log2)
10
12
14
16
18
1
179
Embryos in developmental order

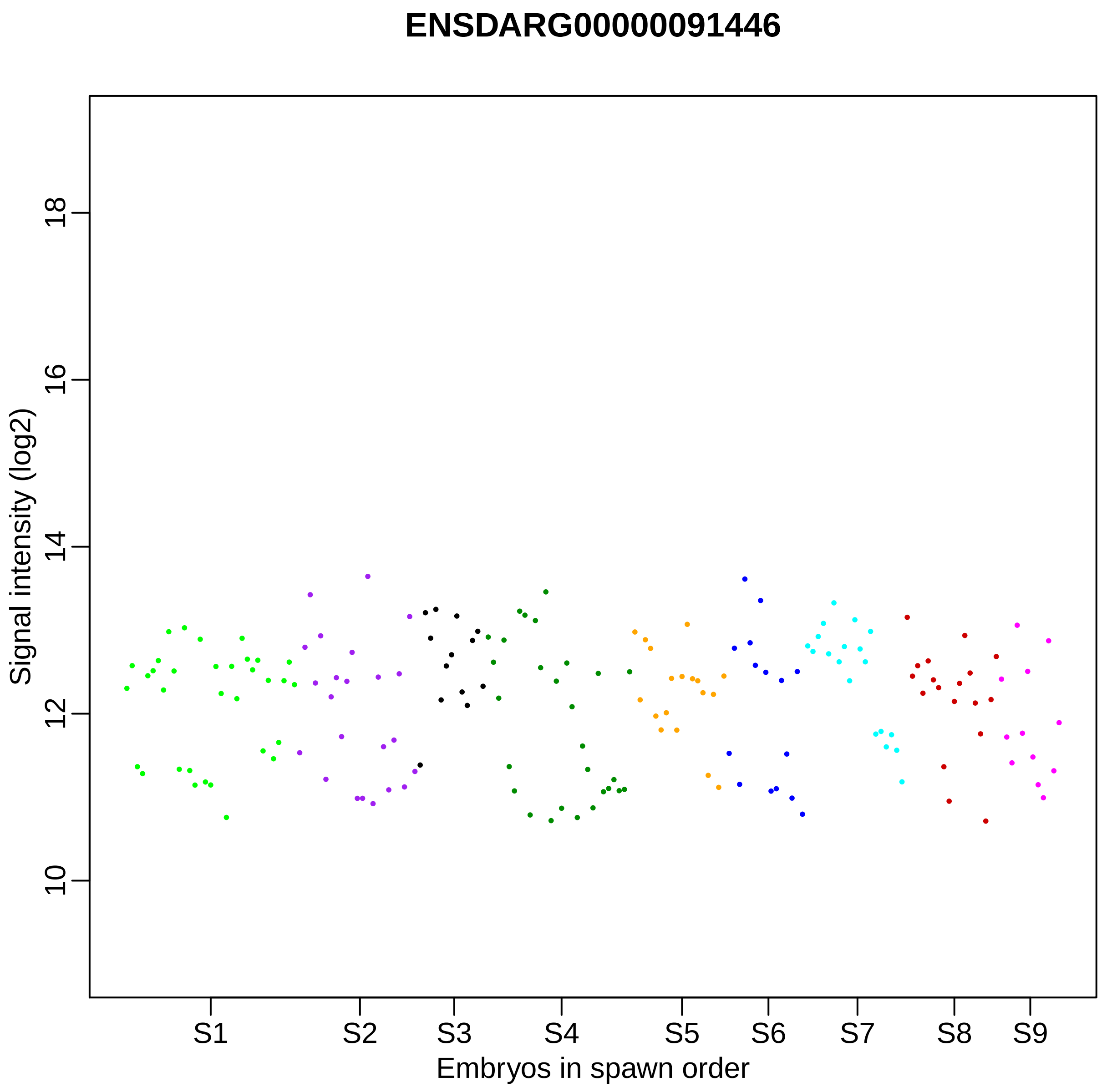

ENSDARG00000091446
Signal intensity (log2)
10
12
14
16
18
S1
S2
S3
S4
S5
S6
S7
S8
S9
Embryos in spawn order

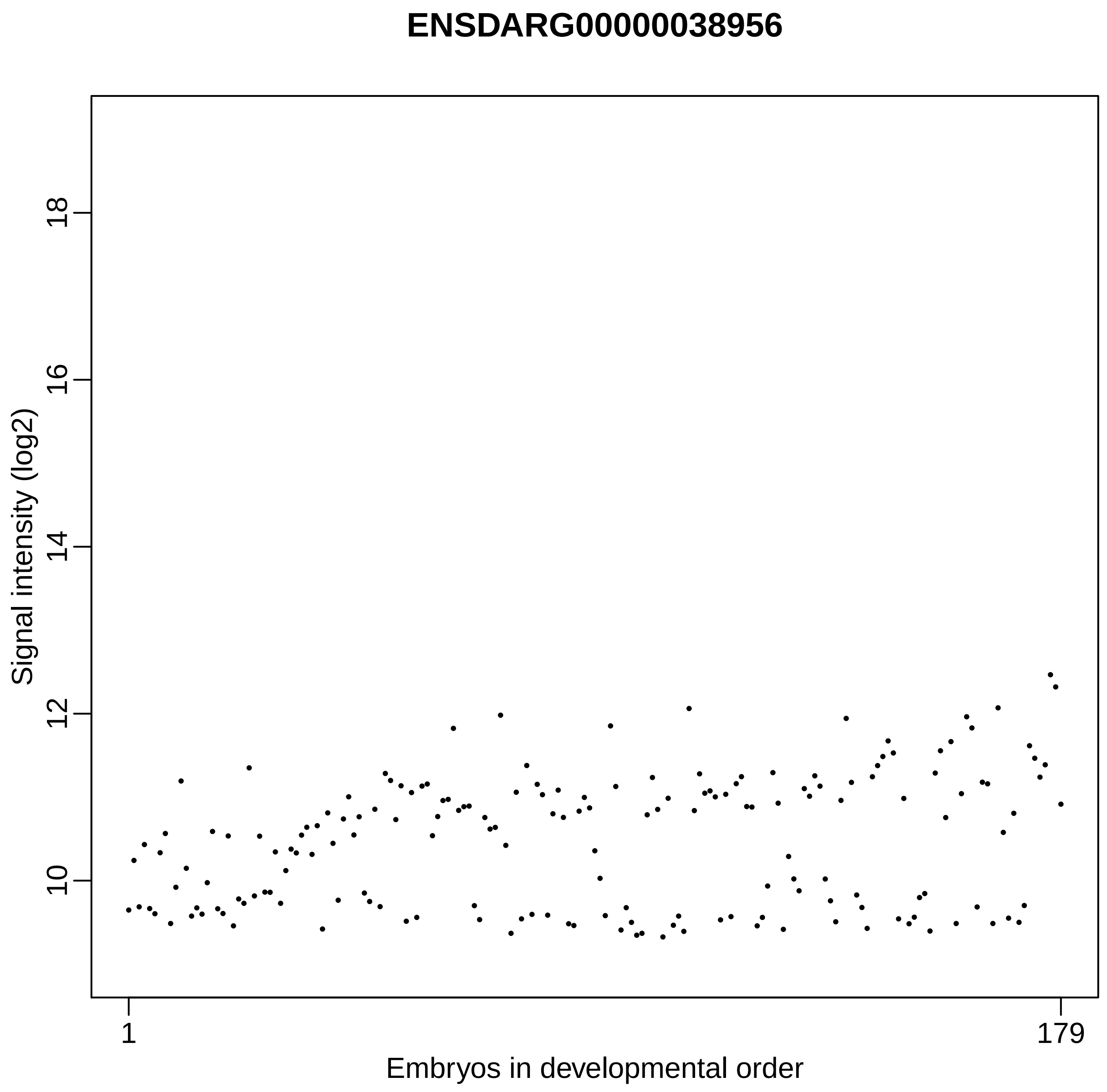
ENSDARG00000038956
Signal intensity (log2)
18
16
14
12
10
1
179
Embryos in developmental order

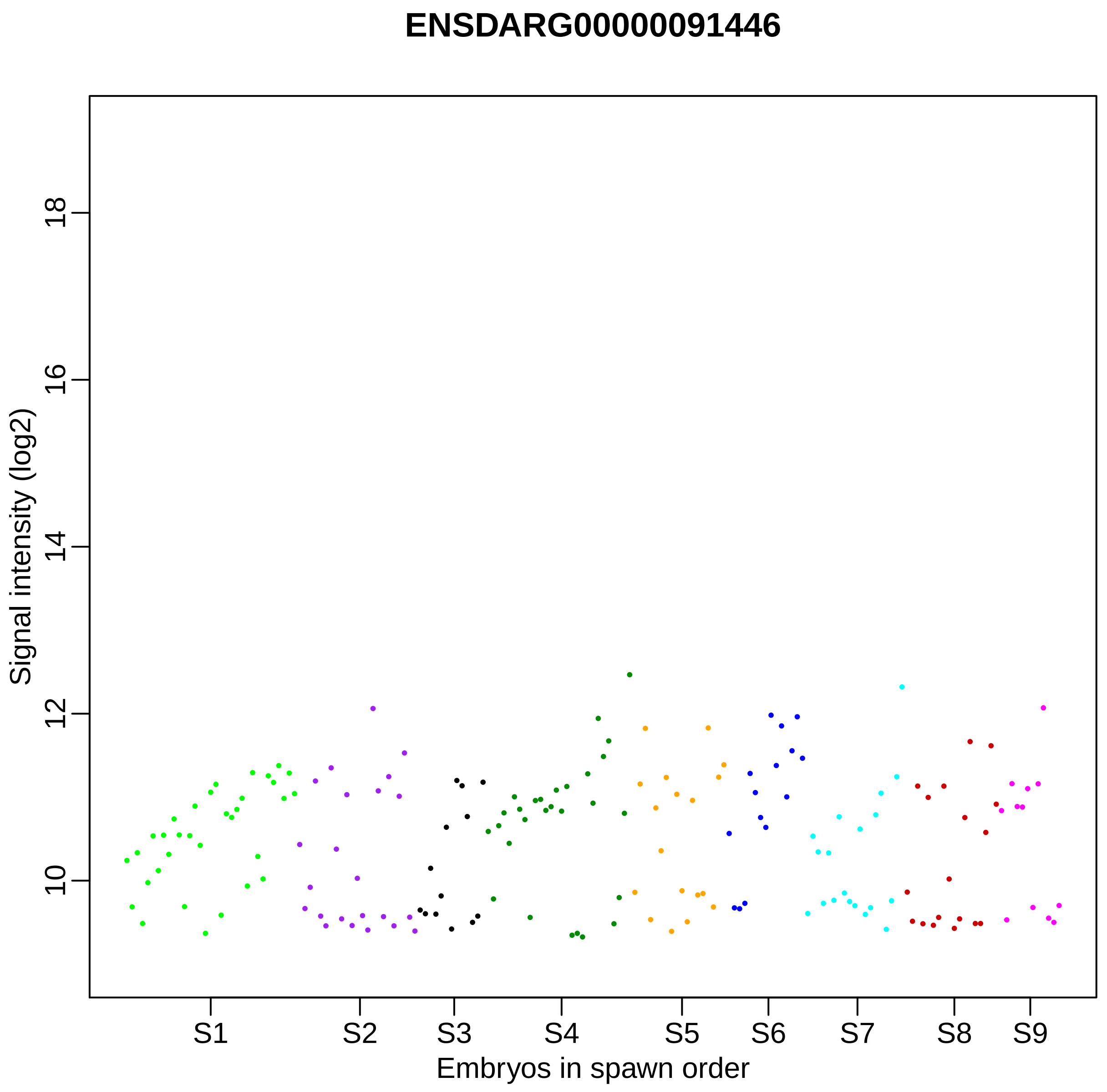
ENSDARG00000091446
Signal intensity (log2)
18
16
14
12
10
S1
S2
S3
S4
S5
S6
S7
S8
S9
Embryos in spawn order

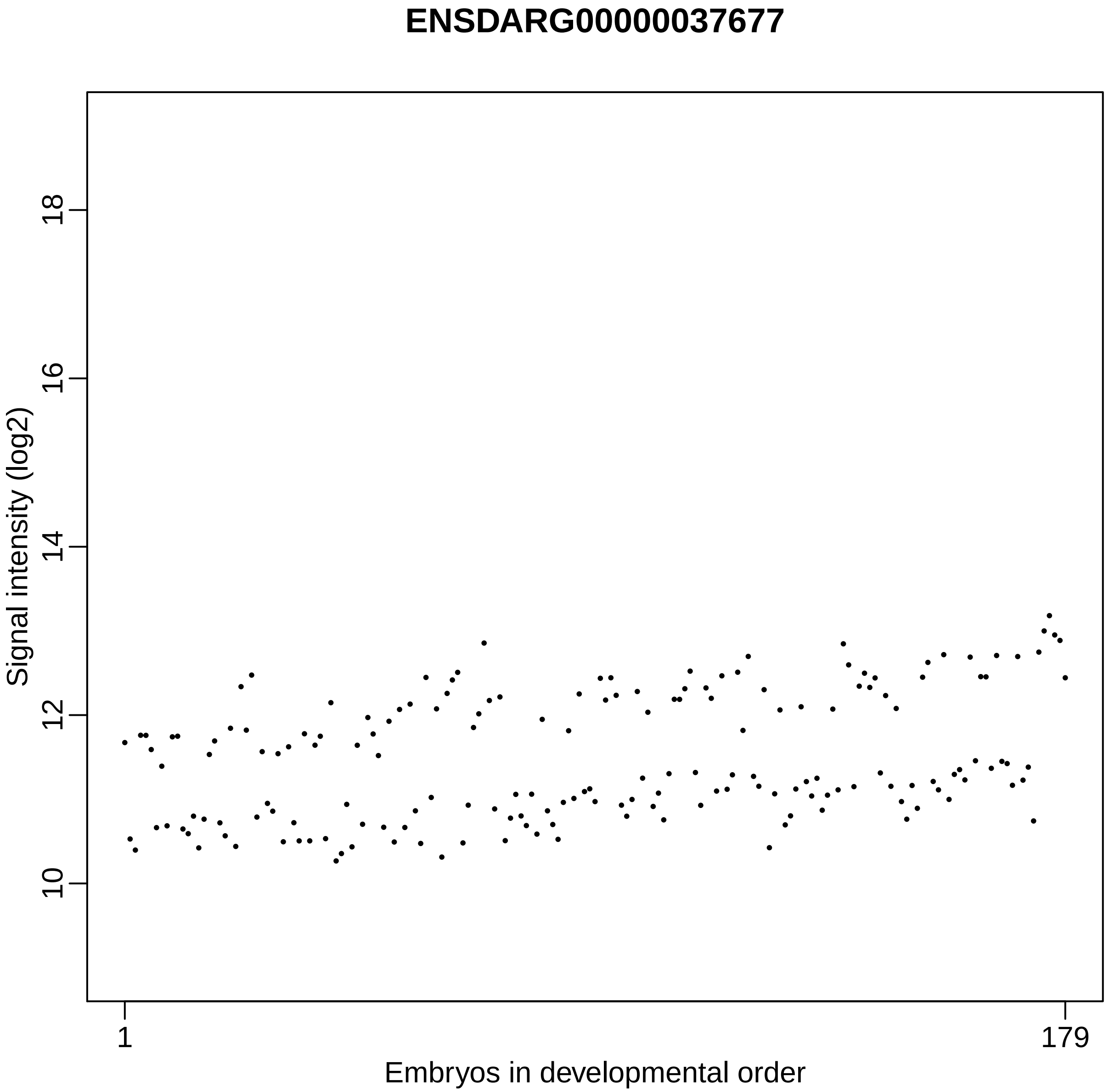
ENSDARG00000037677
Signal intensity (log2)
10
12
14
16
18
1
179
Embryos in developmental order

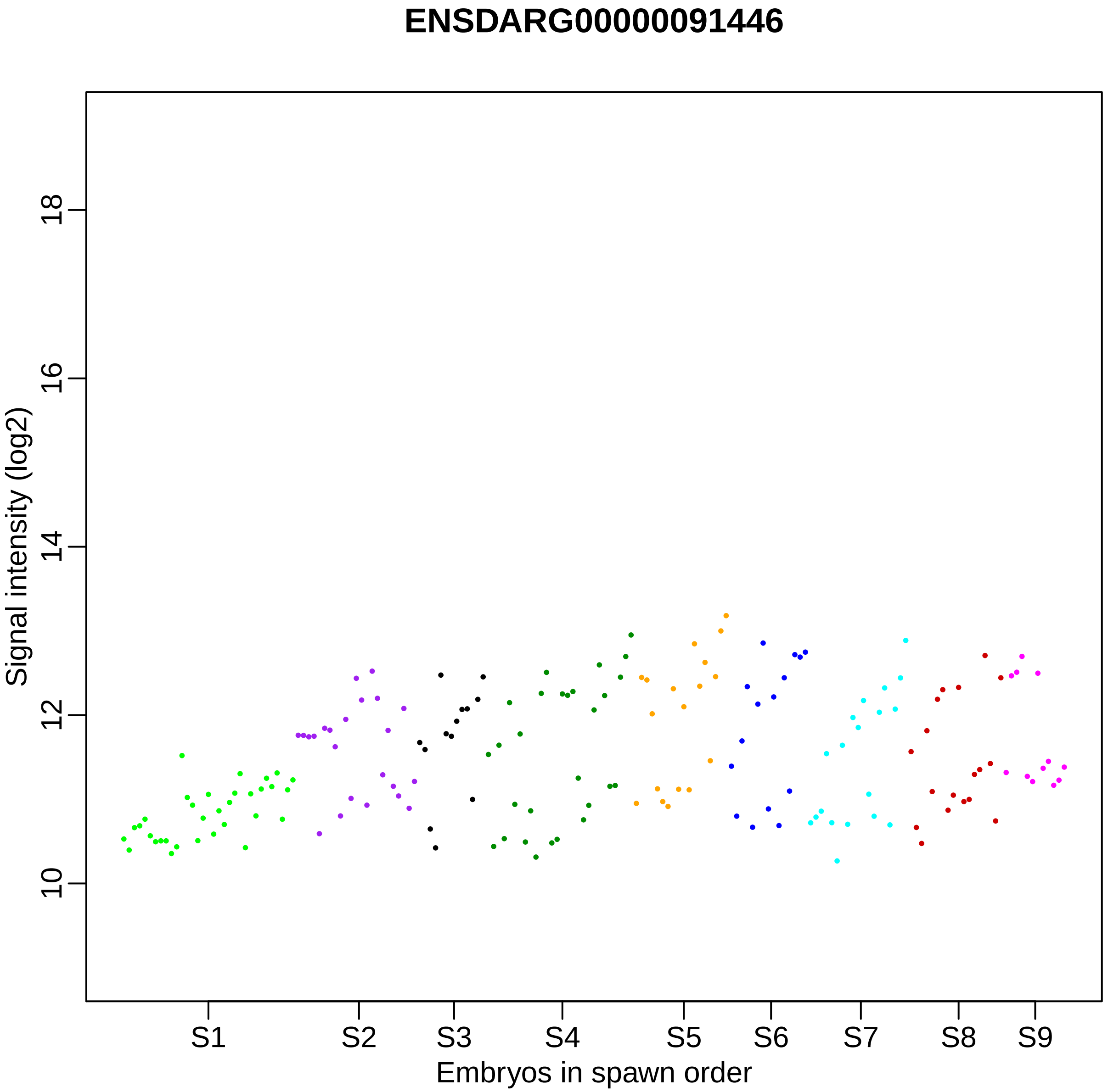
ENSDARG00000091446
Signal intensity (log2)
10
12
14
16
18
S1
S2
S3
S4
S5
S6
S7
S8
S9
Embryos in spawn order

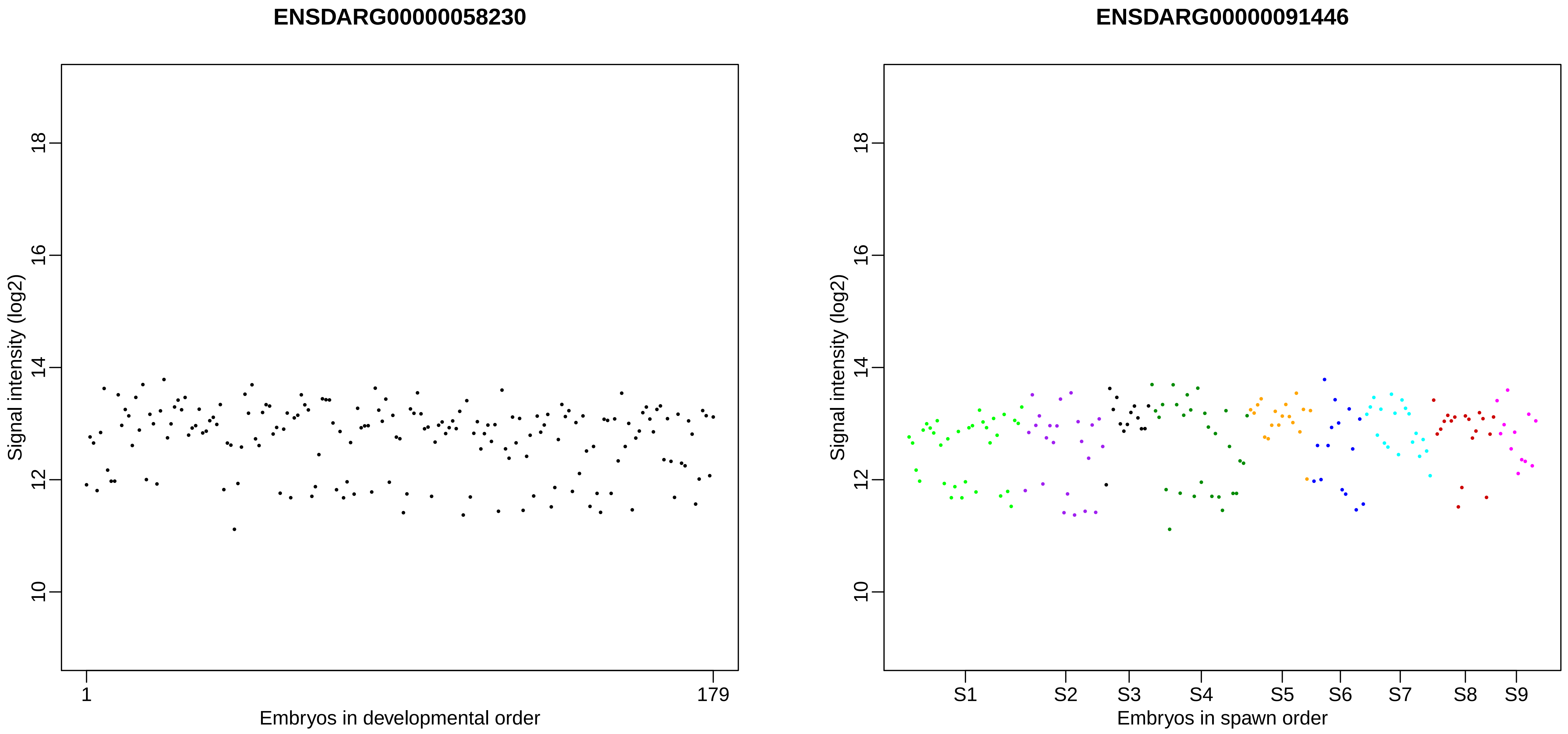
ENSDARG00000058230
Signal intensity (log2)
10
12
14
16
18
1
179
Embryos in developmental order
ENSDARG00000091446
Signal intensity (log2)
10
12
14
16
18
S1
S2
S3
S4
S5
S6
S7
S8
S9
Embryos in spawn order

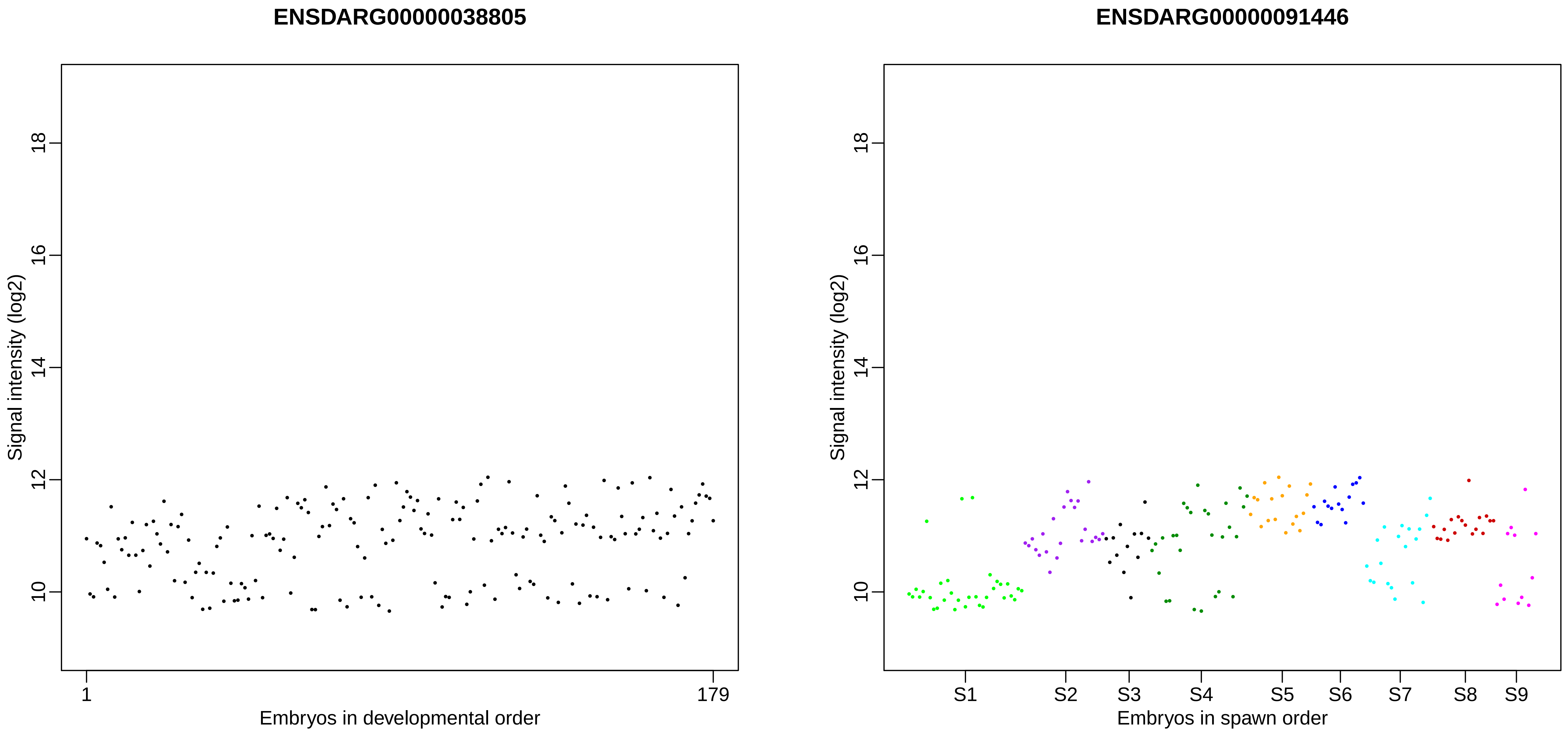
ENSDARG00000038805
Signal intensity (log2)
10
12
14
16
18
1
179
Embryos in developmental order
ENSDARG00000091446
Signal intensity (log2)
10
12
14
16
18
S1
S2
S3
S4
S5
S6
S7
S8
S9
Embryos in spawn order

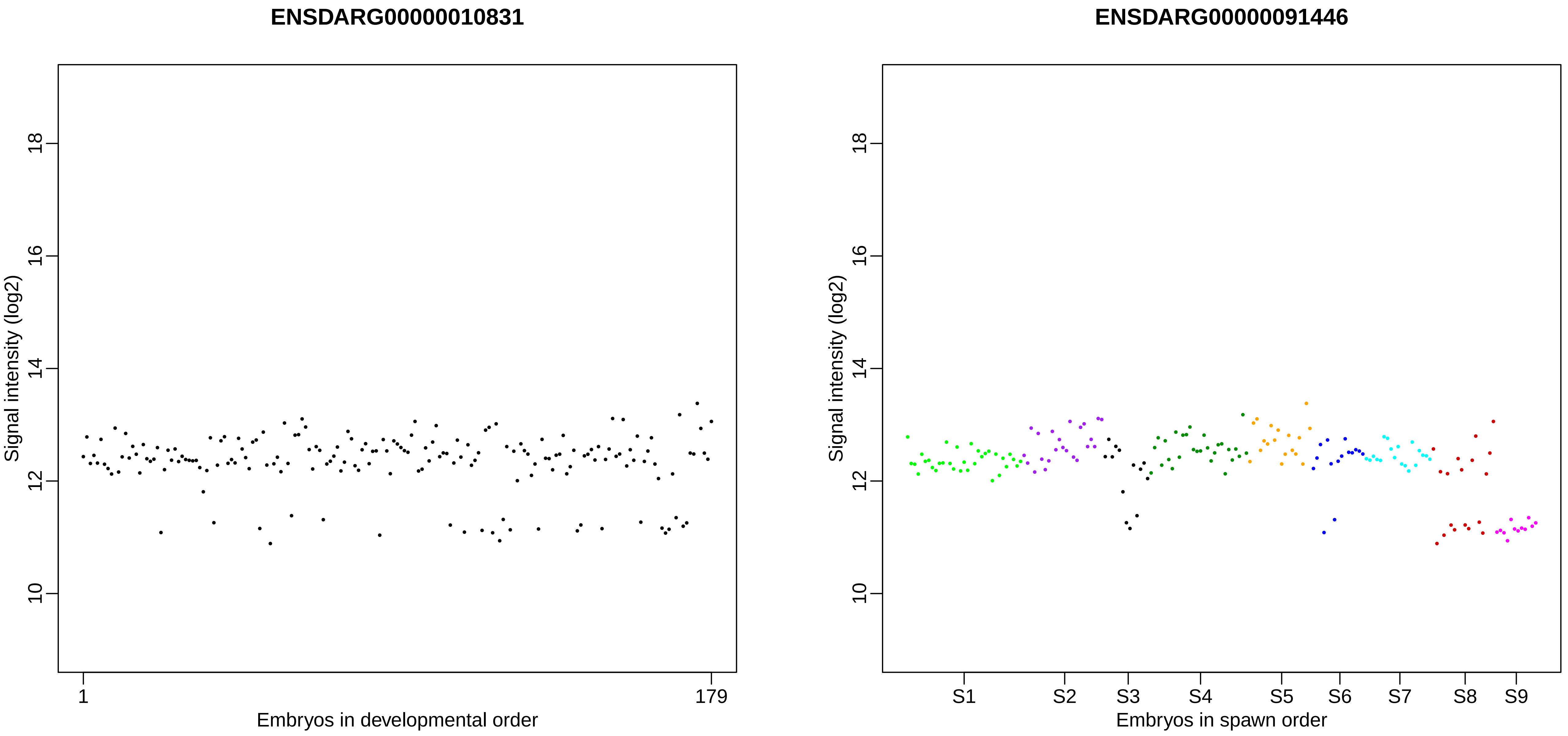

ENSDARG00000010831
Signal intensity (log2)
10
12
14
16
18
1
179
Embryos in developmental order
ENSDARG00000091446
Signal intensity (log2)
10
12
14
16
18
S1
S2
S3
S4
S5
S6
S7
S8
S9
Embryos in spawn order

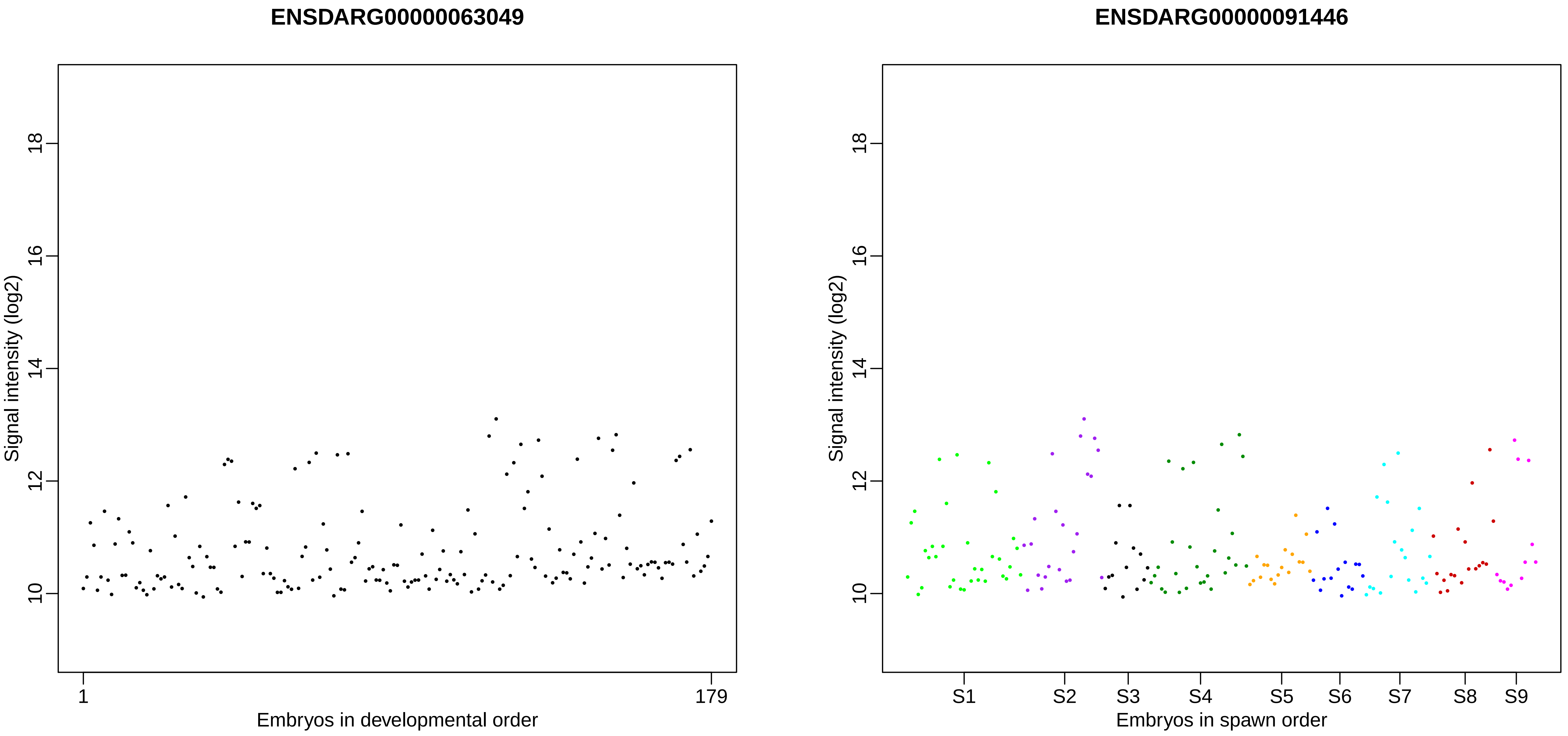

ENSDARG00000063049
Signal intensity (log2)
10
12
14
16
18
1
179
Embryos in developmental order
ENSDARG00000091446
Signal intensity (log2)
10
12
14
16
18
S1
S2
S3
S4
S5
S6
S7
S8
S9
Embryos in spawn order

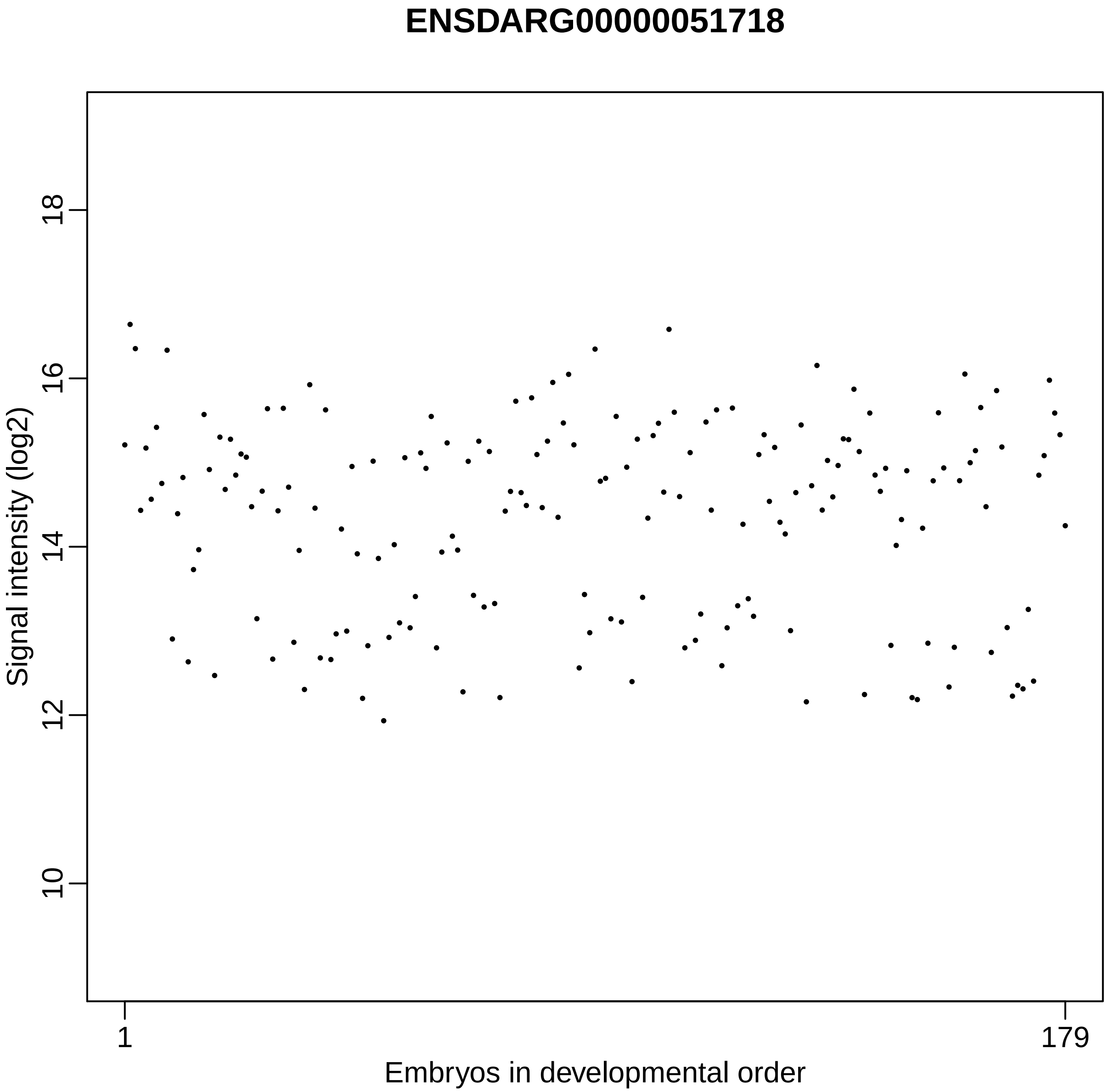
ENSDARG00000051718
Signal intensity (log2)
10
12
14
16
18
1
179
Embryos in developmental order

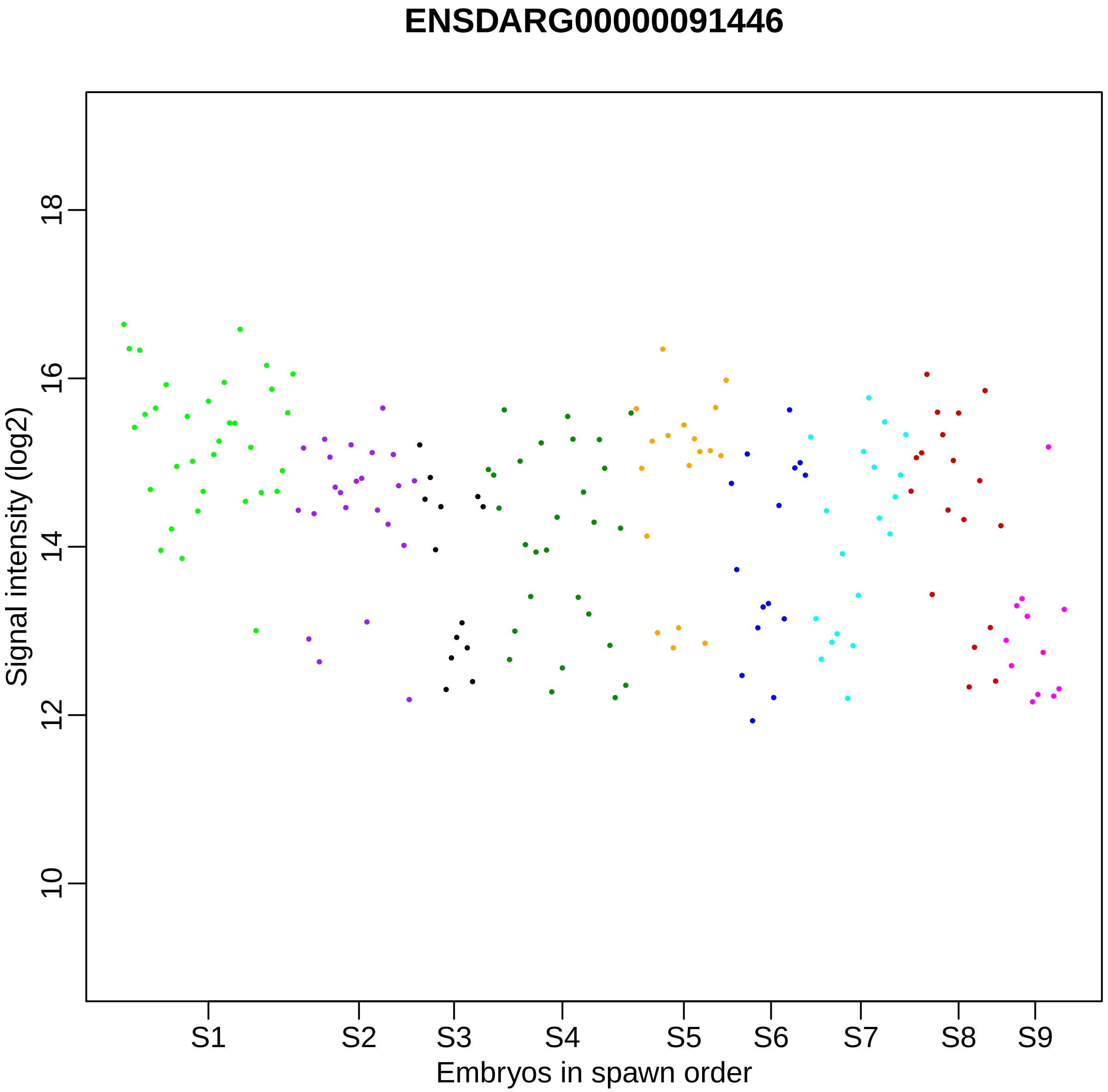
ENSDARG00000091446
Signal intensity (log2)
10
12
14
16
18
S1
S2
S3
S4
S5
S6
S7
S8
S9
Embryos in spawn order

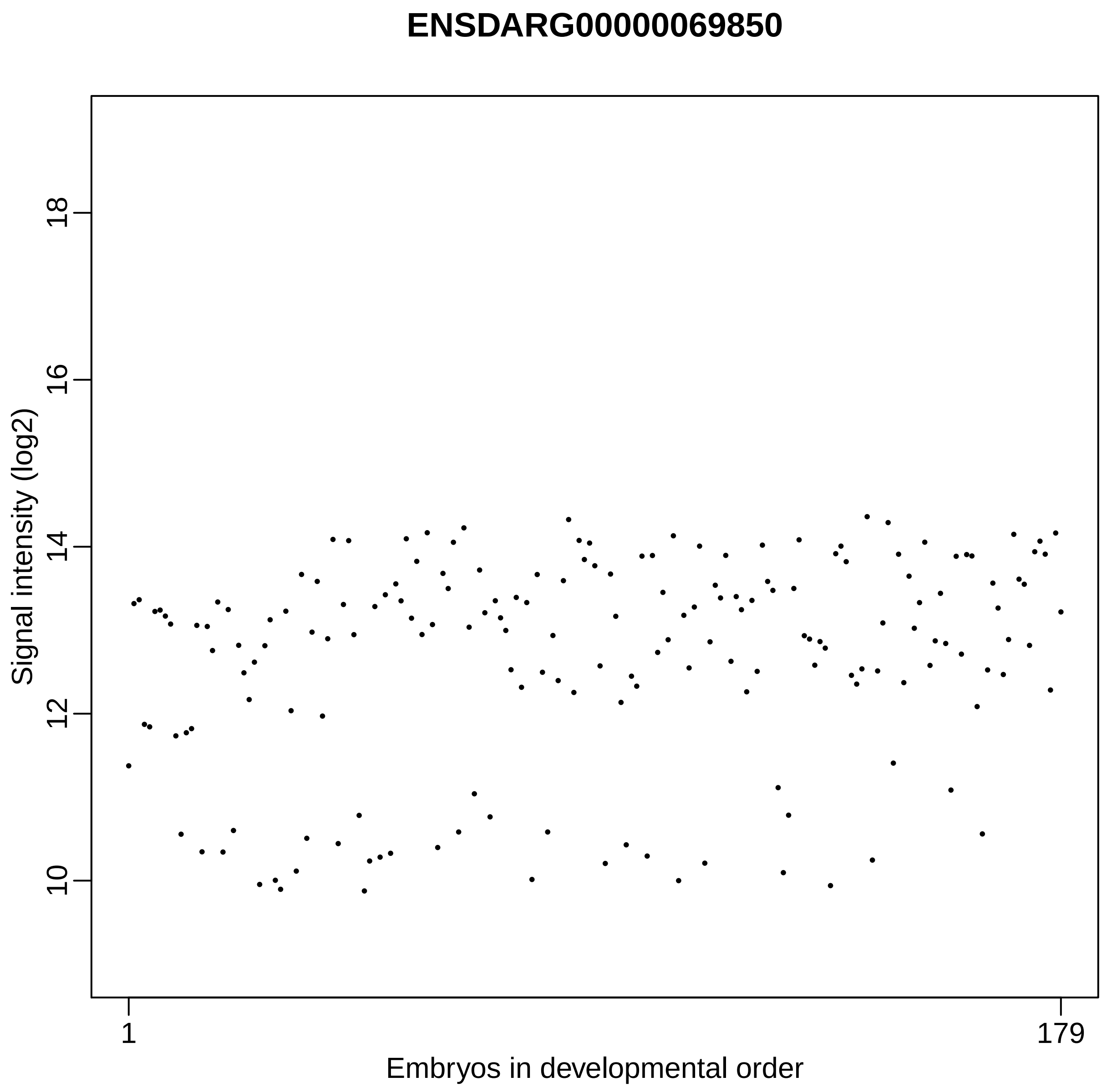

ENSDARG00000069850
Signal intensity (log2)
10
12
14
16
18
1
179
Embryos in developmental order

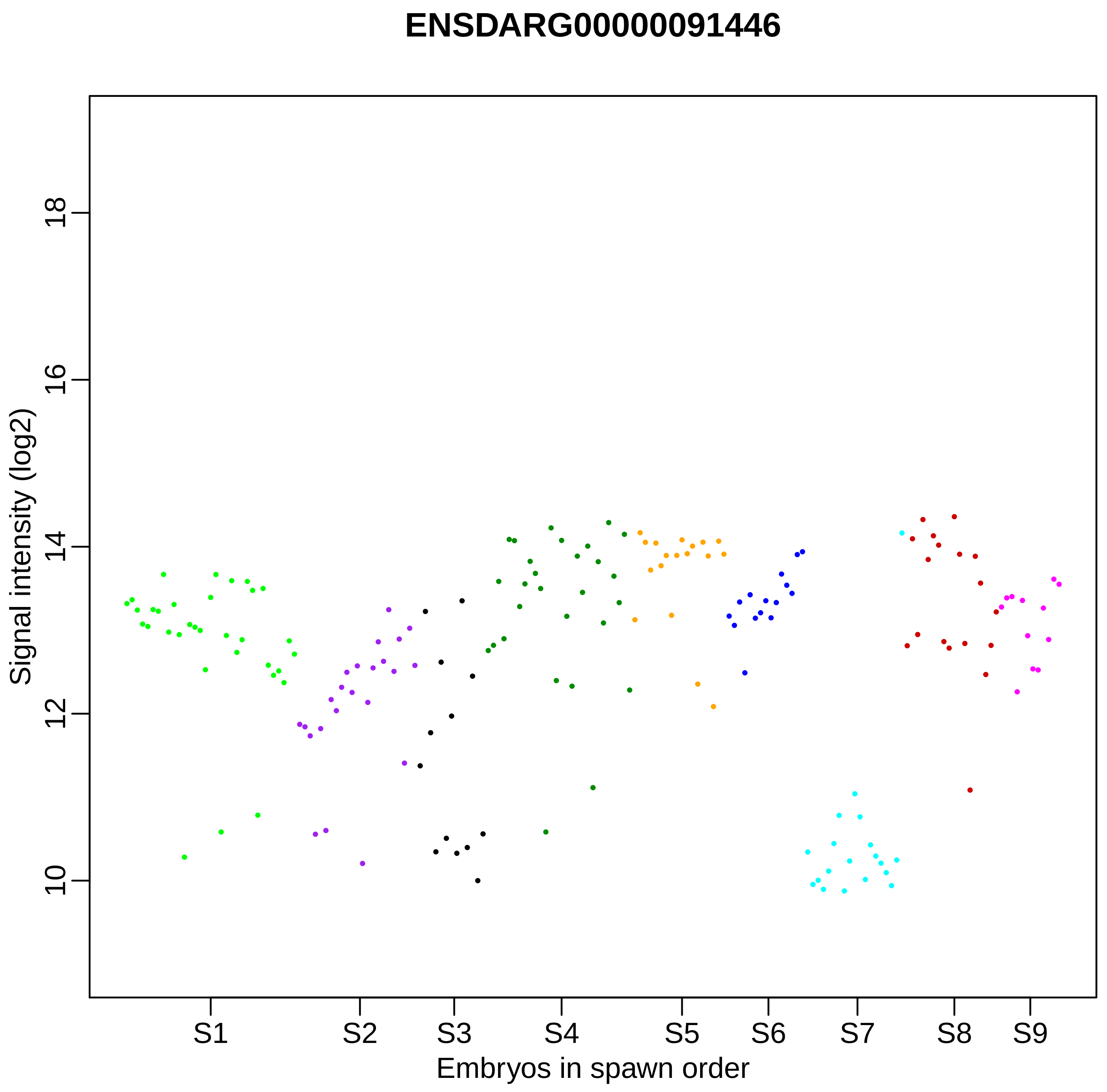

ENSDARG00000091446
Signal intensity (log2)
10
12
14
16
18
S1
S2
S3
S4
S5
S6
S7
S8
S9
Embryos in spawn order

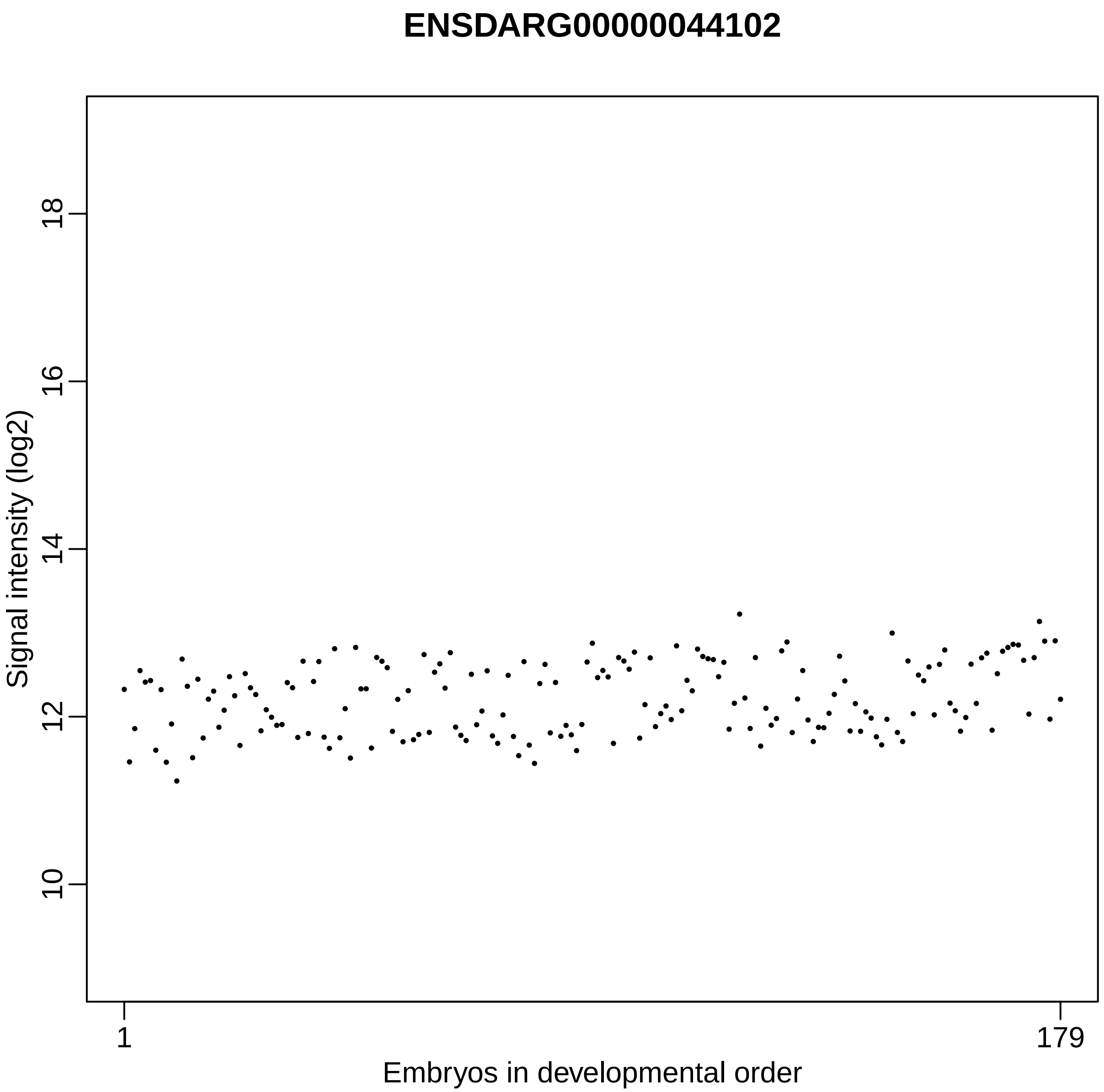
ENSDARG00000044102
Signal intensity (log2)
10
12
14
16
18
1
179
Embryos in developmental order

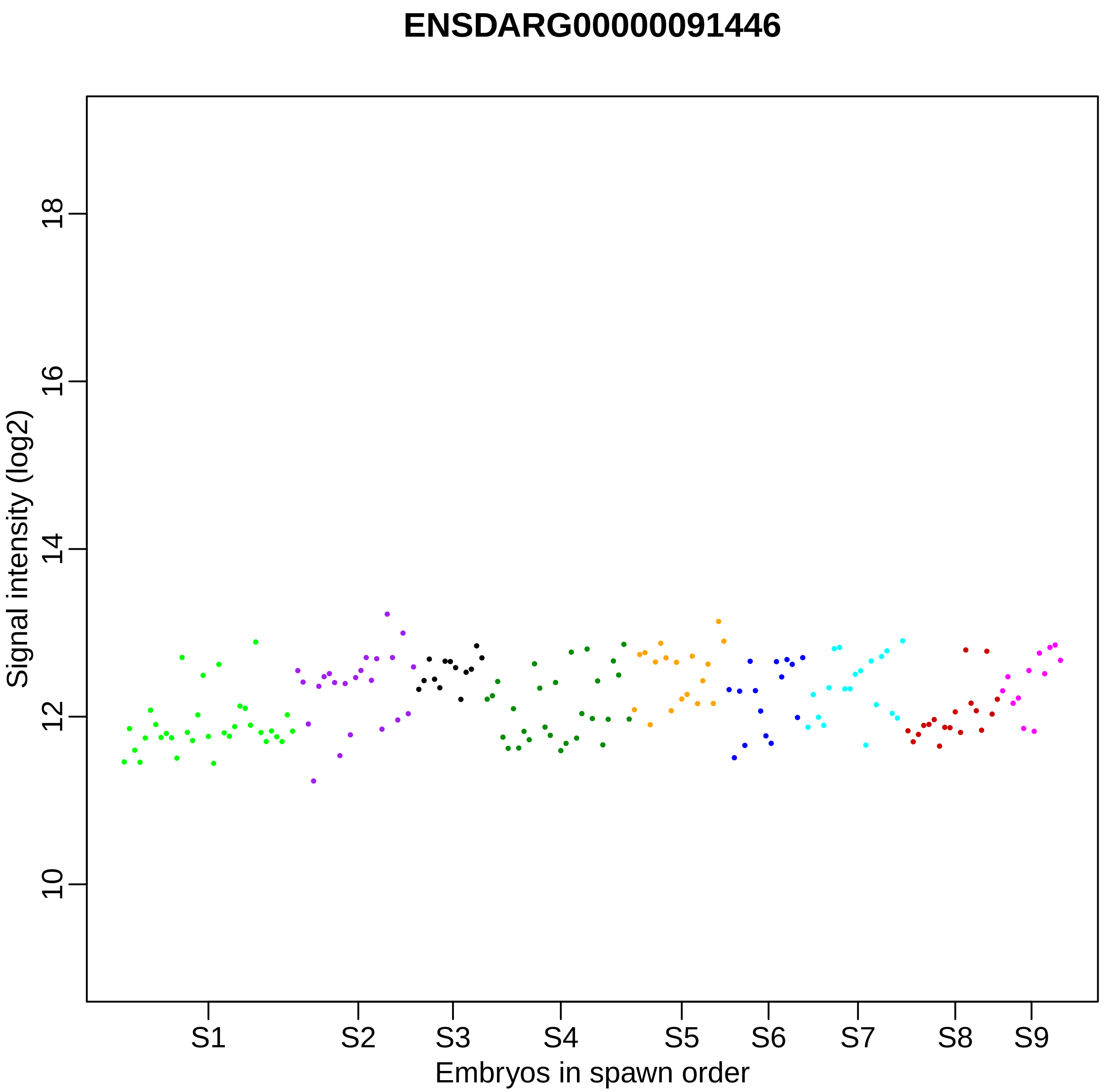
ENSDARG00000091446
Signal intensity (log2)
10
12
14
16
18
S1
S2
S3
S4
S5
S6
S7
S8
S9
Embryos in spawn order

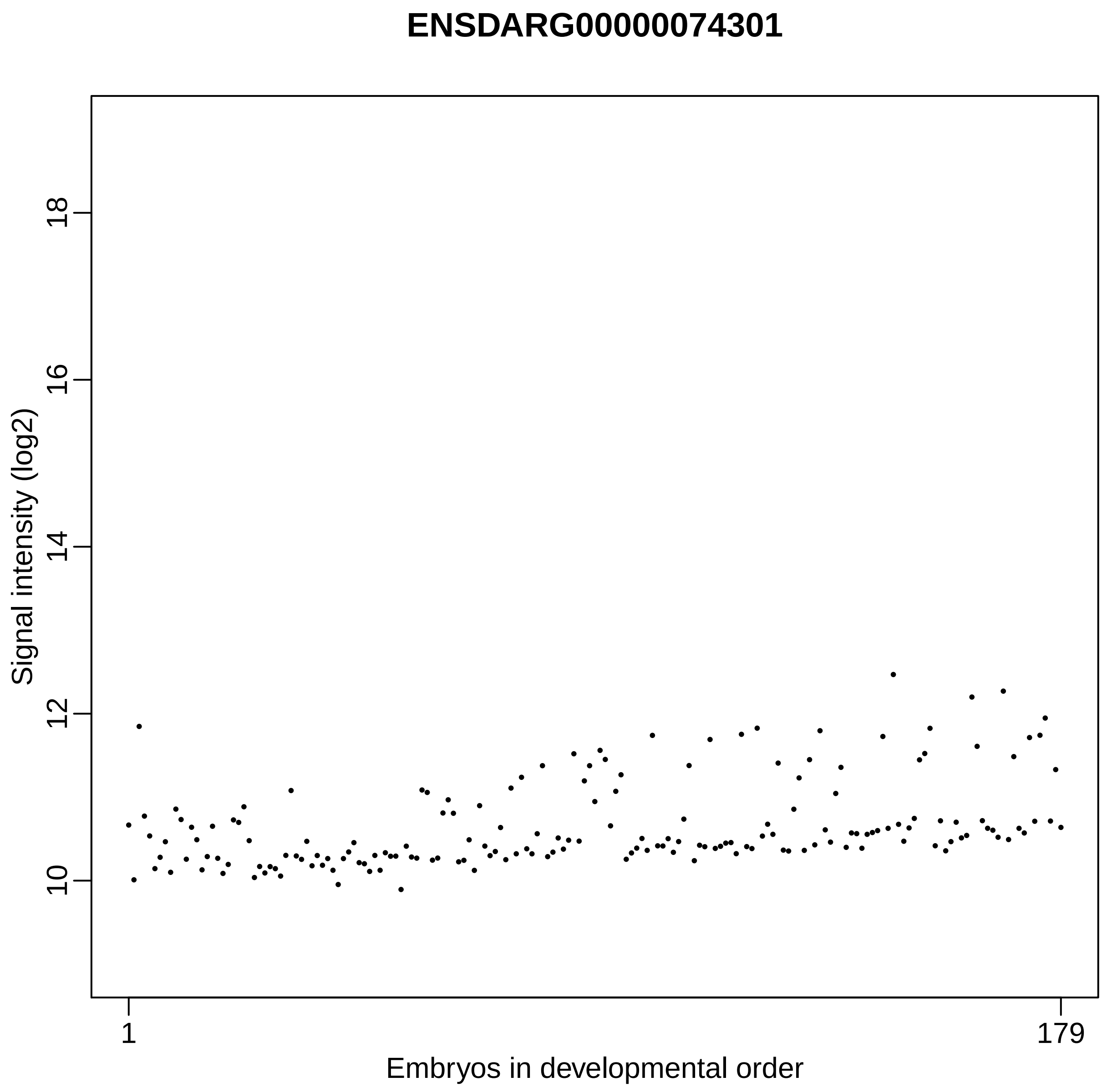

ENSDARG00000074301
Signal intensity (log2)
10
12
14
16
18
1
179
Embryos in developmental order

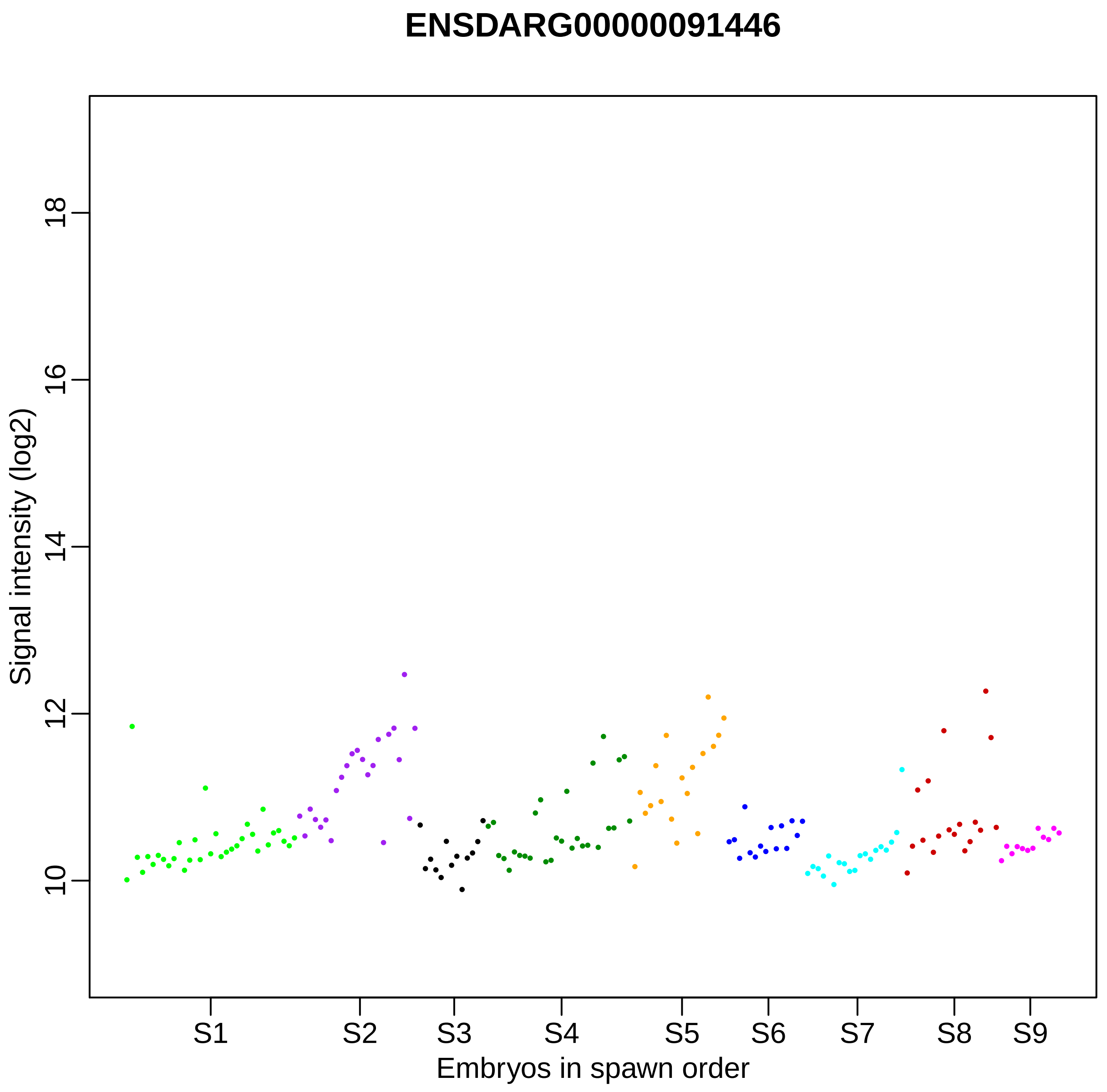

ENSDARG00000091446
Signal intensity (log2)
10
12
14
16
18
S1
S2
S3
S4
S5
S6
S7
S8
S9
Embryos in spawn order

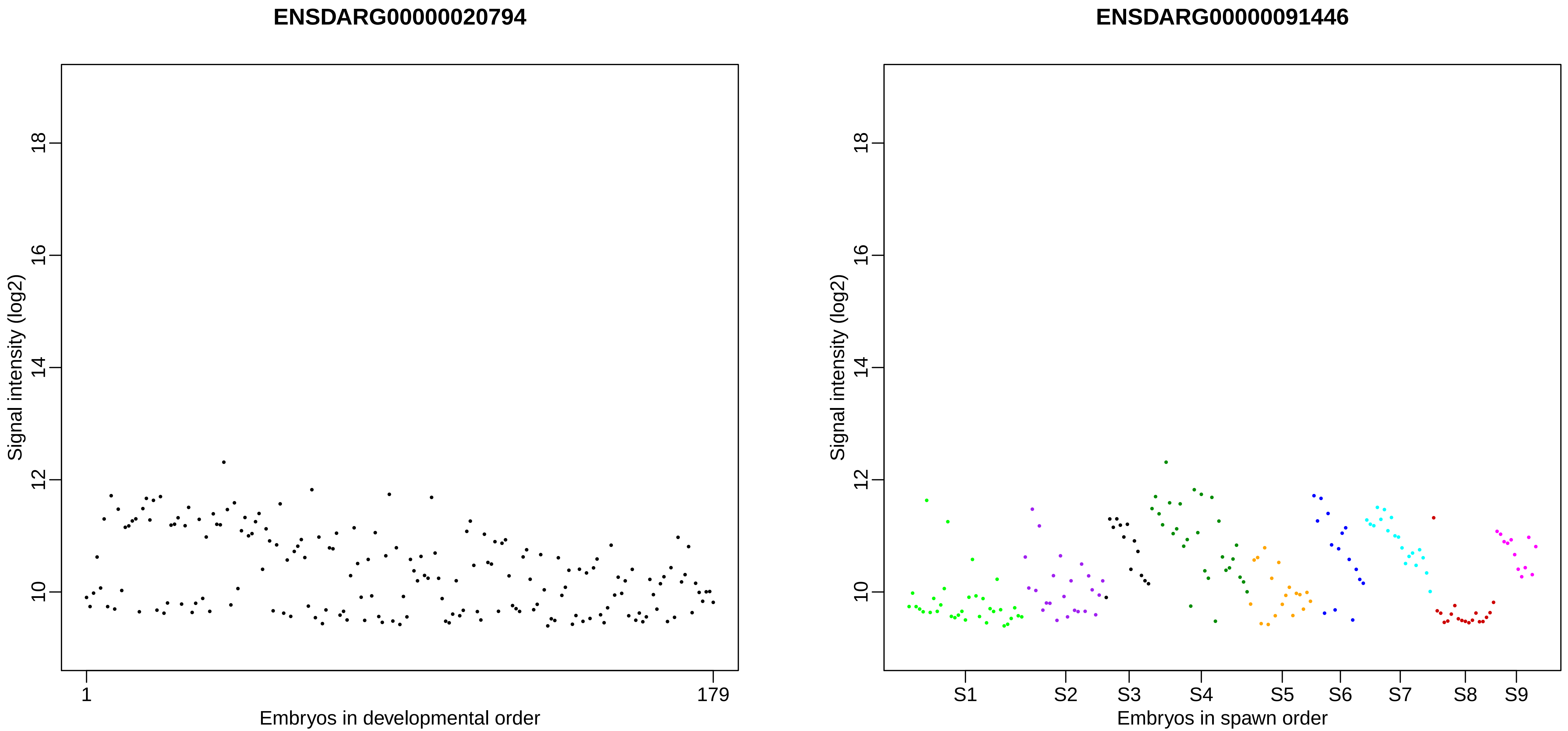

ENSDARG00000020794
Signal intensity (log2)
10
12
14
16
18
1
179
Embryos in developmental order
ENSDARG00000091446
Signal intensity (log2)
10
12
14
16
18
S1
S2
S3
S4
S5
S6
S7
S8
S9
Embryos in spawn order

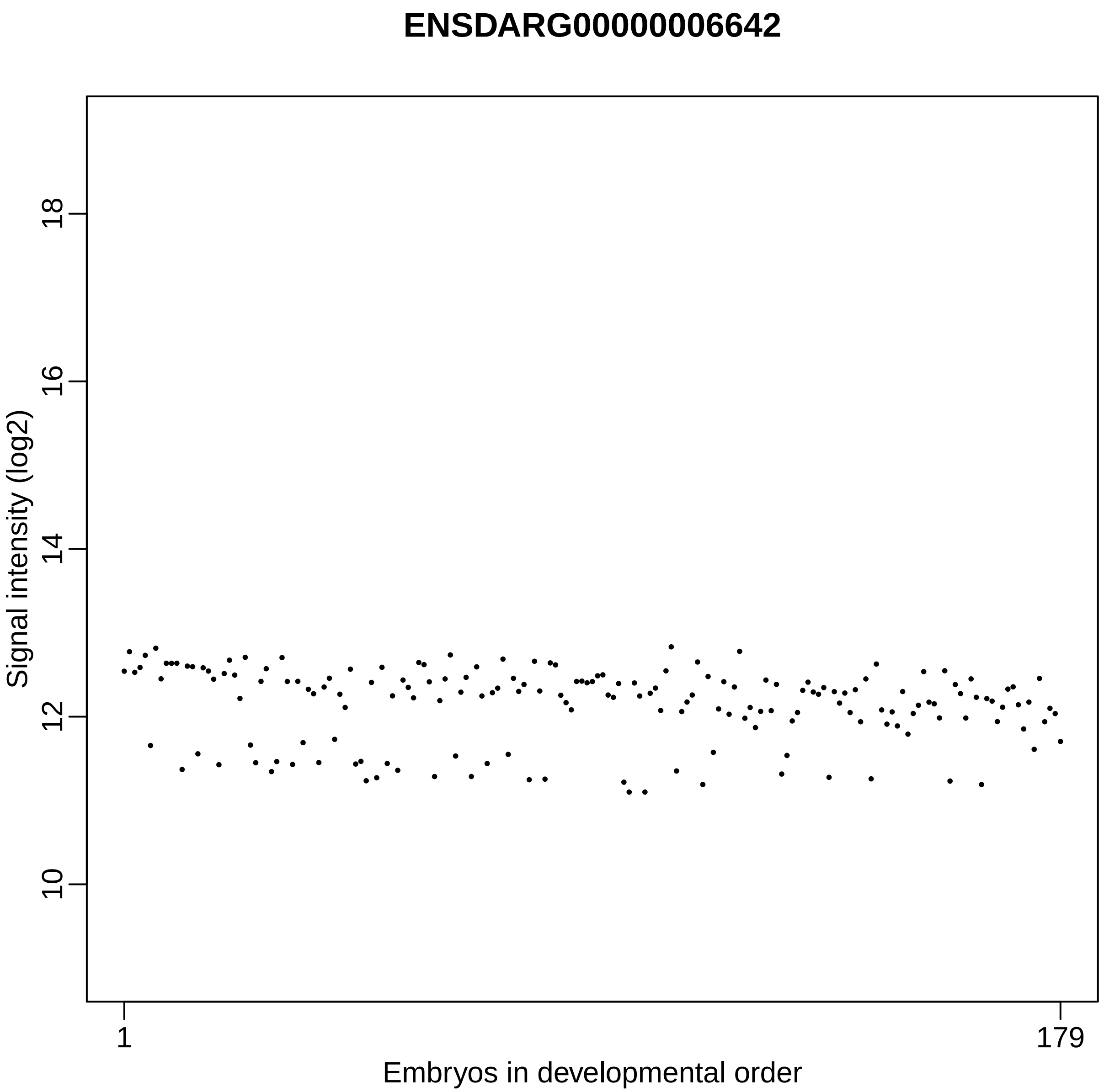
ENSDARG00000006642
Signal intensity (log2)
10
12
14
16
18
1
179
Embryos in developmental order

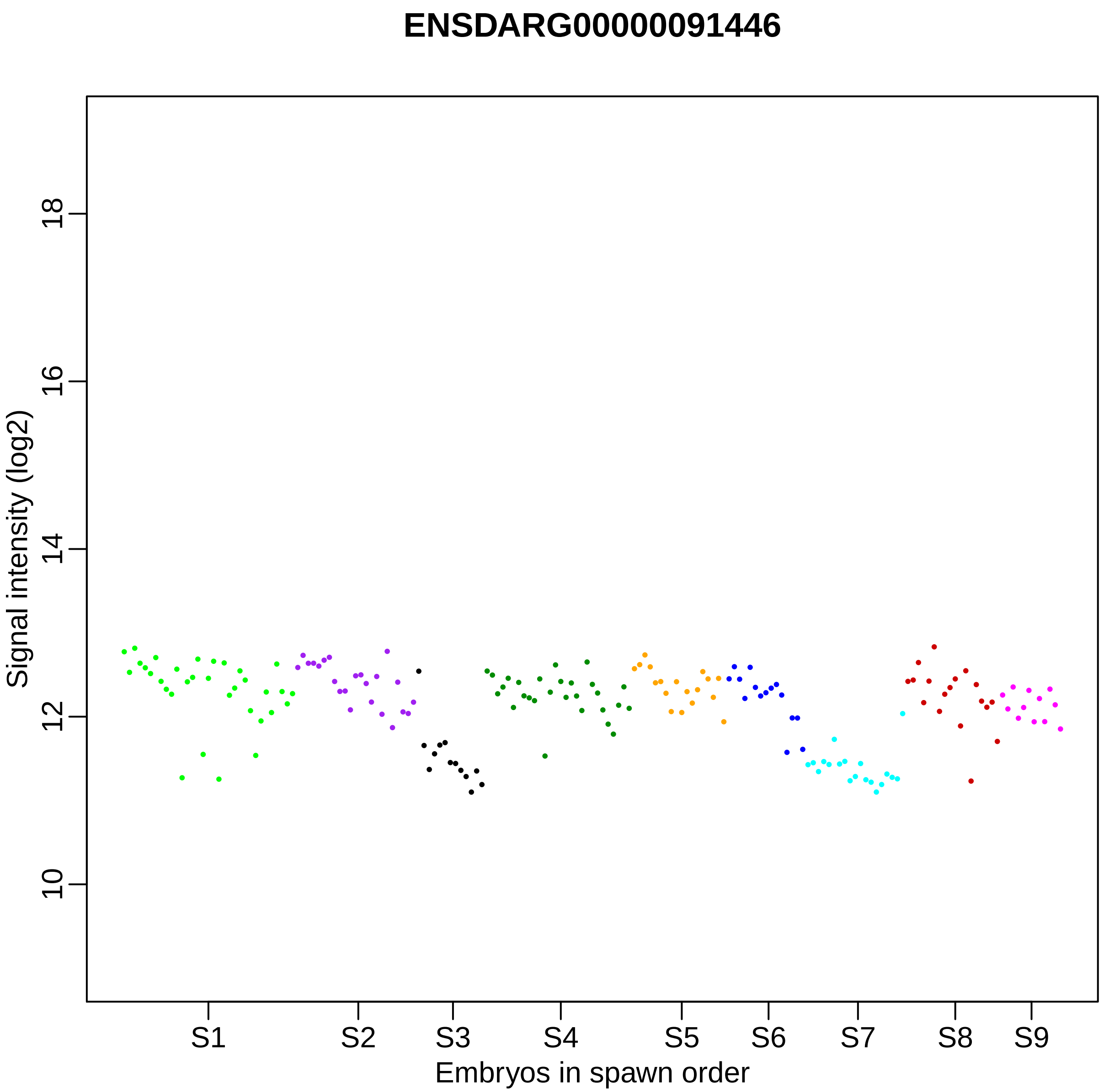
ENSDARG00000091446
Signal intensity (log2)
10
12
14
16
18
S1
S2
S3
S4
S5
S6
S7
S8
S9
Embryos in spawn order

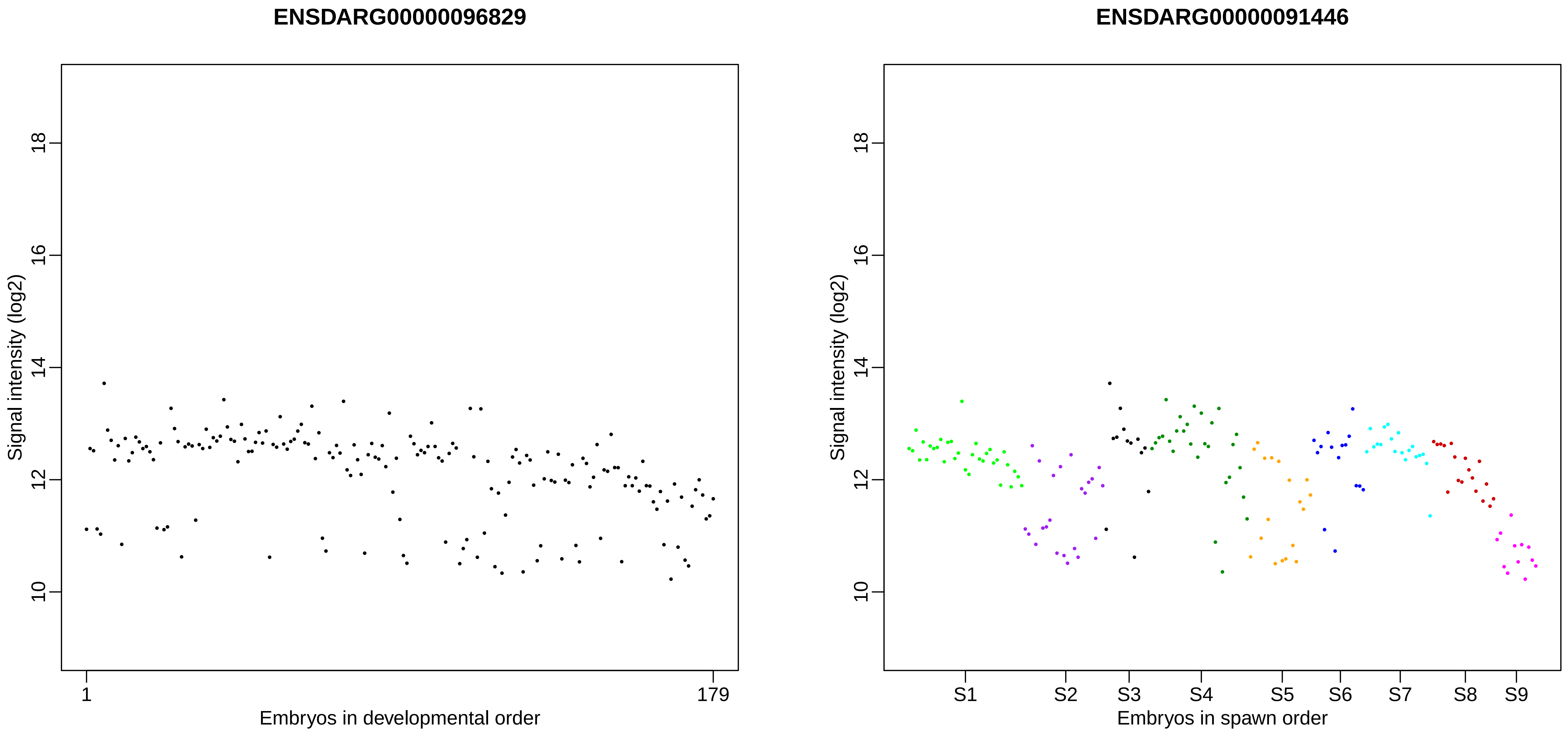
ENSDARG00000096829
Signal intensity (log2)
10
12
14
16
18
1
179
Embryos in developmental order
ENSDARG00000091446
Signal intensity (log2)
10
12
14
16
18
S1
S2
S3
S4
S5
S6
S7
S8
S9
Embryos in spawn order

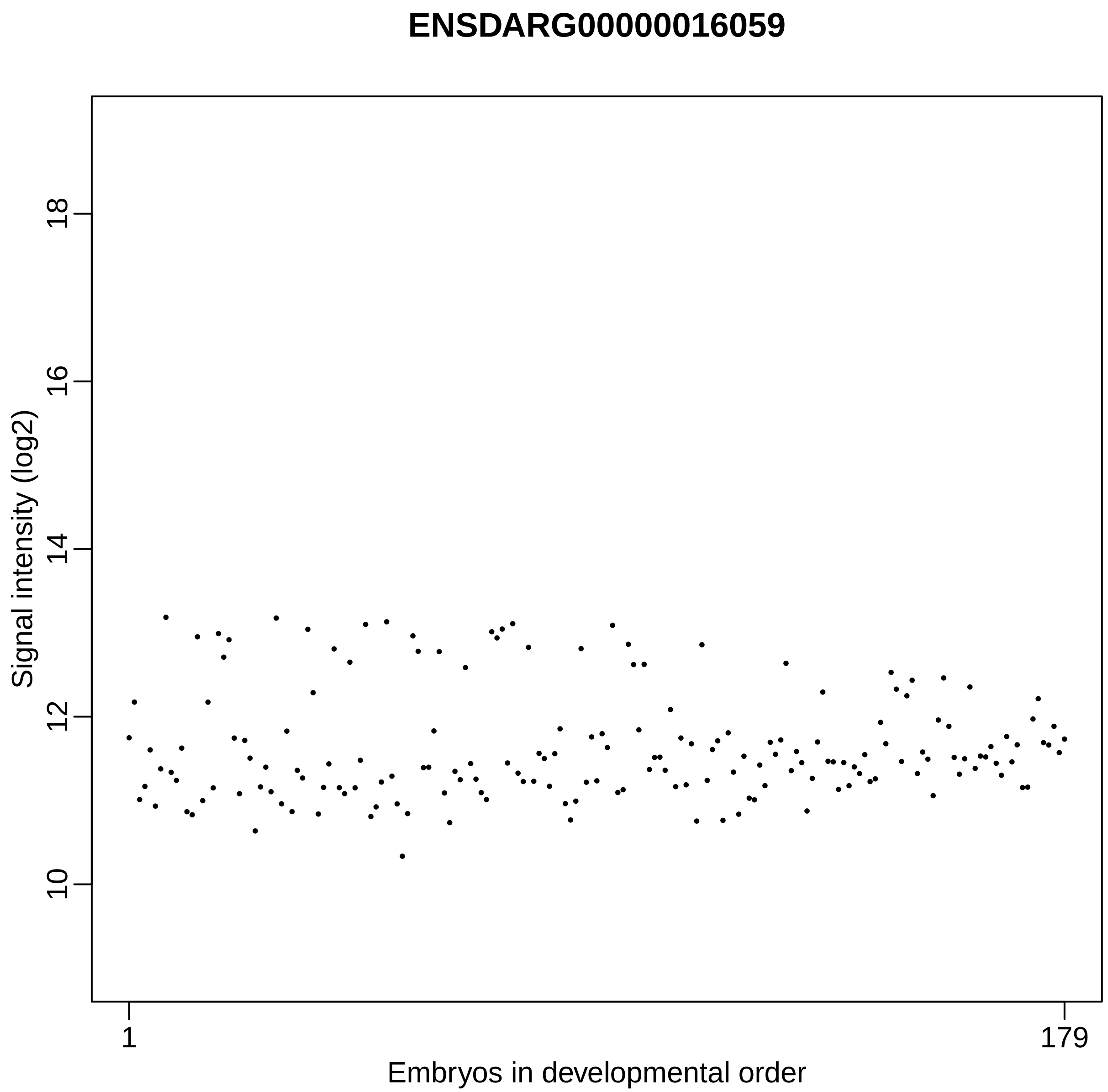
ENSDARG00000016059
Signal intensity (log2)
10
12
14
16
18
1
179
Embryos in developmental order

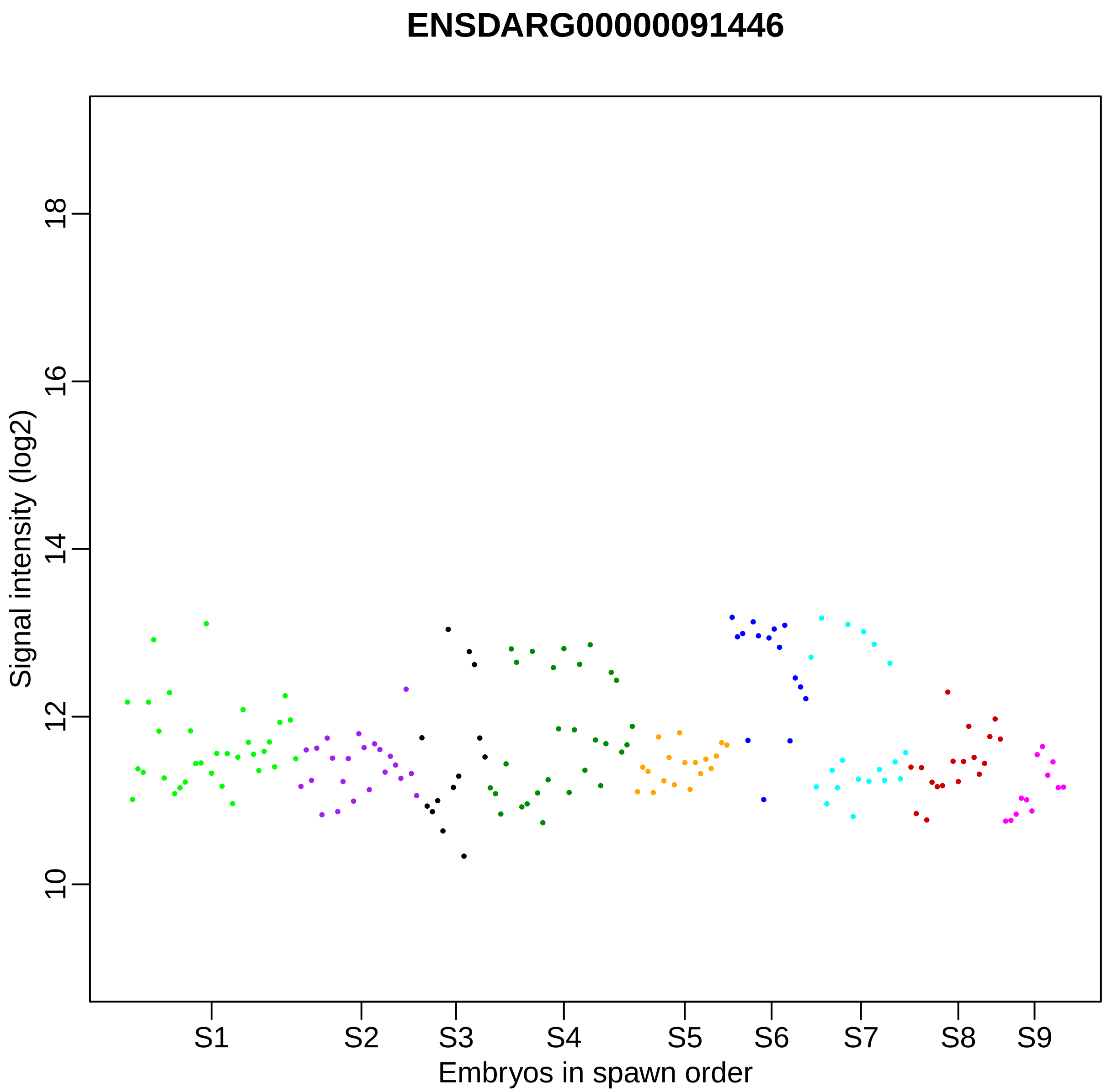
ENSDARG00000091446
Signal intensity (log2)
10
12
14
16
18
S1
S2
S3
S4
S5
S6
S7
S8
S9
Embryos in spawn order

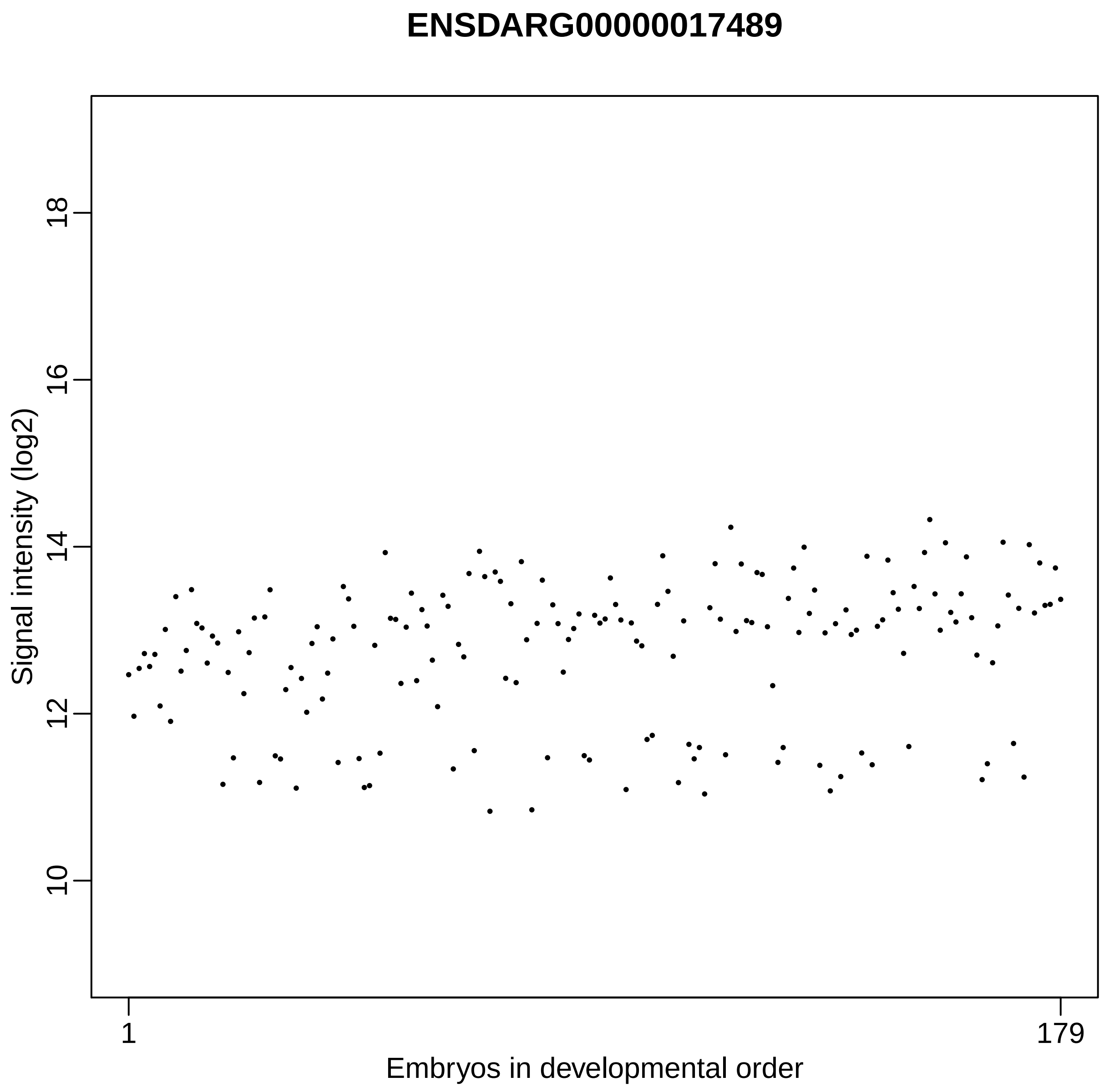
ENSDARG00000017489
Signal intensity (log2)
10
12
14
16
18
1
179
Embryos in developmental order

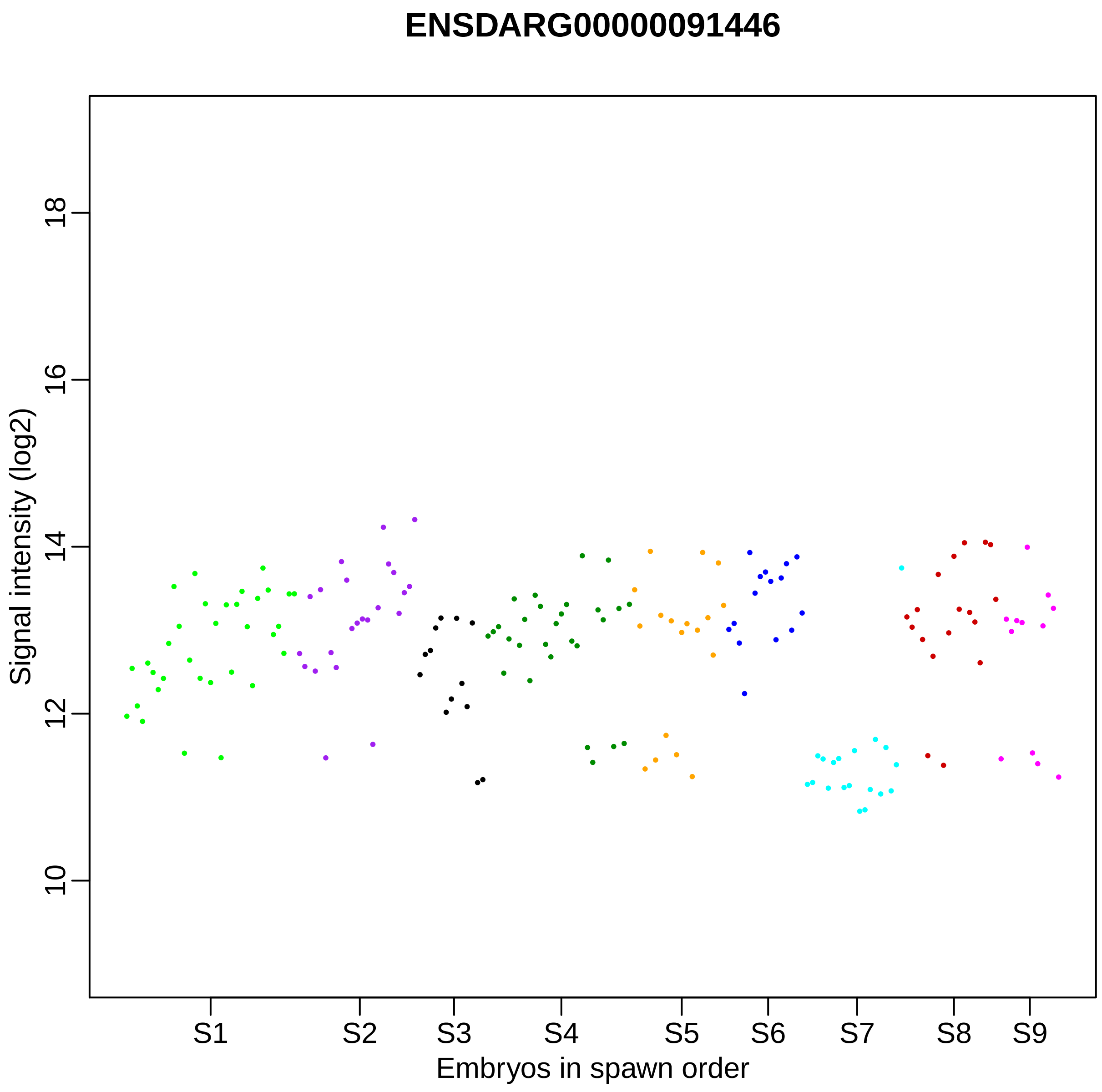
ENSDARG00000091446
Signal intensity (log2)
10
12
14
16
18
S1
S2
S3
S4
S5
S6
S7
S8
S9
Embryos in spawn order

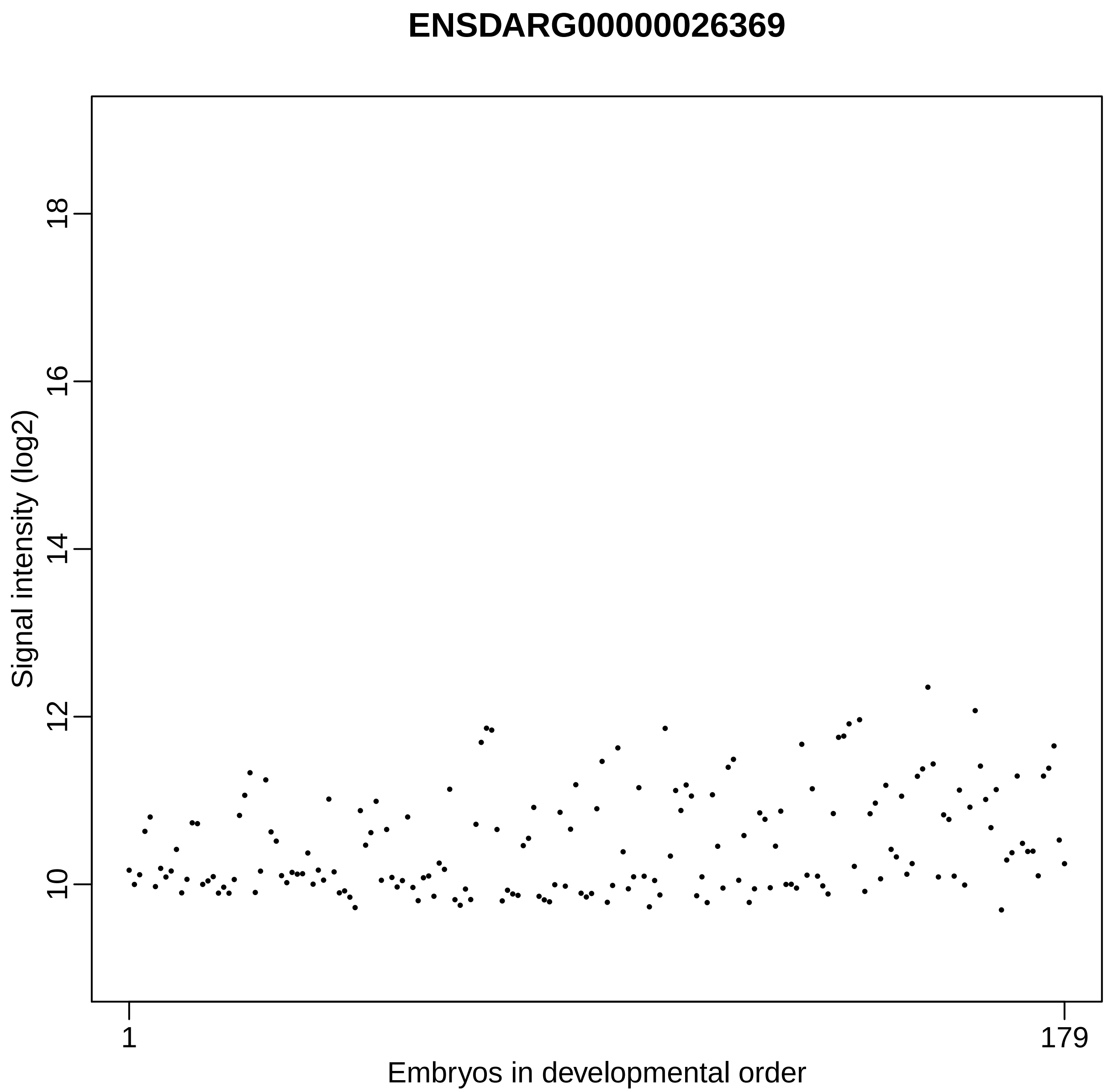
ENSDARG00000026369
Signal intensity (log2)
10
12
14
16
18
1
179
Embryos in developmental order

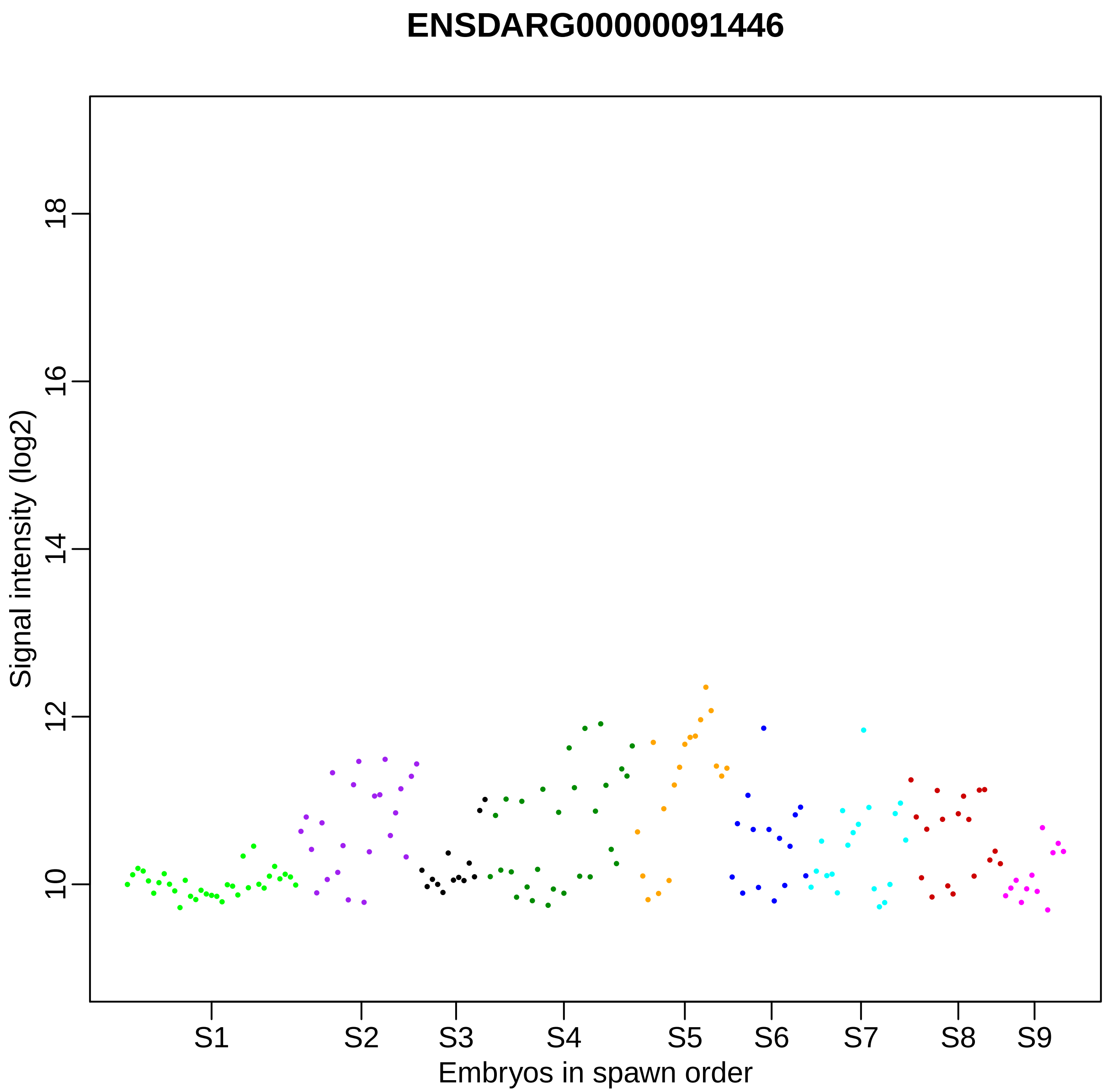
ENSDARG00000091446
Signal intensity (log2)
10
12
14
16
18
S1
S2
S3
S4
S5
S6
S7
S8
S9
Embryos in spawn order

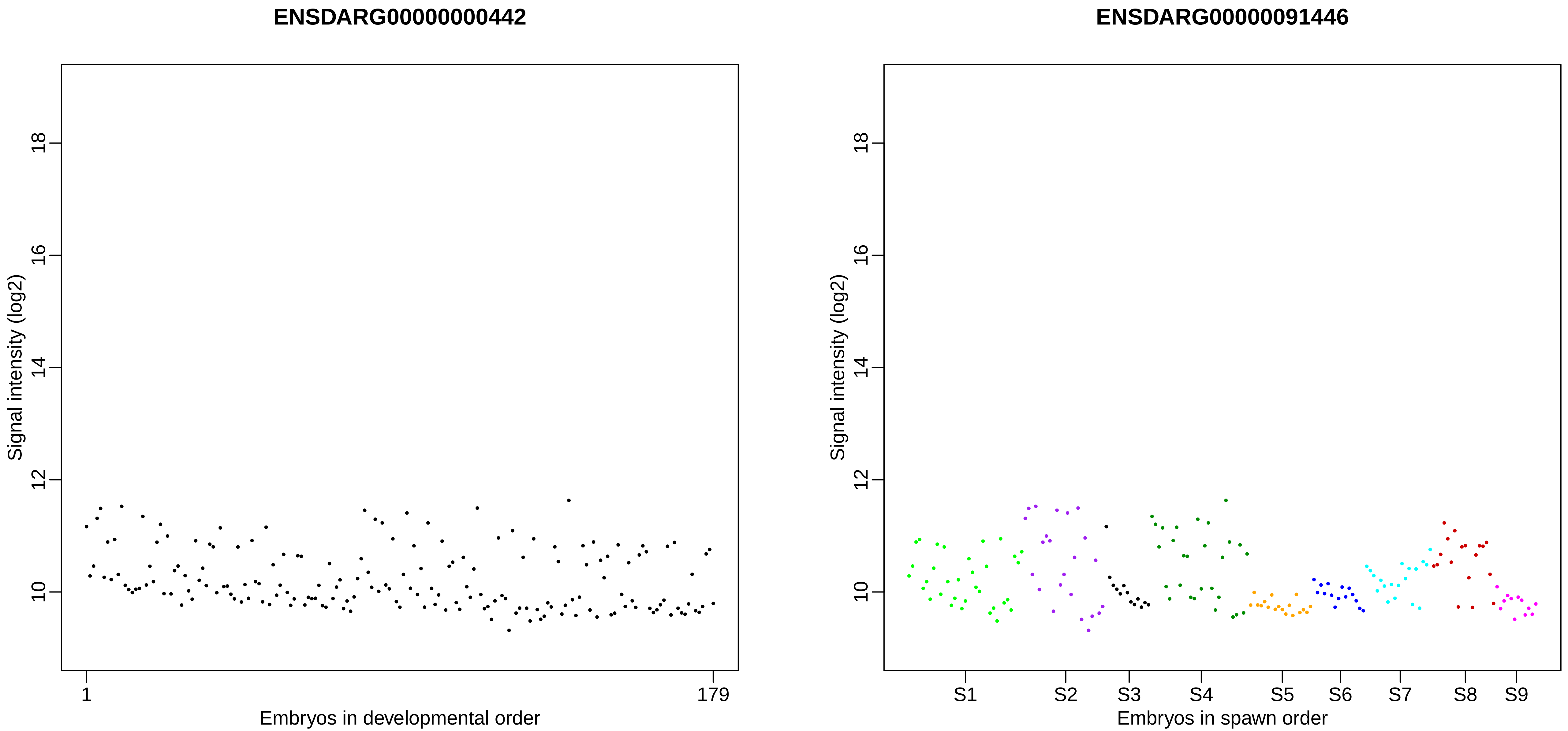

ENSDARG00000000442
Signal intensity (log2)
10
12
14
16
18
1
179
Embryos in developmental order
ENSDARG00000091446
Signal intensity (log2)
10
12
14
16
18
S1
S2
S3
S4
S5
S6
S7
S8
S9
Embryos in spawn order

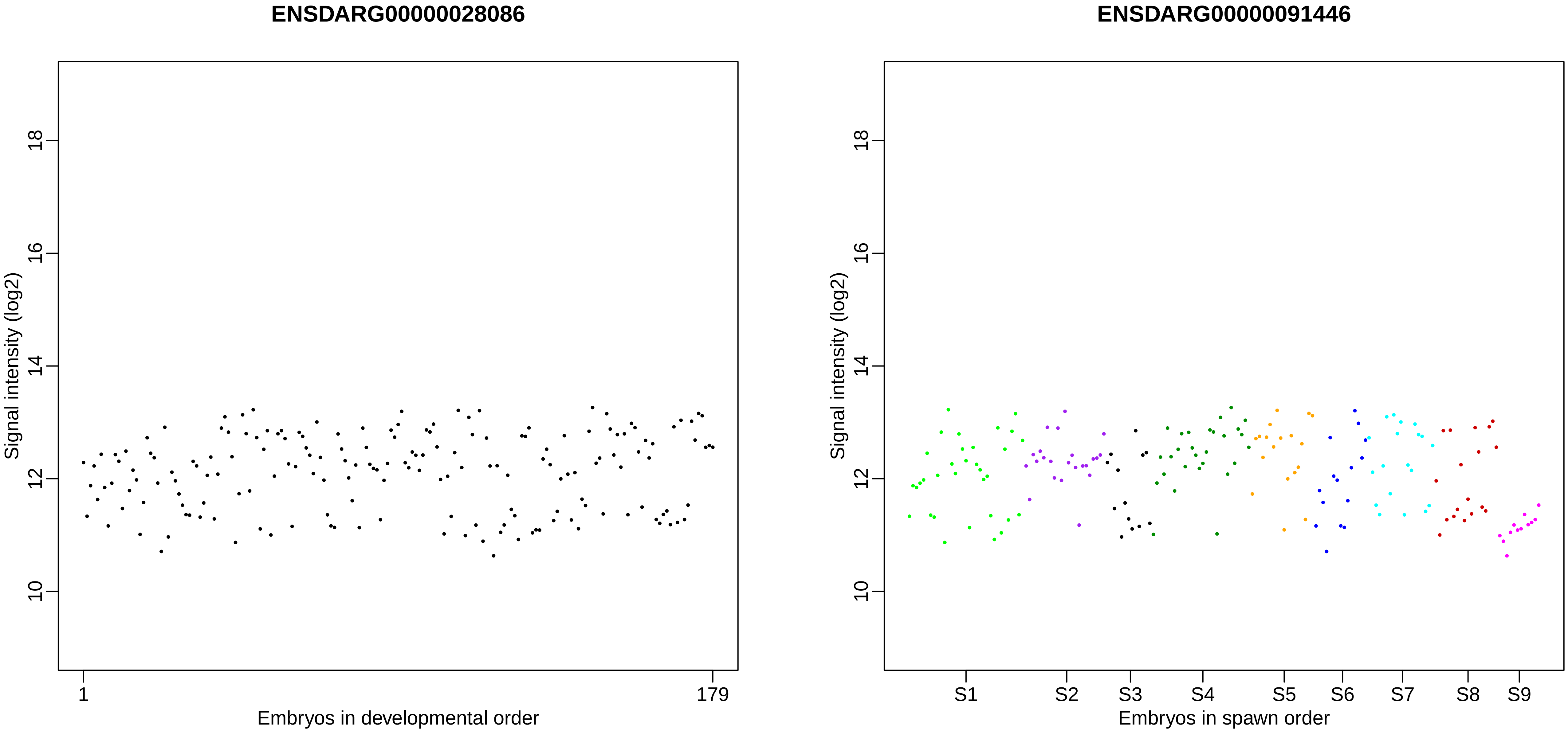
ENSDARG00000028086
ENSDARG00000091446
Signal intensity (log2)
10
12
14
16
18
1
179
Embryos in developmental order
S1
S2
S3
S4
S5
S6
S7
S8
S9
Embryos in spawn order

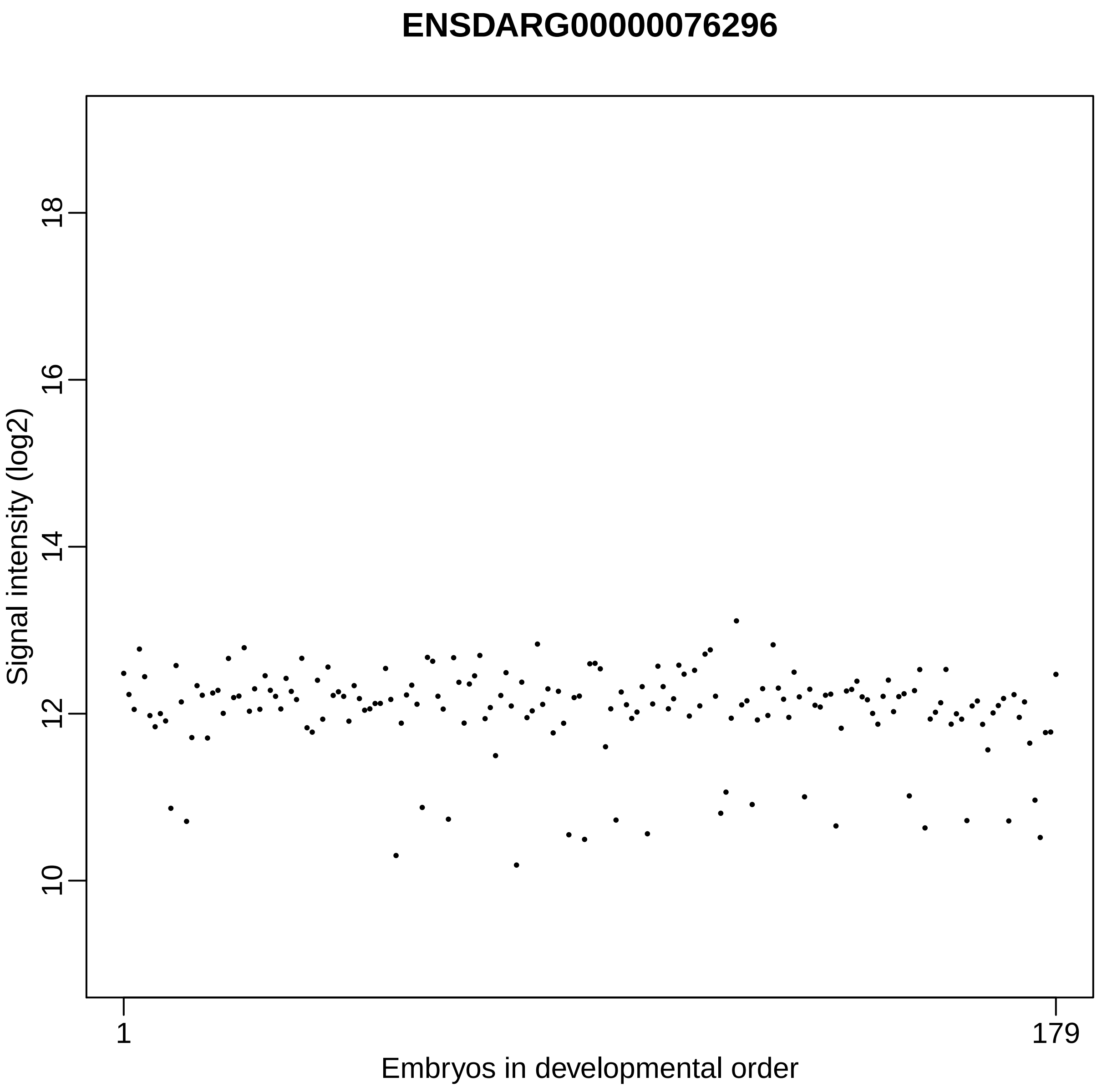
ENSDARG00000076296
Signal intensity (log2)
10
12
14
16
18
1
179
Embryos in developmental order

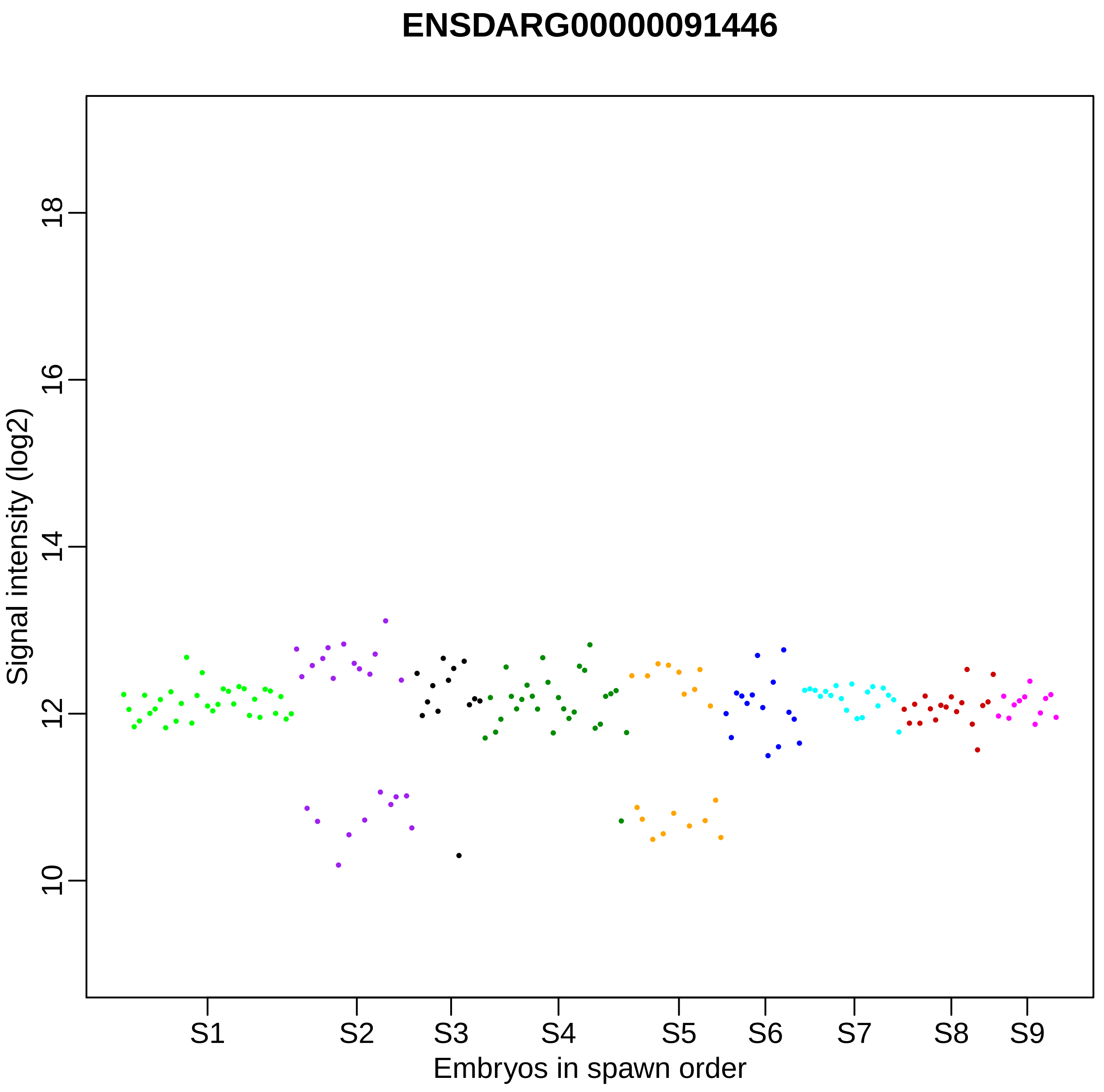
ENSDARG00000091446
Signal intensity (log2)
10
12
14
16
18
S1
S2
S3
S4
S5
S6
S7
S8
S9
Embryos in spawn order

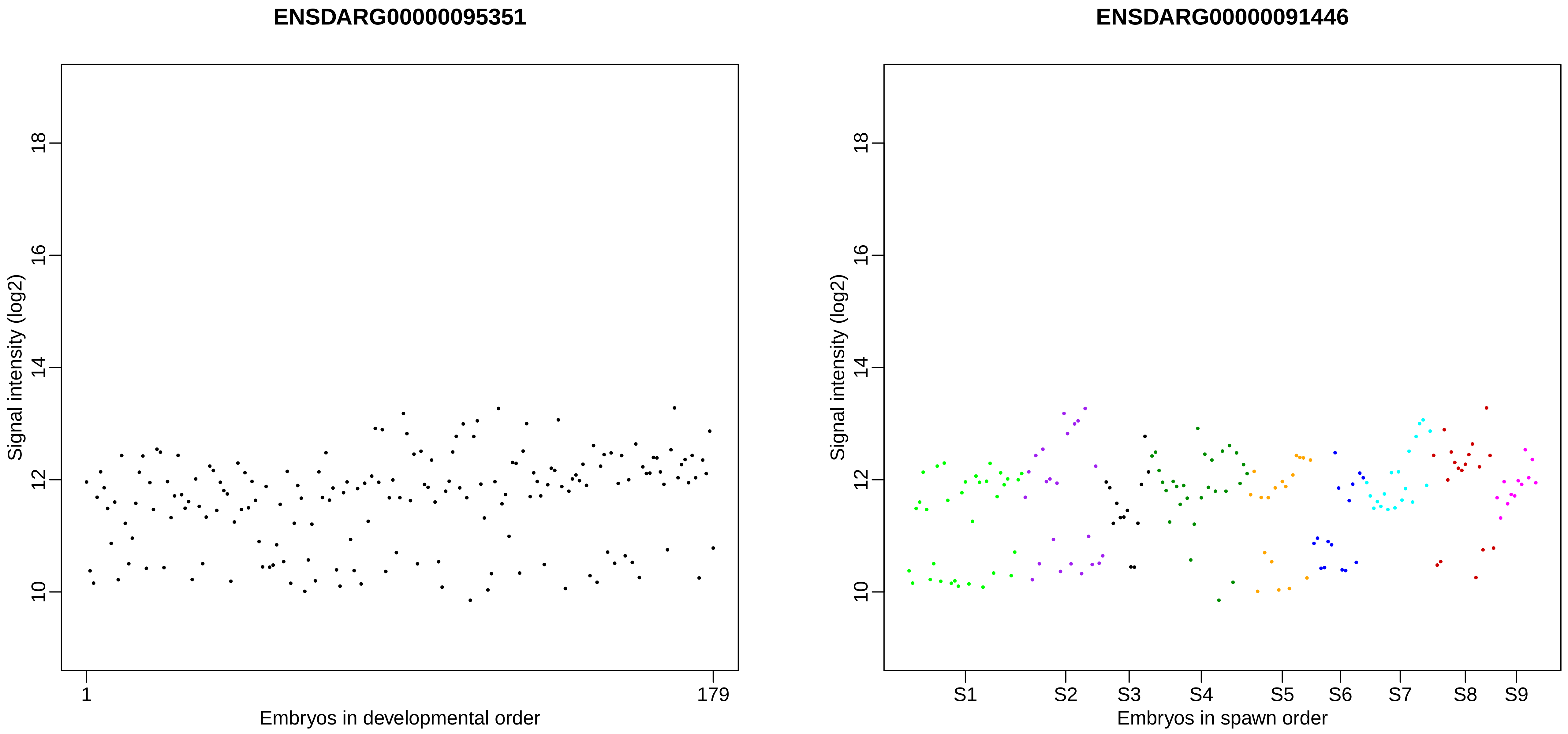

ENSDARG00000095351
Signal intensity (log2)
10
12
14
16
18
1
179
Embryos in developmental order
ENSDARG00000091446
Signal intensity (log2)
10
12
14
16
18
S1
S2
S3
S4
S5
S6
S7
S8
S9
Embryos in spawn order

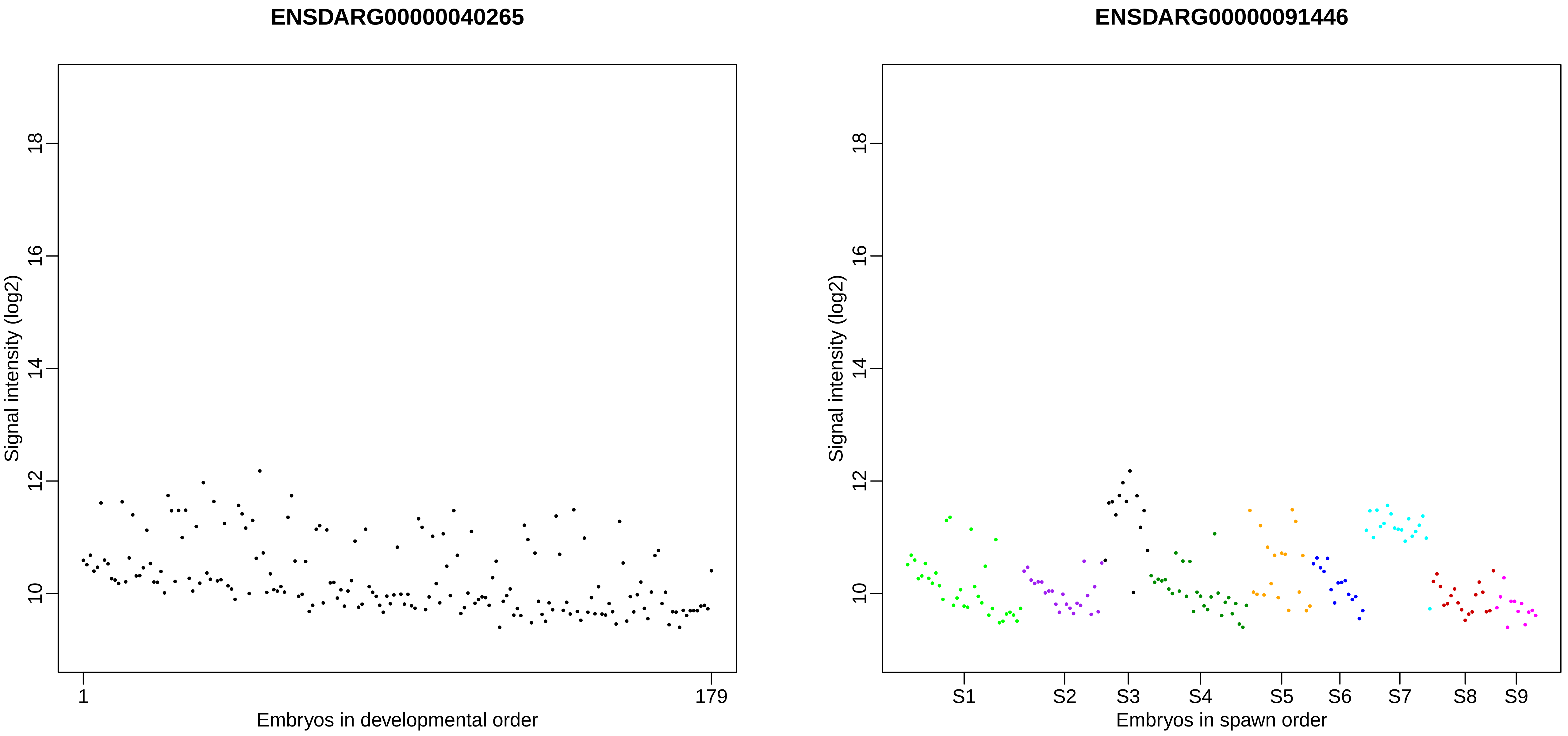

ENSDARG00000040265
Signal intensity (log2)
10
12
14
16
18
1
179
Embryos in developmental order
ENSDARG00000091446
Signal intensity (log2)
10
12
14
16
18
S1
S2
S3
S4
S5
S6
S7
S8
S9
Embryos in spawn order

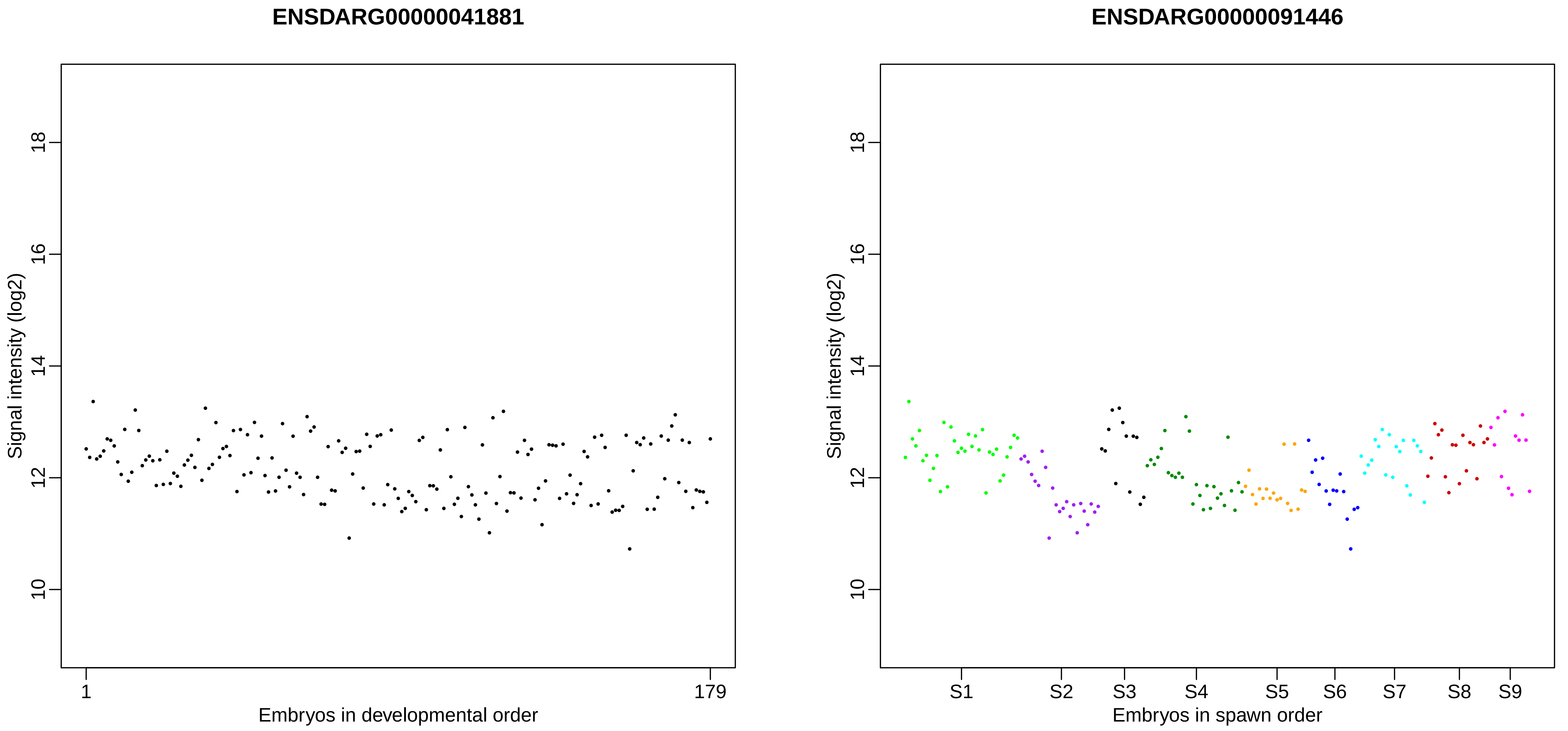
ENSDARG00000041881
Signal intensity (log2)
10
12
14
16
18
1
179
Embryos in developmental order
ENSDARG00000091446
Signal intensity (log2)
10
12
14
16
18
S1
S2
S3
S4
S5
S6
S7
S8
S9
Embryos in spawn order

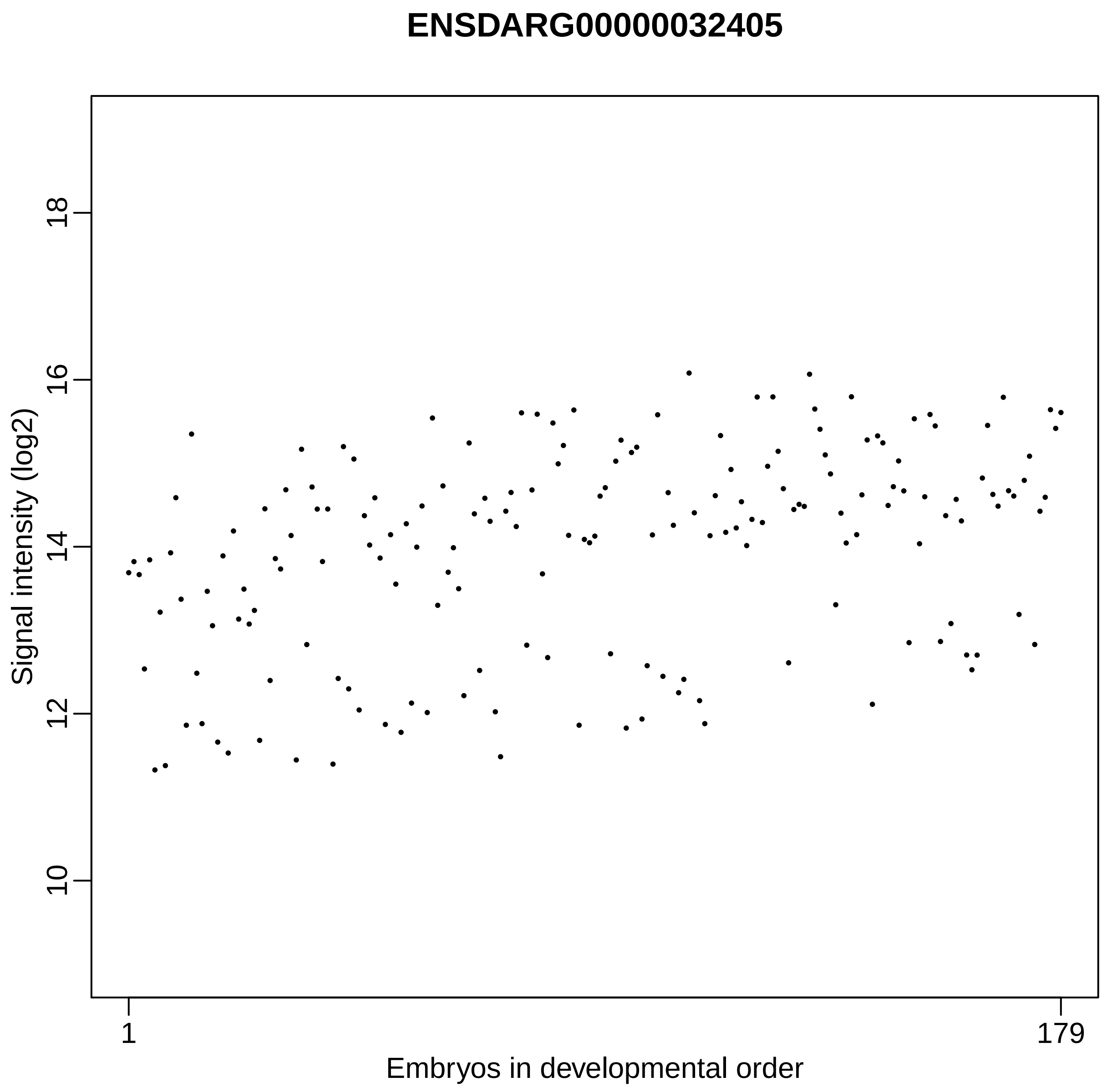
ENSDARG00000032405
Signal intensity (log2)
10
12
14
16
18
1
179
Embryos in developmental order

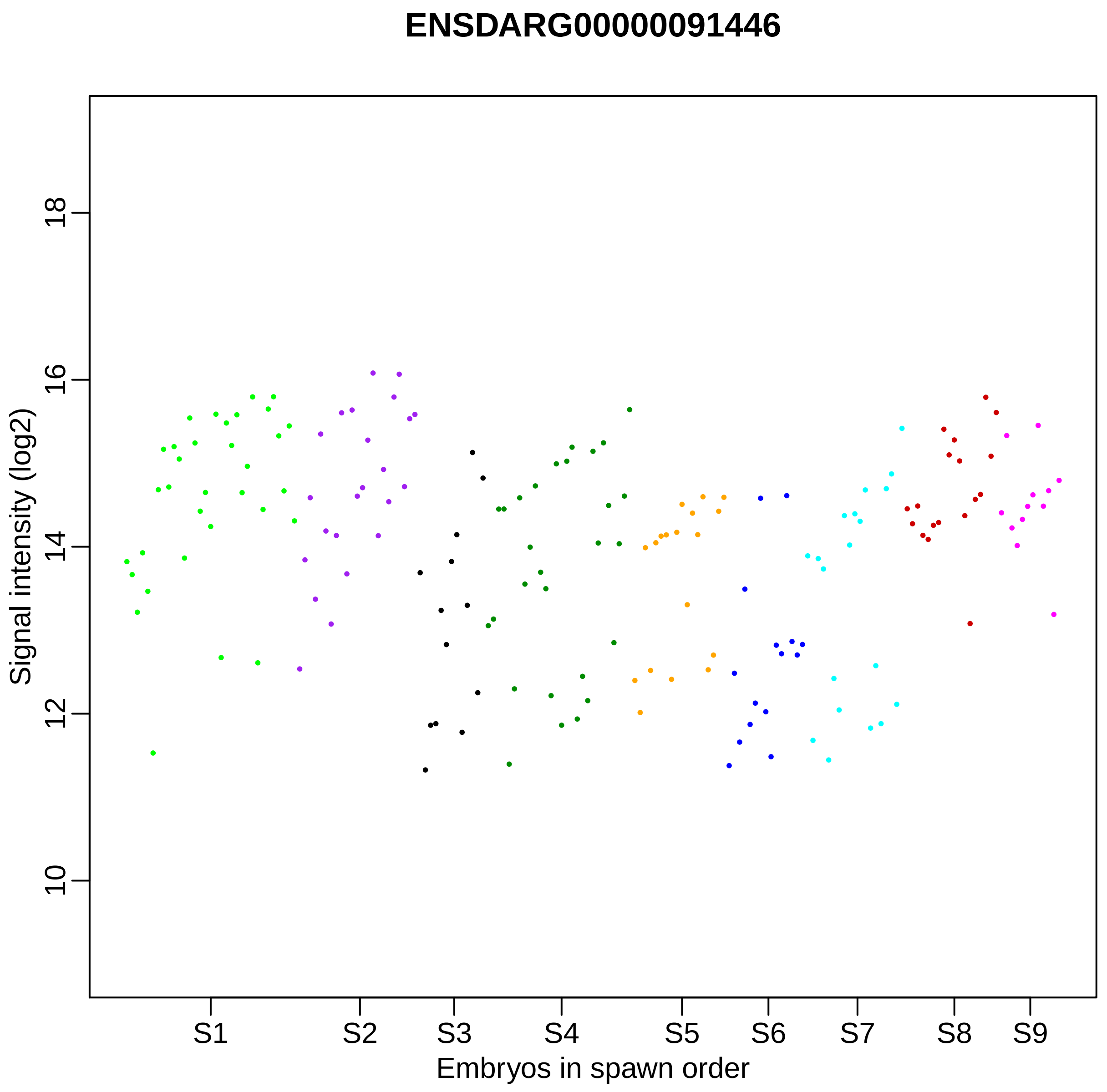
ENSDARG00000091446
Signal intensity (log2)
10
12
14
16
18
S1
S2
S3
S4
S5
S6
S7
S8
S9
Embryos in spawn order

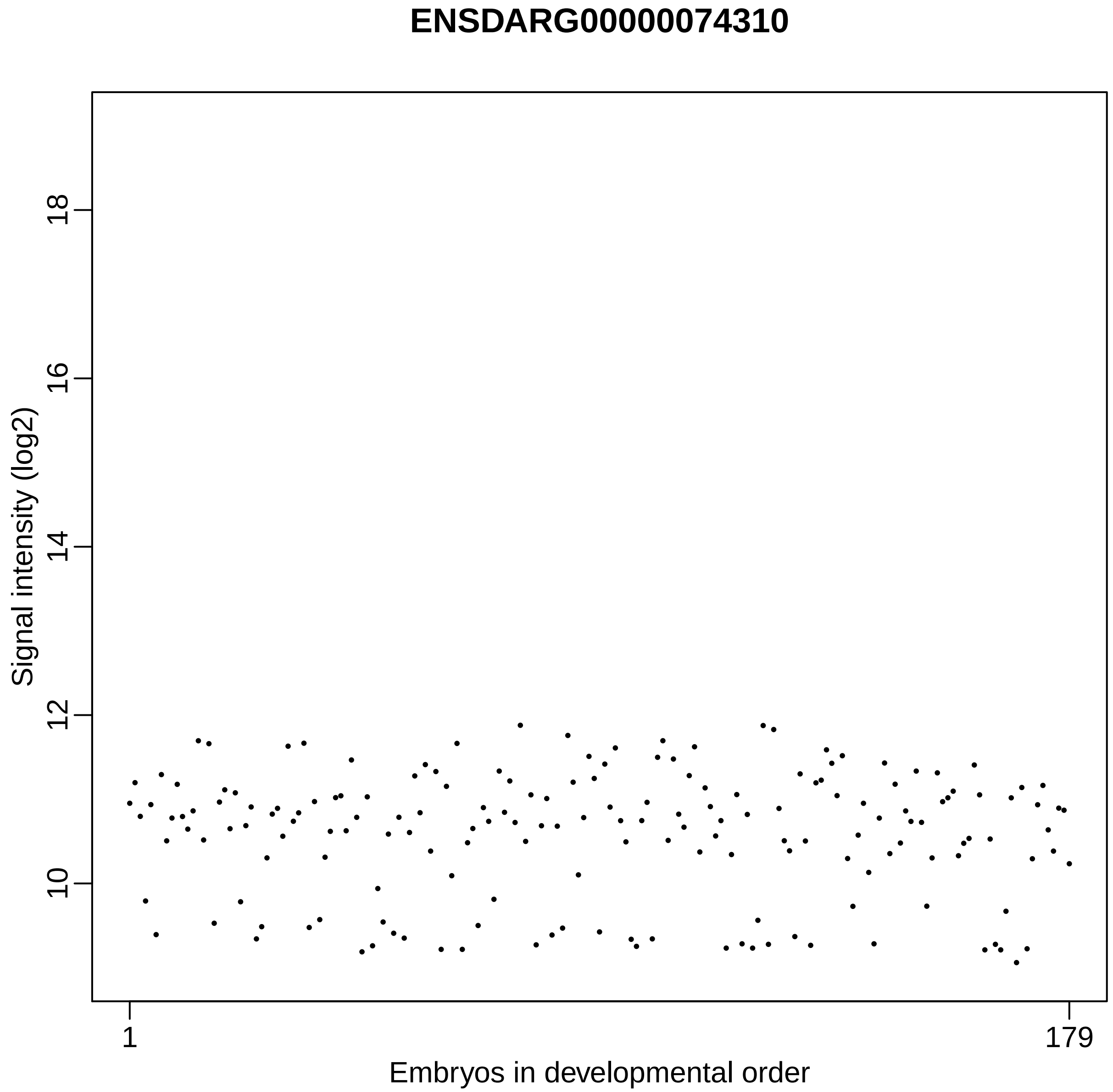

ENSDARG00000074310
Signal intensity (log2)
10
12
14
16
18
1
179
Embryos in developmental order

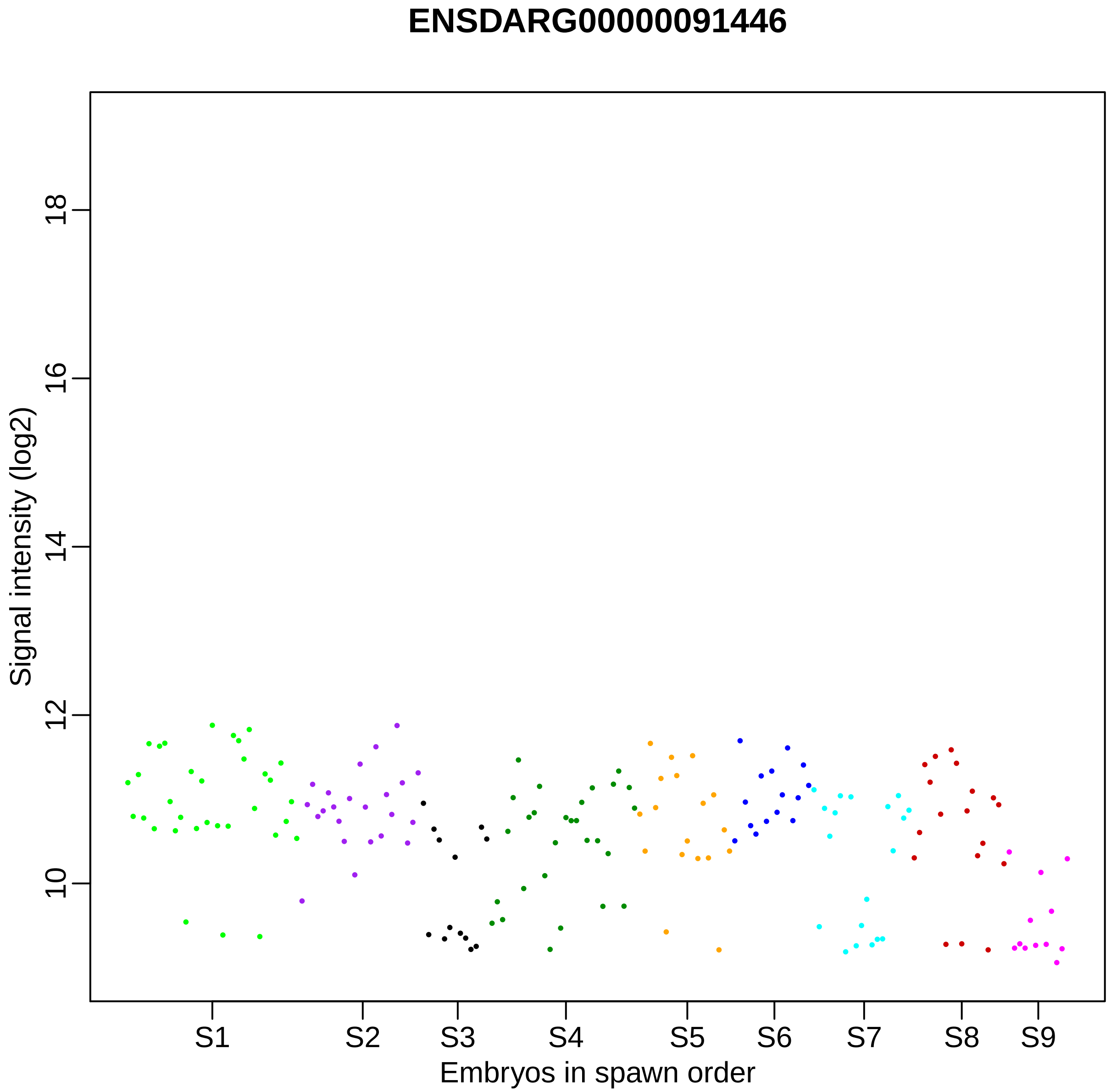

ENSDARG00000091446
Signal intensity (log2)
10
12
14
16
18
S1
S2
S3
S4
S5
S6
S7
S8
S9
Embryos in spawn order

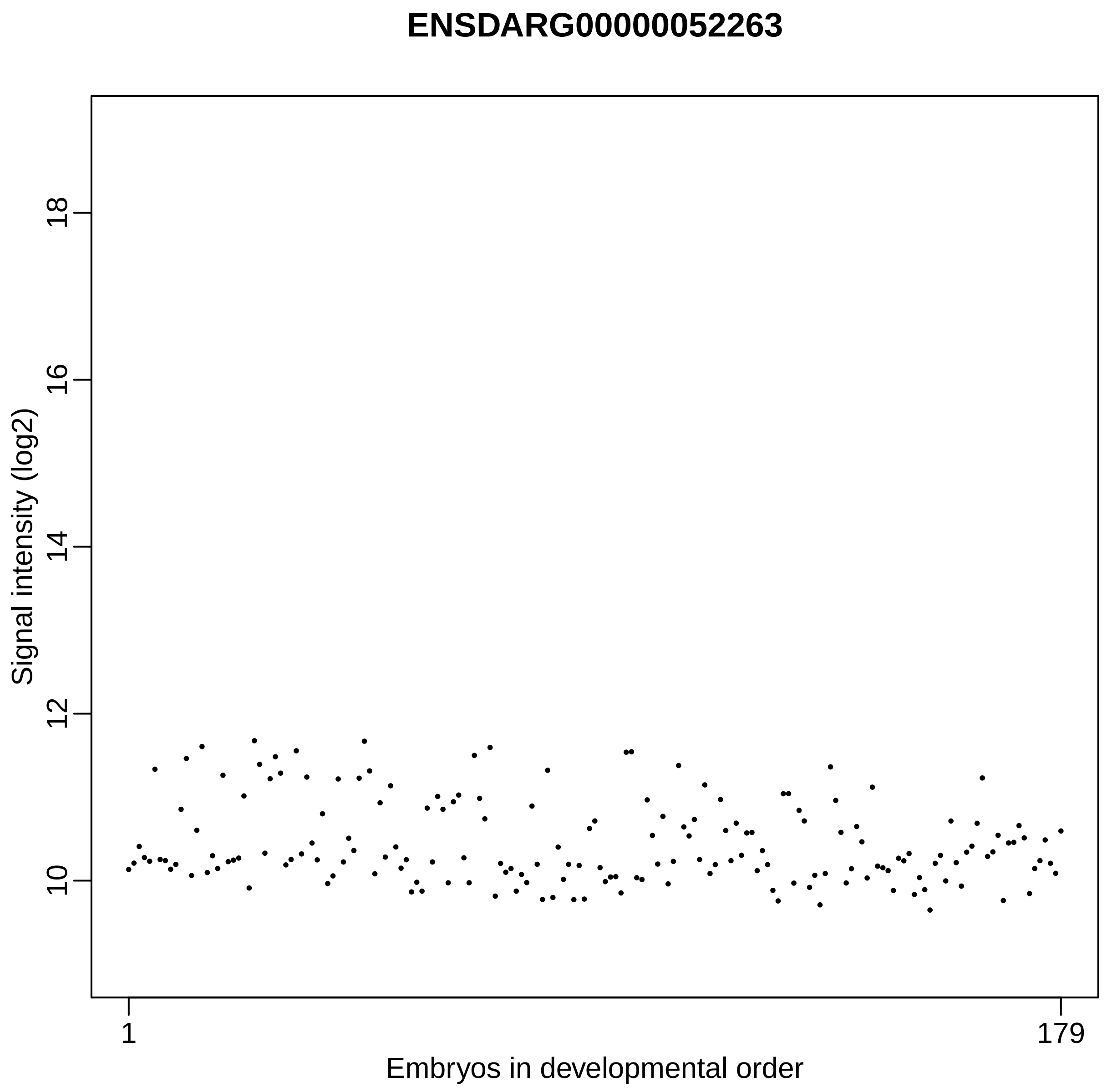
ENSDARG00000052263
Signal intensity (log2)
10
12
14
16
18
1
179
Embryos in developmental order

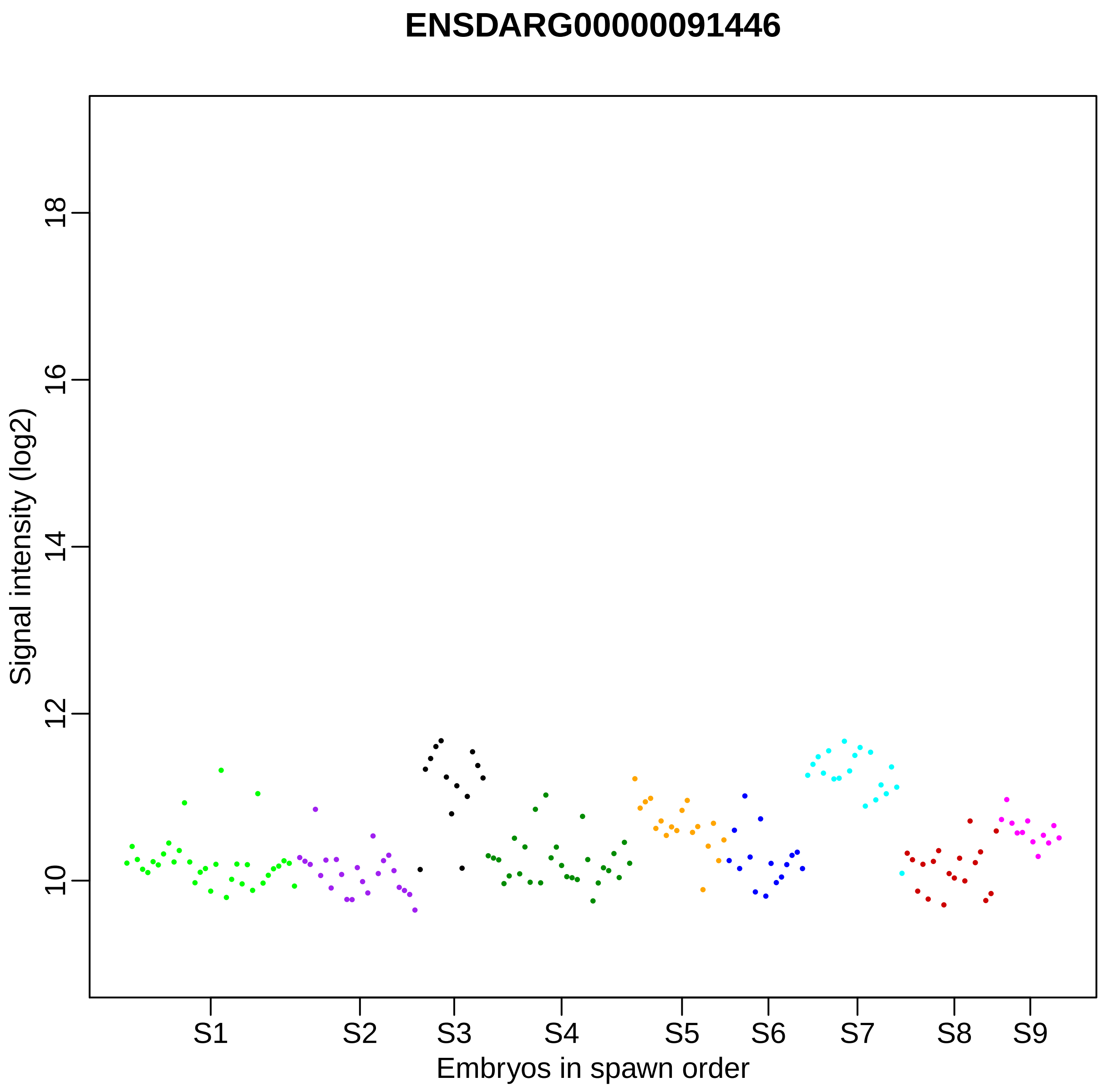
ENSDARG00000091446
Signal intensity (log2)
10
12
14
16
18
S1
S2
S3
S4
S5
S6
S7
S8
S9
Embryos in spawn order

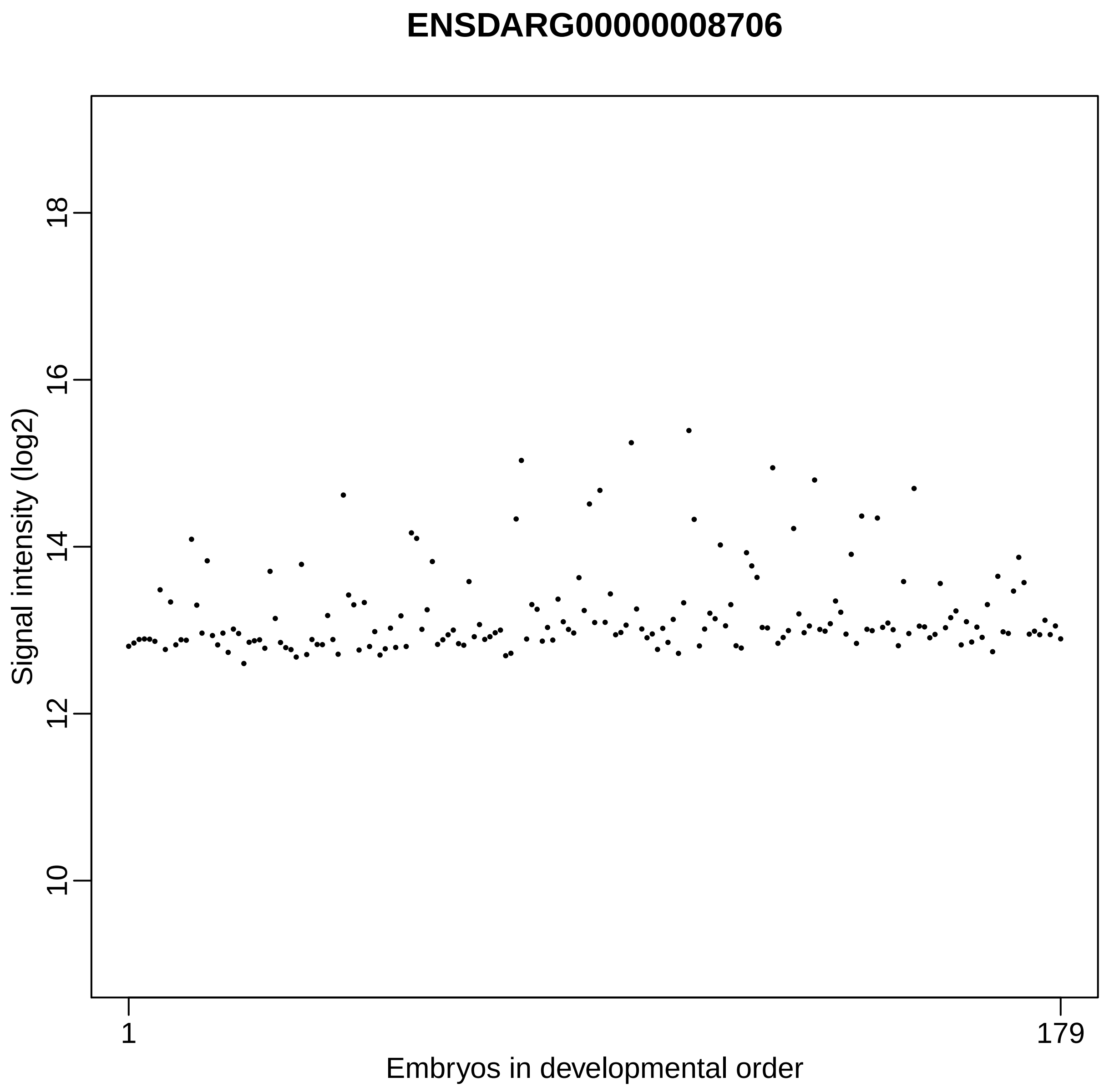
ENSDARG00000008706
Signal intensity (log2)
10
12
14
16
18
1
179
Embryos in developmental order

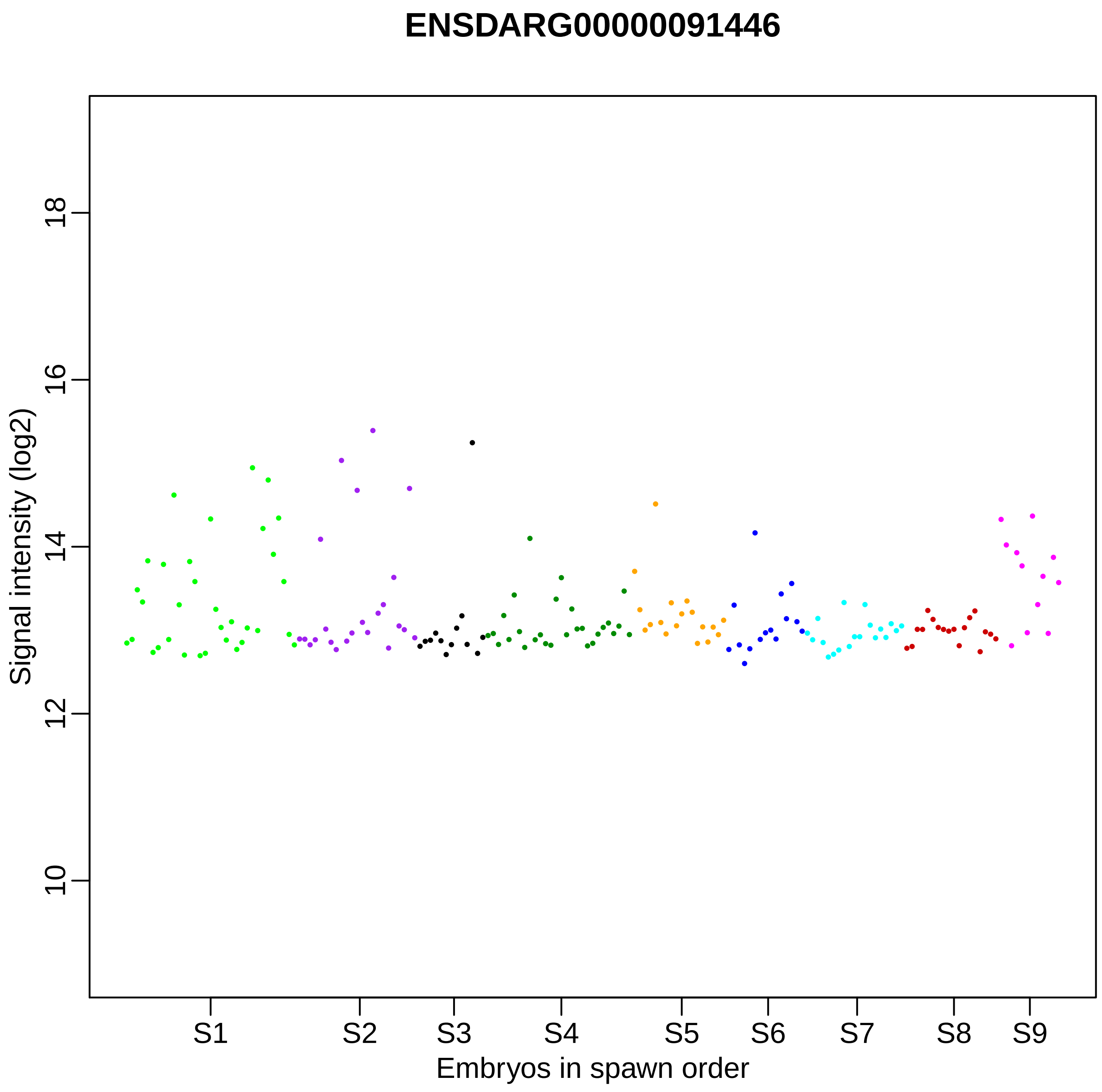
ENSDARG00000091446
Signal intensity (log2)
10
12
14
16
18
S1
S2
S3
S4
S5
S6
S7
S8
S9
Embryos in spawn order

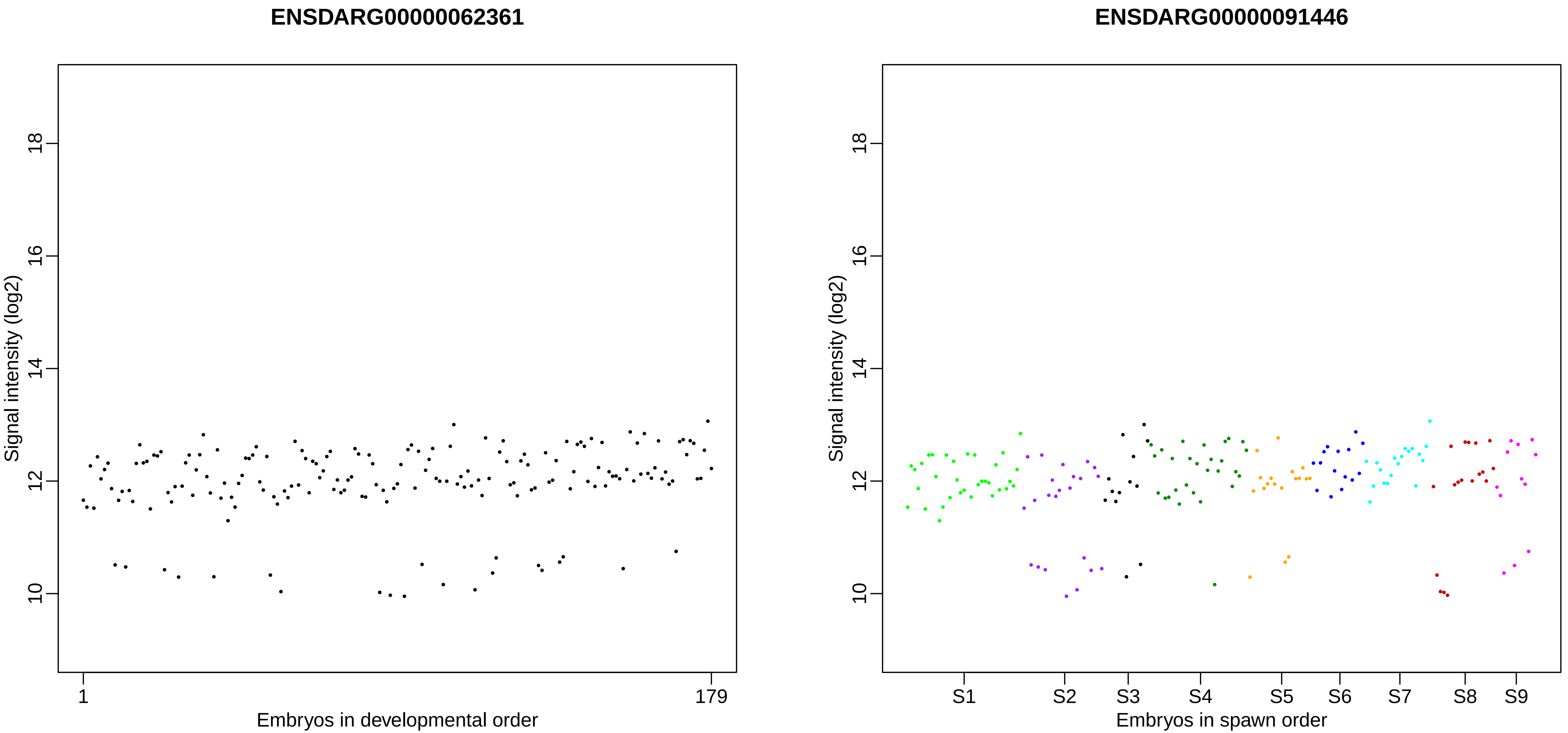

ENSDARG00000062361
Signal intensity (log2)
10
12
14
16
18
1
179
Embryos in developmental order
ENSDARG00000091446
Signal intensity (log2)
10
12
14
16
18
S1
S2
S3
S4
S5
S6
S7
S8
S9
Embryos in spawn order

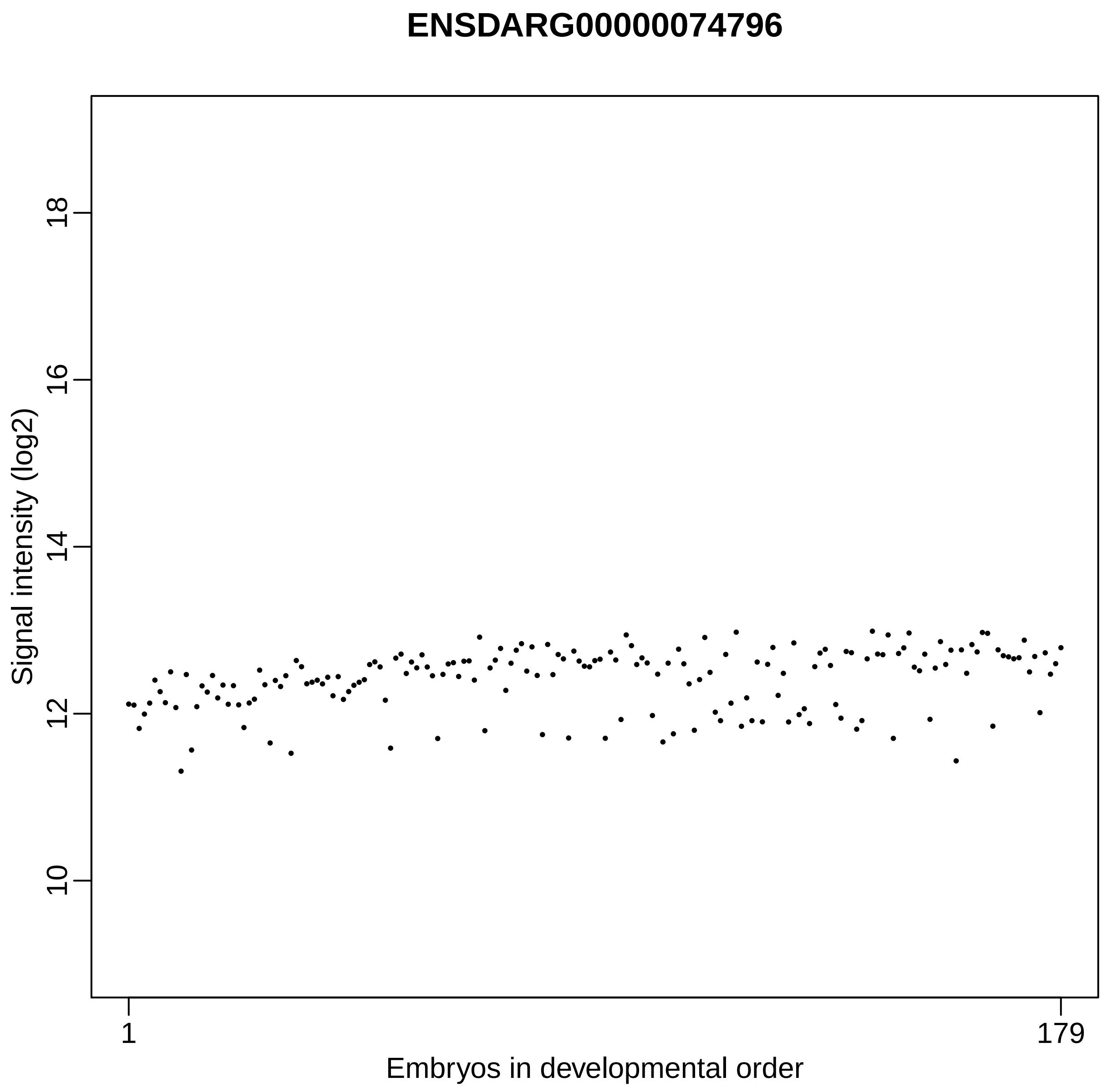
ENSDARG00000074796
Signal intensity (log2)
10
12
14
16
18
1
179
Embryos in developmental order

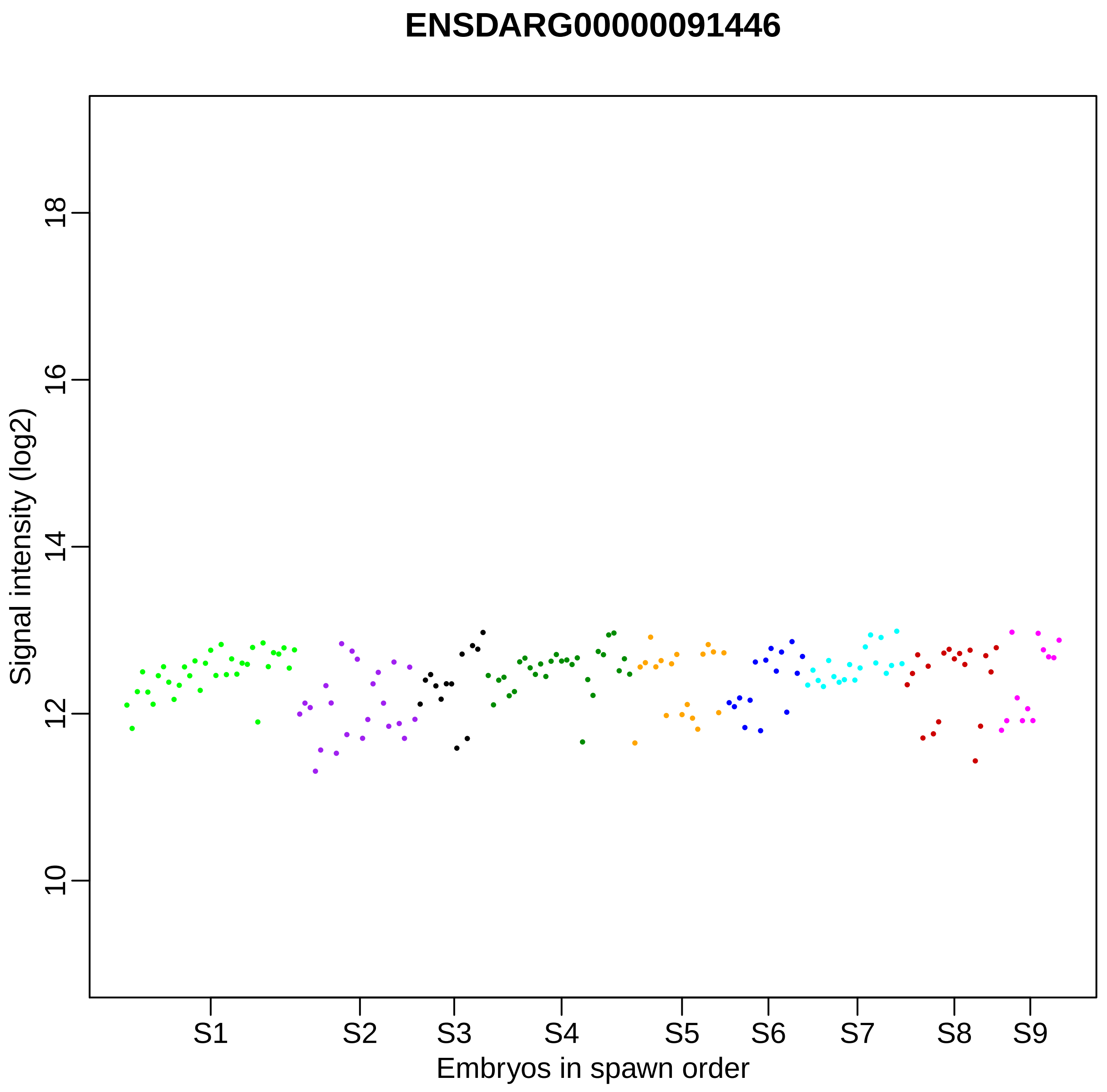
ENSDARG00000091446
Signal intensity (log2)
10
12
14
16
18
S1
S2
S3
S4
S5
S6
S7
S8
S9
Embryos in spawn order

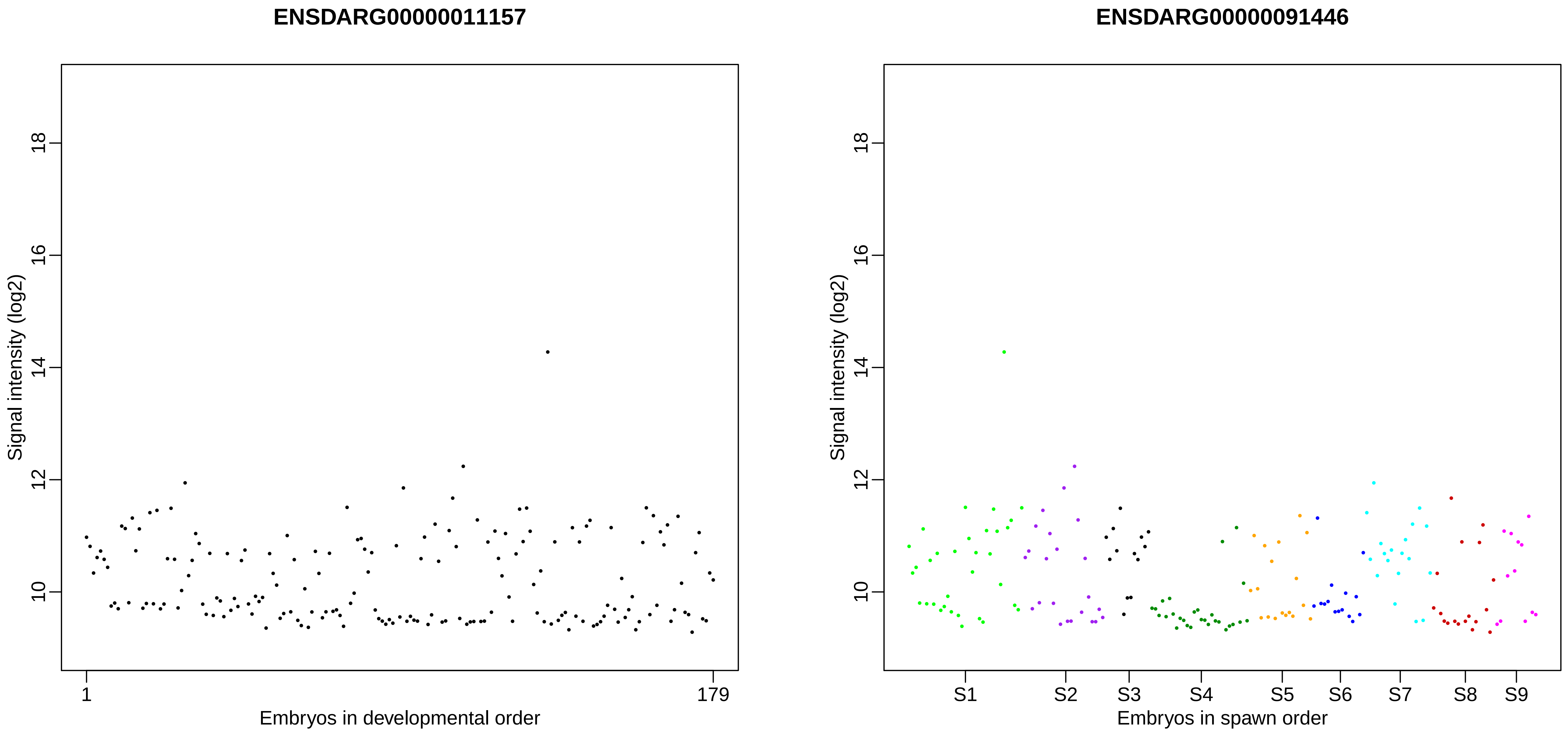

ENSDARG00000011157
Signal intensity (log2)
10
12
14
16
18
1
179
Embryos in developmental order
ENSDARG00000091446
Signal intensity (log2)
10
12
14
16
18
S1
S2
S3
S4
S5
S6
S7
S8
S9
Embryos in spawn order

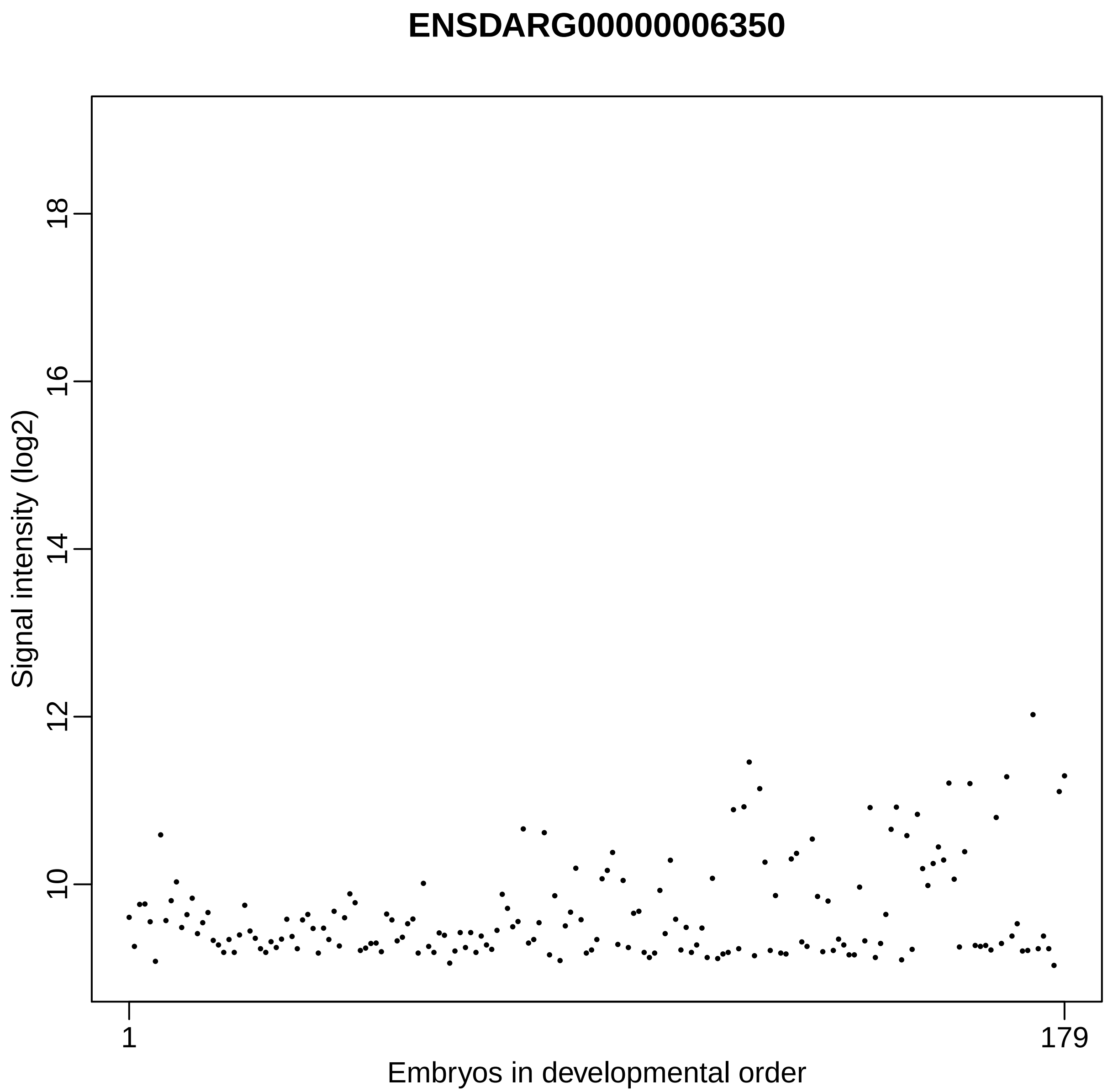

ENSDARG00000006350
Signal intensity (log2)
10
12
14
16
18
1
179
Embryos in developmental order

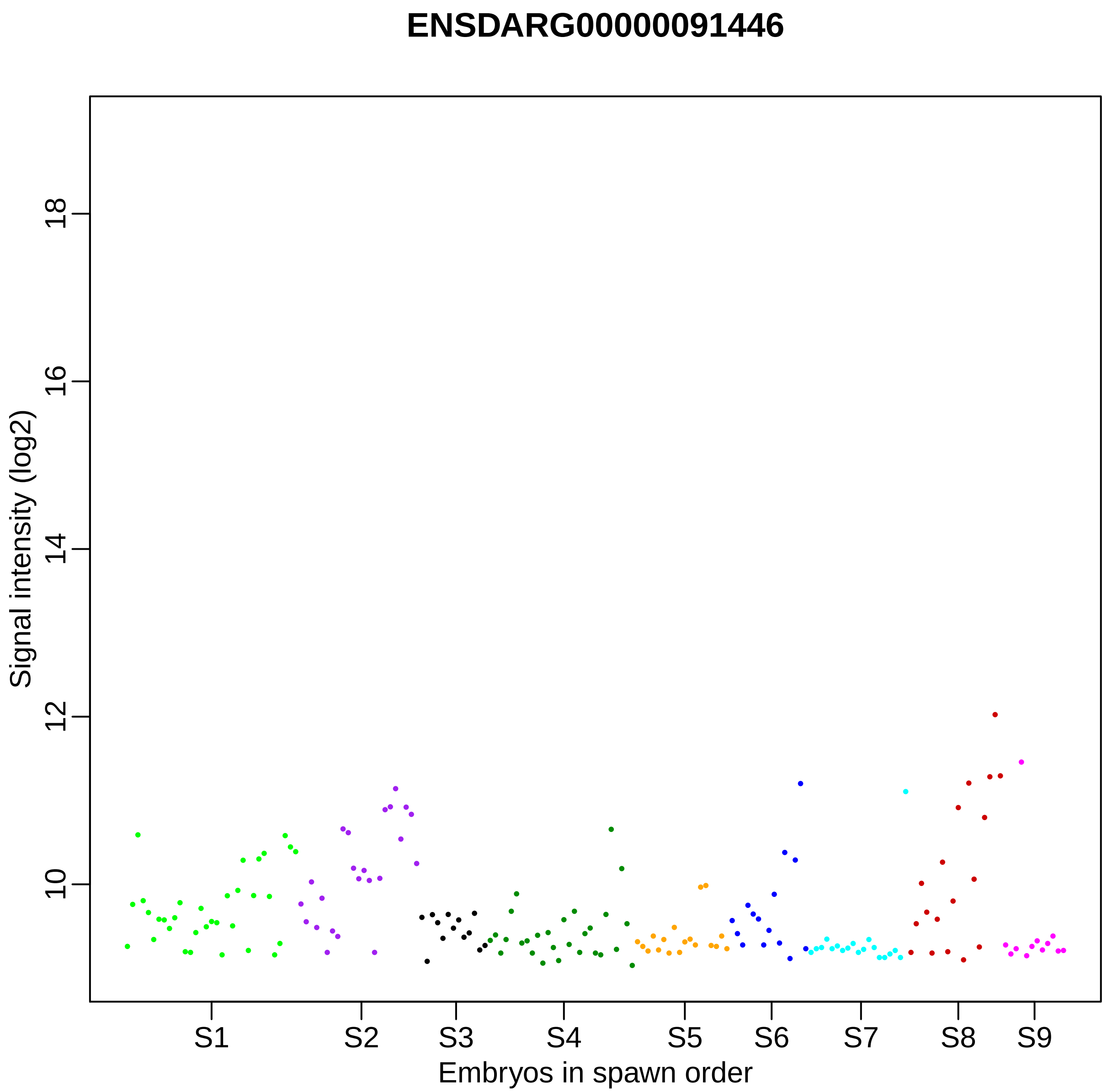

ENSDARG00000091446
Signal intensity (log2)
10
12
14
16
18
S1
S2
S3
S4
S5
S6
S7
S8
S9
Embryos in spawn order

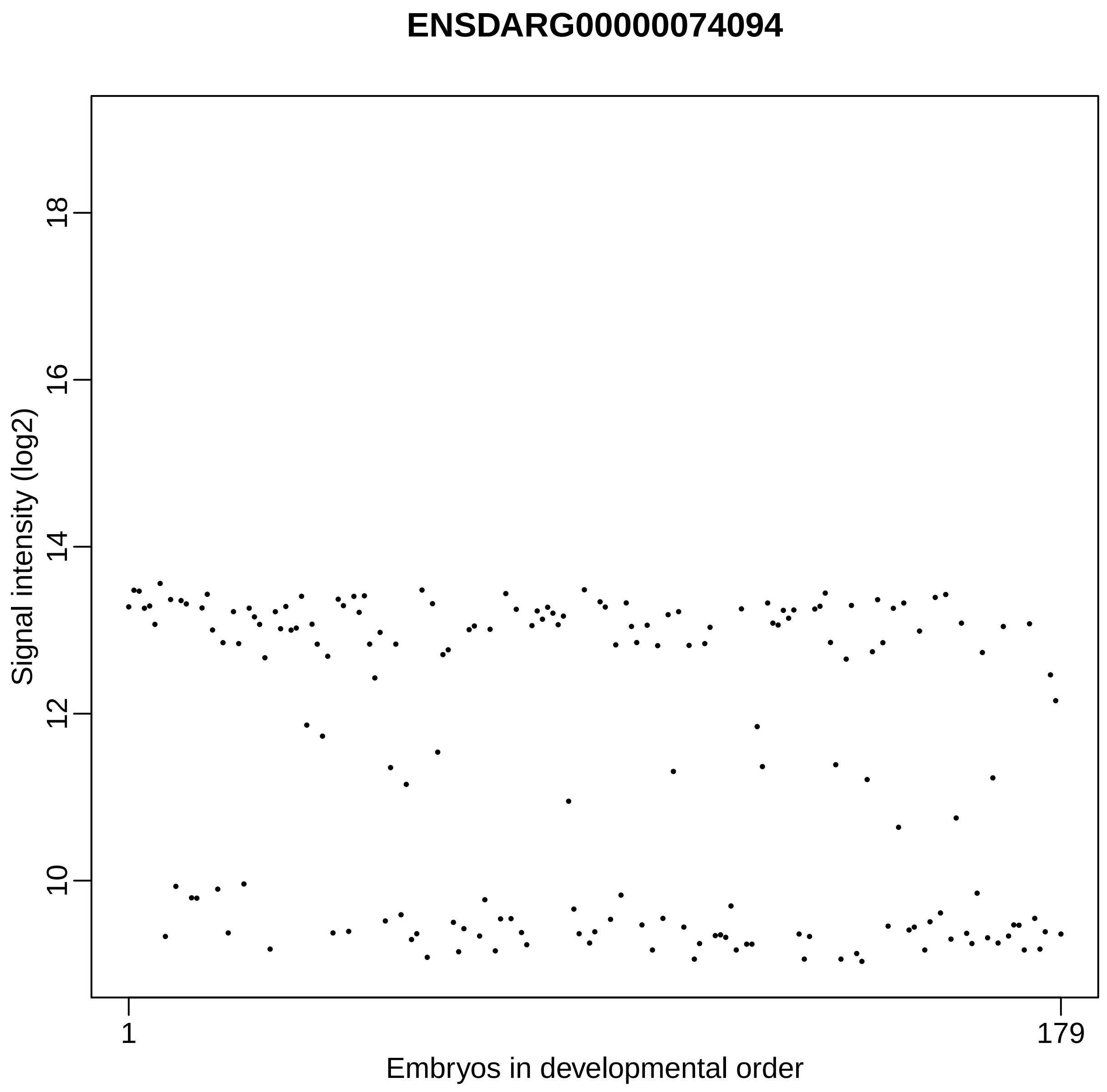
ENSDARG00000074094
Signal intensity (log2)
10
12
14
16
18
1
179
Embryos in developmental order

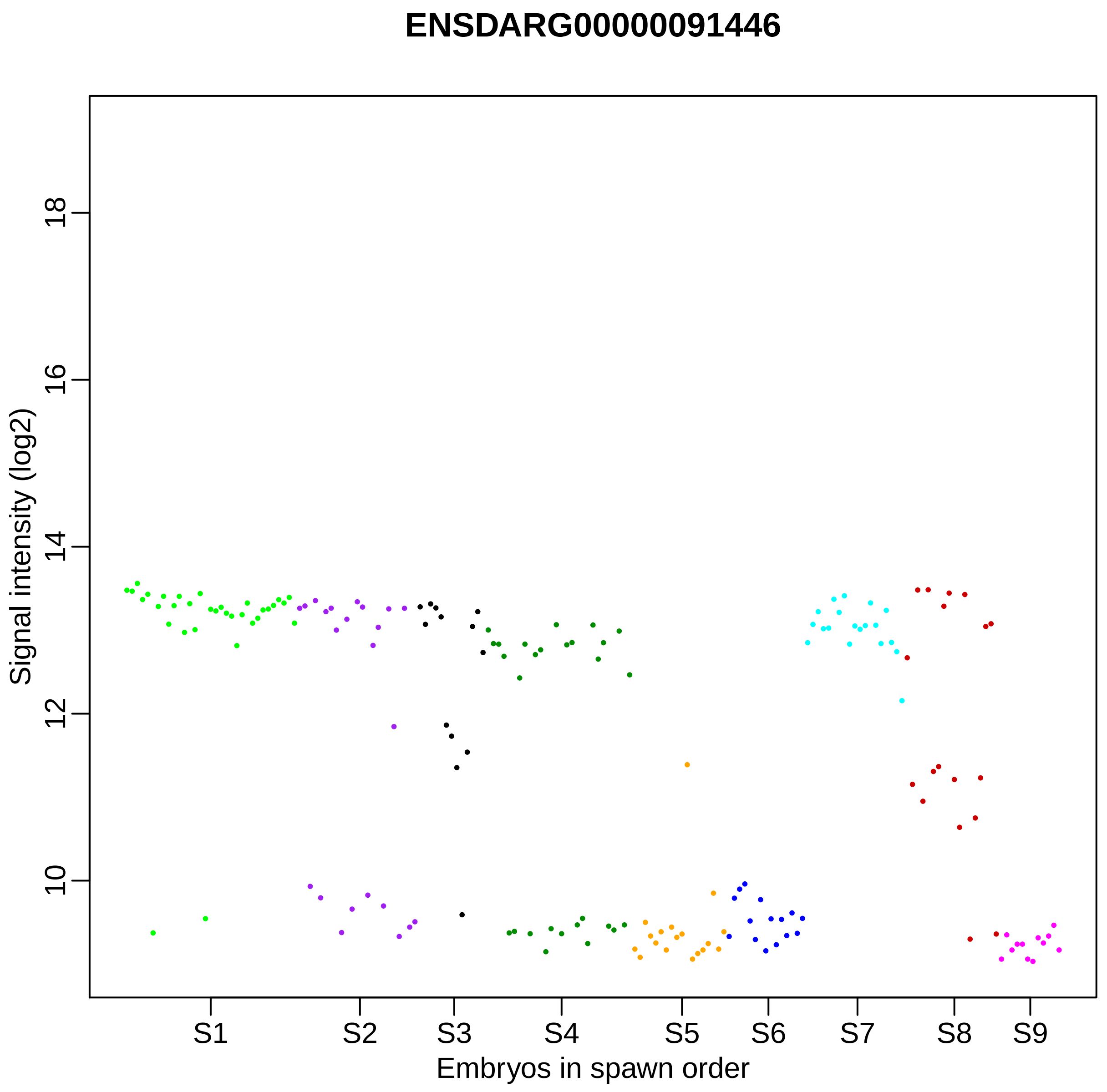
ENSDARG00000091446
Signal intensity (log2)
10
12
14
16
18
S1
S2
S3
S4
S5
S6
S7
S8
S9
Embryos in spawn order

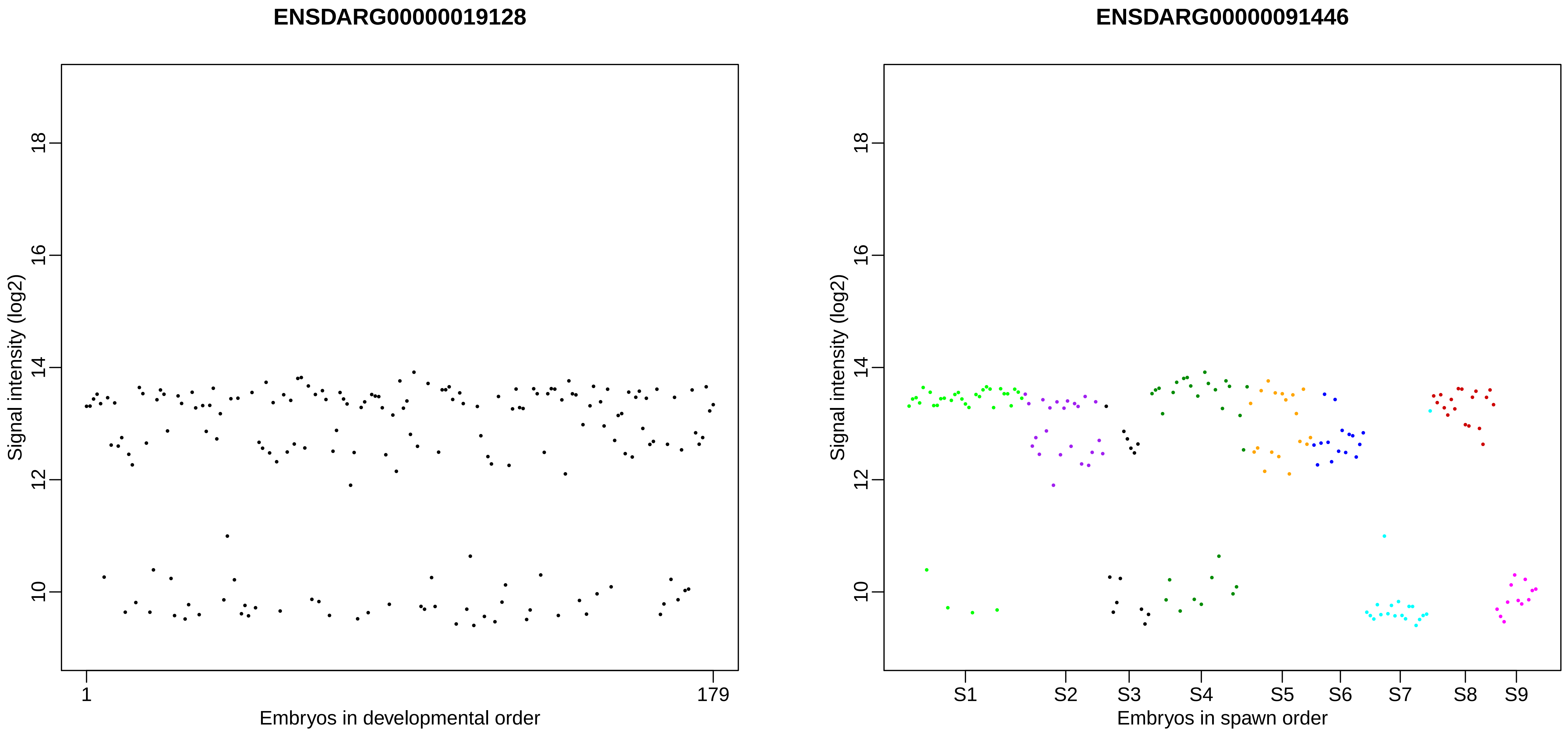

ENSDARG00000019128
Signal intensity (log2)
18
16
14
12
10
1
179
Embryos in developmental order
ENSDARG00000091446
Signal intensity (log2)
18
16
14
12
10
S1
S2
S3
S4
S5
S6
S7
S8
S9
Embryos in spawn order

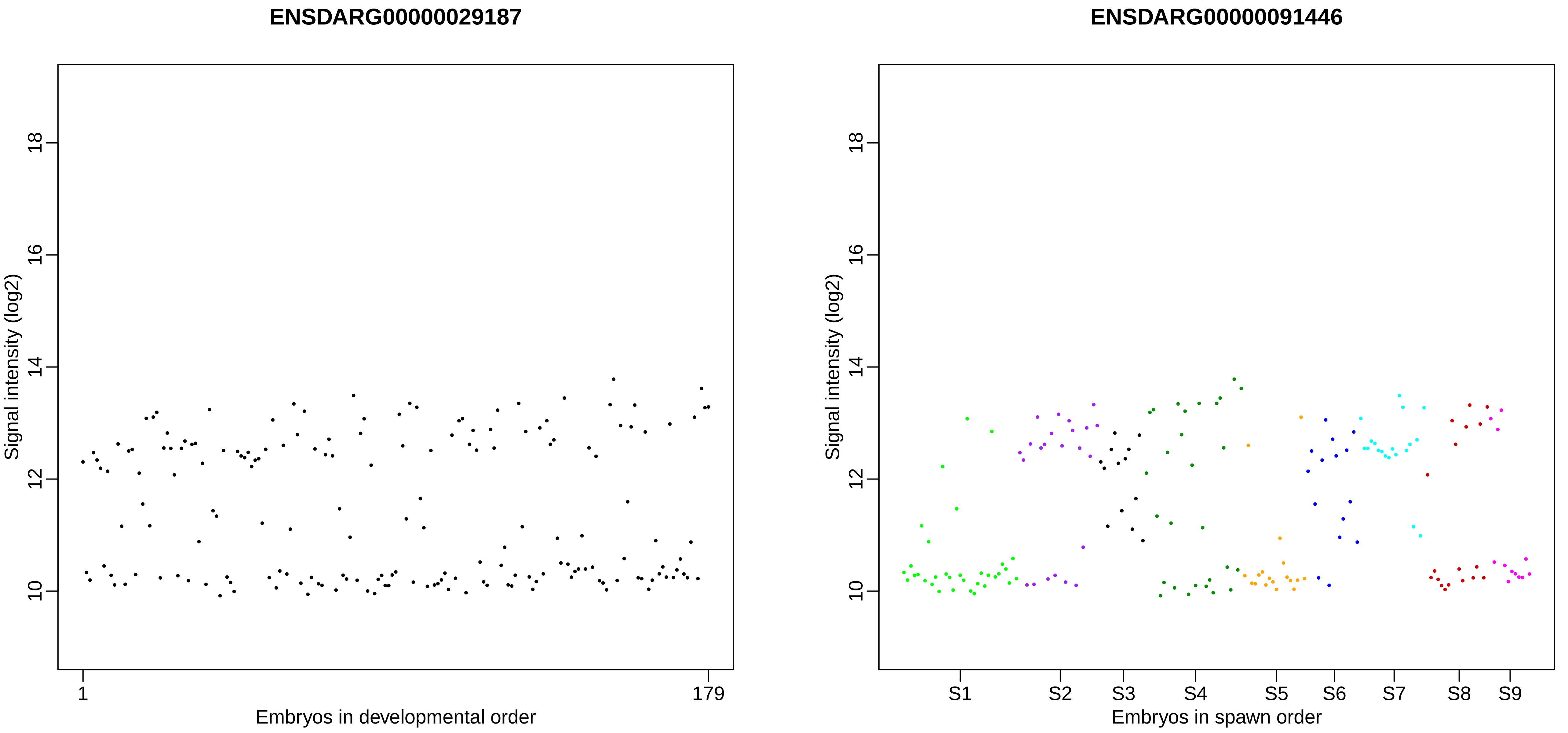
ENSDARG00000029187
Signal intensity (log2)
10
12
14
16
18
1
179
Embryos in developmental order
ENSDARG00000091446
Signal intensity (log2)
10
12
14
16
18
S1
S2
S3
S4
S5
S6
S7
S8
S9
Embryos in spawn order

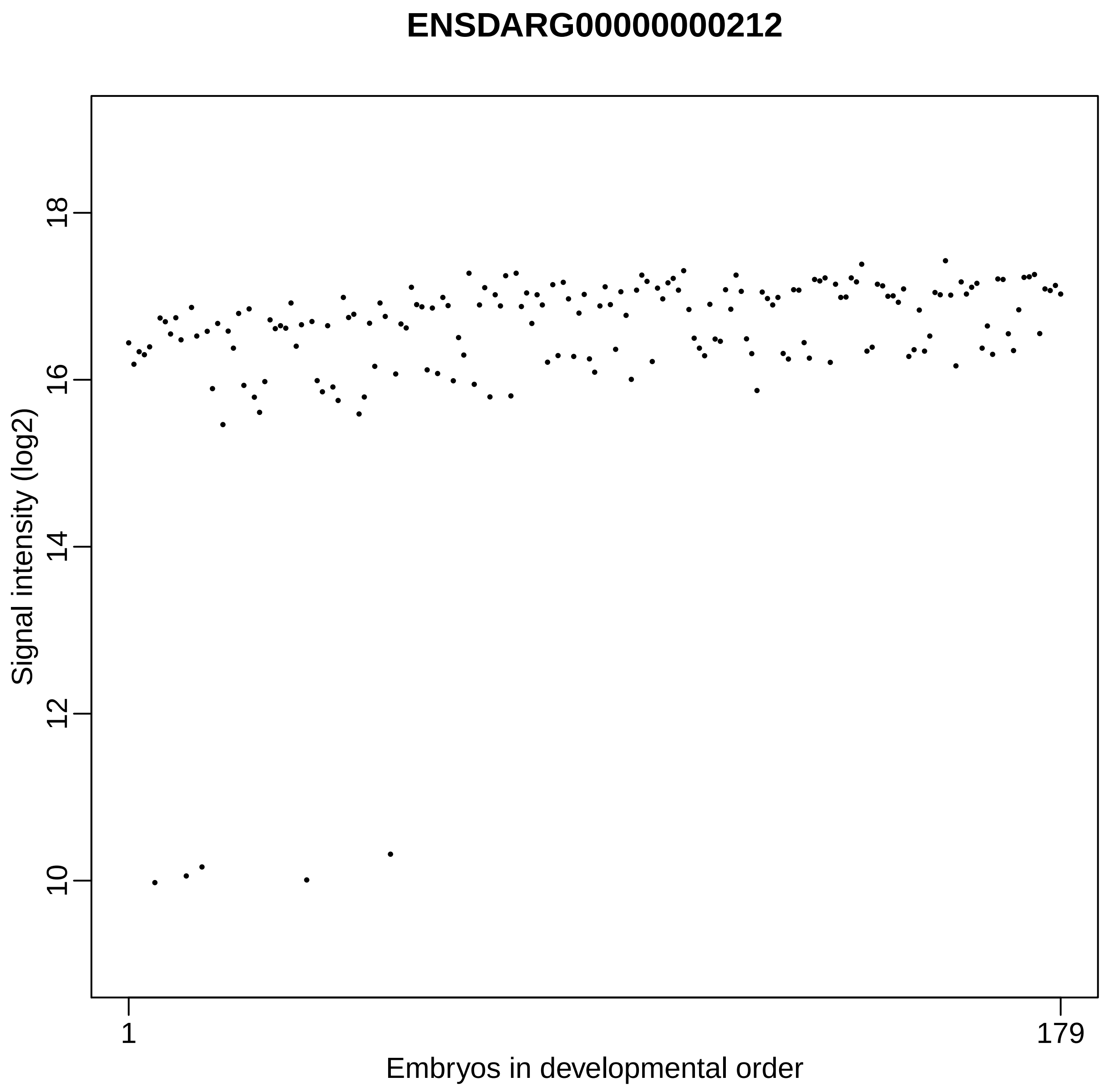
ENSDARG00000000212
Signal intensity (log2)
10
12
14
16
18
1
179
Embryos in developmental order

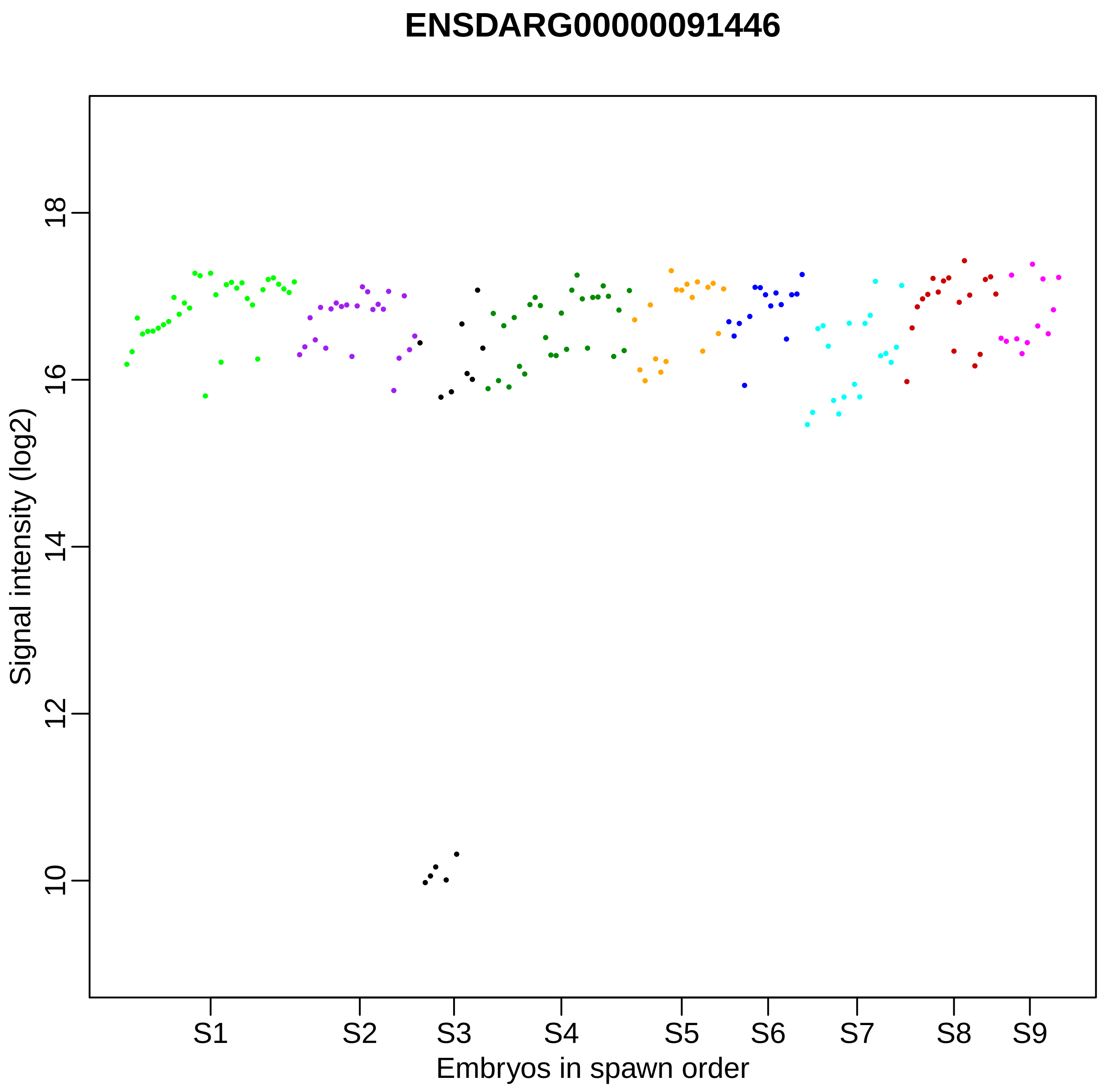
ENSDARG00000091446
Signal intensity (log2)
10
12
14
16
18
S1
S2
S3
S4
S5
S6
S7
S8
S9
Embryos in spawn order

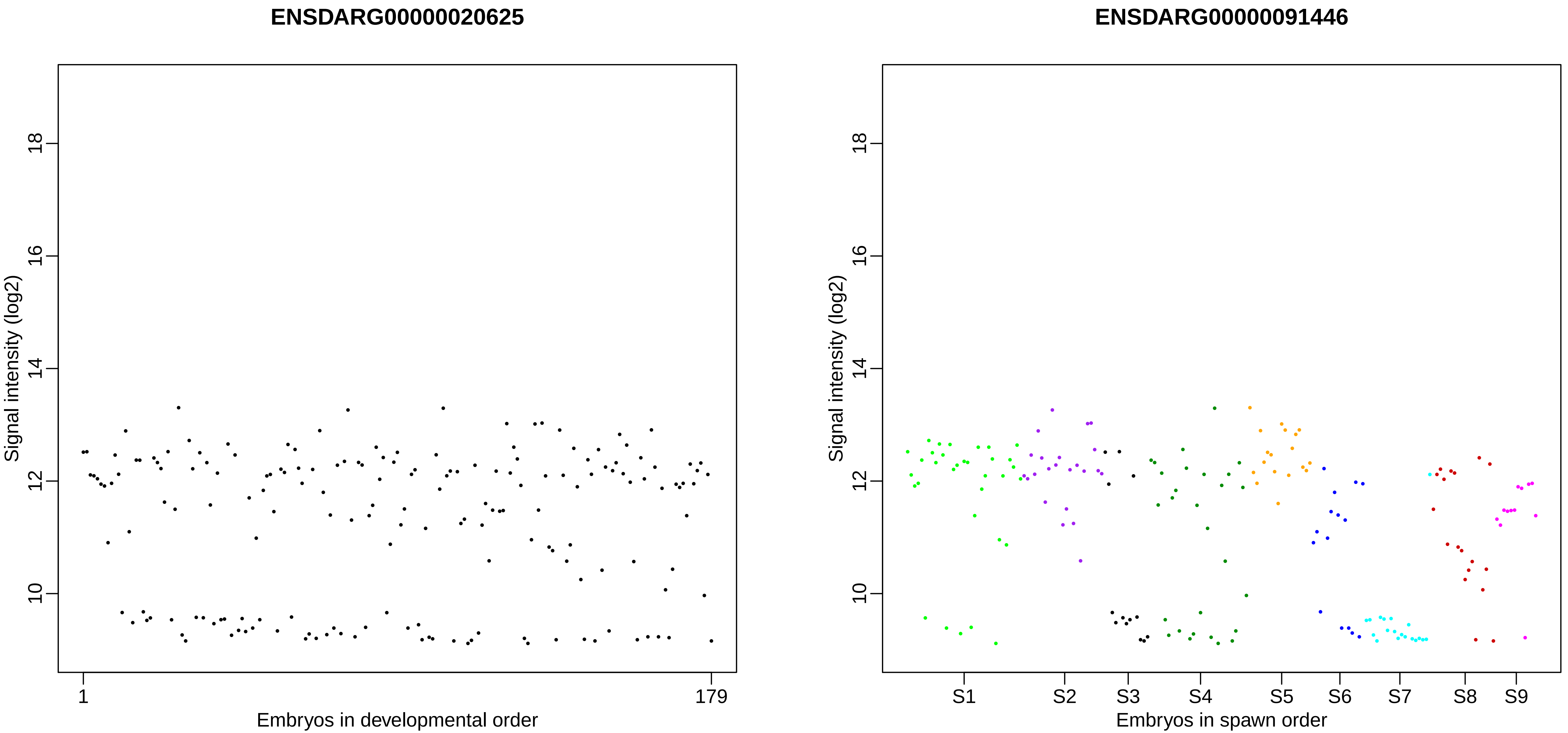

ENSDARG00000020625
Signal intensity (log2)
10
12
14
16
18
1
179
Embryos in developmental order
ENSDARG00000091446
Signal intensity (log2)
10
12
14
16
18
S1
S2
S3
S4
S5
S6
S7
S8
S9
Embryos in spawn order
