# Supplementary material for: Transcriptome dynamics in early zebrafish embryogenesis determined by high-resolution time course analysis of 180 successive, individual zebrafish embryos
Source: BMC Genomics. 2017 Apr 11;18:287. doi: 10.1186/s12864-017-3672-z (PMC5387192; doi:10.1186/s12864-017-3672-z)
Supplement: Supplementary file 13 — First two principal components of the intensity matrix of the expressed Ensembl genes. The variance explained by the components is indicated between brackets as percentages. Each embryo is colored by spawn as indicated. (PDF 26 kb) [file 12864_2017_3672_MOESM13_ESM.pdf]

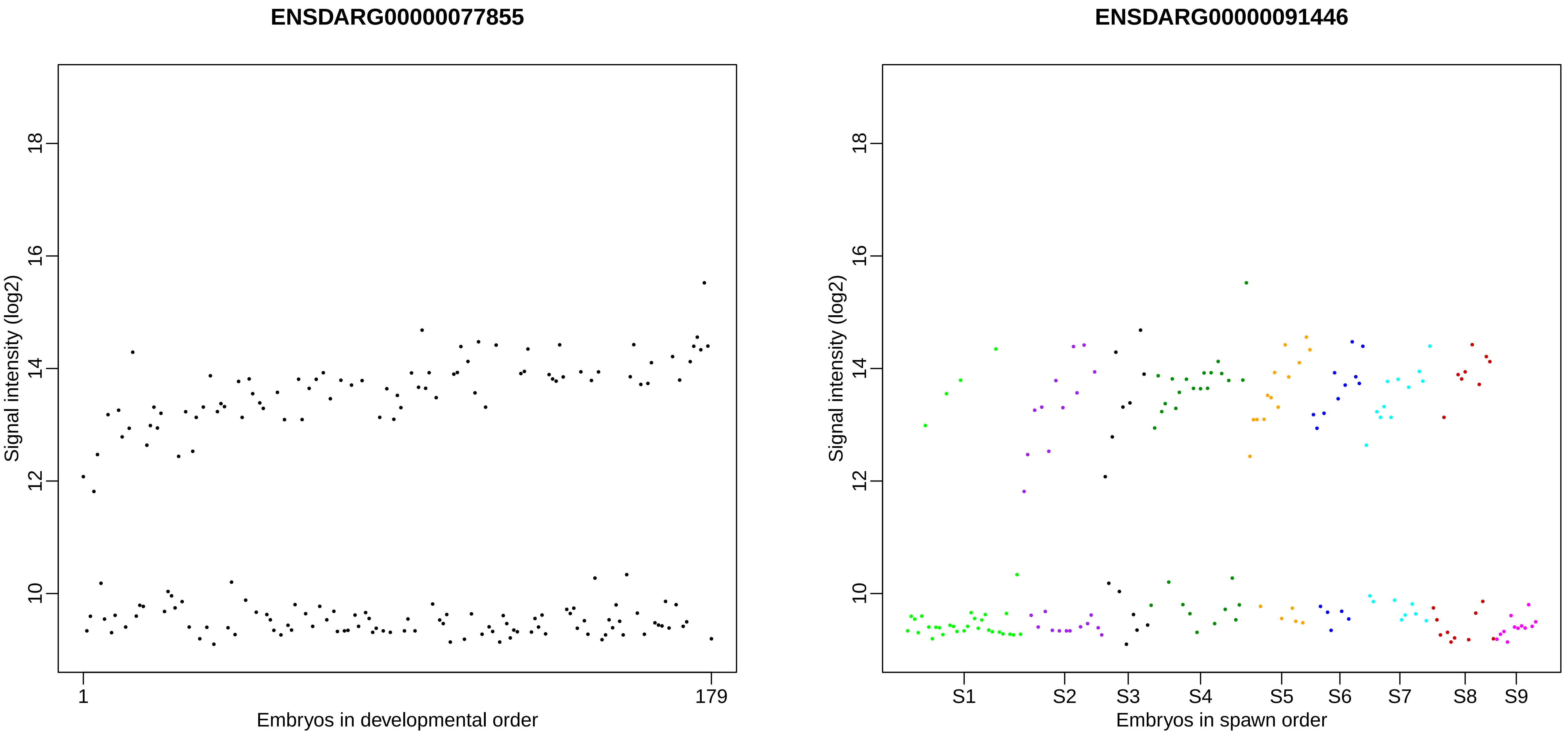

ENSDARG00000077855
Signal intensity (log2)
10
12
14
16
18
1
179
Embryos in developmental order
ENSDARG00000091446
Signal intensity (log2)
10
12
14
16
18
S1
S2
S3
S4
S5
S6
S7
S8
S9
Embryos in spawn order

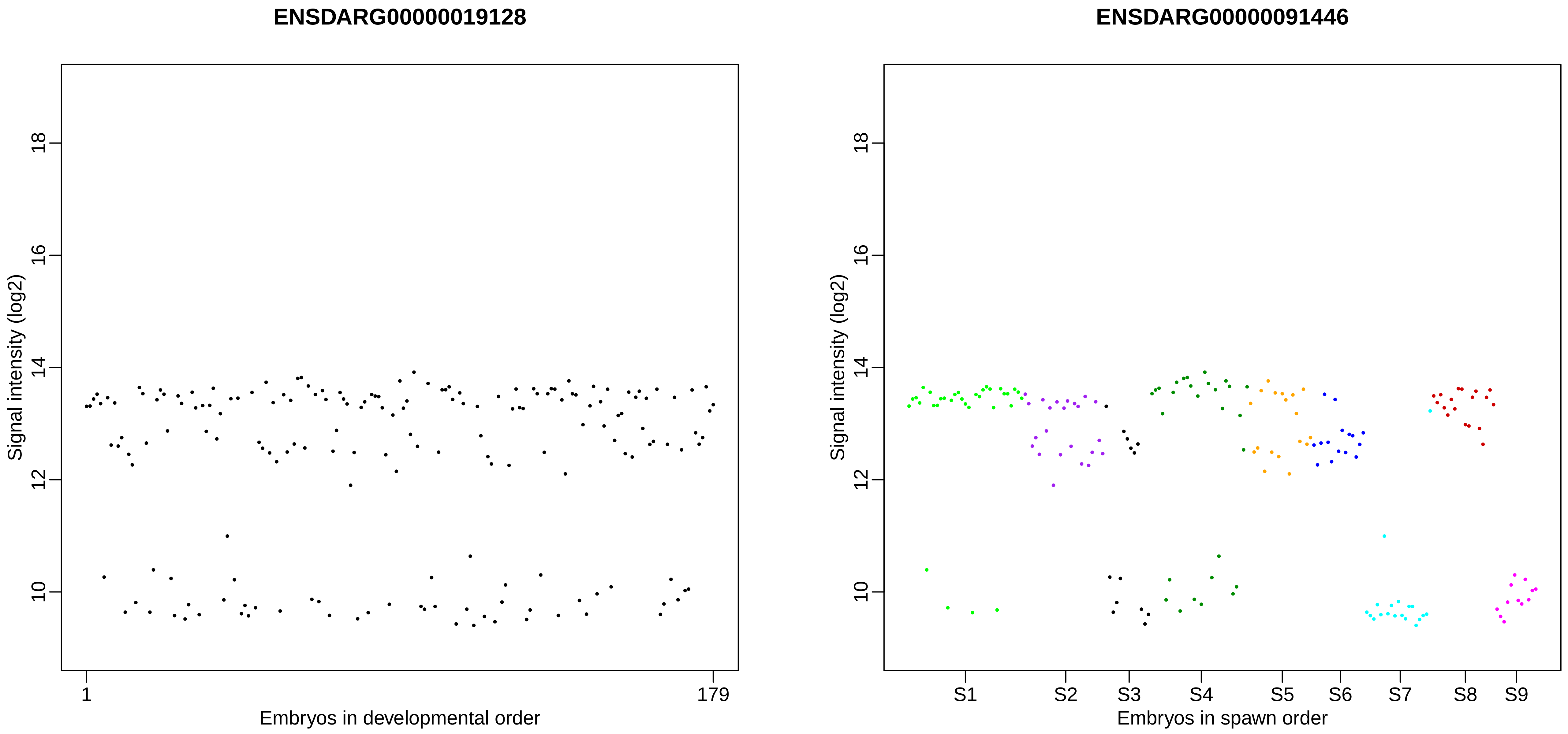
ENSDARG00000019128
Signal intensity (log2)
10
12
14
16
18
1
179
Embryos in developmental order
ENSDARG00000091446
Signal intensity (log2)
10
12
14
16
18
S1
S2
S3
S4
S5
S6
S7
S8
S9
Embryos in spawn order

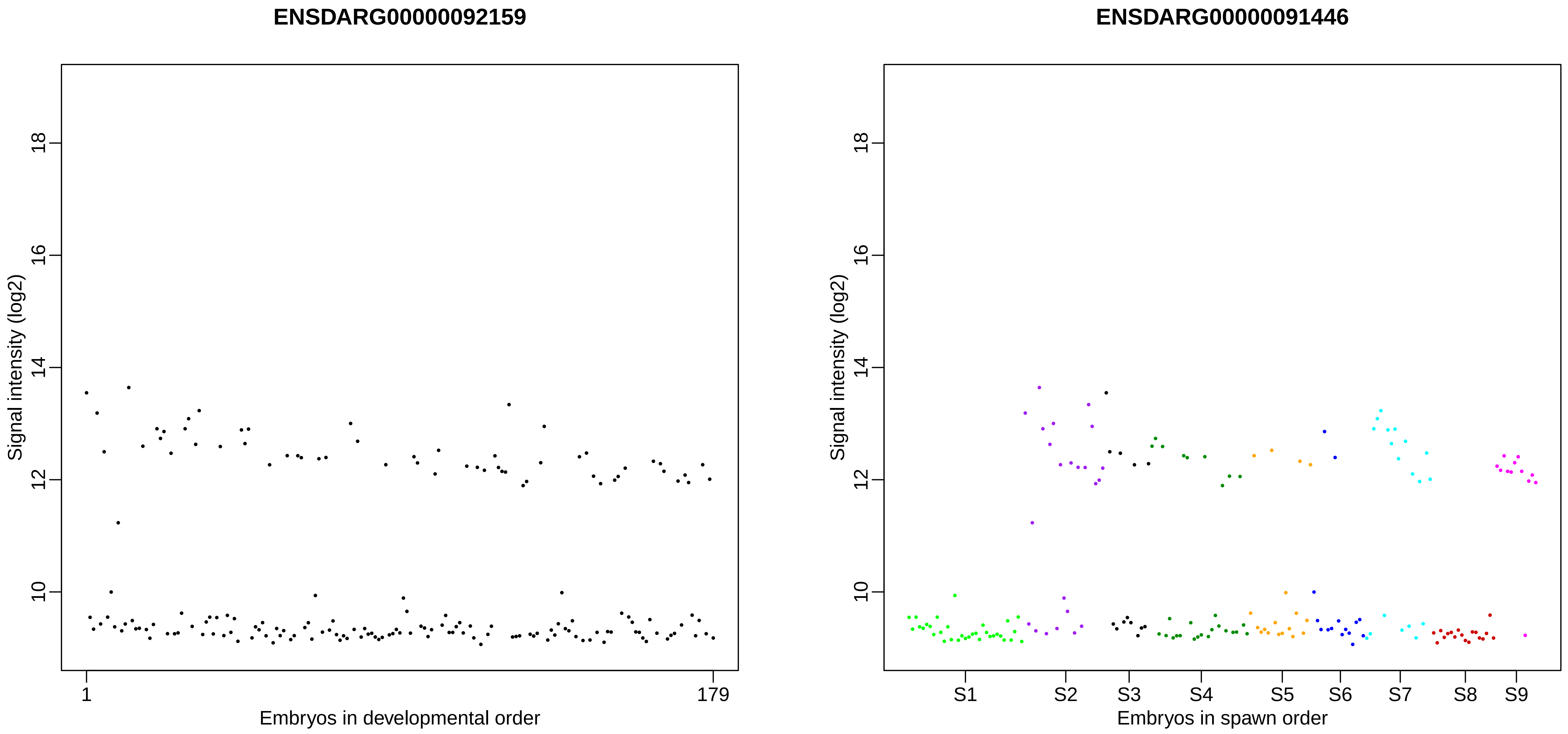
ENSDARG00000092159
Signal intensity (log2)
10
12
14
16
18
1
179
Embryos in developmental order
ENSDARG00000091446
Signal intensity (log2)
10
12
14
16
18
S1
S2
S3
S4
S5
S6
S7
S8
S9
Embryos in spawn order

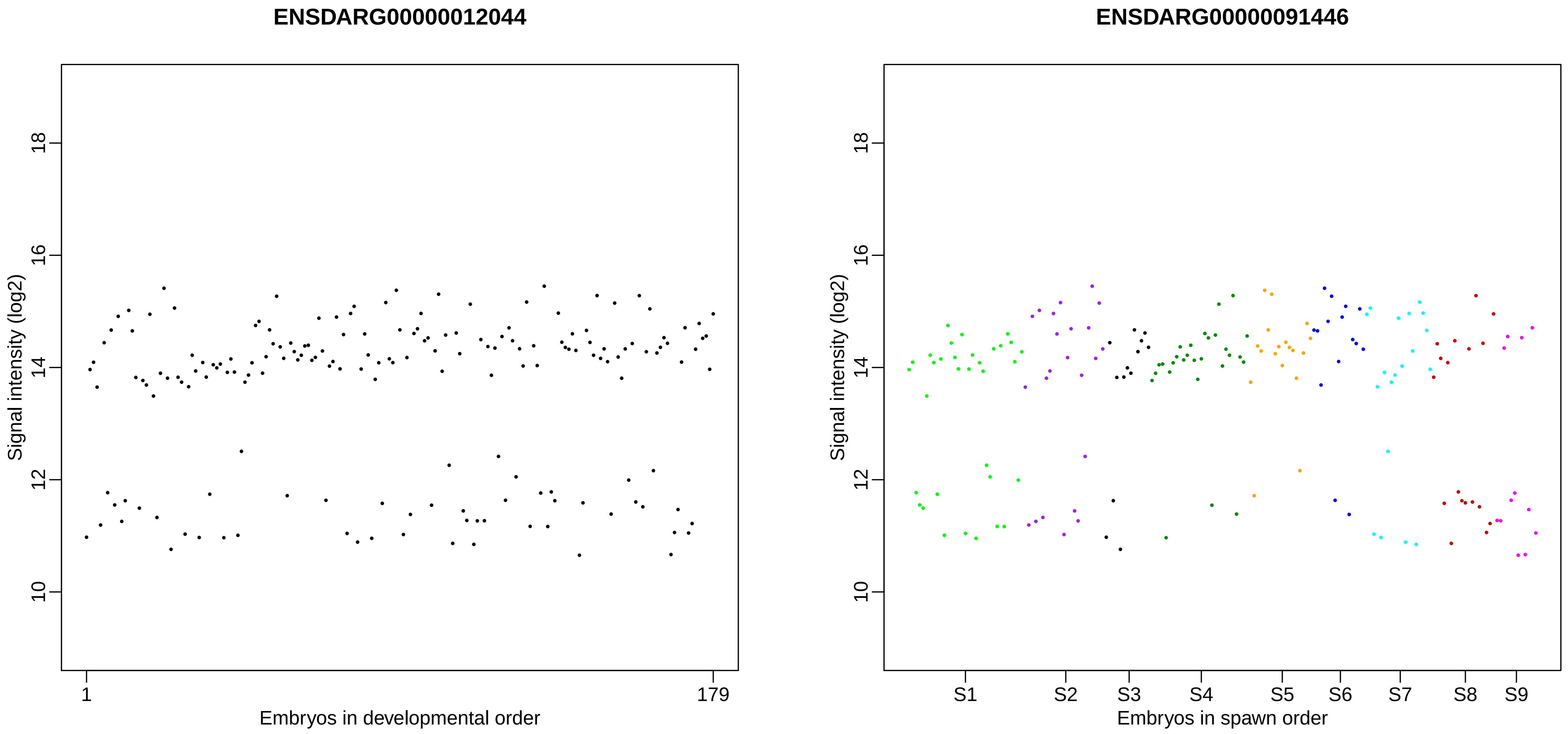
ENSDARG00000012044
Signal intensity (log2)
10
12
14
16
18
1
179
Embryos in developmental order
ENSDARG00000091446
Signal intensity (log2)
10
12
14
16
18
S1
S2
S3
S4
S5
S6
S7
S8
S9
Embryos in spawn order

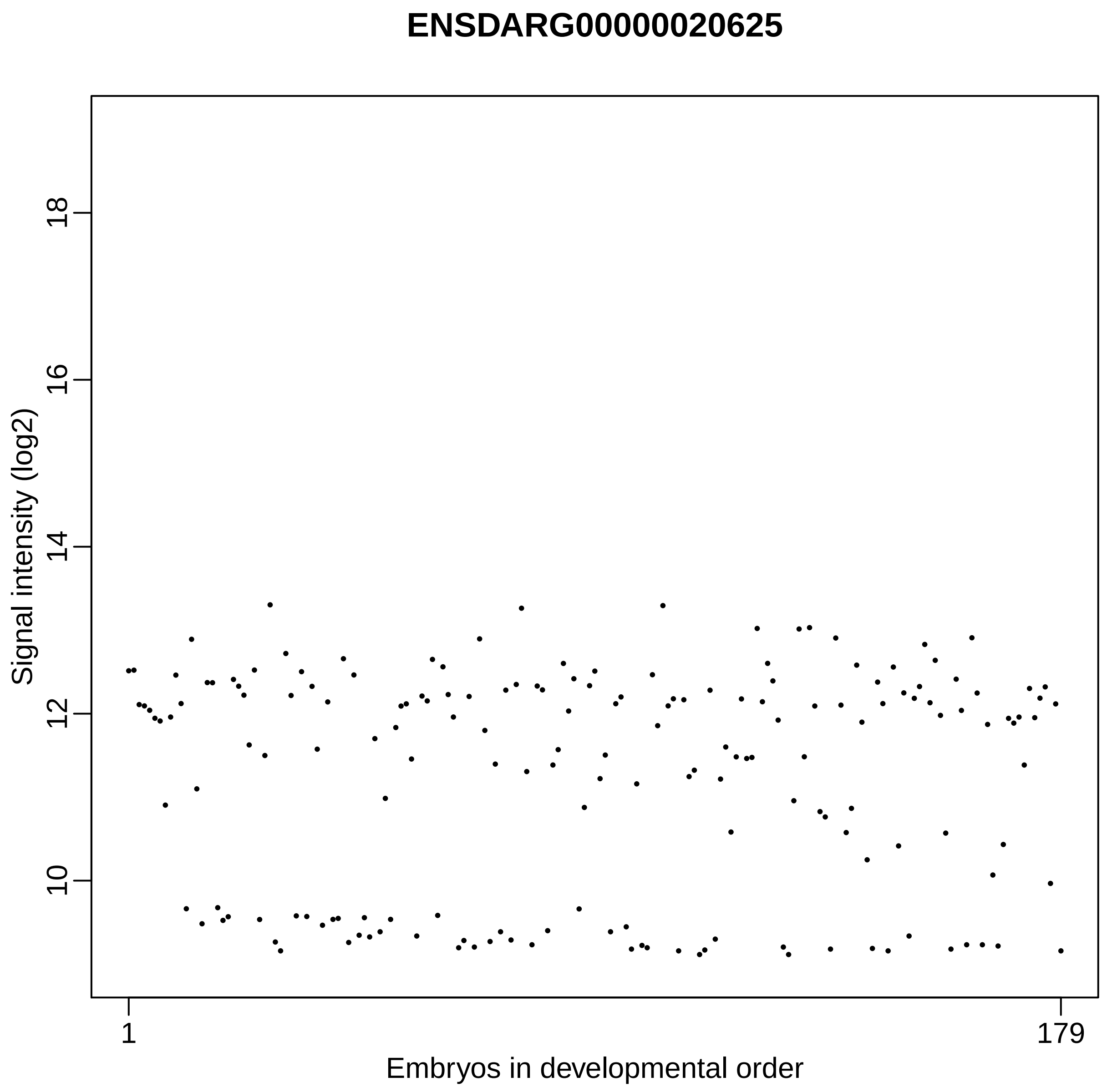

ENSDARG00000020625
Signal intensity (log2)
18
16
14
12
10
1
179
Embryos in developmental order

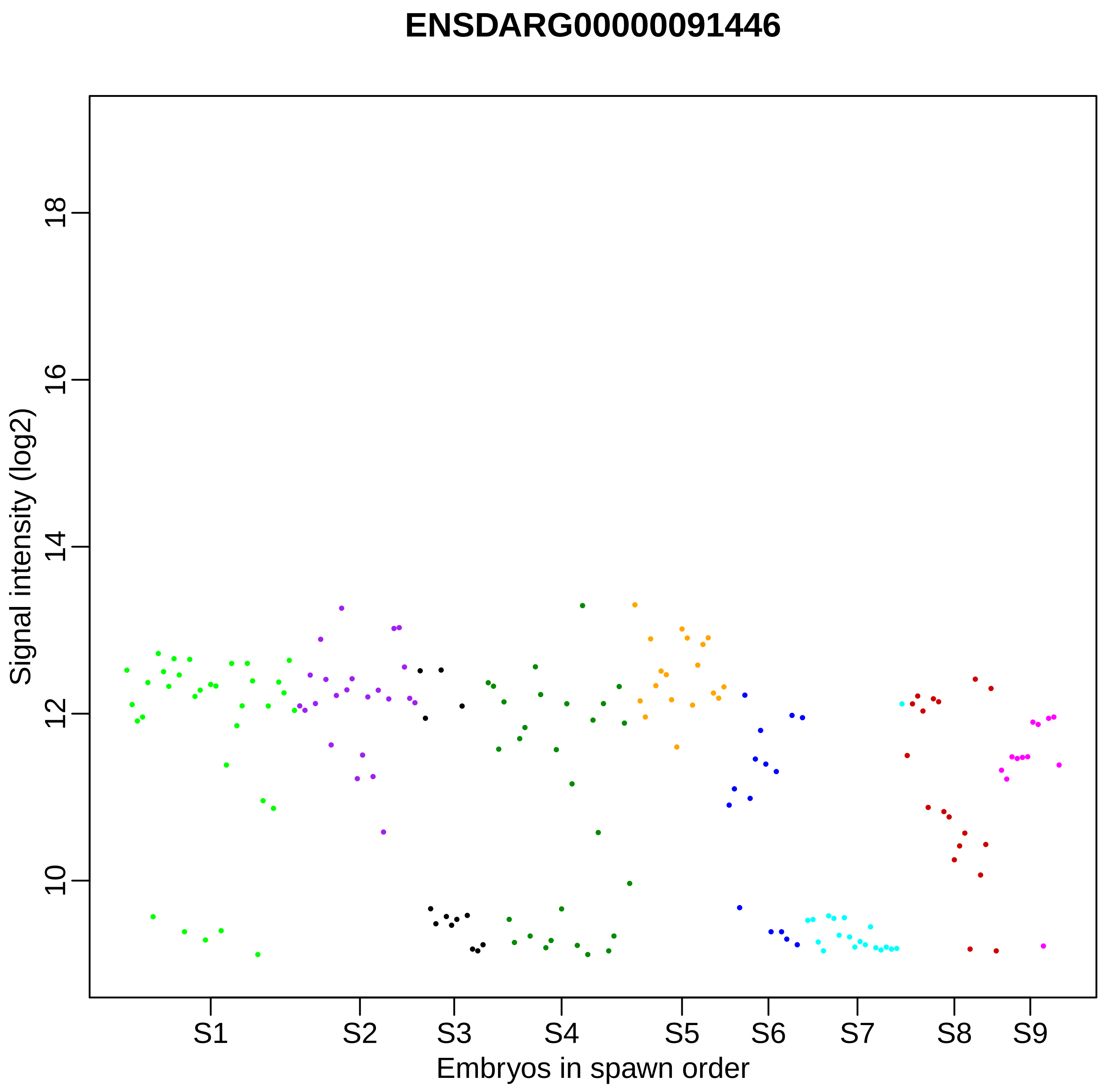

ENSDARG00000091446
Signal intensity (log2)
18
16
14
12
10
S1
S2
S3
S4
S5
S6
S7
S8
S9
Embryos in spawn order

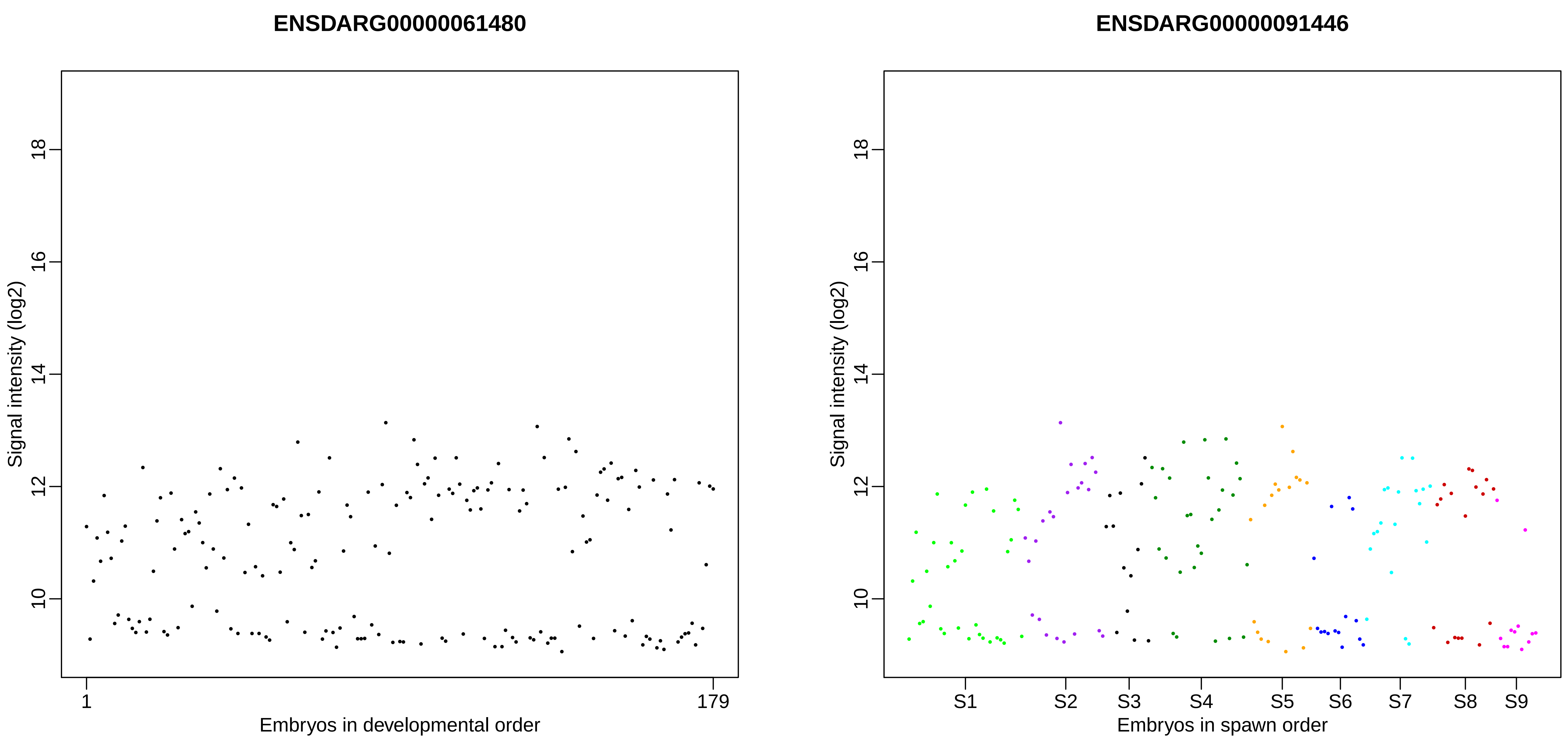
ENSDARG00000061480
Signal intensity (log2)
10
12
14
16
18
1
179
Embryos in developmental order
ENSDARG00000091446
Signal intensity (log2)
10
12
14
16
18
S1
S2
S3
S4
S5
S6
S7
S8
S9
Embryos in spawn order

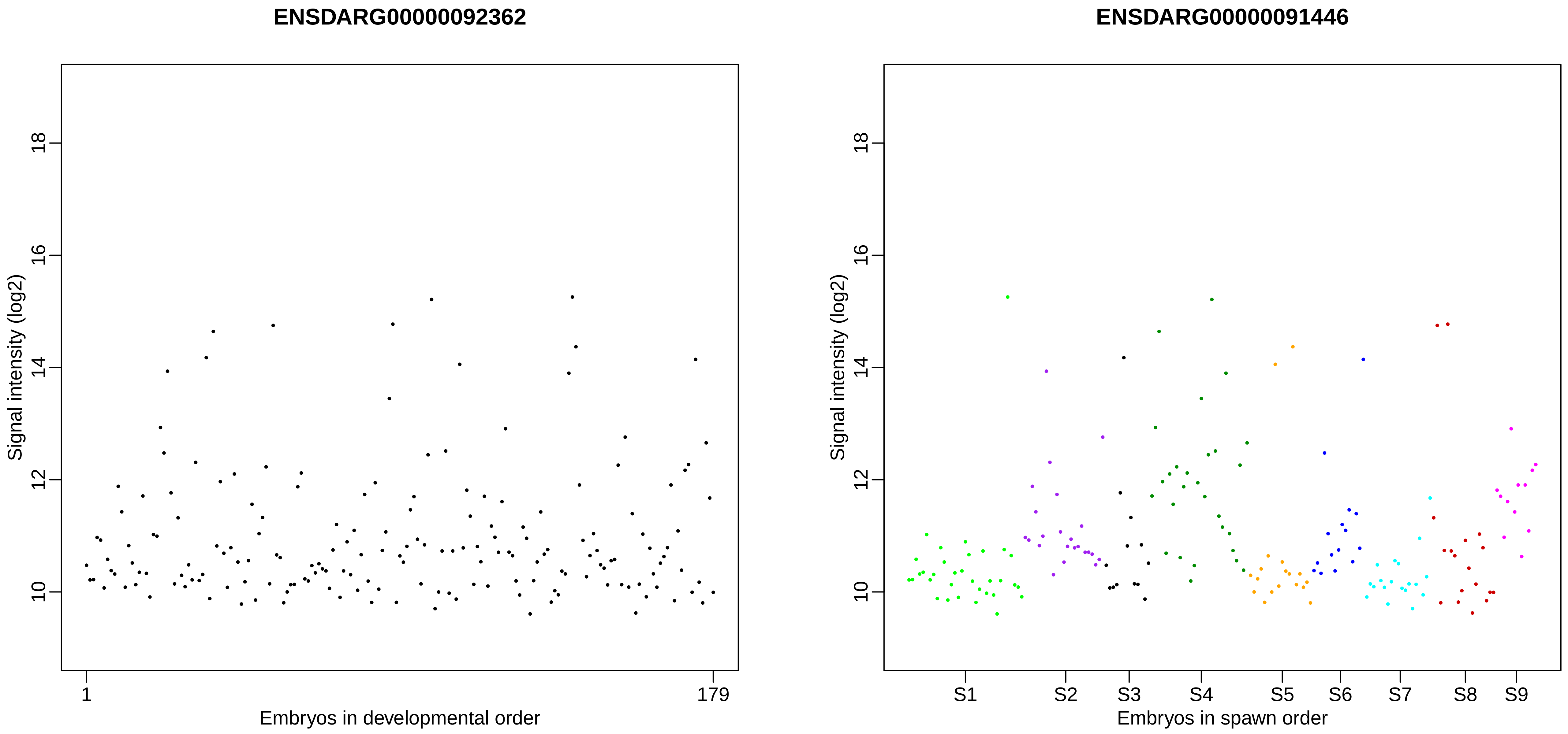
ENSDARG00000092362
Signal intensity (log2)
10
12
14
16
18
1
179
Embryos in developmental order
ENSDARG00000091446
Signal intensity (log2)
10
12
14
16
18
S1
S2
S3
S4
S5
S6
S7
S8
S9
Embryos in spawn order

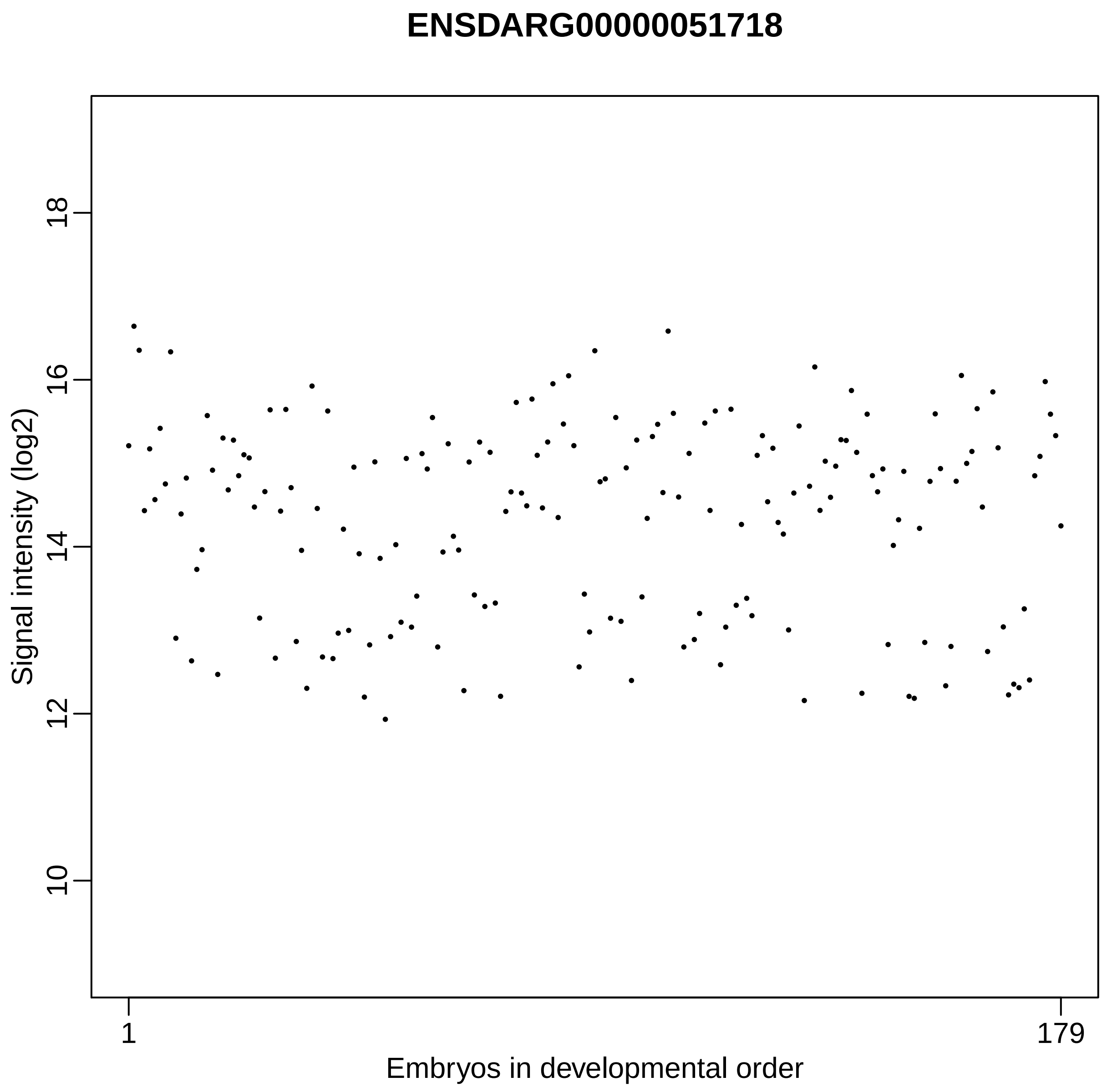
ENSDARG00000051718
Signal intensity (log2)
10
12
14
16
18
1
179
Embryos in developmental order

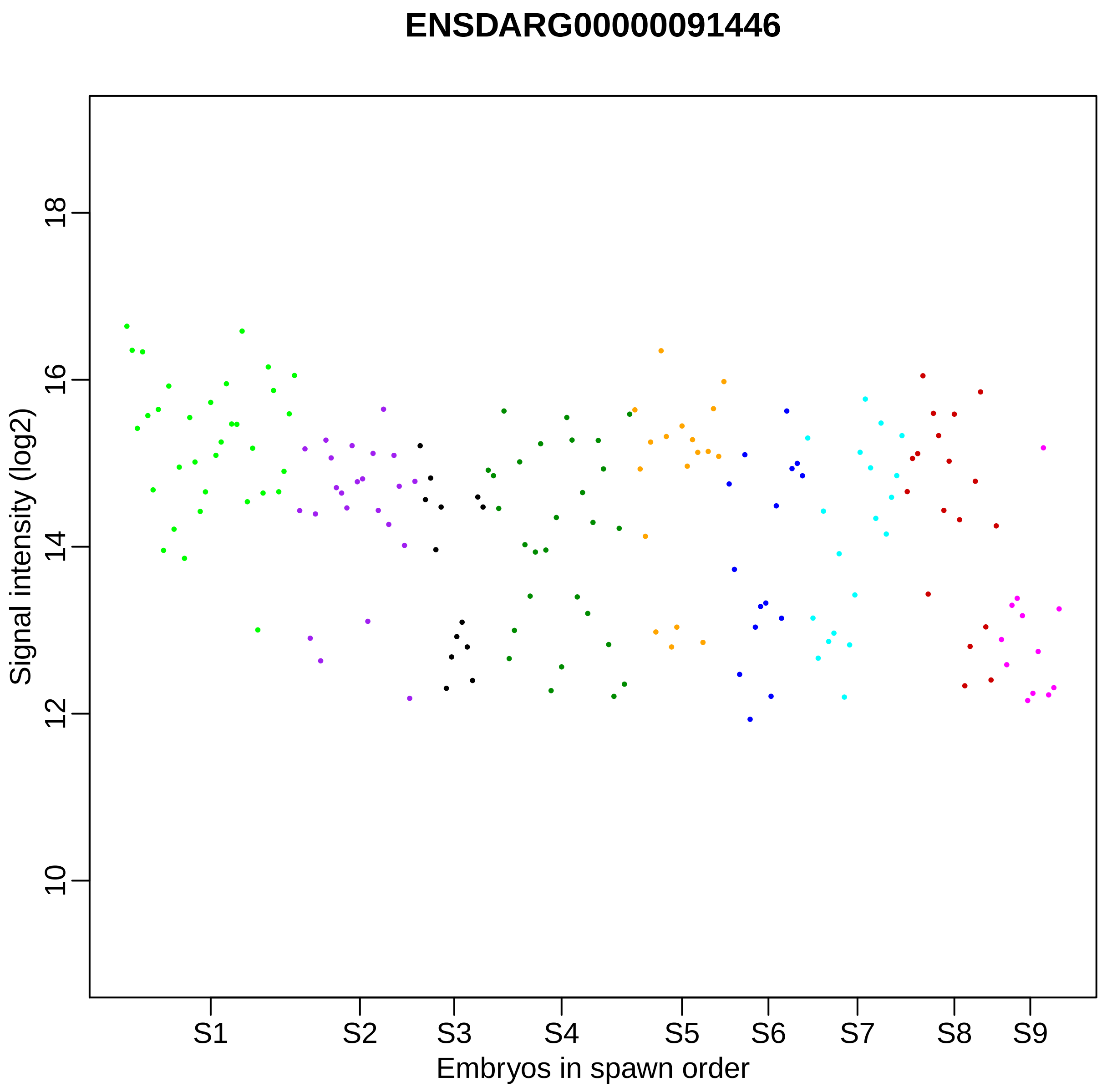
ENSDARG00000091446
Signal intensity (log2)
10
12
14
16
18
S1
S2
S3
S4
S5
S6
S7
S8
S9
Embryos in spawn order

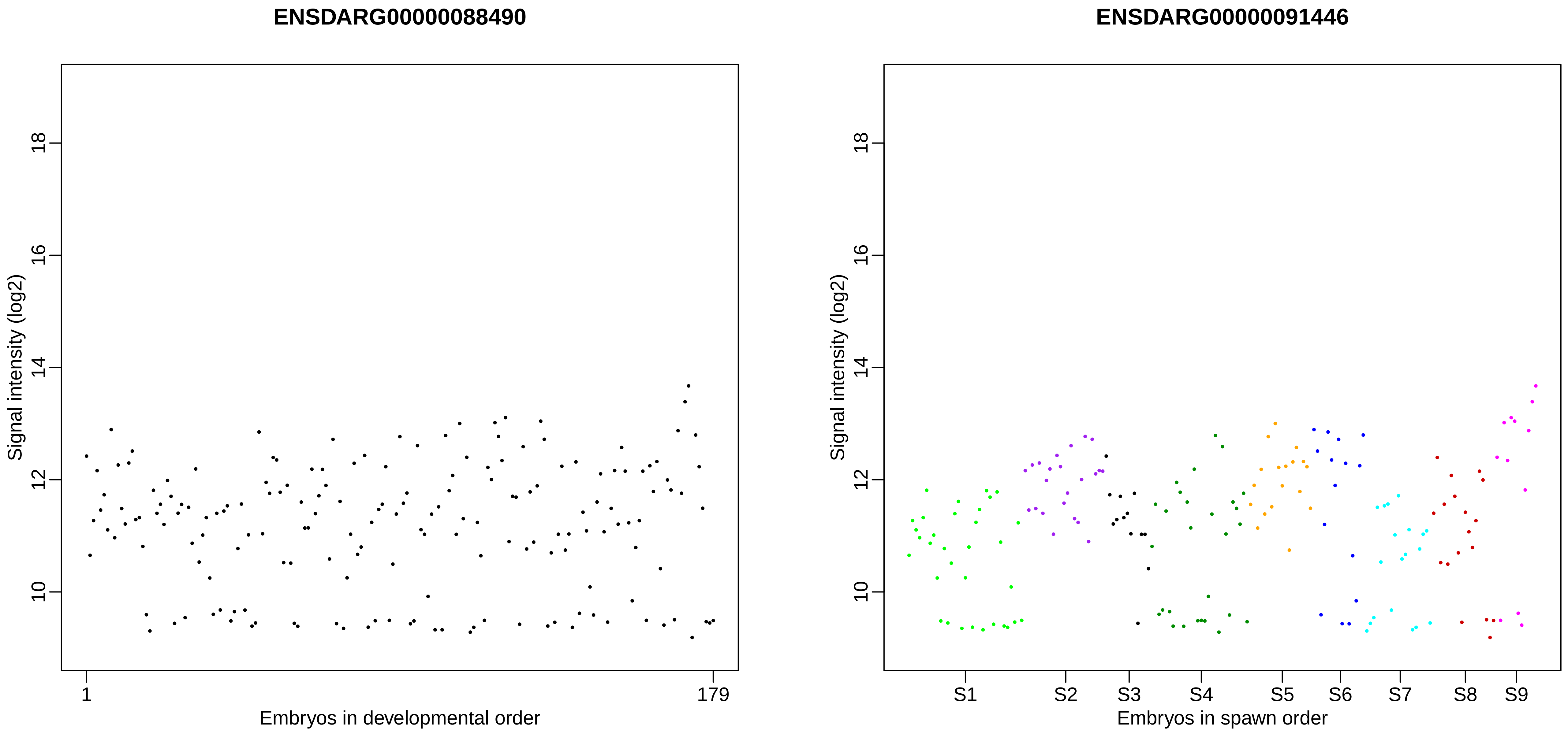

ENSDARG00000088490
Signal intensity (log2)
10
12
14
16
18
1
179
Embryos in developmental order
ENSDARG00000091446
Signal intensity (log2)
10
12
14
16
18
S1
S2
S3
S4
S5
S6
S7
S8
S9
Embryos in spawn order

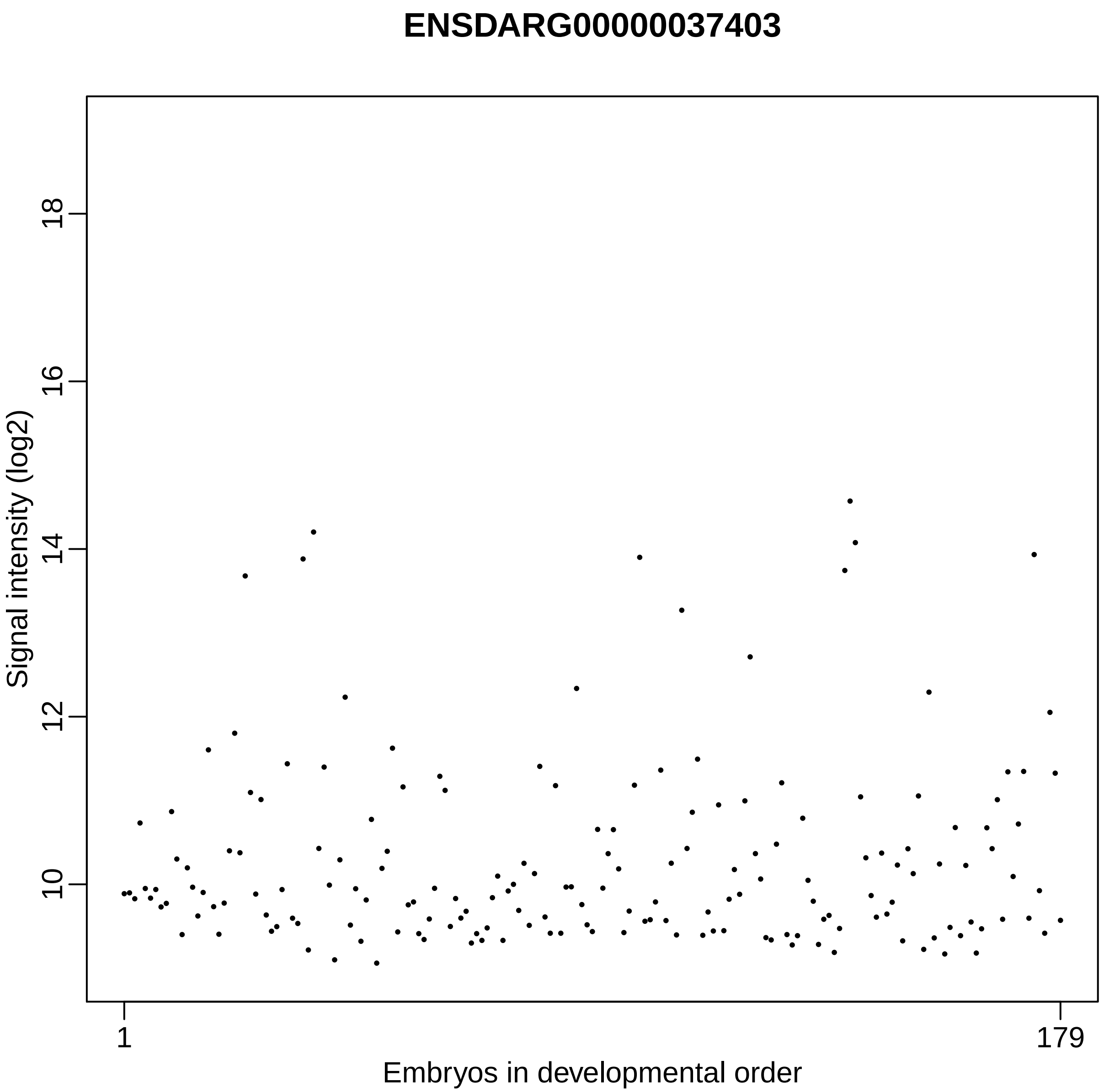
ENSDARG00000037403
Signal intensity (log2)
10
12
14
16
18
1
179
Embryos in developmental order

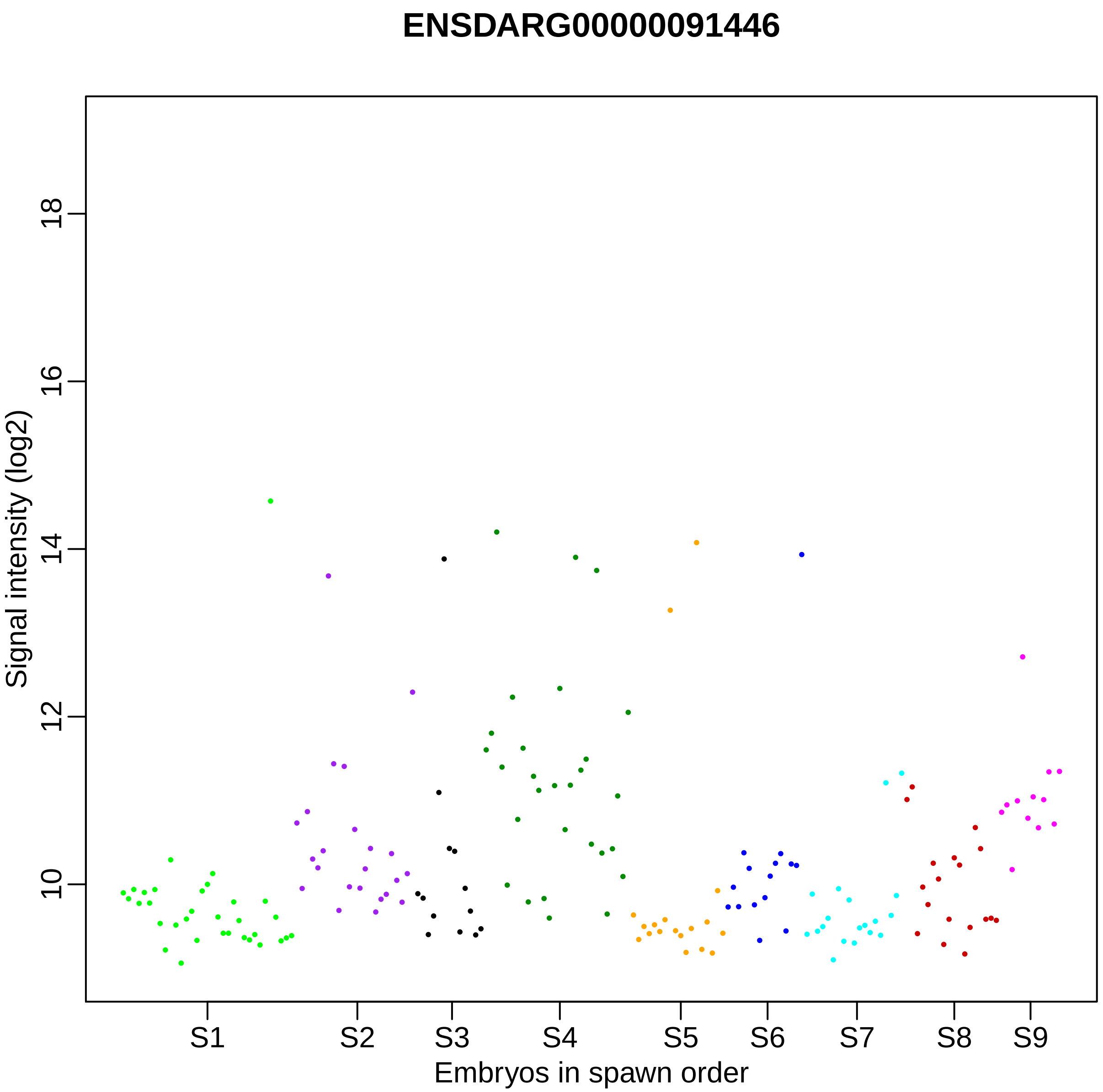
ENSDARG00000091446
Signal intensity (log2)
10
12
14
16
18
S1
S2
S3
S4
S5
S6
S7
S8
S9
Embryos in spawn order

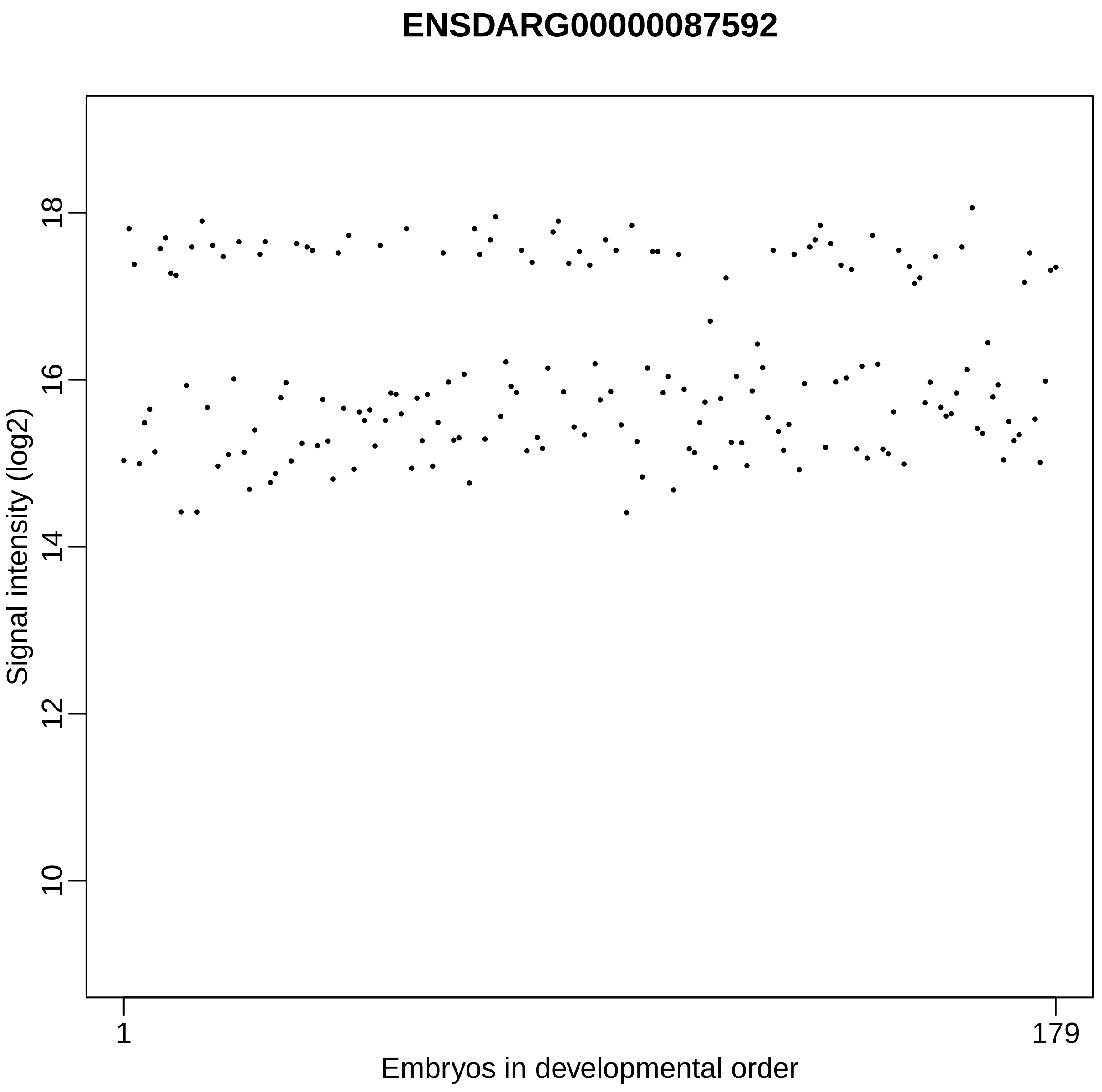

ENSDARG00000087592
Signal intensity (log2)
18
16
14
12
10
1
179
Embryos in developmental order

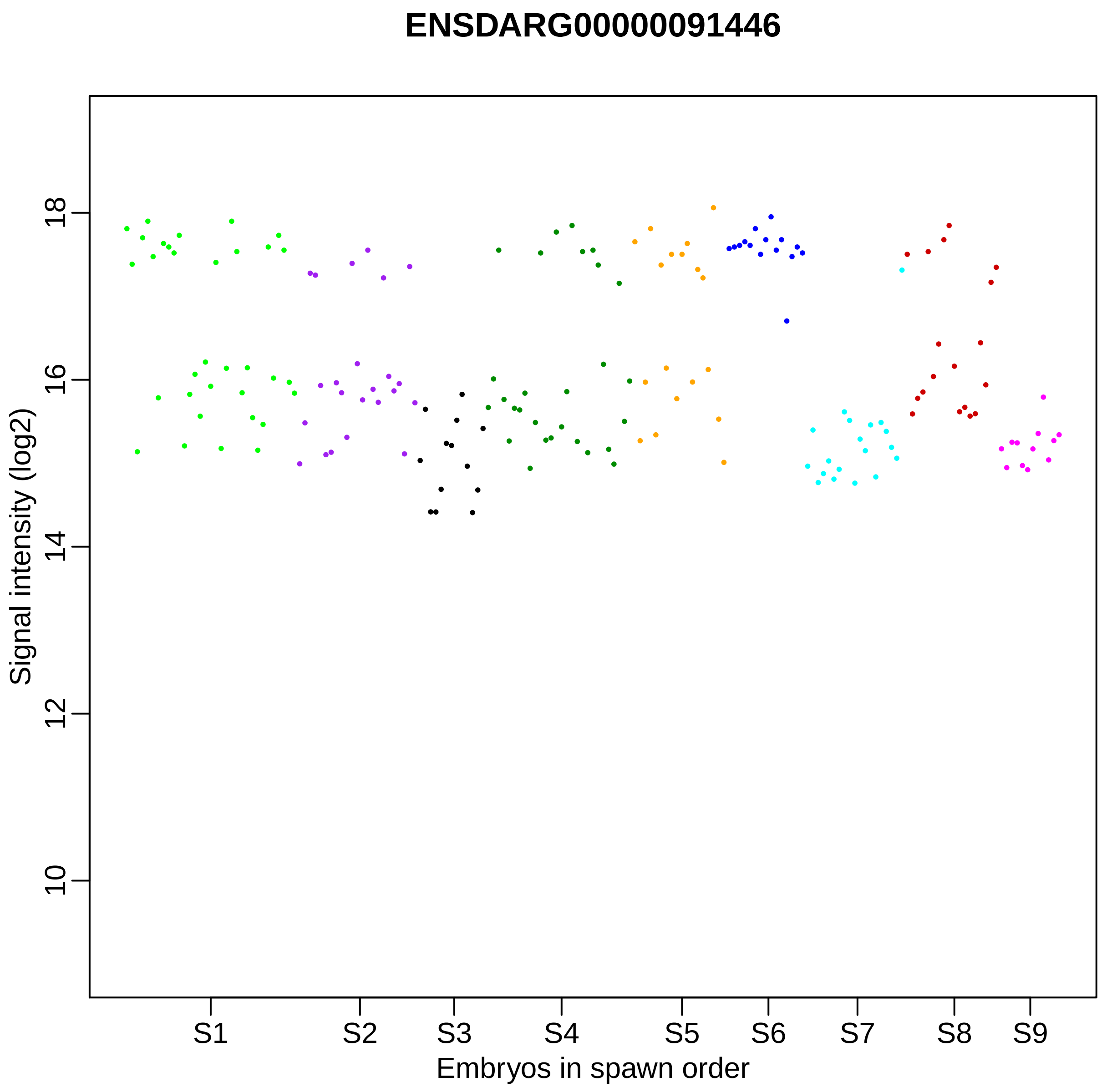

ENSDARG00000091446
Signal intensity (log2)
18
16
14
12
10
S1
S2
S3
S4
S5
S6
S7
S8
S9
Embryos in spawn order

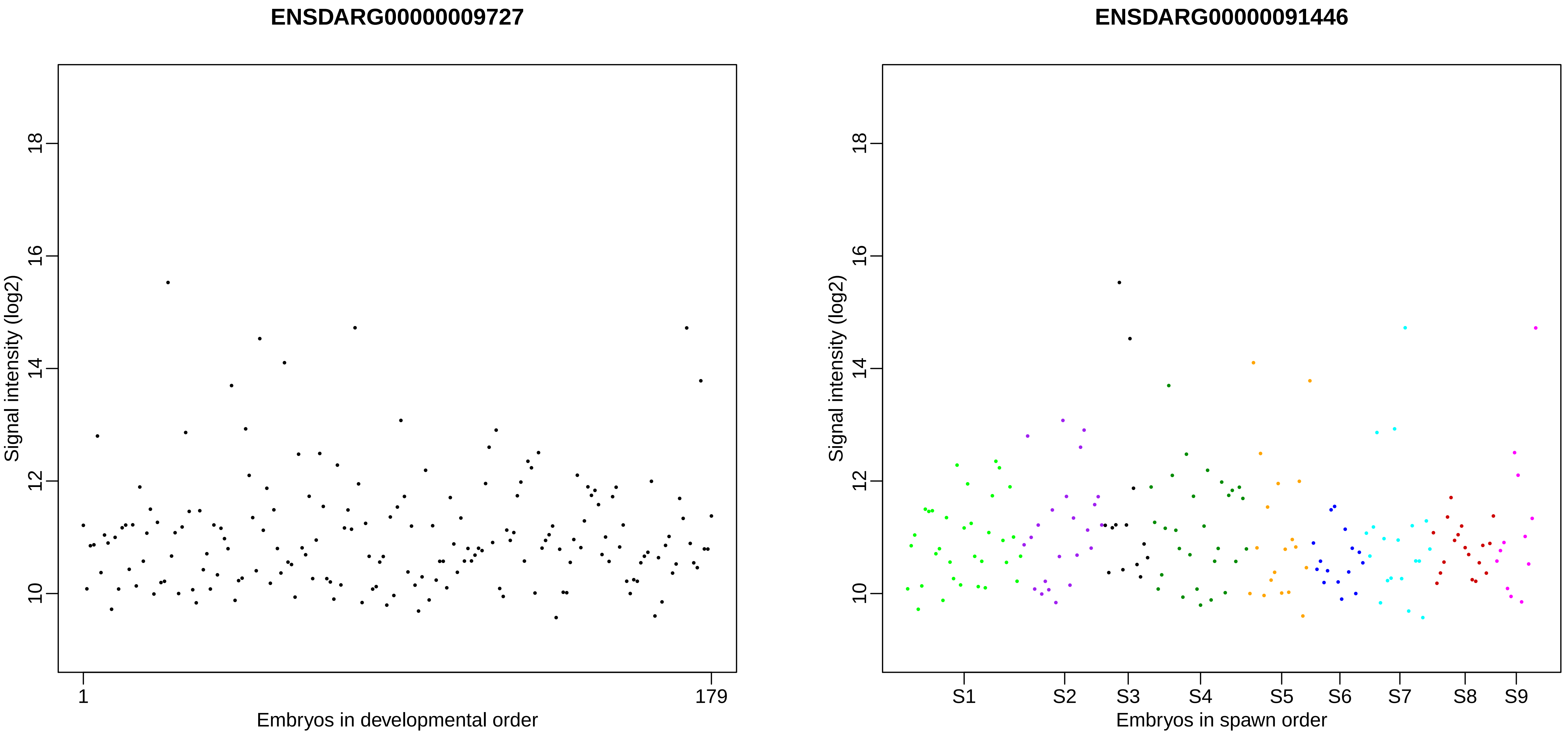

ENSDARG00000009727
Signal intensity (log2)
10
12
14
16
18
1
179
Embryos in developmental order
ENSDARG00000091446
Signal intensity (log2)
10
12
14
16
18
S1
S2
S3
S4
S5
S6
S7
S8
S9
Embryos in spawn order

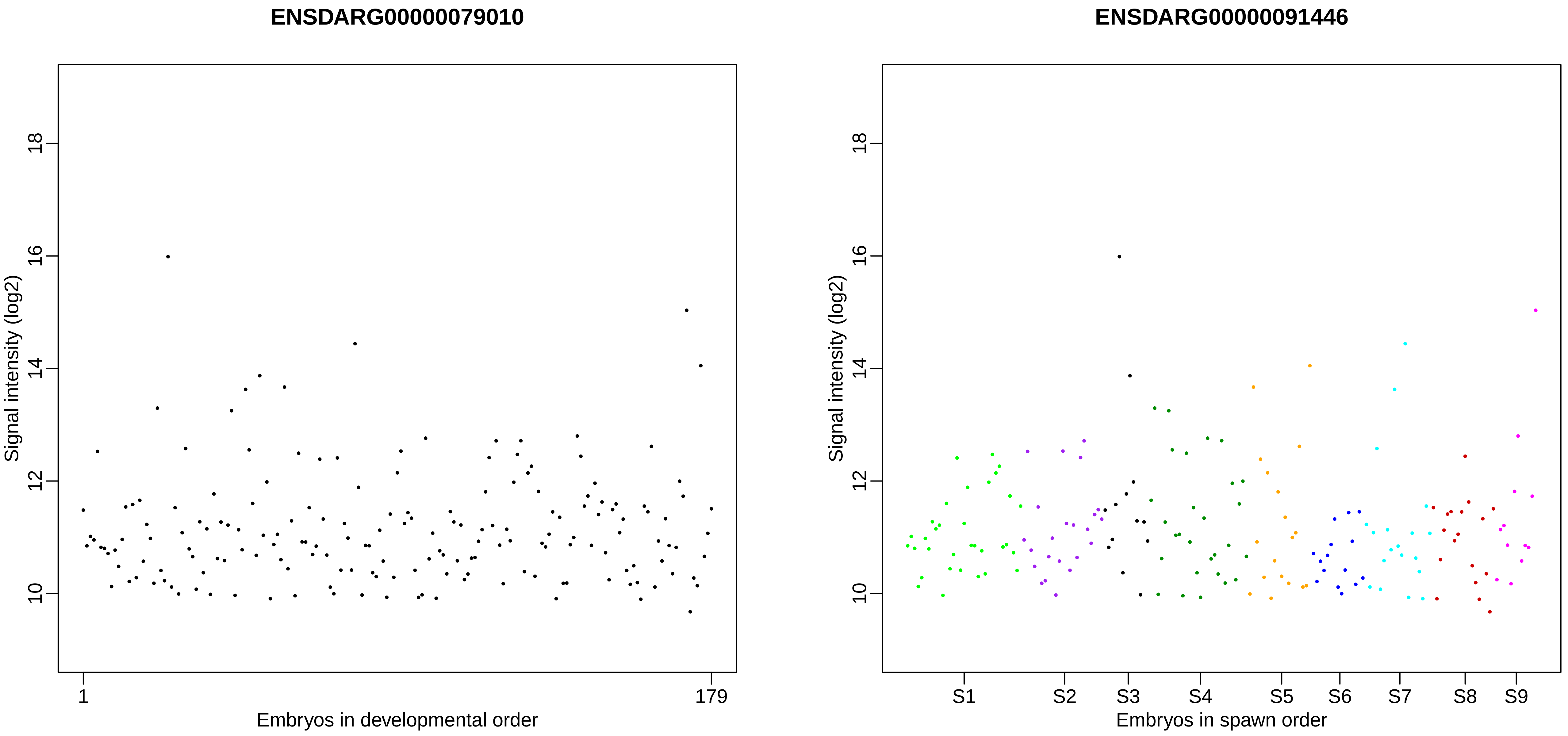
ENSDARG00000079010
Signal intensity (log2)
10
12
14
16
18
1
179
Embryos in developmental order
ENSDARG00000091446
Signal intensity (log2)
10
12
14
16
18
S1
S2
S3
S4
S5
S6
S7
S8
S9
Embryos in spawn order

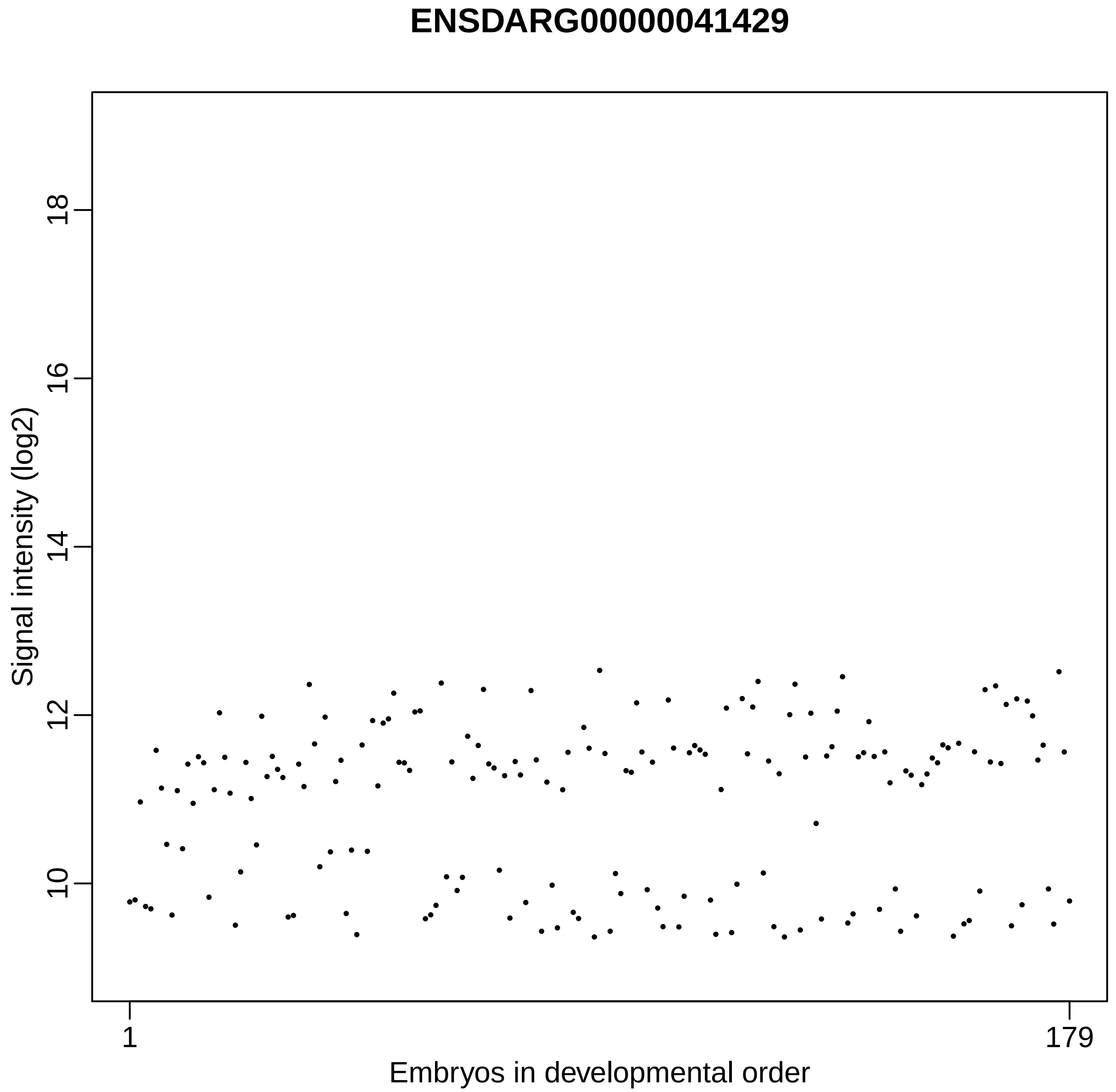

ENSDARG00000041429
Signal intensity (log2)
10
12
14
16
18
1
179
Embryos in developmental order

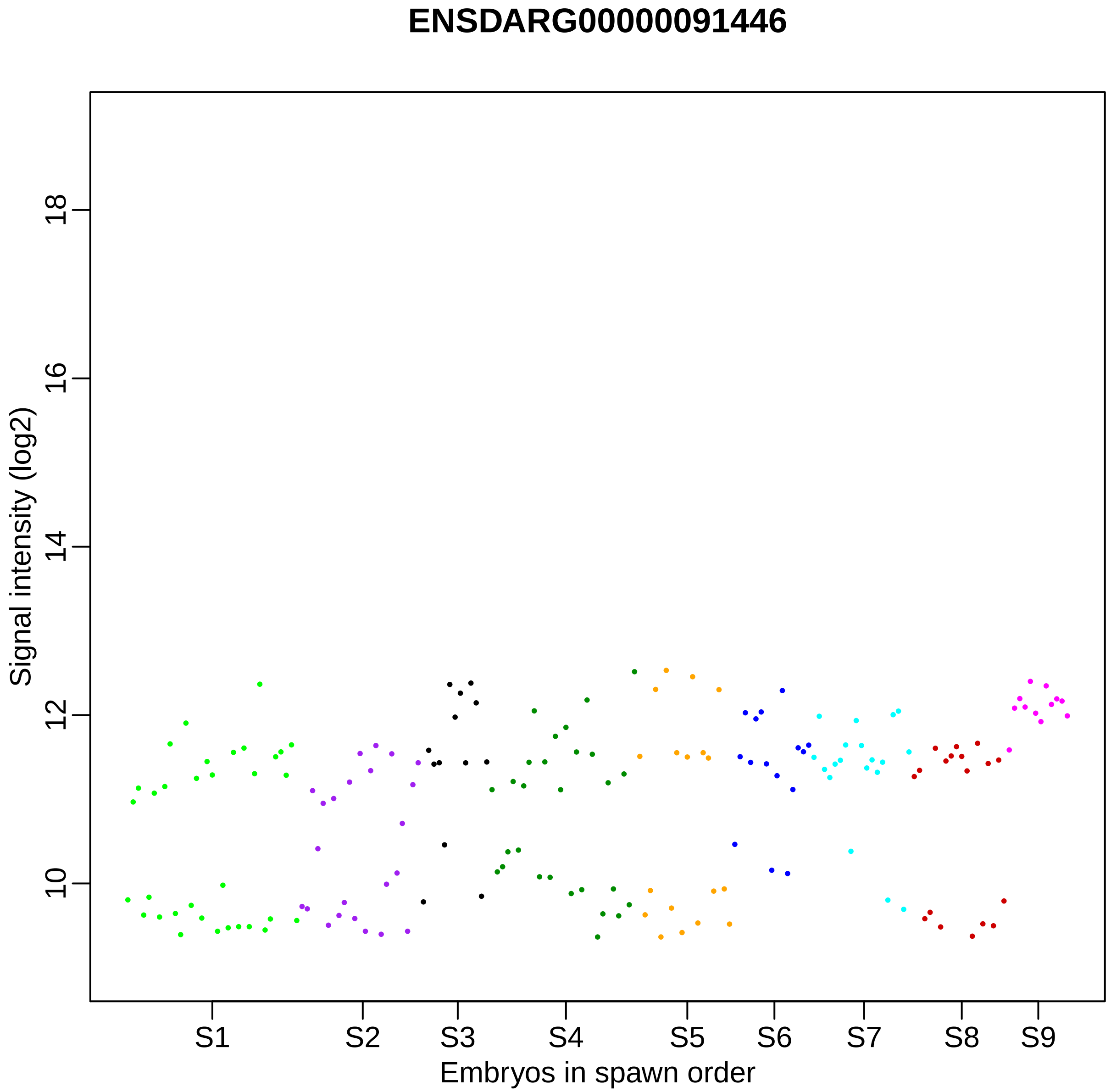

ENSDARG00000091446
Signal intensity (log2)
10
12
14
16
18
S1
S2
S3
S4
S5
S6
S7
S8
S9
Embryos in spawn order

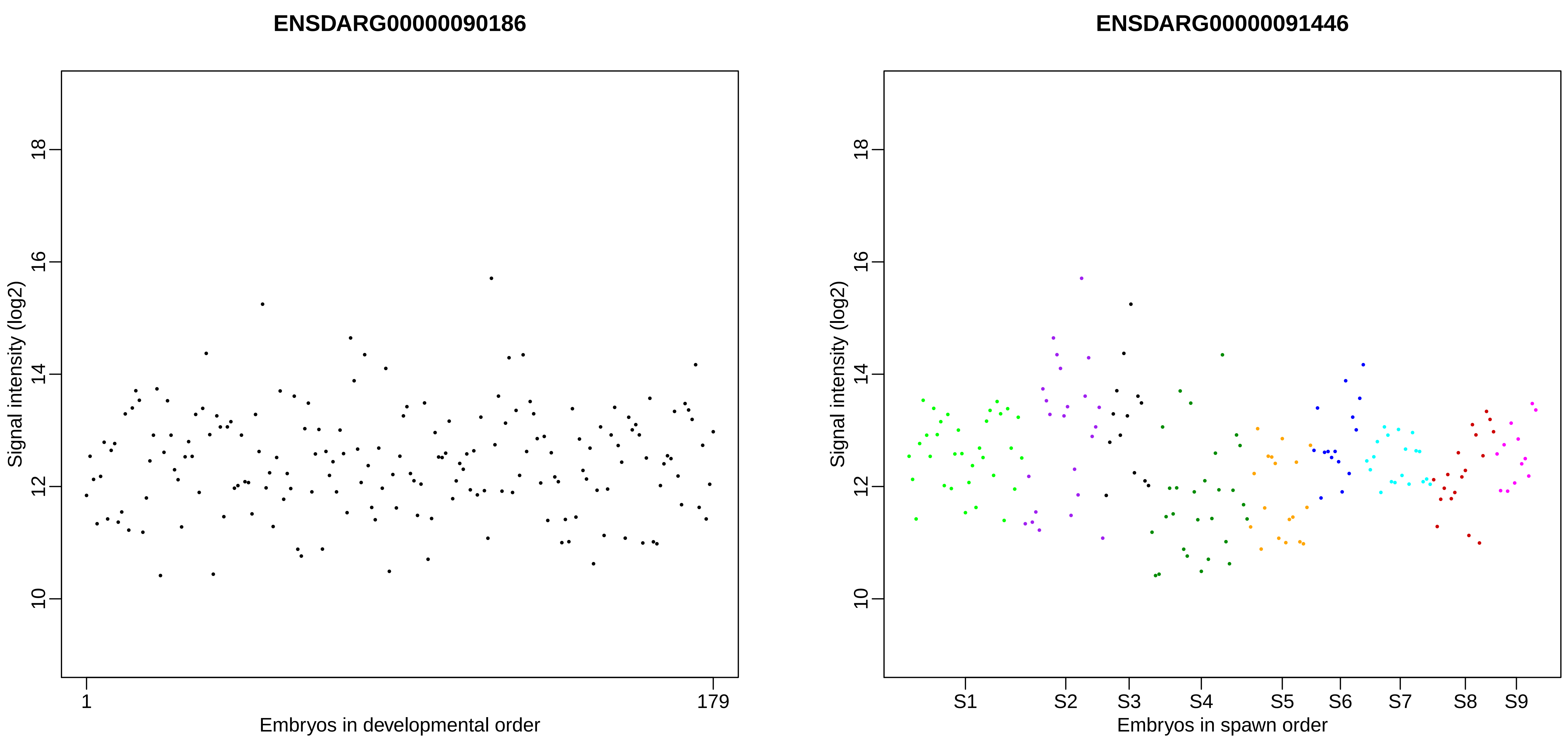
ENSDARG00000090186
ENSDARG00000091446
Signal intensity (log2)
10
12
14
16
18
1
179
Embryos in developmental order
S1
S2
S3
S4
S5
S6
S7
S8
S9
Embryos in spawn order

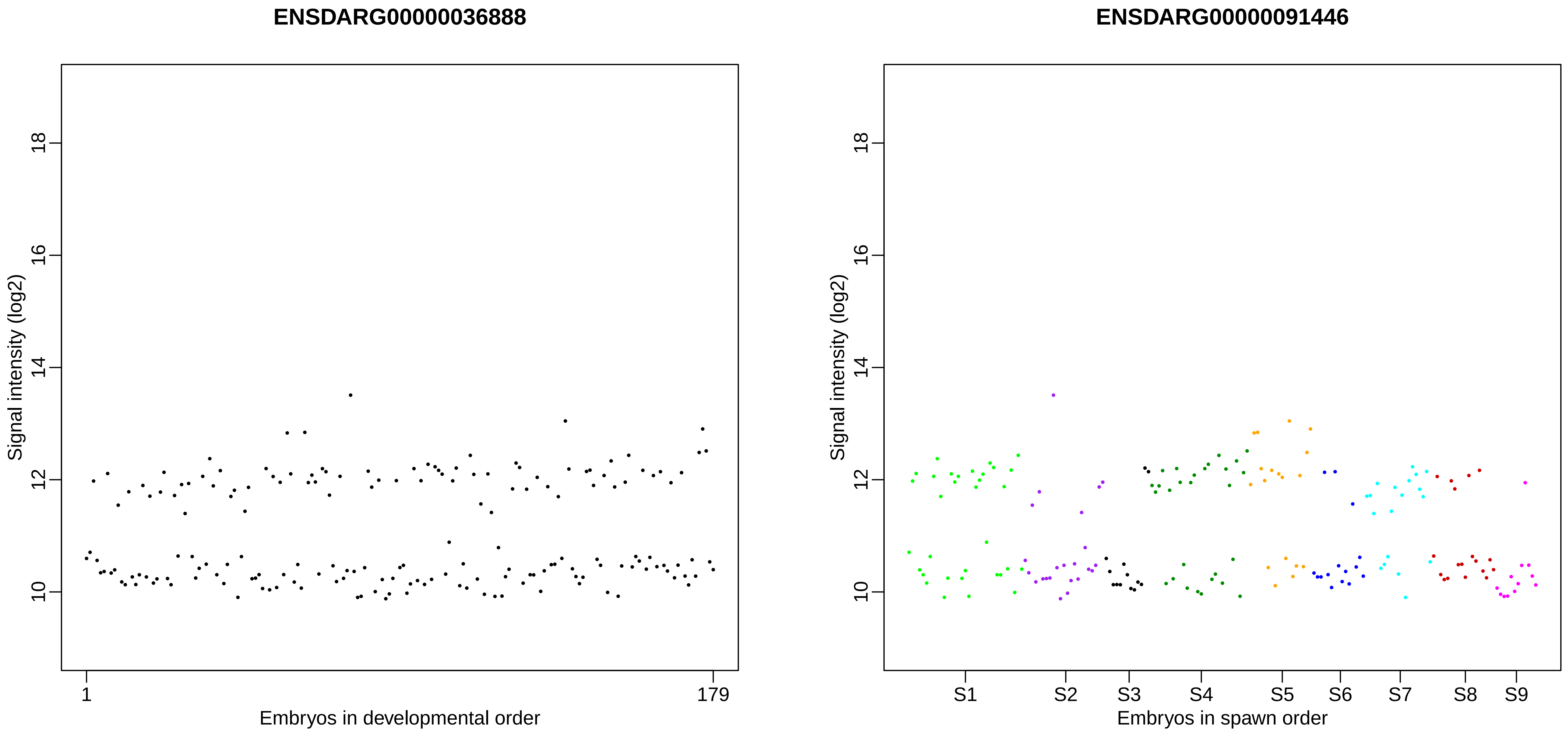

ENSDARG00000036888
Signal intensity (log2)
10
12
14
16
18
1
179
Embryos in developmental order
ENSDARG00000091446
Signal intensity (log2)
10
12
14
16
18
S1
S2
S3
S4
S5
S6
S7
S8
S9
Embryos in spawn order

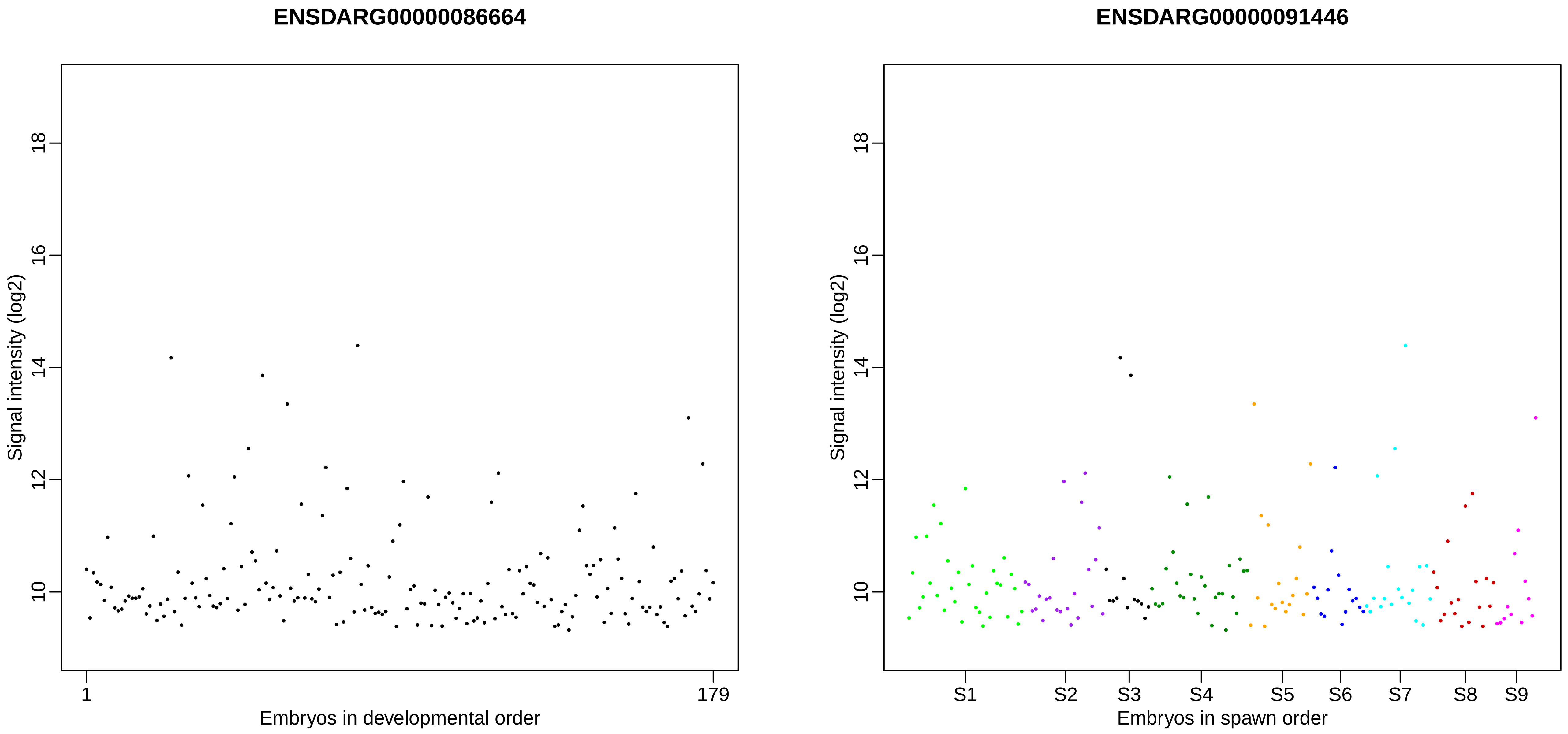
ENSDARG00000086664
Signal intensity (log2)
10
12
14
16
18
1
179
Embryos in developmental order
ENSDARG00000091446
Signal intensity (log2)
10
12
14
16
18
S1
S2
S3
S4
S5
S6
S7
S8
S9
Embryos in spawn order

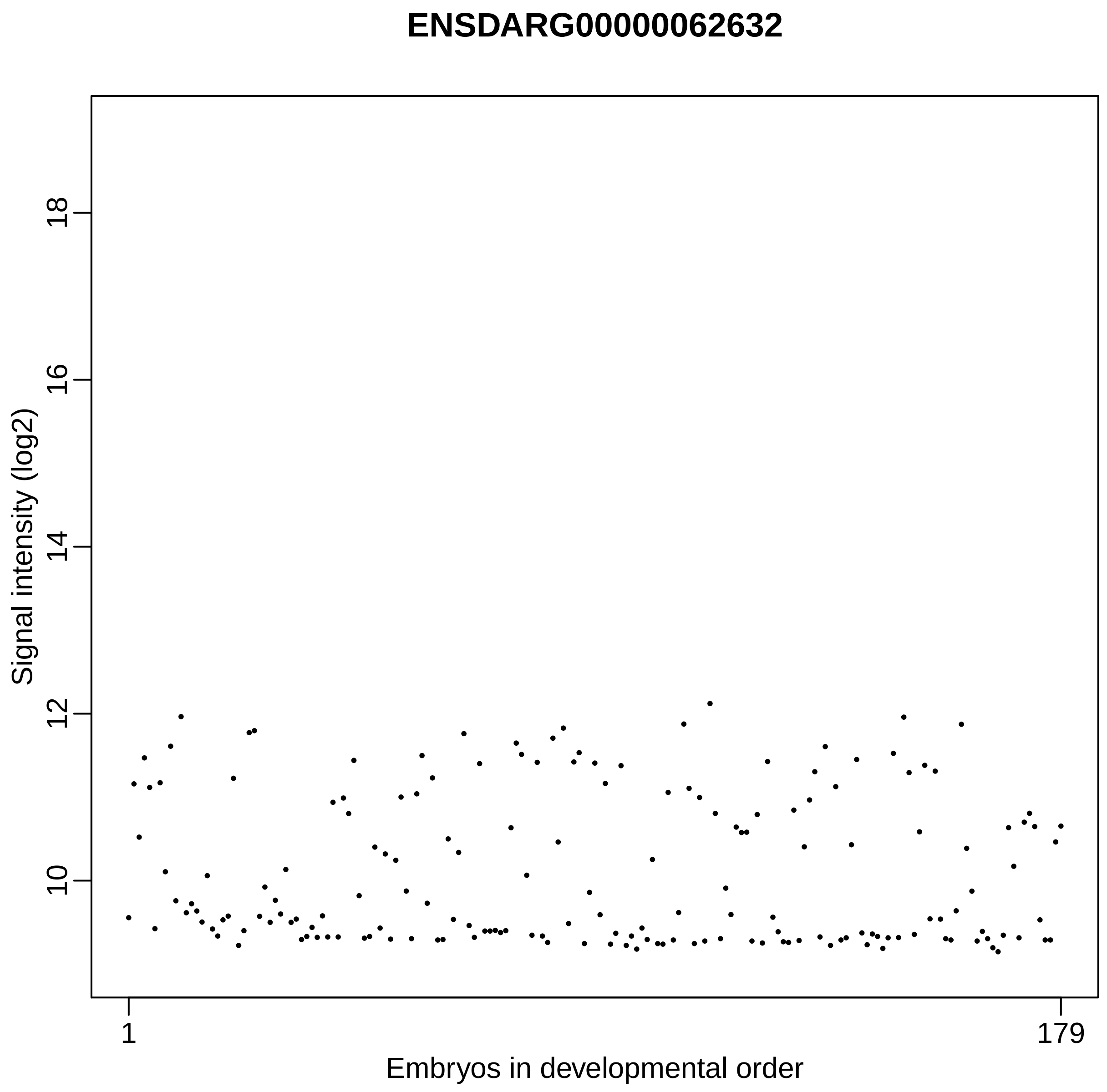
ENSDARG00000062632
Signal intensity (log2)
10
12
14
16
18
1
179
Embryos in developmental order

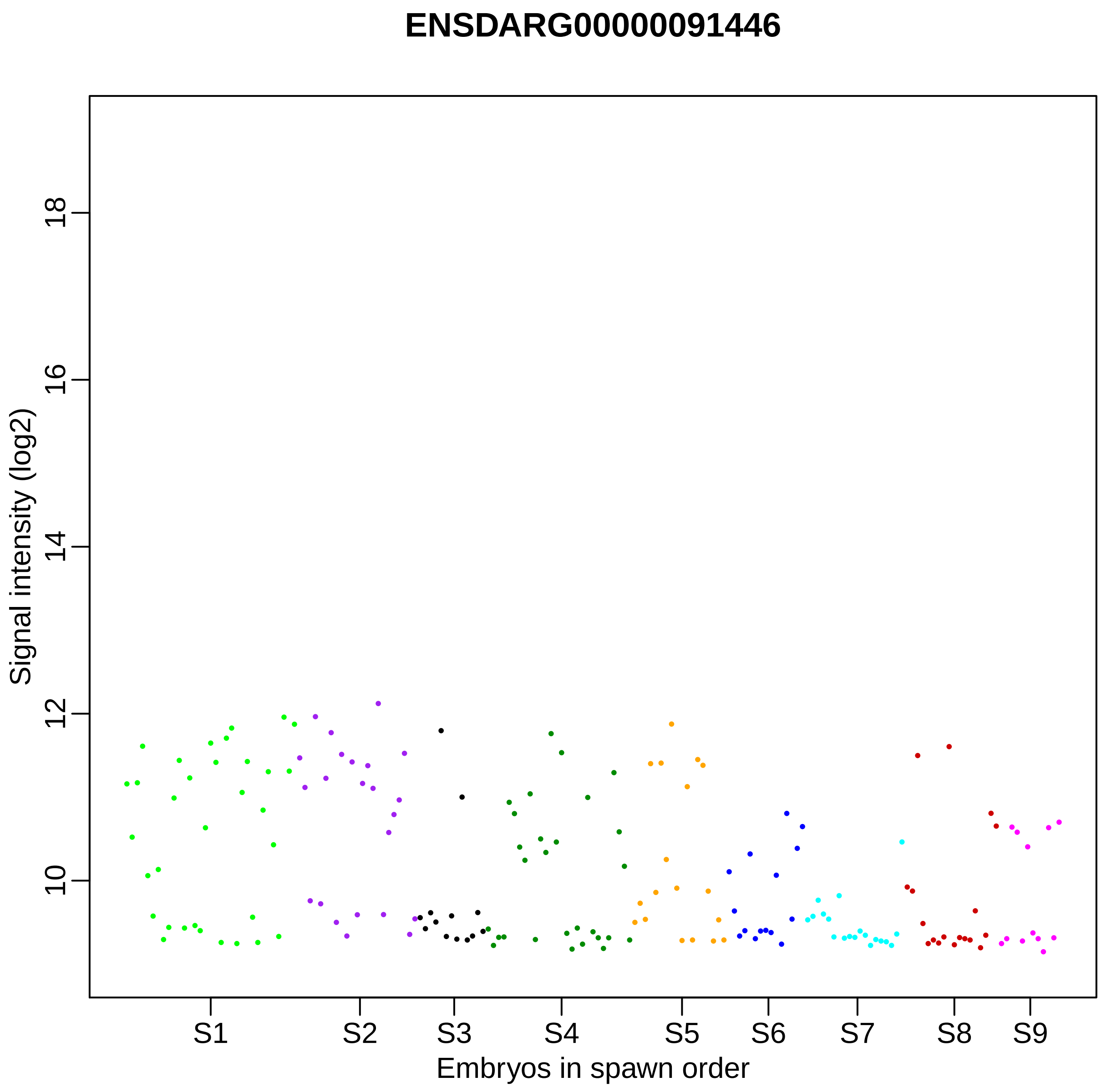
ENSDARG00000091446
Signal intensity (log2)
10
12
14
16
18
S1
S2
S3
S4
S5
S6
S7
S8
S9
Embryos in spawn order

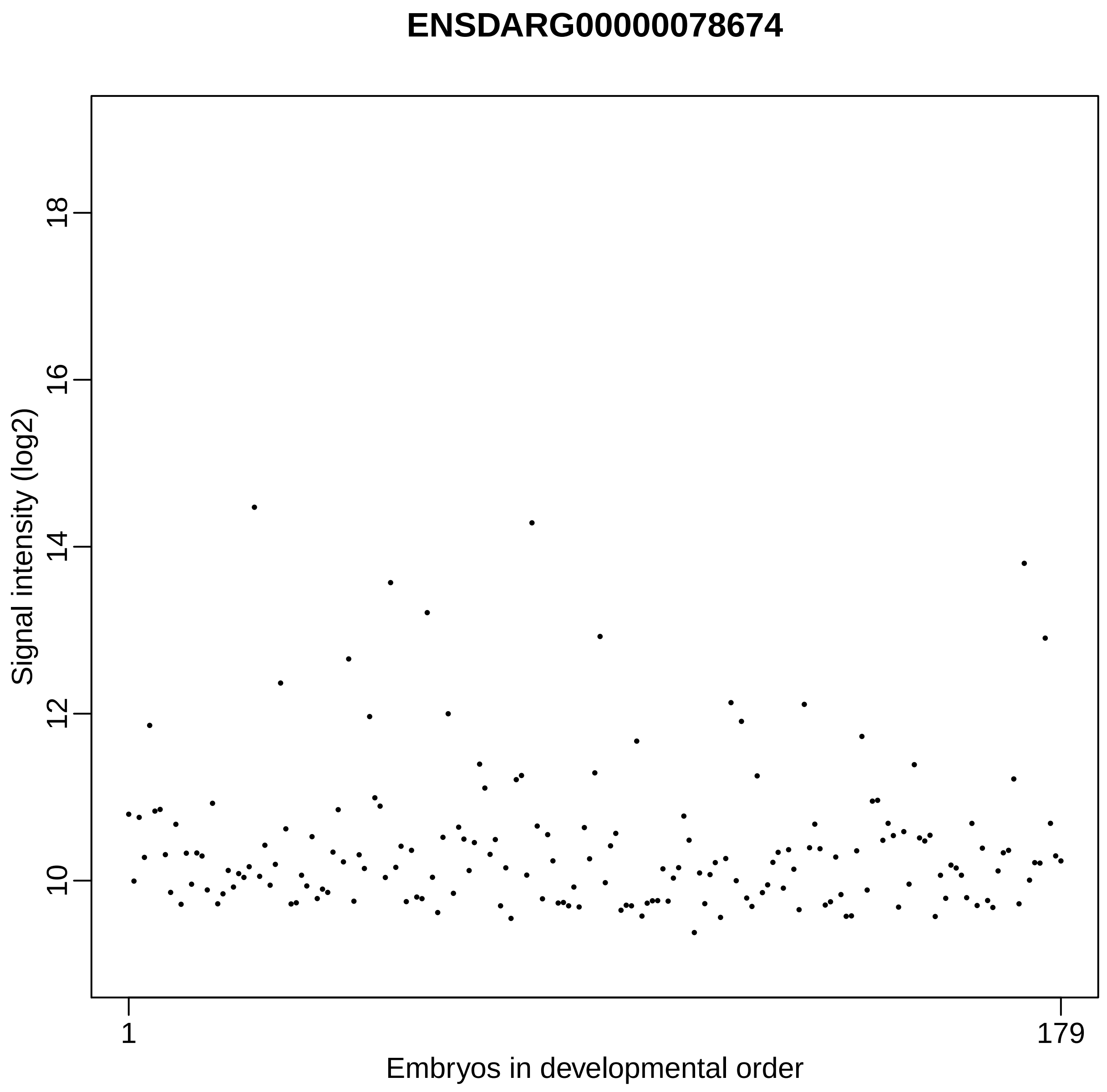
ENSDARG00000078674
Signal intensity (log2)
10
12
14
16
18
1
179
Embryos in developmental order

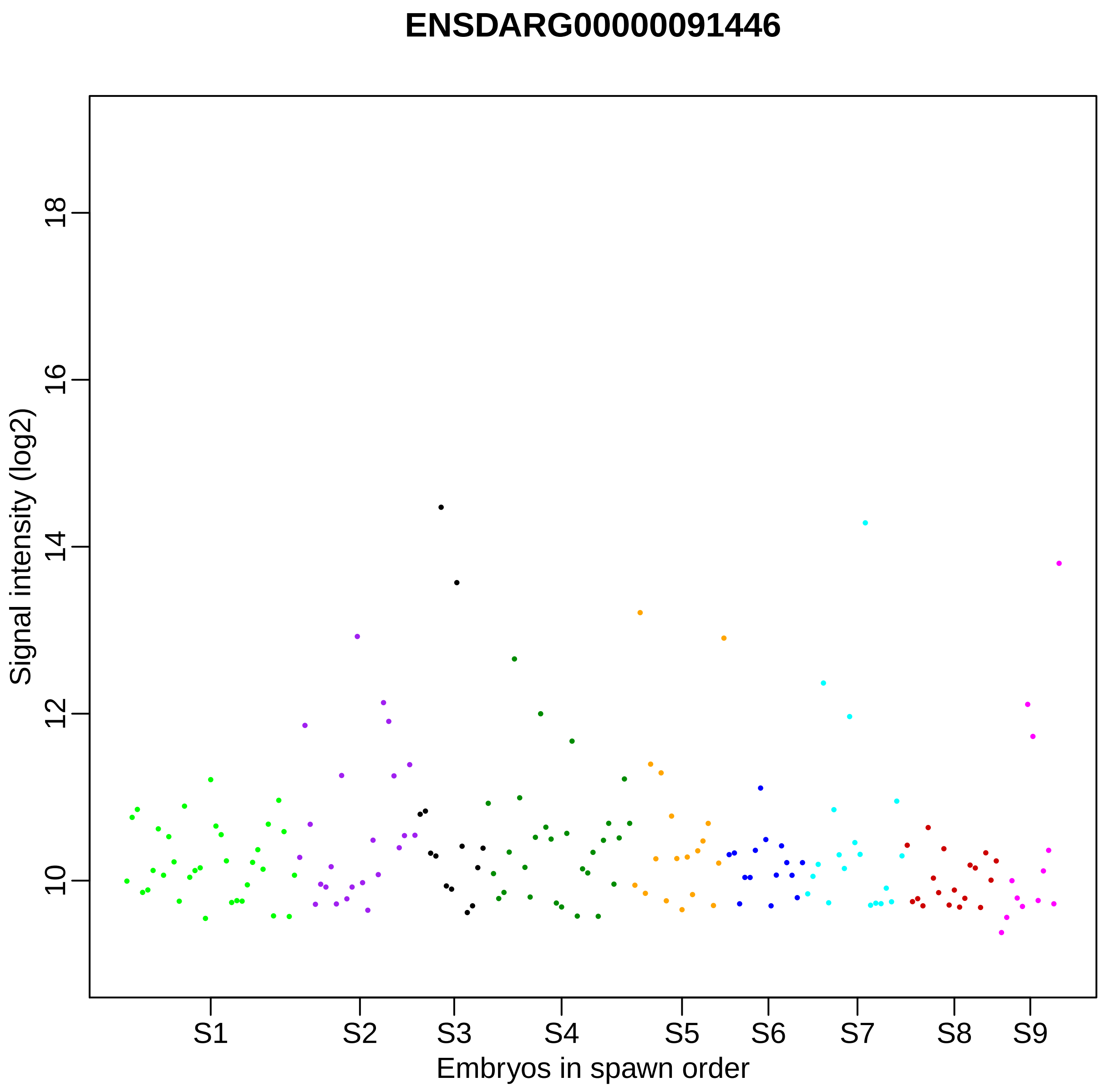
ENSDARG00000091446
Signal intensity (log2)
10
12
14
16
18
S1
S2
S3
S4
S5
S6
S7
S8
S9
Embryos in spawn order

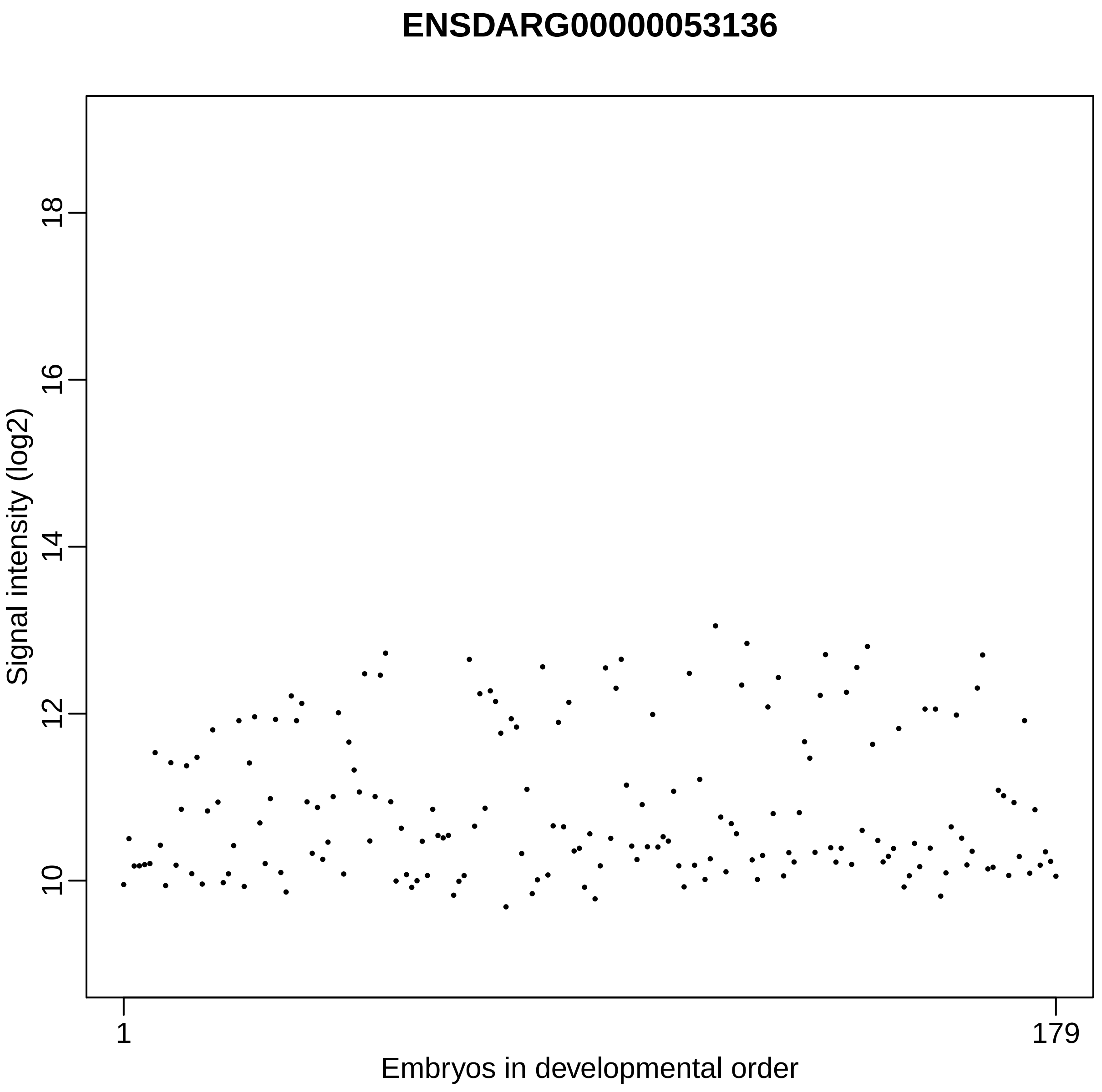

ENSDARG00000053136
Signal intensity (log2)
10
12
14
16
18
1
179
Embryos in developmental order

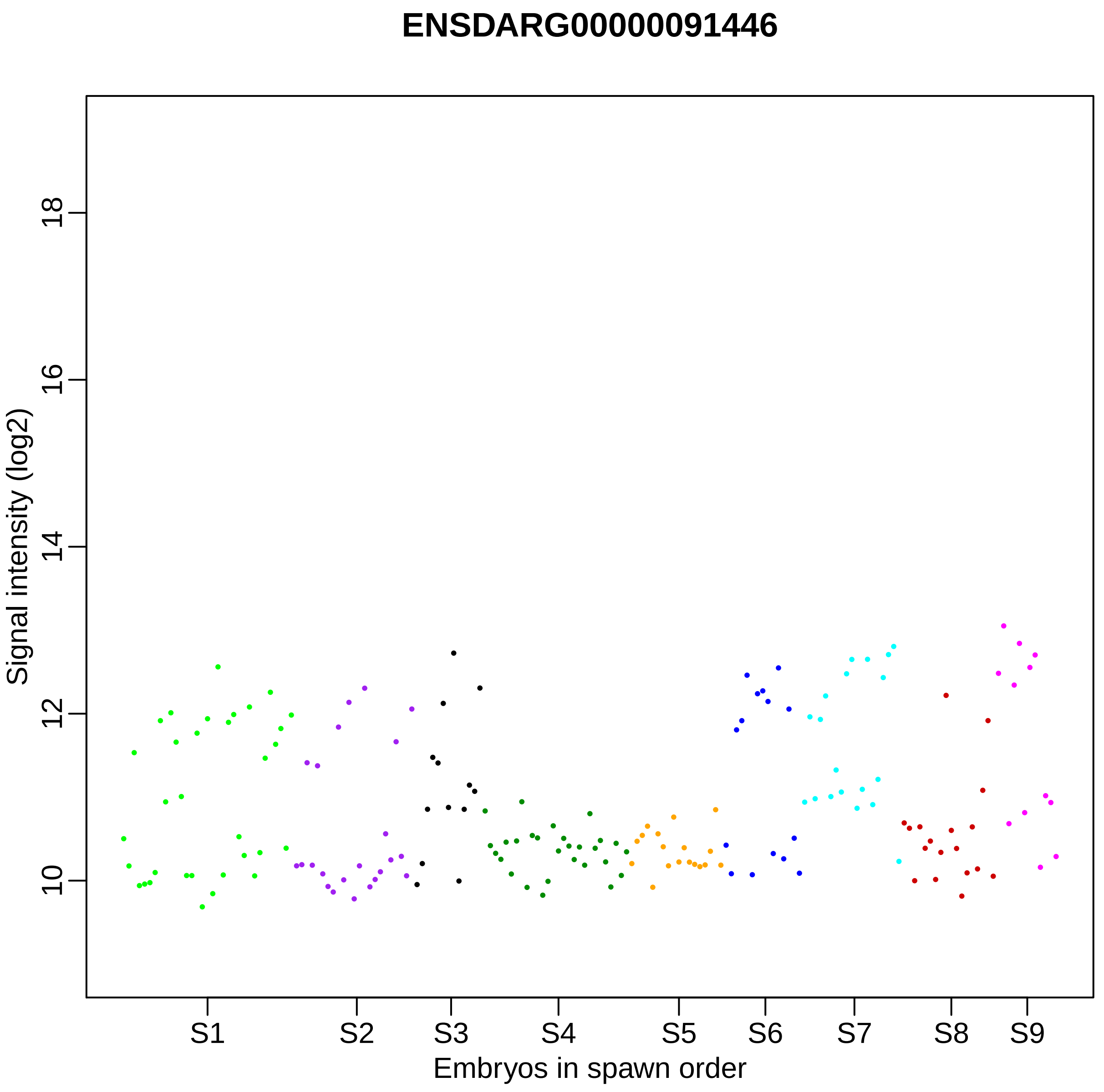

ENSDARG00000091446
Signal intensity (log2)
10
12
14
16
18
S1
S2
S3
S4
S5
S6
S7
S8
S9
Embryos in spawn order

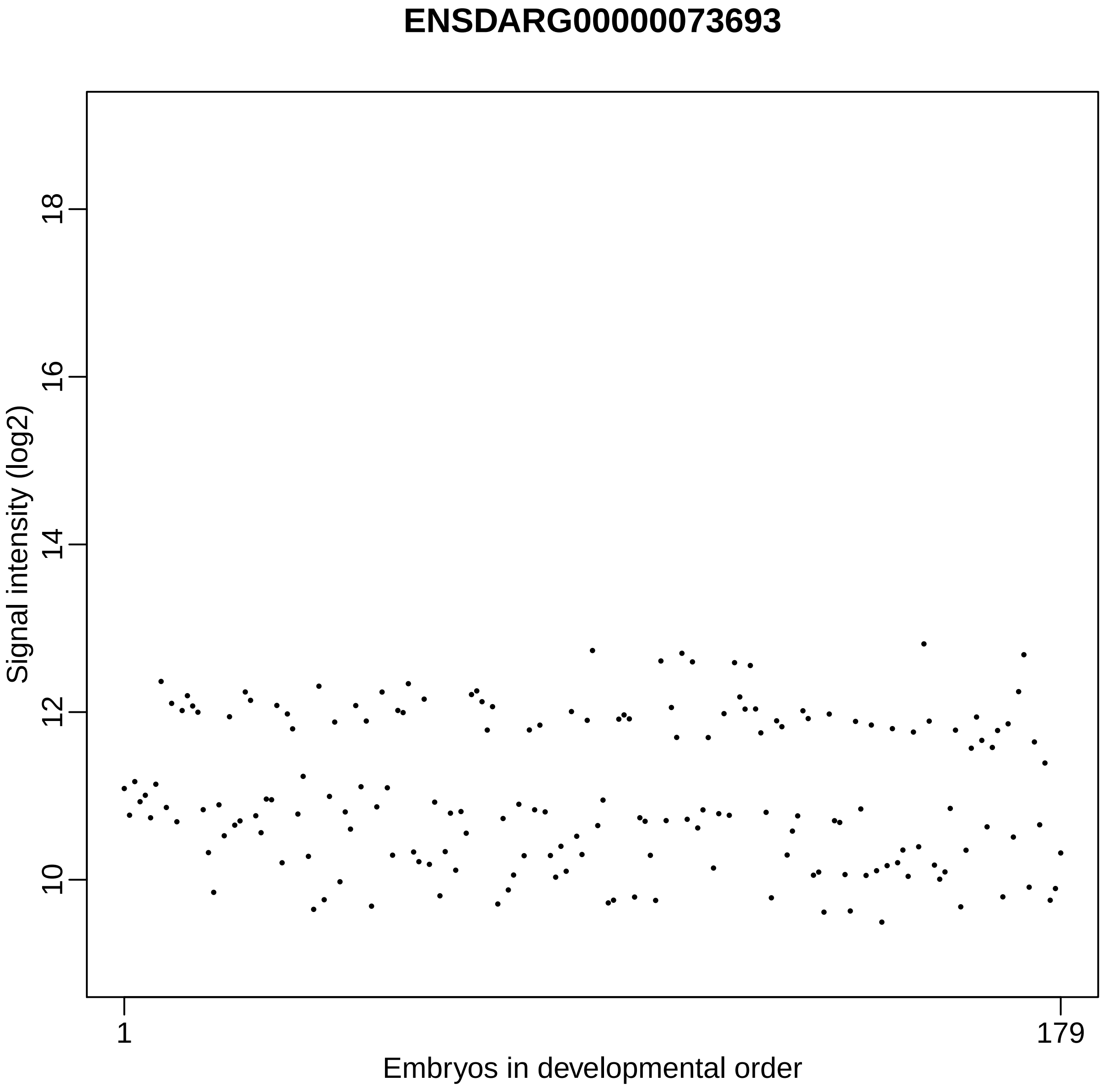

ENSDARG00000073693
Signal intensity (log2)
18
16
14
12
10
1
179
Embryos in developmental order

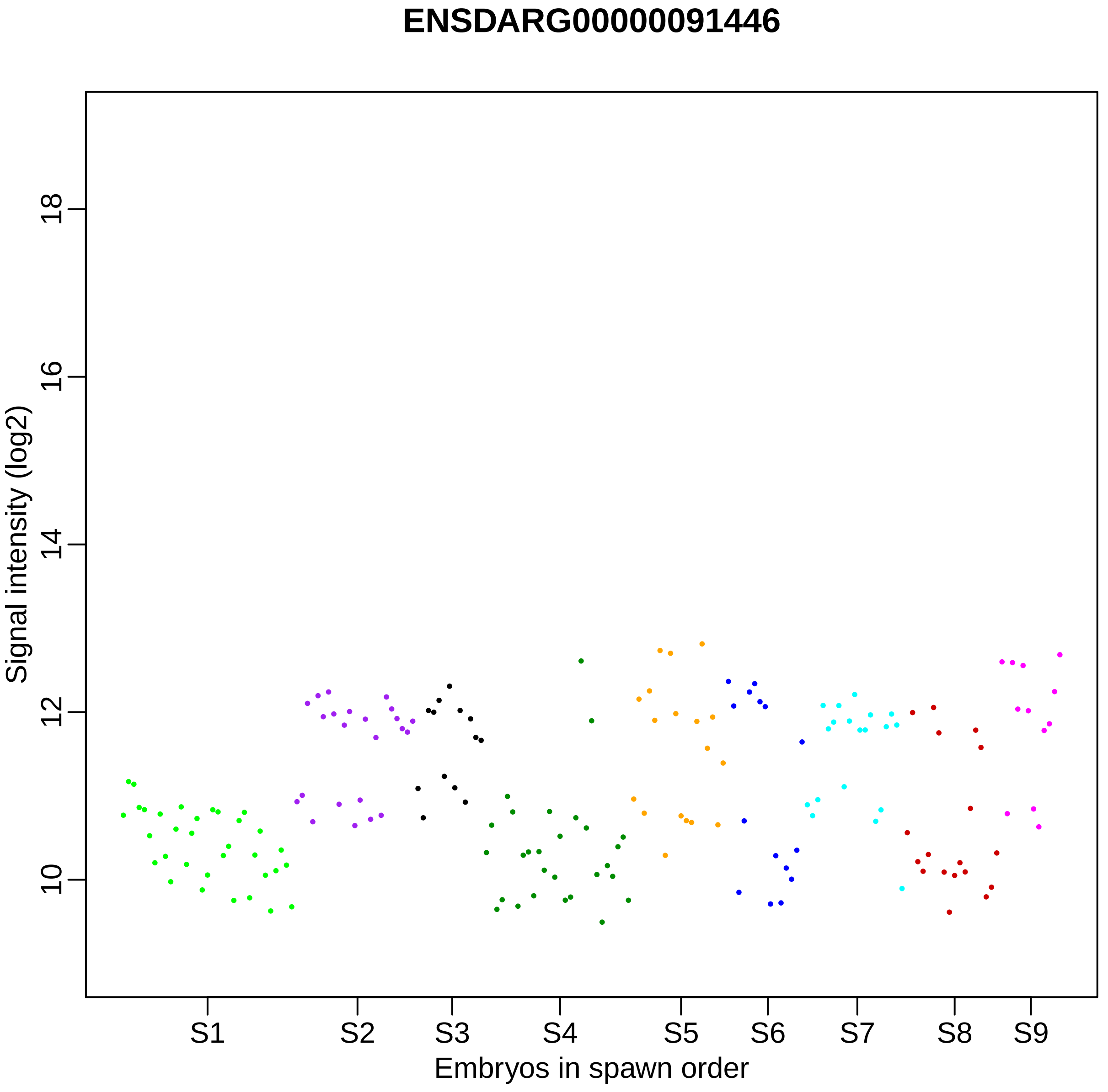

ENSDARG00000091446
Signal intensity (log2)
18
16
14
12
10
S1
S2
S3
S4
S5
S6
S7
S8
S9
Embryos in spawn order

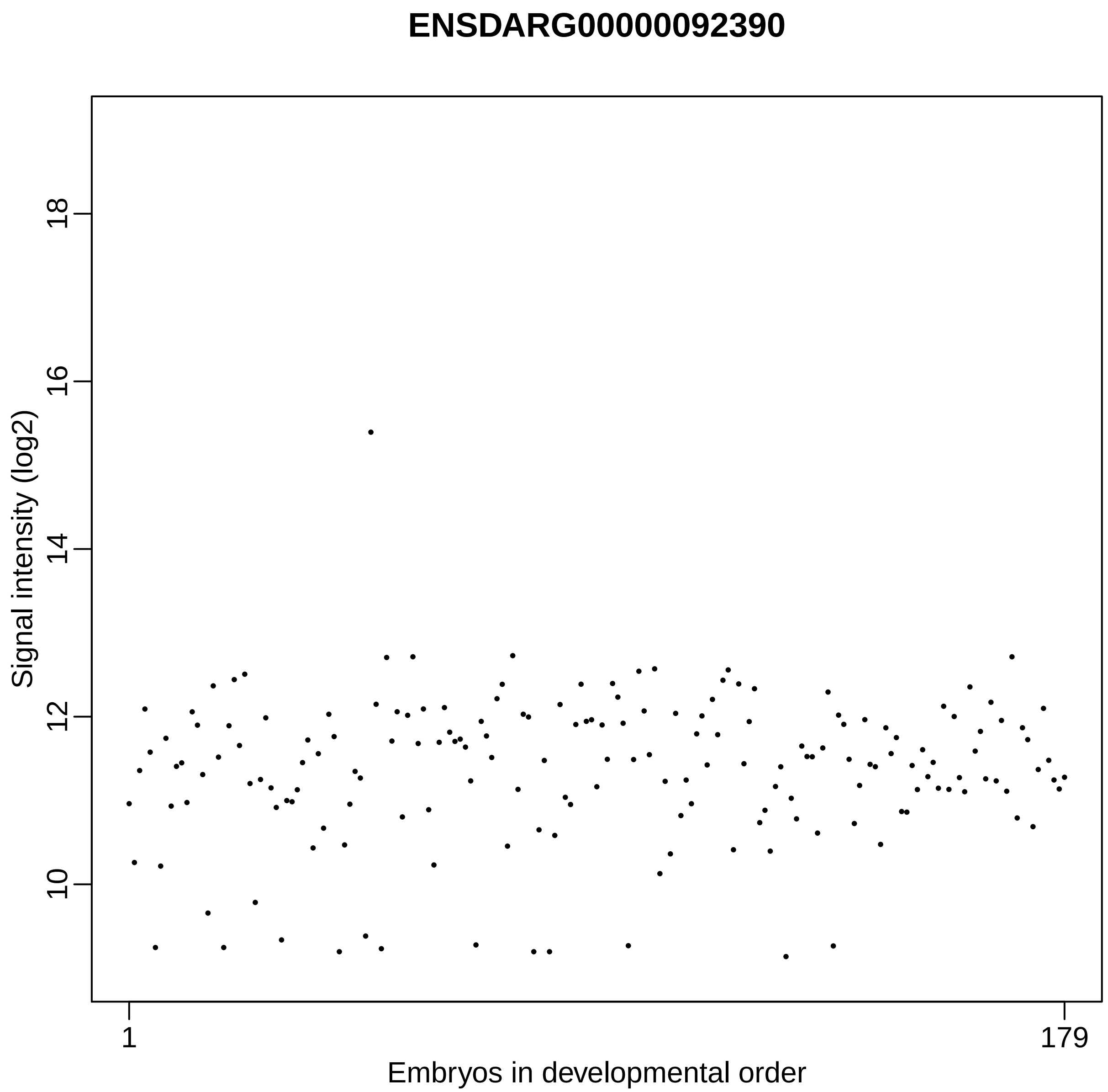
ENSDARG00000092390
Signal intensity (log2)
10
12
14
16
18
1
179
Embryos in developmental order

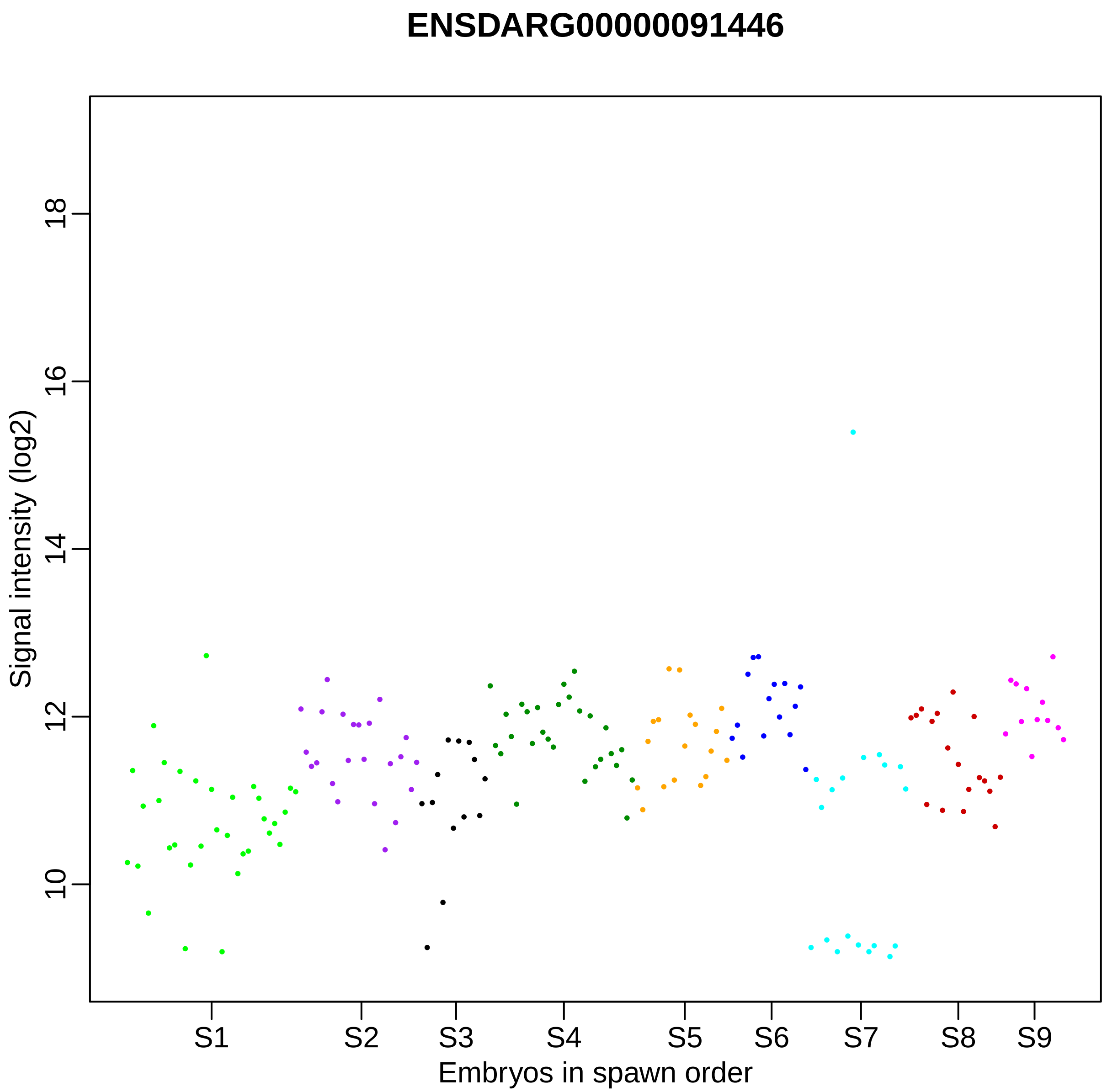
ENSDARG00000091446
Signal intensity (log2)
10
12
14
16
18
S1
S2
S3
S4
S5
S6
S7
S8
S9
Embryos in spawn order

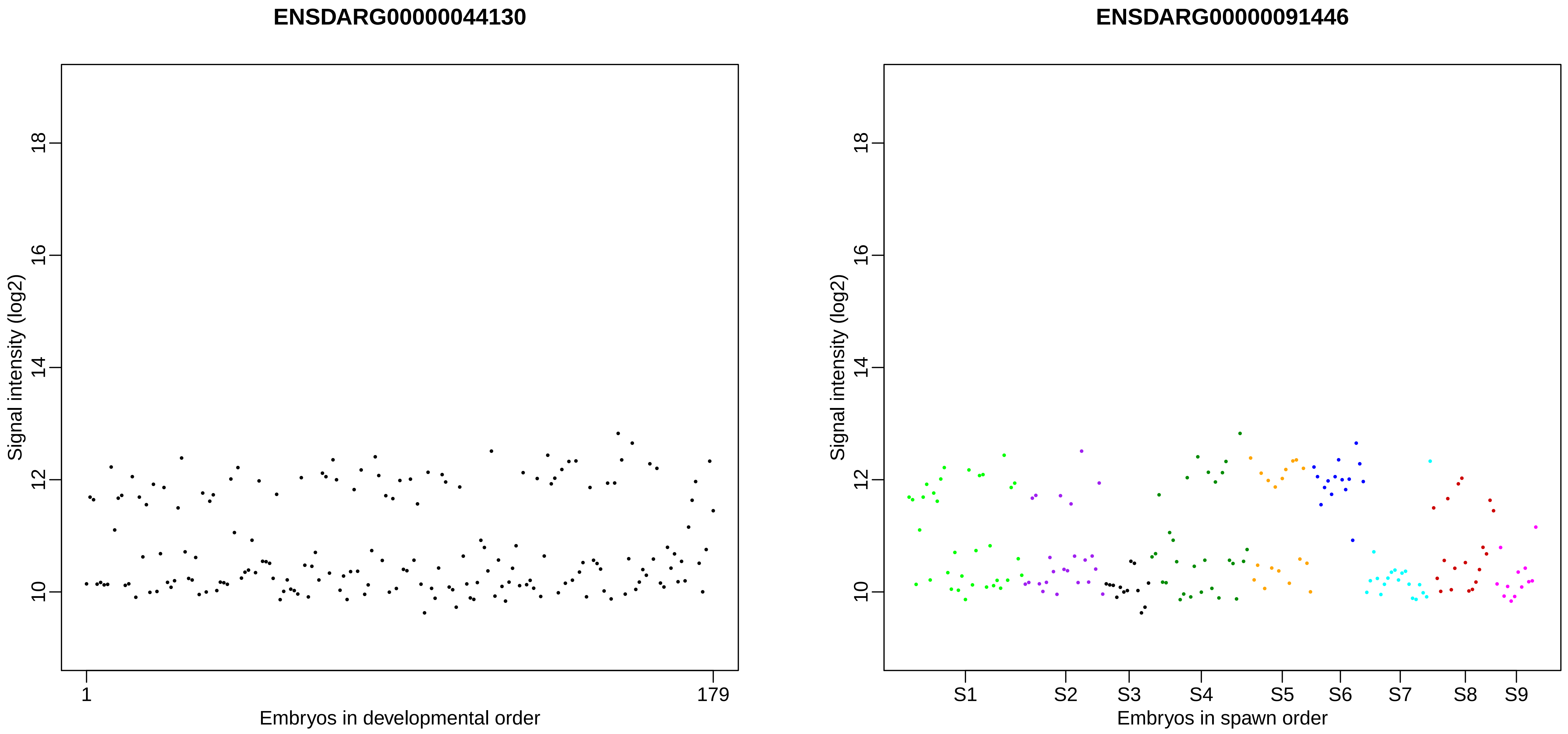
ENSDARG00000044130
Signal intensity (log2)
10
12
14
16
18
1
179
Embryos in developmental order
ENSDARG00000091446
Signal intensity (log2)
10
12
14
16
18
S1
S2
S3
S4
S5
S6
S7
S8
S9
Embryos in spawn order

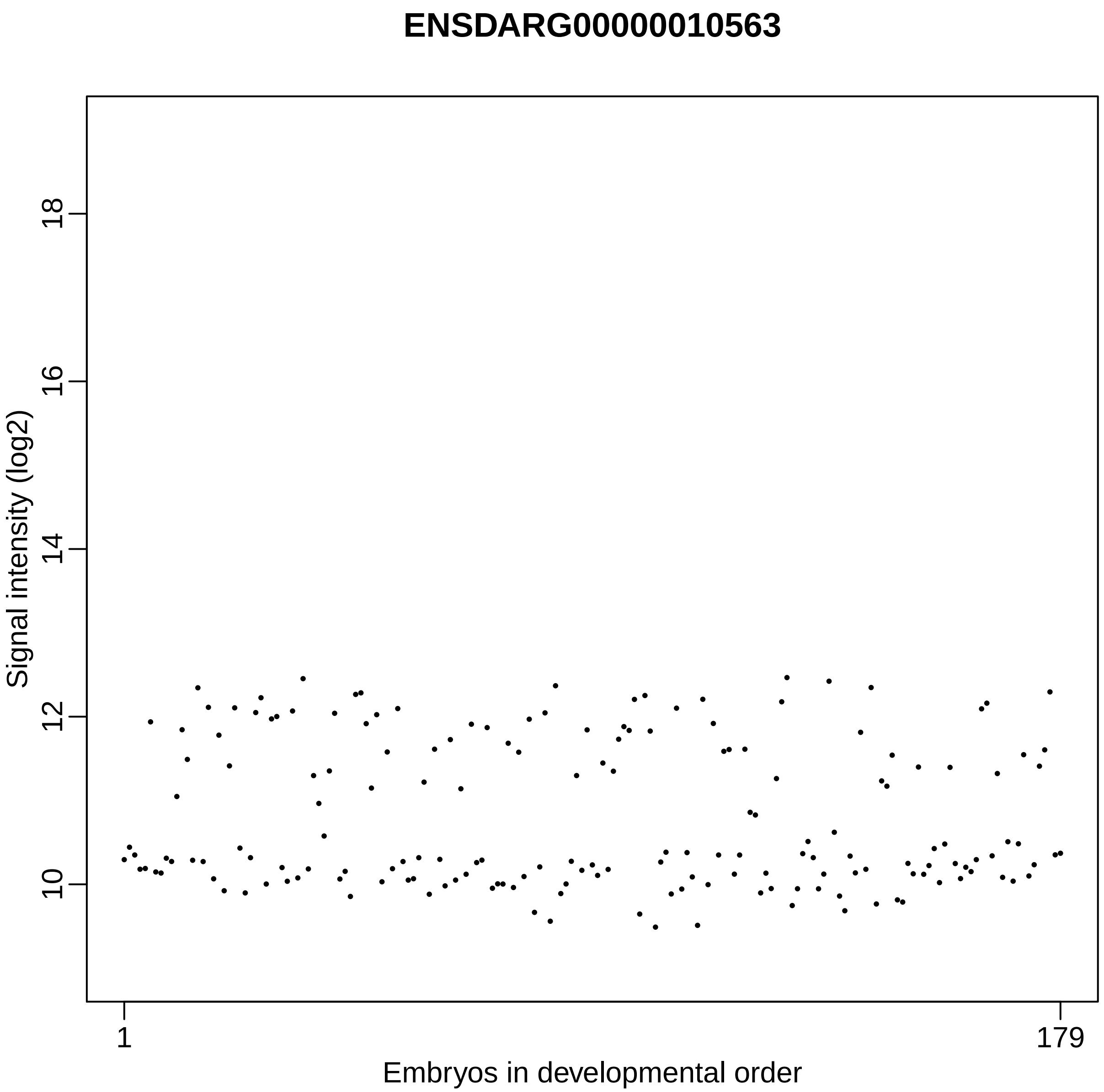
ENSDARG00000010563
Signal intensity (log2)
10
12
14
16
18
1
179
Embryos in developmental order

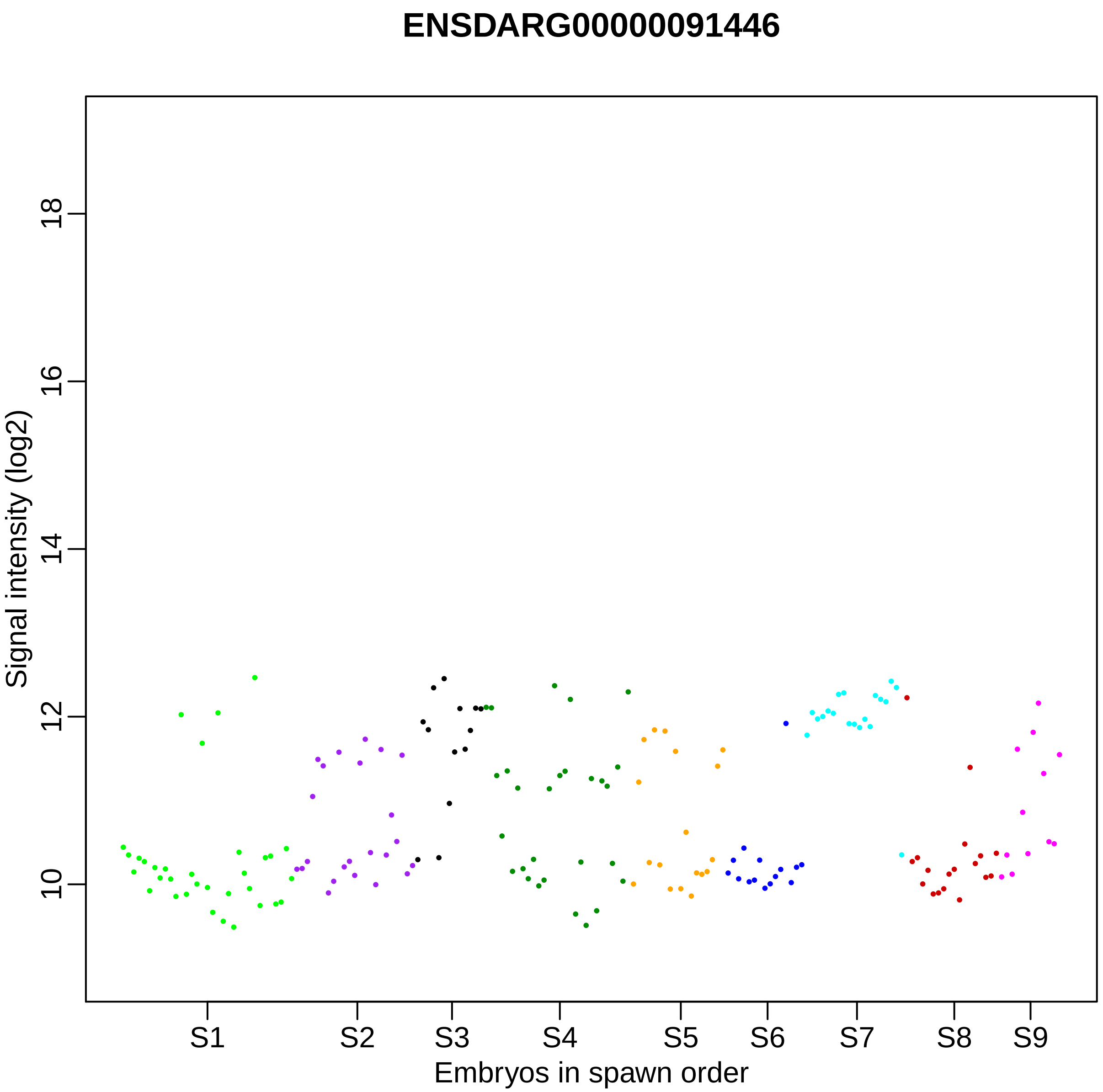
ENSDARG00000091446
Signal intensity (log2)
10
12
14
16
18
S1
S2
S3
S4
S5
S6
S7
S8
S9
Embryos in spawn order

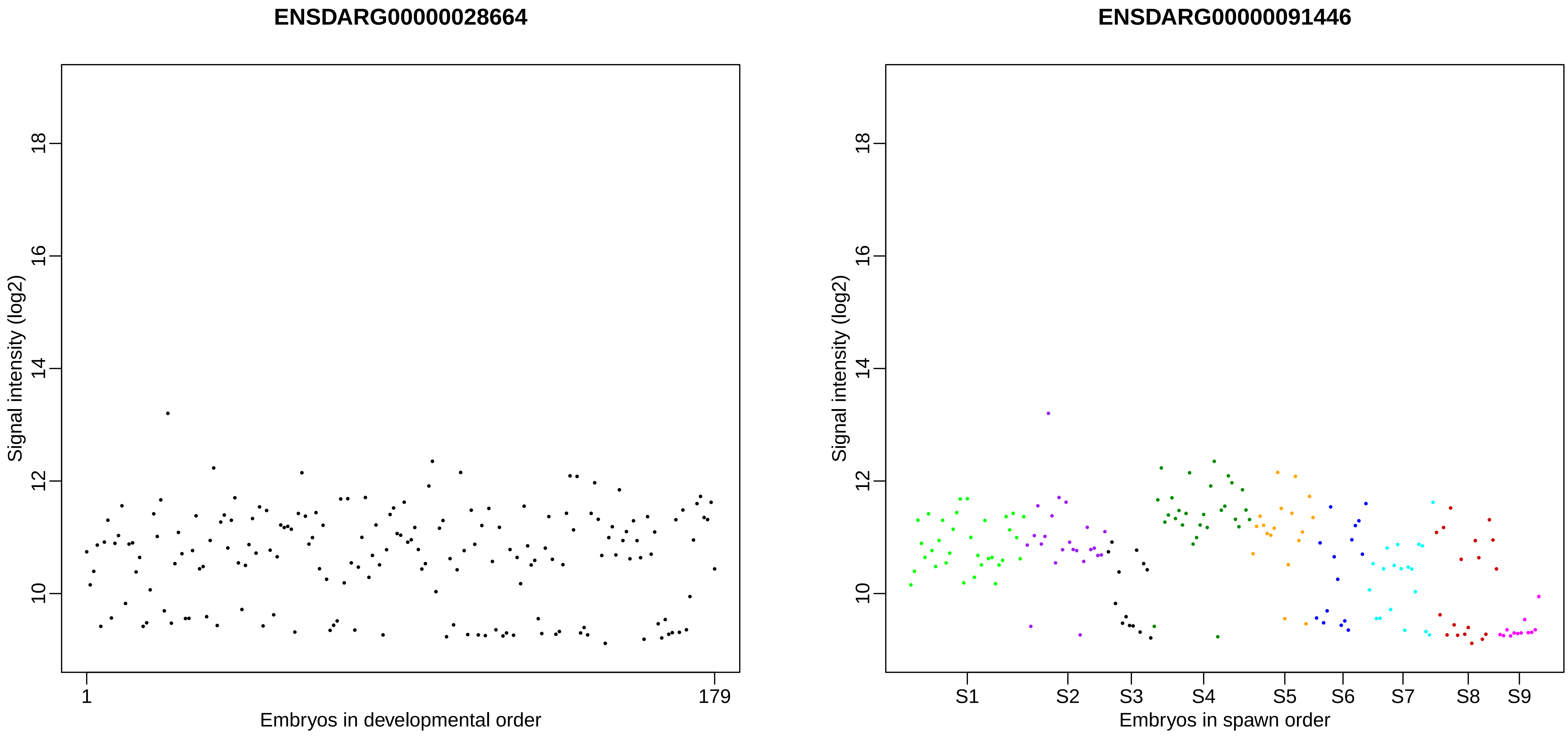

ENSDARG00000028664
Signal intensity (log2)
18
16
14
12
10
1
179
Embryos in developmental order
ENSDARG00000091446
Signal intensity (log2)
18
16
14
12
10
S1
S2
S3
S4
S5
S6
S7
S8
S9
Embryos in spawn order

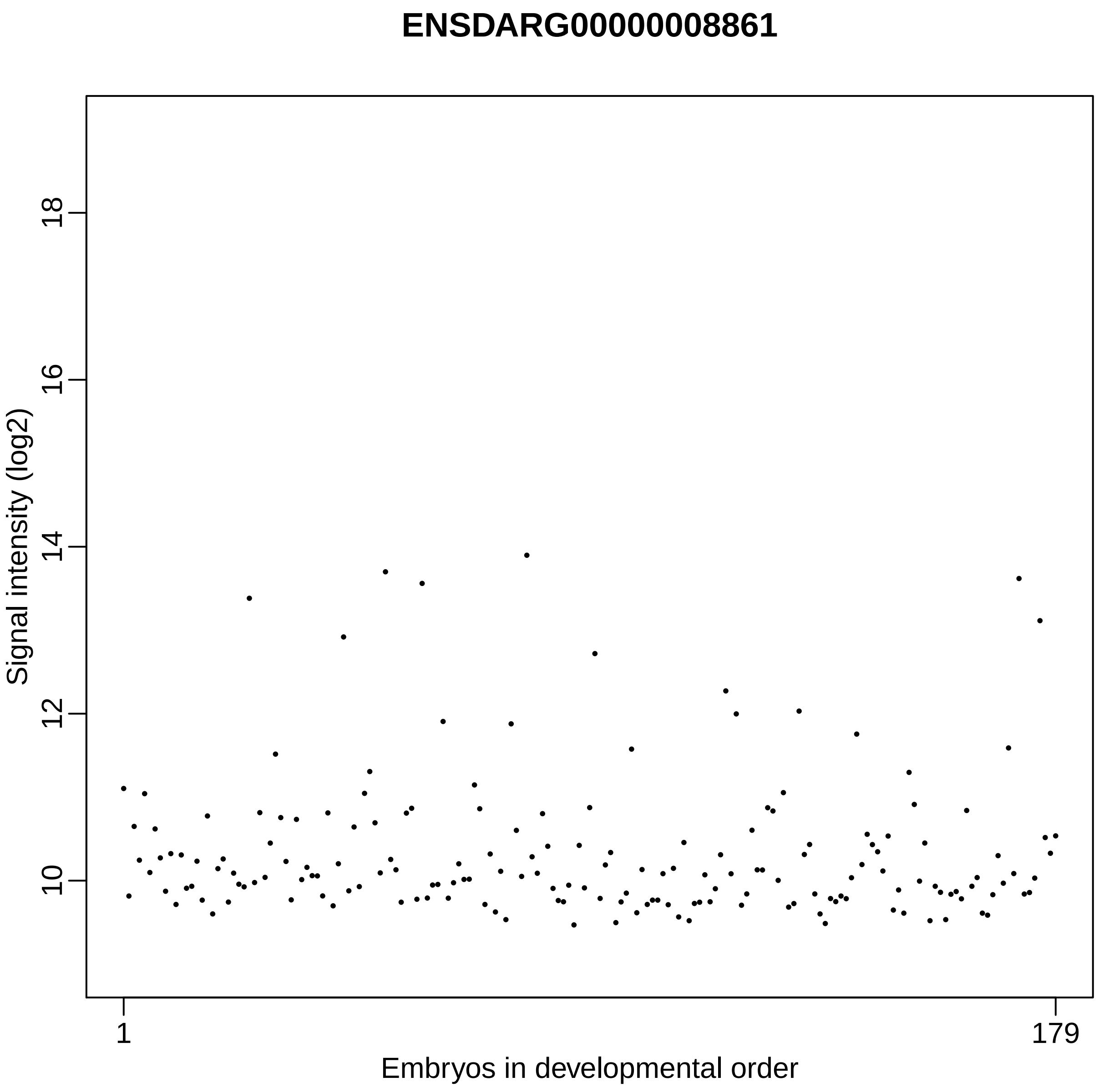

ENSDARG00000008861
Signal intensity (log2)
10
12
14
16
18
1
179
Embryos in developmental order

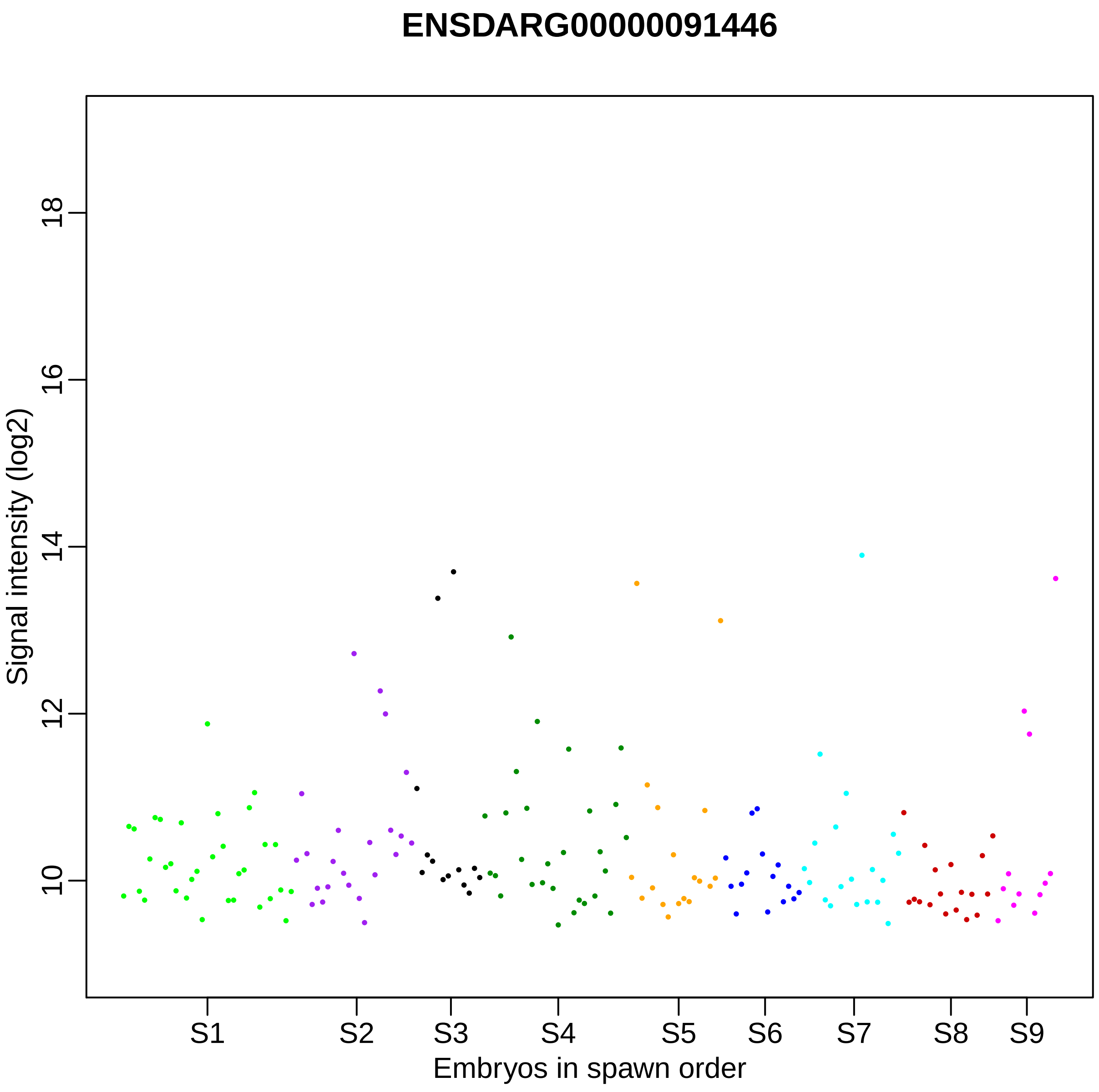

ENSDARG00000091446
Signal intensity (log2)
10
12
14
16
18
S1
S2
S3
S4
S5
S6
S7
S8
S9
Embryos in spawn order

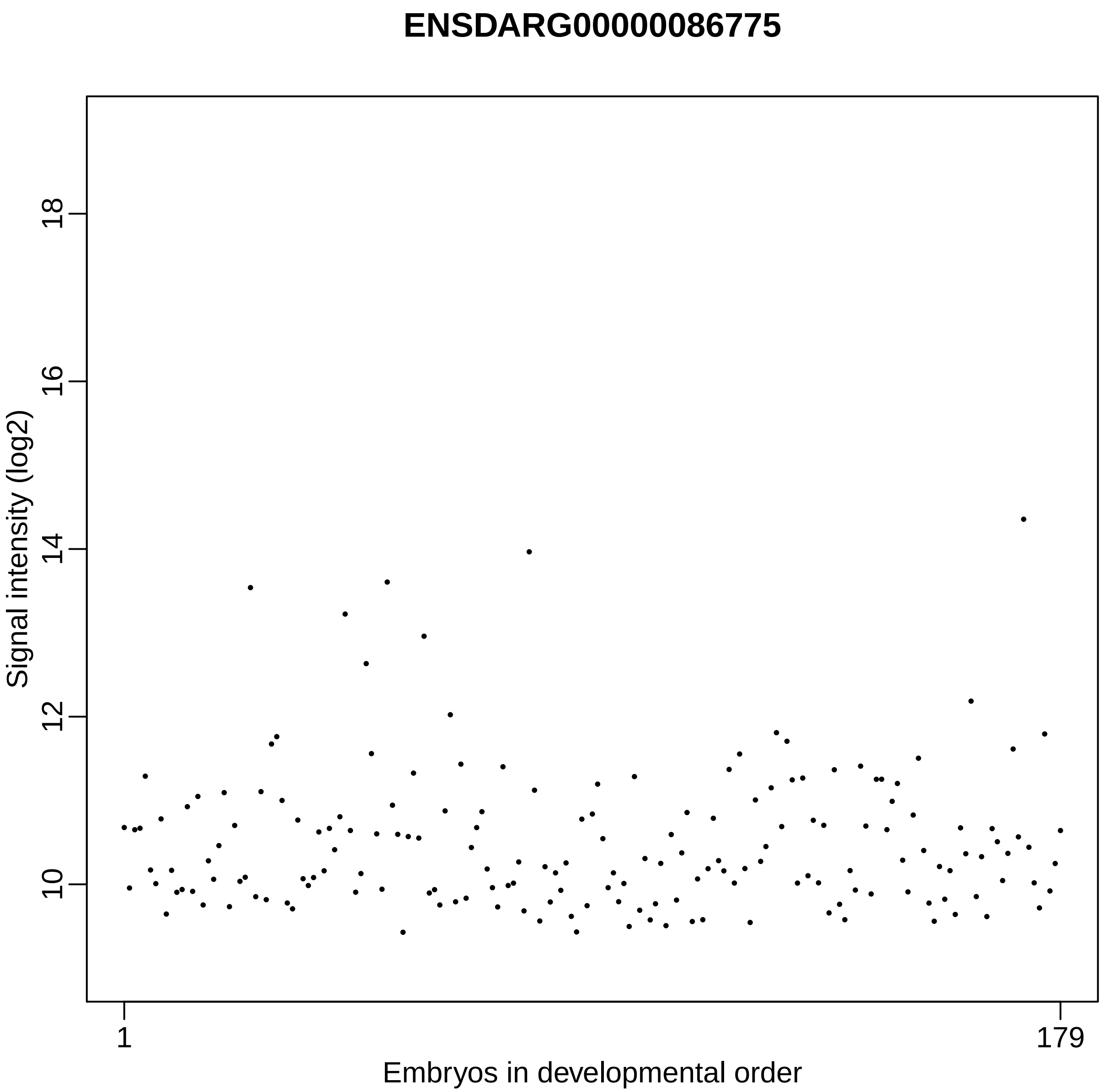
ENSDARG00000086775
Signal intensity (log2)
10
12
14
16
18
1
179
Embryos in developmental order

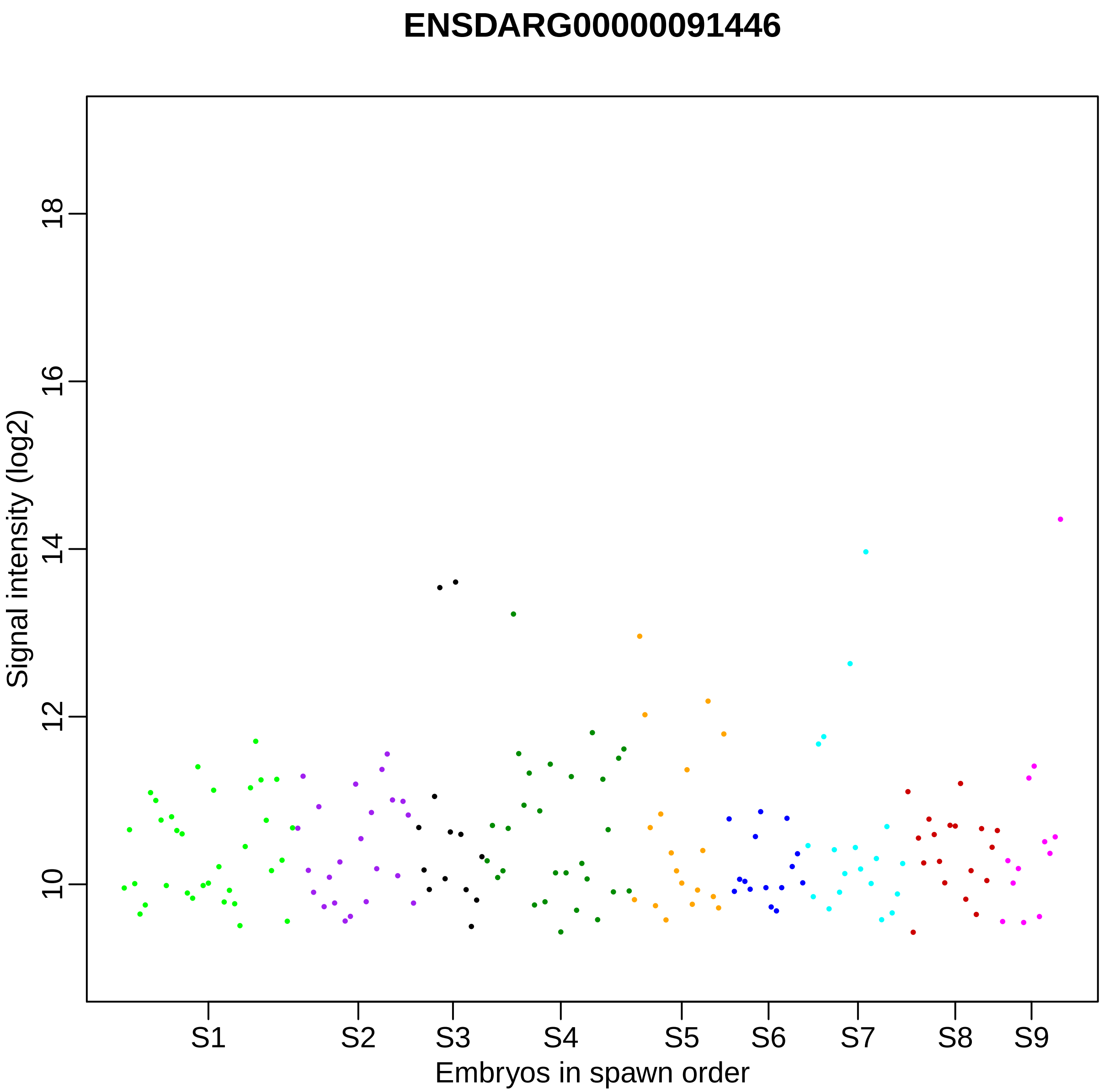
ENSDARG00000091446
Signal intensity (log2)
10
12
14
16
18
S1
S2
S3
S4
S5
S6
S7
S8
S9
Embryos in spawn order

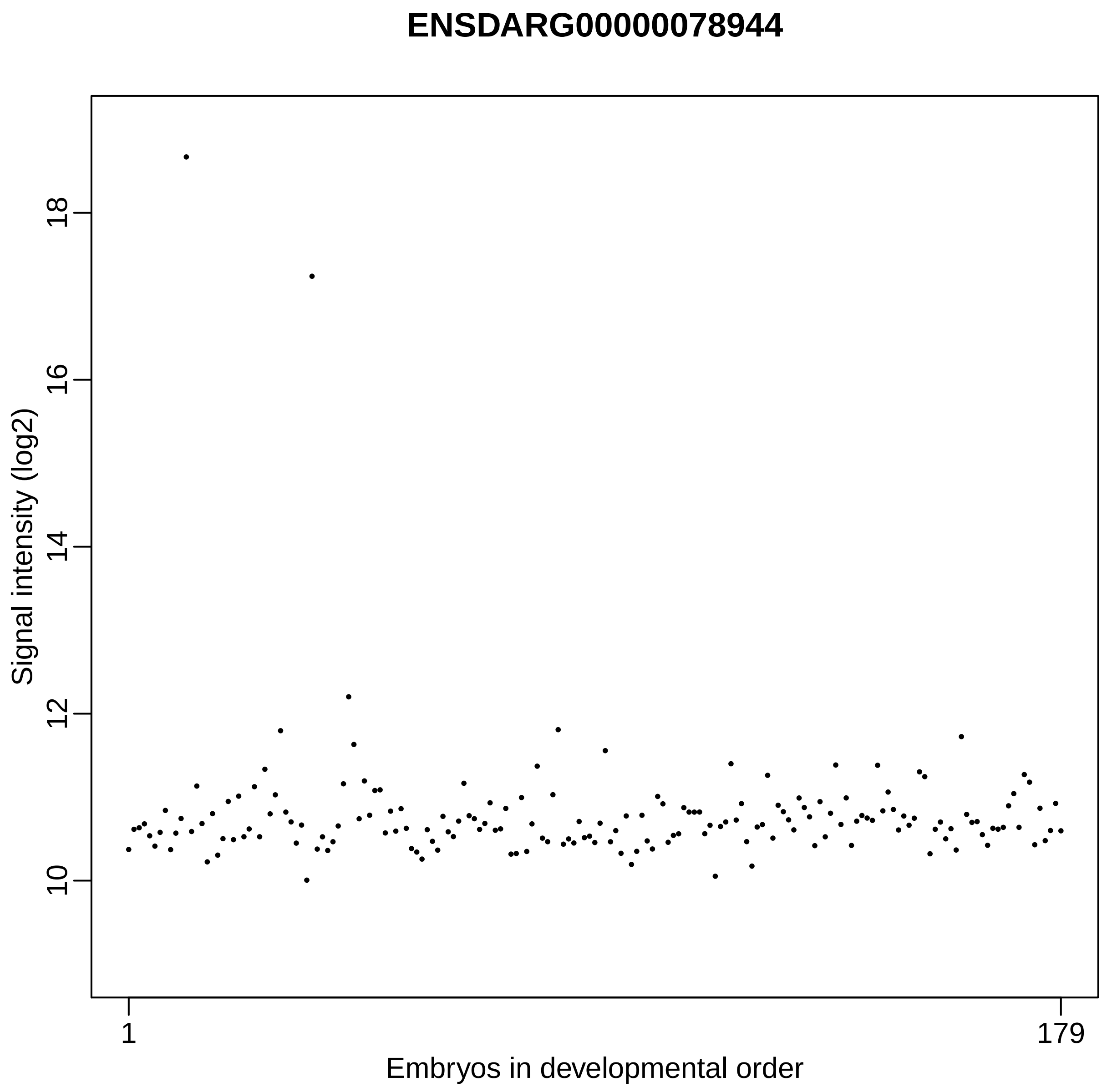
ENSDARG00000078944
Signal intensity (log2)
10
12
14
16
18
1
179
Embryos in developmental order

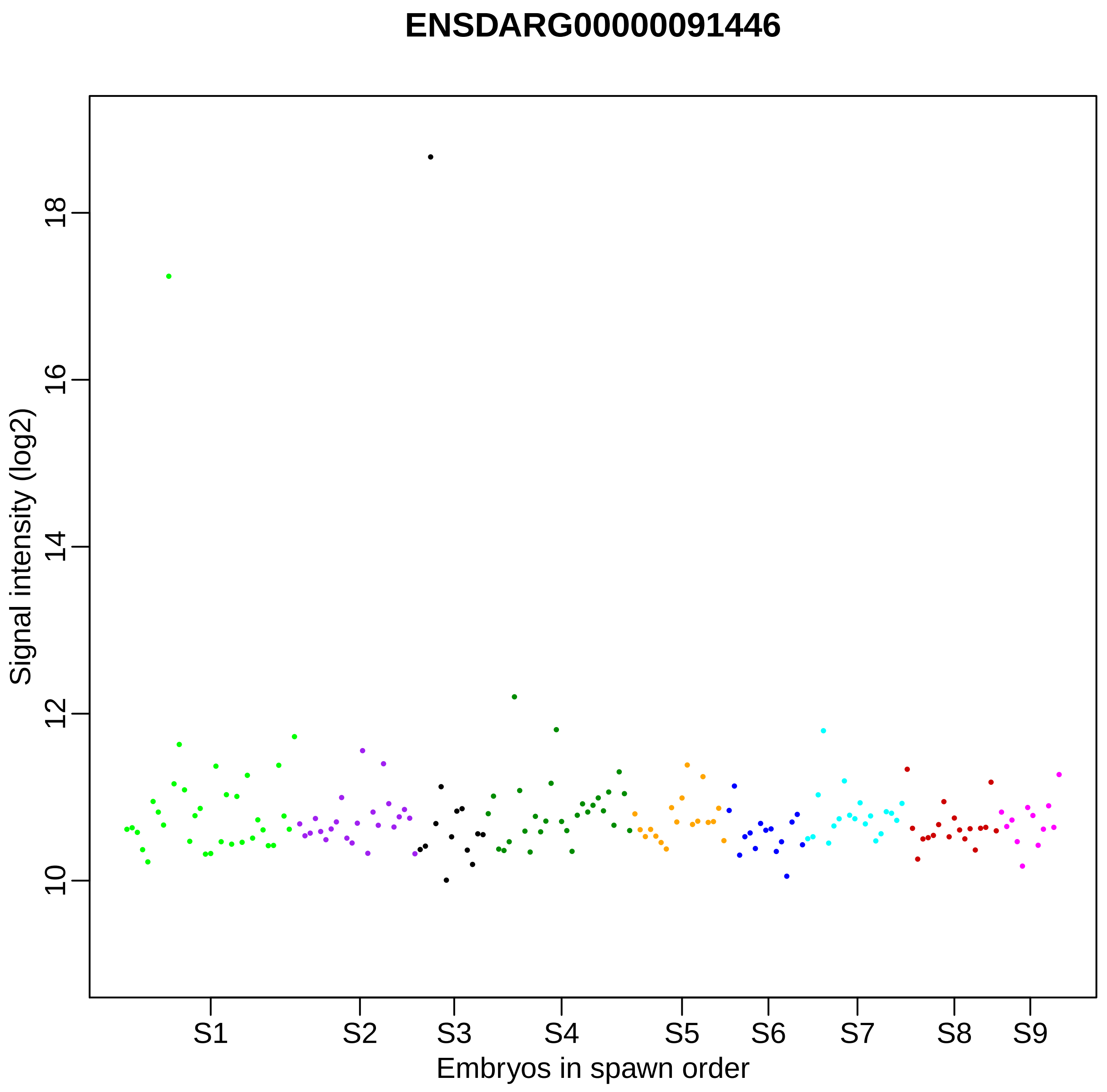
ENSDARG00000091446
Signal intensity (log2)
10
12
14
16
18
S1
S2
S3
S4
S5
S6
S7
S8
S9
Embryos in spawn order

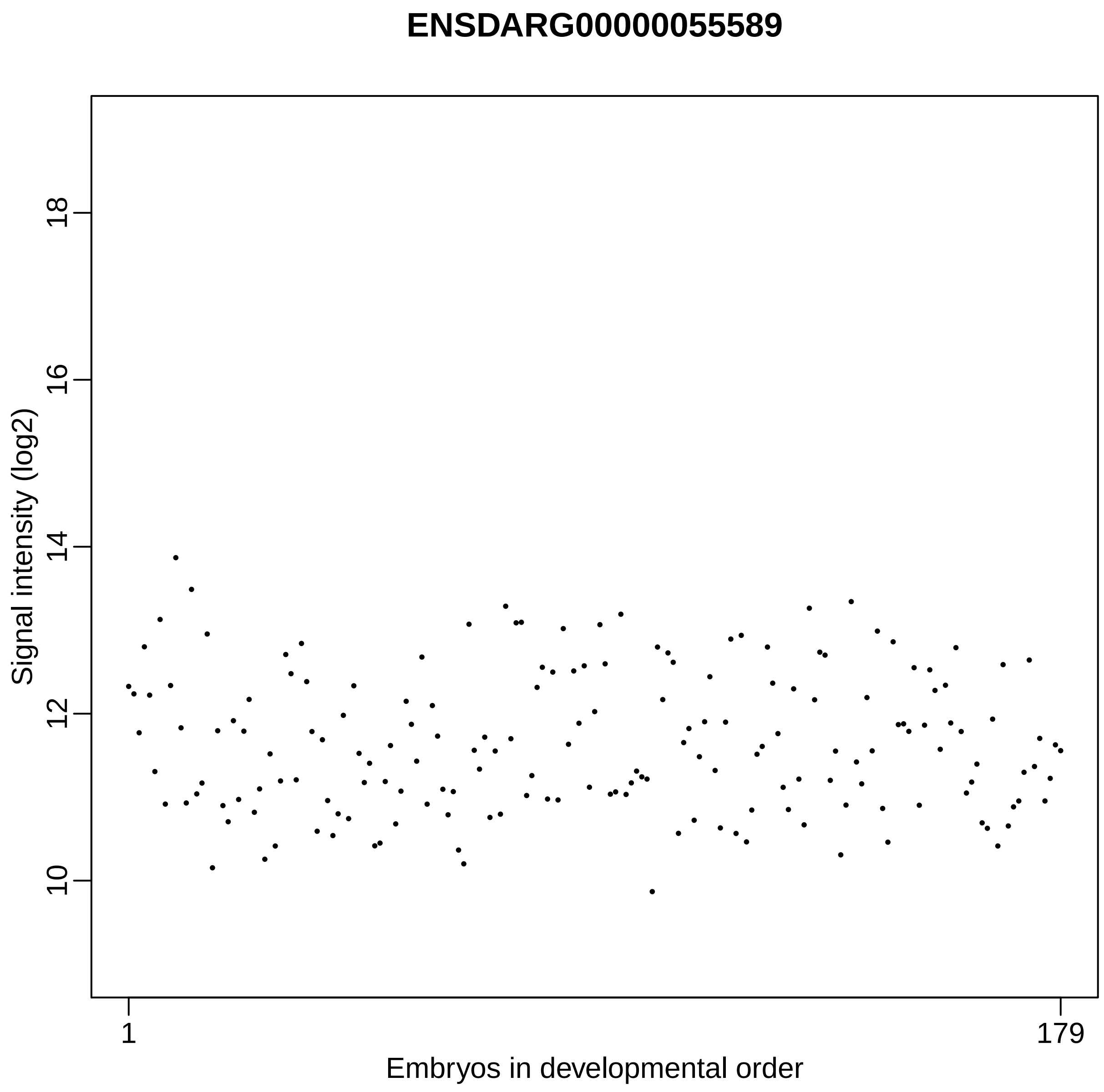

ENSDARG00000055589
Signal intensity (log2)
18
16
14
12
10
1
179
Embryos in developmental order

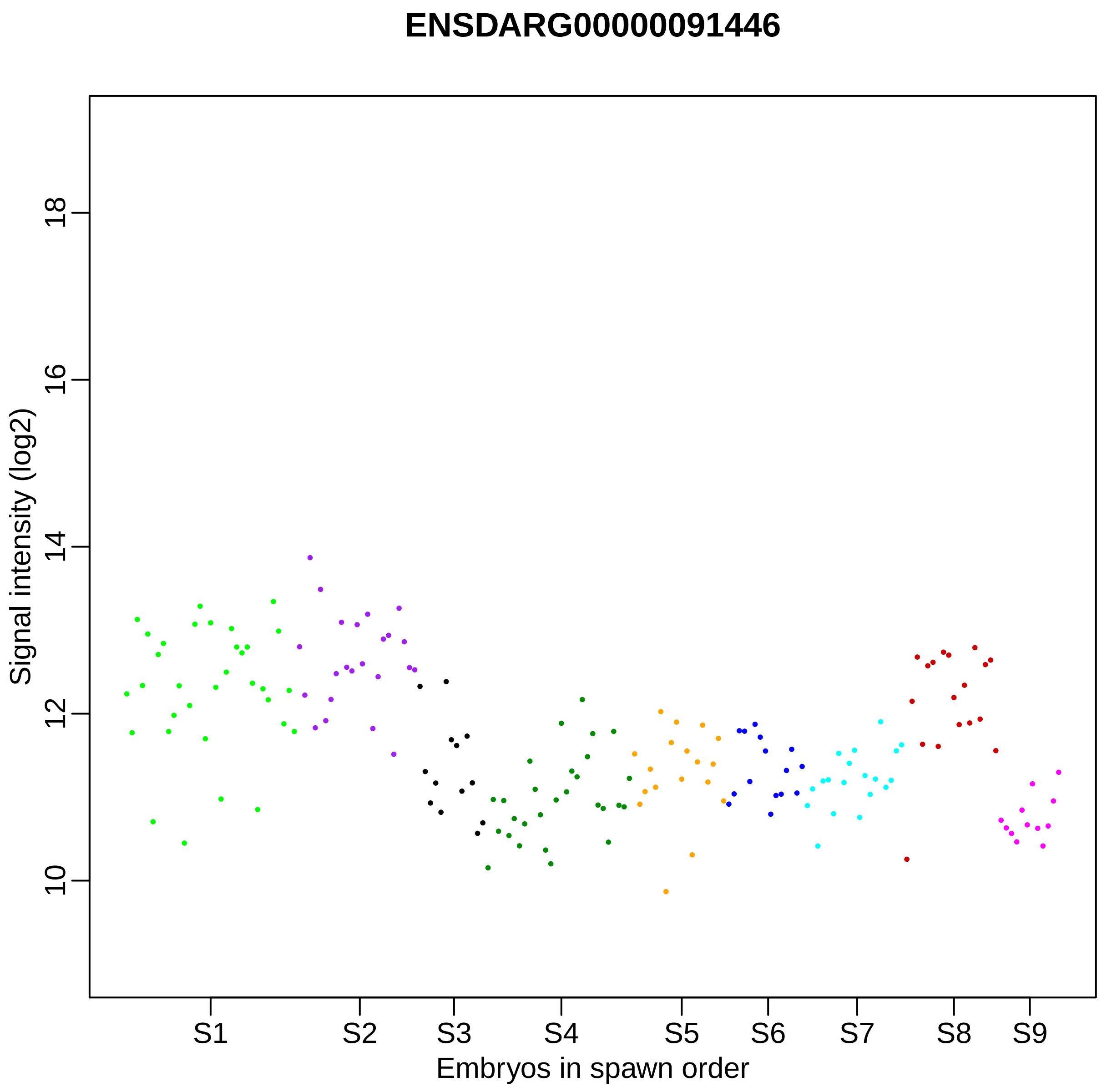

ENSDARG00000091446
Signal intensity (log2)
18
16
14
12
10
S1
S2
S3
S4
S5
S6
S7
S8
S9
Embryos in spawn order

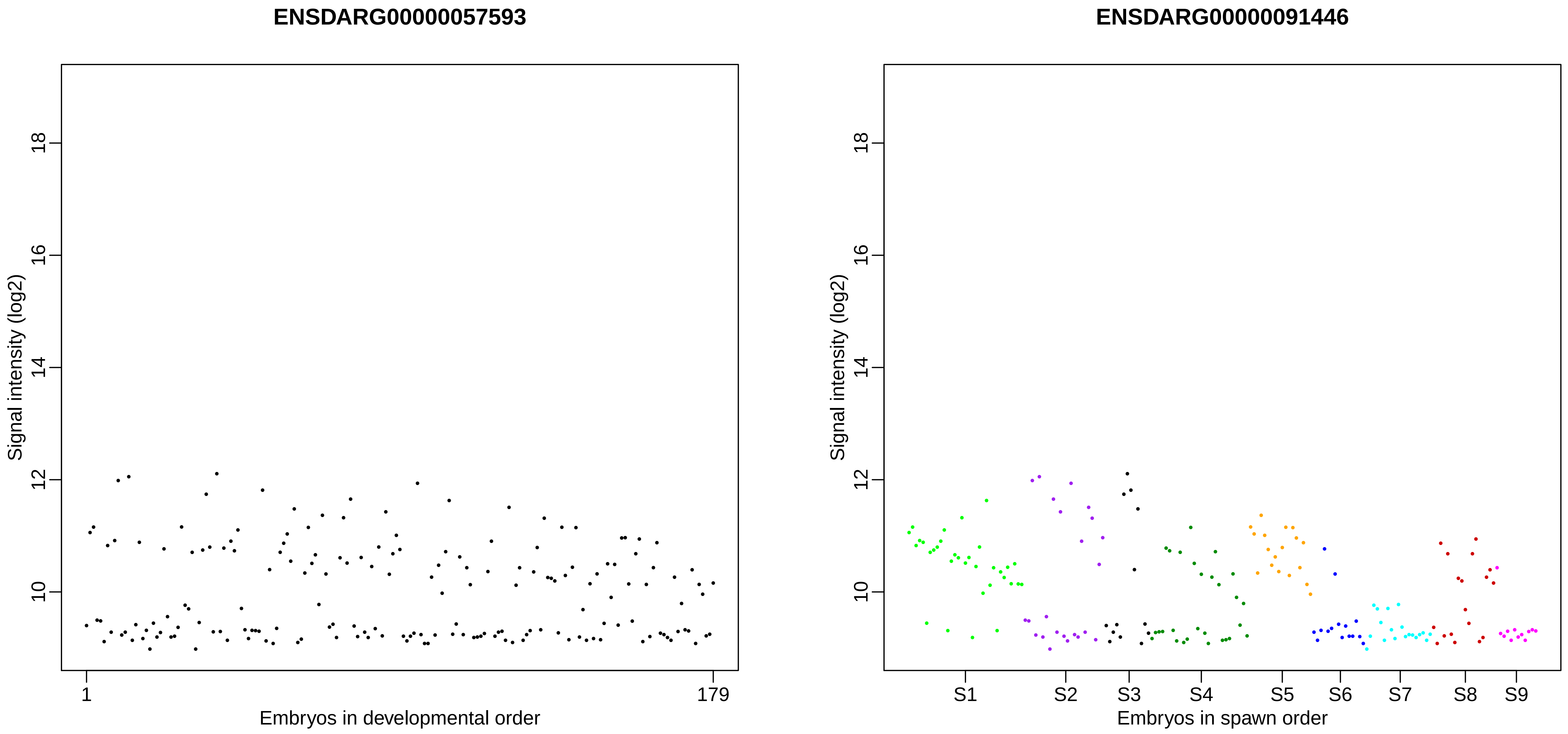
ENSDARG00000057593
Signal intensity (log2)
10
12
14
16
18
1
179
Embryos in developmental order
ENSDARG00000091446
Signal intensity (log2)
10
12
14
16
18
S1
S2
S3
S4
S5
S6
S7
S8
S9
Embryos in spawn order

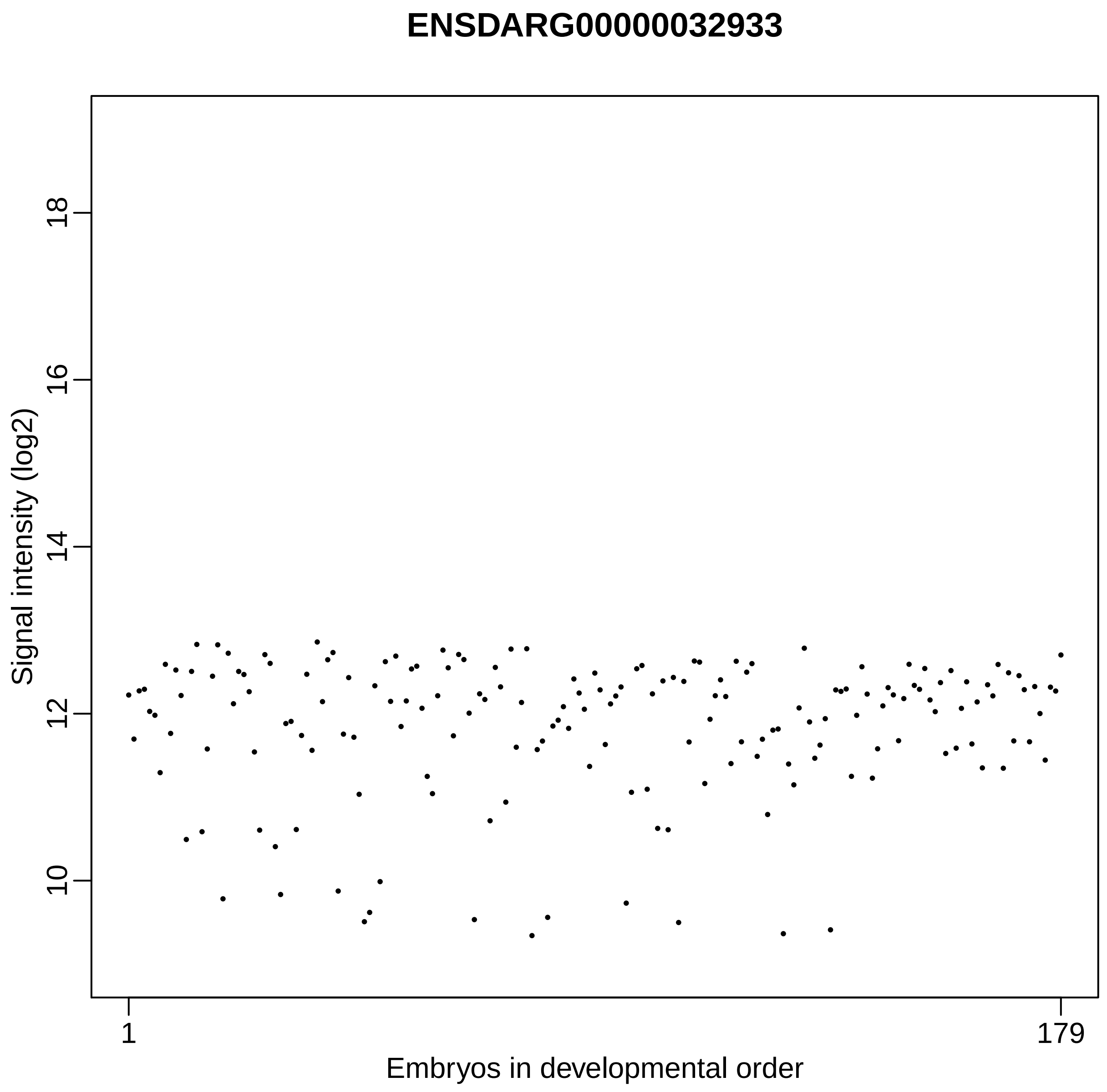
ENSDARG00000032933
Signal intensity (log2)
10
12
14
16
18
1
179
Embryos in developmental order

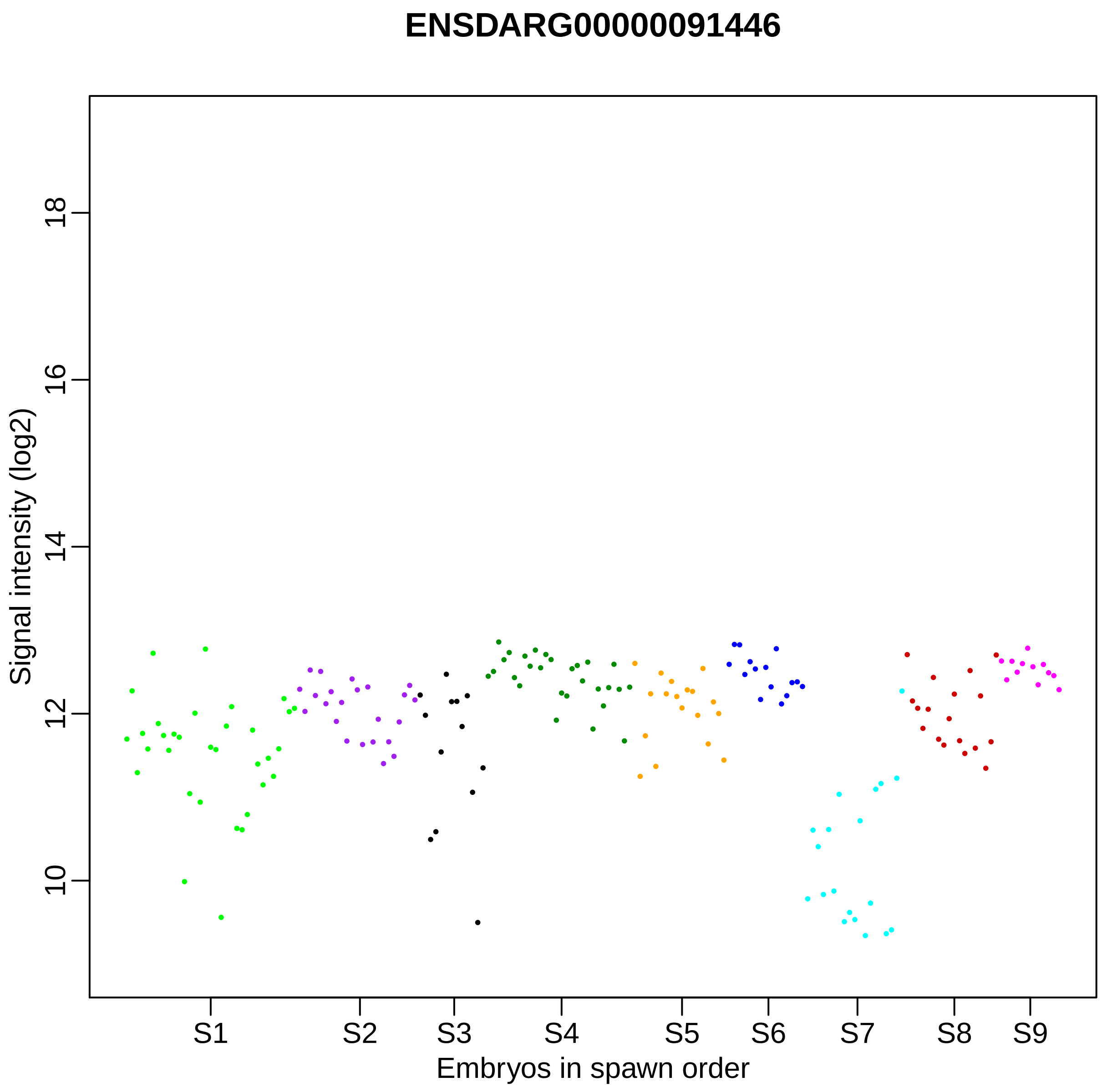
ENSDARG00000091446
Signal intensity (log2)
10
12
14
16
18
S1
S2
S3
S4
S5
S6
S7
S8
S9
Embryos in spawn order

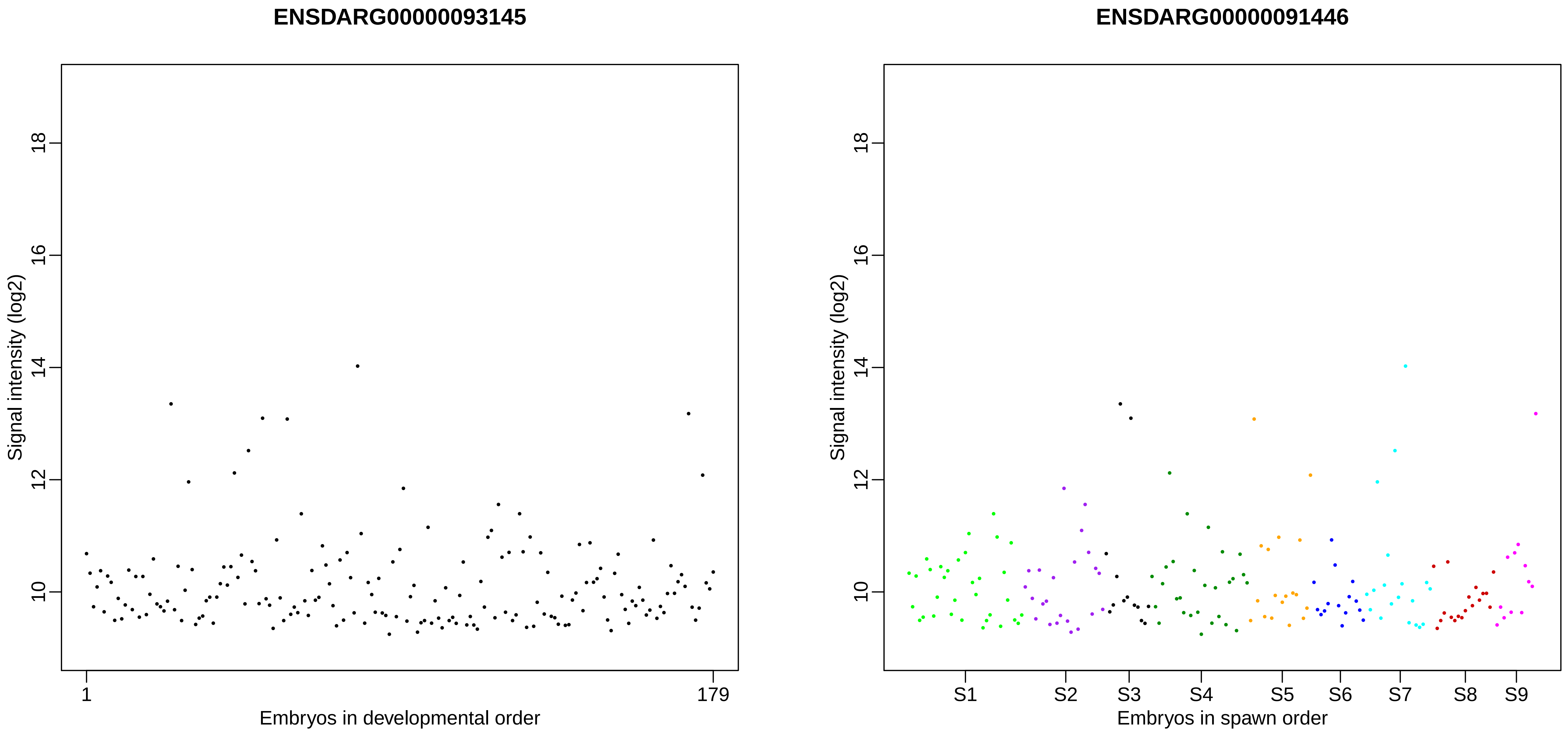

ENSDARG00000093145
Signal intensity (log2)
10
12
14
16
18
1
179
Embryos in developmental order
ENSDARG00000091446
Signal intensity (log2)
10
12
14
16
18
S1
S2
S3
S4
S5
S6
S7
S8
S9
Embryos in spawn order

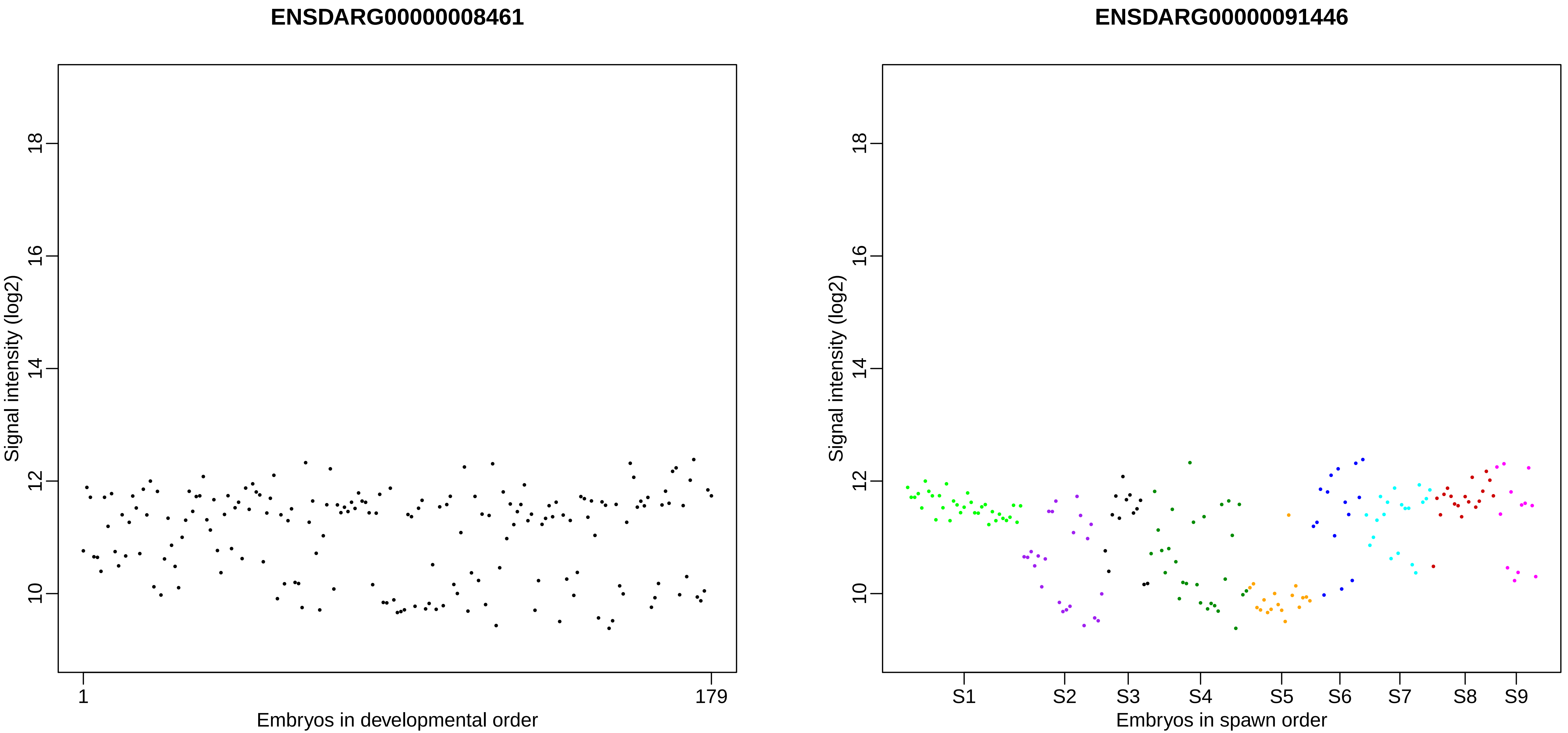

ENSDARG00000008461
Signal intensity (log2)
10
12
14
16
18
1
179
Embryos in developmental order
ENSDARG00000091446
Signal intensity (log2)
10
12
14
16
18
S1
S2
S3
S4
S5
S6
S7
S8
S9
Embryos in spawn order

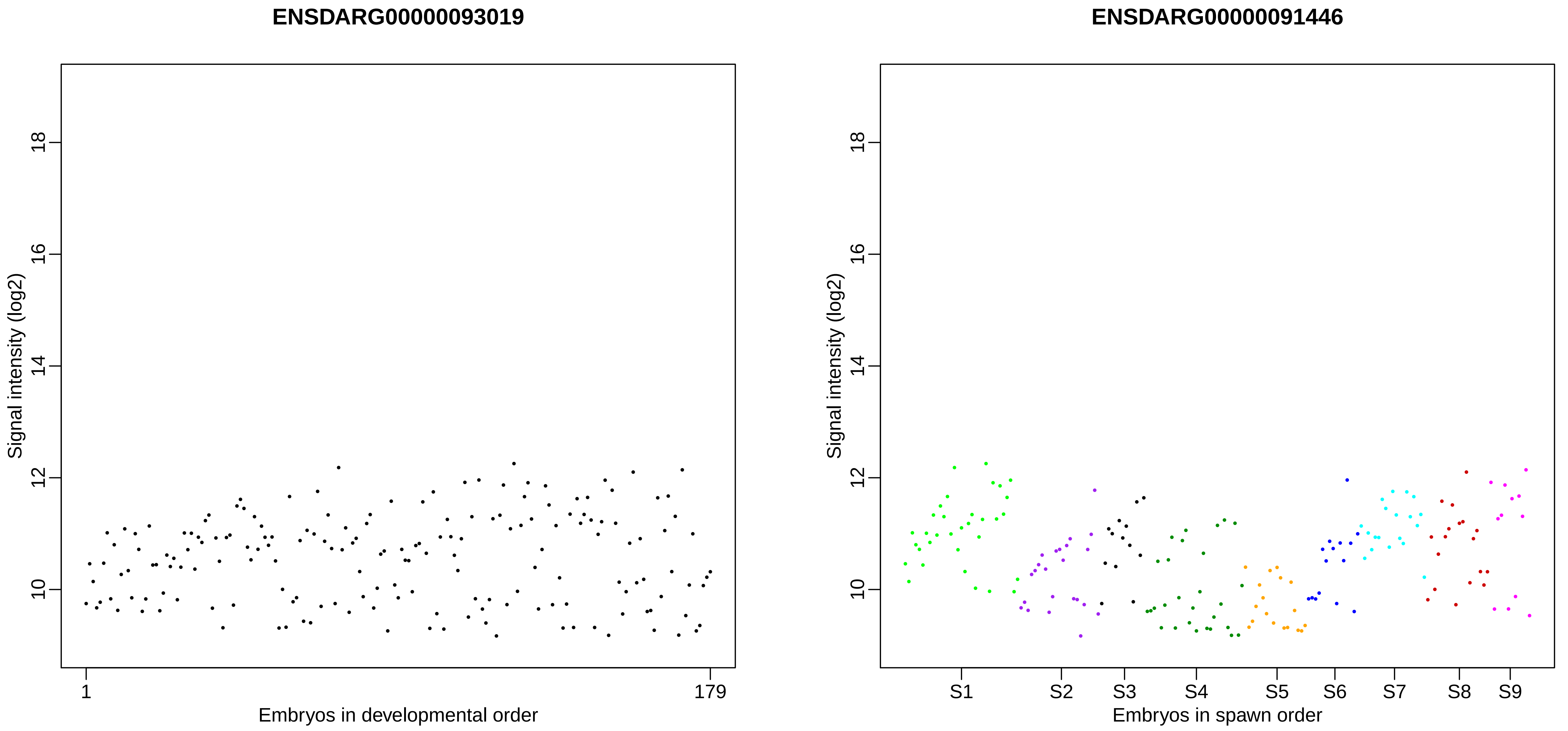
ENSDARG00000093019
Signal intensity (log2)
10
12
14
16
18
1
179
Embryos in developmental order
ENSDARG00000091446
Signal intensity (log2)
10
12
14
16
18
S1
S2
S3
S4
S5
S6
S7
S8
S9
Embryos in spawn order

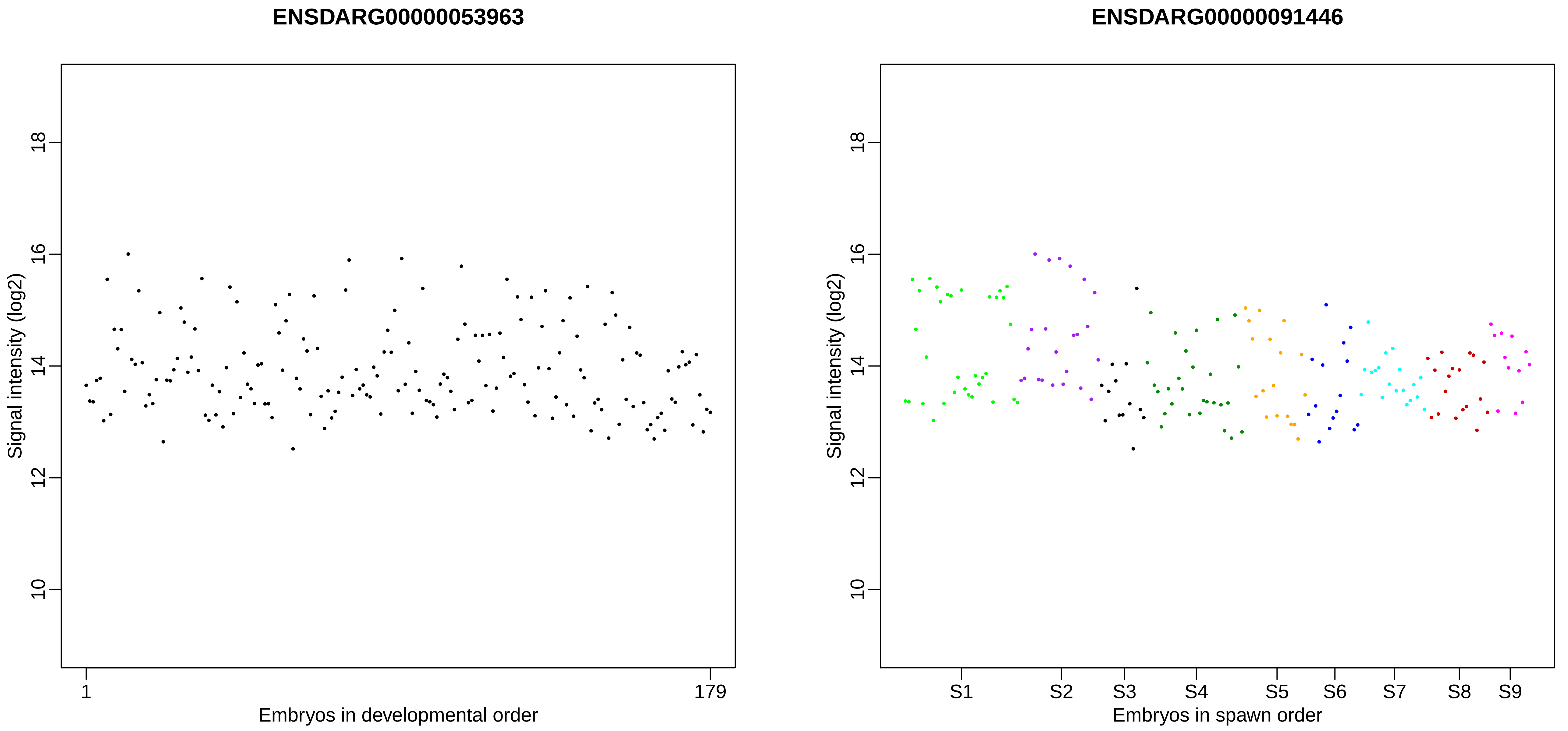

ENSDARG00000053963
Signal intensity (log2)
10
12
14
16
18
1
179
Embryos in developmental order
ENSDARG00000091446
S1
S2
S3
S4
S5
S6
S7
S8
S9
Embryos in spawn order

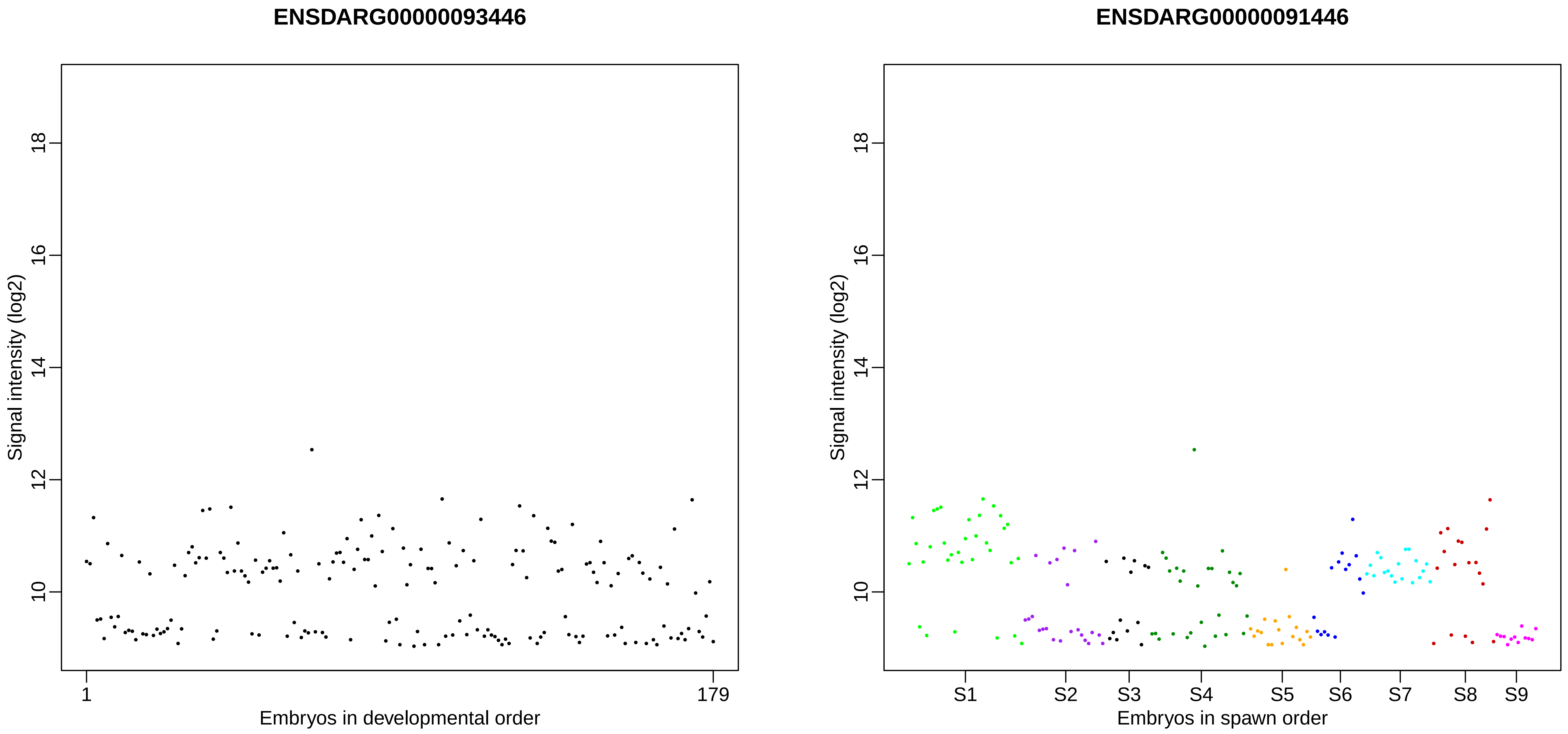
ENSDARG00000093446
Signal intensity (log2)
10
12
14
16
18
1
179
Embryos in developmental order
ENSDARG00000091446
Signal intensity (log2)
10
12
14
16
18
S1
S2
S3
S4
S5
S6
S7
S8
S9
Embryos in spawn order

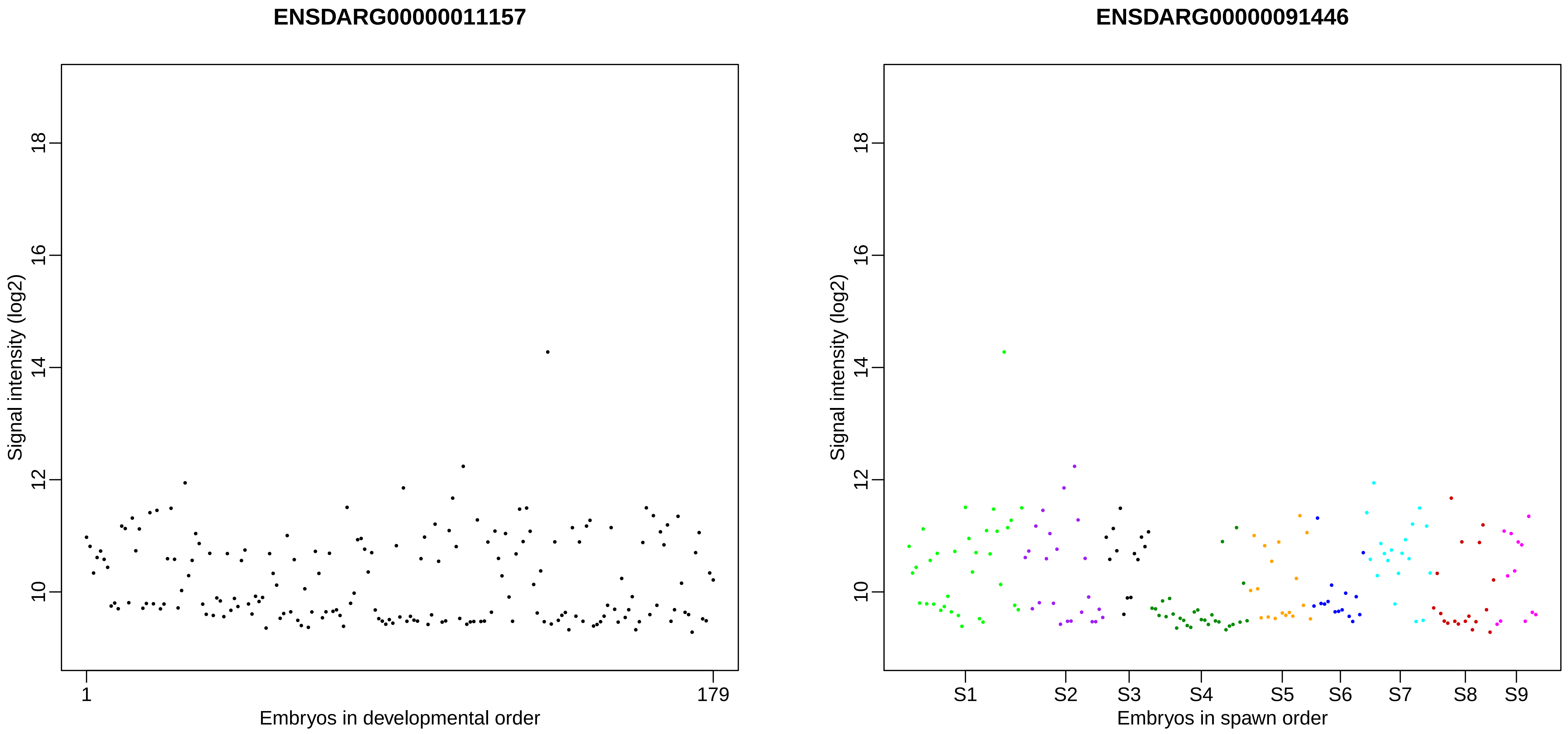
ENSDARG00000011157
Signal intensity (log2)
18
16
14
12
10
1
179
Embryos in developmental order
ENSDARG00000091446
Signal intensity (log2)
18
16
14
12
10
S1
S2
S3
S4
S5
S6
S7
S8
S9
Embryos in spawn order

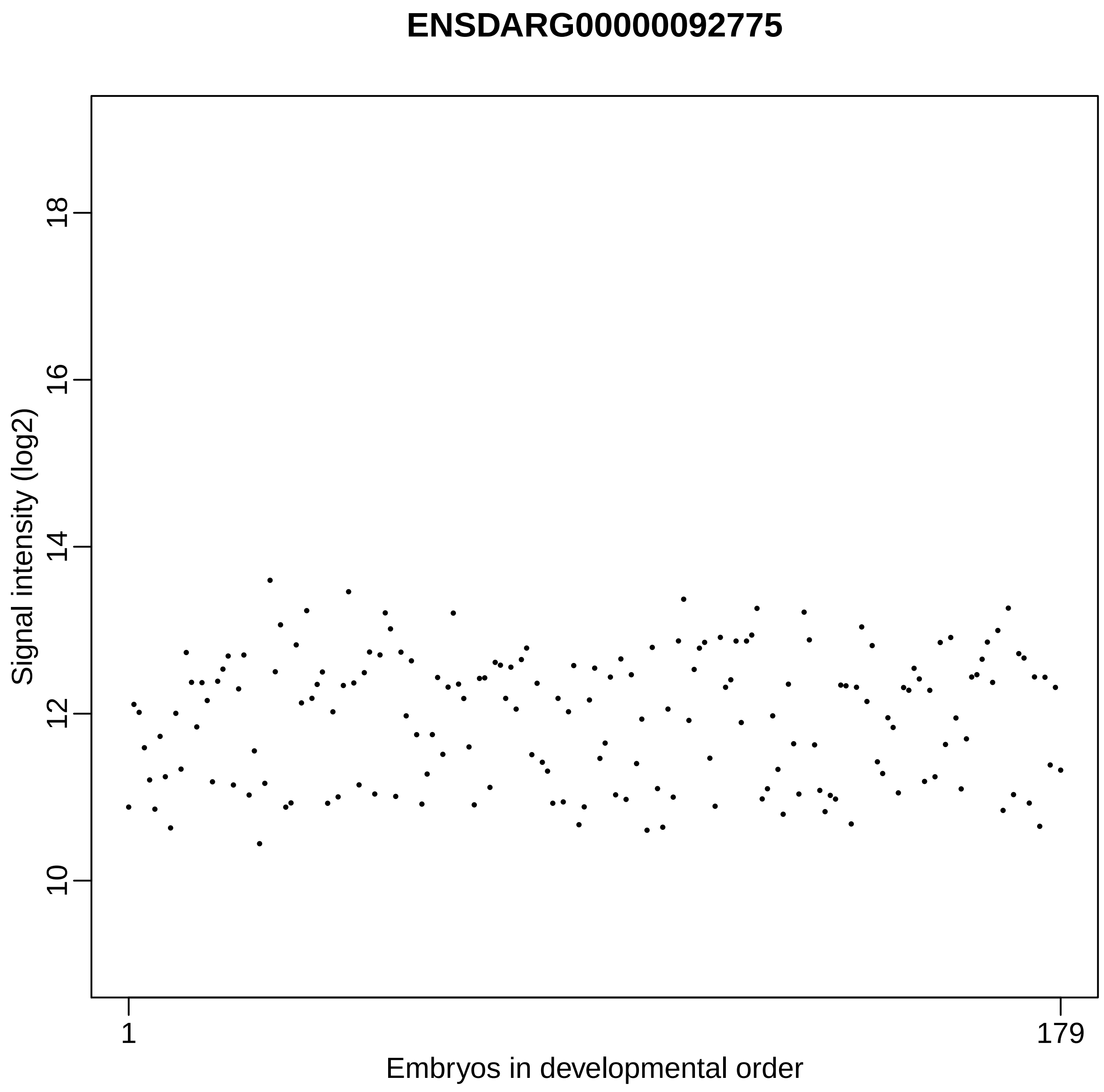
ENSDARG00000092775
Signal intensity (log2)
10
12
14
16
18
1
179
Embryos in developmental order

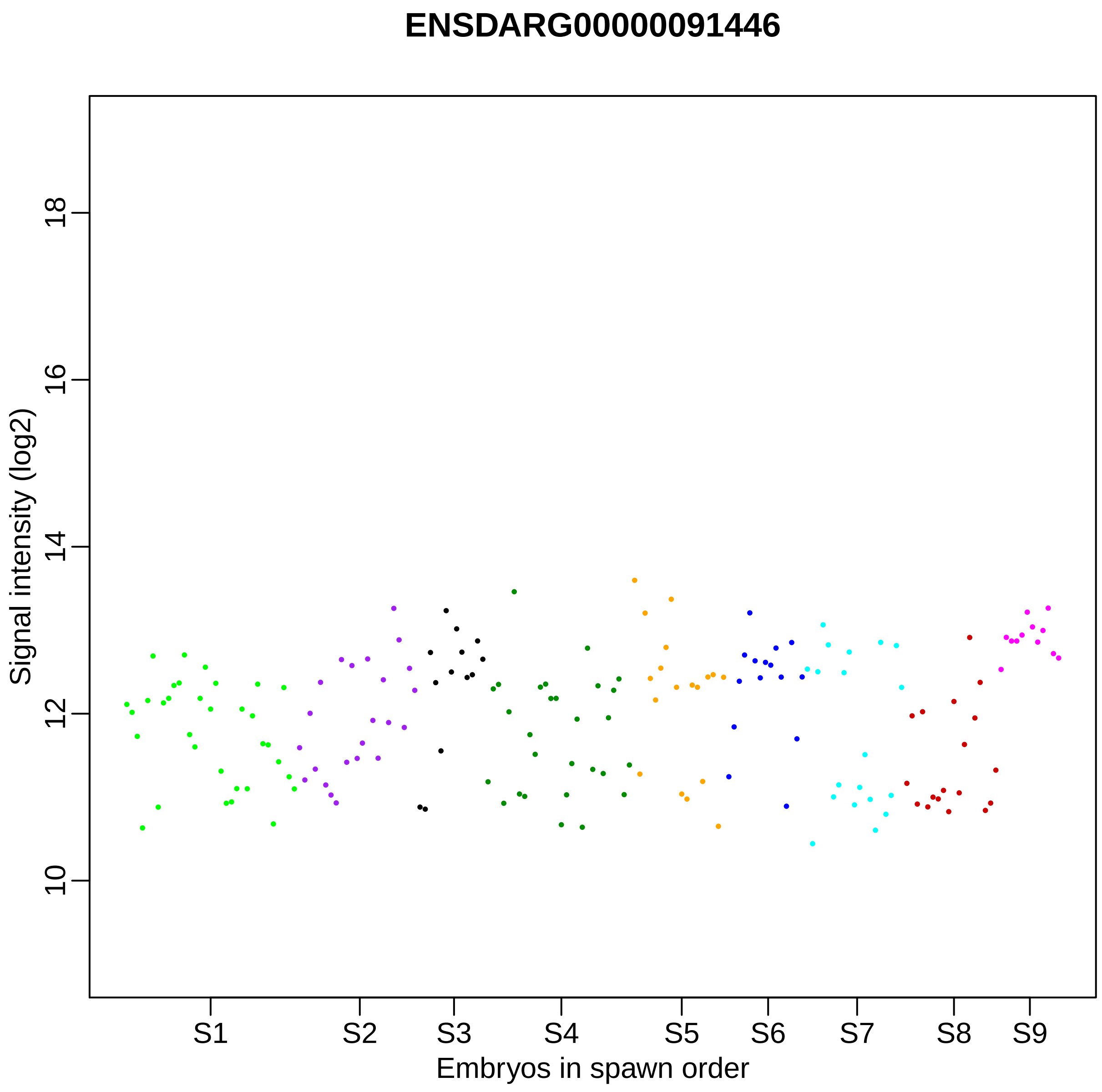
ENSDARG00000091446
Signal intensity (log2)
10
12
14
16
18
S1
S2
S3
S4
S5
S6
S7
S8
S9
Embryos in spawn order

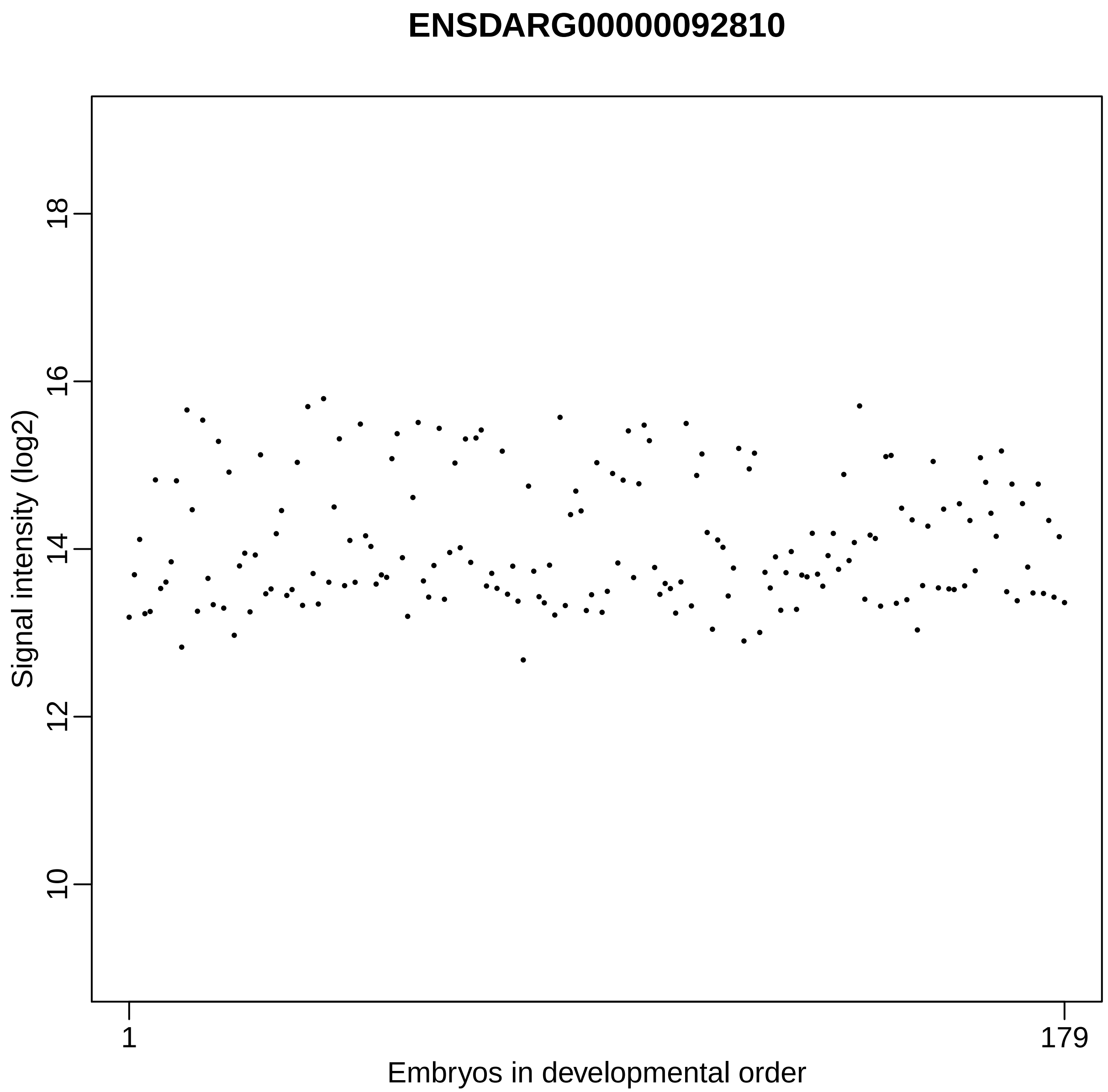
ENSDARG00000092810
Signal intensity (log2)
10
12
14
16
18
1
179
Embryos in developmental order

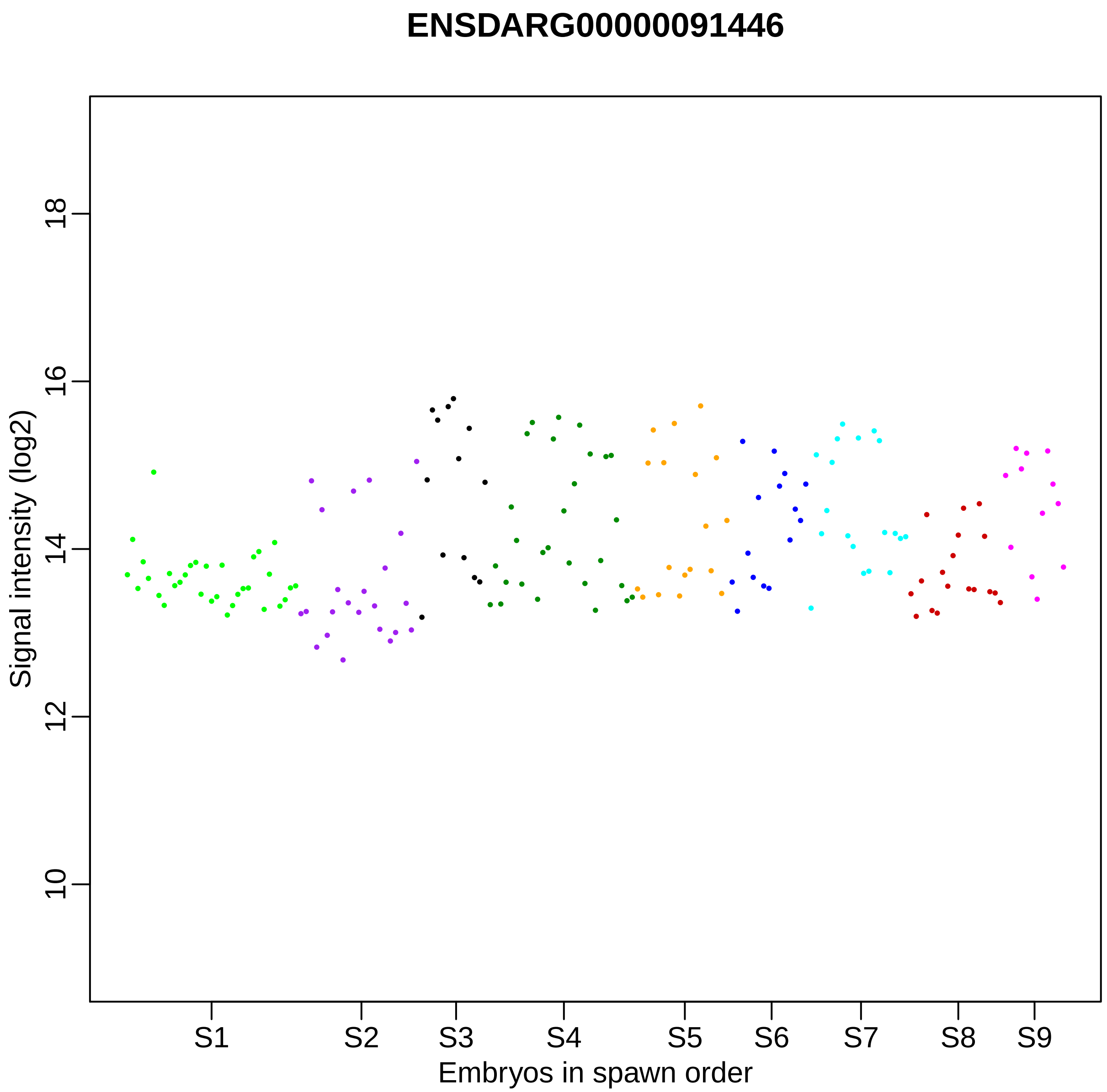
ENSDARG00000091446
Signal intensity (log2)
10
12
14
16
18
S1
S2
S3
S4
S5
S6
S7
S8
S9
Embryos in spawn order

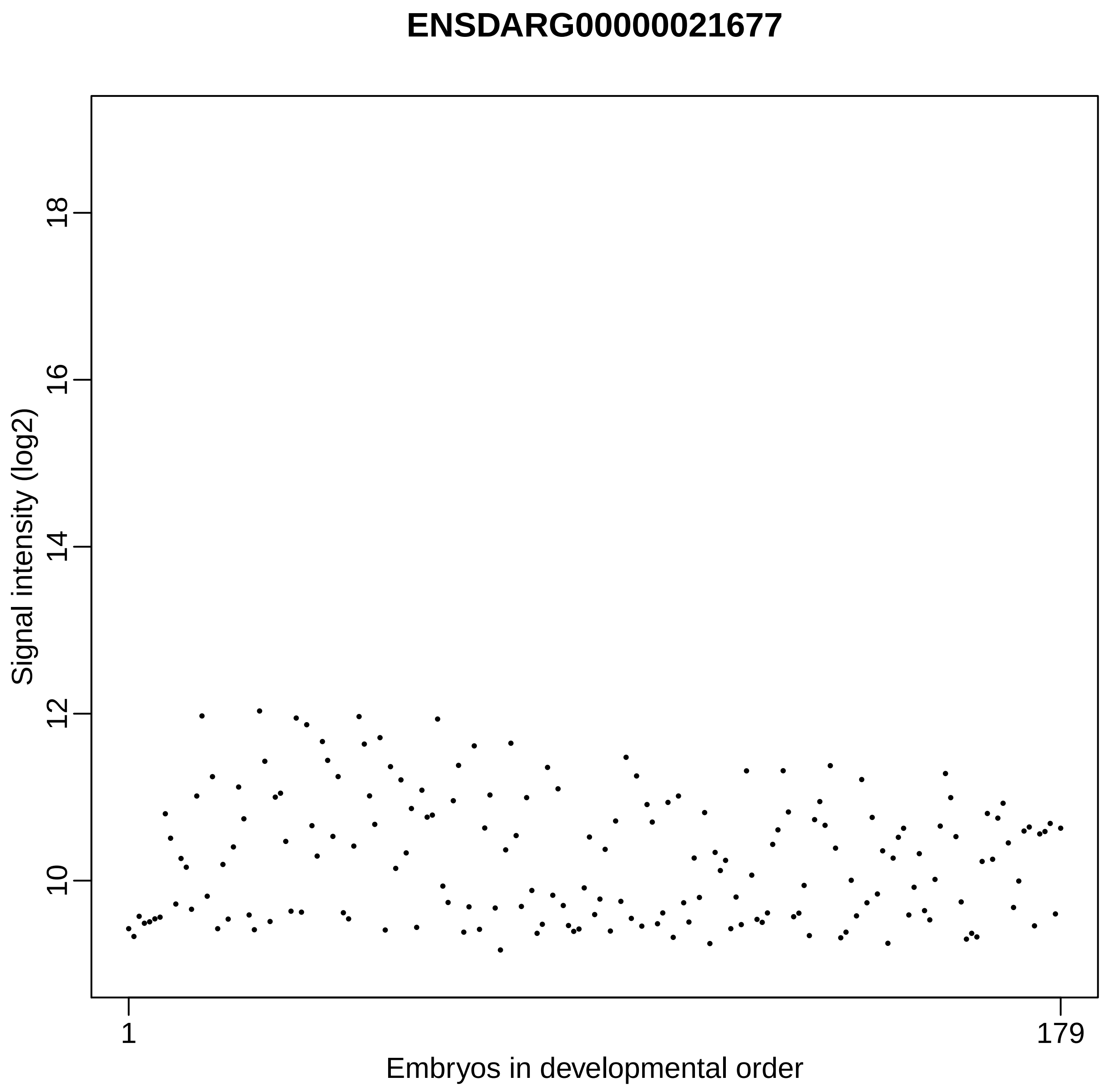
ENSDARG00000021677
Signal intensity (log2)
10
12
14
16
18
1
179
Embryos in developmental order

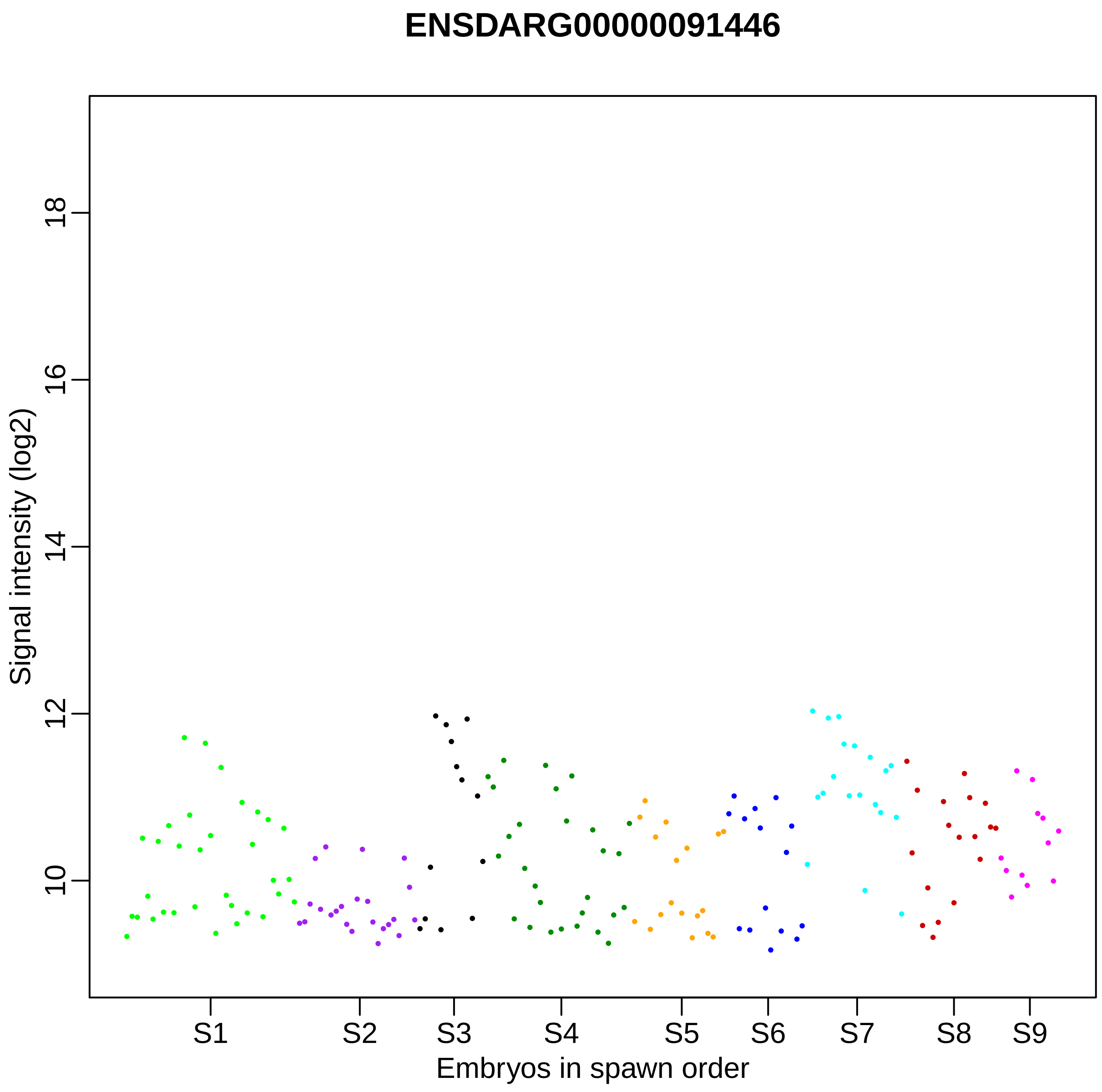
ENSDARG00000091446
Signal intensity (log2)
10
12
14
16
18
S1
S2
S3
S4
S5
S6
S7
S8
S9
Embryos in spawn order

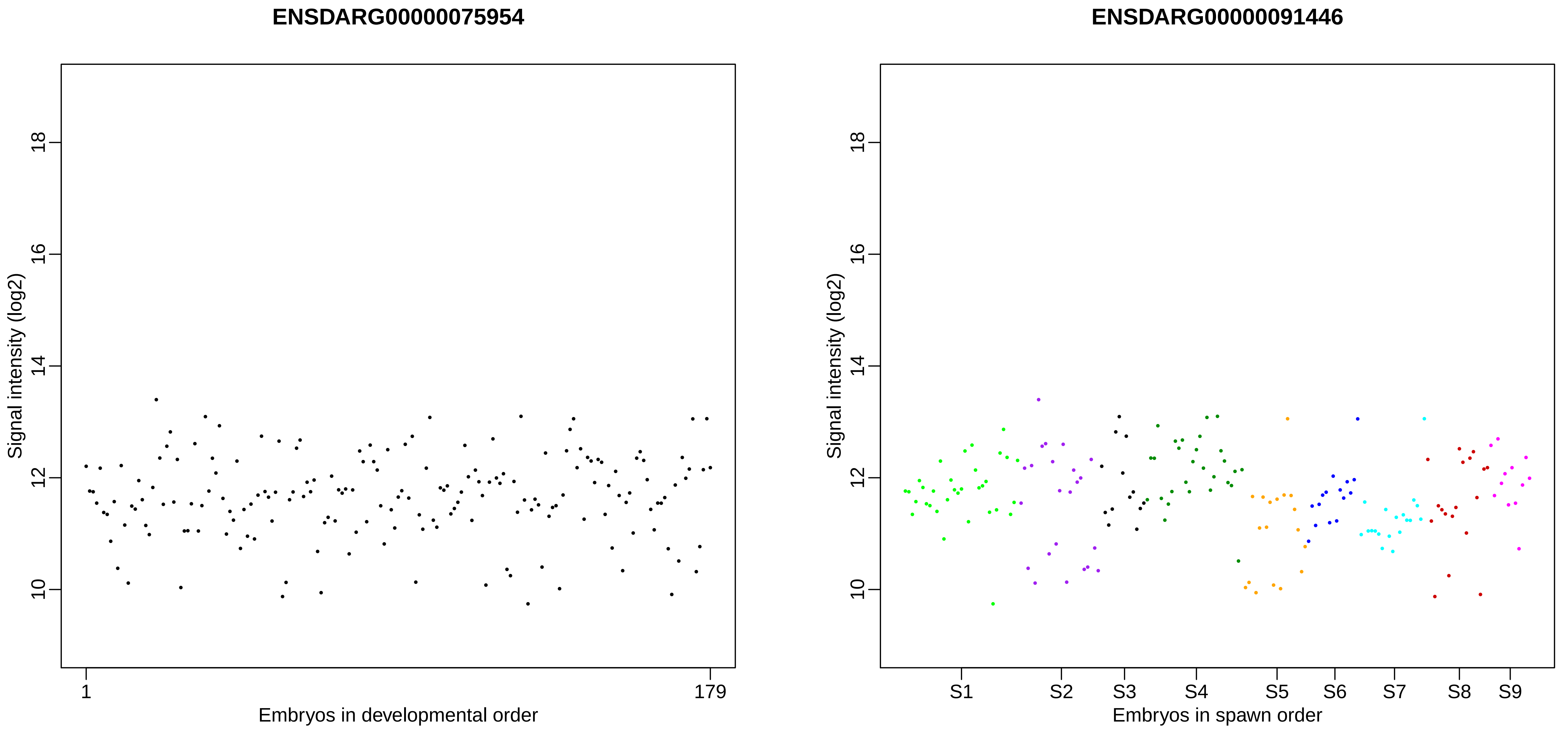
ENSDARG00000075954
Signal intensity (log2)
10
12
14
16
18
1
179
Embryos in developmental order
ENSDARG00000091446
Signal intensity (log2)
10
12
14
16
18
S1
S2
S3
S4
S5
S6
S7
S8
S9
Embryos in spawn order

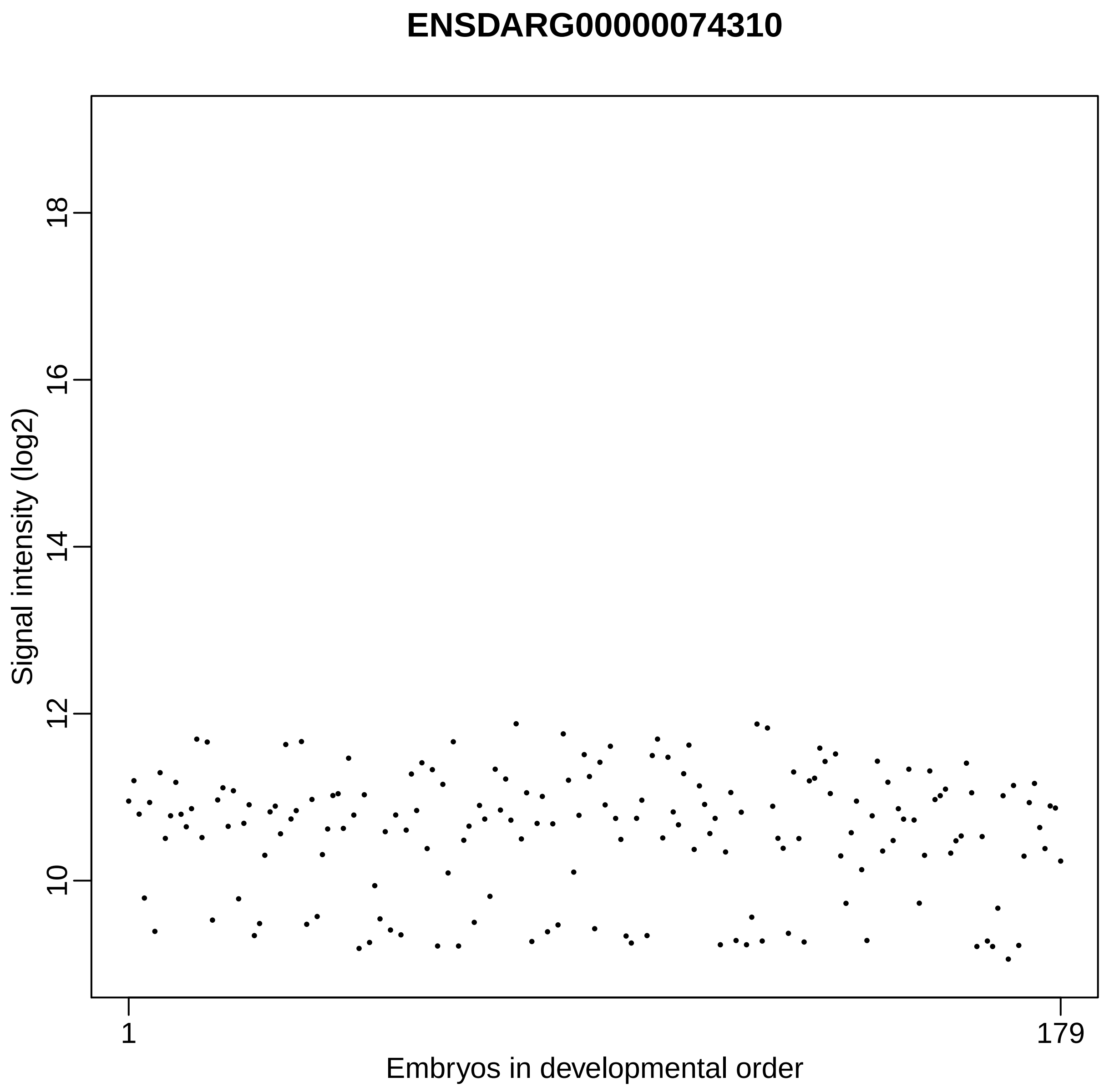
ENSDARG00000074310
Signal intensity (log2)
10
12
14
16
18
1
179
Embryos in developmental order

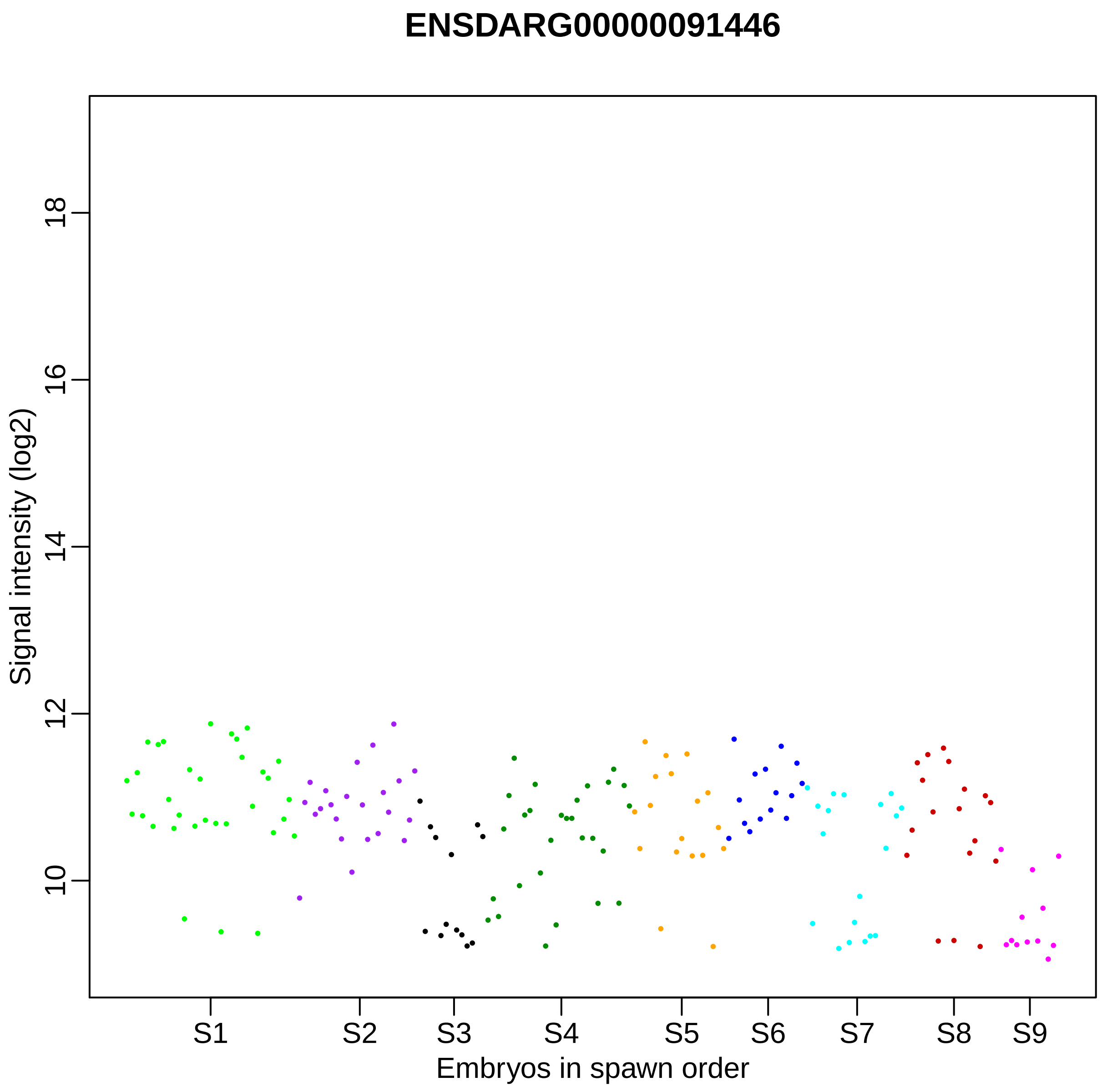
ENSDARG00000091446
Signal intensity (log2)
10
12
14
16
18
S1
S2
S3
S4
S5
S6
S7
S8
S9
Embryos in spawn order

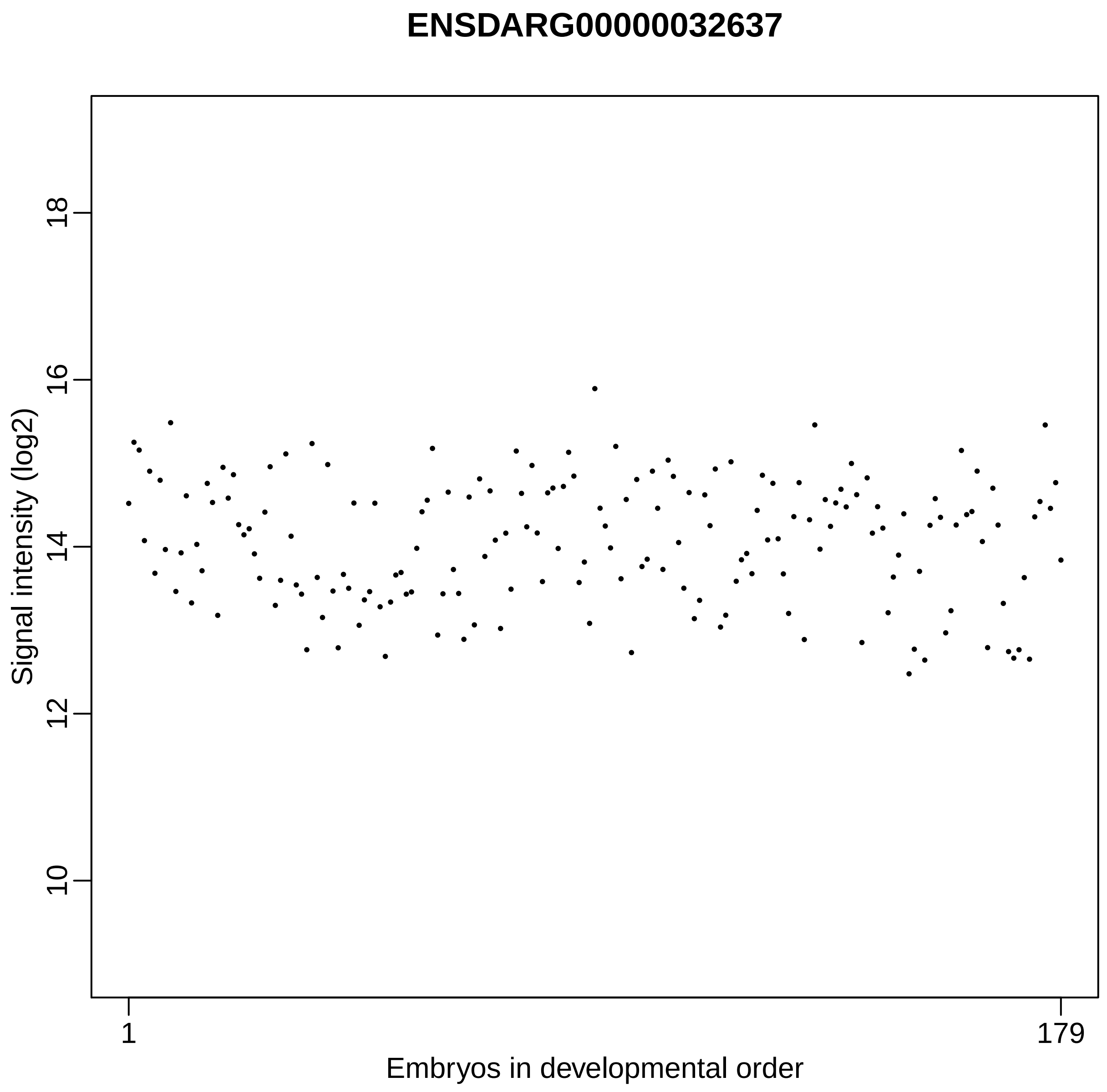

ENSDARG00000032637
Signal intensity (log2)
10
12
14
16
18
1
179
Embryos in developmental order

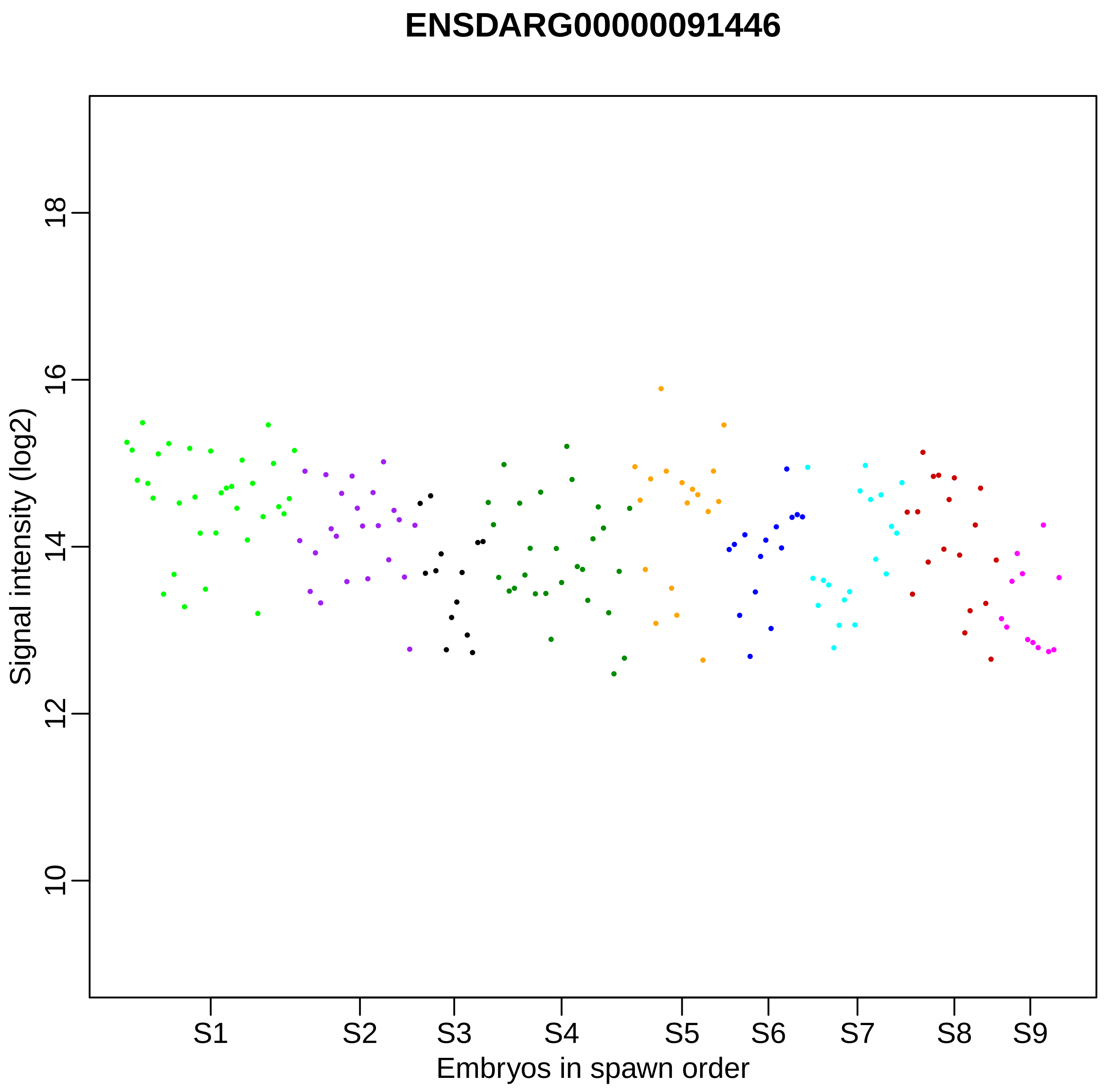

ENSDARG00000091446
Signal intensity (log2)
10
12
14
16
18
S1
S2
S3
S4
S5
S6
S7
S8
S9
Embryos in spawn order

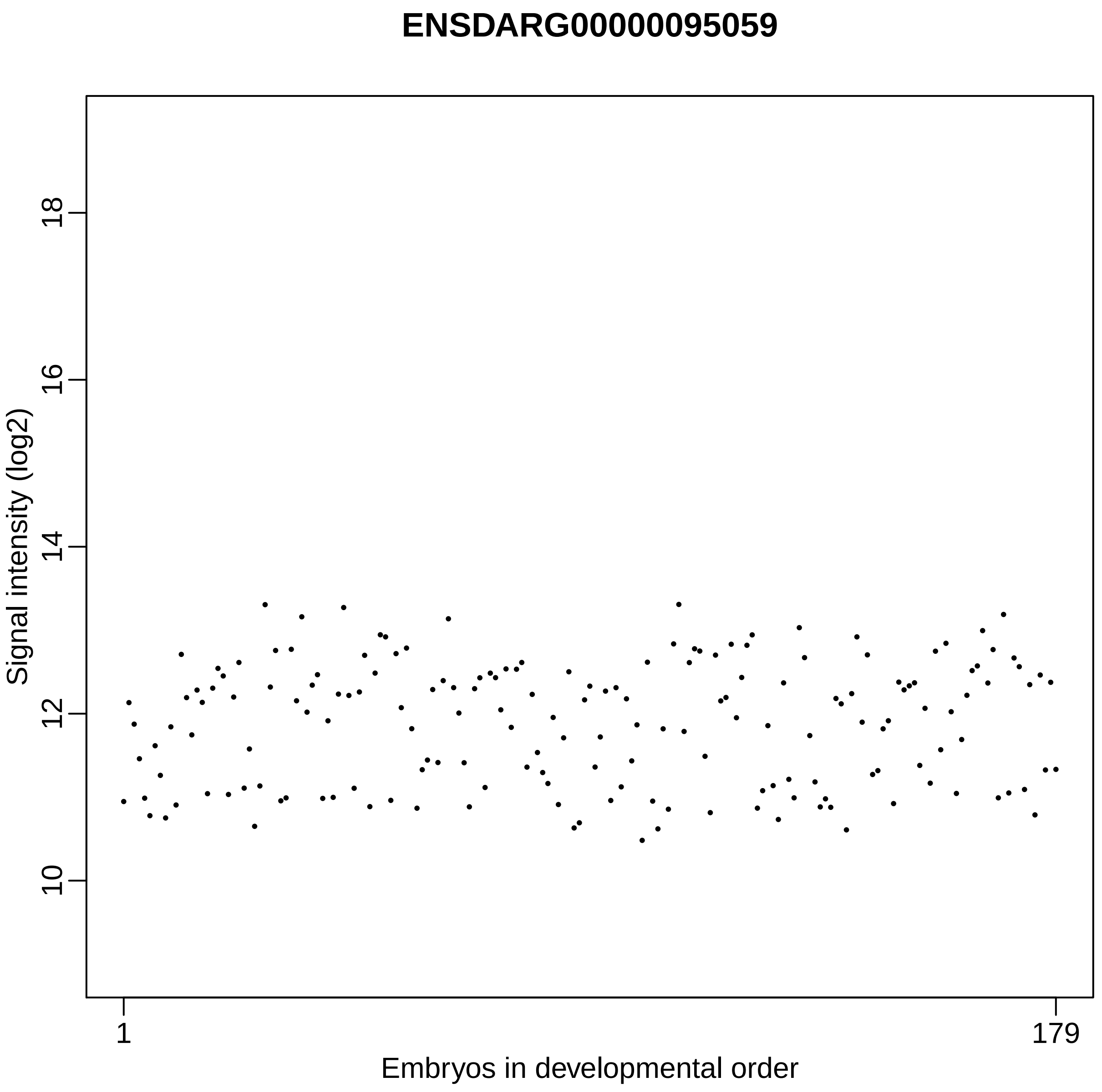
ENSDARG00000095059
Signal intensity (log2)
10
12
14
16
18
1
179
Embryos in developmental order

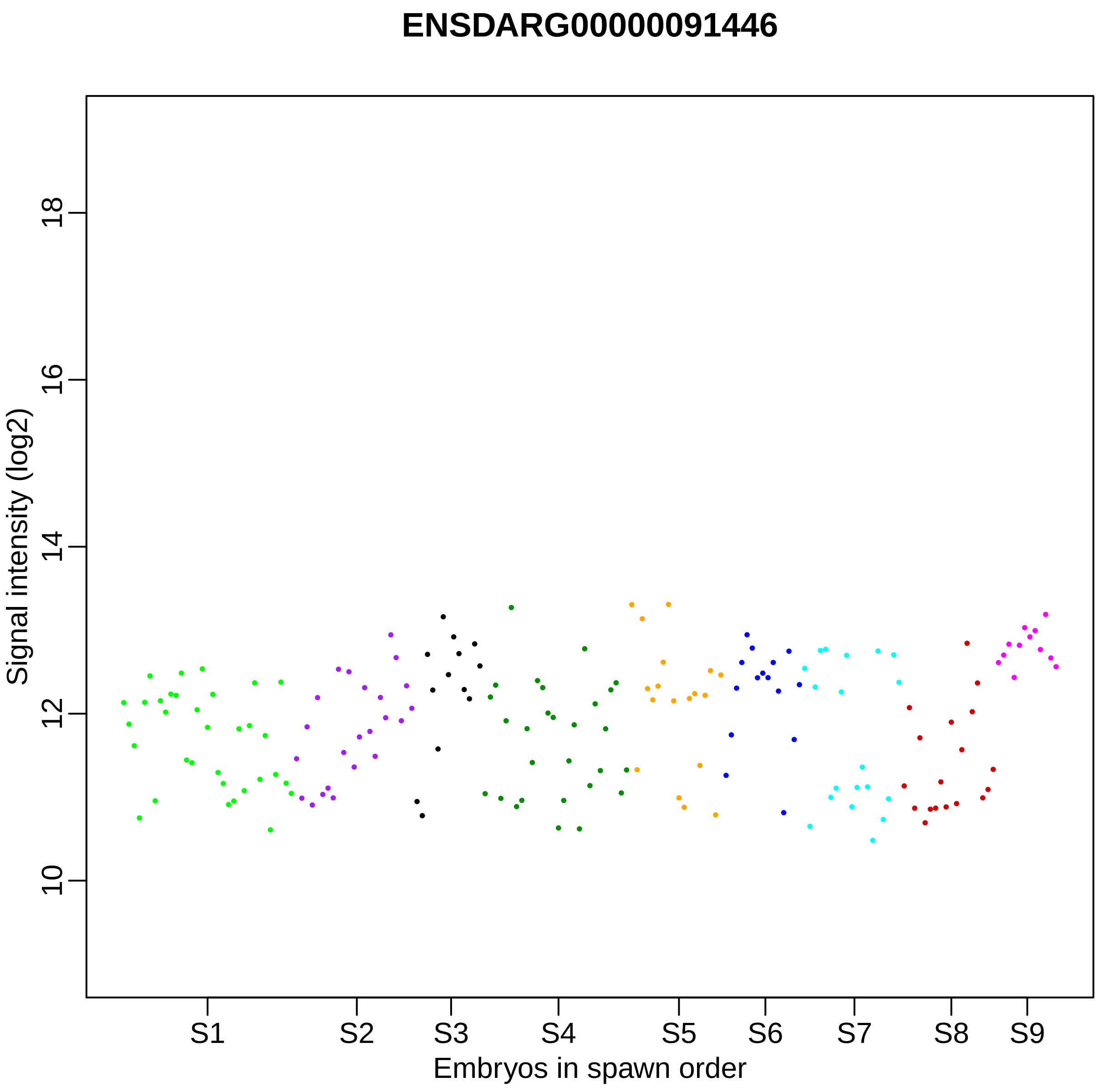
ENSDARG00000091446
Signal intensity (log2)
10
12
14
16
18
S1
S2
S3
S4
S5
S6
S7
S8
S9
Embryos in spawn order

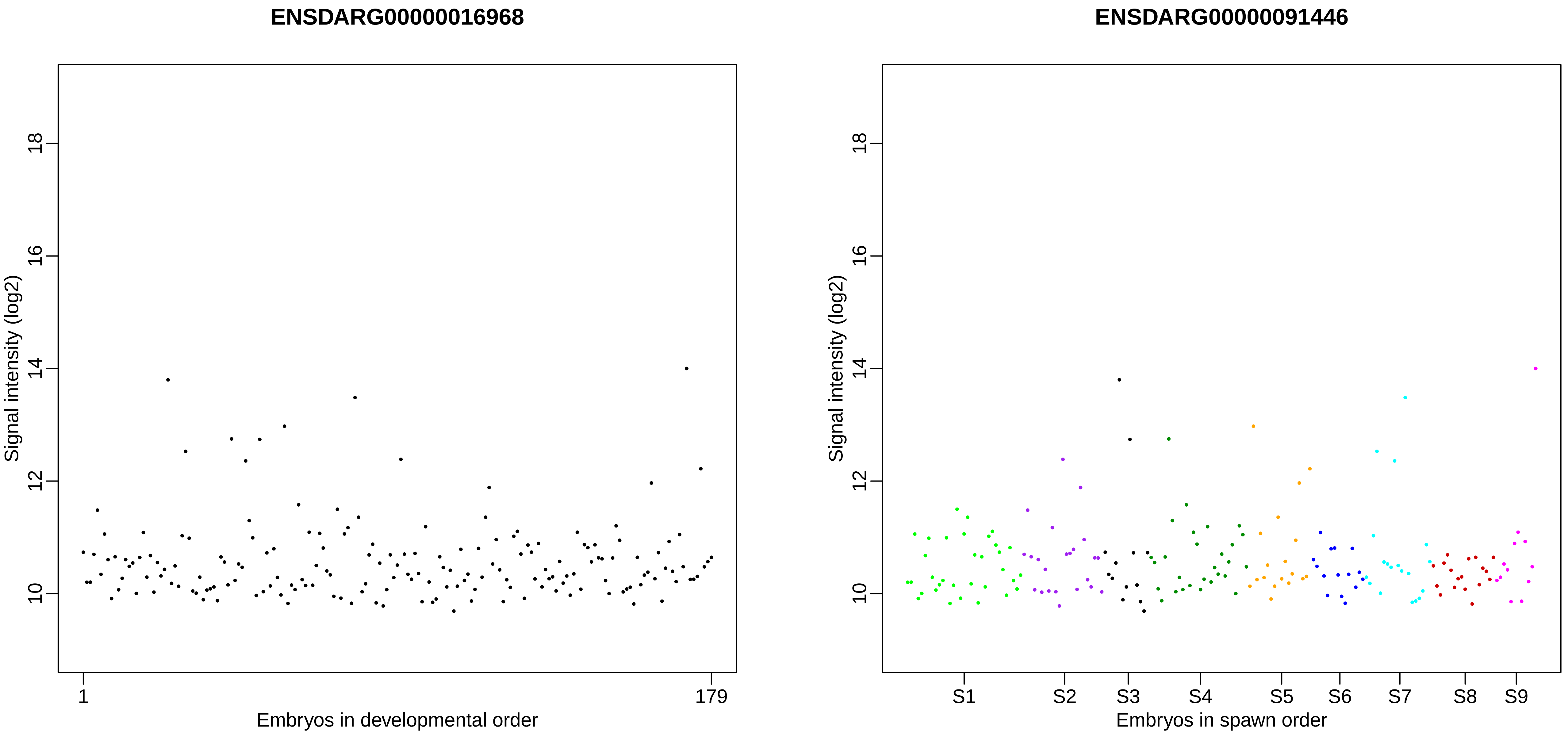

ENSDARG00000016968
Signal intensity (log2)
10
12
14
16
18
1
179
Embryos in developmental order
ENSDARG00000091446
Signal intensity (log2)
10
12
14
16
18
S1
S2
S3
S4
S5
S6
S7
S8
S9
Embryos in spawn order

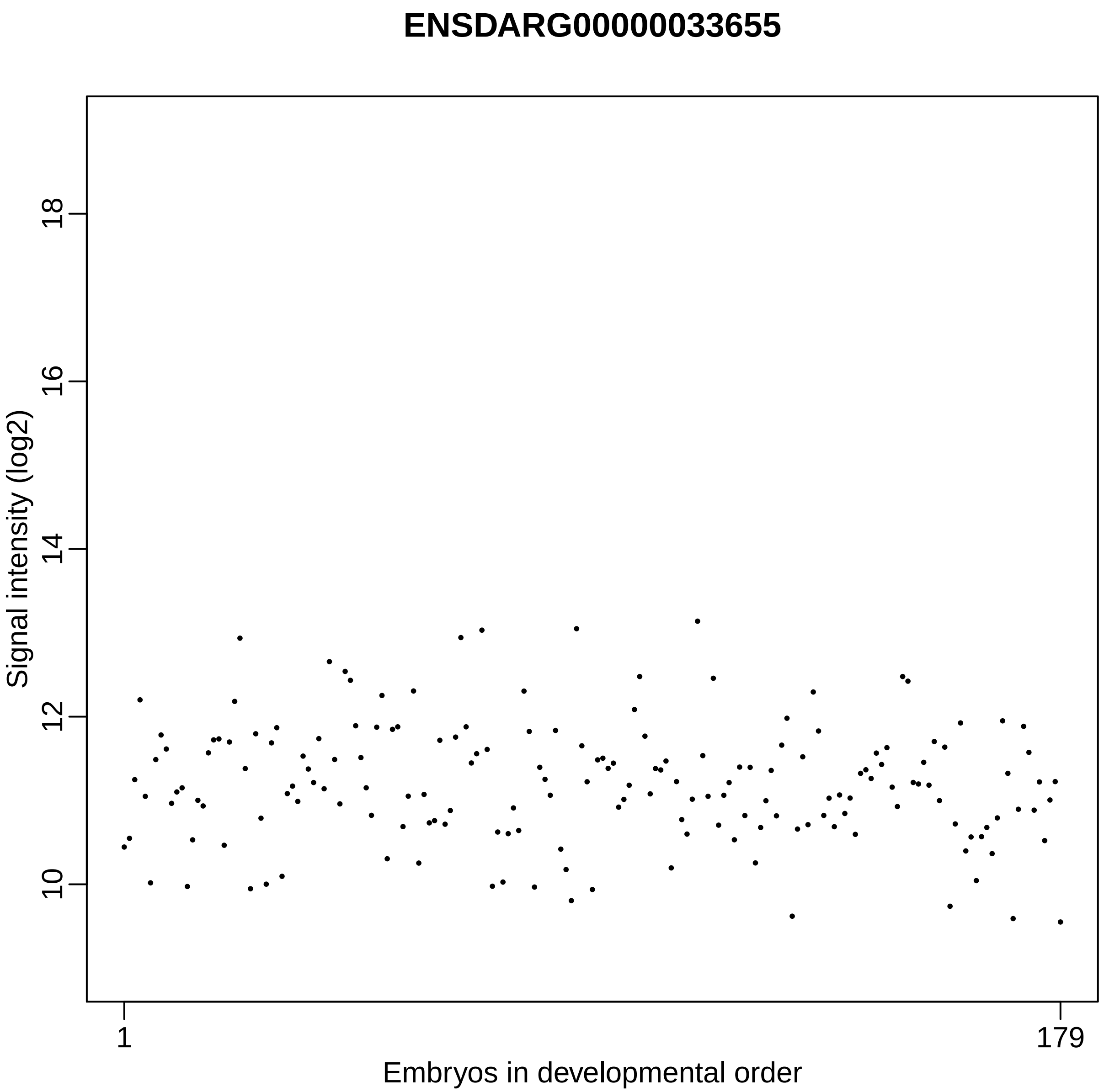
ENSDARG00000033655
Signal intensity (log2)
10
12
14
16
18
1
179
Embryos in developmental order

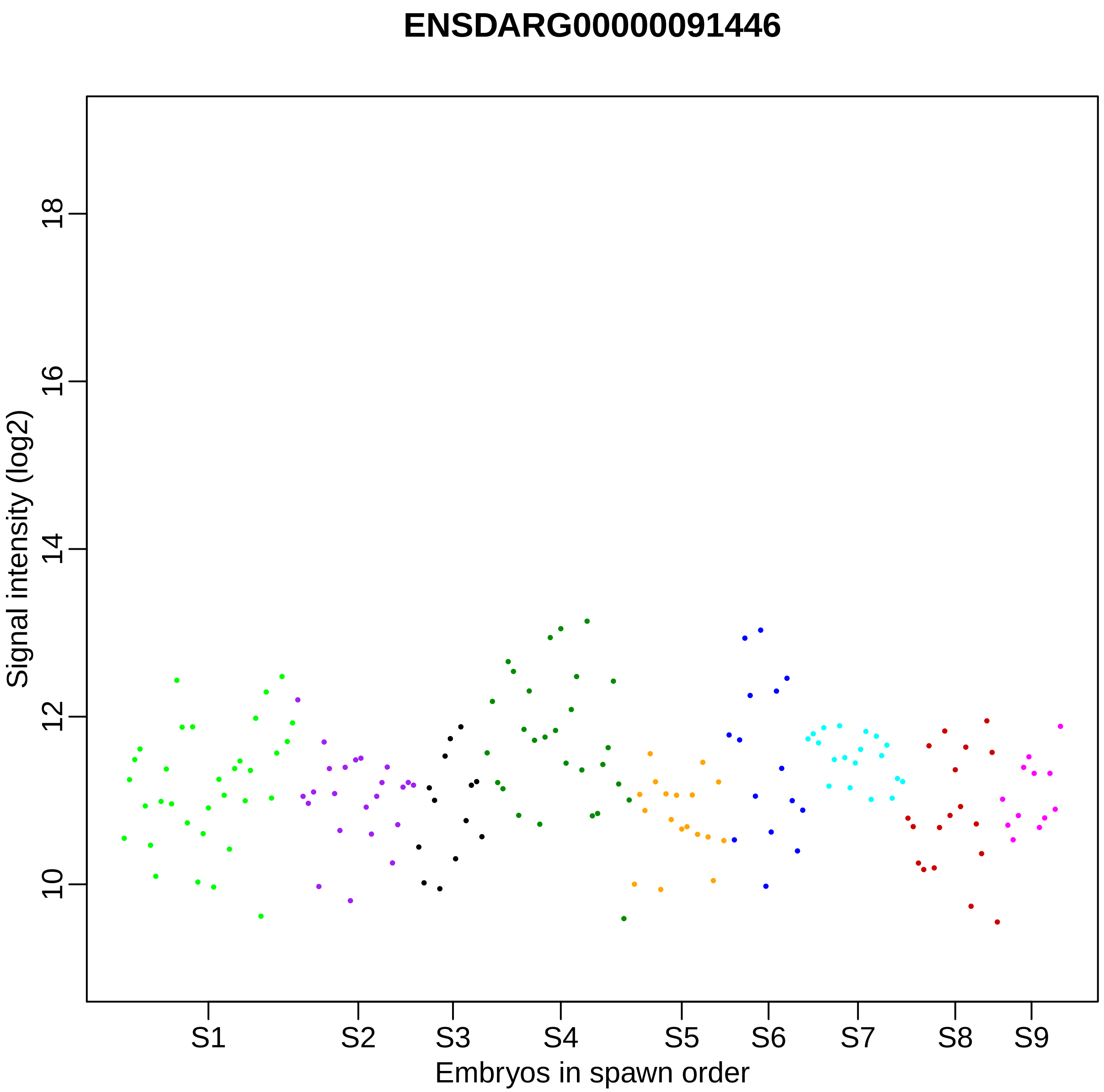
ENSDARG00000091446
Signal intensity (log2)
10
12
14
16
18
S1
S2
S3
S4
S5
S6
S7
S8
S9
Embryos in spawn order

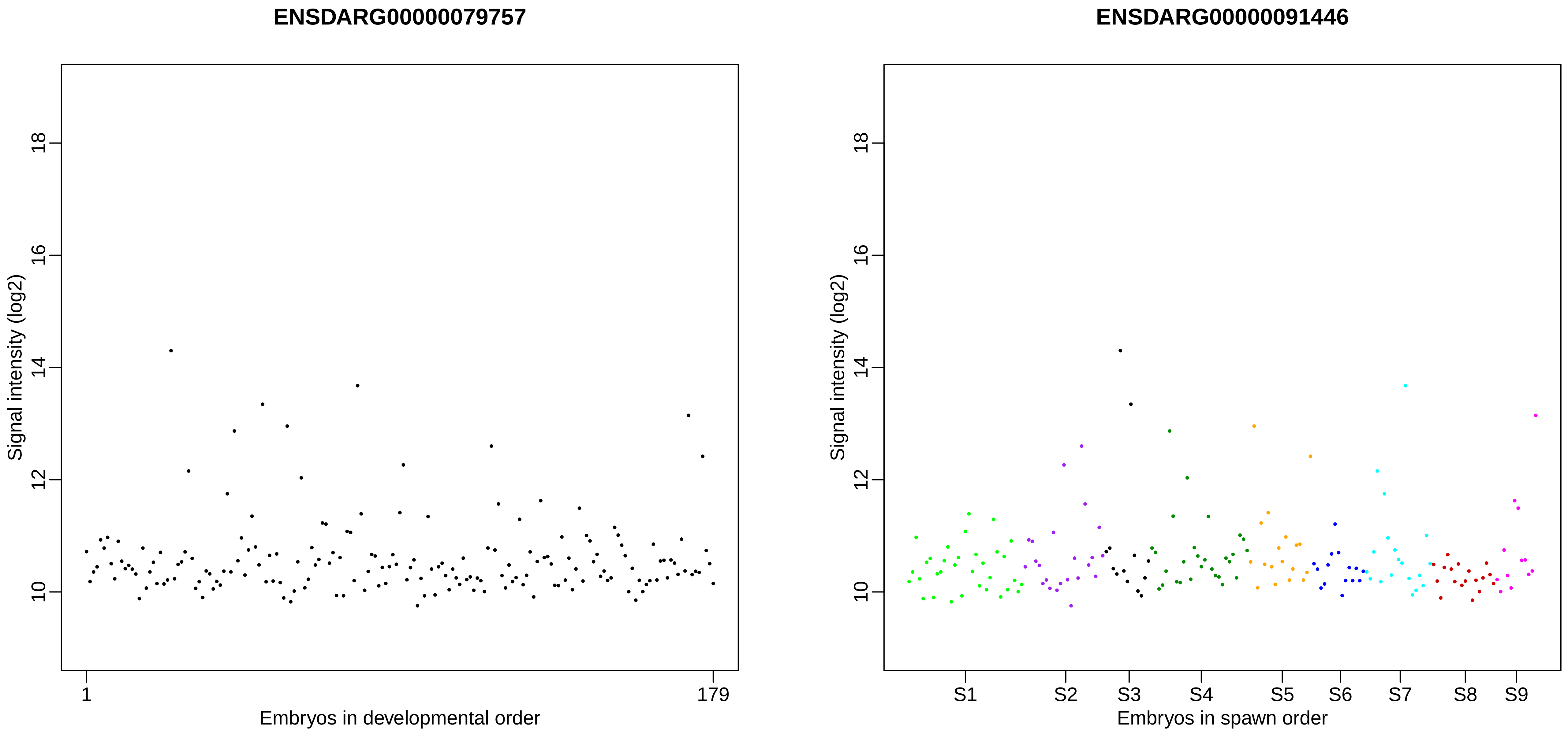

ENSDARG00000079757
Signal intensity (log2)
10
12
14
16
18
1
179
Embryos in developmental order
ENSDARG00000091446
Signal intensity (log2)
10
12
14
16
18
S1
S2
S3
S4
S5
S6
S7
S8
S9
Embryos in spawn order
