# Supplementary material for: Transcriptome dynamics in early zebrafish embryogenesis determined by high-resolution time course analysis of 180 successive, individual zebrafish embryos
Source: BMC Genomics. 2017 Apr 11;18:287. doi: 10.1186/s12864-017-3672-z (PMC5387192; doi:10.1186/s12864-017-3672-z)

Comparison of this study to other studies

Tomography (study T)

A

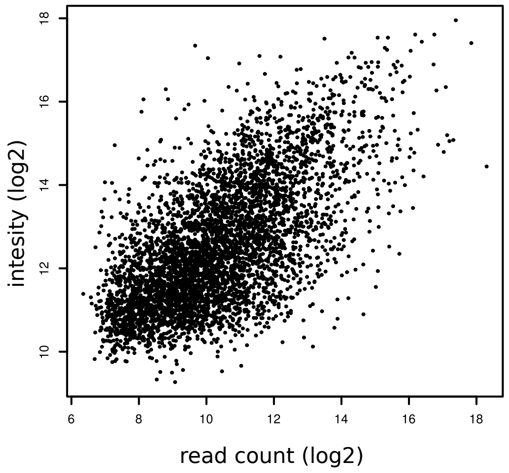

B

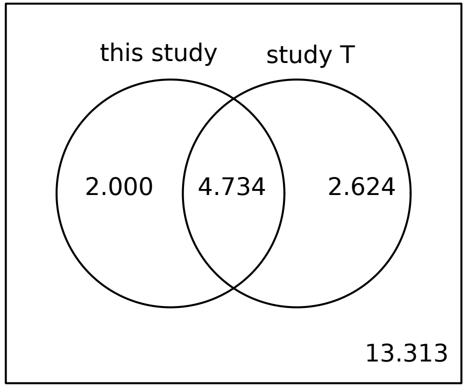

Early embryonic transcriptome (study EET)

C

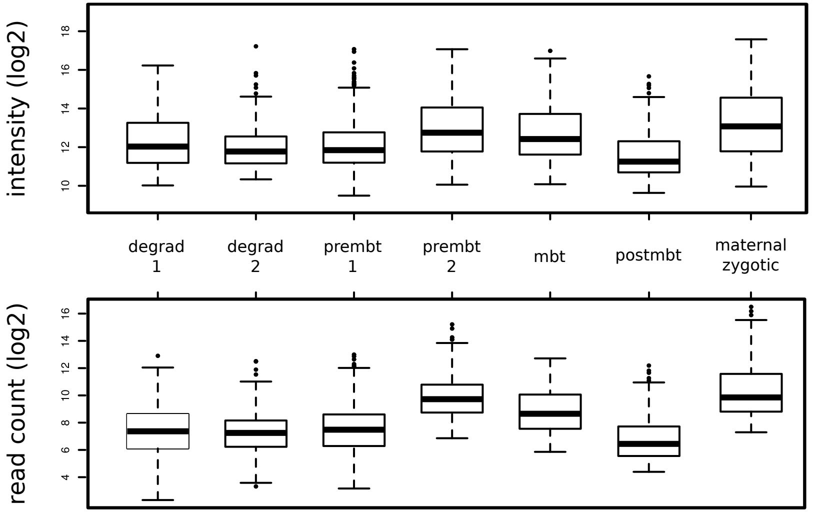

D

	Degrad 1	Degrad 2	Prembt 1	Prembt 2	Mbt	Postmbt	Maternal zygotic
# tot reads of study EET	555	359	2.341	698	164	649	440
# overlap with this study	286	139	1.072	574	113	275	355
% on study EET category	51%	39%	46%	82%	69%	42%	81%
% on tot of this study	4%	2%	16%	9%	2%	4%	5%

Individual egg (study IE)

E

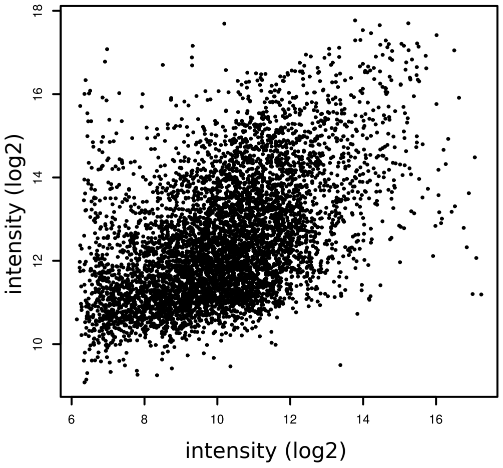

F

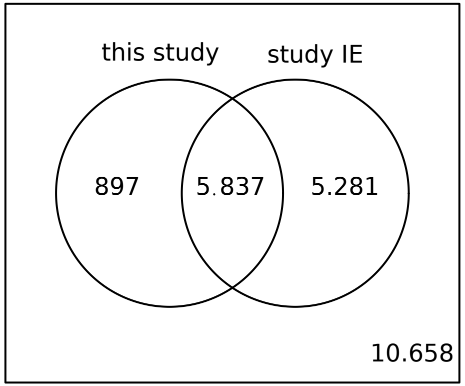

Supplement: Supplementary file 15 — Comparison of this study with the zebrafish tomography study of [30] (study T), with the study on the early embryonic zebrafish transcriptome of [7] (study EET) and with the study on individual zebrafish eggs from five different mothers of [23] (study IE). A) Scatter plot of log2 read counts in the shield stage of study T vs. the log2 expression intensity in this study of genes that are expressed in both studies. Read counts are calculated as the log2 of the sum of counts in all slices in study T and from this study the samples 85 to 95 in the developmental order are taken. The correlation between the two sets is 0.64. B) Venn diagram on Ensembl genes in study T and expressed Ensembl genes in this study. C) The distributions of log2 intensity values of Ensembl genes in this study (upper panel) that are per category in common with the Ensembl genes from study EET; in the lower panel for the same genes the distribution of read counts in the 7 categories of expression clusters in study EET are displayed. D) Per category comparison of number of expressed genes in this study and study EET. E) Scatter plot of log2 expression intensity in study IE (X-axis) and this study (Y-axis) of genes that are expressed in both studies. For this from study IE the median of all 24 eggs was taken and from this study the median of the first five samples was taken. The correlation between the two sets is 0.50. F) Venn diagram on study IE this study. Compared are expressed Ensembl genes from both studies. (PDF 377 kb) [file 12864_2017_3672_MOESM15_ESM.pdf]
