# Supplementary material for: Transcriptome dynamics in early zebrafish embryogenesis determined by high-resolution time course analysis of 180 successive, individual zebrafish embryos
Source: BMC Genomics. 2017 Apr 11;18:287. doi: 10.1186/s12864-017-3672-z (PMC5387192; doi:10.1186/s12864-017-3672-z)
Supplement: Supplementary file 16 — Temporal and spatial behavior of genes in 27 KEGG pathways. Each page displays three plots. The left plot shows the temporal expression in log2 intensity in our time course experiment for a pathway ordered in developmental time. The middle plot shows the spatial expression pattern in the tomography set [30] in log2 read counts. The three peaks represent the three axes in the embryo: respectively the animal-vegetal, left-right and ventral-dorsal axes. The right plot shows the number of genes of the pathway that belong to a spatial expression cluster. The spatial expression cluster 9 is the only spatial expression cluster in which an overrepresentation was found (KEGG pathway “Ribosome”). (PDF 1595 kb) [file 12864_2017_3672_MOESM16_ESM.pdf]

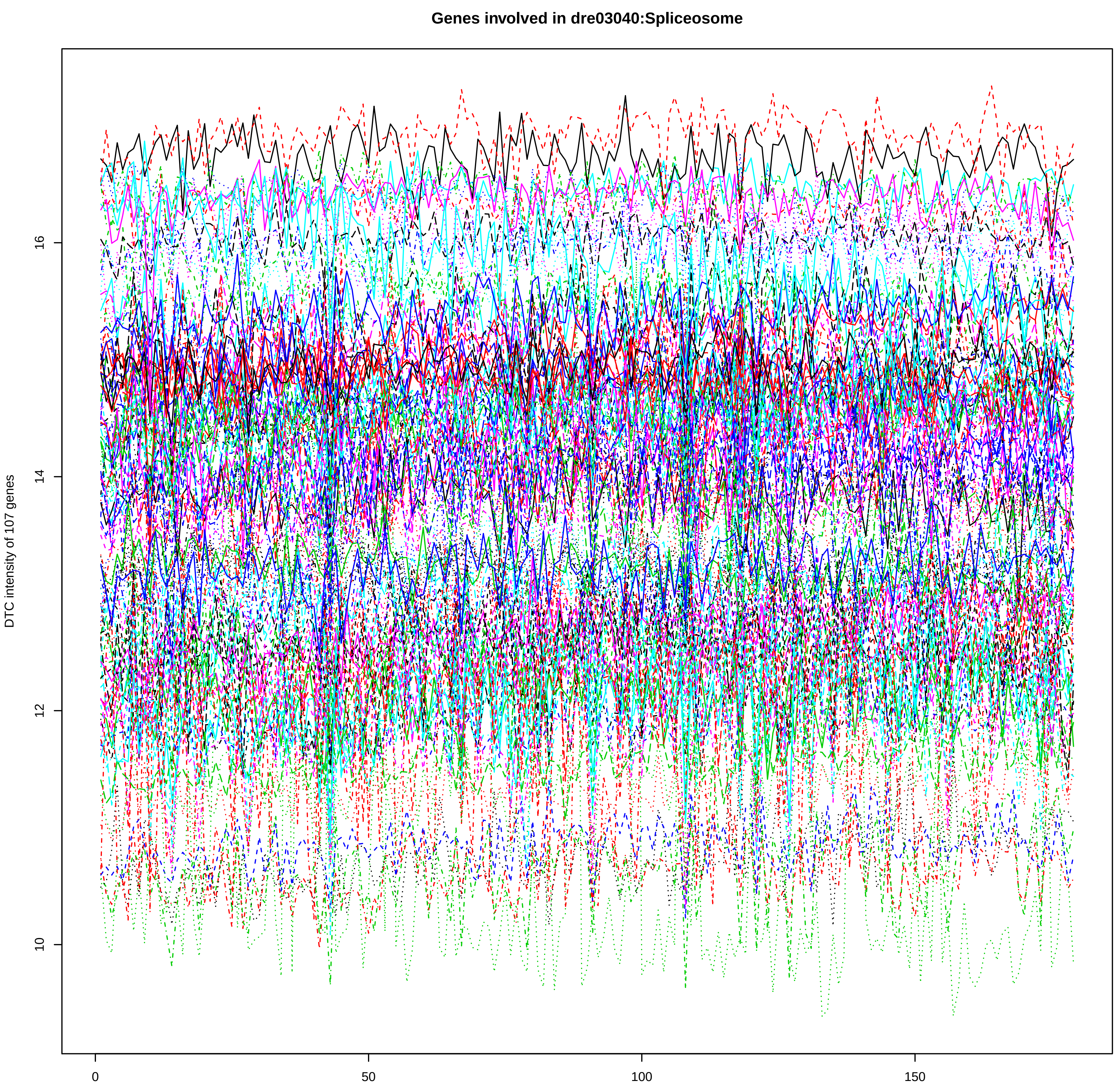
Genes involved in dre03040:Spliceosome
DTC intensity of 107 genes
10
12
14
16
0
50
100
150

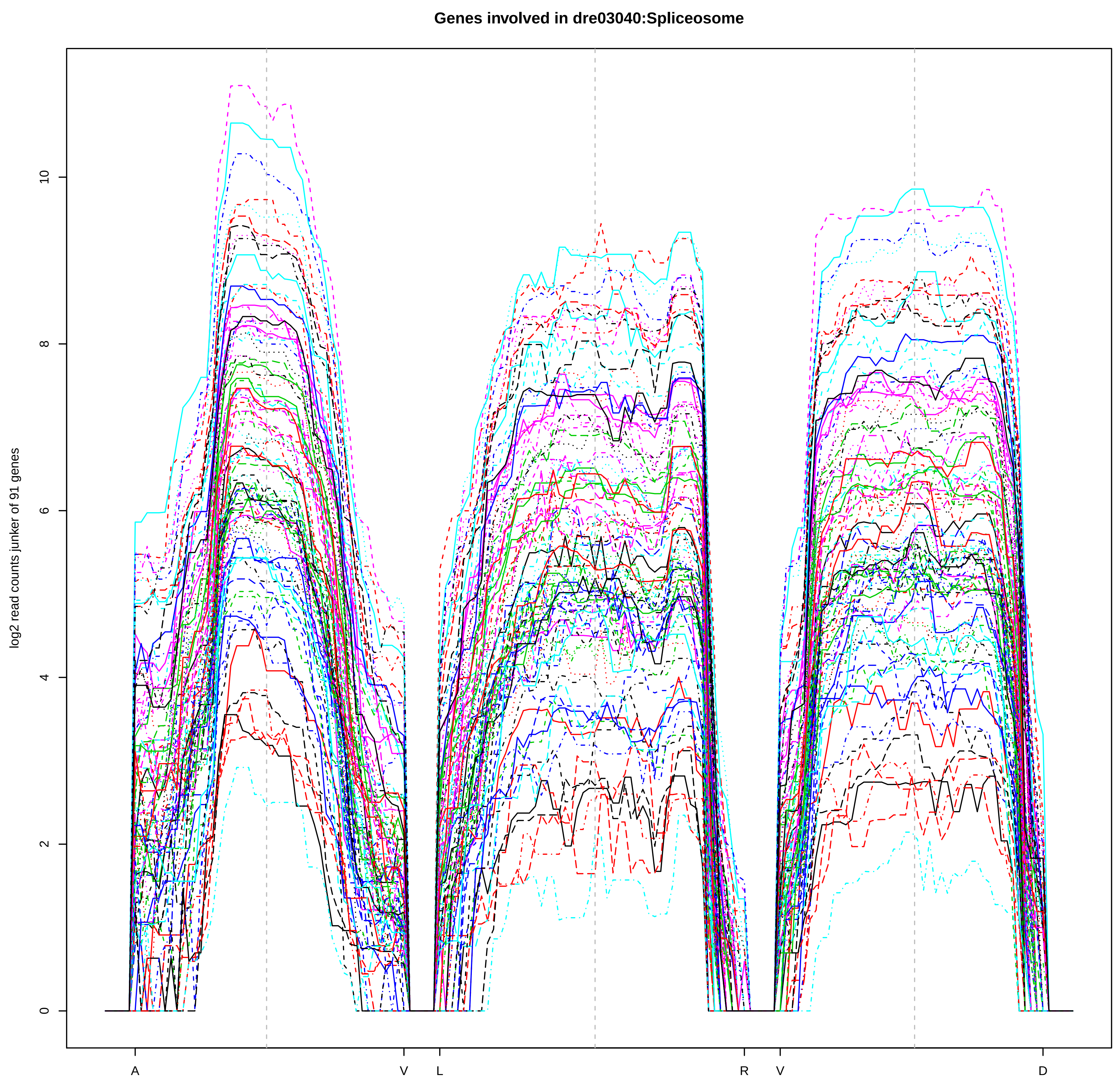
Genes involved in dre03040:Spliceosome
log2 read counts junker of 91 genes
0
2
4
6
8
10
A
V
L
R
V
D

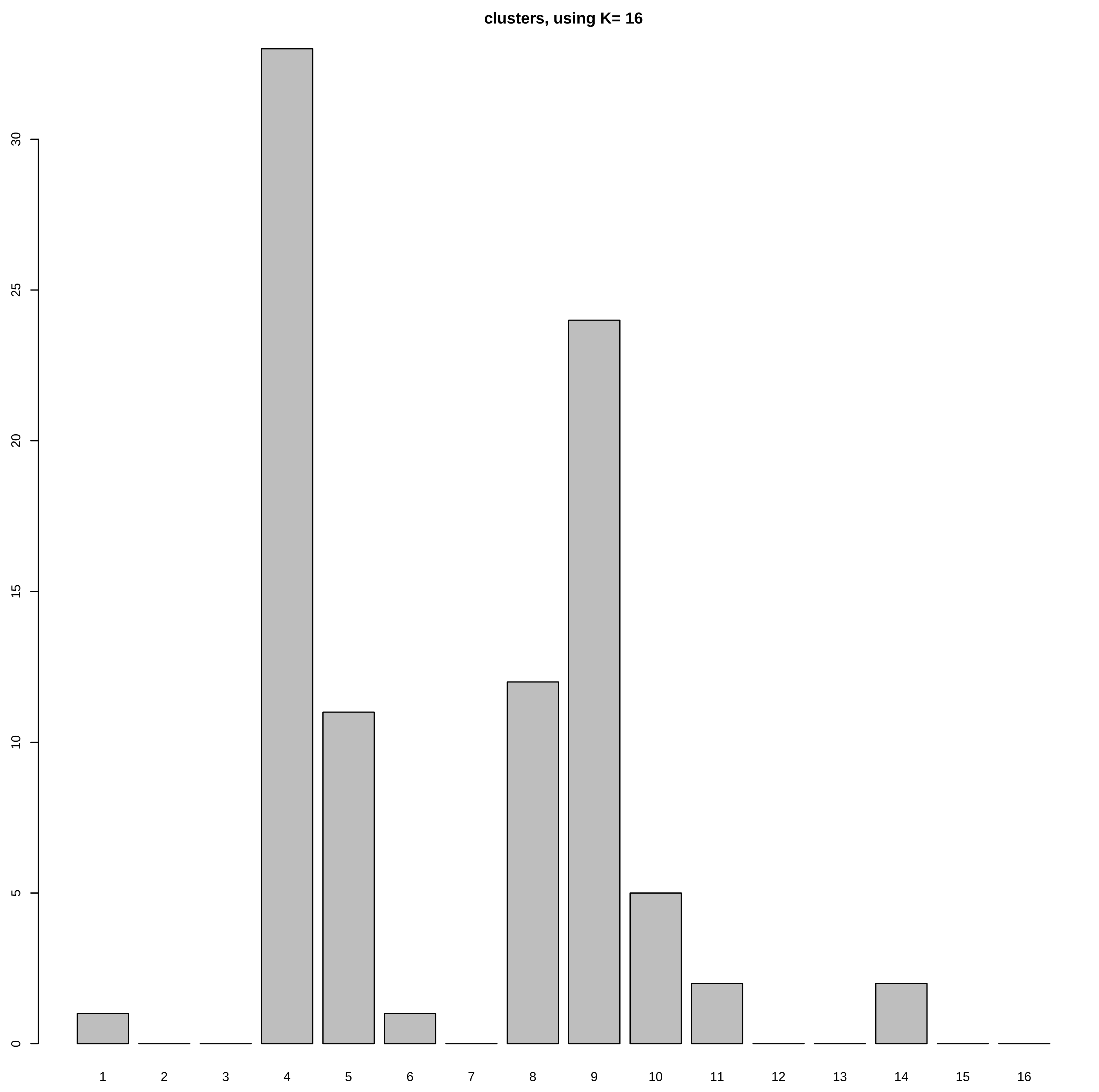
clusters, using K= 16
0
5
10
15
20
25
30
1
2
3
4
5
6
7
8
9
10
11
12
13
14
15
16

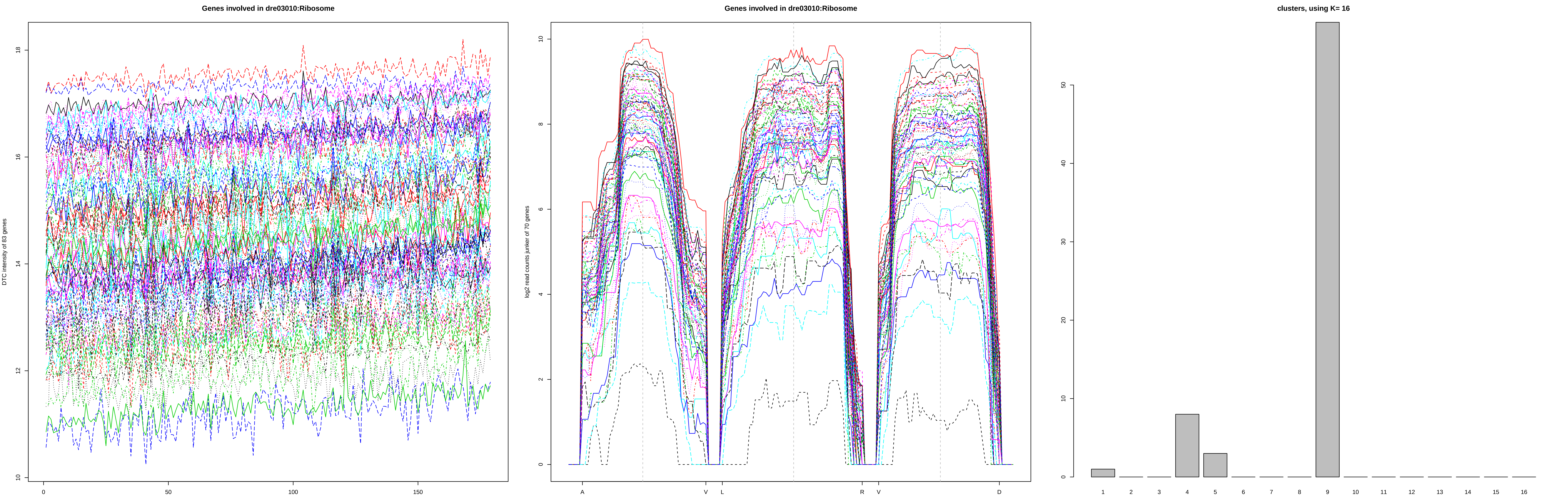

Genes involved in dre03010:Ribosome
DTC intensity of 83 genes
Genes involved in dre03010:Ribosome
log2 read counts junker of 70 genes
A
V
L
R
V
D
clusters, using K= 16

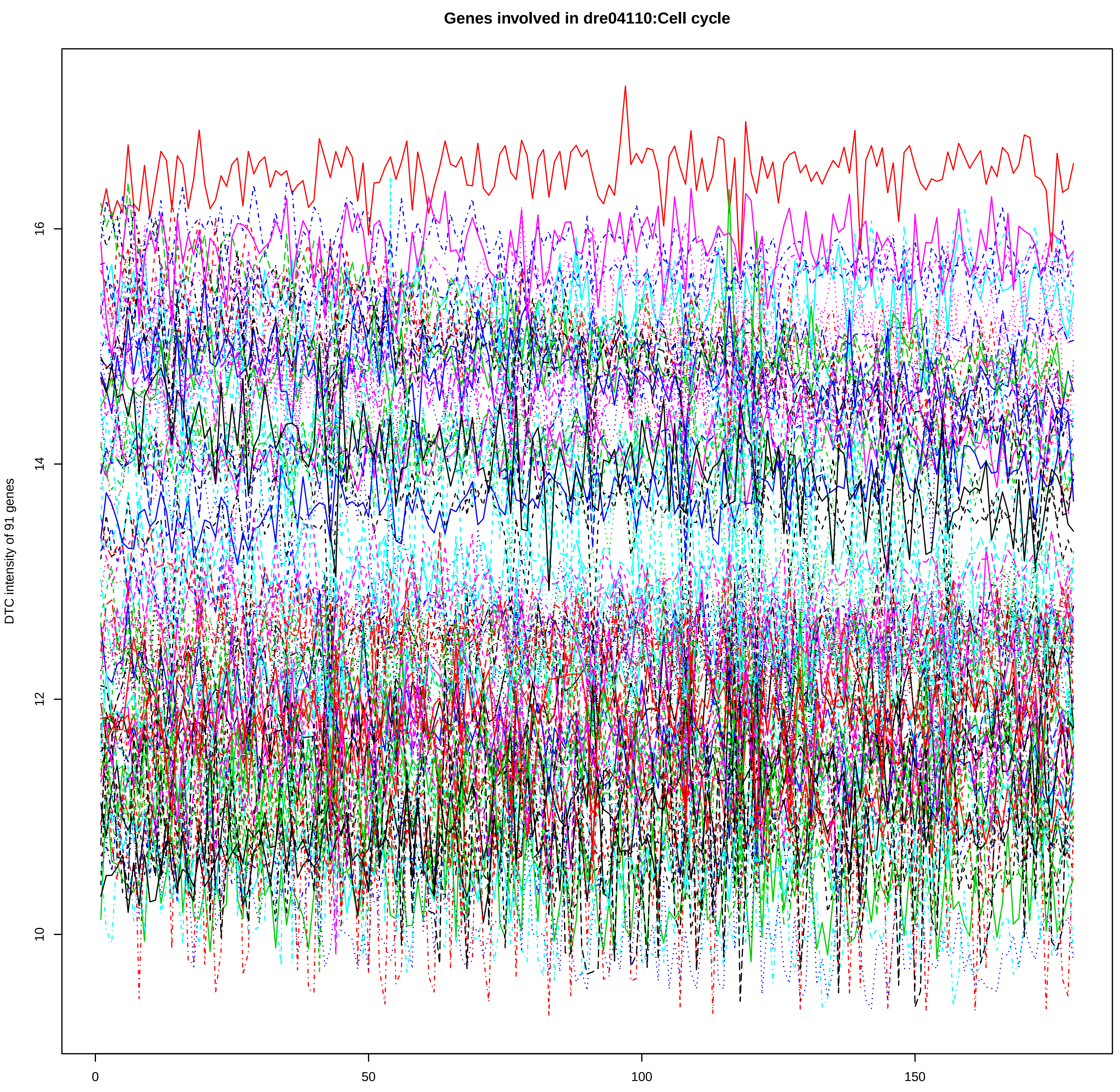

Genes involved in dre04110:Cell cycle
DTC intensity of 91 genes
10
12
14
16
0
50
100
150

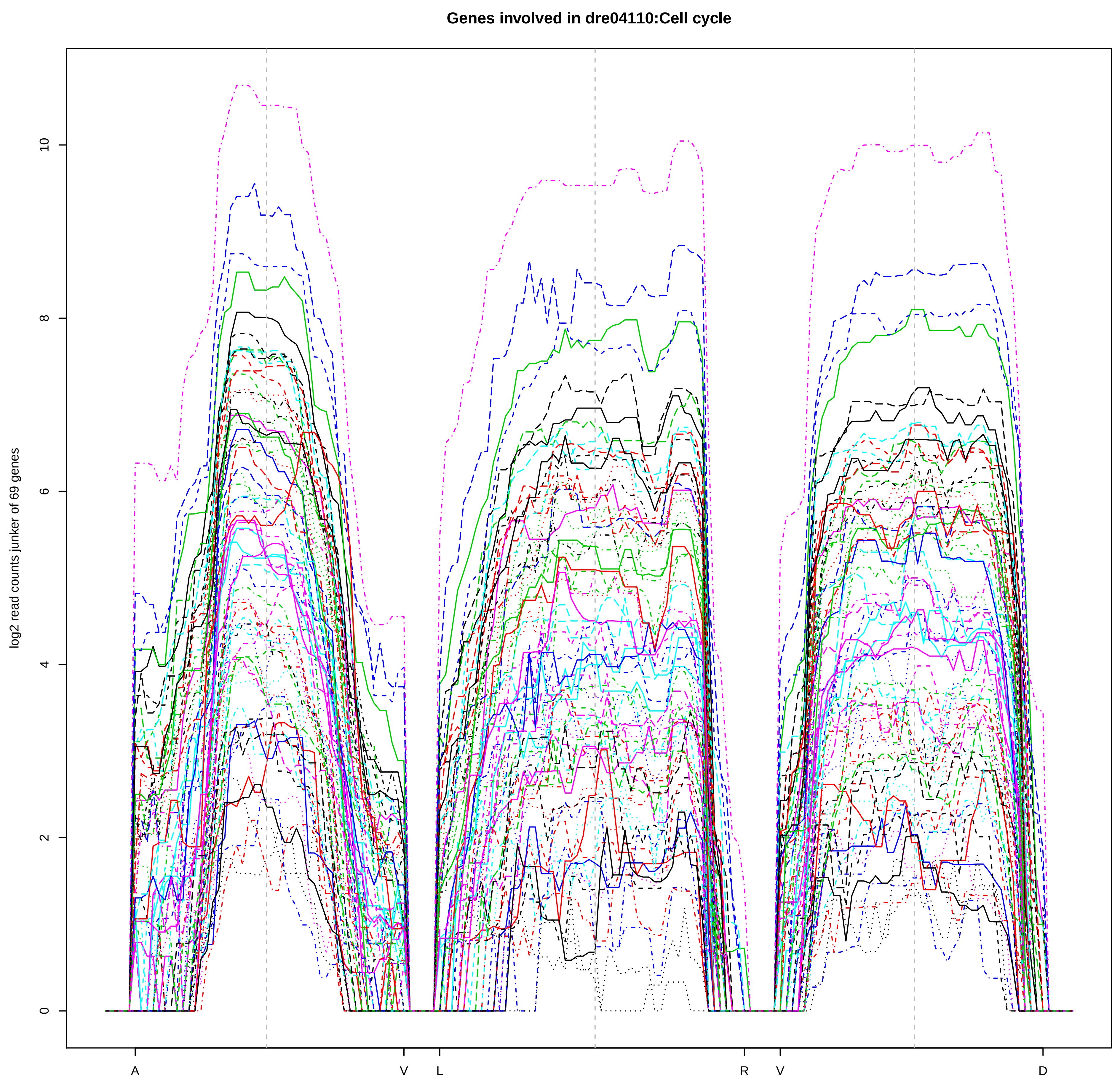

Genes involved in dre04110:Cell cycle
log2 read counts junker of 69 genes
0
2
4
6
8
10
A
V
L
R
V
D

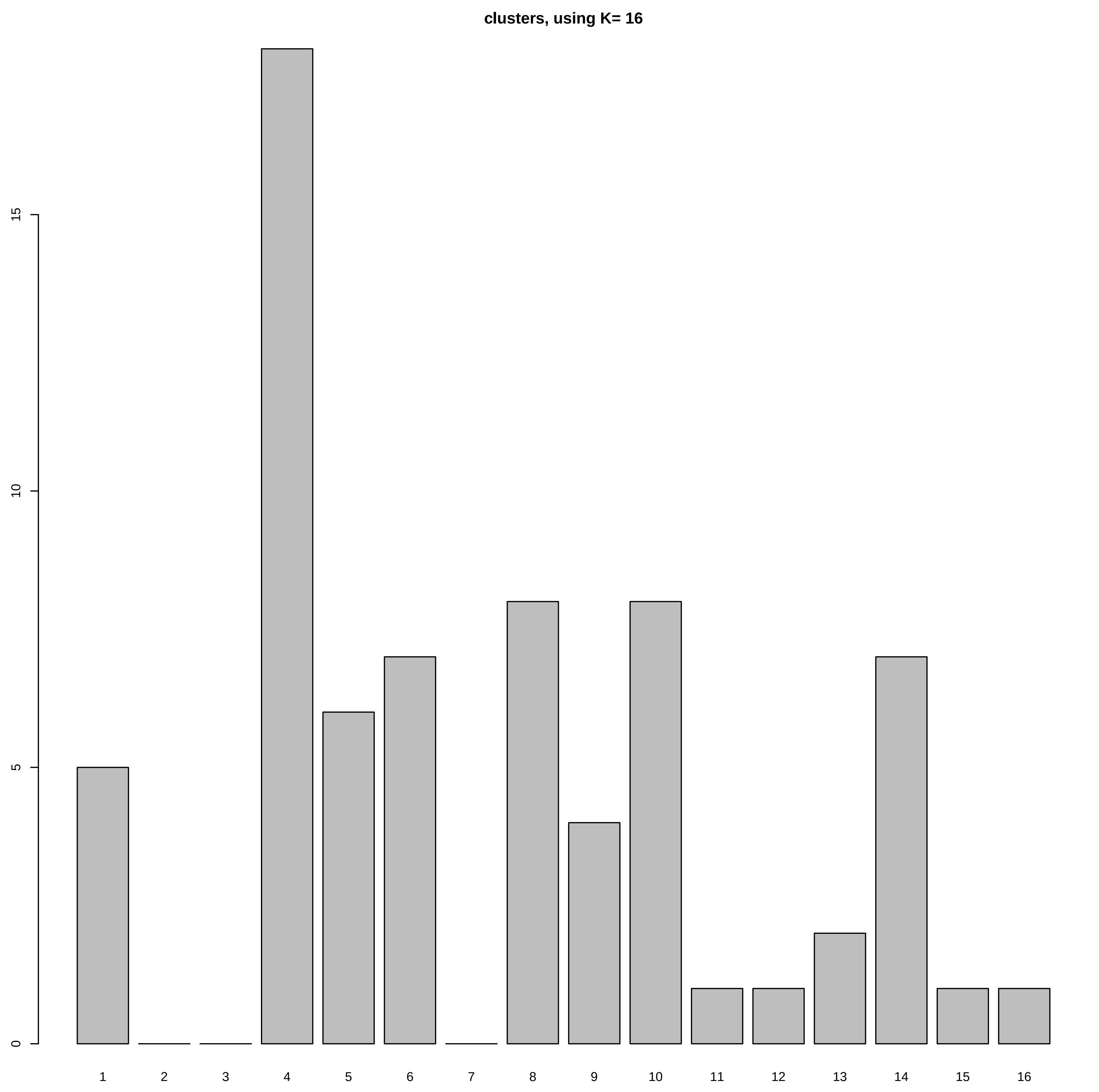

clusters, using K= 16
0
5
10
15
1
2
3
4
5
6
7
8
9
10
11
12
13
14
15
16

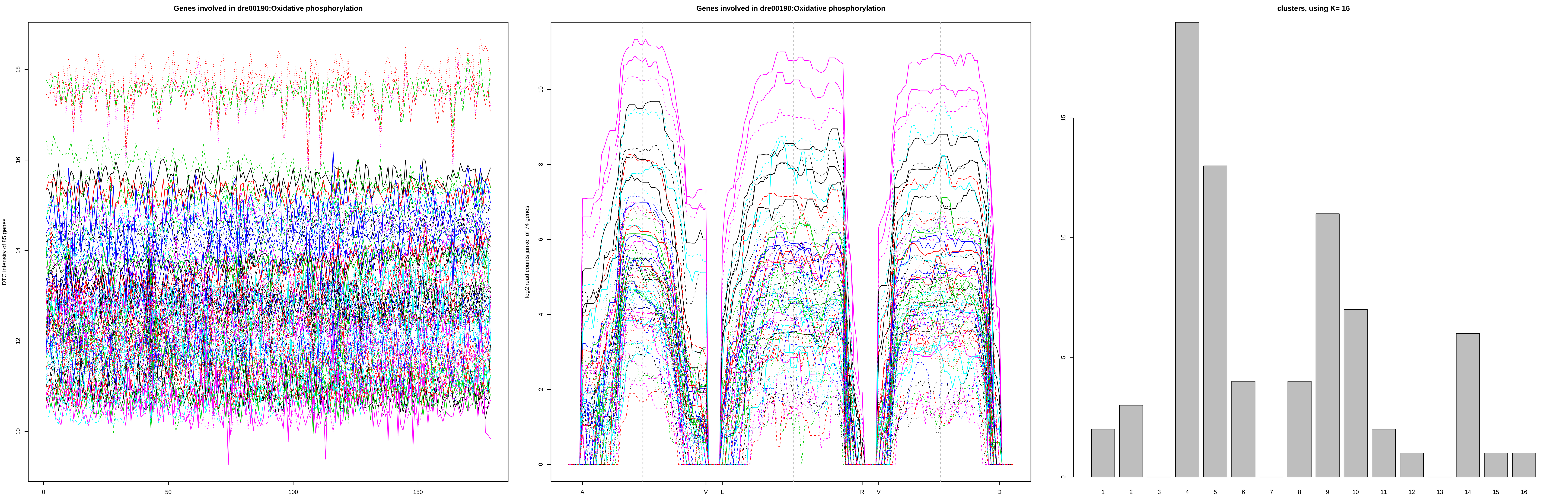
Genes involved in dre00190:Oxidative phosphorylation
DTC intensity of 85 genes
10
12
14
16
18
0
50
100
150
Genes involved in dre00190:Oxidative phosphorylation
log2 read counts junker of 74 genes
0
2
4
6
8
10
A
V
L
R
V
D
clusters, using K= 16
0
5
10
15
1
2
3
4
5
6
7
8
9
10
11
12
13
14
15
16

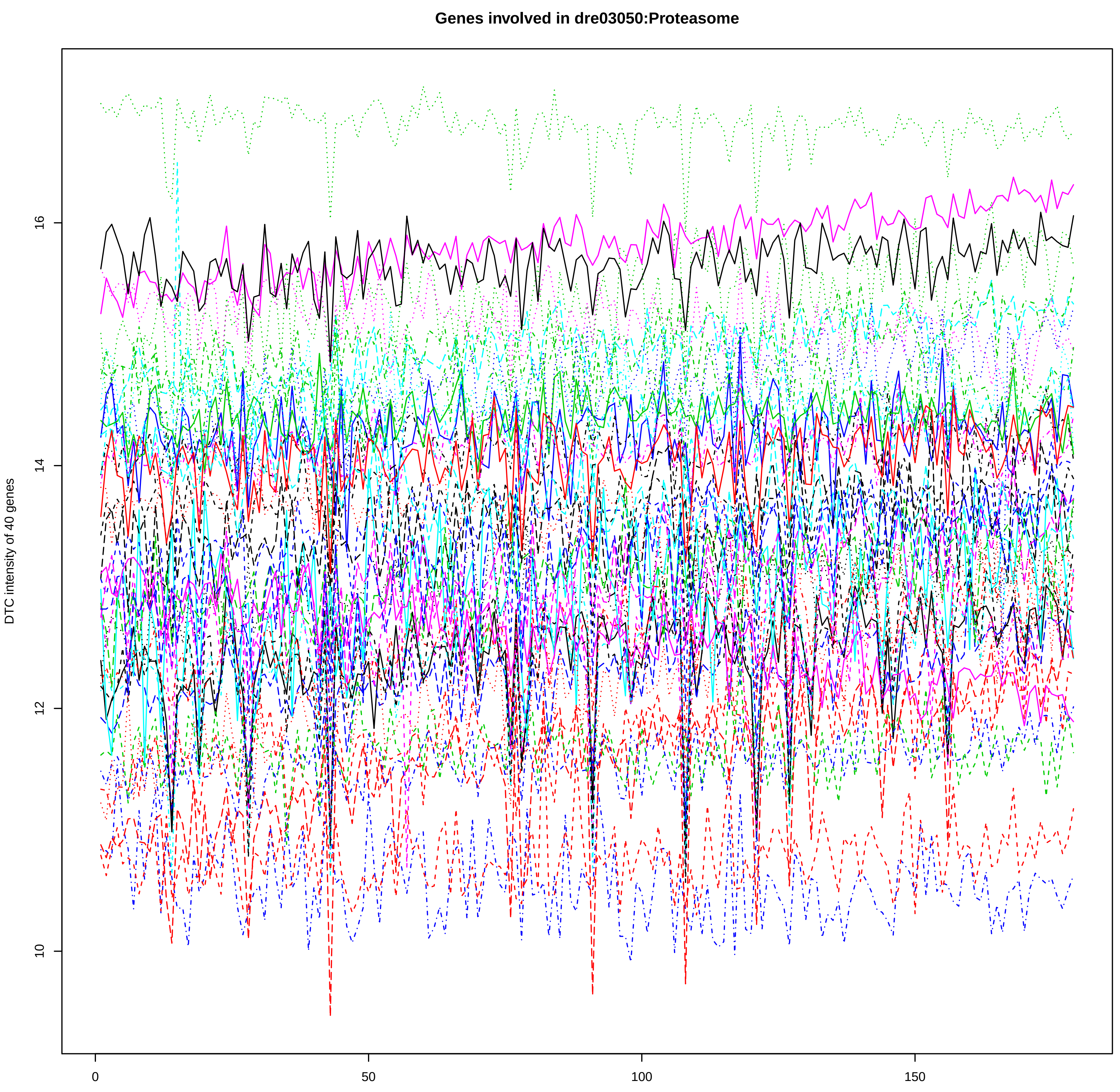

Genes involved in dre03050:Proteasome
DTC intensity of 40 genes
10
12
14
16
0
50
100
150

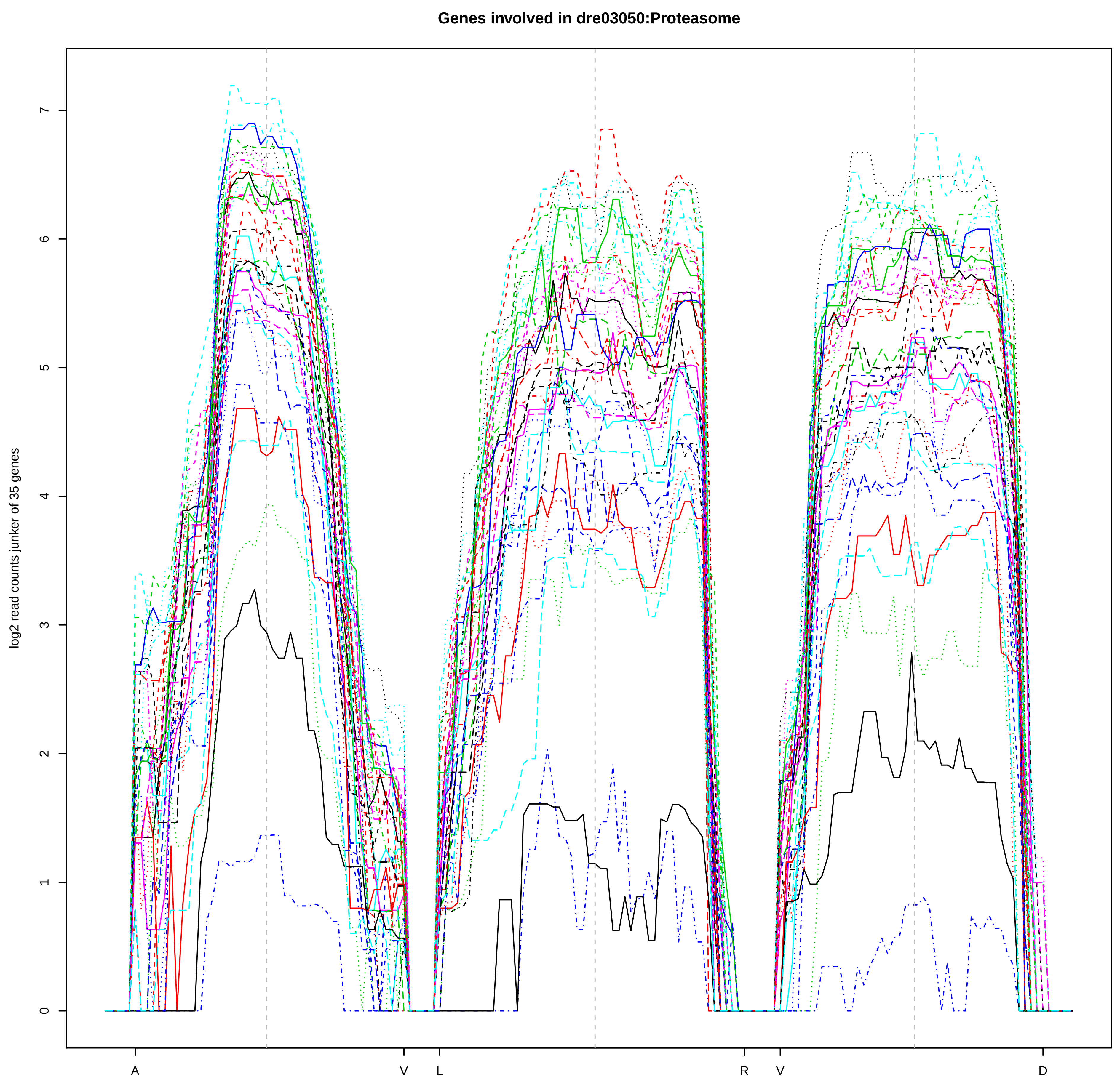

Genes involved in dre03050:Proteasome
log2 read counts junker of 35 genes
0
1
2
3
4
5
6
7
A
V
L
R
V
D

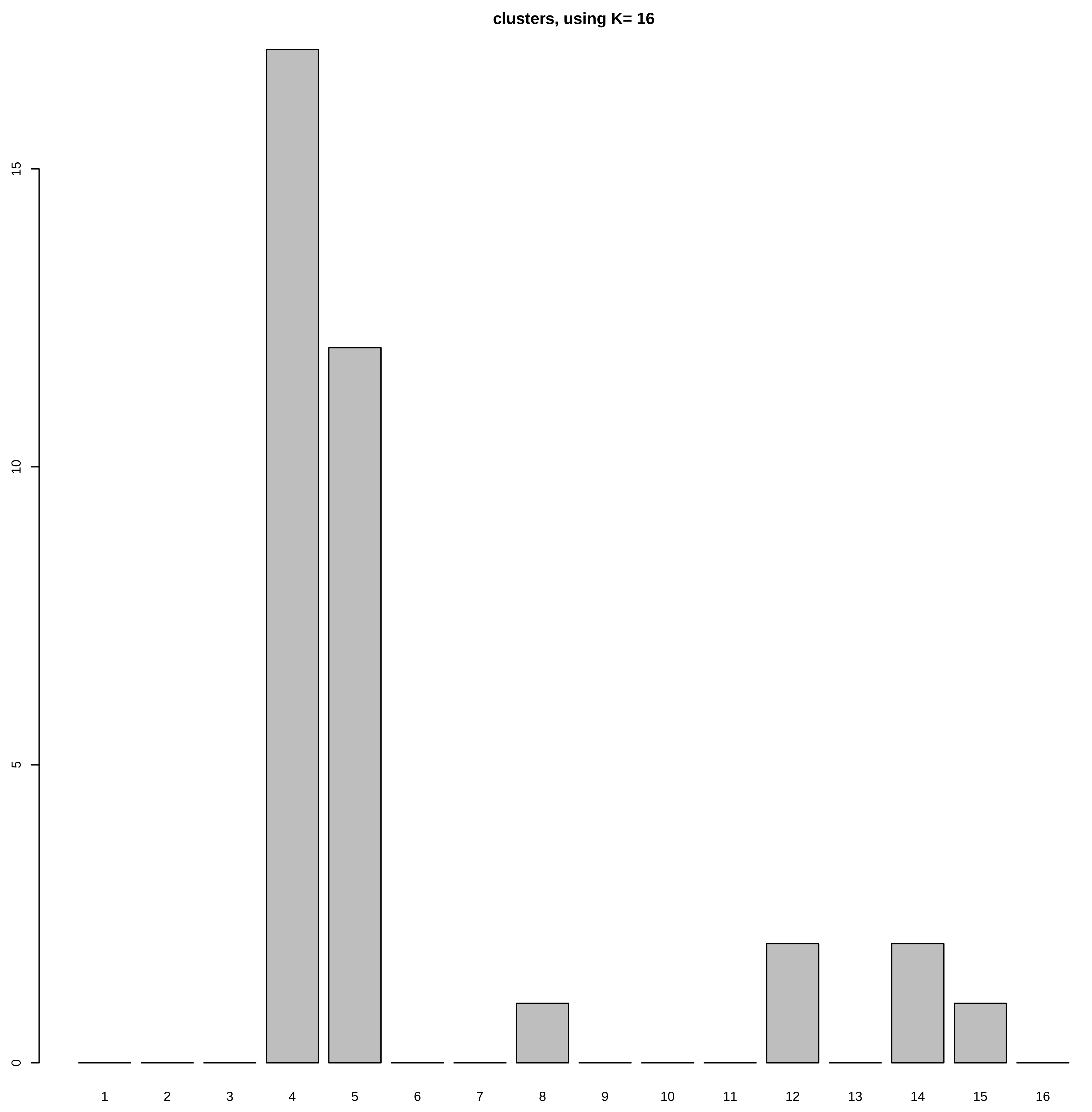

clusters, using K= 16
0
5
10
15
1
2
3
4
5
6
7
8
9
10
11
12
13
14
15
16

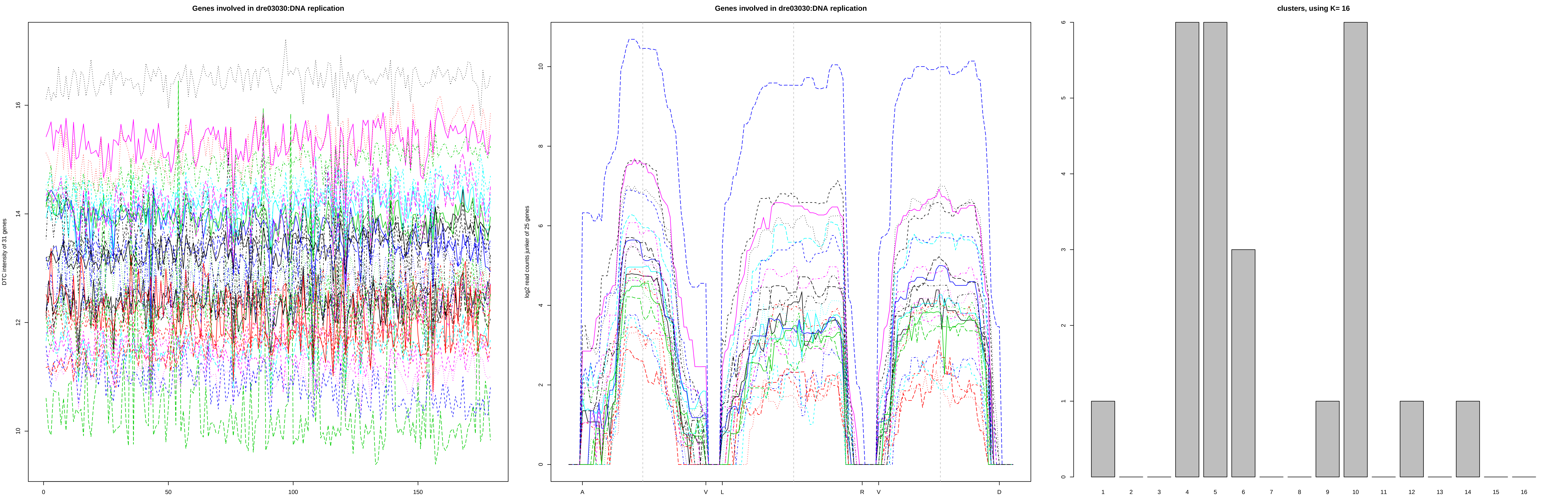

Genes involved in dre03030:DNA replication
DTC intensity of 31 genes
Genes involved in dre03030:DNA replication
log2 read counts junker of 25 genes
A
V
L
R
V
D
clusters, using K= 16

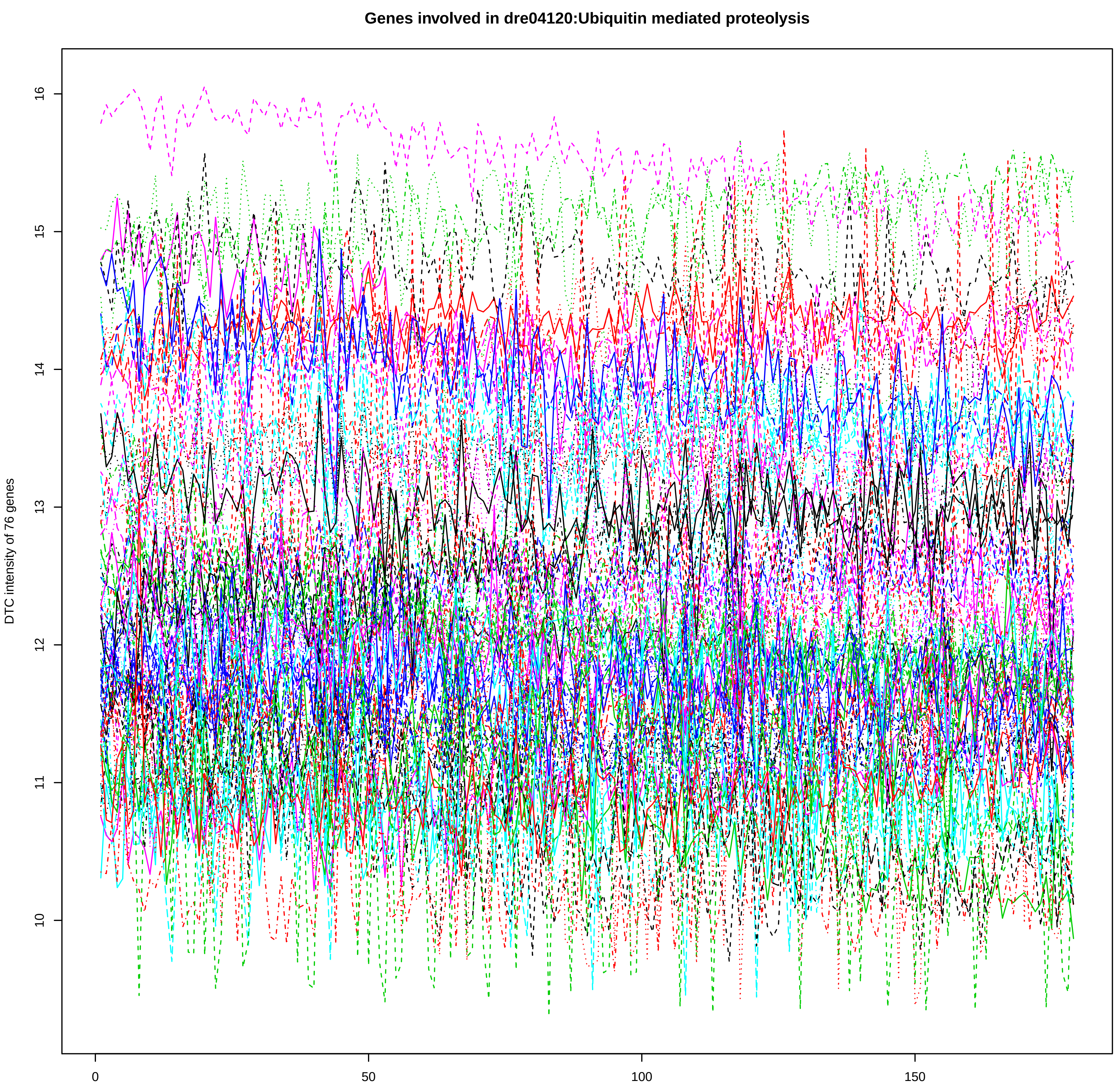
Genes involved in dre04120:Ubiquitin mediated proteolysis
DTC intensity of 76 genes
0
50
100
150
10
11
12
13
14
15
16

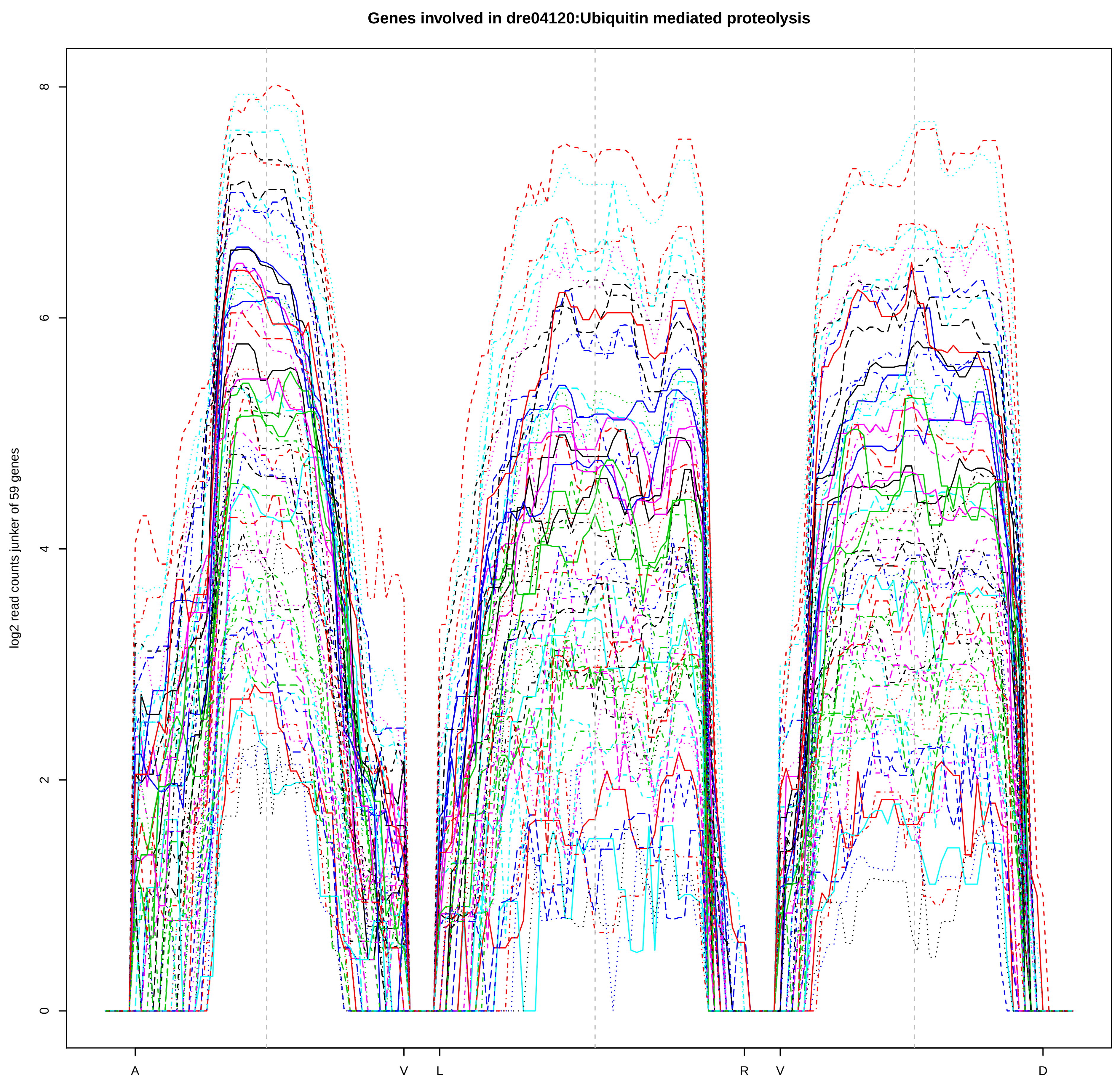
Genes involved in dre04120:Ubiquitin mediated proteolysis
log2 read counts junker of 59 genes
A
V
L
R
V
D
0
2
4
6
8

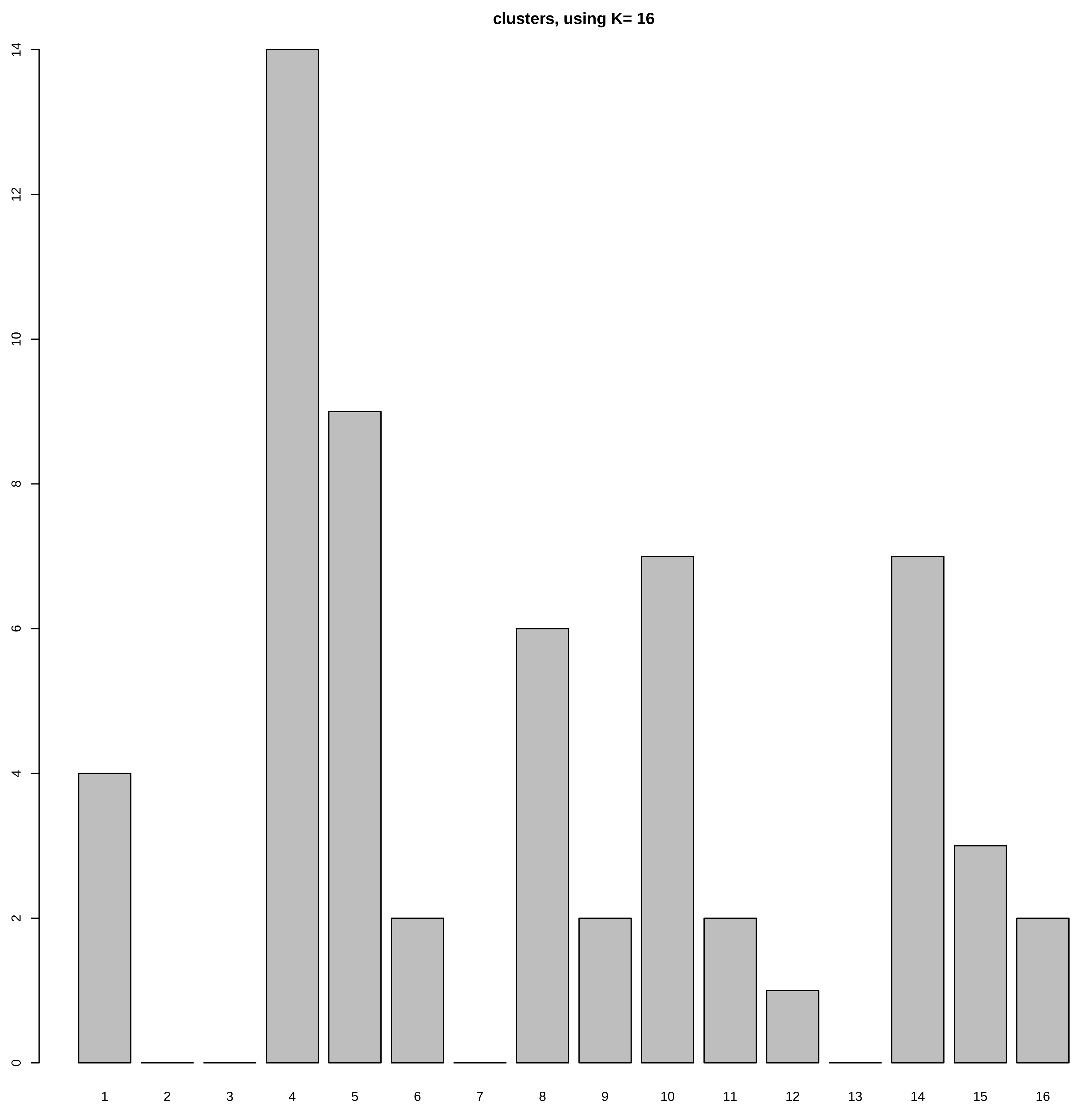
clusters, using K= 16
1
2
3
4
5
6
7
8
9
10
11
12
13
14
15
16
0
2
4
6
8
10
12
14

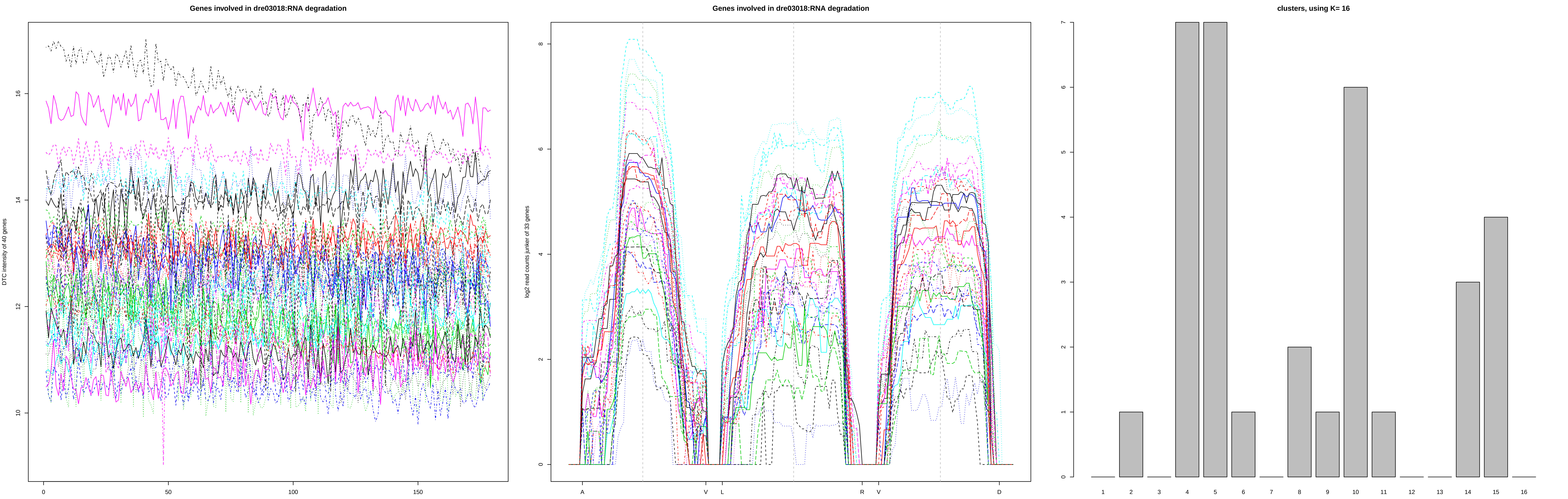

Genes involved in dre03018:RNA degradation
DTC intensity of 40 genes
10
12
14
16
0
50
100
150
Genes involved in dre03018:RNA degradation
log2 read counts junker of 33 genes
0
2
4
6
8
A
V
L
R
V
D
clusters, using K= 16
0
1
2
3
4
5
6
7
1
2
3
4
5
6
7
8
9
10
11
12
13
14
15
16

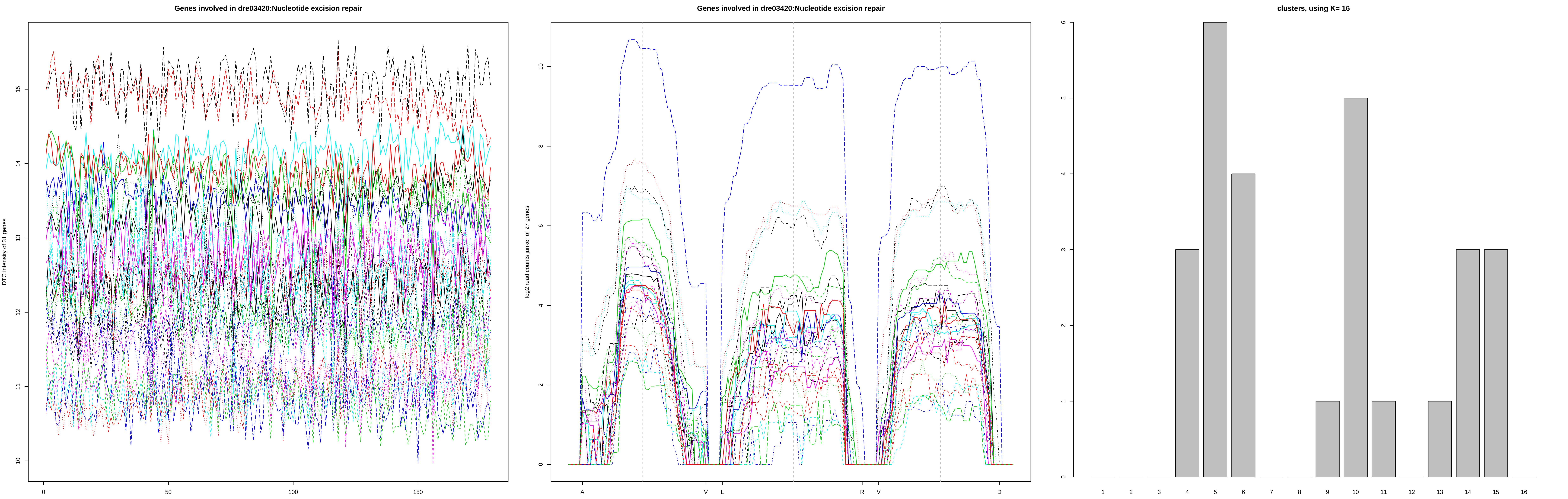

Genes involved in dre03420:Nucleotide excision repair
DTC intensity of 31 genes
Genes involved in dre03420:Nucleotide excision repair
log2 read counts junker of 27 genes
A
V
L
R
V
D
clusters, using K= 16

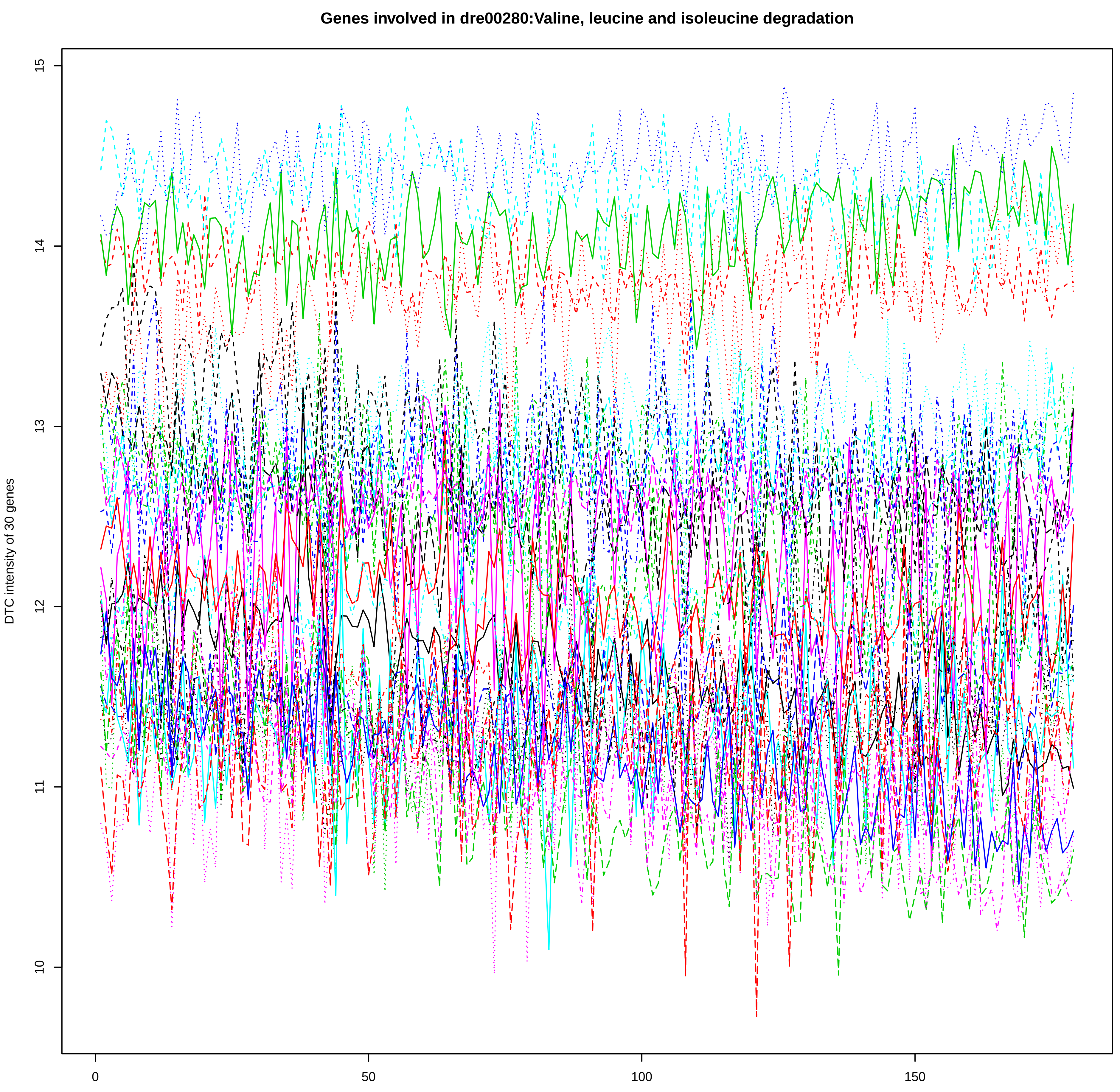

Genes involved in dre00280:Valine, leucine and isoleucine degradation
DTC intensity of 30 genes

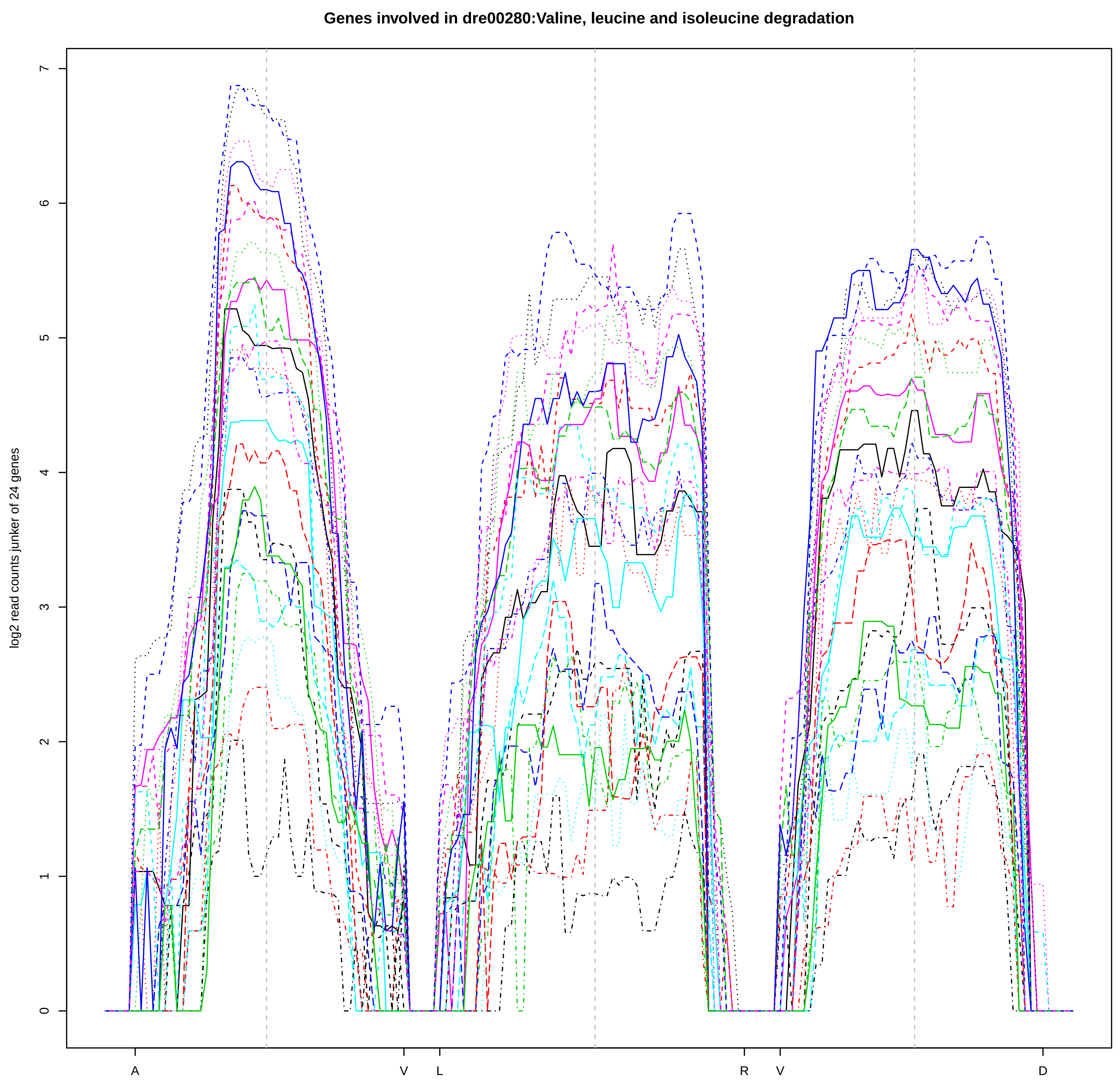

Genes involved in dre00280:Valine, leucine and isoleucine degradation
log2 read counts junker of 24 genes
A
V
L
R
V
D

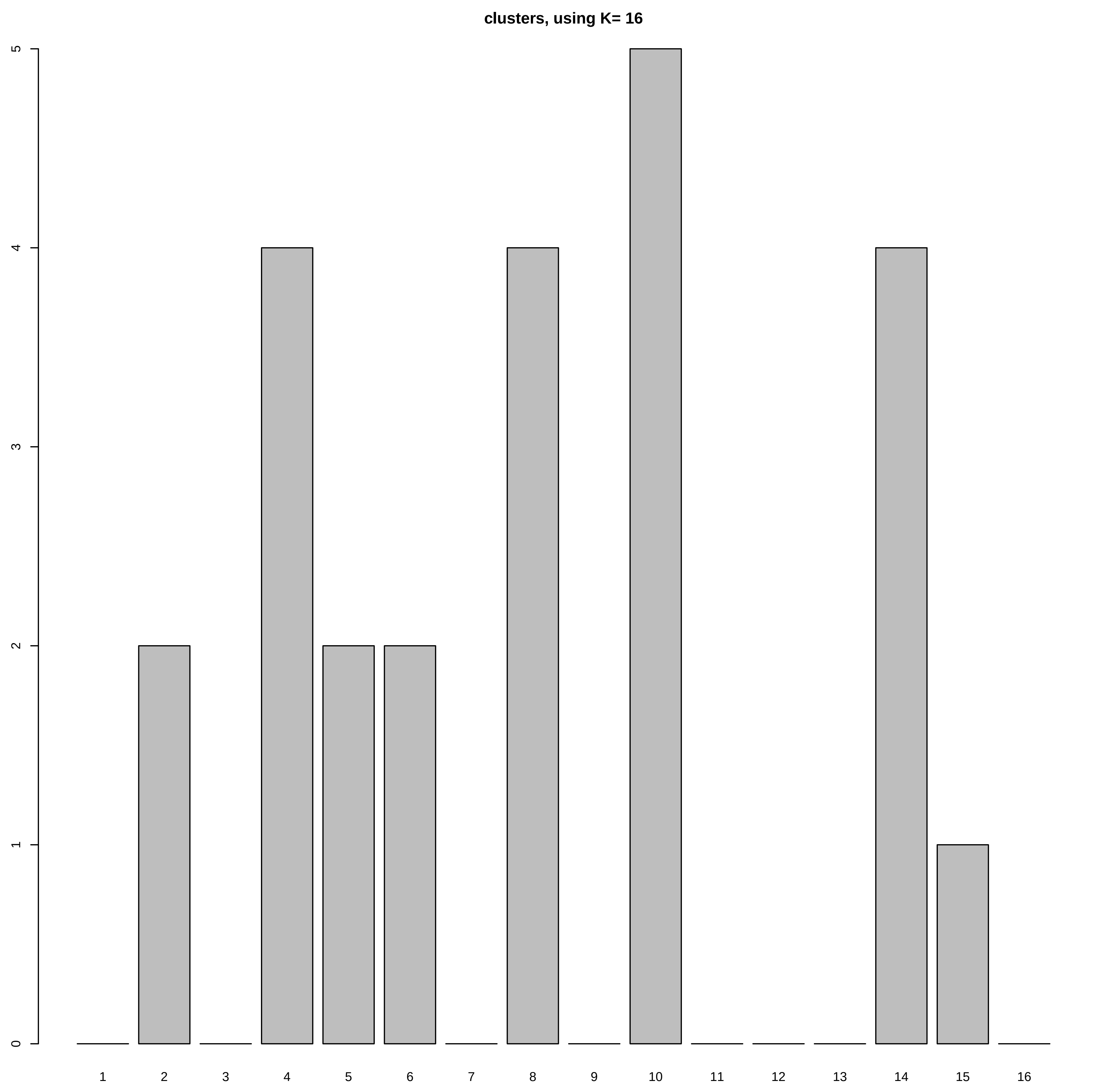

clusters, using K= 16

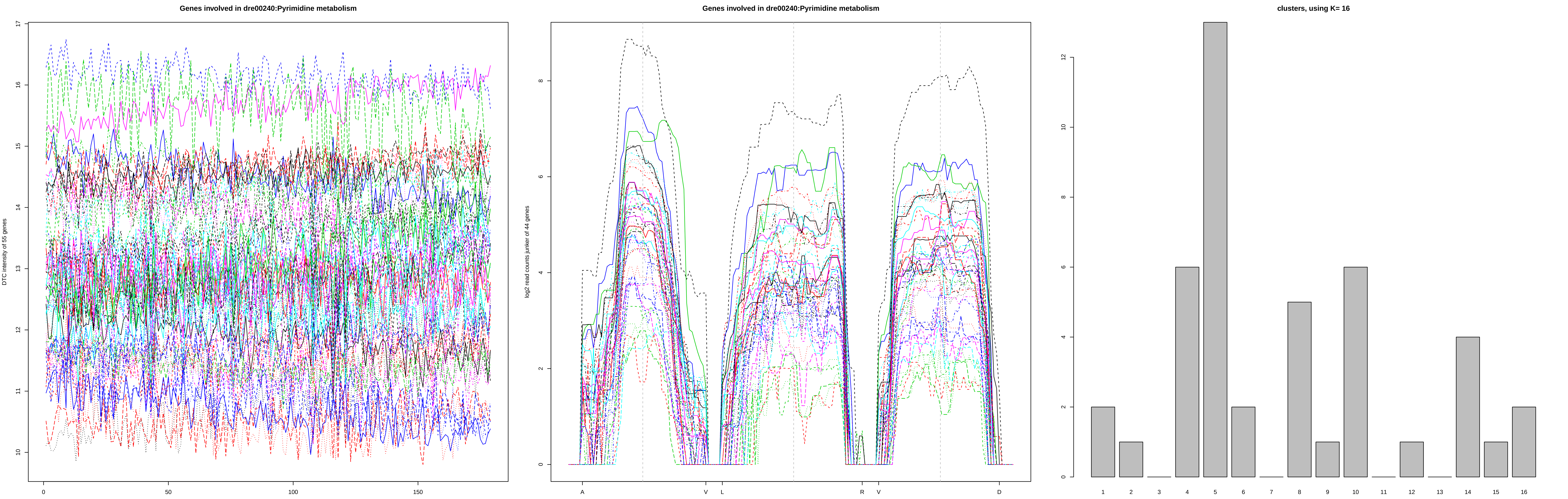

Genes involved in dre00240:Pyrimidine metabolism
DTC intensity of 55 genes
Genes involved in dre00240:Pyrimidine metabolism
log2 read counts junker of 44 genes
A
V
L
R
V
D
clusters, using K= 16

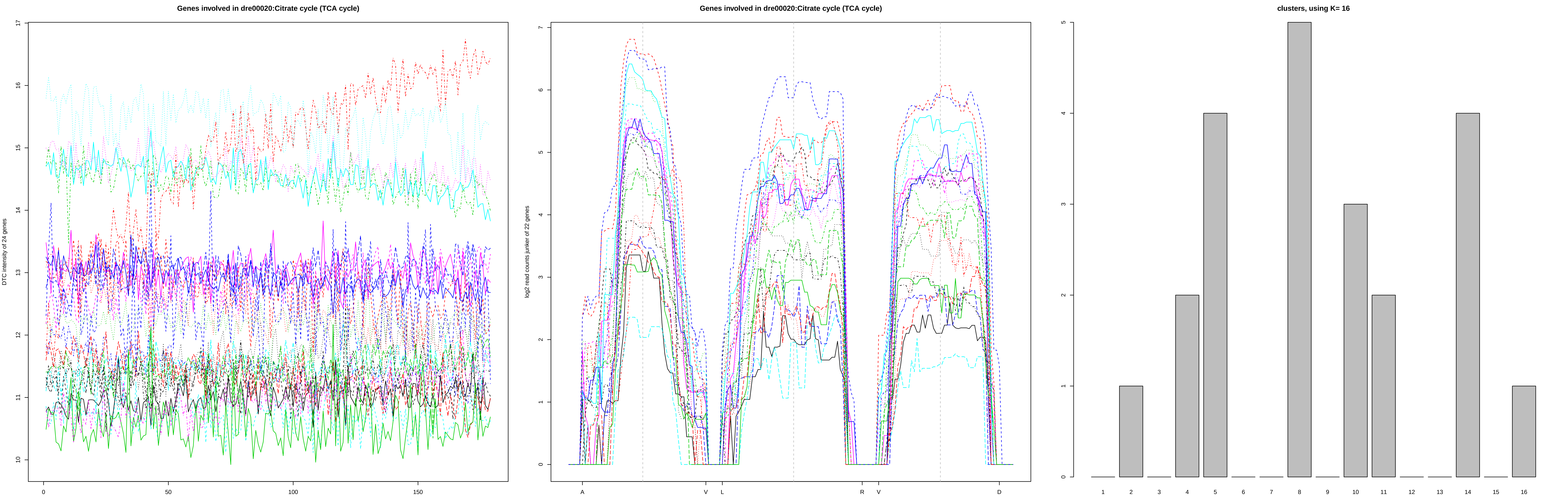
Genes involved in dre00020:Citrate cycle (TCA cycle)
DTC intensity of 24 genes
10
11
12
13
14
15
16
17
0
50
100
150
Genes involved in dre00020:Citrate cycle (TCA cycle)
log2 read counts junker of 22 genes
0
1
2
3
4
5
6
7
A
V
L
R
V
D
clusters, using K= 16
0
1
2
3
4
5
1
2
3
4
5
6
7
8
9
10
11
12
13
14
15
16

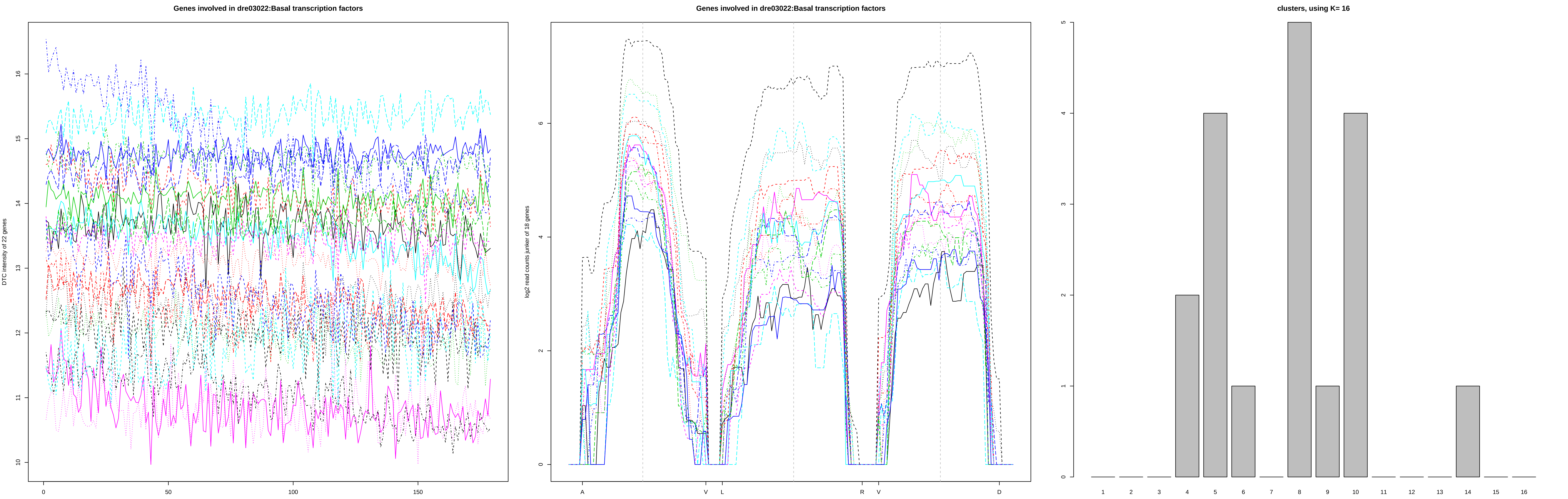

Genes involved in dre03022:Basal transcription factors
DTC intensity of 22 genes
Genes involved in dre03022:Basal transcription factors
log2 read counts junker of 18 genes
A
V
L
R
V
D
clusters, using K= 16

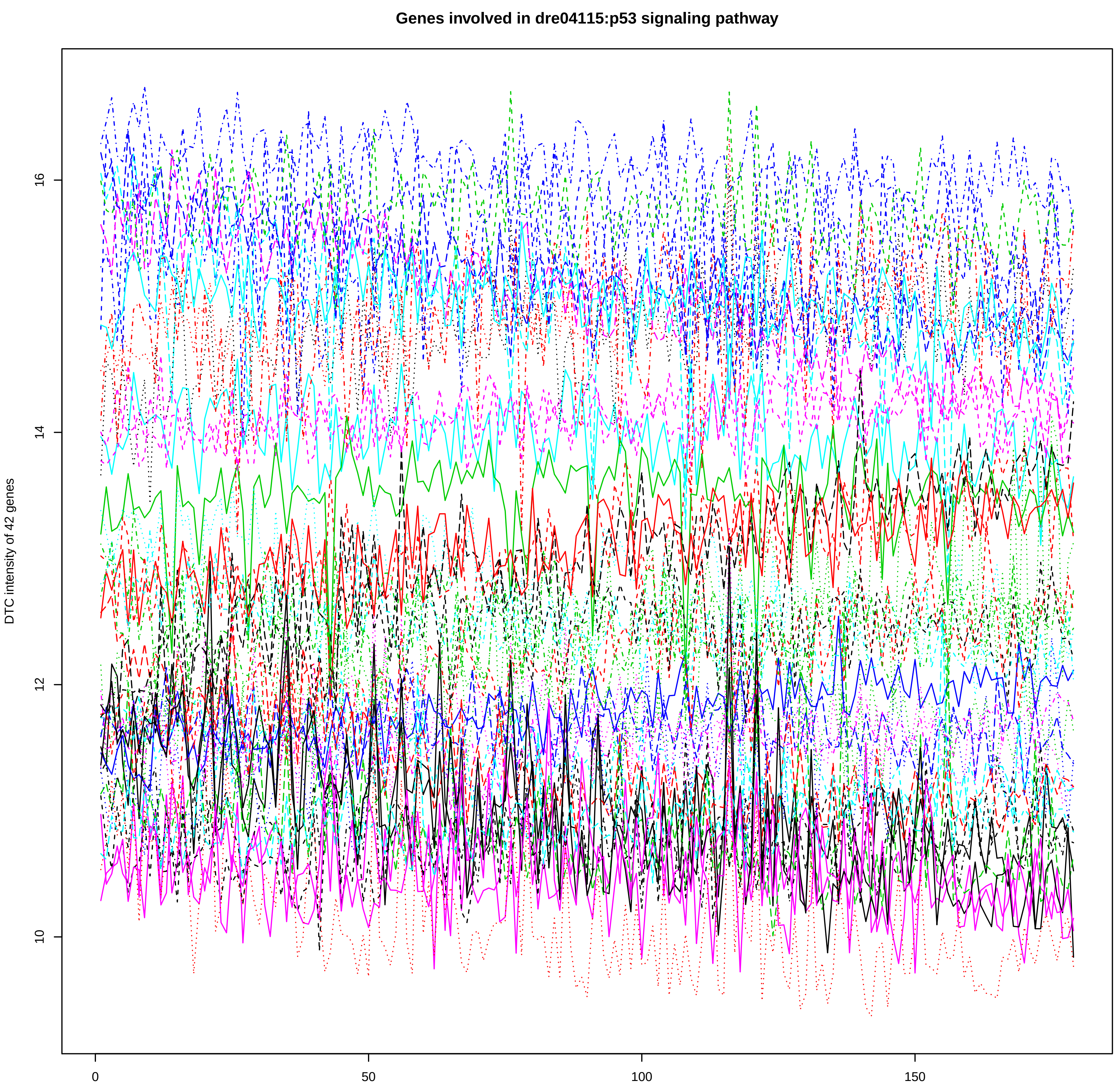

Genes involved in dre04115:p53 signaling pathway
DTC intensity of 42 genes
10
12
14
16
0
50
100
150

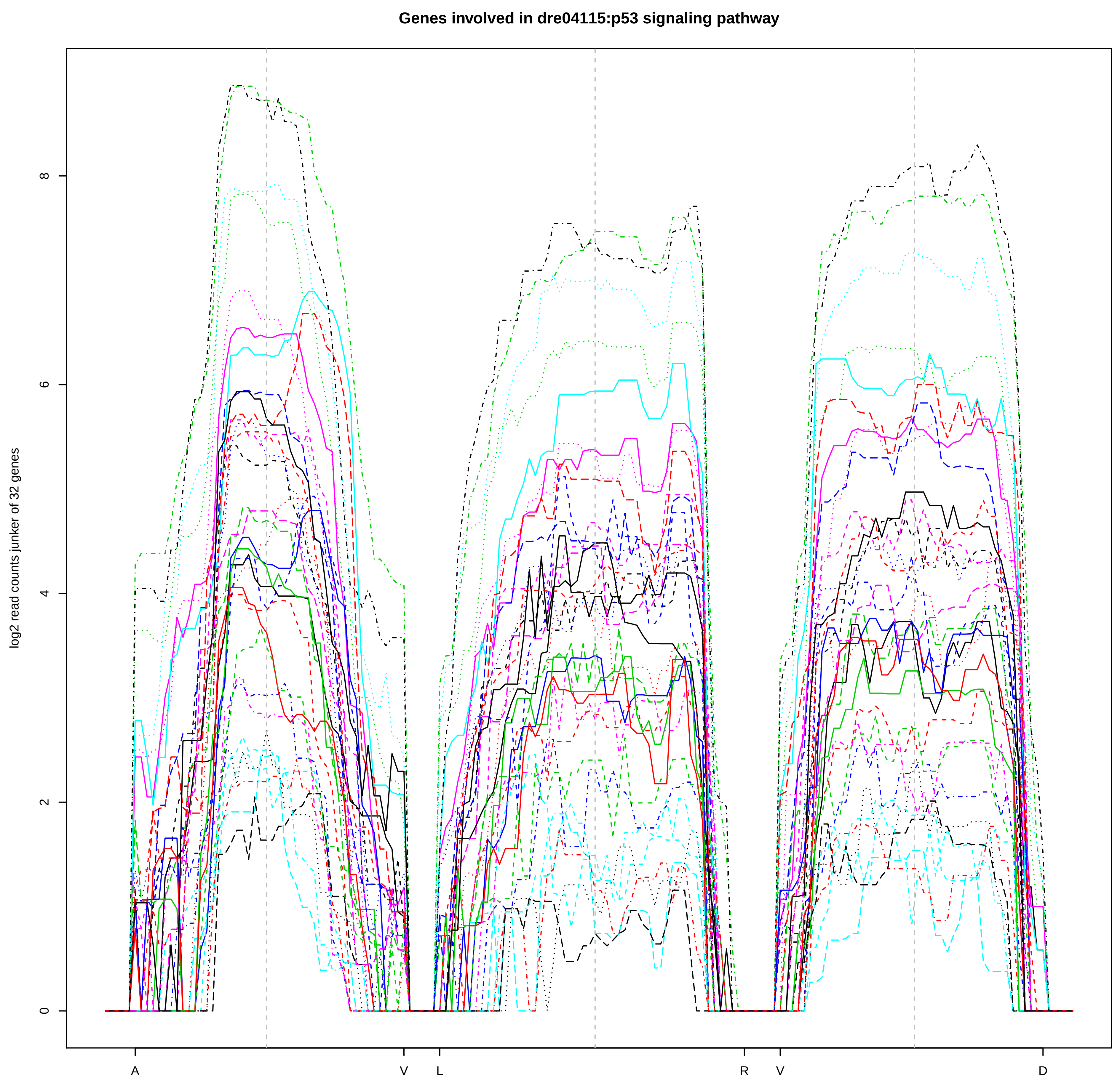

Genes involved in dre04115:p53 signaling pathway
log2 read counts junker of 32 genes
0
2
4
6
8
A
V
L
R
V
D

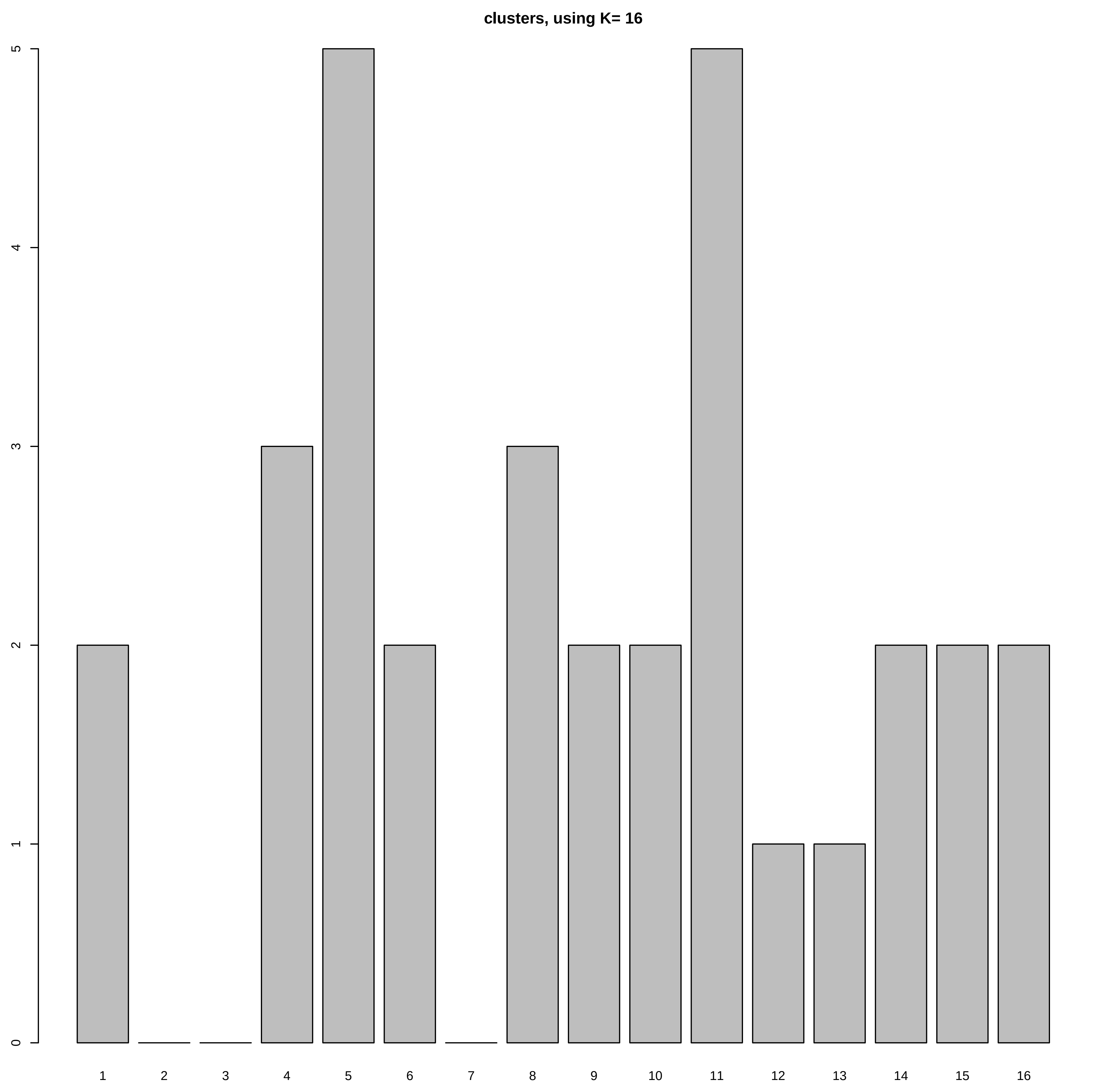

clusters, using K= 16
0
1
2
3
4
5
1
2
3
4
5
6
7
8
9
10
11
12
13
14
15
16

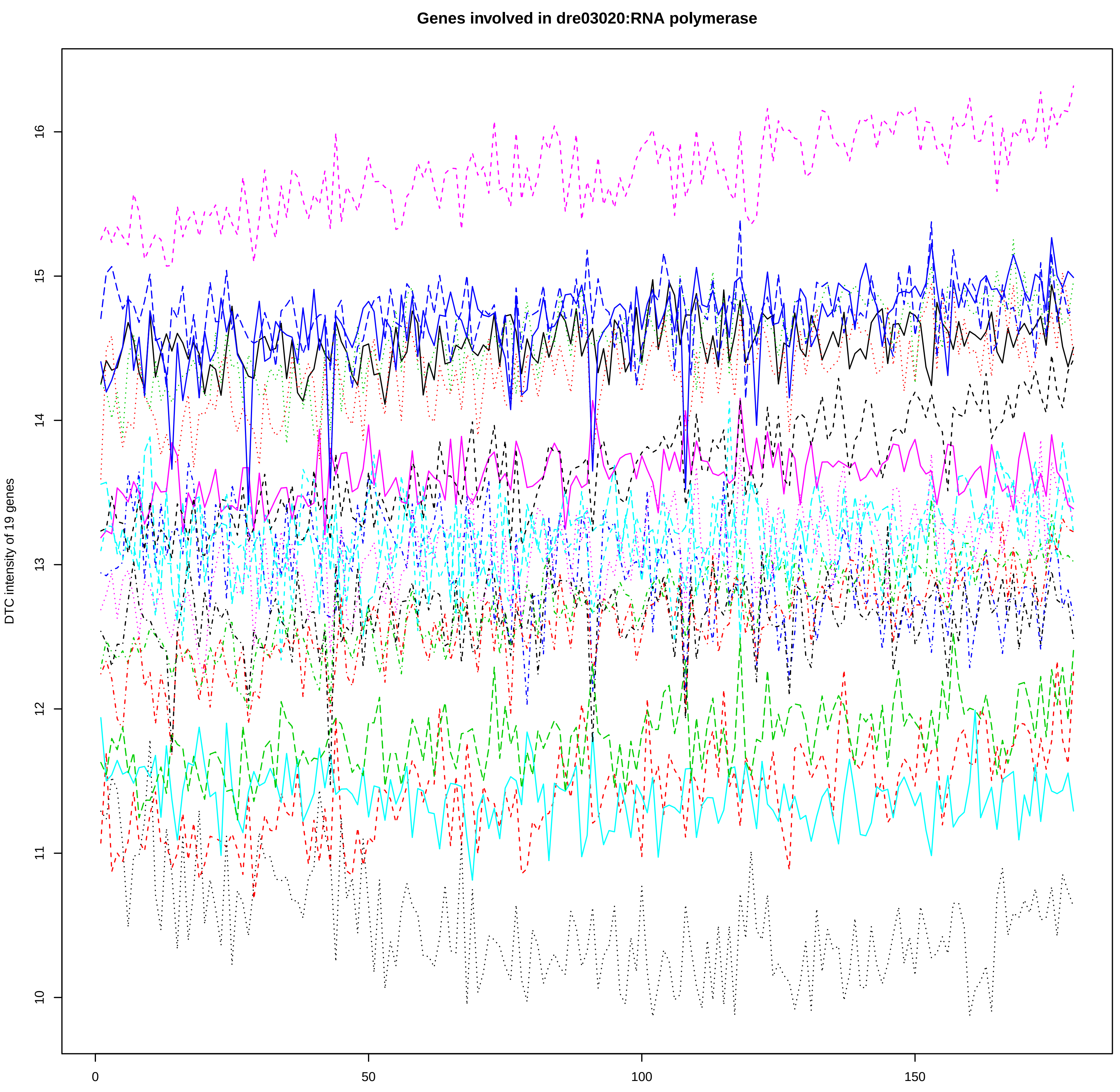

Genes involved in dre03020:RNA polymerase
DTC intensity of 19 genes

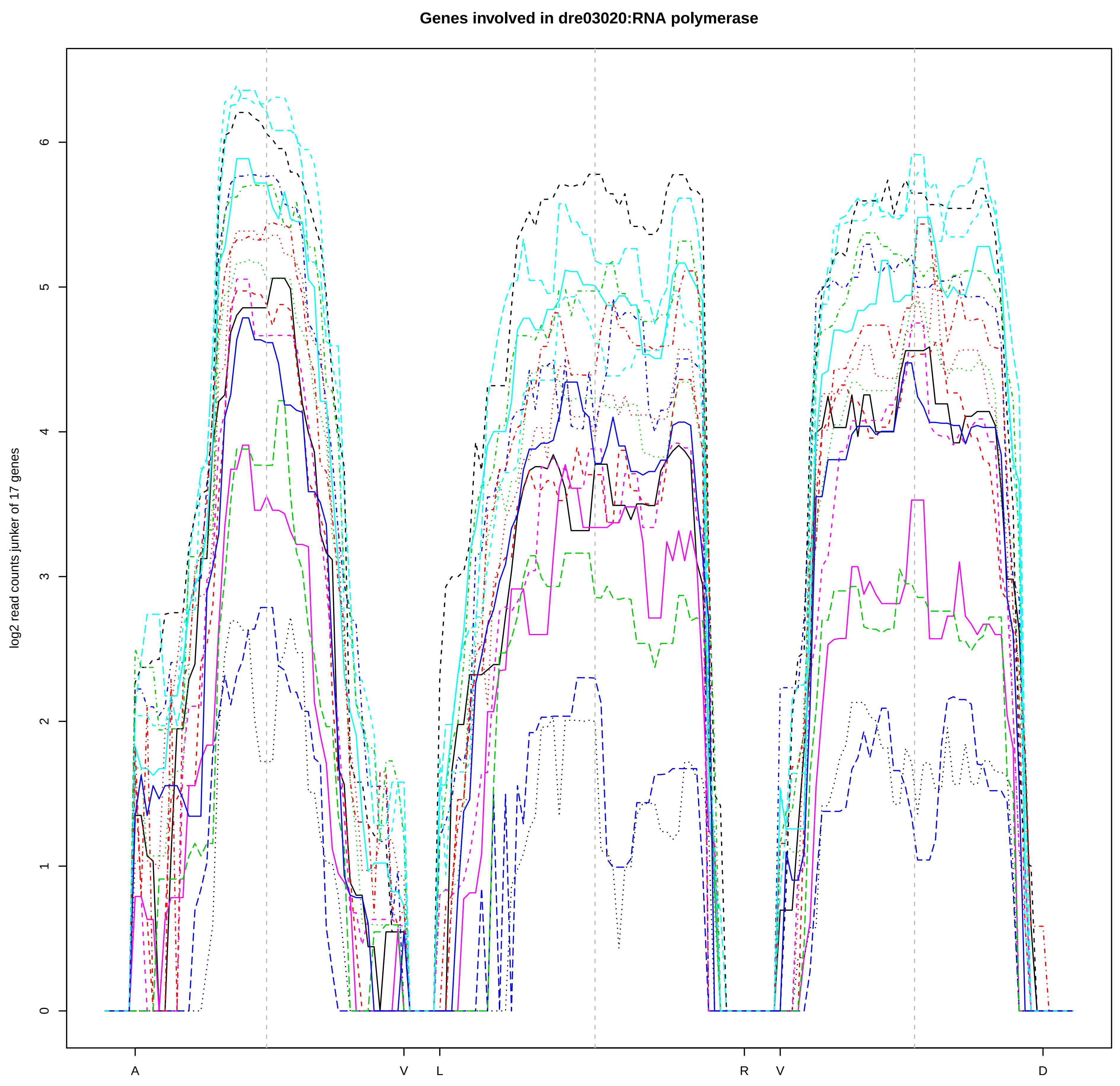

Genes involved in dre03020:RNA polymerase
log2 read counts junker of 17 genes
A
V
L
R
V
D

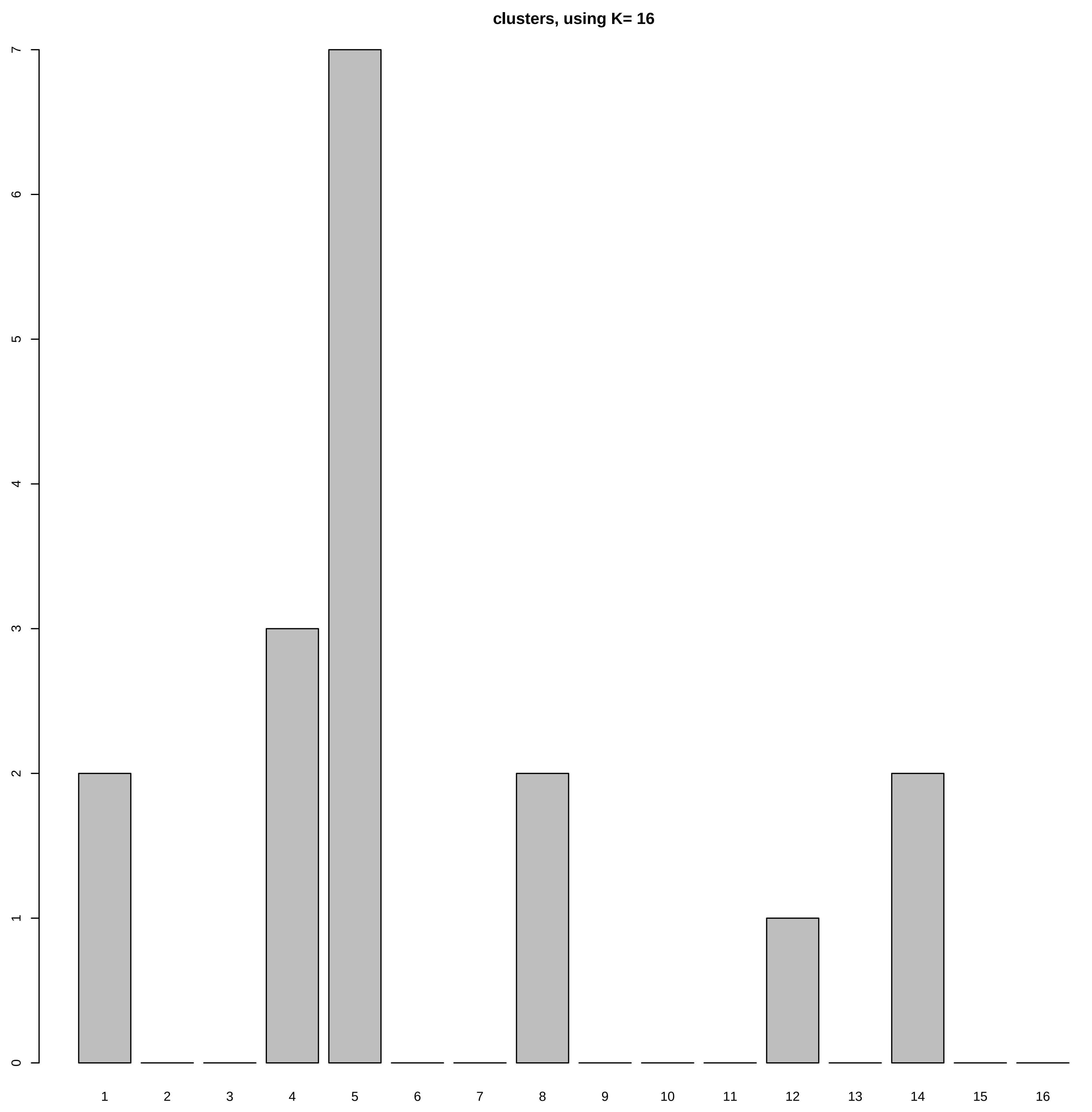

clusters, using K= 16

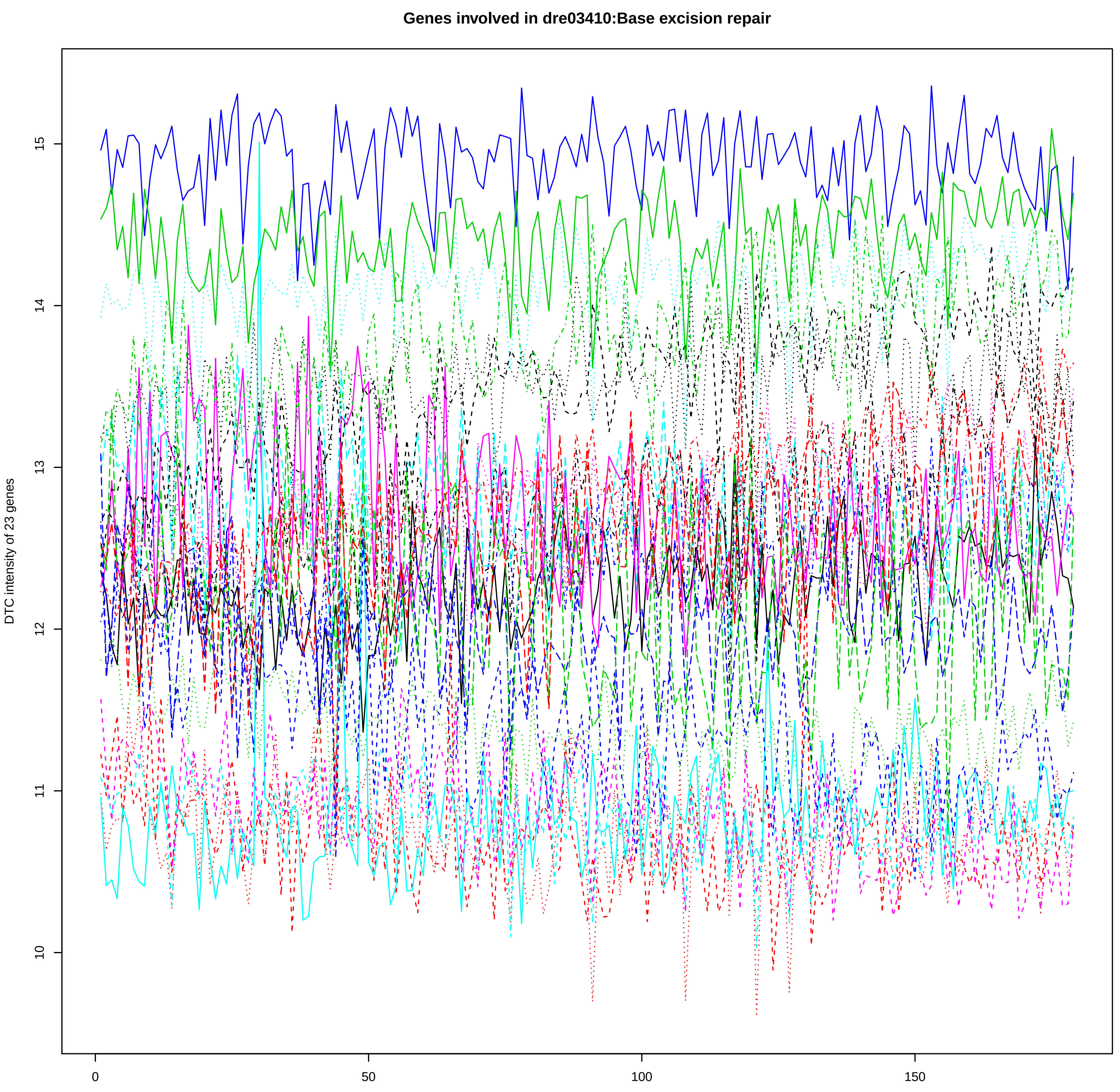
Genes involved in dre03410:Base excision repair
DTC intensity of 23 genes

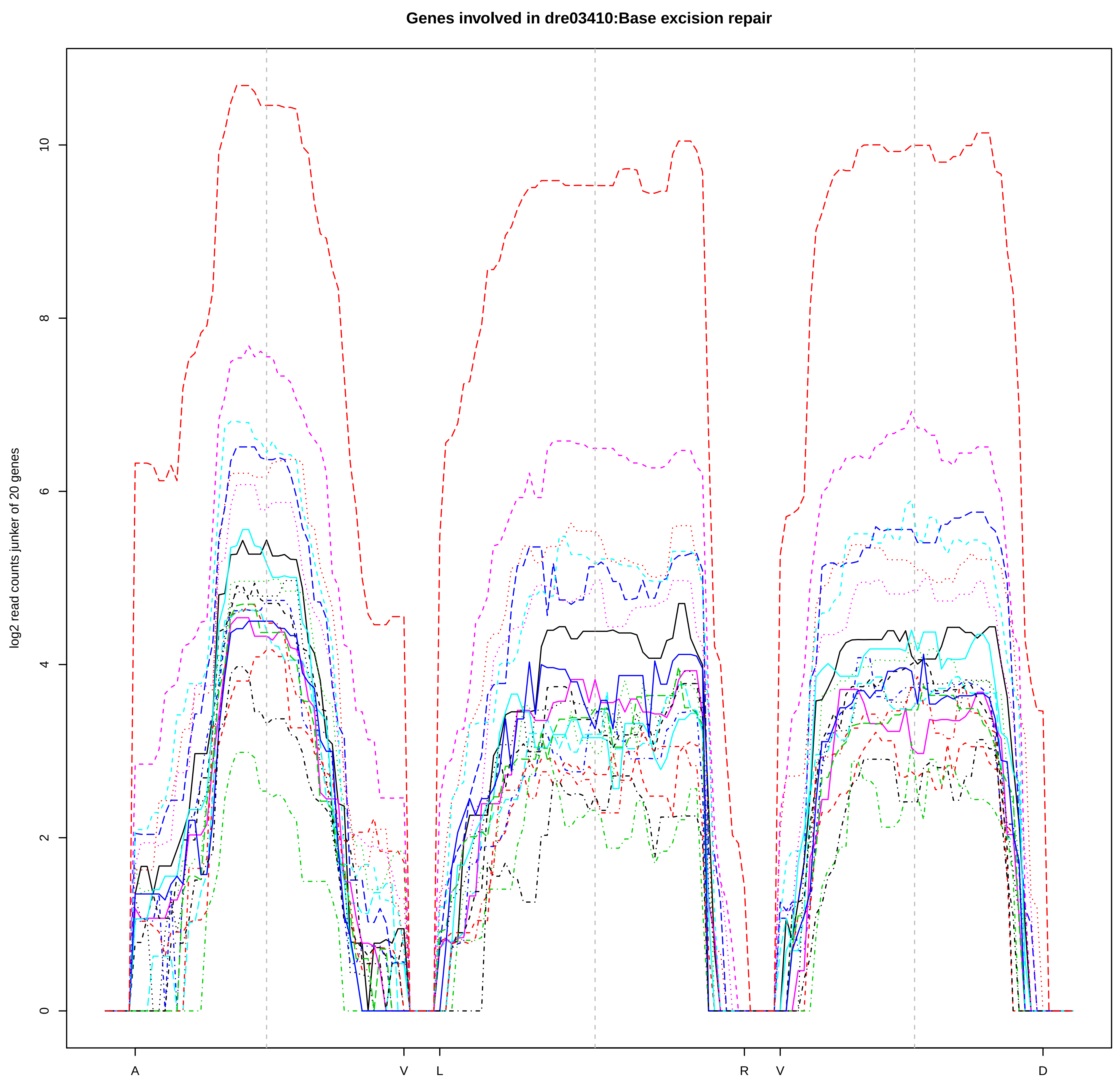
Genes involved in dre03410:Base excision repair
log2 read counts junker of 20 genes
A
V
L
R
V
D

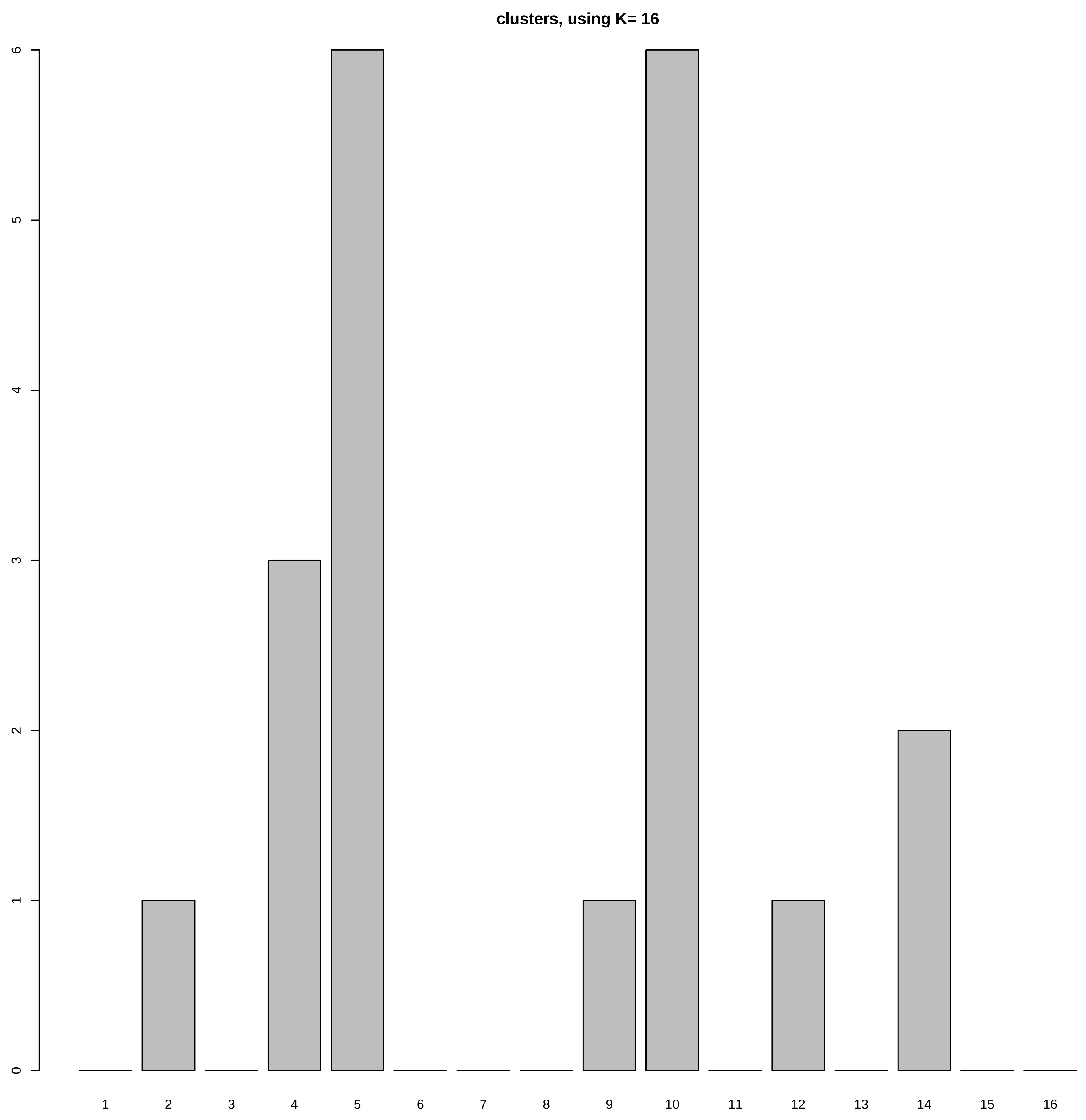
clusters, using K= 16

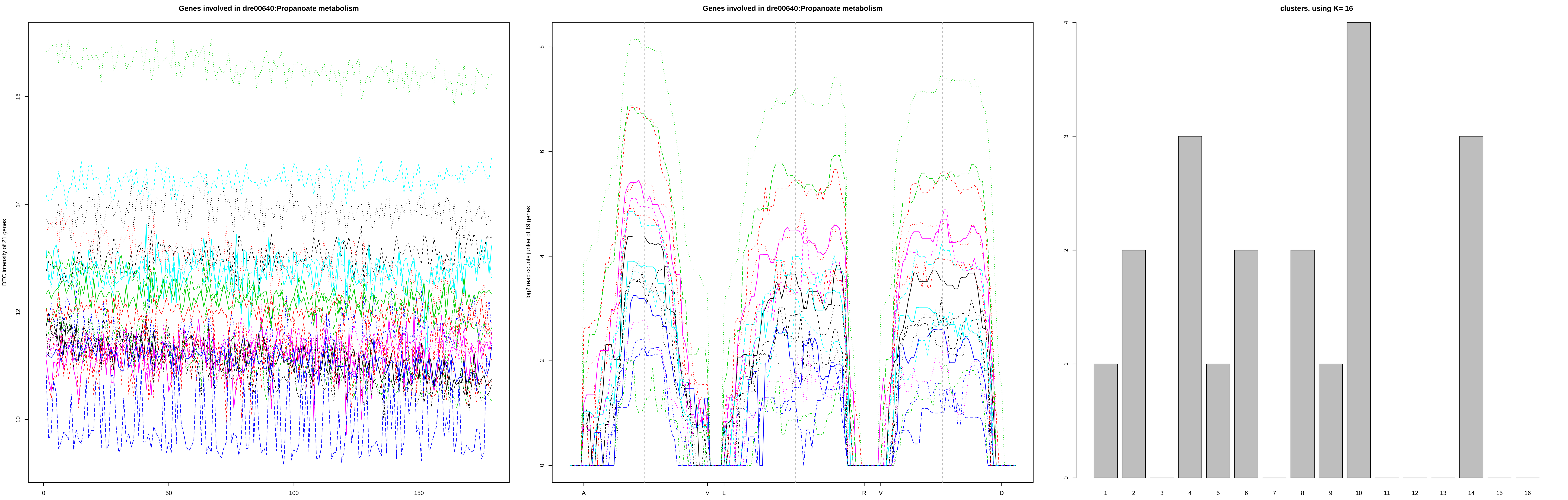
Genes involved in dre00640:Propanoate metabolism
DTC intensity of 21 genes
Genes involved in dre00640:Propanoate metabolism
log2 read counts junker of 19 genes
A
V
L
R
V
D
clusters, using K= 16

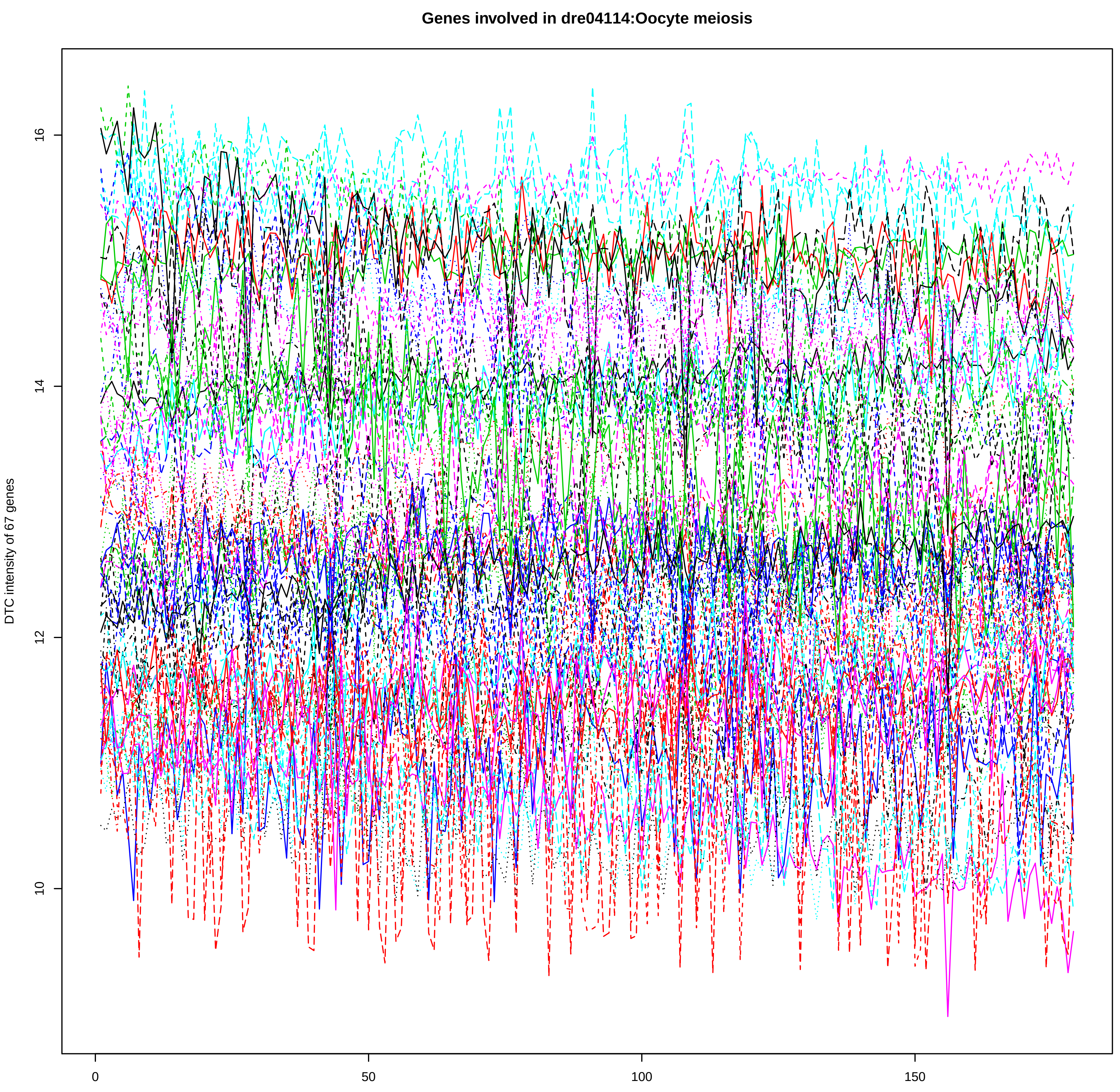
Genes involved in dre04114:Oocyte meiosis
DTC intensity of 67 genes
10
12
14
16
0
50
100
150

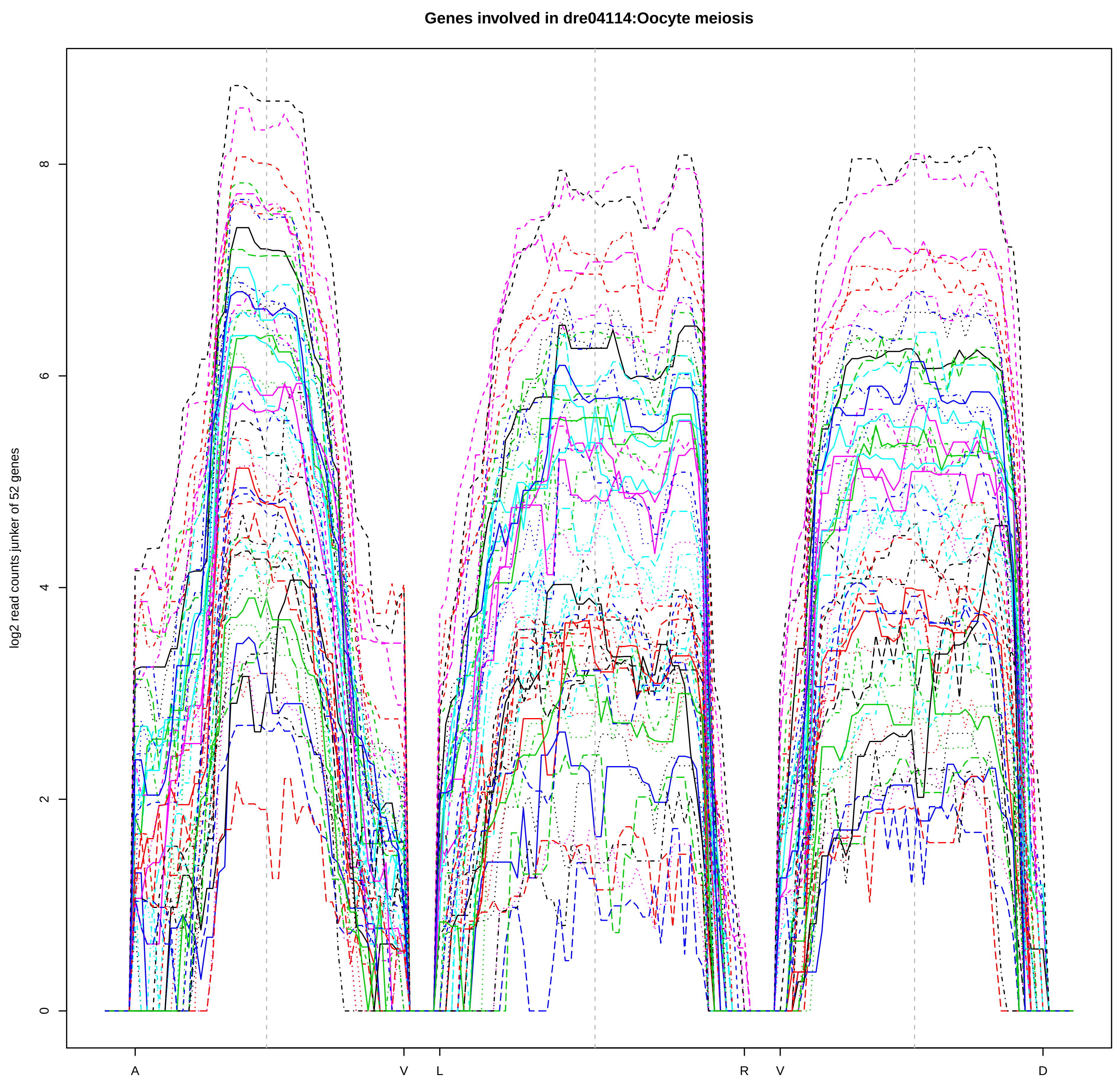
Genes involved in dre04114:Oocyte meiosis
log2 read counts junker of 52 genes
0
2
4
6
8
A
V
L
R
V
D

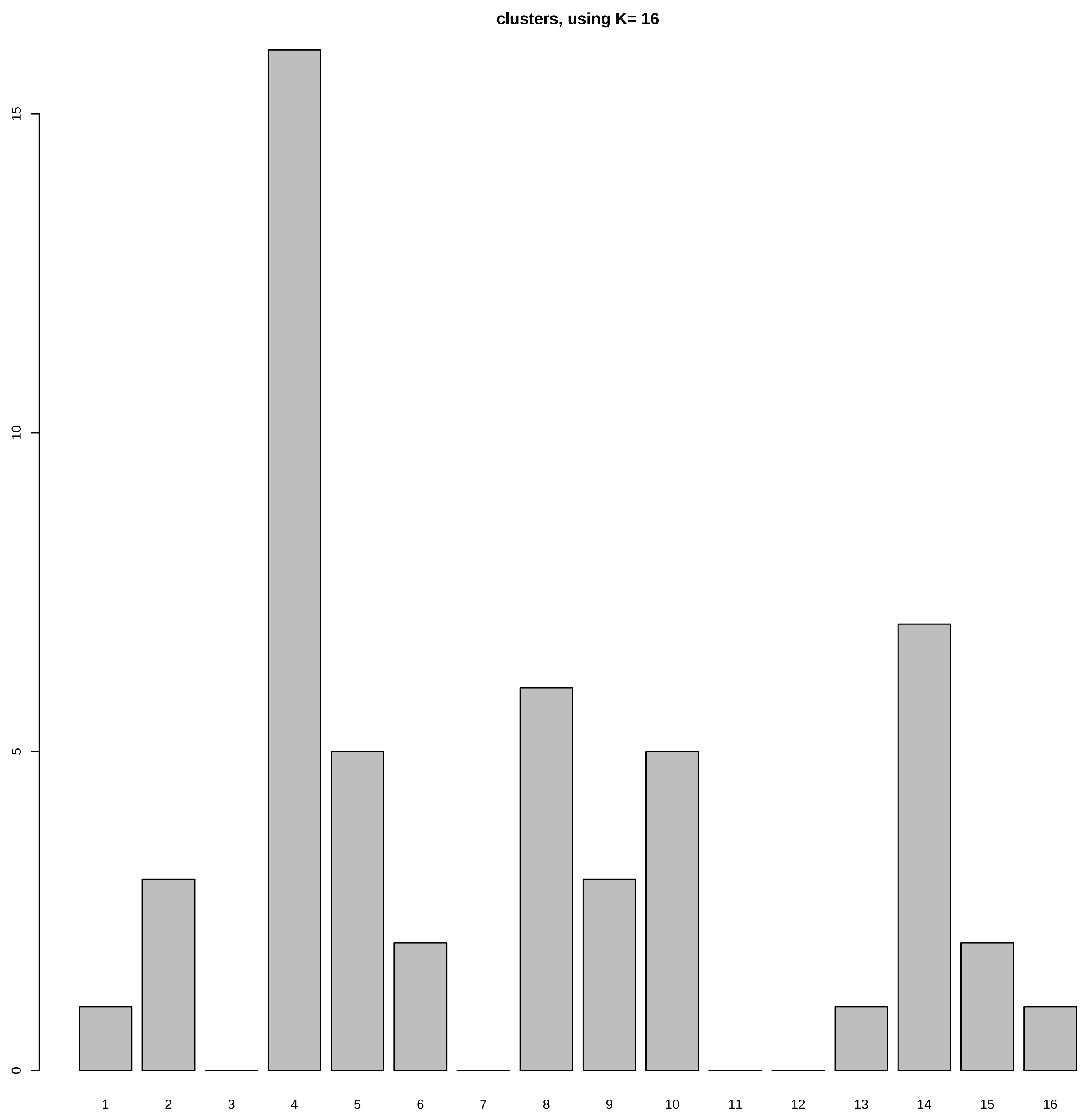
clusters, using K= 16
0
5
10
15
1
2
3
4
5
6
7
8
9
10
11
12
13
14
15
16

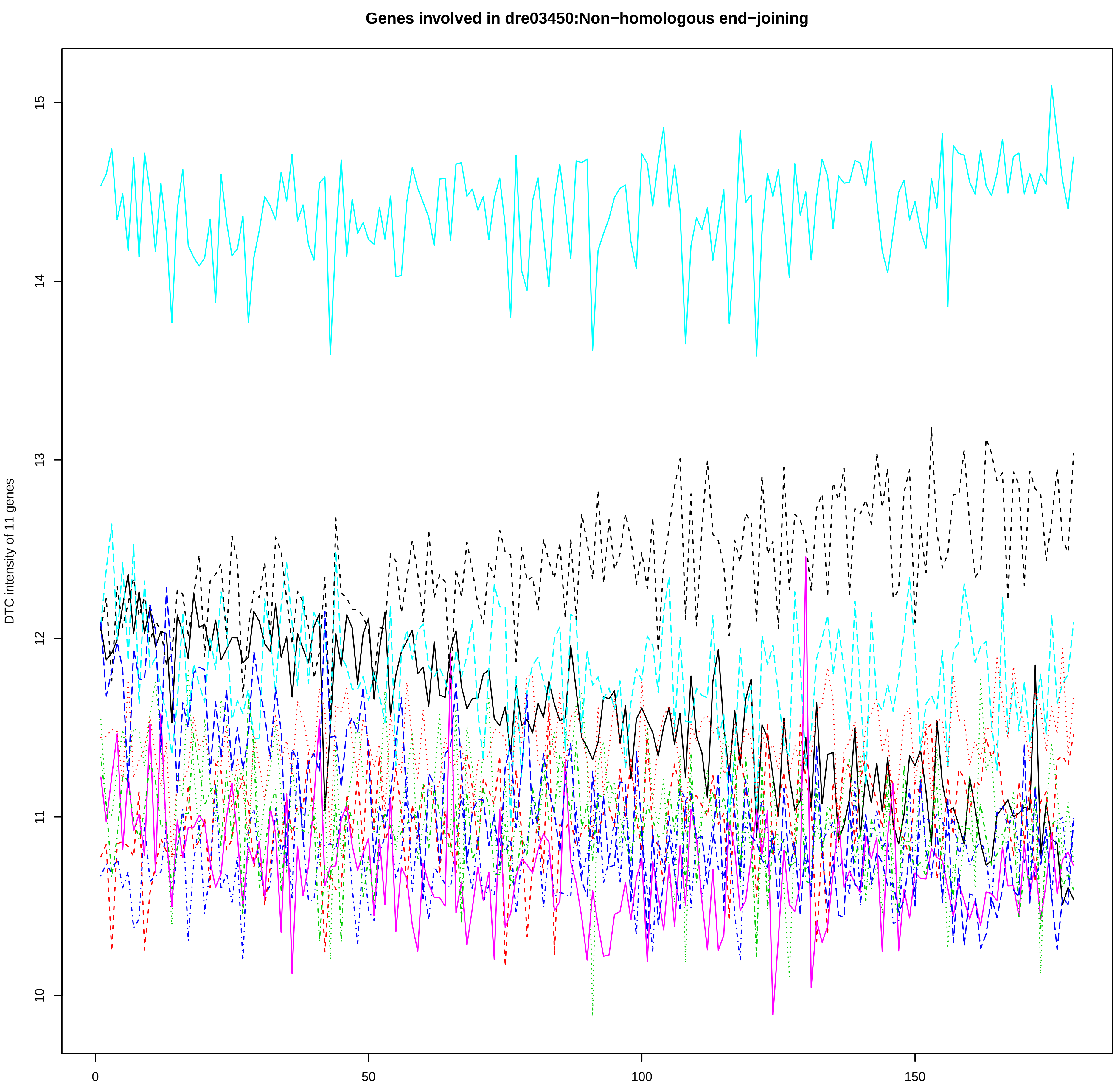
Genes involved in dre03450:Non−homologous end−joining
DTC intensity of 11 genes
10
11
12
13
14
15
0
50
100
150

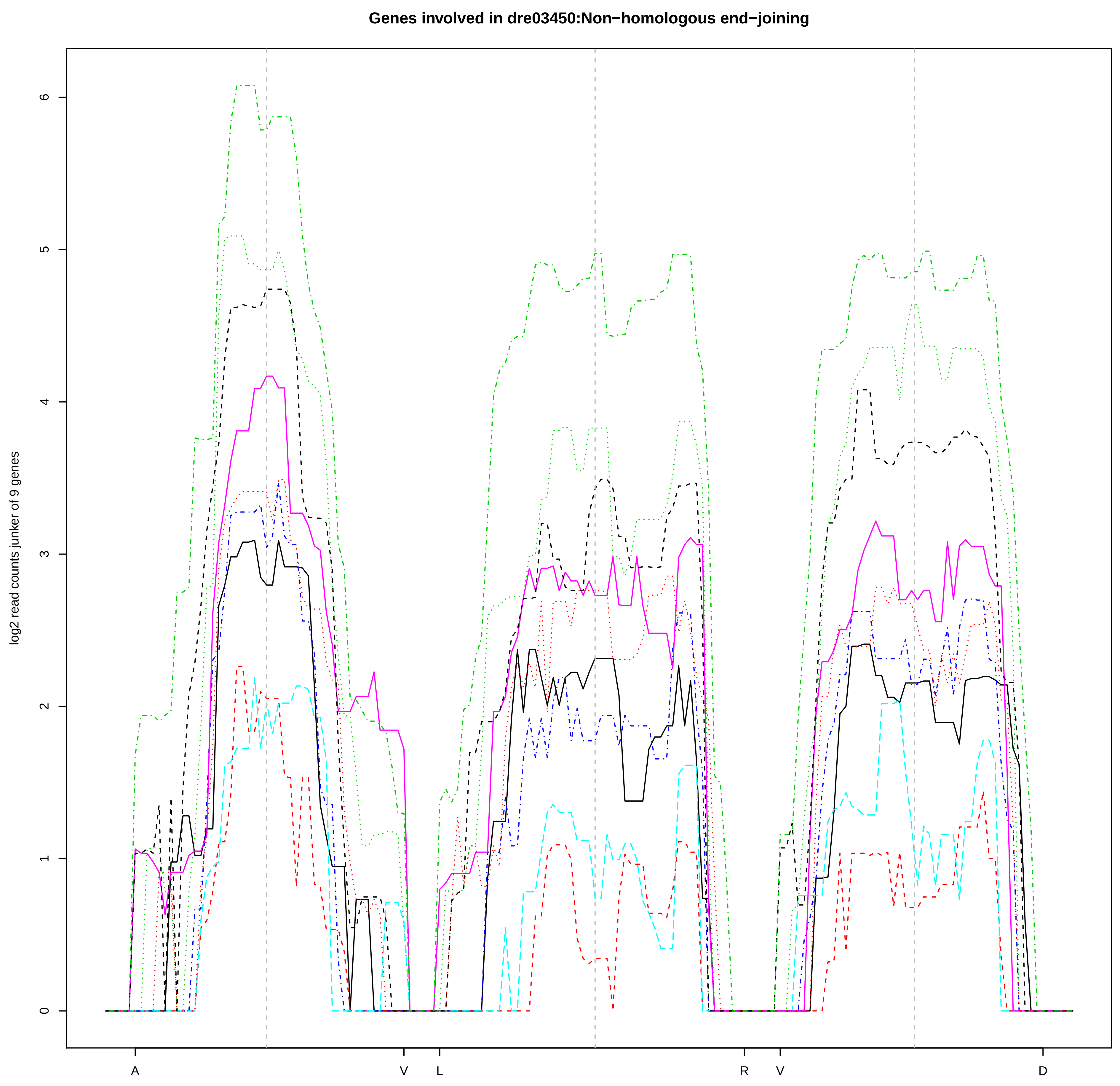
Genes involved in dre03450:Non−homologous end−joining
log2 read counts junker of 9 genes
0
1
2
3
4
5
6
A
V
L
R
V
D

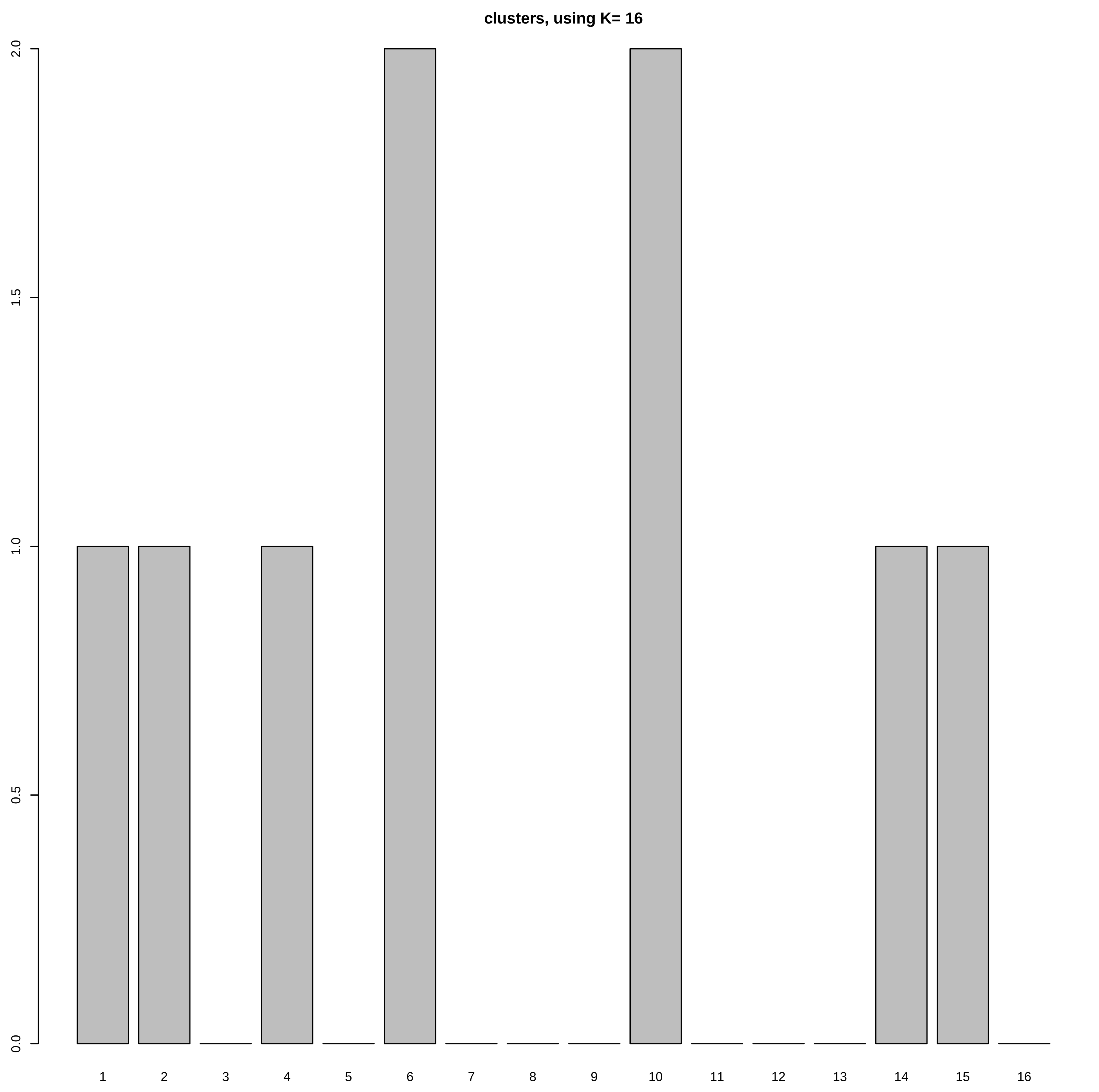
clusters, using K= 16
0.0
0.5
1.0
1.5
2.0
1
2
3
4
5
6
7
8
9
10
11
12
13
14
15
16

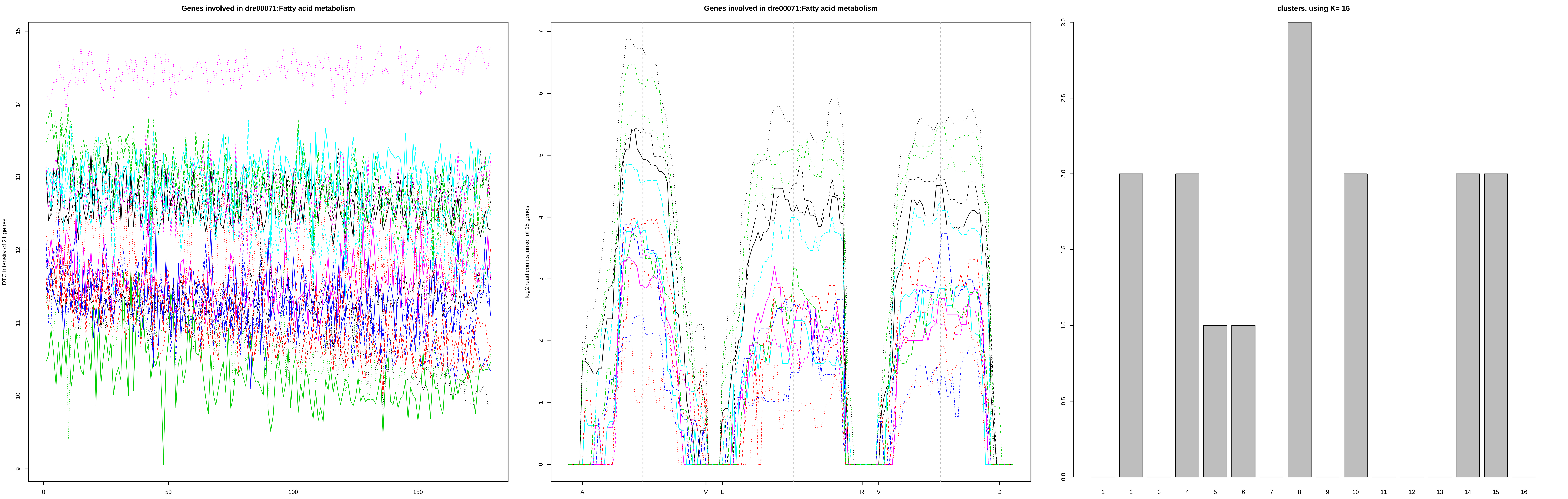
Genes involved in dre00071:Fatty acid metabolism
DTC intensity of 21 genes
Genes involved in dre00071:Fatty acid metabolism
log2 read counts junker of 15 genes
A
V
L
R
V
D
clusters, using K= 16

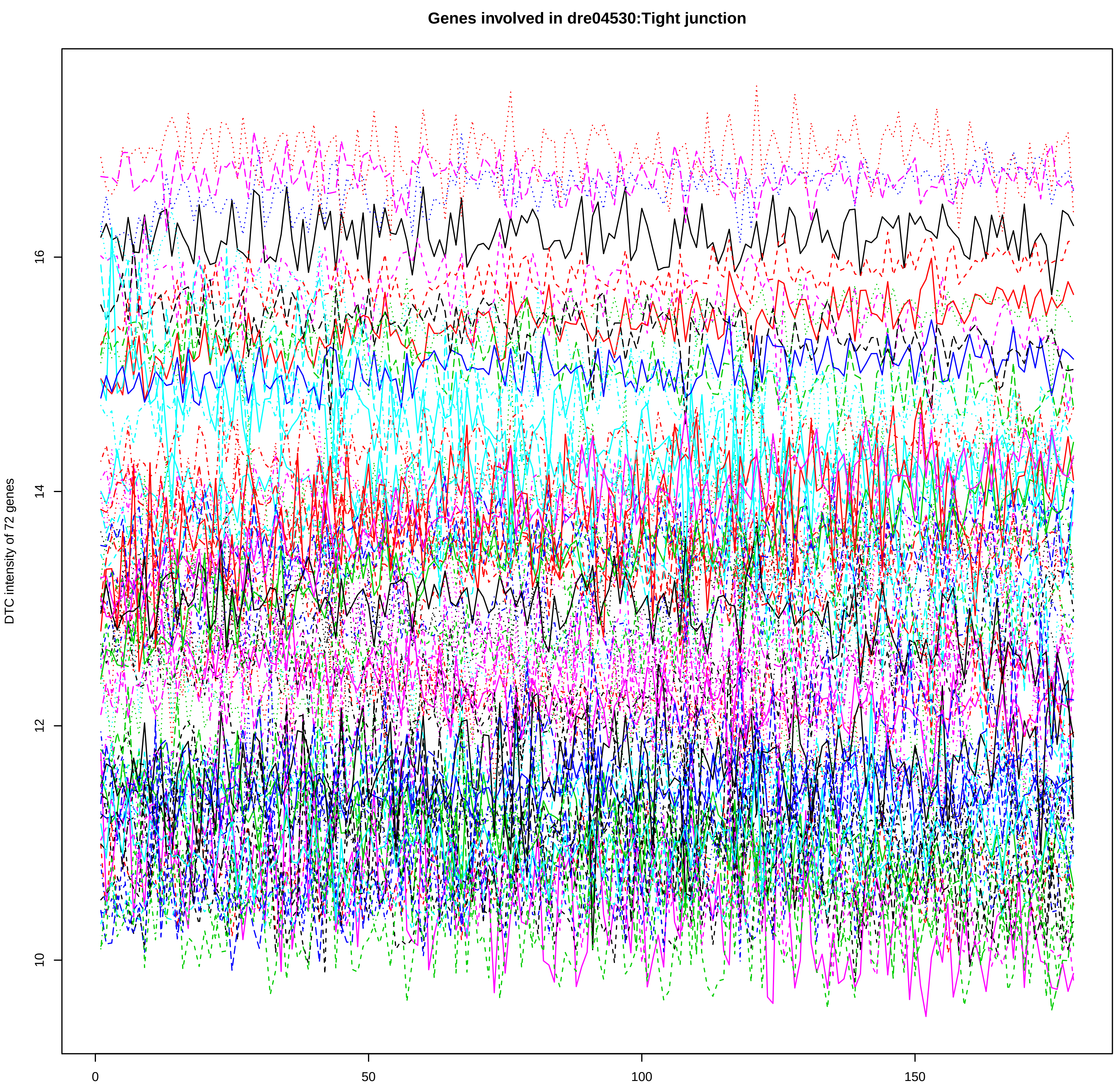
Genes involved in dre04530:Tight junction
DTC intensity of 72 genes
10
12
14
16
0
50
100
150

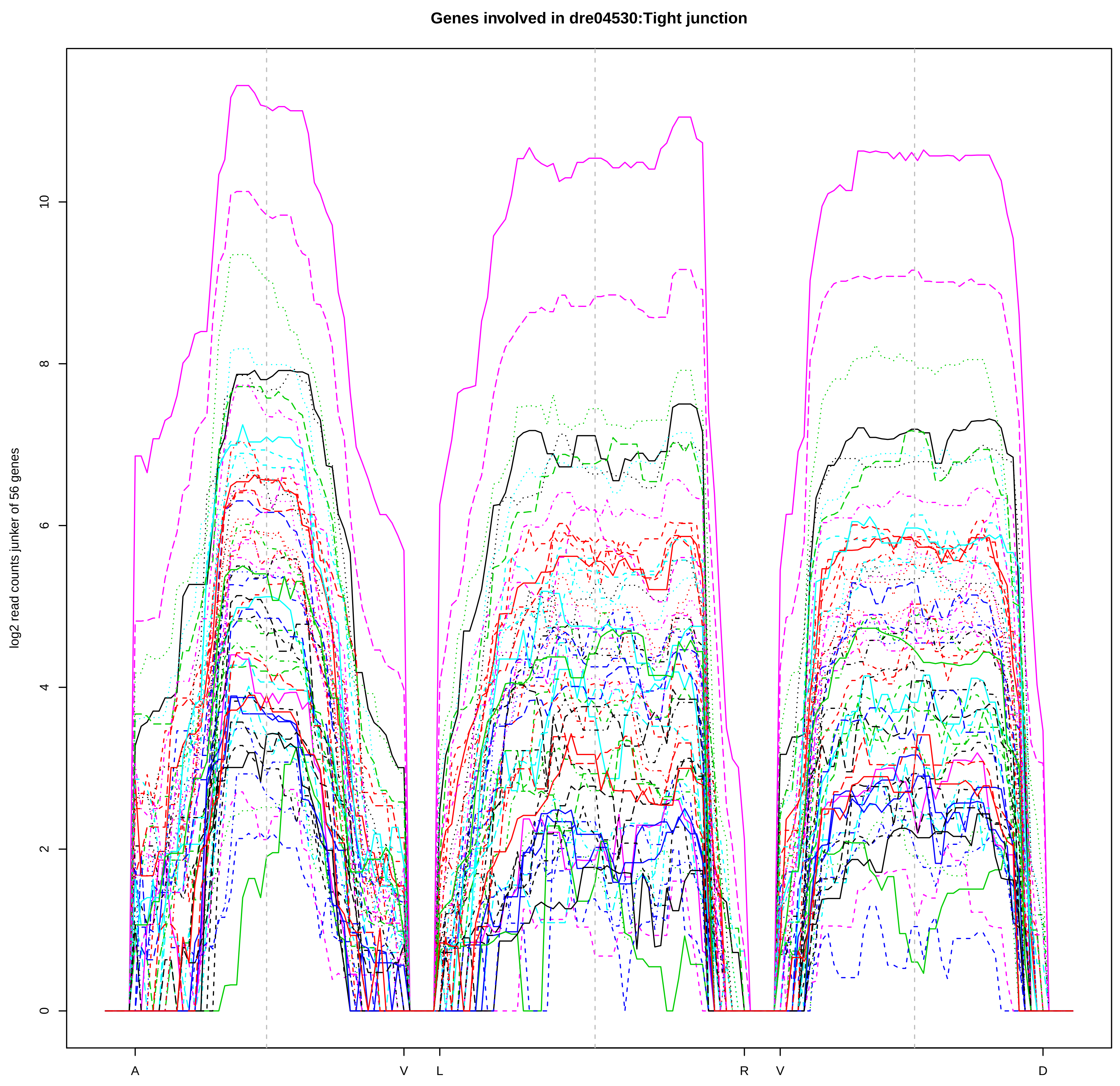
Genes involved in dre04530:Tight junction
log2 read counts junker of 56 genes
0
2
4
6
8
10
A
V
L
R
V
D

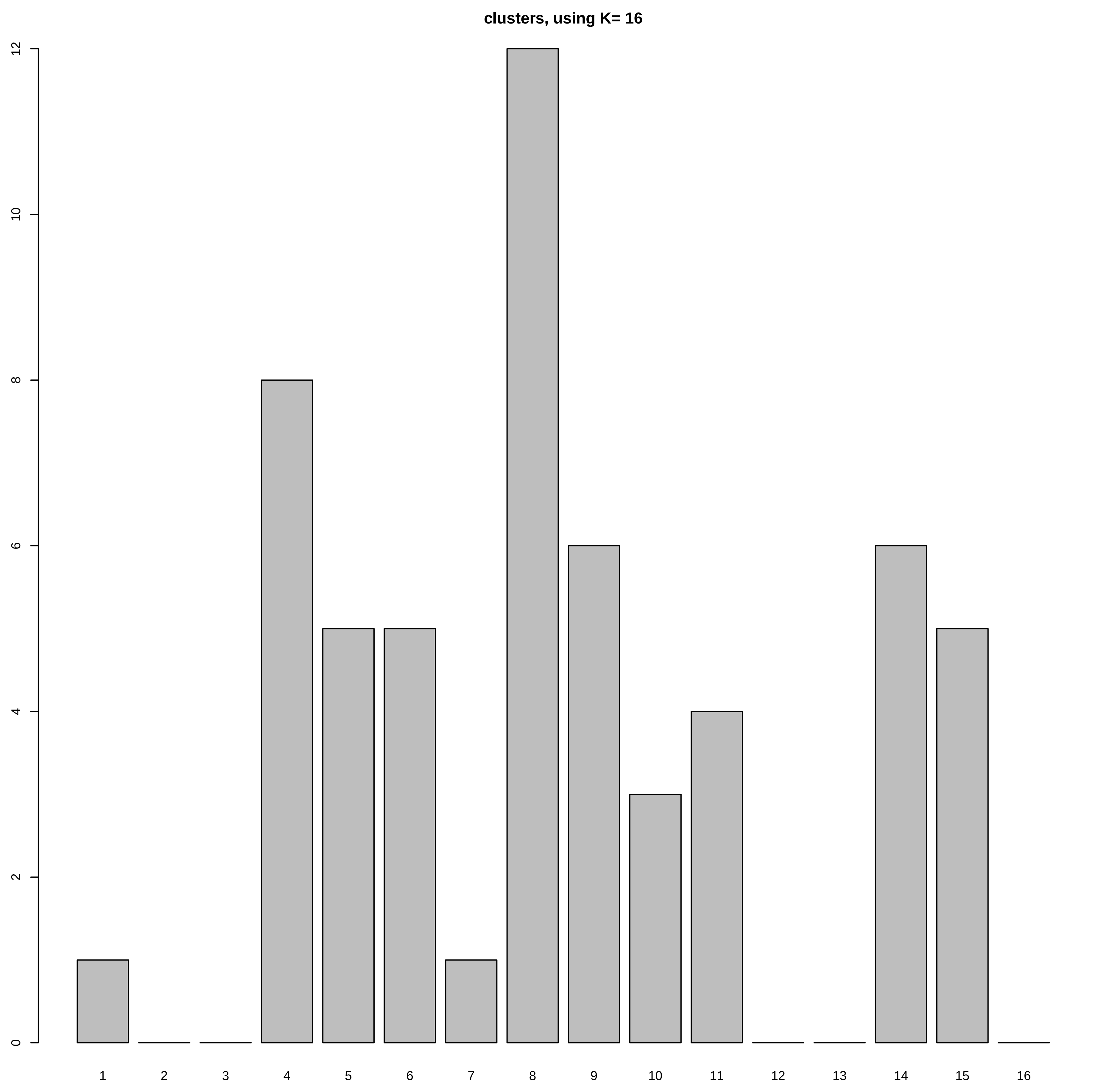
clusters, using K= 16
0
2
4
6
8
10
12
1
2
3
4
5
6
7
8
9
10
11
12
13
14
15
16

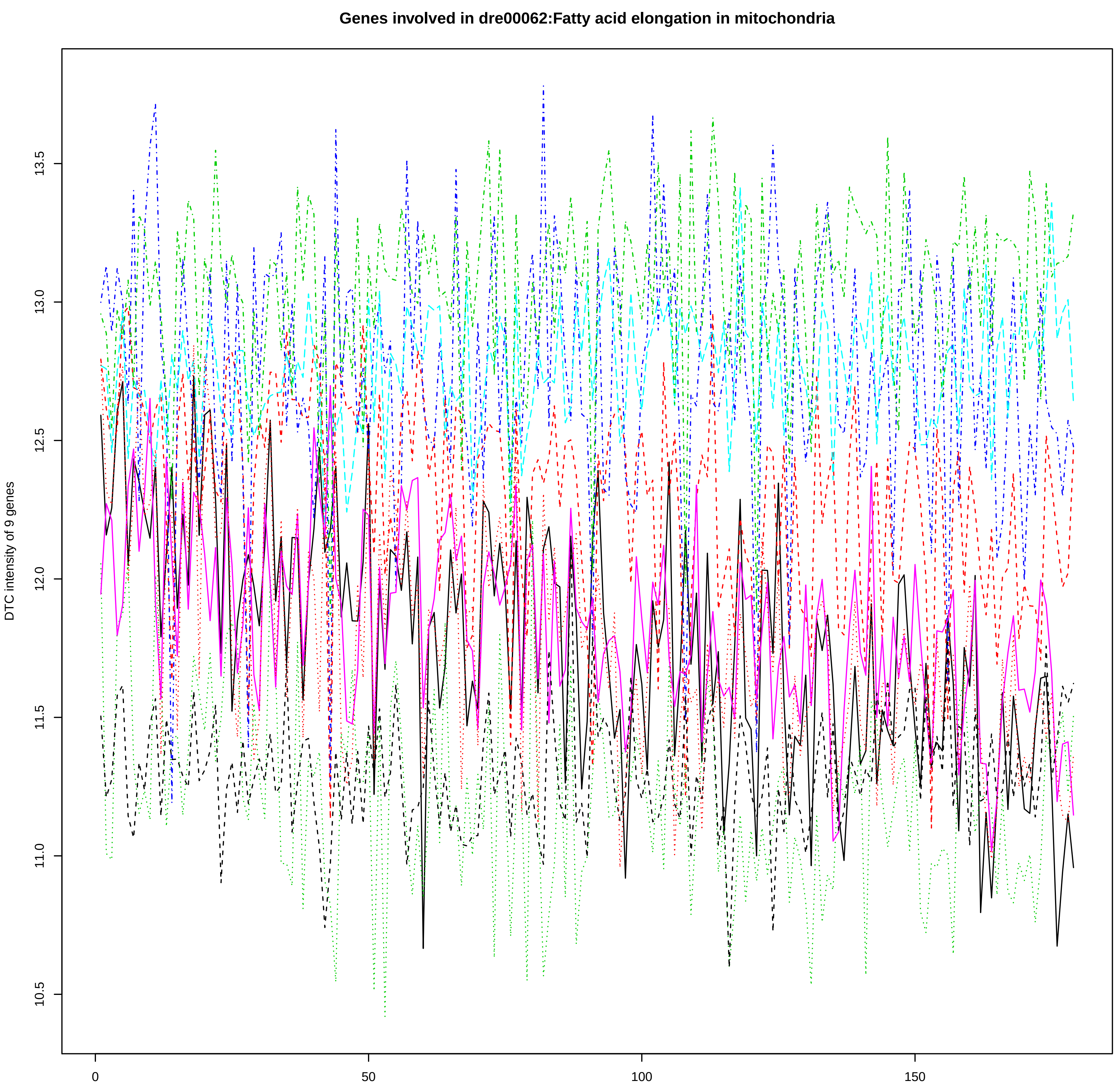
Genes involved in dre00062:Fatty acid elongation in mitochondria
DTC intensity of 9 genes
10.5
11.0
11.5
12.0
12.5
13.0
13.5
0
50
100
150
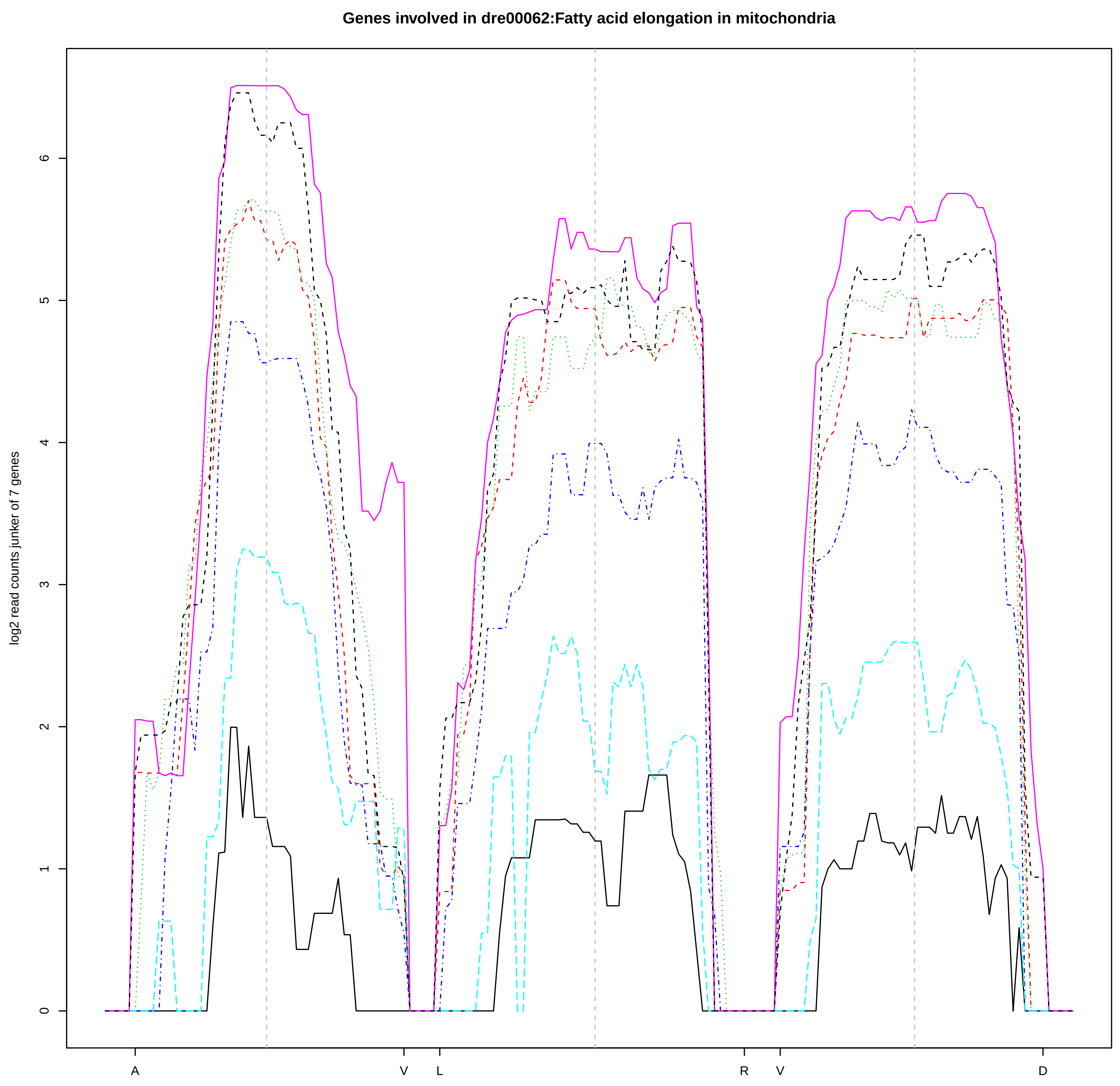
Genes involved in dre00062:Fatty acid elongation in mitochondria
log2 read counts junker of 7 genes
0
1
2
3
4
5
6
A
V
L
R
V
D
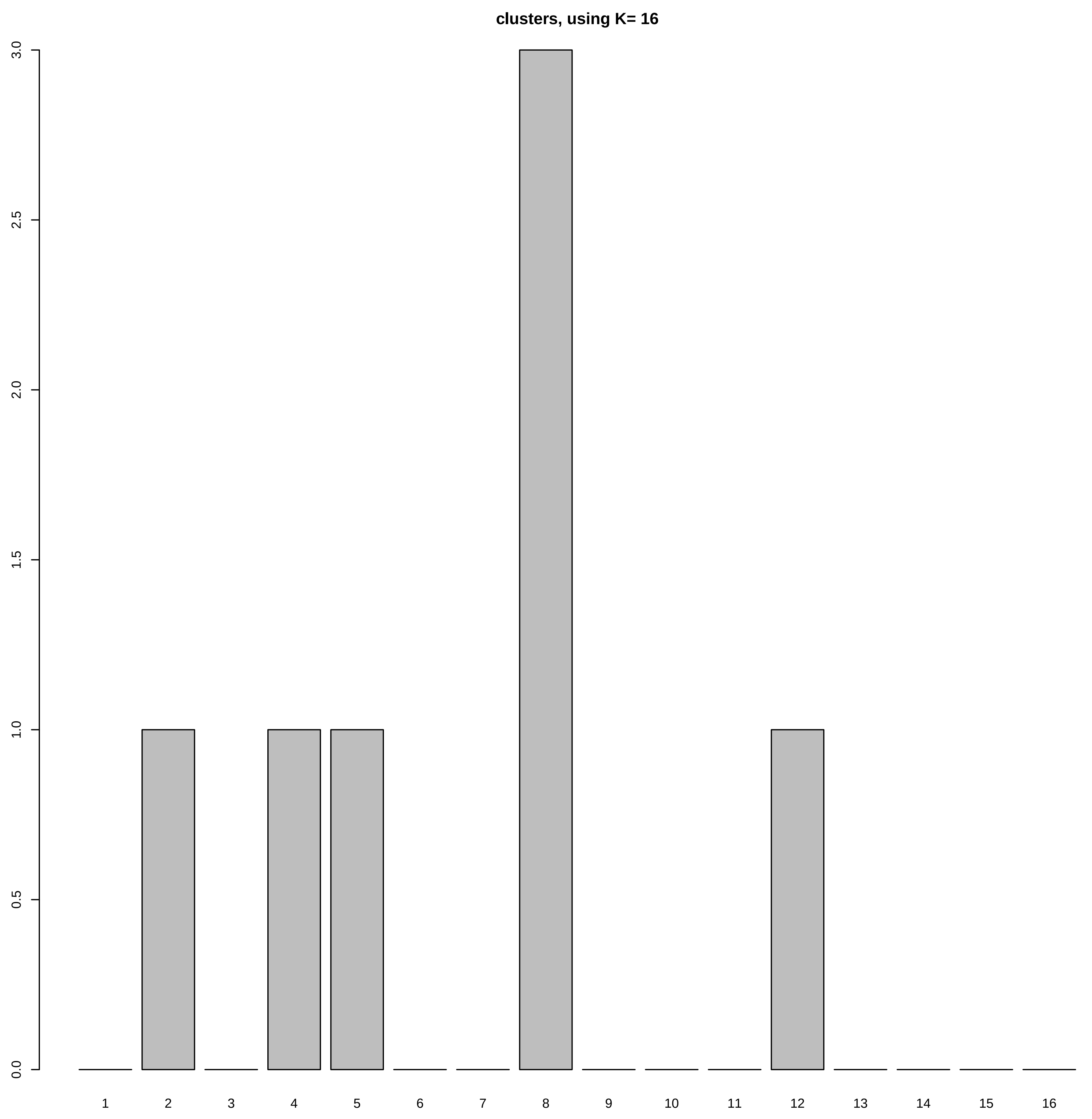
clusters, using K= 16
0.0
0.5
1.0
1.5
2.0
2.5
3.0
1
2
3
4
5
6
7
8
9
10
11
12
13
14
15
16

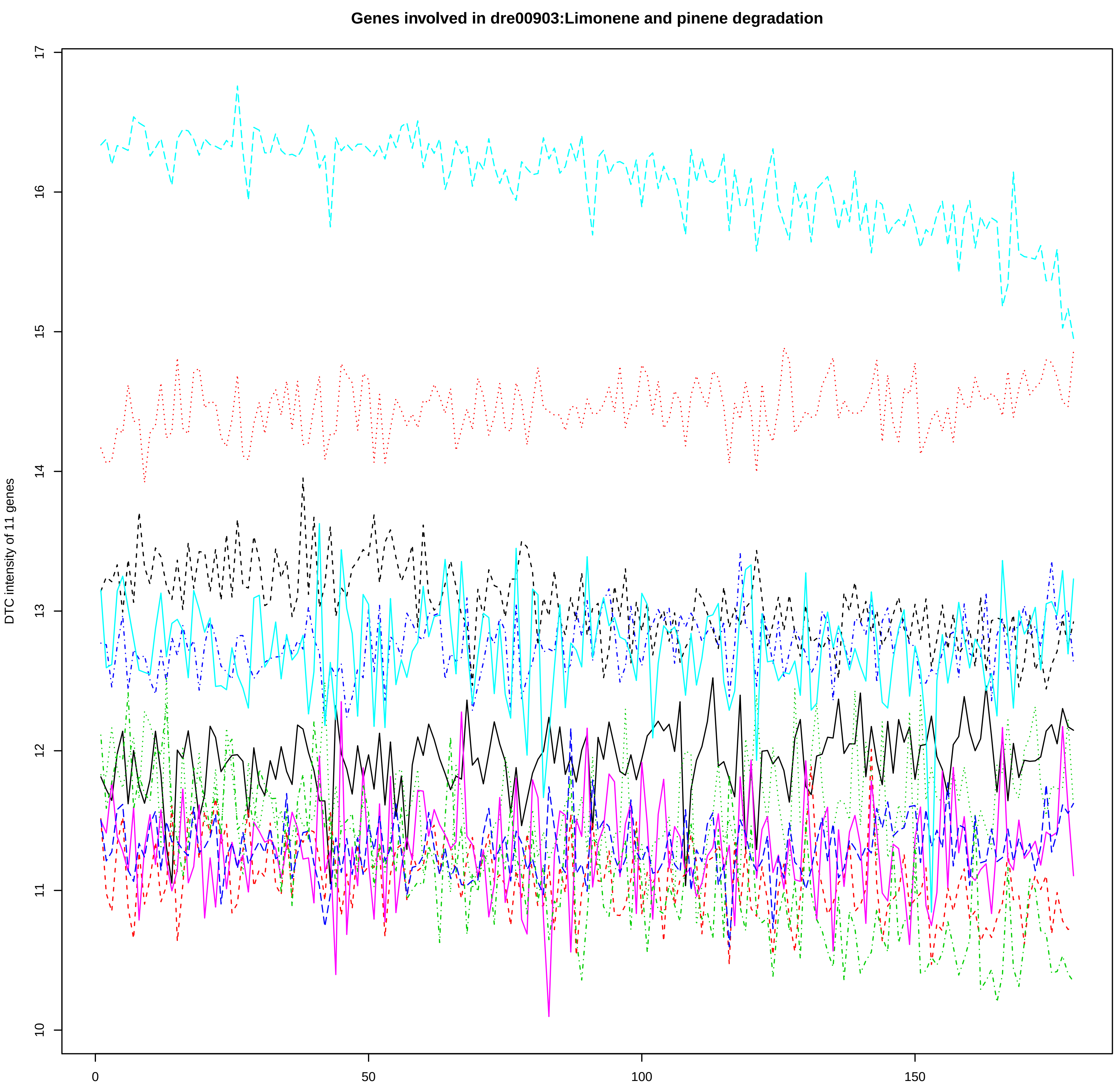

Genes involved in dre00903:Limonene and pinene degradation
DTC intensity of 11 genes

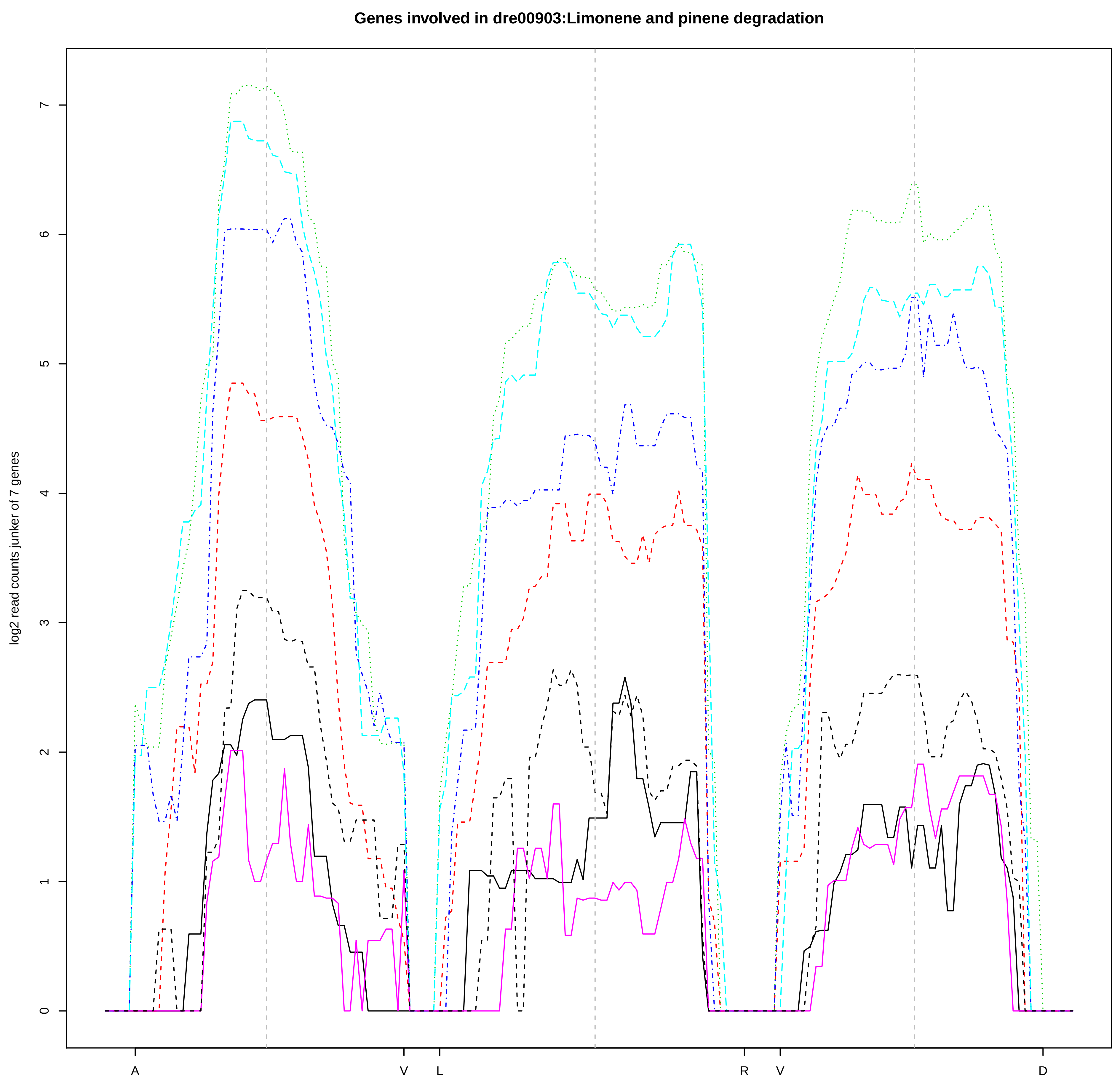

Genes involved in dre00903:Limonene and pinene degradation
log2 read counts junker of 7 genes
A
V
L
R
V
D

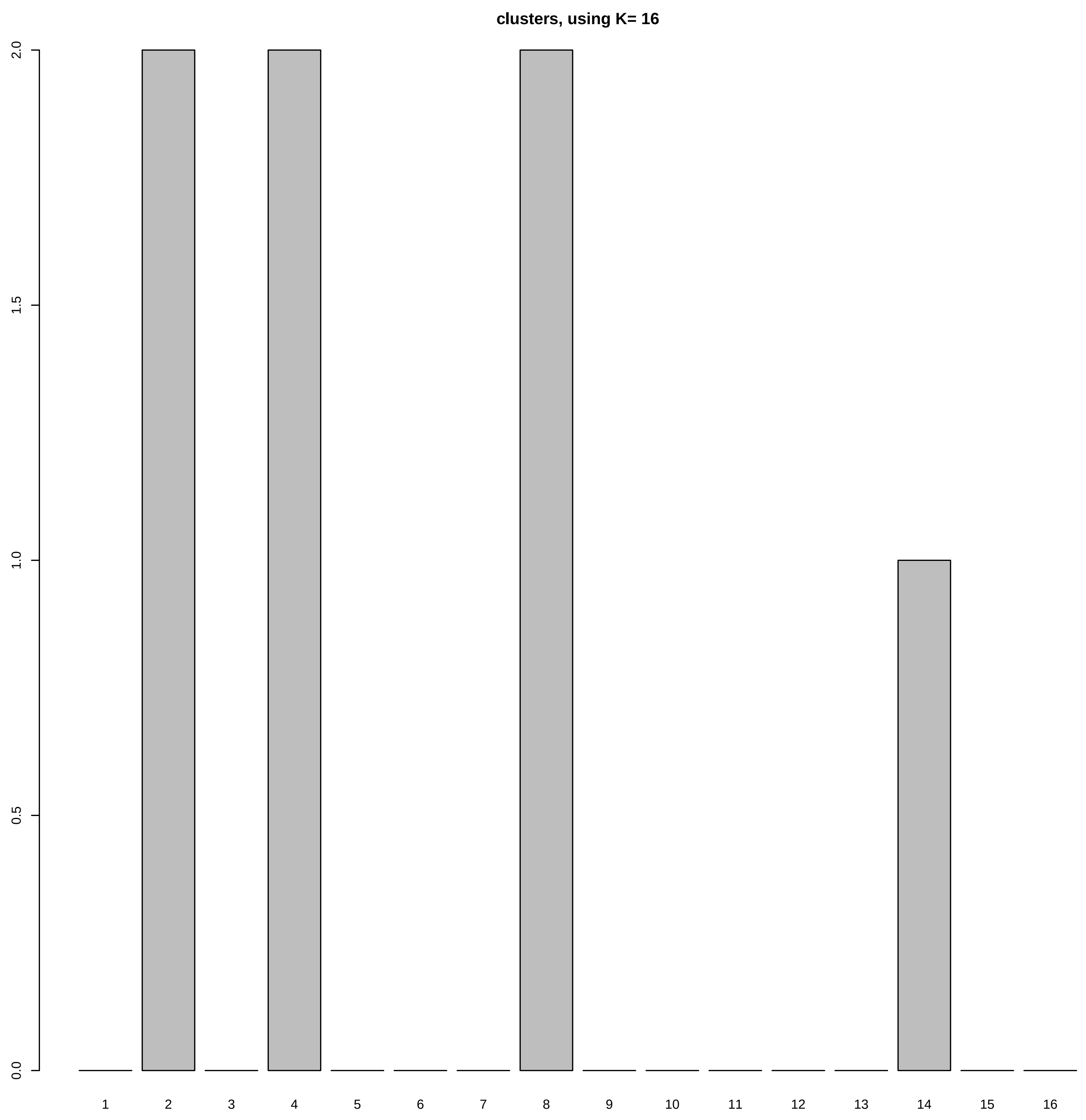

clusters, using K= 16

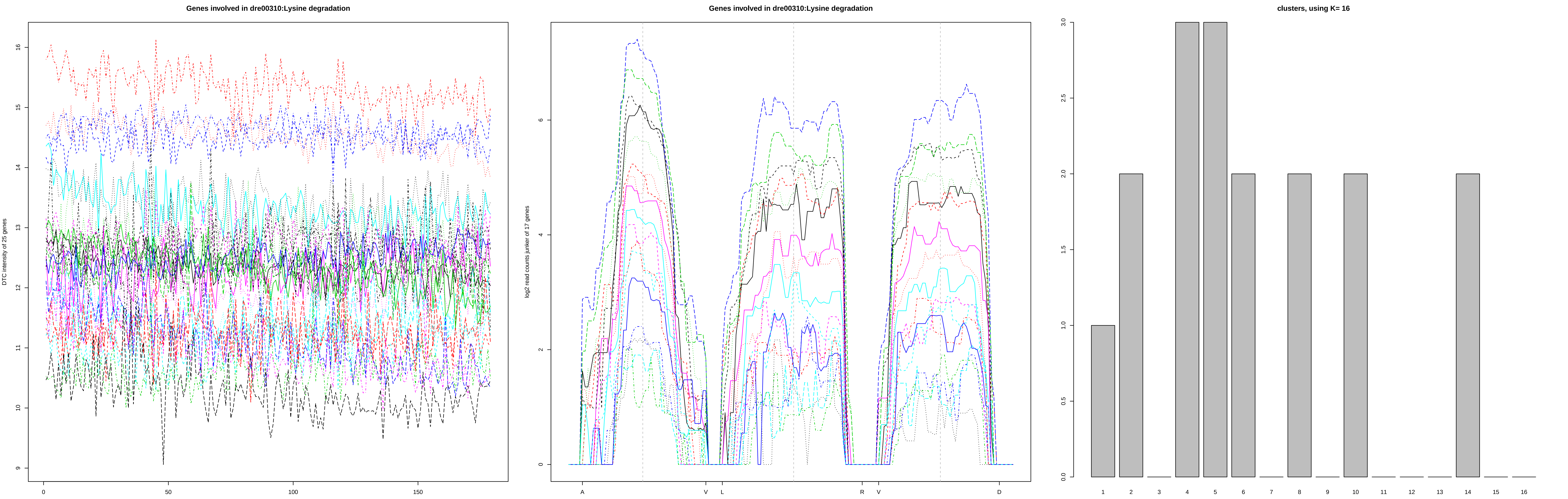
Genes involved in dre00310:Lysine degradation
DTC intensity of 25 genes
9
10
11
12
13
14
15
16
0
50
100
150
Genes involved in dre00310:Lysine degradation
log2 read counts junker of 17 genes
0
2
4
6
A
V
L
R
V
D
clusters, using K= 16
0.0
0.5
1.0
1.5
2.0
2.5
3.0
1
2
3
4
5
6
7
8
9
10
11
12
13
14
15
16

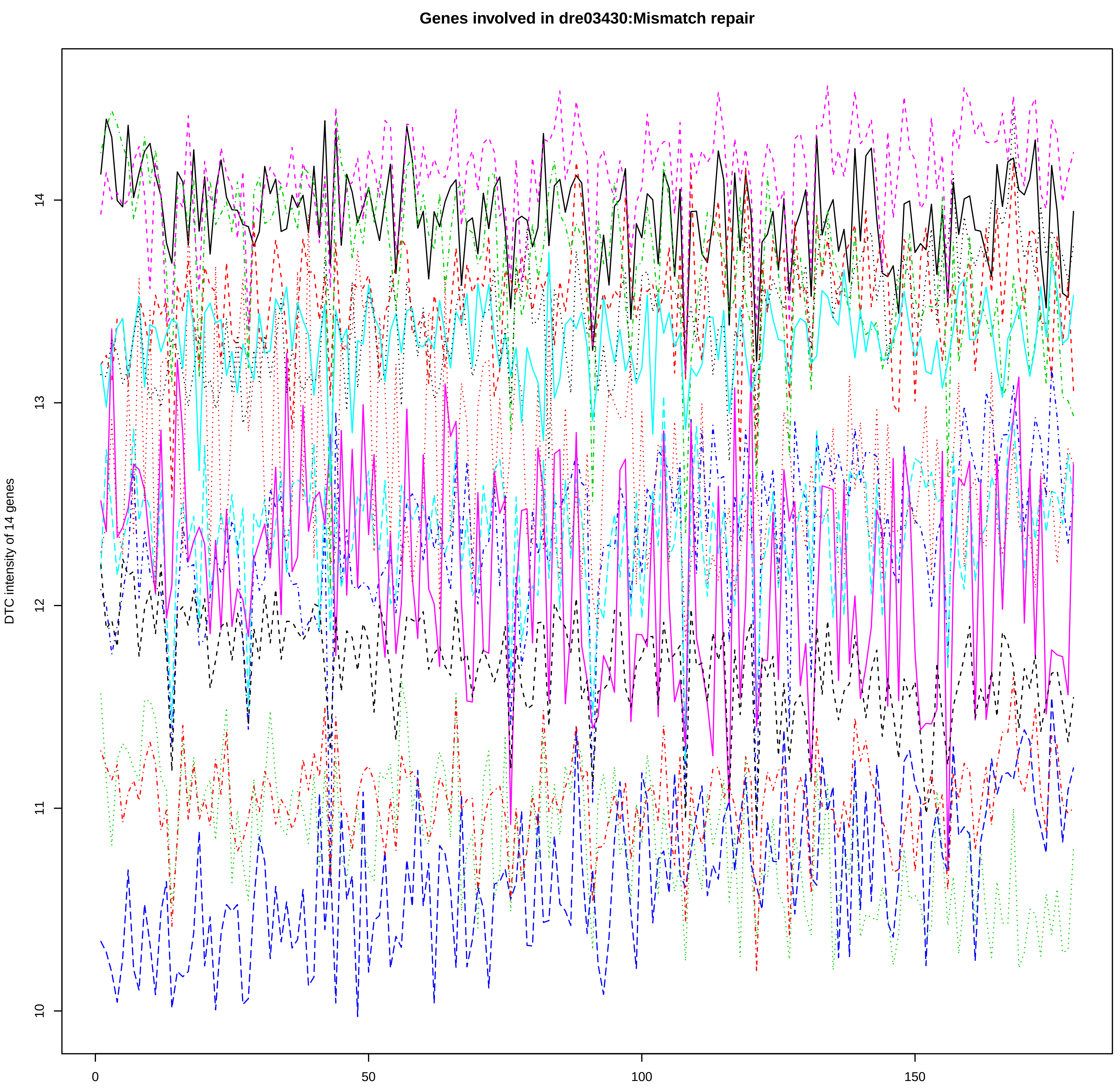
Genes involved in dre03430:Mismatch repair
DTC intensity of 14 genes
10
11
12
13
14
0
50
100
150

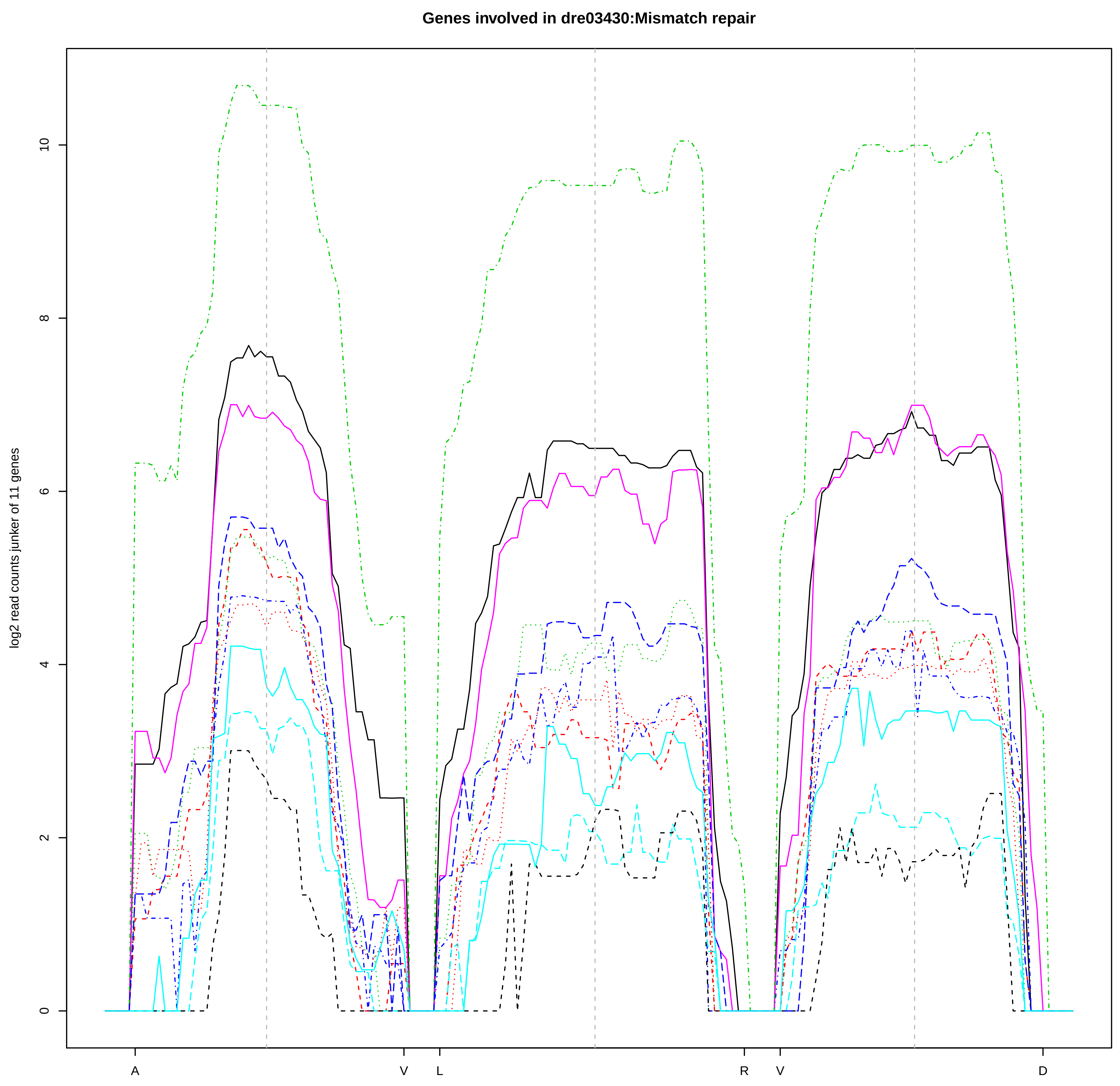
Genes involved in dre03430:Mismatch repair
log2 read counts junker of 11 genes
0
2
4
6
8
10
A
V
L
R
V
D

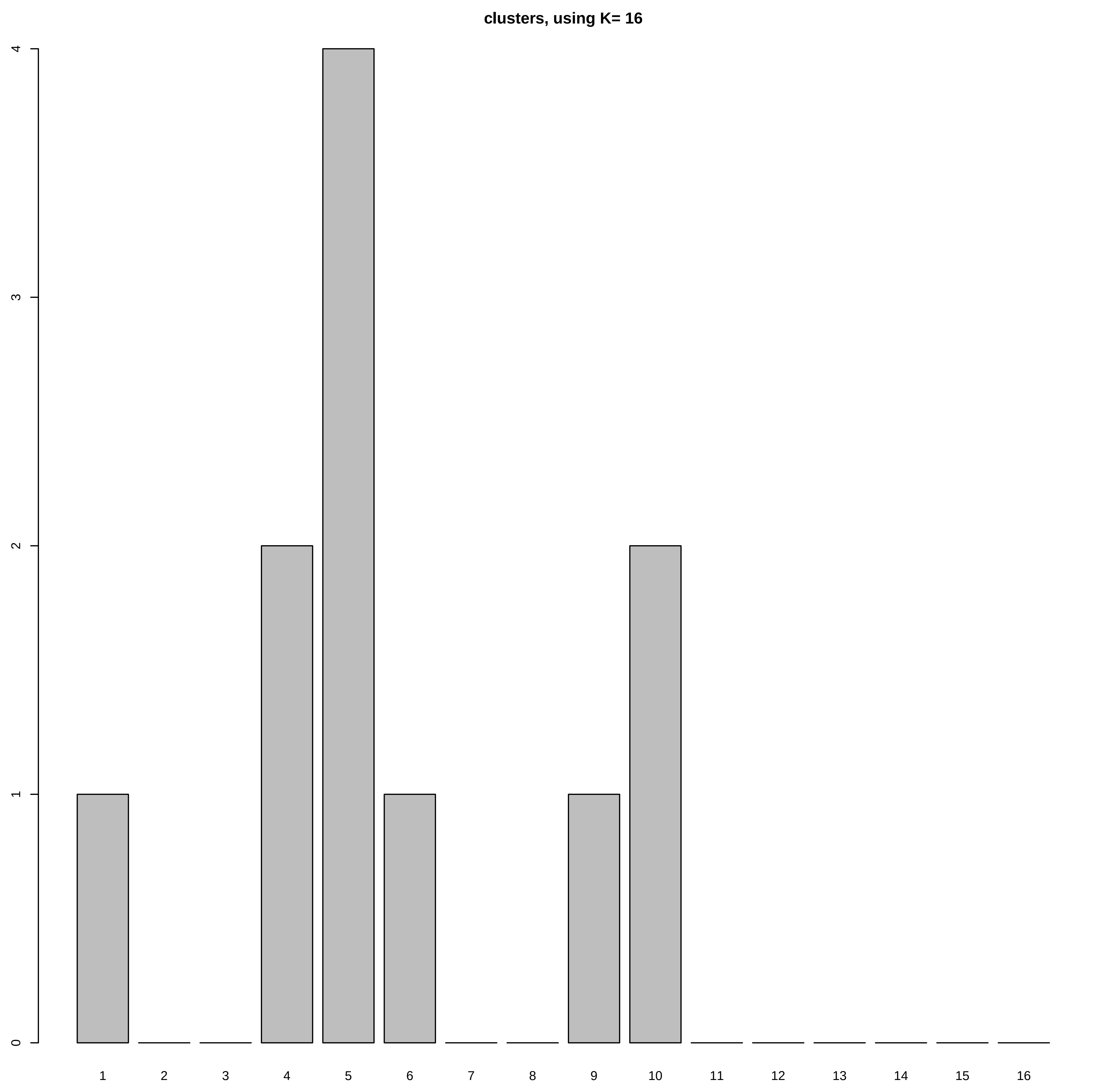
clusters, using K= 16
0
1
2
3
4
1
2
3
4
5
6
7
8
9
10
11
12
13
14
15
16

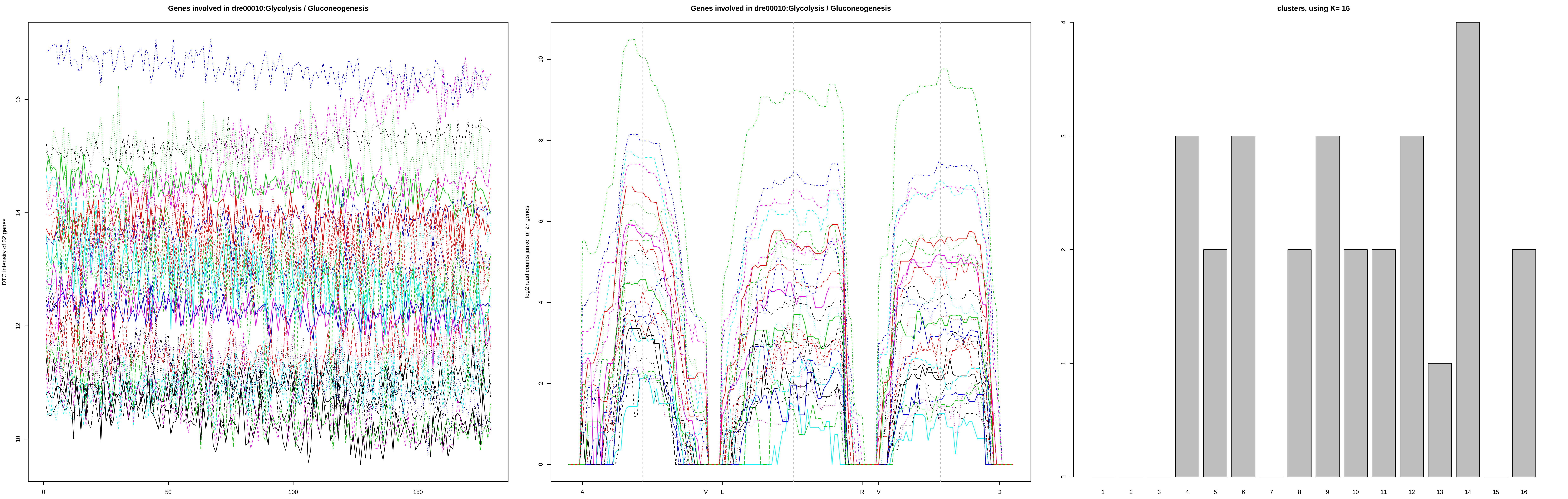

Genes involved in dre00010:Glycolysis / Gluconeogenesis
DTC intensity of 32 genes
10
12
14
16
0
50
100
150
Genes involved in dre00010:Glycolysis / Gluconeogenesis
log2 read counts junker of 27 genes
0
2
4
6
8
10
A
V
L
R
V
D
clusters, using K= 16
0
1
2
3
4
1
2
3
4
5
6
7
8
9
10
11
12
13
14
15
16

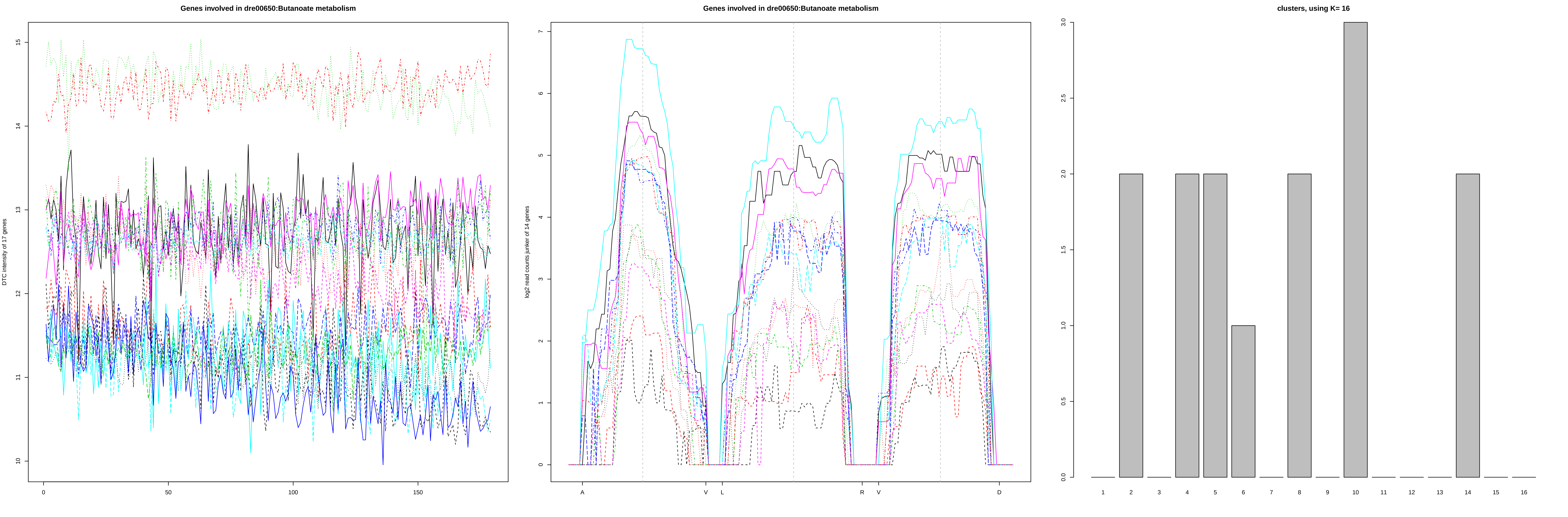

Genes involved in dre00650:Butanoate metabolism
DTC intensity of 17 genes
Genes involved in dre00650:Butanoate metabolism
log2 read counts junker of 14 genes
A
V
L
R
V
D
clusters, using K= 16
