# Supplementary material for: Transcriptome dynamics in early zebrafish embryogenesis determined by high-resolution time course analysis of 180 successive, individual zebrafish embryos
Source: BMC Genomics. 2017 Apr 11;18:287. doi: 10.1186/s12864-017-3672-z (PMC5387192; doi:10.1186/s12864-017-3672-z)

Example of the determination of a stopping and starting gene in this time course

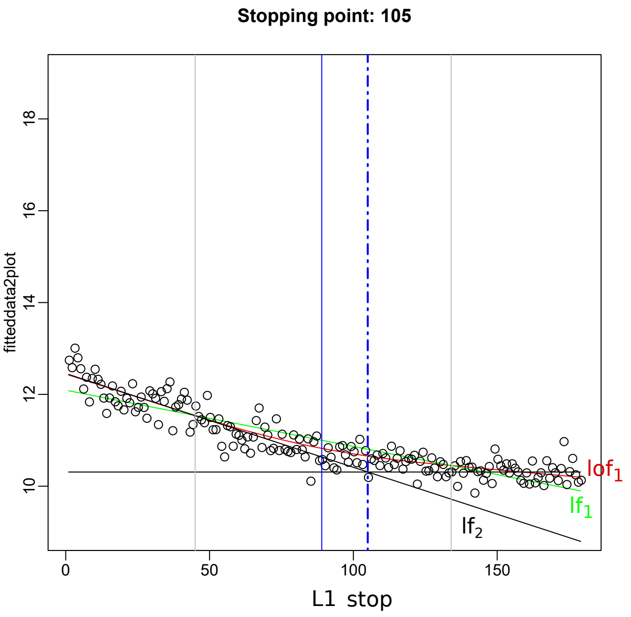

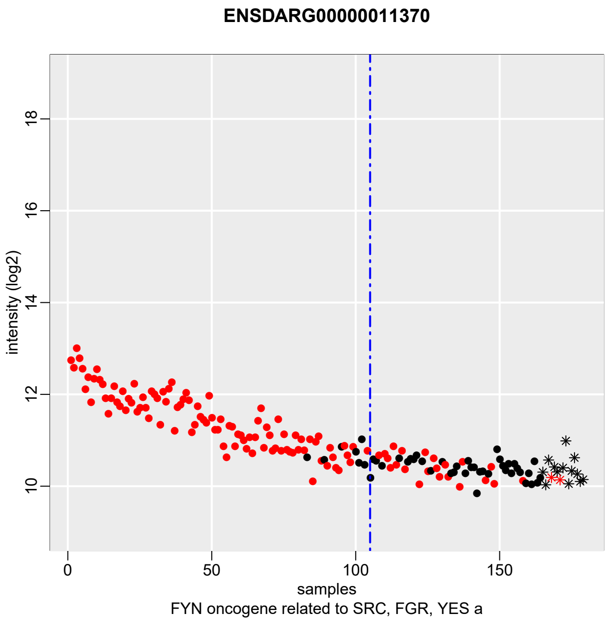

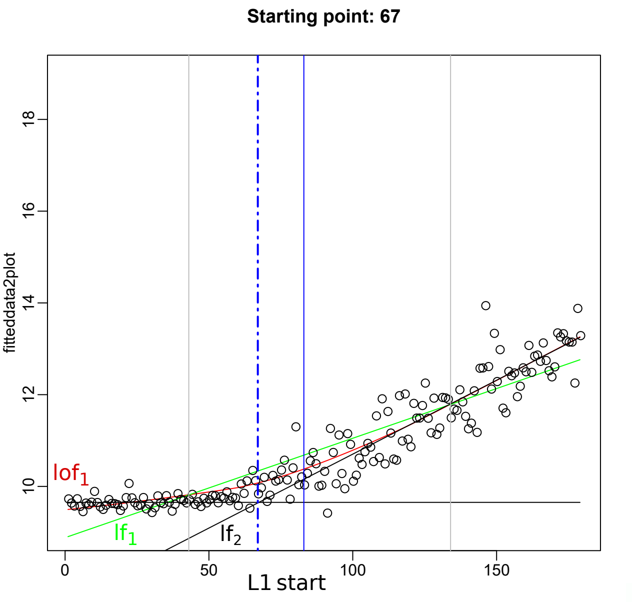

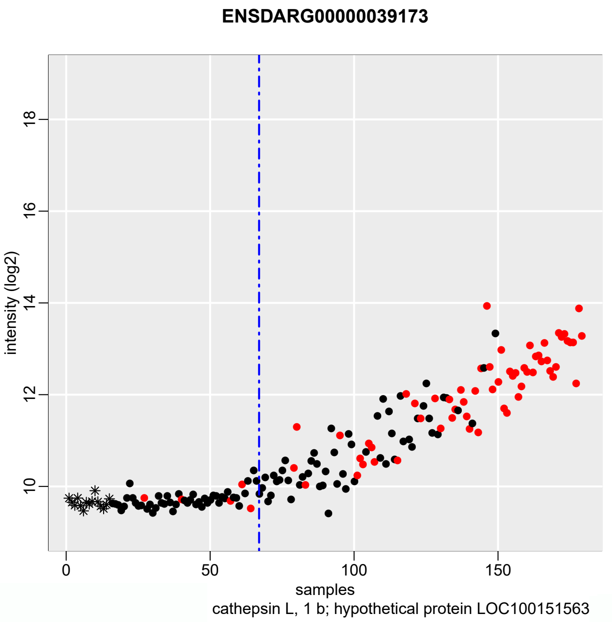

Supplement: Supplementary file 18 — Example of the determination of a stopping point and a starting point. For an explanation see Methods, paragraph “Identification of starting and stopping points”. lf1 = linear fit on all samples; lof1 = lowest fit on all samples; grey lines indicate intersect of lf1 and lof1; L1: embryo with the largest difference between lf1 and lof1; for stopping genes lf2 = linear fit of L1 to 179; for starting genes lf2 = linear fit of 1 to L1; embryos marked with an asterisk: ‘not present’ probes in the last (stopping) 15 respectively in the first (starting) 15 embryos; horizontal black line: median probe intensity of embryos marked with an asterisk; dashed line: intersect of lf2 and the horizontal black line indicating the stopping respectively the starting point. (PDF 131 kb) [file 12864_2017_3672_MOESM18_ESM.pdf]
